# To ERV Is Human: A Phenotype-Wide Scan Linking Polymorphic Human Endogenous Retrovirus-K Insertions to Complex Phenotypes

**DOI:** 10.3389/fgene.2018.00298

**Published:** 2018-08-14

**Authors:** Amelia D. Wallace, George A. Wendt, Lisa F. Barcellos, Adam J. de Smith, Kyle M. Walsh, Catherine Metayer, Joseph F. Costello, Joseph L. Wiemels, Stephen S. Francis

**Affiliations:** ^1^Division of Epidemiology, School of Public Health, University of California, Berkeley, Berkeley, CA, United States; ^2^Division of Epidemiology, School of Community Health Sciences, University of Nevada, Reno, NV, United States; ^3^Department of Epidemiology and Biostatistics, Helen Diller Comprehensive Cancer Center, University of California, San Francisco, San Francisco, CA, United States; ^4^Department of Neurosurgery, Duke University, Durham, NC, United States; ^5^Department of Neurosurgery, Helen Diller Comprehensive Cancer Center, University of California, San Francisco, San Francisco, CA, United States

**Keywords:** HERV-K, GWAS, polymorphism, eQTL, recombination

## Abstract

Approximately 8% of the human genome is comprised of endogenous retroviral insertions (ERVs) originating from historic retroviral integration into germ cells. The function of ERVs as regulators of gene expression is well established. Less well studied are insertional polymorphisms of ERVs and their contribution to the heritability of complex phenotypes. The most recent integration of ERV, HERV-K, is expressed in a range of complex human conditions from cancer to neurologic diseases. Using an in-house computational pipeline and whole-genome sequencing data from the diverse 1,000 Genomes Phase 3 population (*n* = 2,504), we identified 46 polymorphic HERV-K insertions that are tagged by adjacent single nucleotide polymorphisms (SNPs). To test the potential role of polymorphic HERV-K in the heritability of complex diseases, existing databases were queried for enrichment of established relationships between the HERV-K insertion-associated SNPs (hiSNPs), and tissue specific gene expression and disease phenotypes. Overall, hiSNPs for the 46 polymorphic HERV-K sites were statistically enriched (*p* < 1.0E^−16^) for eQTLs across 44 human tissues. Fifteen of the 46 HERV-K insertions had hiSNPs annotated in the EMBL-EBI GWAS Catalog and cumulatively associated with >100 phenotypes. Experimental factor ontology enrichment analysis suggests that polymorphic HERV-K specifically contribute to neurologic and immunologic disease phenotypes, including traits related to intra cranial volume (FDR 2.00E-09), Parkinson's disease (FDR 1.80E-09), and autoimmune diseases (FDR 1.80E-09). These results provide strong candidates for context-specific study of polymorphic HERV-K insertions in disease-related traits, serving as a roadmap for future studies of the heritability of complex disease.

## Introduction

Retroviruses are a class of RNA virus that undergoes reverse transcription to DNA during the infectious cycle inside a host cell. At the proviral stage, retroviral DNA integrates into the host DNA to produce viral proteins. Integration into germ cells can result in endogenization, wherein the virus can be vertically transmitted via standard Mendelian inheritance mechanisms. Endogenous retroviruses (ERV) are ancient examples of proviruses that integrated and endogenized into the human genome >40mya (Bannert and Kurth, 2006). In modern humans, ERVs account for approximately 8% of the genome (Bannert and Kurth, 2006). Their relative stability, as well as the conservation of orthologs in other primate genomes suggests that they induce genome plasticity and can enhance evolutionary fitness (Cordaux and Batzer, 2009; Feschotte and Gilbert, 2012; Grow et al., 2015). Retroviruses are reliant on the fitness of their host for survival and the long-standing evolutionary cooperation between ERVs and humans may represent a symbiotic relationship (Jern and Coffin, 2008). The positive selection of persistent ERVs in the genome may have resulted from increasing the probability of survival to reproductive age [via adaptive effects on placentation (Simpson et al., 1996); and immune (Hurst and Magiorkinis, 2015) and brain development (Mortelmans et al., 2016)]. The phenotypic effects of ERVs on the post-reproductive adult, however, remain unclear and are of growing interest (Li et al., 2015; Bowen et al., 2016; Sekar et al., 2016).

Previous studies have described the potential mechanisms by which ERVs influence human phenotypes. ERV insertions introduce viral genes and, due to their inter-individual homology can generate copy-number variants via non-allelic homologous recombination (Campbell et al., 2014). They modify transcription by adding enhancers (Chuong et al., 2013) and promoters (Fuchs et al., 2013), disrupting intron structure, causing RNA interference (Ling et al., 2003) adding poly-A tails (Kim, 2012), and altering DNA methylation (Kreimer et al., 2013). ERV expression, typically restricted in healthy tissues except during placental development, is detected in diseases including cancers and autoimmune disorders [reviewed in (Cegolon et al., 2013; Nexø et al., 2016)].

While the functional effects of ERVs are well established, the vast majority of ERV insertions are fixed across individuals, and so their potential contribution to phenotypic variation has been largely overlooked. The HERV-K subfamily, however, contains human-specific, unfixed insertions, ranging from fully intact provirus to solo long terminal repeat (LTR) sequences (Wildschutte et al., 2016). HERV-K represents one of the most recent ERV integrations into the human genome and numerous insertions have neither been eliminated from the genome (via negative selection or drift), nor fixed (via positive selection or drift). The polymorphic nature of these insertions suggests a potential contribution to causal variation in the heritability of complex phenotypes. Targeted studies have identified specific HERV-K integrations that affect disease risk, for example a polymorphic HERV-KC4 inserted within the complement component 4 (C4A/B) gene appears to be involved in the genetic risk of schizophrenia (Sekar et al., 2016).

Technical limitations have proved a major obstacle in the untargeted identification of polymorphic HERV-K insertions for application to clinical and epidemiologic studies. With the emergence of next-generation sequencing technologies, methods are being developed for the untargeted identification of ERVs among other mobile genetic elements in human genomes (Witherspoon et al., 2010; Ray and Batzer, 2011; Wildschutte et al., 2016). Here, we examine phenotypic effects of all polymorphic HERV-K insertions identifiable from a large, publically available whole genome sequencing (WGS) dataset. With our computational pipeline, we identified HERV-K insertion locations using data from the diverse 1000 Genomes Phase 3 population (*n* = 2,504). By identifying a subset of polymorphic HERV-K insertions with strong associations to adjacent “tagging” single nucleotide polymorphisms (SNPs), we have leveraged several comprehensive SNP annotation databases to test for enrichment of established relationships between HERV-K insertion-associated SNPs (hiSNPs), tissue-specific gene expression, and diverse disease phenotypes.

## Methods

### HERVnGoSeq computational pipeline

To elucidate the broad phenotypic effects of polymorphic HERV-K insertions, we developed a computational pipeline, HERVnGoSeq, to identify the presence/absence of known and novel HERV-K insertions in individual WGS data (Figure S1). Quality-filtered raw WGS were aligned to HERV-K113, one of the youngest HERV-K elements in the human genome with a conserved intact LTR sequence that is also capable of producing viral particles *in vitro* (Boller et al., 2008). Reads that partially aligned to HERV-K113 - chimeric reads - were trimmed and the non-HERV portions of the reads were extracted. The trimmed chimeric reads were then aligned to the human genome (GRCh37/Hg19). The base-pair position of the trimmed end of the read where HERV-K sequence was removed was called as the insertion point. Insertion points were collected for both the forward and reverse complement alignments separately. Insertion points within 1,000 bp of each other were grouped to represent a single insertion point. The presence of putative HERV-K insertions were assigned to each individual if they had at least one chimeric read that aligned to that insertion point. Absence of an insertion was inferred for individuals when they lacked any chimeric reads representing the specific insertion. The complete pipeline and description is available at https://github.com/unreno/chimera.

### HERV-K identification/validation

Putative polymorphic HERV-K elements were nominated via HERVnGoSeq (Figure S1). Sequence similarity between the reference index, HERV-K113 LTR, and the HERV-K10 LTR, which was ancestrally co-opted to form another mobile element class, Sine-VNTR-Alu composite elements (SVA, Hancks and Kazazian, 2010), resulted in nomination of insertion sites of SVA-A, B, and C in addition to HERV-K when the LTR portion of the SVA element was sufficiently conserved. Thus, true HERV-K insertion sites nominated by HERVnGoSeq were identified as follows: *HERV-K present in reference*—Using the dataset of mobile genetic elements present in the GRCh37 derived from RepBase (Bao et al., 2015) (UCSC RepeatMasker track), HERVnGoSeq nominated sites were confirmed as HERV-K if mapped within a known HERV-K ± 100 bp. *HERV-K absent from reference*—The remaining polymorphic insertion sites were determined to be HERV-K only if the insertion ± 100 bp was previously reported and confirmed with sequencing by a previous study (Dangel et al., 1994; Barbulescu et al., 1999; Mayer et al., 1999; Turner et al., 2001; Bennett et al., 2004; Hughes and Coffin, 2004; Macfarlane and Simmonds, 2004; Mamedov et al., 2004; Belshaw et al., 2005; Moyes et al., 2005; Kidd et al., 2008; Lee et al., 2012; Marchi et al., 2014; Sudmant et al., 2015; Wildschutte et al., 2016). Otherwise, they could not be distinguished from SVAs *in silico*. Additional HERV-K insertions missed by HERVnGoSeq but identified by previous studies and genotyped in the 1KG population were also included (*n* = 5) (Sudmant et al., 2015; Wildschutte et al., 2016). Prevalence of each insertion site was estimated based on either the presence/absence calls from HERVnGoSeq or from genotypes of HERV-K insertions from previous studies (*n* = 5). To ensure that polymorphic HERV-K insertions could not be explained by larger deletions, each insertion site was compared to start and end locations of deletions called in 1KG structural variant VCF. Any HERV-K insertion with a flanking deletion larger than 1,000 bp could not be reliably called polymorphic and was excluded from downstream analyses.

### Identification of SNPs associated with polymorphic HERV-K insertions

Most studies of common genetic disease-risk variants published to-date rely on SNPs, which are easy and cheap to measure compared to other structural genetic variants. These SNP studies rely on linkage disequilibrium (LD) wherein the disease-associated SNP is not necessarily the causal variant but instead tags the causal variant (outlined in Figure S2). To test our underlying hypothesis that disease-associated SNPs are, in some cases, tagging polymorphic HERV-K insertions, which are the true causal variants, we next identified SNPs associated with each HERV-K insertion and queried existing SNP:disease databases for phenotypic associations.

All HERV-K insertion sites were tested for SNP associations. For each of the 2,504 individuals in 1000 Genomes Phase 3, a binary indicator of presence/absence of the HERV-K insertion was generated via HERVnGoSeq or by recoding genotypes generated from previous publications (Sudmant et al., 2015; Wildschutte et al., 2016). Variant files for 1000 Genomes Phase 3 were downloaded from the FTP site (NCBI FTP site: ftp://ftp-trace.ncbi.nih.gov/1000genomes/ftp). After stratifying by continental population and removing related individuals (Gazal et al., 2015), all biallelic SNPs present in the 1,000 Genomes Phase 3 variant files were tested for association with the presence of each of the polymorphic HERV-K insertions using logistic regression adjusted for population stratification by including the first 6 multidimensional scaling (MDS) vectors in all models. MDS components were generated from all 1,000 Genomes variants following pruning for common SNPs (minor allele frequency MAF > 0.05) and for independence followed by random thinning to 10% of variants. All logistic regression modeling and MDS estimation were conducted in Plink 1.9 (Chang et al., 2015).

Manhattan plots were generated to visualize associations between genome-wide SNPs and polymorphic HERV-K insertions. For each of the “*taggable”* HERV-K insertion sites (i.e., those that showed a single, strong association peak in the Manhattan plot), hiSNPs were defined as all SNPs within a 1 Mb window of the insertion with *p*-value for association less than or equal to the Bonferroni adjusted *p*-value threshold for significance (0.05/Total SNPs in 1 Mb window).

### Sensitivity of hiSNP set generation

To confirm that the binary HERV-K insertion presence/absence calls made by HERVnGoSeq generated hiSNP sets similar to hiSNPs for HERV-K insertions called using a different pipeline, we identified 12 HERV-K insertions detected by HERVnGoSeq and the 1000 Genomes by Sudmant et al. (2015). For the 12 HERV-K insertion sites detected by HERVnGoSeq and genotyped by Sudmant et al., logistic regression-based SNP associations were estimated from the binary HERVnGoSeq calls and the dichotomized 1,000 Genomes genotype calls within Europeans. hiSNPs generated by HERVnGoSeq and 1,000 Genomes calls using the method described above were compared. To ensure that hiSNPs associated by logistic regression were representative of SNPs that are in LD with polymorphic HERV-K insertions, complete genotype data for the 12 overlapping HERVnGoSeq/1,000 Genomes sites were used to identify tagging SNPs via the *r*^2^ measure of LD, defined as having an *r*^2^ > 0.2.

### Expression quantitative trait loci (eQTL)

Polymorphic HERV-K insertion hiSNPs across all HERV-K insertion sites were pooled and tested for eQTL (SNP-gene expression association with *p* < 0.05 adjusted for multiple tests) enrichment against all common SNPs included in the tissue-specific Genotype-Tissue Expression (GTEx) Project Version 6 (*n* = 11,555,102) (Carithers and Moore, 2015) using a Fisher's exact test. The null hypothesis for this test is that the odds ratio ([hiSNP&eQTL]x[Not hiSNP&Not eQTL])/([Not hiSNP&eQTL]x[Not eQTL&hiSNP]) = 1. Enrichment of hiSNPs annotated as GTEx eQTLs were also calculated separately by HERV-K insertion site and tissue type using Fisher's exact tests.

### Genome-wide association and experimental factor ontology enrichment

To investigate whether hiSNPs for the polymorphic HERV-K insertion sites have established phenotypic associations, the EMBL-EBI GWAS Catalog (MacArthur et al., 2017) was queried for the presence of hiSNPs. To test for broader phenotypic enrichment across the HERV-K insertion sites, experimental factor ontology enrichment analyses were conducted for all pooled hiSNPs using the XGR online tool (http://galahad.well.ox.ac.uk:3020) with significant enrichments having a false discovery rate <0.05.

### SNP density

To determine whether the absence of hiSNPs for some HERV-K insertions sites was due to the absence of any proximal SNPs, SNP density was calculated for all insertion sites. All SNPs in the 1,000 Genomes Phase 3 dataset were counted within a 1 MB, 500 Kb, and 100 Kb window centered on each polymorphic HERV-K insertion site. The mean SNP densities for HERV-K insertions with hiSNPs was compared to HERV-K insertions with prevalence estimates from 0.2-0.8 and no hiSNPs using a Student's t-Test. Two HERV-K sites located in unlocalized contigs (chr1_gl00192_random) were excluded.

### Hotspot distance

To examine whether some HERV-K insertions lacked hiSNPs due to their proximity to recombination hotspots, we selected two recombination hotspot maps and calculated the distance between the HERV-K insertion sites and recombination hotspots. We identified two datasets mapping the genomic locations of recombination hotspots genome-wide—one using a population-average LD-based mapping method (4,697 hotspots, Genomes Project et al., 2010) and the other using ChIP-seq to identify PRDM9 binding sites among five individuals (62,110 hotspots, Pratto et al., 2014). Repeated random sampling with replacement was used to estimate the distribution of mean distances between randomly selected genomic locations and the nearest recombination hotspot. In order to compare this distribution to the distribution of distances of polymorphic HERV-Ks, a pool of random genomic locations was created wherein locations were matched to the HERV-K sites by chromosome and GC content of flanking 2 Kb region. HERV-K insertion sites on chromosome Y and unmapped contigs were excluded (*n* = 11). First, the percentage GC content of the 2 kb flanking each HERV-K was calculated using data from the UCSC gc5Base Track (Karolchik et al., 2004) in Hg19. Next, all chromosomes were divided into 2 Kb segments and percentage GC content was calculated for each. GC content for all segments was calculated to the tenth of a percent. For each HERV-K insertion, 2 Kb genomic segments were added to the sampling pool if they had identical GC content and were on the same chromosome. This resulted in an average of 444 random locations from which to sample for each HERV-K site (~187,000 total). From this pool, 172 random genomic locations (defined as the center of the 2 Kb fragment), matched 1:1 to respective HERV-K insertions, were sampled and the mean distance to nearest recombination hotspots were calculated. This sampling procedure was repeated 1,000 times. The entire process was carried out independently with each of the recombination hotspot maps.

## Results

### Polymorphic HERV-K identification

Our computational pipeline, HERVnGoSeq, nominated 1,381 putative HERV-K insertion sites among 2,504 human whole genomes from the 1000 Genomes Phase 3 population where sequencing reads partially aligned to the HERV-K113 LTR. HERV-K113 represents the one of the most recent HERV-K integration and thus is most likely to be polymorphic and to have preserved function (Turner et al., 2001). Of the 1,381 sites, 403 HERV-K insertions mapped to reference HERV-K breakpoint sequences in GRCh37/Hg19. A total of 783 putative sites mapped to reference SINE-alu-VNTR (SVA) insertions and were discarded. Of the remaining 195 non-reference putative insertions, 28 had been previously annotated and confirmed as HERV-K via sequencing in recent studies (Sudmant et al., 2015; Wildschutte et al., 2016) and the remainder, many of which are likely SVA, will require future targeted sequencing to confirm. With the rapidly increasing rate of discovery of HERV-K insertions in human genomes, we were able to collate an additional 5 HERV-K insertion sites discovered in a parallel study (Wildschutte et al., 2016), which were also genotyped in the 1000 Genomes population. In total, the 431 (403 reference and 28 non-reference) HERV-K insertions were tested for SNP associations (Table S1). The dichotomized presence/absence, rather than genotypes, of the nominated 431 HERV-K insertion sites were tested for SNP associations to reduce potential misclassification induced by poor sensitivity of calls due to low sequencing depth (~4x). An additional 16 HERV-K insertion sites identified in independent populations of diseased individuals [The Cancer Genome Atlas (Marchi et al., 2014); dbRIP (Wang et al., 2006)] were not detected by HERVnGoSeq, nor any parallel study utilizing the 1000 Genomes Project, suggesting that the diversity of HERV-K insertion sites expands beyond what is represented in the 1000 Genomes population.

### hiSNP identification

All HERV-K insertions detected in more than one individual (*n* = 431) were tested for SNP associations using logistic regression. HERVnGoSeq did not identify any HERV-K insertions occurring in 100% of 2,504 individuals; however, low coverage sequencing data likely resulted in an underestimation of the prevalence of some insertion sites. Thus it is likely that some sites with high prevalence called by HERVnGoSeq are actually fixed across populations. Genome-wide logistic regression stratified by continental population and adjusted for genetic ancestry revealed 46 polymorphic HERV-K insertion sites with significant SNP associations (hiSNPs) in at least one continental population after correction for multiple testing (Table 1, Table S2, Figures S3–S50). Figure 1 shows a Manhattan plot of the genome-wide SNP association results for a polymorphic HERV-K insertion located on chromosome 1.

**Table 1:** 46 Polymorphic HERV-K insertions with hiSNPs.

**Reference HERV-K insertions**			**Non-reference HERV-K insertions**		
**Coordinate GRCh37/hg19**	**Prev^b^**	**Average hiSNP Count^c^**	**Coordinate GRCh37/hg19**	**Prev^b^**	**Average hiSNP Count^c^**
chr3:14132679	0.96	453	chr1:106015875	0.04	436
chr3:125609298^a^	0.54	194	chr1:111802591	0.59	234
chr3:129776131^a^	0.47	203	chr1:223578304	0.01	206
chr3:195654395	0.96	205	chr4:9603240	0.67	969
chr4:120263688	0.68	1416	chr4:9981605	0.02	650
chr5:8937848	0.83	345	chr5:4537604	0.01	176
chr6:32505702^a^	0.13	1817	chr5:64388440	0.07	226
chr6:32746812^a^	0.08	222	chr5:80442266	0.05	49
chr7:16237347^a^	0.81	259	chr6:32648036	0.35	4965
chr7:158029477	0.28	239	chr6:161270899	0.84	576
chr8:7355392	0.14	108	chr7:158773385	0.01	102
chr8:18651453	0.52	199	chr11:60449890	0.07	292
chr8:37050885	0.32	125	chr12:44313657	0.27	593
chr10:135355522	0.18	155	chr12:124066477	0.13	444
chr11:71478951^a^	0.82	379	chr13:90743183	0.12	292
chr11:71875417	0.88	117	chr15:63374594	0.68	238
chr12:55727210	0.76	385	chr19:21841536	0.20	613
chr14:20552746^a^	0.30	144	chr19:22414379	0.43	993
chr17:44361947^a^	0.17	2300	chr19:22457244	0.01	907
chr19:386675^a^	0.10	19	chr19:29855781	0.55	536
chr19:52924209^a^	0.39	37	chr19:57996939	0.02	191
chr20:25215439^a^	0.84	93	chr20:12402387	0.03	271
chr21:15654234^a^	0.65	19	chrX:93606603	0.02	186

a Not previously recognized as polymorphic.

b Prev, prevalence averaged across 5 super populations.

c *Counts averaged across 5 super populations*.

**Figure 1:**
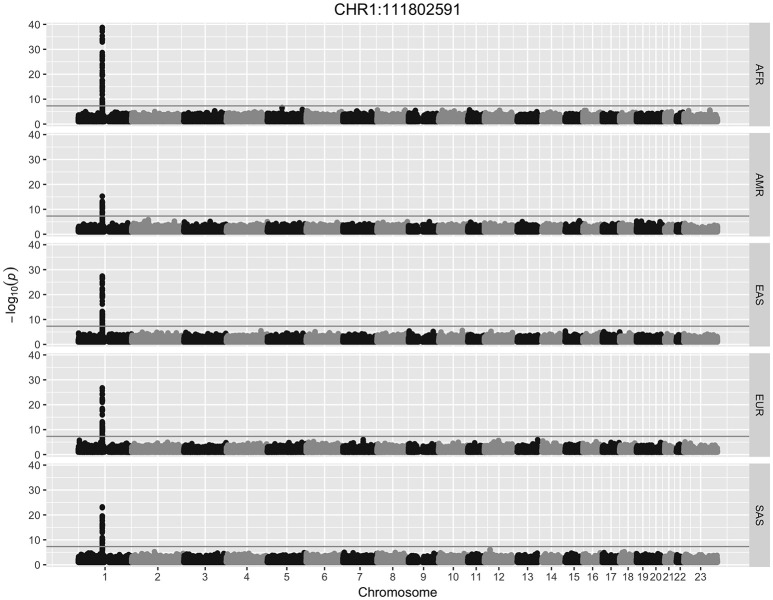
Manhattan plot. Results from a genome-wide association study for a polymorphic HERV-K insertion discovered with HERVnGoSeq at chr1:111802591. The gray horizontal line represents the threshold for genome-wide significance of 5 × 10^−8^. Logistic regression was performed separately within each of the five super populations in the 1,000 Genomes. AFR, African; AMR, Ad Mixed American; EAS, East Asian; EUR, European; SAS, South Asian.

**Figure 2:**
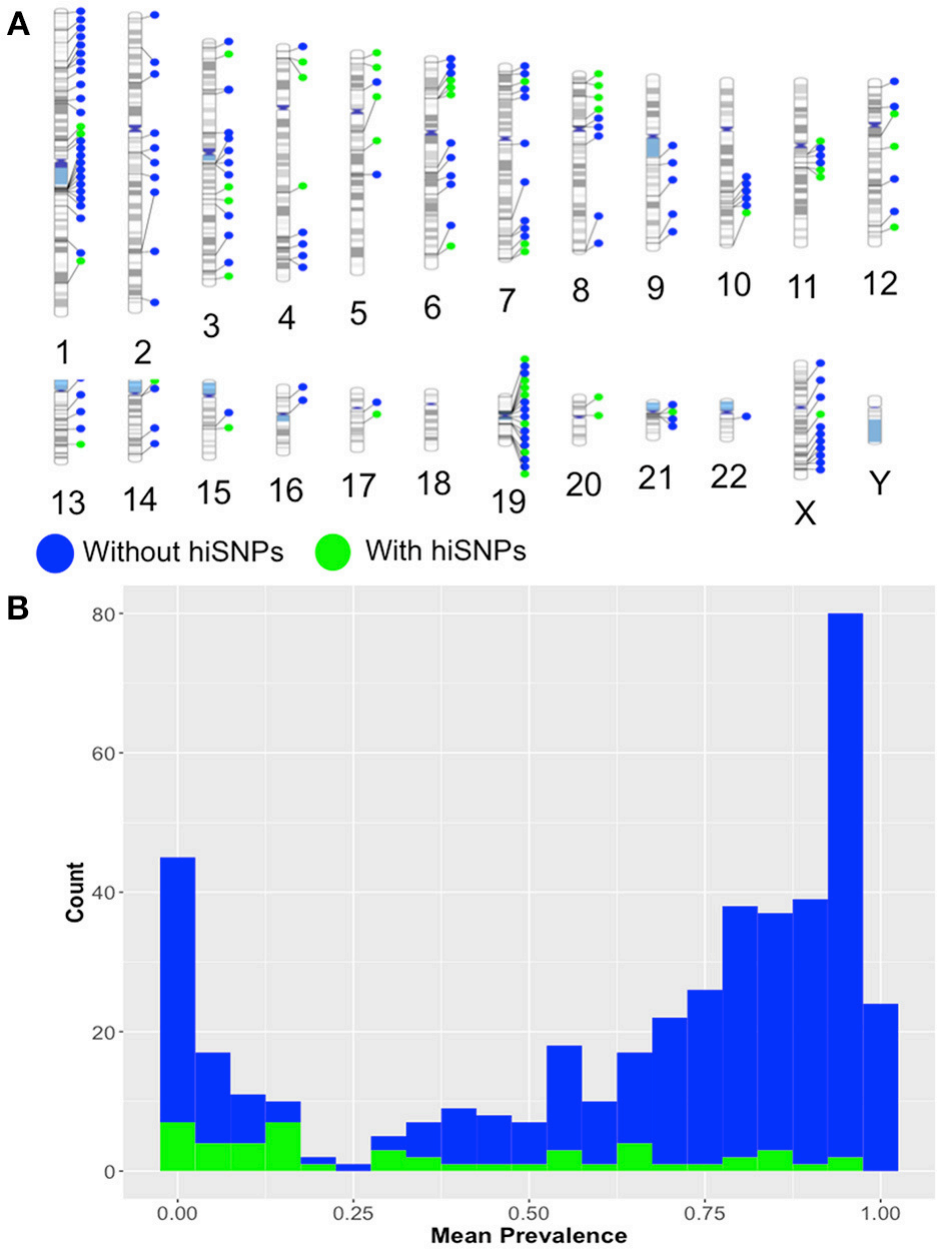
**(A)** Ideogram. Relative genomic locations of HERV-K insertion locations with (green) and without (blue) hiSNPs identified via HERVnGoSeq. **(B)** Histogram. Frequency distribution of identified HERV-K insertion prevalences for insertions with (green) and without (blue) hiSNPs.

Of these, 13 were not previously known to be polymorphic. The majority of the remaining 385 HERV-K insertion sites with no identifiable hiSNPs were rare (prevalence < 0.2, *n* = 48), singletons (*n* = 14), or common and potentially fixed (prevalence > 0.8, *n* = 190), which may explain the lack of association with neighboring SNPs. However, 129 HERV-K sites appear to be common and unfixed (prevalence restricted to 0.2–0.8) yet have no hiSNPs and thus could not be evaluated for phenotype enrichment in this study (Figure 2).

Among the 46 HERV-K insertion sites with strong SNP associations, hiSNP sets were selected within a 1-megabase window to ensure that no strongly associated SNPs were excluded arbitrarily due to genomic distance. The median number of hiSNPs associated with each of these 46 HERV-K insertions was 279 (Table 1). A complete summary including odds ratio and *p*-values for all hiSNP-HERV-K associations can be found in Table S3.

To ensure that the selected hiSNPs represented SNPs in true linkage disequilibrium with the HERV-K insertions, a subset of 12 sites that were identified by HERVnGoSeq and also genotyped in an independent study of structural variation in the 1,000 Genomes Project (Sudmant et al., 2015) were selected for sensitivity analyses. hiSNP sets selected by HERVnGoSeq were compared to hiSNP sets derived from logistic regression with outcome being dichotomized genotypes of insertions called by the 1,000 Genomes, and LD via *r*^2^ values based on maximum likelihood phasing. HERVnGoSeq logistic regression-based hiSNP sets consistently nominated the greatest number of hiSNPs across the 12 sites and, for 10 sites, >75% of HERVnGoSeq hiSNPs were also hiSNPs derived from the genotyped insertions via logistic regression or *r*^2^ (Table S2, Figures S51–S63).

### Tissue-specific differential gene expression and disease enrichment

**Figure 3:**
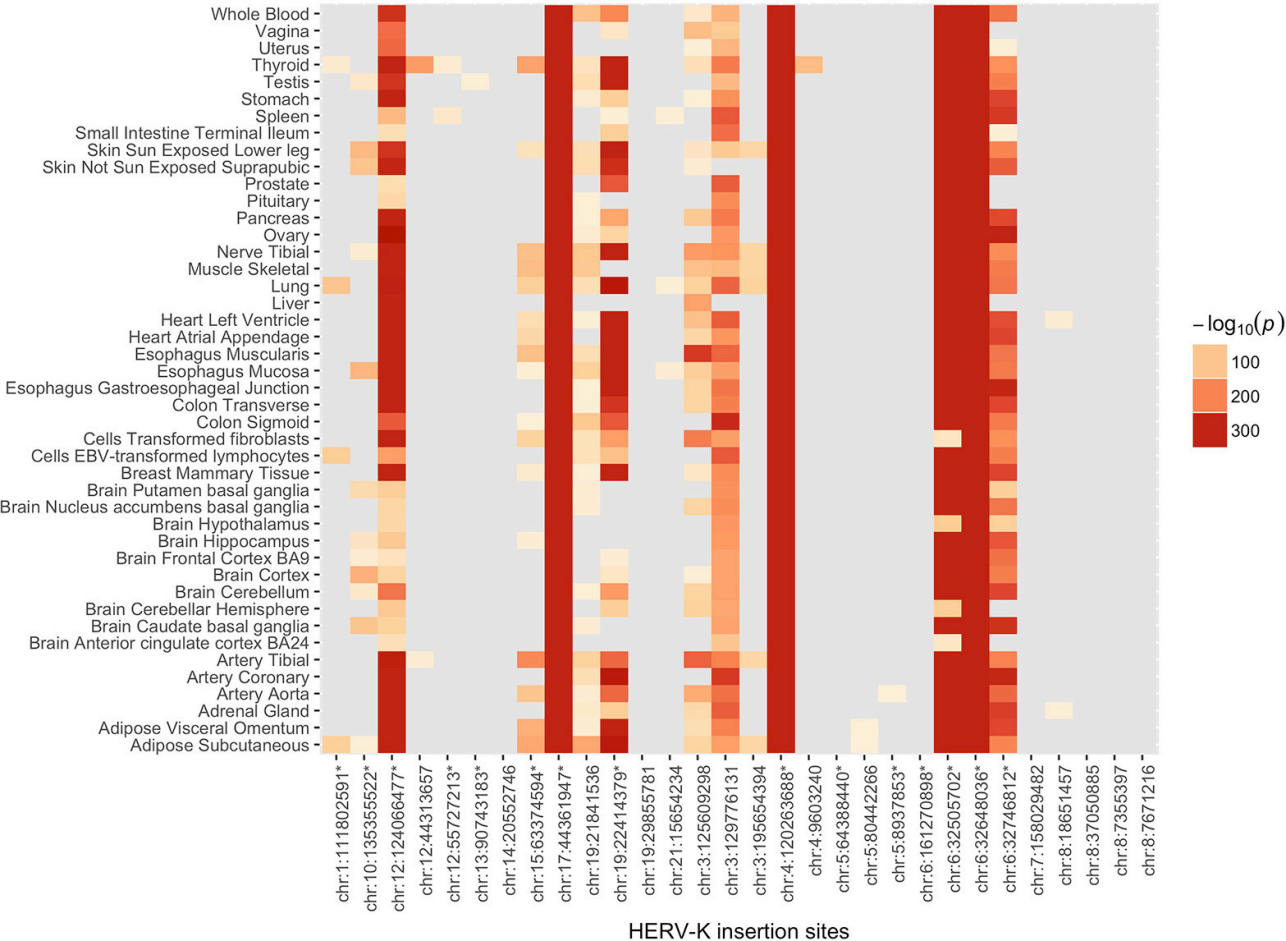
Heat map of log-transformed *p*-values for enrichment of hiSNPs that are eQTLs. Includes the 30 polymorphic HERV-K insertion sites with strong SNP associations in the European continental population. Enrichment of SNPs that are both hiSNPs and eQTLs was determined using Fisher's exact test. Results are stratified across 44 human tissue types.

The Genotype-Tissue expression (GTEx) project provides expression quantitative trait loci (eQTL) analysis results from genotype and gene expression data derived from 449 individuals across 44 human tissues (Carithers and Moore, 2015). We tested whether the HERVnGoSeq derived hiSNPs for each of the HERV-K insertion sites were enriched for eQTLs based on these data. Because the GTEx individuals are ~85% white, we restricted these analyses to the hiSNP sets identified among the 30 polymorphic HERV-K insertion sites in the European continental population (Table S1) to reduce confounding by population stratification. We observed enrichment (*p* < 0.05) of hiSNPs for eQTLs in at least one tissue type for 22 of the 30 sites by Fisher's exact test (Figure 3). HERV-K insertion sites contributed the most eQTL associations to subcutaneous adipose tissue and thyroid tissue with 15 HERV-K sites each. Fifteen of the sites with hiSNP sets enriched for eQTLs also include SNPs associated with disease by GWAS (Figure 3). The number of genes for which individual HERV-K insertion hiSNPs served as eQTLs ranged from 1 to 75 (Table S4). Often in the instances where a large number of genes were affected, the HERV-K insertion and genes occurred on blocks of extended LD (i.e., the major histocompatibility complex).

**Figure 4:**
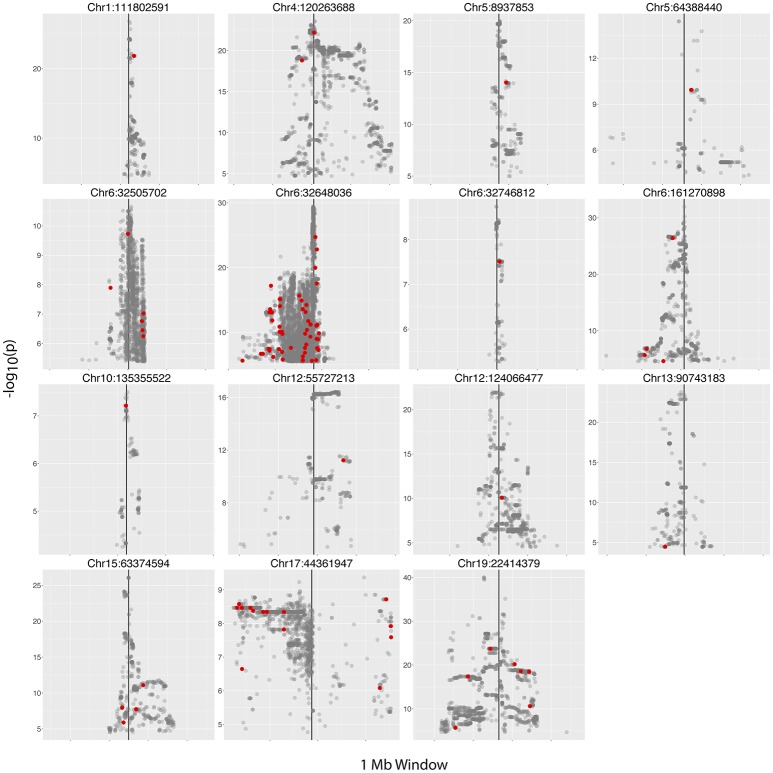
Manhattan plots. Plots of hiSNP sets for 15 HERV-K insertion sites among the 30 polymorphic HERV-K insertion sites with strong SNP associations in the European continental population. Vertical black lines denote HERV-K insertion locations. hiSNPs that are annotated in the NHGRI-EBI GWAS Catalog are represented by red points.

We next examined the 30 hiSNP sets identified in the European continental population for annotation in the NHGRI-EBI GWAS Catalog. Half of the HERV-K insertion sites had at least one hiSNP with a genome-wide significant association with a disease phenotype (Figure 4). In total, European polymorphic HERV-K insertions are associated with 80 human phenotypes (Table 2).

**Table 2:** HERV-K insertions with hiSNPs annotated in NHGRI-EBI GWAS Catalog and associated traits.

**HERV-K insertion**	**Disease/Trait**	**SNP ID^a^**	**PMID^b^**
**Non-MHC HERV-K insertion sites**			
chr1:111802591	Interferon alpha levels in systemic lupus erythematosus	rs7411387^***^ (OR = 1.61)	25338677
chr4:120263688	Corneal astigmatism	rs11098499^**^ (β = 0.048)	23322567
	Educational attainment	rs10028773^***^ (β = 0.02)	25201988
chr5:64388440	Schizophrenia	rs17206232^*^ (β = 0.135)	23212062
chr5:8937853	Obesity-related traits	rs11134338^**^ (β = 0.03)	23251661
chr6:161270898	Lipoprotein (a) - cholesterol levels	rs1620921^***^	25575512
	Lipoprotein (a) levels	rs9355814 (β = 0.33), rs783147 (β = 0.3)	26377243, 21900290
	Protein quantitative trait loci	rs7770628	18464913
chr10:135355522	Obesity-related traits	rs2249694 (β = 0.03)	23251661
chr12:124066477	Pubertal anthropometrics	rs786425^*^ (β = 0.06)	23449627
chr12:55727213	Contrast sensitivity	rs12230513^*^ (β = 2.69)	24152035
chr13:90743183	Longevity	rs2882281	20834067
chr15:63374594	Blood metabolite levels	rs1472631 (β = 0.037)	24816252
	Mean platelet volume	rs11071720 (β = 0.061)	19820697
	Metabolic traits	rs2652822 (β = 0.085)	21886157
	Platelet count	rs3809566 (β = 2.43x10-9)	22139419
	Social communication problems	rs17828380 (β = 0.18)	24564958
chr17:44361947	Bone mineral density	rs1864325 (β = 0.04)	22504420
	Corticobasal degeneration	rs12185268 (OR = 1.3)	26077951
	Epithelial ovarian cancer	rs183211 (OR = 1.11)	25581431
	Idiopathic pulmonary fibrosis	rs17690703 (OR = 1.43)	24429156
	Interstitial lung disease	rs1981997 (OR = 1.41)	23583980
	Intracranial volume	rs9303525 (β = 14.9)	22504418
	Male-pattern baldness	rs12373124 (OR = 1.33)	22693459
	Ovarian cancer in BRCA1 mutation carriers	rs183211 (OR = 1.25)	23544013
	Parkinson's disease	rs12185268 (OR = 3.46), rs17577094 (OR = 1.56), rs17649553 (OR = 1.3), rs183211, rs199515 (OR = 1.32), rs199533 (OR = 1.28), rs415430, rs8070723 (OR = 1.3)	21738487, 24842889, 25064009, 21812969, 22451204, 20711177, 21812969, 21044948
	Progressive supranuclear palsy	rs8070723 (OR = 5.11)	21685912
	Subcortical brain region volumes	rs17689882 (β = 13460.47), rs8072451 (β = 14489.99)	25607358, 25607350
chr19:22414379	Body mass index (change over time)	rs8105895^**^ (β = 1.26)	25378290
	Chagas cardiomyopathy in Tripanosoma cruzi seropositivity	rs2262909	24324551
	Dental caries	rs10404998^**^, rs1865075^**^, rs931608^*^	23259602, 23259602, 23064961
	Response to statin therapy (LDL-C)	rs931608^*^ (β = 4.2)	22331829
	Telomere length	rs1975174^***^ (β = 0.05), rs412658^***^ (β = 0.0497)	20421499, 23001564
**MHC Herv-K insertion sites**			
chr6:32505702	Cervical cancer	rs9272143 (OR = 1.49)	28806749
	Hepatitis B vaccine response	rs3135363 (OR = 1.53)	24282030
	Hepatitis C induced liver cirrhosis	rs3135363 (OR = 1.37)	23321320
	Hepatocellular carcinoma	rs9272105 (OR = 1.28)	22807686
	Leishmaniasis (visceral)	rs9271858 (OR = 1.41)	23291585
	Response to interferon beta therapy	rs9272105 (β = 0.026)	21502966
	Rheumatoid arthritis	rs2157337^*^	21156761
	Systemic sclerosis	rs3129763 (OR = 1.65)	21779181
chr6:32648036	Alzheimer's disease (late onset)	rs9271192 (OR = 1.1)	24162737
	Antinuclear antibody levels	rs2395185^**^ (β = 0.25)	25186300
	Arthritis (juvenile idiopathic)	rs2395148 (OR = 5.37)	18576341
	Asthma	rs3117098 (OR = 1.16), rs7775228^***^ (OR = 1.17), rs9268516^*^ (OR = 1.15), rs9272346^*^ (OR = 1.16)	21804548, 21804548, 23028483, 29273806
	Asthma and hay fever	rs9273373^*^ (OR = 1.24)	24388013
	Atopic dermatitis	rs9469099 (OR = 1.61)	23042114
	Chronic lymphocytic leukemia	rs674313 (OR = 1.69)	21131588
	Circulating myeloperoxidase levels (serum)	rs3134931 (β = 0.05)	23620142
	Cystic fibrosis severity	rs9268905^**^	21602797
	Dementia and core Alzheimer's disease neuropathologic changes	rs7453498^*^ (β = 0.814)	25188341
	Epstein-Barr virus immune response (EBNA-1)	rs477515^**^ (β = 0.28)	23326239
	Follicular lymphoma	rs12195582 (OR = 1.78), rs2647012^*^ (OR = 1.56)	25279986, 21533074
	Hepatitis B vaccine response	rs477515^**^ (OR = 2.05)	24282030
	Hepatitis C induced liver cirrhosis	rs3817963^*^ (OR = 1.3)	23321320
	Hepatocellular carcinoma (hepatitis B virus related)	rs9275319^**^ (OR = 1.49)	23242368
	Hodgkin's lymphoma	rs2395185^**^ (OR = 1.82), rs6903608^*^ (OR = 1.64)	22286212, 24920014
	Hypothyroidism	rs3129720 (OR = 1.16)	22493691
	IgA nephropathy	rs2856717^*^ (OR = 1.27), rs660895^*^ (OR = 1.29), rs7763262 (OR = 1.41), rs9275596^*^ (OR = 1.44)	25305756, 26028593, 25305756, 25305756
	IgE grass sensitization	rs7775228^***^ (OR = 1.33)	22036096
	Inflammatory bowel disease	rs477515^**^ (OR = 1.38)	18758464
	Leprosy	rs9271100 (OR = 1.68)	25642632
	Lung adenocarcinoma	rs3817963^*^ (OR = 1.18)	22797724
	Lung cancer	rs2395185^**^	23143601
	Lupus nephritis in systemic lupus erythematosus	rs2647012^*^ (OR = 1.52)	24925725
	Lymphoma	rs2647045^***^ (OR = 1.69), rs2647046^*^ (OR = 1.25), rs9268853^**^ (OR = 1.56)	23349640, 23349640, 23349640
	Multiple sclerosis (OCB status)	rs3129720 (OR = 1.91), rs3817963^*^ (OR = 1.61), rs9275563	23472185, 23472185, 23472185
	Narcolepsy (age of onset)	rs7744020^*^ (β = 1.9)	24204295
	Nasopharyngeal carcinoma	rs28421666^*^ (OR = 1.49)	20512145
	Nephropathy	rs9275596^*^ (OR = 1.59)	21399633
	Neurofibrillary tangles	rs34075049^**^ (β = 0.59)	25188341
	Parkinson's disease	rs2395163 (OR = 1.24), rs9275326 (OR = 1.18)	22451204, 28892059
	Peanut allergy	rs9275596^*^ (OR = 1.7)	25710614
	Primary biliary cirrhosis	rs7774434^***^ (OR = 1.57)	22961000
	Rheumatoid arthritis	rs12194148^*^, rs12525220 (OR = 2.61), rs660895^*^ (OR = 3.62), rs7748270 (OR = 1.74), rs9268839^*^ (OR = 2.28), rs9275406 (OR = 2.1)	21156761, 24782177,17804836, 24782177, 24390342, 23918589
	Sarcoidosis	rs2076530	22936702
	Schizophrenia	rs9274623 (OR = 1.14)	26198764
	Sjogren's syndrome	rs9271588	24097066
	Systemic lupus erythematosus	rs2647012^*^ (OR = 1.38), rs9271100 (OR = 1.9)	21408207, 19838193
	Systemic sclerosis	rs9275390 (OR = 2.38)	21779181
	Type 1 diabetes	rs9272346^*^ (OR = 5.49)	17554300
	Ulcerative colitis	rs1063355^*^ (OR = 1.43), rs2395185^**^ (OR = 1.49), rs6927022^*^ (OR = 1.44), rs9268480^*^ (OR = 1.82), rs9268853^**^ (OR = 1.37), rs9268877^**^ (OR = 1.45), rs9268923^**^ (OR = 1.45)	24837172, 20228799, 23128233, 19915573, 23511034, 18836448, 20228798
	Vitiligo	rs3806156^**^ (OR = 1.42)	20410501
	Waist-hip ratio	rs2076529 (β = 0.02)	20935629
	Waist-to-hip ratio adjusted for body mass index	rs7759742^*^ (β = 0.02)	28443625
chr6:32746812	Kawasaki disease	rs2857151 (OR = 1.47)	22446962

a ^*^ denotes strength of HERV-K::hiSNP association, all p-values are < bonferroni threshold

* p < 1.0e−10,

** p < 1.0e−15,

*** p < 1.0e−20. OR/β value from SNP: Phenotype association.

b *PMID, Pubmed ID for original research article in GWAS Catalog describing the SNP-phenotype association*.

Experimental factor ontology enrichment analysis suggests that polymorphic HERV-K insertions broadly associate with neurologic and immunologic disease phenotypes, including traits related to intracranial volume (FDR 4.40E-08), Parkinson's disease (FDR 1.80E-09), and autoimmune diseases (FDR 1.80E-09) (Table S5, Figure S64).

### Analyses of “untaggable” HERV-K insertion sites

The majority of polymorphic HERV-K insertions identified via HERVnGoSeq were not associated with any nearby SNPs (*n* = 129) and could not be evaluated for existing phenotypic associations in this study. Fifty-three of these HERV-K insertions (estimated mean prevalence: 45.6%, range: <1–79.7%), occur within genes (Table S6).

The distribution of polymorphic HERV-K in specific chromosomal regions (ex: telomeres, centromeres) did not explain the lack of strong hiSNP associations in the 129 identified polymorphic sites (Figure 2A). However, we suspected that they might differ from sites with strong SNP associations in two respects—proximal SNP density and distance to nearest recombination hotspots, which both effect neighboring patterns of LD (Ardlie et al., 2002; Ke et al., 2004). We found significantly lower SNP densities in the areas around HERV-K insertion sites without hiSNPs (mean 2813.2) than the areas around HERV-K insertions with hiSNPs (mean 3392.7, difference in means: 579.5 SNPs, *p* = 0.0007). However, the presence of SNPs flanking the 129 HERV-K insertions without hiSNPs suggests that SNP density is not a sufficient determining factor.

ERVs can be involved in homologous and non-homologous recombination events (Campbell et al., 2014). Some are enriched for PRDM9 binding motifs (Campbell et al., 2014) and elimination through recombination is a major mechanism by which ERV sequences are removed from the human genome (Katzourakis et al., 2007). We investigated whether recombination at HERV-K insertion sites explained the lack of hiSNP associations with these 129 HERV-K insertions by measuring their proximity to known recombination hotspots.

**Figure 5:**
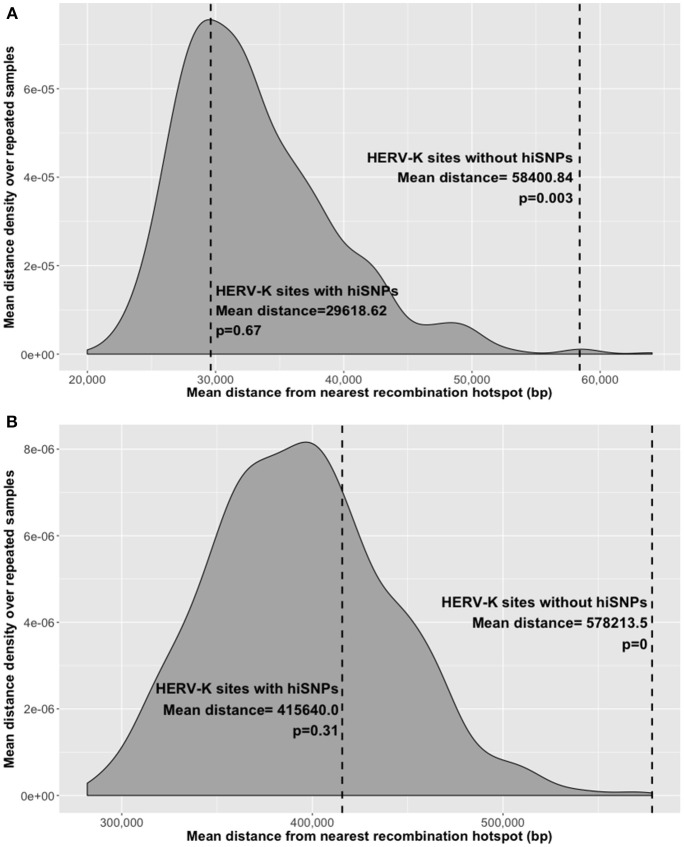
Mean distance to nearest recombination hotspot. Distances indicated for polymorphic HERV-K insertions with and without hiSNPs (dashed lines) and the distribution of mean distances of random genomic locations matched to HERV-K insertions on proximal GC content and chromosome. Distributions were derived from 1000 repeated random samples with replacement. **(A)** Distances from nearest ChIP-seq-based recombination hotspot, **(B)** Distances from nearest LD-based recombination hotspot.

HERV-K insertion sites without hiSNP associations were farther on average from mapped recombination hotspots than their hiSNP-associated counterparts (222.2 kb difference in distance to LD hotspots, *p* = 0.04, and 40.1 kb difference in distance to ChIP-seq hotspots, *p* = 0.008). To determine whether the distance of polymorphic HERV-K insertions from recombination hotspots was greater than expected by chance, we compared the mean distance of these 172 polymorphic HERV-K insertions (46 with hiSNPs and 129 without) to the distances from hotspots of repeated random samples of 172 genomic locations. To mitigate potential confounding, random genomic locations were matched to HERV-K insertions sites on chromosome and flanking 2 kb GC content. Polymorphic HERV-K without hiSNPs were farther from recombination hotspots than randomly selected genomic locations (*p* < 0.005), whereas insertions with hiSNPs were not farther (or closer) to recombination hotspots than expected by chance (Figure 5).

## Discussion

This study shows that polymorphic HERV-K insertions occur in regions of the genome enriched for phenotypic function and, furthermore, that these insertion variants co-occur with established disease-risk variants, providing previously-untested candidates for the functional elements underlying the heritability of numerous complex diseases. Using our computational pipeline, HERVnGoSeq, and the diverse 1,000 Genomes population, we confirmed the presence of 33 known polymorphic HERV-K insertions and identified an additional 13 confirmed sites via strong SNP associations not previously recognized as polymorphic. Of the total 46 HERV-K insertions under investigation, 22 have hiSNP sets enriched for eQTLs and 15 contained disease-associated SNPs identified in prior GWAS.

The collective evidence put forth by annotated hiSNPs supports a role for HERV-K insertions in inducing phenotypic effects. There is previous evidence that polymorphic HERV-K insertions may affect brain function. Previous studies have established links between HERV-K and amyotrophic lateral sclerosis (Alfahad and Nath, 2013), HIV-associated dementia (Garrison et al., 2007), and Schizophrenia (Sekar et al., 2016). Our results further support these links and provide specific candidate polymorphisms that may explain these observations. We found two polymorphic HERV-K insertions (chr5:64388440 and chr6:32648036) whose hiSNP sets include a GWAS hit for schizophrenia. The association of the hiSNP at the insertion chr6:32648036 has already been attributed to the presence of a polymorphic HERV-K at the complement component 4 (C4) locus, resulting in altered expression at *C4A* and *C4B*, which we also observed in our eQTL enrichment results (Table S4) (Sekar et al., 2016). The hiSNP set at the second site at chr5:64388440 contains a SNP that is associated with schizophrenia symptoms relating to hallucination, delusion, and paranoia (Fanous et al., 2012) and both the SNP and the HERV-K are located directly upstream of and serve as eQTLs for *ADAMTS6*, a gene among a family that experimentally induces neurite growth in cultured neurons (Hamel et al., 2008). One of the most strongly associated hiSNPs for another HERV-K at chr4:120263688 was previously identified in a GWAS examining the genetics of cognitive performance using proxy-phenotypes (Rietveld et al., 2014). Experimental factor ontology enrichment analysis of hiSNPs also suggested a largely neurological phenotypic effect of polymorphic HERV-K wherein Parkinson's disease, intracranial volume, and temporal arteritis were the seconds, third, and fourth most significantly enriched terms, respectively. Enrichment analysis also suggests that HERV-K insertion sites may have a functional role in autoimmune diseases. The role of polymorphic HERV-K in immunity, particularly insertions within the HLA, is difficult to delineate. While no specific associations have been established between HERV-K and autoimmunity, strong evidence suggests a link between multiple sclerosis and expression of HERV-W (Schmitt et al., 2013); and the role of ERVs in autoimmunity has been long suspected, but its study has been hindered by technological limitations. With the increasing availability of next-generation sequencing data and computational methods like HERVnGoSeq, the time is ripe for a thorough investigation of polymorphic HERVs in autoimmune disease.

HERV-K expression has frequently been noted in human cancers and has also been of interest as an etiologic factor. We found hiSNP associations with Hepatocellular carcinoma (HCC) tagging two polymorphic HERV-K insertions. Recent studies identified an increase in HERV-K expression in HCC vs. normal tissue (Ma et al., 2016) and also discovered that HCC tumor mutations are frequently caused by APOBEC enzymes, a component of the human innate immune system primarily active against ERVs (Chiu and Greene, 2008). As such, the role of polymorphic HERV-K in interaction with hepatitis viruses and HCC appears warranted.

The HERV-K LTR is known to contain enhancer elements and thus the degree to which hiSNPs were enriched for eQTLs was not unexpected. An advantage of using the GTEx database is the ability to determine tissue-specific eQTL activity, which can help discern the phenotypic effects of polymorphic HERV-K. For example, we observe that the hiSNPs for a polymorphic HERV-K at chr19:22414379 are enriched for eQTLs in adipose tissue, suggesting that the insertion could affect fat storage. Indeed, the hiSNP set for this insertion also contains a GWAS SNP associated with changes in body mass index over time (McQueen et al., 2015).

It is possible that the HERV-K with hiSNP eQTL enrichment is not itself the variant altering *cis*-gene expression, particularly in cases where the HERV is inserted on a large LD block, for example within the HLA region (chr6:32505702, chr6:32648036, chr6:32746812). Often these regions are not fully explored in functional follow-up studies due to their complexity. Since HERV-K LTRs contain functional elements, they serve as strong candidates for eQTLs regardless of regional complexity, warranting additional studies. It is also possible that the observed relationships between eQTLs and HERV-K insertions differ across populations. However, the GTEx consortium primarily consists of samples derived from european individuals and thus the relationship between polymorphic HERV-K and gene expression can currently only be inferred in this population.

We could not leverage SNP annotations to illuminate the function of the majority of polymorphic HERV-K insertions because they did not associate with any neighboring SNPs. In addition to the 46 HERV-K insertions with hiSNPs, we also nominated 129 reference HERV-K insertions that are likely polymorphic and that may have yet-undetected phenotypic associations. It is possible that some of the lower prevalence sites without hiSNPs are fixed and were called polymorphic only because of poor detection sensitivity of HERVnGoSeq. However, this seems unlikely, as our prevalence estimates for previously recognized non-reference polymorphic HERV-K were usually within ~10% of previous studies' estimates (Figure S65). Pairwise correlations of SNPs directly adjacent to these HERV-K insertions suggest that there is LD in these regions (data not shown), but that the HERV-K insertions are “dark variants” that are not correlated with proximal SNPs. One potential explanation why the majority of polymorphic HERV-K insertions fail to have strong SNP associations is the greater than expected distance to the nearest recombination hotspot. Patterns of LD are known to strengthen the closer variants are to a hotspot, with complete loss of LD within the hotspot itself. The observed decay of HERV:SNP LD farther from hotspots requires further study. It is also possible that there is hotspot activity near or within these HERVs that were not identified and included in the two hot spot maps used for this study. Breakdown of LD patterns surrounding these HERV-K insertions may also be explained by other mechanisms that could not be investigated in the present study, including frequent sporadic non-allelic homologous recombination events, evolutionarily recent integration, off-target mutagenic activity of HERV-K repressors such as APOBEC enzymes, or hypermethylation resulting in sporadic deamination of methylated cytosines.

In our survey of phenotypic associations with polymorphic HERV-K insertions, the greatest limitation was the poor sensitivity of detection of HERV-K due to the low coverage of the sequencing data available for the 1,000 Genomes population. Consequently, we were not able to call genotypes or estimate prevalence with high precision. Similar pipelines that have used these data to genotype mobile genetic elements often include an imputation step. Our observation, that a significant number of HERV-K insertions lack SNP associations, likely impedes the ability and reliability of imputation-based methods for genotyping these polymorphisms. This may also explain why so few members of the HERV-K family have been recognized as polymorphic. We anticipate that the accuracy of genotyping HERV-K insertions will greatly increase with higher coverage sequencing data.

Polymorphic HERV-K elements are associated with the germ-line risk of myriad phenotypes. While this study provides a starting point for further investigation, disease-specific epidemiologic and functional studies are needed to elucidate the role of specific polymorphic HERV-K insertions in complex diseases.

## Ethics statement

This study was carried out in accordance with the recommendations of our institutional review board at both the University of California, San Francisco and University of Nevada, Reno. The protocol was approved by the UCSF and UNR IRB (IRB# 1178149-1, 992935-1). Only previously collected data was used in this analysis where original investigators required all subjects gave written informed consent in accordance with the Declaration of Helsinki.

## Author contributions

SF and AW conceived of the study, conducted and assisted in analysis, and wrote the paper. GW developed bioinformatic pipelines, managed data, and assisted in manuscript preparation. LB, AdS, KW, CM, JC, JW assisted in analyses, provided technical expertise and aided in manuscript preparation and writing.

### Conflict of interest statement

The authors declare that the research was conducted in the absence of any commercial or financial relationships that could be construed as a potential conflict of interest.

## Abbreviations

ERV: endogenous retrovirus
HERV-K: human endogenous retrovirus-K
SNP: single nucleotide polymorphism
hiSNP: HERV-K insertion associated single nucleotide polymorphism
SVA: sine-VNTR-alu element
eQTL: expression quantitative trait loci
GWAS: genome-wide association study
WGS: whole genome sequencing
MDS: multi-dimensional scaling
LD: linkage disequilibrium.
