# Supplementary material for: To ERV Is Human: A Phenotype-Wide Scan Linking Polymorphic Human Endogenous Retrovirus-K Insertions to Complex Phenotypes
Source: Front Genet. 2018 Aug 14;9:298. doi: 10.3389/fgene.2018.00298 (PMC6102640; doi:10.3389/fgene.2018.00298)
Supplement: Supplementary file 1 [file Table_1.pdf]

Table S1: Polymorphic HERV-K insertions identified with HERVnGoSeq, prevalence, and hiSNP set size, stratified by continental population

Polymorphic Reference HERV-K Insertions	African		Amerindian		European		East Asian		South Asian	
**Coordinate GRCh37/hg19**	**Prevalence**	**hiSNP Count**	**Prevalence**	**hiSNP Count**	**Prevalence**	**hiSNP Count**	**Prevalence**	**hiSNP Count**	**Prevalence**	**hiSNP Count**
chr3:14132679	0.87	453	0.97	-	0.97	-	0.98	-	1.00	-
chr3:125609298*	0.55	307	0.49	36	0.52	268	0.54	164	0.61	-
chr3:129776131*	0.62	15	0.39	186	0.36	273	0.50	283	0.49	258
chr3:195654395*	0.99	-	0.93	236	0.96	105	0.95	275	0.98	-
chr4:120263688*	0.66	1593	0.63	1253	0.67	1431	0.62	1465	0.79	1337
chr5:8937848	0.82	306	0.81	337	0.73	389	0.91	-	0.88	348
chr6:32505702*, chr6_cox_hap2:3953713, chr6_qbl_hap6:3740216	0.21	2407	0.10	1265	0.11	1862	0.12	1953	0.11	1600
chr6:32746812*, chr6_ssto_hap7:4177515	0.09	-	0.09	-	0.09	222	0.08	-	0.07	-
chr7:16237347*	0.84	130	0.87	-	0.83	-	0.65	387	0.84	-
chr7:158029477	0.19	251	0.29	193	0.33	446	0.33	146	0.28	159
chr8:7355392	0.32	44	0.11	125	0.08	204	0.04	-	0.12	57
chr8:18651453	0.65	203	0.42	116	0.26	238	0.76	263	0.51	175
chr8:37050885	0.48	182	0.22	110	0.18	150	0.33	110	0.40	75
chr10:135355522	0.06	88	0.23	107	0.22	132	0.17	292	0.22	-
chr11:71478951*	0.88	-	0.81	-	0.78	-	0.77	379	0.87	-
chr11:71875417	0.90	93	0.88	-	0.88	-	0.82	187	0.91	72
chr12:55727210	0.68	279	0.84	317	0.83	365	0.60	618	0.83	348
chr14:20552746*	0.49	72	0.24	131	0.18	205	0.29	218	0.30	96
chr17:44361947	0.11	-	0.19	-	0.23	2300	0.11	-	0.21	-
chr19:386675*	0.18	18	0.05	18	0.05	-	0.15	22	0.09	-
chr19:52924209*	0.50	33	0.36	-	0.32	-	0.34	-	0.42	41
chr20:25215439*	0.85	93	0.82	-	0.82	-	0.83	-	0.88	-
chr21:15654234*	0.70	-	0.63	-	0.62	19	0.60	-	0.69	-
**Polymorphic Non-Reference HERV-K Insertions**										
chr1:106015875	0.19	436	0.01	-	0.00	-	0.00	-	0.00	-
chr1:111802592	0.63	193	0.64	173	0.52	268	0.58	331	0.58	206
chr1:223578304	0.06	206	0.01	-	0.00	-	0.00	-	0.00	-
chr4:9603240	0.63	901	0.66	634	0.83	422	0.57	1306	0.66	1580
chr4:9981605	0.08	650	0.00	-	0.00	-	0.00	-	0.00	-
chr5:4537604	0.04	176	0.00	-	0.00	-	0.00	-	0.00	-
chr5:64388440	0.17	400	0.07	94	0.06	184	0.02	-	0.02	-
chr5:80442266	0.03	24	0.08	60	0.08	88	0.00	-	0.05	24
chr6:32648036, chr6_mann_hap4:4099133, chr6_ssto_hap7:4073239	0.26	5287	0.42	3282	0.27	6113	0.33	3938	0.45	6206
chr6:161270899	0.81	446	0.83	472	0.67	874	0.98	-	0.89	513
chr7:158773385	0.04	102	0.00	-	0.00	-	0.00	-	0.00	-
chr11:60449890	0.03	8	0.22	541	0.00	-	0.09	328	0.01	-
chr12:44313657	0.48	540	0.18	507	0.11	451	0.36	887	0.23	580
chr12:124066477	0.32	571	0.09	382	0.12	665	0.03	17	0.07	583
chr13:90743183	0.34	591	0.10	155	0.11	301	0.01	-	0.04	120
chr15:63374594	0.64	224	0.56	156	0.63	481	0.86	-	0.71	91
chr19:21841536	0.32	717	0.08	337	0.08	248	0.24	1342	0.29	423
chr19:22414379	0.64	626	0.35	1016	0.35	1306	0.35	1011	0.44	1007
chr19:22457244	0.04	907	0.00	-	0.00	-	0.00	-	0.00	-
chr19:29855781	0.62	604	0.48	578	0.51	547	0.72	575	0.43	377
chr19:57996939	0.07	191	0.01	-	0.00	-	0.00	-	0.00	-
chr20:12402387	0.12	271	0.01	-	0.00	-	0.00	-	0.00	-
chrX:93606603	0.06	186	0.02	-	0.00	-	0.00	-	0.00	-
**Novel Sites Found in HNGS Not Previously Annotated**										
chr8:7671216*	0.19	49	0.04	-	0.04	112	0.02	-	0.06	19

*Not previously recognized as polymorphic

Table S2: Number of hiSNPs identified via HERVnGoSeq and 1000 Genomes Structural Variant Group; European Super Population Only

Insertion Site Coordinate GRCh37/hg19	1000 Genomes Variant ID	Number HERVnGoSeq hiSNPs	Number 1KG SV hiSNPs	Number SNPS LD > 0.2
chr8_37050885_R_POST	DEL_pindel_25540	96	36	64
chr4_9603240_R_PRE	SVA_umary_SVA_205	321	370	0
chr5_64388440_F_POST	SVA_umary_SVA_245	33	31	42
chr5_80442266_R_PRE	SVA_umary_SVA_252	75	28	8
chr6_161270898_F_POST	SVA_umary_SVA_320	814	736	258
chr1_111802591_R_PRE	SVA_umary_SVA_45	243	240	34
chr12_44313657_F_POST	SVA_umary_SVA_540	377	289	121
chr12_124066477_R_PRE	SVA_umary_SVA_556	394	399	103
chr13_90743183_R_PRE	SVA_umary_SVA_583	221	236	145
chr15_63374594_R_PRE	SVA_umary_SVA_637	431	343	40
chr19_21841536_R_PRE	SVA_umary_SVA_745	193	176	108
chr19_29855781_R_PRE	SVA_umary_SVA_748	468	457	295

Table S3: Associations of hiSNPs with polymprphic HERV-K insertions with African super pc

CHROM	RSID	SNP_POSITION	ALT	OR	P-VAL	HERV_POSITION
10	rs1536828	135342315	C	6.302	1.46E-08	chr:10:135355522
10	rs2480257	135352509	A	6.231	1.80E-08	chr:10:135355522
10	rs2480254	135352869	C	6.154	2.11E-08	chr:10:135355522
10	rs4240522	135355557	T	6.154	2.11E-08	chr:10:135355522
10	rs4363513	135355534	G	6.154	2.11E-08	chr:10:135355522
10	rs4542321	135355481	C	6.154	2.11E-08	chr:10:135355522
10	rs61868336	135350060	G	5.799	3.14E-08	chr:10:135355522
10	rs2987785	135365003	T	5.732	5.29E-08	chr:10:135355522
10	rs8192776	135348187	C	5.593	5.74E-08	chr:10:135355522
10	rs9418990	135337966	T	5.569	7.06E-08	chr:10:135355522
10	rs4512750	135358664	C	5.81	7.38E-08	chr:10:135355522
10	rs9418991	135362114	A	5.801	9.77E-08	chr:10:135355522
10	rs112345496	135382591	T	5.368	1.38E-07	chr:10:135355522
10	rs1536826	135357239	C	5.138	1.41E-07	chr:10:135355522
10	rs2987792	135371466	C	5.415	1.93E-07	chr:10:135355522
10	rs3020513	135364915	C	5.415	1.93E-07	chr:10:135355522
10	rs3020501	135381592	G	4.898	4.49E-07	chr:10:135355522
10	rs9629935	135398306	T	4.604	6.79E-07	chr:10:135355522
10	rs2987796	135379688	T	4.243	1.08E-06	chr:10:135355522
10	rs3020498	135379710	C	4.135	1.80E-06	chr:10:135355522
10	rs4259784	135399192	G	4.648	2.04E-06	chr:10:135355522
10	rs11101867	135400250	G	4.595	2.23E-06	chr:10:135355522
10	rs4133041	135408387	G	4.595	2.23E-06	chr:10:135355522
10	rs4351776	135415572	G	4.572	2.29E-06	chr:10:135355522
10	rs11101857	135395359	A	4.551	2.64E-06	chr:10:135355522
10	rs4129223	135403948	A	4.551	2.64E-06	chr:10:135355522
10	rs4306261	135410989	A	4.551	2.64E-06	chr:10:135355522
10	rs7084384	135412322	A	4.551	2.64E-06	chr:10:135355522
10	rs7084391	135412344	A	4.551	2.64E-06	chr:10:135355522
10	rs7093170	135402904	C	4.551	2.64E-06	chr:10:135355522
10	rs7094588	135401586	A	4.551	2.64E-06	chr:10:135355522
10	rs7907135	135410660	T	4.551	2.64E-06	chr:10:135355522
10	rs3020504	135385782	T	5.923	2.74E-06	chr:10:135355522
10	rs7080705	135426384	G	4.582	2.90E-06	chr:10:135355522
10	rs117220052	135388573	G	4.482	2.91E-06	chr:10:135355522
10	rs4454678	135431118	A	4.56	3.13E-06	chr:10:135355522
10	rs4075721	135429761	G	4.552	3.29E-06	chr:10:135355522
10	rs4379787	135429181	T	4.552	3.29E-06	chr:10:135355522
10	rs59241138	135393140	A	4.368	3.88E-06	chr:10:135355522
10	rs2987791	135370060	C	3.909	4.55E-06	chr:10:135355522
10	rs77382433	135389919	T	4.203	4.67E-06	chr:10:135355522

10	rs9630042	135433765	C	4.433	4.77E-06	chr:10:135355522
10	rs74514207	135386470	T	5.218	4.99E-06	chr:10:135355522
10	rs77360278	135386471	G	5.218	4.99E-06	chr:10:135355522
10	rs9630033	135418505	G	4.091	5.32E-06	chr:10:135355522
10	rs4556495	135422209	C	4.351	5.41E-06	chr:10:135355522
10	rs10857778	135432974	T	4.181	6.12E-06	chr:10:135355522
10	rs4528274	135429564	A	4.216	6.79E-06	chr:10:135355522
10	rs6537613	135412414	T	4.005	7.25E-06	chr:10:135355522
10	rs11101890	135421321	G	4.17	8.47E-06	chr:10:135355522
10	rs2070673	135340567	T	3.236	1.19E-05	chr:10:135355522
10	rs10857769	135405047	T	3.886	1.20E-05	chr:10:135355522
10	rs7075572	135404358	T	3.886	1.20E-05	chr:10:135355522
10	rs4335490	135402350	T	3.865	1.24E-05	chr:10:135355522
10	rs9630012	135397511	A	3.865	1.28E-05	chr:10:135355522
10	rs4450132	135427295	C	4.013	1.32E-05	chr:10:135355522
10	rs10857765	135394613	T	3.858	1.33E-05	chr:10:135355522
10	rs9629936	135398783	A	3.858	1.33E-05	chr:10:135355522
10	rs11101885	135417088	T	3.884	1.42E-05	chr:10:135355522
10	rs4589248	135429336	C	3.982	1.48E-05	chr:10:135355522
10	rs9630038	135429348	T	3.982	1.48E-05	chr:10:135355522
10	rs7922540	135395468	A	3.822	1.54E-05	chr:10:135355522
10	rs10745303	135432865	C	4.018	1.54E-05	chr:10:135355522
10	rs9418996	135423910	T	3.962	1.59E-05	chr:10:135355522
10	rs9629942	135430091	G	3.829	1.88E-05	chr:10:135355522
10	rs2987799	135380499	G	3.559	2.06E-05	chr:10:135355522
10	rs9418988	135327632	T	3.509	2.16E-05	chr:10:135355522
10	rs4463807	135425572	A	3.849	2.30E-05	chr:10:135355522
10	rs7090051	135430810	A	3.852	2.31E-05	chr:10:135355522
10	rs10857779	135432982	C	3.826	2.56E-05	chr:10:135355522
10	rs10857780	135432988	C	3.826	2.56E-05	chr:10:135355522
10	rs11101900	135432003	C	3.826	2.56E-05	chr:10:135355522
10	rs12261676	135433091	A	3.826	2.56E-05	chr:10:135355522
10	rs4628635	135430043	C	3.826	2.56E-05	chr:10:135355522
10	rs9629943	135433387	T	3.826	2.56E-05	chr:10:135355522
10	rs915906	135343738	T	3.108	2.62E-05	chr:10:135355522
10	rs10745304	135434988	G	3.813	2.64E-05	chr:10:135355522
10	rs6537618	135435995	G	3.646	2.86E-05	chr:10:135355522
10	rs7087703	135434565	T	3.789	2.91E-05	chr:10:135355522
10	rs3020502	135381623	C	3.227	3.01E-05	chr:10:135355522
10	rs2987800	135381927	G	3.237	3.04E-05	chr:10:135355522
10	rs3020503	135381766	T	3.237	3.04E-05	chr:10:135355522
10	rs10857776	135428908	A	3.778	3.18E-05	chr:10:135355522
10	rs3020499	135381215	G	3.187	3.20E-05	chr:10:135355522

10	rs3020500	135381276	C	3.187	3.20E-05	chr:10:135355522
10	rs2987797	135379934	A	3.174	4.21E-05	chr:10:135355522
10	rs10857768	135402703	A	3.689	4.47E-05	chr:10:135355522
10	rs9418998	135437279	A	3.112	5.02E-05	chr:10:135355522
11	rs2054849	60364127	C	7.241	6.37E-06	chr:11:60449890
11	rs56183826	60368846	A	6.488	1.22E-05	chr:11:60449890
11	rs75515174	60596312	T	6.594	2.14E-05	chr:11:60449890
11	rs7112307	60599009	T	6.577	2.25E-05	chr:11:60449890
11	rs7112316	60596173	A	6.26	2.73E-05	chr:11:60449890
11	rs61898779	60371542	T	5.716	2.95E-05	chr:11:60449890
11	rs475601	60599397	A	6.009	5.14E-05	chr:11:60449890
11	rs693716	60597793	A	5.957	5.22E-05	chr:11:60449890
11	rs112843408	71879422	T	0.1239	1.38E-11	chr:11:71875416
11	rs148742728	71876704	G	0.1239	1.38E-11	chr:11:71875416
11	rs55693133	71878942	T	0.1239	1.38E-11	chr:11:71875416
11	rs55853463	71878832	T	0.1239	1.38E-11	chr:11:71875416
11	rs57561154	71879181	T	0.1239	1.38E-11	chr:11:71875416
11	rs75576834	71877414	A	0.1239	1.38E-11	chr:11:71875416
11	rs77672783	71880076	C	0.1239	1.38E-11	chr:11:71875416
11	rs79775960	71878496	G	0.1239	1.38E-11	chr:11:71875416
11	rs59894793	71874950	G	0.135	2.59E-11	chr:11:71875416
11	rs61194383	71877877	T	0.1417	1.90E-10	chr:11:71875416
11	rs111594394	71879773	T	0.145	2.57E-10	chr:11:71875416
11	rs116700998	71874984	C	0.144	9.28E-10	chr:11:71875416
11	rs116305805	71874606	T	0.1465	1.29E-09	chr:11:71875416
11	rs117909722	71874660	C	0.1465	1.29E-09	chr:11:71875416
11	rs79147044	71874466	A	0.1465	1.29E-09	chr:11:71875416
11	rs111649414	71852971	C	0.1522	2.57E-09	chr:11:71875416
11	rs142294082	71876741	G	0.1626	1.21E-08	chr:11:71875416
11	rs147259385	71876632	T	0.1626	1.21E-08	chr:11:71875416
11	rs149792914	71880782	G	0.1626	1.21E-08	chr:11:71875416
11	rs192125273	71884785	T	0.1626	1.21E-08	chr:11:71875416
11	rs76789088	71882311	T	0.1626	1.21E-08	chr:11:71875416
11	rs78309837	71884566	G	0.1626	1.21E-08	chr:11:71875416
11	rs116526259	71878266	T	0.1632	1.30E-08	chr:11:71875416
11	rs114319082	71875068	T	0.1653	1.45E-08	chr:11:71875416
11	rs114683364	71852019	C	0.1658	1.53E-08	chr:11:71875416
11	rs77546838	71852460	C	0.1658	1.53E-08	chr:11:71875416
11	rs80194607	71885949	A	0.1721	1.64E-08	chr:11:71875416
11	rs111540831	71859396	G	0.1687	1.98E-08	chr:11:71875416
11	rs146852285	71859549	T	0.1687	1.98E-08	chr:11:71875416
11	rs77210719	71865739	T	0.1687	1.98E-08	chr:11:71875416
11	rs77780241	71876935	C	0.2643	7.19E-07	chr:11:71875416

11	rs75128106	72004156	T	0.1838	7.55E-07	chr:11:71875416
11	rs140631166	71852895	C	0.197	8.09E-07	chr:11:71875416
11	rs147767496	71852892	G	0.197	8.09E-07	chr:11:71875416
11	rs536159778	71852888	T	0.197	8.09E-07	chr:11:71875416
11	rs550493985	71852885	T	0.197	8.09E-07	chr:11:71875416
11	rs568992955	71852886	C	0.197	8.09E-07	chr:11:71875416
11	rs78599455	71884396	A	0.2706	1.13E-06	chr:11:71875416
11	rs78580101	71956877	G	0.1895	1.24E-06	chr:11:71875416
11	rs79908580	71952045	C	0.1895	1.24E-06	chr:11:71875416
11	rs78688516	71995580	A	0.1939	2.30E-06	chr:11:71875416
11	rs115096686	71869064	G	0.1833	2.55E-06	chr:11:71875416
11	rs141992224	71987009	A	0.1957	2.61E-06	chr:11:71875416
11	rs75702062	71982624	C	0.1957	2.61E-06	chr:11:71875416
11	rs77421727	71973793	A	0.1957	2.61E-06	chr:11:71875416
11	rs181342317	72002964	A	0.1975	2.84E-06	chr:11:71875416
11	rs74593868	72010677	G	0.1975	2.84E-06	chr:11:71875416
11	rs75074654	72020705	A	0.1975	2.84E-06	chr:11:71875416
11	rs75570162	72004346	C	0.1975	2.84E-06	chr:11:71875416
11	rs76022773	72016588	C	0.1975	2.84E-06	chr:11:71875416
11	rs76758409	72011870	A	0.1975	2.84E-06	chr:11:71875416
11	rs77881445	72008416	A	0.1975	2.84E-06	chr:11:71875416
11	rs79458542	72004238	G	0.1975	2.84E-06	chr:11:71875416
11	rs80334675	72000581	C	0.1975	2.84E-06	chr:11:71875416
11	rs115105740	71994057	G	0.1985	3.09E-06	chr:11:71875416
11	rs79304956	71937883	A	0.2049	3.28E-06	chr:11:71875416
11	rs75059162	71969869	G	0.2006	3.34E-06	chr:11:71875416
11	rs533755950	71852901	C	0.2079	3.41E-06	chr:11:71875416
11	rs558716278	71852903	T	0.2079	3.41E-06	chr:11:71875416
11	rs576865116	71852904	G	0.2079	3.41E-06	chr:11:71875416
11	rs114261779	71929456	T	0.2071	3.70E-06	chr:11:71875416
11	rs79191067	71931855	A	0.2071	3.70E-06	chr:11:71875416
11	rs114119194	71915580	G	0.2084	4.09E-06	chr:11:71875416
11	rs182654654	71919032	G	0.2084	4.09E-06	chr:11:71875416
11	rs77718722	71909649	C	0.2084	4.09E-06	chr:11:71875416
11	rs115738909	71897200	A	0.1935	4.20E-06	chr:11:71875416
11	rs79023399	71945145	T	0.237	5.59E-06	chr:11:71875416
11	rs78861042	71999717	C	0.2104	5.85E-06	chr:11:71875416
11	rs7939435	71660391	G	0.2037	6.72E-06	chr:11:71875416
11	rs633738	71883540	G	0.2922	7.29E-06	chr:11:71875416
11	rs393561	72281162	A	0.2864	1.49E-05	chr:11:71875416
11	rs113107673	71899266	T	0.3382	1.53E-05	chr:11:71875416
11	rs76985467	71902337	G	0.2256	1.59E-05	chr:11:71875416
11	rs80101434	71881852	G	0.3026	2.12E-05	chr:11:71875416

11	rs113654469	71875351	A	0.3541	2.22E-05	chr:11:71875416
11	rs143497983	71881523	T	0.3045	2.33E-05	chr:11:71875416
11	rs3750912	71726212	T	0.2834	2.55E-05	chr:11:71875416
11	rs78370345	72012797	T	0.2208	2.58E-05	chr:11:71875416
11	rs11235468	71923542	G	0.4074	2.74E-05	chr:11:71875416
11	rs114451879	72054088	G	0.2251	3.13E-05	chr:11:71875416
11	rs75516818	72053758	A	0.2251	3.13E-05	chr:11:71875416
11	rs76421471	72047174	G	0.2251	3.13E-05	chr:11:71875416
11	rs77508235	72046754	C	0.2251	3.13E-05	chr:11:71875416
11	rs116146961	72067052	T	0.2041	3.15E-05	chr:11:71875416
11	rs35567912	71724220	T	0.2689	3.59E-05	chr:11:71875416
11	rs112068862	71885591	T	0.3142	3.70E-05	chr:11:71875416
11	rs114480679	71883291	G	0.3142	3.70E-05	chr:11:71875416
11	rs115282592	71885527	G	0.3142	3.70E-05	chr:11:71875416
11	rs61427200	71882726	T	0.3142	3.70E-05	chr:11:71875416
11	rs74498718	71886281	C	0.3142	3.70E-05	chr:11:71875416
11	rs78222092	71884504	A	0.3142	3.70E-05	chr:11:71875416
11	rs80285294	71883930	T	0.3142	3.70E-05	chr:11:71875416
11	rs112587056	71687488	A	0.2714	3.98E-05	chr:11:71875416
12	rs113088709	124095455	G	46.01	3.12E-41	chr:12:124066477
12	rs138318001	124054104	C	58.85	4.32E-40	chr:12:124066477
12	rs75468015	124054109	C	58.85	4.32E-40	chr:12:124066477
12	rs191430524	124048835	A	44.61	6.11E-40	chr:12:124066477
12	rs75700739	124084424	A	44.97	6.23E-40	chr:12:124066477
12	rs7964934	124058639	A	44.41	8.16E-40	chr:12:124066477
12	rs11829661	124059488	A	47.44	1.06E-39	chr:12:124066477
12	rs78068178	124059962	T	47.44	1.06E-39	chr:12:124066477
12	rs183835928	124048838	T	47.56	1.13E-39	chr:12:124066477
12	rs114613914	124089740	G	59.79	6.51E-39	chr:12:124066477
12	rs3850953	124091412	T	59.79	6.51E-39	chr:12:124066477
12	rs7973689	124090655	T	59.79	6.51E-39	chr:12:124066477
12	rs79847496	124089348	G	59.79	6.51E-39	chr:12:124066477
12	rs113620982	124086656	T	58.82	1.14E-38	chr:12:124066477
12	rs111482432	124078585	G	66.16	1.48E-38	chr:12:124066477
12	rs7965292	124081779	T	66.16	1.48E-38	chr:12:124066477
12	rs12099985	124065667	C	65.79	1.79E-38	chr:12:124066477
12	rs7973187	124068015	C	65.79	1.79E-38	chr:12:124066477
12	rs6488882	124056572	T	48.1	2.71E-37	chr:12:124066477
12	rs80260318	124048074	G	43.37	3.78E-37	chr:12:124066477
12	rs112931584	124044574	A	42.47	5.74E-37	chr:12:124066477
12	rs7297951	124053711	C	46.3	7.63E-37	chr:12:124066477
12	rs7308408	124053542	A	46.3	7.63E-37	chr:12:124066477
12	rs142306340	124048858	G	51.57	1.32E-36	chr:12:124066477

12	rs7309150	124053413	T	49.5	1.82E-36	chr:12:124066477
12	rs7968699	124052243	A	44.98	1.91E-36	chr:12:124066477
12	rs111687827	124051096	C	49.88	1.99E-36	chr:12:124066477
12	rs111853781	124051065	G	49.88	1.99E-36	chr:12:124066477
12	rs113254073	124052945	A	49.88	1.99E-36	chr:12:124066477
12	rs113381757	124052913	C	49.88	1.99E-36	chr:12:124066477
12	rs11832820	124047285	C	49.88	1.99E-36	chr:12:124066477
12	rs145600453	124049196	G	49.88	1.99E-36	chr:12:124066477
12	rs1983274	124049736	A	49.88	1.99E-36	chr:12:124066477
12	rs1983275	124049759	A	49.88	1.99E-36	chr:12:124066477
12	rs7294379	124053430	G	49.88	1.99E-36	chr:12:124066477
12	rs7953401	124050042	A	49.88	1.99E-36	chr:12:124066477
12	rs7137130	124101981	C	23.98	2.19E-36	chr:12:124066477
12	rs78702455	124054015	G	49.8	2.20E-36	chr:12:124066477
12	rs7306906	124100486	A	29.94	3.36E-36	chr:12:124066477
12	rs2333828	124049030	T	47.75	4.49E-36	chr:12:124066477
12	rs112704788	124042091	G	13.52	5.53E-33	chr:12:124066477
12	rs112002563	124077902	T	122.3	4.27E-30	chr:12:124066477
12	rs7965466	124036188	C	10.06	8.42E-30	chr:12:124066477
12	rs7315041	124067244	A	14.87	8.90E-30	chr:12:124066477
12	rs11837236	124065116	G	105.4	5.26E-29	chr:12:124066477
12	rs7954782	124064537	G	105.4	5.26E-29	chr:12:124066477
12	rs7958153	124064998	G	105.4	5.26E-29	chr:12:124066477
12	rs7971779	124064918	T	105.4	5.26E-29	chr:12:124066477
12	rs35917561	124041472	T	9.739	9.88E-29	chr:12:124066477
12	rs112455213	124055507	A	94.53	1.98E-28	chr:12:124066477
12	rs116716383	124055605	G	94.53	1.98E-28	chr:12:124066477
12	rs34827093	124118248	G	8.71	2.61E-28	chr:12:124066477
12	rs4930715	124042949	C	11.49	8.89E-28	chr:12:124066477
12	rs111601834	124116169	G	8.203	9.76E-28	chr:12:124066477
12	rs7301031	124116808	G	8.203	9.76E-28	chr:12:124066477
12	rs79606093	124111062	A	8.119	1.06E-27	chr:12:124066477
12	rs11057298	124057112	A	15.23	1.35E-27	chr:12:124066477
12	rs112298489	124042646	C	9.136	1.37E-27	chr:12:124066477
12	rs11057306	124097766	G	12.05	1.52E-27	chr:12:124066477
12	rs146467055	124131997	T	7.927	1.52E-27	chr:12:124066477
12	rs10846533	124123564	T	7.868	2.06E-27	chr:12:124066477
12	rs11572946	124128650	T	7.868	2.06E-27	chr:12:124066477
12	rs11572939	124126163	C	7.778	2.71E-27	chr:12:124066477
12	rs116795979	124131902	G	7.778	2.71E-27	chr:12:124066477
12	rs7305096	124125481	C	7.778	2.71E-27	chr:12:124066477
12	rs11572945	124128502	T	7.809	2.73E-27	chr:12:124066477
12	rs114627783	124099713	A	9.664	3.13E-27	chr:12:124066477

12	rs61678733	124094174	C	49.58	4.00E-27	chr:12:124066477
12	rs73414262	124093859	G	49.58	4.00E-27	chr:12:124066477
12	rs786437	124090814	G	48.94	5.00E-27	chr:12:124066477
12	rs11615300	124100423	G	7.822	6.09E-27	chr:12:124066477
12	rs6488881	124056441	G	109.3	1.07E-26	chr:12:124066477
12	rs7136101	124056685	A	109.3	1.07E-26	chr:12:124066477
12	rs11057315	124129924	A	7.361	1.10E-26	chr:12:124066477
12	rs7295064	124127725	A	7.361	1.10E-26	chr:12:124066477
12	rs34097324	124098269	G	7.563	1.70E-26	chr:12:124066477
12	rs114225771	124043332	G	10.17	1.88E-26	chr:12:124066477
12	rs7316613	124045580	T	18.27	1.89E-26	chr:12:124066477
12	rs1050448	124105925	A	7.256	3.59E-26	chr:12:124066477
12	rs7314792	124036984	G	7.32	3.66E-26	chr:12:124066477
12	rs786428	124086998	C	56.05	3.99E-26	chr:12:124066477
12	rs786450	124071765	C	56.3	4.02E-26	chr:12:124066477
12	rs786429	124087394	T	56.23	4.08E-26	chr:12:124066477
12	rs786430	124087988	G	56.23	4.08E-26	chr:12:124066477
12	rs786431	124088366	T	56.23	4.08E-26	chr:12:124066477
12	rs786432	124088532	T	56.23	4.08E-26	chr:12:124066477
12	rs786433	124089582	A	56.23	4.08E-26	chr:12:124066477
12	rs786434	124089793	T	56.23	4.08E-26	chr:12:124066477
12	rs786435	124090208	G	56.23	4.08E-26	chr:12:124066477
12	rs786439	124091460	G	56.23	4.08E-26	chr:12:124066477
12	rs74996347	124086528	G	162.6	4.48E-26	chr:12:124066477
12	rs7952920	124085011	T	162.6	4.48E-26	chr:12:124066477
12	rs7970545	124085080	G	162.6	4.48E-26	chr:12:124066477
12	rs786438	124091218	A	55.26	5.08E-26	chr:12:124066477
12	rs78955489	124068910	A	156.6	1.11E-25	chr:12:124066477
12	rs143269373	124071864	A	152.8	1.35E-25	chr:12:124066477
12	rs11057323	124142726	G	6.841	1.78E-25	chr:12:124066477
12	rs111507681	124088418	T	210.7	1.06E-23	chr:12:124066477
12	rs7303554	124079920	G	210.7	1.06E-23	chr:12:124066477
12	rs77592647	124084160	G	210.7	1.06E-23	chr:12:124066477
12	rs11057302	124070013	A	210	1.15E-23	chr:12:124066477
12	rs11610013	124069614	A	210	1.15E-23	chr:12:124066477
12	rs11834369	124059546	T	210	1.15E-23	chr:12:124066477
12	rs12099986	124065978	G	210	1.15E-23	chr:12:124066477
12	rs138708987	124058169	C	210	1.15E-23	chr:12:124066477
12	rs146539578	124071144	T	210	1.15E-23	chr:12:124066477
12	rs146936648	124074744	G	210	1.15E-23	chr:12:124066477
12	rs1877365	124061397	T	210	1.15E-23	chr:12:124066477
12	rs2340691	124061421	T	210	1.15E-23	chr:12:124066477
12	rs74643748	124075915	T	210	1.15E-23	chr:12:124066477

12	rs77895354	124075111	G	210	1.15E-23	chr:12:124066477
12	rs7960812	124062742	T	210	1.15E-23	chr:12:124066477
12	rs7968040	124058937	A	210	1.15E-23	chr:12:124066477
12	rs79933835	124073911	T	210	1.15E-23	chr:12:124066477
12	rs952360	124076806	C	210	1.15E-23	chr:12:124066477
12	rs12099981	124065428	C	115.3	1.40E-23	chr:12:124066477
12	rs7301377	124076254	T	206.8	1.47E-23	chr:12:124066477
12	rs111625397	124058009	A	203.6	1.63E-23	chr:12:124066477
12	rs7308304	124100857	T	5.547	1.90E-23	chr:12:124066477
12	rs35708602	124099704	C	5.574	1.97E-23	chr:12:124066477
12	rs55705511	124105336	A	5.457	2.55E-23	chr:12:124066477
12	rs6488884	124108620	A	5.447	3.09E-23	chr:12:124066477
12	rs15587	124104049	G	5.453	3.25E-23	chr:12:124066477
12	rs3190835	124105328	C	5.453	3.25E-23	chr:12:124066477
12	rs66694750	124103579	T	5.443	3.82E-23	chr:12:124066477
12	rs56675602	124101041	G	5.426	4.03E-23	chr:12:124066477
12	rs3190836	124105226	T	5.416	4.37E-23	chr:12:124066477
12	rs118111778	124083600	T	188.2	4.58E-23	chr:12:124066477
12	rs11831129	124110675	G	5.427	6.25E-23	chr:12:124066477
12	rs6488885	124119155	G	5.427	6.25E-23	chr:12:124066477
12	rs113971302	124102801	T	5.334	6.97E-23	chr:12:124066477
12	rs11833889	124103869	T	5.334	6.97E-23	chr:12:124066477
12	rs3204541	124104686	A	5.334	6.97E-23	chr:12:124066477
12	rs35867744	124103808	A	5.334	6.97E-23	chr:12:124066477
12	rs59126974	124102656	T	5.334	6.97E-23	chr:12:124066477
12	rs7137473	124102216	A	5.334	6.97E-23	chr:12:124066477
12	rs68163141	124102871	C	5.329	8.30E-23	chr:12:124066477
12	rs1050449	124105908	G	5.261	1.06E-22	chr:12:124066477
12	rs34514002	124102920	C	5.3	1.25E-22	chr:12:124066477
12	rs884892	124097528	G	5.722	1.52E-22	chr:12:124066477
12	rs7134190	124116483	C	5.228	2.13E-22	chr:12:124066477
12	rs530657685	124084061	G	10.01	4.06E-22	chr:12:124066477
12	rs6488886	124120205	A	6.413	4.36E-22	chr:12:124066477
12	rs7965502	124137418	G	6.186	6.47E-22	chr:12:124066477
12	rs11572936	124125033	A	6.133	9.29E-22	chr:12:124066477
12	rs116743016	124182567	T	5.816	1.04E-21	chr:12:124066477
12	rs11830487	124178775	T	5.816	1.04E-21	chr:12:124066477
12	rs7134863	124156762	A	5.816	1.04E-21	chr:12:124066477
12	rs7304334	124177865	A	5.816	1.04E-21	chr:12:124066477
12	rs114925278	124149456	A	5.723	2.01E-21	chr:12:124066477
12	rs7965334	124036026	C	4.841	2.97E-21	chr:12:124066477
12	rs28454032	124034703	G	4.436	4.47E-20	chr:12:124066477
12	rs5027860	124032819	G	4.436	4.47E-20	chr:12:124066477

12	rs4534668	124029746	C	4.453	1.18E-18	chr:12:124066477
12	rs28743126	124028131	A	4.425	1.55E-18	chr:12:124066477
12	rs6488889	124144793	C	3.771	5.03E-18	chr:12:124066477
12	rs7139162	124102889	C	0.2538	6.13E-18	chr:12:124066477
12	rs113129322	124027872	A	4.149	8.86E-18	chr:12:124066477
12	rs28627432	124029137	C	4.107	2.73E-17	chr:12:124066477
12	rs139315135	124296141	A	8.272	4.55E-17	chr:12:124066477
12	rs12817861	124046540	C	0.2308	4.98E-17	chr:12:124066477
12	rs4310694	124050760	G	0.2308	4.98E-17	chr:12:124066477
12	rs2458115	124053902	A	0.2301	5.07E-17	chr:12:124066477
12	rs11831217	124167664	A	5.385	5.26E-17	chr:12:124066477
12	rs150012413	124157373	A	5.385	5.26E-17	chr:12:124066477
12	rs2048616	124049966	A	0.2307	5.72E-17	chr:12:124066477
12	rs11572996	124142295	A	5.303	1.02E-16	chr:12:124066477
12	rs11057301	124063905	A	0.2588	1.45E-16	chr:12:124066477
12	rs34369761	124073721	C	0.2601	1.93E-16	chr:12:124066477
12	rs4930714	124067197	A	0.2601	1.93E-16	chr:12:124066477
12	rs7973766	124070991	A	0.2601	1.93E-16	chr:12:124066477
12	rs4930713	124080619	A	0.2632	3.05E-16	chr:12:124066477
12	rs10846528	124052985	G	0.2635	4.89E-16	chr:12:124066477
12	rs10773021	124128424	T	3.007	3.12E-15	chr:12:124066477
12	rs10846534	124126652	T	3.007	3.12E-15	chr:12:124066477
12	rs11057313	124126064	A	3.007	3.12E-15	chr:12:124066477
12	rs11572940	124126180	G	3.007	3.12E-15	chr:12:124066477
12	rs2292702	124130128	C	3.007	3.12E-15	chr:12:124066477
12	rs6488887	124125018	A	3.007	3.12E-15	chr:12:124066477
12	rs7302177	124125757	G	3.007	3.12E-15	chr:12:124066477
12	rs7967780	124128989	T	3.007	3.12E-15	chr:12:124066477
12	rs2942079	124058053	A	4.474	5.99E-15	chr:12:124066477
12	rs57773235	124126090	G	0.3378	1.91E-13	chr:12:124066477
12	rs7974138	124011075	A	3.205	2.28E-13	chr:12:124066477
12	rs7296804	124153863	C	2.833	2.42E-13	chr:12:124066477
12	rs11834141	124150696	C	2.808	3.53E-13	chr:12:124066477
12	rs111682989	124116125	A	0.3495	3.79E-13	chr:12:124066477
12	rs3748270	124115200	A	0.3495	3.79E-13	chr:12:124066477
12	rs3923720	124117446	C	0.3495	3.79E-13	chr:12:124066477
12	rs7298287	124111723	T	0.3495	3.79E-13	chr:12:124066477
12	rs7316410	124116585	A	0.3495	3.79E-13	chr:12:124066477
12	rs114033770	124098056	A	10.47	4.39E-13	chr:12:124066477
12	rs78878823	124016535	A	3.149	4.55E-13	chr:12:124066477
12	rs3748268	124097824	A	0.3162	4.70E-13	chr:12:124066477
12	rs143138404	124019926	C	3.193	6.39E-13	chr:12:124066477
12	rs115152155	124090257	A	10.27	7.60E-13	chr:12:124066477

12	rs11829472	124064580	A	10.27	7.60E-13	chr:12:124066477
12	rs141870968	124084630	T	10.27	7.60E-13	chr:12:124066477
12	rs7960056	124077212	C	10.27	7.60E-13	chr:12:124066477
12	rs115648674	124047990	T	10.08	1.35E-12	chr:12:124066477
12	rs191163652	124049692	T	10.08	1.35E-12	chr:12:124066477
12	rs7315326	124033885	A	0.3573	2.76E-12	chr:12:124066477
12	rs7957883	124064573	C	6.209	3.71E-12	chr:12:124066477
12	rs7968439	124064576	T	6.209	3.71E-12	chr:12:124066477
12	rs28668364	124026482	T	3.291	3.83E-12	chr:12:124066477
12	rs28711796	124008074	C	2.929	4.41E-12	chr:12:124066477
12	rs80351220	124107034	A	10.13	5.01E-12	chr:12:124066477
12	rs71458805	124017254	G	3.15	7.17E-12	chr:12:124066477
12	rs11612479	124155526	G	2.555	7.56E-12	chr:12:124066477
12	rs73416283	124149382	A	2.774	8.14E-12	chr:12:124066477
12	rs786441	124062743	A	0.3089	8.14E-12	chr:12:124066477
12	rs11057319	124140512	G	6.98	8.40E-12	chr:12:124066477
12	rs11572994	124141518	G	6.98	8.40E-12	chr:12:124066477
12	rs145889717	124148637	T	6.98	8.40E-12	chr:12:124066477
12	rs75331857	124153272	C	6.98	8.40E-12	chr:12:124066477
12	rs144036481	124290473	G	3.294	9.33E-12	chr:12:124066477
12	rs112673999	124292569	G	3.293	1.15E-11	chr:12:124066477
12	rs3850952	124090802	T	0.3077	1.47E-11	chr:12:124066477
12	rs79320889	124290233	C	3.234	1.52E-11	chr:12:124066477
12	rs7972380	124065474	G	0.3123	2.12E-11	chr:12:124066477
12	rs75889421	124298787	G	2.884	2.42E-11	chr:12:124066477
12	rs555037713	124281432	C	7.765	2.65E-11	chr:12:124066477
12	rs786426	124086086	C	3.176	2.92E-11	chr:12:124066477
12	rs11057300	124059096	T	0.317	3.02E-11	chr:12:124066477
12	rs577771405	124224753	C	5.735	4.37E-11	chr:12:124066477
12	rs114155449	124115491	C	6.616	6.66E-11	chr:12:124066477
12	rs7135660	124174234	A	2.469	7.15E-11	chr:12:124066477
12	rs79342289	124256095	G	5.47	8.68E-11	chr:12:124066477
12	rs2048802	124098780	T	0.3404	9.99E-11	chr:12:124066477
12	rs149885512	124164576	A	6.312	1.04E-10	chr:12:124066477
12	rs75181091	124292748	A	3.278	1.30E-10	chr:12:124066477
12	rs77682183	124291170	G	3.278	1.30E-10	chr:12:124066477
12	rs78648333	124252117	A	5.242	1.48E-10	chr:12:124066477
12	rs7302183	124104460	C	0.3335	1.72E-10	chr:12:124066477
12	rs112921890	124291095	G	3.013	2.33E-10	chr:12:124066477
12	rs4930708	124145697	T	0.3886	3.42E-10	chr:12:124066477
12	rs7133199	124159185	A	0.3886	3.42E-10	chr:12:124066477
12	rs11836898	124231853	A	4.355	3.87E-10	chr:12:124066477
12	rs114102316	124268435	G	6.384	4.36E-10	chr:12:124066477

12	rs11832319	124269776	A	6.384	4.36E-10	chr:12:124066477
12	rs148051740	124263547	C	6.13	4.72E-10	chr:12:124066477
12	rs7134074	124159885	A	0.3935	5.09E-10	chr:12:124066477
12	rs7313140	124157188	T	0.3935	5.09E-10	chr:12:124066477
12	rs7313261	124166937	G	0.3935	5.09E-10	chr:12:124066477
12	rs115922900	124268788	T	6.073	5.26E-10	chr:12:124066477
12	rs35158437	124160764	C	0.3953	6.03E-10	chr:12:124066477
12	rs116357568	124302912	T	7.661	6.08E-10	chr:12:124066477
12	rs1051793	124144395	A	0.3965	6.13E-10	chr:12:124066477
12	rs116756952	124267615	T	6.421	6.75E-10	chr:12:124066477
12	rs114808264	124270904	G	5.876	7.08E-10	chr:12:124066477
12	rs115350765	124314005	A	7.486	8.32E-10	chr:12:124066477
12	rs112875110	124303111	T	2.557	1.04E-09	chr:12:124066477
12	rs141788165	124263705	C	6.069	1.24E-09	chr:12:124066477
12	rs115354182	124356481	C	4.047	1.26E-09	chr:12:124066477
12	rs150916971	124127485	T	6.118	1.28E-09	chr:12:124066477
12	rs116056046	124072693	G	13.74	1.30E-09	chr:12:124066477
12	rs116048436	124109108	G	6.419	1.37E-09	chr:12:124066477
12	rs6488883	124101347	G	0.3108	1.42E-09	chr:12:124066477
12	rs115259342	124056219	G	11.9	1.48E-09	chr:12:124066477
12	rs4456350	124058489	G	0.3139	1.94E-09	chr:12:124066477
12	rs11057305	124095447	G	2.661	2.03E-09	chr:12:124066477
12	rs150416099	124065185	G	12.97	2.24E-09	chr:12:124066477
12	rs6551	124082240	A	0.3187	2.45E-09	chr:12:124066477
12	rs786448	124070214	G	0.3187	2.45E-09	chr:12:124066477
12	rs786449	124071728	T	0.3187	2.45E-09	chr:12:124066477
12	rs74874384	124067102	A	12.87	3.07E-09	chr:12:124066477
12	rs116338737	124287243	T	6.336	3.11E-09	chr:12:124066477
12	rs151312164	124288849	A	6.336	3.11E-09	chr:12:124066477
12	rs116021417	124297270	T	5.691	3.26E-09	chr:12:124066477
12	rs786443	124063891	C	0.3481	3.77E-09	chr:12:124066477
12	rs7134896	124101858	C	0.326	3.83E-09	chr:12:124066477
12	rs1543075	124086448	A	0.3277	4.37E-09	chr:12:124066477
12	rs1543076	124086449	T	0.3277	4.37E-09	chr:12:124066477
12	rs786422	124083562	A	0.3503	4.58E-09	chr:12:124066477
12	rs786423	124083898	A	0.3503	4.58E-09	chr:12:124066477
12	rs786445	124068361	A	0.3503	4.58E-09	chr:12:124066477
12	rs786447	124069667	G	0.3503	4.58E-09	chr:12:124066477
12	rs114199292	124265429	A	6.112	5.09E-09	chr:12:124066477
12	rs150929624	124265338	G	6.112	5.09E-09	chr:12:124066477
12	rs7313628	124107015	G	0.3304	5.25E-09	chr:12:124066477
12	rs7305701	124150013	T	0.4224	5.67E-09	chr:12:124066477
12	rs116082071	124270365	A	6.013	5.95E-09	chr:12:124066477

12	rs185077712	124340945	A	7.233	6.04E-09	chr:12:124066477
12	rs10846537	124138346	C	0.4256	7.24E-09	chr:12:124066477
12	rs79683601	124307878	T	7.68	7.62E-09	chr:12:124066477
12	rs11057307	124098213	T	0.355	8.18E-09	chr:12:124066477
12	rs3748269	124099925	T	0.355	8.18E-09	chr:12:124066477
12	rs114910324	124330106	T	6.77	8.40E-09	chr:12:124066477
12	rs143454970	124329334	C	6.77	8.40E-09	chr:12:124066477
12	rs146985730	124334700	A	6.77	8.40E-09	chr:12:124066477
12	rs77251237	124329047	T	6.77	8.40E-09	chr:12:124066477
12	rs116167096	124323011	C	4.414	8.74E-09	chr:12:124066477
12	rs114185277	124308237	C	7.568	9.45E-09	chr:12:124066477
12	rs115569365	124228318	C	5.596	1.15E-08	chr:12:124066477
12	rs115756304	124225798	T	5.596	1.15E-08	chr:12:124066477
12	rs115294027	124226320	T	3.909	1.46E-08	chr:12:124066477
12	rs142761113	124225038	A	3.909	1.46E-08	chr:12:124066477
12	rs144294607	124227052	T	3.909	1.46E-08	chr:12:124066477
12	rs77005312	124311563	A	4.542	1.47E-08	chr:12:124066477
12	rs79980997	124310034	T	4.542	1.47E-08	chr:12:124066477
12	rs114520747	124328451	G	6.273	1.48E-08	chr:12:124066477
12	rs192264853	124271218	G	5.293	1.50E-08	chr:12:124066477
12	rs9919	124105839	T	0.4074	1.53E-08	chr:12:124066477
12	rs114594614	124203097	A	5.41	1.56E-08	chr:12:124066477
12	rs76014889	124286312	T	2.649	1.69E-08	chr:12:124066477
12	rs7301065	124154838	T	0.4382	1.69E-08	chr:12:124066477
12	rs1515814	124108699	G	0.3675	1.72E-08	chr:12:124066477
12	rs12305741	124056432	T	0.2296	1.74E-08	chr:12:124066477
12	rs190060224;rs113977710	124299609	A	2.824	1.79E-08	chr:12:124066477
12	rs10846531	124098612	G	0.3809	1.92E-08	chr:12:124066477
12	rs11572935	124124627	A	3.286	2.13E-08	chr:12:124066477
12	rs116703046	124310498	C	4.222	2.32E-08	chr:12:124066477
12	rs541534012	124240646	C	3.27	2.34E-08	chr:12:124066477
12	rs116621164	124308231	T	4.455	2.53E-08	chr:12:124066477
12	rs149260700	124064418	A	9.533	2.60E-08	chr:12:124066477
12	rs11057326	124148478	G	0.4439	2.95E-08	chr:12:124066477
12	rs114272330	124313626	G	4.381	3.21E-08	chr:12:124066477
12	rs7972413	124108296	T	0.3743	3.28E-08	chr:12:124066477
12	rs11610473	124149975	G	2.111	3.33E-08	chr:12:124066477
12	rs11611354	124153639	T	2.111	3.33E-08	chr:12:124066477
12	rs115025821	124302624	G	4.143	3.89E-08	chr:12:124066477
12	rs115483454	124307392	G	4.143	3.89E-08	chr:12:124066477
12	rs142996691	124303149	A	4.143	3.89E-08	chr:12:124066477
12	rs146364969	124303033	A	4.143	3.89E-08	chr:12:124066477
12	rs1022320	124264319	G	2.266	4.04E-08	chr:12:124066477

12	rs141811474	124360917	T	6.953	4.20E-08	chr:12:124066477
12	rs75866956	124256097	T	6.445	4.53E-08	chr:12:124066477
12	rs115362943	124052578	G	6.383	5.93E-08	chr:12:124066477
12	rs116771019	124046353	A	6.383	5.93E-08	chr:12:124066477
12	rs145614580	124043851	T	6.383	5.93E-08	chr:12:124066477
12	rs78440621	124302210	C	2.431	6.99E-08	chr:12:124066477
12	rs7960017	124165059	C	2.043	9.42E-08	chr:12:124066477
12	rs146499491	124309020	C	3.939	9.75E-08	chr:12:124066477
12	rs7294420	124118489	C	3.635	1.03E-07	chr:12:124066477
12	rs7979245	124124907	T	3.635	1.03E-07	chr:12:124066477
12	rs10846527	124043296	C	3.125	1.25E-07	chr:12:124066477
12	rs61154946	124061611	T	0.3251	1.27E-07	chr:12:124066477
12	rs73219037	124184482	C	2.022	1.30E-07	chr:12:124066477
12	rs7973155	124185948	G	2.022	1.30E-07	chr:12:124066477
12	rs11057297	124055090	T	0.3442	1.44E-07	chr:12:124066477
12	rs28834793	124040064	A	0.4605	1.47E-07	chr:12:124066477
12	rs10773034	124234253	G	0.4345	1.53E-07	chr:12:124066477
12	rs118004082	124169240	T	2.012	1.61E-07	chr:12:124066477
12	rs114208992	124169242	C	2.01	1.62E-07	chr:12:124066477
12	rs116040307	124234939	A	5.414	1.67E-07	chr:12:124066477
12	rs35946281	124177016	A	1.998	1.87E-07	chr:12:124066477
12	rs113310757	124161494	G	2.005	1.89E-07	chr:12:124066477
12	rs12820195	124162047	C	2.005	1.89E-07	chr:12:124066477
12	rs12822546	124157247	A	2.005	1.89E-07	chr:12:124066477
12	rs12824075	124157335	T	2.005	1.89E-07	chr:12:124066477
12	rs34534928	124160834	C	2.005	1.89E-07	chr:12:124066477
12	rs6488890	124158971	T	2.005	1.89E-07	chr:12:124066477
12	rs6488891	124163512	T	2.005	1.89E-07	chr:12:124066477
12	rs7133543	124159411	G	2.005	1.89E-07	chr:12:124066477
12	rs7138209	124157052	A	2.005	1.89E-07	chr:12:124066477
12	rs7302649	124159766	A	2.005	1.89E-07	chr:12:124066477
12	rs7953596	124158450	C	2.005	1.89E-07	chr:12:124066477
12	rs7953599	124158455	C	2.005	1.89E-07	chr:12:124066477
12	rs7959553	124162705	T	2.005	1.89E-07	chr:12:124066477
12	rs7963498	124158502	A	2.005	1.89E-07	chr:12:124066477
12	rs7968029	124182120	T	2.005	1.89E-07	chr:12:124066477
12	rs7969130	124161056	G	2.005	1.89E-07	chr:12:124066477
12	rs7969234	124161110	G	2.005	1.89E-07	chr:12:124066477
12	rs7973695	124162477	G	2.005	1.89E-07	chr:12:124066477
12	rs7975119	124157674	T	2.005	1.89E-07	chr:12:124066477
12	rs7975233	124157745	G	2.005	1.89E-07	chr:12:124066477
12	rs7978447	124158080	G	2.005	1.89E-07	chr:12:124066477
12	rs151085897	124037165	C	5.471	1.94E-07	chr:12:124066477

12	rs11837257	124190394	C	1.995	2.08E-07	chr:12:124066477
12	rs11612030	124188064	T	1.994	2.10E-07	chr:12:124066477
12	rs11613632	124184065	A	1.994	2.10E-07	chr:12:124066477
12	rs11614468	124187924	C	1.994	2.10E-07	chr:12:124066477
12	rs11830462	124178665	T	1.994	2.10E-07	chr:12:124066477
12	rs11831536	124176748	G	1.994	2.10E-07	chr:12:124066477
12	rs11833604	124176472	A	1.994	2.10E-07	chr:12:124066477
12	rs11834492	124180011	C	1.994	2.10E-07	chr:12:124066477
12	rs11834808	124190582	T	1.994	2.10E-07	chr:12:124066477
12	rs11837256	124190393	T	1.994	2.10E-07	chr:12:124066477
12	rs12818596	124180474	C	1.994	2.10E-07	chr:12:124066477
12	rs12821242	124178329	A	1.994	2.10E-07	chr:12:124066477
12	rs12823866	124165111	C	1.994	2.10E-07	chr:12:124066477
12	rs12827074	124190836	C	1.994	2.10E-07	chr:12:124066477
12	rs12828408	124198718	C	1.994	2.10E-07	chr:12:124066477
12	rs12829984	124172307	T	1.994	2.10E-07	chr:12:124066477
12	rs12831081	124179739	G	1.994	2.10E-07	chr:12:124066477
12	rs2203500	124194710	G	1.994	2.10E-07	chr:12:124066477
12	rs2222060	124194438	G	1.994	2.10E-07	chr:12:124066477
12	rs35416308	124169461	G	1.994	2.10E-07	chr:12:124066477
12	rs3817309	124198721	T	1.994	2.10E-07	chr:12:124066477
12	rs6488892	124171826	C	1.994	2.10E-07	chr:12:124066477
12	rs6488893	124171903	G	1.994	2.10E-07	chr:12:124066477
12	rs6488894	124185124	T	1.994	2.10E-07	chr:12:124066477
12	rs66471742	124199006	A	1.994	2.10E-07	chr:12:124066477
12	rs66690784	124165677	G	1.994	2.10E-07	chr:12:124066477
12	rs67178535	124196299	A	1.994	2.10E-07	chr:12:124066477
12	rs7135267	124199120	C	1.994	2.10E-07	chr:12:124066477
12	rs71458814	124196891	G	1.994	2.10E-07	chr:12:124066477
12	rs7302788	124167021	C	1.994	2.10E-07	chr:12:124066477
12	rs7304052	124177643	A	1.994	2.10E-07	chr:12:124066477
12	rs7304837	124177538	T	1.994	2.10E-07	chr:12:124066477
12	rs7305838	124193471	A	1.994	2.10E-07	chr:12:124066477
12	rs7307343	124174365	G	1.994	2.10E-07	chr:12:124066477
12	rs7308234	124183804	T	1.994	2.10E-07	chr:12:124066477
12	rs7309528	124171658	T	1.994	2.10E-07	chr:12:124066477
12	rs7310918	124174656	C	1.994	2.10E-07	chr:12:124066477
12	rs7397160	124196219	A	1.994	2.10E-07	chr:12:124066477
12	rs7397230	124182659	G	1.994	2.10E-07	chr:12:124066477
12	rs7397930	124188581	A	1.994	2.10E-07	chr:12:124066477
12	rs7398152	124184639	G	1.994	2.10E-07	chr:12:124066477
12	rs7398298	124192728	T	1.994	2.10E-07	chr:12:124066477
12	rs7399224	124195856	T	1.994	2.10E-07	chr:12:124066477

12	rs7963968	124166041	C	1.994	2.10E-07	chr:12:124066477
12	rs7969964	124165213	A	1.994	2.10E-07	chr:12:124066477
12	rs7979528	124181820	G	1.994	2.10E-07	chr:12:124066477
12	rs34586028	124187082	C	1.99	2.30E-07	chr:12:124066477
12	rs76618338	124302458	G	5.321	2.32E-07	chr:12:124066477
12	rs7977179	124315662	G	5.321	2.32E-07	chr:12:124066477
12	rs10773033	124231368	C	0.4418	2.39E-07	chr:12:124066477
12	rs79687357	124259487	T	2.035	2.57E-07	chr:12:124066477
12	rs112823641	124292928	G	2.072	2.65E-07	chr:12:124066477
12	rs7298440	124181049	T	1.981	2.69E-07	chr:12:124066477
12	rs12817757	124156487	T	1.983	2.76E-07	chr:12:124066477
12	rs10744162	124243707	C	0.4434	2.91E-07	chr:12:124066477
12	rs7976816	124315343	A	2.188	2.93E-07	chr:12:124066477
12	rs7307008	124160021	G	1.977	3.01E-07	chr:12:124066477
12	rs116647224	124282097	A	5.432	3.21E-07	chr:12:124066477
12	rs75844860	124295677	A	2.082	3.34E-07	chr:12:124066477
12	rs78883615	124296519	A	2.082	3.34E-07	chr:12:124066477
12	rs115048185	124146048	G	3.457	3.46E-07	chr:12:124066477
12	rs10161548	124088555	C	0.3151	3.98E-07	chr:12:124066477
12	rs2707	124105638	A	0.315	4.04E-07	chr:12:124066477
12	rs10773019	124104551	G	0.3146	4.06E-07	chr:12:124066477
12	rs10846530	124079831	C	0.3146	4.06E-07	chr:12:124066477
12	rs10846532	124105572	G	0.3146	4.06E-07	chr:12:124066477
12	rs11057299	124058287	A	0.3146	4.06E-07	chr:12:124066477
12	rs11057304	124095426	T	0.3146	4.06E-07	chr:12:124066477
12	rs12231444	124074365	T	0.3146	4.06E-07	chr:12:124066477
12	rs12308953	124080899	A	0.3146	4.06E-07	chr:12:124066477
12	rs12313119	124059924	T	0.3146	4.06E-07	chr:12:124066477
12	rs17840832	124069068	A	0.3146	4.06E-07	chr:12:124066477
12	rs73414223	124063614	A	0.3146	4.06E-07	chr:12:124066477
12	rs7358562	124096245	A	0.3146	4.06E-07	chr:12:124066477
12	rs4930731	124317416	G	2.131	4.76E-07	chr:12:124066477
12	rs4930732	124316091	C	2.131	4.76E-07	chr:12:124066477
12	rs12827680	124240645	C	0.4451	5.26E-07	chr:12:124066477
12	rs116217925	124273882	T	2.971	5.93E-07	chr:12:124066477
12	rs141506842	124274003	C	2.971	5.93E-07	chr:12:124066477
12	rs34996665	124134656	C	0.4961	6.21E-07	chr:12:124066477
12	rs73420364	124251160	T	0.5213	7.84E-07	chr:12:124066477
12	rs114636861	124055954	C	10.43	9.01E-07	chr:12:124066477
12	rs114333487	124004450	A	4.411	9.60E-07	chr:12:124066477
12	rs138487939	123900952	A	3.309	1.04E-06	chr:12:124066477
12	rs114018932	124249783	G	3.969	1.06E-06	chr:12:124066477
12	rs1139320	124197099	T	1.891	1.22E-06	chr:12:124066477

12	rs7135459	124300526	C	2.703	1.33E-06	chr:12:124066477
12	rs35911711	124201087	T	0.4417	1.34E-06	chr:12:124066477
12	rs113771811	124388732	C	3.116	1.39E-06	chr:12:124066477
12	rs2333834	124246004	A	0.4498	1.42E-06	chr:12:124066477
12	rs148052336	124194519	C	6.498	1.54E-06	chr:12:124066477
12	rs149625182	124094218	G	8.675	1.58E-06	chr:12:124066477
12	rs7980300	124258143	C	2.3	1.75E-06	chr:12:124066477
12	rs12815746	124126086	G	0.4602	1.83E-06	chr:12:124066477
12	rs7309510	124342015	A	3.76	2.04E-06	chr:12:124066477
12	rs188907902	124186997	T	4.686	2.15E-06	chr:12:124066477
12	rs116452654	124165541	T	3.292	2.27E-06	chr:12:124066477
12	rs115302188	124083436	T	7.465	2.28E-06	chr:12:124066477
12	rs116232382	124055366	T	7.465	2.28E-06	chr:12:124066477
12	rs149506295	124041628	C	14.44	2.46E-06	chr:12:124066477
12	rs114490301	124161289	T	6.295	2.61E-06	chr:12:124066477
12	rs115385140	124176248	C	6.295	2.61E-06	chr:12:124066477
12	rs141986228	124157053	T	6.295	2.61E-06	chr:12:124066477
12	rs146725051	124164953	A	6.295	2.61E-06	chr:12:124066477
12	rs7302406	124252688	C	0.5361	2.68E-06	chr:12:124066477
12	rs11572957	124131049	C	4.788	2.97E-06	chr:12:124066477
12	rs113767728	124390447	A	2.995	3.05E-06	chr:12:124066477
12	rs77362442	124325302	T	2.304	3.08E-06	chr:12:124066477
12	rs28408980	124026942	A	0.4169	3.26E-06	chr:12:124066477
12	rs7298068	124015832	T	0.4453	3.30E-06	chr:12:124066477
12	rs74560309	124375725	G	1.889	3.43E-06	chr:12:124066477
12	rs61953553	124022068	C	1.865	3.54E-06	chr:12:124066477
12	rs114746659	123957446	A	10.84	4.63E-06	chr:12:124066477
12	rs141364216	123948881	C	10.84	4.63E-06	chr:12:124066477
12	rs150131617	123948369	C	10.84	4.63E-06	chr:12:124066477
12	rs76616545	123956557	C	10.84	4.63E-06	chr:12:124066477
12	rs80183396	123994250	G	3.629	4.65E-06	chr:12:124066477
12	rs114809836	124235217	A	5.676	4.67E-06	chr:12:124066477
12	rs115183932	124244416	C	5.676	4.67E-06	chr:12:124066477
12	rs144080565	124240225	C	5.676	4.67E-06	chr:12:124066477
12	rs145999411	124245289	A	5.676	4.67E-06	chr:12:124066477
12	rs146066975	124234143	T	5.676	4.67E-06	chr:12:124066477
12	rs7297473	124245936	C	5.676	4.67E-06	chr:12:124066477
12	rs7969410	124235775	A	5.676	4.67E-06	chr:12:124066477
12	rs7980215	124243643	A	5.676	4.67E-06	chr:12:124066477
12	rs77070660	124110044	A	2.529	4.73E-06	chr:12:124066477
12	rs114404549	124157298	G	4.002	4.77E-06	chr:12:124066477
12	rs115250621	124173613	G	4.002	4.77E-06	chr:12:124066477
12	rs11572982	124139827	T	4.002	4.77E-06	chr:12:124066477

12	rs116452304	124196758	C	4.002	4.77E-06	chr:12:124066477
12	rs138728238	124166654	C	4.002	4.77E-06	chr:12:124066477
12	rs141540823	124176751	T	4.002	4.77E-06	chr:12:124066477
12	rs561163813	124196886	G	4.002	4.77E-06	chr:12:124066477
12	rs7308639	124162182	G	4.002	4.77E-06	chr:12:124066477
12	rs75514507	124179564	C	4.002	4.77E-06	chr:12:124066477
12	rs76854096	124184877	A	4.002	4.77E-06	chr:12:124066477
12	rs79639515	124162769	G	4.002	4.77E-06	chr:12:124066477
12	rs2333832	124020708	T	1.846	4.87E-06	chr:12:124066477
12	rs6488878	124023932	T	1.846	4.87E-06	chr:12:124066477
12	rs11572993	124141488	C	3.223	4.89E-06	chr:12:124066477
12	rs11572998	124143293	A	3.223	4.89E-06	chr:12:124066477
12	rs74320784	124188434	G	3.223	4.89E-06	chr:12:124066477
12	rs79884091	124166954	A	3.223	4.89E-06	chr:12:124066477
12	rs9723048	124006318	G	0.5098	4.93E-06	chr:12:124066477
12	rs61760955	123793863	C	2.992	5.27E-06	chr:12:124066477
12	rs7959714	124022720	C	1.849	5.42E-06	chr:12:124066477
12	rs76993175	123861292	C	2.662	5.44E-06	chr:12:124066477
12	rs116046657	124187720	A	3.961	5.45E-06	chr:12:124066477
12	rs115767062	124162628	T	4.115	5.74E-06	chr:12:124066477
12	rs147057636	124170576	G	4.115	5.74E-06	chr:12:124066477
12	rs76585413	124169830	C	4.115	5.74E-06	chr:12:124066477
12	rs28673982	124016464	C	0.519	5.85E-06	chr:12:124066477
12	rs7307227	124020098	T	0.4975	5.85E-06	chr:12:124066477
12	rs140212733	123985412	A	9.023	6.14E-06	chr:12:124066477
12	rs189575861	123986238	T	9.023	6.14E-06	chr:12:124066477
12	rs75553354	123973641	A	9.023	6.14E-06	chr:12:124066477
12	rs7980049	124291534	C	1.857	6.20E-06	chr:12:124066477
12	rs7300147	124116833	G	0.4714	6.46E-06	chr:12:124066477
12	rs7966882	124128965	G	0.4659	6.48E-06	chr:12:124066477
12	rs7971452	124126821	A	0.4665	6.84E-06	chr:12:124066477
12	rs7303956	124020050	T	1.83	6.86E-06	chr:12:124066477
12	rs2017181	124117257	T	0.4663	6.89E-06	chr:12:124066477
12	rs908373	124113319	C	0.4663	6.89E-06	chr:12:124066477
12	rs147974993	123936707	T	10.46	7.33E-06	chr:12:124066477
12	rs1533246	124019055	T	1.817	7.41E-06	chr:12:124066477
12	rs142676514	124194569	A	3.116	7.68E-06	chr:12:124066477
12	rs35016092	124022243	A	1.82	7.73E-06	chr:12:124066477
12	rs77765053	124245456	A	3.017	8.30E-06	chr:12:124066477
12	rs35970945	123955358	G	0.427	8.73E-06	chr:12:124066477
12	rs10846535	124130388	A	0.475	1.01E-05	chr:12:124066477
12	rs111260541	124226670	A	3.582	1.01E-05	chr:12:124066477
12	rs111291425	124204277	G	3.582	1.01E-05	chr:12:124066477

12	rs111865336	124210872	T	3.582	1.01E-05	chr:12:124066477
12	rs112095096	124215340	G	3.582	1.01E-05	chr:12:124066477
12	rs112325438	124202498	A	3.582	1.01E-05	chr:12:124066477
12	rs112713519	124208643	C	3.582	1.01E-05	chr:12:124066477
12	rs113038752	124220799	A	3.582	1.01E-05	chr:12:124066477
12	rs113425875	124217119	T	3.582	1.01E-05	chr:12:124066477
12	rs116985162	124211857	A	3.582	1.01E-05	chr:12:124066477
12	rs151281380	124222810	A	3.582	1.01E-05	chr:12:124066477
12	rs74691387	124224357	C	3.582	1.01E-05	chr:12:124066477
12	rs77988284	124225324	T	3.582	1.01E-05	chr:12:124066477
12	rs78856277	124208524	A	3.582	1.01E-05	chr:12:124066477
12	rs79138426	124204768	G	3.582	1.01E-05	chr:12:124066477
12	rs10734904	124204158	G	0.4877	1.14E-05	chr:12:124066477
12	rs10773031	124222909	G	1.795	1.15E-05	chr:12:124066477
12	rs882563	124210549	T	0.4881	1.22E-05	chr:12:124066477
12	rs9788204	124207209	T	0.4881	1.22E-05	chr:12:124066477
12	rs146132801	123986945	T	4.34	1.23E-05	chr:12:124066477
12	rs9697543	124004990	C	0.4517	1.24E-05	chr:12:124066477
12	rs9697721	124005312	C	0.4517	1.24E-05	chr:12:124066477
12	rs13303107	123939487	T	0.4379	1.25E-05	chr:12:124066477
12	rs67516712	124201524	G	0.4897	1.27E-05	chr:12:124066477
12	rs141716858	124002914	T	3.992	1.38E-05	chr:12:124066477
12	rs2063629	124006842	C	0.5314	1.41E-05	chr:12:124066477
12	rs116310078	124270467	T	9.679	1.47E-05	chr:12:124066477
12	rs139909820	123934751	T	7.353	1.67E-05	chr:12:124066477
12	rs10734908	124219036	A	0.497	1.83E-05	chr:12:124066477
12	rs10744160	124225542	G	0.497	1.83E-05	chr:12:124066477
12	rs10773032	124226063	G	0.497	1.83E-05	chr:12:124066477
12	rs2333837	124220522	T	0.497	1.83E-05	chr:12:124066477
12	rs7398851	124215004	G	0.497	1.83E-05	chr:12:124066477
12	rs2292500	124008073	A	0.5372	1.87E-05	chr:12:124066477
12	rs7969641	44310268	T	19.23	2.34E-38	chr:12:44313657
12	rs12301015	44321160	T	18.47	3.17E-38	chr:12:44313657
12	rs12303804	44340018	A	18.27	5.60E-38	chr:12:44313657
12	rs12309681	44343202	T	18.27	5.60E-38	chr:12:44313657
12	rs11182326	44310333	G	18.48	7.91E-38	chr:12:44313657
12	rs11182346	44350097	T	21.44	8.85E-38	chr:12:44313657
12	rs10785477	44318723	G	22.41	9.04E-38	chr:12:44313657
12	rs10880586	44310604	G	22.41	9.04E-38	chr:12:44313657
12	rs11182328	44311277	G	22.41	9.04E-38	chr:12:44313657
12	rs10785478	44320156	T	21.34	9.38E-38	chr:12:44313657
12	rs11182327	44310418	A	21.77	1.21E-37	chr:12:44313657
12	rs11182345	44347202	C	21.19	1.56E-37	chr:12:44313657

12	rs12315094	44345773	A	21.19	1.56E-37	chr:12:44313657
12	rs10785482	44342910	T	22.95	1.57E-37	chr:12:44313657
12	rs10880601	44343768	A	22.9	1.67E-37	chr:12:44313657
12	rs7312373	44332843	C	21.05	1.75E-37	chr:12:44313657
12	rs10880600	44343641	T	21.02	1.85E-37	chr:12:44313657
12	rs11829881	44322395	G	19.36	1.16E-36	chr:12:44313657
12	rs10880597	44336172	A	38.98	2.35E-35	chr:12:44313657
12	rs11182339	44331841	A	38.75	3.54E-35	chr:12:44313657
12	rs4417371	44327407	A	38.62	3.67E-35	chr:12:44313657
12	rs10880605	44349669	A	38.76	3.70E-35	chr:12:44313657
12	rs11182341	44339216	T	38.02	5.86E-35	chr:12:44313657
12	rs10785474	44318428	G	42.26	9.16E-35	chr:12:44313657
12	rs10785473	44316584	G	41.26	1.52E-34	chr:12:44313657
12	rs59709721	44362347	A	10.19	1.18E-32	chr:12:44313657
12	rs12318365	44352760	T	10.5	1.29E-32	chr:12:44313657
12	rs17093971	44359265	T	10.05	2.23E-32	chr:12:44313657
12	rs7308588	44356119	A	9.894	4.17E-32	chr:12:44313657
12	rs4768544	44309919	C	9.446	7.45E-29	chr:12:44313657
12	rs145822768	44350947	T	8.286	1.99E-27	chr:12:44313657
12	rs4499064	44315170	T	105.8	6.12E-26	chr:12:44313657
12	rs12297544	44319679	A	7.957	2.93E-24	chr:12:44313657
12	rs12297529	44319602	A	8.083	2.95E-24	chr:12:44313657
12	rs12301421	44346015	A	7.887	4.92E-24	chr:12:44313657
12	rs12302401	44333816	T	7.88	1.01E-23	chr:12:44313657
12	rs10880599	44337662	T	111.9	2.69E-23	chr:12:44313657
12	rs115692896	44349790	C	7.511	2.81E-23	chr:12:44313657
12	rs76876041	44355481	T	7.511	2.81E-23	chr:12:44313657
12	rs10880604	44349398	T	0.1924	3.36E-23	chr:12:44313657
12	rs11182337	44320875	G	0.1968	4.31E-23	chr:12:44313657
12	rs4238093	44315925	C	0.2002	6.97E-23	chr:12:44313657
12	rs7956825	44313718	G	151.1	1.95E-21	chr:12:44313657
12	rs12305959	44332618	G	148.5	2.20E-21	chr:12:44313657
12	rs10880598	44336375	T	145.2	3.03E-21	chr:12:44313657
12	rs10785479	44320184	G	145.6	3.15E-21	chr:12:44313657
12	rs3925073	44327736	A	145.5	3.16E-21	chr:12:44313657
12	rs3925072	44327565	C	144.4	3.44E-21	chr:12:44313657
12	rs73100975	44339016	T	143.8	3.61E-21	chr:12:44313657
12	rs10785489	44364261	C	4.576	1.16E-20	chr:12:44313657
12	rs78029959	44270446	T	5.028	6.14E-19	chr:12:44313657
12	rs10880592	44315171	A	212.3	7.38E-19	chr:12:44313657
12	rs10785471	44311998	A	207.1	8.70E-19	chr:12:44313657
12	rs12302508	44303914	C	0.2693	1.00E-18	chr:12:44313657
12	rs10748379	44315397	T	204.9	1.02E-18	chr:12:44313657

12	rs10748380	44315492	G	204.9	1.02E-18	chr:12:44313657
12	rs10785470	44311983	A	204.9	1.02E-18	chr:12:44313657
12	rs10785472	44312774	T	204.9	1.02E-18	chr:12:44313657
12	rs10880588	44311787	T	204.9	1.02E-18	chr:12:44313657
12	rs10880589	44313193	T	204.9	1.02E-18	chr:12:44313657
12	rs10880591	44314630	T	204.9	1.02E-18	chr:12:44313657
12	rs11182329	44314564	A	204.9	1.02E-18	chr:12:44313657
12	rs12369569	44313000	T	204.9	1.02E-18	chr:12:44313657
12	rs2031056	44315008	A	204.9	1.02E-18	chr:12:44313657
12	rs73100931	44314388	C	204.9	1.02E-18	chr:12:44313657
12	rs73100933	44314453	T	204.9	1.02E-18	chr:12:44313657
12	rs73100935	44314454	G	204.9	1.02E-18	chr:12:44313657
12	rs7957074	44313906	T	204.9	1.02E-18	chr:12:44313657
12	rs7964465	44312649	G	204.9	1.02E-18	chr:12:44313657
12	rs7971487	44313830	G	204.9	1.02E-18	chr:12:44313657
12	rs7974501	44313794	C	204.9	1.02E-18	chr:12:44313657
12	rs10506232	44316485	A	199.9	1.37E-18	chr:12:44313657
12	rs10785475	44318632	G	199.9	1.37E-18	chr:12:44313657
12	rs10785476	44318633	A	199.9	1.37E-18	chr:12:44313657
12	rs10880590	44313636	G	199.9	1.37E-18	chr:12:44313657
12	rs80098499	44241292	T	4.87	1.68E-18	chr:12:44313657
12	rs11533717	44304261	C	0.2785	2.53E-18	chr:12:44313657
12	rs4238092	44315762	A	0.3037	2.56E-16	chr:12:44313657
12	rs11182294	44231539	T	3.093	2.06E-15	chr:12:44313657
12	rs1623829	44262745	G	0.3027	1.08E-14	chr:12:44313657
12	rs7958314	44302430	T	2.993	1.63E-14	chr:12:44313657
12	rs12311962	44237272	G	0.3064	1.68E-14	chr:12:44313657
12	rs12314126	44236455	A	0.3071	1.78E-14	chr:12:44313657
12	rs73098836	44286253	A	2.948	2.67E-14	chr:12:44313657
12	rs7957146	44306759	G	2.948	2.67E-14	chr:12:44313657
12	rs7971516	44353473	A	0.3741	3.69E-13	chr:12:44313657
12	rs7133994	44331464	C	0.2767	4.04E-13	chr:12:44313657
12	rs7979913	44355025	C	0.2905	8.73E-13	chr:12:44313657
12	rs7296367	44357427	A	0.2939	8.99E-13	chr:12:44313657
12	rs4768545	44313129	C	0.2871	9.30E-13	chr:12:44313657
12	rs7955667	44318739	C	0.2871	9.30E-13	chr:12:44313657
12	rs10748383	44355232	A	0.2941	1.43E-12	chr:12:44313657
12	rs10785486	44359539	A	0.2941	1.43E-12	chr:12:44313657
12	rs10785487	44359593	G	0.2941	1.43E-12	chr:12:44313657
12	rs6582492	44353602	A	0.2941	1.43E-12	chr:12:44313657
12	rs151267396	44246174	T	2.696	2.31E-12	chr:12:44313657
12	rs1731425	44282630	T	2.647	2.45E-12	chr:12:44313657
12	rs10748384	44359086	C	0.3028	3.10E-12	chr:12:44313657

12	rs1675760	44282981	G	2.636	3.20E-12	chr:12:44313657
12	rs1675786	44292760	C	2.636	3.20E-12	chr:12:44313657
12	rs1675791	44290267	G	2.636	3.20E-12	chr:12:44313657
12	rs7966024	44297013	G	2.636	3.20E-12	chr:12:44313657
12	rs1675778	44263162	C	0.3602	3.25E-12	chr:12:44313657
12	rs10785469	44309459	A	0.3621	4.03E-12	chr:12:44313657
12	rs2447115	44254494	C	0.3654	4.63E-12	chr:12:44313657
12	rs1731422	44276369	G	2.586	6.08E-12	chr:12:44313657
12	rs10785468	44309434	T	0.367	8.13E-12	chr:12:44313657
12	rs115584644	44663575	G	5.518	8.24E-12	chr:12:44313657
12	rs10748373	44253386	G	0.3705	8.72E-12	chr:12:44313657
12	rs1675771	44252960	G	0.3705	8.72E-12	chr:12:44313657
12	rs1675774	44266575	T	0.3705	8.72E-12	chr:12:44313657
12	rs1675776	44265242	G	0.3705	8.72E-12	chr:12:44313657
12	rs1675777	44264215	A	0.3705	8.72E-12	chr:12:44313657
12	rs1675784	44257601	G	0.3705	8.72E-12	chr:12:44313657
12	rs1731439	44266559	A	0.3705	8.72E-12	chr:12:44313657
12	rs1731440	44265605	A	0.3705	8.72E-12	chr:12:44313657
12	rs2447116	44262529	G	0.3705	8.72E-12	chr:12:44313657
12	rs2951436	44261618	G	0.3705	8.72E-12	chr:12:44313657
12	rs12297031	44352609	A	0.3094	9.04E-12	chr:12:44313657
12	rs7965330	44309602	G	0.374	1.77E-11	chr:12:44313657
12	rs4768055	44317208	T	0.3382	6.00E-11	chr:12:44313657
12	rs1675782	44258841	T	0.3872	6.33E-11	chr:12:44313657
12	rs4768056	44322826	A	0.3433	7.23E-11	chr:12:44313657
12	rs1731455	44260900	G	0.3969	7.50E-11	chr:12:44313657
12	rs1057486	44260041	C	0.3546	8.94E-11	chr:12:44313657
12	rs10880587	44311477	G	0.343	9.22E-11	chr:12:44313657
12	rs1881926	44319047	C	0.343	9.22E-11	chr:12:44313657
12	rs1881927	44318922	A	0.343	9.22E-11	chr:12:44313657
12	rs1675788	44273779	G	0.3981	9.49E-11	chr:12:44313657
12	rs6582490	44329957	C	0.348	1.08E-10	chr:12:44313657
12	rs7133336	44261915	G	2.413	1.09E-10	chr:12:44313657
12	rs4768057	44332707	A	0.348	1.23E-10	chr:12:44313657
12	rs1851099	44657432	C	2.442	1.28E-10	chr:12:44313657
12	rs1731454	44251107	G	0.3585	1.31E-10	chr:12:44313657
12	rs7305609	44240652	G	0.3585	1.31E-10	chr:12:44313657
12	rs73096934	44241279	T	0.3585	1.31E-10	chr:12:44313657
12	rs11182312	44259319	G	2.407	1.36E-10	chr:12:44313657
12	rs80044299	44266866	A	2.407	1.36E-10	chr:12:44313657
12	rs7974214	44319517	C	0.3478	1.44E-10	chr:12:44313657
12	rs12307644	44273062	G	2.399	1.45E-10	chr:12:44313657
12	rs10785485	44355693	A	0.349	1.67E-10	chr:12:44313657

12	rs12307180	44256942	T	2.383	1.95E-10	chr:12:44313657
12	rs1621279	44249011	T	0.3642	2.17E-10	chr:12:44313657
12	rs1675765	44244855	C	0.3642	2.17E-10	chr:12:44313657
12	rs1675769	44248400	A	0.3642	2.17E-10	chr:12:44313657
12	rs1731450	44246041	C	0.3642	2.17E-10	chr:12:44313657
12	rs1731452	44249745	T	0.3642	2.17E-10	chr:12:44313657
12	rs554923993	44504404	A	2.449	2.26E-10	chr:12:44313657
12	rs12319776	44454041	G	3.958	2.46E-10	chr:12:44313657
12	rs56105344	44318498	T	0.3234	2.58E-10	chr:12:44313657
12	rs61932981	44332524	A	0.3234	2.58E-10	chr:12:44313657
12	rs7969372	44309996	T	0.4021	2.60E-10	chr:12:44313657
12	rs73085708	44347955	T	0.3123	2.87E-10	chr:12:44313657
12	rs7965873	44354797	G	0.3571	3.04E-10	chr:12:44313657
12	rs7971628	44353583	T	0.3571	3.04E-10	chr:12:44313657
12	rs61932976	44317050	G	0.3148	3.06E-10	chr:12:44313657
12	rs61932977	44317683	G	0.3148	3.06E-10	chr:12:44313657
12	rs2407786	44300467	G	0.4081	3.19E-10	chr:12:44313657
12	rs1731458	44285969	C	0.4104	3.53E-10	chr:12:44313657
12	rs55656444	44350421	A	0.3268	3.73E-10	chr:12:44313657
12	rs12299112	44252616	A	2.355	3.82E-10	chr:12:44313657
12	rs58915539	44320865	T	0.327	3.87E-10	chr:12:44313657
12	rs60400993	44643306	C	2.385	3.99E-10	chr:12:44313657
12	rs10748377	44308151	C	0.4118	4.45E-10	chr:12:44313657
12	rs10748378	44308153	G	0.4118	4.45E-10	chr:12:44313657
12	rs10785462	44299067	G	0.4118	4.45E-10	chr:12:44313657
12	rs10785463	44299093	C	0.4118	4.45E-10	chr:12:44313657
12	rs10785466	44308052	G	0.4118	4.45E-10	chr:12:44313657
12	rs10880585	44307982	G	0.4118	4.45E-10	chr:12:44313657
12	rs11182324	44307868	T	0.4118	4.45E-10	chr:12:44313657
12	rs11182325	44307930	C	0.4118	4.45E-10	chr:12:44313657
12	rs1358705	44287850	T	0.4118	4.45E-10	chr:12:44313657
12	rs1609549	44306994	G	0.4118	4.45E-10	chr:12:44313657
12	rs1614042	44290018	T	0.4118	4.45E-10	chr:12:44313657
12	rs1675756	44279540	C	0.4118	4.45E-10	chr:12:44313657
12	rs1675781	44292513	C	0.4118	4.45E-10	chr:12:44313657
12	rs1675789	44290545	G	0.4118	4.45E-10	chr:12:44313657
12	rs1675790	44290447	T	0.4118	4.45E-10	chr:12:44313657
12	rs1731430	44280809	C	0.4118	4.45E-10	chr:12:44313657
12	rs1731456	44286932	G	0.4118	4.45E-10	chr:12:44313657
12	rs1731457	44286499	C	0.4118	4.45E-10	chr:12:44313657
12	rs1731459	44285844	T	0.4118	4.45E-10	chr:12:44313657
12	rs2407783	44300247	C	0.4118	4.45E-10	chr:12:44313657
12	rs2407784	44300347	C	0.4118	4.45E-10	chr:12:44313657

12	rs2407785	44300358	G	0.4118	4.45E-10	chr:12:44313657
12	rs3990338	44300626	C	0.4118	4.45E-10	chr:12:44313657
12	rs4238089	44299688	G	0.4118	4.45E-10	chr:12:44313657
12	rs4238091	44299900	A	0.4118	4.45E-10	chr:12:44313657
12	rs4363674	44307404	G	0.4118	4.45E-10	chr:12:44313657
12	rs4469953	44307349	A	0.4118	4.45E-10	chr:12:44313657
12	rs4572198	44298071	T	0.4118	4.45E-10	chr:12:44313657
12	rs4572199	44298378	T	0.4118	4.45E-10	chr:12:44313657
12	rs4609659	44298550	T	0.4118	4.45E-10	chr:12:44313657
12	rs4768532	44279222	T	0.4118	4.45E-10	chr:12:44313657
12	rs4768535	44298917	T	0.4118	4.45E-10	chr:12:44313657
12	rs4768536	44298961	T	0.4118	4.45E-10	chr:12:44313657
12	rs5019975	44299370	A	0.4118	4.45E-10	chr:12:44313657
12	rs7295151	44308194	A	0.4118	4.45E-10	chr:12:44313657
12	rs11520309	44303103	G	0.4117	4.47E-10	chr:12:44313657
12	rs7974046	44306269	T	0.4122	4.50E-10	chr:12:44313657
12	rs7974045	44306266	A	0.4127	4.91E-10	chr:12:44313657
12	rs17094072	44491766	A	2.402	5.43E-10	chr:12:44313657
12	rs1610291	44307158	T	0.4131	5.70E-10	chr:12:44313657
12	rs10880579	44297491	G	0.4133	5.74E-10	chr:12:44313657
12	rs55900603	44323818	G	0.3327	5.93E-10	chr:12:44313657
12	rs4768538	44300011	G	0.4152	6.19E-10	chr:12:44313657
12	rs113686674	44600008	A	2.368	8.14E-10	chr:12:44313657
12	rs60985254	44596951	A	2.368	8.14E-10	chr:12:44313657
12	rs11830768	44524558	A	2.364	8.59E-10	chr:12:44313657
12	rs17094136	44526895	G	2.364	8.59E-10	chr:12:44313657
12	rs73288071	44525152	C	2.364	8.59E-10	chr:12:44313657
12	rs73288083	44530165	A	2.364	8.59E-10	chr:12:44313657
12	rs7954128	44484284	A	2.364	8.59E-10	chr:12:44313657
12	rs73290244	44558169	T	2.352	9.20E-10	chr:12:44313657
12	rs7956766	44277073	A	2.287	9.37E-10	chr:12:44313657
12	rs7969724	44280202	T	2.287	9.37E-10	chr:12:44313657
12	rs4768539	44303524	T	0.421	1.03E-09	chr:12:44313657
12	rs4768053	44309069	G	0.4214	1.14E-09	chr:12:44313657
12	rs73290254	44562515	C	2.346	1.15E-09	chr:12:44313657
12	rs113174127	44566796	A	2.344	1.18E-09	chr:12:44313657
12	rs113328477	44596665	T	2.344	1.18E-09	chr:12:44313657
12	rs11834090	44580711	C	2.344	1.18E-09	chr:12:44313657
12	rs7132031	44604999	T	2.344	1.18E-09	chr:12:44313657
12	rs73290243	44557607	T	2.344	1.18E-09	chr:12:44313657
12	rs73290245	44558992	T	2.344	1.18E-09	chr:12:44313657
12	rs73290256	44562879	T	2.344	1.18E-09	chr:12:44313657
12	rs73290257	44566508	C	2.344	1.18E-09	chr:12:44313657

12	rs11833168	44548432	T	2.343	1.21E-09	chr:12:44313657
12	rs2062953	44528367	T	2.341	1.23E-09	chr:12:44313657
12	rs73288033	44499108	C	2.341	1.23E-09	chr:12:44313657
12	rs73288040	44499853	T	2.341	1.23E-09	chr:12:44313657
12	rs145249872	44285415	T	2.267	1.30E-09	chr:12:44313657
12	rs12320752	44294435	T	2.269	1.32E-09	chr:12:44313657
12	rs7307888	44280981	A	2.261	1.42E-09	chr:12:44313657
12	rs114920479	44562063	C	2.339	1.44E-09	chr:12:44313657
12	rs11182358	44363335	T	2.181	1.59E-09	chr:12:44313657
12	rs73290241	44557320	G	2.321	1.69E-09	chr:12:44313657
12	rs17094348	44645530	C	2.309	1.80E-09	chr:12:44313657
12	rs7954640	44301081	T	0.4304	1.88E-09	chr:12:44313657
12	rs7972025	44222722	G	0.4432	1.88E-09	chr:12:44313657
12	rs73274135	44651511	G	2.314	2.11E-09	chr:12:44313657
12	rs11182363	44381348	C	3.466	2.39E-09	chr:12:44313657
12	rs17094194	44582003	T	2.298	2.50E-09	chr:12:44313657
12	rs74830372	44588878	A	2.296	2.53E-09	chr:12:44313657
12	rs7954130	44301327	A	0.4374	2.94E-09	chr:12:44313657
12	rs2407787	44300709	A	0.4332	3.67E-09	chr:12:44313657
12	rs11182371	44394939	C	3.379	3.75E-09	chr:12:44313657
12	rs12320155	44394433	T	3.379	3.75E-09	chr:12:44313657
12	rs12299151	44302543	G	2.206	4.03E-09	chr:12:44313657
12	rs73089738	44438342	T	2.665	4.56E-09	chr:12:44313657
12	rs73087623	44395528	T	2.816	4.67E-09	chr:12:44313657
12	rs1406786	44274673	G	2.185	5.01E-09	chr:12:44313657
12	rs79916589	44289680	C	0.4453	5.48E-09	chr:12:44313657
12	rs11838118	44667066	A	2.241	5.54E-09	chr:12:44313657
12	rs11182332	44317599	A	4.204	5.62E-09	chr:12:44313657
12	rs12296939	44426290	T	3.912	7.34E-09	chr:12:44313657
12	rs12303870	44405114	G	3.912	7.34E-09	chr:12:44313657
12	rs74389423	44418402	A	3.912	7.34E-09	chr:12:44313657
12	rs73272295	44628133	G	2.203	7.50E-09	chr:12:44313657
12	rs12303163	44409858	T	3.711	8.35E-09	chr:12:44313657
12	rs11182293	44231482	G	0.401	8.39E-09	chr:12:44313657
12	rs10880578	44291513	T	0.406	8.43E-09	chr:12:44313657
12	rs1675787	44290928	G	0.406	8.43E-09	chr:12:44313657
12	rs1731432	44279783	C	0.406	8.43E-09	chr:12:44313657
12	rs1731435	44292195	G	0.406	8.43E-09	chr:12:44313657
12	rs2447113	44282785	A	0.406	8.43E-09	chr:12:44313657
12	rs2951435	44291174	A	0.406	8.43E-09	chr:12:44313657
12	rs73085705	44345490	T	4.111	9.45E-09	chr:12:44313657
12	rs7969754	44301580	G	0.4387	9.59E-09	chr:12:44313657
12	rs4768049	44256856	G	0.4578	9.81E-09	chr:12:44313657

12	rs56973281	44625856	G	2.18	1.06E-08	chr:12:44313657
12	rs12298970	44237256	C	0.4565	1.09E-08	chr:12:44313657
12	rs1461568	44267583	C	0.4565	1.09E-08	chr:12:44313657
12	rs1731438	44267863	C	0.4565	1.09E-08	chr:12:44313657
12	rs1731448	44244270	A	0.4565	1.09E-08	chr:12:44313657
12	rs10785484	44353210	T	0.4111	1.23E-08	chr:12:44313657
12	rs10161163	44433103	C	3.6	1.28E-08	chr:12:44313657
12	rs10161301	44432919	A	3.6	1.28E-08	chr:12:44313657
12	rs11182376	44402498	G	3.6	1.28E-08	chr:12:44313657
12	rs11182389	44424153	A	3.6	1.28E-08	chr:12:44313657
12	rs114289528	44425403	C	3.6	1.28E-08	chr:12:44313657
12	rs114457407	44426674	T	3.6	1.28E-08	chr:12:44313657
12	rs114631781	44397804	A	3.6	1.28E-08	chr:12:44313657
12	rs114890225	44431883	A	3.6	1.28E-08	chr:12:44313657
12	rs115287223	44439789	G	3.6	1.28E-08	chr:12:44313657
12	rs116190624	44401433	A	3.6	1.28E-08	chr:12:44313657
12	rs12310801	44407385	A	3.6	1.28E-08	chr:12:44313657
12	rs12321149	44399814	G	3.6	1.28E-08	chr:12:44313657
12	rs76842947	44435161	T	3.6	1.28E-08	chr:12:44313657
12	rs7954384	44301587	A	0.4439	1.38E-08	chr:12:44313657
12	rs1675783	44257680	C	0.4627	1.41E-08	chr:12:44313657
12	rs1731442	44262606	C	0.4627	1.41E-08	chr:12:44313657
12	rs1731444	44262392	A	0.4627	1.41E-08	chr:12:44313657
12	rs2447114	44252015	C	0.4627	1.41E-08	chr:12:44313657
12	rs7965240	44309591	G	0.4581	1.77E-08	chr:12:44313657
12	rs138841149	44348027	G	0.3152	1.90E-08	chr:12:44313657
12	rs7961536	44674671	T	2.207	2.13E-08	chr:12:44313657
12	rs10785460	44264906	G	0.466	2.22E-08	chr:12:44313657
12	rs75523074	44635276	A	6.215	2.40E-08	chr:12:44313657
12	rs79894987	44312348	G	6.581	2.61E-08	chr:12:44313657
12	rs1731453	44250528	A	0.4776	3.10E-08	chr:12:44313657
12	rs7965635	44238903	G	0.4776	3.10E-08	chr:12:44313657
12	rs1731449	44244877	A	0.4787	3.37E-08	chr:12:44313657
12	rs115119835	44333536	G	0.3252	3.49E-08	chr:12:44313657
12	rs116245035	44323899	G	0.3252	3.49E-08	chr:12:44313657
12	rs116829442	44323913	G	0.3252	3.49E-08	chr:12:44313657
12	rs59285472	44358961	G	0.3252	3.49E-08	chr:12:44313657
12	rs7139062	44341173	G	0.3252	3.49E-08	chr:12:44313657
12	rs74811852	44380831	T	6.047	4.00E-08	chr:12:44313657
12	rs1675766	44244919	T	0.4825	4.80E-08	chr:12:44313657
12	rs11182396	44431156	A	0.4879	5.07E-08	chr:12:44313657
12	rs7955148	44301433	T	0.4339	5.18E-08	chr:12:44313657
12	rs10506233	44376031	C	2.877	5.24E-08	chr:12:44313657

12	rs114245504	44337287	G	0.3328	5.46E-08	chr:12:44313657
12	rs139815405	44421888	T	5.917	5.87E-08	chr:12:44313657
12	rs114826743	44372500	A	6.308	5.94E-08	chr:12:44313657
12	rs75539343	44369962	T	2.669	5.96E-08	chr:12:44313657
12	rs1731447	44289250	T	0.4831	6.20E-08	chr:12:44313657
12	rs1731424	44282880	G	2.033	6.25E-08	chr:12:44313657
12	rs77544607	44472001	C	5.901	6.93E-08	chr:12:44313657
12	rs80019363	44465318	C	5.901	6.93E-08	chr:12:44313657
12	rs7953229	44239350	A	0.48	6.95E-08	chr:12:44313657
12	rs151082986	44611742	T	6.717	7.87E-08	chr:12:44313657
12	rs76099678	44560102	T	6.717	7.87E-08	chr:12:44313657
12	rs76980159	44609218	G	6.717	7.87E-08	chr:12:44313657
12	rs11182413	44470922	A	5.837	8.77E-08	chr:12:44313657
12	rs1798020	44739393	G	0.4521	8.77E-08	chr:12:44313657
12	rs1623667	44249258	A	0.3799	9.26E-08	chr:12:44313657
12	rs1675767	44247135	A	0.3799	9.26E-08	chr:12:44313657
12	rs1731434	44272915	G	0.3799	9.26E-08	chr:12:44313657
12	rs7971491	44536373	A	1.986	9.62E-08	chr:12:44313657
12	rs575976526	44546500	T	6.127	9.69E-08	chr:12:44313657
12	rs1675759	44280458	T	2.015	1.02E-07	chr:12:44313657
12	rs9668657	44302174	G	0.4538	1.07E-07	chr:12:44313657
12	rs76407496	44465020	A	5.443	1.10E-07	chr:12:44313657
12	rs12313365	44433578	G	0.4899	1.33E-07	chr:12:44313657
12	rs1406787	44274699	A	0.4578	1.33E-07	chr:12:44313657
12	rs80202231	44633337	A	4.489	1.41E-07	chr:12:44313657
12	rs1675755	44278773	A	0.3785	1.48E-07	chr:12:44313657
12	rs17094615	44801627	T	4.842	1.63E-07	chr:12:44313657
12	rs11834865	44582289	G	5.298	1.72E-07	chr:12:44313657
12	rs11835241	44539509	A	5.298	1.72E-07	chr:12:44313657
12	rs7132647	44540494	T	5.298	1.72E-07	chr:12:44313657
12	rs78068362	44570637	G	5.298	1.72E-07	chr:12:44313657
12	rs78906998	44625444	A	5.298	1.72E-07	chr:12:44313657
12	rs79904675	44512645	T	5.298	1.72E-07	chr:12:44313657
12	rs78887319	44358391	G	4.943	1.89E-07	chr:12:44313657
12	rs114285001	44475343	T	5.033	1.90E-07	chr:12:44313657
12	rs1731423	44278469	A	0.4615	2.14E-07	chr:12:44313657
12	rs7968777	44493182	G	0.5325	2.39E-07	chr:12:44313657
12	rs11830711	44500400	G	4.923	2.83E-07	chr:12:44313657
12	rs142242034	44507998	T	4.923	2.83E-07	chr:12:44313657
12	rs7301959	44518177	C	4.923	2.83E-07	chr:12:44313657
12	rs80337269	44497743	G	4.923	2.83E-07	chr:12:44313657
12	rs7972687	44463896	A	5.079	3.35E-07	chr:12:44313657
12	rs1675758	44280027	A	0.3923	3.55E-07	chr:12:44313657

12	rs1731431	44279870	T	0.3923	3.55E-07	chr:12:44313657
12	rs7955615	44295861	T	0.3923	3.55E-07	chr:12:44313657
12	rs277199	44021691	C	1.944	3.59E-07	chr:12:44313657
12	rs55977905	44492668	A	0.5378	3.64E-07	chr:12:44313657
12	rs61930734	44304400	T	0.5045	3.68E-07	chr:12:44313657
12	rs116796240	44363905	G	0.3809	3.73E-07	chr:12:44313657
12	rs11830410	44322978	C	4.222	4.04E-07	chr:12:44313657
12	rs7976722	44312149	T	4.222	4.04E-07	chr:12:44313657
12	rs61933009	44363456	T	0.3312	4.15E-07	chr:12:44313657
12	rs4768054	44309743	A	2.18	4.26E-07	chr:12:44313657
12	rs277198	44019265	G	1.908	4.50E-07	chr:12:44313657
12	rs376975627	44503918	A	2.065	4.57E-07	chr:12:44313657
12	rs10880565	44146248	C	1.861	4.64E-07	chr:12:44313657
12	rs116645597	44656969	G	4.57	5.05E-07	chr:12:44313657
12	rs55782485	44492664	G	0.5436	5.30E-07	chr:12:44313657
12	rs10785467	44309433	C	0.5123	5.81E-07	chr:12:44313657
12	rs144610358	44317647	G	4.674	6.15E-07	chr:12:44313657
12	rs1731426	44281529	G	0.3281	6.30E-07	chr:12:44313657
12	rs10506230	44033769	A	0.4542	6.86E-07	chr:12:44313657
12	rs11182317	44300705	A	1.872	6.87E-07	chr:12:44313657
12	rs10082995	44232340	G	0.5203	7.04E-07	chr:12:44313657
12	rs79820024	44480564	G	2.724	7.40E-07	chr:12:44313657
12	rs1609547	44306939	T	0.3447	7.84E-07	chr:12:44313657
12	rs1675761	44288833	G	0.3447	7.84E-07	chr:12:44313657
12	rs1675763	44286872	A	0.3447	7.84E-07	chr:12:44313657
12	rs4488262	44298417	G	0.3447	7.84E-07	chr:12:44313657
12	rs7136340	44307577	G	0.3447	7.84E-07	chr:12:44313657
12	rs7294490	44307742	G	0.3447	7.84E-07	chr:12:44313657
12	rs10880616	44419249	G	1.869	8.25E-07	chr:12:44313657
12	rs10880617	44423914	T	1.869	8.25E-07	chr:12:44313657
12	rs12296857	44419592	A	1.869	8.25E-07	chr:12:44313657
12	rs184710207	44546292	A	3.864	8.32E-07	chr:12:44313657
12	rs1609548	44306947	A	0.4104	8.82E-07	chr:12:44313657
12	rs76875969	44309760	G	5.436	8.88E-07	chr:12:44313657
12	rs4238088	44299618	G	0.3335	9.20E-07	chr:12:44313657
12	rs1675775	44265524	T	0.5273	9.75E-07	chr:12:44313657
12	rs1731441	44265523	T	0.5273	9.75E-07	chr:12:44313657
12	rs12302873	44146579	T	1.826	1.05E-06	chr:12:44313657
12	rs75316382	44222578	G	8.724	1.12E-06	chr:12:44313657
12	rs551405916	44301953	C	1.731	1.18E-06	chr:12:44313657
12	rs4251483	44168884	T	8.657	1.21E-06	chr:12:44313657
12	rs76955045	44335946	T	4.729	1.25E-06	chr:12:44313657
12	rs1527318	44727080	G	0.4334	1.26E-06	chr:12:44313657

12	rs2220726	44728224	G	0.4334	1.26E-06	chr:12:44313657
12	rs73085709	44350107	C	0.3475	1.32E-06	chr:12:44313657
12	rs840773	44732795	T	0.413	1.34E-06	chr:12:44313657
12	rs10880618	44427686	G	0.4826	1.52E-06	chr:12:44313657
12	rs200335190	44303107	G	0.3759	1.59E-06	chr:12:44313657
12	rs1675764	44285480	C	0.5024	1.91E-06	chr:12:44313657
12	rs1731433	44279776	A	0.5024	1.91E-06	chr:12:44313657
12	rs4537822	44299389	A	0.5024	1.91E-06	chr:12:44313657
12	rs7307771	44729719	G	0.4481	2.10E-06	chr:12:44313657
12	rs60221557	44318175	G	0.3586	2.14E-06	chr:12:44313657
12	rs1701083	44417904	G	0.4886	2.16E-06	chr:12:44313657
12	rs75106774	44665856	G	2.267	2.19E-06	chr:12:44313657
12	rs4768546	44336686	T	0.4196	2.21E-06	chr:12:44313657
12	rs5023324	44327148	T	0.4196	2.21E-06	chr:12:44313657
12	rs7311847	44315590	G	0.4207	2.23E-06	chr:12:44313657
12	rs10880547	44037576	C	0.4748	2.24E-06	chr:12:44313657
12	rs11182194	44034656	C	0.4748	2.24E-06	chr:12:44313657
12	rs10880562	44140311	G	1.784	2.30E-06	chr:12:44313657
12	rs10880573	44226111	C	0.5151	2.35E-06	chr:12:44313657
12	rs2464103	44279211	C	0.4874	2.38E-06	chr:12:44313657
12	rs277208	44025157	T	1.82	2.40E-06	chr:12:44313657
12	rs74567299	44132881	T	2.584	2.48E-06	chr:12:44313657
12	rs2219629	44235406	C	0.5042	2.51E-06	chr:12:44313657
12	rs115050158	44582807	A	7.152	2.55E-06	chr:12:44313657
12	rs79750447	44612456	C	7.152	2.55E-06	chr:12:44313657
12	rs4768543	44308978	G	0.507	2.55E-06	chr:12:44313657
12	rs114674781	44441306	T	4.09	2.57E-06	chr:12:44313657
12	rs115273395	44406871	C	4.09	2.57E-06	chr:12:44313657
12	rs11831160	44421935	A	4.09	2.57E-06	chr:12:44313657
12	rs74842261	44442544	A	4.09	2.57E-06	chr:12:44313657
12	rs75017466	44408294	T	4.09	2.57E-06	chr:12:44313657
12	rs75524777	44442225	C	4.09	2.57E-06	chr:12:44313657
12	rs75890365	44391765	G	4.09	2.57E-06	chr:12:44313657
12	rs78434487	44379562	T	4.09	2.57E-06	chr:12:44313657
12	rs7959602	44445526	C	4.09	2.57E-06	chr:12:44313657
12	rs80285345	44404121	T	4.09	2.57E-06	chr:12:44313657
12	rs550531	44031750	C	0.568	2.66E-06	chr:12:44313657
12	rs77142311	44362408	A	5.255	2.67E-06	chr:12:44313657
12	rs79186285	44361175	T	5.255	2.67E-06	chr:12:44313657
12	rs11182242	44135219	T	0.5621	2.83E-06	chr:12:44313657
12	rs113351059	44677925	G	4.976	2.99E-06	chr:12:44313657
12	rs2120597	44035785	A	0.4794	2.99E-06	chr:12:44313657
12	rs78933883	44419447	A	3.91	3.02E-06	chr:12:44313657

12	rs76787060	44760231	G	4.696	3.06E-06	chr:12:44313657
12	rs115667463	44311743	C	5.009	3.21E-06	chr:12:44313657
12	rs1478617	44023917	G	1.8	3.33E-06	chr:12:44313657
12	rs1827427	44022936	A	1.8	3.33E-06	chr:12:44313657
12	rs202162002	44301820	G	0.4409	3.35E-06	chr:12:44313657
12	rs367898590	44301818	G	0.4409	3.35E-06	chr:12:44313657
12	rs375152458	44301817	C	0.4409	3.35E-06	chr:12:44313657
12	rs74608515	44579565	A	2.219	3.35E-06	chr:12:44313657
12	rs75656875	44589008	C	2.219	3.35E-06	chr:12:44313657
12	rs79000476	44613729	A	2.219	3.35E-06	chr:12:44313657
12	rs59165643	44764543	T	0.4311	3.37E-06	chr:12:44313657
12	rs10880545	44029301	A	0.483	3.39E-06	chr:12:44313657
12	rs79058280	44453973	T	4.022	3.43E-06	chr:12:44313657
12	rs7956667	44452878	G	4.022	3.43E-06	chr:12:44313657
12	rs2699992	44278013	T	0.5445	3.50E-06	chr:12:44313657
12	rs6582497	44476332	A	1.745	3.68E-06	chr:12:44313657
12	rs77141811	44582770	G	4.941	3.74E-06	chr:12:44313657
12	rs117447371	44754802	G	4.897	3.84E-06	chr:12:44313657
12	rs11837483	44761372	C	4.897	3.84E-06	chr:12:44313657
12	rs139453218	44739996	T	4.897	3.84E-06	chr:12:44313657
12	rs142552068	44747852	G	4.897	3.84E-06	chr:12:44313657
12	rs7310993	44739672	G	4.897	3.84E-06	chr:12:44313657
12	rs74318381	44714824	A	4.897	3.84E-06	chr:12:44313657
12	rs75368694	44723623	C	4.897	3.84E-06	chr:12:44313657
12	rs75751564	44782574	A	4.897	3.84E-06	chr:12:44313657
12	rs77126531	44715939	T	4.897	3.84E-06	chr:12:44313657
12	rs77860315	44761285	G	4.897	3.84E-06	chr:12:44313657
12	rs78725908	44717331	G	4.897	3.84E-06	chr:12:44313657
12	rs78835640	44701981	A	4.897	3.84E-06	chr:12:44313657
12	rs79008767	44695168	A	4.897	3.84E-06	chr:12:44313657
12	rs78649011	44785917	G	4.847	3.95E-06	chr:12:44313657
12	rs77526039	44047223	C	11.75	4.05E-06	chr:12:44313657
12	rs7312855	44370379	A	3.731	4.18E-06	chr:12:44313657
12	rs17094013	44421518	T	0.5598	4.22E-06	chr:12:44313657
12	rs7969016	44309726	G	0.5056	4.24E-06	chr:12:44313657
12	rs75577340	44138531	C	11.58	4.29E-06	chr:12:44313657
12	rs12310585	44133972	G	1.75	4.32E-06	chr:12:44313657
12	rs57781916	44367485	G	0.445	4.66E-06	chr:12:44313657
12	rs77894109	44685317	A	4.647	4.76E-06	chr:12:44313657
12	rs74804157	44813166	A	4.13	4.90E-06	chr:12:44313657
12	rs7979391	44295117	T	0.548	5.12E-06	chr:12:44313657
12	rs10880608	44371763	A	0.4861	5.28E-06	chr:12:44313657
12	rs3990339	44300728	A	0.5144	5.32E-06	chr:12:44313657

12	rs75931944	44424831	T	3.66	5.38E-06	chr:12:44313657
12	rs4251481	44168365	G	1.904	5.38E-06	chr:12:44313657
12	rs4251488	44169901	C	1.904	5.38E-06	chr:12:44313657
12	rs7977654	44488570	T	2.105	5.42E-06	chr:12:44313657
12	rs142670960	44436427	C	7.639	5.52E-06	chr:12:44313657
12	rs11182240	44134755	C	1.74	5.57E-06	chr:12:44313657
12	rs7961078	44306167	G	0.5522	5.79E-06	chr:12:44313657
12	rs12308611	44132831	G	1.734	5.86E-06	chr:12:44313657
12	rs74433242	44493794	G	2.159	5.87E-06	chr:12:44313657
12	rs77002463	44489358	A	2.159	5.87E-06	chr:12:44313657
12	rs79143976	44476288	G	2.159	5.87E-06	chr:12:44313657
12	rs7310784	44755510	T	4.503	6.01E-06	chr:12:44313657
12	rs11829049	44763625	C	4.502	6.03E-06	chr:12:44313657
12	rs74569781	44622787	T	7.557	6.14E-06	chr:12:44313657
12	rs10736018	44387942	A	0.4859	6.41E-06	chr:12:44313657
12	rs2407788	44388354	G	0.4859	6.41E-06	chr:12:44313657
12	rs7963981	44306066	C	0.49	6.41E-06	chr:12:44313657
12	rs10467193	44016975	A	1.764	6.76E-06	chr:12:44313657
12	rs73272217	44596334	T	1.794	6.77E-06	chr:12:44313657
12	rs7961986	44604047	A	1.794	6.77E-06	chr:12:44313657
12	rs1731421	44285207	G	0.5549	7.18E-06	chr:12:44313657
12	rs1731429	44280899	G	0.5549	7.18E-06	chr:12:44313657
12	rs2614394	44282166	G	0.5549	7.18E-06	chr:12:44313657
12	rs4238090	44299833	C	0.5549	7.18E-06	chr:12:44313657
12	rs4354753	44293620	A	0.5549	7.18E-06	chr:12:44313657
12	rs4768052	44308910	G	0.5549	7.18E-06	chr:12:44313657
12	rs4768534	44298821	C	0.5549	7.18E-06	chr:12:44313657
12	rs75766105	44721240	C	4.561	7.42E-06	chr:12:44313657
12	rs7973886	44306134	A	0.4624	7.43E-06	chr:12:44313657
12	rs73090673	44764199	T	0.4816	7.77E-06	chr:12:44313657
12	rs7973769	44306028	T	0.4915	7.81E-06	chr:12:44313657
12	rs9669053	44301883	T	0.4481	7.93E-06	chr:12:44313657
12	rs76428704	44252743	T	2.796	8.41E-06	chr:12:44313657
12	rs73272204	44594198	T	1.777	8.98E-06	chr:12:44313657
12	rs10736017	44300037	G	0.5583	9.04E-06	chr:12:44313657
12	rs60165690	44015910	C	2.602	9.47E-06	chr:12:44313657
12	rs1731427	44281449	C	0.5601	9.59E-06	chr:12:44313657
12	rs7956245	44143520	T	1.762	9.67E-06	chr:12:44313657
12	rs9971859	44140776	C	1.799	9.71E-06	chr:12:44313657
12	rs10880580	44297492	A	0.561	1.14E-05	chr:12:44313657
12	rs57291280	44146799	C	1.79	1.15E-05	chr:12:44313657
12	rs77194961	44283770	A	2.702	1.16E-05	chr:12:44313657
12	rs12318086	44143692	G	1.783	1.31E-05	chr:12:44313657

12	rs551422332	44296434	A	0.339	1.43E-05	chr:12:44313657
12	rs111562623	44284404	A	0.3479	1.45E-05	chr:12:44313657
12	rs113528455	44284575	T	0.3479	1.45E-05	chr:12:44313657
12	rs138874015	44296430	T	0.3479	1.45E-05	chr:12:44313657
12	rs151314246	44280746	T	0.3479	1.45E-05	chr:12:44313657
12	rs73098867	44295930	T	0.3479	1.45E-05	chr:12:44313657
12	rs73098870	44296033	A	0.3479	1.45E-05	chr:12:44313657
12	rs766962	44235814	C	0.4584	1.46E-05	chr:12:44313657
12	rs75443333	44053562	C	13.92	1.51E-05	chr:12:44313657
12	rs9668647	44301830	T	0.4589	1.52E-05	chr:12:44313657
12	rs17094314	44617501	G	2.002	1.56E-05	chr:12:44313657
12	rs7969835	44676227	G	2.953	1.56E-05	chr:12:44313657
12	rs7971053	55729947	C	0.03444	1.48E-26	chr:12:55727213
12	rs59466585	55722736	G	0.03436	1.50E-26	chr:12:55727213
12	rs61957887	55721734	T	0.03436	1.50E-26	chr:12:55727213
12	rs7960760	55722311	G	0.03436	1.50E-26	chr:12:55727213
12	rs7960719	55722035	A	0.03453	1.70E-26	chr:12:55727213
12	rs57757372	55718780	G	0.08933	7.29E-26	chr:12:55727213
12	rs58709231	55719090	C	0.08933	7.29E-26	chr:12:55727213
12	rs59097302	55719030	T	0.08933	7.29E-26	chr:12:55727213
12	rs60142559	55719192	G	0.08933	7.29E-26	chr:12:55727213
12	rs60220253	55717410	G	0.08933	7.29E-26	chr:12:55727213
12	rs61957879	55716971	C	0.08933	7.29E-26	chr:12:55727213
12	rs61249728	55728922	G	0.0866	1.62E-24	chr:12:55727213
12	rs11171416	55731490	A	7.164	1.86E-24	chr:12:55727213
12	rs12099811	55731200	T	7.164	1.86E-24	chr:12:55727213
12	rs55659159	55747754	C	7.164	1.86E-24	chr:12:55727213
12	rs59136392	55748204	C	7.164	1.86E-24	chr:12:55727213
12	rs59676560	55745425	T	7.164	1.86E-24	chr:12:55727213
12	rs61957898	55730423	T	7.164	1.86E-24	chr:12:55727213
12	rs61957902	55738064	G	7.164	1.86E-24	chr:12:55727213
12	rs61957924	55744100	T	7.164	1.86E-24	chr:12:55727213
12	rs61957933	55744710	T	7.164	1.86E-24	chr:12:55727213
12	rs61957936	55745719	A	7.164	1.86E-24	chr:12:55727213
12	rs7314118	55737403	A	7.164	1.86E-24	chr:12:55727213
12	rs7955088	55732963	G	7.164	1.86E-24	chr:12:55727213
12	rs11831714	55741742	A	7.047	2.88E-24	chr:12:55727213
12	rs61071395	55721228	C	0.1444	1.76E-23	chr:12:55727213
12	rs61957915	55742266	G	6.2	3.15E-22	chr:12:55727213
12	rs11835321	55725882	C	0.1951	1.41E-19	chr:12:55727213
12	rs201131895	55740725	A	0.2554	2.44E-19	chr:12:55727213
12	rs376987469	55740755	G	0.2554	2.44E-19	chr:12:55727213
12	rs77163619	55726662	G	0.1931	5.63E-19	chr:12:55727213

12	rs4759141	55733274	G	5.179	3.18E-18	chr:12:55727213
12	rs55898514	55736238	G	4.233	4.07E-18	chr:12:55727213
12	rs61957899	55731971	C	4.233	4.07E-18	chr:12:55727213
12	rs73118998	55736659	T	4.233	4.07E-18	chr:12:55727213
12	rs73119000	55736662	G	4.233	4.07E-18	chr:12:55727213
12	rs7313465	55736863	C	4.233	4.07E-18	chr:12:55727213
12	rs7484556	55738427	A	4.244	4.56E-18	chr:12:55727213
12	rs7484522	55738240	A	3.699	1.12E-16	chr:12:55727213
12	rs537017437	55742569	A	3.749	2.77E-16	chr:12:55727213
12	rs7302469	55756809	G	5.235	6.17E-16	chr:12:55727213
12	rs10876746	55741796	A	4.631	8.12E-16	chr:12:55727213
12	rs7486972	55750386	T	5.194	8.22E-16	chr:12:55727213
12	rs12823727	55766379	C	5.186	8.46E-16	chr:12:55727213
12	rs4759143	55765950	A	5.186	8.46E-16	chr:12:55727213
12	rs11171417	55731998	C	4.472	1.25E-15	chr:12:55727213
12	rs4759138	55724302	C	4.472	1.25E-15	chr:12:55727213
12	rs4575375	55721803	T	4.475	1.38E-15	chr:12:55727213
12	rs4759137	55722755	G	4.475	1.38E-15	chr:12:55727213
12	rs6421181	55767525	A	5	1.69E-15	chr:12:55727213
12	rs4759144	55769452	A	4.879	2.40E-15	chr:12:55727213
12	rs556977514	55738632	A	0.2263	1.42E-14	chr:12:55727213
12	rs7485053	55770126	C	0.2832	4.39E-14	chr:12:55727213
12	rs10876748	55749921	T	4.734	8.30E-14	chr:12:55727213
12	rs6581035	55751346	T	4.734	8.30E-14	chr:12:55727213
12	rs7979557	55753210	T	4.734	8.30E-14	chr:12:55727213
12	rs12321043	55719281	A	3.238	1.18E-13	chr:12:55727213
12	rs7488143	55771062	A	4.515	2.21E-13	chr:12:55727213
12	rs7488163	55771125	A	4.515	2.21E-13	chr:12:55727213
12	rs7488164	55771126	A	4.515	2.21E-13	chr:12:55727213
12	rs7972297	55758671	A	3.561	2.39E-12	chr:12:55727213
12	rs11171443	55766882	G	3.534	2.45E-12	chr:12:55727213
12	rs11171442	55766707	A	3.531	3.19E-12	chr:12:55727213
12	rs7314701	55763504	T	3.531	3.19E-12	chr:12:55727213
12	rs7962135	55758832	T	3.531	3.19E-12	chr:12:55727213
12	rs11171435	55758053	T	2.785	6.22E-12	chr:12:55727213
12	rs12296692	55756851	T	2.785	6.22E-12	chr:12:55727213
12	rs12318683	55755024	A	2.785	6.22E-12	chr:12:55727213
12	rs146150551	55751301	T	2.785	6.22E-12	chr:12:55727213
12	rs149898597	55753436	T	2.785	6.22E-12	chr:12:55727213
12	rs56677471	55749967	T	2.785	6.22E-12	chr:12:55727213
12	rs61957943	55749330	A	2.785	6.22E-12	chr:12:55727213
12	rs79104523	55749220	T	2.785	6.22E-12	chr:12:55727213
12	rs58480409	55719221	T	0.3219	6.88E-12	chr:12:55727213

12	rs61957878	55716754	T	0.3219	6.88E-12	chr:12:55727213
12	rs61957884	55720177	G	0.3219	6.88E-12	chr:12:55727213
12	rs61957885	55720876	G	0.3219	6.88E-12	chr:12:55727213
12	rs59347627	55758129	C	2.726	1.25E-11	chr:12:55727213
12	rs12578577	55716443	T	0.3276	1.29E-11	chr:12:55727213
12	rs57606846	55717650	G	0.2701	2.18E-11	chr:12:55727213
12	rs60439574	55716327	A	0.2701	2.18E-11	chr:12:55727213
12	rs10431495	55745942	C	4.557	3.35E-11	chr:12:55727213
12	rs10747741	55736841	C	4.557	3.35E-11	chr:12:55727213
12	rs10783726	55754954	G	4.557	3.35E-11	chr:12:55727213
12	rs4318060	55725690	T	4.557	3.35E-11	chr:12:55727213
12	rs4759139	55728454	T	4.557	3.35E-11	chr:12:55727213
12	rs6581034	55734390	T	4.557	3.35E-11	chr:12:55727213
12	rs74090652	55724831	C	4.557	3.35E-11	chr:12:55727213
12	rs7485740	55752364	G	4.557	3.35E-11	chr:12:55727213
12	rs7486500	55744545	T	4.557	3.35E-11	chr:12:55727213
12	rs7487174	55738093	T	4.557	3.35E-11	chr:12:55727213
12	rs7487798	55758491	C	4.557	3.35E-11	chr:12:55727213
12	rs7977099	55749499	A	4.557	3.35E-11	chr:12:55727213
12	rs7136402	55715837	C	4.568	3.51E-11	chr:12:55727213
12	rs57418635	55715985	C	0.2763	3.79E-11	chr:12:55727213
12	rs116376193	55735215	G	0.2745	3.83E-11	chr:12:55727213
12	rs116809705	55729540	T	0.2745	3.83E-11	chr:12:55727213
12	rs61506742	55734893	A	0.2745	3.83E-11	chr:12:55727213
12	rs74323417	55746784	T	0.2745	3.83E-11	chr:12:55727213
12	rs75098816	55720703	G	0.2745	3.83E-11	chr:12:55727213
12	rs77402184	55721781	T	0.2745	3.83E-11	chr:12:55727213
12	rs78582342	55746945	T	0.2745	3.83E-11	chr:12:55727213
12	rs79259146	55728773	A	0.2745	3.83E-11	chr:12:55727213
12	rs80243347	55728937	C	0.2747	5.14E-11	chr:12:55727213
12	rs114769789	55842426	G	0.3824	7.72E-11	chr:12:55727213
12	rs536367065	55842427	C	0.3824	7.72E-11	chr:12:55727213
12	rs7958605	55767149	G	4.31	8.59E-11	chr:12:55727213
12	rs7132600	55715048	T	4.315	9.84E-11	chr:12:55727213
12	rs55637917	55747565	C	0.3524	2.12E-10	chr:12:55727213
12	rs10747751	55788840	A	4.069	2.35E-10	chr:12:55727213
12	rs10783735	55799842	C	4.069	2.35E-10	chr:12:55727213
12	rs146854480	55752777	A	0.264	2.71E-10	chr:12:55727213
12	rs79963235	55745694	T	0.2976	2.75E-10	chr:12:55727213
12	rs75895729	55770854	C	0.2652	3.14E-10	chr:12:55727213
12	rs6581036	55757196	G	4.409	3.16E-10	chr:12:55727213
12	rs7971195	55785842	T	3.945	3.82E-10	chr:12:55727213
12	rs7311751	55748847	A	2.502	5.85E-10	chr:12:55727213

12	rs10876757	55777863	A	4.246	5.90E-10	chr:12:55727213
12	rs80178612	55756147	C	0.2673	8.52E-10	chr:12:55727213
12	rs7966609	55799349	G	3.989	1.40E-09	chr:12:55727213
12	rs58254871	55750281	A	0.3721	1.42E-09	chr:12:55727213
12	rs7311967	55756278	A	3.436	1.93E-09	chr:12:55727213
12	rs6581037	55767310	T	0.3833	3.91E-09	chr:12:55727213
12	rs12301041	55718970	T	3.472	4.40E-09	chr:12:55727213
12	rs7956183	55785890	A	3.323	4.87E-09	chr:12:55727213
12	rs6581045	55809968	G	4.351	5.90E-09	chr:12:55727213
12	rs4511372	55722251	T	3.398	6.04E-09	chr:12:55727213
12	rs7958843	55726716	A	3.398	6.04E-09	chr:12:55727213
12	rs11171411	55719478	T	3.38	7.11E-09	chr:12:55727213
12	rs7971885	55795576	T	0.4571	8.47E-09	chr:12:55727213
12	rs6421182	55834019	A	4.237	1.06E-08	chr:12:55727213
12	rs10082848	55787328	G	0.464	1.41E-08	chr:12:55727213
12	rs10783733	55797327	C	0.464	1.41E-08	chr:12:55727213
12	rs10876762	55788510	A	0.464	1.41E-08	chr:12:55727213
12	rs10876765	55789546	A	0.464	1.41E-08	chr:12:55727213
12	rs10876766	55789598	T	0.464	1.41E-08	chr:12:55727213
12	rs6581039	55790117	T	0.464	1.41E-08	chr:12:55727213
12	rs6581040	55790945	C	0.464	1.41E-08	chr:12:55727213
12	rs6581041	55790976	A	0.464	1.41E-08	chr:12:55727213
12	rs6581042	55793092	T	0.464	1.41E-08	chr:12:55727213
12	rs6581044	55802724	G	0.464	1.41E-08	chr:12:55727213
12	rs7484724	55787682	A	0.464	1.41E-08	chr:12:55727213
12	rs7484798	55791192	A	0.464	1.41E-08	chr:12:55727213
12	rs7486042	55789332	C	0.464	1.41E-08	chr:12:55727213
12	rs7486820	55793748	T	0.464	1.41E-08	chr:12:55727213
12	rs7487537	55800332	A	0.464	1.41E-08	chr:12:55727213
12	rs7488269	55792543	C	0.464	1.41E-08	chr:12:55727213
12	rs7954814	55790750	T	0.464	1.41E-08	chr:12:55727213
12	rs7967395	55794264	G	0.464	1.41E-08	chr:12:55727213
12	rs7971073	55794976	G	0.464	1.41E-08	chr:12:55727213
12	rs10783736	55799934	T	0.4631	1.48E-08	chr:12:55727213
12	rs7485079	55800382	C	0.4667	1.70E-08	chr:12:55727213
12	rs10783732	55787355	T	0.4687	1.88E-08	chr:12:55727213
12	rs7486057	55796653	C	0.4696	2.05E-08	chr:12:55727213
12	rs56241451	55734249	T	0.4504	2.98E-08	chr:12:55727213
12	rs11171467	55846623	C	4.634	4.38E-08	chr:12:55727213
12	rs79462096	55845066	A	4.445	7.65E-08	chr:12:55727213
12	rs10876777	55832783	T	2.637	2.08E-07	chr:12:55727213
12	rs116723446	55761412	G	0.3173	2.25E-07	chr:12:55727213
12	rs58178495	55780661	C	0.3167	2.25E-07	chr:12:55727213

12	rs11171430	55755254	A	1.987	2.62E-07	chr:12:55727213
12	rs11171431	55757609	G	1.987	2.62E-07	chr:12:55727213
12	rs11171432	55757696	G	1.987	2.62E-07	chr:12:55727213
12	rs11171434	55758018	C	1.987	2.62E-07	chr:12:55727213
12	rs11832308	55755506	C	1.987	2.62E-07	chr:12:55727213
12	rs13377995	55757380	C	1.987	2.62E-07	chr:12:55727213
12	rs59019100	55737459	C	0.4758	2.62E-07	chr:12:55727213
12	rs373941107	55740754	T	0.3846	2.94E-07	chr:12:55727213
12	rs79930882	55784616	C	0.3006	3.14E-07	chr:12:55727213
12	rs115530372	55749031	A	0.3245	4.02E-07	chr:12:55727213
12	rs116530165	55748782	G	0.3245	4.02E-07	chr:12:55727213
12	rs138972006	55766496	G	0.3245	4.02E-07	chr:12:55727213
12	rs75825283	55763461	C	0.3245	4.02E-07	chr:12:55727213
12	rs77870025	55757622	C	0.3245	4.02E-07	chr:12:55727213
12	rs78470098	55768374	A	0.3245	4.02E-07	chr:12:55727213
12	rs78818928	55758506	G	0.3245	4.02E-07	chr:12:55727213
12	rs61956272	55779559	T	0.4341	5.22E-07	chr:12:55727213
12	rs78378859	55867285	G	4.047	5.63E-07	chr:12:55727213
12	rs188428574	55872987	A	4.187	5.72E-07	chr:12:55727213
12	rs190999100	55878997	G	4.187	5.72E-07	chr:12:55727213
12	rs60979899	55879324	T	4.187	5.72E-07	chr:12:55727213
12	rs10783729	55763346	T	2.129	7.13E-07	chr:12:55727213
12	rs562310600	55883108	T	4.128	7.28E-07	chr:12:55727213
12	rs57742580	55879860	A	4.128	7.28E-07	chr:12:55727213
12	rs74844018	55766871	A	0.4383	7.67E-07	chr:12:55727213
12	rs10783727	55762242	T	2.126	7.84E-07	chr:12:55727213
12	rs12582005	55772837	A	0.4409	9.28E-07	chr:12:55727213
12	rs12099665	55731191	T	0.4446	9.98E-07	chr:12:55727213
12	rs12099808	55731178	A	0.4446	9.98E-07	chr:12:55727213
12	rs189794726	55743934	C	0.4446	9.98E-07	chr:12:55727213
12	rs112331945	55781657	T	0.4429	1.08E-06	chr:12:55727213
12	rs61566486	55781694	G	0.4429	1.08E-06	chr:12:55727213
12	rs11171433	55757797	C	0.41	1.12E-06	chr:12:55727213
12	rs59213907	55728911	T	0.4474	1.21E-06	chr:12:55727213
12	rs7959124	55726957	G	0.4474	1.21E-06	chr:12:55727213
12	rs115471673	55766516	G	0.4465	1.30E-06	chr:12:55727213
12	rs12579408	55764348	A	0.4465	1.30E-06	chr:12:55727213
12	rs12581916	55764409	C	0.4465	1.30E-06	chr:12:55727213
12	rs4448777	55724981	C	0.4465	1.30E-06	chr:12:55727213
12	rs4590978	55725086	T	0.4465	1.30E-06	chr:12:55727213
12	rs58780687	55736498	C	0.4465	1.30E-06	chr:12:55727213
12	rs60230359	55736550	C	0.4465	1.30E-06	chr:12:55727213
12	rs60249205	55758333	G	0.4465	1.30E-06	chr:12:55727213

12	rs60586348	55745151	T	0.4465	1.30E-06	chr:12:55727213
12	rs60774348	55757717	G	0.4465	1.30E-06	chr:12:55727213
12	rs61556775	55745640	C	0.4465	1.30E-06	chr:12:55727213
12	rs61957937	55746850	C	0.4465	1.30E-06	chr:12:55727213
12	rs61957938	55747266	G	0.4465	1.30E-06	chr:12:55727213
12	rs61957941	55748058	A	0.4465	1.30E-06	chr:12:55727213
12	rs61957946	55755778	C	0.4465	1.30E-06	chr:12:55727213
12	rs67015069	55759968	A	0.4465	1.30E-06	chr:12:55727213
12	rs73326725	55749222	C	0.4465	1.30E-06	chr:12:55727213
12	rs7489047	55759851	G	0.4465	1.30E-06	chr:12:55727213
12	rs7976828	55726450	T	0.4465	1.30E-06	chr:12:55727213
12	rs11171643	56052997	A	0.2214	1.70E-06	chr:12:55727213
12	rs61956291	55797964	C	0.4528	1.72E-06	chr:12:55727213
12	rs66902680	55724080	C	0.4513	1.74E-06	chr:12:55727213
12	rs61957914	55742041	A	0.4517	1.77E-06	chr:12:55727213
12	rs113676509	56038548	T	0.229	1.80E-06	chr:12:55727213
12	rs79728885	56040066	G	0.229	1.80E-06	chr:12:55727213
12	rs7487419	55769801	G	0.45	1.87E-06	chr:12:55727213
12	rs7132347	55714876	C	0.4492	1.97E-06	chr:12:55727213
12	rs3935215	55792401	A	0.4551	2.30E-06	chr:12:55727213
12	rs61956290	55793833	G	0.4551	2.30E-06	chr:12:55727213
12	rs61956292	55803346	C	0.4551	2.30E-06	chr:12:55727213
12	rs7485979	55789043	A	0.4551	2.30E-06	chr:12:55727213
12	rs7132076	55808469	C	2.504	2.55E-06	chr:12:55727213
12	rs66516917	55749131	G	0.5326	2.80E-06	chr:12:55727213
12	rs115729288	56046717	C	0.2415	2.84E-06	chr:12:55727213
12	rs141508718	56049219	T	0.2415	2.84E-06	chr:12:55727213
12	rs80302115	55744558	A	5.489	3.04E-06	chr:12:55727213
12	rs7132916	55715119	G	0.4529	3.05E-06	chr:12:55727213
12	rs7488530	55902618	G	2.2	3.11E-06	chr:12:55727213
12	rs563055663	55827816	C	0.4493	3.13E-06	chr:12:55727213
12	rs147375537	56039853	A	0.2301	3.29E-06	chr:12:55727213
12	rs6581048	55833861	G	2.467	3.35E-06	chr:12:55727213
12	rs7133317	55824440	T	2.467	3.35E-06	chr:12:55727213
12	rs7295016	56040859	C	0.4381	3.41E-06	chr:12:55727213
12	rs12319326	55774663	C	0.518	3.76E-06	chr:12:55727213
12	rs79072749	55797626	G	4.981	3.79E-06	chr:12:55727213
12	rs61956269	55768023	T	0.4773	4.04E-06	chr:12:55727213
12	rs61957788	55883248	A	0.5002	4.15E-06	chr:12:55727213
12	rs116160069	55712406	C	5.305	4.33E-06	chr:12:55727213
12	rs61957765	55868561	C	0.4999	4.52E-06	chr:12:55727213
12	rs67290413	55869876	C	0.4999	4.52E-06	chr:12:55727213
12	rs7294659	55866519	C	0.5004	5.15E-06	chr:12:55727213

12	rs375349885	55915679	C	2.187	5.38E-06	chr:12:55727213
12	rs112378702	56043445	T	0.2508	5.64E-06	chr:12:55727213
12	rs10876761	55786526	T	0.5465	5.67E-06	chr:12:55727213
12	rs139499289	55871562	G	4.125	5.69E-06	chr:12:55727213
12	rs76329398	55847247	T	4.847	5.98E-06	chr:12:55727213
12	rs4338633	55722153	T	2.149	6.40E-06	chr:12:55727213
12	rs114281059	55702021	A	5.145	7.23E-06	chr:12:55727213
12	rs7971600	55700241	T	5.145	7.23E-06	chr:12:55727213
12	rs6581046	55820121	C	2.052	9.22E-06	chr:12:55727213
12	rs77633032	55830899	T	4.646	9.90E-06	chr:12:55727213
12	rs56906045	55787548	T	3.273	1.06E-05	chr:12:55727213
12	rs12579346	55786856	G	0.4875	1.13E-05	chr:12:55727213
12	rs12580661	55803129	A	0.4875	1.13E-05	chr:12:55727213
12	rs12581060	55797484	C	0.4875	1.13E-05	chr:12:55727213
12	rs61956275	55786423	A	0.4875	1.13E-05	chr:12:55727213
12	rs67934812	55785591	G	0.4875	1.13E-05	chr:12:55727213
12	rs7485991	55789129	C	0.4875	1.13E-05	chr:12:55727213
12	rs7487895	55796720	C	0.4875	1.13E-05	chr:12:55727213
12	rs7488940	55802591	T	0.4875	1.13E-05	chr:12:55727213
12	rs6581038	55785441	T	0.5434	1.17E-05	chr:12:55727213
12	rs56911775	55818173	C	0.3245	1.21E-05	chr:12:55727213
12	rs74092309	55820104	A	0.3245	1.21E-05	chr:12:55727213
12	rs74092310	55820194	T	0.3245	1.21E-05	chr:12:55727213
12	rs78828715	55733980	A	3.164	1.37E-05	chr:12:55727213
12	rs60991522	55781529	T	0.4941	1.43E-05	chr:12:55727213
12	rs7959837	55734511	A	0.5575	1.46E-05	chr:12:55727213
12	rs11495497	55740640	C	0.518	1.70E-05	chr:12:55727213
12	rs12578879	55797773	T	0.4976	1.71E-05	chr:12:55727213
12	rs11830618	55789250	A	3.111	1.76E-05	chr:12:55727213
12	rs74090692	55792877	T	3.111	1.76E-05	chr:12:55727213
12	rs80247641	55797345	C	3.111	1.76E-05	chr:12:55727213
12	rs7977965	55860548	C	2.081	1.86E-05	chr:12:55727213
12	rs142811909	55987010	T	0.2195	1.89E-05	chr:12:55727213
13	rs533464869	90736937	G	33.97	6.47E-36	chr:13:90743183
13	rs7322297	90720522	A	19.27	1.62E-35	chr:13:90743183
13	rs2805699	90723189	A	24.19	3.37E-35	chr:13:90743183
13	rs113894290	90729661	C	45.26	5.56E-31	chr:13:90743183
13	rs1388190	90739471	A	55.85	3.87E-29	chr:13:90743183
13	rs75921720	90708825	C	10.11	2.12E-28	chr:13:90743183
13	rs9588850	90717634	A	7.044	2.20E-27	chr:13:90743183
13	rs2637400	90723289	T	57.03	5.04E-27	chr:13:90743183
13	rs2805701	90723284	A	57.03	5.04E-27	chr:13:90743183
13	rs78721625	90744516	T	9.842	1.02E-26	chr:13:90743183

13	rs112632269	90731067	G	9.552	1.84E-26	chr:13:90743183
13	rs2805748	90747577	A	9.215	3.58E-26	chr:13:90743183
13	rs2637401	90723306	T	68.07	4.43E-26	chr:13:90743183
13	rs2805700	90723275	A	67.21	5.73E-26	chr:13:90743183
13	rs1491024	90715598	G	6.197	7.34E-26	chr:13:90743183
13	rs9588852	90719948	G	6.197	7.34E-26	chr:13:90743183
13	rs2805746	90746594	T	66.34	7.69E-26	chr:13:90743183
13	rs2805725	90741732	G	80.28	1.46E-24	chr:13:90743183
13	rs2637428	90741395	T	79.63	1.60E-24	chr:13:90743183
13	rs2805720	90740791	A	79.63	1.60E-24	chr:13:90743183
13	rs2805722	90741285	T	79.63	1.60E-24	chr:13:90743183
13	rs10161792	90731142	A	79.36	1.67E-24	chr:13:90743183
13	rs16943826	90710255	C	9.349	2.01E-24	chr:13:90743183
13	rs77191234	90719556	G	9.124	3.20E-24	chr:13:90743183
13	rs75863885	90740389	C	57.75	3.68E-24	chr:13:90743183
13	rs2805698	90722081	G	75.49	4.80E-24	chr:13:90743183
13	rs9588859	90725395	A	73.49	7.85E-24	chr:13:90743183
13	rs2637398	90722150	C	0.1904	8.10E-24	chr:13:90743183
13	rs2637426	90741000	G	0.1996	4.65E-23	chr:13:90743183
13	rs2805728	90742666	C	0.1996	4.65E-23	chr:13:90743183
13	rs2637421	90740082	A	0.2018	9.07E-23	chr:13:90743183
13	rs10161626	90731428	C	96.07	1.11E-22	chr:13:90743183
13	rs10161628	90731522	G	96.07	1.11E-22	chr:13:90743183
13	rs10162282	90731420	G	96.07	1.11E-22	chr:13:90743183
13	rs111826912	90730412	A	96.07	1.11E-22	chr:13:90743183
13	rs112726773	90729763	G	96.07	1.11E-22	chr:13:90743183
13	rs115559522	90729810	G	96.07	1.11E-22	chr:13:90743183
13	rs1388189	90739332	A	96.07	1.11E-22	chr:13:90743183
13	rs143968735	90729784	A	96.07	1.11E-22	chr:13:90743183
13	rs1491011	90737989	C	96.07	1.11E-22	chr:13:90743183
13	rs1491013	90738361	A	96.07	1.11E-22	chr:13:90743183
13	rs150882426	90729744	G	96.07	1.11E-22	chr:13:90743183
13	rs1546054	90733331	C	96.07	1.11E-22	chr:13:90743183
13	rs1972000	90733195	T	96.07	1.11E-22	chr:13:90743183
13	rs1972001	90733095	G	96.07	1.11E-22	chr:13:90743183
13	rs2130838	90728244	A	96.07	1.11E-22	chr:13:90743183
13	rs2637402	90723934	A	96.07	1.11E-22	chr:13:90743183
13	rs2637404	90734632	T	96.07	1.11E-22	chr:13:90743183
13	rs2637406	90734868	G	96.07	1.11E-22	chr:13:90743183
13	rs2637407	90735094	C	96.07	1.11E-22	chr:13:90743183
13	rs2637408	90735266	G	96.07	1.11E-22	chr:13:90743183
13	rs2637409	90735308	G	96.07	1.11E-22	chr:13:90743183
13	rs2637411	90735572	T	96.07	1.11E-22	chr:13:90743183

13	rs2637413	90736221	T	96.07	1.11E-22	chr:13:90743183
13	rs2637414	90736401	A	96.07	1.11E-22	chr:13:90743183
13	rs2637415	90736503	T	96.07	1.11E-22	chr:13:90743183
13	rs2637416	90736597	T	96.07	1.11E-22	chr:13:90743183
13	rs2637417	90737783	T	96.07	1.11E-22	chr:13:90743183
13	rs2637418	90737912	G	96.07	1.11E-22	chr:13:90743183
13	rs2637420	90739804	G	96.07	1.11E-22	chr:13:90743183
13	rs2637422	90740092	G	96.07	1.11E-22	chr:13:90743183
13	rs2637423	90740769	G	96.07	1.11E-22	chr:13:90743183
13	rs2637425	90740968	G	96.07	1.11E-22	chr:13:90743183
13	rs2637433	90726357	G	96.07	1.11E-22	chr:13:90743183
13	rs2637443	90731918	C	96.07	1.11E-22	chr:13:90743183
13	rs2637445	90731659	A	96.07	1.11E-22	chr:13:90743183
13	rs2637446	90731613	G	96.07	1.11E-22	chr:13:90743183
13	rs2637452	90753085	G	96.07	1.11E-22	chr:13:90743183
13	rs2637455	90729280	C	96.07	1.11E-22	chr:13:90743183
13	rs2637458	90747295	T	96.07	1.11E-22	chr:13:90743183
13	rs2637459	90729037	A	96.07	1.11E-22	chr:13:90743183
13	rs2637461	90745275	A	96.07	1.11E-22	chr:13:90743183
13	rs2637462	90745244	C	96.07	1.11E-22	chr:13:90743183
13	rs2637463	90744056	A	96.07	1.11E-22	chr:13:90743183
13	rs2637464	90728584	G	96.07	1.11E-22	chr:13:90743183
13	rs2637466	90728427	C	96.07	1.11E-22	chr:13:90743183
13	rs2637467	90728379	A	96.07	1.11E-22	chr:13:90743183
13	rs2637476	90727846	T	96.07	1.11E-22	chr:13:90743183
13	rs2805633	90755098	T	96.07	1.11E-22	chr:13:90743183
13	rs2805703	90724219	T	96.07	1.11E-22	chr:13:90743183
13	rs2805706	90727892	A	96.07	1.11E-22	chr:13:90743183
13	rs2805707	90729005	A	96.07	1.11E-22	chr:13:90743183
13	rs2805708	90731585	A	96.07	1.11E-22	chr:13:90743183
13	rs2805710	90732260	T	96.07	1.11E-22	chr:13:90743183
13	rs2805711	90734616	T	96.07	1.11E-22	chr:13:90743183
13	rs2805712	90734708	A	96.07	1.11E-22	chr:13:90743183
13	rs2805713	90736264	T	96.07	1.11E-22	chr:13:90743183
13	rs2805714	90736468	T	96.07	1.11E-22	chr:13:90743183
13	rs2805715	90737729	G	96.07	1.11E-22	chr:13:90743183
13	rs2805716	90738789	A	96.07	1.11E-22	chr:13:90743183
13	rs2805717	90739820	T	96.07	1.11E-22	chr:13:90743183
13	rs2805718	90739830	T	96.07	1.11E-22	chr:13:90743183
13	rs2805719	90740285	T	96.07	1.11E-22	chr:13:90743183
13	rs2805721	90740809	C	96.07	1.11E-22	chr:13:90743183
13	rs2805724	90741683	T	96.07	1.11E-22	chr:13:90743183
13	rs2805727	90742608	C	96.07	1.11E-22	chr:13:90743183

13	rs2805729	90743323	T	96.07	1.11E-22	chr:13:90743183
13	rs2805730	90743490	A	96.07	1.11E-22	chr:13:90743183
13	rs2805731	90743620	C	96.07	1.11E-22	chr:13:90743183
13	rs2805732	90744055	C	96.07	1.11E-22	chr:13:90743183
13	rs2805733	90744227	G	96.07	1.11E-22	chr:13:90743183
13	rs2805734	90744235	A	96.07	1.11E-22	chr:13:90743183
13	rs2805735	90744305	A	96.07	1.11E-22	chr:13:90743183
13	rs2805736	90744645	T	96.07	1.11E-22	chr:13:90743183
13	rs2805738	90745081	G	96.07	1.11E-22	chr:13:90743183
13	rs2805739	90745491	T	96.07	1.11E-22	chr:13:90743183
13	rs2805741	90745796	A	96.07	1.11E-22	chr:13:90743183
13	rs2805743	90746158	G	96.07	1.11E-22	chr:13:90743183
13	rs2805747	90747181	A	96.07	1.11E-22	chr:13:90743183
13	rs2805750	90747917	C	96.07	1.11E-22	chr:13:90743183
13	rs2805754	90748891	G	96.07	1.11E-22	chr:13:90743183
13	rs2805755	90749022	T	96.07	1.11E-22	chr:13:90743183
13	rs2805758	90749261	A	96.07	1.11E-22	chr:13:90743183
13	rs2805759	90749514	A	96.07	1.11E-22	chr:13:90743183
13	rs2805761	90750031	G	96.07	1.11E-22	chr:13:90743183
13	rs28495463	90734124	A	96.07	1.11E-22	chr:13:90743183
13	rs28508421	90743437	T	96.07	1.11E-22	chr:13:90743183
13	rs73591319	90725907	A	96.07	1.11E-22	chr:13:90743183
13	rs73591321	90727136	G	96.07	1.11E-22	chr:13:90743183
13	rs76998954	90743210	A	96.07	1.11E-22	chr:13:90743183
13	rs78067562	90743391	C	96.07	1.11E-22	chr:13:90743183
13	rs9588860	90727484	G	96.07	1.11E-22	chr:13:90743183
13	rs9588861	90727552	A	96.07	1.11E-22	chr:13:90743183
13	rs79785623	90752549	T	9.184	1.24E-22	chr:13:90743183
13	rs2805627	90752272	A	95.4	1.30E-22	chr:13:90743183
13	rs2805740	90745675	A	94.92	1.39E-22	chr:13:90743183
13	rs2637439	90732217	A	0.2039	1.66E-22	chr:13:90743183
13	rs2805704	90727635	G	0.208	2.12E-22	chr:13:90743183
13	rs73593652	90755595	A	91.64	2.66E-22	chr:13:90743183
13	rs80233688	90723496	G	10.65	5.12E-21	chr:13:90743183
13	rs78975634	90720218	G	10.46	1.10E-20	chr:13:90743183
13	rs116763682	90743619	A	8.908	1.19E-20	chr:13:90743183
13	rs74507286	90738994	T	8.908	1.19E-20	chr:13:90743183
13	rs75805202	90735664	G	8.908	1.19E-20	chr:13:90743183
13	rs78645261	90754325	G	8.908	1.19E-20	chr:13:90743183
13	rs79110442	90748308	G	8.908	1.19E-20	chr:13:90743183
13	rs76202013	90714744	C	8.741	2.59E-20	chr:13:90743183
13	rs76936079	90744717	T	11.21	5.60E-20	chr:13:90743183
13	rs2805689	90715153	T	0.2522	1.54E-18	chr:13:90743183

13	rs115029999	90669505	G	8.046	4.28E-18	chr:13:90743183
13	rs75635330	90668043	C	8.046	4.28E-18	chr:13:90743183
13	rs112945355	90680625	T	7.821	1.01E-17	chr:13:90743183
13	rs113169741	90685650	A	7.821	1.01E-17	chr:13:90743183
13	rs114498245	90667379	A	7.821	1.01E-17	chr:13:90743183
13	rs137989181	90676229	T	7.821	1.01E-17	chr:13:90743183
13	rs1987095	90681873	C	7.821	1.01E-17	chr:13:90743183
13	rs1987156	90681662	A	7.821	1.01E-17	chr:13:90743183
13	rs57346044	90704533	C	7.821	1.01E-17	chr:13:90743183
13	rs74565296	90691322	T	7.821	1.01E-17	chr:13:90743183
13	rs75410608	90693188	A	7.821	1.01E-17	chr:13:90743183
13	rs76901628	90699709	C	7.821	1.01E-17	chr:13:90743183
13	rs78545676	90677712	G	7.821	1.01E-17	chr:13:90743183
13	rs78585383	90673119	T	7.821	1.01E-17	chr:13:90743183
13	rs78693129	90673027	T	7.821	1.01E-17	chr:13:90743183
13	rs79671279	90682347	T	7.821	1.01E-17	chr:13:90743183
13	rs7985369	90671522	A	7.821	1.01E-17	chr:13:90743183
13	rs80014975	90678526	G	7.821	1.01E-17	chr:13:90743183
13	rs2350299	90728500	T	0.2789	1.54E-16	chr:13:90743183
13	rs2637419	90738557	A	0.2789	1.54E-16	chr:13:90743183
13	rs2637453	90752931	A	0.2789	1.54E-16	chr:13:90743183
13	rs2637456	90749441	C	0.2789	1.54E-16	chr:13:90743183
13	rs2637460	90745311	T	0.2789	1.54E-16	chr:13:90743183
13	rs2805628	90752802	T	0.2789	1.54E-16	chr:13:90743183
13	rs2805629	90753329	C	0.2789	1.54E-16	chr:13:90743183
13	rs2805631	90753606	A	0.2789	1.54E-16	chr:13:90743183
13	rs2805737	90745031	C	0.2789	1.54E-16	chr:13:90743183
13	rs2805751	90748336	T	0.2789	1.54E-16	chr:13:90743183
13	rs2805752	90748567	A	0.2789	1.54E-16	chr:13:90743183
13	rs2805753	90748842	A	0.2789	1.54E-16	chr:13:90743183
13	rs2805756	90749045	T	0.2789	1.54E-16	chr:13:90743183
13	rs9522840	90726082	A	0.2789	1.54E-16	chr:13:90743183
13	rs2637399	90722860	C	0.2816	2.20E-16	chr:13:90743183
13	rs7989242	90746294	T	6.503	3.58E-16	chr:13:90743183
13	rs80247605	90664690	G	4.121	3.05E-15	chr:13:90743183
13	rs16943737	90663135	G	5.226	3.18E-15	chr:13:90743183
13	rs41322051	90646840	G	5.226	3.18E-15	chr:13:90743183
13	rs41361347	90652322	G	5.226	3.18E-15	chr:13:90743183
13	rs74387961	90647017	A	5.226	3.18E-15	chr:13:90743183
13	rs74422803	90653835	T	5.226	3.18E-15	chr:13:90743183
13	rs74568779	90660687	A	5.226	3.18E-15	chr:13:90743183
13	rs76111549	90651195	A	5.226	3.18E-15	chr:13:90743183
13	rs76925939	90646777	T	5.226	3.18E-15	chr:13:90743183

13	rs77555222	90662826	C	5.226	3.18E-15	chr:13:90743183
13	rs78076983	90651105	G	5.226	3.18E-15	chr:13:90743183
13	rs78172895	90663325	A	5.226	3.18E-15	chr:13:90743183
13	rs78641456	90653738	G	5.226	3.18E-15	chr:13:90743183
13	rs78680587	90654218	A	5.226	3.18E-15	chr:13:90743183
13	rs78905293	90647677	T	5.226	3.18E-15	chr:13:90743183
13	rs78992908	90651318	A	5.226	3.18E-15	chr:13:90743183
13	rs79494540	90648492	C	5.226	3.18E-15	chr:13:90743183
13	rs79603316	90660753	A	5.226	3.18E-15	chr:13:90743183
13	rs80350675	90647983	G	5.226	3.18E-15	chr:13:90743183
13	rs4142563	90722674	C	0.2827	6.64E-15	chr:13:90743183
13	rs78078484	90646090	A	5.036	6.94E-15	chr:13:90743183
13	rs2259699	90724908	G	0.3077	6.16E-14	chr:13:90743183
13	rs2805634	90755657	C	0.3077	6.16E-14	chr:13:90743183
13	rs2805686	90714000	T	0.2983	1.24E-13	chr:13:90743183
13	rs9560516	90648080	G	4.903	2.93E-13	chr:13:90743183
13	rs16943738	90664242	G	4.211	3.39E-13	chr:13:90743183
13	rs77851009	90633937	A	4.513	8.28E-13	chr:13:90743183
13	rs7330958	90724514	C	0.3205	1.33E-12	chr:13:90743183
13	rs9515679	90723917	C	0.3205	1.33E-12	chr:13:90743183
13	rs12858780	90735185	C	0.3206	1.47E-12	chr:13:90743183
13	rs1491012	90738253	T	0.3206	1.47E-12	chr:13:90743183
13	rs1586740	90747887	G	0.3206	1.47E-12	chr:13:90743183
13	rs2350300	90733811	C	0.3206	1.47E-12	chr:13:90743183
13	rs35414148	90749088	G	0.3206	1.47E-12	chr:13:90743183
13	rs3864991	90752677	A	0.3206	1.47E-12	chr:13:90743183
13	rs4093505	90755052	T	0.3206	1.47E-12	chr:13:90743183
13	rs4357809	90752644	A	0.3206	1.47E-12	chr:13:90743183
13	rs4773550	90754699	G	0.3206	1.47E-12	chr:13:90743183
13	rs7992099	90737810	C	0.3206	1.47E-12	chr:13:90743183
13	rs7994352	90737852	G	0.3206	1.47E-12	chr:13:90743183
13	rs9515680	90726064	A	0.3206	1.47E-12	chr:13:90743183
13	rs9515684	90735792	G	0.3206	1.47E-12	chr:13:90743183
13	rs9522843	90735691	C	0.3206	1.47E-12	chr:13:90743183
13	rs9522844	90738863	C	0.3206	1.47E-12	chr:13:90743183
13	rs9560547	90725963	C	0.3206	1.47E-12	chr:13:90743183
13	rs2262007	90719805	G	0.3134	1.49E-12	chr:13:90743183
13	rs2350296	90718492	T	0.3134	1.49E-12	chr:13:90743183
13	rs2805696	90720160	G	0.317	2.15E-12	chr:13:90743183
13	rs2637412	90735700	T	0.3227	3.69E-12	chr:13:90743183
13	rs2805705	90727717	G	0.3227	3.69E-12	chr:13:90743183
13	rs6492470	90731362	A	0.3227	3.69E-12	chr:13:90743183
13	rs7332177	90725070	T	0.3227	3.69E-12	chr:13:90743183

13	rs2805757	90749182	T	0.3251	4.80E-12	chr:13:90743183
13	rs1361601	90651537	T	2.966	4.91E-12	chr:13:90743183
13	rs1417931	90652059	C	2.966	4.91E-12	chr:13:90743183
13	rs1417932	90651802	C	2.966	4.91E-12	chr:13:90743183
13	rs1857016	90653003	C	2.966	4.91E-12	chr:13:90743183
13	rs1935181	90649956	C	2.966	4.91E-12	chr:13:90743183
13	rs1935182	90649766	T	2.966	4.91E-12	chr:13:90743183
13	rs2805658	90652699	A	2.966	4.91E-12	chr:13:90743183
13	rs2805659	90652722	A	2.966	4.91E-12	chr:13:90743183
13	rs4773545	90650689	T	2.966	4.91E-12	chr:13:90743183
13	rs6492467	90646581	T	2.966	4.91E-12	chr:13:90743183
13	rs6492468	90650308	T	2.966	4.91E-12	chr:13:90743183
13	rs6492469	90650326	A	2.966	4.91E-12	chr:13:90743183
13	rs7987787	90648902	A	2.966	4.91E-12	chr:13:90743183
13	rs7986744	90693163	T	2.559	6.44E-12	chr:13:90743183
13	rs1388186	90720423	T	0.332	7.29E-12	chr:13:90743183
13	rs4771804	90756276	A	0.3304	8.08E-12	chr:13:90743183
13	rs2805632	90753714	A	0.3293	9.08E-12	chr:13:90743183
13	rs13378305	90713920	A	6.119	9.75E-12	chr:13:90743183
13	rs13378483	90717030	A	6.119	9.75E-12	chr:13:90743183
13	rs9583777	90715968	G	6.119	9.75E-12	chr:13:90743183
13	rs9583778	90716191	T	6.119	9.75E-12	chr:13:90743183
13	rs9588846	90710793	G	6.119	9.75E-12	chr:13:90743183
13	rs10161983	90732800	C	6.118	1.02E-11	chr:13:90743183
13	rs10161992	90733101	A	6.118	1.02E-11	chr:13:90743183
13	rs111863575	90726069	A	6.118	1.02E-11	chr:13:90743183
13	rs143776399	90746276	G	6.118	1.02E-11	chr:13:90743183
13	rs28693886	90743678	A	6.118	1.02E-11	chr:13:90743183
13	rs73591334	90729098	G	6.118	1.02E-11	chr:13:90743183
13	rs9583782	90726722	T	6.118	1.02E-11	chr:13:90743183
13	rs9588865	90740747	A	6.118	1.02E-11	chr:13:90743183
13	rs9588868	90752403	G	6.118	1.02E-11	chr:13:90743183
13	rs1341647	90653212	G	2.867	1.12E-11	chr:13:90743183
13	rs77736962	90558819	G	4.252	1.31E-11	chr:13:90743183
13	rs8001217	90717865	T	0.3385	1.47E-11	chr:13:90743183
13	rs9515678	90718301	G	0.3385	1.47E-11	chr:13:90743183
13	rs9522836	90718114	A	0.3385	1.47E-11	chr:13:90743183
13	rs1578539	90647385	A	2.841	1.56E-11	chr:13:90743183
13	rs7987946	90648926	A	2.841	1.56E-11	chr:13:90743183
13	rs7987947	90648931	A	2.841	1.56E-11	chr:13:90743183
13	rs9522811	90647140	C	2.841	1.56E-11	chr:13:90743183
13	rs16943521	90522552	T	4.199	1.66E-11	chr:13:90743183
13	rs143214514	90539432	T	4.151	1.67E-11	chr:13:90743183

13	rs9583783	90742832	T	5.837	1.75E-11	chr:13:90743183
13	rs1388200	90712935	C	2.419	2.70E-11	chr:13:90743183
13	rs4379942	90821658	C	3.214	4.34E-11	chr:13:90743183
13	rs7982527	90819058	G	3.214	4.34E-11	chr:13:90743183
13	rs13378373	90722854	C	5.072	8.25E-11	chr:13:90743183
13	rs80012736	90756512	C	5.148	8.42E-11	chr:13:90743183
13	rs73593622	90745520	A	5.923	1.57E-10	chr:13:90743183
13	rs73593624	90746672	T	5.923	1.57E-10	chr:13:90743183
13	rs8000208	90738628	G	5.923	1.57E-10	chr:13:90743183
13	rs9588869	90754103	G	5.923	1.57E-10	chr:13:90743183
13	rs9588853	90721316	T	0.3372	2.14E-10	chr:13:90743183
13	rs1341654	90661565	A	2.824	2.53E-10	chr:13:90743183
13	rs74381025	90532314	T	3.384	6.13E-10	chr:13:90743183
13	rs140305879	90539479	A	3.329	6.60E-10	chr:13:90743183
13	rs9583774	90702237	C	5.162	8.10E-10	chr:13:90743183
13	rs2637482	90708243	C	0.4385	1.77E-09	chr:13:90743183
13	rs73591378	90736516	G	7.69	2.19E-09	chr:13:90743183
13	rs75979017	90631052	T	6.912	2.22E-09	chr:13:90743183
13	rs6492463	90630714	A	2.393	3.13E-09	chr:13:90743183
13	rs6492464	90630756	A	2.393	3.13E-09	chr:13:90743183
13	rs6492465	90630793	T	2.393	3.13E-09	chr:13:90743183
13	rs12323116	90788866	T	2.299	3.23E-09	chr:13:90743183
13	rs73591393	90740254	G	7.492	3.73E-09	chr:13:90743183
13	rs73593648	90754798	G	7.492	3.73E-09	chr:13:90743183
13	rs67627188	90791970	G	2.814	5.87E-09	chr:13:90743183
13	rs6492466	90642785	T	2.432	6.76E-09	chr:13:90743183
13	rs7330798	90643122	T	2.432	6.76E-09	chr:13:90743183
13	rs7336804	90643171	G	2.432	6.76E-09	chr:13:90743183
13	rs9555961	90643256	C	2.432	6.76E-09	chr:13:90743183
13	rs66977832	90814025	T	0.4474	7.83E-09	chr:13:90743183
13	rs34788777	90640624	G	2.625	7.93E-09	chr:13:90743183
13	rs7329747	90643225	A	2.379	9.73E-09	chr:13:90743183
13	rs9555962	90643465	T	2.379	9.73E-09	chr:13:90743183
13	rs115378073	90550877	C	8.813	1.02E-08	chr:13:90743183
13	rs11620232	90642326	G	2.565	1.34E-08	chr:13:90743183
13	rs11841913	90637307	T	2.565	1.34E-08	chr:13:90743183
13	rs11842101	90633545	G	2.565	1.34E-08	chr:13:90743183
13	rs12855674	90638634	G	2.565	1.34E-08	chr:13:90743183
13	rs12857525	90638814	G	2.565	1.34E-08	chr:13:90743183
13	rs35159467	90631767	A	2.565	1.34E-08	chr:13:90743183
13	rs2637430	90769517	C	2.683	1.36E-08	chr:13:90743183
13	rs190443016	90628332	C	8.033	1.40E-08	chr:13:90743183
13	rs73589586	90700899	T	8.109	1.62E-08	chr:13:90743183

13	rs36107022	90633068	G	2.265	1.74E-08	chr:13:90743183
13	rs7322351	90639552	T	2.523	2.31E-08	chr:13:90743183
13	rs11617103	90631248	T	2.252	2.45E-08	chr:13:90743183
13	rs11840615	90633462	T	2.252	2.45E-08	chr:13:90743183
13	rs11842409	90633533	C	2.252	2.45E-08	chr:13:90743183
13	rs33978002	90631956	C	2.252	2.45E-08	chr:13:90743183
13	rs34913089	90631879	A	2.252	2.45E-08	chr:13:90743183
13	rs35389000	90631733	A	2.252	2.45E-08	chr:13:90743183
13	rs35478160	90631588	A	2.252	2.45E-08	chr:13:90743183
13	rs35544140	90632802	C	2.252	2.45E-08	chr:13:90743183
13	rs36035336	90632808	G	2.252	2.45E-08	chr:13:90743183
13	rs36088073	90632180	G	2.252	2.45E-08	chr:13:90743183
13	rs4483712	90631681	T	2.252	2.45E-08	chr:13:90743183
13	rs67925817	90634231	C	2.252	2.45E-08	chr:13:90743183
13	rs75355243	90632450	C	2.252	2.45E-08	chr:13:90743183
13	rs2637451	90756983	T	2.2	3.48E-08	chr:13:90743183
13	rs11842540	90634897	G	2.484	3.49E-08	chr:13:90743183
13	rs113533011	90712744	T	6.339	3.49E-08	chr:13:90743183
13	rs7332839	90552147	A	2.604	3.52E-08	chr:13:90743183
13	rs9560548	90732315	G	0.3697	4.49E-08	chr:13:90743183
13	rs138876275	90584825	T	3.95	5.30E-08	chr:13:90743183
13	rs150248833	90584824	T	3.95	5.30E-08	chr:13:90743183
13	rs73590700	90585491	A	3.95	5.30E-08	chr:13:90743183
13	rs73590702	90586548	T	3.95	5.30E-08	chr:13:90743183
13	rs73592606	90591394	G	3.95	5.30E-08	chr:13:90743183
13	rs6492462	90630593	G	2.203	5.49E-08	chr:13:90743183
13	rs11616837	90550930	C	2.54	5.88E-08	chr:13:90743183
13	rs34177160	90557890	A	2.54	5.88E-08	chr:13:90743183
13	rs73589556	90667440	G	6.18	5.99E-08	chr:13:90743183
13	rs141481669	90536135	C	2.541	6.62E-08	chr:13:90743183
13	rs16943606	90568012	C	2.511	7.11E-08	chr:13:90743183
13	rs34025086	90561699	T	2.511	7.11E-08	chr:13:90743183
13	rs35016897	90561702	C	2.511	7.11E-08	chr:13:90743183
13	rs7491874	90564889	A	2.511	7.11E-08	chr:13:90743183
13	rs4365161	90727687	G	0.3762	7.27E-08	chr:13:90743183
13	rs4630407	90727551	T	0.3762	7.27E-08	chr:13:90743183
13	rs7322597	90727900	T	0.3762	7.27E-08	chr:13:90743183
13	rs7323161	90728164	G	0.3762	7.27E-08	chr:13:90743183
13	rs764152	90739700	C	0.3762	7.27E-08	chr:13:90743183
13	rs9555976	90725792	A	0.3762	7.27E-08	chr:13:90743183
13	rs9560549	90732413	T	0.3762	7.27E-08	chr:13:90743183
13	rs73594629	90613906	A	3.831	7.85E-08	chr:13:90743183
13	rs11843666	90541866	C	2.506	8.27E-08	chr:13:90743183

13	rs12860268	90540632	A	2.506	8.27E-08	chr:13:90743183
13	rs72635728	90549311	C	2.506	8.27E-08	chr:13:90743183
13	rs35536220	90721995	T	0.3331	8.76E-08	chr:13:90743183
13	rs35818222	90721996	T	0.3331	8.76E-08	chr:13:90743183
13	rs67813670	90635824	T	2.373	8.94E-08	chr:13:90743183
13	rs112268157	90602971	C	3.8	9.03E-08	chr:13:90743183
13	rs7332183	90690405	C	5.724	1.01E-07	chr:13:90743183
13	rs11841910	90637232	T	2.347	1.08E-07	chr:13:90743183
13	rs73592671	90594126	G	3.825	1.11E-07	chr:13:90743183
13	rs3962877	90743898	G	0.3854	1.20E-07	chr:13:90743183
13	rs3962897	90743725	G	0.3854	1.20E-07	chr:13:90743183
13	rs4033089	90743876	A	0.3854	1.20E-07	chr:13:90743183
13	rs4142564	90742508	G	0.3854	1.20E-07	chr:13:90743183
13	rs4397974	90744196	A	0.3854	1.20E-07	chr:13:90743183
13	rs4575402	90759420	G	0.4795	1.20E-07	chr:13:90743183
13	rs56085483	90742927	C	0.3854	1.20E-07	chr:13:90743183
13	rs9560552	90741176	T	0.3854	1.20E-07	chr:13:90743183
13	rs67849531	90635826	C	2.363	1.22E-07	chr:13:90743183
13	rs9560555	90759323	C	0.4808	1.25E-07	chr:13:90743183
13	rs112923054	90648444	G	6.365	1.30E-07	chr:13:90743183
13	rs113736761	90664093	G	6.365	1.30E-07	chr:13:90743183
13	rs114082106	90655972	A	6.365	1.30E-07	chr:13:90743183
13	rs114844703	90655971	T	6.365	1.30E-07	chr:13:90743183
13	rs73589536	90649073	T	6.365	1.30E-07	chr:13:90743183
13	rs73589537	90650192	G	6.365	1.30E-07	chr:13:90743183
13	rs11069955	90636907	C	2.326	1.41E-07	chr:13:90743183
13	rs11839010	90637082	T	2.326	1.41E-07	chr:13:90743183
13	rs35140659	90635765	G	2.326	1.41E-07	chr:13:90743183
13	rs7318758	90636318	A	2.326	1.41E-07	chr:13:90743183
13	rs7323349	90636352	A	2.326	1.41E-07	chr:13:90743183
13	rs7323973	90636677	A	2.326	1.41E-07	chr:13:90743183
13	rs9560506	90637400	T	2.201	1.62E-07	chr:13:90743183
13	rs73589592	90705949	T	5.345	1.68E-07	chr:13:90743183
13	rs12861166	90640920	G	2.136	1.78E-07	chr:13:90743183
13	rs12866996	90640832	A	2.136	1.78E-07	chr:13:90743183
13	rs12867329	90641063	C	2.136	1.78E-07	chr:13:90743183
13	rs34234059	90640734	C	2.136	1.78E-07	chr:13:90743183
13	rs34411621	90641297	A	2.136	1.78E-07	chr:13:90743183
13	rs34813353	90640801	A	2.136	1.78E-07	chr:13:90743183
13	rs4145191	90639915	G	2.136	1.78E-07	chr:13:90743183
13	rs4145192	90639854	T	2.136	1.78E-07	chr:13:90743183
13	rs7320872	90639825	A	2.136	1.78E-07	chr:13:90743183
13	rs9555960	90640282	G	2.136	1.78E-07	chr:13:90743183

13	rs9560512	90641900	G	2.136	1.78E-07	chr:13:90743183
13	rs4512967	90759397	T	0.4865	1.96E-07	chr:13:90743183
13	rs12585871	90736906	G	0.3788	2.10E-07	chr:13:90743183
13	rs9583784	90745973	C	0.3788	2.10E-07	chr:13:90743183
13	rs7993692	90642252	G	2.129	2.17E-07	chr:13:90743183
13	rs7320537	90639720	C	2.109	2.21E-07	chr:13:90743183
13	rs9588800	90592361	A	0.4345	2.25E-07	chr:13:90743183
13	rs7984840	90719301	T	0.3866	2.31E-07	chr:13:90743183
13	rs4145193	90640172	G	2.117	2.39E-07	chr:13:90743183
13	rs111267159	90682583	T	4.926	2.69E-07	chr:13:90743183
13	rs7981899	90713975	G	0.3564	2.74E-07	chr:13:90743183
13	rs73591316	90724951	A	0.3831	2.83E-07	chr:13:90743183
13	rs111593143	90749467	G	10.42	3.17E-07	chr:13:90743183
13	rs1539155	90544523	C	0.5084	3.81E-07	chr:13:90743183
13	rs1578536	90543914	T	0.5084	3.81E-07	chr:13:90743183
13	rs2637449	90759178	A	2.437	5.45E-07	chr:13:90743183
13	rs9583779	90717701	G	0.3623	5.55E-07	chr:13:90743183
13	rs11618576	90531160	A	1.98	5.81E-07	chr:13:90743183
13	rs73593654	90756729	C	0.3984	5.94E-07	chr:13:90743183
13	rs9555953	90537427	T	0.5138	6.43E-07	chr:13:90743183
13	rs9515657	90557478	T	1.935	6.57E-07	chr:13:90743183
13	rs9560546	90720086	T	0.3942	6.66E-07	chr:13:90743183
13	rs12872730	90628330	T	2.231	7.38E-07	chr:13:90743183
13	rs1907428	90718633	C	0.3945	7.43E-07	chr:13:90743183
13	rs7992865	90709499	G	0.3945	7.43E-07	chr:13:90743183
13	rs9560544	90716307	C	0.3945	7.43E-07	chr:13:90743183
13	rs1388192	90767388	A	1.96	7.77E-07	chr:13:90743183
13	rs34711780	90536294	T	1.985	7.90E-07	chr:13:90743183
13	rs11619601	90630160	G	2.203	7.92E-07	chr:13:90743183
13	rs73590694	90584912	G	8.591	8.29E-07	chr:13:90743183
13	rs73594605	90606905	T	8.591	8.29E-07	chr:13:90743183
13	rs73589555	90665968	G	3.548	8.87E-07	chr:13:90743183
13	rs113523606	90770034	C	4.226	9.04E-07	chr:13:90743183
13	rs73593674	90764791	A	4.226	9.04E-07	chr:13:90743183
13	rs73593695	90769565	T	4.226	9.04E-07	chr:13:90743183
13	rs7981055	90765430	T	4.226	9.04E-07	chr:13:90743183
13	rs7997171	90764661	C	4.226	9.04E-07	chr:13:90743183
13	rs9515656	90556978	A	1.898	9.83E-07	chr:13:90743183
13	rs9522839	90725273	A	0.4063	9.98E-07	chr:13:90743183
13	rs950956	90581710	C	2.199	1.02E-06	chr:13:90743183
13	rs2637429	90765946	G	1.948	1.05E-06	chr:13:90743183
13	rs2637431	90773811	A	0.4664	1.08E-06	chr:13:90743183
13	rs971011	90777278	A	0.4664	1.08E-06	chr:13:90743183

13	rs34721005	90628659	C	2.204	1.11E-06	chr:13:90743183
13	rs9560508	90637437	C	2.021	1.28E-06	chr:13:90743183
13	rs2637432	90774493	A	0.4692	1.31E-06	chr:13:90743183
13	rs2637437	90776365	C	0.4692	1.31E-06	chr:13:90743183
13	rs2637440	90777847	C	0.4692	1.31E-06	chr:13:90743183
13	rs724793	90778898	C	0.4692	1.31E-06	chr:13:90743183
13	rs9588826	90641387	T	0.5293	1.36E-06	chr:13:90743183
13	rs73589597	90708041	C	3.89	1.44E-06	chr:13:90743183
13	rs12585482	90562222	A	0.5274	1.46E-06	chr:13:90743183
13	rs35152474	90623202	T	2.181	1.50E-06	chr:13:90743183
13	rs67181673	90624858	G	2.181	1.50E-06	chr:13:90743183
13	rs11839415	90579846	T	2.18	1.52E-06	chr:13:90743183
13	rs12854096	90614128	G	2.18	1.52E-06	chr:13:90743183
13	rs12870592	90616121	T	2.18	1.52E-06	chr:13:90743183
13	rs17633242	90603523	A	2.18	1.52E-06	chr:13:90743183
13	rs34072176	90595015	G	2.18	1.52E-06	chr:13:90743183
13	rs35019682	90600049	G	2.18	1.52E-06	chr:13:90743183
13	rs36012183	90604597	C	2.18	1.52E-06	chr:13:90743183
13	rs67842803	90583574	G	2.18	1.52E-06	chr:13:90743183
13	rs8001179	90613977	G	2.18	1.52E-06	chr:13:90743183
13	rs9560491	90542236	T	0.5286	1.55E-06	chr:13:90743183
13	rs9560492	90542402	T	0.5286	1.55E-06	chr:13:90743183
13	rs35160629	90641021	G	1.974	1.57E-06	chr:13:90743183
13	rs9560507	90637423	A	1.994	1.59E-06	chr:13:90743183
13	rs73594641	90617801	T	5.765	1.70E-06	chr:13:90743183
13	rs74102220	90772886	G	4.18	1.75E-06	chr:13:90743183
13	rs9560542	90707966	A	0.4163	1.82E-06	chr:13:90743183
13	rs115159618	90747104	G	7.216	1.83E-06	chr:13:90743183
13	rs9588824	90633423	G	0.5407	1.87E-06	chr:13:90743183
13	rs59478734	90825311	A	3.057	1.98E-06	chr:13:90743183
13	rs73597471	90821777	T	3.057	1.98E-06	chr:13:90743183
13	rs73597491	90830101	T	3.048	2.04E-06	chr:13:90743183
13	rs61960173	90773350	G	0.4692	2.16E-06	chr:13:90743183
13	rs12856882	90638971	T	1.995	2.22E-06	chr:13:90743183
13	rs9560511	90637932	C	1.995	2.22E-06	chr:13:90743183
13	rs7984830	90718586	T	0.3798	2.26E-06	chr:13:90743183
13	rs115483767	90657352	C	5.992	2.55E-06	chr:13:90743183
13	rs60749084	90662586	G	5.992	2.55E-06	chr:13:90743183
13	rs73597482	90825069	G	3.055	2.58E-06	chr:13:90743183
13	rs74102250	90830341	C	2.888	2.66E-06	chr:13:90743183
13	rs57953905	90872130	G	2.712	2.76E-06	chr:13:90743183
13	rs1491010	90837913	A	2.282	2.86E-06	chr:13:90743183
13	rs2805646	90772617	G	0.4866	2.90E-06	chr:13:90743183

13	rs2637478	90709431	C	0.5184	2.92E-06	chr:13:90743183
13	rs2805684	90712517	T	0.5184	2.92E-06	chr:13:90743183
13	rs55654710	90782777	T	4.688	2.96E-06	chr:13:90743183
13	rs74102230	90792973	T	4.688	2.96E-06	chr:13:90743183
13	rs112363253	90592057	T	5.574	3.02E-06	chr:13:90743183
13	rs113260853	90627086	G	5.574	3.02E-06	chr:13:90743183
13	rs113575165	90598445	T	5.574	3.02E-06	chr:13:90743183
13	rs73590665	90579572	A	5.574	3.02E-06	chr:13:90743183
13	rs73590670	90580024	C	5.574	3.02E-06	chr:13:90743183
13	rs73590682	90582229	G	5.574	3.02E-06	chr:13:90743183
13	rs73590684	90583224	A	5.574	3.02E-06	chr:13:90743183
13	rs73590691	90584739	A	5.574	3.02E-06	chr:13:90743183
13	rs73592604	90588007	G	5.574	3.02E-06	chr:13:90743183
13	rs73594610	90609667	A	5.574	3.02E-06	chr:13:90743183
13	rs73594672	90623800	A	5.574	3.02E-06	chr:13:90743183
13	rs9560509	90637790	G	1.966	3.17E-06	chr:13:90743183
13	rs55759961	90862725	A	2.824	3.23E-06	chr:13:90743183
13	rs7331225	90646484	C	0.4941	3.32E-06	chr:13:90743183
13	rs1907429	90772910	A	0.4895	3.46E-06	chr:13:90743183
13	rs2260330	90772341	G	0.4895	3.46E-06	chr:13:90743183
13	rs73583123	90863956	T	2.703	3.68E-06	chr:13:90743183
13	rs73583107	90847543	T	2.815	4.04E-06	chr:13:90743183
13	rs79414261	90843550	A	2.815	4.04E-06	chr:13:90743183
13	rs7491812	90752437	G	0.3748	4.22E-06	chr:13:90743183
13	rs73595615	90773157	G	3.653	4.28E-06	chr:13:90743183
13	rs2805641	90761259	G	1.907	4.65E-06	chr:13:90743183
13	rs113227446	90807925	G	3.759	4.87E-06	chr:13:90743183
13	rs113236793	90792905	T	3.759	4.87E-06	chr:13:90743183
13	rs113988025	90807223	T	3.759	4.87E-06	chr:13:90743183
13	rs116167725	90783382	C	3.759	4.87E-06	chr:13:90743183
13	rs116651730	90783384	G	3.759	4.87E-06	chr:13:90743183
13	rs144588438	90783823	G	3.759	4.87E-06	chr:13:90743183
13	rs73595636	90782881	A	3.759	4.87E-06	chr:13:90743183
13	rs73595658	90788533	A	3.759	4.87E-06	chr:13:90743183
13	rs73595666	90791754	A	3.759	4.87E-06	chr:13:90743183
13	rs73595678	90794729	C	3.759	4.87E-06	chr:13:90743183
13	rs73595692	90803673	A	3.759	4.87E-06	chr:13:90743183
13	rs73597431	90813633	G	3.759	4.87E-06	chr:13:90743183
13	rs75879175	90810098	T	3.759	4.87E-06	chr:13:90743183
13	rs77034014	90791980	T	3.759	4.87E-06	chr:13:90743183
13	rs80143767	90810663	G	3.759	4.87E-06	chr:13:90743183
13	rs9522837	90724010	C	0.3756	5.16E-06	chr:13:90743183
13	rs74102232	90794536	A	4.098	5.18E-06	chr:13:90743183

13	rs955322	90568994	C	0.5541	5.52E-06	chr:13:90743183
13	rs574520393	90810145	A	4.716	6.56E-06	chr:13:90743183
13	rs7318897	90541300	T	0.381	6.64E-06	chr:13:90743183
13	rs74102239	90814460	A	3.794	6.81E-06	chr:13:90743183
13	rs9560569	90823825	T	0.531	7.21E-06	chr:13:90743183
13	rs73583129	90869753	G	2.465	7.33E-06	chr:13:90743183
13	rs72639682	90735400	A	0.4144	7.38E-06	chr:13:90743183
13	rs1539156	90548249	T	0.5484	7.44E-06	chr:13:90743183
13	rs9522835	90715112	G	0.3868	7.45E-06	chr:13:90743183
13	rs7996031	90822896	C	0.5301	7.55E-06	chr:13:90743183
13	rs8002817	90822912	C	0.5301	7.55E-06	chr:13:90743183
13	rs2805643	90762364	T	1.872	7.61E-06	chr:13:90743183
13	rs9560495	90562947	T	0.5595	7.84E-06	chr:13:90743183
13	rs9560497	90567654	T	0.5595	7.84E-06	chr:13:90743183
13	rs9583747	90567048	T	0.5595	7.84E-06	chr:13:90743183
13	rs9588794	90562433	A	0.5595	7.84E-06	chr:13:90743183
13	rs111375518	90775792	A	3.577	7.92E-06	chr:13:90743183
13	rs2637475	90712256	T	0.5354	8.18E-06	chr:13:90743183
13	rs12429707	90678379	C	0.4193	8.87E-06	chr:13:90743183
13	rs28837503	90675852	A	0.4193	8.87E-06	chr:13:90743183
13	rs72636938	90669783	G	0.4193	8.87E-06	chr:13:90743183
13	rs9555971	90697612	A	0.4193	8.87E-06	chr:13:90743183
13	rs9560523	90672204	C	0.4193	8.87E-06	chr:13:90743183
13	rs9560524	90674539	A	0.4193	8.87E-06	chr:13:90743183
13	rs9560537	90690924	G	0.4193	8.87E-06	chr:13:90743183
13	rs9560538	90691450	A	0.4193	8.87E-06	chr:13:90743183
13	rs9634613	90668112	G	0.4193	8.87E-06	chr:13:90743183
13	rs4142565	90742687	G	0.419	9.39E-06	chr:13:90743183
13	rs75426841	90822252	T	4.879	9.46E-06	chr:13:90743183
13	rs75197852	90814283	A	3.01	9.61E-06	chr:13:90743183
13	rs73595700	90804104	T	3.708	9.63E-06	chr:13:90743183
13	rs10161633	90653542	A	0.4585	1.01E-05	chr:13:90743183
13	rs1578543	90655155	A	0.4585	1.01E-05	chr:13:90743183
13	rs17664303	90659602	A	0.4585	1.01E-05	chr:13:90743183
13	rs60185893	90649244	G	0.4585	1.01E-05	chr:13:90743183
13	rs66656579	90656550	A	0.4585	1.01E-05	chr:13:90743183
13	rs9588831	90652104	G	0.4585	1.01E-05	chr:13:90743183
13	rs12428396	90753177	G	0.4192	1.02E-05	chr:13:90743183
13	rs9560554	90755079	G	0.4192	1.02E-05	chr:13:90743183
13	rs9560568	90823449	C	0.5372	1.03E-05	chr:13:90743183
13	rs141255381	90787561	T	3.95	1.04E-05	chr:13:90743183
13	rs80043791	90787299	G	3.95	1.04E-05	chr:13:90743183
13	rs9560486	90531354	A	0.5554	1.06E-05	chr:13:90743183

13	rs72641506	90757010	C	0.5151	1.07E-05	chr:13:90743183
13	rs552641606	90795958	A	3.778	1.12E-05	chr:13:90743183
13	rs56987740	90687516	T	2.905	1.17E-05	chr:13:90743183
13	rs12184683	90618940	G	0.4404	1.22E-05	chr:13:90743183
13	rs7994080	90715892	C	0.4145	1.23E-05	chr:13:90743183
13	rs9515652	90541852	G	1.794	1.27E-05	chr:13:90743183
13	rs9522782	90539818	G	1.794	1.27E-05	chr:13:90743183
13	rs2805749	90747622	A	0.4014	1.45E-05	chr:13:90743183
13	rs9588821	90628299	T	0.4443	1.50E-05	chr:13:90743183
13	rs13378871	90632416	A	0.4411	1.57E-05	chr:13:90743183
13	rs12585323	90593476	C	0.4458	1.60E-05	chr:13:90743183
13	rs56166032	90599344	A	0.4458	1.60E-05	chr:13:90743183
13	rs7994571	90603122	A	0.4458	1.60E-05	chr:13:90743183
13	rs9301639	90591226	T	0.4458	1.60E-05	chr:13:90743183
13	rs9588801	90593093	T	0.4458	1.60E-05	chr:13:90743183
13	rs9588805	90604479	T	0.4458	1.60E-05	chr:13:90743183
13	rs9588807	90605464	C	0.4458	1.60E-05	chr:13:90743183
13	rs9588809	90615236	A	0.4458	1.60E-05	chr:13:90743183
13	rs9805197	90600767	T	0.4458	1.60E-05	chr:13:90743183
13	rs9805592	90600637	G	0.4458	1.60E-05	chr:13:90743183
13	rs9515665	90664547	G	0.448	1.67E-05	chr:13:90743183
13	rs9588825	90639634	G	0.4454	1.70E-05	chr:13:90743183
14	rs6573756	20555675	G	17.05	1.15E-29	chr:14:20552746
14	rs4360850	20558013	A	14.95	1.16E-29	chr:14:20552746
14	rs4438225	20557810	A	14.8	1.45E-29	chr:14:20552746
14	rs6573762	20555920	A	16.75	1.75E-29	chr:14:20552746
14	rs7401094	20555950	C	16.75	1.75E-29	chr:14:20552746
14	rs4297617	20557961	T	15.67	2.29E-29	chr:14:20552746
14	rs4572311	20557971	C	15.67	2.29E-29	chr:14:20552746
14	rs11158659	20556212	A	17.88	3.45E-29	chr:14:20552746
14	rs11158660	20556259	C	17.88	3.45E-29	chr:14:20552746
14	rs12434437	20556447	T	17.88	3.45E-29	chr:14:20552746
14	rs12434463	20556513	T	17.88	3.45E-29	chr:14:20552746
14	rs6573757	20555744	A	17.88	3.45E-29	chr:14:20552746
14	rs6573759	20555817	C	17.88	3.45E-29	chr:14:20552746
14	rs7151579	20556265	G	17.88	3.45E-29	chr:14:20552746
14	rs10136466	20550347	C	6.826	8.51E-28	chr:14:20552746
14	rs1958722	20562590	G	5.664	1.51E-25	chr:14:20552746
14	rs4981728	20560512	A	0.1873	3.00E-25	chr:14:20552746
14	rs12433363	20561341	T	0.1857	3.11E-25	chr:14:20552746
14	rs12586923	20559937	G	0.1857	3.11E-25	chr:14:20552746
14	rs12587536	20559906	C	0.1857	3.11E-25	chr:14:20552746
14	rs12881697	20560434	A	0.1857	3.11E-25	chr:14:20552746

14	rs4144239	20561922	G	0.1857	3.11E-25	chr:14:20552746
14	rs4144240	20561753	T	0.1857	3.11E-25	chr:14:20552746
14	rs57793822	20561481	C	0.1857	3.11E-25	chr:14:20552746
14	rs7148411	20559282	A	0.1857	3.11E-25	chr:14:20552746
14	rs7158086	20559451	A	0.1857	3.11E-25	chr:14:20552746
14	rs969774	20562080	C	0.1857	3.11E-25	chr:14:20552746
14	rs11158661	20556366	C	0.1833	3.54E-25	chr:14:20552746
14	rs4144238	20561971	T	0.2071	3.02E-22	chr:14:20552746
14	rs12890274	20560354	C	0.21	3.10E-22	chr:14:20552746
14	rs58618658	20549983	A	4.4	2.09E-20	chr:14:20552746
14	rs74037224	20549898	A	4.4	2.09E-20	chr:14:20552746
14	rs7153404	20550111	A	0.2275	1.60E-18	chr:14:20552746
14	rs12590521	20558876	G	0.2404	1.77E-17	chr:14:20552746
14	rs78783511	20555396	C	3.665	1.02E-16	chr:14:20552746
14	rs7152272	20559234	A	2.528	5.35E-13	chr:14:20552746
14	rs58368472	20561378	T	2.245	1.54E-10	chr:14:20552746
14	rs10150358	20552946	A	0.478	2.15E-10	chr:14:20552746
14	rs58438833	20552415	A	0.478	2.15E-10	chr:14:20552746
14	rs113445938	20550943	C	0.4963	2.88E-10	chr:14:20552746
14	rs114454848	20557868	C	3.418	1.04E-09	chr:14:20552746
14	rs75471823	20557468	C	3.418	1.04E-09	chr:14:20552746
14	rs12588910	20558938	T	0.501	2.85E-08	chr:14:20552746
14	rs56023234	20550049	A	2.882	6.90E-08	chr:14:20552746
14	rs8020394	20550495	C	2.882	6.90E-08	chr:14:20552746
14	rs182788079	20555663	T	2.79	1.15E-07	chr:14:20552746
14	rs190564533	20555662	A	2.79	1.15E-07	chr:14:20552746
14	rs374481570	20550995	A	0.1298	1.36E-07	chr:14:20552746
14	rs80060711	20549750	A	2.787	2.87E-07	chr:14:20552746
14	rs10141155	20557742	G	0.4067	5.49E-07	chr:14:20552746
14	rs74037232	20561953	T	2.588	6.65E-07	chr:14:20552746
14	rs7144218	20563139	G	2.449	9.39E-07	chr:14:20552746
14	rs10136940	20553166	G	0.6104	9.57E-07	chr:14:20552746
14	rs73575313	20550544	T	0.4201	1.09E-06	chr:14:20552746
14	rs10137015	20555241	T	0.611	1.29E-06	chr:14:20552746
14	rs12897224	20560235	C	2.989	6.29E-06	chr:14:20552746
14	rs138182926	20563062	A	4.449	7.81E-06	chr:14:20552746
14	rs2262356	20533972	A	0.5	1.29E-05	chr:14:20552746
14	rs138465439	20572188	T	4.299	1.29E-05	chr:14:20552746
14	rs578237295	20567485	C	4.299	1.29E-05	chr:14:20552746
14	rs78025773	20667485	A	2.536	1.49E-05	chr:14:20552746
14	rs141544050	20598438	A	3.767	1.61E-05	chr:14:20552746
14	rs72669100	20669659	T	2.602	1.81E-05	chr:14:20552746
14	rs77455669;rs573148833	20601712	A	3.855	1.94E-05	chr:14:20552746

14	rs115548693	20669341	G	2.497	2.08E-05	chr:14:20552746
14	rs77527268	20668468	T	2.497	2.08E-05	chr:14:20552746
14	rs138738400	20594248	C	3.956	2.19E-05	chr:14:20552746
14	rs28404937	20556487	T	3.247	2.58E-05	chr:14:20552746
14	rs1805277	20533442	G	0.5549	2.96E-05	chr:14:20552746
14	rs1958719	20533586	A	0.5549	2.96E-05	chr:14:20552746
14	rs2775256	20533836	G	0.5549	2.96E-05	chr:14:20552746
14	rs562809940	20572294	T	3.746	3.01E-05	chr:14:20552746
15	rs2937855	63374677	G	0.1314	1.11E-27	chr:15:63374594
15	rs2652804	63374256	T	0.1687	4.80E-25	chr:15:63374594
15	rs2652805	63374129	A	0.1687	4.80E-25	chr:15:63374594
15	rs2652806	63374127	C	0.1687	4.80E-25	chr:15:63374594
15	rs2729805	63374289	A	0.1722	1.25E-24	chr:15:63374594
15	rs2729806	63373329	T	0.1729	2.10E-24	chr:15:63374594
15	rs2652832	63396928	T	0.2581	1.80E-18	chr:15:63374594
15	rs2729833	63397044	G	0.2581	1.80E-18	chr:15:63374594
15	rs2729801	63381095	A	0.2583	2.28E-18	chr:15:63374594
15	rs7182618	63398622	T	0.2642	3.71E-18	chr:15:63374594
15	rs78015421	63391406	A	0.2664	5.64E-18	chr:15:63374594
15	rs68088462	63397617	T	0.2624	7.66E-18	chr:15:63374594
15	rs28849975	63393922	T	0.2677	8.90E-18	chr:15:63374594
15	rs4344684	63397318	C	0.2844	8.68E-17	chr:15:63374594
15	rs2729804	63376659	T	3.449	1.87E-16	chr:15:63374594
15	rs34575783	63397686	G	0.3033	1.02E-15	chr:15:63374594
15	rs28872293	63394148	G	0.3035	1.16E-15	chr:15:63374594
15	rs2414811	63397725	C	0.3016	1.47E-15	chr:15:63374594
15	rs2729789	63400345	G	0.3085	1.79E-15	chr:15:63374594
15	rs8032104	63400584	A	0.3085	1.79E-15	chr:15:63374594
15	rs11637852	63397151	G	0.3088	1.97E-15	chr:15:63374594
15	rs2652833	63396885	C	0.3088	1.97E-15	chr:15:63374594
15	rs2652838	63396088	A	0.3088	1.97E-15	chr:15:63374594
15	rs2652840	63393220	T	0.3088	1.97E-15	chr:15:63374594
15	rs2652842	63391939	C	0.3088	1.97E-15	chr:15:63374594
15	rs2652843	63391648	T	0.3088	1.97E-15	chr:15:63374594
15	rs2652844	63391647	T	0.3088	1.97E-15	chr:15:63374594
15	rs2729830	63395812	G	0.3088	1.97E-15	chr:15:63374594
15	rs28768697	63394095	A	0.3088	1.97E-15	chr:15:63374594
15	rs34786874	63397504	A	0.3088	1.97E-15	chr:15:63374594
15	rs36116701	63397494	T	0.3088	1.97E-15	chr:15:63374594
15	rs76127683	63391362	G	0.3088	1.97E-15	chr:15:63374594
15	rs2134265	63398196	G	0.3101	2.05E-15	chr:15:63374594
15	rs7181141	63398552	A	0.3101	2.05E-15	chr:15:63374594
15	rs2652836	63396301	C	0.3091	2.27E-15	chr:15:63374594

15	rs2729776	63392222	T	0.3091	2.27E-15	chr:15:63374594
15	rs2729834	63397047	G	0.3091	2.27E-15	chr:15:63374594
15	rs4775621	63395062	G	0.3091	2.27E-15	chr:15:63374594
15	rs8034620	63397155	C	0.3091	2.27E-15	chr:15:63374594
15	rs28860327	63393824	A	0.31	2.49E-15	chr:15:63374594
15	rs2729787	63401493	G	0.3211	2.84E-15	chr:15:63374594
15	rs2652835	63396408	T	0.3143	3.77E-15	chr:15:63374594
15	rs2729832	63396401	A	0.3146	4.35E-15	chr:15:63374594
15	rs2729788	63400378	G	0.3218	4.62E-15	chr:15:63374594
15	rs8034945	63397207	G	0.3214	4.78E-15	chr:15:63374594
15	rs12917357	63399793	C	0.3222	5.30E-15	chr:15:63374594
15	rs11637947	63397538	C	0.3227	6.04E-15	chr:15:63374594
15	rs12912721	63397385	T	0.3227	6.04E-15	chr:15:63374594
15	rs2652839	63395158	C	0.3227	6.04E-15	chr:15:63374594
15	rs2729829	63395484	C	0.3227	6.04E-15	chr:15:63374594
15	rs2729831	63396039	A	0.3227	6.04E-15	chr:15:63374594
15	rs35963767	63397601	G	0.3227	6.04E-15	chr:15:63374594
15	rs2652785	63397890	T	0.3202	6.66E-15	chr:15:63374594
15	rs9646207	63391235	C	0.3302	7.08E-15	chr:15:63374594
15	rs2652815	63401345	T	0.3253	9.98E-15	chr:15:63374594
15	rs4775620	63395043	C	0.3304	1.37E-14	chr:15:63374594
15	rs11071727	63390808	T	0.3448	1.93E-14	chr:15:63374594
15	rs4774472	63353760	C	0.2999	6.31E-14	chr:15:63374594
15	rs11071725	63390608	C	0.3508	7.36E-14	chr:15:63374594
15	rs10162675	63370358	C	0.3121	2.52E-13	chr:15:63374594
15	rs2729781	63404265	T	0.3543	2.79E-13	chr:15:63374594
15	rs2652816	63403368	C	0.3555	3.51E-13	chr:15:63374594
15	rs2729782	63403354	G	0.3555	3.51E-13	chr:15:63374594
15	rs2729783	63403316	G	0.3555	3.51E-13	chr:15:63374594
15	rs2729784	63402735	T	0.3555	3.51E-13	chr:15:63374594
15	rs6494394	63440938	A	0.3551	5.33E-13	chr:15:63374594
15	rs2729785	63402574	G	0.3595	5.89E-13	chr:15:63374594
15	rs2652813	63405366	G	0.3657	6.64E-13	chr:15:63374594
15	rs2937859	63366282	G	0.3257	7.44E-13	chr:15:63374594
15	rs2729780	63404639	C	0.3625	7.65E-13	chr:15:63374594
15	rs7175602	63367032	C	0.3265	1.01E-12	chr:15:63374594
15	rs2652834	63396867	A	0.3418	1.29E-12	chr:15:63374594
15	rs8038648	63379643	G	0.3674	1.42E-12	chr:15:63374594
15	rs7165016	63439590	T	0.389	3.44E-12	chr:15:63374594
15	rs12912328	63494235	A	0.3799	1.13E-11	chr:15:63374594
15	rs12595312	63494159	T	0.3821	1.15E-11	chr:15:63374594
15	rs7176597	63494482	G	0.3821	1.15E-11	chr:15:63374594
15	rs11071726	63390622	T	0.404	1.19E-11	chr:15:63374594

15	rs11855609	63522691	C	0.3984	4.44E-11	chr:15:63374594
15	rs2729828	63361307	A	0.3284	4.84E-11	chr:15:63374594
15	rs16946416	63356721	T	0.3293	6.53E-11	chr:15:63374594
15	rs11071723	63355275	A	0.3379	1.21E-10	chr:15:63374594
15	rs28583444	63357852	T	0.3419	1.71E-10	chr:15:63374594
15	rs4238372	63351488	G	0.3419	1.71E-10	chr:15:63374594
15	rs6494396	63441248	C	0.3836	2.04E-10	chr:15:63374594
15	rs7170462	63345622	A	0.343	2.32E-10	chr:15:63374594
15	rs4774474	63405580	G	0.4083	2.67E-10	chr:15:63374594
15	rs12593078	63517347	A	0.4191	2.93E-10	chr:15:63374594
15	rs4775614	63351687	A	0.3504	3.10E-10	chr:15:63374594
15	rs2652824	63420001	C	0.4012	3.57E-10	chr:15:63374594
15	rs8032506	63371644	A	0.3489	3.61E-10	chr:15:63374594
15	rs4774478	63434763	A	0.4216	4.16E-10	chr:15:63374594
15	rs2729837	63430330	C	0.4032	4.36E-10	chr:15:63374594
15	rs2729827	63360564	G	0.4279	4.75E-10	chr:15:63374594
15	rs11853804	63528256	C	0.4301	7.66E-10	chr:15:63374594
15	rs11071735	63468114	T	0.3932	1.02E-09	chr:15:63374594
15	rs4238370	63347363	T	0.4149	1.13E-09	chr:15:63374594
15	rs2729779	63407173	T	0.4094	1.39E-09	chr:15:63374594
15	rs146054382	63508433	A	0.4356	1.60E-09	chr:15:63374594
15	rs78356111	63436955	C	0.435	1.63E-09	chr:15:63374594
15	rs6494391	63406785	T	0.4305	1.70E-09	chr:15:63374594
15	rs72747098	63525249	A	2.296	2.12E-09	chr:15:63374594
15	rs7163254	63439403	C	0.4179	2.31E-09	chr:15:63374594
15	rs6494392	63406863	G	0.4158	2.41E-09	chr:15:63374594
15	rs8468	63434110	C	0.4301	5.24E-09	chr:15:63374594
15	rs2652817	63483290	A	0.3964	5.47E-09	chr:15:63374594
15	rs2729791	63493057	G	0.3964	5.47E-09	chr:15:63374594
15	rs2729790	63466427	T	0.4556	6.68E-09	chr:15:63374594
15	rs13313493	63523855	C	0.3993	6.95E-09	chr:15:63374594
15	rs16946692	63531915	G	0.3993	6.95E-09	chr:15:63374594
15	rs2588862	63500195	A	0.3993	6.95E-09	chr:15:63374594
15	rs55976323	63523586	A	0.3993	6.95E-09	chr:15:63374594
15	rs7175974	63510113	G	0.3993	6.95E-09	chr:15:63374594
15	rs8032533	63516033	A	0.3993	6.95E-09	chr:15:63374594
15	rs4774476	63407390	T	2.346	1.66E-08	chr:15:63374594
15	rs7174466	63425019	A	0.4379	1.74E-08	chr:15:63374594
15	rs7175973	63425027	T	0.4379	1.74E-08	chr:15:63374594
15	rs12904537	63379726	T	2.595	1.95E-08	chr:15:63374594
15	rs9672258	63532459	C	0.4205	2.60E-08	chr:15:63374594
15	rs7177692	63379733	T	2.555	2.79E-08	chr:15:63374594
15	rs7177694	63379737	T	2.555	2.79E-08	chr:15:63374594

15	rs2652795	63409265	T	0.4464	2.85E-08	chr:15:63374594
15	rs2652796	63408042	T	0.4464	2.85E-08	chr:15:63374594
15	rs2652797	63407578	C	0.4464	2.85E-08	chr:15:63374594
15	rs2652798	63407446	C	0.4464	2.85E-08	chr:15:63374594
15	rs2729778	63407332	T	0.4464	2.85E-08	chr:15:63374594
15	rs4775624	63408548	T	0.4464	2.85E-08	chr:15:63374594
15	rs10152434	63545352	C	0.4236	3.52E-08	chr:15:63374594
15	rs112196574	63545156	A	0.4236	3.52E-08	chr:15:63374594
15	rs12439988	63547217	A	0.4236	3.52E-08	chr:15:63374594
15	rs2062727	63472681	A	0.4435	3.85E-08	chr:15:63374594
15	rs16946690	63530492	A	0.428	4.04E-08	chr:15:63374594
15	rs2652818	63502378	C	0.428	4.04E-08	chr:15:63374594
15	rs4774475	63407357	T	0.4693	5.23E-08	chr:15:63374594
15	rs6494397	63460009	A	0.4801	5.96E-08	chr:15:63374594
15	rs62011182	63413115	A	0.4615	7.78E-08	chr:15:63374594
15	rs9888638	63410349	T	0.4613	8.53E-08	chr:15:63374594
15	rs9888735	63410141	G	0.4613	8.53E-08	chr:15:63374594
15	rs2652787	63415843	G	0.4622	8.61E-08	chr:15:63374594
15	rs2729819	63415849	C	0.4622	8.61E-08	chr:15:63374594
15	rs2652802	63377307	G	0.2694	9.61E-08	chr:15:63374594
15	rs28369547	63528924	T	0.4417	9.75E-08	chr:15:63374594
15	rs2652789	63415223	A	0.4649	1.05E-07	chr:15:63374594
15	rs11071733	63416481	G	2.268	1.12E-07	chr:15:63374594
15	rs2652819	63431175	A	0.4705	1.31E-07	chr:15:63374594
15	rs2652820	63430901	T	0.4705	1.31E-07	chr:15:63374594
15	rs10851724	63416702	T	0.4678	1.36E-07	chr:15:63374594
15	rs2652792	63411492	G	0.4678	1.36E-07	chr:15:63374594
15	rs2652794	63410872	C	0.4678	1.36E-07	chr:15:63374594
15	rs2729813	63412154	A	0.4678	1.36E-07	chr:15:63374594
15	rs2729815	63412843	A	0.4678	1.36E-07	chr:15:63374594
15	rs2729820	63416193	A	0.4678	1.36E-07	chr:15:63374594
15	rs34317102	63414083	A	0.4678	1.36E-07	chr:15:63374594
15	rs4551986	63424400	T	0.4678	1.36E-07	chr:15:63374594
15	rs2652812	63406170	C	0.4829	1.44E-07	chr:15:63374594
15	rs2729823	63420660	A	0.4718	1.50E-07	chr:15:63374594
15	rs2652807	63373942	G	0.2742	1.54E-07	chr:15:63374594
15	rs59768255	63436960	A	0.5112	1.80E-07	chr:15:63374594
15	rs61187203	63436958	C	0.5112	1.80E-07	chr:15:63374594
15	rs966331	63411927	T	0.4883	1.86E-07	chr:15:63374594
15	rs4775625	63410577	G	0.4867	1.87E-07	chr:15:63374594
15	rs1007075	63413501	A	0.49	2.16E-07	chr:15:63374594
15	rs1007076	63413371	T	0.49	2.16E-07	chr:15:63374594
15	rs12902237	63418188	C	0.4899	2.18E-07	chr:15:63374594

15	rs55719615	63344167	G	0.4438	2.73E-07	chr:15:63374594
15	rs1972041	63360847	C	2.024	2.77E-07	chr:15:63374594
15	rs2729793	63483977	T	0.4659	3.11E-07	chr:15:63374594
15	rs12591282	63539475	G	0.4638	3.88E-07	chr:15:63374594
15	rs2173982	63361262	C	1.989	4.76E-07	chr:15:63374594
15	rs3784673	63547859	C	0.4748	4.86E-07	chr:15:63374594
15	rs11071721	63350218	T	0.4735	5.18E-07	chr:15:63374594
15	rs7166624	63348594	A	0.4735	5.18E-07	chr:15:63374594
15	rs4468558	63346681	T	0.4765	6.33E-07	chr:15:63374594
15	rs2062726	63472745	A	0.5171	7.82E-07	chr:15:63374594
15	rs62012880	63527162	G	2.009	8.77E-07	chr:15:63374594
15	rs113519012	63362525	A	2.191	9.27E-07	chr:15:63374594
15	rs4775628	63426463	A	0.5135	9.29E-07	chr:15:63374594
15	rs7175185	63425337	A	0.5135	9.29E-07	chr:15:63374594
15	rs920864	63474391	G	0.5192	9.81E-07	chr:15:63374594
15	rs1976433	63426912	T	0.5216	1.17E-06	chr:15:63374594
15	rs2729777	63468964	T	0.5253	1.27E-06	chr:15:63374594
15	rs10851723	63416335	G	0.5182	1.32E-06	chr:15:63374594
15	rs28565028	63397038	T	2.123	1.34E-06	chr:15:63374594
15	rs2588859	63469846	A	0.5249	1.35E-06	chr:15:63374594
15	rs2588860	63471333	G	0.525	1.39E-06	chr:15:63374594
15	rs11071739	63585896	T	0.5164	1.40E-06	chr:15:63374594
15	rs1017545	63553994	C	1.975	1.66E-06	chr:15:63374594
15	rs6494393	63426435	A	0.5249	1.72E-06	chr:15:63374594
15	rs4633662	63424286	G	0.5284	1.72E-06	chr:15:63374594
15	rs28656270	63374294	C	3.334	2.01E-06	chr:15:63374594
15	rs2729826	63360446	G	0.5001	2.18E-06	chr:15:63374594
15	rs1566660	63429258	C	0.5354	2.59E-06	chr:15:63374594
15	rs1126312	63427638	A	0.5396	3.11E-06	chr:15:63374594
15	rs9302231	63427394	G	0.5396	3.11E-06	chr:15:63374594
15	rs1126310	63428765	G	0.5422	3.76E-06	chr:15:63374594
15	rs11858666	63526239	C	1.941	3.91E-06	chr:15:63374594
15	rs2729803	63376661	T	2.014	6.27E-06	chr:15:63374594
15	rs147111390	63374643	T	0.2212	6.51E-06	chr:15:63374594
15	rs2134263	63365394	T	1.901	6.95E-06	chr:15:63374594
15	rs12324711	63409679	T	0.2692	1.11E-05	chr:15:63374594
15	rs7172246	63554824	A	0.5775	1.35E-05	chr:15:63374594
15	rs7170846	63449543	C	2.451	1.43E-05	chr:15:63374594
15	rs55862039	63451563	T	2.223	1.48E-05	chr:15:63374594
15	rs28394803	63443968	C	1.944	1.57E-05	chr:15:63374594
15	rs953978	63445144	T	1.944	1.57E-05	chr:15:63374594
15	rs56208666	63451858	G	2.227	1.69E-05	chr:15:63374594
15	rs34367270	63410912	A	1.805	1.72E-05	chr:15:63374594

15	rs7162890	63492027	A	2.13	1.88E-05	chr:15:63374594
15	rs17829340	63546414	C	2.271	2.08E-05	chr:15:63374594
15	rs8041454	63485976	C	2.151	2.23E-05	chr:15:63374594
15	rs11071724	63370843	G	2.136	2.23E-05	chr:15:63374594
15	rs74025269	63543279	C	0.2534	2.28E-05	chr:15:63374594
15	rs2899691	63519088	A	2.364	2.49E-05	chr:15:63374594
15	rs74021919	63418773	T	0.2618	2.57E-05	chr:15:63374594
15	rs74024349	63395543	C	0.3003	2.60E-05	chr:15:63374594
15	rs74568001	63394792	T	0.3003	2.60E-05	chr:15:63374594
15	rs959125	63583159	G	0.5664	2.66E-05	chr:15:63374594
15	rs11636105	63371220	A	2.125	2.73E-05	chr:15:63374594
15	rs4984257	63561160	T	1.905	2.81E-05	chr:15:63374594
15	rs369161040	63573742	C	0.5582	3.04E-05	chr:15:63374594
15	rs4984273	63472714	T	2.335	3.15E-05	chr:15:63374594
15	rs4984274	63472715	T	2.335	3.15E-05	chr:15:63374594
15	rs17762798	63600603	A	0.3762	3.27E-05	chr:15:63374594
15	rs2937857	63369571	T	1.709	3.34E-05	chr:15:63374594
19	rs146500963	21849138	G	53.34	4.48E-44	chr:19:21841536
19	rs12460252	21846543	A	50.83	6.51E-44	chr:19:21841536
19	rs12461654	21849302	A	50.83	6.51E-44	chr:19:21841536
19	rs1829501	21847506	C	50.83	6.51E-44	chr:19:21841536
19	rs59984853	21848526	A	50.83	6.51E-44	chr:19:21841536
19	rs8105433	21840606	A	47.15	7.84E-44	chr:19:21841536
19	rs28669973	21842253	A	49.2	8.25E-44	chr:19:21841536
19	rs113277169	21843728	T	48.98	4.99E-43	chr:19:21841536
19	rs186258664	21844201	T	48.98	4.99E-43	chr:19:21841536
19	rs12052180	21850463	C	46.09	7.87E-43	chr:19:21841536
19	rs58339212	21861075	T	46.46	7.95E-43	chr:19:21841536
19	rs111517079	21838995	T	46.05	8.42E-43	chr:19:21841536
19	rs138320974	21834230	C	46.05	8.42E-43	chr:19:21841536
19	rs34069114	21838272	C	46.05	8.42E-43	chr:19:21841536
19	rs78554651	21838518	G	46.05	8.42E-43	chr:19:21841536
19	rs80147175	21839022	G	46.05	8.42E-43	chr:19:21841536
19	rs73546506	21860575	A	44.29	8.55E-43	chr:19:21841536
19	rs79961580	21860730	A	44.29	8.55E-43	chr:19:21841536
19	rs12462750	21850703	G	45.82	9.96E-43	chr:19:21841536
19	rs60312928	21838295	G	43.91	1.44E-42	chr:19:21841536
19	rs117182815	21825628	A	25.52	4.95E-37	chr:19:21841536
19	rs34891363	21825550	C	24.82	4.97E-37	chr:19:21841536
19	rs12460950	21824781	C	25.63	5.74E-37	chr:19:21841536
19	rs67673622	21825103	C	25.63	5.74E-37	chr:19:21841536
19	rs1589279	21847351	C	22.65	7.86E-37	chr:19:21841536
19	rs58621185	21858531	A	22.01	1.77E-36	chr:19:21841536

19	rs116684909	21832378	T	28.49	2.17E-36	chr:19:21841536
19	rs111714111	21827774	A	28.43	2.51E-36	chr:19:21841536
19	rs113033092	21827530	C	28.96	2.76E-36	chr:19:21841536
19	rs77728877	21827833	G	28.96	2.76E-36	chr:19:21841536
19	rs7258332	21829769	G	28.89	2.86E-36	chr:19:21841536
19	rs112867628	21828269	A	29.47	3.21E-36	chr:19:21841536
19	rs35933524	21828299	G	29.47	3.21E-36	chr:19:21841536
19	rs4809168	21828794	A	29.47	3.21E-36	chr:19:21841536
19	rs7253097	21828936	C	29.47	3.21E-36	chr:19:21841536
19	rs7253361	21829123	G	29.47	3.21E-36	chr:19:21841536
19	rs7254224	21828967	C	29.47	3.21E-36	chr:19:21841536
19	rs7257314	21829542	A	29.47	3.21E-36	chr:19:21841536
19	rs7257929	21829488	C	29.47	3.21E-36	chr:19:21841536
19	rs58039129	21827133	A	30.74	3.68E-36	chr:19:21841536
19	rs2139533	21832590	T	27.54	4.15E-36	chr:19:21841536
19	rs111811317	21831260	A	32.35	8.74E-36	chr:19:21841536
19	rs12232857	21832320	A	32.35	8.74E-36	chr:19:21841536
19	rs35188146	21831014	G	32.35	8.74E-36	chr:19:21841536
19	rs35837179	21831140	A	32.35	8.74E-36	chr:19:21841536
19	rs57155685	21831116	T	32.35	8.74E-36	chr:19:21841536
19	rs57973828	21831115	C	32.35	8.74E-36	chr:19:21841536
19	rs59571905	21830875	T	32.35	8.74E-36	chr:19:21841536
19	rs7250456	21831585	T	32.35	8.74E-36	chr:19:21841536
19	rs7250875	21831905	T	32.35	8.74E-36	chr:19:21841536
19	rs61517956	21833407	A	33.06	1.19E-35	chr:19:21841536
19	rs1520075	21833741	C	32.72	2.85E-35	chr:19:21841536
19	rs75322575	21833798	A	36.1	3.56E-35	chr:19:21841536
19	rs115824809	21831481	T	11.2	4.71E-34	chr:19:21841536
19	rs2139534	21832567	G	11.41	1.24E-33	chr:19:21841536
19	rs12232815	21832363	C	10.96	1.29E-33	chr:19:21841536
19	rs2139531	21832736	A	11.14	1.35E-33	chr:19:21841536
19	rs7253108	21828955	G	10.46	1.44E-33	chr:19:21841536
19	rs117740054	21825672	T	10.02	1.53E-33	chr:19:21841536
19	rs2139532	21832597	C	11.32	1.69E-33	chr:19:21841536
19	rs111405107	21824157	A	185	3.12E-33	chr:19:21841536
19	rs111977553	21824165	T	185	3.12E-33	chr:19:21841536
19	rs7250867	21831884	T	40.55	9.92E-32	chr:19:21841536
19	rs59896423	21848375	T	11.79	8.00E-31	chr:19:21841536
19	rs7259645	21842436	T	139.8	8.39E-25	chr:19:21841536
19	rs144847660	21823693	T	594.7	1.02E-23	chr:19:21841536
19	rs10414424	21858966	C	5.143	1.75E-23	chr:19:21841536
19	rs145365305	21843419	A	17.23	6.93E-23	chr:19:21841536
19	rs7508279	21832973	T	0.2297	1.23E-22	chr:19:21841536

19	rs12461975	21862108	A	8.038	2.02E-22	chr:19:21841536
19	rs144768538	21848501	G	15.05	6.13E-22	chr:19:21841536
19	rs12461397	21861309	G	4.928	7.79E-22	chr:19:21841536
19	rs2359397	21854270	A	4.918	9.52E-22	chr:19:21841536
19	rs115046417	21854616	G	14.91	1.06E-21	chr:19:21841536
19	rs116818044	21857932	A	14.91	1.06E-21	chr:19:21841536
19	rs12461437	21861314	A	4.827	1.13E-21	chr:19:21841536
19	rs1983379	21860203	T	4.637	4.88E-21	chr:19:21841536
19	rs1818694	21858673	T	4.559	6.77E-21	chr:19:21841536
19	rs1850933	21855208	C	4.559	6.77E-21	chr:19:21841536
19	rs2359391	21854015	A	4.559	6.77E-21	chr:19:21841536
19	rs12459639	21852800	T	4.475	9.29E-21	chr:19:21841536
19	rs10854058	21858846	T	4.459	1.25E-20	chr:19:21841536
19	rs9917005	21853097	G	4.404	1.40E-20	chr:19:21841536
19	rs114434735	21866989	T	10.24	9.37E-20	chr:19:21841536
19	rs116277680	21862920	T	13.16	1.49E-19	chr:19:21841536
19	rs116205961	21848939	A	13.08	2.11E-19	chr:19:21841536
19	rs1520074	21833928	C	0.2023	5.92E-18	chr:19:21841536
19	rs113367038	21827121	G	6.817	1.45E-17	chr:19:21841536
19	rs12984244	21833966	T	0.2232	2.08E-17	chr:19:21841536
19	rs116521177	21831596	T	6.688	2.37E-17	chr:19:21841536
19	rs113005217	21845172	T	633.2	2.77E-17	chr:19:21841536
19	rs113115412	21815922	T	7.55	3.22E-17	chr:19:21841536
19	rs113865693	21816425	T	5.231	5.01E-17	chr:19:21841536
19	rs145545138	21816252	A	5.231	5.01E-17	chr:19:21841536
19	rs146923867	21812763	T	7.252	5.90E-17	chr:19:21841536
19	rs7245831	21812746	C	7.198	6.34E-17	chr:19:21841536
19	rs10423041	21933943	C	3.651	8.17E-17	chr:19:21841536
19	rs12978641	21843780	A	0.2622	8.44E-17	chr:19:21841536
19	rs112769786	21895211	C	6.744	9.22E-17	chr:19:21841536
19	rs4467196	21950795	C	3.65	9.75E-17	chr:19:21841536
19	rs376690624	21817575	C	7.035	1.05E-16	chr:19:21841536
19	rs4808291	21841588	G	0.2647	1.17E-16	chr:19:21841536
19	rs6511282	21841076	T	0.2647	1.17E-16	chr:19:21841536
19	rs12971429	21843346	A	0.2679	1.78E-16	chr:19:21841536
19	rs12973368	21843305	T	0.2679	1.78E-16	chr:19:21841536
19	rs12977844	21843501	A	0.2679	1.78E-16	chr:19:21841536
19	rs12979639	21843427	T	0.2679	1.78E-16	chr:19:21841536
19	rs7253077	21844512	C	0.2679	1.78E-16	chr:19:21841536
19	rs7253101	21844592	A	0.2679	1.78E-16	chr:19:21841536
19	rs7253231	21844658	A	0.2679	1.78E-16	chr:19:21841536
19	rs7253259	21844719	A	0.2679	1.78E-16	chr:19:21841536
19	rs8100335	21842862	T	0.2707	2.60E-16	chr:19:21841536

19	rs4352176	21924392	C	3.571	2.60E-16	chr:19:21841536
19	rs4569404	21924445	A	3.571	2.60E-16	chr:19:21841536
19	rs113293163	21914189	A	3.606	2.67E-16	chr:19:21841536
19	rs12978741	21843568	T	0.2483	2.77E-16	chr:19:21841536
19	rs12978776	21843616	A	0.2483	2.77E-16	chr:19:21841536
19	rs12980130	21843569	G	0.2483	2.77E-16	chr:19:21841536
19	rs28432246	21842163	C	0.2483	2.77E-16	chr:19:21841536
19	rs28520521	21842340	C	0.2483	2.77E-16	chr:19:21841536
19	rs115218875	21905585	G	7.306	2.85E-16	chr:19:21841536
19	rs12984552	21834092	G	0.2535	3.21E-16	chr:19:21841536
19	rs4808292	21841626	G	0.2484	3.22E-16	chr:19:21841536
19	rs4809171	21841170	C	0.2484	3.22E-16	chr:19:21841536
19	rs8105369	21840734	T	0.2548	3.34E-16	chr:19:21841536
19	rs8105649	21840765	C	0.2548	3.34E-16	chr:19:21841536
19	rs12979269	21843781	A	0.2483	3.51E-16	chr:19:21841536
19	rs12979139	21843901	T	0.2497	3.55E-16	chr:19:21841536
19	rs57699265	21844083	G	0.2497	3.55E-16	chr:19:21841536
19	rs8101035	21934556	C	3.5	4.46E-16	chr:19:21841536
19	rs11085493	21948748	T	3.483	4.51E-16	chr:19:21841536
19	rs56314966	21815935	A	4.6	4.59E-16	chr:19:21841536
19	rs10403795	21919081	A	3.534	4.60E-16	chr:19:21841536
19	rs10405142	21918928	T	3.534	4.60E-16	chr:19:21841536
19	rs10422982	21918221	C	3.534	4.60E-16	chr:19:21841536
19	rs4330044	21905838	T	3.534	4.60E-16	chr:19:21841536
19	rs77464908	21898805	A	3.534	4.60E-16	chr:19:21841536
19	rs10418334	21823931	C	0.2603	5.35E-16	chr:19:21841536
19	rs4809170	21836678	C	0.2585	5.42E-16	chr:19:21841536
19	rs10401456	21834185	G	0.2575	5.49E-16	chr:19:21841536
19	rs4808293	21841796	T	0.2536	5.52E-16	chr:19:21841536
19	rs10404030	21839783	G	0.2584	5.90E-16	chr:19:21841536
19	rs10405733	21834482	T	0.2584	5.90E-16	chr:19:21841536
19	rs10422946	21838785	T	0.2584	5.90E-16	chr:19:21841536
19	rs4808287	21835411	C	0.2584	5.90E-16	chr:19:21841536
19	rs4808289	21836844	G	0.2584	5.90E-16	chr:19:21841536
19	rs4808290	21837075	T	0.2584	5.90E-16	chr:19:21841536
19	rs6511279	21838240	A	0.2584	5.90E-16	chr:19:21841536
19	rs6511280	21838492	G	0.2584	5.90E-16	chr:19:21841536
19	rs6511281	21838565	C	0.2584	5.90E-16	chr:19:21841536
19	rs7351087	21838147	G	0.2584	5.90E-16	chr:19:21841536
19	rs7351088	21838146	T	0.2584	5.90E-16	chr:19:21841536
19	rs8106449	21835586	A	0.2584	5.90E-16	chr:19:21841536
19	rs9807876	21837709	A	0.2584	5.90E-16	chr:19:21841536
19	rs10422804	21838830	A	0.2789	5.95E-16	chr:19:21841536

19	rs1520066	21836294	G	0.2789	5.95E-16	chr:19:21841536
19	rs2139528	21839114	G	0.2789	5.95E-16	chr:19:21841536
19	rs4808285	21835126	T	0.2789	5.95E-16	chr:19:21841536
19	rs4808286	21835201	C	0.2789	5.95E-16	chr:19:21841536
19	rs7351091	21838002	G	0.2789	5.95E-16	chr:19:21841536
19	rs7508494	21838657	G	0.2789	5.95E-16	chr:19:21841536
19	rs8101233	21839603	C	0.2789	5.95E-16	chr:19:21841536
19	rs8104094	21837228	C	0.2789	5.95E-16	chr:19:21841536
19	rs8106619	21840994	T	0.2789	5.95E-16	chr:19:21841536
19	rs9807872	21837639	C	0.2789	5.95E-16	chr:19:21841536
19	rs3795247	21906428	C	3.507	6.42E-16	chr:19:21841536
19	rs7252777	21907988	T	3.507	6.42E-16	chr:19:21841536
19	rs112092047	21911328	T	6.785	6.43E-16	chr:19:21841536
19	rs12979778	21843988	A	0.2549	6.59E-16	chr:19:21841536
19	rs78183809	21827463	A	6.619	6.60E-16	chr:19:21841536
19	rs2139526	21839252	T	0.2791	6.77E-16	chr:19:21841536
19	rs1520067	21836159	G	0.2792	6.98E-16	chr:19:21841536
19	rs4372817	21922642	A	3.523	7.37E-16	chr:19:21841536
19	rs4808288	21836742	A	0.282	8.53E-16	chr:19:21841536
19	rs8105808	21840952	G	0.2824	9.32E-16	chr:19:21841536
19	rs7508059	21838351	A	0.2827	1.17E-15	chr:19:21841536
19	rs4808297	21935700	A	3.384	1.30E-15	chr:19:21841536
19	rs115955398	21880407	G	8.783	1.35E-15	chr:19:21841536
19	rs4381720	21916526	A	3.44	1.51E-15	chr:19:21841536
19	rs73540393	21822034	C	6.793	2.03E-15	chr:19:21841536
19	rs113716628	21927468	A	7.536	3.46E-15	chr:19:21841536
19	rs113881962	21893988	G	7.536	3.46E-15	chr:19:21841536
19	rs115055800	21917565	A	7.536	3.46E-15	chr:19:21841536
19	rs115256934	21909875	A	7.536	3.46E-15	chr:19:21841536
19	rs116579333	21939341	G	7.536	3.46E-15	chr:19:21841536
19	rs141125734	21918157	A	7.536	3.46E-15	chr:19:21841536
19	rs143955976	21911765	G	7.536	3.46E-15	chr:19:21841536
19	rs10469371	21893614	T	3.333	6.06E-15	chr:19:21841536
19	rs114301054	21946383	C	7.253	1.16E-14	chr:19:21841536
19	rs58748216	21914122	T	3.27	1.28E-14	chr:19:21841536
19	rs58886507	21885499	G	8.206	1.32E-14	chr:19:21841536
19	rs56742849	21914115	T	3.261	1.57E-14	chr:19:21841536
19	rs4319879	21916767	G	3.224	1.71E-14	chr:19:21841536
19	rs4547465	21901056	A	3.224	1.71E-14	chr:19:21841536
19	rs8105002	21919934	A	3.224	1.71E-14	chr:19:21841536
19	rs8111464	21917939	G	3.224	1.71E-14	chr:19:21841536
19	rs10416553	21823771	C	0.3093	1.93E-14	chr:19:21841536
19	rs4436888	21916489	C	3.218	1.97E-14	chr:19:21841536

19	rs7258859	21942723	G	3.182	2.01E-14	chr:19:21841536
19	rs566027145	21813440	C	4.702	2.22E-14	chr:19:21841536
19	rs115218914	21950948	G	6.473	2.44E-14	chr:19:21841536
19	rs112816324	21820984	T	4.188	2.81E-14	chr:19:21841536
19	rs141526653	21894950	A	5.915	3.04E-14	chr:19:21841536
19	rs115586860	21899429	G	6.542	3.18E-14	chr:19:21841536
19	rs116345357	21923374	T	6.542	3.18E-14	chr:19:21841536
19	rs116654331	21918591	A	6.542	3.18E-14	chr:19:21841536
19	rs144690685	21897717	G	6.542	3.18E-14	chr:19:21841536
19	rs150203019	21916698	A	6.542	3.18E-14	chr:19:21841536
19	rs56168029	21942877	A	0.2875	3.49E-14	chr:19:21841536
19	rs112450146	21821327	A	8.407	3.76E-14	chr:19:21841536
19	rs141892265	21965652	C	4.815	4.06E-14	chr:19:21841536
19	rs10421113	21929885	T	3.108	4.33E-14	chr:19:21841536
19	rs10421342	21930003	T	3.108	4.33E-14	chr:19:21841536
19	rs4808295	21930317	A	3.108	4.33E-14	chr:19:21841536
19	rs4809175	21938744	G	3.108	4.33E-14	chr:19:21841536
19	rs11085492	21945186	G	3.116	4.52E-14	chr:19:21841536
19	rs4613201	21927706	C	3.098	5.47E-14	chr:19:21841536
19	rs10405075	21943707	C	3.073	6.62E-14	chr:19:21841536
19	rs10412225	21944450	G	3.073	6.62E-14	chr:19:21841536
19	rs112439638	21962740	A	5.483	9.24E-14	chr:19:21841536
19	rs66562280	21833014	C	0.1039	1.08E-13	chr:19:21841536
19	rs115368414	22032475	T	9.643	1.13E-13	chr:19:21841536
19	rs114079879	21955405	A	5.491	1.15E-13	chr:19:21841536
19	rs114219279	21956161	A	5.491	1.15E-13	chr:19:21841536
19	rs115947529	21837849	T	19.13	1.48E-13	chr:19:21841536
19	rs116257175	21843303	A	19.13	1.48E-13	chr:19:21841536
19	rs111445324	21732011	A	7.219	1.67E-13	chr:19:21841536
19	rs113702555	21744151	G	7.219	1.67E-13	chr:19:21841536
19	rs61623191	21985264	A	10.69	1.84E-13	chr:19:21841536
19	rs139923873	22000997	A	11.18	2.84E-13	chr:19:21841536
19	rs145633375	22005464	A	11.18	2.84E-13	chr:19:21841536
19	rs57484704	21905719	G	6.154	3.05E-13	chr:19:21841536
19	rs116509034	21926427	A	6.13	3.42E-13	chr:19:21841536
19	rs60856228	21979630	C	10.38	3.99E-13	chr:19:21841536
19	rs562143535	21958356	T	9.304	4.29E-13	chr:19:21841536
19	rs111824582	21888348	T	6.056	4.31E-13	chr:19:21841536
19	rs113790434	21888363	A	6.056	4.31E-13	chr:19:21841536
19	rs148627677	21887779	G	6.056	4.31E-13	chr:19:21841536
19	rs183423536	21891022	G	6.056	4.31E-13	chr:19:21841536
19	rs150056857	21978913	A	10.31	4.37E-13	chr:19:21841536
19	rs73536651	21805053	C	4.626	4.49E-13	chr:19:21841536

19	rs113232162	21897187	T	5.833	4.72E-13	chr:19:21841536
19	rs115624329	21961229	C	9.276	4.93E-13	chr:19:21841536
19	rs111585880	21913436	A	5.933	5.31E-13	chr:19:21841536
19	rs111610027	21927126	A	5.933	5.31E-13	chr:19:21841536
19	rs111733303	21894790	A	5.933	5.31E-13	chr:19:21841536
19	rs112621399	21933096	T	5.933	5.31E-13	chr:19:21841536
19	rs113489374	21897562	A	5.933	5.31E-13	chr:19:21841536
19	rs114302517	21922619	C	5.933	5.31E-13	chr:19:21841536
19	rs115620407	21923380	C	5.933	5.31E-13	chr:19:21841536
19	rs183171803	21937845	A	5.933	5.31E-13	chr:19:21841536
19	rs61467203	21925770	A	5.933	5.31E-13	chr:19:21841536
19	rs7250493	21941207	G	5.933	5.31E-13	chr:19:21841536
19	rs3942413	21848383	T	0.3406	5.43E-13	chr:19:21841536
19	rs12978078	21813444	G	4.135	5.92E-13	chr:19:21841536
19	rs1829500	21849298	T	0.3375	6.03E-13	chr:19:21841536
19	rs4278872	21850903	T	0.3375	6.03E-13	chr:19:21841536
19	rs7248709	21848686	T	0.3375	6.03E-13	chr:19:21841536
19	rs8108393	21846846	C	0.3375	6.03E-13	chr:19:21841536
19	rs141542153	21734291	C	2.691	6.15E-13	chr:19:21841536
19	rs58560061	21950179	T	5.945	7.68E-13	chr:19:21841536
19	rs9755064	21891183	G	5.963	7.98E-13	chr:19:21841536
19	rs148992091	21751206	C	6.306	8.95E-13	chr:19:21841536
19	rs10425489	21856926	A	0.3442	9.96E-13	chr:19:21841536
19	rs6511283	21867819	T	0.3631	1.01E-12	chr:19:21841536
19	rs60179888	21804408	G	4.433	1.11E-12	chr:19:21841536
19	rs2359402	21855832	C	0.3443	1.49E-12	chr:19:21841536
19	rs2359393	21854152	A	0.3508	1.50E-12	chr:19:21841536
19	rs116095496	21938262	C	5.613	1.54E-12	chr:19:21841536
19	rs76195912	21784376	G	4.065	1.54E-12	chr:19:21841536
19	rs60451543	21786214	A	4.108	1.54E-12	chr:19:21841536
19	rs8111885	21733334	G	2.672	1.57E-12	chr:19:21841536
19	rs1401926	21740932	T	2.649	2.17E-12	chr:19:21841536
19	rs1879237	21738727	G	2.649	2.17E-12	chr:19:21841536
19	rs1879238	21738753	C	2.649	2.17E-12	chr:19:21841536
19	rs7253559	21740602	A	2.649	2.17E-12	chr:19:21841536
19	rs8109536	21736351	C	2.649	2.17E-12	chr:19:21841536
19	rs8110459	21732379	A	2.649	2.17E-12	chr:19:21841536
19	rs57908208	21844047	A	0.3407	2.27E-12	chr:19:21841536
19	rs11085456	21725386	T	2.642	2.59E-12	chr:19:21841536
19	rs12973084	21723197	T	2.642	2.59E-12	chr:19:21841536
19	rs7251540	21727927	G	2.642	2.59E-12	chr:19:21841536
19	rs7251669	21728019	G	2.642	2.59E-12	chr:19:21841536
19	rs9789284	21727511	C	2.642	2.59E-12	chr:19:21841536

19	rs73552497	21784106	C	3.93	2.85E-12	chr:19:21841536
19	rs2562496	21739870	C	2.603	2.91E-12	chr:19:21841536
19	rs4611587	21743971	G	2.603	2.91E-12	chr:19:21841536
19	rs78823434	21982409	G	4.508	3.04E-12	chr:19:21841536
19	rs115965563	21953738	T	6.747	3.18E-12	chr:19:21841536
19	rs34040704	21739464	G	0.3807	4.37E-12	chr:19:21841536
19	rs12710186	21958004	C	0.3395	5.12E-12	chr:19:21841536
19	rs12461802	21725407	A	0.3816	5.19E-12	chr:19:21841536
19	rs12973147	21860793	G	0.3456	5.51E-12	chr:19:21841536
19	rs12975447	21860974	G	0.3456	5.51E-12	chr:19:21841536
19	rs12985995	21723036	T	0.3819	5.74E-12	chr:19:21841536
19	rs149800591	22007547	G	4.39	1.07E-11	chr:19:21841536
19	rs114427176	22008878	C	4.59	1.15E-11	chr:19:21841536
19	rs114999467	22014978	G	4.59	1.15E-11	chr:19:21841536
19	rs115539747	21994401	C	4.59	1.15E-11	chr:19:21841536
19	rs116255808	22009296	C	4.59	1.15E-11	chr:19:21841536
19	rs116584441	21995470	T	4.59	1.15E-11	chr:19:21841536
19	rs116636330	22025850	C	4.59	1.15E-11	chr:19:21841536
19	rs8108565	21980435	T	4.577	1.26E-11	chr:19:21841536
19	rs116749192	21845739	A	11.6	1.57E-11	chr:19:21841536
19	rs116480376	22005732	C	4.451	1.63E-11	chr:19:21841536
19	rs62108422	21813454	G	2.894	1.72E-11	chr:19:21841536
19	rs11085494	21952631	T	0.3863	1.76E-11	chr:19:21841536
19	rs2015706	21858208	T	2.659	1.79E-11	chr:19:21841536
19	rs145764433	21834212	T	19.74	2.67E-11	chr:19:21841536
19	rs146308222	21842944	T	19.74	2.67E-11	chr:19:21841536
19	rs8103024	21835228	G	19.74	2.67E-11	chr:19:21841536
19	rs58952031	21804541	C	2.557	2.73E-11	chr:19:21841536
19	rs62109860	21833294	C	0.2649	2.87E-11	chr:19:21841536
19	rs116736619	22017198	C	4.379	2.88E-11	chr:19:21841536
19	rs111522182	21835996	C	4.572	3.27E-11	chr:19:21841536
19	rs113981438	21737410	G	3.902	3.44E-11	chr:19:21841536
19	rs57706029	21953161	T	0.3325	3.45E-11	chr:19:21841536
19	rs116811099	22034436	A	4.177	3.66E-11	chr:19:21841536
19	rs12610154	21676357	T	2.519	3.85E-11	chr:19:21841536
19	rs28898308	21696860	T	0.398	4.13E-11	chr:19:21841536
19	rs10408050	21852423	C	0.3416	4.23E-11	chr:19:21841536
19	rs115454713	21822330	T	3.834	4.30E-11	chr:19:21841536
19	rs116464036	21822329	T	3.834	4.30E-11	chr:19:21841536
19	rs12462082	21974486	T	3.659	4.45E-11	chr:19:21841536
19	rs1464295	21757867	C	2.549	4.67E-11	chr:19:21841536
19	rs114275324	22029050	T	4.372	4.73E-11	chr:19:21841536
19	rs116234413	22028375	A	4.372	4.73E-11	chr:19:21841536

19	rs7248444	22032146	T	4.372	4.73E-11	chr:19:21841536
19	rs8113074	21775494	T	2.619	4.83E-11	chr:19:21841536
19	rs116340972	21876058	C	12.07	5.21E-11	chr:19:21841536
19	rs73552476	21776939	G	4.343	6.51E-11	chr:19:21841536
19	rs12975424	21698865	T	2.509	8.06E-11	chr:19:21841536
19	rs144386887	21825423	T	6.065	8.26E-11	chr:19:21841536
19	rs4381721	21951581	T	0.3969	8.33E-11	chr:19:21841536
19	rs4522535	21951615	T	0.3972	8.49E-11	chr:19:21841536
19	rs116605460	21847926	C	10.62	9.21E-11	chr:19:21841536
19	rs4574048	21743921	C	2.444	1.10E-10	chr:19:21841536
19	rs9676253	21969452	A	0.3579	1.16E-10	chr:19:21841536
19	rs73012155	21813922	G	2.484	1.20E-10	chr:19:21841536
19	rs149207035	21856312	T	7.774	1.60E-10	chr:19:21841536
19	rs12972593	21932574	C	0.3921	1.65E-10	chr:19:21841536
19	rs10412709	21865359	A	0.4012	1.91E-10	chr:19:21841536
19	rs11879643	21816374	A	2.449	2.06E-10	chr:19:21841536
19	rs11883066	21816179	T	2.449	2.06E-10	chr:19:21841536
19	rs11883127	21816337	A	2.449	2.06E-10	chr:19:21841536
19	rs4809160	21815548	A	2.449	2.06E-10	chr:19:21841536
19	rs4809161	21815823	G	2.449	2.06E-10	chr:19:21841536
19	rs55802558	21814240	T	2.449	2.06E-10	chr:19:21841536
19	rs56208843	21814116	A	2.449	2.06E-10	chr:19:21841536
19	rs56311814	21814190	T	2.449	2.06E-10	chr:19:21841536
19	rs7256368	21842236	G	0.3756	2.27E-10	chr:19:21841536
19	rs6511291	21950402	C	0.3945	2.31E-10	chr:19:21841536
19	rs35717293	21912755	G	0.3846	2.66E-10	chr:19:21841536
19	rs62108416	21812729	T	2.546	2.71E-10	chr:19:21841536
19	rs7245671	21812597	C	2.546	2.71E-10	chr:19:21841536
19	rs7260048	21812636	T	2.546	2.71E-10	chr:19:21841536
19	rs4319878	21924452	T	0.3861	2.73E-10	chr:19:21841536
19	rs7251810	21782572	A	2.45	3.10E-10	chr:19:21841536
19	rs113009301	21768087	A	4.087	3.54E-10	chr:19:21841536
19	rs73550367	21758542	G	4.087	3.54E-10	chr:19:21841536
19	rs73552443	21765384	G	4.087	3.54E-10	chr:19:21841536
19	rs73552446	21766756	G	4.087	3.54E-10	chr:19:21841536
19	rs62108418	21813273	G	2.536	3.84E-10	chr:19:21841536
19	rs62108419	21813299	T	2.536	3.84E-10	chr:19:21841536
19	rs62108420	21813354	C	2.536	3.84E-10	chr:19:21841536
19	rs73012144	21812991	A	2.536	3.84E-10	chr:19:21841536
19	rs113472792	21877404	T	5.92	3.98E-10	chr:19:21841536
19	rs8113652	21921709	C	0.3989	4.05E-10	chr:19:21841536
19	rs562777226	21812683	A	2.505	4.15E-10	chr:19:21841536
19	rs62108415	21812707	T	2.505	4.15E-10	chr:19:21841536

19	rs11665855	21784516	C	2.385	4.37E-10	chr:19:21841536
19	rs34810593	21825360	G	0.2819	4.93E-10	chr:19:21841536
19	rs11881486	21813528	A	2.414	4.98E-10	chr:19:21841536
19	rs11881496	21813801	G	2.414	4.98E-10	chr:19:21841536
19	rs62108423	21813710	A	2.414	4.98E-10	chr:19:21841536
19	rs73552469	21772391	A	3.877	5.00E-10	chr:19:21841536
19	rs73536671	21813150	T	2.5	5.56E-10	chr:19:21841536
19	rs59039597	21877989	A	27.47	5.71E-10	chr:19:21841536
19	rs7246400	21909948	A	0.3937	5.83E-10	chr:19:21841536
19	rs56177049	21774271	G	3.789	6.12E-10	chr:19:21841536
19	rs61242234	21776305	T	3.789	6.12E-10	chr:19:21841536
19	rs7252798	21908053	C	0.4052	6.26E-10	chr:19:21841536
19	rs61037485	21924189	A	0.3952	7.17E-10	chr:19:21841536
19	rs74755925	21813062	T	2.467	7.84E-10	chr:19:21841536
19	rs8102320	21936885	G	0.4095	8.66E-10	chr:19:21841536
19	rs58267832	21864436	G	9.546	9.07E-10	chr:19:21841536
19	rs6511285	21890339	A	0.2536	9.62E-10	chr:19:21841536
19	rs115229801	21828695	G	11.86	1.03E-09	chr:19:21841536
19	rs58691880	21804749	G	3.9	1.19E-09	chr:19:21841536
19	rs34497957	21693426	C	0.431	1.42E-09	chr:19:21841536
19	rs7245571	21768899	G	2.28	1.43E-09	chr:19:21841536
19	rs4462731	21689603	T	0.4315	1.47E-09	chr:19:21841536
19	rs147044062	21694415	A	0.4332	1.55E-09	chr:19:21841536
19	rs35782355	21719401	C	0.434	1.56E-09	chr:19:21841536
19	rs7250360	21718822	G	0.434	1.56E-09	chr:19:21841536
19	rs12974329	21700400	T	0.4304	1.70E-09	chr:19:21841536
19	rs60559953	21793334	G	3.857	1.86E-09	chr:19:21841536
19	rs73931207	21794073	A	3.857	1.86E-09	chr:19:21841536
19	rs75788756	21755046	A	3.95	1.95E-09	chr:19:21841536
19	rs10411885	21840889	G	0.3321	2.14E-09	chr:19:21841536
19	rs6511255	21692027	G	0.4352	2.18E-09	chr:19:21841536
19	rs7252254	21687561	A	0.4352	2.18E-09	chr:19:21841536
19	rs7257186	21697444	A	0.4352	2.18E-09	chr:19:21841536
19	rs73017722	21688495	T	0.4352	2.18E-09	chr:19:21841536
19	rs116659024	21843617	C	0.3229	2.25E-09	chr:19:21841536
19	rs2435000	22011115	T	0.2699	2.27E-09	chr:19:21841536
19	rs8101900	21699047	C	0.4346	2.38E-09	chr:19:21841536
19	rs10406256	21839958	C	0.3332	2.41E-09	chr:19:21841536
19	rs11085489	21862213	G	0.4188	2.58E-09	chr:19:21841536
19	rs111470979	21761884	G	3.649	2.58E-09	chr:19:21841536
19	rs188552296	21760265	G	3.649	2.58E-09	chr:19:21841536
19	rs61563055	21761609	T	3.649	2.58E-09	chr:19:21841536
19	rs7254417	21771069	A	3.649	2.58E-09	chr:19:21841536

19	rs2133822	21685102	A	0.437	2.78E-09	chr:19:21841536
19	rs7256389	21695797	T	0.4358	2.88E-09	chr:19:21841536
19	rs8106840	21696458	A	0.4358	2.88E-09	chr:19:21841536
19	rs10407311	21834510	C	0.3368	3.25E-09	chr:19:21841536
19	rs10409373	21836608	C	0.3368	3.25E-09	chr:19:21841536
19	rs8103138	21835313	G	0.3368	3.25E-09	chr:19:21841536
19	rs10407896	22019444	G	0.288	3.39E-09	chr:19:21841536
19	rs2244127	22016695	A	0.288	3.39E-09	chr:19:21841536
19	rs8103969	21835257	C	0.3367	3.43E-09	chr:19:21841536
19	rs8104073	21835293	C	0.3367	3.43E-09	chr:19:21841536
19	rs57073163	21876331	A	2.837	3.45E-09	chr:19:21841536
19	rs10416719	21823729	T	0.3438	3.62E-09	chr:19:21841536
19	rs10418097	21823792	G	0.3438	3.62E-09	chr:19:21841536
19	rs11085488	21862210	G	0.4231	3.64E-09	chr:19:21841536
19	rs201468350	21959256	T	3.021	3.91E-09	chr:19:21841536
19	rs111727989	21765946	T	3.573	3.95E-09	chr:19:21841536
19	rs12975399	21765794	C	2.252	4.08E-09	chr:19:21841536
19	rs8101089	21676173	A	0.4405	4.10E-09	chr:19:21841536
19	rs73550372	21760873	A	3.561	4.21E-09	chr:19:21841536
19	rs2457792	22030057	G	0.2911	4.47E-09	chr:19:21841536
19	rs11666680	21681788	T	0.4416	4.84E-09	chr:19:21841536
19	rs10416598	21823852	T	0.3308	5.39E-09	chr:19:21841536
19	rs116273517	21859087	C	4.997	5.45E-09	chr:19:21841536
19	rs116378385	21857155	A	4.997	5.45E-09	chr:19:21841536
19	rs9304991	21856732	T	0.4409	5.51E-09	chr:19:21841536
19	rs2457784	22026514	A	0.2939	5.87E-09	chr:19:21841536
19	rs58686910	21799119	A	3.604	6.36E-09	chr:19:21841536
19	rs10419466	21842094	G	0.332	6.38E-09	chr:19:21841536
19	rs9304990	21841379	C	0.332	6.38E-09	chr:19:21841536
19	rs62110790	21867868	C	0.4218	6.57E-09	chr:19:21841536
19	rs113759090	21850317	A	7.505	9.30E-09	chr:19:21841536
19	rs140359291	21853986	G	7.505	9.30E-09	chr:19:21841536
19	rs10425631	21839322	G	0.3367	1.04E-08	chr:19:21841536
19	rs1397579	21976964	C	2.118	1.41E-08	chr:19:21841536
19	rs12979686	21975817	C	0.3161	1.43E-08	chr:19:21841536
19	rs7258901	21701504	A	0.4614	1.71E-08	chr:19:21841536
19	rs58399826	21783395	T	3.298	1.78E-08	chr:19:21841536
19	rs114842582	22008572	C	3.801	1.83E-08	chr:19:21841536
19	rs35230828	21738391	T	2.185	2.05E-08	chr:19:21841536
19	rs12983787	21591035	T	0.4575	2.61E-08	chr:19:21841536
19	rs12462022	21502836	A	0.4666	3.21E-08	chr:19:21841536
19	rs7259993	21812403	T	2.291	3.36E-08	chr:19:21841536
19	rs138592457	21826867	T	13.72	3.46E-08	chr:19:21841536

19	rs1818695	21858390	T	0.4673	3.49E-08	chr:19:21841536
19	rs10414467	21866004	A	0.3401	5.33E-08	chr:19:21841536
19	rs57197225	21722663	C	2.899	5.44E-08	chr:19:21841536
19	rs79602756	22045020	G	2.781	6.51E-08	chr:19:21841536
19	rs9304989	21824313	T	0.1537	7.33E-08	chr:19:21841536
19	rs35506714	21738138	C	2.298	1.01E-07	chr:19:21841536
19	rs140947336	21856697	A	8.175	1.03E-07	chr:19:21841536
19	rs7253749	21953828	T	0.4275	1.15E-07	chr:19:21841536
19	rs8101143	21956136	G	0.4297	1.24E-07	chr:19:21841536
19	rs111515495	21870482	T	3.361	1.38E-07	chr:19:21841536
19	rs12461329	21860597	T	0.3264	1.46E-07	chr:19:21841536
19	rs12973337	21860723	A	0.3264	1.46E-07	chr:19:21841536
19	rs7246819	21529440	G	0.4829	1.55E-07	chr:19:21841536
19	rs7257326	21532065	C	0.4839	1.67E-07	chr:19:21841536
19	rs10164399	21856722	T	0.3196	1.79E-07	chr:19:21841536
19	rs116497973	21860513	T	25.11	1.79E-07	chr:19:21841536
19	rs8110690	21549668	G	0.485	1.91E-07	chr:19:21841536
19	rs4075278	21961172	G	0.4325	2.19E-07	chr:19:21841536
19	rs11085450	21637051	T	1.983	2.22E-07	chr:19:21841536
19	rs189340667	21718301	T	2.75	2.27E-07	chr:19:21841536
19	rs73550345	21752141	T	0.4642	2.27E-07	chr:19:21841536
19	rs12459069	21852101	G	0.3275	2.29E-07	chr:19:21841536
19	rs2359394	21854169	G	0.3296	2.35E-07	chr:19:21841536
19	rs2359395	21854176	A	0.3296	2.35E-07	chr:19:21841536
19	rs2359396	21854179	A	0.3296	2.35E-07	chr:19:21841536
19	rs1080829	21461135	A	0.4878	2.42E-07	chr:19:21841536
19	rs7259739	21960407	C	0.4384	2.52E-07	chr:19:21841536
19	rs114757397	21976932	G	7.522	2.56E-07	chr:19:21841536
19	rs4808279	21752009	A	0.4831	2.72E-07	chr:19:21841536
19	rs2359401	21855732	A	0.3319	2.74E-07	chr:19:21841536
19	rs5924665	21865290	A	0.4336	2.80E-07	chr:19:21841536
19	rs7259051	21529237	A	0.4902	2.85E-07	chr:19:21841536
19	rs12986333	21821460	A	0.429	2.92E-07	chr:19:21841536
19	rs12982471	21816186	A	0.4284	2.96E-07	chr:19:21841536
19	rs10422054	21985317	T	0.4437	3.03E-07	chr:19:21841536
19	rs12459622	21852799	G	0.3322	3.04E-07	chr:19:21841536
19	rs12459640	21852807	T	0.3322	3.04E-07	chr:19:21841536
19	rs10164402	21856407	C	0.3319	3.10E-07	chr:19:21841536
19	rs10164404	21856699	C	0.3319	3.10E-07	chr:19:21841536
19	rs10854059	21859805	A	0.3319	3.10E-07	chr:19:21841536
19	rs12459105	21852322	C	0.3319	3.10E-07	chr:19:21841536
19	rs12459145	21852274	C	0.3319	3.10E-07	chr:19:21841536
19	rs12459146	21852286	A	0.3319	3.10E-07	chr:19:21841536

19	rs12459607	21852601	T	0.3319	3.10E-07	chr:19:21841536
19	rs12459653	21852679	A	0.3319	3.10E-07	chr:19:21841536
19	rs12459663	21852921	A	0.3319	3.10E-07	chr:19:21841536
19	rs12460255	21853648	A	0.3319	3.10E-07	chr:19:21841536
19	rs12460259	21853849	G	0.3319	3.10E-07	chr:19:21841536
19	rs12460275	21853896	T	0.3319	3.10E-07	chr:19:21841536
19	rs12463364	21851610	G	0.3319	3.10E-07	chr:19:21841536
19	rs12972399	21850149	C	0.3319	3.10E-07	chr:19:21841536
19	rs12982600	21847694	A	0.3319	3.10E-07	chr:19:21841536
19	rs12983456	21855124	A	0.3319	3.10E-07	chr:19:21841536
19	rs12984472	21847713	C	0.3319	3.10E-07	chr:19:21841536
19	rs1818696	21850359	T	0.3319	3.10E-07	chr:19:21841536
19	rs1850931	21855226	A	0.3319	3.10E-07	chr:19:21841536
19	rs1850932	21855223	T	0.3319	3.10E-07	chr:19:21841536
19	rs1850934	21854928	C	0.3319	3.10E-07	chr:19:21841536
19	rs1850935	21854623	C	0.3319	3.10E-07	chr:19:21841536
19	rs2015715	21858115	T	0.3319	3.10E-07	chr:19:21841536
19	rs2015736	21857927	T	0.3319	3.10E-07	chr:19:21841536
19	rs2037356	21849559	T	0.3319	3.10E-07	chr:19:21841536
19	rs2359388	21851081	C	0.3319	3.10E-07	chr:19:21841536
19	rs2359389	21851157	G	0.3319	3.10E-07	chr:19:21841536
19	rs2359390	21851459	A	0.3319	3.10E-07	chr:19:21841536
19	rs2359392	21854136	G	0.3319	3.10E-07	chr:19:21841536
19	rs2359398	21854389	T	0.3319	3.10E-07	chr:19:21841536
19	rs2359399	21854394	T	0.3319	3.10E-07	chr:19:21841536
19	rs2359400	21855574	T	0.3319	3.10E-07	chr:19:21841536
19	rs2359405	21856187	T	0.3319	3.10E-07	chr:19:21841536
19	rs3946575	21848698	T	0.3319	3.10E-07	chr:19:21841536
19	rs4302166	21850861	T	0.3319	3.10E-07	chr:19:21841536
19	rs4310994	21850923	G	0.3319	3.10E-07	chr:19:21841536
19	rs4321314	21849668	A	0.3319	3.10E-07	chr:19:21841536
19	rs4490112	21850753	A	0.3319	3.10E-07	chr:19:21841536
19	rs4500887	21850985	A	0.3319	3.10E-07	chr:19:21841536
19	rs7259640	21848238	A	0.3319	3.10E-07	chr:19:21841536
19	rs8108289	21846845	G	0.3319	3.10E-07	chr:19:21841536
19	rs181740008	21844179	T	16.32	3.13E-07	chr:19:21841536
19	rs10084114	21821966	G	0.4304	3.14E-07	chr:19:21841536
19	rs58679179	21879499	A	16.36	3.28E-07	chr:19:21841536
19	rs143149382	21823568	T	23.07	3.75E-07	chr:19:21841536
19	rs1533675	21777706	A	2.247	3.76E-07	chr:19:21841536
19	rs7256700	21820763	T	0.4341	3.82E-07	chr:19:21841536
19	rs10164296	21856328	T	0.3352	3.93E-07	chr:19:21841536
19	rs12461611	21856280	T	0.3352	3.93E-07	chr:19:21841536

19	rs34449776	21856309	G	0.3352	3.93E-07	chr:19:21841536
19	rs35091166	21856300	A	0.3352	3.93E-07	chr:19:21841536
19	rs12972511	21822010	T	0.434	3.95E-07	chr:19:21841536
19	rs2435023	22066349	C	0.4792	3.95E-07	chr:19:21841536
19	rs2435024	22066306	C	0.4792	3.95E-07	chr:19:21841536
19	rs2359403	21855836	T	0.3361	3.99E-07	chr:19:21841536
19	rs2359404	21855839	A	0.3361	3.99E-07	chr:19:21841536
19	rs4026206	21855856	A	0.3361	3.99E-07	chr:19:21841536
19	rs4075277	21961173	C	0.443	4.11E-07	chr:19:21841536
19	rs192866411	21890362	G	7.331	4.20E-07	chr:19:21841536
19	rs148893136	21824764	T	9.732	4.35E-07	chr:19:21841536
19	rs74762678	22019157	C	9.335	4.48E-07	chr:19:21841536
19	rs75937847	22031912	A	9.335	4.48E-07	chr:19:21841536
19	rs8111923	22021828	A	9.335	4.48E-07	chr:19:21841536
19	rs11085466	21751811	C	0.4754	4.96E-07	chr:19:21841536
19	rs11668774	21859863	G	0.342	5.00E-07	chr:19:21841536
19	rs1818693	21858687	G	0.342	5.00E-07	chr:19:21841536
19	rs2015476	21859110	C	0.342	5.00E-07	chr:19:21841536
19	rs2015481	21858994	G	0.342	5.00E-07	chr:19:21841536
19	rs68086564	21813877	A	0.4395	5.34E-07	chr:19:21841536
19	rs12710189	22006625	A	0.453	5.41E-07	chr:19:21841536
19	rs7251082	21669802	G	1.948	5.59E-07	chr:19:21841536
19	rs147394420	21836414	G	22.11	6.06E-07	chr:19:21841536
19	rs139828537	21823298	G	12.19	6.07E-07	chr:19:21841536
19	rs1829503	21869343	G	1.94	6.15E-07	chr:19:21841536
19	rs12609186	21984052	C	0.5079	6.36E-07	chr:19:21841536
19	rs11668682	21666161	C	1.939	6.81E-07	chr:19:21841536
19	rs56346453	21665484	G	1.939	6.81E-07	chr:19:21841536
19	rs7250838	21669642	G	1.939	6.81E-07	chr:19:21841536
19	rs7251620	21669681	T	1.939	6.81E-07	chr:19:21841536
19	rs9917101	21854552	G	0.3745	7.36E-07	chr:19:21841536
19	rs113751886	21713937	C	2.115	7.65E-07	chr:19:21841536
19	rs10425298	21847634	T	0.5026	7.94E-07	chr:19:21841536
19	rs60083403	21634949	T	2.589	8.36E-07	chr:19:21841536
19	rs115973453	21833961	T	11.23	9.15E-07	chr:19:21841536
19	rs73550355	21755034	A	2.706	9.92E-07	chr:19:21841536
19	rs557545096	21718308	C	5.488	1.01E-06	chr:19:21841536
19	rs1581300	21867835	G	0.4822	1.01E-06	chr:19:21841536
19	rs8107706	21636997	A	2.436	1.06E-06	chr:19:21841536
19	rs56217825	21709797	A	2.681	1.10E-06	chr:19:21841536
19	rs61115928	21692055	G	2.681	1.10E-06	chr:19:21841536
19	rs61663351	21709444	T	2.681	1.10E-06	chr:19:21841536
19	rs139810035	21858645	C	15.32	1.12E-06	chr:19:21841536

19	rs8108605	21683011	G	2.604	1.37E-06	chr:19:21841536
19	rs113751359	21721174	T	2.632	1.57E-06	chr:19:21841536
19	rs12971307	21813063	C	0.4914	1.57E-06	chr:19:21841536
19	rs7257308	21644822	T	2.575	1.61E-06	chr:19:21841536
19	rs111409905	21838129	T	14.06	1.74E-06	chr:19:21841536
19	rs113249059	21687009	A	2.604	1.93E-06	chr:19:21841536
19	rs73926349	21686226	C	2.619	1.98E-06	chr:19:21841536
19	rs150234937	21913596	A	6.81	2.13E-06	chr:19:21841536
19	rs150575565	21915047	G	6.81	2.13E-06	chr:19:21841536
19	rs7248761	21866423	G	0.5332	2.14E-06	chr:19:21841536
19	rs140259531	21693877	T	2.308	2.44E-06	chr:19:21841536
19	rs4462730	21689276	G	2.308	2.44E-06	chr:19:21841536
19	rs1589280	21863419	C	0.5346	2.45E-06	chr:19:21841536
19	rs7249701	21866865	T	0.5346	2.45E-06	chr:19:21841536
19	rs8108212	21845320	A	0.308	2.48E-06	chr:19:21841536
19	rs150430144	21972159	G	3.66	2.48E-06	chr:19:21841536
19	rs138499271	21871425	T	8.939	2.57E-06	chr:19:21841536
19	rs138521730	21874443	T	32.78	2.69E-06	chr:19:21841536
19	rs34573540	21821470	C	0.496	2.86E-06	chr:19:21841536
19	rs59543267	21704335	C	2.287	3.08E-06	chr:19:21841536
19	rs7254975	21700744	T	2.287	3.08E-06	chr:19:21841536
19	rs73548606	21697397	G	2.287	3.08E-06	chr:19:21841536
19	rs73548618	21707058	A	2.287	3.08E-06	chr:19:21841536
19	rs73548628	21721540	C	2.287	3.08E-06	chr:19:21841536
19	rs7255176	21700682	C	2.273	3.27E-06	chr:19:21841536
19	rs11668453	21759357	A	0.4826	3.41E-06	chr:19:21841536
19	rs75079001	21757375	G	0.4826	3.41E-06	chr:19:21841536
19	rs75211977	21760201	A	0.4826	3.41E-06	chr:19:21841536
19	rs76881507	21762189	T	0.4826	3.41E-06	chr:19:21841536
19	rs8103528	21761273	T	0.4826	3.41E-06	chr:19:21841536
19	rs112471014	21683716	A	2.513	3.49E-06	chr:19:21841536
19	rs4462729	21689023	T	2.513	3.49E-06	chr:19:21841536
19	rs7259339	21693263	T	2.513	3.49E-06	chr:19:21841536
19	rs73546766	21673034	G	2.513	3.49E-06	chr:19:21841536
19	rs73546769	21675043	T	2.513	3.49E-06	chr:19:21841536
19	rs73546782	21683487	T	2.513	3.49E-06	chr:19:21841536
19	rs73546785	21684151	A	2.513	3.49E-06	chr:19:21841536
19	rs73546787	21684230	A	2.513	3.49E-06	chr:19:21841536
19	rs73546789	21687603	C	2.513	3.49E-06	chr:19:21841536
19	rs144073556	21872570	G	31.47	3.61E-06	chr:19:21841536
19	rs73552468	21772097	A	0.4852	3.85E-06	chr:19:21841536
19	rs10415147	21953645	C	0.4103	3.88E-06	chr:19:21841536
19	rs1818698	21867499	A	0.5436	4.13E-06	chr:19:21841536

19	rs10423827	21957137	T	0.4099	4.32E-06	chr:19:21841536
19	rs13346947	21957309	T	0.4099	4.32E-06	chr:19:21841536
19	rs76695906	21766710	A	0.4859	4.40E-06	chr:19:21841536
19	rs1818697	21867497	T	0.5445	4.48E-06	chr:19:21841536
19	rs10413799	21953773	G	0.4105	4.50E-06	chr:19:21841536
19	rs10414913	21954204	T	0.4105	4.50E-06	chr:19:21841536
19	rs114861132	21852398	T	0.3261	4.53E-06	chr:19:21841536
19	rs73546778	21681944	G	2.47	4.91E-06	chr:19:21841536
19	rs3935082	21960911	T	0.4	5.04E-06	chr:19:21841536
19	rs3935083	21960619	T	0.4	5.04E-06	chr:19:21841536
19	rs4452078	21960577	A	0.4	5.04E-06	chr:19:21841536
19	rs6511294	21978569	G	1.845	5.07E-06	chr:19:21841536
19	rs12463222	21863913	T	0.2909	5.43E-06	chr:19:21841536
19	rs12985113	21864344	T	0.2909	5.43E-06	chr:19:21841536
19	rs112199629	21594395	A	2.433	5.70E-06	chr:19:21841536
19	rs73539898	21526435	C	2.42	5.70E-06	chr:19:21841536
19	rs112689818	21824443	C	9.358	6.11E-06	chr:19:21841536
19	rs543408085	21718140	C	2.303	6.14E-06	chr:19:21841536
19	rs58214336	21958607	A	0.4039	6.27E-06	chr:19:21841536
19	rs113878987	21491216	A	2.394	6.36E-06	chr:19:21841536
19	rs60543700	21473099	T	2.394	6.36E-06	chr:19:21841536
19	rs59391927	21783487	T	0.4973	6.37E-06	chr:19:21841536
19	rs115753254	21970269	C	6.311	7.01E-06	chr:19:21841536
19	rs563762240	21959213	T	6.311	7.01E-06	chr:19:21841536
19	rs9973270	21814919	T	0.5047	7.10E-06	chr:19:21841536
19	rs4399664	21778026	T	0.5017	7.93E-06	chr:19:21841536
19	rs10402184	21842973	A	0.3649	7.93E-06	chr:19:21841536
19	rs12985935	21821448	G	0.512	8.30E-06	chr:19:21841536
19	rs113788337	21850952	G	27.72	8.58E-06	chr:19:21841536
19	rs10084110	21821537	A	0.5131	8.79E-06	chr:19:21841536
19	rs146835848	21525805	T	2.379	8.92E-06	chr:19:21841536
19	rs59645800	21538453	C	2.379	8.92E-06	chr:19:21841536
19	rs6511236	21570590	T	2.379	8.92E-06	chr:19:21841536
19	rs73541828	21530791	C	2.379	8.92E-06	chr:19:21841536
19	rs73547786	21568888	G	2.379	8.92E-06	chr:19:21841536
19	rs73926973	21494541	A	2.379	8.92E-06	chr:19:21841536
19	rs11669043	21614714	T	1.802	8.99E-06	chr:19:21841536
19	rs73535998	21482386	T	2.336	9.04E-06	chr:19:21841536
19	rs113381212	21868580	T	3.515	9.11E-06	chr:19:21841536
19	rs2292986	21776332	A	0.5034	9.19E-06	chr:19:21841536
19	rs7252817	21783157	A	0.4696	9.31E-06	chr:19:21841536
19	rs12985947	21820111	C	0.5272	9.41E-06	chr:19:21841536
19	rs2435036	22039105	T	0.4484	1.00E-05	chr:19:21841536

19	rs7257080	21820858	A	0.5148	1.02E-05	chr:19:21841536
19	rs12609376	21817102	G	0.5186	1.02E-05	chr:19:21841536
19	rs12611302	21817486	C	0.5186	1.02E-05	chr:19:21841536
19	rs144197730	21832349	T	6.411	1.08E-05	chr:19:21841536
19	rs112300130	21866056	C	26.76	1.11E-05	chr:19:21841536
19	rs141251254	21843366	T	26.76	1.11E-05	chr:19:21841536
19	rs142410630	21842348	T	26.76	1.11E-05	chr:19:21841536
19	rs148504899	21853608	T	26.76	1.11E-05	chr:19:21841536
19	rs182785858	21853425	T	26.76	1.11E-05	chr:19:21841536
19	rs549257374	21864596	A	26.76	1.11E-05	chr:19:21841536
19	rs60585877	21864195	G	26.76	1.11E-05	chr:19:21841536
19	rs12462709	21989391	C	6.539	1.11E-05	chr:19:21841536
19	rs58664716	21993467	G	6.539	1.11E-05	chr:19:21841536
19	rs76621431	22014914	C	6.539	1.11E-05	chr:19:21841536
19	rs77216709	21992940	G	6.539	1.11E-05	chr:19:21841536
19	rs12979060	21843727	A	0.3405	1.12E-05	chr:19:21841536
19	rs77115676	21985611	A	7.19	1.14E-05	chr:19:21841536
19	rs79327994	21993765	T	7.19	1.14E-05	chr:19:21841536
19	rs28498118	21647585	T	1.791	1.14E-05	chr:19:21841536
19	rs139946230	21851689	G	4.762	1.15E-05	chr:19:21841536
19	rs189120125	21851671	G	4.762	1.15E-05	chr:19:21841536
19	rs572947750	21851672	T	4.762	1.15E-05	chr:19:21841536
19	rs111549912	22051478	C	2.551	1.16E-05	chr:19:21841536
19	rs112444568	22051581	T	2.551	1.16E-05	chr:19:21841536
19	rs114651586	21978760	A	5.599	1.19E-05	chr:19:21841536
19	rs16997799	21567787	C	2.344	1.19E-05	chr:19:21841536
19	rs12971926	21837430	T	0.3402	1.22E-05	chr:19:21841536
19	rs12975766	21840005	C	0.3402	1.22E-05	chr:19:21841536
19	rs1850930	21839462	G	0.3402	1.22E-05	chr:19:21841536
19	rs2139527	21839211	T	0.3402	1.22E-05	chr:19:21841536
19	rs187935733	22059949	T	2.559	1.23E-05	chr:19:21841536
19	rs12972184	21835628	C	0.3397	1.34E-05	chr:19:21841536
19	rs11669130	21654213	C	1.773	1.35E-05	chr:19:21841536
19	rs138491703	21965859	G	3.352	1.36E-05	chr:19:21841536
19	rs12978917	21813834	A	0.5381	1.40E-05	chr:19:21841536
19	rs12979910	21814189	A	0.5381	1.40E-05	chr:19:21841536
19	rs11883081	21816114	G	0.5361	1.40E-05	chr:19:21841536
19	rs12985836	21819861	G	0.5361	1.40E-05	chr:19:21841536
19	rs4808284	21815748	G	0.5361	1.40E-05	chr:19:21841536
19	rs113704561	21828995	T	4.83	1.42E-05	chr:19:21841536
19	rs149036654	21828173	G	4.83	1.42E-05	chr:19:21841536
19	rs35605606	22059609	A	2.253	1.46E-05	chr:19:21841536
19	rs12150872	21793510	T	0.5139	1.50E-05	chr:19:21841536

19	rs12150881	21793561	T	0.5139	1.50E-05	chr:19:21841536
19	rs12151328	21793265	A	0.5139	1.50E-05	chr:19:21841536
19	rs12972549	21822054	G	0.5205	1.54E-05	chr:19:21841536
19	rs150844285	22411568	G	0.01025	1.04E-31	chr:19:22414379
19	rs10425845	22370672	G	0.02516	8.60E-31	chr:19:22414379
19	rs55992911	22397853	C	25.02	1.88E-26	chr:19:22414379
19	rs463200	22327489	T	0.004768	1.51E-24	chr:19:22414379
19	rs557388	22353206	A	0.0039	2.24E-24	chr:19:22414379
19	rs10419384	22391239	G	20.94	6.87E-24	chr:19:22414379
19	rs1918161	22374023	T	20.94	6.87E-24	chr:19:22414379
19	rs2360002	22383362	A	20.94	6.87E-24	chr:19:22414379
19	rs281181	22396809	T	20.94	6.87E-24	chr:19:22414379
19	rs3934143	22392238	G	20.94	6.87E-24	chr:19:22414379
19	rs436431	22360919	T	20.94	6.87E-24	chr:19:22414379
19	rs7343077	22367925	T	20.94	6.87E-24	chr:19:22414379
19	rs281158	22416013	T	23.33	7.19E-24	chr:19:22414379
19	rs2606266	22409041	T	21.5	8.07E-24	chr:19:22414379
19	rs2606267	22409260	A	21.5	8.07E-24	chr:19:22414379
19	rs2606274	22413650	G	21.5	8.07E-24	chr:19:22414379
19	rs2694445	22408997	C	21.5	8.07E-24	chr:19:22414379
19	rs2548937	22413188	T	21.47	8.84E-24	chr:19:22414379
19	rs4025837	22413189	A	21.47	8.84E-24	chr:19:22414379
19	rs2694449	22404047	A	20.94	9.41E-24	chr:19:22414379
19	rs281193	22400609	A	20.94	9.41E-24	chr:19:22414379
19	rs281194	22400802	A	20.94	9.41E-24	chr:19:22414379
19	rs281195	22402480	T	20.94	9.41E-24	chr:19:22414379
19	rs281199	22405501	T	20.94	9.41E-24	chr:19:22414379
19	rs379619	22341905	C	21.42	2.39E-23	chr:19:22414379
19	rs113493783	22333975	G	21.33	2.71E-23	chr:19:22414379
19	rs142745046	22334261	C	21.33	2.71E-23	chr:19:22414379
19	rs411557	22333324	C	21.33	2.71E-23	chr:19:22414379
19	rs459017	22330531	C	21.33	2.71E-23	chr:19:22414379
19	rs459539	22330699	T	21.33	2.71E-23	chr:19:22414379
19	rs462577	22335500	A	21.33	2.71E-23	chr:19:22414379
19	rs462671	22328850	T	21.33	2.71E-23	chr:19:22414379
19	rs465978	22342421	A	21.33	2.71E-23	chr:19:22414379
19	rs78408635	22339824	G	21.33	2.71E-23	chr:19:22414379
19	rs79196382	22339817	A	21.33	2.71E-23	chr:19:22414379
19	rs281156	22414691	T	13	6.71E-23	chr:19:22414379
19	rs121382	22348657	C	0.004717	9.10E-23	chr:19:22414379
19	rs2522318	22350385	C	0.004717	9.10E-23	chr:19:22414379
19	rs366501	22352375	T	0.004717	9.10E-23	chr:19:22414379
19	rs399307	22345794	C	0.004717	9.10E-23	chr:19:22414379

19	rs408240	22346403	A	0.004717	9.10E-23	chr:19:22414379
19	rs427343	22346138	G	0.004717	9.10E-23	chr:19:22414379
19	rs445788	22354323	G	0.004717	9.10E-23	chr:19:22414379
19	rs451905	22345649	G	0.004717	9.10E-23	chr:19:22414379
19	rs112297561	22341110	A	0.004739	9.37E-23	chr:19:22414379
19	rs112826254	22334192	G	0.004739	9.37E-23	chr:19:22414379
19	rs371540	22344391	A	0.004739	9.37E-23	chr:19:22414379
19	rs395410	22336307	C	0.004739	9.37E-23	chr:19:22414379
19	rs420977	22335762	G	0.004739	9.37E-23	chr:19:22414379
19	rs421180	22335719	G	0.004739	9.37E-23	chr:19:22414379
19	rs457504	22331109	G	0.004739	9.37E-23	chr:19:22414379
19	rs461312	22333288	T	0.004739	9.37E-23	chr:19:22414379
19	rs461804	22328560	T	0.004739	9.37E-23	chr:19:22414379
19	rs465150	22342486	T	0.004739	9.37E-23	chr:19:22414379
19	rs465380	22335068	A	0.004739	9.37E-23	chr:19:22414379
19	rs56256419	22345457	C	0.004739	9.37E-23	chr:19:22414379
19	rs56284384	22345461	A	0.004739	9.37E-23	chr:19:22414379
19	rs76608558	22334370	T	0.004739	9.37E-23	chr:19:22414379
19	rs400419	22345405	C	0.004729	1.02E-22	chr:19:22414379
19	rs400661	22345337	C	0.004729	1.02E-22	chr:19:22414379
19	rs416928	22345369	G	0.004729	1.02E-22	chr:19:22414379
19	rs447533	22345393	G	0.004729	1.02E-22	chr:19:22414379
19	rs458172	22329400	C	0.01101	1.13E-22	chr:19:22414379
19	rs2204581	22325017	C	0.04556	1.38E-22	chr:19:22414379
19	rs382357	22343507	A	0.03135	1.63E-22	chr:19:22414379
19	rs386720	22342796	A	0.03135	1.63E-22	chr:19:22414379
19	rs414385	22338577	G	0.03135	1.63E-22	chr:19:22414379
19	rs421261	22334418	T	0.03135	1.63E-22	chr:19:22414379
19	rs546256	22339777	C	0.03135	1.63E-22	chr:19:22414379
19	rs78957365	22341032	T	0.03135	1.63E-22	chr:19:22414379
19	rs111471672	22410364	G	0.003013	3.03E-22	chr:19:22414379
19	rs449891	22352161	T	0.06121	6.71E-22	chr:19:22414379
19	rs430560	22353560	T	0.01489	1.15E-21	chr:19:22414379
19	rs456246	22327192	G	0.06004	1.21E-21	chr:19:22414379
19	rs281190	22399788	A	0.04143	2.64E-21	chr:19:22414379
19	rs281189	22399448	G	0.0415	2.90E-21	chr:19:22414379
19	rs281185	22398525	A	0.04177	3.93E-21	chr:19:22414379
19	rs281201	22406201	T	0.03725	1.17E-20	chr:19:22414379
19	rs461661	22327559	C	0.003494	1.50E-20	chr:19:22414379
19	rs1852417	22324637	G	0.03973	1.52E-20	chr:19:22414379
19	rs605085	22355832	T	0.08661	2.11E-20	chr:19:22414379
19	rs1852418	22324418	C	0.1307	3.18E-20	chr:19:22414379
19	rs2214352	22350938	A	0.02706	3.36E-20	chr:19:22414379

19	rs10421426	22366423	C	0.02322	3.95E-20	chr:19:22414379
19	rs2360001	22379587	G	0.02322	3.95E-20	chr:19:22414379
19	rs281184	22397099	T	0.02322	3.95E-20	chr:19:22414379
19	rs4932963	22388931	C	0.02322	3.95E-20	chr:19:22414379
19	rs2606273	22413654	G	0.002202	5.05E-20	chr:19:22414379
19	rs2606275	22413622	G	0.002202	5.05E-20	chr:19:22414379
19	rs281204	22407056	G	0.002202	5.05E-20	chr:19:22414379
19	rs281205	22407824	T	0.002202	5.05E-20	chr:19:22414379
19	rs4932966	22412232	A	0.002202	5.05E-20	chr:19:22414379
19	rs281202	22406331	A	0.00224	7.76E-20	chr:19:22414379
19	rs281161	22418243	G	0.04166	8.79E-20	chr:19:22414379
19	rs281197	22404604	A	0.00219	8.84E-20	chr:19:22414379
19	rs10403375	22389248	G	0.002187	8.87E-20	chr:19:22414379
19	rs10411854	22377604	C	0.002187	8.87E-20	chr:19:22414379
19	rs10412921	22386545	C	0.002187	8.87E-20	chr:19:22414379
19	rs10419666	22364492	A	0.002187	8.87E-20	chr:19:22414379
19	rs10419926	22364620	A	0.002187	8.87E-20	chr:19:22414379
19	rs12459032	22365552	G	0.002187	8.87E-20	chr:19:22414379
19	rs1852426	22385097	C	0.002187	8.87E-20	chr:19:22414379
19	rs1852428	22380017	G	0.002187	8.87E-20	chr:19:22414379
19	rs1918162	22373949	T	0.002187	8.87E-20	chr:19:22414379
19	rs1988038	22356993	T	0.002187	8.87E-20	chr:19:22414379
19	rs281176	22394319	C	0.002187	8.87E-20	chr:19:22414379
19	rs281178	22395581	G	0.002187	8.87E-20	chr:19:22414379
19	rs281179	22396158	C	0.002187	8.87E-20	chr:19:22414379
19	rs281180	22396190	A	0.002187	8.87E-20	chr:19:22414379
19	rs281183	22397080	C	0.002187	8.87E-20	chr:19:22414379
19	rs28760999	22365862	G	0.002187	8.87E-20	chr:19:22414379
19	rs2884867	22380662	G	0.002187	8.87E-20	chr:19:22414379
19	rs412658	22359440	C	0.002187	8.87E-20	chr:19:22414379
19	rs414983	22357747	T	0.002187	8.87E-20	chr:19:22414379
19	rs427945	22356325	T	0.002187	8.87E-20	chr:19:22414379
19	rs428455	22357247	G	0.002187	8.87E-20	chr:19:22414379
19	rs4584959	22368977	T	0.002187	8.87E-20	chr:19:22414379
19	rs8104929	22375868	C	0.002187	8.87E-20	chr:19:22414379
19	rs281186	22398960	G	0.002198	9.22E-20	chr:19:22414379
19	rs281187	22399362	G	0.002198	9.22E-20	chr:19:22414379
19	rs281188	22399389	T	0.002198	9.22E-20	chr:19:22414379
19	rs281191	22400166	T	0.002198	9.22E-20	chr:19:22414379
19	rs2021560	22373470	C	7.969	9.65E-20	chr:19:22414379
19	rs440539	22359330	G	0.002376	1.15E-19	chr:19:22414379
19	rs4371283	22373810	C	0.002342	1.29E-19	chr:19:22414379
19	rs11668081	22425615	A	0.1354	2.54E-19	chr:19:22414379

19	rs4932965	22412224	T	0.01987	2.62E-19	chr:19:22414379
19	rs17470104	22424418	A	0.1265	2.87E-19	chr:19:22414379
19	rs10418058	22391275	C	0.002572	1.47E-18	chr:19:22414379
19	rs2522319	22356056	T	0.002531	1.48E-18	chr:19:22414379
19	rs111581976	22370897	C	0.002554	1.65E-18	chr:19:22414379
19	rs1526883	22366973	C	0.002554	1.65E-18	chr:19:22414379
19	rs1830172	22388211	A	0.002554	1.65E-18	chr:19:22414379
19	rs1852427	22375002	A	0.002554	1.65E-18	chr:19:22414379
19	rs2522321	22358014	T	0.002554	1.65E-18	chr:19:22414379
19	rs2522322	22358015	T	0.002554	1.65E-18	chr:19:22414379
19	rs281182	22396907	C	0.002554	1.65E-18	chr:19:22414379
19	rs28790613	22378639	C	0.002554	1.65E-18	chr:19:22414379
19	rs3855858	22385901	T	0.002554	1.65E-18	chr:19:22414379
19	rs409627	22357936	G	0.002554	1.65E-18	chr:19:22414379
19	rs427500	22356757	G	0.002554	1.65E-18	chr:19:22414379
19	rs446754	22361761	A	0.002554	1.65E-18	chr:19:22414379
19	rs453104	22354405	T	0.002554	1.65E-18	chr:19:22414379
19	rs682866	22360309	C	0.002554	1.65E-18	chr:19:22414379
19	rs7343076	22367903	G	0.002554	1.65E-18	chr:19:22414379
19	rs7343146	22369930	G	0.002554	1.65E-18	chr:19:22414379
19	rs8105545	22376246	G	0.002554	1.65E-18	chr:19:22414379
19	rs8106346	22376315	T	0.002554	1.65E-18	chr:19:22414379
19	rs281200	22405888	A	0.002593	2.04E-18	chr:19:22414379
19	rs56144642	22421516	C	0.1289	4.43E-18	chr:19:22414379
19	rs10417143	22373303	T	0.009177	9.25E-18	chr:19:22414379
19	rs281174	22392420	G	0.009177	9.25E-18	chr:19:22414379
19	rs383678	22361741	T	0.009177	9.25E-18	chr:19:22414379
19	rs436744	22355575	T	0.009177	9.25E-18	chr:19:22414379
19	rs4614861	22373841	T	0.009177	9.25E-18	chr:19:22414379
19	rs1922685	22287970	T	0.1684	1.38E-17	chr:19:22414379
19	rs11671130	22329455	C	0.1341	9.82E-17	chr:19:22414379
19	rs11671580	22326439	A	0.1341	9.82E-17	chr:19:22414379
19	rs149070103	22340413	G	0.1341	9.82E-17	chr:19:22414379
19	rs1830174	22344502	A	0.1341	9.82E-17	chr:19:22414379
19	rs4381717	22338981	A	0.1341	9.82E-17	chr:19:22414379
19	rs4602157	22339036	C	0.1341	9.82E-17	chr:19:22414379
19	rs4616412	22339005	G	0.1341	9.82E-17	chr:19:22414379
19	rs4932750	22333226	T	0.1341	9.82E-17	chr:19:22414379
19	rs4123726	22419677	T	0.1353	9.88E-17	chr:19:22414379
19	rs281172	22424697	T	0.1716	1.09E-16	chr:19:22414379
19	rs12609473	22378606	G	0.1614	1.24E-16	chr:19:22414379
19	rs1830175	22382080	A	0.1614	1.24E-16	chr:19:22414379
19	rs3951502	22368852	A	0.1614	1.24E-16	chr:19:22414379

19	rs431628	22359024	T	0.1614	1.24E-16	chr:19:22414379
19	rs9304995	22366432	A	0.1614	1.24E-16	chr:19:22414379
19	rs11672659	22394767	T	0.1368	1.58E-16	chr:19:22414379
19	rs12609320	22390462	C	0.1368	1.58E-16	chr:19:22414379
19	rs1609037	22380261	C	0.1368	1.58E-16	chr:19:22414379
19	rs4390714	22369043	A	0.1368	1.58E-16	chr:19:22414379
19	rs4578792	22368982	G	0.1368	1.58E-16	chr:19:22414379
19	rs4932962	22372000	T	0.1368	1.58E-16	chr:19:22414379
19	rs1852420	22361902	T	0.1388	2.25E-16	chr:19:22414379
19	rs2522317	22339626	G	0.177	2.34E-16	chr:19:22414379
19	rs404793	22329632	A	0.177	2.34E-16	chr:19:22414379
19	rs424912	22347371	C	0.177	2.34E-16	chr:19:22414379
19	rs435638	22333121	A	0.177	2.34E-16	chr:19:22414379
19	rs448861	22353606	A	0.177	2.34E-16	chr:19:22414379
19	rs458515	22330126	T	0.177	2.34E-16	chr:19:22414379
19	rs2548936	22410178	C	0.001518	6.48E-16	chr:19:22414379
19	rs281192	22400404	T	446	1.54E-15	chr:19:22414379
19	rs2168491	22425589	G	0.2017	2.34E-15	chr:19:22414379
19	rs8111771	22287861	A	0.2075	5.30E-15	chr:19:22414379
19	rs10412312	22427134	C	5.597	8.16E-15	chr:19:22414379
19	rs172183	22405532	A	0.1965	8.27E-15	chr:19:22414379
19	rs281196	22403293	G	0.1965	8.27E-15	chr:19:22414379
19	rs281198	22405169	G	0.1965	8.27E-15	chr:19:22414379
19	rs281203	22406377	C	0.1965	8.27E-15	chr:19:22414379
19	rs464038	22335569	G	0.2141	9.41E-15	chr:19:22414379
19	rs62119970	22417828	G	6.305	1.42E-14	chr:19:22414379
19	rs61616269	22397866	C	0.2234	1.58E-14	chr:19:22414379
19	rs76490109	22330225	A	0.1689	2.39E-14	chr:19:22414379
19	rs150310626	22336147	T	0.162	7.93E-14	chr:19:22414379
19	rs281169	22423276	T	0.2184	9.03E-14	chr:19:22414379
19	rs10407936	22312059	A	0.2017	1.56E-13	chr:19:22414379
19	rs10415817	22313214	C	0.2017	1.56E-13	chr:19:22414379
19	rs281164	22419523	T	0.2114	2.00E-13	chr:19:22414379
19	rs366849	22234941	G	0.2051	2.08E-13	chr:19:22414379
19	rs7248898	22245354	G	0.2377	2.36E-13	chr:19:22414379
19	rs1978718	22293126	T	0.2095	2.69E-13	chr:19:22414379
19	rs2116895	22266669	T	0.2095	2.69E-13	chr:19:22414379
19	rs10403779	22258396	C	0.2108	3.34E-13	chr:19:22414379
19	rs2043318	22241615	G	0.2108	3.34E-13	chr:19:22414379
19	rs28881618	22254692	G	0.2108	3.34E-13	chr:19:22414379
19	rs3752163	22256396	G	0.2108	3.34E-13	chr:19:22414379
19	rs4932953	22249045	G	0.2108	3.34E-13	chr:19:22414379
19	rs6511318	22245745	A	0.2108	3.34E-13	chr:19:22414379

19	rs7251108	22250689	G	0.2108	3.34E-13	chr:19:22414379
19	rs7255666	22257264	T	0.2108	3.34E-13	chr:19:22414379
19	rs7258080	22257625	A	0.2108	3.34E-13	chr:19:22414379
19	rs8107039	22238990	G	0.2108	3.34E-13	chr:19:22414379
19	rs403850	22229593	T	0.2117	3.67E-13	chr:19:22414379
19	rs417331	22234478	T	0.2117	3.67E-13	chr:19:22414379
19	rs632709	22233096	C	0.2117	3.67E-13	chr:19:22414379
19	rs4244913	22239919	A	0.2129	4.41E-13	chr:19:22414379
19	rs2099347	22282614	T	0.2124	4.89E-13	chr:19:22414379
19	rs10411951	22309055	C	0.2321	5.20E-13	chr:19:22414379
19	rs6511334	22307267	C	0.2321	5.20E-13	chr:19:22414379
19	rs7258192	22264658	G	0.2155	5.83E-13	chr:19:22414379
19	rs7245790	22320009	C	0.2137	6.71E-13	chr:19:22414379
19	rs1036235	22279541	T	0.2168	6.97E-13	chr:19:22414379
19	rs8100438	22301003	C	0.238	7.61E-13	chr:19:22414379
19	rs987711	22300007	T	0.238	7.61E-13	chr:19:22414379
19	rs7254363	22257048	C	0.2186	9.24E-13	chr:19:22414379
19	rs6511332	22288391	A	0.2209	9.67E-13	chr:19:22414379
19	rs8103471	22317314	T	0.2173	1.11E-12	chr:19:22414379
19	rs10404620	22316577	A	0.2484	3.00E-12	chr:19:22414379
19	rs4244914	22315389	A	0.2484	3.00E-12	chr:19:22414379
19	rs8112637	22435375	T	0.2329	3.02E-12	chr:19:22414379
19	rs11085518	22434898	C	0.2358	3.99E-12	chr:19:22414379
19	rs11085520	22434988	G	0.2358	3.99E-12	chr:19:22414379
19	rs11085521	22435049	T	0.2358	3.99E-12	chr:19:22414379
19	rs8109519	22435128	T	0.2358	3.99E-12	chr:19:22414379
19	rs8109766	22435340	T	0.2358	3.99E-12	chr:19:22414379
19	rs8110568	22435291	C	0.2358	3.99E-12	chr:19:22414379
19	rs281166	22420798	G	0.2373	4.25E-12	chr:19:22414379
19	rs281168	22422638	A	0.2373	4.25E-12	chr:19:22414379
19	rs4123729	22418142	C	0.1436	6.62E-12	chr:19:22414379
19	rs55758909	22420558	A	0.1436	6.62E-12	chr:19:22414379
19	rs4372816	22328376	G	0.1405	6.99E-12	chr:19:22414379
19	rs74416263	22342767	T	0.1405	6.99E-12	chr:19:22414379
19	rs281155	22414594	G	0.0006619	7.33E-12	chr:19:22414379
19	rs4932747	22279252	C	0.2821	8.51E-12	chr:19:22414379
19	rs1378915	22362527	C	0.0006537	8.67E-12	chr:19:22414379
19	rs381995	22361666	T	0.0006537	8.67E-12	chr:19:22414379
19	rs11666326	22377109	A	0.1451	1.02E-11	chr:19:22414379
19	rs11667657	22393601	C	0.1454	1.04E-11	chr:19:22414379
19	rs1840718	22427586	T	3.499	1.15E-11	chr:19:22414379
19	rs10426106	22323268	T	0.2313	1.40E-11	chr:19:22414379
19	rs1865067	22323069	T	0.2313	1.40E-11	chr:19:22414379

19	rs111684505	22401097	G	0.1516	1.53E-11	chr:19:22414379
19	rs11672740	22394859	A	0.1499	1.65E-11	chr:19:22414379
19	rs452707	22304145	T	0.2452	2.12E-11	chr:19:22414379
19	rs57051328	22435951	A	0.2557	2.20E-11	chr:19:22414379
19	rs57684949	22435886	C	0.2557	2.20E-11	chr:19:22414379
19	rs61387248	22435806	A	0.2557	2.20E-11	chr:19:22414379
19	rs617609	22326000	A	0.2318	2.26E-11	chr:19:22414379
19	rs28456336	22268779	T	0.2692	3.00E-11	chr:19:22414379
19	rs60200804	22283368	T	0.2695	4.36E-11	chr:19:22414379
19	rs1019938	22280954	T	0.2716	5.44E-11	chr:19:22414379
19	rs2359995	22280060	C	0.2716	5.44E-11	chr:19:22414379
19	rs4553749	22285063	C	0.2716	5.44E-11	chr:19:22414379
19	rs4932748	22279318	T	0.2716	5.44E-11	chr:19:22414379
19	rs4932954	22272454	T	0.2716	5.44E-11	chr:19:22414379
19	rs6511320	22267122	C	0.2716	5.44E-11	chr:19:22414379
19	rs6511323	22281914	T	0.2716	5.44E-11	chr:19:22414379
19	rs6511325	22284357	A	0.2716	5.44E-11	chr:19:22414379
19	rs6511326	22286679	T	0.2716	5.44E-11	chr:19:22414379
19	rs6511329	22287905	T	0.2716	5.44E-11	chr:19:22414379
19	rs9304994	22272743	G	0.2716	5.44E-11	chr:19:22414379
19	rs8109649	22435344	G	0.2891	5.96E-11	chr:19:22414379
19	rs1560718	22292212	C	0.275	7.86E-11	chr:19:22414379
19	rs3813590	22321273	T	0.1616	7.90E-11	chr:19:22414379
19	rs3813592	22321245	G	0.1616	7.90E-11	chr:19:22414379
19	rs12460025	22318230	G	10.65	1.19E-10	chr:19:22414379
19	rs281157	22415509	A	0.001215	1.32E-10	chr:19:22414379
19	rs281159	22416987	G	0.001217	1.35E-10	chr:19:22414379
19	rs281163	22419302	A	0.3091	1.69E-10	chr:19:22414379
19	rs1830173	22388037	T	10.2	1.87E-10	chr:19:22414379
19	rs281177	22394727	T	10.2	1.87E-10	chr:19:22414379
19	rs451793	22361286	G	10.2	1.87E-10	chr:19:22414379
19	rs62112446	22368828	A	10.2	1.87E-10	chr:19:22414379
19	rs7343144	22369824	T	10.2	1.87E-10	chr:19:22414379
19	rs387604	22332265	A	9.116	1.92E-10	chr:19:22414379
19	rs7253425	22381476	G	10.18	1.93E-10	chr:19:22414379
19	rs420218	22353589	T	10.18	1.93E-10	chr:19:22414379
19	rs281162	22418472	A	7.922	3.20E-10	chr:19:22414379
19	rs3813591	22321256	C	13.37	3.38E-10	chr:19:22414379
19	rs111352604	22314567	G	0.1733	4.13E-10	chr:19:22414379
19	rs281160	22418046	T	6.782	4.17E-10	chr:19:22414379
19	rs17554725	22317759	G	13.06	4.37E-10	chr:19:22414379
19	rs7257051	22319292	G	13.06	4.37E-10	chr:19:22414379
19	rs8113700	22319421	A	13.06	4.37E-10	chr:19:22414379

19	rs4932951	22246352	C	6.202	4.76E-10	chr:19:22414379
19	rs12610367	22320848	T	0.1794	5.03E-10	chr:19:22414379
19	rs12460610	22291277	C	6.156	5.06E-10	chr:19:22414379
19	rs73019874	22267259	G	6.156	5.06E-10	chr:19:22414379
19	rs10422039	22422539	A	3.271	5.46E-10	chr:19:22414379
19	rs368652	22356811	A	0.2535	9.60E-10	chr:19:22414379
19	rs437284	22354875	T	0.2535	9.60E-10	chr:19:22414379
19	rs4436884	22285163	C	5.356	1.06E-09	chr:19:22414379
19	rs4932955	22272845	A	5.356	1.06E-09	chr:19:22414379
19	rs4932956	22273797	G	5.356	1.06E-09	chr:19:22414379
19	rs12610034	22314528	A	0.1771	1.08E-09	chr:19:22414379
19	rs73021832	22316144	C	0.1771	1.08E-09	chr:19:22414379
19	rs190030543	22291079	T	5.579	1.22E-09	chr:19:22414379
19	rs73021803	22286898	T	5.339	1.29E-09	chr:19:22414379
19	rs281170	22423336	T	6.197	1.43E-09	chr:19:22414379
19	rs11673195	22313814	C	0.1825	1.64E-09	chr:19:22414379
19	rs111270824	22234403	T	0.2052	2.07E-09	chr:19:22414379
19	rs61268244	22310130	T	0.188	2.35E-09	chr:19:22414379
19	rs112688861	22227973	A	0.2093	2.93E-09	chr:19:22414379
19	rs807954	22117663	C	0.3618	3.78E-09	chr:19:22414379
19	rs397538	22329889	A	0.2631	4.19E-09	chr:19:22414379
19	rs2548932	22436172	G	3.133	4.36E-09	chr:19:22414379
19	rs28702014	22436174	G	3.133	4.36E-09	chr:19:22414379
19	rs1526884	22303651	G	0.1921	5.22E-09	chr:19:22414379
19	rs4932957	22304745	T	0.1921	5.22E-09	chr:19:22414379
19	rs11669885	22397422	A	0.2714	6.42E-09	chr:19:22414379
19	rs56320331	22297657	T	4.263	6.43E-09	chr:19:22414379
19	rs57346017	22427336	G	2.917	6.63E-09	chr:19:22414379
19	rs11671416	22427254	T	0.3932	9.57E-09	chr:19:22414379
19	rs10420558	22422430	T	2.884	1.27E-08	chr:19:22414379
19	rs12461719	22292841	T	3.875	1.48E-08	chr:19:22414379
19	rs56242139	22295279	A	3.875	1.48E-08	chr:19:22414379
19	rs55635227	22298116	C	3.88	1.54E-08	chr:19:22414379
19	rs283146	22438180	C	0.3276	1.60E-08	chr:19:22414379
19	rs377054171	22290374	C	5.192	1.84E-08	chr:19:22414379
19	rs11561612	22432845	A	0.1982	1.89E-08	chr:19:22414379
19	rs147620331	22077709	T	0.1911	1.91E-08	chr:19:22414379
19	rs12980808	22374080	G	0.2729	2.28E-08	chr:19:22414379
19	rs34364151	22393367	T	0.2729	2.28E-08	chr:19:22414379
19	rs78993648	22230665	A	0.1574	2.61E-08	chr:19:22414379
19	rs57489232	22078207	T	0.198	3.22E-08	chr:19:22414379
19	rs11085516	22431751	T	0.2089	3.35E-08	chr:19:22414379
19	rs55784860	22431132	C	0.2089	3.35E-08	chr:19:22414379

19	rs8106199	22421457	T	2.733	4.87E-08	chr:19:22414379
19	rs111575233	22430482	A	0.2158	5.05E-08	chr:19:22414379
19	rs74586061	22480025	A	0.3146	5.28E-08	chr:19:22414379
19	rs74459753	22458131	T	0.3293	5.56E-08	chr:19:22414379
19	rs11085517	22433283	T	0.2083	6.59E-08	chr:19:22414379
19	rs11085519	22434908	T	0.2083	6.59E-08	chr:19:22414379
19	rs55771377	22433422	G	0.2083	6.59E-08	chr:19:22414379
19	rs7253490	22293706	C	0.4112	6.90E-08	chr:19:22414379
19	rs12327815	22477891	C	0.3194	7.54E-08	chr:19:22414379
19	rs114200971	22459204	T	0.3212	8.16E-08	chr:19:22414379
19	rs74583609	22471607	T	0.3212	8.16E-08	chr:19:22414379
19	rs111451157	22480494	C	0.3214	8.85E-08	chr:19:22414379
19	rs150285219	22500252	G	0.3214	8.85E-08	chr:19:22414379
19	rs113226782	22468272	G	0.3355	9.04E-08	chr:19:22414379
19	rs7259785	22419378	A	6.565	9.96E-08	chr:19:22414379
19	rs568047338	22450475	A	0.2638	1.01E-07	chr:19:22414379
19	rs112194091	22421144	C	6.564	1.01E-07	chr:19:22414379
19	rs112285940	22462360	G	0.3241	1.05E-07	chr:19:22414379
19	rs114485288	22456655	A	0.3258	1.10E-07	chr:19:22414379
19	rs34312380	22390071	T	0.2898	1.27E-07	chr:19:22414379
19	rs34407605	22390084	T	0.2898	1.27E-07	chr:19:22414379
19	rs1961316	22426154	A	3.45	1.28E-07	chr:19:22414379
19	rs114487296	22450493	C	0.2671	1.30E-07	chr:19:22414379
19	rs115211114	22450434	G	0.2671	1.30E-07	chr:19:22414379
19	rs115402237	22446268	G	0.2671	1.30E-07	chr:19:22414379
19	rs116095172	22447297	A	0.2671	1.30E-07	chr:19:22414379
19	rs116116710	22450695	G	0.2671	1.30E-07	chr:19:22414379
19	rs116290379	22446479	A	0.2671	1.30E-07	chr:19:22414379
19	rs116505167	22450685	C	0.2671	1.30E-07	chr:19:22414379
19	rs116721114	22450482	T	0.2671	1.30E-07	chr:19:22414379
19	rs138350421	22447920	C	0.2671	1.30E-07	chr:19:22414379
19	rs139771475	22448256	T	0.2671	1.30E-07	chr:19:22414379
19	rs141860818	22448149	G	0.2671	1.30E-07	chr:19:22414379
19	rs183369013	22446254	A	0.2671	1.30E-07	chr:19:22414379
19	rs542721643	22447006	T	0.2671	1.30E-07	chr:19:22414379
19	rs560680679	22447007	G	0.2671	1.30E-07	chr:19:22414379
19	rs75134450	22447605	T	0.2671	1.30E-07	chr:19:22414379
19	rs76451239	22446557	T	0.2671	1.30E-07	chr:19:22414379
19	rs76963327	22446051	T	0.2671	1.30E-07	chr:19:22414379
19	rs78665120	22446079	T	0.2671	1.30E-07	chr:19:22414379
19	rs79135252	22446546	A	0.2671	1.30E-07	chr:19:22414379
19	rs80081358	22450977	G	0.2671	1.30E-07	chr:19:22414379
19	rs111639362	22463115	C	0.328	1.36E-07	chr:19:22414379

19	rs10416663	22425363	A	9.463	1.36E-07	chr:19:22414379
19	rs114210862	22460366	A	0.3295	1.41E-07	chr:19:22414379
19	rs112569701	22473830	G	0.3323	1.50E-07	chr:19:22414379
19	rs116524376	22352448	A	0.2115	1.71E-07	chr:19:22414379
19	rs79490176	22351851	T	0.2115	1.71E-07	chr:19:22414379
19	rs139826378	22452654	G	0.2718	1.81E-07	chr:19:22414379
19	rs116565754	22456104	T	0.3327	1.82E-07	chr:19:22414379
19	rs112567736	22424051	T	9.218	1.86E-07	chr:19:22414379
19	rs73019902	22286707	A	9.197	1.93E-07	chr:19:22414379
19	rs73540924	22212627	G	0.2327	1.99E-07	chr:19:22414379
19	rs12461499	22304581	A	8.086	2.13E-07	chr:19:22414379
19	rs59020942	22427750	T	0.2375	2.20E-07	chr:19:22414379
19	rs59574273	22427752	T	0.2375	2.20E-07	chr:19:22414379
19	rs111724992	22282718	C	9.097	2.22E-07	chr:19:22414379
19	rs12459299	22296261	G	9.097	2.22E-07	chr:19:22414379
19	rs12460028	22284598	C	9.097	2.22E-07	chr:19:22414379
19	rs12460058	22284531	A	9.097	2.22E-07	chr:19:22414379
19	rs12461745	22262667	T	9.097	2.22E-07	chr:19:22414379
19	rs56138218	22279031	A	9.097	2.22E-07	chr:19:22414379
19	rs56183023	22269366	C	9.097	2.22E-07	chr:19:22414379
19	rs6511324	22283994	T	9.097	2.22E-07	chr:19:22414379
19	rs7260631	22282906	G	9.097	2.22E-07	chr:19:22414379
19	rs73019896	22286350	A	9.097	2.22E-07	chr:19:22414379
19	rs115943909	22452135	C	0.2747	2.26E-07	chr:19:22414379
19	rs115059058	22459742	G	0.3365	2.32E-07	chr:19:22414379
19	rs12461491	22304761	G	9.3	2.44E-07	chr:19:22414379
19	rs73019812	22232291	T	9.041	2.48E-07	chr:19:22414379
19	rs78455852	22446210	T	0.1926	2.52E-07	chr:19:22414379
19	rs34878133	22230331	A	9.014	2.55E-07	chr:19:22414379
19	rs12459277	22235908	T	9.015	2.55E-07	chr:19:22414379
19	rs3752168	22235478	C	9.015	2.55E-07	chr:19:22414379
19	rs73019829	22239600	A	9.015	2.55E-07	chr:19:22414379
19	rs73021825	22313577	A	11.23	2.61E-07	chr:19:22414379
19	rs56036277	22257574	A	8.987	2.62E-07	chr:19:22414379
19	rs73019862	22260969	T	8.987	2.62E-07	chr:19:22414379
19	rs8108310	22287184	T	8.926	2.80E-07	chr:19:22414379
19	rs76995772	22311909	A	11.16	2.81E-07	chr:19:22414379
19	rs73021823	22310164	A	11.11	2.92E-07	chr:19:22414379
19	rs114289956	22454001	T	0.2786	2.93E-07	chr:19:22414379
19	rs114422668	22453355	T	0.2786	2.93E-07	chr:19:22414379
19	rs114551844	22452452	T	0.2786	2.93E-07	chr:19:22414379
19	rs114612477	22451904	T	0.2786	2.93E-07	chr:19:22414379
19	rs115083572	22452285	G	0.2786	2.93E-07	chr:19:22414379

19	rs116119620	22453015	T	0.2786	2.93E-07	chr:19:22414379
19	rs116279903	22452173	C	0.2786	2.93E-07	chr:19:22414379
19	rs139000614	22451845	T	0.2786	2.93E-07	chr:19:22414379
19	rs139097230	22452489	T	0.2786	2.93E-07	chr:19:22414379
19	rs141440964	22451251	T	0.2786	2.93E-07	chr:19:22414379
19	rs60604817	22427565	G	0.2437	3.15E-07	chr:19:22414379
19	rs56359043	22295283	G	8.839	3.22E-07	chr:19:22414379
19	rs114471536	22444439	G	0.2098	3.46E-07	chr:19:22414379
19	rs73019871	22265915	T	8.744	3.65E-07	chr:19:22414379
19	rs142536481	22451222	A	0.2824	3.66E-07	chr:19:22414379
19	rs141785726	22432469	T	0.2765	3.89E-07	chr:19:22414379
19	rs193035916	22453827	T	0.2842	4.11E-07	chr:19:22414379
19	rs73019831	22242013	T	8.659	4.23E-07	chr:19:22414379
19	rs148603054	22433487	T	0.2803	4.98E-07	chr:19:22414379
19	rs73550926	22378553	T	10.48	5.50E-07	chr:19:22414379
19	rs58752579	22109593	A	0.4416	5.66E-07	chr:19:22414379
19	rs9676804	22427959	C	0.2816	5.76E-07	chr:19:22414379
19	rs9676823	22427987	C	0.2816	5.76E-07	chr:19:22414379
19	rs9676835	22428179	A	0.2816	5.76E-07	chr:19:22414379
19	rs7253840	22160161	A	0.2448	5.92E-07	chr:19:22414379
19	rs6511312	22205349	T	2.464	6.63E-07	chr:19:22414379
19	rs115059290	22451632	A	0.2887	6.79E-07	chr:19:22414379
19	rs143243514	22451798	G	0.289	6.94E-07	chr:19:22414379
19	rs149576034	22451779	A	0.289	6.94E-07	chr:19:22414379
19	rs137981753	22434357	T	0.2869	6.99E-07	chr:19:22414379
19	rs11085515	22426520	C	0.4451	7.53E-07	chr:19:22414379
19	rs116577376	22453691	A	0.2897	7.54E-07	chr:19:22414379
19	rs77463219	22447877	C	0.2795	7.86E-07	chr:19:22414379
19	rs114837217	22446650	C	0.2798	7.89E-07	chr:19:22414379
19	rs114073147	22451639	T	0.2924	8.54E-07	chr:19:22414379
19	rs138243368	22451652	G	0.2924	8.54E-07	chr:19:22414379
19	rs149312129	22453905	G	0.2409	8.71E-07	chr:19:22414379
19	rs139037369	22423799	T	0.2068	9.95E-07	chr:19:22414379
19	rs73933927	22422487	C	6.266	1.02E-06	chr:19:22414379
19	rs75418407	22421000	G	6.266	1.02E-06	chr:19:22414379
19	rs75794199	22420031	A	6.222	1.12E-06	chr:19:22414379
19	rs10410168	22302211	A	3.22	1.13E-06	chr:19:22414379
19	rs113140998	22468903	C	0.3543	1.21E-06	chr:19:22414379
19	rs10419058	22309972	A	0.4495	1.23E-06	chr:19:22414379
19	rs10422317	22306604	A	3.193	1.35E-06	chr:19:22414379
19	rs149056579	22425321	C	0.2151	1.45E-06	chr:19:22414379
19	rs73548422	22306063	T	3.173	1.57E-06	chr:19:22414379
19	rs113181884	22586084	C	0.3836	1.61E-06	chr:19:22414379

19	rs56768795	22586073	A	0.3836	1.61E-06	chr:19:22414379
19	rs10518283	22306086	G	3.177	1.65E-06	chr:19:22414379
19	rs114397370	22452941	C	0.2998	1.69E-06	chr:19:22414379
19	rs192010013	22310622	T	0.1952	1.72E-06	chr:19:22414379
19	rs112366241	22423026	G	5.396	1.86E-06	chr:19:22414379
19	rs150083716	22423796	C	5.396	1.86E-06	chr:19:22414379
19	rs73933930	22423321	T	5.396	1.86E-06	chr:19:22414379
19	rs75979312	22423590	C	5.396	1.86E-06	chr:19:22414379
19	rs28803922	22619836	G	0.4493	1.88E-06	chr:19:22414379
19	rs143591281	22494122	C	0.2472	1.88E-06	chr:19:22414379
19	rs11673212	22536050	C	0.4391	1.97E-06	chr:19:22414379
19	rs6511344	22535673	C	0.4391	1.97E-06	chr:19:22414379
19	rs555185638	22290846	C	3.668	1.98E-06	chr:19:22414379
19	rs12608536	22542058	A	0.4419	2.04E-06	chr:19:22414379
19	rs149005977	22452903	G	0.3036	2.11E-06	chr:19:22414379
19	rs2188969	22202783	A	0.1891	2.17E-06	chr:19:22414379
19	rs146011557	22327592	G	0.2758	2.23E-06	chr:19:22414379
19	rs543737598	22327590	T	0.2758	2.23E-06	chr:19:22414379
19	rs576238286	22327589	G	0.2758	2.23E-06	chr:19:22414379
19	rs115893105	22452937	A	0.3053	2.25E-06	chr:19:22414379
19	rs201113571	22290840	T	4.364	2.31E-06	chr:19:22414379
19	rs283150	22435172	G	2.839	2.35E-06	chr:19:22414379
19	rs147438265	22139021	T	0.2741	2.45E-06	chr:19:22414379
19	rs10422880	22314083	T	3.152	2.48E-06	chr:19:22414379
19	rs28552094	22312865	T	3.152	2.48E-06	chr:19:22414379
19	rs114469495	22343743	A	0.2043	2.72E-06	chr:19:22414379
19	rs146480504	22325896	T	0.2043	2.72E-06	chr:19:22414379
19	rs138748164	22432402	T	0.2558	2.81E-06	chr:19:22414379
19	rs112141066	22095496	T	0.2426	2.88E-06	chr:19:22414379
19	rs283149	22436268	G	2.496	3.13E-06	chr:19:22414379
19	rs140062581	22396642	G	0.2064	3.15E-06	chr:19:22414379
19	rs140589863	22368673	C	0.2064	3.15E-06	chr:19:22414379
19	rs142135317	22364627	C	0.2064	3.15E-06	chr:19:22414379
19	rs151087034	22383146	T	0.2064	3.15E-06	chr:19:22414379
19	rs145383649	22237089	T	0.218	3.20E-06	chr:19:22414379
19	rs115052425	22428855	T	0.2587	3.33E-06	chr:19:22414379
19	rs1654508	22219276	A	0.1832	3.37E-06	chr:19:22414379
19	rs11085524	22536422	G	0.4502	3.81E-06	chr:19:22414379
19	rs11879791	22536939	A	0.4502	3.81E-06	chr:19:22414379
19	rs12459636	22542439	A	0.4502	3.81E-06	chr:19:22414379
19	rs59598661	22537029	T	0.4502	3.81E-06	chr:19:22414379
19	rs113984339	22455231	A	0.3422	4.33E-06	chr:19:22414379
19	rs114951439	22226661	C	0.2039	4.54E-06	chr:19:22414379

19	rs115104032	22232501	G	0.2039	4.54E-06	chr:19:22414379
19	rs116314323	22263188	G	0.2039	4.54E-06	chr:19:22414379
19	rs116684615	22258574	G	0.2039	4.54E-06	chr:19:22414379
19	rs116689744	22249435	T	0.2039	4.54E-06	chr:19:22414379
19	rs138062894	22240079	G	0.2039	4.54E-06	chr:19:22414379
19	rs138262894	22304541	T	0.2039	4.54E-06	chr:19:22414379
19	rs139498146	22259577	T	0.2039	4.54E-06	chr:19:22414379
19	rs139706424	22245204	A	0.2039	4.54E-06	chr:19:22414379
19	rs141718625	22301482	G	0.2039	4.54E-06	chr:19:22414379
19	rs144107018	22228069	T	0.2039	4.54E-06	chr:19:22414379
19	rs146174182	22246554	T	0.2039	4.54E-06	chr:19:22414379
19	rs146898264	22228968	T	0.2039	4.54E-06	chr:19:22414379
19	rs147115194	22261333	T	0.2039	4.54E-06	chr:19:22414379
19	rs147734923	22261485	G	0.2039	4.54E-06	chr:19:22414379
19	rs149531591	22305037	A	0.2039	4.54E-06	chr:19:22414379
19	rs182075189	22287899	T	0.2039	4.54E-06	chr:19:22414379
19	rs185089748	22229367	A	0.2039	4.54E-06	chr:19:22414379
19	rs189880041	22250567	T	0.2039	4.54E-06	chr:19:22414379
19	rs562407245	22287558	G	0.2039	4.54E-06	chr:19:22414379
19	rs61743214	22235321	A	0.2039	4.54E-06	chr:19:22414379
19	rs75477882	22307782	A	0.2039	4.54E-06	chr:19:22414379
19	rs76624361	22265310	A	0.2039	4.54E-06	chr:19:22414379
19	rs115822146	22351637	C	0.2157	4.90E-06	chr:19:22414379
19	rs138593069	22359173	G	0.2157	4.90E-06	chr:19:22414379
19	rs145669542	22349883	G	0.2157	4.90E-06	chr:19:22414379
19	rs151297934	22351897	G	0.2157	4.90E-06	chr:19:22414379
19	rs184806947	22349913	A	0.2157	4.90E-06	chr:19:22414379
19	rs189229342	22349917	A	0.2157	4.90E-06	chr:19:22414379
19	rs78880255	22377092	C	0.2157	4.90E-06	chr:19:22414379
19	rs150941289	22294382	C	0.2054	5.03E-06	chr:19:22414379
19	rs114710457	22439048	T	0.2356	5.05E-06	chr:19:22414379
19	rs10420483	22190378	A	0.1944	5.38E-06	chr:19:22414379
19	rs11085509	22204573	T	0.1944	5.38E-06	chr:19:22414379
19	rs2017962	22208751	T	0.1944	5.38E-06	chr:19:22414379
19	rs2078089	22209753	A	0.1944	5.38E-06	chr:19:22414379
19	rs2106869	22184693	C	0.1944	5.38E-06	chr:19:22414379
19	rs2188970	22200382	G	0.1944	5.38E-06	chr:19:22414379
19	rs2359821	22208314	C	0.1944	5.38E-06	chr:19:22414379
19	rs374537	22222802	G	0.1944	5.38E-06	chr:19:22414379
19	rs404822	22215967	A	0.1944	5.38E-06	chr:19:22414379
19	rs410794	22226159	A	0.1944	5.38E-06	chr:19:22414379
19	rs4244912	22203237	T	0.1944	5.38E-06	chr:19:22414379
19	rs429184	22214352	A	0.1944	5.38E-06	chr:19:22414379

19	rs436039	22214873	T	0.1944	5.38E-06	chr:19:22414379
19	rs7249976	22183920	A	0.1944	5.38E-06	chr:19:22414379
19	rs149772697	22459904	T	0.2754	5.64E-06	chr:19:22414379
19	rs371889380	22289791	T	0.1605	5.68E-06	chr:19:22414379
19	rs73930533	22382933	A	6.07	5.83E-06	chr:19:22414379
19	rs10424854	22314278	C	2.951	5.84E-06	chr:19:22414379
19	rs11882755	22413432	C	6.066	5.86E-06	chr:19:22414379
19	rs114106545	22443451	C	0.194	5.95E-06	chr:19:22414379
19	rs114858177	22442210	T	0.194	5.95E-06	chr:19:22414379
19	rs114941840	22443558	G	0.194	5.95E-06	chr:19:22414379
19	rs115829048	22443428	G	0.194	5.95E-06	chr:19:22414379
19	rs116071570	22441750	T	0.194	5.95E-06	chr:19:22414379
19	rs116835709	22442196	A	0.194	5.95E-06	chr:19:22414379
19	rs138359687	22441889	T	0.194	5.95E-06	chr:19:22414379
19	rs142199394	22441613	A	0.194	5.95E-06	chr:19:22414379
19	rs142993500	22442940	G	0.194	5.95E-06	chr:19:22414379
19	rs150828150	22444017	T	0.194	5.95E-06	chr:19:22414379
19	rs151201308	22441615	G	0.194	5.95E-06	chr:19:22414379
19	rs74794076	22443741	C	0.194	5.95E-06	chr:19:22414379
19	rs76781024	22441776	C	0.194	5.95E-06	chr:19:22414379
19	rs78001040	22442909	C	0.194	5.95E-06	chr:19:22414379
19	rs62120010	22428480	A	2.726	6.25E-06	chr:19:22414379
19	rs13346237	22317838	C	2.982	6.33E-06	chr:19:22414379
19	rs79706549	22368499	C	6.021	6.39E-06	chr:19:22414379
19	rs7255829	22537311	C	0.4605	6.47E-06	chr:19:22414379
19	rs115560278	22117181	A	0.3108	6.80E-06	chr:19:22414379
19	rs139107619	22330909	T	0.1874	6.83E-06	chr:19:22414379
19	rs150671714	22671868	T	0.2372	6.83E-06	chr:19:22414379
19	rs80327586	22376285	T	0.2241	7.19E-06	chr:19:22414379
19	rs7248242	22575994	G	0.4627	7.86E-06	chr:19:22414379
19	rs10420646	22319061	T	2.888	8.22E-06	chr:19:22414379
19	rs10409819	22602919	T	0.4612	8.35E-06	chr:19:22414379
19	rs10406742	22323974	G	2.877	9.05E-06	chr:19:22414379
19	rs807952	22117758	A	0.4714	9.14E-06	chr:19:22414379
19	rs807953	22117746	G	0.4714	9.14E-06	chr:19:22414379
19	rs390730	22233509	C	0.2493	9.46E-06	chr:19:22414379
19	rs11669110	22296485	G	0.2495	9.49E-06	chr:19:22414379
19	rs12609368	22263996	C	0.2495	9.49E-06	chr:19:22414379
19	rs149975047	22287938	A	0.2495	9.49E-06	chr:19:22414379
19	rs17459246	22278121	C	0.2495	9.49E-06	chr:19:22414379
19	rs191067451	22288201	A	0.2495	9.49E-06	chr:19:22414379
19	rs1978719	22266101	A	0.2495	9.49E-06	chr:19:22414379
19	rs367972720	22291197	C	0.2495	9.49E-06	chr:19:22414379

19	rs372140746	22291198	C	0.2495	9.49E-06	chr:19:22414379
19	rs372252339	22291126	T	0.2495	9.49E-06	chr:19:22414379
19	rs55728714	22262899	G	0.2495	9.49E-06	chr:19:22414379
19	rs55886600	22276437	A	0.2495	9.49E-06	chr:19:22414379
19	rs56073035	22276298	G	0.2495	9.49E-06	chr:19:22414379
19	rs56207853	22292357	T	0.2495	9.49E-06	chr:19:22414379
19	rs56369285	22267185	C	0.2495	9.49E-06	chr:19:22414379
19	rs72481609	22296819	C	0.2495	9.49E-06	chr:19:22414379
19	rs73019889	22277826	G	0.2495	9.49E-06	chr:19:22414379
19	rs8103088	22298322	G	0.4747	9.70E-06	chr:19:22414379
19	rs183543741	22289845	T	0.2596	9.72E-06	chr:19:22414379
19	rs193152926	22289796	A	0.2596	9.72E-06	chr:19:22414379
19	rs375012052	22289851	A	0.2596	9.72E-06	chr:19:22414379
19	rs808246	22430516	A	2.709	9.98E-06	chr:19:22414379
19	rs543507823	22444547	G	0.3428	1.02E-05	chr:19:22414379
19	rs554187827	22398607	T	0.29	1.04E-05	chr:19:22414379
19	rs59448173	22251554	T	0.2609	1.04E-05	chr:19:22414379
19	rs10426859	22400733	C	7.74	1.05E-05	chr:19:22414379
19	rs7248538	22393348	G	7.74	1.05E-05	chr:19:22414379
19	rs77684496	22123382	C	0.2233	1.06E-05	chr:19:22414379
19	rs10418173	22418962	T	9.681	1.10E-05	chr:19:22414379
19	rs10419517	22418957	A	9.681	1.10E-05	chr:19:22414379
19	rs10417756	22418917	C	9.642	1.11E-05	chr:19:22414379
19	rs10417759	22418919	C	9.642	1.11E-05	chr:19:22414379
19	rs114009852	22441147	T	0.1833	1.15E-05	chr:19:22414379
19	rs78326546	22441140	T	0.1833	1.15E-05	chr:19:22414379
19	rs78401147	22441182	A	0.1833	1.15E-05	chr:19:22414379
19	rs147395021	22217756	A	87.79	2.31E-21	chr:19:22457244
19	rs115365441	22441932	A	76.01	3.65E-20	chr:19:22457244
19	rs116421315	22441374	G	76.01	3.65E-20	chr:19:22457244
19	rs114106545	22443451	C	54.61	6.25E-20	chr:19:22457244
19	rs114858177	22442210	T	54.61	6.25E-20	chr:19:22457244
19	rs114941840	22443558	G	54.61	6.25E-20	chr:19:22457244
19	rs115829048	22443428	G	54.61	6.25E-20	chr:19:22457244
19	rs116071570	22441750	T	54.61	6.25E-20	chr:19:22457244
19	rs116835709	22442196	A	54.61	6.25E-20	chr:19:22457244
19	rs138359687	22441889	T	54.61	6.25E-20	chr:19:22457244
19	rs142199394	22441613	A	54.61	6.25E-20	chr:19:22457244
19	rs142993500	22442940	G	54.61	6.25E-20	chr:19:22457244
19	rs150828150	22444017	T	54.61	6.25E-20	chr:19:22457244
19	rs151201308	22441615	G	54.61	6.25E-20	chr:19:22457244
19	rs74794076	22443741	C	54.61	6.25E-20	chr:19:22457244
19	rs76781024	22441776	C	54.61	6.25E-20	chr:19:22457244

19	rs78001040	22442909	C	54.61	6.25E-20	chr:19:22457244
19	rs142179493	22422064	A	63.25	9.48E-20	chr:19:22457244
19	rs114859869	22440062	T	44.89	1.74E-19	chr:19:22457244
19	rs116060696	22440074	G	44.89	1.74E-19	chr:19:22457244
19	rs116654604	22440069	C	44.89	1.74E-19	chr:19:22457244
19	rs79734070	22439863	G	44.89	1.74E-19	chr:19:22457244
19	rs115063713	22420253	C	54.08	3.10E-19	chr:19:22457244
19	rs141998357	22443528	A	48.26	3.62E-19	chr:19:22457244
19	rs116591487	22419605	C	56.94	4.86E-19	chr:19:22457244
19	rs75133779	22440947	C	67.28	6.46E-19	chr:19:22457244
19	rs114009852	22441147	T	89.48	8.73E-19	chr:19:22457244
19	rs78326546	22441140	T	89.48	8.73E-19	chr:19:22457244
19	rs78401147	22441182	A	89.48	8.73E-19	chr:19:22457244
19	rs184984485	22383032	T	82.21	1.75E-18	chr:19:22457244
19	rs114516156	22422372	C	175.1	8.05E-18	chr:19:22457244
19	rs115621962	22447031	G	175.1	8.05E-18	chr:19:22457244
19	rs115964434	22446473	A	175.1	8.05E-18	chr:19:22457244
19	rs139118076	22453443	C	175.1	8.05E-18	chr:19:22457244
19	rs148109247	22448147	G	175.1	8.05E-18	chr:19:22457244
19	rs150771286	22452177	G	175.1	8.05E-18	chr:19:22457244
19	rs151079335	22453101	A	175.1	8.05E-18	chr:19:22457244
19	rs375615347	22446472	C	175.1	8.05E-18	chr:19:22457244
19	rs74475732	22446215	T	175.1	8.05E-18	chr:19:22457244
19	rs75556934	22451107	A	175.1	8.05E-18	chr:19:22457244
19	rs115052365	22445846	G	144.3	8.60E-18	chr:19:22457244
19	rs115561260	22457093	A	142.8	9.22E-18	chr:19:22457244
19	rs114009036	22420460	C	145.1	1.22E-17	chr:19:22457244
19	rs145532709	22450480	C	145.1	1.22E-17	chr:19:22457244
19	rs114710457	22439048	T	24.55	1.59E-17	chr:19:22457244
19	rs116725110	22527192	T	74.49	1.81E-17	chr:19:22457244
19	rs116084977	22506409	T	68.73	7.13E-17	chr:19:22457244
19	rs146786232	22415272	A	245	7.68E-17	chr:19:22457244
19	rs78455852	22446210	T	26.9	7.79E-17	chr:19:22457244
19	rs115429983	22452748	T	246.3	8.09E-17	chr:19:22457244
19	rs181160079	22618570	A	39.54	8.45E-17	chr:19:22457244
19	rs141659586	22429781	C	23.32	8.84E-17	chr:19:22457244
19	rs187112112	22571835	C	56.86	1.11E-16	chr:19:22457244
19	rs111606427	22404384	T	64.16	1.30E-16	chr:19:22457244
19	rs115667831	22457297	A	41.21	1.43E-16	chr:19:22457244
19	rs111597600	22411958	C	57.54	1.56E-16	chr:19:22457244
19	rs111879339	22406839	C	57.54	1.56E-16	chr:19:22457244
19	rs112780443	22409102	C	57.54	1.56E-16	chr:19:22457244
19	rs113019152	22405484	G	57.54	1.56E-16	chr:19:22457244

19	rs113231070	22404630	C	57.54	1.56E-16	chr:19:22457244
19	rs113678644	22405415	A	57.54	1.56E-16	chr:19:22457244
19	rs531059495	22450755	G	83.2	2.14E-16	chr:19:22457244
19	rs115179543	22417596	T	220	2.18E-16	chr:19:22457244
19	rs187117413	22414551	G	119.9	2.61E-16	chr:19:22457244
19	rs375269842	22544016	T	57.23	3.24E-16	chr:19:22457244
19	rs113984339	22455231	A	46.76	6.21E-16	chr:19:22457244
19	rs149415387	22437751	A	47.8	8.45E-16	chr:19:22457244
19	rs111645354	22347290	G	56.76	8.50E-16	chr:19:22457244
19	rs111896267	22337151	A	56.76	8.50E-16	chr:19:22457244
19	rs112380854	22330408	T	56.76	8.50E-16	chr:19:22457244
19	rs112885394	22335107	T	56.76	8.50E-16	chr:19:22457244
19	rs113246191	22323300	A	50.49	1.00E-15	chr:19:22457244
19	rs111231589	22359280	A	50.9	1.12E-15	chr:19:22457244
19	rs111405458	22383509	T	50.9	1.12E-15	chr:19:22457244
19	rs111451901	22384757	C	50.9	1.12E-15	chr:19:22457244
19	rs111497034	22395521	A	50.9	1.12E-15	chr:19:22457244
19	rs111649524	22387942	A	50.9	1.12E-15	chr:19:22457244
19	rs111843291	22383785	A	50.9	1.12E-15	chr:19:22457244
19	rs111847233	22357736	T	50.9	1.12E-15	chr:19:22457244
19	rs111943746	22382564	A	50.9	1.12E-15	chr:19:22457244
19	rs112045666	22378363	G	50.9	1.12E-15	chr:19:22457244
19	rs112135580	22385238	C	50.9	1.12E-15	chr:19:22457244
19	rs112197764	22394487	A	50.9	1.12E-15	chr:19:22457244
19	rs112203564	22365870	G	50.9	1.12E-15	chr:19:22457244
19	rs112282469	22390612	C	50.9	1.12E-15	chr:19:22457244
19	rs112294993	22373901	A	50.9	1.12E-15	chr:19:22457244
19	rs112408934	22382671	G	50.9	1.12E-15	chr:19:22457244
19	rs112526435	22393321	C	50.9	1.12E-15	chr:19:22457244
19	rs112583340	22392029	G	50.9	1.12E-15	chr:19:22457244
19	rs112722708	22375596	C	50.9	1.12E-15	chr:19:22457244
19	rs112745821	22364556	T	50.9	1.12E-15	chr:19:22457244
19	rs112813869	22380889	G	50.9	1.12E-15	chr:19:22457244
19	rs113029510	22367849	T	50.9	1.12E-15	chr:19:22457244
19	rs113191315	22368273	T	50.9	1.12E-15	chr:19:22457244
19	rs113279822	22375736	T	50.9	1.12E-15	chr:19:22457244
19	rs113528164	22369653	T	50.9	1.12E-15	chr:19:22457244
19	rs113582747	22359316	A	50.9	1.12E-15	chr:19:22457244
19	rs113679901	22396847	C	50.9	1.12E-15	chr:19:22457244
19	rs113695641	22380800	C	50.9	1.12E-15	chr:19:22457244
19	rs113820130	22392425	G	50.9	1.12E-15	chr:19:22457244
19	rs113832043	22371460	T	50.9	1.12E-15	chr:19:22457244
19	rs113847498	22359079	G	50.9	1.12E-15	chr:19:22457244

19	rs143032389	22370874	T	50.9	1.12E-15	chr:19:22457244
19	rs145894434	22365290	G	50.9	1.12E-15	chr:19:22457244
19	rs182740216	22366972	G	50.9	1.12E-15	chr:19:22457244
19	rs188552081	22383063	T	50.9	1.12E-15	chr:19:22457244
19	rs113296199	22382233	A	46.06	1.24E-15	chr:19:22457244
19	rs112326121	22374471	A	46.13	1.27E-15	chr:19:22457244
19	rs111716202	22389004	C	58.96	1.28E-15	chr:19:22457244
19	rs368868006;rs113543677	22356888	T	50.11	1.50E-15	chr:19:22457244
19	rs148613793	22549722	G	149.5	2.50E-15	chr:19:22457244
19	rs191121560	22571615	A	138.3	3.21E-15	chr:19:22457244
19	rs111445140	22415931	G	151.7	3.84E-15	chr:19:22457244
19	rs112319065	22532631	C	131.9	3.96E-15	chr:19:22457244
19	rs113011252	22532632	A	131.9	3.96E-15	chr:19:22457244
19	rs146534067	22464950	T	139.6	5.26E-15	chr:19:22457244
19	rs367813024	22612132	T	36.73	9.05E-15	chr:19:22457244
19	rs112273271	22550142	T	220	9.98E-15	chr:19:22457244
19	rs116117409	22422841	C	49.52	1.38E-14	chr:19:22457244
19	rs111947032	22356371	G	136.4	1.83E-14	chr:19:22457244
19	rs112370669	22360739	G	136.4	1.83E-14	chr:19:22457244
19	rs112930128	22395349	G	136.4	1.83E-14	chr:19:22457244
19	rs113445585	22374230	G	136.4	1.83E-14	chr:19:22457244
19	rs113912819	22382874	C	136.4	1.83E-14	chr:19:22457244
19	rs138778030	22384180	C	136.4	1.83E-14	chr:19:22457244
19	rs150364857	22386816	C	136.4	1.83E-14	chr:19:22457244
19	rs111229177	22416439	A	197	2.63E-14	chr:19:22457244
19	rs112747233	22414061	G	197	2.63E-14	chr:19:22457244
19	rs113228771	22423407	T	197	2.63E-14	chr:19:22457244
19	rs113913094	22422857	T	197	2.63E-14	chr:19:22457244
19	rs181917915	22412277	C	197	2.63E-14	chr:19:22457244
19	rs111337812	22608572	A	201.1	2.77E-14	chr:19:22457244
19	rs111379851	22553834	A	201.1	2.77E-14	chr:19:22457244
19	rs111533424	22601263	C	201.1	2.77E-14	chr:19:22457244
19	rs111537669	22545140	T	201.1	2.77E-14	chr:19:22457244
19	rs111538984	22554080	G	201.1	2.77E-14	chr:19:22457244
19	rs111575423	22593969	T	201.1	2.77E-14	chr:19:22457244
19	rs111602907	22564622	C	201.1	2.77E-14	chr:19:22457244
19	rs111619544	22550550	G	201.1	2.77E-14	chr:19:22457244
19	rs111626548	22560244	T	201.1	2.77E-14	chr:19:22457244
19	rs111765860	22625345	A	201.1	2.77E-14	chr:19:22457244
19	rs111814553	22567536	T	201.1	2.77E-14	chr:19:22457244
19	rs111816256	22542169	G	201.1	2.77E-14	chr:19:22457244
19	rs111830349	22555613	A	201.1	2.77E-14	chr:19:22457244
19	rs111912694	22597899	A	201.1	2.77E-14	chr:19:22457244

19	rs112095922	22553682	G	201.1	2.77E-14	chr:19:22457244
19	rs112229850	22557479	A	201.1	2.77E-14	chr:19:22457244
19	rs112258846	22545879	A	201.1	2.77E-14	chr:19:22457244
19	rs112481678	22595822	C	201.1	2.77E-14	chr:19:22457244
19	rs112509518	22552293	T	201.1	2.77E-14	chr:19:22457244
19	rs112561498	22541476	A	201.1	2.77E-14	chr:19:22457244
19	rs112823838	22605733	T	201.1	2.77E-14	chr:19:22457244
19	rs112866031	22590900	T	201.1	2.77E-14	chr:19:22457244
19	rs112869442	22549291	T	201.1	2.77E-14	chr:19:22457244
19	rs112928214	22549578	G	201.1	2.77E-14	chr:19:22457244
19	rs112968058	22539254	C	201.1	2.77E-14	chr:19:22457244
19	rs113042013	22593547	A	201.1	2.77E-14	chr:19:22457244
19	rs113119758	22566695	C	201.1	2.77E-14	chr:19:22457244
19	rs113123935	22551210	A	201.1	2.77E-14	chr:19:22457244
19	rs113127476	22594838	C	201.1	2.77E-14	chr:19:22457244
19	rs113164398	22551597	C	201.1	2.77E-14	chr:19:22457244
19	rs113176625	22545460	A	201.1	2.77E-14	chr:19:22457244
19	rs113272479	22557052	A	201.1	2.77E-14	chr:19:22457244
19	rs113340875	22547756	G	201.1	2.77E-14	chr:19:22457244
19	rs113425349	22550135	G	201.1	2.77E-14	chr:19:22457244
19	rs113646091	22543329	A	201.1	2.77E-14	chr:19:22457244
19	rs113656212	22593993	A	201.1	2.77E-14	chr:19:22457244
19	rs113726304	22551534	G	201.1	2.77E-14	chr:19:22457244
19	rs113730727	22545305	C	201.1	2.77E-14	chr:19:22457244
19	rs113810256	22548456	T	201.1	2.77E-14	chr:19:22457244
19	rs113821661	22539951	G	201.1	2.77E-14	chr:19:22457244
19	rs113823121	22544913	T	201.1	2.77E-14	chr:19:22457244
19	rs113977100	22552401	A	201.1	2.77E-14	chr:19:22457244
19	rs113978061	22554526	T	201.1	2.77E-14	chr:19:22457244
19	rs138835526	22569391	T	201.1	2.77E-14	chr:19:22457244
19	rs139066965	22552874	G	201.1	2.77E-14	chr:19:22457244
19	rs139210906	22641104	T	201.1	2.77E-14	chr:19:22457244
19	rs142060148	22549939	G	201.1	2.77E-14	chr:19:22457244
19	rs142323997	22554030	T	201.1	2.77E-14	chr:19:22457244
19	rs142810110	22545244	A	201.1	2.77E-14	chr:19:22457244
19	rs145673919	22568076	T	201.1	2.77E-14	chr:19:22457244
19	rs146624571	22553225	T	201.1	2.77E-14	chr:19:22457244
19	rs146893961	22642685	A	201.1	2.77E-14	chr:19:22457244
19	rs149149769	22569253	T	201.1	2.77E-14	chr:19:22457244
19	rs149646486	22563903	C	201.1	2.77E-14	chr:19:22457244
19	rs151261211	22571631	T	201.1	2.77E-14	chr:19:22457244
19	rs181891009	22532666	A	201.1	2.77E-14	chr:19:22457244
19	rs184624181	22553364	A	201.1	2.77E-14	chr:19:22457244

19	rs186202171	22562458	G	201.1	2.77E-14	chr:19:22457244
19	rs188759472	22580731	A	201.1	2.77E-14	chr:19:22457244
19	rs190471430	22582187	G	201.1	2.77E-14	chr:19:22457244
19	rs538927712	22544940	A	201.1	2.77E-14	chr:19:22457244
19	rs550663184	22569428	A	201.1	2.77E-14	chr:19:22457244
19	rs555985277	22571044	G	201.1	2.77E-14	chr:19:22457244
19	rs558563281	22566100	C	201.1	2.77E-14	chr:19:22457244
19	rs562237600	22584717	G	201.1	2.77E-14	chr:19:22457244
19	rs571264982	22544939	C	201.1	2.77E-14	chr:19:22457244
19	rs571649137	22580395	T	201.1	2.77E-14	chr:19:22457244
19	rs78672622	22560817	A	201.1	2.77E-14	chr:19:22457244
19	rs74522409	22357958	T	57.12	3.81E-14	chr:19:22457244
19	rs183883031	22504759	A	178.2	4.59E-14	chr:19:22457244
19	rs116255882	22392527	A	46.58	5.83E-14	chr:19:22457244
19	rs143862226	22397331	A	46.58	5.83E-14	chr:19:22457244
19	rs184983951	22390797	C	46.58	5.83E-14	chr:19:22457244
19	rs111243802	22337716	G	177.3	9.99E-14	chr:19:22457244
19	rs111680755	22359378	T	177.3	9.99E-14	chr:19:22457244
19	rs112590977	22337679	G	177.3	9.99E-14	chr:19:22457244
19	rs113190051	22398150	A	177.3	9.99E-14	chr:19:22457244
19	rs113680427	22348139	T	177.3	9.99E-14	chr:19:22457244
19	rs113777797	22338914	A	177.3	9.99E-14	chr:19:22457244
19	rs145893290	22334144	G	177.3	9.99E-14	chr:19:22457244
19	rs186365374	22348627	T	177.3	9.99E-14	chr:19:22457244
19	rs77293225	22333577	T	177.3	9.99E-14	chr:19:22457244
19	rs112252305	22370021	G	176.1	1.07E-13	chr:19:22457244
19	rs186030003	22385984	C	41.49	1.61E-13	chr:19:22457244
19	rs116187005	22324206	C	44.12	1.84E-13	chr:19:22457244
19	rs147947686	22331745	T	44.12	1.84E-13	chr:19:22457244
19	rs372815775	22584624	C	15.34	3.10E-13	chr:19:22457244
19	rs150652573	22123378	A	24.49	3.36E-13	chr:19:22457244
19	rs114336280	22230853	G	37.58	9.75E-13	chr:19:22457244
19	rs114390555	22234503	A	37.58	9.75E-13	chr:19:22457244
19	rs115126226	22231618	A	37.58	9.75E-13	chr:19:22457244
19	rs115234699	22228872	C	37.58	9.75E-13	chr:19:22457244
19	rs115375842	22230349	T	37.58	9.75E-13	chr:19:22457244
19	rs116147909	22235388	C	37.58	9.75E-13	chr:19:22457244
19	rs116208631	22225820	A	37.58	9.75E-13	chr:19:22457244
19	rs116220073	22240979	G	37.58	9.75E-13	chr:19:22457244
19	rs116607420	22223810	A	37.58	9.75E-13	chr:19:22457244
19	rs116618266	22240054	A	37.58	9.75E-13	chr:19:22457244
19	rs138553855	22228574	C	37.58	9.75E-13	chr:19:22457244
19	rs182149206	22242278	A	37.58	9.75E-13	chr:19:22457244

19	rs185846159	22238913	A	37.58	9.75E-13	chr:19:22457244
19	rs185852651	22243109	G	37.58	9.75E-13	chr:19:22457244
19	rs539325267	22224292	A	37.58	9.75E-13	chr:19:22457244
19	rs545672737	22249240	G	37.58	9.75E-13	chr:19:22457244
19	rs560401882	22240262	C	37.58	9.75E-13	chr:19:22457244
19	rs577835819	22236102	G	37.58	9.75E-13	chr:19:22457244
19	rs74924740	22228262	T	37.58	9.75E-13	chr:19:22457244
19	rs77041084	22244488	A	37.58	9.75E-13	chr:19:22457244
19	rs79357837	22231665	G	37.58	9.75E-13	chr:19:22457244
19	rs79459406	22227540	G	37.58	9.75E-13	chr:19:22457244
19	rs531553383	22234434	T	32.89	1.43E-12	chr:19:22457244
19	rs111331902	22523438	A	307	1.62E-12	chr:19:22457244
19	rs111347114	22528269	T	307	1.62E-12	chr:19:22457244
19	rs111347986	22465740	G	307	1.62E-12	chr:19:22457244
19	rs111348116	22491181	G	307	1.62E-12	chr:19:22457244
19	rs111353460	22470418	A	307	1.62E-12	chr:19:22457244
19	rs111392601	22466364	C	307	1.62E-12	chr:19:22457244
19	rs111405729	22461219	A	307	1.62E-12	chr:19:22457244
19	rs111584628	22472494	C	307	1.62E-12	chr:19:22457244
19	rs111589497	22468024	C	307	1.62E-12	chr:19:22457244
19	rs111599921	22462253	G	307	1.62E-12	chr:19:22457244
19	rs111628544	22475742	C	307	1.62E-12	chr:19:22457244
19	rs111675305	22476369	G	307	1.62E-12	chr:19:22457244
19	rs111685365	22467591	G	307	1.62E-12	chr:19:22457244
19	rs111791096	22486821	G	307	1.62E-12	chr:19:22457244
19	rs111794618	22512833	T	307	1.62E-12	chr:19:22457244
19	rs111833085	22503371	C	307	1.62E-12	chr:19:22457244
19	rs111841655	22458159	G	307	1.62E-12	chr:19:22457244
19	rs111878997	22458921	G	307	1.62E-12	chr:19:22457244
19	rs111881871	22463368	A	307	1.62E-12	chr:19:22457244
19	rs111928225	22465110	C	307	1.62E-12	chr:19:22457244
19	rs111935865	22468643	A	307	1.62E-12	chr:19:22457244
19	rs111980271	22468993	C	307	1.62E-12	chr:19:22457244
19	rs112079358	22466375	C	307	1.62E-12	chr:19:22457244
19	rs112086155	22523441	A	307	1.62E-12	chr:19:22457244
19	rs112093011	22508537	A	307	1.62E-12	chr:19:22457244
19	rs112117248	22461875	G	307	1.62E-12	chr:19:22457244
19	rs112279189	22482252	T	307	1.62E-12	chr:19:22457244
19	rs112318231	22508846	G	307	1.62E-12	chr:19:22457244
19	rs112330264	22463147	G	307	1.62E-12	chr:19:22457244
19	rs112366721	22467813	C	307	1.62E-12	chr:19:22457244
19	rs112369737	22482092	T	307	1.62E-12	chr:19:22457244
19	rs112484153	22463235	G	307	1.62E-12	chr:19:22457244

19	rs112511003	22487779	C	307	1.62E-12	chr:19:22457244
19	rs112558538	22506445	T	307	1.62E-12	chr:19:22457244
19	rs112611796	22480481	G	307	1.62E-12	chr:19:22457244
19	rs112616360	22463970	C	307	1.62E-12	chr:19:22457244
19	rs112732764	22506984	G	307	1.62E-12	chr:19:22457244
19	rs112748886	22523715	T	307	1.62E-12	chr:19:22457244
19	rs112760151	22518116	A	307	1.62E-12	chr:19:22457244
19	rs112773780	22461453	A	307	1.62E-12	chr:19:22457244
19	rs112778286	22467575	A	307	1.62E-12	chr:19:22457244
19	rs112837545	22460829	G	307	1.62E-12	chr:19:22457244
19	rs112869551	22492820	T	307	1.62E-12	chr:19:22457244
19	rs112875406	22478544	G	307	1.62E-12	chr:19:22457244
19	rs112933157	22482100	T	307	1.62E-12	chr:19:22457244
19	rs112935650	22514292	T	307	1.62E-12	chr:19:22457244
19	rs112975904	22493695	G	307	1.62E-12	chr:19:22457244
19	rs112979619	22466267	G	307	1.62E-12	chr:19:22457244
19	rs113007464	22488683	A	307	1.62E-12	chr:19:22457244
19	rs113012175	22463167	C	307	1.62E-12	chr:19:22457244
19	rs113017256	22457513	A	307	1.62E-12	chr:19:22457244
19	rs113106657	22456984	A	307	1.62E-12	chr:19:22457244
19	rs113183382	22476736	A	307	1.62E-12	chr:19:22457244
19	rs113218411	22477083	A	307	1.62E-12	chr:19:22457244
19	rs113225857	22502165	C	307	1.62E-12	chr:19:22457244
19	rs113278147	22472449	A	307	1.62E-12	chr:19:22457244
19	rs113408156	22523766	G	307	1.62E-12	chr:19:22457244
19	rs113455707	22457553	A	307	1.62E-12	chr:19:22457244
19	rs113505685	22516109	T	307	1.62E-12	chr:19:22457244
19	rs113661007	22502029	A	307	1.62E-12	chr:19:22457244
19	rs113661444	22468085	C	307	1.62E-12	chr:19:22457244
19	rs113678276	22457485	C	307	1.62E-12	chr:19:22457244
19	rs113757693	22490645	C	307	1.62E-12	chr:19:22457244
19	rs113759458	22465492	A	307	1.62E-12	chr:19:22457244
19	rs113760836	22459182	A	307	1.62E-12	chr:19:22457244
19	rs113883320	22468005	G	307	1.62E-12	chr:19:22457244
19	rs113983011	22458912	T	307	1.62E-12	chr:19:22457244
19	rs138641734	22458090	T	307	1.62E-12	chr:19:22457244
19	rs139826831	22482639	C	307	1.62E-12	chr:19:22457244
19	rs139851550	22464361	T	307	1.62E-12	chr:19:22457244
19	rs141591722	22456499	A	307	1.62E-12	chr:19:22457244
19	rs141659125	22461369	C	307	1.62E-12	chr:19:22457244
19	rs142383733	22481007	A	307	1.62E-12	chr:19:22457244
19	rs142685011	22462196	A	307	1.62E-12	chr:19:22457244
19	rs143231508	22457794	G	307	1.62E-12	chr:19:22457244

19	rs144875635	22457334	C	307	1.62E-12	chr:19:22457244
19	rs145152229	22521124	A	307	1.62E-12	chr:19:22457244
19	rs146187646	22456587	G	307	1.62E-12	chr:19:22457244
19	rs147035832	22505163	T	307	1.62E-12	chr:19:22457244
19	rs147918037	22457338	C	307	1.62E-12	chr:19:22457244
19	rs150670728	22463994	A	307	1.62E-12	chr:19:22457244
19	rs180721768	22458935	C	307	1.62E-12	chr:19:22457244
19	rs181394053	22461788	C	307	1.62E-12	chr:19:22457244
19	rs185929242	22458952	A	307	1.62E-12	chr:19:22457244
19	rs187550228	22459794	T	307	1.62E-12	chr:19:22457244
19	rs188358832	22489165	G	307	1.62E-12	chr:19:22457244
19	rs190514044	22461779	C	307	1.62E-12	chr:19:22457244
19	rs558518009	22465851	A	307	1.62E-12	chr:19:22457244
19	rs570102314	22466800	T	307	1.62E-12	chr:19:22457244
19	rs572997851	22463516	A	307	1.62E-12	chr:19:22457244
19	rs576832782	22465852	A	307	1.62E-12	chr:19:22457244
19	rs62120059	22504646	T	307	1.62E-12	chr:19:22457244
19	rs145605064	22433722	G	10.05	2.23E-12	chr:19:22457244
19	rs545861890	22172046	G	38.59	2.33E-12	chr:19:22457244
19	rs114037048	22221311	G	33.73	2.72E-12	chr:19:22457244
19	rs114154518	22209922	A	33.73	2.72E-12	chr:19:22457244
19	rs114336104	22206569	T	33.73	2.72E-12	chr:19:22457244
19	rs114376748	22187308	A	33.73	2.72E-12	chr:19:22457244
19	rs114581494	22196629	T	33.73	2.72E-12	chr:19:22457244
19	rs115108750	22219863	A	33.73	2.72E-12	chr:19:22457244
19	rs115237687	22215491	A	33.73	2.72E-12	chr:19:22457244
19	rs115279951	22161913	A	33.73	2.72E-12	chr:19:22457244
19	rs115313005	22153742	C	33.73	2.72E-12	chr:19:22457244
19	rs115714685	22143377	T	33.73	2.72E-12	chr:19:22457244
19	rs115749454	22132841	T	33.73	2.72E-12	chr:19:22457244
19	rs116451139	22206670	A	33.73	2.72E-12	chr:19:22457244
19	rs116549366	22129081	A	33.73	2.72E-12	chr:19:22457244
19	rs116635050	22214144	T	33.73	2.72E-12	chr:19:22457244
19	rs139372710	22208245	T	33.73	2.72E-12	chr:19:22457244
19	rs139712084	22134263	G	33.73	2.72E-12	chr:19:22457244
19	rs139827854	22164537	C	33.73	2.72E-12	chr:19:22457244
19	rs140692670	22135897	T	33.73	2.72E-12	chr:19:22457244
19	rs141102799	22210911	C	33.73	2.72E-12	chr:19:22457244
19	rs142038975	22218223	G	33.73	2.72E-12	chr:19:22457244
19	rs143587172	22153672	T	33.73	2.72E-12	chr:19:22457244
19	rs143881598	22144783	A	33.73	2.72E-12	chr:19:22457244
19	rs143988685	22139456	G	33.73	2.72E-12	chr:19:22457244
19	rs144949352	22210627	C	33.73	2.72E-12	chr:19:22457244

19	rs145551125	22154377	A	33.73	2.72E-12	chr:19:22457244
19	rs146152908	22220009	G	33.73	2.72E-12	chr:19:22457244
19	rs146173015	22219565	T	33.73	2.72E-12	chr:19:22457244
19	rs146209419	22139387	C	33.73	2.72E-12	chr:19:22457244
19	rs146855686	22214960	A	33.73	2.72E-12	chr:19:22457244
19	rs147885468	22196623	A	33.73	2.72E-12	chr:19:22457244
19	rs149665725	22135542	C	33.73	2.72E-12	chr:19:22457244
19	rs149777346	22174483	A	33.73	2.72E-12	chr:19:22457244
19	rs150225897	22165950	G	33.73	2.72E-12	chr:19:22457244
19	rs187824736	22139170	G	33.73	2.72E-12	chr:19:22457244
19	rs190926616	22127113	A	33.73	2.72E-12	chr:19:22457244
19	rs373553310	22172557	T	33.73	2.72E-12	chr:19:22457244
19	rs536004240	22221702	T	33.73	2.72E-12	chr:19:22457244
19	rs553282539	22180445	C	33.73	2.72E-12	chr:19:22457244
19	rs567799262	22166136	C	33.73	2.72E-12	chr:19:22457244
19	rs572272889	22136204	A	33.73	2.72E-12	chr:19:22457244
19	rs113995532	22311003	A	35.39	3.06E-12	chr:19:22457244
19	rs114265091	22305581	T	35.39	3.06E-12	chr:19:22457244
19	rs114268107	22299269	A	35.39	3.06E-12	chr:19:22457244
19	rs114333361	22254473	G	35.39	3.06E-12	chr:19:22457244
19	rs114536726	22277396	G	35.39	3.06E-12	chr:19:22457244
19	rs114912357	22304372	C	35.39	3.06E-12	chr:19:22457244
19	rs115315543	22312815	T	35.39	3.06E-12	chr:19:22457244
19	rs116364866	22257200	T	35.39	3.06E-12	chr:19:22457244
19	rs137982685	22308435	A	35.39	3.06E-12	chr:19:22457244
19	rs138520987	22284469	C	35.39	3.06E-12	chr:19:22457244
19	rs140411548	22274933	A	35.39	3.06E-12	chr:19:22457244
19	rs140940314	22286794	T	35.39	3.06E-12	chr:19:22457244
19	rs142024462	22251053	G	35.39	3.06E-12	chr:19:22457244
19	rs142376559	22283429	T	35.39	3.06E-12	chr:19:22457244
19	rs143226803	22252902	G	35.39	3.06E-12	chr:19:22457244
19	rs144582683	22281876	T	35.39	3.06E-12	chr:19:22457244
19	rs144804825	22280914	G	35.39	3.06E-12	chr:19:22457244
19	rs145432030	22252143	A	35.39	3.06E-12	chr:19:22457244
19	rs146601906	22268795	C	35.39	3.06E-12	chr:19:22457244
19	rs147630102	22279149	T	35.39	3.06E-12	chr:19:22457244
19	rs148716112	22252267	G	35.39	3.06E-12	chr:19:22457244
19	rs148864740	22283247	A	35.39	3.06E-12	chr:19:22457244
19	rs149144636	22307223	T	35.39	3.06E-12	chr:19:22457244
19	rs151294558	22303104	T	35.39	3.06E-12	chr:19:22457244
19	rs527521821	22287784	T	35.39	3.06E-12	chr:19:22457244
19	rs527538855	22276237	T	35.39	3.06E-12	chr:19:22457244
19	rs531901473	22308841	G	35.39	3.06E-12	chr:19:22457244

19	rs572201624	22288568	G	35.39	3.06E-12	chr:19:22457244
19	rs74546435	22286868	C	35.39	3.06E-12	chr:19:22457244
19	rs79716546	22278072	A	35.39	3.06E-12	chr:19:22457244
19	rs80197259	22268610	T	35.39	3.06E-12	chr:19:22457244
19	rs1808668	22518657	A	8.192	3.19E-12	chr:19:22457244
19	rs10221484	22455170	G	7.707	3.19E-12	chr:19:22457244
19	rs192813876	22503939	C	295.9	3.33E-12	chr:19:22457244
19	rs563897534	22504020	A	295.9	3.33E-12	chr:19:22457244
19	rs1397578	22112401	T	8.544	3.62E-12	chr:19:22457244
19	rs539718842	22288647	C	31.52	3.89E-12	chr:19:22457244
19	rs79980977	22275829	C	32.48	4.07E-12	chr:19:22457244
19	rs114864357	22261886	T	29.22	5.89E-12	chr:19:22457244
19	rs114053624	22315055	A	28.54	6.07E-12	chr:19:22457244
19	rs115212498	22315714	T	28.54	6.07E-12	chr:19:22457244
19	rs112883809	22360222	C	13.55	6.61E-12	chr:19:22457244
19	rs115445273	22320251	G	25.45	7.45E-12	chr:19:22457244
19	rs139237351	22444018	T	66.79	7.71E-12	chr:19:22457244
19	rs142883534	22414632	C	66.79	7.71E-12	chr:19:22457244
19	rs113726774	22610993	A	260.2	1.15E-11	chr:19:22457244
19	rs142612704	22612156	C	260.2	1.15E-11	chr:19:22457244
19	rs144689364	22610728	G	260.2	1.15E-11	chr:19:22457244
19	rs111580267	22531550	G	6.704	1.26E-11	chr:19:22457244
19	rs10221483	22455112	G	6.533	1.60E-11	chr:19:22457244
19	rs137956372	22252532	C	29.38	1.77E-11	chr:19:22457244
19	rs112823671	22404330	T	228.2	2.28E-11	chr:19:22457244
19	rs115841775	22378130	A	15.98	2.93E-11	chr:19:22457244
19	rs141333367	22550832	T	248.7	3.00E-11	chr:19:22457244
19	rs144186431	22653654	A	248.7	3.00E-11	chr:19:22457244
19	rs145714062	22621527	T	248.7	3.00E-11	chr:19:22457244
19	rs148604204	22569715	T	248.7	3.00E-11	chr:19:22457244
19	rs571428100	22244680	G	18.43	3.73E-11	chr:19:22457244
19	rs188116615	22660198	A	254.3	3.86E-11	chr:19:22457244
19	rs7249555	22114930	T	6.731	6.43E-11	chr:19:22457244
19	rs112140424	22358384	A	196.6	7.23E-11	chr:19:22457244
19	rs113985894	22344306	A	196.6	7.23E-11	chr:19:22457244
19	rs138892114	22341235	A	196.6	7.23E-11	chr:19:22457244
19	rs150434239	22329422	T	196.6	7.23E-11	chr:19:22457244
19	rs569524431;rs536123462	22398606	G	196.6	7.23E-11	chr:19:22457244
19	rs187985394	22764542	G	26.09	1.32E-10	chr:19:22457244
19	rs181154523	22580076	A	29.08	1.35E-10	chr:19:22457244
19	rs114487296	22450493	C	7.763	1.82E-10	chr:19:22457244
19	rs115211114	22450434	G	7.763	1.82E-10	chr:19:22457244
19	rs115402237	22446268	G	7.763	1.82E-10	chr:19:22457244

19	rs116095172	22447297	A	7.763	1.82E-10	chr:19:22457244
19	rs116116710	22450695	G	7.763	1.82E-10	chr:19:22457244
19	rs116290379	22446479	A	7.763	1.82E-10	chr:19:22457244
19	rs116505167	22450685	C	7.763	1.82E-10	chr:19:22457244
19	rs116721114	22450482	T	7.763	1.82E-10	chr:19:22457244
19	rs138350421	22447920	C	7.763	1.82E-10	chr:19:22457244
19	rs139771475	22448256	T	7.763	1.82E-10	chr:19:22457244
19	rs141860818	22448149	G	7.763	1.82E-10	chr:19:22457244
19	rs183369013	22446254	A	7.763	1.82E-10	chr:19:22457244
19	rs542721643	22447006	T	7.763	1.82E-10	chr:19:22457244
19	rs560680679	22447007	G	7.763	1.82E-10	chr:19:22457244
19	rs75134450	22447605	T	7.763	1.82E-10	chr:19:22457244
19	rs76451239	22446557	T	7.763	1.82E-10	chr:19:22457244
19	rs76963327	22446051	T	7.763	1.82E-10	chr:19:22457244
19	rs78665120	22446079	T	7.763	1.82E-10	chr:19:22457244
19	rs79135252	22446546	A	7.763	1.82E-10	chr:19:22457244
19	rs80081358	22450977	G	7.763	1.82E-10	chr:19:22457244
19	rs140081547	22739626	A	16.38	1.99E-10	chr:19:22457244
19	rs144287912	22539692	G	31.4	2.08E-10	chr:19:22457244
19	rs144173274	22112846	G	6.239	2.32E-10	chr:19:22457244
19	rs184140040	22112850	A	6.239	2.32E-10	chr:19:22457244
19	rs188931733	22112851	C	6.239	2.32E-10	chr:19:22457244
19	rs58270429	22109607	T	6.239	2.32E-10	chr:19:22457244
19	rs73933106	22102242	C	6.239	2.32E-10	chr:19:22457244
19	rs73933107	22104518	C	6.239	2.32E-10	chr:19:22457244
19	rs114485832	22108198	C	20.26	2.85E-10	chr:19:22457244
19	rs138388412	22104647	C	20.26	2.85E-10	chr:19:22457244
19	rs139775535	22109977	C	20.26	2.85E-10	chr:19:22457244
19	rs142388513	22112305	T	20.26	2.85E-10	chr:19:22457244
19	rs143241439	22094493	A	20.26	2.85E-10	chr:19:22457244
19	rs145211946	22105330	A	20.26	2.85E-10	chr:19:22457244
19	rs145243020	22108175	G	20.26	2.85E-10	chr:19:22457244
19	rs149721195	22103306	A	20.26	2.85E-10	chr:19:22457244
19	rs149783246	22104896	G	20.26	2.85E-10	chr:19:22457244
19	rs541067957	22100366	T	20.26	2.85E-10	chr:19:22457244
19	rs114289956	22454001	T	7.546	3.28E-10	chr:19:22457244
19	rs114422668	22453355	T	7.546	3.28E-10	chr:19:22457244
19	rs114551844	22452452	T	7.546	3.28E-10	chr:19:22457244
19	rs114612477	22451904	T	7.546	3.28E-10	chr:19:22457244
19	rs115083572	22452285	G	7.546	3.28E-10	chr:19:22457244
19	rs116119620	22453015	T	7.546	3.28E-10	chr:19:22457244
19	rs116279903	22452173	C	7.546	3.28E-10	chr:19:22457244
19	rs139000614	22451845	T	7.546	3.28E-10	chr:19:22457244

19	rs139097230	22452489	T	7.546	3.28E-10	chr:19:22457244
19	rs141440964	22451251	T	7.546	3.28E-10	chr:19:22457244
19	rs191403348	22559796	T	21.59	3.30E-10	chr:19:22457244
19	rs142536481	22451222	A	7.426	4.11E-10	chr:19:22457244
19	rs193035916	22453827	T	7.425	4.21E-10	chr:19:22457244
19	rs139399139	22416692	G	61.75	5.35E-10	chr:19:22457244
19	rs113750672	22327418	G	8.838	5.48E-10	chr:19:22457244
19	rs187062178	22559795	T	18.89	6.84E-10	chr:19:22457244
19	rs189961352	22177388	A	126.8	8.36E-10	chr:19:22457244
19	rs568047338	22450475	A	7.046	9.31E-10	chr:19:22457244
19	rs150780501	22233452	A	127.4	9.61E-10	chr:19:22457244
19	rs138189903	22567136	A	17.03	9.99E-10	chr:19:22457244
19	rs2957811	22658228	T	8.571	1.12E-09	chr:19:22457244
19	rs182106268	22803406	T	23.29	1.12E-09	chr:19:22457244
19	rs149005977	22452903	G	7.056	1.14E-09	chr:19:22457244
19	rs148003905	22492889	G	28.31	1.24E-09	chr:19:22457244
19	rs116015221	22293659	G	24.58	1.38E-09	chr:19:22457244
19	rs139816022	22310284	G	24.58	1.38E-09	chr:19:22457244
19	rs191142508	22337203	T	24.58	1.38E-09	chr:19:22457244
19	rs545003608	22311890	A	24.58	1.38E-09	chr:19:22457244
19	rs76194015	22353141	C	24.58	1.38E-09	chr:19:22457244
19	rs116577376	22453691	A	6.978	1.42E-09	chr:19:22457244
19	rs115943909	22452135	C	6.895	1.56E-09	chr:19:22457244
19	rs114073147	22451639	T	6.876	1.58E-09	chr:19:22457244
19	rs138243368	22451652	G	6.876	1.58E-09	chr:19:22457244
19	rs77463219	22447877	C	7.323	2.35E-09	chr:19:22457244
19	rs143224857	22427423	C	10.12	2.45E-09	chr:19:22457244
19	rs115687200	22157778	T	11.5	3.05E-09	chr:19:22457244
19	rs149966838	22756003	C	38.71	3.20E-09	chr:19:22457244
19	rs114939584	22175083	A	21.05	3.70E-09	chr:19:22457244
19	rs115215406	22149164	C	21.05	3.70E-09	chr:19:22457244
19	rs116608223	22182776	G	21.05	3.70E-09	chr:19:22457244
19	rs140414488	22159440	G	21.05	3.70E-09	chr:19:22457244
19	rs142063237	22138016	T	21.05	3.70E-09	chr:19:22457244
19	rs114397370	22452941	C	6.442	5.57E-09	chr:19:22457244
19	rs66562925	22158713	A	3.903	6.79E-09	chr:19:22457244
19	rs115893105	22452937	A	6.344	6.94E-09	chr:19:22457244
19	rs115059290	22451632	A	6.284	7.66E-09	chr:19:22457244
19	rs143243514	22451798	G	6.276	7.84E-09	chr:19:22457244
19	rs149576034	22451779	A	6.276	7.84E-09	chr:19:22457244
19	rs139826378	22452654	G	6.256	8.04E-09	chr:19:22457244
19	rs141299705	22055302	A	17.67	8.91E-09	chr:19:22457244
19	rs186354431	22791195	G	14.74	1.09E-08	chr:19:22457244

19	rs112112152	22523100	C	539.1	1.10E-08	chr:19:22457244
19	rs145589124	22487608	A	539.1	1.10E-08	chr:19:22457244
19	rs148806686	22512253	A	539.1	1.10E-08	chr:19:22457244
19	rs149714315	22449905	T	539.1	1.10E-08	chr:19:22457244
19	rs150383049	22455754	T	539.1	1.10E-08	chr:19:22457244
19	rs563742399	22466626	A	539.1	1.10E-08	chr:19:22457244
19	rs11668081	22425615	A	4.284	1.71E-08	chr:19:22457244
19	rs540580081	22148238	T	63.62	2.20E-08	chr:19:22457244
19	rs532150113	22300732	A	34.12	2.48E-08	chr:19:22457244
19	rs116663366	22452335	G	114.2	2.61E-08	chr:19:22457244
19	rs190597217	22531263	A	114.2	2.61E-08	chr:19:22457244
19	rs190823937	22487007	C	114.2	2.61E-08	chr:19:22457244
19	rs191104857	22506678	G	114.2	2.61E-08	chr:19:22457244
19	rs538482571	22516648	T	114.2	2.61E-08	chr:19:22457244
19	rs557883619	22504635	T	114.2	2.61E-08	chr:19:22457244
19	rs2905572	22651073	T	6.711	3.12E-08	chr:19:22457244
19	rs1975172	22534603	A	5.443	3.41E-08	chr:19:22457244
19	rs114837217	22446650	C	5.968	3.86E-08	chr:19:22457244
19	rs137916796	22223262	A	235.6	4.85E-08	chr:19:22457244
19	rs138065010	22221080	G	235.6	4.85E-08	chr:19:22457244
19	rs138136455	22235094	G	235.6	4.85E-08	chr:19:22457244
19	rs138944622	22218813	A	235.6	4.85E-08	chr:19:22457244
19	rs139224264	22287539	C	235.6	4.85E-08	chr:19:22457244
19	rs139384201	22262701	G	235.6	4.85E-08	chr:19:22457244
19	rs139697093	22254360	A	235.6	4.85E-08	chr:19:22457244
19	rs139937682	22251675	C	235.6	4.85E-08	chr:19:22457244
19	rs140163563	22241747	G	235.6	4.85E-08	chr:19:22457244
19	rs140373029	22236373	G	235.6	4.85E-08	chr:19:22457244
19	rs140727836	22256795	T	235.6	4.85E-08	chr:19:22457244
19	rs140928310	22169601	T	235.6	4.85E-08	chr:19:22457244
19	rs140938169	22225775	C	235.6	4.85E-08	chr:19:22457244
19	rs141037224	22234862	T	235.6	4.85E-08	chr:19:22457244
19	rs141123787	22261209	T	235.6	4.85E-08	chr:19:22457244
19	rs141317016	22269142	T	235.6	4.85E-08	chr:19:22457244
19	rs141486459	22177998	G	235.6	4.85E-08	chr:19:22457244
19	rs142038665	22272262	C	235.6	4.85E-08	chr:19:22457244
19	rs142133953	22228297	T	235.6	4.85E-08	chr:19:22457244
19	rs142357912	22223320	T	235.6	4.85E-08	chr:19:22457244
19	rs142752732	22213290	A	235.6	4.85E-08	chr:19:22457244
19	rs142768412	22215751	G	235.6	4.85E-08	chr:19:22457244
19	rs143288664	22295865	G	235.6	4.85E-08	chr:19:22457244
19	rs143385332	22264278	C	235.6	4.85E-08	chr:19:22457244
19	rs144077097	22305273	C	235.6	4.85E-08	chr:19:22457244

19	rs144122273	22216039	C	235.6	4.85E-08	chr:19:22457244
19	rs144220574	22236453	T	235.6	4.85E-08	chr:19:22457244
19	rs144361746	22268651	T	235.6	4.85E-08	chr:19:22457244
19	rs144418402	22218366	G	235.6	4.85E-08	chr:19:22457244
19	rs144604323	22300079	C	235.6	4.85E-08	chr:19:22457244
19	rs144620049	22284129	A	235.6	4.85E-08	chr:19:22457244
19	rs144621274	22213695	T	235.6	4.85E-08	chr:19:22457244
19	rs144960409	22260428	C	235.6	4.85E-08	chr:19:22457244
19	rs145325958	22252462	T	235.6	4.85E-08	chr:19:22457244
19	rs145394286	22168575	T	235.6	4.85E-08	chr:19:22457244
19	rs145757686	22218652	T	235.6	4.85E-08	chr:19:22457244
19	rs145962074	22198724	A	235.6	4.85E-08	chr:19:22457244
19	rs146027114	22230310	T	235.6	4.85E-08	chr:19:22457244
19	rs146184496	22310922	G	235.6	4.85E-08	chr:19:22457244
19	rs146400349	22312632	T	235.6	4.85E-08	chr:19:22457244
19	rs147113029	22257396	T	235.6	4.85E-08	chr:19:22457244
19	rs147412239	22270321	G	235.6	4.85E-08	chr:19:22457244
19	rs147785793	22263476	A	235.6	4.85E-08	chr:19:22457244
19	rs147884937	22246105	C	235.6	4.85E-08	chr:19:22457244
19	rs148436093	22293894	T	235.6	4.85E-08	chr:19:22457244
19	rs149151089	22172676	G	235.6	4.85E-08	chr:19:22457244
19	rs149208334	22214718	T	235.6	4.85E-08	chr:19:22457244
19	rs149759540	22254509	T	235.6	4.85E-08	chr:19:22457244
19	rs150070177	22168257	C	235.6	4.85E-08	chr:19:22457244
19	rs150165200	22282864	G	235.6	4.85E-08	chr:19:22457244
19	rs150370139	22249619	G	235.6	4.85E-08	chr:19:22457244
19	rs150572210	22263326	G	235.6	4.85E-08	chr:19:22457244
19	rs150850724	22226907	T	235.6	4.85E-08	chr:19:22457244
19	rs181537500	22277333	G	235.6	4.85E-08	chr:19:22457244
19	rs181602841	22310308	C	235.6	4.85E-08	chr:19:22457244
19	rs183209446	22213538	A	235.6	4.85E-08	chr:19:22457244
19	rs184357408	22284609	T	235.6	4.85E-08	chr:19:22457244
19	rs184379199	22264886	T	235.6	4.85E-08	chr:19:22457244
19	rs184412193	22154331	G	235.6	4.85E-08	chr:19:22457244
19	rs185830182	22282815	T	235.6	4.85E-08	chr:19:22457244
19	rs187083067	22284465	T	235.6	4.85E-08	chr:19:22457244
19	rs187681883	22155362	C	235.6	4.85E-08	chr:19:22457244
19	rs188101230	22270986	G	235.6	4.85E-08	chr:19:22457244
19	rs189140686	22286969	T	235.6	4.85E-08	chr:19:22457244
19	rs189304390	22171804	A	235.6	4.85E-08	chr:19:22457244
19	rs190107251	22286396	A	235.6	4.85E-08	chr:19:22457244
19	rs190155891	22282841	T	235.6	4.85E-08	chr:19:22457244
19	rs190466491	22237645	A	235.6	4.85E-08	chr:19:22457244

19	rs192242698	22217954	T	235.6	4.85E-08	chr:19:22457244
19	rs540350961	22238826	T	235.6	4.85E-08	chr:19:22457244
19	rs549637656	22235566	G	235.6	4.85E-08	chr:19:22457244
19	rs561145349	22291241	T	235.6	4.85E-08	chr:19:22457244
19	rs578101931	22218970	A	235.6	4.85E-08	chr:19:22457244
19	rs2905574	22655531	G	6.482	5.45E-08	chr:19:22457244
19	rs2905575	22656127	A	6.482	5.45E-08	chr:19:22457244
19	rs2360621	22548669	C	5.212	5.81E-08	chr:19:22457244
19	rs8106611	22569618	G	3.737	6.60E-08	chr:19:22457244
19	rs1989120	22205528	T	4.146	7.81E-08	chr:19:22457244
19	rs114714889	22110780	G	7.02	8.89E-08	chr:19:22457244
19	rs114783112	22098748	A	7.02	8.89E-08	chr:19:22457244
19	rs114809292	22094232	T	7.02	8.89E-08	chr:19:22457244
19	rs115231855	22095693	G	7.02	8.89E-08	chr:19:22457244
19	rs192776996	22476682	G	89.23	1.14E-07	chr:19:22457244
19	rs187405198	22194270	C	190.6	1.30E-07	chr:19:22457244
19	rs11671804	22591216	C	4.784	1.43E-07	chr:19:22457244
19	rs12973769	22593223	C	4.784	1.43E-07	chr:19:22457244
19	rs150054286	22116842	G	6.636	1.46E-07	chr:19:22457244
19	rs1651572	22109563	A	3.997	1.47E-07	chr:19:22457244
19	rs11669254	22544805	A	4.469	1.65E-07	chr:19:22457244
19	rs140212785	22121675	T	290.8	1.77E-07	chr:19:22457244
19	rs147165335	22272039	C	290.8	1.77E-07	chr:19:22457244
19	rs148566913	22142435	A	290.8	1.77E-07	chr:19:22457244
19	rs68168074	22120333	T	3.653	1.91E-07	chr:19:22457244
19	rs138450162	22148674	A	6.09	2.52E-07	chr:19:22457244
19	rs146657049	22138227	C	6.044	2.57E-07	chr:19:22457244
19	rs138324788	22127166	A	6.11	2.58E-07	chr:19:22457244
19	rs76319388	22123380	A	3.506	2.73E-07	chr:19:22457244
19	rs114684241	22159941	A	5.681	2.76E-07	chr:19:22457244
19	rs115190567	22175786	T	5.681	2.76E-07	chr:19:22457244
19	rs115804972	22194494	T	5.681	2.76E-07	chr:19:22457244
19	rs116141646	22161755	T	5.681	2.76E-07	chr:19:22457244
19	rs116394195	22188702	G	5.681	2.76E-07	chr:19:22457244
19	rs141590519	22166246	A	5.681	2.76E-07	chr:19:22457244
19	rs145236020	22159069	T	5.681	2.76E-07	chr:19:22457244
19	rs146289217	22155844	T	5.681	2.76E-07	chr:19:22457244
19	rs146758677	22178933	G	5.681	2.76E-07	chr:19:22457244
19	rs148680320	22184686	G	5.681	2.76E-07	chr:19:22457244
19	rs150812540	22187753	C	5.681	2.76E-07	chr:19:22457244
19	rs151164349	22159776	T	5.681	2.76E-07	chr:19:22457244
19	rs151216289	22155922	A	5.681	2.76E-07	chr:19:22457244
19	rs189198732	22181216	T	5.681	2.76E-07	chr:19:22457244

19	rs10421680	22614451	T	4.656	2.90E-07	chr:19:22457244
19	rs114405470	22150913	T	5.967	3.10E-07	chr:19:22457244
19	rs114443803	22135979	G	5.967	3.10E-07	chr:19:22457244
19	rs115563563	22132411	T	5.967	3.10E-07	chr:19:22457244
19	rs116267189	22134427	C	5.967	3.10E-07	chr:19:22457244
19	rs138257968	22138742	A	5.967	3.10E-07	chr:19:22457244
19	rs140726533	22138265	G	5.967	3.10E-07	chr:19:22457244
19	rs147703981	22142989	G	5.967	3.10E-07	chr:19:22457244
19	rs150619604	22134247	C	5.967	3.10E-07	chr:19:22457244
19	rs1973597	22142202	A	5.967	3.10E-07	chr:19:22457244
19	rs61659969	22133446	T	5.967	3.10E-07	chr:19:22457244
19	rs10409819	22602919	T	3.49	3.34E-07	chr:19:22457244
19	rs114091543	22183039	A	5.57	3.41E-07	chr:19:22457244
19	rs10405555	22617622	T	4.109	3.59E-07	chr:19:22457244
19	rs28787019	22617223	A	4.109	3.59E-07	chr:19:22457244
19	rs186894845	22452402	T	293.9	3.61E-07	chr:19:22457244
19	rs115855159	22100503	A	5.861	3.70E-07	chr:19:22457244
19	rs1828452	22112137	T	5.861	3.70E-07	chr:19:22457244
19	rs6511298	22111469	C	5.861	3.70E-07	chr:19:22457244
19	rs570488053	22413076	A	76	3.83E-07	chr:19:22457244
19	rs377000167	22190372	A	5.478	4.42E-07	chr:19:22457244
19	rs116474134	22090746	C	5.87	5.56E-07	chr:19:22457244
19	rs145444878	22096073	G	13.43	5.58E-07	chr:19:22457244
19	rs190626140	22093710	C	13.43	5.58E-07	chr:19:22457244
19	rs62119972	22425682	C	4.898	5.64E-07	chr:19:22457244
19	rs73924264	22730161	C	4.536	5.99E-07	chr:19:22457244
19	rs2885037	22606672	A	4.591	6.07E-07	chr:19:22457244
19	rs145146444	22451345	G	4.896	6.27E-07	chr:19:22457244
19	rs56927650	22805247	C	5.434	7.21E-07	chr:19:22457244
19	rs7253558	22101672	C	3.229	7.59E-07	chr:19:22457244
19	rs281155	22414594	G	3.411	7.98E-07	chr:19:22457244
19	rs111440289	22532918	A	4.199	8.25E-07	chr:19:22457244
19	rs56935667	22798098	T	5.475	9.85E-07	chr:19:22457244
19	rs140714593	22505024	C	3.891	1.11E-06	chr:19:22457244
19	rs190571209	22505025	G	3.891	1.11E-06	chr:19:22457244
19	rs192221778	22443087	T	225.6	1.11E-06	chr:19:22457244
19	rs115644378	22323470	A	68.72	1.11E-06	chr:19:22457244
19	rs145451705	22323004	T	68.72	1.11E-06	chr:19:22457244
19	rs180736225	22342040	A	68.72	1.11E-06	chr:19:22457244
19	rs187298748	22341468	T	68.72	1.11E-06	chr:19:22457244
19	rs113269615	22606977	A	4.18	1.12E-06	chr:19:22457244
19	rs10407093	22618285	A	3.844	1.33E-06	chr:19:22457244
19	rs10415112	22619052	C	3.844	1.33E-06	chr:19:22457244

19	rs7257489	22113083	C	5.516	1.34E-06	chr:19:22457244
19	rs112258788	22669331	A	14.44	1.42E-06	chr:19:22457244
19	rs188121447	22666103	T	14.44	1.42E-06	chr:19:22457244
19	rs144168791	22150963	C	5.388	1.42E-06	chr:19:22457244
19	rs576475899	22572221	A	40.53	1.49E-06	chr:19:22457244
19	rs113181884	22586084	C	3.275	1.53E-06	chr:19:22457244
19	rs56768795	22586073	A	3.275	1.53E-06	chr:19:22457244
19	rs62121331	22809119	A	5.157	1.59E-06	chr:19:22457244
19	rs281173	22424997	A	3.17	1.81E-06	chr:19:22457244
19	rs115740412	22104738	G	3.1	1.82E-06	chr:19:22457244
19	rs7254293	22105469	C	3.1	1.82E-06	chr:19:22457244
19	rs73933109	22104602	T	3.1	1.82E-06	chr:19:22457244
19	rs73933112	22104993	T	3.1	1.82E-06	chr:19:22457244
19	rs78781407	22716995	G	4.644	2.04E-06	chr:19:22457244
19	rs111343581	22505121	A	3.759	2.11E-06	chr:19:22457244
19	rs111394256	22521445	G	3.759	2.11E-06	chr:19:22457244
19	rs111398872	22487132	C	3.759	2.11E-06	chr:19:22457244
19	rs111402663	22511428	T	3.759	2.11E-06	chr:19:22457244
19	rs111403022	22491809	G	3.759	2.11E-06	chr:19:22457244
19	rs111430028	22507923	T	3.759	2.11E-06	chr:19:22457244
19	rs111589669	22493334	C	3.759	2.11E-06	chr:19:22457244
19	rs111602603	22519137	G	3.759	2.11E-06	chr:19:22457244
19	rs111626129	22515060	T	3.759	2.11E-06	chr:19:22457244
19	rs111659459	22488651	C	3.759	2.11E-06	chr:19:22457244
19	rs111840114	22489991	C	3.759	2.11E-06	chr:19:22457244
19	rs111878522	22480384	C	3.759	2.11E-06	chr:19:22457244
19	rs111915770	22496189	C	3.759	2.11E-06	chr:19:22457244
19	rs111920900	22480534	G	3.759	2.11E-06	chr:19:22457244
19	rs112033021	22505136	T	3.759	2.11E-06	chr:19:22457244
19	rs112038831	22480770	A	3.759	2.11E-06	chr:19:22457244
19	rs112075571	22500405	T	3.759	2.11E-06	chr:19:22457244
19	rs112099028	22488225	A	3.759	2.11E-06	chr:19:22457244
19	rs112147518	22492556	G	3.759	2.11E-06	chr:19:22457244
19	rs112405385	22468863	C	3.759	2.11E-06	chr:19:22457244
19	rs112493995	22500985	T	3.759	2.11E-06	chr:19:22457244
19	rs112535242	22490303	T	3.759	2.11E-06	chr:19:22457244
19	rs112562889	22510641	T	3.759	2.11E-06	chr:19:22457244
19	rs112614989	22495601	T	3.759	2.11E-06	chr:19:22457244
19	rs112738992	22487087	T	3.759	2.11E-06	chr:19:22457244
19	rs112771592	22481643	A	3.759	2.11E-06	chr:19:22457244
19	rs112839081	22503731	T	3.759	2.11E-06	chr:19:22457244
19	rs112925452	22475485	G	3.759	2.11E-06	chr:19:22457244
19	rs112934506	22500998	G	3.759	2.11E-06	chr:19:22457244

19	rs112976811	22470822	G	3.759	2.11E-06	chr:19:22457244
19	rs113010780	22494716	A	3.759	2.11E-06	chr:19:22457244
19	rs113099868	22508558	C	3.759	2.11E-06	chr:19:22457244
19	rs113106398	22488360	A	3.759	2.11E-06	chr:19:22457244
19	rs113179295	22489112	G	3.759	2.11E-06	chr:19:22457244
19	rs113183858	22480158	C	3.759	2.11E-06	chr:19:22457244
19	rs113277011	22506028	A	3.759	2.11E-06	chr:19:22457244
19	rs113345334	22480739	A	3.759	2.11E-06	chr:19:22457244
19	rs113427950	22482205	G	3.759	2.11E-06	chr:19:22457244
19	rs113443194	22470891	C	3.759	2.11E-06	chr:19:22457244
19	rs113493517	22491195	T	3.759	2.11E-06	chr:19:22457244
19	rs113525304	22506218	T	3.759	2.11E-06	chr:19:22457244
19	rs113588745	22514004	T	3.759	2.11E-06	chr:19:22457244
19	rs113594810	22487473	A	3.759	2.11E-06	chr:19:22457244
19	rs113675059	22488659	G	3.759	2.11E-06	chr:19:22457244
19	rs113880235	22530533	A	3.759	2.11E-06	chr:19:22457244
19	rs113978372	22519613	A	3.759	2.11E-06	chr:19:22457244
19	rs114769949	22489408	G	3.759	2.11E-06	chr:19:22457244
19	rs142839721	22504617	C	3.759	2.11E-06	chr:19:22457244
19	rs150276670	22503794	C	3.759	2.11E-06	chr:19:22457244
19	rs183724818	22504740	T	3.759	2.11E-06	chr:19:22457244
19	rs188095427	22504742	T	3.759	2.11E-06	chr:19:22457244
19	rs57772560	22474380	G	3.759	2.11E-06	chr:19:22457244
19	rs58410881	22492992	T	3.759	2.11E-06	chr:19:22457244
19	rs58457864	22503016	A	3.759	2.11E-06	chr:19:22457244
19	rs58512684	22503246	C	3.759	2.11E-06	chr:19:22457244
19	rs59332835	22503156	G	3.759	2.11E-06	chr:19:22457244
19	rs59578914	22505820	T	3.759	2.11E-06	chr:19:22457244
19	rs59621371	22472621	C	3.759	2.11E-06	chr:19:22457244
19	rs60233053	22472668	A	3.759	2.11E-06	chr:19:22457244
19	rs60583994	22518193	C	3.759	2.11E-06	chr:19:22457244
19	rs60837647	22511651	A	3.759	2.11E-06	chr:19:22457244
19	rs61377758	22487928	T	3.759	2.11E-06	chr:19:22457244
19	rs72481610	22521300	A	3.759	2.11E-06	chr:19:22457244
19	rs74578514	22512257	T	3.759	2.11E-06	chr:19:22457244
19	rs74583053	22516393	A	3.759	2.11E-06	chr:19:22457244
19	rs74583407	22495342	T	3.759	2.11E-06	chr:19:22457244
19	rs74724191	22514863	T	3.759	2.11E-06	chr:19:22457244
19	rs75167566	22510652	G	3.759	2.11E-06	chr:19:22457244
19	rs75351296	22514818	T	3.759	2.11E-06	chr:19:22457244
19	rs75451854	22513005	G	3.759	2.11E-06	chr:19:22457244
19	rs75855320	22514666	A	3.759	2.11E-06	chr:19:22457244
19	rs75917018	22514169	T	3.759	2.11E-06	chr:19:22457244

19	rs76181558	22509045	G	3.759	2.11E-06	chr:19:22457244
19	rs76285566	22515640	C	3.759	2.11E-06	chr:19:22457244
19	rs76618973	22512967	G	3.759	2.11E-06	chr:19:22457244
19	rs76619922	22496282	T	3.759	2.11E-06	chr:19:22457244
19	rs76882085	22513715	G	3.759	2.11E-06	chr:19:22457244
19	rs76899249	22488129	C	3.759	2.11E-06	chr:19:22457244
19	rs77262019	22512544	A	3.759	2.11E-06	chr:19:22457244
19	rs77279152	22480135	G	3.759	2.11E-06	chr:19:22457244
19	rs77336446	22511992	A	3.759	2.11E-06	chr:19:22457244
19	rs77364752	22495292	T	3.759	2.11E-06	chr:19:22457244
19	rs77408464	22475802	T	3.759	2.11E-06	chr:19:22457244
19	rs77892334	22514175	C	3.759	2.11E-06	chr:19:22457244
19	rs77936818	22510441	T	3.759	2.11E-06	chr:19:22457244
19	rs77987565	22513394	T	3.759	2.11E-06	chr:19:22457244
19	rs78300029	22499853	G	3.759	2.11E-06	chr:19:22457244
19	rs78511685	22500713	T	3.759	2.11E-06	chr:19:22457244
19	rs78770026	22479009	T	3.759	2.11E-06	chr:19:22457244
19	rs78807780	22469259	T	3.759	2.11E-06	chr:19:22457244
19	rs78829247	22482490	C	3.759	2.11E-06	chr:19:22457244
19	rs78833242	22472768	G	3.759	2.11E-06	chr:19:22457244
19	rs79205652	22512138	C	3.759	2.11E-06	chr:19:22457244
19	rs79291199	22510138	G	3.759	2.11E-06	chr:19:22457244
19	rs79523019	22507475	G	3.759	2.11E-06	chr:19:22457244
19	rs80068572	22514201	G	3.759	2.11E-06	chr:19:22457244
19	rs639884	22104847	A	3.068	2.19E-06	chr:19:22457244
19	rs187257161	21977190	T	16.52	2.32E-06	chr:19:22457244
19	rs113212530	22490432	C	3.734	2.39E-06	chr:19:22457244
19	rs112661895	22480668	G	3.73	2.43E-06	chr:19:22457244
19	rs10420033	22611191	T	4.295	2.46E-06	chr:19:22457244
19	rs281202	22406331	A	3.164	2.90E-06	chr:19:22457244
19	rs62121869	22504698	C	3.61	2.91E-06	chr:19:22457244
19	rs111346474	22516745	A	3.597	2.94E-06	chr:19:22457244
19	rs2606273	22413654	G	3.129	3.25E-06	chr:19:22457244
19	rs2606275	22413622	G	3.129	3.25E-06	chr:19:22457244
19	rs281204	22407056	G	3.129	3.25E-06	chr:19:22457244
19	rs281205	22407824	T	3.129	3.25E-06	chr:19:22457244
19	rs4932966	22412232	A	3.129	3.25E-06	chr:19:22457244
19	rs112137052	22299648	T	3.734	3.56E-06	chr:19:22457244
19	rs11880424	22541239	C	3.291	3.61E-06	chr:19:22457244
19	rs111471672	22410364	G	3.088	3.82E-06	chr:19:22457244
19	rs75303808	22726897	C	4.427	3.88E-06	chr:19:22457244
19	rs2359817	22205523	A	3.006	3.94E-06	chr:19:22457244
19	rs115422672	22725140	G	4.431	4.02E-06	chr:19:22457244

19	rs10423736	22596132	T	2.987	4.03E-06	chr:19:22457244
19	rs6511360	22727289	A	4.39	4.31E-06	chr:19:22457244
19	rs112913380	22626103	A	3.698	4.60E-06	chr:19:22457244
19	rs113719399	22628520	A	3.698	4.60E-06	chr:19:22457244
19	rs113768322	22626678	G	3.698	4.60E-06	chr:19:22457244
19	rs187858863	22447018	G	164	4.64E-06	chr:19:22457244
19	rs115004803	22103990	A	4.398	4.78E-06	chr:19:22457244
19	rs150844285	22411568	G	3.072	4.83E-06	chr:19:22457244
19	rs543630191	22504027	C	3.635	4.86E-06	chr:19:22457244
19	rs545769504	22504019	C	3.635	4.86E-06	chr:19:22457244
19	rs115961265	22629483	A	3.674	5.19E-06	chr:19:22457244
19	rs2195964	22538338	C	2.961	5.40E-06	chr:19:22457244
19	rs4932760	22540455	T	2.961	5.40E-06	chr:19:22457244
19	rs113616597	22621797	A	3.654	5.66E-06	chr:19:22457244
19	rs141397890	22504492	C	3.58	6.12E-06	chr:19:22457244
19	rs535198870	22504470	C	3.58	6.12E-06	chr:19:22457244
19	rs112235889	22623216	T	3.62	6.47E-06	chr:19:22457244
19	rs281197	22404604	A	2.991	6.51E-06	chr:19:22457244
19	rs73556461	22629854	A	3.62	6.72E-06	chr:19:22457244
19	rs463200	22327489	T	2.947	7.07E-06	chr:19:22457244
19	rs13382114	22545739	G	2.919	7.09E-06	chr:19:22457244
19	rs112485492	22488990	C	3.518	7.26E-06	chr:19:22457244
19	rs113454521	22488964	C	3.518	7.26E-06	chr:19:22457244
19	rs543507823	22444547	G	4.015	7.83E-06	chr:19:22457244
19	rs12608842	22548013	G	2.884	7.85E-06	chr:19:22457244
19	rs111616991	22508462	A	3.496	7.92E-06	chr:19:22457244
19	rs3867187	22570220	C	3.563	8.38E-06	chr:19:22457244
19	rs8107513	22564534	C	2.851	8.78E-06	chr:19:22457244
19	rs8107704	22564531	G	2.851	8.78E-06	chr:19:22457244
19	rs7508099	22556203	T	2.85	8.80E-06	chr:19:22457244
19	rs10402396	22601809	C	2.849	9.07E-06	chr:19:22457244
19	rs10406199	22584530	G	2.849	9.07E-06	chr:19:22457244
19	rs10407050	22584914	G	2.849	9.07E-06	chr:19:22457244
19	rs10408428	22578023	T	2.849	9.07E-06	chr:19:22457244
19	rs10414591	22600122	A	2.849	9.07E-06	chr:19:22457244
19	rs10417380	22596625	T	2.849	9.07E-06	chr:19:22457244
19	rs10420380	22600624	T	2.849	9.07E-06	chr:19:22457244
19	rs12151261	22587929	T	2.849	9.07E-06	chr:19:22457244
19	rs16999542	22573092	A	2.849	9.07E-06	chr:19:22457244
19	rs17391718	22554154	A	2.849	9.07E-06	chr:19:22457244
19	rs4024624	22568264	C	2.849	9.07E-06	chr:19:22457244
19	rs56027264	22554354	T	2.849	9.07E-06	chr:19:22457244
19	rs58374468	22562522	C	2.849	9.07E-06	chr:19:22457244

19	rs7247220	22589489	G	2.849	9.07E-06	chr:19:22457244
19	rs7247735	22575488	T	2.849	9.07E-06	chr:19:22457244
19	rs7250710	22557231	G	2.849	9.07E-06	chr:19:22457244
19	rs7253315	22594439	T	2.849	9.07E-06	chr:19:22457244
19	rs7255934	22596865	C	2.849	9.07E-06	chr:19:22457244
19	rs75653200	22579503	T	2.849	9.07E-06	chr:19:22457244
19	rs8100077	22593112	T	2.849	9.07E-06	chr:19:22457244
19	rs8111665	22588559	T	2.849	9.07E-06	chr:19:22457244
19	rs2957848	22583747	G	2.846	9.52E-06	chr:19:22457244
19	rs7249374	22533465	C	3.39	1.02E-05	chr:19:22457244
19	rs11085514	22425837	A	0.2628	1.04E-05	chr:19:22457244
19	rs807952	22117758	A	2.861	1.06E-05	chr:19:22457244
19	rs807953	22117746	G	2.861	1.06E-05	chr:19:22457244
19	rs557388	22353206	A	2.875	1.12E-05	chr:19:22457244
19	rs1378915	22362527	C	2.886	1.25E-05	chr:19:22457244
19	rs381995	22361666	T	2.886	1.25E-05	chr:19:22457244
19	rs181072468	22590742	A	48.91	1.34E-05	chr:19:22457244
19	rs529629136	22584157	T	48.91	1.34E-05	chr:19:22457244
19	rs532030191	22568566	G	48.91	1.34E-05	chr:19:22457244
19	rs545541353	22467138	A	48.91	1.34E-05	chr:19:22457244
19	rs548682242	22625428	C	48.91	1.34E-05	chr:19:22457244
19	rs563059259	22584711	A	48.91	1.34E-05	chr:19:22457244
19	rs146669501	22427433	C	4.367	1.36E-05	chr:19:22457244
19	rs1634469	22101863	T	2.768	1.48E-05	chr:19:22457244
19	rs4024619	22586839	G	2.797	1.50E-05	chr:19:22457244
19	rs1230299	22624040	C	3.384	1.52E-05	chr:19:22457244
19	rs4932932	22203214	C	0.3102	1.54E-05	chr:19:22457244
19	rs8109237	22202739	A	0.3102	1.54E-05	chr:19:22457244
19	rs8100117	22195322	T	0.3101	1.57E-05	chr:19:22457244
19	rs4371283	22373810	C	2.852	1.58E-05	chr:19:22457244
19	rs8112041	22604565	T	2.751	1.61E-05	chr:19:22457244
19	rs201163831	29845670	T	54.36	1.78E-44	chr:19:29855781
19	rs200026439	29845668	T	54.13	2.11E-44	chr:19:29855781
19	rs62109919	29845666	T	46.38	3.71E-43	chr:19:29855781
19	rs62109918	29845664	T	50.06	5.97E-43	chr:19:29855781
19	rs12608817	29808821	A	37.53	1.13E-42	chr:19:29855781
19	rs12610707	29808907	C	37.53	1.13E-42	chr:19:29855781
19	rs12978558	29807938	C	28.08	4.23E-42	chr:19:29855781
19	rs200009518	29841360	G	32.82	1.04E-40	chr:19:29855781
19	rs200273877	29841554	A	97.46	1.56E-39	chr:19:29855781
19	rs201714995	29841536	A	96.59	2.05E-39	chr:19:29855781
19	rs10416302	29851854	C	26.8	7.36E-39	chr:19:29855781
19	rs7249741	29849562	G	26.73	8.39E-39	chr:19:29855781

19	rs9789288	29806349	T	23.71	3.32E-38	chr:19:29855781
19	rs11881314	29830193	A	105.9	3.13E-37	chr:19:29855781
19	rs62108163	29828752	T	114.8	3.41E-37	chr:19:29855781
19	rs62108164	29828755	C	114.3	3.76E-37	chr:19:29855781
19	rs10401431	29829429	G	113.7	4.10E-37	chr:19:29855781
19	rs10407240	29829500	C	113.7	4.10E-37	chr:19:29855781
19	rs10409875	29827166	A	113.7	4.10E-37	chr:19:29855781
19	rs10410499	29827426	T	113.7	4.10E-37	chr:19:29855781
19	rs10419949	29828341	G	113.7	4.10E-37	chr:19:29855781
19	rs11881407	29829948	T	113.7	4.10E-37	chr:19:29855781
19	rs11881459	29830092	T	113.7	4.10E-37	chr:19:29855781
19	rs11881508	29830309	G	113.7	4.10E-37	chr:19:29855781
19	rs12610959	29827626	C	113.7	4.10E-37	chr:19:29855781
19	rs10413728	29833591	C	22.92	4.15E-37	chr:19:29855781
19	rs2193501	29834999	A	22.92	4.15E-37	chr:19:29855781
19	rs10853798	29821124	A	22.6	7.71E-37	chr:19:29855781
19	rs1119169	29850778	T	23.2	5.96E-36	chr:19:29855781
19	rs10409090	29823614	G	135.7	1.52E-35	chr:19:29855781
19	rs8099873	29808683	A	19.1	2.55E-35	chr:19:29855781
19	rs12150940	29840750	C	128.3	2.60E-35	chr:19:29855781
19	rs12151167	29840748	T	128.3	2.60E-35	chr:19:29855781
19	rs2111498	29822858	A	140.2	5.27E-35	chr:19:29855781
19	rs35184298	29830558	A	21.54	1.78E-34	chr:19:29855781
19	rs28488633	29823953	C	159.7	1.79E-34	chr:19:29855781
19	rs4805368	29828621	G	21.58	1.86E-34	chr:19:29855781
19	rs8102393	29820560	C	149.5	2.63E-34	chr:19:29855781
19	rs8107784	29824307	T	154.6	4.94E-34	chr:19:29855781
19	rs979413	29818635	A	148.1	5.14E-34	chr:19:29855781
19	rs28792508	29841216	C	17.13	1.60E-33	chr:19:29855781
19	rs200136359	29843211	T	0.08887	3.44E-33	chr:19:29855781
19	rs200658849	29843213	C	0.08887	3.44E-33	chr:19:29855781
19	rs76108985	29843218	T	0.08887	3.44E-33	chr:19:29855781
19	rs2216340	29823183	G	159.9	4.30E-33	chr:19:29855781
19	rs2865870	29821980	C	174.9	4.75E-33	chr:19:29855781
19	rs10417724	29819252	A	174.9	4.94E-33	chr:19:29855781
19	rs10404772	29821253	A	174	5.31E-33	chr:19:29855781
19	rs10408183	29823239	T	174	5.31E-33	chr:19:29855781
19	rs10423259	29820105	A	174	5.31E-33	chr:19:29855781
19	rs10853797	29821094	C	174	5.31E-33	chr:19:29855781
19	rs10853800	29821178	C	174	5.31E-33	chr:19:29855781
19	rs11083916	29821200	C	174	5.31E-33	chr:19:29855781
19	rs11083917	29821301	C	174	5.31E-33	chr:19:29855781
19	rs2111496	29822748	A	174	5.31E-33	chr:19:29855781

19	rs2160716	29822600	C	174	5.31E-33	chr:19:29855781
19	rs2193497	29821737	A	174	5.31E-33	chr:19:29855781
19	rs2216339	29821569	G	174	5.31E-33	chr:19:29855781
19	rs28546871	29821491	C	174	5.31E-33	chr:19:29855781
19	rs2865869	29821979	T	174	5.31E-33	chr:19:29855781
19	rs2865871	29822061	T	174	5.31E-33	chr:19:29855781
19	rs5007513	29821553	A	174	5.31E-33	chr:19:29855781
19	rs8107378	29823430	C	174	5.31E-33	chr:19:29855781
19	rs8113732	29820742	G	174	5.31E-33	chr:19:29855781
19	rs1558363	29865313	C	21.13	5.97E-33	chr:19:29855781
19	rs142004081	29841268	A	19.89	6.13E-33	chr:19:29855781
19	rs979412	29818376	A	168.7	6.84E-33	chr:19:29855781
19	rs79585427	29843225	T	0.09233	1.14E-32	chr:19:29855781
19	rs10415948	29824022	G	198.5	9.87E-32	chr:19:29855781
19	rs10416173	29824125	T	198.5	9.87E-32	chr:19:29855781
19	rs2865875	29825068	A	198.5	9.87E-32	chr:19:29855781
19	rs2865876	29825093	A	198.5	9.87E-32	chr:19:29855781
19	rs2865877	29825180	C	198.5	9.87E-32	chr:19:29855781
19	rs727798	29811828	C	21.43	1.01E-31	chr:19:29855781
19	rs10425611	29813263	T	21.46	1.04E-31	chr:19:29855781
19	rs10419653	29812484	G	21.35	1.12E-31	chr:19:29855781
19	rs10425007	29812397	C	21.35	1.12E-31	chr:19:29855781
19	rs2216341	29812294	T	21.35	1.12E-31	chr:19:29855781
19	rs2865874	29825020	A	189.3	1.28E-31	chr:19:29855781
19	rs11880387	29810986	A	20.92	1.83E-31	chr:19:29855781
19	rs10422587	29869112	A	20.06	2.04E-31	chr:19:29855781
19	rs10411752	29826775	T	0.09285	3.47E-31	chr:19:29855781
19	rs10405460	29840781	A	194.6	1.26E-30	chr:19:29855781
19	rs10410829	29840787	T	194.6	1.26E-30	chr:19:29855781
19	rs10401757	29866045	T	17.54	1.90E-30	chr:19:29855781
19	rs10403590	29844676	C	196.7	3.09E-30	chr:19:29855781
19	rs8109855	29828233	A	0.05409	3.23E-30	chr:19:29855781
19	rs4591272	29841167	G	0.1595	3.85E-30	chr:19:29855781
19	rs10426495	29866098	C	21.14	4.22E-30	chr:19:29855781
19	rs1981676	29868320	A	21.08	4.68E-30	chr:19:29855781
19	rs28857523	29844100	G	205	5.17E-30	chr:19:29855781
19	rs28870995	29844110	C	205	5.17E-30	chr:19:29855781
19	rs200964938	29841291	G	18.5	9.46E-30	chr:19:29855781
19	rs34235250	29852282	C	18.84	1.33E-29	chr:19:29855781
19	rs1558364	29865345	A	17.34	1.99E-29	chr:19:29855781
19	rs2080613	29811982	A	21.17	2.26E-29	chr:19:29855781
19	rs1558365	29865746	C	17.19	3.11E-29	chr:19:29855781
19	rs1558366	29865847	T	17.19	3.11E-29	chr:19:29855781

19	rs8100333	29866136	T	17.19	3.11E-29	chr:19:29855781
19	rs8100334	29866137	T	17.19	3.11E-29	chr:19:29855781
19	rs9916951	29865388	G	17.19	3.11E-29	chr:19:29855781
19	rs9749537	29844679	C	0.04853	4.69E-29	chr:19:29855781
19	rs28573811	29867469	T	21.16	6.26E-29	chr:19:29855781
19	rs74838486	29842509	G	0.02811	6.35E-29	chr:19:29855781
19	rs758770	29870136	G	19.07	7.90E-29	chr:19:29855781
19	rs12973626	29867364	C	20.89	8.26E-29	chr:19:29855781
19	rs12979587	29867327	A	20.89	8.26E-29	chr:19:29855781
19	rs12980588	29867736	A	20.89	8.26E-29	chr:19:29855781
19	rs12981862	29867527	G	20.89	8.26E-29	chr:19:29855781
19	rs12981877	29867555	T	20.89	8.26E-29	chr:19:29855781
19	rs1981675	29868188	T	20.89	8.26E-29	chr:19:29855781
19	rs1981677	29868499	A	20.89	8.26E-29	chr:19:29855781
19	rs4271695	29866742	A	20.89	8.26E-29	chr:19:29855781
19	rs4398199	29866833	C	20.89	8.26E-29	chr:19:29855781
19	rs4558517	29866993	T	20.89	8.26E-29	chr:19:29855781
19	rs4605284	29866673	T	20.89	8.26E-29	chr:19:29855781
19	rs8103925	29866287	G	20.89	8.26E-29	chr:19:29855781
19	rs35900872	29871153	A	18.65	8.37E-29	chr:19:29855781
19	rs4605285	29866853	G	20.83	9.41E-29	chr:19:29855781
19	rs10422614	29835503	G	247.8	1.04E-28	chr:19:29855781
19	rs10422144	29868886	A	20.72	1.20E-28	chr:19:29855781
19	rs17624933	29867883	T	20.39	1.39E-28	chr:19:29855781
19	rs10408748	29837390	T	17.99	1.79E-28	chr:19:29855781
19	rs2193500	29834892	G	17.99	1.79E-28	chr:19:29855781
19	rs10411884	29814875	T	19.38	2.02E-28	chr:19:29855781
19	rs10411489	29846018	C	16.16	2.04E-28	chr:19:29855781
19	rs13343404	29826240	A	17.92	2.09E-28	chr:19:29855781
19	rs10419817	29828620	T	15.25	2.22E-28	chr:19:29855781
19	rs10416313	29831461	G	228.4	2.33E-28	chr:19:29855781
19	rs13345888	29842781	C	18.65	2.76E-28	chr:19:29855781
19	rs28581734	29813167	T	19.01	3.14E-28	chr:19:29855781
19	rs10405922	29813264	C	18.81	5.10E-28	chr:19:29855781
19	rs10424492	29813431	A	18.81	5.10E-28	chr:19:29855781
19	rs17773647	29813079	G	18.81	5.10E-28	chr:19:29855781
19	rs8102048	29814240	A	18.81	5.10E-28	chr:19:29855781
19	rs8113604	29813727	G	18.81	5.10E-28	chr:19:29855781
19	rs10418201	29810677	G	18.79	5.13E-28	chr:19:29855781
19	rs2111497	29822857	T	0.04176	5.17E-28	chr:19:29855781
19	rs10423930	29810687	G	18.67	5.42E-28	chr:19:29855781
19	rs74181477	29840703	A	232.9	6.40E-28	chr:19:29855781
19	rs11880160	29810859	A	18.52	8.25E-28	chr:19:29855781

19	rs8111179	29809968	C	18.52	8.25E-28	chr:19:29855781
19	rs10408576	29818131	A	18.61	9.40E-28	chr:19:29855781
19	rs10426484	29866090	A	19.65	9.51E-28	chr:19:29855781
19	rs10424375	29839089	A	18.54	1.04E-27	chr:19:29855781
19	rs11879307	29815370	A	18.54	1.04E-27	chr:19:29855781
19	rs2193499	29821859	A	18.54	1.04E-27	chr:19:29855781
19	rs7251831	29849926	G	0.03814	1.69E-27	chr:19:29855781
19	rs10410330	29827372	C	18.16	1.79E-27	chr:19:29855781
19	rs10413814	29859044	A	17.95	2.26E-27	chr:19:29855781
19	rs10413925	29851859	T	17.95	2.26E-27	chr:19:29855781
19	rs2865883	29837946	C	0.03885	2.38E-27	chr:19:29855781
19	rs1558362	29865141	T	16.13	2.39E-27	chr:19:29855781
19	rs7253279	29864696	A	16.13	2.39E-27	chr:19:29855781
19	rs13344183	29841038	A	0.03888	2.44E-27	chr:19:29855781
19	rs144395360	29843445	C	0.03902	2.72E-27	chr:19:29855781
19	rs8108111	29809851	G	16	4.95E-27	chr:19:29855781
19	rs6417157	29858422	C	6.261	5.21E-27	chr:19:29855781
19	rs12979074	29867103	A	19.87	6.48E-27	chr:19:29855781
19	rs10407026	29848625	T	0.03485	8.82E-27	chr:19:29855781
19	rs2159564	29851751	G	0.03485	8.82E-27	chr:19:29855781
19	rs2159565	29847643	G	0.03485	8.82E-27	chr:19:29855781
19	rs4804875	29853497	A	0.03485	8.82E-27	chr:19:29855781
19	rs4805373	29853498	A	0.03485	8.82E-27	chr:19:29855781
19	rs7252072	29855010	A	0.03485	8.82E-27	chr:19:29855781
19	rs10421910	29868808	A	21.02	1.14E-26	chr:19:29855781
19	rs8103862	29830747	C	0.03553	1.15E-26	chr:19:29855781
19	rs10404828	29826537	C	0.03571	1.26E-26	chr:19:29855781
19	rs10411272	29826524	T	0.03571	1.26E-26	chr:19:29855781
19	rs2865878	29825358	T	0.03571	1.26E-26	chr:19:29855781
19	rs8111162	29824369	T	0.03571	1.26E-26	chr:19:29855781
19	rs78286516	29843274	G	0.03566	1.31E-26	chr:19:29855781
19	rs78902520	29843271	T	0.03566	1.31E-26	chr:19:29855781
19	rs10853799	29821146	C	0.0357	1.34E-26	chr:19:29855781
19	rs2193498	29821753	C	0.0357	1.34E-26	chr:19:29855781
19	rs2865872	29822103	T	0.0357	1.34E-26	chr:19:29855781
19	rs2903113	29821703	C	0.0357	1.34E-26	chr:19:29855781
19	rs8113496	29820529	A	0.0357	1.34E-26	chr:19:29855781
19	rs10407922	29833571	A	0.0358	1.47E-26	chr:19:29855781
19	rs10421823	29835487	C	0.0358	1.47E-26	chr:19:29855781
19	rs8101272	29840005	G	0.0358	1.47E-26	chr:19:29855781
19	rs8105692	29840119	T	0.0358	1.47E-26	chr:19:29855781
19	rs8112567	29838763	T	0.0358	1.47E-26	chr:19:29855781
19	rs9749055	29845247	A	0.0358	1.47E-26	chr:19:29855781

19	rs7258038	29844001	A	0.03576	1.53E-26	chr:19:29855781
19	rs12978201	29805534	G	14.36	3.09E-26	chr:19:29855781
19	rs150522295	29841311	G	4.564	3.85E-26	chr:19:29855781
19	rs10415631	29810562	C	16.7	4.16E-26	chr:19:29855781
19	rs10412642	29871720	A	17.94	4.40E-26	chr:19:29855781
19	rs10420835	29856760	C	0.03161	5.64E-26	chr:19:29855781
19	rs62108213	29830870	A	293	1.92E-25	chr:19:29855781
19	rs62108214	29830872	C	293	1.92E-25	chr:19:29855781
19	rs62108215	29830873	T	293	1.92E-25	chr:19:29855781
19	rs7260198	29813253	G	0.1685	1.19E-24	chr:19:29855781
19	rs72623834	29842510	A	135.1	1.57E-24	chr:19:29855781
19	rs11878559	29814222	A	0.1703	2.36E-24	chr:19:29855781
19	rs58676754	29810034	C	0.1707	2.68E-24	chr:19:29855781
19	rs73536008	29814409	A	0.171	3.63E-24	chr:19:29855781
19	rs28888132	29845104	T	0.1757	9.01E-24	chr:19:29855781
19	rs10402522	29861084	A	4.838	4.18E-23	chr:19:29855781
19	rs10403684	29860887	T	4.838	4.18E-23	chr:19:29855781
19	rs10404520	29860937	G	4.838	4.18E-23	chr:19:29855781
19	rs10404941	29861103	C	4.838	4.18E-23	chr:19:29855781
19	rs10422443	29860582	A	4.838	4.18E-23	chr:19:29855781
19	rs10424790	29860630	G	4.838	4.18E-23	chr:19:29855781
19	rs11880983	29859865	C	4.838	4.18E-23	chr:19:29855781
19	rs11882773	29859829	A	4.838	4.18E-23	chr:19:29855781
19	rs6509385	29861469	C	4.838	4.18E-23	chr:19:29855781
19	rs11880239	29816464	T	0.1925	4.27E-23	chr:19:29855781
19	rs16962709	29817187	T	0.1925	4.27E-23	chr:19:29855781
19	rs6509388	29861882	G	4.769	6.42E-23	chr:19:29855781
19	rs12709912	29859443	C	4.753	8.35E-23	chr:19:29855781
19	rs13344917	29859503	C	4.722	9.87E-23	chr:19:29855781
19	rs4805382	29873748	A	4.915	2.42E-22	chr:19:29855781
19	rs10405131	29845482	G	415.4	2.56E-22	chr:19:29855781
19	rs9797726	29842789	T	404.6	2.88E-22	chr:19:29855781
19	rs7258057	29844039	G	413.5	3.13E-22	chr:19:29855781
19	rs35379433	29834698	A	393.2	3.87E-22	chr:19:29855781
19	rs10405226	29840658	A	388.9	4.13E-22	chr:19:29855781
19	rs740880	29878436	C	0.2346	4.19E-22	chr:19:29855781
19	rs10500238	29818674	G	0.2039	4.91E-22	chr:19:29855781
19	rs10405212	29832150	G	364.3	1.15E-21	chr:19:29855781
19	rs150191335	29843058	T	14.71	1.55E-21	chr:19:29855781
19	rs12610720	29808999	C	13.54	2.49E-21	chr:19:29855781
19	rs7508357	29841738	C	13.68	2.76E-21	chr:19:29855781
19	rs67987964	29845533	G	13.56	3.30E-21	chr:19:29855781
19	rs62104320	29866335	A	0.1928	4.71E-21	chr:19:29855781

19	rs10406127	29826758	T	13.24	5.18E-21	chr:19:29855781
19	rs1122022	29825214	A	13.24	5.18E-21	chr:19:29855781
19	rs10500239	29829980	C	13.13	5.19E-21	chr:19:29855781
19	rs1558864	29823261	T	0.193	6.25E-21	chr:19:29855781
19	rs35308361	29830552	G	0.193	6.25E-21	chr:19:29855781
19	rs929691	29818042	A	0.193	6.25E-21	chr:19:29855781
19	rs7249123	29870685	A	0.1947	6.67E-21	chr:19:29855781
19	rs2903115	29837927	C	0.1926	7.17E-21	chr:19:29855781
19	rs10404303	29860663	T	0.1937	7.41E-21	chr:19:29855781
19	rs10421211	29852874	G	0.1937	7.41E-21	chr:19:29855781
19	rs4804876	29853605	G	0.1937	7.41E-21	chr:19:29855781
19	rs61631634	29852485	A	0.1937	7.41E-21	chr:19:29855781
19	rs7248644	29856939	T	0.1937	7.41E-21	chr:19:29855781
19	rs7252679	29863745	T	0.1937	7.41E-21	chr:19:29855781
19	rs7258342	29859947	A	0.1937	7.41E-21	chr:19:29855781
19	rs7257439	29816242	C	0.194	9.26E-21	chr:19:29855781
19	rs6509383	29857637	T	0.1955	1.01E-20	chr:19:29855781
19	rs10420966	29816663	G	0.1964	1.28E-20	chr:19:29855781
19	rs8107741	29858602	T	0.1999	1.81E-20	chr:19:29855781
19	rs8107456	29810347	G	0.2	1.82E-20	chr:19:29855781
19	rs7351109	29816574	A	0.1974	2.06E-20	chr:19:29855781
19	rs7249507	29858152	T	0.191	3.85E-20	chr:19:29855781
19	rs75470832	29846159	G	0.1918	4.46E-20	chr:19:29855781
19	rs7254145	29851810	G	10.23	6.75E-20	chr:19:29855781
19	rs8107989	29867796	C	0.1981	7.02E-20	chr:19:29855781
19	rs7255472	29811968	A	0.2086	8.26E-20	chr:19:29855781
19	rs73027078;rs531011943	29845662	C	0.2002	9.32E-20	chr:19:29855781
19	rs4805374	29862430	A	0.2087	9.50E-20	chr:19:29855781
19	rs73925542	29832199	T	0.1952	1.03E-19	chr:19:29855781
19	rs8100899	29808524	A	0.2115	1.09E-19	chr:19:29855781
19	rs10413763	29873400	A	13.34	1.26E-19	chr:19:29855781
19	rs73925543	29833506	C	0.194	1.86E-19	chr:19:29855781
19	rs73925544	29839016	G	0.1991	2.05E-19	chr:19:29855781
19	rs75568982	29819020	G	0.1967	2.10E-19	chr:19:29855781
19	rs77954688	29845223	T	0.197	2.12E-19	chr:19:29855781
19	rs16962671	29810905	T	0.2016	3.07E-19	chr:19:29855781
19	rs73925540	29818545	T	0.2017	3.11E-19	chr:19:29855781
19	rs10404884	29839279	T	0.2064	3.88E-19	chr:19:29855781
19	rs73925527	29804447	A	0.197	4.11E-19	chr:19:29855781
19	rs74604731	29845371	T	0.2007	5.10E-19	chr:19:29855781
19	rs7253662	29815722	C	0.2236	1.12E-18	chr:19:29855781
19	rs7258463	29869545	A	0.1979	1.99E-18	chr:19:29855781
19	rs56792186	29862953	T	0.2044	2.42E-18	chr:19:29855781

19	rs1860573	29879749	A	7.55	2.98E-18	chr:19:29855781
19	rs12327590	29879321	T	0.2082	4.23E-18	chr:19:29855781
19	rs16962804	29876726	T	0.2574	4.53E-18	chr:19:29855781
19	rs2160717	29822729	T	3.803	5.27E-18	chr:19:29855781
19	rs17773922	29830629	C	605.6	5.92E-18	chr:19:29855781
19	rs111486428	29842638	T	605.5	5.97E-18	chr:19:29855781
19	rs140915866	29843043	G	605.5	5.97E-18	chr:19:29855781
19	rs147519792	29843490	G	605.5	5.97E-18	chr:19:29855781
19	rs148091885	29842847	A	605.5	5.97E-18	chr:19:29855781
19	rs2865888	29845818	C	605.5	5.97E-18	chr:19:29855781
19	rs62109915	29843088	T	605.5	5.97E-18	chr:19:29855781
19	rs7507405	29841956	C	605.5	5.97E-18	chr:19:29855781
19	rs7507511	29842044	T	605.5	5.97E-18	chr:19:29855781
19	rs7507537	29841906	G	605.5	5.97E-18	chr:19:29855781
19	rs7507869	29841938	C	605.5	5.97E-18	chr:19:29855781
19	rs9748962	29844809	A	605.5	5.97E-18	chr:19:29855781
19	rs10403475	29826676	C	602.4	6.24E-18	chr:19:29855781
19	rs10405069	29833324	A	602.4	6.24E-18	chr:19:29855781
19	rs10405105	29833373	T	602.4	6.24E-18	chr:19:29855781
19	rs10405177	29840592	C	602.4	6.24E-18	chr:19:29855781
19	rs10405550	29839527	C	602.4	6.24E-18	chr:19:29855781
19	rs10406530	29832689	C	602.4	6.24E-18	chr:19:29855781
19	rs10406567	29832755	C	602.4	6.24E-18	chr:19:29855781
19	rs10406707	29840277	C	602.4	6.24E-18	chr:19:29855781
19	rs10406756	29840591	T	602.4	6.24E-18	chr:19:29855781
19	rs10407234	29833261	C	602.4	6.24E-18	chr:19:29855781
19	rs10409965	29837251	T	602.4	6.24E-18	chr:19:29855781
19	rs10411073	29826427	C	602.4	6.24E-18	chr:19:29855781
19	rs10413525	29833498	C	602.4	6.24E-18	chr:19:29855781
19	rs10415713	29831541	T	602.4	6.24E-18	chr:19:29855781
19	rs10416004	29831681	T	602.4	6.24E-18	chr:19:29855781
19	rs10416725	29831649	C	602.4	6.24E-18	chr:19:29855781
19	rs10422084	29835604	G	602.4	6.24E-18	chr:19:29855781
19	rs10423486	29839393	A	602.4	6.24E-18	chr:19:29855781
19	rs10423994	29839675	A	602.4	6.24E-18	chr:19:29855781
19	rs10424081	29832472	A	602.4	6.24E-18	chr:19:29855781
19	rs10424145	29839694	T	602.4	6.24E-18	chr:19:29855781
19	rs10425325	29839190	C	602.4	6.24E-18	chr:19:29855781
19	rs10425546	29832398	T	602.4	6.24E-18	chr:19:29855781
19	rs10425705	29839737	T	602.4	6.24E-18	chr:19:29855781
19	rs10425993	29832603	G	602.4	6.24E-18	chr:19:29855781
19	rs111393122	29839832	C	602.4	6.24E-18	chr:19:29855781
19	rs13346790	29825511	A	602.4	6.24E-18	chr:19:29855781

19	rs2865884	29837991	C	602.4	6.24E-18	chr:19:29855781
19	rs35615835	29827061	A	602.4	6.24E-18	chr:19:29855781
19	rs8103034	29830764	A	602.4	6.24E-18	chr:19:29855781
19	rs8106354	29831100	A	602.4	6.24E-18	chr:19:29855781
19	rs8108995	29838275	G	602.4	6.24E-18	chr:19:29855781
19	rs8109315	29838529	C	602.4	6.24E-18	chr:19:29855781
19	rs8112403	29838791	G	602.4	6.24E-18	chr:19:29855781
19	rs10410622	29826229	G	599.1	6.60E-18	chr:19:29855781
19	rs13345551	29826197	G	599.1	6.60E-18	chr:19:29855781
19	rs10406849	29833391	A	602.5	6.99E-18	chr:19:29855781
19	rs10402726	29826292	T	587.4	8.22E-18	chr:19:29855781
19	rs10405159	29826311	G	587.4	8.22E-18	chr:19:29855781
19	rs10404259	29840188	C	575.2	8.77E-18	chr:19:29855781
19	rs10410585	29837528	A	575.9	8.86E-18	chr:19:29855781
19	rs8113733	29807306	T	0.229	9.09E-18	chr:19:29855781
19	rs73925556	29872773	A	0.2142	9.57E-18	chr:19:29855781
19	rs7249606	29866089	T	0.2783	9.72E-18	chr:19:29855781
19	rs1981678	29868519	A	0.2321	1.02E-17	chr:19:29855781
19	rs35349289	29830485	G	559.5	1.04E-17	chr:19:29855781
19	rs8107043	29864213	A	0.2804	1.25E-17	chr:19:29855781
19	rs7253602	29864638	A	0.2808	1.45E-17	chr:19:29855781
19	rs10403689	29862797	A	3.631	1.48E-17	chr:19:29855781
19	rs10423850	29862444	A	3.631	1.48E-17	chr:19:29855781
19	rs11883246	29862074	G	3.631	1.48E-17	chr:19:29855781
19	rs56048673	29862967	A	3.631	1.48E-17	chr:19:29855781
19	rs6509386	29861649	T	3.631	1.48E-17	chr:19:29855781
19	rs6509387	29861707	A	3.631	1.48E-17	chr:19:29855781
19	rs6509389	29861958	G	3.631	1.48E-17	chr:19:29855781
19	rs6509390	29862034	T	3.631	1.48E-17	chr:19:29855781
19	rs7246620	29861360	C	3.631	1.48E-17	chr:19:29855781
19	rs7248809	29863240	G	3.631	1.48E-17	chr:19:29855781
19	rs7248884	29863662	T	3.631	1.48E-17	chr:19:29855781
19	rs7252555	29863630	C	3.631	1.48E-17	chr:19:29855781
19	rs7258498	29860387	T	3.631	1.48E-17	chr:19:29855781
19	rs7259512	29862045	G	3.631	1.48E-17	chr:19:29855781
19	rs7507344	29863087	A	3.631	1.48E-17	chr:19:29855781
19	rs7507798	29863083	C	3.631	1.48E-17	chr:19:29855781
19	rs8107249	29834648	G	549.4	1.61E-17	chr:19:29855781
19	rs10404170	29863044	C	3.63	1.66E-17	chr:19:29855781
19	rs4804878	29873579	A	3.447	3.83E-17	chr:19:29855781
19	rs57598720	29911930	G	0.3001	5.75E-17	chr:19:29855781
19	rs10420190	29873997	A	5.789	7.91E-17	chr:19:29855781
19	rs111546324	29885636	A	0.2472	2.21E-16	chr:19:29855781

19	rs34786514	29878333	G	11.94	2.70E-16	chr:19:29855781
19	rs2865896	29872800	A	0.3041	5.26E-16	chr:19:29855781
19	rs4805381	29872627	T	0.3141	1.79E-15	chr:19:29855781
19	rs735285	29879116	G	0.3411	3.77E-15	chr:19:29855781
19	rs1990390	29859020	T	0.2719	4.24E-15	chr:19:29855781
19	rs58075202	29883248	A	0.2523	8.70E-15	chr:19:29855781
19	rs7258087	29869254	C	0.2903	4.86E-14	chr:19:29855781
19	rs8103504	29808196	T	0.3165	4.93E-14	chr:19:29855781
19	rs11882038	29894089	A	0.2932	1.49E-13	chr:19:29855781
19	rs10407706	29799613	T	0.2739	1.60E-13	chr:19:29855781
19	rs10407716	29799624	T	0.2739	1.60E-13	chr:19:29855781
19	rs2012095	29801230	A	0.2738	1.95E-13	chr:19:29855781
19	rs16962834	29888216	C	0.305	2.27E-13	chr:19:29855781
19	rs8102388	29863179	T	0.2989	3.74E-13	chr:19:29855781
19	rs8109517	29863857	A	0.2989	3.74E-13	chr:19:29855781
19	rs60913159	29887808	C	0.3064	3.93E-13	chr:19:29855781
19	rs7249485	29865998	C	0.3069	7.61E-13	chr:19:29855781
19	rs62109922	29852078	T	1633	1.27E-12	chr:19:29855781
19	rs4805366	29799264	A	0.2893	1.38E-12	chr:19:29855781
19	rs8105295	29846290	G	1307	3.15E-12	chr:19:29855781
19	rs8108803	29846308	G	1307	3.15E-12	chr:19:29855781
19	rs78869484	29876215	A	0.2035	3.46E-12	chr:19:29855781
19	rs74929396	29835148	T	0.2199	4.70E-12	chr:19:29855781
19	rs62104321	29866339	A	0.2996	5.72E-12	chr:19:29855781
19	rs12691091	29859430	T	0.3807	6.25E-12	chr:19:29855781
19	rs61584558	29839440	A	0.3503	1.36E-11	chr:19:29855781
19	rs114213191	29819476	G	0.2314	1.38E-11	chr:19:29855781
19	rs112652178	29839576	T	7.272	1.71E-11	chr:19:29855781
19	rs57627371	29825582	A	7.272	1.72E-11	chr:19:29855781
19	rs16962720	29822546	G	7.219	2.11E-11	chr:19:29855781
19	rs57891947	29835004	G	7.219	2.11E-11	chr:19:29855781
19	rs8103202	29823667	A	7.219	2.11E-11	chr:19:29855781
19	rs7254046	29910750	C	0.4167	2.42E-11	chr:19:29855781
19	rs7259598	29886029	C	3.101	2.97E-11	chr:19:29855781
19	rs61072957	29889358	T	0.2546	4.18E-11	chr:19:29855781
19	rs73539004	29890512	T	0.404	4.19E-11	chr:19:29855781
19	rs8107175	29864319	G	0.3477	4.42E-11	chr:19:29855781
19	rs892074	29912414	G	0.4262	5.48E-11	chr:19:29855781
19	rs957166	29882508	T	4.157	8.99E-11	chr:19:29855781
19	rs55637461	29890797	A	0.4153	1.14E-10	chr:19:29855781
19	rs11880044	29894462	C	0.3683	1.43E-10	chr:19:29855781
19	rs112201099	29831256	G	6.793	1.57E-10	chr:19:29855781
19	rs77053698	29834175	G	6.793	1.57E-10	chr:19:29855781

19	rs78222687	29826354	G	6.793	1.57E-10	chr:19:29855781
19	rs8113093	29820574	G	6.793	1.57E-10	chr:19:29855781
19	rs9749184	29845316	C	6.793	1.57E-10	chr:19:29855781
19	rs36000040	29886930	A	4.585	1.64E-10	chr:19:29855781
19	rs35698420	29893992	G	0.3701	2.02E-10	chr:19:29855781
19	rs12984061	29885279	A	2.975	2.67E-10	chr:19:29855781
19	rs4805387	29885553	C	2.975	2.67E-10	chr:19:29855781
19	rs4805389	29885643	C	2.975	2.67E-10	chr:19:29855781
19	rs4805390	29885676	G	2.975	2.67E-10	chr:19:29855781
19	rs4805391	29885783	C	2.975	2.67E-10	chr:19:29855781
19	rs35788644	29886919	G	5.082	3.36E-10	chr:19:29855781
19	rs7259050	29799035	A	2.314	3.46E-10	chr:19:29855781
19	rs16962711	29817497	C	6.186	3.57E-10	chr:19:29855781
19	rs62109920	29846656	G	6.296	4.81E-10	chr:19:29855781
19	rs4592772	29825009	T	5.617	5.15E-10	chr:19:29855781
19	rs10401849	29860785	G	0.4245	7.73E-10	chr:19:29855781
19	rs1990391	29859164	C	0.4245	7.73E-10	chr:19:29855781
19	rs73925525	29800294	A	0.2642	9.89E-10	chr:19:29855781
19	rs73537194	29888691	T	0.4387	1.02E-09	chr:19:29855781
19	rs10409760	29927476	T	2.744	1.10E-09	chr:19:29855781
19	rs10408364	29927536	A	2.743	1.20E-09	chr:19:29855781
19	rs727741	29889308	C	0.4401	1.39E-09	chr:19:29855781
19	rs2011890	29801011	A	2.79	1.42E-09	chr:19:29855781
19	rs73018534	29925518	A	0.2074	2.43E-09	chr:19:29855781
19	rs111433330	29887508	C	4.027	2.88E-09	chr:19:29855781
19	rs34023514	29887063	G	4.027	2.88E-09	chr:19:29855781
19	rs34371256	29886875	G	4.027	2.88E-09	chr:19:29855781
19	rs34859114	29887178	T	4.027	2.88E-09	chr:19:29855781
19	rs35116778	29887181	T	4.027	2.88E-09	chr:19:29855781
19	rs36004537	29886979	C	4.027	2.88E-09	chr:19:29855781
19	rs74998164	29887409	A	4.027	2.88E-09	chr:19:29855781
19	rs79593711	29886856	G	4.027	2.88E-09	chr:19:29855781
19	rs111387261	29851256	T	6.61	2.92E-09	chr:19:29855781
19	rs58278243	29848560	A	6.61	2.92E-09	chr:19:29855781
19	rs58498480	29857056	C	6.61	2.92E-09	chr:19:29855781
19	rs75402952	29850166	T	6.61	2.92E-09	chr:19:29855781
19	rs58096217	29883779	T	4.149	4.54E-09	chr:19:29855781
19	rs16962829	29886534	A	4.073	4.75E-09	chr:19:29855781
19	rs59708874	29884080	T	4.109	5.85E-09	chr:19:29855781
19	rs114338054	29884514	T	4.105	5.92E-09	chr:19:29855781
19	rs115868467	29884511	A	4.105	5.92E-09	chr:19:29855781
19	rs76868820	29884335	G	4.105	5.92E-09	chr:19:29855781
19	rs77221124	29884276	G	4.105	5.92E-09	chr:19:29855781

19	rs16962828	29886506	T	4.068	6.38E-09	chr:19:29855781
19	rs77348851	29887598	A	4.069	6.38E-09	chr:19:29855781
19	rs36032197	29813521	T	0.4549	6.57E-09	chr:19:29855781
19	rs71363971	29813517	C	0.4549	6.57E-09	chr:19:29855781
19	rs57992272	29884754	A	4.065	7.62E-09	chr:19:29855781
19	rs6509399	29874546	C	2.16	9.13E-09	chr:19:29855781
19	rs73923816	29907840	T	0.4042	1.04E-08	chr:19:29855781
19	rs78714471	29887469	T	3.905	1.05E-08	chr:19:29855781
19	rs34913300	29886724	G	4.114	1.30E-08	chr:19:29855781
19	rs10424195	29883577	T	3.401	1.55E-08	chr:19:29855781
19	rs181784529	29840974	A	0.4578	1.76E-08	chr:19:29855781
19	rs1012613	29796344	G	0.4033	1.79E-08	chr:19:29855781
19	rs17703419	29898487	G	5.969	1.81E-08	chr:19:29855781
19	rs79806042	29805218	C	5.488	1.84E-08	chr:19:29855781
19	rs10412806	29910330	A	0.4192	2.27E-08	chr:19:29855781
19	rs892073	29912294	T	0.2511	2.51E-08	chr:19:29855781
19	rs8103091	29923548	A	2.318	3.34E-08	chr:19:29855781
19	rs12150922	29879068	T	2.48	3.74E-08	chr:19:29855781
19	rs55976149	29911362	A	0.4308	4.68E-08	chr:19:29855781
19	rs10412090	30003113	T	3.433	4.80E-08	chr:19:29855781
19	rs59914048	29906011	T	0.2655	5.64E-08	chr:19:29855781
19	rs3960886	29878149	T	0.4212	6.57E-08	chr:19:29855781
19	rs13343981	29915466	G	2.22	9.76E-08	chr:19:29855781
19	rs881270	29927370	G	0.2603	1.07E-07	chr:19:29855781
19	rs10775550	29920202	G	2.216	1.09E-07	chr:19:29855781
19	rs1079335	29918435	T	2.216	1.09E-07	chr:19:29855781
19	rs1079336	29918517	G	2.216	1.09E-07	chr:19:29855781
19	rs10853808	29920041	C	2.216	1.09E-07	chr:19:29855781
19	rs10853809	29920113	T	2.216	1.09E-07	chr:19:29855781
19	rs17625222	29915445	T	2.216	1.09E-07	chr:19:29855781
19	rs4603687	29919576	T	2.216	1.09E-07	chr:19:29855781
19	rs873859	29919206	A	2.216	1.09E-07	chr:19:29855781
19	rs964314	29920424	T	2.216	1.09E-07	chr:19:29855781
19	rs6509401	29877643	T	0.4279	1.10E-07	chr:19:29855781
19	rs8109600	29877328	A	0.4279	1.10E-07	chr:19:29855781
19	rs10423905	29928853	C	2.171	1.13E-07	chr:19:29855781
19	rs10410292	29906715	A	2.284	1.18E-07	chr:19:29855781
19	rs73925555	29866763	T	0.1947	1.22E-07	chr:19:29855781
19	rs76847806	29809912	G	5.024	1.28E-07	chr:19:29855781
19	rs60623657	29792990	T	3.47	1.50E-07	chr:19:29855781
19	rs8110952	29919832	C	2.191	1.55E-07	chr:19:29855781
19	rs138628360	29881734	C	0.3982	2.47E-07	chr:19:29855781
19	rs377492537	29881785	C	0.3982	2.47E-07	chr:19:29855781

19	rs76206311	29878417	A	7.098	2.79E-07	chr:19:29855781
19	rs8107933	29919507	T	0.2792	2.85E-07	chr:19:29855781
19	rs10413487	29911836	A	2.571	2.90E-07	chr:19:29855781
19	rs10417711	29928712	T	2.1	4.22E-07	chr:19:29855781
19	rs16962822	29884508	T	3.713	4.54E-07	chr:19:29855781
19	rs62111119	29795071	T	1.92	4.67E-07	chr:19:29855781
19	rs76928648	29883569	C	3.91	4.73E-07	chr:19:29855781
19	rs2111519	29794684	C	0.4375	4.75E-07	chr:19:29855781
19	rs58785326	29911925	A	1.878	5.00E-07	chr:19:29855781
19	rs59317515	29925332	A	0.2458	5.63E-07	chr:19:29855781
19	rs12983004	29931341	G	0.4996	5.81E-07	chr:19:29855781
19	rs115818685	29921786	A	0.244	6.62E-07	chr:19:29855781
19	rs114872814	29911326	G	0.2673	6.85E-07	chr:19:29855781
19	rs79425781	29883399	A	3.827	7.00E-07	chr:19:29855781
19	rs112068505	29932295	G	0.2475	7.37E-07	chr:19:29855781
19	rs881269	29927390	A	0.3067	7.46E-07	chr:19:29855781
19	rs73539045	29928129	T	0.3439	7.53E-07	chr:19:29855781
19	rs75380460	29637459	C	0.4035	8.14E-07	chr:19:29855781
19	rs2111518	29794622	T	0.4456	9.81E-07	chr:19:29855781
19	rs77502496	29883350	C	3.756	9.88E-07	chr:19:29855781
19	rs78912590	29883341	T	3.756	9.88E-07	chr:19:29855781
19	rs79489332	29883312	C	3.756	9.88E-07	chr:19:29855781
19	rs4805384	29874653	A	7.299	1.00E-06	chr:19:29855781
19	rs11671532	29888823	C	0.5297	1.01E-06	chr:19:29855781
19	rs73539047	29928466	A	0.3507	1.06E-06	chr:19:29855781
19	rs11882502	29847717	A	0.3638	1.20E-06	chr:19:29855781
19	rs57265693	29820612	T	0.3057	1.35E-06	chr:19:29855781
19	rs73543427	29866512	C	0.3057	1.35E-06	chr:19:29855781
19	rs56269880	29907259	G	2.423	1.37E-06	chr:19:29855781
19	rs9304679	29917132	A	2.361	1.47E-06	chr:19:29855781
19	rs61643444	29824416	G	0.3031	1.48E-06	chr:19:29855781
19	rs10402892	29961630	T	2.044	1.56E-06	chr:19:29855781
19	rs10404366	29956972	C	2.044	1.56E-06	chr:19:29855781
19	rs10404529	29956983	A	2.044	1.56E-06	chr:19:29855781
19	rs11667306	29879868	C	1.941	1.82E-06	chr:19:29855781
19	rs11880642	29847581	G	0.3699	1.88E-06	chr:19:29855781
19	rs11882449	29847512	A	0.3699	1.88E-06	chr:19:29855781
19	rs11882621	29847571	T	0.3699	1.88E-06	chr:19:29855781
19	rs59406130	29850574	G	0.3699	1.88E-06	chr:19:29855781
19	rs7259246	29853762	T	0.3699	1.88E-06	chr:19:29855781
19	rs73541380	29853023	T	0.3699	1.88E-06	chr:19:29855781
19	rs73542048	29849389	A	0.3699	1.88E-06	chr:19:29855781
19	rs73542050	29849800	A	0.3699	1.88E-06	chr:19:29855781

19	rs73542053	29850273	T	0.3699	1.88E-06	chr:19:29855781
19	rs73542056	29851283	T	0.3699	1.88E-06	chr:19:29855781
19	rs73542063	29851794	C	0.3699	1.88E-06	chr:19:29855781
19	rs8105384	29854818	T	0.3699	1.88E-06	chr:19:29855781
19	rs77445168	29803772	T	9.522	1.97E-06	chr:19:29855781
19	rs73534196	29809176	A	0.3152	2.07E-06	chr:19:29855781
19	rs61680354	29922071	A	0.4683	2.23E-06	chr:19:29855781
19	rs8107813	29919427	A	0.4683	2.23E-06	chr:19:29855781
19	rs9676501	29918545	T	0.4683	2.23E-06	chr:19:29855781
19	rs9676571	29915029	T	0.4683	2.23E-06	chr:19:29855781
19	rs112194147	29837534	A	0.313	2.30E-06	chr:19:29855781
19	rs10409972	29905297	C	2.322	2.45E-06	chr:19:29855781
19	rs114483161	29881250	G	4.888	2.48E-06	chr:19:29855781
19	rs114911433	29881069	C	4.888	2.48E-06	chr:19:29855781
19	rs59336688	29841432	G	0.3103	2.51E-06	chr:19:29855781
19	rs114506886	29856995	C	0.3784	2.52E-06	chr:19:29855781
19	rs115392433	29856997	C	0.3784	2.52E-06	chr:19:29855781
19	rs116708338	29856993	C	0.3784	2.52E-06	chr:19:29855781
19	rs73541383	29855391	G	0.3784	2.52E-06	chr:19:29855781
19	rs73541385	29855644	A	0.3784	2.52E-06	chr:19:29855781
19	rs73541386	29856702	G	0.3784	2.52E-06	chr:19:29855781
19	rs73541387	29856831	G	0.3784	2.52E-06	chr:19:29855781
19	rs73541390	29856897	G	0.3784	2.52E-06	chr:19:29855781
19	rs74670598	29856961	C	0.3784	2.52E-06	chr:19:29855781
19	rs78225345	29856430	A	0.3784	2.52E-06	chr:19:29855781
19	rs79412487	29856954	T	0.3784	2.52E-06	chr:19:29855781
19	rs73923820	29914440	T	0.4699	2.81E-06	chr:19:29855781
19	rs55782117	29830846	C	0.3065	2.92E-06	chr:19:29855781
19	rs77841426	29878776	A	6.674	2.96E-06	chr:19:29855781
19	rs61282357	29850798	T	0.38	3.13E-06	chr:19:29855781
19	rs61094332	29809681	A	0.3742	3.14E-06	chr:19:29855781
19	rs73534200	29810221	A	0.3227	3.45E-06	chr:19:29855781
19	rs114182535	29812567	T	4.66	3.73E-06	chr:19:29855781
19	rs74880260	29799563	A	3.84	4.39E-06	chr:19:29855781
19	rs10422964	30007626	G	2.333	5.04E-06	chr:19:29855781
19	rs11880396	29803819	T	0.3845	5.21E-06	chr:19:29855781
19	rs73534194	29808022	T	0.3845	5.21E-06	chr:19:29855781
19	rs73539063	29932080	G	0.3762	5.42E-06	chr:19:29855781
19	rs11882858	29895527	T	3.761	5.68E-06	chr:19:29855781
19	rs8106906	29894204	C	3.761	5.68E-06	chr:19:29855781
19	rs76017112	29672619	A	0.4107	6.29E-06	chr:19:29855781
19	rs76420107	29672658	T	0.4107	6.29E-06	chr:19:29855781
19	rs73539049	29928755	G	0.3919	6.57E-06	chr:19:29855781

19	rs73534191	29805756	C	0.3921	7.39E-06	chr:19:29855781
19	rs11668561	29886533	T	0.2491	8.51E-06	chr:19:29855781
19	rs78081748	29883523	G	2.999	9.21E-06	chr:19:29855781
19	rs4444446	29732702	T	2.251	1.06E-05	chr:19:29855781
19	rs59431805	29836706	C	0.4069	1.24E-05	chr:19:29855781
19	rs73542021	29833539	T	0.4069	1.24E-05	chr:19:29855781
19	rs73542025	29836159	A	0.4069	1.24E-05	chr:19:29855781
19	rs73542028	29836591	C	0.4069	1.24E-05	chr:19:29855781
19	rs74477241	29835720	G	0.4069	1.24E-05	chr:19:29855781
19	rs77977515	29835434	G	0.4069	1.24E-05	chr:19:29855781
19	rs8102327	29840232	T	0.4069	1.24E-05	chr:19:29855781
19	rs8103129	29840525	G	0.4069	1.24E-05	chr:19:29855781
19	rs116030630	29673309	T	0.42	1.27E-05	chr:19:29855781
19	rs75040595	29806368	A	4.046	1.29E-05	chr:19:29855781
19	rs58623111	29801673	A	0.4369	1.33E-05	chr:19:29855781
19	rs112905684	29864870	A	0.3311	1.34E-05	chr:19:29855781
19	rs113678597	29864992	T	0.3311	1.34E-05	chr:19:29855781
19	rs7253694	29858529	C	0.545	1.68E-05	chr:19:29855781
19	rs116423283	29666952	G	0.4329	1.71E-05	chr:19:29855781
19	rs77810432	29647026	T	0.4329	1.71E-05	chr:19:29855781
19	rs79351088	29660319	T	0.4329	1.71E-05	chr:19:29855781
19	rs80139316	29637756	T	0.4329	1.71E-05	chr:19:29855781
19	rs4545933	30005861	G	0.5867	1.72E-05	chr:19:29855781
19	rs112488290	29844372	G	0.4145	1.74E-05	chr:19:29855781
19	rs139736193	29843907	T	0.4145	1.74E-05	chr:19:29855781
19	rs142442997	29843149	T	0.4145	1.74E-05	chr:19:29855781
19	rs28767801	29845057	G	0.4145	1.74E-05	chr:19:29855781
19	rs73542039	29844055	A	0.4145	1.74E-05	chr:19:29855781
19	rs149724344	29813571	A	6.536	1.80E-05	chr:19:29855781
19	rs59806259	29872263	T	0.4619	1.90E-05	chr:19:29855781
19	rs58831774	29823559	T	0.4148	1.97E-05	chr:19:29855781
19	rs73540021	29822304	T	0.4148	1.97E-05	chr:19:29855781
19	rs73540047	29827049	G	0.4148	1.97E-05	chr:19:29855781
19	rs111857853	387614	G	2.943	4.07E-10	chr:19:386675
19	rs144524516	388358	G	2.908	2.37E-09	chr:19:386675
19	rs148483422	388359	G	2.908	2.37E-09	chr:19:386675
19	rs73916946	387864	C	0.3813	2.32E-08	chr:19:386675
19	rs62102076	389134	C	0.3997	8.88E-08	chr:19:386675
19	rs55749078	387847	T	0.4299	6.92E-07	chr:19:386675
19	rs62102075	389088	C	2.217	1.09E-06	chr:19:386675
19	rs112912797	389216	C	0.4256	1.42E-06	chr:19:386675
19	rs79881403	389217	A	0.4256	1.42E-06	chr:19:386675
19	rs369380681	388030	C	2.173	3.13E-06	chr:19:386675

19	rs78540374	389290	T	2.103	3.50E-06	chr:19:386675
19	rs529560773	388349	C	0.4482	4.57E-06	chr:19:386675
19	rs549371497	388347	C	0.4482	4.57E-06	chr:19:386675
19	rs562006336	388348	T	0.4482	4.57E-06	chr:19:386675
19	rs77887742	389289	T	2.061	6.02E-06	chr:19:386675
19	rs111688272	388012	A	0.4194	8.66E-06	chr:19:386675
19	rs200520198	387268	A	0.4642	1.83E-05	chr:19:386675
19	rs62102074	389061	A	0.4434	2.95E-05	chr:19:386675
19	rs28462149	52925088	G	2.769	2.68E-14	chr:19:52924209
19	rs28375755	52925067	A	2.742	3.96E-14	chr:19:52924209
19	rs73934459	52924049	G	2.698	1.15E-13	chr:19:52924209
19	rs112694418	52924249	C	2.73	1.27E-13	chr:19:52924209
19	rs73934458	52924044	G	2.713	1.34E-13	chr:19:52924209
19	rs28528760	52924984	C	2.639	1.75E-13	chr:19:52924209
19	rs113590683	52924340	G	2.621	1.87E-13	chr:19:52924209
19	rs59950484	52924130	A	0.3837	2.09E-13	chr:19:52924209
19	rs61689498	52924136	G	0.3837	2.09E-13	chr:19:52924209
19	rs111240956	52924334	C	2.687	2.55E-13	chr:19:52924209
19	rs59318859	52924127	C	0.3917	7.49E-13	chr:19:52924209
19	rs73934456	52923808	G	2.626	9.12E-13	chr:19:52924209
19	rs558610644	52924875	T	2.557	2.16E-12	chr:19:52924209
19	rs60439793	52923753	C	2.548	2.27E-12	chr:19:52924209
19	rs73934457	52923971	G	2.548	2.27E-12	chr:19:52924209
19	rs369961637	52924884	A	2.529	3.35E-12	chr:19:52924209
19	rs371913657	52924859	A	2.529	3.35E-12	chr:19:52924209
19	rs7247111	52925118	G	0.3975	8.29E-12	chr:19:52924209
19	rs16983796	52925179	A	2.464	1.05E-11	chr:19:52924209
19	rs4085090	52924507	T	2.416	1.13E-11	chr:19:52924209
19	rs12461528	52925285	C	2.439	1.65E-11	chr:19:52924209
19	rs56902760	52924208	G	0.3993	1.71E-11	chr:19:52924209
19	rs55952958	52923189	G	0.4236	5.80E-11	chr:19:52924209
19	rs56236739	52923275	T	0.4236	5.80E-11	chr:19:52924209
19	rs548489689	52924755	T	2.112	2.79E-08	chr:19:52924209
19	rs567005277	52924766	C	2.112	2.79E-08	chr:19:52924209
19	rs146678829	52924581	C	1.957	1.27E-07	chr:19:52924209
19	rs572684466	52924733	T	2.043	4.51E-07	chr:19:52924209
19	rs324091	52905067	C	0.5099	3.35E-06	chr:19:52924209
19	rs569677324	52924640	T	1.941	7.42E-06	chr:19:52924209
19	rs140283810	52924629	G	1.922	8.75E-06	chr:19:52924209
19	rs375010966	52924628	C	1.922	8.75E-06	chr:19:52924209
19	rs8106133	52921057	G	0.4741	5.02E-05	chr:19:52924209
19	rs111934070	58038908	G	72.68	9.85E-20	chr:19:57996939
19	rs112656982	58040171	T	37.09	3.48E-18	chr:19:57996939

19	rs186701804	58041008	G	37.09	3.48E-18	chr:19:57996939
19	rs199529440	57959782	A	72.9	9.84E-18	chr:19:57996939
19	rs139217049	57957290	A	68.72	1.14E-17	chr:19:57996939
19	rs142468478	57959266	A	68.72	1.14E-17	chr:19:57996939
19	rs114548890	58044044	A	32.25	3.01E-17	chr:19:57996939
19	rs193080271	58011847	G	126.6	7.79E-17	chr:19:57996939
19	rs113149928	57962253	C	28.31	1.15E-16	chr:19:57996939
19	rs114280019	58046047	T	27.02	1.46E-16	chr:19:57996939
19	rs75911968	58049368	C	27.02	1.46E-16	chr:19:57996939
19	rs76307110	58054737	C	27.02	1.46E-16	chr:19:57996939
19	rs79588559	58047419	T	27.02	1.46E-16	chr:19:57996939
19	rs115056216	58045466	C	26.76	2.00E-16	chr:19:57996939
19	rs150316756	58039634	G	26.27	2.02E-16	chr:19:57996939
19	rs529767732	57959794	T	25.5	8.71E-16	chr:19:57996939
19	rs111887346	58047732	C	21.57	1.48E-15	chr:19:57996939
19	rs142572042	57964713	G	17.26	3.40E-15	chr:19:57996939
19	rs7249326	57963238	G	17.26	3.40E-15	chr:19:57996939
19	rs2240037	57955719	A	21.87	6.12E-15	chr:19:57996939
19	rs141683327	57999717	G	23.06	9.12E-15	chr:19:57996939
19	rs76997017	57966578	C	14.37	2.32E-14	chr:19:57996939
19	rs117470965	58003359	T	11.65	3.27E-14	chr:19:57996939
19	rs2240038	57955729	A	15.45	1.62E-13	chr:19:57996939
19	rs2240039	57955971	T	15.15	2.32E-13	chr:19:57996939
19	rs78257981	58038933	C	16.6	6.15E-13	chr:19:57996939
19	rs144254809	58048573	A	24.27	2.10E-12	chr:19:57996939
19	rs58718038	57960628	T	8.515	5.19E-12	chr:19:57996939
19	rs560805565	57959791	T	36.44	5.87E-12	chr:19:57996939
19	rs151275866	57961406	T	60.23	1.74E-10	chr:19:57996939
19	rs143367206	58039265	T	22.15	3.01E-10	chr:19:57996939
19	rs116713673	57995726	G	80.4	6.24E-10	chr:19:57996939
19	rs139765863	57986056	C	80.4	6.24E-10	chr:19:57996939
19	rs140647909	57988960	A	80.4	6.24E-10	chr:19:57996939
19	rs568624310	57982399	T	80.4	6.24E-10	chr:19:57996939
19	rs74442817	58055907	C	11.47	9.58E-10	chr:19:57996939
19	rs12976560	57990916	C	4.792	1.01E-09	chr:19:57996939
19	rs12984815	57990569	C	4.792	1.01E-09	chr:19:57996939
19	rs12985337	57982983	C	4.792	1.01E-09	chr:19:57996939
19	rs2074066	57988396	T	4.792	1.01E-09	chr:19:57996939
19	rs34837812	57990115	G	4.792	1.01E-09	chr:19:57996939
19	rs35859595	57979815	A	4.792	1.01E-09	chr:19:57996939
19	rs7257644	57985889	C	4.792	1.01E-09	chr:19:57996939
19	rs139637370	58015322	C	144.1	1.54E-09	chr:19:57996939
19	rs144872330	58052509	A	20.37	1.91E-09	chr:19:57996939

19	rs137980030	58030474	A	75.92	1.96E-09	chr:19:57996939
19	rs115366794	58057807	T	13.33	2.00E-09	chr:19:57996939
19	rs558258289	58010894	A	85.21	2.32E-09	chr:19:57996939
19	rs578220955	58010895	T	85.21	2.32E-09	chr:19:57996939
19	rs7254867	57989075	T	4.388	4.13E-09	chr:19:57996939
19	rs181196402	58055230	T	52.42	7.29E-09	chr:19:57996939
19	rs76623573	58040781	A	6.891	7.78E-09	chr:19:57996939
19	rs116069926	58042529	T	6.863	8.48E-09	chr:19:57996939
19	rs114738978	58103550	C	45.06	1.05E-08	chr:19:57996939
19	rs140265363	58111979	G	45.06	1.05E-08	chr:19:57996939
19	rs140453879	58074643	C	45.06	1.05E-08	chr:19:57996939
19	rs144851961	58067894	C	45.06	1.05E-08	chr:19:57996939
19	rs144917593	57987973	T	45.06	1.05E-08	chr:19:57996939
19	rs146388366	58103549	G	45.06	1.05E-08	chr:19:57996939
19	rs147657507	58084837	C	45.06	1.05E-08	chr:19:57996939
19	rs149472061	58103008	C	45.06	1.05E-08	chr:19:57996939
19	rs182411760	58059723	C	45.06	1.05E-08	chr:19:57996939
19	rs185241277	58097224	G	45.06	1.05E-08	chr:19:57996939
19	rs189139501	58085347	A	45.06	1.05E-08	chr:19:57996939
19	rs190672375	58104655	A	45.06	1.05E-08	chr:19:57996939
19	rs538973691	58090499	G	45.06	1.05E-08	chr:19:57996939
19	rs558702226	58068436	T	45.06	1.05E-08	chr:19:57996939
19	rs74451589	57987696	G	45.06	1.05E-08	chr:19:57996939
19	rs75462744	57987494	T	45.06	1.05E-08	chr:19:57996939
19	rs7259134	58062311	T	35.15	1.14E-08	chr:19:57996939
19	rs112258496	58044432	T	71.86	1.58E-08	chr:19:57996939
19	rs115113770	58044725	A	71.86	1.58E-08	chr:19:57996939
19	rs190095598	58052166	T	71.86	1.58E-08	chr:19:57996939
19	rs111373384	58065183	A	23.01	2.70E-08	chr:19:57996939
19	rs147266930	58068257	C	23.01	2.70E-08	chr:19:57996939
19	rs1548476	58058536	A	4.211	2.92E-08	chr:19:57996939
19	rs141763996	58052042	C	6.009	3.38E-08	chr:19:57996939
19	rs111637349	58070101	C	20.46	3.60E-08	chr:19:57996939
19	rs115123894	58114634	A	14.88	3.62E-08	chr:19:57996939
19	rs112656017	58116334	G	7.006	4.10E-08	chr:19:57996939
19	rs137934258	58069247	A	91.53	7.88E-08	chr:19:57996939
19	rs141955887	58085765	T	91.53	7.88E-08	chr:19:57996939
19	rs193172049	57974021	C	91.53	7.88E-08	chr:19:57996939
19	rs113721107	58056083	A	9.584	8.47E-08	chr:19:57996939
19	rs12978765	57970368	G	3.668	1.10E-07	chr:19:57996939
19	rs191182363	58026207	A	91.25	1.32E-07	chr:19:57996939
19	rs10425274	57995784	T	4.053	2.01E-07	chr:19:57996939
19	rs4801225	57993186	A	4.053	2.01E-07	chr:19:57996939

19	rs6510078	57997218	T	4.053	2.01E-07	chr:19:57996939
19	rs7255357	57998012	C	4.053	2.06E-07	chr:19:57996939
19	rs3810132	57998879	C	4.053	2.07E-07	chr:19:57996939
19	rs115762769	58069603	C	5.818	3.72E-07	chr:19:57996939
19	rs144856611	58109979	C	11.61	9.20E-07	chr:19:57996939
19	rs147006795	58139866	T	10.87	1.20E-06	chr:19:57996939
19	rs6510070	57961612	A	0.2595	1.50E-06	chr:19:57996939
19	rs148982903	58021448	A	56.2	1.53E-06	chr:19:57996939
19	rs180863935	58029394	T	176.2	3.45E-06	chr:19:57996939
19	rs537906242	58036834	G	176.2	3.45E-06	chr:19:57996939
19	rs569344648	58036832	A	176.2	3.45E-06	chr:19:57996939
19	rs67586127	57969810	G	3.037	4.05E-06	chr:19:57996939
19	rs12981785	57965453	T	2.985	4.42E-06	chr:19:57996939
19	rs145749832	58060826	T	7.169	4.89E-06	chr:19:57996939
19	rs8109760	57968550	T	2.96	5.01E-06	chr:19:57996939
19	rs368071146	58011500	T	154	5.21E-06	chr:19:57996939
19	rs7254946	57964491	C	2.945	5.51E-06	chr:19:57996939
19	rs75763025	58231575	G	4.347	5.78E-06	chr:19:57996939
19	rs114686987	58069658	T	61.35	5.81E-06	chr:19:57996939
19	rs115397199	58099510	T	61.35	5.81E-06	chr:19:57996939
19	rs115443337	58004644	G	61.35	5.81E-06	chr:19:57996939
19	rs143191422	58066185	A	61.35	5.81E-06	chr:19:57996939
19	rs150631932	58041883	T	61.35	5.81E-06	chr:19:57996939
19	rs151014926	58016099	A	61.35	5.81E-06	chr:19:57996939
19	rs181200370	58085770	A	61.35	5.81E-06	chr:19:57996939
19	rs528301114	58059696	C	61.35	5.81E-06	chr:19:57996939
19	rs556667965	58027910	C	61.35	5.81E-06	chr:19:57996939
19	rs561682422	58036530	G	61.35	5.81E-06	chr:19:57996939
19	rs7245637	58036125	G	61.35	5.81E-06	chr:19:57996939
19	rs7247851	58091247	C	61.35	5.81E-06	chr:19:57996939
19	rs80005447	58086009	A	61.35	5.81E-06	chr:19:57996939
19	rs8110884	58035519	A	61.35	5.81E-06	chr:19:57996939
19	rs8112653	58032436	C	61.35	5.81E-06	chr:19:57996939
19	rs140933142	58073972	A	8.181	6.08E-06	chr:19:57996939
19	rs12973393	57971753	G	2.935	6.18E-06	chr:19:57996939
19	rs12974160	57971398	G	2.935	6.18E-06	chr:19:57996939
19	rs12975239	57971803	C	2.935	6.18E-06	chr:19:57996939
19	rs12979514	57971812	G	2.935	6.18E-06	chr:19:57996939
19	rs12981036	57972474	G	2.935	6.18E-06	chr:19:57996939
19	rs12981524	57971878	A	2.935	6.18E-06	chr:19:57996939
19	rs67307910	57999846	A	0.2819	7.20E-06	chr:19:57996939
19	rs140239177	58079063	G	14.65	8.19E-06	chr:19:57996939
19	rs188500682	58088656	A	14.65	8.19E-06	chr:19:57996939

19	rs189728557	58066926	G	14.65	8.19E-06	chr:19:57996939
19	rs79747118	58060141	T	14.65	8.19E-06	chr:19:57996939
19	rs9676252	58117022	C	5.914	8.57E-06	chr:19:57996939
19	rs2240022	58038624	C	3.115	8.71E-06	chr:19:57996939
19	rs2051765	58035645	C	2.958	9.83E-06	chr:19:57996939
19	rs2051766	58035875	G	2.958	9.83E-06	chr:19:57996939
19	rs56076852	58035509	T	2.958	9.83E-06	chr:19:57996939
19	rs114688947	58130706	A	25.78	1.11E-05	chr:19:57996939
19	rs116193782	58130373	T	25.78	1.11E-05	chr:19:57996939
19	rs139480699	58121695	T	25.78	1.11E-05	chr:19:57996939
19	rs190588533	58136346	C	25.78	1.11E-05	chr:19:57996939
19	rs528742160	58123196	T	25.78	1.11E-05	chr:19:57996939
19	rs566246472	58123195	T	25.78	1.11E-05	chr:19:57996939
19	rs80323195	58128932	C	25.78	1.11E-05	chr:19:57996939
19	rs9304794	58129725	G	25.78	1.11E-05	chr:19:57996939
19	rs9676620	58124289	A	25.78	1.11E-05	chr:19:57996939
19	rs559349729	58149392	A	6.252	1.21E-05	chr:19:57996939
19	rs112485279	58084911	T	6.532	1.22E-05	chr:19:57996939
19	rs55903117	58019583	G	2.879	1.42E-05	chr:19:57996939
19	rs71333245	58003141	A	2.956	1.43E-05	chr:19:57996939
19	rs2106824	58028152	G	2.875	1.49E-05	chr:19:57996939
19	rs56366060	58019482	C	2.875	1.49E-05	chr:19:57996939
19	rs56796260	58020259	C	2.875	1.49E-05	chr:19:57996939
19	rs2360755	58025922	C	2.873	1.53E-05	chr:19:57996939
19	rs9676259	58117677	A	5.637	1.60E-05	chr:19:57996939
19	rs10409092	58009514	A	2.881	2.16E-05	chr:19:57996939
19	rs10409635	58009510	G	2.881	2.16E-05	chr:19:57996939
19	rs10221501	58006675	G	2.878	2.24E-05	chr:19:57996939
19	rs10221524	58006952	G	2.878	2.24E-05	chr:19:57996939
19	rs10853895	58008450	G	2.878	2.24E-05	chr:19:57996939
19	rs12978437	58007453	C	2.878	2.24E-05	chr:19:57996939
19	rs12979154	58008956	C	2.878	2.24E-05	chr:19:57996939
19	rs2074074	58003873	C	2.878	2.24E-05	chr:19:57996939
19	rs2074076	58004346	G	2.878	2.24E-05	chr:19:57996939
19	rs2360761	58007785	A	2.878	2.24E-05	chr:19:57996939
19	rs4801490	58006239	A	2.878	2.24E-05	chr:19:57996939
19	rs58155371	58009212	T	2.878	2.24E-05	chr:19:57996939
19	rs6510084	58002665	A	2.878	2.24E-05	chr:19:57996939
19	rs6510085	58002819	T	2.878	2.24E-05	chr:19:57996939
19	rs7248267	58005668	C	2.878	2.24E-05	chr:19:57996939
19	rs12461717	58009020	A	2.877	2.30E-05	chr:19:57996939
19	rs2074071	58003580	A	2.876	2.33E-05	chr:19:57996939
19	rs2074072	58003613	C	2.876	2.33E-05	chr:19:57996939

19	rs2074073	58003728	G	2.876	2.33E-05	chr:19:57996939
19	rs2074075	58004158	C	2.876	2.33E-05	chr:19:57996939
19	rs2074077	58004931	A	2.876	2.33E-05	chr:19:57996939
19	rs7253260	58001096	G	2.876	2.36E-05	chr:19:57996939
19	rs368257628	58121788	A	3.834	2.40E-05	chr:19:57996939
19	rs12460048	58041703	C	2.886	2.49E-05	chr:19:57996939
19	rs545252681	58375359	T	32.51	2.54E-05	chr:19:57996939
19	rs73573431	58044998	C	6.061	2.63E-05	chr:19:57996939
19	rs12609704	58060538	T	2.927	2.67E-05	chr:19:57996939
19	rs150151592	58140290	G	11.16	2.75E-05	chr:19:57996939
19	rs2019475	57999738	G	2.924	2.76E-05	chr:19:57996939
19	rs573268019	58245334	C	6.113	2.82E-05	chr:19:57996939
19	rs61138724	58081065	C	2.779	3.04E-05	chr:19:57996939
19	rs111377500	58078770	T	6.453	3.24E-05	chr:19:57996939
19	rs138572320	58071615	G	6.453	3.24E-05	chr:19:57996939
19	rs139218209	58076804	A	6.453	3.24E-05	chr:19:57996939
19	rs144124750	58078046	T	6.453	3.24E-05	chr:19:57996939
1	rs1613391	106042790	T	55.49	7.27E-34	chr:1:106015875
1	rs115832392	106074857	T	50.05	4.01E-33	chr:1:106015875
1	rs116494820	106075693	T	50.05	4.01E-33	chr:1:106015875
1	rs1710796	106078422	T	50.05	4.01E-33	chr:1:106015875
1	rs11801857	106080328	A	47.96	5.35E-33	chr:1:106015875
1	rs1710794	106080269	A	47.96	5.35E-33	chr:1:106015875
1	rs1721566	106072947	G	43.72	7.03E-33	chr:1:106015875
1	rs1710788	106072791	T	46.58	7.94E-33	chr:1:106015875
1	rs116125878	106075962	A	45.14	8.69E-33	chr:1:106015875
1	rs1710777	106081969	G	47.43	9.96E-33	chr:1:106015875
1	rs777065	106084022	A	47.43	9.96E-33	chr:1:106015875
1	rs1850246	106068484	A	75.92	1.19E-32	chr:1:106015875
1	rs1623730	106079717	G	40.96	1.67E-32	chr:1:106015875
1	rs2501604	106000162	T	67.91	1.82E-32	chr:1:106015875
1	rs1710792	106071859	T	74.74	1.89E-32	chr:1:106015875
1	rs550158236	106064983	C	74.74	1.89E-32	chr:1:106015875
1	rs777098	106056842	C	74.74	1.89E-32	chr:1:106015875
1	rs777093	106060811	C	73.56	3.28E-32	chr:1:106015875
1	rs1971395	106038763	C	852.6	4.90E-31	chr:1:106015875
1	rs1516147	106004541	T	19.03	9.44E-30	chr:1:106015875
1	rs1516148	106004460	A	19.03	9.44E-30	chr:1:106015875
1	rs2222088	106008245	G	19.09	1.22E-29	chr:1:106015875
1	rs1516162	106028919	G	1555	1.61E-29	chr:1:106015875
1	rs1710769	106032610	A	1555	1.61E-29	chr:1:106015875
1	rs1710771	106032381	G	1555	1.61E-29	chr:1:106015875
1	rs151171804	106020156	T	43.74	3.56E-28	chr:1:106015875

1	rs777086	106022269	C	43.93	3.70E-28	chr:1:106015875
1	rs2502907	106018534	A	45.61	3.89E-28	chr:1:106015875
1	rs1721516	106028315	G	180.9	2.37E-27	chr:1:106015875
1	rs991009	106027353	A	180.9	2.37E-27	chr:1:106015875
1	rs2501579	105974218	C	20.75	2.05E-26	chr:1:106015875
1	rs115558666	105962319	G	17.68	8.25E-26	chr:1:106015875
1	rs2459321	105964755	A	17.68	8.25E-26	chr:1:106015875
1	rs12096040	106070032	C	776.9	3.05E-25	chr:1:106015875
1	rs1721541	106065836	T	776.9	3.05E-25	chr:1:106015875
1	rs2459330	106069314	T	776.9	3.05E-25	chr:1:106015875
1	rs6691386	106068934	G	776.9	3.05E-25	chr:1:106015875
1	rs79131044	106070515	T	776.9	3.05E-25	chr:1:106015875
1	rs116577727	105985398	G	11.76	2.98E-24	chr:1:106015875
1	rs2459319	106048674	C	560	1.97E-23	chr:1:106015875
1	rs11579317	106034326	A	20.63	2.23E-23	chr:1:106015875
1	rs12733456	106034035	G	20.63	2.23E-23	chr:1:106015875
1	rs12733465	106034046	G	20.63	2.23E-23	chr:1:106015875
1	rs1516154	106034669	A	20.63	2.23E-23	chr:1:106015875
1	rs1516155	106034597	G	20.63	2.23E-23	chr:1:106015875
1	rs1524123	106034389	A	20.63	2.23E-23	chr:1:106015875
1	rs1949338	106035398	A	20.63	2.23E-23	chr:1:106015875
1	rs2203575	106033671	T	20.63	2.23E-23	chr:1:106015875
1	rs2203576	106033660	C	20.63	2.23E-23	chr:1:106015875
1	rs2880133	106023408	C	19.79	4.77E-23	chr:1:106015875
1	rs1916824	106024122	G	21.7	9.91E-23	chr:1:106015875
1	rs1516163	106029319	T	24.51	1.50E-22	chr:1:106015875
1	rs1516164	106029434	T	24.51	1.50E-22	chr:1:106015875
1	rs2203577	106025524	A	24.51	1.50E-22	chr:1:106015875
1	rs4847045	106026809	A	24.51	1.50E-22	chr:1:106015875
1	rs11184582	106037458	C	6.685	2.59E-22	chr:1:106015875
1	rs11184583	106037459	C	6.685	2.59E-22	chr:1:106015875
1	rs11184581	106037417	G	6.688	2.71E-22	chr:1:106015875
1	rs12084140	106036483	C	6.688	2.71E-22	chr:1:106015875
1	rs1404034	106037161	G	6.688	2.71E-22	chr:1:106015875
1	rs1404036	106036839	C	6.688	2.71E-22	chr:1:106015875
1	rs28431449	106036281	C	6.688	2.71E-22	chr:1:106015875
1	rs28473029	106036486	A	6.688	2.71E-22	chr:1:106015875
1	rs12563882	106036145	A	6.685	3.11E-22	chr:1:106015875
1	rs2341710	106032936	C	28.13	3.69E-22	chr:1:106015875
1	rs9651162	106032690	G	27.8	5.42E-22	chr:1:106015875
1	rs1613491	106042753	T	25.29	9.31E-22	chr:1:106015875
1	rs2203573	106008029	C	7.95	7.27E-21	chr:1:106015875
1	rs2203574	106008091	G	7.996	8.16E-21	chr:1:106015875

1	rs7546059	106035197	T	5.634	8.67E-21	chr:1:106015875
1	rs777103	106054535	A	1319	1.02E-20	chr:1:106015875
1	rs2501587	105959269	T	8.247	7.67E-20	chr:1:106015875
1	rs12037064	106048945	T	6.337	2.95E-19	chr:1:106015875
1	rs1400100	106053322	A	6.337	2.95E-19	chr:1:106015875
1	rs1721531	106045217	A	6.337	2.95E-19	chr:1:106015875
1	rs1721533	106043860	A	6.337	2.95E-19	chr:1:106015875
1	rs2255422	106052011	C	6.337	2.95E-19	chr:1:106015875
1	rs2459318	106048663	C	6.337	2.95E-19	chr:1:106015875
1	rs2501598	106038410	G	6.337	2.95E-19	chr:1:106015875
1	rs777099	106056576	A	6.337	2.95E-19	chr:1:106015875
1	rs777102	106054543	T	6.337	2.95E-19	chr:1:106015875
1	rs1710772	106058864	A	6.146	4.23E-19	chr:1:106015875
1	rs12045534	106064926	A	5.965	7.67E-19	chr:1:106015875
1	rs1721542	106066105	A	5.965	7.67E-19	chr:1:106015875
1	rs2090336	106068107	T	5.965	7.67E-19	chr:1:106015875
1	rs2501596	106066693	G	5.965	7.67E-19	chr:1:106015875
1	rs777095	106063610	A	5.965	7.67E-19	chr:1:106015875
1	rs1721556	106081420	G	5.319	9.51E-19	chr:1:106015875
1	rs777070	106087275	T	8.526	1.06E-18	chr:1:106015875
1	rs1710790	106072410	G	5.921	1.36E-18	chr:1:106015875
1	rs10458526	106020558	G	0.2215	2.51E-16	chr:1:106015875
1	rs10458527	106020963	A	0.2213	2.66E-16	chr:1:106015875
1	rs6701452	106017505	T	0.245	6.68E-16	chr:1:106015875
1	rs2459316	106009279	G	4.548	2.15E-15	chr:1:106015875
1	rs2502867	106009415	C	4.558	2.23E-15	chr:1:106015875
1	rs2502869	106009370	T	4.285	1.04E-14	chr:1:106015875
1	rs6698864	105974470	C	4.136	1.16E-14	chr:1:106015875
1	rs951064	105975229	A	4.136	1.16E-14	chr:1:106015875
1	rs1160236	105958462	A	4.1	1.65E-14	chr:1:106015875
1	rs1516149	105956749	A	4.1	1.65E-14	chr:1:106015875
1	rs1516150	105956591	A	4.1	1.65E-14	chr:1:106015875
1	rs2018927	105958336	T	4.1	1.65E-14	chr:1:106015875
1	rs2018940	105958192	C	4.1	1.65E-14	chr:1:106015875
1	rs2501586	105959459	G	4.1	1.65E-14	chr:1:106015875
1	rs2459328	105968239	A	4.094	1.66E-14	chr:1:106015875
1	rs2459329	105968586	G	4.094	1.66E-14	chr:1:106015875
1	rs2501583	105967946	A	4.094	1.66E-14	chr:1:106015875
1	rs1546627	105950316	T	4.158	1.68E-14	chr:1:106015875
1	rs4356105	105954220	G	4.158	1.68E-14	chr:1:106015875
1	rs2459323	105961094	A	4.08	1.96E-14	chr:1:106015875
1	rs4522039	105963002	A	4.08	1.96E-14	chr:1:106015875
1	rs4847042	105963120	A	4.08	1.96E-14	chr:1:106015875

1	rs2459322	105961795	A	3.99	2.90E-14	chr:1:106015875
1	rs1516167	105944997	T	4.065	2.94E-14	chr:1:106015875
1	rs2341703	105952310	G	4.065	2.94E-14	chr:1:106015875
1	rs2501592	105950855	C	4.065	2.94E-14	chr:1:106015875
1	rs2502913	105948633	G	4.065	2.94E-14	chr:1:106015875
1	rs4423065	105962843	T	4.045	3.44E-14	chr:1:106015875
1	rs1516160	106011680	A	4.039	4.81E-14	chr:1:106015875
1	rs1616562	106033932	A	2746	5.12E-14	chr:1:106015875
1	rs1710763	106036401	C	2746	5.12E-14	chr:1:106015875
1	rs1710764	106036371	T	2746	5.12E-14	chr:1:106015875
1	rs1721588	106036430	A	2746	5.12E-14	chr:1:106015875
1	rs1721591	106034215	T	2746	5.12E-14	chr:1:106015875
1	rs1710762	106036654	C	2718	5.46E-14	chr:1:106015875
1	rs1721584	106036800	T	2718	5.46E-14	chr:1:106015875
1	rs1721585	106036787	T	2718	5.46E-14	chr:1:106015875
1	rs1516165	106039504	T	2699	5.58E-14	chr:1:106015875
1	rs10881295	105937528	T	3.953	6.58E-14	chr:1:106015875
1	rs11184550	105936193	G	3.953	6.58E-14	chr:1:106015875
1	rs77199289	105867471	G	11.51	7.54E-14	chr:1:106015875
1	rs11811336	105937215	A	3.823	8.06E-14	chr:1:106015875
1	rs2502915	106016319	T	0.235	1.25E-13	chr:1:106015875
1	rs1516169	105942480	T	3.713	1.50E-13	chr:1:106015875
1	rs1516170	105942265	C	3.713	1.50E-13	chr:1:106015875
1	rs2459310	106006312	T	0.2859	1.45E-12	chr:1:106015875
1	rs1913979	106026332	G	0.2804	1.84E-12	chr:1:106015875
1	rs61164181	105751026	T	4.047	1.88E-12	chr:1:106015875
1	rs3935799	106013859	A	0.2926	5.29E-12	chr:1:106015875
1	rs115474157	106083384	A	74.92	6.07E-12	chr:1:106015875
1	rs2502850	106012498	T	0.3007	7.96E-12	chr:1:106015875
1	rs139174365	105763925	C	8.757	8.33E-12	chr:1:106015875
1	rs12410557	105945707	C	3.317	8.88E-12	chr:1:106015875
1	rs2048839	105944301	A	3.317	8.88E-12	chr:1:106015875
1	rs66461932	105753684	C	3.792	8.93E-12	chr:1:106015875
1	rs202075201	106015815	T	0.3001	1.23E-11	chr:1:106015875
1	rs35570551	106066311	T	0.2865	1.34E-11	chr:1:106015875
1	rs7546711	106060014	C	0.2865	1.34E-11	chr:1:106015875
1	rs11184551	105937403	T	3.296	1.54E-11	chr:1:106015875
1	rs7520366	106071542	C	0.2967	1.94E-11	chr:1:106015875
1	rs58273107	106072932	A	0.2912	2.19E-11	chr:1:106015875
1	rs7520462	106071601	C	0.2912	2.19E-11	chr:1:106015875
1	rs34282524	106039986	T	0.2995	2.30E-11	chr:1:106015875
1	rs7416522	106056427	A	0.2959	2.77E-11	chr:1:106015875
1	rs11184575	106014131	T	0.2772	2.92E-11	chr:1:106015875

1	rs12736550	106037135	C	0.2913	3.01E-11	chr:1:106015875
1	rs12740117	106035061	G	0.2913	3.01E-11	chr:1:106015875
1	rs34763646	106037800	A	0.2913	3.01E-11	chr:1:106015875
1	rs11583556	106043955	A	0.3019	3.10E-11	chr:1:106015875
1	rs11585459	106049997	C	0.3019	3.10E-11	chr:1:106015875
1	rs12731640	106053411	T	0.3019	3.10E-11	chr:1:106015875
1	rs1524110	106056983	A	0.3019	3.10E-11	chr:1:106015875
1	rs17016041	106057104	T	0.3019	3.10E-11	chr:1:106015875
1	rs34086078	106045540	T	0.3019	3.10E-11	chr:1:106015875
1	rs35057076	106066099	A	0.3019	3.10E-11	chr:1:106015875
1	rs35663660	106052782	A	0.3019	3.10E-11	chr:1:106015875
1	rs35719854	106043937	G	0.3019	3.10E-11	chr:1:106015875
1	rs56672462	106065612	A	0.3019	3.10E-11	chr:1:106015875
1	rs2502905	105980618	G	3.28	3.35E-11	chr:1:106015875
1	rs11580189	106034938	G	0.2914	3.38E-11	chr:1:106015875
1	rs57386757	106065793	C	0.3023	3.90E-11	chr:1:106015875
1	rs11589718	106074279	C	0.3026	4.21E-11	chr:1:106015875
1	rs4847128	106073581	A	0.3026	4.21E-11	chr:1:106015875
1	rs4847129	106073809	A	0.3026	4.21E-11	chr:1:106015875
1	rs6582935	106074848	T	0.3026	4.21E-11	chr:1:106015875
1	rs7530092	106077085	A	0.3026	4.21E-11	chr:1:106015875
1	rs12739959	106035632	A	0.2983	5.48E-11	chr:1:106015875
1	rs34713228	106066393	A	0.3115	6.57E-11	chr:1:106015875
1	rs78465248	105742310	T	5.456	1.31E-10	chr:1:106015875
1	rs61799668	105821235	G	4.741	1.35E-10	chr:1:106015875
1	rs61801985	105797351	G	4.741	1.35E-10	chr:1:106015875
1	rs12025612	105747413	T	3.733	1.44E-10	chr:1:106015875
1	rs111784611	105918850	A	6.78	2.09E-10	chr:1:106015875
1	rs112735666	105922596	C	6.78	2.09E-10	chr:1:106015875
1	rs113509697	105921450	C	6.78	2.09E-10	chr:1:106015875
1	rs113749066	105914937	C	6.78	2.09E-10	chr:1:106015875
1	rs6691077	105913642	T	6.78	2.09E-10	chr:1:106015875
1	rs112274969	105923747	A	9.333	2.23E-10	chr:1:106015875
1	rs66706038	105741549	T	3.677	2.35E-10	chr:1:106015875
1	rs6681292	105913357	A	6.5	3.26E-10	chr:1:106015875
1	rs75169870	105770091	A	4.692	3.37E-10	chr:1:106015875
1	rs2653958	105794365	A	4.517	3.40E-10	chr:1:106015875
1	rs2251261	105903564	G	6.453	3.52E-10	chr:1:106015875
1	rs2455694	105894556	C	6.453	3.52E-10	chr:1:106015875
1	rs2491594	105903400	T	6.453	3.52E-10	chr:1:106015875
1	rs2491598	105898549	C	6.453	3.52E-10	chr:1:106015875
1	rs2491595	105902535	T	6.179	3.73E-10	chr:1:106015875
1	rs74397125	105858256	T	5.39	4.61E-10	chr:1:106015875

1	rs777064	106083957	T	2.689	5.08E-10	chr:1:106015875
1	rs2501584	105965571	C	3.005	5.44E-10	chr:1:106015875
1	rs75819272	105741790	T	4.614	6.23E-10	chr:1:106015875
1	rs114523650	105751676	T	4.743	6.66E-10	chr:1:106015875
1	rs7552941	105770337	C	4.077	6.99E-10	chr:1:106015875
1	rs141855967	105931476	T	8.811	7.13E-10	chr:1:106015875
1	rs987243	105957249	G	2.761	7.15E-10	chr:1:106015875
1	rs4598542	105724879	A	3.854	7.19E-10	chr:1:106015875
1	rs11576686	106023978	C	0.3159	7.54E-10	chr:1:106015875
1	rs2016033	106023753	C	0.3159	7.54E-10	chr:1:106015875
1	rs35921147	106026496	A	0.3159	7.54E-10	chr:1:106015875
1	rs61232173	106015774	T	0.3159	7.54E-10	chr:1:106015875
1	rs7415139	106026464	C	0.3159	7.54E-10	chr:1:106015875
1	rs4598541	106085602	A	0.327	8.27E-10	chr:1:106015875
1	rs11580550	106031366	G	0.3176	9.30E-10	chr:1:106015875
1	rs1913977	106035092	G	0.3175	9.52E-10	chr:1:106015875
1	rs61814796	106084764	A	0.3315	9.74E-10	chr:1:106015875
1	rs34695077	106082923	A	0.3328	1.08E-09	chr:1:106015875
1	rs143651669	105730556	T	4.532	1.18E-09	chr:1:106015875
1	rs61802870	105721358	A	3.827	1.59E-09	chr:1:106015875
1	rs11184553	105938462	C	0.3423	1.88E-09	chr:1:106015875
1	rs72685575	105742799	G	4.303	2.06E-09	chr:1:106015875
1	rs77726109	105754593	C	4.202	2.22E-09	chr:1:106015875
1	rs10785749	106024798	G	0.3133	2.94E-09	chr:1:106015875
1	rs11184558	105940737	G	2.566	3.34E-09	chr:1:106015875
1	rs2842202	105785079	G	4.09	3.49E-09	chr:1:106015875
1	rs61204110	105787019	A	4.256	3.91E-09	chr:1:106015875
1	rs17410084	105729213	G	4.227	3.98E-09	chr:1:106015875
1	rs2455670	105863366	A	5.276	4.09E-09	chr:1:106015875
1	rs2491634	105859257	A	5.276	4.09E-09	chr:1:106015875
1	rs2502887	105862250	G	5.276	4.09E-09	chr:1:106015875
1	rs113219798	105762498	G	3.72	5.33E-09	chr:1:106015875
1	rs115647603	105753833	G	3.72	5.33E-09	chr:1:106015875
1	rs1622623	106019590	A	0.2792	6.06E-09	chr:1:106015875
1	rs1710801	106019165	G	0.2792	6.06E-09	chr:1:106015875
1	rs1710800	106019088	A	0.2333	6.17E-09	chr:1:106015875
1	rs144456983	105849597	T	5.565	6.34E-09	chr:1:106015875
1	rs1400095	105998181	C	2.656	6.75E-09	chr:1:106015875
1	rs12217056	105726684	T	3.242	7.02E-09	chr:1:106015875
1	rs12217119	105726638	T	3.242	7.02E-09	chr:1:106015875
1	rs17015538	105727945	G	3.242	7.02E-09	chr:1:106015875
1	rs61100528	105727653	A	3.242	7.02E-09	chr:1:106015875
1	rs2341705	106022632	T	0.2774	7.03E-09	chr:1:106015875

1	rs2459334	105941906	G	2.697	7.44E-09	chr:1:106015875
1	rs10458525	106020469	A	0.2793	7.77E-09	chr:1:106015875
1	rs4387226	105940467	T	2.574	8.02E-09	chr:1:106015875
1	rs68122168	105728011	A	3.606	1.71E-08	chr:1:106015875
1	rs527895871	106018882	C	71.53	1.90E-08	chr:1:106015875
1	rs2491644	105853300	G	4.667	3.18E-08	chr:1:106015875
1	rs777096	106063748	T	0.3612	4.49E-08	chr:1:106015875
1	rs777071	106087588	C	4.021	4.99E-08	chr:1:106015875
1	rs994077	105946899	T	2.492	5.16E-08	chr:1:106015875
1	rs994078	105946707	T	2.492	5.16E-08	chr:1:106015875
1	rs6583129	105741765	A	2.353	5.93E-08	chr:1:106015875
1	rs61802371	106010993	G	0.3059	6.44E-08	chr:1:106015875
1	rs7515518	106007433	G	0.3142	8.38E-08	chr:1:106015875
1	rs60011126	106012101	G	0.3118	9.81E-08	chr:1:106015875
1	rs114611383	105722969	T	3.733	1.06E-07	chr:1:106015875
1	rs116221399	105722338	A	3.733	1.06E-07	chr:1:106015875
1	rs6694723	105914819	A	3.903	1.07E-07	chr:1:106015875
1	rs58901097	106012090	T	0.314	1.14E-07	chr:1:106015875
1	rs11184580	106031090	A	0.3127	1.23E-07	chr:1:106015875
1	rs1516156	106032070	G	0.3127	1.23E-07	chr:1:106015875
1	rs11184477	105754803	T	13.23	1.32E-07	chr:1:106015875
1	rs11184491	105762971	T	13.23	1.32E-07	chr:1:106015875
1	rs11184499	105775895	G	13.23	1.32E-07	chr:1:106015875
1	rs12057709	105759497	C	13.23	1.32E-07	chr:1:106015875
1	rs12062311	105801635	A	13.23	1.32E-07	chr:1:106015875
1	rs12066245	105809418	A	13.23	1.32E-07	chr:1:106015875
1	rs12073157	105777047	A	13.23	1.32E-07	chr:1:106015875
1	rs12079151	105794849	G	13.23	1.32E-07	chr:1:106015875
1	rs12088275	105801859	G	13.23	1.32E-07	chr:1:106015875
1	rs138392752	105793935	A	13.23	1.32E-07	chr:1:106015875
1	rs145270872	105787697	A	13.23	1.32E-07	chr:1:106015875
1	rs182198854	105792234	A	13.23	1.32E-07	chr:1:106015875
1	rs575693642	105732177	A	13.23	1.32E-07	chr:1:106015875
1	rs2501603	106007141	A	0.3229	1.54E-07	chr:1:106015875
1	rs12024969	106010402	C	0.3171	1.56E-07	chr:1:106015875
1	rs6583152	105939231	G	0.3925	1.61E-07	chr:1:106015875
1	rs17015519	105725199	C	2.825	1.62E-07	chr:1:106015875
1	rs12037250	106028291	G	0.317	1.64E-07	chr:1:106015875
1	rs1400098	106027601	G	0.317	1.64E-07	chr:1:106015875
1	rs1721555	106081513	A	2.183	1.71E-07	chr:1:106015875
1	rs11184578	106025908	A	0.3189	1.88E-07	chr:1:106015875
1	rs12089773	105799511	T	11.13	2.00E-07	chr:1:106015875
1	rs57204684	105741143	G	2.465	2.26E-07	chr:1:106015875

1	rs11184645	106146803	C	2.885	2.77E-07	chr:1:106015875
1	rs12061093	106142124	C	2.885	2.77E-07	chr:1:106015875
1	rs12061385	106142741	C	2.885	2.77E-07	chr:1:106015875
1	rs12073177	106143049	A	2.885	2.77E-07	chr:1:106015875
1	rs12095228	106145138	C	2.885	2.77E-07	chr:1:106015875
1	rs72978342	106147715	G	2.885	2.77E-07	chr:1:106015875
1	rs11184595	106057086	G	204.2	2.82E-07	chr:1:106015875
1	rs11184601	106068615	G	204.2	2.82E-07	chr:1:106015875
1	rs116119372	106048511	C	204.2	2.82E-07	chr:1:106015875
1	rs116403148	106076295	C	204.2	2.82E-07	chr:1:106015875
1	rs12088963	106052211	C	204.2	2.82E-07	chr:1:106015875
1	rs12096856	106050831	T	204.2	2.82E-07	chr:1:106015875
1	rs145993505	106076328	A	204.2	2.82E-07	chr:1:106015875
1	rs147177659	106070936	C	204.2	2.82E-07	chr:1:106015875
1	rs74465823	106056508	C	204.2	2.82E-07	chr:1:106015875
1	rs76840597	106055476	A	204.2	2.82E-07	chr:1:106015875
1	rs78421232	106044091	C	204.2	2.82E-07	chr:1:106015875
1	rs1710775	106081424	A	2.151	2.94E-07	chr:1:106015875
1	rs111944817	105686231	T	6.329	3.45E-07	chr:1:106015875
1	rs12095857	105824486	T	7.127	3.58E-07	chr:1:106015875
1	rs143822842	105834597	A	7.127	3.58E-07	chr:1:106015875
1	rs150869865	105833358	C	7.127	3.58E-07	chr:1:106015875
1	rs114208439	105716495	G	3.44	4.01E-07	chr:1:106015875
1	rs1710774	106081299	G	2.127	4.39E-07	chr:1:106015875
1	rs10881292	105887769	C	3.692	4.61E-07	chr:1:106015875
1	rs2455689	105888503	G	3.692	4.61E-07	chr:1:106015875
1	rs2455692	105893000	A	3.692	4.61E-07	chr:1:106015875
1	rs2491605	105879717	A	3.692	4.61E-07	chr:1:106015875
1	rs2502854	105891417	G	3.692	4.61E-07	chr:1:106015875
1	rs2502855	105890506	A	3.692	4.61E-07	chr:1:106015875
1	rs2502857	105888045	C	3.692	4.61E-07	chr:1:106015875
1	rs2502859	105886620	A	3.692	4.61E-07	chr:1:106015875
1	rs2502862	105881603	T	3.692	4.61E-07	chr:1:106015875
1	rs115086517	105817986	C	6.842	5.17E-07	chr:1:106015875
1	rs4554770	105997926	A	2.285	5.41E-07	chr:1:106015875
1	rs10127670	106012521	A	2.198	5.85E-07	chr:1:106015875
1	rs2340895	105886108	C	3.618	6.21E-07	chr:1:106015875
1	rs2653963	105793111	G	2.241	6.46E-07	chr:1:106015875
1	rs9787043	105729825	C	2.553	6.53E-07	chr:1:106015875
1	rs144007422	105902428	A	14.75	6.61E-07	chr:1:106015875
1	rs4381251	105761912	C	0.3662	6.72E-07	chr:1:106015875
1	rs4847106	105750245	C	0.3679	6.75E-07	chr:1:106015875
1	rs4471309	105742392	C	0.3692	7.11E-07	chr:1:106015875

1	rs12084501	105983963	G	2.167	7.99E-07	chr:1:106015875
1	rs12739546	106042643	A	0.3995	8.43E-07	chr:1:106015875
1	rs34671917	106042838	T	0.3995	8.43E-07	chr:1:106015875
1	rs35172839	106042833	A	0.3995	8.43E-07	chr:1:106015875
1	rs35725714	106043584	A	0.3995	8.43E-07	chr:1:106015875
1	rs61800477	106043510	T	0.3995	8.43E-07	chr:1:106015875
1	rs10785750	106032404	A	0.3282	8.82E-07	chr:1:106015875
1	rs4847124	106006734	T	0.3183	9.16E-07	chr:1:106015875
1	rs10158067	105982990	A	2.148	9.62E-07	chr:1:106015875
1	rs12025614	105747427	C	0.3892	1.06E-06	chr:1:106015875
1	rs2455684	105870249	T	3.463	1.07E-06	chr:1:106015875
1	rs4240951	105743667	C	0.3767	1.12E-06	chr:1:106015875
1	rs12120660	106086980	G	0.437	1.16E-06	chr:1:106015875
1	rs12062978	106026152	A	154.5	1.21E-06	chr:1:106015875
1	rs10736042	105764122	A	0.3939	1.41E-06	chr:1:106015875
1	rs7544453	105762322	C	0.3939	1.41E-06	chr:1:106015875
1	rs11184522	105848138	T	10.11	1.51E-06	chr:1:106015875
1	rs114503512	105852165	T	10.11	1.51E-06	chr:1:106015875
1	rs12068306	105847403	T	10.11	1.51E-06	chr:1:106015875
1	rs527649208	105848778	A	10.11	1.51E-06	chr:1:106015875
1	rs77248660	105854424	G	10.11	1.51E-06	chr:1:106015875
1	rs111375949	105685211	A	4.295	1.61E-06	chr:1:106015875
1	rs2455674	105867559	A	3.36	1.67E-06	chr:1:106015875
1	rs2502871	105868987	G	3.36	1.67E-06	chr:1:106015875
1	rs2502876	105867088	G	3.36	1.67E-06	chr:1:106015875
1	rs2502916	105941966	A	2.21	1.69E-06	chr:1:106015875
1	rs7540344	105766289	C	0.3982	1.76E-06	chr:1:106015875
1	rs2102765	105980430	C	2.115	1.81E-06	chr:1:106015875
1	rs6664964	105822668	A	2.379	2.08E-06	chr:1:106015875
1	rs12142840	105998320	C	0.4283	2.13E-06	chr:1:106015875
1	rs72690944	106034336	T	0.3381	2.51E-06	chr:1:106015875
1	rs2653970	105790671	C	2.129	2.54E-06	chr:1:106015875
1	rs2842205	105790865	G	2.129	2.54E-06	chr:1:106015875
1	rs17015975	106045394	G	0.4114	2.74E-06	chr:1:106015875
1	rs2653964	105792654	G	2.114	2.88E-06	chr:1:106015875
1	rs114176247	106036218	A	0.3413	2.97E-06	chr:1:106015875
1	rs12090701	106046094	T	0.3413	2.97E-06	chr:1:106015875
1	rs11184445	105678189	A	12.39	3.04E-06	chr:1:106015875
1	rs11184542	105899962	A	35.37	3.29E-06	chr:1:106015875
1	rs116477821	105884616	T	35.37	3.29E-06	chr:1:106015875
1	rs12073774	105856736	C	35.37	3.29E-06	chr:1:106015875
1	rs12081910	105867598	C	35.37	3.29E-06	chr:1:106015875
1	rs12089174	105872838	G	35.37	3.29E-06	chr:1:106015875

1	rs12094194	105880758	G	35.37	3.29E-06	chr:1:106015875
1	rs147587583	105863503	C	35.37	3.29E-06	chr:1:106015875
1	rs61799719	105921633	T	2.125	3.45E-06	chr:1:106015875
1	rs4520462	105724511	G	2.339	3.50E-06	chr:1:106015875
1	rs4520463	105724777	C	2.339	3.50E-06	chr:1:106015875
1	rs5023774	105724840	T	2.339	3.50E-06	chr:1:106015875
1	rs5023775	105724847	G	2.339	3.50E-06	chr:1:106015875
1	rs6695016	106040066	T	0.344	3.60E-06	chr:1:106015875
1	rs11184605	106078805	G	0.3543	3.68E-06	chr:1:106015875
1	rs12066943	105846636	T	7.305	3.99E-06	chr:1:106015875
1	rs2491592	105904728	A	21.02	4.10E-06	chr:1:106015875
1	rs4494191	105759207	G	2.09	5.09E-06	chr:1:106015875
1	rs2842215	105803955	T	2.216	5.11E-06	chr:1:106015875
1	rs12025289	106011628	G	0.4206	6.05E-06	chr:1:106015875
1	rs11184498	105775864	T	32.11	6.66E-06	chr:1:106015875
1	rs116086800	105771040	A	32.11	6.66E-06	chr:1:106015875
1	rs12072886	105829792	T	32.11	6.66E-06	chr:1:106015875
1	rs11184597	106064241	G	0.3663	6.68E-06	chr:1:106015875
1	rs12023854	106064845	A	0.3663	6.68E-06	chr:1:106015875
1	rs11184560	105947701	A	2.025	6.85E-06	chr:1:106015875
1	rs17420076	105949341	T	2.025	6.85E-06	chr:1:106015875
1	rs4847115	105949664	A	2.025	6.85E-06	chr:1:106015875
1	rs4847116	105949895	C	2.025	6.85E-06	chr:1:106015875
1	rs17015610	105821331	C	2.205	7.63E-06	chr:1:106015875
1	rs61802869	105716761	A	2.299	7.65E-06	chr:1:106015875
1	rs4356104	105940304	C	0.4474	8.27E-06	chr:1:106015875
1	rs12073686	105689610	T	3.118	8.32E-06	chr:1:106015875
1	rs12095042	105686805	G	3.118	8.32E-06	chr:1:106015875
1	rs1400099	106052389	C	0.4051	9.00E-06	chr:1:106015875
1	rs11184455	105705082	T	30.4	9.39E-06	chr:1:106015875
1	rs28408426	105715318	T	30.4	9.39E-06	chr:1:106015875
1	rs11184449	105691844	G	3.901	9.46E-06	chr:1:106015875
1	rs12091417	105542166	C	18.63	9.60E-06	chr:1:106015875
1	rs189019094	105538480	G	18.63	9.60E-06	chr:1:106015875
1	rs2262604	105795657	A	2.158	1.05E-05	chr:1:106015875
1	rs2653946	105801055	G	2.158	1.05E-05	chr:1:106015875
1	rs2653952	105797758	T	2.158	1.05E-05	chr:1:106015875
1	rs2653955	105796743	T	2.158	1.05E-05	chr:1:106015875
1	rs2653962	105793150	C	2.158	1.05E-05	chr:1:106015875
1	rs2809308	105806892	A	2.158	1.05E-05	chr:1:106015875
1	rs2809309	105804239	C	2.158	1.05E-05	chr:1:106015875
1	rs2809314	105802092	C	2.158	1.05E-05	chr:1:106015875
1	rs2809315	105801615	G	2.158	1.05E-05	chr:1:106015875

1	rs2809316	105801603	G	2.158	1.05E-05	chr:1:106015875
1	rs2809319	105800889	A	2.158	1.05E-05	chr:1:106015875
1	rs2809323	105800294	C	2.158	1.05E-05	chr:1:106015875
1	rs2809325	105799204	G	2.158	1.05E-05	chr:1:106015875
1	rs2809326	105799111	T	2.158	1.05E-05	chr:1:106015875
1	rs2809330	105796736	T	2.158	1.05E-05	chr:1:106015875
1	rs2809333	105795642	C	2.158	1.05E-05	chr:1:106015875
1	rs2842208	105795113	C	2.158	1.05E-05	chr:1:106015875
1	rs2842209	105795246	A	2.158	1.05E-05	chr:1:106015875
1	rs9435375	105795431	C	2.158	1.05E-05	chr:1:106015875
1	rs2653942	105803548	A	2.148	1.19E-05	chr:1:106015875
1	rs13374559	106082557	A	0.4164	1.29E-05	chr:1:106015875
1	rs1516168	105942573	G	0.3674	1.37E-05	chr:1:106015875
1	rs2501595	105942599	G	0.3674	1.37E-05	chr:1:106015875
1	rs11184651	106173159	A	2.462	1.39E-05	chr:1:106015875
1	rs12071793	106163378	G	2.462	1.39E-05	chr:1:106015875
1	rs12078346	106172976	T	2.462	1.39E-05	chr:1:106015875
1	rs2809329	105797309	C	2.132	1.41E-05	chr:1:106015875
1	rs11184450	105692228	G	3.749	1.47E-05	chr:1:106015875
1	rs7528406	105727098	C	0.438	1.60E-05	chr:1:106015875
1	rs2842216	105804507	A	2.136	1.60E-05	chr:1:106015875
1	rs149544970	106100694	T	8.498	1.61E-05	chr:1:106015875
1	rs11804803	106040837	T	0.4197	1.61E-05	chr:1:106015875
1	rs12048213	111821434	T	23.85	1.65E-39	chr:1:111802591
1	rs10857867	111810878	A	35.34	2.04E-39	chr:1:111802591
1	rs12139505	111816865	A	24.14	1.06E-38	chr:1:111802591
1	rs12034487	111805474	G	55.15	7.67E-38	chr:1:111802591
1	rs10857869	111822699	T	23.4	4.05E-36	chr:1:111802591
1	rs3118208	111812709	A	43.04	6.20E-35	chr:1:111802591
1	rs4259643	111814404	A	43	6.73E-35	chr:1:111802591
1	rs3118209	111812196	T	42.72	7.11E-35	chr:1:111802591
1	rs2104408	111810753	C	46.59	1.93E-34	chr:1:111802591
1	rs2820057	111811175	G	46.59	1.93E-34	chr:1:111802591
1	rs2820058	111811160	C	46.59	1.93E-34	chr:1:111802591
1	rs4240529	111824744	A	16.23	1.23E-33	chr:1:111802591
1	rs10857866	111810876	A	11.86	1.95E-29	chr:1:111802591
1	rs3122591	111810174	C	0.1024	1.36E-28	chr:1:111802591
1	rs2764548	111808585	T	0.07971	2.14E-28	chr:1:111802591
1	rs2820059	111808392	G	0.07971	2.14E-28	chr:1:111802591
1	rs3122593	111810965	A	0.1117	2.95E-28	chr:1:111802591
1	rs2494013	111813821	A	0.1292	9.10E-28	chr:1:111802591
1	rs3118206	111814461	G	0.1292	9.10E-28	chr:1:111802591
1	rs3122594	111813735	T	0.1292	9.10E-28	chr:1:111802591

1	rs3122595	111813951	T	0.1292	9.10E-28	chr:1:111802591
1	rs11102231	111817468	A	0.1468	6.64E-27	chr:1:111802591
1	rs4304581	111801635	G	0.1518	1.25E-26	chr:1:111802591
1	rs12139408	111816538	A	9.881	2.29E-26	chr:1:111802591
1	rs12728726	111819239	C	9.852	2.46E-26	chr:1:111802591
1	rs2820082	111800472	T	0.177	8.68E-25	chr:1:111802591
1	rs6687390	111825226	T	0.1805	3.61E-24	chr:1:111802591
1	rs10776720	111808276	A	207.7	2.13E-23	chr:1:111802591
1	rs10776722	111828464	C	5.801	4.08E-22	chr:1:111802591
1	rs7517252	111828817	A	7.161	3.12E-21	chr:1:111802591
1	rs6683292	111801402	A	4.379	9.84E-21	chr:1:111802591
1	rs4240528	111802958	C	0.2218	1.42E-20	chr:1:111802591
1	rs10776719	111808180	T	280.5	1.91E-20	chr:1:111802591
1	rs1853784	111804717	C	280.5	1.91E-20	chr:1:111802591
1	rs2494012	111808937	T	280.5	1.91E-20	chr:1:111802591
1	rs2764538	111804370	A	280.5	1.91E-20	chr:1:111802591
1	rs2764549	111808546	C	280.5	1.91E-20	chr:1:111802591
1	rs2820062	111805966	G	280.5	1.91E-20	chr:1:111802591
1	rs12140893	111812832	A	7.927	3.93E-20	chr:1:111802591
1	rs1590499	111804651	A	260.5	4.37E-20	chr:1:111802591
1	rs11809538	111801853	A	0.2536	2.28E-18	chr:1:111802591
1	rs6698373	111816497	A	0.2578	3.02E-18	chr:1:111802591
1	rs3927471	111802912	C	0.2552	3.03E-18	chr:1:111802591
1	rs61808280	111807687	C	0.2552	3.03E-18	chr:1:111802591
1	rs7550145	111804157	T	0.2552	3.03E-18	chr:1:111802591
1	rs9660405	111808946	C	0.2552	3.03E-18	chr:1:111802591
1	rs6702380	111818052	A	0.2586	4.47E-18	chr:1:111802591
1	rs6655936	111821516	G	0.2587	4.49E-18	chr:1:111802591
1	rs6671713	111815790	T	0.2654	2.03E-17	chr:1:111802591
1	rs7512322	111818550	A	0.272	6.32E-17	chr:1:111802591
1	rs41282488	111828423	T	0.28	3.61E-16	chr:1:111802591
1	rs10494131	111824711	C	0.2833	1.05E-15	chr:1:111802591
1	rs6688201	111826023	G	0.2833	1.05E-15	chr:1:111802591
1	rs6688325	111826175	T	0.2833	1.05E-15	chr:1:111802591
1	rs6537690	111824209	A	0.2848	1.05E-15	chr:1:111802591
1	rs6537691	111824255	T	0.2848	1.05E-15	chr:1:111802591
1	rs9659635	111808642	A	0.2878	3.06E-15	chr:1:111802591
1	rs11800770	111813650	T	0.2912	8.24E-15	chr:1:111802591
1	rs10857871	111829633	C	6.415	4.12E-14	chr:1:111802591
1	rs6703883	111820644	G	0.3053	4.67E-14	chr:1:111802591
1	rs6694890	111827901	T	5.355	9.82E-14	chr:1:111802591
1	rs4838897	111833661	T	5.213	1.05E-12	chr:1:111802591
1	rs4348720	111806066	A	0.28	1.35E-12	chr:1:111802591

1	rs4418594	111825261	A	0.3526	1.53E-12	chr:1:111802591
1	rs41281400	111827898	A	0.3258	1.78E-12	chr:1:111802591
1	rs4575084	111826609	A	2.427	7.29E-11	chr:1:111802591
1	rs11102233	111827749	T	4.029	7.92E-11	chr:1:111802591
1	rs4838899	111834050	G	3.451	1.12E-10	chr:1:111802591
1	rs6685226	111780604	G	3.946	1.21E-10	chr:1:111802591
1	rs12023459	111832973	T	3.454	1.21E-10	chr:1:111802591
1	rs41282484	111828379	C	0.3025	1.43E-10	chr:1:111802591
1	rs12724976	111813174	C	0.2612	4.86E-10	chr:1:111802591
1	rs35884994	111816144	C	0.2594	5.80E-10	chr:1:111802591
1	rs1575721	111804605	C	0.2951	8.27E-10	chr:1:111802591
1	rs1575723	111803817	T	0.2951	8.27E-10	chr:1:111802591
1	rs72981857	111812089	T	5.299	9.93E-10	chr:1:111802591
1	rs11102240	111844621	G	2.642	1.24E-09	chr:1:111802591
1	rs2820061	111806570	T	0.3071	2.04E-09	chr:1:111802591
1	rs7411387	111837135	C	2.579	2.35E-09	chr:1:111802591
1	rs7515475	111801948	C	0.3124	2.50E-09	chr:1:111802591
1	rs11588487	111812241	T	0.2621	2.78E-09	chr:1:111802591
1	rs75132719	111800925	C	7.011	3.75E-09	chr:1:111802591
1	rs11102229	111808446	G	0.2686	4.21E-09	chr:1:111802591
1	rs12733155	111812412	T	0.2578	4.41E-09	chr:1:111802591
1	rs59772828	111808783	A	0.2668	6.21E-09	chr:1:111802591
1	rs34487304	111804011	A	0.2646	6.77E-09	chr:1:111802591
1	rs12135216	111809857	G	0.2838	8.22E-09	chr:1:111802591
1	rs72981843	111808146	C	5.025	9.46E-09	chr:1:111802591
1	rs4838905	111840470	T	2.847	1.05E-08	chr:1:111802591
1	rs3118205	111814475	C	0.3681	1.07E-08	chr:1:111802591
1	rs3891601	111805489	G	0.2728	1.11E-08	chr:1:111802591
1	rs12738135	111809571	G	0.3124	1.14E-08	chr:1:111802591
1	rs4376736	111841944	T	2.846	1.19E-08	chr:1:111802591
1	rs2764541	111811430	C	0.3774	2.20E-08	chr:1:111802591
1	rs2147787	111820225	A	0.3364	5.33E-08	chr:1:111802591
1	rs11589375	111822887	G	0.3398	5.95E-08	chr:1:111802591
1	rs12758301	111822132	C	0.3398	5.95E-08	chr:1:111802591
1	rs4323713	111823220	T	0.3398	5.95E-08	chr:1:111802591
1	rs5029527	111818923	T	0.3398	5.95E-08	chr:1:111802591
1	rs10857872	111838382	A	2.356	7.02E-08	chr:1:111802591
1	rs11102232	111818450	C	0.3465	8.87E-08	chr:1:111802591
1	rs35693821	111816294	G	0.3469	1.06E-07	chr:1:111802591
1	rs6675853	111841598	C	4.791	1.46E-07	chr:1:111802591
1	rs6676797	111843174	T	4.791	1.46E-07	chr:1:111802591
1	rs12140873	111826055	G	5.059	1.58E-07	chr:1:111802591
1	rs12411206	111822962	C	0.348	1.71E-07	chr:1:111802591

1	rs12567865	111817250	A	0.348	1.71E-07	chr:1:111802591
1	rs58982671	111826007	T	0.348	1.71E-07	chr:1:111802591
1	rs6694264	111848628	G	0.4459	1.88E-07	chr:1:111802591
1	rs11589353	111822680	G	0.3541	2.73E-07	chr:1:111802591
1	rs74108969	111848742	G	0.4534	3.09E-07	chr:1:111802591
1	rs74108968	111847695	C	0.4253	3.30E-07	chr:1:111802591
1	rs6682169	111848975	T	0.4526	3.91E-07	chr:1:111802591
1	rs6697360	111849106	C	0.4526	3.91E-07	chr:1:111802591
1	rs7518496	111790311	G	1.926	4.80E-07	chr:1:111802591
1	rs6657000	111848961	A	0.4559	4.89E-07	chr:1:111802591
1	rs6657192	111849107	A	0.4559	4.89E-07	chr:1:111802591
1	rs12086232	111851186	T	0.4556	5.43E-07	chr:1:111802591
1	rs12128628	111850085	A	0.4556	5.43E-07	chr:1:111802591
1	rs114140388	111813706	T	6.134	5.66E-07	chr:1:111802591
1	rs7412290	111851040	A	0.4589	6.78E-07	chr:1:111802591
1	rs12071109	111851342	C	0.4603	7.35E-07	chr:1:111802591
1	rs28480609	111851572	T	0.4603	7.35E-07	chr:1:111802591
1	rs28531702	111851860	G	0.4603	7.35E-07	chr:1:111802591
1	rs56146935	111851403	T	0.4603	7.35E-07	chr:1:111802591
1	rs7531210	111852352	T	0.4603	7.35E-07	chr:1:111802591
1	rs7543074	111852600	G	0.4603	7.35E-07	chr:1:111802591
1	rs7545065	111852255	A	0.4603	7.35E-07	chr:1:111802591
1	rs7545343	111852527	G	0.4603	7.35E-07	chr:1:111802591
1	rs12125099	111855621	G	0.4604	7.48E-07	chr:1:111802591
1	rs12401376	111828474	A	0.3752	7.65E-07	chr:1:111802591
1	rs79950640	111787045	A	0.4519	9.05E-07	chr:1:111802591
1	rs10494133	111854688	C	0.4647	9.74E-07	chr:1:111802591
1	rs11102243	111854044	T	0.4647	9.74E-07	chr:1:111802591
1	rs11102244	111854076	T	0.4647	9.74E-07	chr:1:111802591
1	rs12128422	111855454	C	0.4647	9.74E-07	chr:1:111802591
1	rs12129604	111856561	A	0.4647	9.74E-07	chr:1:111802591
1	rs12131907	111853376	A	0.4647	9.74E-07	chr:1:111802591
1	rs12136028	111855396	T	0.4647	9.74E-07	chr:1:111802591
1	rs12137027	111856269	T	0.4647	9.74E-07	chr:1:111802591
1	rs12139473	111853377	T	0.4647	9.74E-07	chr:1:111802591
1	rs41282490	111854741	G	0.4647	9.74E-07	chr:1:111802591
1	rs41282494	111854895	A	0.4647	9.74E-07	chr:1:111802591
1	rs41282496	111854938	T	0.4647	9.74E-07	chr:1:111802591
1	rs74108971	111855149	T	0.4647	9.74E-07	chr:1:111802591
1	rs11583210	111781886	A	3.221	1.10E-06	chr:1:111802591
1	rs35599356	111782540	G	3.221	1.10E-06	chr:1:111802591
1	rs28536723	111851749	G	0.4669	1.14E-06	chr:1:111802591
1	rs12132538	111865013	A	0.4615	1.52E-06	chr:1:111802591

1	rs76388937	111785979	C	4.534	1.73E-06	chr:1:111802591
1	rs4284263	111823414	C	6.914	2.09E-06	chr:1:111802591
1	rs1056825	111785930	C	2.339	2.10E-06	chr:1:111802591
1	rs55737011	111852824	A	0.4747	2.12E-06	chr:1:111802591
1	rs76275224	111823994	T	0.3722	2.53E-06	chr:1:111802591
1	rs75367073	111791052	A	0.4684	2.54E-06	chr:1:111802591
1	rs115563824	111816883	C	6.798	2.59E-06	chr:1:111802591
1	rs41282492	111854889	G	0.4821	2.60E-06	chr:1:111802591
1	rs3738303	111894180	A	2.83	3.33E-06	chr:1:111802591
1	rs3738304	111890941	A	2.83	3.33E-06	chr:1:111802591
1	rs116638080	111764490	T	5.057	3.42E-06	chr:1:111802591
1	rs75868085	111762769	A	5.057	3.42E-06	chr:1:111802591
1	rs7541787	111850452	C	1.853	3.69E-06	chr:1:111802591
1	rs114382749	111803341	A	0.1844	4.12E-06	chr:1:111802591
1	rs17027081	111743468	T	0.5546	4.95E-06	chr:1:111802591
1	rs41281398	111827466	T	0.3871	5.22E-06	chr:1:111802591
1	rs41281404	111828148	C	0.3871	5.22E-06	chr:1:111802591
1	rs6696410	111840987	A	0.5455	5.34E-06	chr:1:111802591
1	rs11102249	111864700	A	0.4825	5.98E-06	chr:1:111802591
1	rs942692	111746697	T	1.949	7.18E-06	chr:1:111802591
1	rs12072791	111787872	G	0.5469	7.35E-06	chr:1:111802591
1	rs11102234	111832773	T	7.116	7.36E-06	chr:1:111802591
1	rs2820072	111857485	A	0.5523	7.90E-06	chr:1:111802591
1	rs113703527	111840317	A	0.5423	9.69E-06	chr:1:111802591
1	rs10857870	111829220	C	0.5382	9.95E-06	chr:1:111802591
1	rs79725626	111762028	T	3.642	1.04E-05	chr:1:111802591
1	rs12030461	111759662	T	3.106	1.34E-05	chr:1:111802591
1	rs9662740	111844526	C	1.814	1.34E-05	chr:1:111802591
1	rs12735522	111765722	A	3.314	1.64E-05	chr:1:111802591
1	rs112975376	111812293	C	0.3279	1.64E-05	chr:1:111802591
1	rs12077359	111787847	A	0.5384	1.81E-05	chr:1:111802591
1	rs60895025	112001163	A	3.665	2.02E-05	chr:1:111802591
1	rs12076798	111857512	A	0.5061	2.10E-05	chr:1:111802591
1	rs12094378	111862078	T	0.5061	2.10E-05	chr:1:111802591
1	rs7517352	111859050	G	0.5061	2.10E-05	chr:1:111802591
1	rs10494130	111797385	G	2.579	2.12E-05	chr:1:111802591
1	rs17014713	111763153	T	3.023	2.18E-05	chr:1:111802591
1	rs3887445	111761427	A	3.023	2.18E-05	chr:1:111802591
1	rs78462286	111763635	A	3.023	2.18E-05	chr:1:111802591
1	rs1541156	111858687	T	0.5223	2.19E-05	chr:1:111802591
1	rs12027550	111821946	G	4.587	2.24E-05	chr:1:111802591
1	rs12047673	111823173	C	4.587	2.24E-05	chr:1:111802591
1	rs58013456	111826483	G	4.574	2.27E-05	chr:1:111802591

1	rs116694952	111752904	A	3.891	2.49E-05	chr:1:111802591
1	rs113282546	223589739	G	131.9	4.82E-18	chr:1:223578304
1	rs148497163	223589636	A	122.7	7.72E-18	chr:1:223578304
1	rs113023892	223574695	T	197.3	8.44E-18	chr:1:223578304
1	rs148171220	223526935	G	381.4	2.99E-17	chr:1:223578304
1	rs139446310	223564326	G	103.3	4.37E-17	chr:1:223578304
1	rs142553074	223563828	T	103.3	4.37E-17	chr:1:223578304
1	rs149091195	223565422	G	103.3	4.37E-17	chr:1:223578304
1	rs150542653	223563940	A	103.3	4.37E-17	chr:1:223578304
1	rs114113533	223562286	A	46.96	5.41E-17	chr:1:223578304
1	rs113302908	223551701	C	163.9	1.84E-15	chr:1:223578304
1	rs112020523	223526862	T	237.4	2.64E-15	chr:1:223578304
1	rs113853048	223574880	T	110.8	4.29E-15	chr:1:223578304
1	rs141620413	223575930	G	110.8	4.29E-15	chr:1:223578304
1	rs146220066	223576091	T	110.8	4.29E-15	chr:1:223578304
1	rs544155730	223569138	T	212.5	1.12E-14	chr:1:223578304
1	rs113816059	223584961	G	236.7	1.85E-14	chr:1:223578304
1	rs115769114	223586270	T	213.8	8.14E-14	chr:1:223578304
1	rs548918742	223569460	G	190.1	1.45E-13	chr:1:223578304
1	rs555558273	223566737	C	190.1	1.45E-13	chr:1:223578304
1	rs564773873	223574253	A	190.1	1.45E-13	chr:1:223578304
1	rs572064621	223569123	C	190.1	1.45E-13	chr:1:223578304
1	rs1772270	223479060	C	24.35	1.48E-13	chr:1:223578304
1	rs1772271	223480141	C	24.35	1.48E-13	chr:1:223578304
1	rs1356480	223490036	T	23.3	1.63E-13	chr:1:223578304
1	rs1772273	223482173	G	23.3	1.63E-13	chr:1:223578304
1	rs1772275	223486502	C	23.3	1.63E-13	chr:1:223578304
1	rs825124	223488337	A	23.3	1.63E-13	chr:1:223578304
1	rs184232855	223549832	A	181.6	2.12E-13	chr:1:223578304
1	rs192160429	223549233	T	181.6	2.12E-13	chr:1:223578304
1	rs1713206	223486505	T	20.65	5.71E-13	chr:1:223578304
1	rs825125	223489079	T	20.49	6.38E-13	chr:1:223578304
1	rs112175683	223574758	A	145.1	9.21E-13	chr:1:223578304
1	rs148639077	223568849	C	145.1	9.21E-13	chr:1:223578304
1	rs111921471	223524835	A	27.87	1.08E-12	chr:1:223578304
1	rs111941553	223523663	G	27.87	1.08E-12	chr:1:223578304
1	rs116663149	223509790	T	27.87	1.08E-12	chr:1:223578304
1	rs1400962	223478509	A	19.41	2.88E-12	chr:1:223578304
1	rs115764077	223514984	A	25.16	2.98E-12	chr:1:223578304
1	rs1400960	223502811	T	15.39	3.69E-12	chr:1:223578304
1	rs377753813	223538113	T	34.74	3.97E-12	chr:1:223578304
1	rs142409482	223547357	G	27.34	9.44E-12	chr:1:223578304
1	rs564670573	223541689	G	27.34	9.44E-12	chr:1:223578304

1	rs825113	223498145	A	13.12	1.41E-11	chr:1:223578304
1	rs146999469	223631923	T	30.48	1.52E-11	chr:1:223578304
1	rs825114	223495647	A	13.37	1.96E-11	chr:1:223578304
1	rs17162003	223523808	C	19.1	2.75E-11	chr:1:223578304
1	rs142891705	223538778	A	179.9	1.02E-10	chr:1:223578304
1	rs144427708	223538543	T	179.9	1.02E-10	chr:1:223578304
1	rs187934431	223540037	G	179.9	1.02E-10	chr:1:223578304
1	rs541503964	223538000	T	179.9	1.02E-10	chr:1:223578304
1	rs11261268	223574537	C	7.981	1.89E-10	chr:1:223578304
1	rs12074782	223575111	C	7.981	1.89E-10	chr:1:223578304
1	rs12082133	223575202	C	7.981	1.89E-10	chr:1:223578304
1	rs6679661	223567267	T	7.981	1.89E-10	chr:1:223578304
1	rs79205040	223575371	T	7.978	1.98E-10	chr:1:223578304
1	rs144105833	223484757	T	44.36	1.11E-09	chr:1:223578304
1	rs184809316	223520175	A	44.36	1.11E-09	chr:1:223578304
1	rs187476584	223512123	C	44.36	1.11E-09	chr:1:223578304
1	rs4661241	223529815	C	6.632	3.05E-09	chr:1:223578304
1	rs1772285	223574646	G	6.24	4.63E-09	chr:1:223578304
1	rs111306873	223566859	C	6.302	6.58E-09	chr:1:223578304
1	rs114322253	223511018	T	17.35	6.74E-09	chr:1:223578304
1	rs182702369	223460069	T	37.74	9.00E-09	chr:1:223578304
1	rs188693245	223444688	A	37.74	9.00E-09	chr:1:223578304
1	rs541636260	223455262	G	37.74	9.00E-09	chr:1:223578304
1	rs2282399	223531803	T	6.058	1.23E-08	chr:1:223578304
1	rs145296796	223542049	C	4.042	1.33E-08	chr:1:223578304
1	rs2501982	223574483	G	5.434	1.39E-08	chr:1:223578304
1	rs569846351	223607681	C	46.72	2.22E-08	chr:1:223578304
1	rs144991841	223574229	C	5.519	2.46E-08	chr:1:223578304
1	rs1547788	223532570	C	5.214	4.49E-08	chr:1:223578304
1	rs145118686	223420257	A	33.81	4.65E-08	chr:1:223578304
1	rs1619227	223549683	C	4.87	5.09E-08	chr:1:223578304
1	rs185186335	223401837	G	26.56	6.33E-08	chr:1:223578304
1	rs1713219	223574801	T	4.72	7.71E-08	chr:1:223578304
1	rs1772284	223574563	G	4.72	7.71E-08	chr:1:223578304
1	rs116211782	223402343	C	24.61	8.65E-08	chr:1:223578304
1	rs7412054	223544706	G	4.869	9.21E-08	chr:1:223578304
1	rs9442046	223528843	G	4.837	9.87E-08	chr:1:223578304
1	rs870486	223525848	C	5.075	1.03E-07	chr:1:223578304
1	rs17163831	223526993	A	4.837	1.10E-07	chr:1:223578304
1	rs4611006	223545114	C	4.778	1.38E-07	chr:1:223578304
1	rs7555607	223547630	G	4.778	1.38E-07	chr:1:223578304
1	rs146954194	223367503	C	18.52	1.40E-07	chr:1:223578304
1	rs1713220	223562254	A	4.802	1.42E-07	chr:1:223578304

1	rs2487521	223561802	C	4.799	1.55E-07	chr:1:223578304
1	rs528799671	223623712	T	327	1.78E-07	chr:1:223578304
1	rs111544996	223603203	A	40.3	1.79E-07	chr:1:223578304
1	rs112387175	223606850	T	40.3	1.79E-07	chr:1:223578304
1	rs113564518	223602358	T	40.3	1.79E-07	chr:1:223578304
1	rs1772281	223556136	G	4.728	1.79E-07	chr:1:223578304
1	rs75549686	223606264	T	40.3	1.79E-07	chr:1:223578304
1	rs6604709	223549347	C	4.653	1.94E-07	chr:1:223578304
1	rs825116	223587515	A	4.369	2.06E-07	chr:1:223578304
1	rs1095167	223589689	C	4.368	2.12E-07	chr:1:223578304
1	rs368997392	223578085	C	268.6	2.55E-07	chr:1:223578304
1	rs2678377	223561839	G	4.547	2.65E-07	chr:1:223578304
1	rs12089137	223540478	A	4.719	3.18E-07	chr:1:223578304
1	rs190956088	223334678	A	21.58	3.69E-07	chr:1:223578304
1	rs114561020	223615999	A	278.5	4.26E-07	chr:1:223578304
1	rs115839657	223612161	G	278.5	4.26E-07	chr:1:223578304
1	rs140601483	223610098	C	278.5	4.26E-07	chr:1:223578304
1	rs141557867	223616035	T	278.5	4.26E-07	chr:1:223578304
1	rs142652637	223609064	C	278.5	4.26E-07	chr:1:223578304
1	rs148343754	223619557	C	278.5	4.26E-07	chr:1:223578304
1	rs151310629	223631012	C	278.5	4.26E-07	chr:1:223578304
1	rs185279945	223619577	C	278.5	4.26E-07	chr:1:223578304
1	rs188205217	223620022	A	278.5	4.26E-07	chr:1:223578304
1	rs189126138	223607106	T	278.5	4.26E-07	chr:1:223578304
1	rs192051318	223620487	T	278.5	4.26E-07	chr:1:223578304
1	rs545549072	223625224	T	278.5	4.26E-07	chr:1:223578304
1	rs549272720	223621320	T	278.5	4.26E-07	chr:1:223578304
1	rs571603807	223613100	C	278.5	4.26E-07	chr:1:223578304
1	rs1341286	223557615	A	4.382	4.60E-07	chr:1:223578304
1	rs531513655	223346577	G	19.22	4.78E-07	chr:1:223578304
1	rs533140692	223345701	T	19.22	4.78E-07	chr:1:223578304
1	rs1095175	223581814	G	4.409	5.69E-07	chr:1:223578304
1	rs1713208	223577888	C	4.472	5.90E-07	chr:1:223578304
1	rs2138964	223577741	G	4.472	5.90E-07	chr:1:223578304
1	rs825115	223586638	A	4.472	5.90E-07	chr:1:223578304
1	rs825130	223584719	T	4.472	5.90E-07	chr:1:223578304
1	rs825129	223584267	G	4.474	5.94E-07	chr:1:223578304
1	rs147650606	223387621	G	25.32	6.41E-07	chr:1:223578304
1	rs190773497	223385479	C	25.32	6.41E-07	chr:1:223578304
1	rs79163656	223599457	T	8.365	6.93E-07	chr:1:223578304
1	rs79992920	223599029	A	8.365	6.93E-07	chr:1:223578304
1	rs115065751	223431616	T	36.25	7.27E-07	chr:1:223578304
1	rs17163828	223524567	T	4.38	7.96E-07	chr:1:223578304

1	rs11800458	223614676	G	3.689	8.68E-07	chr:1:223578304
1	rs143874675	223428458	C	18.72	8.84E-07	chr:1:223578304
1	rs149377286	223372354	A	21.85	9.34E-07	chr:1:223578304
1	rs193298999	223382245	A	21.85	9.34E-07	chr:1:223578304
1	rs187443405	223375768	T	16.57	1.13E-06	chr:1:223578304
1	rs368556337	223392473	C	16.57	1.13E-06	chr:1:223578304
1	rs116237191	223428180	A	12.87	1.18E-06	chr:1:223578304
1	rs141325273	223585727	T	207.9	1.20E-06	chr:1:223578304
1	rs182453047	223581941	T	207.9	1.20E-06	chr:1:223578304
1	rs184127550	223551626	T	207.9	1.20E-06	chr:1:223578304
1	rs184872353	223583665	C	207.9	1.20E-06	chr:1:223578304
1	rs528523441	223552117	G	207.9	1.20E-06	chr:1:223578304
1	rs529704607	223574456	A	207.9	1.20E-06	chr:1:223578304
1	rs548354976	223613018	C	67.16	1.20E-06	chr:1:223578304
1	rs1713209	223578627	T	4.161	1.41E-06	chr:1:223578304
1	rs825131	223518146	C	4.615	1.61E-06	chr:1:223578304
1	rs113300713	223600333	T	7.613	1.61E-06	chr:1:223578304
1	rs1772294	223579973	G	4.082	1.64E-06	chr:1:223578304
1	rs116112887	223406902	A	39.57	1.81E-06	chr:1:223578304
1	rs142866025	223393056	G	39.57	1.81E-06	chr:1:223578304
1	rs146827894	223403403	A	29.19	1.85E-06	chr:1:223578304
1	rs150564478	223402681	T	29.19	1.85E-06	chr:1:223578304
1	rs180955231	223401559	G	29.19	1.85E-06	chr:1:223578304
1	rs182250497	223403453	T	29.19	1.85E-06	chr:1:223578304
1	rs540887665	223400590	T	29.19	1.85E-06	chr:1:223578304
1	rs577931892	223402174	C	29.19	1.85E-06	chr:1:223578304
1	rs61838249	223523185	G	4.259	1.87E-06	chr:1:223578304
1	rs2459849	223561765	A	3.851	2.08E-06	chr:1:223578304
1	rs17163824	223514945	T	4.053	3.27E-06	chr:1:223578304
1	rs140557200	223400001	C	13.78	3.58E-06	chr:1:223578304
1	rs141029080	223400712	G	13.78	3.58E-06	chr:1:223578304
1	rs184027824	223400086	G	13.78	3.58E-06	chr:1:223578304
1	rs186797349	223399925	G	13.78	3.58E-06	chr:1:223578304
1	rs564837872	223573293	G	183.4	4.50E-06	chr:1:223578304
1	rs145231501	223398704	T	16.51	6.75E-06	chr:1:223578304
1	rs185629877	223398693	T	16.51	6.75E-06	chr:1:223578304
1	rs5744134	223311003	A	55.57	6.78E-06	chr:1:223578304
1	rs11799394	223614397	A	3.223	8.80E-06	chr:1:223578304
1	rs1417748	223613551	T	3.223	8.80E-06	chr:1:223578304
1	rs6699902	223616212	C	3.223	8.80E-06	chr:1:223578304
1	rs17163825	223523854	C	3.89	9.53E-06	chr:1:223578304
1	rs185188251	223400662	C	15.46	9.88E-06	chr:1:223578304
1	rs191519537	223400591	C	15.46	9.88E-06	chr:1:223578304

1	rs193141990	223401085	G	15.46	9.88E-06	chr:1:223578304
1	rs191167295	223562023	G	135.1	1.18E-05	chr:1:223578304
1	rs547598164	223537580	C	135.1	1.18E-05	chr:1:223578304
1	rs144789273	223423878	G	14.7	1.18E-05	chr:1:223578304
1	rs7551911	223617182	C	3.12	1.25E-05	chr:1:223578304
1	rs11577440	223621225	G	3.105	1.37E-05	chr:1:223578304
1	rs139633125	223407949	C	30.21	1.43E-05	chr:1:223578304
1	rs61838244	223519871	C	3.867	1.52E-05	chr:1:223578304
1	rs60933808	223563508	T	3.706	1.52E-05	chr:1:223578304
1	rs114791159	223439849	C	13.19	1.68E-05	chr:1:223578304
1	rs187375815	223371302	G	22.35	1.85E-05	chr:1:223578304
1	rs115335457	223497418	T	119.7	1.93E-05	chr:1:223578304
1	rs561556683	223476949	A	119.7	1.93E-05	chr:1:223578304
1	rs549926600	223613079	C	5.279	2.17E-05	chr:1:223578304
1	rs844069	223596251	T	3.085	2.23E-05	chr:1:223578304
1	rs2678370	223594054	T	3.081	2.31E-05	chr:1:223578304
1	rs2678369	223594072	G	3.06	2.72E-05	chr:1:223578304
1	rs61837868	223619301	T	3.328	3.28E-05	chr:1:223578304
1	rs536770077	223720607	C	15.2	3.30E-05	chr:1:223578304
1	rs141506504	223475098	G	11.24	3.40E-05	chr:1:223578304
1	rs145845253	223448121	T	11.24	3.40E-05	chr:1:223578304
1	rs147038656	223475370	C	11.24	3.40E-05	chr:1:223578304
1	rs111900949	223611619	G	3.769	3.69E-05	chr:1:223578304
1	rs112545135	223611178	C	3.769	3.69E-05	chr:1:223578304
1	rs113133820	223611836	T	3.769	3.69E-05	chr:1:223578304
1	rs113261136	223609917	C	3.769	3.69E-05	chr:1:223578304
1	rs114929456	223611018	G	3.769	3.69E-05	chr:1:223578304
1	rs17162092	223611148	C	3.769	3.69E-05	chr:1:223578304
1	rs182653141	223611943	T	3.769	3.69E-05	chr:1:223578304
1	rs185487965	223611944	C	3.769	3.69E-05	chr:1:223578304
1	rs74861303	223611353	G	3.769	3.69E-05	chr:1:223578304
1	rs77204463	223610460	G	3.769	3.69E-05	chr:1:223578304
1	rs77396066	223611100	C	3.769	3.69E-05	chr:1:223578304
1	rs78077769	223611604	G	3.769	3.69E-05	chr:1:223578304
1	rs79629094	223611702	T	3.769	3.69E-05	chr:1:223578304
20	rs6041366	12444867	A	70.46	1.84E-30	chr:20:12402387
20	rs1158442	12381568	T	55.19	6.83E-30	chr:20:12402387
20	rs6041319	12390696	T	52.02	9.52E-30	chr:20:12402387
20	rs112628379	12412353	T	151.4	1.35E-29	chr:20:12402387
20	rs6041328	12405323	T	151.4	1.35E-29	chr:20:12402387
20	rs78442554	12408034	A	151.4	1.35E-29	chr:20:12402387
20	rs111299735	12403766	A	302.3	2.67E-29	chr:20:12402387
20	rs113033767	12402863	A	302.3	2.67E-29	chr:20:12402387

20	rs113465587	12407728	G	302.3	2.67E-29	chr:20:12402387
20	rs113863736	12406435	T	302.3	2.67E-29	chr:20:12402387
20	rs115347228	12402868	T	302.3	2.67E-29	chr:20:12402387
20	rs6041324	12400987	A	302.3	2.67E-29	chr:20:12402387
20	rs6041327	12404063	G	302.3	2.67E-29	chr:20:12402387
20	rs6041329	12405489	C	302.3	2.67E-29	chr:20:12402387
20	rs6041331	12406866	T	302.3	2.67E-29	chr:20:12402387
20	rs6041332	12407362	C	302.3	2.67E-29	chr:20:12402387
20	rs6041334	12408582	T	302.3	2.67E-29	chr:20:12402387
20	rs6041335	12408807	T	302.3	2.67E-29	chr:20:12402387
20	rs6041336	12409056	T	302.3	2.67E-29	chr:20:12402387
20	rs76202039	12408077	T	302.3	2.67E-29	chr:20:12402387
20	rs76670390	12402832	T	302.3	2.67E-29	chr:20:12402387
20	rs79419664	12400697	A	302.3	2.67E-29	chr:20:12402387
20	rs6033415	12427403	G	123.7	9.14E-29	chr:20:12402387
20	rs6033418	12432061	C	123.7	9.14E-29	chr:20:12402387
20	rs6033419	12432357	C	123.7	9.14E-29	chr:20:12402387
20	rs6041345	12416478	T	123.7	9.14E-29	chr:20:12402387
20	rs75859773	12413891	T	45.97	1.27E-28	chr:20:12402387
20	rs79077592	12414053	G	136.1	2.04E-28	chr:20:12402387
20	rs6033411	12419544	A	117.9	3.14E-28	chr:20:12402387
20	rs6041346	12417695	T	48.33	5.05E-28	chr:20:12402387
20	rs78412784	12416970	A	30.65	1.33E-26	chr:20:12402387
20	rs6041343	12414696	T	26.14	7.53E-25	chr:20:12402387
20	rs6041344	12414723	C	26.14	7.53E-25	chr:20:12402387
20	rs79480718	12414804	T	25.44	1.20E-24	chr:20:12402387
20	rs79942557	12414865	G	25.44	1.20E-24	chr:20:12402387
20	rs112182228	12440251	G	17.47	1.54E-22	chr:20:12402387
20	rs76044905	12400273	G	19.11	1.82E-20	chr:20:12402387
20	rs6033404	12392718	G	12.57	2.38E-19	chr:20:12402387
20	rs982720	12415702	G	10.85	1.02E-18	chr:20:12402387
20	rs6033413	12420731	A	8.471	6.94E-18	chr:20:12402387
20	rs2208973	12394990	T	9.824	9.93E-18	chr:20:12402387
20	rs75214329	12394923	G	75.15	1.71E-16	chr:20:12402387
20	rs1543522	12378028	T	70.04	4.39E-16	chr:20:12402387
20	rs6033402	12385137	A	70.04	4.39E-16	chr:20:12402387
20	rs6041322	12393909	T	70.04	4.39E-16	chr:20:12402387
20	rs78031920	12381352	A	70.04	4.39E-16	chr:20:12402387
20	rs6041365	12443879	A	22.45	1.01E-15	chr:20:12402387
20	rs6033412	12420695	G	48.31	1.01E-15	chr:20:12402387
20	rs76913143	12423701	A	48.31	1.01E-15	chr:20:12402387
20	rs77315664	12440583	G	48.31	1.01E-15	chr:20:12402387
20	rs6041369	12448314	G	8.062	3.03E-15	chr:20:12402387

20	rs79420216	12436078	T	46.06	3.08E-15	chr:20:12402387
20	rs6041403	12482361	A	31.16	4.04E-15	chr:20:12402387
20	rs114758761	12452082	G	36.03	5.34E-15	chr:20:12402387
20	rs116348168	12474818	G	36.03	5.34E-15	chr:20:12402387
20	rs6041397	12475659	T	36.03	5.34E-15	chr:20:12402387
20	rs79008745	12449661	A	42.52	1.01E-14	chr:20:12402387
20	rs75117931	12402099	G	3309	7.07E-14	chr:20:12402387
20	rs114584836	12495394	A	21.68	7.58E-14	chr:20:12402387
20	rs6033436	12491921	G	21.68	7.58E-14	chr:20:12402387
20	rs115598781	12484619	G	54.87	1.08E-13	chr:20:12402387
20	rs150802997	12477176	G	54.87	1.08E-13	chr:20:12402387
20	rs76084571	12479767	C	54.87	1.08E-13	chr:20:12402387
20	rs75164900	12391011	T	78.33	1.46E-13	chr:20:12402387
20	rs77796060	12397152	G	78.33	1.46E-13	chr:20:12402387
20	rs7347049	12426444	A	11	5.26E-13	chr:20:12402387
20	rs7347050	12426446	A	11	5.26E-13	chr:20:12402387
20	rs115757547	12485027	A	25.15	5.84E-13	chr:20:12402387
20	rs116466224	12455509	C	72.63	9.51E-13	chr:20:12402387
20	rs76075008	12455108	C	72.63	9.51E-13	chr:20:12402387
20	rs6074428	12421622	A	29.55	1.55E-12	chr:20:12402387
20	rs77796184	12464618	A	67.55	1.81E-12	chr:20:12402387
20	rs115249017	12482675	T	15.08	3.54E-12	chr:20:12402387
20	rs75995150	12412941	T	99.29	4.38E-12	chr:20:12402387
20	rs138242853	12427450	C	96.92	7.38E-12	chr:20:12402387
20	rs74748099	12367068	G	96.92	7.38E-12	chr:20:12402387
20	rs78485531	12422395	A	96.92	7.38E-12	chr:20:12402387
20	rs74597927	12301234	A	13.57	1.44E-11	chr:20:12402387
20	rs79405946	12345614	T	17.17	2.98E-11	chr:20:12402387
20	rs77957262	12353052	G	16.21	4.30E-11	chr:20:12402387
20	rs79322698	12352997	T	16.21	4.30E-11	chr:20:12402387
20	rs150663868	12461148	C	8.657	4.34E-11	chr:20:12402387
20	rs183697438	12468251	C	8.657	4.34E-11	chr:20:12402387
20	rs4814115	12406658	T	4.196	1.21E-10	chr:20:12402387
20	rs7273589	12318863	C	8.771	4.38E-10	chr:20:12402387
20	rs6033422	12440983	T	3.382	4.77E-10	chr:20:12402387
20	rs189911827	12325521	T	8.525	4.79E-10	chr:20:12402387
20	rs200749087	12324302	C	8.212	6.65E-10	chr:20:12402387
20	rs2423652	12414721	G	3.772	7.08E-10	chr:20:12402387
20	rs75283889	12456906	A	6.054	1.25E-09	chr:20:12402387
20	rs6033423	12441485	A	3.435	2.49E-09	chr:20:12402387
20	rs1076791	12414548	C	24.41	4.14E-09	chr:20:12402387
20	rs6078576	12418150	A	24.41	4.14E-09	chr:20:12402387
20	rs6041325	12401841	G	0.2717	5.43E-09	chr:20:12402387

20	rs8121916	12401325	C	0.2751	6.74E-09	chr:20:12402387
20	rs961900	12412558	G	0.275	7.15E-09	chr:20:12402387
20	rs6109442	12492496	C	25.27	7.69E-09	chr:20:12402387
20	rs2423653	12415277	C	0.2809	9.04E-09	chr:20:12402387
20	rs6078572	12413317	A	3.136	9.44E-09	chr:20:12402387
20	rs73257928	12363286	T	5.053	2.04E-08	chr:20:12402387
20	rs17811681	12435803	C	6.38	3.09E-08	chr:20:12402387
20	rs62217077	12434581	A	6.38	3.09E-08	chr:20:12402387
20	rs2423650	12407307	C	0.2768	4.17E-08	chr:20:12402387
20	rs116927816	12430382	A	5.881	4.25E-08	chr:20:12402387
20	rs17225851	12433428	T	5.881	4.25E-08	chr:20:12402387
20	rs191153854	12430462	T	5.881	4.25E-08	chr:20:12402387
20	rs370510432	12430850	C	5.881	4.25E-08	chr:20:12402387
20	rs6078584	12427317	G	5.881	4.25E-08	chr:20:12402387
20	rs6078595	12433726	T	5.881	4.25E-08	chr:20:12402387
20	rs6104895	12423634	A	5.881	4.25E-08	chr:20:12402387
20	rs6104902	12431869	T	5.881	4.25E-08	chr:20:12402387
20	rs6109412	12427919	C	5.881	4.25E-08	chr:20:12402387
20	rs6109413	12429618	A	5.881	4.25E-08	chr:20:12402387
20	rs6109415	12429732	C	5.881	4.25E-08	chr:20:12402387
20	rs6109417	12433285	C	5.881	4.25E-08	chr:20:12402387
20	rs62217062	12429652	A	5.881	4.25E-08	chr:20:12402387
20	rs62217068	12430532	C	5.881	4.25E-08	chr:20:12402387
20	rs62217070	12430736	A	5.881	4.25E-08	chr:20:12402387
20	rs62217071	12430839	C	5.881	4.25E-08	chr:20:12402387
20	rs62217073	12431110	A	5.881	4.25E-08	chr:20:12402387
20	rs62217074	12431224	C	5.881	4.25E-08	chr:20:12402387
20	rs62217075	12431404	A	5.881	4.25E-08	chr:20:12402387
20	rs73086467	12429783	T	5.881	4.25E-08	chr:20:12402387
20	rs7354487	12433661	T	5.881	4.25E-08	chr:20:12402387
20	rs6033391	12357536	T	4.991	5.03E-08	chr:20:12402387
20	rs6078561	12400804	C	0.3039	5.35E-08	chr:20:12402387
20	rs180908033	12430463	C	5.643	6.44E-08	chr:20:12402387
20	rs62217072	12431100	A	5.588	6.81E-08	chr:20:12402387
20	rs6104897	12427834	G	5.592	7.04E-08	chr:20:12402387
20	rs6104898	12427840	T	5.592	7.04E-08	chr:20:12402387
20	rs115418369	12339013	G	18.75	8.51E-08	chr:20:12402387
20	rs142448219	12345691	G	18.75	8.51E-08	chr:20:12402387
20	rs151284840	12349804	G	18.75	8.51E-08	chr:20:12402387
20	rs184200562	12334974	A	18.75	8.51E-08	chr:20:12402387
20	rs184808643	12331832	C	18.75	8.51E-08	chr:20:12402387
20	rs188888725	12331858	A	18.75	8.51E-08	chr:20:12402387
20	rs189345805	12347114	C	18.75	8.51E-08	chr:20:12402387

20	rs190202636	12337629	T	18.75	8.51E-08	chr:20:12402387
20	rs191702285	12352826	C	18.75	8.51E-08	chr:20:12402387
20	rs534143730	12340682	T	18.75	8.51E-08	chr:20:12402387
20	rs115446612	12330972	G	12.73	8.84E-08	chr:20:12402387
20	rs188647098	12325753	G	12.73	8.84E-08	chr:20:12402387
20	rs2423658	12418229	C	0.2903	1.17E-07	chr:20:12402387
20	rs532712527	12502632	A	9.606	1.20E-07	chr:20:12402387
20	rs180675070	12431897	A	60.4	1.24E-07	chr:20:12402387
20	rs184342578	12413537	A	60.4	1.24E-07	chr:20:12402387
20	rs192472764	12404826	C	60.4	1.24E-07	chr:20:12402387
20	rs549726688	12428639	C	60.4	1.24E-07	chr:20:12402387
20	rs75230420	12434749	A	60.4	1.24E-07	chr:20:12402387
20	rs7260674	12437164	C	0.309	1.95E-07	chr:20:12402387
20	rs557077506	12429449	T	61.9	2.55E-07	chr:20:12402387
20	rs761795	12413671	T	0.2981	2.65E-07	chr:20:12402387
20	rs761796	12413967	T	0.2991	2.92E-07	chr:20:12402387
20	rs2423654	12415338	C	0.3096	3.66E-07	chr:20:12402387
20	rs961902	12412258	A	2.768	4.10E-07	chr:20:12402387
20	rs117135667	12429471	T	5.286	4.34E-07	chr:20:12402387
20	rs372438680	12429595	A	5.286	4.34E-07	chr:20:12402387
20	rs76164157	12334041	G	11.49	4.70E-07	chr:20:12402387
20	rs2423669	12431381	C	0.3082	4.95E-07	chr:20:12402387
20	rs2423660	12418517	T	0.3137	5.17E-07	chr:20:12402387
20	rs6074432	12435756	T	0.2982	5.73E-07	chr:20:12402387
20	rs6078587	12428279	G	5.096	5.95E-07	chr:20:12402387
20	rs141045617	12284519	T	13.19	6.01E-07	chr:20:12402387
20	rs141365476	12274133	C	13.19	6.01E-07	chr:20:12402387
20	rs186764661	12373373	T	29.03	6.22E-07	chr:20:12402387
20	rs2423670	12431787	A	0.3542	6.50E-07	chr:20:12402387
20	rs536548796	12566222	A	21.32	7.29E-07	chr:20:12402387
20	rs114184436	12409313	T	51.04	7.39E-07	chr:20:12402387
20	rs112010423	12265725	T	11.5	8.57E-07	chr:20:12402387
20	rs113420386	12259124	T	11.5	8.57E-07	chr:20:12402387
20	rs116741844	12390032	A	31.08	9.26E-07	chr:20:12402387
20	rs145800996	12294403	T	10.27	1.22E-06	chr:20:12402387
20	rs112240038	12493179	C	2.687	1.47E-06	chr:20:12402387
20	rs6041350	12419332	G	0.3755	1.59E-06	chr:20:12402387
20	rs372486425	12365849	C	44.19	1.59E-06	chr:20:12402387
20	rs6033392	12361355	T	3.602	1.74E-06	chr:20:12402387
20	rs6033407	12397454	T	0.3532	1.86E-06	chr:20:12402387
20	rs142054272	12168523	G	20.71	1.94E-06	chr:20:12402387
20	rs2423667	12430414	G	0.3293	1.96E-06	chr:20:12402387
20	rs2423668	12430673	C	0.3293	1.96E-06	chr:20:12402387

20	rs6078585	12428260	T	0.3293	1.96E-06	chr:20:12402387
20	rs6131338	12427932	T	0.3293	1.96E-06	chr:20:12402387
20	rs6131339	12427947	G	0.3293	1.96E-06	chr:20:12402387
20	rs16993185	12436775	T	0.3644	2.01E-06	chr:20:12402387
20	rs76871121	12488960	G	4.583	2.21E-06	chr:20:12402387
20	rs112814140	12326209	A	7.108	2.55E-06	chr:20:12402387
20	rs113352545	12326235	A	7.108	2.55E-06	chr:20:12402387
20	rs187957479	12529522	C	18.84	2.74E-06	chr:20:12402387
20	rs6109403	12421885	A	4.011	2.92E-06	chr:20:12402387
20	rs116158280	12389524	G	41.88	3.00E-06	chr:20:12402387
20	rs6109397	12401534	G	0.3934	3.27E-06	chr:20:12402387
20	rs6131334	12389853	T	3.872	3.50E-06	chr:20:12402387
20	rs113000297	12466277	G	0.2736	4.18E-06	chr:20:12402387
20	rs7264732	12481860	C	0.407	4.36E-06	chr:20:12402387
20	rs6041398	12478969	A	2.811	4.90E-06	chr:20:12402387
20	rs28458384	12371527	G	0.3534	4.93E-06	chr:20:12402387
20	rs6134540	12389312	C	0.2881	5.18E-06	chr:20:12402387
20	rs6134541	12389437	T	0.2881	5.18E-06	chr:20:12402387
20	rs184906859	12385101	C	39.62	5.84E-06	chr:20:12402387
20	rs6041395	12474644	T	2.772	5.88E-06	chr:20:12402387
20	rs6041396	12475525	C	2.772	5.88E-06	chr:20:12402387
20	rs6131348	12470922	A	2.772	5.88E-06	chr:20:12402387
20	rs6134573	12480470	T	2.772	5.88E-06	chr:20:12402387
20	rs6041318	12388209	A	2.367	7.12E-06	chr:20:12402387
20	rs747146	12396788	G	0.3843	7.46E-06	chr:20:12402387
20	rs2327547	12377703	A	0.377	7.48E-06	chr:20:12402387
20	rs137994263	12475296	C	2.699	7.59E-06	chr:20:12402387
20	rs142578022	12475299	G	2.699	7.59E-06	chr:20:12402387
20	rs28808402	12475302	T	2.699	7.59E-06	chr:20:12402387
20	rs6033405	12396625	A	0.3844	7.79E-06	chr:20:12402387
20	rs4814122	12452159	G	0.2788	8.10E-06	chr:20:12402387
20	rs6078613	12452346	C	0.2788	8.10E-06	chr:20:12402387
20	rs980615	12399726	A	0.303	8.29E-06	chr:20:12402387
20	rs6041378	12459832	G	0.304	8.78E-06	chr:20:12402387
20	rs6078558	12389058	C	2.339	8.81E-06	chr:20:12402387
20	rs6033434	12476495	G	0.2888	8.90E-06	chr:20:12402387
20	rs6104893	12390159	T	2.334	9.17E-06	chr:20:12402387
20	rs77577273	12179302	A	17.34	9.26E-06	chr:20:12402387
20	rs6134535	12375894	G	0.2565	1.08E-05	chr:20:12402387
20	rs6041374	12452895	T	2.599	1.16E-05	chr:20:12402387
20	rs6074421	12398729	G	2.313	1.35E-05	chr:20:12402387
20	rs6074420	12393920	G	0.3016	1.45E-05	chr:20:12402387
20	rs6041393	12473634	C	0.2927	1.47E-05	chr:20:12402387

20	rs6134567	12470891	C	0.3013	1.50E-05	chr:20:12402387
20	rs6078568	12409405	C	0.3243	1.59E-05	chr:20:12402387
20	rs6041381	12463411	G	0.3433	1.60E-05	chr:20:12402387
20	rs6134564	12457214	T	0.2917	1.61E-05	chr:20:12402387
20	rs6041380	12463039	G	0.3436	1.61E-05	chr:20:12402387
20	rs2208974	12395136	C	0.3733	1.62E-05	chr:20:12402387
20	rs2006906	12412378	A	0.3251	1.66E-05	chr:20:12402387
20	rs2208972	12384473	A	0.2879	1.70E-05	chr:20:12402387
20	rs6078627	12476999	G	0.3396	1.70E-05	chr:20:12402387
20	rs6041361	12429416	T	2.976	1.72E-05	chr:20:12402387
20	rs6041385	12464327	C	0.3039	1.75E-05	chr:20:12402387
20	rs7273044	12485868	T	0.4219	1.77E-05	chr:20:12402387
20	rs7273048	12485891	G	0.4219	1.77E-05	chr:20:12402387
20	rs7352320	12476943	C	0.3017	1.78E-05	chr:20:12402387
20	rs13044477	12391123	T	0.3064	1.79E-05	chr:20:12402387
20	rs111506184	12461299	G	2.504	1.83E-05	chr:20:12402387
20	rs59788428	12473097	T	2.504	1.83E-05	chr:20:12402387
20	rs6041394	12473710	C	2.504	1.83E-05	chr:20:12402387
20	rs6041391	12471984	T	0.3105	1.86E-05	chr:20:12402387
20	rs6078636	12485481	C	0.4029	1.87E-05	chr:20:12402387
20	rs6041375	12453961	C	0.3027	1.90E-05	chr:20:12402387
20	rs1156962	12465287	A	0.3058	1.90E-05	chr:20:12402387
20	rs4814118	12451752	T	0.2951	1.91E-05	chr:20:12402387
20	rs4814120	12451800	A	0.2951	1.91E-05	chr:20:12402387
20	rs139677565	12431206	A	37.46	1.98E-05	chr:20:12402387
20	rs141378564	12432111	G	37.46	1.98E-05	chr:20:12402387
20	rs145523895	12416152	G	37.46	1.98E-05	chr:20:12402387
20	rs146334973	12421169	G	37.46	1.98E-05	chr:20:12402387
20	rs147927851	12419846	A	37.46	1.98E-05	chr:20:12402387
20	rs149752488	12422163	A	37.46	1.98E-05	chr:20:12402387
20	rs552496158	12422634	A	37.46	1.98E-05	chr:20:12402387
20	rs7263958	12484678	C	2.767	1.99E-05	chr:20:12402387
20	rs185987507	12432233	A	37.45	2.00E-05	chr:20:12402387
20	rs6033435	12482080	C	0.3067	2.11E-05	chr:20:12402387
20	rs73252166	12465468	T	2.483	2.20E-05	chr:20:12402387
20	rs1009414	12368613	T	0.3117	2.33E-05	chr:20:12402387
20	rs6078574	12414684	C	0.3403	2.51E-05	chr:20:12402387
20	rs533883396	12605663	G	13.03	2.54E-05	chr:20:12402387
20	rs13043996	12493242	G	0.4271	2.78E-05	chr:20:12402387
20	rs13045325	12493233	A	0.4271	2.78E-05	chr:20:12402387
20	rs2024776	12492186	G	0.4297	3.04E-05	chr:20:12402387
20	rs2024777	12492241	A	0.4297	3.04E-05	chr:20:12402387
20	rs2024778	12492397	C	0.4297	3.04E-05	chr:20:12402387

20	rs2024779	12492398	A	0.4297	3.04E-05	chr:20:12402387
20	rs2024780	12492513	C	0.4297	3.04E-05	chr:20:12402387
20	rs2024781	12492607	T	0.4297	3.04E-05	chr:20:12402387
20	rs34825222	12491730	T	0.4297	3.04E-05	chr:20:12402387
20	rs141342790	12647822	A	3.574	3.13E-05	chr:20:12402387
20	rs75840140	25249311	C	0.3064	4.83E-09	chr:20:25215439
20	rs6037070	25241262	A	0.3038	5.50E-09	chr:20:25215439
20	rs6050510	25251815	T	0.306	6.41E-09	chr:20:25215439
20	rs11907515	25222282	A	0.3071	7.86E-09	chr:20:25215439
20	rs141382289	25222225	G	0.3071	7.86E-09	chr:20:25215439
20	rs28496800	25225171	A	0.3071	7.86E-09	chr:20:25215439
20	rs6050474	25220154	C	0.3071	7.86E-09	chr:20:25215439
20	rs6050476	25221010	C	0.3071	7.86E-09	chr:20:25215439
20	rs6050478	25221913	G	0.3071	7.86E-09	chr:20:25215439
20	rs7344105	25225945	G	0.3071	7.86E-09	chr:20:25215439
20	rs75374071	25226575	A	0.3071	7.86E-09	chr:20:25215439
20	rs111540814	25250437	C	0.3088	8.25E-09	chr:20:25215439
20	rs2387734	25248229	T	0.3088	8.25E-09	chr:20:25215439
20	rs28770021	25244243	C	0.3088	8.25E-09	chr:20:25215439
20	rs28881233	25244100	T	0.3088	8.25E-09	chr:20:25215439
20	rs6037071	25241483	G	0.3088	8.25E-09	chr:20:25215439
20	rs6037073	25245133	C	0.3088	8.25E-09	chr:20:25215439
20	rs6037075	25251855	A	0.3088	8.25E-09	chr:20:25215439
20	rs6050492	25241711	A	0.3088	8.25E-09	chr:20:25215439
20	rs6050493	25241749	G	0.3088	8.25E-09	chr:20:25215439
20	rs6050494	25242248	G	0.3088	8.25E-09	chr:20:25215439
20	rs6050502	25247253	C	0.3088	8.25E-09	chr:20:25215439
20	rs6050503	25247463	G	0.3088	8.25E-09	chr:20:25215439
20	rs6050504	25247557	G	0.3088	8.25E-09	chr:20:25215439
20	rs6050507	25250988	A	0.3088	8.25E-09	chr:20:25215439
20	rs6050508	25251190	G	0.3088	8.25E-09	chr:20:25215439
20	rs6050511	25252000	C	0.3088	8.25E-09	chr:20:25215439
20	rs7269045	25251580	G	0.3088	8.25E-09	chr:20:25215439
20	rs8118763	25239781	T	0.3088	8.25E-09	chr:20:25215439
20	rs8125437	25239780	G	0.3088	8.25E-09	chr:20:25215439
20	rs6050485	25229230	T	0.3049	9.74E-09	chr:20:25215439
20	rs6050486	25230841	G	0.3113	1.05E-08	chr:20:25215439
20	rs74407052	25238982	G	0.311	1.05E-08	chr:20:25215439
20	rs113949969	25237479	A	0.3122	1.17E-08	chr:20:25215439
20	rs116672236	25237036	C	0.3122	1.17E-08	chr:20:25215439
20	rs2145125	25231125	C	0.3122	1.17E-08	chr:20:25215439
20	rs2180596	25234777	A	0.3122	1.17E-08	chr:20:25215439
20	rs2180597	25235321	T	0.3122	1.17E-08	chr:20:25215439

20	rs6037068	25229589	T	0.3122	1.17E-08	chr:20:25215439
20	rs6050487	25230924	G	0.3122	1.17E-08	chr:20:25215439
20	rs6050488	25232353	C	0.3122	1.17E-08	chr:20:25215439
20	rs6050490	25238235	T	0.3122	1.17E-08	chr:20:25215439
20	rs74749140	25237767	C	0.3122	1.17E-08	chr:20:25215439
20	rs74967589	25234732	G	0.3122	1.17E-08	chr:20:25215439
20	rs77959670	25237615	T	0.3122	1.17E-08	chr:20:25215439
20	rs79311385	25233074	G	0.3122	1.17E-08	chr:20:25215439
20	rs79783587	25231486	C	0.3122	1.17E-08	chr:20:25215439
20	rs79986863	25237531	G	0.3122	1.17E-08	chr:20:25215439
20	rs8125980	25236501	C	0.3122	1.17E-08	chr:20:25215439
20	rs77769033	25235235	G	0.3126	1.25E-08	chr:20:25215439
20	rs6050509	25251796	T	0.3231	2.21E-08	chr:20:25215439
20	rs6050495	25242336	T	0.321	2.41E-08	chr:20:25215439
20	rs6050505	25248982	C	0.321	2.41E-08	chr:20:25215439
20	rs6050512	25252899	G	0.3354	3.05E-08	chr:20:25215439
20	rs7269479	25216156	T	0.3176	4.53E-08	chr:20:25215439
20	rs79719043	25237559	G	0.3305	5.18E-08	chr:20:25215439
20	rs6050473	25219993	G	0.3284	1.09E-07	chr:20:25215439
20	rs6037061	25202863	T	0.3468	2.04E-07	chr:20:25215439
20	rs7273544	25253996	A	0.3581	5.81E-07	chr:20:25215439
20	rs11906864	25203460	A	0.3603	6.17E-07	chr:20:25215439
20	rs11906892	25203627	A	0.3603	6.17E-07	chr:20:25215439
20	rs6050459	25206290	C	0.3603	6.17E-07	chr:20:25215439
20	rs111795731	25221821	T	0.4149	1.91E-06	chr:20:25215439
20	rs6050460	25206497	T	0.379	2.24E-06	chr:20:25215439
20	rs111895469	25210163	C	0.4111	2.55E-06	chr:20:25215439
20	rs7274919	25254095	T	0.4075	2.70E-06	chr:20:25215439
20	rs113746429	25210002	T	0.4154	3.39E-06	chr:20:25215439
20	rs6050457	25202639	G	0.4311	3.97E-06	chr:20:25215439
20	rs115475072	25651557	T	0.366	4.92E-06	chr:20:25215439
20	rs77415996	25646484	C	0.366	4.92E-06	chr:20:25215439
20	rs6050471	25218814	G	0.4315	6.05E-06	chr:20:25215439
20	rs2227894	25255338	T	0.4411	8.11E-06	chr:20:25215439
20	rs6050515	25255551	G	0.4411	8.11E-06	chr:20:25215439
20	rs6050470	25217083	A	0.4478	1.25E-05	chr:20:25215439
20	rs111599962	25211363	A	0.4522	1.50E-05	chr:20:25215439
20	rs115815357	25415232	G	0.3873	1.91E-05	chr:20:25215439
20	rs143705499	25381805	T	0.3873	1.91E-05	chr:20:25215439
20	rs111291203	25213040	G	0.4639	2.74E-05	chr:20:25215439
20	rs111496137	25209726	A	0.4639	2.74E-05	chr:20:25215439
20	rs112367052	25212962	A	0.4639	2.74E-05	chr:20:25215439
20	rs113155740	25212521	A	0.4639	2.74E-05	chr:20:25215439

20	rs6037064	25206894	C	0.4639	2.74E-05	chr:20:25215439
20	rs6050461	25207011	G	0.4639	2.74E-05	chr:20:25215439
20	rs6050462	25207722	A	0.4639	2.74E-05	chr:20:25215439
20	rs74630628	25210321	A	0.4639	2.74E-05	chr:20:25215439
20	rs76026090	25212428	C	0.4639	2.74E-05	chr:20:25215439
20	rs78169178	25211157	A	0.4639	2.74E-05	chr:20:25215439
20	rs7272959	25263756	A	0.4763	2.78E-05	chr:20:25215439
20	rs112837676	25214740	C	0.4643	2.81E-05	chr:20:25215439
20	rs145676605	25214659	G	0.4643	2.81E-05	chr:20:25215439
20	rs1044573	25206654	G	0.4919	2.93E-05	chr:20:25215439
20	rs45587239	25255415	T	0.4699	3.26E-05	chr:20:25215439
20	rs6050513	25254688	G	0.4693	3.58E-05	chr:20:25215439
3	rs6767408	125623666	T	3.39	2.20E-11	chr:3:125609298
3	rs6767618	125623941	G	3.39	2.20E-11	chr:3:125609298
3	rs74470729	125621098	T	3.39	2.20E-11	chr:3:125609298
3	rs79143534	125622688	G	3.39	2.20E-11	chr:3:125609298
3	rs79647471	125622158	A	3.39	2.20E-11	chr:3:125609298
3	rs11925437	125599216	A	3.4	2.23E-11	chr:3:125609298
3	rs12633485	125605577	T	3.4	2.23E-11	chr:3:125609298
3	rs12637469	125600407	T	3.4	2.23E-11	chr:3:125609298
3	rs6772193	125597432	A	3.4	2.23E-11	chr:3:125609298
3	rs75313595	125601479	A	3.4	2.23E-11	chr:3:125609298
3	rs75687938	125600858	T	3.4	2.23E-11	chr:3:125609298
3	rs77342220	125608463	T	3.4	2.23E-11	chr:3:125609298
3	rs78438430	125607169	C	3.4	2.23E-11	chr:3:125609298
3	rs79584539	125600024	T	3.4	2.23E-11	chr:3:125609298
3	rs79780713	125596589	G	3.4	2.23E-11	chr:3:125609298
3	rs75431854	125601942	A	3.343	3.50E-11	chr:3:125609298
3	rs9714319	125612678	T	3.314	3.84E-11	chr:3:125609298
3	rs200006127	125610368	G	3.288	5.24E-11	chr:3:125609298
3	rs74494751	125615642	G	3.288	5.24E-11	chr:3:125609298
3	rs78795877	125617617	T	3.288	5.24E-11	chr:3:125609298
3	rs138126565	125649194	A	3.023	5.80E-11	chr:3:125609298
3	rs11917669	125599068	T	3.298	5.91E-11	chr:3:125609298
3	rs3828357	125648356	C	2.973	8.06E-11	chr:3:125609298
3	rs76697478	125622811	A	3.256	8.35E-11	chr:3:125609298
3	rs60487455	125619863	G	3.171	1.49E-10	chr:3:125609298
3	rs57115172	125635679	A	0.3013	4.98E-10	chr:3:125609298
3	rs34721907	125637285	A	3.309	6.26E-10	chr:3:125609298
3	rs58198667	125635719	T	3.309	6.26E-10	chr:3:125609298
3	rs75837707	125630833	T	3.309	6.26E-10	chr:3:125609298
3	rs80022059	125641856	A	3.309	6.26E-10	chr:3:125609298
3	rs74402623	125643173	A	3.271	8.15E-10	chr:3:125609298

3	rs73858993	125633175	A	0.2931	8.69E-10	chr:3:125609298
3	rs12634512	125631766	T	2.815	8.98E-10	chr:3:125609298
3	rs138475236	125630752	G	0.3056	1.81E-09	chr:3:125609298
3	rs78614478	125631020	G	0.3056	1.81E-09	chr:3:125609298
3	rs11721242	125578642	T	3.336	1.82E-09	chr:3:125609298
3	rs141959419	125572267	G	3.336	1.82E-09	chr:3:125609298
3	rs145364321	125633605	T	0.3116	2.85E-09	chr:3:125609298
3	rs148981044	125621428	A	0.3159	4.97E-09	chr:3:125609298
3	rs2134521	125609297	A	0.3159	4.97E-09	chr:3:125609298
3	rs4263243	125609585	C	0.3159	4.97E-09	chr:3:125609298
3	rs4467397	125609586	A	0.3159	4.97E-09	chr:3:125609298
3	rs4574234	125609062	C	0.3159	4.97E-09	chr:3:125609298
3	rs57062978	125597586	G	0.3159	4.97E-09	chr:3:125609298
3	rs59221171	125622286	A	0.3159	4.97E-09	chr:3:125609298
3	rs145754213	125595882	A	0.3192	5.00E-09	chr:3:125609298
3	rs2979929	125541332	T	3.007	5.23E-09	chr:3:125609298
3	rs73858992	125633146	T	0.3258	5.53E-09	chr:3:125609298
3	rs2947690	125547912	A	2.98	5.88E-09	chr:3:125609298
3	rs573653999	125632831	A	0.3333	5.98E-09	chr:3:125609298
3	rs143065094	125643940	G	3.012	6.28E-09	chr:3:125609298
3	rs148384950	125631858	C	0.3254	6.80E-09	chr:3:125609298
3	rs115967814	125583032	T	0.3257	7.43E-09	chr:3:125609298
3	rs373861381	125585240	T	0.3257	7.43E-09	chr:3:125609298
3	rs57632767	125594520	G	0.3257	7.43E-09	chr:3:125609298
3	rs2979947	125552507	T	2.985	8.39E-09	chr:3:125609298
3	rs60374286	125630173	T	2.575	8.69E-09	chr:3:125609298
3	rs115617027	125635484	T	0.3372	1.01E-08	chr:3:125609298
3	rs140980442	125632547	A	0.3379	1.02E-08	chr:3:125609298
3	rs145326567	125632497	A	0.3379	1.02E-08	chr:3:125609298
3	rs145899983	125632793	G	0.3379	1.02E-08	chr:3:125609298
3	rs147699921	125632533	G	0.3379	1.02E-08	chr:3:125609298
3	rs138816824	125637500	C	0.3316	1.05E-08	chr:3:125609298
3	rs140011834	125592925	A	0.33	1.12E-08	chr:3:125609298
3	rs138110582	125626294	C	0.3847	1.13E-08	chr:3:125609298
3	rs55799821	125628440	G	0.3847	1.13E-08	chr:3:125609298
3	rs34209763	125599595	A	0.4488	1.19E-08	chr:3:125609298
3	rs144451371	125581114	T	0.3961	1.24E-08	chr:3:125609298
3	rs1976462	125628199	T	0.3914	1.36E-08	chr:3:125609298
3	rs111743409	125637546	C	0.344	1.55E-08	chr:3:125609298
3	rs140098024	125636854	G	0.344	1.55E-08	chr:3:125609298
3	rs140551936	125634217	A	0.344	1.55E-08	chr:3:125609298
3	rs144371013	125634299	G	0.344	1.55E-08	chr:3:125609298
3	rs147184359	125635840	T	0.344	1.55E-08	chr:3:125609298

3	rs147995646	125635637	T	0.344	1.55E-08	chr:3:125609298
3	rs181799363	125634788	T	0.344	1.55E-08	chr:3:125609298
3	rs182646434	125635472	T	0.344	1.55E-08	chr:3:125609298
3	rs186969735	125635531	T	0.344	1.55E-08	chr:3:125609298
3	rs2333375	125636956	A	0.344	1.55E-08	chr:3:125609298
3	rs61669680	125635949	A	0.344	1.55E-08	chr:3:125609298
3	rs112294864	125630740	T	0.3894	1.63E-08	chr:3:125609298
3	rs11711436	125629149	T	2.535	1.68E-08	chr:3:125609298
3	rs13319798	125605232	A	0.4519	1.70E-08	chr:3:125609298
3	rs35390120	125599809	A	0.4519	1.70E-08	chr:3:125609298
3	rs149422308	125593018	G	0.4006	1.81E-08	chr:3:125609298
3	rs58678427	125587076	G	0.4021	1.87E-08	chr:3:125609298
3	rs79344588	125632117	A	2.762	1.87E-08	chr:3:125609298
3	rs57811915	125628849	G	0.4058	1.89E-08	chr:3:125609298
3	rs145810852	125632668	A	0.3399	1.93E-08	chr:3:125609298
3	rs6775423	125598255	A	0.453	2.06E-08	chr:3:125609298
3	rs144234599	125627279	G	0.3965	2.18E-08	chr:3:125609298
3	rs111243139	125586513	T	0.4305	2.28E-08	chr:3:125609298
3	rs149454477	125582797	T	0.4305	2.28E-08	chr:3:125609298
3	rs56401450	125585471	A	0.4305	2.28E-08	chr:3:125609298
3	rs9758507	125616428	T	0.4589	2.64E-08	chr:3:125609298
3	rs16834637	125605471	T	0.4544	2.65E-08	chr:3:125609298
3	rs115941261	125626815	C	0.4007	3.12E-08	chr:3:125609298
3	rs141213249	125626084	C	0.4007	3.12E-08	chr:3:125609298
3	rs141277436	125627531	T	0.4007	3.12E-08	chr:3:125609298
3	rs144154148	125626708	C	0.4007	3.12E-08	chr:3:125609298
3	rs146378940	125627019	G	0.4007	3.12E-08	chr:3:125609298
3	rs148261048	125626762	A	0.4007	3.12E-08	chr:3:125609298
3	rs28590611	125630308	A	0.4007	3.12E-08	chr:3:125609298
3	rs55869762	125625263	G	0.4007	3.12E-08	chr:3:125609298
3	rs55902320	125625416	T	0.4007	3.12E-08	chr:3:125609298
3	rs56389777	125625327	A	0.4007	3.12E-08	chr:3:125609298
3	rs565164860	125626618	A	0.4007	3.12E-08	chr:3:125609298
3	rs58316089	125625520	A	0.4007	3.12E-08	chr:3:125609298
3	rs60975495	125628365	T	0.4007	3.12E-08	chr:3:125609298
3	rs72973373	125627659	A	0.4007	3.12E-08	chr:3:125609298
3	rs72973375	125629675	G	0.4007	3.12E-08	chr:3:125609298
3	rs73858989	125626195	C	0.4007	3.12E-08	chr:3:125609298
3	rs77486061	125626922	G	0.4007	3.12E-08	chr:3:125609298
3	rs571842515	125636115	G	0.3531	3.49E-08	chr:3:125609298
3	rs58654168	125635668	T	0.3531	3.49E-08	chr:3:125609298
3	rs140061761	125573221	A	2.575	3.73E-08	chr:3:125609298
3	rs143347021	125642315	G	0.3325	3.82E-08	chr:3:125609298

3	rs144610597	125637650	A	0.3325	3.82E-08	chr:3:125609298
3	rs4414795	125628001	T	0.4019	4.72E-08	chr:3:125609298
3	rs35321002	125612065	C	0.4615	5.45E-08	chr:3:125609298
3	rs75212493	125633523	C	2.508	5.65E-08	chr:3:125609298
3	rs574674152	125585290	T	0.3993	5.75E-08	chr:3:125609298
3	rs148099594	125590940	A	0.4244	6.02E-08	chr:3:125609298
3	rs115157504	125596364	T	0.337	6.53E-08	chr:3:125609298
3	rs116579261	125617222	T	0.337	6.53E-08	chr:3:125609298
3	rs143740725	125613881	A	0.337	6.53E-08	chr:3:125609298
3	rs149956886	125620247	T	0.337	6.53E-08	chr:3:125609298
3	rs151153255	125614821	A	0.337	6.53E-08	chr:3:125609298
3	rs2202238	125602871	A	0.337	6.53E-08	chr:3:125609298
3	rs7430375	125600976	C	0.337	6.53E-08	chr:3:125609298
3	rs55918505	125635660	A	0.3651	6.71E-08	chr:3:125609298
3	rs181960287	125634374	T	0.3468	6.89E-08	chr:3:125609298
3	rs139355261	125627118	A	0.415	7.99E-08	chr:3:125609298
3	rs2979977	125599006	T	2.019	9.52E-08	chr:3:125609298
3	rs56888543	125628470	A	0.4182	9.56E-08	chr:3:125609298
3	rs61479612	125628441	C	0.4182	9.56E-08	chr:3:125609298
3	rs13071955	125624345	C	0.482	1.03E-07	chr:3:125609298
3	rs13321217	125631771	A	0.5006	1.10E-07	chr:3:125609298
3	rs34507765	125612424	T	0.4889	1.17E-07	chr:3:125609298
3	rs115888633	125608013	G	0.3504	1.32E-07	chr:3:125609298
3	rs9832909	125588352	T	2.414	1.42E-07	chr:3:125609298
3	rs12492188	125582423	C	0.5063	1.72E-07	chr:3:125609298
3	rs2971298	125602713	A	0.4947	2.04E-07	chr:3:125609298
3	rs112567450	125592200	T	2.383	2.10E-07	chr:3:125609298
3	rs11922653	125591569	A	2.383	2.10E-07	chr:3:125609298
3	rs35540257	125590518	A	2.383	2.10E-07	chr:3:125609298
3	rs138581232	125587746	A	0.4653	2.64E-07	chr:3:125609298
3	rs59014841	125584640	A	2.356	2.93E-07	chr:3:125609298
3	rs7624806	125599074	A	0.5104	3.74E-07	chr:3:125609298
3	rs9873689	125611796	A	0.5025	3.77E-07	chr:3:125609298
3	rs149827337	125571747	A	0.4097	4.19E-07	chr:3:125609298
3	rs4306815	125633863	T	0.5238	4.30E-07	chr:3:125609298
3	rs11915699	125632684	G	0.5241	4.35E-07	chr:3:125609298
3	rs13088308	125632263	C	0.5241	4.35E-07	chr:3:125609298
3	rs2979934	125546915	T	2.326	4.45E-07	chr:3:125609298
3	rs9840926	125635356	C	0.5251	4.49E-07	chr:3:125609298
3	rs77524584	125558110	C	2.436	4.53E-07	chr:3:125609298
3	rs9848299	125557678	C	2.436	4.53E-07	chr:3:125609298
3	rs36074052	125554457	A	2.451	4.59E-07	chr:3:125609298
3	rs36052298	125554425	A	2.441	5.14E-07	chr:3:125609298

3	rs151185137	125559746	T	0.4451	5.51E-07	chr:3:125609298
3	rs146152284	125579209	A	0.4636	5.52E-07	chr:3:125609298
3	rs71327727	125632977	C	0.5253	5.62E-07	chr:3:125609298
3	rs143658483	125567789	A	0.4158	5.69E-07	chr:3:125609298
3	rs12498003	125636189	G	0.5294	5.98E-07	chr:3:125609298
3	rs4047922	125636595	G	0.5294	5.98E-07	chr:3:125609298
3	rs4047937	125636446	C	0.5294	5.98E-07	chr:3:125609298
3	rs7644102	125634825	C	0.5294	5.98E-07	chr:3:125609298
3	rs7644184	125634873	C	0.5294	5.98E-07	chr:3:125609298
3	rs9841142	125635541	A	0.5294	5.98E-07	chr:3:125609298
3	rs28603219	125581860	C	2.293	6.27E-07	chr:3:125609298
3	rs76765944	125554804	A	2.408	6.34E-07	chr:3:125609298
3	rs73858982	125579723	G	0.4873	6.47E-07	chr:3:125609298
3	rs148413344	125576954	A	0.4161	6.49E-07	chr:3:125609298
3	rs568638136	125573937	A	0.4161	6.49E-07	chr:3:125609298
3	rs57837602	125560471	C	0.4313	6.55E-07	chr:3:125609298
3	rs78718797	125556575	C	2.397	6.68E-07	chr:3:125609298
3	rs60014637	125642557	G	1.812	7.82E-07	chr:3:125609298
3	rs115956393	125569904	C	0.4089	8.29E-07	chr:3:125609298
3	rs115904560	125599999	T	0.3681	8.52E-07	chr:3:125609298
3	rs9859596	125593349	T	2.154	8.62E-07	chr:3:125609298
3	rs11914298	125643772	G	1.855	8.72E-07	chr:3:125609298
3	rs534418903	125573938	C	0.4212	8.98E-07	chr:3:125609298
3	rs79335314	125643915	C	0.3442	9.68E-07	chr:3:125609298
3	rs533185188	125574482	G	0.4193	9.85E-07	chr:3:125609298
3	rs563914125	125574480	A	0.4193	9.85E-07	chr:3:125609298
3	rs142956830	125574053	G	2.345	1.03E-06	chr:3:125609298
3	rs79330036	125586222	A	2.265	1.07E-06	chr:3:125609298
3	rs12695469	125635682	G	0.5395	1.13E-06	chr:3:125609298
3	rs13100358	125643202	G	1.92	1.29E-06	chr:3:125609298
3	rs6764584	125564726	A	2.305	1.41E-06	chr:3:125609298
3	rs75945393	125566855	T	2.305	1.41E-06	chr:3:125609298
3	rs77521522	125566651	A	2.305	1.41E-06	chr:3:125609298
3	rs9847495	125566153	A	2.305	1.41E-06	chr:3:125609298
3	rs113525069	125573023	G	2.301	1.60E-06	chr:3:125609298
3	rs11708613	125577595	C	2.301	1.60E-06	chr:3:125609298
3	rs11713267	125578350	T	2.301	1.60E-06	chr:3:125609298
3	rs11720087	125577524	T	2.301	1.60E-06	chr:3:125609298
3	rs143489229	125573812	G	2.301	1.60E-06	chr:3:125609298
3	rs2877920	125578987	A	2.301	1.60E-06	chr:3:125609298
3	rs57482370	125577906	A	2.301	1.60E-06	chr:3:125609298
3	rs7614815	125571436	T	2.301	1.60E-06	chr:3:125609298
3	rs79951679	125575493	T	2.301	1.60E-06	chr:3:125609298

3	rs9872316	125576232	T	2.301	1.60E-06	chr:3:125609298
3	rs9883344	125569292	A	2.301	1.60E-06	chr:3:125609298
3	rs80067333	125559464	T	2.343	1.63E-06	chr:3:125609298
3	rs190156584	125577438	T	0.4347	1.66E-06	chr:3:125609298
3	rs191463608	125645394	T	0.3327	1.66E-06	chr:3:125609298
3	rs2062773	125639648	T	1.872	1.75E-06	chr:3:125609298
3	rs2333384	125640487	G	1.872	1.75E-06	chr:3:125609298
3	rs59757827	125571036	A	0.5236	2.02E-06	chr:3:125609298
3	rs151134413	125618379	T	0.3083	2.07E-06	chr:3:125609298
3	rs114237101	125567024	A	0.4388	2.14E-06	chr:3:125609298
3	rs6797253	125643329	G	1.854	2.17E-06	chr:3:125609298
3	rs6772482	125643332	A	1.843	2.19E-06	chr:3:125609298
3	rs59259948	125642901	A	1.85	2.30E-06	chr:3:125609298
3	rs2089011	125640396	T	1.858	2.30E-06	chr:3:125609298
3	rs11921945	125643719	A	1.87	2.49E-06	chr:3:125609298
3	rs2947695	125551048	A	2.203	2.49E-06	chr:3:125609298
3	rs2947696	125551372	A	2.203	2.49E-06	chr:3:125609298
3	rs2947699	125552053	G	2.203	2.49E-06	chr:3:125609298
3	rs2947701	125553160	A	2.203	2.49E-06	chr:3:125609298
3	rs2971246	125552502	C	2.203	2.49E-06	chr:3:125609298
3	rs2971247	125553347	C	2.203	2.49E-06	chr:3:125609298
3	rs2979952	125553514	T	2.203	2.49E-06	chr:3:125609298
3	rs141307437	125584187	C	0.3898	2.60E-06	chr:3:125609298
3	rs10934731	125649804	A	0.3428	2.62E-06	chr:3:125609298
3	rs13094642	125642587	A	1.845	2.71E-06	chr:3:125609298
3	rs13094793	125642621	C	1.845	2.71E-06	chr:3:125609298
3	rs13095106	125642614	G	1.845	2.71E-06	chr:3:125609298
3	rs13315434	125641394	T	1.845	2.71E-06	chr:3:125609298
3	rs4340656	125642350	A	1.845	2.71E-06	chr:3:125609298
3	rs4377449	125642330	G	1.844	2.86E-06	chr:3:125609298
3	rs13099404	125642929	A	1.826	3.01E-06	chr:3:125609298
3	rs184564121	125629157	T	0.5059	4.00E-06	chr:3:125609298
3	rs13074445	125642534	T	1.767	4.68E-06	chr:3:125609298
3	rs6793220	125641894	C	1.808	4.74E-06	chr:3:125609298
3	rs2947694	125549914	C	2.116	4.85E-06	chr:3:125609298
3	rs142168300	125459601	G	4.338	5.43E-06	chr:3:125609298
3	rs2947693	125549639	A	2.132	6.59E-06	chr:3:125609298
3	rs11706818	125579822	A	2.055	6.75E-06	chr:3:125609298
3	rs4465900	125580464	T	2.055	6.75E-06	chr:3:125609298
3	rs142313792	125477703	T	4.274	7.00E-06	chr:3:125609298
3	rs138332423	125557124	T	0.4673	7.17E-06	chr:3:125609298
3	rs138647908	125563160	A	0.3358	7.58E-06	chr:3:125609298
3	rs4334594	125642101	A	1.79	7.86E-06	chr:3:125609298

3	rs141585111	125560472	A	0.4635	8.01E-06	chr:3:125609298
3	rs151206333	125447850	T	4.222	8.03E-06	chr:3:125609298
3	rs188079237	125439780	A	4.222	8.03E-06	chr:3:125609298
3	rs4679462	125449701	A	4.222	8.03E-06	chr:3:125609298
3	rs2062772	125639885	C	1.779	9.22E-06	chr:3:125609298
3	rs2333387	125640610	G	1.779	9.22E-06	chr:3:125609298
3	rs28541533	125640490	C	1.779	9.22E-06	chr:3:125609298
3	rs62270469	125468544	C	3.641	9.66E-06	chr:3:125609298
3	rs62270472	125472187	T	3.641	9.66E-06	chr:3:125609298
3	rs62271932	125461055	T	3.641	9.66E-06	chr:3:125609298
3	rs60465123	125465458	T	0.5938	9.66E-06	chr:3:125609298
3	rs115135873	125551405	A	0.4705	9.90E-06	chr:3:125609298
3	rs143210907	125553118	G	0.4705	9.90E-06	chr:3:125609298
3	rs34428329	125552195	T	0.4705	9.90E-06	chr:3:125609298
3	rs35665654	125552213	C	0.4705	9.90E-06	chr:3:125609298
3	rs55797702	125624481	A	0.5261	1.02E-05	chr:3:125609298
3	rs60925467	125624482	A	0.5261	1.02E-05	chr:3:125609298
3	rs74589163	125651239	C	0.49	1.03E-05	chr:3:125609298
3	rs148827978	125461946	A	0.5694	1.04E-05	chr:3:125609298
3	rs73858994	125643933	G	1.837	1.06E-05	chr:3:125609298
3	rs146002711	125541483	A	0.4724	1.07E-05	chr:3:125609298
3	rs199860792	125459352	C	0.5832	1.11E-05	chr:3:125609298
3	rs116610378	125540021	T	0.4723	1.18E-05	chr:3:125609298
3	rs146575920	125560345	A	0.4711	1.21E-05	chr:3:125609298
3	rs28893337	125462572	T	0.5775	1.22E-05	chr:3:125609298
3	rs60718422	125624343	C	0.5179	1.22E-05	chr:3:125609298
3	rs28410080	125630331	G	0.5698	1.26E-05	chr:3:125609298
3	rs140492652	125560325	A	0.4739	1.29E-05	chr:3:125609298
3	rs112466111	125466546	G	0.639	1.34E-05	chr:3:125609298
3	rs113990589	125466549	C	0.639	1.34E-05	chr:3:125609298
3	rs2947651	125454228	A	0.5768	1.34E-05	chr:3:125609298
3	rs35396288	125640852	C	1.757	1.34E-05	chr:3:125609298
3	rs4047900	125640973	A	1.757	1.34E-05	chr:3:125609298
3	rs112104357	125464294	A	0.5832	1.41E-05	chr:3:125609298
3	rs148709673	125540599	A	0.4789	1.44E-05	chr:3:125609298
3	rs57481916	125549584	C	0.4789	1.44E-05	chr:3:125609298
3	rs112627655	125564745	A	0.546	1.47E-05	chr:3:125609298
3	rs147266302	125564877	T	0.546	1.47E-05	chr:3:125609298
3	rs147918515	125564955	T	0.546	1.47E-05	chr:3:125609298
3	rs111677873	125562984	T	0.5461	1.48E-05	chr:3:125609298
3	rs7622672	125459012	C	0.5835	1.51E-05	chr:3:125609298
3	rs2976825	125454769	T	0.581	1.56E-05	chr:3:125609298
3	rs2971275	125452679	A	0.5827	1.56E-05	chr:3:125609298

3	rs2947649	125453782	T	0.5813	1.66E-05	chr:3:125609298
3	rs2976821	125454299	T	0.5821	1.74E-05	chr:3:125609298
3	rs11916518	125462329	T	0.5852	1.76E-05	chr:3:125609298
3	rs35029093	125454162	G	0.5933	1.77E-05	chr:3:125609298
3	rs138718192	125464260	T	0.5868	1.81E-05	chr:3:125609298
3	rs2971254	125560461	A	1.74	1.83E-05	chr:3:125609298
3	rs12485974	125644009	G	1.739	1.85E-05	chr:3:125609298
3	rs2971272	125451353	T	0.5847	1.87E-05	chr:3:125609298
3	rs2976808	125451344	G	0.5847	1.87E-05	chr:3:125609298
3	rs112116911	125569717	G	0.5565	1.88E-05	chr:3:125609298
3	rs112824336	125569001	A	0.5565	1.88E-05	chr:3:125609298
3	rs113695174	125569248	A	0.5565	1.88E-05	chr:3:125609298
3	rs13079414	125642936	T	1.741	1.93E-05	chr:3:125609298
3	rs9825926	125454681	A	0.5785	1.93E-05	chr:3:125609298
3	rs144324739	125571352	G	0.4571	1.93E-05	chr:3:125609298
3	rs28699626	125630320	A	0.5767	1.94E-05	chr:3:125609298
3	rs7622267	125458591	C	0.5881	1.94E-05	chr:3:125609298
3	rs138407862	125461945	C	0.5953	1.95E-05	chr:3:125609298
3	rs11714778	129762082	C	0.2834	2.05E-12	chr:3:129776131
3	rs73208686	129762898	C	0.2834	2.05E-12	chr:3:129776131
3	rs12495049	129810234	T	0.3024	4.09E-11	chr:3:129776131
3	rs11718709	129811323	A	0.3132	6.35E-11	chr:3:129776131
3	rs111717650	129814301	A	0.3237	7.92E-11	chr:3:129776131
3	rs73210759	129813745	T	0.3237	7.92E-11	chr:3:129776131
3	rs11716622	129809592	A	0.2984	2.82E-09	chr:3:129776131
3	rs12634803	129807284	T	0.4623	1.17E-07	chr:3:129776131
3	rs146549952	129816552	T	0.3994	1.44E-06	chr:3:129776131
3	rs9713334	129822494	T	0.4118	2.71E-06	chr:3:129776131
3	rs150822181	129756230	T	0.3471	1.38E-05	chr:3:129776131
3	rs9758049	129814863	A	0.3969	1.55E-05	chr:3:129776131
3	rs150790892	129752818	T	0.3499	1.56E-05	chr:3:129776131
3	rs55891359	129760185	T	0.5504	1.72E-05	chr:3:129776131
3	rs9713340	129822492	A	0.5002	3.35E-05	chr:3:129776131
3	rs111649280	14112363	C	0.03653	1.61E-21	chr:3:14132684
3	rs9815552	14112593	C	0.03653	1.61E-21	chr:3:14132684
3	rs9812004	14145704	A	0.0538	7.80E-21	chr:3:14132684
3	rs140999500	14146696	G	0.06186	9.47E-21	chr:3:14132684
3	rs78278386	14144792	T	0.06186	9.47E-21	chr:3:14132684
3	rs150494767	14134606	G	0.03713	1.38E-20	chr:3:14132684
3	rs372874849	14134618	C	0.03713	1.38E-20	chr:3:14132684
3	rs6800440	14126660	A	0.03228	1.94E-20	chr:3:14132684
3	rs6775826	14126847	C	0.03003	1.99E-20	chr:3:14132684
3	rs9310438	14105048	T	0.03143	2.18E-20	chr:3:14132684

3	rs569399702	14134859	A	0.03256	2.56E-20	chr:3:14132684
3	rs13314202	14141969	A	0.03248	2.63E-20	chr:3:14132684
3	rs13325293	14142078	A	0.03248	2.63E-20	chr:3:14132684
3	rs13326583	14143915	A	0.03248	2.63E-20	chr:3:14132684
3	rs28590293	14137568	T	0.03248	2.63E-20	chr:3:14132684
3	rs28657962	14137572	C	0.03248	2.63E-20	chr:3:14132684
3	rs28691764	14137871	T	0.03248	2.63E-20	chr:3:14132684
3	rs28729783	14137587	C	0.03248	2.63E-20	chr:3:14132684
3	rs6767978	14136755	A	0.03248	2.63E-20	chr:3:14132684
3	rs6768033	14137003	A	0.03248	2.63E-20	chr:3:14132684
3	rs6768047	14137044	T	0.03248	2.63E-20	chr:3:14132684
3	rs6770834	14137266	A	0.03248	2.63E-20	chr:3:14132684
3	rs6770939	14137398	C	0.03248	2.63E-20	chr:3:14132684
3	rs6774176	14138199	A	0.03248	2.63E-20	chr:3:14132684
3	rs6774208	14138377	G	0.03248	2.63E-20	chr:3:14132684
3	rs6780911	14137037	A	0.03248	2.63E-20	chr:3:14132684
3	rs6781010	14137119	C	0.03248	2.63E-20	chr:3:14132684
3	rs6787198	14138394	C	0.03248	2.63E-20	chr:3:14132684
3	rs6792123	14136737	G	0.03248	2.63E-20	chr:3:14132684
3	rs9824627	14135965	G	0.03248	2.63E-20	chr:3:14132684
3	rs9829180	14136563	T	0.03248	2.63E-20	chr:3:14132684
3	rs9844764	14139269	G	0.03248	2.63E-20	chr:3:14132684
3	rs9866433	14136054	A	0.03248	2.63E-20	chr:3:14132684
3	rs9866963	14136380	A	0.03248	2.63E-20	chr:3:14132684
3	rs1816425	13982518	G	0.04999	2.71E-20	chr:3:14132684
3	rs12330591	13981061	C	0.05016	2.79E-20	chr:3:14132684
3	rs1816424	13982379	T	0.05016	2.79E-20	chr:3:14132684
3	rs28810528	13991453	A	0.05016	2.79E-20	chr:3:14132684
3	rs9816802	13973637	T	0.05016	2.79E-20	chr:3:14132684
3	rs9825702	13980411	T	0.05016	2.79E-20	chr:3:14132684
3	rs9842881	13978272	T	0.05016	2.79E-20	chr:3:14132684
3	rs9863071	13987353	A	0.05016	2.79E-20	chr:3:14132684
3	rs9816646	13973502	G	0.05508	2.92E-20	chr:3:14132684
3	rs9867949	14009276	C	0.05821	3.79E-20	chr:3:14132684
3	rs6763196	14131474	G	0.02801	4.68E-20	chr:3:14132684
3	rs114404429	14100931	C	0.02786	4.72E-20	chr:3:14132684
3	rs9841035	14101872	T	0.02786	4.72E-20	chr:3:14132684
3	rs9841145	14101782	C	0.02786	4.72E-20	chr:3:14132684
3	rs9878216	14101678	G	0.02786	4.72E-20	chr:3:14132684
3	rs10510425	14131634	T	0.02803	5.03E-20	chr:3:14132684
3	rs11128682	14135592	G	0.02803	5.03E-20	chr:3:14132684
3	rs111878348	14124270	G	0.02803	5.03E-20	chr:3:14132684
3	rs113510537	14127749	A	0.02803	5.03E-20	chr:3:14132684

3	rs113797200	14133687	T	0.02803	5.03E-20	chr:3:14132684
3	rs116434506	14124497	G	0.02803	5.03E-20	chr:3:14132684
3	rs137878649	14127432	C	0.02803	5.03E-20	chr:3:14132684
3	rs138479933	14127602	T	0.02803	5.03E-20	chr:3:14132684
3	rs144847211	14124364	T	0.02803	5.03E-20	chr:3:14132684
3	rs149488650	14127543	G	0.02803	5.03E-20	chr:3:14132684
3	rs150291853	14127391	C	0.02803	5.03E-20	chr:3:14132684
3	rs17038878	14126963	A	0.02803	5.03E-20	chr:3:14132684
3	rs17038888	14128537	G	0.02803	5.03E-20	chr:3:14132684
3	rs17038890	14128694	A	0.02803	5.03E-20	chr:3:14132684
3	rs17038897	14133753	G	0.02803	5.03E-20	chr:3:14132684
3	rs17038898	14133938	A	0.02803	5.03E-20	chr:3:14132684
3	rs28413653	14131866	T	0.02803	5.03E-20	chr:3:14132684
3	rs28459558	14131867	G	0.02803	5.03E-20	chr:3:14132684
3	rs57419163	14129180	C	0.02803	5.03E-20	chr:3:14132684
3	rs60912473	14129207	A	0.02803	5.03E-20	chr:3:14132684
3	rs6764655	14126881	C	0.02803	5.03E-20	chr:3:14132684
3	rs6765773	14130989	A	0.02803	5.03E-20	chr:3:14132684
3	rs6774501	14131459	G	0.02803	5.03E-20	chr:3:14132684
3	rs6800207	14126643	A	0.02803	5.03E-20	chr:3:14132684
3	rs75472977	14134186	G	0.02803	5.03E-20	chr:3:14132684
3	rs76800068	14127818	T	0.02803	5.03E-20	chr:3:14132684
3	rs76802227	14135538	A	0.02803	5.03E-20	chr:3:14132684
3	rs78737595	14127206	G	0.02803	5.03E-20	chr:3:14132684
3	rs79248858	14134365	C	0.02803	5.03E-20	chr:3:14132684
3	rs79551783	14127817	A	0.02803	5.03E-20	chr:3:14132684
3	rs79844615	14133898	C	0.02803	5.03E-20	chr:3:14132684
3	rs9814706	14128484	G	0.02803	5.03E-20	chr:3:14132684
3	rs9820661	14129934	G	0.02803	5.03E-20	chr:3:14132684
3	rs9823101	14135029	G	0.02803	5.03E-20	chr:3:14132684
3	rs9826257	14123547	A	0.02803	5.03E-20	chr:3:14132684
3	rs9835016	14130585	T	0.02803	5.03E-20	chr:3:14132684
3	rs9835296	14130573	A	0.02803	5.03E-20	chr:3:14132684
3	rs9851936	14128139	T	0.02803	5.03E-20	chr:3:14132684
3	rs9855211	14130686	C	0.02803	5.03E-20	chr:3:14132684
3	rs9855536	14130891	C	0.02803	5.03E-20	chr:3:14132684
3	rs9857759	14129422	T	0.02803	5.03E-20	chr:3:14132684
3	rs9857881	14122390	G	0.02803	5.03E-20	chr:3:14132684
3	rs9859522	14130999	G	0.02803	5.03E-20	chr:3:14132684
3	rs9865779	14132640	C	0.02803	5.03E-20	chr:3:14132684
3	rs9871949	14128174	C	0.02803	5.03E-20	chr:3:14132684
3	rs9872093	14128271	C	0.02803	5.03E-20	chr:3:14132684
3	rs9872723	14130606	C	0.02803	5.03E-20	chr:3:14132684

3	rs9877696	14129399	C	0.02803	5.03E-20	chr:3:14132684
3	rs9878167	14129703	C	0.02803	5.03E-20	chr:3:14132684
3	rs144390287	14114504	G	0.02797	5.06E-20	chr:3:14132684
3	rs28629229	14109145	G	0.02797	5.06E-20	chr:3:14132684
3	rs4459883	14121381	C	0.02797	5.06E-20	chr:3:14132684
3	rs4603938	14121539	A	0.02797	5.06E-20	chr:3:14132684
3	rs9857415	14104960	T	0.02797	5.06E-20	chr:3:14132684
3	rs9872481	14107553	T	0.02797	5.06E-20	chr:3:14132684
3	rs112864358	13973257	A	0.05235	5.22E-20	chr:3:14132684
3	rs28729904	13992324	G	0.04658	5.28E-20	chr:3:14132684
3	rs115977684	14134096	T	0.02803	5.29E-20	chr:3:14132684
3	rs77316475	14089067	C	0.04706	5.87E-20	chr:3:14132684
3	rs9810909	14090930	A	0.04706	5.87E-20	chr:3:14132684
3	rs9812023	14085576	C	0.04706	5.87E-20	chr:3:14132684
3	rs9849894	14085904	G	0.04706	5.87E-20	chr:3:14132684
3	rs2101447	13992784	T	0.04656	5.97E-20	chr:3:14132684
3	rs28441118	14014066	T	0.04656	5.97E-20	chr:3:14132684
3	rs4435622	13996248	C	0.04656	5.97E-20	chr:3:14132684
3	rs77194165	14019828	T	0.04656	5.97E-20	chr:3:14132684
3	rs77801428	14017928	T	0.04656	5.97E-20	chr:3:14132684
3	rs80033779	14007913	C	0.04656	5.97E-20	chr:3:14132684
3	rs9848315	14009228	A	0.04656	5.97E-20	chr:3:14132684
3	rs9849692	13997131	G	0.04656	5.97E-20	chr:3:14132684
3	rs9862470	13992947	A	0.04656	5.97E-20	chr:3:14132684
3	rs9863644	14008860	C	0.04656	5.97E-20	chr:3:14132684
3	rs13318940	14100831	A	0.04708	6.34E-20	chr:3:14132684
3	rs2085488	14083660	G	0.04725	6.40E-20	chr:3:14132684
3	rs113645764	14006276	T	0.04675	6.53E-20	chr:3:14132684
3	rs28824550	14016250	A	0.04675	6.53E-20	chr:3:14132684
3	rs4047229	14004277	G	0.04675	6.53E-20	chr:3:14132684
3	rs9817521	14028186	A	0.04675	6.53E-20	chr:3:14132684
3	rs9822017	14004685	T	0.04675	6.53E-20	chr:3:14132684
3	rs9829954	14018283	G	0.04675	6.53E-20	chr:3:14132684
3	rs9833209	14006848	A	0.04675	6.53E-20	chr:3:14132684
3	rs9837062	14007143	T	0.04675	6.53E-20	chr:3:14132684
3	rs9842826	14008193	A	0.04675	6.53E-20	chr:3:14132684
3	rs9863652	14010799	G	0.04675	6.53E-20	chr:3:14132684
3	rs9852507	14091226	G	0.04642	6.65E-20	chr:3:14132684
3	rs28884184	14066612	C	0.04705	7.63E-20	chr:3:14132684
3	rs6803143	14044421	A	0.04705	7.63E-20	chr:3:14132684
3	rs7428486	14078749	T	0.04705	7.63E-20	chr:3:14132684
3	rs9817658	14049363	C	0.04705	7.63E-20	chr:3:14132684
3	rs9840631	14071508	T	0.04705	7.63E-20	chr:3:14132684

3	rs9867120	14076266	A	0.04705	7.63E-20	chr:3:14132684
3	rs9880508	14052114	C	0.04705	7.63E-20	chr:3:14132684
3	rs9823450	14052571	G	0.04701	7.87E-20	chr:3:14132684
3	rs13322742	14104301	A	0.04708	8.29E-20	chr:3:14132684
3	rs150787267	14116452	G	0.04708	8.29E-20	chr:3:14132684
3	rs183483454	14116524	C	0.04708	8.29E-20	chr:3:14132684
3	rs28696078	14103504	A	0.04708	8.29E-20	chr:3:14132684
3	rs12108069	14055655	A	0.04724	8.34E-20	chr:3:14132684
3	rs12330658	14046295	T	0.04724	8.34E-20	chr:3:14132684
3	rs28590512	14080655	G	0.04724	8.34E-20	chr:3:14132684
3	rs6767254	14044385	G	0.04724	8.34E-20	chr:3:14132684
3	rs6776717	14061611	T	0.04724	8.34E-20	chr:3:14132684
3	rs9810787	14053241	A	0.04724	8.34E-20	chr:3:14132684
3	rs9812495	14057393	C	0.04724	8.34E-20	chr:3:14132684
3	rs9831699	14064613	T	0.04724	8.34E-20	chr:3:14132684
3	rs9865978	14053226	T	0.04734	9.02E-20	chr:3:14132684
3	rs7611337	14043474	G	0.04724	9.75E-20	chr:3:14132684
3	rs28761305	13998961	T	0.04822	9.88E-20	chr:3:14132684
3	rs7633184	13992358	A	14.38	1.26E-19	chr:3:14132684
3	rs6797200	14067250	A	0.05181	1.85E-19	chr:3:14132684
3	rs114748241	13997390	A	0.04991	2.06E-19	chr:3:14132684
3	rs9876468	14134697	C	0.03649	4.09E-19	chr:3:14132684
3	rs6767928	14136907	G	0.09675	1.64E-17	chr:3:14132684
3	rs145085143	14037234	T	0.09896	1.88E-17	chr:3:14132684
3	rs142887561	14021272	T	0.09611	3.15E-17	chr:3:14132684
3	rs76773010	14019928	G	0.09611	3.15E-17	chr:3:14132684
3	rs77000306	14017655	T	0.09611	3.15E-17	chr:3:14132684
3	rs79849273	14006999	A	0.09611	3.15E-17	chr:3:14132684
3	rs181388240	14032254	C	0.09021	3.51E-17	chr:3:14132684
3	rs111462297	14025712	G	0.09851	3.87E-17	chr:3:14132684
3	rs111981004	14012555	T	0.09851	3.87E-17	chr:3:14132684
3	rs112071682	14038589	G	0.09851	3.87E-17	chr:3:14132684
3	rs112594602	14027090	G	0.09851	3.87E-17	chr:3:14132684
3	rs113226436	14023913	C	0.09851	3.87E-17	chr:3:14132684
3	rs139015636	14035616	G	0.09851	3.87E-17	chr:3:14132684
3	rs112085579	14084458	G	0.1028	4.58E-17	chr:3:14132684
3	rs112507992	14099531	A	0.1028	4.58E-17	chr:3:14132684
3	rs17038849	14093586	A	0.1028	4.58E-17	chr:3:14132684
3	rs28870410	14085234	T	0.1028	4.58E-17	chr:3:14132684
3	rs74323071	14084576	C	0.1028	4.58E-17	chr:3:14132684
3	rs868305	14087896	A	0.1028	4.58E-17	chr:3:14132684
3	rs113152170	13984589	A	0.1037	6.21E-17	chr:3:14132684
3	rs17038787	13978137	T	0.1037	6.21E-17	chr:3:14132684

3	rs112225787	14118633	G	0.1022	6.38E-17	chr:3:14132684
3	rs112488436	14110763	T	0.1022	6.38E-17	chr:3:14132684
3	rs113237279	14105023	G	0.1022	6.38E-17	chr:3:14132684
3	rs113415393	14049786	A	0.1022	6.38E-17	chr:3:14132684
3	rs113842726	14131971	G	0.1022	6.38E-17	chr:3:14132684
3	rs139614460	14112839	G	0.1022	6.38E-17	chr:3:14132684
3	rs145715963	14115465	G	0.1022	6.38E-17	chr:3:14132684
3	rs183941725	14117290	G	0.1022	6.38E-17	chr:3:14132684
3	rs558409036	14106203	A	0.1022	6.38E-17	chr:3:14132684
3	rs6800457	14044027	A	0.1022	6.38E-17	chr:3:14132684
3	rs77497299	14080626	G	0.1022	6.38E-17	chr:3:14132684
3	rs77513529	14047268	G	0.1022	6.38E-17	chr:3:14132684
3	rs78283775	14109923	T	0.1022	6.38E-17	chr:3:14132684
3	rs78379417	14064551	A	0.1022	6.38E-17	chr:3:14132684
3	rs80026241	14058780	T	0.1022	6.38E-17	chr:3:14132684
3	rs80039308	14134322	G	0.1022	6.38E-17	chr:3:14132684
3	rs80136382	14069151	G	0.1022	6.38E-17	chr:3:14132684
3	rs116446927	13952877	T	0.1061	7.58E-17	chr:3:14132684
3	rs28378280	13981010	G	0.1061	7.58E-17	chr:3:14132684
3	rs137936132	14023282	C	0.09062	9.04E-17	chr:3:14132684
3	rs149445713	14092523	G	0.1114	9.10E-17	chr:3:14132684
3	rs535198208	14106202	A	0.1044	1.03E-16	chr:3:14132684
3	rs75217305	14080912	A	0.09821	1.54E-16	chr:3:14132684
3	rs9756008	14115991	C	0.1134	1.56E-16	chr:3:14132684
3	rs6790932	14141537	G	0.1092	1.80E-16	chr:3:14132684
3	rs112384621	14055310	A	0.09977	2.08E-16	chr:3:14132684
3	rs113771892	13985798	T	0.09866	3.14E-16	chr:3:14132684
3	rs149117984	13985955	A	0.09866	3.14E-16	chr:3:14132684
3	rs567859105	13988444	T	0.09866	3.14E-16	chr:3:14132684
3	rs4487225	14079133	A	0.09938	3.71E-16	chr:3:14132684
3	rs9866178	14023272	T	0.1561	4.78E-16	chr:3:14132684
3	rs1077525	13929225	C	0.1189	5.55E-16	chr:3:14132684
3	rs564631976	14117370	G	0.1152	7.24E-16	chr:3:14132684
3	rs6783302	14063432	C	0.1604	1.83E-15	chr:3:14132684
3	rs570206947	14096727	A	0.1229	2.81E-15	chr:3:14132684
3	rs2731323	14125860	C	0.03776	5.25E-15	chr:3:14132684
3	rs189040807	14035562	T	0.1411	8.45E-15	chr:3:14132684
3	rs61976959	13968603	T	0.1138	1.19E-14	chr:3:14132684
3	rs9863367	14123434	G	0.1024	1.39E-13	chr:3:14132684
3	rs4685070	14126238	T	7.09	1.04E-12	chr:3:14132684
3	rs2879889	14100627	T	7.076	1.05E-12	chr:3:14132684
3	rs2607741	14147892	C	0.2138	2.83E-12	chr:3:14132684
3	rs79296261	13961704	A	0.2065	3.74E-12	chr:3:14132684

3	rs9859264	14147316	G	0.1682	3.78E-12	chr:3:14132684
3	rs9883700	13926173	T	0.1689	7.21E-12	chr:3:14132684
3	rs141104612	14029807	T	0.1484	8.79E-12	chr:3:14132684
3	rs147847103	14032386	G	0.1484	8.79E-12	chr:3:14132684
3	rs150975467	14031399	C	0.1484	8.79E-12	chr:3:14132684
3	rs114894105	13977047	T	0.1491	9.15E-12	chr:3:14132684
3	rs114253217	14013750	A	0.1515	1.23E-11	chr:3:14132684
3	rs115249832	14013505	T	0.1515	1.23E-11	chr:3:14132684
3	rs116745088	14013702	T	0.1515	1.23E-11	chr:3:14132684
3	rs13323884	14075680	C	0.1515	1.23E-11	chr:3:14132684
3	rs13326796	14025875	A	0.1515	1.23E-11	chr:3:14132684
3	rs140684053	14037007	A	0.1515	1.23E-11	chr:3:14132684
3	rs143632765	14001634	A	0.1515	1.23E-11	chr:3:14132684
3	rs184888567	14035078	G	0.1515	1.23E-11	chr:3:14132684
3	rs28786533	14014309	G	0.1515	1.23E-11	chr:3:14132684
3	rs28817689	14068151	T	0.1515	1.23E-11	chr:3:14132684
3	rs549372188	14000614	C	0.1515	1.23E-11	chr:3:14132684
3	rs60724048	14058133	A	0.1515	1.23E-11	chr:3:14132684
3	rs74696324	14087986	A	0.1515	1.23E-11	chr:3:14132684
3	rs9810605	14023044	C	0.1515	1.23E-11	chr:3:14132684
3	rs9811854	14085401	A	0.1515	1.23E-11	chr:3:14132684
3	rs9818209	14051646	G	0.1515	1.23E-11	chr:3:14132684
3	rs9818431	14058942	A	0.1515	1.23E-11	chr:3:14132684
3	rs9823761	14022668	G	0.1515	1.23E-11	chr:3:14132684
3	rs9824059	13992720	G	0.1515	1.23E-11	chr:3:14132684
3	rs9832794	13976688	T	0.1515	1.23E-11	chr:3:14132684
3	rs9843754	14084399	G	0.1515	1.23E-11	chr:3:14132684
3	rs9857436	14069069	G	0.1515	1.23E-11	chr:3:14132684
3	rs9862109	13992794	A	0.1515	1.23E-11	chr:3:14132684
3	rs9863140	14047776	T	0.1515	1.23E-11	chr:3:14132684
3	rs9870470	13976748	G	0.1515	1.23E-11	chr:3:14132684
3	rs9876149	14000562	T	0.1515	1.23E-11	chr:3:14132684
3	rs9876776	13995109	T	0.1515	1.23E-11	chr:3:14132684
3	rs4684181	14126626	T	5.308	1.28E-11	chr:3:14132684
3	rs78541509	14126272	A	0.1726	1.39E-11	chr:3:14132684
3	rs9850096	13939593	T	0.1526	1.41E-11	chr:3:14132684
3	rs1816428	13962544	G	0.15	1.61E-11	chr:3:14132684
3	rs2607779	14106499	A	4.951	1.65E-11	chr:3:14132684
3	rs6773588	14113458	A	4.951	1.65E-11	chr:3:14132684
3	rs577819290	14113432	C	0.174	1.71E-11	chr:3:14132684
3	rs59247191	14115568	T	0.174	1.71E-11	chr:3:14132684
3	rs115668091	14103512	A	0.1547	1.74E-11	chr:3:14132684
3	rs13317006	14123362	T	0.1547	1.74E-11	chr:3:14132684

3	rs137910229	14116926	C	0.1547	1.74E-11	chr:3:14132684
3	rs1840944	14109740	A	0.1547	1.74E-11	chr:3:14132684
3	rs77845295	14127928	C	0.1547	1.74E-11	chr:3:14132684
3	rs78496810	14126389	A	0.1547	1.74E-11	chr:3:14132684
3	rs9864588	14141076	A	0.1547	1.74E-11	chr:3:14132684
3	rs9865105	14132215	C	0.1547	1.74E-11	chr:3:14132684
3	rs9866363	14136194	T	0.1547	1.74E-11	chr:3:14132684
3	rs9866424	14102935	C	0.1547	1.74E-11	chr:3:14132684
3	rs9990360	14107071	T	0.1547	1.74E-11	chr:3:14132684
3	rs2607778	14108543	A	4.916	1.93E-11	chr:3:14132684
3	rs4607093	13957262	C	0.177	2.32E-11	chr:3:14132684
3	rs9812276	13932069	A	0.177	2.32E-11	chr:3:14132684
3	rs9813081	13967417	A	0.177	2.32E-11	chr:3:14132684
3	rs9836126	13954830	G	0.177	2.32E-11	chr:3:14132684
3	rs9837169	13949585	C	0.177	2.32E-11	chr:3:14132684
3	rs9841847	13925930	G	0.177	2.32E-11	chr:3:14132684
3	rs9845208	13931668	G	0.177	2.32E-11	chr:3:14132684
3	rs9866731	13963237	A	0.177	2.32E-11	chr:3:14132684
3	rs115679038	13951421	G	0.1585	2.65E-11	chr:3:14132684
3	rs13326555	13970839	A	0.1585	2.65E-11	chr:3:14132684
3	rs1836062	13927838	G	0.1585	2.65E-11	chr:3:14132684
3	rs4100202	13927553	G	0.1585	2.65E-11	chr:3:14132684
3	rs75058833	13937762	A	0.1585	2.65E-11	chr:3:14132684
3	rs9809239	13956505	A	0.1585	2.65E-11	chr:3:14132684
3	rs9809732	13956896	A	0.1585	2.65E-11	chr:3:14132684
3	rs9817282	13954033	C	0.1585	2.65E-11	chr:3:14132684
3	rs9828438	13926238	C	0.1585	2.65E-11	chr:3:14132684
3	rs9829583	13953037	T	0.1585	2.65E-11	chr:3:14132684
3	rs9846432	13940098	C	0.1585	2.65E-11	chr:3:14132684
3	rs9850906	13957016	G	0.1585	2.65E-11	chr:3:14132684
3	rs9850966	13939923	C	0.1585	2.65E-11	chr:3:14132684
3	rs9868342	13944541	C	0.1585	2.65E-11	chr:3:14132684
3	rs9870914	13936149	G	0.1585	2.65E-11	chr:3:14132684
3	rs9875035	13949553	T	0.1585	2.65E-11	chr:3:14132684
3	rs9859458	14110031	T	0.1779	2.85E-11	chr:3:14132684
3	rs116165837	14141476	T	0.1798	2.99E-11	chr:3:14132684
3	rs28611125	13942862	T	0.1794	3.00E-11	chr:3:14132684
3	rs575748852	13952289	T	0.1794	3.00E-11	chr:3:14132684
3	rs79210652	13951065	G	0.1794	3.00E-11	chr:3:14132684
3	rs9812797	13967277	C	0.1794	3.00E-11	chr:3:14132684
3	rs9817937	13954402	C	0.1794	3.00E-11	chr:3:14132684
3	rs9818205	13961369	G	0.1794	3.00E-11	chr:3:14132684
3	rs9819140	13968995	A	0.1794	3.00E-11	chr:3:14132684

3	rs9830847	13953841	G	0.1794	3.00E-11	chr:3:14132684
3	rs9859141	13946543	T	0.1794	3.00E-11	chr:3:14132684
3	rs9833375	14142867	G	0.1597	3.31E-11	chr:3:14132684
3	rs1433360	13923058	A	0.1311	3.63E-11	chr:3:14132684
3	rs2733568	14137824	A	4.648	6.07E-11	chr:3:14132684
3	rs2733571	14140737	A	4.648	6.07E-11	chr:3:14132684
3	rs9866332	14023358	T	0.1557	1.25E-10	chr:3:14132684
3	rs528884598	14145075	C	0.191	2.49E-10	chr:3:14132684
3	rs79616045	13969716	A	0.2601	4.85E-10	chr:3:14132684
3	rs11924189	14037014	T	7.754	6.06E-10	chr:3:14132684
3	rs1386932	14061369	G	7.697	6.99E-10	chr:3:14132684
3	rs112517663	14037066	G	7.668	7.19E-10	chr:3:14132684
3	rs4684172	14036514	G	7.668	7.19E-10	chr:3:14132684
3	rs4685057	14028949	G	7.668	7.19E-10	chr:3:14132684
3	rs4685058	14030384	T	7.662	7.23E-10	chr:3:14132684
3	rs190558237	14034113	G	6.225	8.82E-10	chr:3:14132684
3	rs7636901	14030958	G	6.905	1.20E-09	chr:3:14132684
3	rs2607761	14091428	A	0.2848	3.04E-09	chr:3:14132684
3	rs7630488	13998984	C	0.29	3.74E-09	chr:3:14132684
3	rs11718142	14018793	G	0.3001	7.30E-09	chr:3:14132684
3	rs4684171	14028190	T	0.3001	7.30E-09	chr:3:14132684
3	rs9867041	14014615	T	0.3001	7.30E-09	chr:3:14132684
3	rs11927267	14073992	T	0.2999	7.35E-09	chr:3:14132684
3	rs7653289	13986333	C	0.2993	7.74E-09	chr:3:14132684
3	rs1077325	13992649	G	0.3004	7.87E-09	chr:3:14132684
3	rs2878160	14059571	A	0.3004	7.87E-09	chr:3:14132684
3	rs4685061	14043823	G	0.3004	7.87E-09	chr:3:14132684
3	rs6797895	14059009	T	0.3004	7.87E-09	chr:3:14132684
3	rs7645409	14056172	A	0.3004	7.87E-09	chr:3:14132684
3	rs9882317	14026356	T	0.1801	7.91E-09	chr:3:14132684
3	rs7651599	14010974	C	0.2974	8.19E-09	chr:3:14132684
3	rs4359796	14027681	T	0.3018	8.24E-09	chr:3:14132684
3	rs11128679	14075245	C	0.3025	9.19E-09	chr:3:14132684
3	rs2731305	14081342	T	0.3025	9.19E-09	chr:3:14132684
3	rs4684168	13981591	C	0.303	9.40E-09	chr:3:14132684
3	rs7373709	14114549	C	4.116	9.85E-09	chr:3:14132684
3	rs2607774	14112612	T	4.121	1.07E-08	chr:3:14132684
3	rs2607777	14110377	G	4.121	1.07E-08	chr:3:14132684
3	rs6773534	14113581	T	4.121	1.07E-08	chr:3:14132684
3	rs2733564	14134662	A	4.058	1.37E-08	chr:3:14132684
3	rs9874530	13915940	C	0.3219	3.58E-08	chr:3:14132684
3	rs12485272	13926324	A	5.922	6.71E-08	chr:3:14132684
3	rs12498112	13926288	A	5.898	6.88E-08	chr:3:14132684

3	rs12629691	13927124	T	5.848	8.17E-08	chr:3:14132684
3	rs2607754	14123418	C	8.493	9.92E-08	chr:3:14132684
3	rs12488625	13926215	G	5.43	1.02E-07	chr:3:14132684
3	rs1873003	13926117	A	5.381	1.35E-07	chr:3:14132684
3	rs72624492	14273657	A	0.3269	1.60E-07	chr:3:14132684
3	rs12630468	13928145	T	6.042	1.61E-07	chr:3:14132684
3	rs2259815	14129625	T	8.028	1.98E-07	chr:3:14132684
3	rs2731321	14127075	T	7.957	2.19E-07	chr:3:14132684
3	rs7371922	14000094	A	6.936	2.34E-07	chr:3:14132684
3	rs111910231	13985222	A	7.781	2.89E-07	chr:3:14132684
3	rs2129882	13980817	G	7.781	2.89E-07	chr:3:14132684
3	rs9883041	13987555	T	7.781	2.89E-07	chr:3:14132684
3	rs1488375	14061563	G	7.741	3.17E-07	chr:3:14132684
3	rs1586514	14050157	G	7.741	3.17E-07	chr:3:14132684
3	rs2733559	14103235	G	7.741	3.17E-07	chr:3:14132684
3	rs28865280	14064958	C	7.741	3.17E-07	chr:3:14132684
3	rs12330714	14040342	A	7.722	3.22E-07	chr:3:14132684
3	rs1906538	14014169	C	7.722	3.22E-07	chr:3:14132684
3	rs7373624	14000032	C	7.722	3.22E-07	chr:3:14132684
3	rs7648345	14039901	G	7.722	3.22E-07	chr:3:14132684
3	rs9831774	14002932	T	7.722	3.22E-07	chr:3:14132684
3	rs1844108	14024328	A	7.675	3.47E-07	chr:3:14132684
3	rs56071023	14075920	G	2.919	3.48E-07	chr:3:14132684
3	rs9848934	13972580	G	0.2562	4.04E-07	chr:3:14132684
3	rs79626378	14031735	G	6.628	4.74E-07	chr:3:14132684
3	rs9811978	13972699	T	0.2593	4.96E-07	chr:3:14132684
3	rs80109649	14047674	C	0.1777	5.31E-07	chr:3:14132684
3	rs186563622	13973964	C	0.08816	5.33E-07	chr:3:14132684
3	rs2733573	14143515	G	3.388	5.50E-07	chr:3:14132684
3	rs2733574	14146811	A	3.468	6.31E-07	chr:3:14132684
3	rs4685075	14174146	T	3.201	8.26E-07	chr:3:14132684
3	rs2733555	14099649	C	6.326	8.93E-07	chr:3:14132684
3	rs116501880	14162865	G	0.26	1.69E-06	chr:3:14132684
3	rs1077524	13930133	C	3.727	1.96E-06	chr:3:14132684
3	rs2168718	14084157	G	2.781	2.08E-06	chr:3:14132684
3	rs7632937	14169079	T	2.737	2.24E-06	chr:3:14132684
3	rs11914294	14097070	C	2.757	2.38E-06	chr:3:14132684
3	rs1825902	14007730	A	3.256	2.47E-06	chr:3:14132684
3	rs66716710	13938728	G	6.551	2.56E-06	chr:3:14132684
3	rs1579551	13945571	C	6.501	2.79E-06	chr:3:14132684
3	rs6778297	13932143	T	6.476	2.97E-06	chr:3:14132684
3	rs62234597	13963780	A	6.459	3.01E-06	chr:3:14132684
3	rs9835402	13954396	A	2.744	3.21E-06	chr:3:14132684

3	rs60536565	13989554	A	3.707	3.28E-06	chr:3:14132684
3	rs7638190	14182743	T	0.2723	3.31E-06	chr:3:14132684
3	rs11128674	14023301	C	2.704	3.33E-06	chr:3:14132684
3	rs12497139	14069587	C	2.701	3.53E-06	chr:3:14132684
3	rs6775534	14051001	T	2.701	3.53E-06	chr:3:14132684
3	rs6778884	14044681	G	2.701	3.53E-06	chr:3:14132684
3	rs2117039	13963330	T	2.721	3.62E-06	chr:3:14132684
3	rs76516763	14288865	T	0.2955	3.71E-06	chr:3:14132684
3	rs2607733	14144634	T	3.809	3.84E-06	chr:3:14132684
3	rs58484785	14025912	G	3.538	4.05E-06	chr:3:14132684
3	rs114945814	14155639	T	0.317	4.05E-06	chr:3:14132684
3	rs1545630	14154982	A	0.317	4.05E-06	chr:3:14132684
3	rs77941327	14154701	T	0.317	4.05E-06	chr:3:14132684
3	rs9875589	13961157	T	2.693	4.22E-06	chr:3:14132684
3	rs3731163	14189750	A	0.2769	4.33E-06	chr:3:14132684
3	rs78804891	14195939	A	0.2769	4.33E-06	chr:3:14132684
3	rs58619368	13983224	A	3.533	4.34E-06	chr:3:14132684
3	rs2125033	14121045	G	3.527	4.44E-06	chr:3:14132684
3	rs9681156	14116410	A	3.527	4.44E-06	chr:3:14132684
3	rs4684177	14070433	G	3.508	4.62E-06	chr:3:14132684
3	rs1586515	14007451	G	3.512	4.74E-06	chr:3:14132684
3	rs1601870	14024162	T	7.23	4.79E-06	chr:3:14132684
3	rs114645233	14164555	A	0.3198	4.84E-06	chr:3:14132684
3	rs114995436	14155753	T	0.3203	4.84E-06	chr:3:14132684
3	rs79249693	14162197	T	0.3203	4.84E-06	chr:3:14132684
3	rs79376214	14162212	C	0.3203	4.84E-06	chr:3:14132684
3	rs12490597	14131281	T	3.501	4.96E-06	chr:3:14132684
3	rs151198621	14166091	T	0.3258	4.98E-06	chr:3:14132684
3	rs80234167	14163921	G	0.3205	5.03E-06	chr:3:14132684
3	rs58025061	13988979	A	3.508	5.07E-06	chr:3:14132684
3	rs12490233	13977365	A	2.631	5.32E-06	chr:3:14132684
3	rs77851036	14238763	T	0.2812	5.48E-06	chr:3:14132684
3	rs2733577	14165929	C	2.593	5.50E-06	chr:3:14132684
3	rs80318592	13915410	T	0.3166	5.58E-06	chr:3:14132684
3	rs2341652	14118907	C	3.462	5.74E-06	chr:3:14132684
3	rs114671838	14269630	C	0.2857	6.58E-06	chr:3:14132684
3	rs75381945	14284754	A	0.3061	6.61E-06	chr:3:14132684
3	rs76958605	14278137	T	0.3061	6.61E-06	chr:3:14132684
3	rs79346909	14277369	T	0.3061	6.61E-06	chr:3:14132684
3	rs114248491	14267791	G	0.2862	6.94E-06	chr:3:14132684
3	rs6776709	13978803	G	3.437	6.96E-06	chr:3:14132684
3	rs2733566	14136506	G	6.927	7.56E-06	chr:3:14132684
3	rs9681152	14137485	A	3.32	7.73E-06	chr:3:14132684

3	rs112418665	13918467	C	0.3273	9.28E-06	chr:3:14132684
3	rs2340920	14100412	A	2.827	9.29E-06	chr:3:14132684
3	rs114274907	14151299	T	0.2803	1.04E-05	chr:3:14132684
3	rs115199886	14151236	C	0.2803	1.04E-05	chr:3:14132684
3	rs6775584	13925728	C	3.017	1.04E-05	chr:3:14132684
3	rs76922809	14291338	C	0.3207	1.07E-05	chr:3:14132684
3	rs9823738	13934580	A	0.3995	1.09E-05	chr:3:14132684
3	rs9823887	13934666	A	0.3995	1.09E-05	chr:3:14132684
3	rs115308741	14294531	A	0.3025	1.67E-05	chr:3:14132684
3	rs114913553	13901623	T	0.2718	2.05E-05	chr:3:14132684
3	rs142954409	14271479	C	0.3345	2.26E-05	chr:3:14132684
3	rs115584242	14307401	G	0.3037	2.34E-05	chr:3:14132684
3	rs2128161	14308120	C	0.3037	2.34E-05	chr:3:14132684
4	rs201965045	120254952	G	0.134	1.96E-33	chr:4:120263688
4	rs13128774	120246282	T	0.1412	2.00E-33	chr:4:120263688
4	rs13128933	120246249	A	0.1311	8.28E-33	chr:4:120263688
4	rs190883674	120269780	G	0.1223	8.38E-33	chr:4:120263688
4	rs201764844	120269768	C	0.1223	8.38E-33	chr:4:120263688
4	rs77466269	120269755	A	0.1206	1.17E-32	chr:4:120263688
4	rs140305554	120247693	C	0.1271	2.12E-32	chr:4:120263688
4	rs373735748	120244580	T	0.128	3.37E-32	chr:4:120263688
4	rs55884612	120244577	T	0.128	3.37E-32	chr:4:120263688
4	rs12510923	120247381	G	0.1148	4.39E-32	chr:4:120263688
4	rs113263911	120255134	T	0.136	4.69E-32	chr:4:120263688
4	rs10006495	120263315	G	0.1334	5.37E-32	chr:4:120263688
4	rs200947437	120254755	C	0.1123	1.14E-31	chr:4:120263688
4	rs12503309	120247397	A	0.1214	1.35E-31	chr:4:120263688
4	rs10017001	120263293	G	0.1336	1.66E-31	chr:4:120263688
4	rs9994691	120263298	A	0.1336	1.66E-31	chr:4:120263688
4	rs12503276	120247329	A	0.1042	2.45E-31	chr:4:120263688
4	rs12503360	120247506	C	0.1041	3.47E-31	chr:4:120263688
4	rs534745130	120246059	C	0.1169	3.70E-31	chr:4:120263688
4	rs55816173	120247014	T	0.1398	5.07E-31	chr:4:120263688
4	rs10013042	120244482	T	0.09842	7.30E-31	chr:4:120263688
4	rs13101526	120244456	T	0.09842	7.30E-31	chr:4:120263688
4	rs140584472	120246695	G	0.1314	1.18E-30	chr:4:120263688
4	rs185481756	120246689	C	0.1314	1.18E-30	chr:4:120263688
4	rs143457155	120263622	C	0.07849	4.80E-30	chr:4:120263688
4	rs10006688	120263526	C	0.07878	5.57E-30	chr:4:120263688
4	rs34481394	120248374	T	0.09109	6.75E-30	chr:4:120263688
4	rs35231872	120248376	T	0.09109	6.75E-30	chr:4:120263688
4	rs35653026	120248378	G	0.09109	6.75E-30	chr:4:120263688
4	rs7672372	120248406	G	0.08788	1.22E-29	chr:4:120263688

4	rs75420021	120246729	A	0.08856	1.23E-29	chr:4:120263688
4	rs7671759	120248094	C	0.09662	1.40E-29	chr:4:120263688
4	rs7672357	120248340	T	0.0883	1.41E-29	chr:4:120263688
4	rs28369503	120238925	A	0.08037	1.56E-29	chr:4:120263688
4	rs28458294	120238924	C	0.08037	1.56E-29	chr:4:120263688
4	rs9996501	120238918	A	0.08037	1.56E-29	chr:4:120263688
4	rs12503171	120247196	G	0.08913	1.79E-29	chr:4:120263688
4	rs12507283	120247218	A	0.08913	1.79E-29	chr:4:120263688
4	rs72676046	120254681	C	0.0847	1.85E-29	chr:4:120263688
4	rs7438634	120245754	C	0.085	1.90E-29	chr:4:120263688
4	rs13122153	120266585	C	0.07656	2.12E-29	chr:4:120263688
4	rs7671797	120248157	G	0.08499	2.16E-29	chr:4:120263688
4	rs28735977	120261738	T	0.07672	2.18E-29	chr:4:120263688
4	rs10212956	120254343	G	0.08139	2.62E-29	chr:4:120263688
4	rs1849457	120254355	G	0.08139	2.62E-29	chr:4:120263688
4	rs9996494	120238880	A	0.07712	2.64E-29	chr:4:120263688
4	rs9996644	120238877	T	0.07712	2.64E-29	chr:4:120263688
4	rs12507387	120247590	C	0.07692	2.70E-29	chr:4:120263688
4	rs7671978	120248218	G	0.08561	2.77E-29	chr:4:120263688
4	rs7672129	120248176	T	0.08561	2.77E-29	chr:4:120263688
4	rs10003931	120259052	G	0.08143	2.79E-29	chr:4:120263688
4	rs2036856	120249288	A	0.0813	2.87E-29	chr:4:120263688
4	rs2036857	120249240	C	0.0813	2.87E-29	chr:4:120263688
4	rs4443261	120249301	C	0.0813	2.87E-29	chr:4:120263688
4	rs4422403	120258194	T	0.07758	3.68E-29	chr:4:120263688
4	rs13121928	120266518	C	0.06881	3.78E-29	chr:4:120263688
4	rs1814814	120258124	G	0.07758	3.90E-29	chr:4:120263688
4	rs10213480	120254561	T	0.07774	4.05E-29	chr:4:120263688
4	rs35280960	120257059	A	0.07734	4.09E-29	chr:4:120263688
4	rs10024933	120253582	A	0.07757	4.11E-29	chr:4:120263688
4	rs10518300	120249499	G	0.07772	4.26E-29	chr:4:120263688
4	rs28643450	120245242	A	0.07765	4.34E-29	chr:4:120263688
4	rs28502463	120257023	C	0.07788	4.66E-29	chr:4:120263688
4	rs28622913	120261723	A	0.06912	4.97E-29	chr:4:120263688
4	rs10003567	120241674	T	0.07387	5.82E-29	chr:4:120263688
4	rs10005237	120237897	C	0.07387	5.82E-29	chr:4:120263688
4	rs10006259	120242145	T	0.07387	5.82E-29	chr:4:120263688
4	rs10212775	120239244	C	0.07387	5.82E-29	chr:4:120263688
4	rs10213267	120239074	G	0.07387	5.82E-29	chr:4:120263688
4	rs12506610	120241659	C	0.07387	5.82E-29	chr:4:120263688
4	rs12711071	120240934	C	0.07387	5.82E-29	chr:4:120263688
4	rs28652763	120242312	T	0.07387	5.82E-29	chr:4:120263688
4	rs2964	120240131	C	0.07387	5.82E-29	chr:4:120263688

4	rs878372	120238780	C	0.07387	5.82E-29	chr:4:120263688
4	rs907204	120238654	A	0.07387	5.82E-29	chr:4:120263688
4	rs907205	120238664	C	0.07387	5.82E-29	chr:4:120263688
4	rs9991166	120237851	A	0.07387	5.82E-29	chr:4:120263688
4	rs10213221	120255926	A	0.07387	5.84E-29	chr:4:120263688
4	rs10023641	120258410	G	0.07401	6.36E-29	chr:4:120263688
4	rs114866537	120258608	T	0.07401	6.36E-29	chr:4:120263688
4	rs10212714	120254302	C	0.07406	6.80E-29	chr:4:120263688
4	rs10212719	120254437	C	0.07406	6.80E-29	chr:4:120263688
4	rs10213158	120255829	T	0.07406	6.80E-29	chr:4:120263688
4	rs11098501	120252017	G	0.07406	6.80E-29	chr:4:120263688
4	rs11098502	120252063	A	0.07406	6.80E-29	chr:4:120263688
4	rs11724409	120252361	G	0.07406	6.80E-29	chr:4:120263688
4	rs11732087	120239831	G	0.07406	6.80E-29	chr:4:120263688
4	rs11732686	120252330	A	0.07406	6.80E-29	chr:4:120263688
4	rs12498994	120250818	C	0.07406	6.80E-29	chr:4:120263688
4	rs12507565	120251121	A	0.07406	6.80E-29	chr:4:120263688
4	rs12509621	120249660	C	0.07406	6.80E-29	chr:4:120263688
4	rs12511640	120251248	C	0.07406	6.80E-29	chr:4:120263688
4	rs12513083	120249612	T	0.07406	6.80E-29	chr:4:120263688
4	rs140387830	120247916	G	0.07406	6.80E-29	chr:4:120263688
4	rs145027390	120247827	G	0.07406	6.80E-29	chr:4:120263688
4	rs1511017	120250805	A	0.07406	6.80E-29	chr:4:120263688
4	rs1511025	120240238	C	0.07406	6.80E-29	chr:4:120263688
4	rs17049949	120255290	G	0.07406	6.80E-29	chr:4:120263688
4	rs1980024	120253047	T	0.07406	6.80E-29	chr:4:120263688
4	rs1980025	120252806	T	0.07406	6.80E-29	chr:4:120263688
4	rs1980026	120251643	A	0.07406	6.80E-29	chr:4:120263688
4	rs2002047	120257228	G	0.07406	6.80E-29	chr:4:120263688
4	rs2002049	120257417	G	0.07406	6.80E-29	chr:4:120263688
4	rs2036860	120248934	G	0.07406	6.80E-29	chr:4:120263688
4	rs28363929	120257103	G	0.07406	6.80E-29	chr:4:120263688
4	rs28373117	120253465	T	0.07406	6.80E-29	chr:4:120263688
4	rs28713555	120251995	T	0.07406	6.80E-29	chr:4:120263688
4	rs34425882	120253177	A	0.07406	6.80E-29	chr:4:120263688
4	rs3956464	120256764	C	0.07406	6.80E-29	chr:4:120263688
4	rs4107728	120253910	A	0.07406	6.80E-29	chr:4:120263688
4	rs7672594	120248543	C	0.07406	6.80E-29	chr:4:120263688
4	rs7672778	120248585	G	0.07406	6.80E-29	chr:4:120263688
4	rs7689729	120256192	G	0.07406	6.80E-29	chr:4:120263688
4	rs7690338	120256468	G	0.07406	6.80E-29	chr:4:120263688
4	rs9991959	120253773	C	0.07406	6.80E-29	chr:4:120263688
4	rs1546503	120241167	C	0.07409	6.90E-29	chr:4:120263688

4	rs1546506	120241240	C	0.07409	6.90E-29	chr:4:120263688
4	rs12510269	120241646	G	0.07416	7.01E-29	chr:4:120263688
4	rs11735886	120290215	C	0.09901	7.31E-29	chr:4:120263688
4	rs4434233;rs542273973	120258229	A	0.07434	7.74E-29	chr:4:120263688
4	rs10005973	120271958	T	0.1081	8.18E-29	chr:4:120263688
4	rs13108589	120268102	C	0.1081	8.18E-29	chr:4:120263688
4	rs17595608	120250506	T	0.1081	8.18E-29	chr:4:120263688
4	rs1980027	120251577	T	0.1081	8.18E-29	chr:4:120263688
4	rs3864141	120266007	A	0.1081	8.18E-29	chr:4:120263688
4	rs66900435	120249425	A	0.1081	8.18E-29	chr:4:120263688
4	rs714899	120243035	A	0.1081	8.18E-29	chr:4:120263688
4	rs9999724	120239944	T	0.1081	8.18E-29	chr:4:120263688
4	rs7673476	120248683	A	0.07431	8.78E-29	chr:4:120263688
4	rs9993347	120258955	G	0.1166	9.51E-29	chr:4:120263688
4	rs148893841	120263621	G	0.06587	1.04E-28	chr:4:120263688
4	rs10213554	120260785	A	0.1088	1.19E-28	chr:4:120263688
4	rs3864143	120266428	C	0.06607	1.44E-28	chr:4:120263688
4	rs6815934	120267310	C	0.06664	1.92E-28	chr:4:120263688
4	rs12502393	120271228	T	0.06668	1.96E-28	chr:4:120263688
4	rs12506445	120271237	C	0.06668	1.96E-28	chr:4:120263688
4	rs6838457	120267367	G	0.06666	2.07E-28	chr:4:120263688
4	rs10000544	120270705	A	0.06689	2.45E-28	chr:4:120263688
4	rs10005542	120268302	C	0.06689	2.45E-28	chr:4:120263688
4	rs10006192	120263022	C	0.06689	2.45E-28	chr:4:120263688
4	rs10010355	120261043	G	0.06689	2.45E-28	chr:4:120263688
4	rs10012408	120270771	A	0.06689	2.45E-28	chr:4:120263688
4	rs10015579	120271802	C	0.06689	2.45E-28	chr:4:120263688
4	rs10015965	120268237	G	0.06689	2.45E-28	chr:4:120263688
4	rs10016241	120268553	C	0.06689	2.45E-28	chr:4:120263688
4	rs10022448	120270421	G	0.06689	2.45E-28	chr:4:120263688
4	rs10025925	120271744	T	0.06689	2.45E-28	chr:4:120263688
4	rs10028773	120265259	G	0.06689	2.45E-28	chr:4:120263688
4	rs12498657	120262866	C	0.06689	2.45E-28	chr:4:120263688
4	rs12502524	120271414	A	0.06689	2.45E-28	chr:4:120263688
4	rs12506487	120271361	G	0.06689	2.45E-28	chr:4:120263688
4	rs12510133	120271344	C	0.06689	2.45E-28	chr:4:120263688
4	rs17009144	120270795	G	0.06689	2.45E-28	chr:4:120263688
4	rs17517414	120262101	C	0.06689	2.45E-28	chr:4:120263688
4	rs2136911	120265859	A	0.06689	2.45E-28	chr:4:120263688
4	rs2175381	120265967	C	0.06689	2.45E-28	chr:4:120263688
4	rs28369518	120271630	C	0.06689	2.45E-28	chr:4:120263688
4	rs28396837	120271541	A	0.06689	2.45E-28	chr:4:120263688
4	rs28437684	120264849	A	0.06689	2.45E-28	chr:4:120263688

4	rs28546691	120262832	A	0.06689	2.45E-28	chr:4:120263688
4	rs28551750	120264901	T	0.06689	2.45E-28	chr:4:120263688
4	rs28589345	120269080	A	0.06689	2.45E-28	chr:4:120263688
4	rs28599836	120268809	T	0.06689	2.45E-28	chr:4:120263688
4	rs28616635	120261861	C	0.06689	2.45E-28	chr:4:120263688
4	rs28691602	120264770	C	0.06689	2.45E-28	chr:4:120263688
4	rs28714087	120270966	G	0.06689	2.45E-28	chr:4:120263688
4	rs28726222	120262672	C	0.06689	2.45E-28	chr:4:120263688
4	rs3864142	120266191	T	0.06689	2.45E-28	chr:4:120263688
4	rs3864144	120272605	T	0.06689	2.45E-28	chr:4:120263688
4	rs3986377	120260270	A	0.06689	2.45E-28	chr:4:120263688
4	rs4001390	120265783	C	0.06689	2.45E-28	chr:4:120263688
4	rs4473640	120260437	A	0.06689	2.45E-28	chr:4:120263688
4	rs55820750	120269168	C	0.06689	2.45E-28	chr:4:120263688
4	rs6815291	120266982	A	0.06689	2.45E-28	chr:4:120263688
4	rs6833140	120266822	A	0.06689	2.45E-28	chr:4:120263688
4	rs6838009	120267034	A	0.06689	2.45E-28	chr:4:120263688
4	rs79606892	120259606	C	0.06689	2.45E-28	chr:4:120263688
4	rs9307470	120261214	A	0.06689	2.45E-28	chr:4:120263688
4	rs9307471	120261262	A	0.06689	2.45E-28	chr:4:120263688
4	rs9995716	120264996	C	0.06689	2.45E-28	chr:4:120263688
4	rs1814813	120258207	G	0.1223	2.70E-28	chr:4:120263688
4	rs28572003	120268891	G	0.067	2.77E-28	chr:4:120263688
4	rs555773279	120269424	T	0.06701	2.77E-28	chr:4:120263688
4	rs6838968	120267311	A	0.06725	2.98E-28	chr:4:120263688
4	rs17595727	120262074	C	0.06721	3.10E-28	chr:4:120263688
4	rs12506421	120271167	C	0.0629	4.00E-28	chr:4:120263688
4	rs878376	120237702	A	0.1372	1.48E-26	chr:4:120263688
4	rs938056	120237453	T	0.1372	1.48E-26	chr:4:120263688
4	rs34818745	120257055	C	0.1462	2.24E-26	chr:4:120263688
4	rs371048191	120246107	C	0.156	3.22E-26	chr:4:120263688
4	rs4472123	120236630	A	0.1592	4.97E-25	chr:4:120263688
4	rs373699192	120246109	A	0.1467	7.65E-25	chr:4:120263688
4	rs9685057	120272333	T	5.445	2.43E-24	chr:4:120263688
4	rs78422072	120281042	T	0.1697	2.43E-24	chr:4:120263688
4	rs78971550	120281041	T	0.1697	2.43E-24	chr:4:120263688
4	rs10026436	120288904	A	0.1627	2.49E-24	chr:4:120263688
4	rs11735878	120290177	C	0.1862	3.45E-24	chr:4:120263688
4	rs6846966	120293208	G	0.1675	3.57E-24	chr:4:120263688
4	rs11722183	120280597	C	0.1664	4.45E-24	chr:4:120263688
4	rs4296662	120313440	G	0.166	4.46E-24	chr:4:120263688
4	rs4560394	120313435	G	0.166	4.46E-24	chr:4:120263688
4	rs10002914	120292081	A	0.1674	4.67E-24	chr:4:120263688

4	rs10013652	120292256	G	0.1674	4.67E-24	chr:4:120263688
4	rs10029303	120286755	A	0.1674	4.67E-24	chr:4:120263688
4	rs10517834	120286783	C	0.1674	4.67E-24	chr:4:120263688
4	rs10857066	120286596	T	0.1674	4.67E-24	chr:4:120263688
4	rs11098505	120284627	T	0.1674	4.67E-24	chr:4:120263688
4	rs11098506	120284971	G	0.1674	4.67E-24	chr:4:120263688
4	rs11098507	120288286	A	0.1674	4.67E-24	chr:4:120263688
4	rs12374244	120281972	T	0.1674	4.67E-24	chr:4:120263688
4	rs12374245	120282059	T	0.1674	4.67E-24	chr:4:120263688
4	rs12374346	120281705	A	0.1674	4.67E-24	chr:4:120263688
4	rs12374352	120281977	T	0.1674	4.67E-24	chr:4:120263688
4	rs12374396	120281619	C	0.1674	4.67E-24	chr:4:120263688
4	rs12499602	120284387	A	0.1674	4.67E-24	chr:4:120263688
4	rs12501602	120287935	T	0.1674	4.67E-24	chr:4:120263688
4	rs12503082	120284317	G	0.1674	4.67E-24	chr:4:120263688
4	rs12510451	120289143	T	0.1674	4.67E-24	chr:4:120263688
4	rs12510487	120289231	T	0.1674	4.67E-24	chr:4:120263688
4	rs12513310	120288039	A	0.1674	4.67E-24	chr:4:120263688
4	rs13104572	120285568	C	0.1674	4.67E-24	chr:4:120263688
4	rs13125526	120288019	A	0.1674	4.67E-24	chr:4:120263688
4	rs28470894	120289405	G	0.1674	4.67E-24	chr:4:120263688
4	rs28488261	120289396	C	0.1674	4.67E-24	chr:4:120263688
4	rs28501434	120283773	T	0.1674	4.67E-24	chr:4:120263688
4	rs28581362	120283548	A	0.1674	4.67E-24	chr:4:120263688
4	rs28594160	120289456	G	0.1674	4.67E-24	chr:4:120263688
4	rs28668103	120291372	T	0.1674	4.67E-24	chr:4:120263688
4	rs28687057	120280812	G	0.1674	4.67E-24	chr:4:120263688
4	rs55845118	120290913	C	0.1674	4.67E-24	chr:4:120263688
4	rs60378962	120280991	A	0.1674	4.67E-24	chr:4:120263688
4	rs67281037	120281157	C	0.1674	4.67E-24	chr:4:120263688
4	rs6824111	120285968	A	0.1674	4.67E-24	chr:4:120263688
4	rs6824169	120293175	C	0.1674	4.67E-24	chr:4:120263688
4	rs6847586	120286069	A	0.1674	4.67E-24	chr:4:120263688
4	rs6847778	120293582	G	0.1674	4.67E-24	chr:4:120263688
4	rs6847862	120286371	T	0.1674	4.67E-24	chr:4:120263688
4	rs6848081	120286443	T	0.1674	4.67E-24	chr:4:120263688
4	rs6848380	120286403	A	0.1674	4.67E-24	chr:4:120263688
4	rs6857892	120282696	T	0.1674	4.67E-24	chr:4:120263688
4	rs71610270	120287436	G	0.1674	4.67E-24	chr:4:120263688
4	rs72676059	120290828	G	0.1674	4.67E-24	chr:4:120263688
4	rs73842616	120290683	A	0.1674	4.67E-24	chr:4:120263688
4	rs7677068	120291704	T	0.1674	4.67E-24	chr:4:120263688
4	rs9991221	120292107	C	0.1674	4.67E-24	chr:4:120263688

4	rs9995136	120286845	G	0.1674	4.67E-24	chr:4:120263688
4	rs10003742	120271798	G	5.445	4.94E-24	chr:4:120263688
4	rs10015883	120268146	A	5.445	4.94E-24	chr:4:120263688
4	rs10213542	120260650	C	5.445	4.94E-24	chr:4:120263688
4	rs28439855	120262256	G	5.445	4.94E-24	chr:4:120263688
4	rs4643791	120265619	G	5.445	4.94E-24	chr:4:120263688
4	rs9307472	120261415	T	5.445	4.94E-24	chr:4:120263688
4	rs9637624	120274911	A	5.445	4.94E-24	chr:4:120263688
4	rs10857068	120317549	G	0.1679	5.92E-24	chr:4:120263688
4	rs11098513	120316075	G	0.1679	5.92E-24	chr:4:120263688
4	rs11098514	120317374	C	0.1679	5.92E-24	chr:4:120263688
4	rs11098516	120317978	C	0.1679	5.92E-24	chr:4:120263688
4	rs13116768	120318254	C	0.1679	5.92E-24	chr:4:120263688
4	rs28572238	120316686	G	0.1679	5.92E-24	chr:4:120263688
4	rs150131449	120288605	G	0.1665	5.99E-24	chr:4:120263688
4	rs13105020	120285688	T	0.1696	6.03E-24	chr:4:120263688
4	rs13132082	120285657	A	0.1696	6.03E-24	chr:4:120263688
4	rs10002083	120311152	T	0.1675	6.20E-24	chr:4:120263688
4	rs10010801	120310028	C	0.1675	6.20E-24	chr:4:120263688
4	rs10014748	120300988	G	0.1675	6.20E-24	chr:4:120263688
4	rs10014845	120301077	G	0.1675	6.20E-24	chr:4:120263688
4	rs10024844	120311528	G	0.1675	6.20E-24	chr:4:120263688
4	rs10025054	120311697	C	0.1675	6.20E-24	chr:4:120263688
4	rs10027498	120311987	C	0.1675	6.20E-24	chr:4:120263688
4	rs10027856	120304874	A	0.1675	6.20E-24	chr:4:120263688
4	rs10027976	120305042	T	0.1675	6.20E-24	chr:4:120263688
4	rs10222794	120302351	T	0.1675	6.20E-24	chr:4:120263688
4	rs10222801	120302350	T	0.1675	6.20E-24	chr:4:120263688
4	rs10440343	120309787	C	0.1675	6.20E-24	chr:4:120263688
4	rs10440402	120307813	G	0.1675	6.20E-24	chr:4:120263688
4	rs11098508	120306607	G	0.1675	6.20E-24	chr:4:120263688
4	rs11098509	120306670	C	0.1675	6.20E-24	chr:4:120263688
4	rs11098510	120306793	T	0.1675	6.20E-24	chr:4:120263688
4	rs11726097	120301499	T	0.1675	6.20E-24	chr:4:120263688
4	rs12498908	120308261	A	0.1675	6.20E-24	chr:4:120263688
4	rs12506452	120308083	C	0.1675	6.20E-24	chr:4:120263688
4	rs13113483	120309039	T	0.1675	6.20E-24	chr:4:120263688
4	rs13151285	120314741	A	0.1675	6.20E-24	chr:4:120263688
4	rs138611376	120306434	G	0.1675	6.20E-24	chr:4:120263688
4	rs140816585	120306228	C	0.1675	6.20E-24	chr:4:120263688
4	rs146523888	120308804	G	0.1675	6.20E-24	chr:4:120263688
4	rs150376940	120305404	A	0.1675	6.20E-24	chr:4:120263688
4	rs28751034	120307097	A	0.1675	6.20E-24	chr:4:120263688

4	rs28793658	120307214	C	0.1675	6.20E-24	chr:4:120263688
4	rs28812279	120304152	C	0.1675	6.20E-24	chr:4:120263688
4	rs28884220	120307211	G	0.1675	6.20E-24	chr:4:120263688
4	rs4309825	120314881	G	0.1675	6.20E-24	chr:4:120263688
4	rs4345162	120312959	A	0.1675	6.20E-24	chr:4:120263688
4	rs4373140	120307698	G	0.1675	6.20E-24	chr:4:120263688
4	rs4463052	120313258	C	0.1675	6.20E-24	chr:4:120263688
4	rs4507344	120307485	A	0.1675	6.20E-24	chr:4:120263688
4	rs4604043	120314921	T	0.1675	6.20E-24	chr:4:120263688
4	rs7437420	120312903	G	0.1675	6.20E-24	chr:4:120263688
4	rs7674544	120303714	G	0.1675	6.20E-24	chr:4:120263688
4	rs7677836	120310638	A	0.1675	6.20E-24	chr:4:120263688
4	rs7678238	120310832	C	0.1675	6.20E-24	chr:4:120263688
4	rs76990001	120302639	T	0.1675	6.20E-24	chr:4:120263688
4	rs78036070	120302802	A	0.1675	6.20E-24	chr:4:120263688
4	rs79704848	120309351	A	0.1675	6.20E-24	chr:4:120263688
4	rs9307474	120302496	A	0.1675	6.20E-24	chr:4:120263688
4	rs9994889	120305056	G	0.1675	6.20E-24	chr:4:120263688
4	rs6848580	120286492	A	0.1705	6.62E-24	chr:4:120263688
4	rs10004114	120259213	A	5.362	7.76E-24	chr:4:120263688
4	rs114019672	120306475	G	0.1685	8.12E-24	chr:4:120263688
4	rs7658181	120310873	G	0.1677	8.14E-24	chr:4:120263688
4	rs10011097	120310359	G	0.1686	8.33E-24	chr:4:120263688
4	rs13127114	120314664	C	0.1685	8.39E-24	chr:4:120263688
4	rs28668716	120309875	C	0.169	8.77E-24	chr:4:120263688
4	rs10021601	120282563	A	0.1724	8.84E-24	chr:4:120263688
4	rs7674500	120303593	T	0.1693	8.95E-24	chr:4:120263688
4	rs1814815	120258091	C	5.362	8.97E-24	chr:4:120263688
4	rs2017058	120257739	T	5.362	8.97E-24	chr:4:120263688
4	rs7665192	120247065	A	0.1744	9.02E-24	chr:4:120263688
4	rs10005644	120298477	C	0.1702	9.07E-24	chr:4:120263688
4	rs10016060	120298412	G	0.1702	9.07E-24	chr:4:120263688
4	rs10016448	120298845	G	0.1702	9.07E-24	chr:4:120263688
4	rs10020034	120294331	G	0.1702	9.07E-24	chr:4:120263688
4	rs10028423	120294909	T	0.1702	9.07E-24	chr:4:120263688
4	rs10031718	120295892	T	0.1702	9.07E-24	chr:4:120263688
4	rs1112817	120297800	C	0.1702	9.07E-24	chr:4:120263688
4	rs11729050	120300066	C	0.1702	9.07E-24	chr:4:120263688
4	rs2322021	120295030	A	0.1702	9.07E-24	chr:4:120263688
4	rs28429722	120300093	A	0.1702	9.07E-24	chr:4:120263688
4	rs28491261	120294900	A	0.1702	9.07E-24	chr:4:120263688
4	rs59394118	120295551	G	0.1702	9.07E-24	chr:4:120263688
4	rs59866101	120296591	G	0.1702	9.07E-24	chr:4:120263688

4	rs6832410	120296800	G	0.1702	9.07E-24	chr:4:120263688
4	rs6838617	120297423	T	0.1702	9.07E-24	chr:4:120263688
4	rs6858383	120296772	C	0.1702	9.07E-24	chr:4:120263688
4	rs9307473	120300221	T	0.1702	9.07E-24	chr:4:120263688
4	rs9996417	120295862	C	0.1702	9.07E-24	chr:4:120263688
4	rs9996569	120299004	C	0.1702	9.07E-24	chr:4:120263688
4	rs9996677	120299148	A	0.1702	9.07E-24	chr:4:120263688
4	rs9998953	120296169	C	0.1702	9.07E-24	chr:4:120263688
4	rs28714195	120316950	T	0.1725	9.82E-24	chr:4:120263688
4	rs12511625	120318135	G	0.172	1.12E-23	chr:4:120263688
4	rs10222798	120302419	T	0.1728	1.24E-23	chr:4:120263688
4	rs11724758	120239878	G	5.309	1.26E-23	chr:4:120263688
4	rs13117947	120257060	A	5.251	1.43E-23	chr:4:120263688
4	rs11723757	120299669	T	0.1636	1.55E-23	chr:4:120263688
4	rs56155624	120291026	G	0.1771	1.60E-23	chr:4:120263688
4	rs10434028	120294464	G	0.1763	1.81E-23	chr:4:120263688
4	rs7658368	120310922	T	0.1747	2.46E-23	chr:4:120263688
4	rs10017189	120263523	A	4.887	2.56E-23	chr:4:120263688
4	rs2036858	120249237	A	5.168	3.14E-23	chr:4:120263688
4	rs552865294	120246034	C	4.2	4.94E-23	chr:4:120263688
4	rs35239065	120325130	C	0.1813	8.07E-23	chr:4:120263688
4	rs35265692	120325135	C	0.1813	8.07E-23	chr:4:120263688
4	rs72676074	120359841	C	0.2016	9.03E-23	chr:4:120263688
4	rs3956463	120245748	G	4.896	9.81E-23	chr:4:120263688
4	rs200290730	120287492	A	0.1681	1.34E-22	chr:4:120263688
4	rs10020256	120287265	T	0.1698	1.94E-22	chr:4:120263688
4	rs11723550	120289961	C	0.1906	2.88E-22	chr:4:120263688
4	rs10440403	120308047	T	0.195	5.32E-22	chr:4:120263688
4	rs4001305	120359236	T	0.2143	5.42E-22	chr:4:120263688
4	rs71627788	120255728	C	4.076	6.05E-22	chr:4:120263688
4	rs71610285	120329953	C	0.2073	1.45E-21	chr:4:120263688
4	rs72916543	120254961	T	0.2906	1.75E-21	chr:4:120263688
4	rs10019309	120327085	T	0.2094	3.37E-21	chr:4:120263688
4	rs368938172	120330272	G	0.2108	5.06E-21	chr:4:120263688
4	rs71595380	120329294	T	0.2048	5.75E-21	chr:4:120263688
4	rs114860385	120355584	C	0.2108	9.97E-21	chr:4:120263688
4	rs28499576	120386677	A	0.2121	9.98E-21	chr:4:120263688
4	rs7356491	120381974	T	0.2121	9.98E-21	chr:4:120263688
4	rs7692994	120427489	A	0.2294	1.06E-20	chr:4:120263688
4	rs2389809	120377399	C	0.2117	1.14E-20	chr:4:120263688
4	rs73844769	120254829	A	3.534	1.16E-20	chr:4:120263688
4	rs10018726	120394010	G	0.2167	1.88E-20	chr:4:120263688
4	rs10020027	120381879	C	0.2167	1.88E-20	chr:4:120263688

4	rs13107475	120393011	T	0.2167	1.88E-20	chr:4:120263688
4	rs1809406	120377122	A	0.2167	1.88E-20	chr:4:120263688
4	rs2389803	120393511	A	0.2167	1.88E-20	chr:4:120263688
4	rs9994810	120381590	G	0.2167	1.88E-20	chr:4:120263688
4	rs11098534	120556772	G	0.2378	2.29E-20	chr:4:120263688
4	rs28408407	120376030	G	0.2146	2.29E-20	chr:4:120263688
4	rs28587175	120373172	C	0.2146	2.29E-20	chr:4:120263688
4	rs10034623	120397829	A	0.2215	4.15E-20	chr:4:120263688
4	rs6849171	120409549	A	0.2215	4.15E-20	chr:4:120263688
4	rs4001207	120331782	C	0.2192	4.37E-20	chr:4:120263688
4	rs9997340	120364809	C	0.2192	4.37E-20	chr:4:120263688
4	rs9998174	120366312	G	0.2192	4.37E-20	chr:4:120263688
4	rs3733520	120423480	G	0.2211	4.44E-20	chr:4:120263688
4	rs72918577	120326701	A	0.2219	4.66E-20	chr:4:120263688
4	rs7679468	120328333	C	0.2218	5.59E-20	chr:4:120263688
4	rs7654611	120328304	G	0.2218	5.63E-20	chr:4:120263688
4	rs34237553	120325273	C	0.2204	5.63E-20	chr:4:120263688
4	rs72918584	120362445	C	0.2226	6.32E-20	chr:4:120263688
4	rs201764925	120334210	A	0.1804	7.41E-20	chr:4:120263688
4	rs878374	120237564	G	0.2492	7.85E-20	chr:4:120263688
4	rs72918579	120333222	C	0.2236	8.14E-20	chr:4:120263688
4	rs10004484	120442428	T	0.2289	8.18E-20	chr:4:120263688
4	rs1397613	120242929	G	4.187	8.60E-20	chr:4:120263688
4	rs4834770	120241849	A	4.187	8.60E-20	chr:4:120263688
4	rs4834771	120242772	C	4.187	8.60E-20	chr:4:120263688
4	rs6823963	120242164	A	4.187	8.60E-20	chr:4:120263688
4	rs10518336	120522934	A	0.244	1.01E-19	chr:4:120263688
4	rs10034579	120244029	A	4.183	1.03E-19	chr:4:120263688
4	rs1973598	120243419	A	4.183	1.03E-19	chr:4:120263688
4	rs2282688	120243722	T	4.183	1.03E-19	chr:4:120263688
4	rs6857641	120243511	T	4.183	1.03E-19	chr:4:120263688
4	rs7655177	120328622	G	0.2266	1.06E-19	chr:4:120263688
4	rs6832740	120546136	C	0.235	1.08E-19	chr:4:120263688
4	rs7693919	120540571	A	0.235	1.08E-19	chr:4:120263688
4	rs28567749	120329211	C	0.22	1.28E-19	chr:4:120263688
4	rs17050695	120489527	T	0.2339	1.38E-19	chr:4:120263688
4	rs28735358	120482049	C	0.2339	1.38E-19	chr:4:120263688
4	rs6858592	120458692	A	0.2339	1.38E-19	chr:4:120263688
4	rs10050092	120532085	C	0.237	1.55E-19	chr:4:120263688
4	rs6855918	120546299	T	0.2365	1.61E-19	chr:4:120263688
4	rs1155576	120450159	C	0.2355	1.91E-19	chr:4:120263688
4	rs147267946	120307639	T	0.1494	1.94E-19	chr:4:120263688
4	rs10018280	120478139	G	0.2357	1.95E-19	chr:4:120263688

4	rs2127823	120485670	T	0.2357	1.95E-19	chr:4:120263688
4	rs3775858	120486028	G	0.2357	1.95E-19	chr:4:120263688
4	rs7680063	120479889	G	0.2357	1.95E-19	chr:4:120263688
4	rs9997631	120469995	G	0.2357	1.95E-19	chr:4:120263688
4	rs6534141	120485223	T	0.2396	2.52E-19	chr:4:120263688
4	rs28850368	120554313	A	0.2632	3.39E-19	chr:4:120263688
4	rs34835603	120553428	T	0.2632	3.39E-19	chr:4:120263688
4	rs75199754	120365198	A	0.2304	3.75E-19	chr:4:120263688
4	rs4145952	120155806	C	4.113	1.87E-18	chr:4:120263688
4	rs10014822	120280143	C	3.488	6.66E-18	chr:4:120263688
4	rs28787668	120554687	A	0.2753	1.20E-17	chr:4:120263688
4	rs7699346	120310542	T	0.2492	1.49E-17	chr:4:120263688
4	rs2389880	120559870	A	0.2798	1.69E-17	chr:4:120263688
4	rs6820115	120398182	C	0.2857	3.72E-17	chr:4:120263688
4	rs67265404	120359270	T	0.2693	4.01E-17	chr:4:120263688
4	rs3806808	120551085	C	0.2889	4.04E-17	chr:4:120263688
4	rs7676296	120555687	G	0.2904	5.13E-17	chr:4:120263688
4	rs10009566	120558608	C	0.2934	6.08E-17	chr:4:120263688
4	rs10012252	120559139	C	0.2934	6.08E-17	chr:4:120263688
4	rs55661135	120506153	A	3.62	6.62E-17	chr:4:120263688
4	rs55777923	120553264	T	0.2822	7.26E-17	chr:4:120263688
4	rs9998585	120396802	A	0.2546	8.09E-17	chr:4:120263688
4	rs10006793	120390791	G	0.2566	1.11E-16	chr:4:120263688
4	rs11098526	120390359	T	0.2566	1.11E-16	chr:4:120263688
4	rs11098527	120399906	G	0.2566	1.11E-16	chr:4:120263688
4	rs11934940	120389369	G	0.2566	1.11E-16	chr:4:120263688
4	rs12499324	120393786	T	0.2566	1.11E-16	chr:4:120263688
4	rs12505953	120385681	A	0.2566	1.11E-16	chr:4:120263688
4	rs34004415	120381140	A	0.2566	1.11E-16	chr:4:120263688
4	rs6822679	120402702	C	0.2566	1.11E-16	chr:4:120263688
4	rs6842762	120398236	T	0.2566	1.11E-16	chr:4:120263688
4	rs6848389	120402622	C	0.2566	1.11E-16	chr:4:120263688
4	rs71614443	120379388	C	0.2566	1.11E-16	chr:4:120263688
4	rs9994730	120381564	C	0.2566	1.11E-16	chr:4:120263688
4	rs368193355	120327846	C	0.2339	1.34E-16	chr:4:120263688
4	rs9995244	120321858	G	0.2644	1.40E-16	chr:4:120263688
4	rs13134132	120324615	A	0.2981	1.57E-16	chr:4:120263688
4	rs10017371	120293776	G	0.2598	2.00E-16	chr:4:120263688
4	rs11729991	120292700	T	0.2598	2.00E-16	chr:4:120263688
4	rs12505469	120249585	T	0.2598	2.00E-16	chr:4:120263688
4	rs10020141	120318870	C	0.2645	2.04E-16	chr:4:120263688
4	rs3775842	120427732	G	3.807	2.19E-16	chr:4:120263688
4	rs6844263	120433807	A	3.807	2.19E-16	chr:4:120263688

4	rs140982995	120455483	A	3.805	2.57E-16	chr:4:120263688
4	rs10008386	120318809	A	0.2669	2.78E-16	chr:4:120263688
4	rs10008392	120318839	A	0.2669	2.78E-16	chr:4:120263688
4	rs12504149	120318577	A	0.2669	2.78E-16	chr:4:120263688
4	rs12508173	120318526	C	0.2669	2.78E-16	chr:4:120263688
4	rs12509234	120319434	C	0.2669	2.78E-16	chr:4:120263688
4	rs6833815	120318921	A	0.2669	2.78E-16	chr:4:120263688
4	rs13104396	120285649	T	0.2623	2.82E-16	chr:4:120263688
4	rs1022145	120230979	G	0.2623	2.84E-16	chr:4:120263688
4	rs7674713	120322257	C	0.2633	2.86E-16	chr:4:120263688
4	rs10518328	120401779	A	0.2612	2.86E-16	chr:4:120263688
4	rs11940028	120411907	C	0.2649	3.72E-16	chr:4:120263688
4	rs4336213	120236469	T	0.3106	3.75E-16	chr:4:120263688
4	rs11098512	120314161	T	0.2642	3.77E-16	chr:4:120263688
4	rs71629403	120372567	T	0.2626	3.94E-16	chr:4:120263688
4	rs10006877	120242793	T	0.2648	4.00E-16	chr:4:120263688
4	rs1002152	120273387	A	0.2648	4.00E-16	chr:4:120263688
4	rs1546502	120235898	G	0.2648	4.00E-16	chr:4:120263688
4	rs878373	120237484	T	0.2648	4.00E-16	chr:4:120263688
4	rs3955380	120334410	A	0.2629	4.00E-16	chr:4:120263688
4	rs10014719	120320715	A	0.2677	4.00E-16	chr:4:120263688
4	rs12506148	120320348	T	0.2677	4.00E-16	chr:4:120263688
4	rs10028669	120295197	T	0.2643	4.09E-16	chr:4:120263688
4	rs71614437	120370156	C	0.2634	4.24E-16	chr:4:120263688
4	rs10006706	120409152	C	0.2656	5.38E-16	chr:4:120263688
4	rs10031483	120422636	C	0.2656	5.38E-16	chr:4:120263688
4	rs10031665	120422852	C	0.2656	5.38E-16	chr:4:120263688
4	rs11098530	120413154	T	0.2656	5.38E-16	chr:4:120263688
4	rs11729521	120416788	C	0.2656	5.38E-16	chr:4:120263688
4	rs11734241	120416872	G	0.2656	5.38E-16	chr:4:120263688
4	rs11935596	120412457	C	0.2656	5.38E-16	chr:4:120263688
4	rs12507964	120413061	A	0.2656	5.38E-16	chr:4:120263688
4	rs12510138	120423935	C	0.2656	5.38E-16	chr:4:120263688
4	rs2306455	120421969	A	0.2656	5.38E-16	chr:4:120263688
4	rs28685688	120420334	C	0.2656	5.38E-16	chr:4:120263688
4	rs28845498	120405186	A	0.2656	5.38E-16	chr:4:120263688
4	rs3733523	120423719	T	0.2656	5.38E-16	chr:4:120263688
4	rs4577559	120404043	G	0.2656	5.38E-16	chr:4:120263688
4	rs56270433	120406030	T	0.2656	5.38E-16	chr:4:120263688
4	rs6834796	120414693	C	0.2656	5.38E-16	chr:4:120263688
4	rs6849561	120409694	C	0.2656	5.38E-16	chr:4:120263688
4	rs7656252	120404268	C	0.2656	5.38E-16	chr:4:120263688
4	rs7681544	120411255	C	0.2656	5.38E-16	chr:4:120263688

4	rs7687843	120421211	T	0.2656	5.38E-16	chr:4:120263688
4	rs6838236	120267229	G	0.267	5.54E-16	chr:4:120263688
4	rs13435162	120304634	A	0.267	5.63E-16	chr:4:120263688
4	rs28551662	120309556	A	0.267	5.63E-16	chr:4:120263688
4	rs7674556	120303729	G	0.267	5.63E-16	chr:4:120263688
4	rs7695996	120322033	T	0.2701	5.63E-16	chr:4:120263688
4	rs28441656	120376115	G	0.2653	5.65E-16	chr:4:120263688
4	rs1546504	120241179	T	0.2675	5.76E-16	chr:4:120263688
4	rs1546505	120241224	G	0.2675	5.76E-16	chr:4:120263688
4	rs71627800	120360532	G	0.2116	6.04E-16	chr:4:120263688
4	rs71610279	120326643	C	0.2683	6.75E-16	chr:4:120263688
4	rs79446934	120326713	C	0.2683	6.75E-16	chr:4:120263688
4	rs7696465	120322114	A	0.2689	7.18E-16	chr:4:120263688
4	rs7696649	120322177	A	0.2689	7.18E-16	chr:4:120263688
4	rs10016944	120320894	A	0.274	7.41E-16	chr:4:120263688
4	rs550069983;rs77653089	120359650	G	0.2453	7.47E-16	chr:4:120263688
4	rs7672493	120248260	A	0.269	7.47E-16	chr:4:120263688
4	rs2017057	120257711	G	0.2693	7.71E-16	chr:4:120263688
4	rs73842633	120375464	A	0.2674	7.85E-16	chr:4:120263688
4	rs79057525	120375389	G	0.2674	7.85E-16	chr:4:120263688
4	rs200149630	120356941	C	0.2676	7.94E-16	chr:4:120263688
4	rs34792640	120368627	A	0.2676	7.94E-16	chr:4:120263688
4	rs34913743	120324918	G	0.2676	7.94E-16	chr:4:120263688
4	rs3890049	120326283	A	0.2676	7.94E-16	chr:4:120263688
4	rs79244813	120354847	C	0.2676	7.94E-16	chr:4:120263688
4	rs9760442	120367951	G	0.2676	7.94E-16	chr:4:120263688
4	rs10017543	120321420	A	0.2708	7.96E-16	chr:4:120263688
4	rs28571712	120375980	A	0.2678	8.01E-16	chr:4:120263688
4	rs11723839	120299673	A	0.2693	8.18E-16	chr:4:120263688
4	rs28753180	120405367	T	0.2699	9.92E-16	chr:4:120263688
4	rs28789446	120405366	C	0.2699	9.92E-16	chr:4:120263688
4	rs202150896	120367650	T	0.2689	1.01E-15	chr:4:120263688
4	rs7696857	120322321	A	0.2716	1.05E-15	chr:4:120263688
4	rs199863129	120334013	T	0.2622	1.07E-15	chr:4:120263688
4	rs7654762	120328377	C	0.2701	1.11E-15	chr:4:120263688
4	rs9683450	120232144	G	3.972	1.39E-15	chr:4:120263688
4	rs12502423	120424172	C	0.2699	1.40E-15	chr:4:120263688
4	rs10019406	120364619	G	0.2718	1.48E-15	chr:4:120263688
4	rs35494005	120361142	C	0.2718	1.48E-15	chr:4:120263688
4	rs9685784	120366008	T	0.2718	1.48E-15	chr:4:120263688
4	rs2715026	120616805	C	3.587	1.98E-15	chr:4:120263688
4	rs4001198	120325264	T	0.2762	2.00E-15	chr:4:120263688
4	rs1480931	120395809	A	0.2659	2.15E-15	chr:4:120263688

4	rs1480933	120433248	C	3.497	2.76E-15	chr:4:120263688
4	rs71614435	120369711	A	0.2753	3.03E-15	chr:4:120263688
4	rs4563482	120354480	G	0.3329	3.06E-15	chr:4:120263688
4	rs185413498	120327707	T	0.2769	3.08E-15	chr:4:120263688
4	rs1155577	120449810	T	3.494	3.26E-15	chr:4:120263688
4	rs13104219	120438174	G	3.494	3.26E-15	chr:4:120263688
4	rs11098500	120219239	A	0.2691	3.88E-15	chr:4:120263688
4	rs58583086	120556362	G	3.471	3.90E-15	chr:4:120263688
4	rs2583609	120623111	T	3.502	4.07E-15	chr:4:120263688
4	rs2245564	120625812	T	3.502	4.56E-15	chr:4:120263688
4	rs2583608	120620379	C	3.502	4.56E-15	chr:4:120263688
4	rs7696875	120322358	T	0.2727	4.58E-15	chr:4:120263688
4	rs11726229	120211580	T	0.2698	4.73E-15	chr:4:120263688
4	rs12499598	120189303	C	0.2698	4.73E-15	chr:4:120263688
4	rs28419773	120211061	C	0.2698	4.73E-15	chr:4:120263688
4	rs28555550	120211040	G	0.2698	4.73E-15	chr:4:120263688
4	rs7659194	120207147	G	0.2698	4.73E-15	chr:4:120263688
4	rs56173225	120509972	A	3.46	4.91E-15	chr:4:120263688
4	rs2306457	120472839	A	0.2853	5.09E-15	chr:4:120263688
4	rs11722872	120233030	C	0.271	5.15E-15	chr:4:120263688
4	rs142328963	120498647	A	3.432	5.92E-15	chr:4:120263688
4	rs66887589	120509279	C	3.432	5.92E-15	chr:4:120263688
4	rs2892869	120521297	C	3.43	6.74E-15	chr:4:120263688
4	rs72920701	120513230	A	3.43	6.74E-15	chr:4:120263688
4	rs7678973	120511817	C	3.43	6.74E-15	chr:4:120263688
4	rs2248806	120621423	A	3.456	7.50E-15	chr:4:120263688
4	rs10012456	120274039	T	0.2741	7.56E-15	chr:4:120263688
4	rs6847075	120286043	T	0.2741	7.56E-15	chr:4:120263688
4	rs199816242	120199351	T	0.2718	8.02E-15	chr:4:120263688
4	rs13435370	120334666	C	0.271	8.82E-15	chr:4:120263688
4	rs11934328	120376744	T	0.2733	8.94E-15	chr:4:120263688
4	rs11098499	120187611	T	0.2746	9.08E-15	chr:4:120263688
4	rs58601355	120186367	C	0.2746	9.08E-15	chr:4:120263688
4	rs56843000	120516597	C	3.403	9.24E-15	chr:4:120263688
4	rs4504231	120586891	A	0.3241	9.50E-15	chr:4:120263688
4	rs12498539	120468370	T	0.2906	1.04E-14	chr:4:120263688
4	rs34308924	120481431	C	0.2906	1.04E-14	chr:4:120263688
4	rs3775854	120471971	T	0.2906	1.04E-14	chr:4:120263688
4	rs3822194	120471648	G	0.2906	1.04E-14	chr:4:120263688
4	rs3822195	120471660	T	0.2906	1.04E-14	chr:4:120263688
4	rs6829903	120506884	T	0.2906	1.04E-14	chr:4:120263688
4	rs7669520	120515278	C	0.2906	1.04E-14	chr:4:120263688
4	rs9884402	120489982	G	0.2906	1.04E-14	chr:4:120263688

4	rs9995026	120490499	A	0.2906	1.04E-14	chr:4:120263688
4	rs10026736	120384322	C	0.2746	1.05E-14	chr:4:120263688
4	rs12506546	120301618	C	0.2766	1.07E-14	chr:4:120263688
4	rs6853998	120475860	A	0.2909	1.07E-14	chr:4:120263688
4	rs6858777	120475966	G	0.2909	1.07E-14	chr:4:120263688
4	rs71614422	120359340	A	0.32	1.08E-14	chr:4:120263688
4	rs76525361	120359608	T	0.32	1.08E-14	chr:4:120263688
4	rs76651228	120359559	G	0.32	1.08E-14	chr:4:120263688
4	rs72676045	120254663	A	0.2787	1.10E-14	chr:4:120263688
4	rs2252565	120613111	T	3.431	1.20E-14	chr:4:120263688
4	rs6836219	120597941	T	3.431	1.20E-14	chr:4:120263688
4	rs34585914	120189194	T	0.2772	1.27E-14	chr:4:120263688
4	rs2389873	120554714	G	3.278	1.45E-14	chr:4:120263688
4	rs11731756	120478696	G	0.2936	1.53E-14	chr:4:120263688
4	rs11737395	120520704	G	0.2936	1.53E-14	chr:4:120263688
4	rs201236087	120474546	T	0.2936	1.53E-14	chr:4:120263688
4	rs2170276	120485824	C	0.2936	1.53E-14	chr:4:120263688
4	rs62322293	120330655	T	3.52	1.65E-14	chr:4:120263688
4	rs7658561	120479985	C	0.2935	1.68E-14	chr:4:120263688
4	rs7437666	120653063	C	3.394	1.95E-14	chr:4:120263688
4	rs201085338	120347661	A	0.2877	1.96E-14	chr:4:120263688
4	rs199865201	120332408	G	3.461	2.14E-14	chr:4:120263688
4	rs80259299	120336497	G	0.2232	2.20E-14	chr:4:120263688
4	rs9759478	120367998	A	0.2803	2.25E-14	chr:4:120263688
4	rs10027517	120372234	A	3.462	2.59E-14	chr:4:120263688
4	rs12505876	120385472	G	3.462	2.59E-14	chr:4:120263688
4	rs9307477	120421809	G	3.462	2.59E-14	chr:4:120263688
4	rs9684327	120365441	A	3.462	2.59E-14	chr:4:120263688
4	rs6843229	120424087	T	3.518	2.62E-14	chr:4:120263688
4	rs9762870	120367769	A	0.3053	2.77E-14	chr:4:120263688
4	rs2583616	120629473	A	3.384	2.83E-14	chr:4:120263688
4	rs9799664	120326678	C	3.44	2.87E-14	chr:4:120263688
4	rs75690486	120359788	A	0.3234	3.21E-14	chr:4:120263688
4	rs2714961	120628627	C	3.322	3.50E-14	chr:4:120263688
4	rs2583605	120659992	A	3.317	3.70E-14	chr:4:120263688
4	rs201664317	120330379	C	0.3121	3.85E-14	chr:4:120263688
4	rs4834792	120555696	A	3.184	4.01E-14	chr:4:120263688
4	rs2714978	120670213	G	3.087	4.15E-14	chr:4:120263688
4	rs2250346	120648078	T	3.292	4.42E-14	chr:4:120263688
4	rs2714980	120668765	T	3.092	4.54E-14	chr:4:120263688
4	rs6534135	120406302	A	3.401	4.67E-14	chr:4:120263688
4	rs2583617	120644194	A	3.292	4.85E-14	chr:4:120263688
4	rs2714960	120630872	C	3.292	4.85E-14	chr:4:120263688

4	rs9996586	120326942	A	3.38	4.85E-14	chr:4:120263688
4	rs41464847	120532401	G	3.259	4.98E-14	chr:4:120263688
4	rs59590064	120539753	C	3.259	4.98E-14	chr:4:120263688
4	rs6817317	120542827	G	3.259	4.98E-14	chr:4:120263688
4	rs7672519	120544112	T	3.259	4.98E-14	chr:4:120263688
4	rs7681980	120533223	A	3.259	4.98E-14	chr:4:120263688
4	rs78709015	120367728	T	0.3103	5.07E-14	chr:4:120263688
4	rs2583604	120660715	A	3.32	5.14E-14	chr:4:120263688
4	rs2714976	120670555	G	3.287	5.15E-14	chr:4:120263688
4	rs17006190	120418838	G	0.2847	5.16E-14	chr:4:120263688
4	rs3733519	120423448	G	0.2847	5.16E-14	chr:4:120263688
4	rs9683520	120368199	G	3.247	5.24E-14	chr:4:120263688
4	rs7680748	120479978	A	0.3035	5.40E-14	chr:4:120263688
4	rs725449	120656892	T	3.249	6.21E-14	chr:4:120263688
4	rs7661498	120408108	T	3.373	6.32E-14	chr:4:120263688
4	rs4001211	120331544	G	0.302	6.57E-14	chr:4:120263688
4	rs2715016	120452356	C	3.304	6.65E-14	chr:4:120263688
4	rs2256054	120669517	C	3.05	7.24E-14	chr:4:120263688
4	rs2583596	120670120	A	3.045	8.53E-14	chr:4:120263688
4	rs2583597	120670150	A	3.045	8.53E-14	chr:4:120263688
4	rs28571649	120388867	A	0.3175	8.57E-14	chr:4:120263688
4	rs13134517	120405262	G	3.337	8.57E-14	chr:4:120263688
4	rs17005489	120649070	A	3.206	8.98E-14	chr:4:120263688
4	rs11098524	120390032	G	0.3183	9.03E-14	chr:4:120263688
4	rs531826432	120327073	T	0.313	9.87E-14	chr:4:120263688
4	rs77222221	120329212	A	0.2509	1.02E-13	chr:4:120263688
4	rs10019421	120327198	C	3.118	1.13E-13	chr:4:120263688
4	rs10031129	120327201	T	3.118	1.13E-13	chr:4:120263688
4	rs550821123	120327079	T	0.3174	1.25E-13	chr:4:120263688
4	rs74529999	120327854	C	0.3155	1.25E-13	chr:4:120263688
4	rs2127820	120394534	A	0.3209	1.49E-13	chr:4:120263688
4	rs2127821	120394535	C	0.3209	1.49E-13	chr:4:120263688
4	rs10026493	120384194	G	0.321	1.51E-13	chr:4:120263688
4	rs28665282	120384186	T	0.321	1.51E-13	chr:4:120263688
4	rs2583619	120652805	T	3.18	1.54E-13	chr:4:120263688
4	rs74693727	120330155	T	0.3238	1.69E-13	chr:4:120263688
4	rs4511986	120354674	C	0.3172	1.71E-13	chr:4:120263688
4	rs10019522	120364728	G	0.321	1.83E-13	chr:4:120263688
4	rs4337717	120643040	C	0.3575	1.84E-13	chr:4:120263688
4	rs10009450	120391258	G	0.3233	2.02E-13	chr:4:120263688
4	rs10017335	120381523	G	0.3233	2.02E-13	chr:4:120263688
4	rs10017389	120381412	A	0.3233	2.02E-13	chr:4:120263688
4	rs10026748	120384357	A	0.3233	2.02E-13	chr:4:120263688

4	rs10026952	120384564	A	0.3233	2.02E-13	chr:4:120263688
4	rs10032151	120391628	T	0.3233	2.02E-13	chr:4:120263688
4	rs10032158	120391632	T	0.3233	2.02E-13	chr:4:120263688
4	rs11098525	120390152	A	0.3233	2.02E-13	chr:4:120263688
4	rs11941899	120382758	A	0.3233	2.02E-13	chr:4:120263688
4	rs11943877	120389047	T	0.3233	2.02E-13	chr:4:120263688
4	rs12502909	120377529	T	0.3233	2.02E-13	chr:4:120263688
4	rs12505878	120385485	A	0.3233	2.02E-13	chr:4:120263688
4	rs148358770	120382262	C	0.3233	2.02E-13	chr:4:120263688
4	rs150657109	120377707	T	0.3233	2.02E-13	chr:4:120263688
4	rs17046116	120387259	A	0.3233	2.02E-13	chr:4:120263688
4	rs17046118	120387496	G	0.3233	2.02E-13	chr:4:120263688
4	rs185466280	120377740	T	0.3233	2.02E-13	chr:4:120263688
4	rs28374462	120386597	G	0.3233	2.02E-13	chr:4:120263688
4	rs28444327	120387946	G	0.3233	2.02E-13	chr:4:120263688
4	rs28461559	120387932	A	0.3233	2.02E-13	chr:4:120263688
4	rs28479042	120386876	C	0.3233	2.02E-13	chr:4:120263688
4	rs28559989	120386627	T	0.3233	2.02E-13	chr:4:120263688
4	rs28587142	120386753	C	0.3233	2.02E-13	chr:4:120263688
4	rs28632018	120386730	C	0.3233	2.02E-13	chr:4:120263688
4	rs2892848	120381341	T	0.3233	2.02E-13	chr:4:120263688
4	rs34858317	120387110	C	0.3233	2.02E-13	chr:4:120263688
4	rs34858405	120381081	A	0.3233	2.02E-13	chr:4:120263688
4	rs35063680	120387102	C	0.3233	2.02E-13	chr:4:120263688
4	rs4001190	120377619	A	0.3233	2.02E-13	chr:4:120263688
4	rs6837828	120392084	T	0.3233	2.02E-13	chr:4:120263688
4	rs6838814	120392443	A	0.3233	2.02E-13	chr:4:120263688
4	rs71614442	120379346	T	0.3233	2.02E-13	chr:4:120263688
4	rs71614448	120387026	A	0.3233	2.02E-13	chr:4:120263688
4	rs71614449	120387055	T	0.3233	2.02E-13	chr:4:120263688
4	rs7436506	120393769	C	0.3233	2.02E-13	chr:4:120263688
4	rs74602693	120377647	T	0.3233	2.02E-13	chr:4:120263688
4	rs7654587	120388406	T	0.3233	2.02E-13	chr:4:120263688
4	rs7659336	120385341	C	0.3233	2.02E-13	chr:4:120263688
4	rs7681214	120385320	A	0.3233	2.02E-13	chr:4:120263688
4	rs7681978	120383775	C	0.3233	2.02E-13	chr:4:120263688
4	rs7688613	120384803	C	0.3233	2.02E-13	chr:4:120263688
4	rs9993199	120392873	A	0.3233	2.02E-13	chr:4:120263688
4	rs7664591	120627116	C	0.3694	2.08E-13	chr:4:120263688
4	rs2714973	120662172	A	3.109	2.17E-13	chr:4:120263688
4	rs4513552	120354657	C	2.954	2.27E-13	chr:4:120263688
4	rs9684723	120367735	A	2.999	2.30E-13	chr:4:120263688
4	rs58416902	120374639	G	0.3215	2.48E-13	chr:4:120263688

4	rs9684878	120368175	A	3.076	2.54E-13	chr:4:120263688
4	rs3736115	120488703	A	0.3114	2.64E-13	chr:4:120263688
4	rs58452170	120459674	T	0.3094	2.68E-13	chr:4:120263688
4	rs76568951	120355570	C	0.3235	2.69E-13	chr:4:120263688
4	rs1383533	120434576	A	0.3115	2.71E-13	chr:4:120263688
4	rs79870274	120382190	T	0.3256	2.75E-13	chr:4:120263688
4	rs10008459	120394231	C	0.3257	2.76E-13	chr:4:120263688
4	rs4834806	120656656	C	0.3654	2.99E-13	chr:4:120263688
4	rs187271184	120330448	A	0.325	3.13E-13	chr:4:120263688
4	rs190803766	120330449	A	0.325	3.13E-13	chr:4:120263688
4	rs7654529	120388230	A	0.3294	3.33E-13	chr:4:120263688
4	rs7654546	120388255	A	0.3294	3.33E-13	chr:4:120263688
4	rs10008791	120431469	C	0.3122	3.70E-13	chr:4:120263688
4	rs10009626	120470005	C	0.3122	3.70E-13	chr:4:120263688
4	rs10013305	120450424	A	0.3122	3.70E-13	chr:4:120263688
4	rs10030660	120436704	A	0.3122	3.70E-13	chr:4:120263688
4	rs10034450	120455649	G	0.3122	3.70E-13	chr:4:120263688
4	rs1010739	120463471	T	0.3122	3.70E-13	chr:4:120263688
4	rs1010740	120463409	C	0.3122	3.70E-13	chr:4:120263688
4	rs11098531	120465001	A	0.3122	3.70E-13	chr:4:120263688
4	rs11098532	120490726	A	0.3122	3.70E-13	chr:4:120263688
4	rs11736416	120431661	T	0.3122	3.70E-13	chr:4:120263688
4	rs12498599	120468503	T	0.3122	3.70E-13	chr:4:120263688
4	rs12502389	120454191	G	0.3122	3.70E-13	chr:4:120263688
4	rs13128602	120459366	G	0.3122	3.70E-13	chr:4:120263688
4	rs13134665	120426430	C	0.3122	3.70E-13	chr:4:120263688
4	rs13149407	120437825	G	0.3122	3.70E-13	chr:4:120263688
4	rs1383532	120434404	A	0.3122	3.70E-13	chr:4:120263688
4	rs1480936	120462861	T	0.3122	3.70E-13	chr:4:120263688
4	rs1480939	120456927	C	0.3122	3.70E-13	chr:4:120263688
4	rs1480940	120457682	C	0.3122	3.70E-13	chr:4:120263688
4	rs1552092	120488496	T	0.3122	3.70E-13	chr:4:120263688
4	rs1552095	120460306	C	0.3122	3.70E-13	chr:4:120263688
4	rs2291185	120434833	A	0.3122	3.70E-13	chr:4:120263688
4	rs2306456	120472422	G	0.3122	3.70E-13	chr:4:120263688
4	rs28718422	120466304	A	0.3122	3.70E-13	chr:4:120263688
4	rs34868248	120442430	G	0.3122	3.70E-13	chr:4:120263688
4	rs3733525	120447048	G	0.3122	3.70E-13	chr:4:120263688
4	rs3775843	120427844	C	0.3122	3.70E-13	chr:4:120263688
4	rs3775844	120428033	A	0.3122	3.70E-13	chr:4:120263688
4	rs3775845	120432447	C	0.3122	3.70E-13	chr:4:120263688
4	rs3775847	120447642	C	0.3122	3.70E-13	chr:4:120263688
4	rs3775848	120447724	T	0.3122	3.70E-13	chr:4:120263688

4	rs3775849	120450908	C	0.3122	3.70E-13	chr:4:120263688
4	rs3775852	120454556	G	0.3122	3.70E-13	chr:4:120263688
4	rs3822190	120428098	A	0.3122	3.70E-13	chr:4:120263688
4	rs3822191	120428101	T	0.3122	3.70E-13	chr:4:120263688
4	rs4596225	120496444	G	0.3122	3.70E-13	chr:4:120263688
4	rs6534140	120455311	T	0.3122	3.70E-13	chr:4:120263688
4	rs6835635	120458867	C	0.3122	3.70E-13	chr:4:120263688
4	rs6847818	120469250	T	0.3122	3.70E-13	chr:4:120263688
4	rs7657849	120455494	C	0.3122	3.70E-13	chr:4:120263688
4	rs7664986	120429952	C	0.3122	3.70E-13	chr:4:120263688
4	rs7678400	120461957	A	0.3122	3.70E-13	chr:4:120263688
4	rs7688802	120451668	A	0.3122	3.70E-13	chr:4:120263688
4	rs7695620	120452776	A	0.3122	3.70E-13	chr:4:120263688
4	rs7699064	120428309	T	0.3122	3.70E-13	chr:4:120263688
4	rs71612595	120331428	T	0.3275	4.34E-13	chr:4:120263688
4	rs142609610	120327032	A	0.3278	4.42E-13	chr:4:120263688
4	rs11728616	120440676	A	0.3143	4.83E-13	chr:4:120263688
4	rs35111518	120440682	G	0.3143	4.83E-13	chr:4:120263688
4	rs79026312	120440677	A	0.3143	4.83E-13	chr:4:120263688
4	rs71610280	120326648	G	0.3311	5.16E-13	chr:4:120263688
4	rs77299866	120326716	T	0.3311	5.16E-13	chr:4:120263688
4	rs11933966	120476715	A	0.3158	5.50E-13	chr:4:120263688
4	rs11947234	120474859	G	0.3158	5.50E-13	chr:4:120263688
4	rs13140391	120503437	G	0.3158	5.50E-13	chr:4:120263688
4	rs13140409	120503460	G	0.3158	5.50E-13	chr:4:120263688
4	rs2086450	120496217	A	0.3158	5.50E-13	chr:4:120263688
4	rs36040693	120477616	G	0.3158	5.50E-13	chr:4:120263688
4	rs55825515	120486402	G	0.3158	5.50E-13	chr:4:120263688
4	rs3872807	120360251	T	3.015	5.72E-13	chr:4:120263688
4	rs2714970	120657355	T	3.041	5.87E-13	chr:4:120263688
4	rs9683604	120363592	T	0.3305	5.87E-13	chr:4:120263688
4	rs9684938	120363595	G	0.3305	5.87E-13	chr:4:120263688
4	rs4001195	120329676	G	0.3325	6.12E-13	chr:4:120263688
4	rs10023569	120323316	T	0.3307	6.29E-13	chr:4:120263688
4	rs10518331	120323595	T	0.3307	6.29E-13	chr:4:120263688
4	rs11934314	120376540	A	0.3307	6.29E-13	chr:4:120263688
4	rs13133522	120324424	T	0.3307	6.29E-13	chr:4:120263688
4	rs139379776	120332234	A	0.3307	6.29E-13	chr:4:120263688
4	rs140750089	120330539	A	0.3307	6.29E-13	chr:4:120263688
4	rs149185281	120369277	A	0.3307	6.29E-13	chr:4:120263688
4	rs149826784	120330643	A	0.3307	6.29E-13	chr:4:120263688
4	rs200822665	120357250	A	0.3307	6.29E-13	chr:4:120263688
4	rs201742833	120356668	T	0.3307	6.29E-13	chr:4:120263688

4	rs2389802	120325732	A	0.3307	6.29E-13	chr:4:120263688
4	rs28394590	120373738	G	0.3307	6.29E-13	chr:4:120263688
4	rs28529639	120373104	T	0.3307	6.29E-13	chr:4:120263688
4	rs28653183	120372276	G	0.3307	6.29E-13	chr:4:120263688
4	rs28714318	120375830	T	0.3307	6.29E-13	chr:4:120263688
4	rs34378623	120368744	T	0.3307	6.29E-13	chr:4:120263688
4	rs35091806	120325529	A	0.3307	6.29E-13	chr:4:120263688
4	rs35111011	120366526	T	0.3307	6.29E-13	chr:4:120263688
4	rs35165976	120325630	A	0.3307	6.29E-13	chr:4:120263688
4	rs4001185	120376381	C	0.3307	6.29E-13	chr:4:120263688
4	rs66506550	120371445	A	0.3307	6.29E-13	chr:4:120263688
4	rs6820376	120324476	G	0.3307	6.29E-13	chr:4:120263688
4	rs6845714	120326138	T	0.3307	6.29E-13	chr:4:120263688
4	rs6846442	120326323	A	0.3307	6.29E-13	chr:4:120263688
4	rs71612596	120331743	A	0.3307	6.29E-13	chr:4:120263688
4	rs71612597	120331845	C	0.3307	6.29E-13	chr:4:120263688
4	rs71612601	120332595	T	0.3307	6.29E-13	chr:4:120263688
4	rs71612602	120332781	A	0.3307	6.29E-13	chr:4:120263688
4	rs71614434	120368975	T	0.3307	6.29E-13	chr:4:120263688
4	rs71614438	120371252	A	0.3307	6.29E-13	chr:4:120263688
4	rs71629404	120372724	G	0.3307	6.29E-13	chr:4:120263688
4	rs74772011	120332334	C	0.3307	6.29E-13	chr:4:120263688
4	rs7659501	120371552	T	0.3307	6.29E-13	chr:4:120263688
4	rs7659551	120371729	T	0.3307	6.29E-13	chr:4:120263688
4	rs7664458	120371967	G	0.3307	6.29E-13	chr:4:120263688
4	rs77934861	120324707	C	0.3307	6.29E-13	chr:4:120263688
4	rs35985971	120361596	G	0.2885	6.30E-13	chr:4:120263688
4	rs76837541	120375428	G	0.3311	6.41E-13	chr:4:120263688
4	rs71610281	120328001	C	0.3337	6.50E-13	chr:4:120263688
4	rs10026516	120384253	A	0.3341	6.87E-13	chr:4:120263688
4	rs115044484	120326887	G	0.3331	7.87E-13	chr:4:120263688
4	rs370222454	120359691	G	0.3332	8.46E-13	chr:4:120263688
4	rs532415426	120359632	A	0.3332	8.46E-13	chr:4:120263688
4	rs545772518	120359643	G	0.3332	8.46E-13	chr:4:120263688
4	rs559457335	120359644	G	0.3332	8.46E-13	chr:4:120263688
4	rs563816604	120359627	G	0.3332	8.46E-13	chr:4:120263688
4	rs28634456	120375778	G	0.3337	8.51E-13	chr:4:120263688
4	rs78332141	120375782	T	0.3337	8.51E-13	chr:4:120263688
4	rs12508504	120410607	C	0.3357	8.57E-13	chr:4:120263688
4	rs28626372	120373262	T	0.3333	8.60E-13	chr:4:120263688
4	rs7654984	120328520	G	0.3337	8.65E-13	chr:4:120263688
4	rs4834772	120272316	G	2.358	9.00E-13	chr:4:120263688
4	rs28394116	120524283	G	0.322	9.24E-13	chr:4:120263688

4	rs10027431	120372142	A	2.846	9.45E-13	chr:4:120263688
4	rs10027432	120372143	A	2.846	9.45E-13	chr:4:120263688
4	rs28655325	120372999	G	0.3325	9.56E-13	chr:4:120263688
4	rs28567426	120373354	G	0.3371	1.04E-12	chr:4:120263688
4	rs4368585	120354482	G	0.336	1.04E-12	chr:4:120263688
4	rs72489565	120326819	C	0.3353	1.08E-12	chr:4:120263688
4	rs34566984	120361270	A	0.3358	1.12E-12	chr:4:120263688
4	rs9683709	120365714	A	0.3358	1.12E-12	chr:4:120263688
4	rs9685777	120365965	A	0.3358	1.12E-12	chr:4:120263688
4	rs28495013	120375831	C	0.3359	1.13E-12	chr:4:120263688
4	rs75396180	120375727	A	0.3359	1.13E-12	chr:4:120263688
4	rs80242894	120375752	A	0.3359	1.13E-12	chr:4:120263688
4	rs192039339	120333191	C	0.3361	1.15E-12	chr:4:120263688
4	rs4001175	120500667	A	0.3193	1.16E-12	chr:4:120263688
4	rs10006525	120408931	G	0.3369	1.19E-12	chr:4:120263688
4	rs13145352	120409963	C	0.337	1.21E-12	chr:4:120263688
4	rs17005535	120411562	T	0.3373	1.25E-12	chr:4:120263688
4	rs181331234	120327890	C	0.3359	1.31E-12	chr:4:120263688
4	rs7655048	120328284	A	0.3381	1.40E-12	chr:4:120263688
4	rs76798497	120667915	C	3.56	1.40E-12	chr:4:120263688
4	rs6846620	120326404	A	0.3395	1.43E-12	chr:4:120263688
4	rs7655723	120328666	A	0.3384	1.43E-12	chr:4:120263688
4	rs71610282	120329093	C	0.337	1.43E-12	chr:4:120263688
4	rs7654700	120328125	A	0.3382	1.44E-12	chr:4:120263688
4	rs9683609	120363692	G	0.3381	1.49E-12	chr:4:120263688
4	rs115227179	120355773	T	0.339	1.51E-12	chr:4:120263688
4	rs75122014	120362426	C	0.3389	1.57E-12	chr:4:120263688
4	rs370990577	120327271	C	0.3252	1.82E-12	chr:4:120263688
4	rs11735819	120290194	C	2.557	1.86E-12	chr:4:120263688
4	rs76541370	120498280	C	2.892	2.06E-12	chr:4:120263688
4	rs116952757	120362969	C	0.3412	2.08E-12	chr:4:120263688
4	rs17009122	120362403	A	0.3412	2.08E-12	chr:4:120263688
4	rs527513020	120358891	C	0.242	2.26E-12	chr:4:120263688
4	rs201087782	120327650	G	0.3449	2.36E-12	chr:4:120263688
4	rs12648182	120503891	A	2.877	2.37E-12	chr:4:120263688
4	rs9760422	120354126	A	2.816	2.50E-12	chr:4:120263688
4	rs59182778	120374798	C	0.3436	2.94E-12	chr:4:120263688
4	rs28663988	120620388	C	0.3846	3.06E-12	chr:4:120263688
4	rs146531121	120174794	A	0.306	3.68E-12	chr:4:120263688
4	rs6814412	120170851	A	0.306	3.68E-12	chr:4:120263688
4	rs34147052	120361591	C	0.3504	3.85E-12	chr:4:120263688
4	rs34965784	120361586	C	0.3504	3.85E-12	chr:4:120263688
4	rs6841872	120158685	T	3.018	4.00E-12	chr:4:120263688

4	rs200207680	120338383	T	0.3453	4.24E-12	chr:4:120263688
4	rs370208580	120333619	G	0.2168	4.42E-12	chr:4:120263688
4	rs12512517	120173245	G	0.3093	5.22E-12	chr:4:120263688
4	rs28549914	120322781	A	0.3555	6.77E-12	chr:4:120263688
4	rs1511018	120161580	T	0.3113	6.85E-12	chr:4:120263688
4	rs1511019	120166007	G	0.3113	6.85E-12	chr:4:120263688
4	rs6837898	120179154	T	0.3113	6.85E-12	chr:4:120263688
4	rs56372521	120374431	T	0.3354	7.07E-12	chr:4:120263688
4	rs78958430	120338339	T	0.3501	7.13E-12	chr:4:120263688
4	rs79262354	120338335	T	0.3501	7.13E-12	chr:4:120263688
4	rs12644843	120169477	T	2.996	8.37E-12	chr:4:120263688
4	rs113574635	120279291	G	0.2536	1.02E-11	chr:4:120263688
4	rs9637625	120276345	G	0.2536	1.02E-11	chr:4:120263688
4	rs9637626	120276692	A	0.2536	1.02E-11	chr:4:120263688
4	rs6833334	120504227	T	0.3287	1.05E-11	chr:4:120263688
4	rs12509811	120635685	C	0.3451	1.18E-11	chr:4:120263688
4	rs6534139	120449456	A	0.3384	1.25E-11	chr:4:120263688
4	rs1511016	120170355	A	2.86	1.35E-11	chr:4:120263688
4	rs4496567	120175625	A	2.86	1.35E-11	chr:4:120263688
4	rs6534131	120180195	A	2.86	1.35E-11	chr:4:120263688
4	rs6823331	120180790	T	2.86	1.35E-11	chr:4:120263688
4	rs7676969	120173724	C	2.86	1.35E-11	chr:4:120263688
4	rs28504370	120388890	A	0.362	1.49E-11	chr:4:120263688
4	rs35434465	120169378	G	0.3268	1.69E-11	chr:4:120263688
4	rs3756156	120524841	A	0.3396	2.28E-11	chr:4:120263688
4	rs7656644	120228035	G	2.707	2.46E-11	chr:4:120263688
4	rs34954036	120366927	A	0.3612	2.73E-11	chr:4:120263688
4	rs13113112	120156040	T	0.3258	2.76E-11	chr:4:120263688
4	rs565835685	120358886	G	0.2564	2.94E-11	chr:4:120263688
4	rs13136462	120543173	T	0.3424	3.02E-11	chr:4:120263688
4	rs11723090	120490592	C	0.3346	3.09E-11	chr:4:120263688
4	rs4998488	120500841	C	0.3346	3.09E-11	chr:4:120263688
4	rs59732491	120489588	C	0.3346	3.09E-11	chr:4:120263688
4	rs200940395	120333454	C	0.3213	3.13E-11	chr:4:120263688
4	rs143700375	120254198	A	0.2645	3.44E-11	chr:4:120263688
4	rs12505735	120532956	A	0.3454	4.11E-11	chr:4:120263688
4	rs117030010	120333095	C	0.3401	4.75E-11	chr:4:120263688
4	rs10007283	120619656	G	0.3982	4.81E-11	chr:4:120263688
4	rs3956479	120360863	G	2.472	4.98E-11	chr:4:120263688
4	rs3956480	120360861	C	2.472	4.98E-11	chr:4:120263688
4	rs4001200	120378665	G	0.1842	5.29E-11	chr:4:120263688
4	rs4079144	120325432	T	0.3962	5.61E-11	chr:4:120263688
4	rs10034661	120244085	A	2.966	5.81E-11	chr:4:120263688

4	rs28535956	120536858	C	0.3496	6.05E-11	chr:4:120263688
4	rs6849889	120186348	C	0.3954	6.48E-11	chr:4:120263688
4	rs6534133	120270703	C	3.303	7.99E-11	chr:4:120263688
4	rs376876178	120333615	G	0.2907	8.31E-11	chr:4:120263688
4	rs13116504	120209412	C	0.3978	8.51E-11	chr:4:120263688
4	rs13133044	120201252	C	0.3978	8.51E-11	chr:4:120263688
4	rs35643470	120184948	C	0.3978	8.51E-11	chr:4:120263688
4	rs201495048	120333126	A	0.3484	9.10E-11	chr:4:120263688
4	rs28690296	120685139	C	0.2494	9.27E-11	chr:4:120263688
4	rs4834802	120614296	C	0.408	9.29E-11	chr:4:120263688
4	rs146743180	120497433	T	2.572	9.59E-11	chr:4:120263688
4	rs80052400	120498134	C	2.572	9.59E-11	chr:4:120263688
4	rs28578366	120536905	T	0.35	9.77E-11	chr:4:120263688
4	rs10023652	120662947	C	0.4122	1.02E-10	chr:4:120263688
4	rs36173322	120496491	T	2.566	1.15E-10	chr:4:120263688
4	rs2389903	120646281	G	0.4199	1.25E-10	chr:4:120263688
4	rs78716734	120642117	G	0.4168	1.36E-10	chr:4:120263688
4	rs59516282	120502016	A	2.543	1.40E-10	chr:4:120263688
4	rs202019903	120344304	C	0.3175	1.56E-10	chr:4:120263688
4	rs10518337	120574874	C	2.853	1.58E-10	chr:4:120263688
4	rs4094	120619421	G	0.4211	1.63E-10	chr:4:120263688
4	rs57597755	120640021	C	0.4184	1.66E-10	chr:4:120263688
4	rs7683474	120612853	C	0.4115	1.83E-10	chr:4:120263688
4	rs55876624	120629576	C	0.4209	1.84E-10	chr:4:120263688
4	rs35138262	120361169	G	2.546	2.28E-10	chr:4:120263688
4	rs569705290	120333521	C	0.3638	2.43E-10	chr:4:120263688
4	rs13122709	120555356	A	0.3682	3.26E-10	chr:4:120263688
4	rs12512646	120585733	A	0.4145	3.58E-10	chr:4:120263688
4	rs11737400	120279335	G	0.4404	4.45E-10	chr:4:120263688
4	rs13149954	120279041	A	0.4404	4.45E-10	chr:4:120263688
4	rs13150541	120279284	A	0.4404	4.45E-10	chr:4:120263688
4	rs28580295	120278873	T	0.4404	4.45E-10	chr:4:120263688
4	rs62320738	120279420	A	0.4404	4.45E-10	chr:4:120263688
4	rs62320739	120279459	C	0.4404	4.45E-10	chr:4:120263688
4	rs71627791	120278765	G	0.4404	4.45E-10	chr:4:120263688
4	rs28450134	120376318	C	0.3883	4.49E-10	chr:4:120263688
4	rs12499377	120587215	A	0.4006	4.66E-10	chr:4:120263688
4	rs4434234	120258230	T	3.153	5.09E-10	chr:4:120263688
4	rs11734863	120673259	G	2.401	5.15E-10	chr:4:120263688
4	rs62320740	120279486	A	0.4417	5.25E-10	chr:4:120263688
4	rs10029669	120619666	T	0.4276	5.64E-10	chr:4:120263688
4	rs9994488	120587781	T	0.4229	5.72E-10	chr:4:120263688
4	rs12504773	120562149	C	0.3749	6.36E-10	chr:4:120263688

4	rs7694773	120652993	A	0.4111	6.70E-10	chr:4:120263688
4	rs10014947	120280077	A	0.4464	7.29E-10	chr:4:120263688
4	rs2389875	120557014	T	2.63	8.00E-10	chr:4:120263688
4	rs558588080	120348891	A	0.2044	8.44E-10	chr:4:120263688
4	rs138401876	120370298	A	0.3951	9.84E-10	chr:4:120263688
4	rs7680914	120563053	G	0.3805	1.07E-09	chr:4:120263688
4	rs150043944	120278513	T	3.11	1.09E-09	chr:4:120263688
4	rs151273612	120288457	T	3.11	1.09E-09	chr:4:120263688
4	rs56082510	120288041	T	3.11	1.09E-09	chr:4:120263688
4	rs57842733	120285718	T	3.11	1.09E-09	chr:4:120263688
4	rs78716570	120281377	A	3.11	1.09E-09	chr:4:120263688
4	rs9994354	120263158	C	3.11	1.09E-09	chr:4:120263688
4	rs10026625	120280136	C	0.4545	1.15E-09	chr:4:120263688
4	rs28866816	120553935	T	0.1823	1.41E-09	chr:4:120263688
4	rs4833613	120150156	A	0.4434	1.42E-09	chr:4:120263688
4	rs1799883	120241902	T	3.077	1.49E-09	chr:4:120263688
4	rs11722660	120643106	C	0.4259	1.54E-09	chr:4:120263688
4	rs28651206	120316172	T	0.1814	1.74E-09	chr:4:120263688
4	rs4513553	120298549	G	3.051	1.97E-09	chr:4:120263688
4	rs4613552	120296250	A	3.051	1.97E-09	chr:4:120263688
4	rs57516093	120299780	G	3.051	1.97E-09	chr:4:120263688
4	rs74942216	120295084	G	3.051	1.97E-09	chr:4:120263688
4	rs368648502	120331079	G	0.4226	2.03E-09	chr:4:120263688
4	rs4476582	120313014	C	3.507	2.25E-09	chr:4:120263688
4	rs4833624	120585497	T	0.43	2.32E-09	chr:4:120263688
4	rs3775841	120425777	C	0.4213	2.38E-09	chr:4:120263688
4	rs1825061	120388902	T	0.4143	2.50E-09	chr:4:120263688
4	rs28877102	120555266	T	0.1939	2.60E-09	chr:4:120263688
4	rs4637383	120305966	A	3.021	2.61E-09	chr:4:120263688
4	rs12331776	120617412	A	0.4447	2.67E-09	chr:4:120263688
4	rs10155235	120544391	G	0.1873	2.77E-09	chr:4:120263688
4	rs60308574	120526037	C	0.1873	2.77E-09	chr:4:120263688
4	rs6816375	120297670	T	3.414	2.92E-09	chr:4:120263688
4	rs62321169	120591368	T	2.322	2.93E-09	chr:4:120263688
4	rs9994651	120587803	A	0.4409	2.96E-09	chr:4:120263688
4	rs374110121	120254786	A	0.1977	2.97E-09	chr:4:120263688
4	rs11946739	120266472	T	4.08	3.09E-09	chr:4:120263688
4	rs186992684	120336473	T	0.4665	3.24E-09	chr:4:120263688
4	rs2389874	120554991	A	0.4496	3.35E-09	chr:4:120263688
4	rs12646466	120268321	G	4.107	3.44E-09	chr:4:120263688
4	rs11935130	120241725	A	4.12	3.49E-09	chr:4:120263688
4	rs574704366	120246403	G	4.12	3.49E-09	chr:4:120263688
4	rs17295253	120613018	A	0.4532	3.50E-09	chr:4:120263688

4	rs11726524	120643124	C	0.4418	3.89E-09	chr:4:120263688
4	rs150332522	120361870	G	0.2257	4.18E-09	chr:4:120263688
4	rs78624933	120338651	C	0.3925	4.87E-09	chr:4:120263688
4	rs2389870	120522038	C	0.3886	6.32E-09	chr:4:120263688
4	rs6821717	120599661	C	2.263	6.64E-09	chr:4:120263688
4	rs6857105	120222298	G	0.455	6.87E-09	chr:4:120263688
4	rs11722624	120673096	T	2.251	7.13E-09	chr:4:120263688
4	rs9997209	120683244	G	2.262	7.40E-09	chr:4:120263688
4	rs6840053	120292233	A	3.995	7.59E-09	chr:4:120263688
4	rs62328426	120247732	C	4.007	7.75E-09	chr:4:120263688
4	rs115200920	120314057	A	0.2363	8.49E-09	chr:4:120263688
4	rs190330001	120386251	C	0.2363	8.49E-09	chr:4:120263688
4	rs548685104	120303483	C	0.2363	8.49E-09	chr:4:120263688
4	rs2252621	120675026	C	2.382	9.03E-09	chr:4:120263688
4	rs4001284	120334957	A	1.963	9.38E-09	chr:4:120263688
4	rs4440203	120265438	G	3.479	9.89E-09	chr:4:120263688
4	rs180772254	120257545	C	0.2188	1.00E-08	chr:4:120263688
4	rs10006850	120212521	G	2.616	1.02E-08	chr:4:120263688
4	rs11944880	120159334	T	0.4478	1.06E-08	chr:4:120263688
4	rs7675857	120680581	C	2.239	1.11E-08	chr:4:120263688
4	rs4001176	120500705	G	0.4481	1.17E-08	chr:4:120263688
4	rs11937425	120235999	C	2.441	1.18E-08	chr:4:120263688
4	rs6822909	120691218	T	2.503	1.29E-08	chr:4:120263688
4	rs7681373	120207076	G	2.59	1.29E-08	chr:4:120263688
4	rs142970247	120499656	C	2.234	1.46E-08	chr:4:120263688
4	rs59319494	120696060	A	2.523	1.56E-08	chr:4:120263688
4	rs7693769	120658676	C	0.4642	1.70E-08	chr:4:120263688
4	rs11725321	120611912	G	0.4356	1.77E-08	chr:4:120263688
4	rs6534149	120559292	G	2.191	1.78E-08	chr:4:120263688
4	rs10029750	120172543	G	0.4488	1.86E-08	chr:4:120263688
4	rs4834765	120168233	C	2.525	2.08E-08	chr:4:120263688
4	rs7356185	120167111	T	2.525	2.08E-08	chr:4:120263688
4	rs10005548	120298406	C	0.1718	2.11E-08	chr:4:120263688
4	rs10021584	120310390	G	0.1718	2.11E-08	chr:4:120263688
4	rs28590285	120314988	A	0.1718	2.11E-08	chr:4:120263688
4	rs28622207	120312520	A	0.1718	2.11E-08	chr:4:120263688
4	rs28805329	120305330	C	0.1718	2.11E-08	chr:4:120263688
4	rs28819763	120306716	C	0.1718	2.11E-08	chr:4:120263688
4	rs28847696	120307385	G	0.1718	2.11E-08	chr:4:120263688
4	rs28876736	120301564	T	0.1718	2.11E-08	chr:4:120263688
4	rs536614865	120305383	A	0.1718	2.11E-08	chr:4:120263688
4	rs72918555	120312875	A	0.1718	2.11E-08	chr:4:120263688
4	rs10000726	120210764	G	2.523	2.30E-08	chr:4:120263688

4	rs10010174	120206572	A	2.523	2.30E-08	chr:4:120263688
4	rs80162044	120229263	T	0.2488	2.41E-08	chr:4:120263688
4	rs77857888	120667614	A	0.4041	2.42E-08	chr:4:120263688
4	rs190040520	120269282	T	0.2496	2.43E-08	chr:4:120263688
4	rs2127822	120430787	G	0.2775	2.46E-08	chr:4:120263688
4	rs75475204	120442748	A	0.2775	2.46E-08	chr:4:120263688
4	rs146990217	120258389	C	0.2499	2.49E-08	chr:4:120263688
4	rs7694483	120405929	T	2.199	2.52E-08	chr:4:120263688
4	rs13139045	120168588	C	0.4553	2.70E-08	chr:4:120263688
4	rs10010696	120164303	C	0.4547	2.97E-08	chr:4:120263688
4	rs10022262	120175152	G	0.4547	2.97E-08	chr:4:120263688
4	rs10213342	120175237	G	0.4547	2.97E-08	chr:4:120263688
4	rs11098496	120167466	G	0.4547	2.97E-08	chr:4:120263688
4	rs11729897	120174453	T	0.4547	2.97E-08	chr:4:120263688
4	rs11734519	120171071	G	0.4547	2.97E-08	chr:4:120263688
4	rs12501610	120175763	T	0.4547	2.97E-08	chr:4:120263688
4	rs1397608	120161744	G	0.4547	2.97E-08	chr:4:120263688
4	rs1511015	120170473	C	0.4547	2.97E-08	chr:4:120263688
4	rs34630083	120174569	G	0.4547	2.97E-08	chr:4:120263688
4	rs35197422	120169314	G	0.4547	2.97E-08	chr:4:120263688
4	rs7684942	120162201	T	0.4547	2.97E-08	chr:4:120263688
4	rs7685268	120162188	A	0.4547	2.97E-08	chr:4:120263688
4	rs59867181	120556467	T	2.151	2.99E-08	chr:4:120263688
4	rs7699252	120659198	G	0.4713	3.14E-08	chr:4:120263688
4	rs2714985	120664142	G	2.172	3.36E-08	chr:4:120263688
4	rs5008452	120621752	T	0.4648	3.43E-08	chr:4:120263688
4	rs11737671	120686198	T	0.447	3.57E-08	chr:4:120263688
4	rs56324051	120633203	A	0.269	3.60E-08	chr:4:120263688
4	rs72680938	120629974	C	0.269	3.60E-08	chr:4:120263688
4	rs35271032	120156659	T	0.454	3.61E-08	chr:4:120263688
4	rs115559320	120277729	C	0.257	3.64E-08	chr:4:120263688
4	rs146281946	120277665	G	0.257	3.64E-08	chr:4:120263688
4	rs17050351	120233152	G	0.257	3.64E-08	chr:4:120263688
4	rs74628006	120238963	G	0.257	3.64E-08	chr:4:120263688
4	rs13435802	120177960	T	0.4574	3.73E-08	chr:4:120263688
4	rs12506395	120185094	T	0.4576	3.82E-08	chr:4:120263688
4	rs28374891	120183550	A	0.4576	3.82E-08	chr:4:120263688
4	rs878375	120237574	C	0.4806	3.83E-08	chr:4:120263688
4	rs953966	120171342	G	2.459	3.88E-08	chr:4:120263688
4	rs34616304	120169435	T	0.4566	3.90E-08	chr:4:120263688
4	rs114798996	120690303	G	0.2508	4.05E-08	chr:4:120263688
4	rs34209241	120634499	C	0.4079	4.12E-08	chr:4:120263688
4	rs4834804	120633428	T	0.4079	4.12E-08	chr:4:120263688

4	rs2389879	120557684	A	2.128	4.18E-08	chr:4:120263688
4	rs10857065	120183959	G	2.457	4.29E-08	chr:4:120263688
4	rs10000230	120261439	C	0.1782	4.36E-08	chr:4:120263688
4	rs10006040	120294970	G	0.1782	4.36E-08	chr:4:120263688
4	rs10010547	120261226	G	0.1782	4.36E-08	chr:4:120263688
4	rs10012406	120296884	G	0.1782	4.36E-08	chr:4:120263688
4	rs10022551	120300655	T	0.1782	4.36E-08	chr:4:120263688
4	rs10028634	120320939	A	0.1782	4.36E-08	chr:4:120263688
4	rs11098518	120319718	C	0.1782	4.36E-08	chr:4:120263688
4	rs11098519	120321396	G	0.1782	4.36E-08	chr:4:120263688
4	rs11098520	120321601	A	0.1782	4.36E-08	chr:4:120263688
4	rs28399227	120316205	A	0.1782	4.36E-08	chr:4:120263688
4	rs28619175	120273102	A	0.1782	4.36E-08	chr:4:120263688
4	rs55905129	120318838	T	0.1782	4.36E-08	chr:4:120263688
4	rs57469640	120291308	T	0.1782	4.36E-08	chr:4:120263688
4	rs58691470	120272539	C	0.1782	4.36E-08	chr:4:120263688
4	rs59116083	120317260	C	0.1782	4.36E-08	chr:4:120263688
4	rs61015090	120229944	T	0.1782	4.36E-08	chr:4:120263688
4	rs72914446	120236185	T	0.1782	4.36E-08	chr:4:120263688
4	rs72914498	120250420	G	0.1782	4.36E-08	chr:4:120263688
4	rs142536044	120344540	A	0.2056	4.55E-08	chr:4:120263688
4	rs114698504	120283063	A	0.2566	4.81E-08	chr:4:120263688
4	rs116283501	120281326	A	0.2566	4.81E-08	chr:4:120263688
4	rs116545339	120251911	A	0.2566	4.81E-08	chr:4:120263688
4	rs145397100	120297459	G	0.2566	4.81E-08	chr:4:120263688
4	rs1511024	120240181	T	0.2566	4.81E-08	chr:4:120263688
4	rs17050397	120226765	C	0.2566	4.81E-08	chr:4:120263688
4	rs78026991	120249930	T	0.2566	4.81E-08	chr:4:120263688
4	rs186438419	120268957	A	0.2568	4.83E-08	chr:4:120263688
4	rs74878216	120425940	C	0.3225	4.84E-08	chr:4:120263688
4	rs71610284	120329583	C	0.2679	4.98E-08	chr:4:120263688
4	rs146272386	120329743	A	0.172	5.20E-08	chr:4:120263688
4	rs188978315	120329666	A	0.172	5.20E-08	chr:4:120263688
4	rs28470408	120555668	T	0.172	5.20E-08	chr:4:120263688
4	rs6842973	120694059	A	2.056	5.25E-08	chr:4:120263688
4	rs184550151	120362915	A	0.1743	5.61E-08	chr:4:120263688
4	rs55685100	120641568	C	0.4817	5.72E-08	chr:4:120263688
4	rs1551	120158500	G	0.4613	5.72E-08	chr:4:120263688
4	rs111741197	120222451	G	0.1947	5.78E-08	chr:4:120263688
4	rs9307475	120368556	G	2.105	6.17E-08	chr:4:120263688
4	rs2714965	120676814	T	2.416	6.30E-08	chr:4:120263688
4	rs11931312	120159023	C	0.4645	6.33E-08	chr:4:120263688
4	rs13114751	120156617	A	0.4645	6.33E-08	chr:4:120263688

4	rs35916640	120155852	C	0.4645	6.33E-08	chr:4:120263688
4	rs4145951	120155817	G	0.4645	6.33E-08	chr:4:120263688
4	rs56386062	120155135	C	0.4645	6.33E-08	chr:4:120263688
4	rs6815725	120158410	A	0.4645	6.33E-08	chr:4:120263688
4	rs2583602	120679347	A	2.277	6.58E-08	chr:4:120263688
4	rs113380886	120386537	G	0.1905	7.36E-08	chr:4:120263688
4	rs79319059	120275733	T	0.1827	7.48E-08	chr:4:120263688
4	rs10023107	120328669	T	0.1827	7.56E-08	chr:4:120263688
4	rs184941991	120327437	T	0.1827	7.56E-08	chr:4:120263688
4	rs28552853	120329126	C	0.1827	7.56E-08	chr:4:120263688
4	rs66792078	120112924	C	2.195	7.77E-08	chr:4:120263688
4	rs138958717	120634176	C	0.2799	7.84E-08	chr:4:120263688
4	rs28510336	120262453	A	0.1835	7.89E-08	chr:4:120263688
4	rs2256834	120663939	T	2.125	8.00E-08	chr:4:120263688
4	rs11098515	120317844	G	5.061	8.47E-08	chr:4:120263688
4	rs2389957	120695322	C	2.141	8.96E-08	chr:4:120263688
4	rs4311295	120695280	A	2.141	8.96E-08	chr:4:120263688
4	rs2389958	120695256	C	2.141	8.99E-08	chr:4:120263688
4	rs2892892	120697553	G	2.019	9.33E-08	chr:4:120263688
4	rs539494480	120688288	A	0.2678	9.65E-08	chr:4:120263688
4	rs11732719	120611865	A	0.4438	9.87E-08	chr:4:120263688
4	rs145764064	120373542	G	0.2666	1.03E-07	chr:4:120263688
4	rs184130735	120380121	C	0.2666	1.03E-07	chr:4:120263688
4	rs4447844	120696623	A	2.014	1.07E-07	chr:4:120263688
4	rs6534134	120309946	A	4.989	1.09E-07	chr:4:120263688
4	rs13129661	120152909	G	0.4702	1.10E-07	chr:4:120263688
4	rs1962195	120575866	G	0.4994	1.14E-07	chr:4:120263688
4	rs62321166	120577103	A	0.4994	1.14E-07	chr:4:120263688
4	rs9991735	120572128	T	0.4994	1.14E-07	chr:4:120263688
4	rs925387	120111190	G	2.164	1.19E-07	chr:4:120263688
4	rs116517149	120366951	A	0.2758	1.19E-07	chr:4:120263688
4	rs540083319	120327712	T	0.2208	1.21E-07	chr:4:120263688
4	rs7655035	120617004	T	0.4827	1.25E-07	chr:4:120263688
4	rs6534150	120685502	A	2.218	1.26E-07	chr:4:120263688
4	rs539522533	120329584	A	0.1951	1.29E-07	chr:4:120263688
4	rs184933717	120362885	G	0.218	1.34E-07	chr:4:120263688
4	rs140053614	120380141	A	0.2161	1.37E-07	chr:4:120263688
4	rs150995791	120380540	C	0.2161	1.37E-07	chr:4:120263688
4	rs13103043	120665371	C	0.4871	1.41E-07	chr:4:120263688
4	rs10020420	120287431	C	3.745	1.42E-07	chr:4:120263688
4	rs7681328	120274496	T	3.745	1.42E-07	chr:4:120263688
4	rs72680927	120627177	C	0.275	1.44E-07	chr:4:120263688
4	rs72680937	120629959	T	0.275	1.44E-07	chr:4:120263688

4	rs72680944	120634198	C	0.275	1.44E-07	chr:4:120263688
4	rs2252625	120674899	T	2.195	1.51E-07	chr:4:120263688
4	rs72924312	120584019	A	0.4215	1.52E-07	chr:4:120263688
4	rs10213498	120421607	G	0.2241	1.54E-07	chr:4:120263688
4	rs115128297	120369947	A	0.2757	1.60E-07	chr:4:120263688
4	rs149721973	120386905	G	0.2757	1.60E-07	chr:4:120263688
4	rs377586222	120328165	G	0.2757	1.60E-07	chr:4:120263688
4	rs17049978	120112049	G	2.144	1.73E-07	chr:4:120263688
4	rs4834776	120306578	G	4.875	1.74E-07	chr:4:120263688
4	rs749526	120663649	T	0.4775	1.75E-07	chr:4:120263688
4	rs11731571	120221185	G	1.953	1.82E-07	chr:4:120263688
4	rs192625463	120359735	A	0.2133	1.82E-07	chr:4:120263688
4	rs541659266	120354371	C	0.2133	1.82E-07	chr:4:120263688
4	rs10032392	120515966	A	0.2135	1.86E-07	chr:4:120263688
4	rs28736368	120425076	G	0.1815	1.89E-07	chr:4:120263688
4	rs79549478	120632887	C	0.4301	2.05E-07	chr:4:120263688
4	rs7665086	120394985	G	5.036	2.21E-07	chr:4:120263688
4	rs72680928	120627183	T	0.284	2.24E-07	chr:4:120263688
4	rs72680929	120627288	C	0.284	2.24E-07	chr:4:120263688
4	rs147812813	120325093	T	0.2274	2.26E-07	chr:4:120263688
4	rs28610991	120569861	C	0.5084	2.45E-07	chr:4:120263688
4	rs186394904	120327983	A	0.2856	2.53E-07	chr:4:120263688
4	rs567467134	120359164	C	0.2281	2.59E-07	chr:4:120263688
4	rs17358860	120518064	A	0.4347	2.64E-07	chr:4:120263688
4	rs114276127	120484174	C	0.2963	2.66E-07	chr:4:120263688
4	rs149494214	120506268	C	0.2963	2.66E-07	chr:4:120263688
4	rs373952860	120499913	T	0.2963	2.66E-07	chr:4:120263688
4	rs28615474	120420932	A	0.2299	2.66E-07	chr:4:120263688
4	rs28864814	120404759	C	0.2299	2.66E-07	chr:4:120263688
4	rs28870820	120407023	G	0.2299	2.66E-07	chr:4:120263688
4	rs57295141	120403128	C	0.2299	2.66E-07	chr:4:120263688
4	rs72920607	120396853	T	0.2299	2.66E-07	chr:4:120263688
4	rs72920619	120409668	G	0.2299	2.66E-07	chr:4:120263688
4	rs12331661	120509981	T	0.2232	2.67E-07	chr:4:120263688
4	rs182295274	120364972	C	0.2258	2.69E-07	chr:4:120263688
4	rs185524670	120365146	T	0.2258	2.69E-07	chr:4:120263688
4	rs185987967	120361331	T	0.2258	2.69E-07	chr:4:120263688
4	rs186090164	120365072	A	0.2258	2.69E-07	chr:4:120263688
4	rs189764750	120361087	G	0.2258	2.69E-07	chr:4:120263688
4	rs28535588	120362194	C	0.2258	2.69E-07	chr:4:120263688
4	rs555275051	120365212	T	0.2258	2.69E-07	chr:4:120263688
4	rs56841438	120365478	T	0.2258	2.69E-07	chr:4:120263688
4	rs9993996	120363669	A	0.2258	2.69E-07	chr:4:120263688

4	rs564630005	120351897	T	0.2379	2.71E-07	chr:4:120263688
4	rs3905917	120268435	A	3.647	2.72E-07	chr:4:120263688
4	rs4834773	120277395	G	3.647	2.72E-07	chr:4:120263688
4	rs199515440	120372424	T	0.3952	2.75E-07	chr:4:120263688
4	rs10003953	120438731	C	0.2239	2.81E-07	chr:4:120263688
4	rs10010096	120440411	C	0.2239	2.81E-07	chr:4:120263688
4	rs10010097	120440413	C	0.2239	2.81E-07	chr:4:120263688
4	rs10015714	120509190	A	0.2239	2.81E-07	chr:4:120263688
4	rs10015999	120393442	G	0.2239	2.81E-07	chr:4:120263688
4	rs10021318	120507479	C	0.2239	2.81E-07	chr:4:120263688
4	rs10023384	120471256	G	0.2239	2.81E-07	chr:4:120263688
4	rs10027735	120490305	G	0.2239	2.81E-07	chr:4:120263688
4	rs10031328	120431656	T	0.2239	2.81E-07	chr:4:120263688
4	rs10031493	120366627	A	0.2239	2.81E-07	chr:4:120263688
4	rs10471013	120420247	G	0.2239	2.81E-07	chr:4:120263688
4	rs138837790	120496366	G	0.2239	2.81E-07	chr:4:120263688
4	rs141077493	120366930	T	0.2239	2.81E-07	chr:4:120263688
4	rs181233686	120374932	A	0.2239	2.81E-07	chr:4:120263688
4	rs183139683	120375913	A	0.2239	2.81E-07	chr:4:120263688
4	rs184784499	120371685	A	0.2239	2.81E-07	chr:4:120263688
4	rs187861314	120388040	A	0.2239	2.81E-07	chr:4:120263688
4	rs188395833	120338340	G	0.2239	2.81E-07	chr:4:120263688
4	rs189259285	120329870	T	0.2239	2.81E-07	chr:4:120263688
4	rs189415167	120389621	T	0.2239	2.81E-07	chr:4:120263688
4	rs28380652	120430952	C	0.2239	2.81E-07	chr:4:120263688
4	rs28419430	120547822	T	0.2239	2.81E-07	chr:4:120263688
4	rs28436005	120390576	A	0.2239	2.81E-07	chr:4:120263688
4	rs28461667	120435203	G	0.2239	2.81E-07	chr:4:120263688
4	rs28469881	120418490	C	0.2239	2.81E-07	chr:4:120263688
4	rs28576329	120456845	G	0.2239	2.81E-07	chr:4:120263688
4	rs28634062	120432555	G	0.2239	2.81E-07	chr:4:120263688
4	rs28639769	120388271	C	0.2239	2.81E-07	chr:4:120263688
4	rs28654414	120391595	A	0.2239	2.81E-07	chr:4:120263688
4	rs28661222	120377892	T	0.2239	2.81E-07	chr:4:120263688
4	rs28669906	120412590	A	0.2239	2.81E-07	chr:4:120263688
4	rs529382998	120338515	G	0.2239	2.81E-07	chr:4:120263688
4	rs537132872	120333467	T	0.2239	2.81E-07	chr:4:120263688
4	rs540253240	120338387	T	0.2239	2.81E-07	chr:4:120263688
4	rs548399597	120336898	T	0.2239	2.81E-07	chr:4:120263688
4	rs548630365	120341300	A	0.2239	2.81E-07	chr:4:120263688
4	rs556748979	120369383	T	0.2239	2.81E-07	chr:4:120263688
4	rs56191916	120407746	G	0.2239	2.81E-07	chr:4:120263688
4	rs564413275	120351192	C	0.2239	2.81E-07	chr:4:120263688

4	rs566386400	120379510	A	0.2239	2.81E-07	chr:4:120263688
4	rs57516186	120373425	T	0.2239	2.81E-07	chr:4:120263688
4	rs61655565	120497863	A	0.2239	2.81E-07	chr:4:120263688
4	rs61729464	120432277	G	0.2239	2.81E-07	chr:4:120263688
4	rs72918590	120375759	A	0.2239	2.81E-07	chr:4:120263688
4	rs72918597	120387237	C	0.2239	2.81E-07	chr:4:120263688
4	rs80029731	120386563	G	0.2239	2.81E-07	chr:4:120263688
4	rs9307478	120460662	T	0.2239	2.81E-07	chr:4:120263688
4	rs9884290	120458361	C	0.2239	2.81E-07	chr:4:120263688
4	rs9884542	120392244	G	0.2239	2.81E-07	chr:4:120263688
4	rs9996803	120452328	G	0.2239	2.81E-07	chr:4:120263688
4	rs9997423	120519207	C	0.2239	2.81E-07	chr:4:120263688
4	rs9998244	120500686	T	0.2239	2.81E-07	chr:4:120263688
4	rs28405434	120426371	G	0.2165	2.84E-07	chr:4:120263688
4	rs13101722	120555975	A	1.99	3.17E-07	chr:4:120263688
4	rs11731675	120212514	C	0.5134	3.35E-07	chr:4:120263688
4	rs1022146	120236103	G	0.509	3.68E-07	chr:4:120263688
4	rs2389747	120236102	T	0.509	3.68E-07	chr:4:120263688
4	rs12108288	120483022	G	0.2951	3.84E-07	chr:4:120263688
4	rs17050700	120489535	C	0.2951	3.84E-07	chr:4:120263688
4	rs80327561	120487478	A	0.2951	3.84E-07	chr:4:120263688
4	rs28639494	120512193	T	0.2428	4.01E-07	chr:4:120263688
4	rs10034691	120328726	C	0.2342	4.19E-07	chr:4:120263688
4	rs115479057	120324932	C	0.2342	4.19E-07	chr:4:120263688
4	rs188378849	120325022	A	0.2342	4.19E-07	chr:4:120263688
4	rs28619539	120328217	G	0.2342	4.19E-07	chr:4:120263688
4	rs28665114	120328177	C	0.2342	4.19E-07	chr:4:120263688
4	rs28709577	120324121	C	0.2342	4.19E-07	chr:4:120263688
4	rs61696977	120326010	T	0.2342	4.19E-07	chr:4:120263688
4	rs28450144	120418685	G	0.2628	4.52E-07	chr:4:120263688
4	rs6827780	120406415	G	4.584	4.80E-07	chr:4:120263688
4	rs6845402	120421158	G	4.584	4.80E-07	chr:4:120263688
4	rs78364210	120421396	A	4.584	4.80E-07	chr:4:120263688
4	rs187011726	120379474	T	4.819	4.96E-07	chr:4:120263688
4	rs6534136	120406771	T	4.819	4.96E-07	chr:4:120263688
4	rs6848530	120381808	G	4.819	4.96E-07	chr:4:120263688
4	rs72680912	120625246	C	0.301	5.00E-07	chr:4:120263688
4	rs572028672	120348896	T	0.2305	5.07E-07	chr:4:120263688
4	rs10029882	120466070	A	0.2305	5.08E-07	chr:4:120263688
4	rs545490844	120330912	T	0.2316	5.48E-07	chr:4:120263688
4	rs200974711	120468265	G	0.5248	5.61E-07	chr:4:120263688
4	rs111687566	120393977	A	4.274	6.19E-07	chr:4:120263688
4	rs141773781	120192032	G	0.1815	6.32E-07	chr:4:120263688

4	rs72680956	120641924	G	0.3891	6.32E-07	chr:4:120263688
4	rs9995234	120321827	G	0.4174	6.51E-07	chr:4:120263688
4	rs112880930	120327377	T	0.2454	6.59E-07	chr:4:120263688
4	rs752650	120632387	C	0.4781	6.94E-07	chr:4:120263688
4	rs12649773	120305262	A	2.539	7.11E-07	chr:4:120263688
4	rs142534042	120308936	C	2.539	7.11E-07	chr:4:120263688
4	rs4833617	120305176	A	2.539	7.11E-07	chr:4:120263688
4	rs55827028	120297059	A	2.539	7.11E-07	chr:4:120263688
4	rs62322286	120307242	G	2.539	7.11E-07	chr:4:120263688
4	rs56199127	120641341	C	0.3926	7.54E-07	chr:4:120263688
4	rs189022116	120375119	C	0.2452	7.71E-07	chr:4:120263688
4	rs11731926	120653012	G	0.3773	7.86E-07	chr:4:120263688
4	rs13122890	120601731	T	1.995	7.89E-07	chr:4:120263688
4	rs62322289	120314122	T	2.502	8.07E-07	chr:4:120263688
4	rs60899768	120320771	A	2.869	8.68E-07	chr:4:120263688
4	rs28723049	120620507	A	0.5128	8.88E-07	chr:4:120263688
4	rs62322280	120301120	A	2.498	9.21E-07	chr:4:120263688
4	rs11724055	120470035	A	0.3483	9.35E-07	chr:4:120263688
4	rs9684358	120622560	C	0.5122	9.44E-07	chr:4:120263688
4	rs28869613	120620588	A	0.5132	9.87E-07	chr:4:120263688
4	rs11098511	120306879	A	0.4226	9.98E-07	chr:4:120263688
4	rs113918125	120581639	G	0.4727	1.03E-06	chr:4:120263688
4	rs56905341	120576818	C	2.712	1.19E-06	chr:4:120263688
4	rs7684853	120581633	G	0.5367	1.19E-06	chr:4:120263688
4	rs10019674	120443489	C	0.4232	1.22E-06	chr:4:120263688
4	rs3822192	120445720	C	0.4232	1.22E-06	chr:4:120263688
4	rs7665125	120402079	G	1.945	1.23E-06	chr:4:120263688
4	rs13128673	120297304	A	0.3271	1.26E-06	chr:4:120263688
4	rs555486587	120351800	A	0.2599	1.30E-06	chr:4:120263688
4	rs559693063	120341279	C	0.2608	1.30E-06	chr:4:120263688
4	rs11732173	120327824	T	0.3367	1.32E-06	chr:4:120263688
4	rs114513627	120535428	T	0.3125	1.34E-06	chr:4:120263688
4	rs116420214	120557873	G	0.3125	1.34E-06	chr:4:120263688
4	rs75483765	120546586	T	0.3125	1.34E-06	chr:4:120263688
4	rs79141760	120555974	G	0.3125	1.34E-06	chr:4:120263688
4	rs2389871	120550639	A	3.81	1.35E-06	chr:4:120263688
4	rs6854072	120549435	T	3.81	1.35E-06	chr:4:120263688
4	rs9307480	120533707	T	3.81	1.35E-06	chr:4:120263688
4	rs10518329	120401835	A	1.949	1.35E-06	chr:4:120263688
4	rs874425	120664362	C	0.518	1.39E-06	chr:4:120263688
4	rs3775850	120451018	A	0.3464	1.43E-06	chr:4:120263688
4	rs2714966	120677425	A	2.066	1.45E-06	chr:4:120263688
4	rs2389891	120576177	G	0.5408	1.51E-06	chr:4:120263688

4	rs577516977	120264366	G	0.1691	1.55E-06	chr:4:120263688
4	rs13126596	120140729	G	0.4114	1.56E-06	chr:4:120263688
4	rs68128210	120137819	G	0.4114	1.56E-06	chr:4:120263688
4	rs2389872	120554040	C	3.593	1.64E-06	chr:4:120263688
4	rs28654836	120599090	T	0.4794	1.65E-06	chr:4:120263688
4	rs11732621	120212883	G	0.5371	1.68E-06	chr:4:120263688
4	rs2389883	120567635	C	0.542	1.70E-06	chr:4:120263688
4	rs2389892	120577583	C	0.542	1.70E-06	chr:4:120263688
4	rs7670803	120579321	C	0.542	1.70E-06	chr:4:120263688
4	rs7671191	120579548	C	0.542	1.70E-06	chr:4:120263688
4	rs17005515	120649610	C	0.3191	1.72E-06	chr:4:120263688
4	rs2036862	120135531	G	2.107	1.76E-06	chr:4:120263688
4	rs67110248	120139522	C	2.107	1.76E-06	chr:4:120263688
4	rs72673792	120141616	A	2.107	1.76E-06	chr:4:120263688
4	rs7678043	120698652	T	1.989	1.78E-06	chr:4:120263688
4	rs10013852	120533838	C	3.626	1.79E-06	chr:4:120263688
4	rs10021469	120540908	C	3.626	1.79E-06	chr:4:120263688
4	rs12504983	120532626	G	3.626	1.79E-06	chr:4:120263688
4	rs2389876	120557179	C	3.626	1.79E-06	chr:4:120263688
4	rs2389877	120557187	G	3.626	1.79E-06	chr:4:120263688
4	rs2389878	120557268	G	3.626	1.79E-06	chr:4:120263688
4	rs2953293	120547986	T	3.626	1.79E-06	chr:4:120263688
4	rs2953294	120547987	T	3.626	1.79E-06	chr:4:120263688
4	rs3109370	120546307	A	3.626	1.79E-06	chr:4:120263688
4	rs4287988	120543998	A	3.626	1.79E-06	chr:4:120263688
4	rs4833621	120544884	T	3.626	1.79E-06	chr:4:120263688
4	rs6534145	120537384	A	3.626	1.79E-06	chr:4:120263688
4	rs6534148	120544218	T	3.626	1.79E-06	chr:4:120263688
4	rs6831062	120544784	C	3.626	1.79E-06	chr:4:120263688
4	rs17051339	120573222	T	0.3713	1.81E-06	chr:4:120263688
4	rs10434034	120621676	T	0.3089	1.95E-06	chr:4:120263688
4	rs114569900	120624053	T	0.3089	1.95E-06	chr:4:120263688
4	rs115807288	120624153	A	0.3089	1.95E-06	chr:4:120263688
4	rs141894037	120620858	C	0.3089	1.95E-06	chr:4:120263688
4	rs72678599	120619817	C	0.3089	1.95E-06	chr:4:120263688
4	rs72678601	120619868	T	0.3089	1.95E-06	chr:4:120263688
4	rs72678602	120619989	A	0.3089	1.95E-06	chr:4:120263688
4	rs72680904	120622192	A	0.3089	1.95E-06	chr:4:120263688
4	rs72680906	120622479	A	0.3089	1.95E-06	chr:4:120263688
4	rs72680907	120622607	C	0.3089	1.95E-06	chr:4:120263688
4	rs72680908	120623268	G	0.3089	1.95E-06	chr:4:120263688
4	rs72680911	120625215	A	0.3089	1.95E-06	chr:4:120263688
4	rs72680914	120625473	C	0.3089	1.95E-06	chr:4:120263688

4	rs72680917	120625878	A	0.3089	1.95E-06	chr:4:120263688
4	rs72680918	120626166	C	0.3089	1.95E-06	chr:4:120263688
4	rs7659472	120620910	A	0.3089	1.95E-06	chr:4:120263688
4	rs7682153	120621278	G	0.3089	1.95E-06	chr:4:120263688
4	rs7682717	120621417	A	0.3089	1.95E-06	chr:4:120263688
4	rs10016938	120606183	C	0.4819	1.97E-06	chr:4:120263688
4	rs73842698	120602428	T	0.4819	1.97E-06	chr:4:120263688
4	rs17005698	120414009	A	3.486	2.02E-06	chr:4:120263688
4	rs77408616	120423274	G	3.486	2.02E-06	chr:4:120263688
4	rs2928991	120476677	C	3.724	2.03E-06	chr:4:120263688
4	rs73842685	120584839	A	0.4655	2.07E-06	chr:4:120263688
4	rs12233881	120318169	G	2.397	2.13E-06	chr:4:120263688
4	rs372426471	120344927	G	0.3303	2.20E-06	chr:4:120263688
4	rs4834787	120521526	T	3.705	2.20E-06	chr:4:120263688
4	rs2953290	120549138	A	3.585	2.22E-06	chr:4:120263688
4	rs11098497	120187068	T	0.5407	2.25E-06	chr:4:120263688
4	rs7659403	120207254	A	0.5407	2.25E-06	chr:4:120263688
4	rs62322295	120338592	C	0.3452	2.29E-06	chr:4:120263688
4	rs9996940	120364542	C	1.907	2.33E-06	chr:4:120263688
4	rs28633994	120276037	G	0.4032	2.38E-06	chr:4:120263688
4	rs13103899	120438302	G	0.3538	2.41E-06	chr:4:120263688
4	rs28562576	120760193	G	0.5112	2.46E-06	chr:4:120263688
4	rs67073020	120152557	T	0.5069	2.54E-06	chr:4:120263688
4	rs35264119	120422738	A	0.3367	2.56E-06	chr:4:120263688
4	rs62319578	120408421	G	0.3367	2.56E-06	chr:4:120263688
4	rs2256645	120458932	C	3.798	2.61E-06	chr:4:120263688
4	rs2622497	120463856	A	3.798	2.61E-06	chr:4:120263688
4	rs7694813	120445321	T	3.798	2.61E-06	chr:4:120263688
4	rs13119104	120523140	C	3.667	2.65E-06	chr:4:120263688
4	rs13125819	120523904	A	3.667	2.65E-06	chr:4:120263688
4	rs7439678	120526862	G	3.667	2.65E-06	chr:4:120263688
4	rs80125345	120447079	C	3.667	2.65E-06	chr:4:120263688
4	rs56838032	120329894	G	1.815	2.66E-06	chr:4:120263688
4	rs9996382	120151012	A	0.4685	2.73E-06	chr:4:120263688
4	rs34878806	120235904	A	2.281	2.76E-06	chr:4:120263688
4	rs116497468	120574824	C	0.3206	2.77E-06	chr:4:120263688
4	rs76490003	120568947	C	0.3206	2.77E-06	chr:4:120263688
4	rs372864183	120305645	G	0.345	2.79E-06	chr:4:120263688
4	rs72924305	120581152	C	0.4162	2.81E-06	chr:4:120263688
4	rs71614444	120379436	G	0.4052	2.89E-06	chr:4:120263688
4	rs907736	120289943	T	0.5504	2.92E-06	chr:4:120263688
4	rs12512171	120662564	T	0.5362	2.96E-06	chr:4:120263688
4	rs62319649	120522579	C	2.038	2.97E-06	chr:4:120263688

4	rs13140150	120473191	C	0.3733	3.08E-06	chr:4:120263688
4	rs139839559	120640908	G	0.4085	3.09E-06	chr:4:120263688
4	rs72680953	120640435	G	0.4085	3.09E-06	chr:4:120263688
4	rs72680959	120643960	C	0.4085	3.09E-06	chr:4:120263688
4	rs12331938	120679356	T	0.29	3.11E-06	chr:4:120263688
4	rs10031702	120625231	A	0.2713	3.16E-06	chr:4:120263688
4	rs73842702	120611013	C	0.4904	3.26E-06	chr:4:120263688
4	rs13126170	120323188	A	0.3467	3.28E-06	chr:4:120263688
4	rs62319570	120370058	T	0.3467	3.28E-06	chr:4:120263688
4	rs62319574	120392364	T	0.3467	3.28E-06	chr:4:120263688
4	rs28403131	120285806	T	0.408	3.35E-06	chr:4:120263688
4	rs138790146	120626770	T	0.3173	3.48E-06	chr:4:120263688
4	rs72680920	120626314	T	0.3173	3.48E-06	chr:4:120263688
4	rs72680921	120626325	G	0.3173	3.48E-06	chr:4:120263688
4	rs72680923	120626383	C	0.3173	3.48E-06	chr:4:120263688
4	rs2389884	120530449	C	3.492	3.53E-06	chr:4:120263688
4	rs4146266	120524990	C	3.492	3.53E-06	chr:4:120263688
4	rs143726128	120348512	A	1.7	3.54E-06	chr:4:120263688
4	rs28504181	120622530	G	0.4842	3.54E-06	chr:4:120263688
4	rs10434033	120582336	T	0.4949	3.56E-06	chr:4:120263688
4	rs62328420	120238189	C	2.543	3.59E-06	chr:4:120263688
4	rs2953291	120550144	A	3.391	3.62E-06	chr:4:120263688
4	rs201916895	120199348	C	0.3176	3.74E-06	chr:4:120263688
4	rs372896807	120566079	A	0.5554	3.87E-06	chr:4:120263688
4	rs375123133	120566082	A	0.5554	3.87E-06	chr:4:120263688
4	rs56381021	120566077	C	0.5554	3.87E-06	chr:4:120263688
4	rs7695292	120558446	G	0.4111	3.90E-06	chr:4:120263688
4	rs2036859	120249184	T	2.542	3.96E-06	chr:4:120263688
4	rs988146	120399505	G	0.347	3.97E-06	chr:4:120263688
4	rs56122576	120129336	G	0.426	3.99E-06	chr:4:120263688
4	rs200898556	120500426	T	0.407	4.01E-06	chr:4:120263688
4	rs3733524	120423729	C	0.407	4.01E-06	chr:4:120263688
4	rs9799694	120357017	T	0.3388	4.02E-06	chr:4:120263688
4	rs147174618	120247897	G	0.2098	4.10E-06	chr:4:120263688
4	rs554405164	120330730	A	4.439	4.39E-06	chr:4:120263688
4	rs13133969	120495479	C	0.3641	4.49E-06	chr:4:120263688
4	rs62319609	120491640	G	0.3641	4.49E-06	chr:4:120263688
4	rs4834786	120521360	G	3.423	4.56E-06	chr:4:120263688
4	rs17595769	120275103	T	2.498	4.97E-06	chr:4:120263688
4	rs59225699	120582821	T	0.5023	4.98E-06	chr:4:120263688
4	rs72920685	120506082	C	0.158	5.27E-06	chr:4:120263688
4	rs62320719	120274210	G	2.551	5.32E-06	chr:4:120263688
4	rs28478532	120622231	C	0.4907	5.34E-06	chr:4:120263688

4	rs9995045	120321636	T	0.4256	5.38E-06	chr:4:120263688
4	rs951570	120150467	T	0.4858	5.42E-06	chr:4:120263688
4	rs115001838	120686401	A	0.2528	5.42E-06	chr:4:120263688
4	rs115269783	120683979	G	0.2528	5.42E-06	chr:4:120263688
4	rs74577464	120689419	C	0.2528	5.42E-06	chr:4:120263688
4	rs75584233	120687002	C	0.2528	5.42E-06	chr:4:120263688
4	rs12641176	120688248	A	0.5027	5.42E-06	chr:4:120263688
4	rs10006304	120124305	G	0.4318	5.45E-06	chr:4:120263688
4	rs13138782	120124474	A	0.4318	5.45E-06	chr:4:120263688
4	rs28428953	120334911	T	4.364	5.56E-06	chr:4:120263688
4	rs541516897	120622296	C	0.2949	5.67E-06	chr:4:120263688
4	rs1011054	120202387	A	0.4143	5.68E-06	chr:4:120263688
4	rs79834186	120607253	G	0.4975	5.97E-06	chr:4:120263688
4	rs114974538	120640794	G	0.2889	6.05E-06	chr:4:120263688
4	rs6843509	120220196	A	0.5599	6.12E-06	chr:4:120263688
4	rs116343718	120277281	G	2.474	6.19E-06	chr:4:120263688
4	rs11727068	120277809	A	2.474	6.19E-06	chr:4:120263688
4	rs11728178	120272969	T	2.474	6.19E-06	chr:4:120263688
4	rs11735260	120277811	G	2.474	6.19E-06	chr:4:120263688
4	rs13122306	120275801	T	2.474	6.19E-06	chr:4:120263688
4	rs13139711	120265747	A	2.474	6.19E-06	chr:4:120263688
4	rs17517470	120276316	G	2.474	6.19E-06	chr:4:120263688
4	rs17595713	120261571	G	2.474	6.19E-06	chr:4:120263688
4	rs17595790	120276220	C	2.474	6.19E-06	chr:4:120263688
4	rs3817230	120275499	C	2.474	6.19E-06	chr:4:120263688
4	rs3817242	120274926	G	2.474	6.19E-06	chr:4:120263688
4	rs56818816	120275663	G	2.474	6.19E-06	chr:4:120263688
4	rs62320717	120272087	G	2.474	6.19E-06	chr:4:120263688
4	rs62320718	120273269	A	2.474	6.19E-06	chr:4:120263688
4	rs62320720	120274349	C	2.474	6.19E-06	chr:4:120263688
4	rs62320721	120274612	G	2.474	6.19E-06	chr:4:120263688
4	rs62320722	120274661	T	2.474	6.19E-06	chr:4:120263688
4	rs62320723	120274816	G	2.474	6.19E-06	chr:4:120263688
4	rs62320724	120274878	C	2.474	6.19E-06	chr:4:120263688
4	rs62320725	120275268	G	2.474	6.19E-06	chr:4:120263688
4	rs62320726	120275344	T	2.474	6.19E-06	chr:4:120263688
4	rs62320731	120276840	T	2.474	6.19E-06	chr:4:120263688
4	rs62320732	120276920	G	2.474	6.19E-06	chr:4:120263688
4	rs62320733	120277115	G	2.474	6.19E-06	chr:4:120263688
4	rs62320735	120277500	G	2.474	6.19E-06	chr:4:120263688
4	rs71610263	120261416	A	2.474	6.19E-06	chr:4:120263688
4	rs72918504	120275130	G	2.474	6.19E-06	chr:4:120263688
4	rs75186513	120275052	T	2.474	6.19E-06	chr:4:120263688

4	rs78504630	120275010	C	2.474	6.19E-06	chr:4:120263688
4	rs7436509	120120569	C	1.992	6.32E-06	chr:4:120263688
4	rs113988766	120632631	G	0.4281	6.54E-06	chr:4:120263688
4	rs3749591	120214030	G	0.4224	6.73E-06	chr:4:120263688
4	rs6856110	120759381	C	0.529	6.88E-06	chr:4:120263688
4	rs10002072	120626513	C	0.2726	6.91E-06	chr:4:120263688
4	rs4834775	120301232	A	1.968	6.91E-06	chr:4:120263688
4	rs10013032	120149419	C	0.4892	7.13E-06	chr:4:120263688
4	rs4833612	120147596	A	0.4892	7.13E-06	chr:4:120263688
4	rs115717214	120674999	C	0.2708	7.36E-06	chr:4:120263688
4	rs115320472	120015556	G	0.3087	7.40E-06	chr:4:120263688
4	rs78380139	120015922	C	0.3087	7.40E-06	chr:4:120263688
4	rs1052633	120215314	G	0.5626	7.40E-06	chr:4:120263688
4	rs2175383	120215836	A	0.5626	7.40E-06	chr:4:120263688
4	rs62320716	120271922	A	2.47	7.54E-06	chr:4:120263688
4	rs10022508	120115228	G	0.4388	7.56E-06	chr:4:120263688
4	rs28768140	120623578	C	0.4951	7.80E-06	chr:4:120263688
4	rs11727963	120278100	A	2.424	8.09E-06	chr:4:120263688
4	rs72680925	120626727	G	0.3371	8.10E-06	chr:4:120263688
4	rs10001829	120665293	C	0.5518	8.47E-06	chr:4:120263688
4	rs11098498	120187119	C	0.5631	8.49E-06	chr:4:120263688
4	rs191937876	120383692	C	4.263	8.78E-06	chr:4:120263688
4	rs11098517	120318039	C	4.241	8.82E-06	chr:4:120263688
4	rs116371598	120354769	G	4.241	8.82E-06	chr:4:120263688
4	rs11932826	120382707	C	4.241	8.82E-06	chr:4:120263688
4	rs140706157	120323724	G	4.241	8.82E-06	chr:4:120263688
4	rs182185985	120367355	T	4.241	8.82E-06	chr:4:120263688
4	rs188943095	120364004	C	4.241	8.82E-06	chr:4:120263688
4	rs4092729	120358556	C	4.241	8.82E-06	chr:4:120263688
4	rs61269662	120324317	A	4.241	8.82E-06	chr:4:120263688
4	rs75192334	120424699	C	4.241	8.82E-06	chr:4:120263688
4	rs7697294	120306313	T	4.241	8.82E-06	chr:4:120263688
4	rs9759494	120368186	A	4.241	8.82E-06	chr:4:120263688
4	rs9759724	120368997	A	4.241	8.82E-06	chr:4:120263688
4	rs6817738	120417022	T	4.023	8.87E-06	chr:4:120263688
4	rs561829266	120338531	T	0.1751	8.91E-06	chr:4:120263688
4	rs13107334	120424580	G	0.3567	9.12E-06	chr:4:120263688
4	rs547446471	120381152	C	0.1623	9.18E-06	chr:4:120263688
4	rs113643839	120561944	G	0.3604	9.39E-06	chr:4:120263688
4	rs2252708	120612158	A	0.5326	9.50E-06	chr:4:120263688
4	rs6832670	120118792	G	0.4372	9.56E-06	chr:4:120263688
4	rs6851169	120117510	C	0.4372	9.56E-06	chr:4:120263688
4	rs57391877	120587108	C	0.4986	1.01E-05	chr:4:120263688

4	rs13119815	120662321	A	0.5444	1.01E-05	chr:4:120263688
4	rs111663206	120122939	C	1.949	1.04E-05	chr:4:120263688
4	rs17049975	120115461	C	1.949	1.04E-05	chr:4:120263688
4	rs17051345	120576634	G	0.4138	1.04E-05	chr:4:120263688
4	rs73842682	120567388	G	0.4138	1.04E-05	chr:4:120263688
4	rs73842683	120573143	A	0.4138	1.04E-05	chr:4:120263688
4	rs4348074	120302119	G	4.192	1.04E-05	chr:4:120263688
4	rs71614451	120404460	T	0.3571	1.04E-05	chr:4:120263688
4	rs72680934	120628956	A	0.4228	1.05E-05	chr:4:120263688
4	rs17292047	120465851	A	0.3187	1.09E-05	chr:4:120263688
4	rs72680941	120631878	C	0.4368	1.09E-05	chr:4:120263688
4	rs12502503	120116255	T	0.4439	1.30E-05	chr:4:120263688
4	rs12509054	120115075	C	0.4439	1.30E-05	chr:4:120263688
4	rs6822498	120120183	C	0.4439	1.30E-05	chr:4:120263688
4	rs6838674	120119376	G	0.4439	1.30E-05	chr:4:120263688
4	rs2902921	9591436	C	13.58	3.77E-34	chr:4:9603240
4	rs78121242	9591433	C	13.29	1.13E-33	chr:4:9603240
4	rs138499118	9631591	C	0.147	7.19E-33	chr:4:9603240
4	rs28851772	9630974	A	0.1496	1.15E-32	chr:4:9603240
4	rs56787100	9631153	T	0.1496	1.15E-32	chr:4:9603240
4	rs10031125	9625879	A	0.156	2.59E-32	chr:4:9603240
4	rs10049735	9626409	C	0.156	2.59E-32	chr:4:9603240
4	rs28648949	9626132	C	0.156	2.59E-32	chr:4:9603240
4	rs10005582	9626791	A	0.1671	2.84E-32	chr:4:9603240
4	rs28491253	9629512	T	0.1556	2.95E-32	chr:4:9603240
4	rs73082825	9627676	A	0.1561	3.64E-32	chr:4:9603240
4	rs115870066	9622163	A	0.1562	4.16E-32	chr:4:9603240
4	rs151134492	9623039	A	0.1561	5.37E-32	chr:4:9603240
4	rs7692046	9620128	G	0.1566	6.28E-32	chr:4:9603240
4	rs114980491	9623328	T	0.1566	6.61E-32	chr:4:9603240
4	rs147074482	9623653	T	0.1566	6.61E-32	chr:4:9603240
4	rs6856351	9590718	T	11.56	1.02E-30	chr:4:9603240
4	rs1842487	9597264	T	46.27	1.19E-30	chr:4:9603240
4	rs6448859	9596488	T	46.27	1.19E-30	chr:4:9603240
4	rs6837173	9590700	T	11.21	2.04E-30	chr:4:9603240
4	rs28784595	9646182	C	0.04457	3.43E-30	chr:4:9603240
4	rs9714994	9589698	T	11.02	8.83E-30	chr:4:9603240
4	rs6811235	9594627	A	59.76	1.56E-29	chr:4:9603240
4	rs2865075	9591384	A	10.35	7.31E-29	chr:4:9603240
4	rs10019130	9597356	G	10.32	7.78E-29	chr:4:9603240
4	rs13103207	9603672	T	9.85	1.14E-28	chr:4:9603240
4	rs1842488	9601268	A	9.85	1.14E-28	chr:4:9603240
4	rs2865079	9600388	A	9.85	1.16E-28	chr:4:9603240

4	rs6855316	9596183	G	9.989	1.20E-28	chr:4:9603240
4	rs11728108	9590027	T	10.06	1.22E-28	chr:4:9603240
4	rs10212854	9590489	A	10.07	1.27E-28	chr:4:9603240
4	rs9714725	9589901	C	8.497	9.19E-28	chr:4:9603240
4	rs13136903	9694522	T	6.66	2.13E-26	chr:4:9603240
4	rs7700164	9690605	C	7.86	2.20E-26	chr:4:9603240
4	rs10017364	9692924	T	7.803	3.91E-26	chr:4:9603240
4	rs13434918	9692356	A	7.612	6.68E-26	chr:4:9603240
4	rs6843107	9692300	A	7.612	6.68E-26	chr:4:9603240
4	rs6843499	9692462	A	7.612	6.68E-26	chr:4:9603240
4	rs35735545	9600181	A	0.01146	3.00E-24	chr:4:9603240
4	rs12645445	9599283	G	0.01153	3.38E-24	chr:4:9603240
4	rs11729986	9605023	A	7.619	7.35E-24	chr:4:9603240
4	rs111921266	9591246	A	0.1917	7.58E-23	chr:4:9603240
4	rs34968063	9599535	T	0.009451	1.54E-22	chr:4:9603240
4	rs28728462	9700392	G	4.969	1.88E-22	chr:4:9603240
4	rs150602585	9621799	G	0.1693	6.55E-22	chr:4:9603240
4	rs10020849	9702608	T	0.2198	4.08E-21	chr:4:9603240
4	rs6852419	9711314	G	4.316	1.50E-20	chr:4:9603240
4	rs12501341	9589915	A	0.2214	1.70E-20	chr:4:9603240
4	rs12649073	9694490	A	4.56	2.17E-20	chr:4:9603240
4	rs6858200	9709531	C	4.3	2.25E-20	chr:4:9603240
4	rs111613333	9592284	A	0.2109	2.27E-20	chr:4:9603240
4	rs61485794	9592346	G	0.2109	2.27E-20	chr:4:9603240
4	rs11723205	9718677	G	4.234	2.40E-20	chr:4:9603240
4	rs55912423	9715435	G	4.234	2.40E-20	chr:4:9603240
4	rs6836384	9713148	C	4.234	2.40E-20	chr:4:9603240
4	rs10939465	9702053	C	4.29	2.84E-20	chr:4:9603240
4	rs6448856	9595797	A	0.227	3.36E-20	chr:4:9603240
4	rs6448858	9595918	T	0.227	3.36E-20	chr:4:9603240
4	rs7672211	9597825	A	0.227	3.36E-20	chr:4:9603240
4	rs6858393	9709577	C	4.228	3.38E-20	chr:4:9603240
4	rs73097911	9601929	A	0.2146	4.97E-20	chr:4:9603240
4	rs74183920	9603786	A	0.2146	4.97E-20	chr:4:9603240
4	rs3890174	9700774	C	4.399	5.24E-20	chr:4:9603240
4	rs6820385	9606357	G	0.2315	6.60E-20	chr:4:9603240
4	rs7683574	9719148	C	4.162	6.76E-20	chr:4:9603240
4	rs60761027	9645910	C	6.729	6.97E-20	chr:4:9603240
4	rs9992908	9649931	T	6.729	6.97E-20	chr:4:9603240
4	rs576487641	9712952	C	4.127	9.47E-20	chr:4:9603240
4	rs10003639	9591535	C	8.284	1.01E-19	chr:4:9603240
4	rs6825142	9592142	G	8.282	1.01E-19	chr:4:9603240
4	rs141950396	9589589	G	8.254	1.08E-19	chr:4:9603240

4	rs12645339	9596615	T	8.227	1.17E-19	chr:4:9603240
4	rs73805539	9591699	C	0.2196	1.47E-19	chr:4:9603240
4	rs7664612	9595727	A	0.2316	1.62E-19	chr:4:9603240
4	rs28872608	9600754	T	8.123	1.73E-19	chr:4:9603240
4	rs10034929	9602625	C	7.689	1.78E-19	chr:4:9603240
4	rs9684176	9716386	G	4.658	1.91E-19	chr:4:9603240
4	rs61267391	9600955	G	0.2185	2.02E-19	chr:4:9603240
4	rs9291584	9588616	T	8.387	2.84E-19	chr:4:9603240
4	rs59430272	9601484	C	0.2211	2.99E-19	chr:4:9603240
4	rs112839156	9601686	A	0.2234	3.20E-19	chr:4:9603240
4	rs12509677	9602724	A	0.2234	3.20E-19	chr:4:9603240
4	rs59507413	9606018	T	0.2234	3.20E-19	chr:4:9603240
4	rs60296945	9604068	T	0.2234	3.20E-19	chr:4:9603240
4	rs60990443	9601319	T	0.2234	3.20E-19	chr:4:9603240
4	rs12510176	9599593	G	0.2247	4.04E-19	chr:4:9603240
4	rs9917965	9614520	A	0.2455	4.26E-19	chr:4:9603240
4	rs112018355	9611157	A	0.2251	5.74E-19	chr:4:9603240
4	rs11723945	9735299	A	0.2533	7.68E-19	chr:4:9603240
4	rs28652624	9671614	G	7.189	8.12E-19	chr:4:9603240
4	rs113890812	9677773	T	7.183	8.39E-19	chr:4:9603240
4	rs11944562	9674314	A	7.183	8.39E-19	chr:4:9603240
4	rs150445004	9669197	G	7.183	8.39E-19	chr:4:9603240
4	rs36110035	9667699	A	7.183	8.39E-19	chr:4:9603240
4	rs6819225	9668489	T	7.183	8.39E-19	chr:4:9603240
4	rs6848322	9668369	A	7.183	8.39E-19	chr:4:9603240
4	rs10032448	9673179	T	7.164	8.64E-19	chr:4:9603240
4	rs6819589	9670971	A	7.164	8.64E-19	chr:4:9603240
4	rs6851901	9672668	T	7.164	8.64E-19	chr:4:9603240
4	rs55737007	9678275	C	7.014	9.01E-19	chr:4:9603240
4	rs10007313	9611894	G	7.702	1.28E-18	chr:4:9603240
4	rs28531949	9612273	G	7.702	1.28E-18	chr:4:9603240
4	rs139639883	9668986	A	7.07	1.39E-18	chr:4:9603240
4	rs28773308	9681879	A	6.748	1.55E-18	chr:4:9603240
4	rs28496830	9678778	A	6.702	2.32E-18	chr:4:9603240
4	rs148730952	9647892	T	0.245	2.52E-18	chr:4:9603240
4	rs6831476	9708183	T	4.381	3.13E-18	chr:4:9603240
4	rs6821653	9658781	A	7.164	3.32E-18	chr:4:9603240
4	rs11943931	9706478	A	4.386	3.36E-18	chr:4:9603240
4	rs7671464	9689915	A	6.595	3.57E-18	chr:4:9603240
4	rs6813074	9664848	A	7.122	3.82E-18	chr:4:9603240
4	rs28498401	9685887	A	6.303	4.48E-18	chr:4:9603240
4	rs28630379	9684419	A	6.303	4.48E-18	chr:4:9603240
4	rs35847996	9685530	T	6.303	4.48E-18	chr:4:9603240

4	rs36119953	9685253	G	6.303	4.48E-18	chr:4:9603240
4	rs6836851	9661477	A	7.039	5.77E-18	chr:4:9603240
4	rs28571083	9660883	G	7.015	6.17E-18	chr:4:9603240
4	rs6834366	9658004	A	7.015	6.17E-18	chr:4:9603240
4	rs6448961	9686837	A	6.252	6.35E-18	chr:4:9603240
4	rs1871717	9594100	G	0.2498	8.22E-18	chr:4:9603240
4	rs1871718	9594106	A	0.2498	8.22E-18	chr:4:9603240
4	rs11937931	9613699	C	7.106	8.80E-18	chr:4:9603240
4	rs6835200	9658382	T	5.111	9.22E-18	chr:4:9603240
4	rs73097914	9602324	T	0.2497	9.55E-18	chr:4:9603240
4	rs6842974	9654882	T	6.766	9.65E-18	chr:4:9603240
4	rs11721978	9643139	G	6.609	1.07E-17	chr:4:9603240
4	rs6830692	9589873	T	0.2342	1.09E-17	chr:4:9603240
4	rs10939395	9595105	T	0.2514	1.10E-17	chr:4:9603240
4	rs10939406	9603769	T	0.2514	1.10E-17	chr:4:9603240
4	rs1809400	9599703	T	0.2514	1.10E-17	chr:4:9603240
4	rs1809401	9599790	T	0.2514	1.10E-17	chr:4:9603240
4	rs1842489	9601365	C	0.2514	1.10E-17	chr:4:9603240
4	rs1871720	9594372	A	0.2514	1.10E-17	chr:4:9603240
4	rs4404541	9600193	G	0.2514	1.10E-17	chr:4:9603240
4	rs4974820	9604794	G	0.2514	1.10E-17	chr:4:9603240
4	rs4974821	9602901	A	0.2514	1.10E-17	chr:4:9603240
4	rs6814674	9594719	T	0.2514	1.10E-17	chr:4:9603240
4	rs6815282	9595018	T	0.2514	1.10E-17	chr:4:9603240
4	rs6855527	9596313	C	0.2514	1.10E-17	chr:4:9603240
4	rs7672393	9597912	A	0.2514	1.10E-17	chr:4:9603240
4	rs9993066	9602631	T	0.2514	1.10E-17	chr:4:9603240
4	rs11727674	9719981	C	3.668	1.16E-17	chr:4:9603240
4	rs6812634	9639831	T	7.062	1.18E-17	chr:4:9603240
4	rs55948575	9648084	A	6.898	1.21E-17	chr:4:9603240
4	rs10032063	9650944	A	6.596	1.24E-17	chr:4:9603240
4	rs11945043	9646794	T	6.708	1.35E-17	chr:4:9603240
4	rs4974822	9602787	A	0.2535	1.44E-17	chr:4:9603240
4	rs4974862	9602780	T	0.2535	1.44E-17	chr:4:9603240
4	rs13131693	9593418	T	0.2537	1.47E-17	chr:4:9603240
4	rs28735278	9614378	A	0.2437	1.65E-17	chr:4:9603240
4	rs6839392	9613589	T	0.2437	1.65E-17	chr:4:9603240
4	rs11736694	9607834	C	0.2559	1.68E-17	chr:4:9603240
4	rs13122952	9707740	A	4.249	1.72E-17	chr:4:9603240
4	rs10005638	9653852	A	6.227	1.80E-17	chr:4:9603240
4	rs10029398	9650482	C	6.635	2.02E-17	chr:4:9603240
4	rs10035053	9649605	G	6.635	2.02E-17	chr:4:9603240
4	rs55935416	9648989	T	6.635	2.02E-17	chr:4:9603240

4	rs9992888	9649863	T	6.635	2.02E-17	chr:4:9603240
4	rs4974860	9604201	T	0.2609	2.04E-17	chr:4:9603240
4	rs111802886	9643840	T	6.618	2.05E-17	chr:4:9603240
4	rs10939411	9606906	T	0.256	2.15E-17	chr:4:9603240
4	rs10939413	9607827	T	0.256	2.15E-17	chr:4:9603240
4	rs2004334	9609566	A	0.256	2.15E-17	chr:4:9603240
4	rs4974857	9607133	T	0.256	2.15E-17	chr:4:9603240
4	rs6849083	9606355	A	0.256	2.15E-17	chr:4:9603240
4	rs6855150	9606433	T	0.256	2.15E-17	chr:4:9603240
4	rs6855313	9606475	T	0.256	2.15E-17	chr:4:9603240
4	rs6855323	9606482	T	0.256	2.15E-17	chr:4:9603240
4	rs11737872	9608680	C	0.2512	2.15E-17	chr:4:9603240
4	rs28680396	9646707	A	6.479	2.21E-17	chr:4:9603240
4	rs9291586	9605658	A	0.2546	2.36E-17	chr:4:9603240
4	rs4974855	9608931	T	0.2583	3.07E-17	chr:4:9603240
4	rs145566624	9642951	A	6.566	3.20E-17	chr:4:9603240
4	rs10007322	9611939	T	0.2594	3.85E-17	chr:4:9603240
4	rs28546389	9703880	T	0.2764	3.88E-17	chr:4:9603240
4	rs6842111	9612572	A	0.2598	3.89E-17	chr:4:9603240
4	rs6448969	9700056	A	4.176	4.65E-17	chr:4:9603240
4	rs734628	9610085	G	0.2667	6.95E-17	chr:4:9603240
4	rs13141772	9575916	T	4.899	7.66E-17	chr:4:9603240
4	rs61199897	9657621	T	0.2764	1.31E-16	chr:4:9603240
4	rs4974814	9614162	T	0.2702	1.31E-16	chr:4:9603240
4	rs73095794	9596566	A	0.2657	1.37E-16	chr:4:9603240
4	rs61118685	9650877	A	0.2612	1.38E-16	chr:4:9603240
4	rs12506692	9596059	T	0.2677	1.40E-16	chr:4:9603240
4	rs6835175	9658351	T	5.113	1.88E-16	chr:4:9603240
4	rs56934553	9594735	A	0.2699	1.94E-16	chr:4:9603240
4	rs7691087	9595443	T	0.2699	1.94E-16	chr:4:9603240
4	rs111288527	9651346	G	0.2645	2.10E-16	chr:4:9603240
4	rs6831341	9592225	T	0.2616	3.35E-16	chr:4:9603240
4	rs111656532	9632606	G	2.682	3.42E-16	chr:4:9603240
4	rs13152842	9588960	T	0.26	3.49E-16	chr:4:9603240
4	rs58688978	9693903	A	0.2687	3.65E-16	chr:4:9603240
4	rs73802959	9694487	A	0.2687	3.65E-16	chr:4:9603240
4	rs57685354	9655782	T	0.265	3.71E-16	chr:4:9603240
4	rs145014366	9588718	C	0.2716	3.93E-16	chr:4:9603240
4	rs151311132	9646020	C	0.2677	4.43E-16	chr:4:9603240
4	rs113133387	9642181	T	0.2809	4.65E-16	chr:4:9603240
4	rs10939407	9604018	G	0.2623	4.79E-16	chr:4:9603240
4	rs1967000	9592111	G	0.2623	4.79E-16	chr:4:9603240
4	rs2086430	9598327	C	0.2623	4.79E-16	chr:4:9603240

4	rs2101074	9598414	A	0.2623	4.79E-16	chr:4:9603240
4	rs6448855	9595781	G	0.2623	4.79E-16	chr:4:9603240
4	rs6853345	9589192	A	0.2623	4.79E-16	chr:4:9603240
4	rs12512543	9608445	A	0.2761	4.86E-16	chr:4:9603240
4	rs6855818	9635008	A	4.115	5.48E-16	chr:4:9603240
4	rs149050104	9661523	T	0.2683	5.67E-16	chr:4:9603240
4	rs35352906	9670773	T	0.2683	5.67E-16	chr:4:9603240
4	rs4259062	9674245	T	0.2683	5.67E-16	chr:4:9603240
4	rs4417968	9667070	T	0.2683	5.67E-16	chr:4:9603240
4	rs4446308	9678379	A	0.2683	5.67E-16	chr:4:9603240
4	rs6822895	9681222	T	0.2683	5.67E-16	chr:4:9603240
4	rs6858190	9679430	G	0.2683	5.67E-16	chr:4:9603240
4	rs79367718	9673458	C	0.2683	5.67E-16	chr:4:9603240
4	rs61165916	9655464	A	0.2685	5.78E-16	chr:4:9603240
4	rs74821633	9690069	C	0.2712	6.64E-16	chr:4:9603240
4	rs10012992	9594197	A	4.644	8.00E-16	chr:4:9603240
4	rs10000206	9611952	C	0.2668	9.24E-16	chr:4:9603240
4	rs10939410	9606851	T	0.2668	9.24E-16	chr:4:9603240
4	rs2004333	9609411	T	0.2668	9.24E-16	chr:4:9603240
4	rs9917942	9612378	T	0.2668	9.24E-16	chr:4:9603240
4	rs28762742	9646007	G	3.972	1.07E-15	chr:4:9603240
4	rs112013141	9611339	G	5.288	1.24E-15	chr:4:9603240
4	rs9917943	9612413	C	5.288	1.24E-15	chr:4:9603240
4	rs58786763	9649755	A	0.2747	1.77E-15	chr:4:9603240
4	rs146531882	9653818	T	0.2777	2.23E-15	chr:4:9603240
4	rs13141264	9698240	A	4.082	2.57E-15	chr:4:9603240
4	rs60568234	9648674	T	0.2879	2.61E-15	chr:4:9603240
4	rs6824573	9589256	A	0.293	2.62E-15	chr:4:9603240
4	rs11730320	9719901	C	0.3204	2.75E-15	chr:4:9603240
4	rs4974817	9613000	A	0.2844	3.01E-15	chr:4:9603240
4	rs28886660	9644540	G	0.2885	3.70E-15	chr:4:9603240
4	rs58724508	9642207	T	3.831	3.94E-15	chr:4:9603240
4	rs6828382	9634594	C	3.819	4.30E-15	chr:4:9603240
4	rs28627624	9637607	C	3.795	4.89E-15	chr:4:9603240
4	rs9917891	9614633	C	5.147	5.32E-15	chr:4:9603240
4	rs9917966	9614537	G	5.147	5.32E-15	chr:4:9603240
4	rs4974852	9612877	C	0.2797	5.63E-15	chr:4:9603240
4	rs77118731	9633558	G	0.2823	6.94E-15	chr:4:9603240
4	rs2902922	9609380	G	5.089	7.31E-15	chr:4:9603240
4	rs143992647	9639055	T	0.2833	7.50E-15	chr:4:9603240
4	rs4974813	9614492	T	0.2833	7.50E-15	chr:4:9603240
4	rs142877628	9670746	T	0.2905	7.65E-15	chr:4:9603240
4	rs10032442	9578122	C	0.2883	8.71E-15	chr:4:9603240

4	rs13105133	9577464	T	0.2883	8.71E-15	chr:4:9603240
4	rs13120121	9577601	G	0.2883	8.71E-15	chr:4:9603240
4	rs13124988	9582385	C	0.2883	8.71E-15	chr:4:9603240
4	rs13135278	9582422	A	0.2883	8.71E-15	chr:4:9603240
4	rs13141294	9582314	T	0.2883	8.71E-15	chr:4:9603240
4	rs7439868	9575415	A	0.2883	8.71E-15	chr:4:9603240
4	rs7439897	9575506	A	0.2883	8.71E-15	chr:4:9603240
4	rs7683563	9574835	A	0.2883	8.71E-15	chr:4:9603240
4	rs9683873	9585416	A	0.2883	8.71E-15	chr:4:9603240
4	rs6827717	9695525	T	0.304	1.00E-14	chr:4:9603240
4	rs143900004	9636160	T	0.2936	1.02E-14	chr:4:9603240
4	rs142797284	9634436	T	0.2872	1.06E-14	chr:4:9603240
4	rs12648085	9572407	G	0.2883	1.07E-14	chr:4:9603240
4	rs35722426	9570769	C	0.2883	1.07E-14	chr:4:9603240
4	rs57504588	9570006	A	0.2883	1.07E-14	chr:4:9603240
4	rs72491232	9658479	G	0.2857	1.16E-14	chr:4:9603240
4	rs2046112	9584367	T	0.2911	1.27E-14	chr:4:9603240
4	rs150196562	9642725	G	0.2875	1.35E-14	chr:4:9603240
4	rs113258723	9643382	C	0.2865	1.80E-14	chr:4:9603240
4	rs73084845	9671025	T	0.2984	1.84E-14	chr:4:9603240
4	rs12505906	9636906	A	0.2984	1.85E-14	chr:4:9603240
4	rs73082901	9636762	C	0.2984	1.85E-14	chr:4:9603240
4	rs76266909	9636303	C	0.2984	1.85E-14	chr:4:9603240
4	rs57610759	9642093	G	3.577	2.45E-14	chr:4:9603240
4	rs10014826	9635408	C	0.3006	2.47E-14	chr:4:9603240
4	rs150921853	9639169	T	0.3006	2.47E-14	chr:4:9603240
4	rs137952419	9613766	G	5.022	2.53E-14	chr:4:9603240
4	rs10005931	9714347	C	3.25	2.73E-14	chr:4:9603240
4	rs28575773	9656525	A	0.3042	3.35E-14	chr:4:9603240
4	rs12501304	9682548	T	0.3028	3.44E-14	chr:4:9603240
4	rs73084842	9671015	T	0.3028	3.44E-14	chr:4:9603240
4	rs201078836	9680043	T	0.3036	3.76E-14	chr:4:9603240
4	rs182962990	9642562	T	0.2856	3.93E-14	chr:4:9603240
4	rs139020598	9656876	T	0.2949	3.93E-14	chr:4:9603240
4	rs57868742	9694076	G	0.2994	4.43E-14	chr:4:9603240
4	rs73802957	9694235	T	0.2994	4.43E-14	chr:4:9603240
4	rs73802958	9694236	T	0.2994	4.43E-14	chr:4:9603240
4	rs10026706	9674011	A	0.305	4.58E-14	chr:4:9603240
4	rs12171434	9647060	T	0.305	4.58E-14	chr:4:9603240
4	rs142277140	9648076	C	0.305	4.58E-14	chr:4:9603240
4	rs143079510	9665042	T	0.305	4.58E-14	chr:4:9603240
4	rs143459149	9674515	G	0.305	4.58E-14	chr:4:9603240
4	rs150693830	9665588	T	0.305	4.58E-14	chr:4:9603240

4	rs28380563	9658392	T	0.305	4.58E-14	chr:4:9603240
4	rs28577902	9650657	A	0.305	4.58E-14	chr:4:9603240
4	rs28643508	9659221	C	0.305	4.58E-14	chr:4:9603240
4	rs4518243	9664634	C	0.305	4.58E-14	chr:4:9603240
4	rs59312718	9669434	T	0.305	4.58E-14	chr:4:9603240
4	rs73084818	9648875	T	0.305	4.58E-14	chr:4:9603240
4	rs7684741	9661843	A	0.305	4.58E-14	chr:4:9603240
4	rs7693691	9652934	C	0.305	4.58E-14	chr:4:9603240
4	rs77025480	9657754	A	0.305	4.58E-14	chr:4:9603240
4	rs73084810	9642699	G	0.3052	4.62E-14	chr:4:9603240
4	rs74532861	9656067	C	0.2956	4.67E-14	chr:4:9603240
4	rs75965288	9656832	G	0.2956	4.67E-14	chr:4:9603240
4	rs7679526	9653286	T	0.2956	4.67E-14	chr:4:9603240
4	rs111953893	9645341	T	3.636	4.78E-14	chr:4:9603240
4	rs28546201	9684768	T	0.3061	5.06E-14	chr:4:9603240
4	rs28691200	9684552	A	0.3061	5.06E-14	chr:4:9603240
4	rs57478100	9652472	A	0.2878	5.49E-14	chr:4:9603240
4	rs141992178	9645431	A	3.579	6.85E-14	chr:4:9603240
4	rs111322624	9682780	T	0.2993	7.04E-14	chr:4:9603240
4	rs112611007	9667420	A	0.2993	7.04E-14	chr:4:9603240
4	rs35199477	9674995	A	0.2993	7.04E-14	chr:4:9603240
4	rs368426688	9667600	T	0.2993	7.04E-14	chr:4:9603240
4	rs4260536	9678079	G	0.2993	7.04E-14	chr:4:9603240
4	rs4264827	9664677	A	0.2993	7.04E-14	chr:4:9603240
4	rs4362821	9668941	T	0.2993	7.04E-14	chr:4:9603240
4	rs4550924	9674437	T	0.2993	7.04E-14	chr:4:9603240
4	rs6822851	9681151	A	0.2993	7.04E-14	chr:4:9603240
4	rs6838107	9679002	C	0.2993	7.04E-14	chr:4:9603240
4	rs75729554	9676179	T	0.2993	7.04E-14	chr:4:9603240
4	rs28718562	9659961	G	0.3067	7.20E-14	chr:4:9603240
4	rs9684206	9585751	T	0.3203	8.66E-14	chr:4:9603240
4	rs9996676	9678661	C	0.3109	9.21E-14	chr:4:9603240
4	rs150730953	9697831	C	0.3027	9.28E-14	chr:4:9603240
4	rs73802943	9663020	C	0.3075	1.03E-13	chr:4:9603240
4	rs28851574	9644580	C	0.3121	1.06E-13	chr:4:9603240
4	rs145156790	9671365	A	0.2994	1.06E-13	chr:4:9603240
4	rs10001941	9688516	A	0.3126	1.10E-13	chr:4:9603240
4	rs76078762	9656034	A	0.3031	1.15E-13	chr:4:9603240
4	rs79487290	9663694	A	0.3034	1.20E-13	chr:4:9603240
4	rs73088893	9695306	A	0.3148	1.44E-13	chr:4:9603240
4	rs116345678	9601756	G	5.209	1.76E-13	chr:4:9603240
4	rs58673888	9601558	T	5.199	1.80E-13	chr:4:9603240
4	rs116485634	9753365	T	0.3652	1.86E-13	chr:4:9603240

4	rs10024971	9694013	G	0.3178	2.05E-13	chr:4:9603240
4	rs6812913	9757848	T	0.3609	2.08E-13	chr:4:9603240
4	rs57553236	9688140	C	0.3082	2.17E-13	chr:4:9603240
4	rs6836522	9686823	T	0.3082	2.17E-13	chr:4:9603240
4	rs201440390	9697882	A	0.3111	2.27E-13	chr:4:9603240
4	rs12504246	9692932	A	0.321	2.44E-13	chr:4:9603240
4	rs57402803	9691353	T	0.3148	2.98E-13	chr:4:9603240
4	rs16898588	9595751	T	0.2143	3.25E-13	chr:4:9603240
4	rs9995735	9658789	T	0.3254	3.30E-13	chr:4:9603240
4	rs10006684	9694706	T	5.014	3.71E-13	chr:4:9603240
4	rs28765135	9644325	A	3.653	4.01E-13	chr:4:9603240
4	rs138647140	9621141	A	3.272	4.20E-13	chr:4:9603240
4	rs6816115	9585104	C	0.305	4.29E-13	chr:4:9603240
4	rs10012404	9621365	T	3.275	4.63E-13	chr:4:9603240
4	rs186527246	9621870	A	3.275	4.63E-13	chr:4:9603240
4	rs58820969	9607071	A	4.958	4.83E-13	chr:4:9603240
4	rs28783176	9645220	G	3.649	4.86E-13	chr:4:9603240
4	rs28866958	9644586	G	3.649	4.86E-13	chr:4:9603240
4	rs28624152	9626792	T	2.919	4.91E-13	chr:4:9603240
4	rs113905817	9626025	G	3.276	5.06E-13	chr:4:9603240
4	rs10013863	9667436	T	0.3286	5.74E-13	chr:4:9603240
4	rs9884683	9677137	A	0.3286	5.74E-13	chr:4:9603240
4	rs56264714	9624087	A	3.277	5.76E-13	chr:4:9603240
4	rs144989810	9612047	T	4.923	6.31E-13	chr:4:9603240
4	rs150289378	9753368	G	0.373	6.58E-13	chr:4:9603240
4	rs73802916	9604035	C	0.2177	6.68E-13	chr:4:9603240
4	rs113883509	9620223	A	3.214	6.92E-13	chr:4:9603240
4	rs149583330	9662001	T	0.3007	6.93E-13	chr:4:9603240
4	rs28840705	9631170	A	3.211	7.11E-13	chr:4:9603240
4	rs11729255	9744933	A	0.3664	8.01E-13	chr:4:9603240
4	rs11734448	9563381	C	4.286	8.29E-13	chr:4:9603240
4	rs10012148	9664294	A	0.3321	8.39E-13	chr:4:9603240
4	rs28796640	9663788	T	0.3321	8.39E-13	chr:4:9603240
4	rs115914537	9753369	C	0.3753	9.72E-13	chr:4:9603240
4	rs12503033	9732370	C	0.3689	1.32E-12	chr:4:9603240
4	rs6448846	9580937	G	0.3639	1.42E-12	chr:4:9603240
4	rs139901069	9582722	A	0.2303	1.53E-12	chr:4:9603240
4	rs77648749	9583407	A	0.2303	1.53E-12	chr:4:9603240
4	rs3864192	9573978	A	0.3626	1.76E-12	chr:4:9603240
4	rs7666771	9739268	T	0.371	1.89E-12	chr:4:9603240
4	rs10011678	9688689	C	0.3399	1.95E-12	chr:4:9603240
4	rs10021058	9733051	T	0.374	2.02E-12	chr:4:9603240
4	rs28711515	9731651	G	0.374	2.02E-12	chr:4:9603240

4	rs55768848	9740885	A	0.3747	2.27E-12	chr:4:9603240
4	rs11723541	9556165	T	4.075	2.46E-12	chr:4:9603240
4	rs11728441	9558041	C	4.075	2.46E-12	chr:4:9603240
4	rs12233678	9549951	C	4.075	2.46E-12	chr:4:9603240
4	rs12233771	9552965	A	4.075	2.46E-12	chr:4:9603240
4	rs12233884	9550045	A	4.075	2.46E-12	chr:4:9603240
4	rs80077135	9681299	G	0.2357	3.06E-12	chr:4:9603240
4	rs28673739	9677529	T	0.234	3.19E-12	chr:4:9603240
4	rs9291600	9675669	A	0.234	3.19E-12	chr:4:9603240
4	rs12233677	9549872	T	4.034	3.46E-12	chr:4:9603240
4	rs13152653	9556775	A	4.034	3.46E-12	chr:4:9603240
4	rs28793127	9660482	T	0.3539	3.51E-12	chr:4:9603240
4	rs7442338	9560945	T	3.967	3.73E-12	chr:4:9603240
4	rs10003452	9736893	A	0.3765	3.98E-12	chr:4:9603240
4	rs13107393	9562217	T	3.969	4.16E-12	chr:4:9603240
4	rs13116764	9561759	G	3.969	4.16E-12	chr:4:9603240
4	rs9715222	9564999	C	3.969	4.16E-12	chr:4:9603240
4	rs2030288	9751331	C	0.387	5.39E-12	chr:4:9603240
4	rs28695459	9750721	T	0.387	5.39E-12	chr:4:9603240
4	rs13152465	9556695	A	3.927	5.89E-12	chr:4:9603240
4	rs12508809	9748034	A	0.3936	6.30E-12	chr:4:9603240
4	rs12645989	9722133	T	0.3838	6.36E-12	chr:4:9603240
4	rs12233701	9552436	T	3.764	7.32E-12	chr:4:9603240
4	rs2176642	9752894	G	0.3917	7.71E-12	chr:4:9603240
4	rs6840249	9578971	G	0.3815	9.17E-12	chr:4:9603240
4	rs13136075	9714968	A	3.299	1.21E-11	chr:4:9603240
4	rs13151395	9709144	T	3.311	1.41E-11	chr:4:9603240
4	rs66534644	9711634	G	3.264	1.75E-11	chr:4:9603240
4	rs12510091	9708661	A	3.276	1.99E-11	chr:4:9603240
4	rs6843276	9714147	T	3.232	2.04E-11	chr:4:9603240
4	rs6813395	9758132	A	0.4095	2.37E-11	chr:4:9603240
4	rs12500632	9554039	A	0.3496	2.51E-11	chr:4:9603240
4	rs116386123	9593389	A	4.487	2.59E-11	chr:4:9603240
4	rs11722945	9758822	A	0.4145	3.82E-11	chr:4:9603240
4	rs34611252	9743467	T	0.4099	4.08E-11	chr:4:9603240
4	rs3900665	9756491	T	0.4131	4.12E-11	chr:4:9603240
4	rs28479165	9731689	T	0.4088	4.27E-11	chr:4:9603240
4	rs73802936	9629733	T	0.2248	5.89E-11	chr:4:9603240
4	rs11733200	9743037	C	0.4132	6.05E-11	chr:4:9603240
4	rs7667340	9739575	T	0.4132	6.05E-11	chr:4:9603240
4	rs10033951	9779580	T	0.4183	6.71E-11	chr:4:9603240
4	rs75086253	9588904	C	6.503	6.87E-11	chr:4:9603240
4	rs141814667	9634087	A	4.314	7.11E-11	chr:4:9603240

4	rs56044722	9717939	A	0.3744	7.84E-11	chr:4:9603240
4	rs35454046	9545545	A	0.3611	8.04E-11	chr:4:9603240
4	rs116422411	9632691	T	3.094	8.97E-11	chr:4:9603240
4	rs55978512	9594743	A	6.388	1.01E-10	chr:4:9603240
4	rs6448836	9577290	G	0.4236	1.18E-10	chr:4:9603240
4	rs113638151	9625676	T	3.182	1.49E-10	chr:4:9603240
4	rs139524483	9624221	G	3.182	1.49E-10	chr:4:9603240
4	rs183790214	9624079	A	3.182	1.49E-10	chr:4:9603240
4	rs188557791	9624282	A	3.182	1.49E-10	chr:4:9603240
4	rs189357301	9625543	T	3.182	1.49E-10	chr:4:9603240
4	rs73082817	9626178	A	3.182	1.49E-10	chr:4:9603240
4	rs73082823	9627543	A	3.182	1.49E-10	chr:4:9603240
4	rs73082831	9629850	A	3.182	1.49E-10	chr:4:9603240
4	rs13137196	9715456	A	3.946	1.69E-10	chr:4:9603240
4	rs10032846	9602708	G	5.682	1.76E-10	chr:4:9603240
4	rs34972086	9603493	T	5.682	1.76E-10	chr:4:9603240
4	rs112271793	9631492	G	2.177	1.96E-10	chr:4:9603240
4	rs12509448	9577884	T	3.339	2.24E-10	chr:4:9603240
4	rs111476545	9593148	G	5.627	2.26E-10	chr:4:9603240
4	rs12508394	9702186	T	3.94	2.36E-10	chr:4:9603240
4	rs111705356	9675394	C	5.622	2.87E-10	chr:4:9603240
4	rs12649271	9674750	G	5.622	2.87E-10	chr:4:9603240
4	rs56001555	9675049	T	5.622	2.87E-10	chr:4:9603240
4	rs61213314	9541933	G	0.3778	3.27E-10	chr:4:9603240
4	rs10027164	9574763	A	0.4073	3.40E-10	chr:4:9603240
4	rs28475805	9628806	G	2.146	3.45E-10	chr:4:9603240
4	rs1842497	9579673	G	3.258	3.53E-10	chr:4:9603240
4	rs149685592	9624404	G	3.03	3.77E-10	chr:4:9603240
4	rs9684707	9569057	C	3.977	3.85E-10	chr:4:9603240
4	rs142093617	9679891	T	5.554	3.87E-10	chr:4:9603240
4	rs144106725	9689812	G	5.554	3.87E-10	chr:4:9603240
4	rs6847920	9668143	T	5.554	3.87E-10	chr:4:9603240
4	rs7699789	9679534	T	5.554	3.87E-10	chr:4:9603240
4	rs10939476	9730105	G	0.3763	4.05E-10	chr:4:9603240
4	rs113032361	9654782	G	5.522	4.19E-10	chr:4:9603240
4	rs12639876	9648354	T	5.522	4.19E-10	chr:4:9603240
4	rs13115550	9651413	T	5.522	4.19E-10	chr:4:9603240
4	rs148454816	9654199	T	5.522	4.19E-10	chr:4:9603240
4	rs35050866	9654001	A	5.522	4.19E-10	chr:4:9603240
4	rs73221507	9661031	G	5.522	4.19E-10	chr:4:9603240
4	rs12640950	9674332	G	5.29	4.83E-10	chr:4:9603240
4	rs58667761	9524632	C	0.3873	5.57E-10	chr:4:9603240
4	rs9761072	9530984	T	0.3873	5.57E-10	chr:4:9603240

4	rs1871719	9594339	T	5.788	5.82E-10	chr:4:9603240
4	rs112846020	9639531	T	5.434	6.00E-10	chr:4:9603240
4	rs113007587	9637175	G	5.434	6.00E-10	chr:4:9603240
4	rs141954958	9636492	A	5.434	6.00E-10	chr:4:9603240
4	rs11735096	9747398	T	0.4411	6.73E-10	chr:4:9603240
4	rs34056993	9514776	T	0.3924	7.03E-10	chr:4:9603240
4	rs58522119	9525010	T	0.3885	7.40E-10	chr:4:9603240
4	rs112219402	9562271	G	0.3778	7.51E-10	chr:4:9603240
4	rs6448882	9621131	A	2.732	8.46E-10	chr:4:9603240
4	rs532514174	9632907	A	0.1367	8.60E-10	chr:4:9603240
4	rs564924473	9632906	A	0.1367	8.60E-10	chr:4:9603240
4	rs59229779	9750259	C	0.4439	9.43E-10	chr:4:9603240
4	rs28846731	9623693	G	2.085	1.29E-09	chr:4:9603240
4	rs7658772	9512477	A	0.4004	1.35E-09	chr:4:9603240
4	rs28578840	9629427	C	2.077	1.41E-09	chr:4:9603240
4	rs4698055	9627821	T	2.064	1.74E-09	chr:4:9603240
4	rs58768205	9511390	A	0.4029	1.75E-09	chr:4:9603240
4	rs181796209	9621911	A	4.901	1.84E-09	chr:4:9603240
4	rs10939425	9627380	G	2.052	2.21E-09	chr:4:9603240
4	rs6846234	9577203	C	0.4422	2.42E-09	chr:4:9603240
4	rs56306649	9510873	C	0.4092	2.54E-09	chr:4:9603240
4	rs56340722	9510913	G	0.4092	2.54E-09	chr:4:9603240
4	rs12505958	9560131	G	3.487	2.64E-09	chr:4:9603240
4	rs34004016	9697965	C	0.3359	2.79E-09	chr:4:9603240
4	rs9998193	9760106	A	0.4617	2.80E-09	chr:4:9603240
4	rs149036428	9619440	A	0.3261	3.29E-09	chr:4:9603240
4	rs375351318	9619439	C	0.3261	3.29E-09	chr:4:9603240
4	rs10939437	9647252	G	3.529	3.89E-09	chr:4:9603240
4	rs9994111	9558344	G	2.224	4.19E-09	chr:4:9603240
4	rs140186320	9595829	C	6.078	4.39E-09	chr:4:9603240
4	rs148856374	9604143	T	6.078	4.39E-09	chr:4:9603240
4	rs57631263	9601537	A	6.078	4.39E-09	chr:4:9603240
4	rs56202639	9725168	C	0.4648	5.30E-09	chr:4:9603240
4	rs113444477	9609487	A	6.015	5.50E-09	chr:4:9603240
4	rs61319849	9607459	A	6.015	5.50E-09	chr:4:9603240
4	rs4974827	9588288	C	0.4281	5.56E-09	chr:4:9603240
4	rs57867579	9725344	A	0.4649	5.60E-09	chr:4:9603240
4	rs13141297	9602103	T	5.715	5.65E-09	chr:4:9603240
4	rs111750522	9597976	A	6.004	5.71E-09	chr:4:9603240
4	rs11724606	9720444	A	0.276	6.08E-09	chr:4:9603240
4	rs11732596	9569361	T	2.935	6.97E-09	chr:4:9603240
4	rs28785196	9623514	G	1.996	7.73E-09	chr:4:9603240
4	rs28866204	9623513	G	1.996	7.73E-09	chr:4:9603240

4	rs61031859	9684806	T	0.3259	7.93E-09	chr:4:9603240
4	rs11734804	9734103	C	0.2818	8.58E-09	chr:4:9603240
4	rs4077928	9698985	C	2.467	1.11E-08	chr:4:9603240
4	rs12233883	9549927	C	3.358	1.37E-08	chr:4:9603240
4	rs7683831	9845079	T	2.944	1.38E-08	chr:4:9603240
4	rs2037314	9724578	T	0.4756	1.44E-08	chr:4:9603240
4	rs13110253	9558556	T	3.341	1.47E-08	chr:4:9603240
4	rs4613568	9862902	T	2.931	1.53E-08	chr:4:9603240
4	rs6852425	9856859	G	2.931	1.53E-08	chr:4:9603240
4	rs73802973	9722401	A	0.2954	1.54E-08	chr:4:9603240
4	rs189438022	9660440	G	3.728	1.55E-08	chr:4:9603240
4	rs1842490	9568457	T	3.337	1.63E-08	chr:4:9603240
4	rs13132153	9561479	A	3.317	1.82E-08	chr:4:9603240
4	rs13138276	9562004	A	3.317	1.82E-08	chr:4:9603240
4	rs1880831	9568145	T	3.317	1.82E-08	chr:4:9603240
4	rs1980219	9850621	T	2.911	1.90E-08	chr:4:9603240
4	rs6812007	9850220	C	2.911	1.90E-08	chr:4:9603240
4	rs6828040	9850480	T	2.911	1.90E-08	chr:4:9603240
4	rs6847858	9853059	T	2.911	1.90E-08	chr:4:9603240
4	rs6847871	9853074	T	2.911	1.90E-08	chr:4:9603240
4	rs10939561	9851542	T	2.894	2.16E-08	chr:4:9603240
4	rs60403942	9603452	C	0.2362	2.16E-08	chr:4:9603240
4	rs6448880	9621023	T	2.169	2.35E-08	chr:4:9603240
4	rs28591077	9863678	A	2.865	2.47E-08	chr:4:9603240
4	rs9684729	9862036	C	2.865	2.47E-08	chr:4:9603240
4	rs10939428	9630041	A	2.8	2.48E-08	chr:4:9603240
4	rs552244216	9633025	T	0.2649	2.70E-08	chr:4:9603240
4	rs10006208	9621927	G	2.152	2.75E-08	chr:4:9603240
4	rs10019518	9621923	A	2.152	2.75E-08	chr:4:9603240
4	rs6831137	9633547	C	0.2468	2.84E-08	chr:4:9603240
4	rs114237113	9547812	A	0.3007	3.22E-08	chr:4:9603240
4	rs13120116	9694454	T	0.4841	3.32E-08	chr:4:9603240
4	rs113251875	9621763	T	1.961	3.32E-08	chr:4:9603240
4	rs59551372	9718895	T	0.2488	3.36E-08	chr:4:9603240
4	rs4102942	9568478	T	3.239	3.56E-08	chr:4:9603240
4	rs192886753	9622824	A	4.381	3.62E-08	chr:4:9603240
4	rs4697691	9858485	A	2.843	3.67E-08	chr:4:9603240
4	rs4697900	9859976	A	2.843	3.67E-08	chr:4:9603240
4	rs190746279	9632929	G	0.1861	3.78E-08	chr:4:9603240
4	rs11726722	9637477	G	3.16	4.06E-08	chr:4:9603240
4	rs149367087	9609347	A	7.211	4.26E-08	chr:4:9603240
4	rs55876301	9608833	A	0.2554	4.76E-08	chr:4:9603240
4	rs4504249	9704927	T	0.2566	4.92E-08	chr:4:9603240

4	rs61045106	9702341	G	0.2566	4.92E-08	chr:4:9603240
4	rs7673959	9620145	G	2.109	4.95E-08	chr:4:9603240
4	rs138681522	9619447	A	0.2832	5.36E-08	chr:4:9603240
4	rs145781005	9619446	C	0.2832	5.36E-08	chr:4:9603240
4	rs147877123	9721552	G	0.2585	5.77E-08	chr:4:9603240
4	rs6448996	9791254	T	0.4934	5.82E-08	chr:4:9603240
4	rs73805506	9548880	T	0.3046	5.84E-08	chr:4:9603240
4	rs4622998	9621659	C	2.087	6.23E-08	chr:4:9603240
4	rs7686230	9657345	T	3.345	6.39E-08	chr:4:9603240
4	rs9685427	9793380	C	0.4963	6.78E-08	chr:4:9603240
4	rs9761429	9530799	A	2.041	6.93E-08	chr:4:9603240
4	rs73082809	9623322	A	3.73	7.06E-08	chr:4:9603240
4	rs12510906	9509153	T	0.4325	7.27E-08	chr:4:9603240
4	rs142793639	9594543	T	0.3178	7.87E-08	chr:4:9603240
4	rs55892720	9585091	G	0.2648	7.94E-08	chr:4:9603240
4	rs112905485	9621041	T	3.694	8.47E-08	chr:4:9603240
4	rs141186559	9632118	G	3.694	8.47E-08	chr:4:9603240
4	rs144061159	9622297	T	3.694	8.47E-08	chr:4:9603240
4	rs149842121	9622099	T	3.694	8.47E-08	chr:4:9603240
4	rs190262728	9624191	T	3.694	8.47E-08	chr:4:9603240
4	rs57999276	9630949	T	3.694	8.47E-08	chr:4:9603240
4	rs6833119	9520138	C	0.3097	9.07E-08	chr:4:9603240
4	rs57304521	9753374	C	0.3432	9.63E-08	chr:4:9603240
4	rs9291612	9735891	T	0.42	1.06E-07	chr:4:9603240
4	rs6815602	9592093	T	4.808	1.11E-07	chr:4:9603240
4	rs73097919	9603468	G	7.536	1.22E-07	chr:4:9603240
4	rs6856401	9664027	G	3.255	1.26E-07	chr:4:9603240
4	rs139582668	9671180	T	5.755	1.34E-07	chr:4:9603240
4	rs61216331	9605453	A	7.433	1.50E-07	chr:4:9603240
4	rs141703666	9510151	G	0.4451	1.58E-07	chr:4:9603240
4	rs78329286	9538878	A	0.3198	1.58E-07	chr:4:9603240
4	rs62409886	9642713	T	3.514	1.60E-07	chr:4:9603240
4	rs111965781	9595298	C	5.675	1.61E-07	chr:4:9603240
4	rs57995767	9510711	A	0.4457	1.62E-07	chr:4:9603240
4	rs149045417	9596105	T	0.3392	1.83E-07	chr:4:9603240
4	rs181297539	9652092	T	5.636	1.87E-07	chr:4:9603240
4	rs10939509	9772309	A	0.5125	1.91E-07	chr:4:9603240
4	rs13150941	9694401	A	0.5137	2.16E-07	chr:4:9603240
4	rs2004245	9532422	T	0.3284	2.20E-07	chr:4:9603240
4	rs11941979	9664162	T	5.598	2.20E-07	chr:4:9603240
4	rs149010044	9593436	A	5.231	2.33E-07	chr:4:9603240
4	rs58434858	9501245	A	0.3084	2.42E-07	chr:4:9603240
4	rs28450736	9410035	T	4.903	2.51E-07	chr:4:9603240

4	rs7655090	9765875	G	0.5171	2.55E-07	chr:4:9603240
4	rs2001392	9533092	C	0.3406	2.61E-07	chr:4:9603240
4	rs34172197	9634195	A	3.384	2.63E-07	chr:4:9603240
4	rs13123739	9604667	T	5.619	2.65E-07	chr:4:9603240
4	rs149920424	9700202	T	6.431	2.72E-07	chr:4:9603240
4	rs141319276	9622203	C	2.705	2.88E-07	chr:4:9603240
4	rs150327213	9624677	G	2.705	2.88E-07	chr:4:9603240
4	rs57881200	9518764	T	0.3184	2.97E-07	chr:4:9603240
4	rs10002614	9626095	T	2.1	3.05E-07	chr:4:9603240
4	rs6836718	9626703	A	2.1	3.05E-07	chr:4:9603240
4	rs6836875	9626757	A	2.1	3.05E-07	chr:4:9603240
4	rs7654517	9620028	A	2.105	3.14E-07	chr:4:9603240
4	rs147340410	9106171	A	0.4712	3.15E-07	chr:4:9603240
4	rs11730577	9592342	T	5.566	3.22E-07	chr:4:9603240
4	rs11732839	9597885	G	5.566	3.22E-07	chr:4:9603240
4	rs11737375	9607387	G	5.566	3.22E-07	chr:4:9603240
4	rs13105082	9604504	G	5.566	3.22E-07	chr:4:9603240
4	rs13108259	9603571	G	5.566	3.22E-07	chr:4:9603240
4	rs13120086	9599365	T	5.566	3.22E-07	chr:4:9603240
4	rs13124589	9590540	T	5.566	3.22E-07	chr:4:9603240
4	rs62409962	9600872	T	5.566	3.22E-07	chr:4:9603240
4	rs2865076	9591550	C	0.514	3.27E-07	chr:4:9603240
4	rs2867383	9787935	A	0.5202	3.28E-07	chr:4:9603240
4	rs2903564	9787937	A	0.5202	3.28E-07	chr:4:9603240
4	rs6813899	9654942	A	5.153	3.34E-07	chr:4:9603240
4	rs13147553	9610951	A	5.019	3.57E-07	chr:4:9603240
4	rs62293221	9763306	C	0.5203	3.58E-07	chr:4:9603240
4	rs4352476	9621475	C	2.081	3.58E-07	chr:4:9603240
4	rs4504250	9621700	T	2.081	3.58E-07	chr:4:9603240
4	rs4558872	9621642	A	2.081	3.58E-07	chr:4:9603240
4	rs13119059	9847860	C	3.072	3.62E-07	chr:4:9603240
4	rs60678461	9501499	G	0.3098	3.64E-07	chr:4:9603240
4	rs28826976	9631246	A	2.079	3.71E-07	chr:4:9603240
4	rs13120469	9623671	C	2.076	4.20E-07	chr:4:9603240
4	rs6448878	9620623	G	2.076	4.20E-07	chr:4:9603240
4	rs6448879	9620994	T	2.076	4.20E-07	chr:4:9603240
4	rs7661238	9620481	T	2.076	4.20E-07	chr:4:9603240
4	rs7693408	9623110	G	2.076	4.20E-07	chr:4:9603240
4	rs79887705	9622505	T	2.076	4.20E-07	chr:4:9603240
4	rs113222128	9626999	A	2.631	4.21E-07	chr:4:9603240
4	rs11729458	9630071	A	2.075	4.25E-07	chr:4:9603240
4	rs11933525	9630504	G	2.075	4.25E-07	chr:4:9603240
4	rs11939928	9630491	T	2.075	4.25E-07	chr:4:9603240

4	rs12505789	9630602	A	2.075	4.25E-07	chr:4:9603240
4	rs142275227	9631513	T	2.075	4.25E-07	chr:4:9603240
4	rs7666466	9629503	G	2.075	4.25E-07	chr:4:9603240
4	rs78469741	9631526	C	2.075	4.25E-07	chr:4:9603240
4	rs11733687	9643374	T	3.379	4.26E-07	chr:4:9603240
4	rs10939515	9773296	A	0.4827	4.66E-07	chr:4:9603240
4	rs10939600	9886931	A	2.454	4.66E-07	chr:4:9603240
4	rs6449089	9884258	G	2.454	4.66E-07	chr:4:9603240
4	rs6449090	9884536	T	2.454	4.66E-07	chr:4:9603240
4	rs6838891	9884114	C	2.454	4.66E-07	chr:4:9603240
4	rs6848689	9884197	A	2.454	4.66E-07	chr:4:9603240
4	rs4697908	9893282	T	2.425	4.70E-07	chr:4:9603240
4	rs13134147	9611368	G	5.182	5.02E-07	chr:4:9603240
4	rs116305119	9692931	T	4.346	5.06E-07	chr:4:9603240
4	rs7669444	9893577	A	2.424	5.07E-07	chr:4:9603240
4	rs141451880	9572788	T	7.636	5.30E-07	chr:4:9603240
4	rs28490020	9425351	T	5.676	5.54E-07	chr:4:9603240
4	rs6856161	9633826	G	2.574	5.73E-07	chr:4:9603240
4	rs6448960	9686586	T	3.089	5.74E-07	chr:4:9603240
4	rs7680572	9687543	A	3.089	5.74E-07	chr:4:9603240
4	rs145096741	9697413	T	4.71	5.94E-07	chr:4:9603240
4	rs4974864	9588259	G	0.4916	5.96E-07	chr:4:9603240
4	rs62409891	9643131	C	2.573	6.11E-07	chr:4:9603240
4	rs1947868	9584808	C	4.765	6.12E-07	chr:4:9603240
4	rs13114702	9895176	C	3.086	6.17E-07	chr:4:9603240
4	rs142574103	9646506	C	4.295	6.19E-07	chr:4:9603240
4	rs143069896	9647474	A	4.295	6.19E-07	chr:4:9603240
4	rs191365255	9645629	G	4.295	6.19E-07	chr:4:9603240
4	rs11733921	9632876	T	2.064	6.41E-07	chr:4:9603240
4	rs55901556	9492888	A	0.4506	6.95E-07	chr:4:9603240
4	rs6810574	9589675	A	0.5201	6.98E-07	chr:4:9603240
4	rs10011580	9634989	A	2.554	7.08E-07	chr:4:9603240
4	rs59114318	9634777	A	2.554	7.08E-07	chr:4:9603240
4	rs59245820	9634816	G	2.554	7.08E-07	chr:4:9603240
4	rs6813740	9635102	T	2.554	7.08E-07	chr:4:9603240
4	rs6836184	9633711	A	2.554	7.08E-07	chr:4:9603240
4	rs6837618	9634403	A	2.554	7.08E-07	chr:4:9603240
4	rs9291589	9633410	C	2.554	7.08E-07	chr:4:9603240
4	rs7688167	9893506	G	2.38	7.23E-07	chr:4:9603240
4	rs28430858	9626149	A	2.022	7.23E-07	chr:4:9603240
4	rs6842855	9633990	T	2.549	7.34E-07	chr:4:9603240
4	rs6448959	9683927	T	3.053	7.49E-07	chr:4:9603240
4	rs11727601	9637799	G	2.543	7.76E-07	chr:4:9603240

4	rs7696971	9884528	T	2.349	7.86E-07	chr:4:9603240
4	rs6843869	9634451	T	2.553	7.92E-07	chr:4:9603240
4	rs56653599	9642038	T	2.541	8.35E-07	chr:4:9603240
4	rs2061995	9561739	C	1.913	8.75E-07	chr:4:9603240
4	rs140698773	9656963	A	4.056	8.78E-07	chr:4:9603240
4	rs145155902	9655859	T	4.056	8.78E-07	chr:4:9603240
4	rs148558268	9650816	C	4.056	8.78E-07	chr:4:9603240
4	rs149901527	9650222	T	4.056	8.78E-07	chr:4:9603240
4	rs151010672	9653136	T	4.056	8.78E-07	chr:4:9603240
4	rs143116957	9710108	A	5.961	9.06E-07	chr:4:9603240
4	rs3864193	9584474	A	4.66	9.12E-07	chr:4:9603240
4	rs4102945	9581863	T	4.656	9.27E-07	chr:4:9603240
4	rs1811570	9587999	A	0.5224	9.75E-07	chr:4:9603240
4	rs563515175	9138173	G	0.518	1.05E-06	chr:4:9603240
4	rs9760761	9565469	C	1.902	1.07E-06	chr:4:9603240
4	rs11734592	9641381	T	3.25	1.10E-06	chr:4:9603240
4	rs62411071	9640314	A	3.25	1.10E-06	chr:4:9603240
4	rs190136506	9588618	T	5.9	1.11E-06	chr:4:9603240
4	rs576330935	9678540	G	0.312	1.11E-06	chr:4:9603240
4	rs143075413	9640102	T	4.15	1.15E-06	chr:4:9603240
4	rs148263591	9685524	A	4.003	1.15E-06	chr:4:9603240
4	rs150765292	9685713	C	4.003	1.15E-06	chr:4:9603240
4	rs151050464	9682970	C	4.003	1.15E-06	chr:4:9603240
4	rs4440216	9681531	T	3.011	1.16E-06	chr:4:9603240
4	rs79728548	9527286	G	0.3604	1.19E-06	chr:4:9603240
4	rs193204674	9138282	G	0.522	1.21E-06	chr:4:9603240
4	rs141494277	9675657	C	3.983	1.22E-06	chr:4:9603240
4	rs144614831	9671929	T	3.983	1.22E-06	chr:4:9603240
4	rs145492372	9675554	G	3.983	1.22E-06	chr:4:9603240
4	rs147715620	9672824	A	3.983	1.22E-06	chr:4:9603240
4	rs147423360	9138296	C	0.5247	1.23E-06	chr:4:9603240
4	rs11729203	9569091	T	3.385	1.28E-06	chr:4:9603240
4	rs13129232	9573407	T	4.564	1.31E-06	chr:4:9603240
4	rs13144634	9571273	G	4.564	1.31E-06	chr:4:9603240
4	rs114016786	9635321	T	4.106	1.40E-06	chr:4:9603240
4	rs148519502	9634313	C	4.106	1.40E-06	chr:4:9603240
4	rs112714712	9643479	G	3.03	1.44E-06	chr:4:9603240
4	rs77272440	9521226	C	0.4723	1.50E-06	chr:4:9603240
4	rs10006409	9559593	C	1.938	1.52E-06	chr:4:9603240
4	rs11728002	9632904	A	2.712	1.54E-06	chr:4:9603240
4	rs11731299	9632932	G	2.712	1.54E-06	chr:4:9603240
4	rs139494050	9576026	C	4.721	1.55E-06	chr:4:9603240
4	rs144095124	9578290	G	4.721	1.55E-06	chr:4:9603240

4	rs144745779	9582675	C	4.721	1.55E-06	chr:4:9603240
4	rs146486292	9579767	T	4.721	1.55E-06	chr:4:9603240
4	rs148129824	9578976	T	4.721	1.55E-06	chr:4:9603240
4	rs148656483	9579785	T	4.721	1.55E-06	chr:4:9603240
4	rs10005684	9626897	T	2.008	1.56E-06	chr:4:9603240
4	rs36027609	9541970	T	0.3354	1.58E-06	chr:4:9603240
4	rs9884669	9563294	C	1.876	1.60E-06	chr:4:9603240
4	rs114251526	9664763	A	3.924	1.63E-06	chr:4:9603240
4	rs141555885	9678916	A	3.924	1.63E-06	chr:4:9603240
4	rs141621221	9680742	T	3.924	1.63E-06	chr:4:9603240
4	rs148876755	9664792	C	3.924	1.63E-06	chr:4:9603240
4	rs80194314	9682611	T	3.924	1.63E-06	chr:4:9603240
4	rs10805307	9580141	A	4.5	1.70E-06	chr:4:9603240
4	rs10939373	9579571	T	4.5	1.70E-06	chr:4:9603240
4	rs11722510	9585639	A	4.5	1.70E-06	chr:4:9603240
4	rs11736658	9585855	T	4.5	1.70E-06	chr:4:9603240
4	rs13125486	9575367	T	4.5	1.70E-06	chr:4:9603240
4	rs6817939	9588339	A	2.004	1.73E-06	chr:4:9603240
4	rs13132135	9583284	T	4.527	1.75E-06	chr:4:9603240
4	rs141971639	9127659	C	0.5204	1.75E-06	chr:4:9603240
4	rs148459006	9138330	T	0.5227	1.77E-06	chr:4:9603240
4	rs13127386	9636309	T	3.127	1.82E-06	chr:4:9603240
4	rs6841523	9549516	G	1.903	1.89E-06	chr:4:9603240
4	rs1519094	9817286	T	4.365	1.90E-06	chr:4:9603240
4	rs12510498	9606546	A	5.311	1.91E-06	chr:4:9603240
4	rs4974831	9525674	T	0.544	1.98E-06	chr:4:9603240
4	rs6855095	9819977	G	2.44	1.98E-06	chr:4:9603240
4	rs190003305	9709175	T	4.052	2.00E-06	chr:4:9603240
4	rs11730777	9768556	T	0.3566	2.08E-06	chr:4:9603240
4	rs1904944	9144568	G	0.5229	2.14E-06	chr:4:9603240
4	rs1904945	9144504	G	0.5229	2.14E-06	chr:4:9603240
4	rs1964557	9565668	A	0.3433	2.17E-06	chr:4:9603240
4	rs34523133	9565010	C	0.3433	2.17E-06	chr:4:9603240
4	rs34672750	9551410	T	0.3433	2.17E-06	chr:4:9603240
4	rs34899372	9561627	T	0.3433	2.17E-06	chr:4:9603240
4	rs35848567	9549756	A	0.3433	2.17E-06	chr:4:9603240
4	rs35888628	9551409	A	0.3433	2.17E-06	chr:4:9603240
4	rs4974870	9566083	C	0.3433	2.17E-06	chr:4:9603240
4	rs55634110	9643904	T	2.925	2.35E-06	chr:4:9603240
4	rs10939258	9525869	A	0.5474	2.36E-06	chr:4:9603240
4	rs11727040	9657302	C	0.5289	2.39E-06	chr:4:9603240
4	rs188897845	9411622	A	6.14	2.39E-06	chr:4:9603240
4	rs34658640	9137757	T	0.5259	2.41E-06	chr:4:9603240

4	rs149139210	9146298	T	0.5244	2.42E-06	chr:4:9603240
4	rs35881783	9140558	T	0.5262	2.46E-06	chr:4:9603240
4	rs34377195	9140410	A	0.5245	2.46E-06	chr:4:9603240
4	rs187748995	9490915	T	0.3218	2.52E-06	chr:4:9603240
4	rs2865051	9552430	G	1.898	2.70E-06	chr:4:9603240
4	rs28408800	9565507	C	1.84	2.74E-06	chr:4:9603240
4	rs142595860	9138358	C	0.5283	2.84E-06	chr:4:9603240
4	rs13434403	9415059	T	6.06	2.87E-06	chr:4:9603240
4	rs11727477	9768621	A	0.3697	2.88E-06	chr:4:9603240
4	rs57480541	9712078	T	4.498	2.89E-06	chr:4:9603240
4	rs4274842	9676030	A	0.5339	2.91E-06	chr:4:9603240
4	rs4370138	9667026	T	0.5339	2.91E-06	chr:4:9603240
4	rs6449056	9377525	T	5.541	2.94E-06	chr:4:9603240
4	rs61166583	9778530	A	0.5257	2.96E-06	chr:4:9603240
4	rs10032544	9556417	G	1.887	2.97E-06	chr:4:9603240
4	rs7668047	9424418	T	2.912	3.00E-06	chr:4:9603240
4	rs116588617	9577543	G	0.3618	3.03E-06	chr:4:9603240
4	rs2086493	9135431	C	0.5289	3.08E-06	chr:4:9603240
4	rs10009248	9413239	A	6.022	3.09E-06	chr:4:9603240
4	rs115759716	9421961	G	6.022	3.09E-06	chr:4:9603240
4	rs9991609	9416843	C	6.022	3.09E-06	chr:4:9603240
4	rs10010621	9668706	G	2.743	3.20E-06	chr:4:9603240
4	rs4532233	9668924	G	2.743	3.20E-06	chr:4:9603240
4	rs6848526	9670487	T	2.743	3.20E-06	chr:4:9603240
4	rs7434744	9672688	A	2.743	3.20E-06	chr:4:9603240
4	rs9685755	9670178	T	2.743	3.20E-06	chr:4:9603240
4	rs143296337	9142775	T	0.5281	3.26E-06	chr:4:9603240
4	rs141420764	9137720	T	0.5299	3.27E-06	chr:4:9603240
4	rs34105649	9137737	A	0.5299	3.27E-06	chr:4:9603240
4	rs56000261	9704355	T	0.416	3.33E-06	chr:4:9603240
4	rs34433551	9556102	T	0.3532	3.41E-06	chr:4:9603240
4	rs143069383	9594711	G	4.708	3.44E-06	chr:4:9603240
4	rs11731224	9640971	G	2.396	3.58E-06	chr:4:9603240
4	rs184909457	9639179	A	3.898	3.60E-06	chr:4:9603240
4	rs28729253	9602291	A	7.573	3.65E-06	chr:4:9603240
4	rs62409885	9642489	T	3.567	3.66E-06	chr:4:9603240
4	rs13136319	9685017	T	0.5373	3.68E-06	chr:4:9603240
4	rs7658414	9687969	T	0.5373	3.68E-06	chr:4:9603240
4	rs11727873	9758469	C	0.5292	3.69E-06	chr:4:9603240
4	rs139337524	9414895	A	5.939	3.73E-06	chr:4:9603240
4	rs6825744	9553832	G	1.873	3.74E-06	chr:4:9603240
4	rs6849024	9553433	A	1.873	3.74E-06	chr:4:9603240
4	rs9683933	9594670	A	2.425	3.85E-06	chr:4:9603240

4	rs12108245	9418546	G	5.921	3.85E-06	chr:4:9603240
4	rs149732552	9417907	C	5.921	3.85E-06	chr:4:9603240
4	rs28617591	9417770	T	5.921	3.85E-06	chr:4:9603240
4	rs35554314	9148327	C	0.538	3.88E-06	chr:4:9603240
4	rs151336389	9146467	G	0.5326	3.91E-06	chr:4:9603240
4	rs28757760	9411386	A	5.916	3.92E-06	chr:4:9603240
4	rs149108994	9144349	C	0.5342	4.01E-06	chr:4:9603240
4	rs114982594	9599260	G	7.478	4.06E-06	chr:4:9603240
4	rs9291575	9550798	A	1.896	4.09E-06	chr:4:9603240
4	rs10011621	9760239	C	0.5264	4.10E-06	chr:4:9603240
4	rs1947867	9584773	A	0.5432	4.11E-06	chr:4:9603240
4	rs9684038	9585837	A	0.5432	4.11E-06	chr:4:9603240
4	rs34907188	9534556	T	0.3487	4.15E-06	chr:4:9603240
4	rs4974840	9143368	A	0.5347	4.19E-06	chr:4:9603240
4	rs4974841	9143357	G	0.5347	4.19E-06	chr:4:9603240
4	rs4260537	9678215	A	2.708	4.29E-06	chr:4:9603240
4	rs4485817	9674732	G	2.708	4.29E-06	chr:4:9603240
4	rs114198265	9699093	A	3.602	4.30E-06	chr:4:9603240
4	rs35143284	9139875	A	0.5322	4.41E-06	chr:4:9603240
4	rs1904942	9144778	A	0.5352	4.44E-06	chr:4:9603240
4	rs151275640	9138407	G	0.5342	4.45E-06	chr:4:9603240
4	rs11732272	9768258	G	0.3734	4.47E-06	chr:4:9603240
4	rs143633314	9126186	G	0.5166	4.48E-06	chr:4:9603240
4	rs62409887	9642748	G	2.421	4.55E-06	chr:4:9603240
4	rs13116244	9678901	C	0.5405	4.57E-06	chr:4:9603240
4	rs71509133	9138551	G	0.5389	4.62E-06	chr:4:9603240
4	rs13118016	9585215	A	0.544	4.66E-06	chr:4:9603240
4	rs13136183	9683883	T	0.5423	4.70E-06	chr:4:9603240
4	rs28537474	9594779	T	8.71	4.81E-06	chr:4:9603240
4	rs28504038	9603671	T	7.358	4.92E-06	chr:4:9603240
4	rs35007886	9526152	A	0.3586	5.04E-06	chr:4:9603240
4	rs4974877	9525399	A	0.3586	5.04E-06	chr:4:9603240
4	rs13434688	9415633	A	5.803	5.06E-06	chr:4:9603240
4	rs113919186	9561488	G	1.856	5.08E-06	chr:4:9603240
4	rs11942505	9564504	C	1.856	5.08E-06	chr:4:9603240
4	rs28733833	9564338	T	1.856	5.08E-06	chr:4:9603240
4	rs6812931	9566656	C	1.856	5.08E-06	chr:4:9603240
4	rs9884691	9561348	T	1.856	5.08E-06	chr:4:9603240
4	rs35860603	9141447	A	0.539	5.19E-06	chr:4:9603240
4	rs142185832	9144352	G	0.5396	5.32E-06	chr:4:9603240
4	rs146530806	9144304	C	0.5396	5.32E-06	chr:4:9603240
4	rs113534025	9590971	C	2.701	5.37E-06	chr:4:9603240
4	rs143713351	9142597	C	0.5382	5.42E-06	chr:4:9603240

4	rs112166230	9564464	C	1.877	5.60E-06	chr:4:9603240
4	rs113137430	9562106	T	1.877	5.60E-06	chr:4:9603240
4	rs113785368	9562216	C	1.877	5.60E-06	chr:4:9603240
4	rs9884664	9563037	C	1.877	5.60E-06	chr:4:9603240
4	rs9884684	9561232	T	1.877	5.60E-06	chr:4:9603240
4	rs9997903	9555405	A	1.849	5.62E-06	chr:4:9603240
4	rs138241899	9143654	G	0.5395	5.78E-06	chr:4:9603240
4	rs6820665	9553569	T	0.337	5.87E-06	chr:4:9603240
4	rs28857279	9416476	T	5.731	5.91E-06	chr:4:9603240
4	rs28445426	9412201	T	5.732	5.93E-06	chr:4:9603240
4	rs10222812	9424045	T	7.212	6.28E-06	chr:4:9603240
4	rs13435504	9415239	A	7.212	6.28E-06	chr:4:9603240
4	rs141859376	9421945	G	7.212	6.28E-06	chr:4:9603240
4	rs143093608	9422266	G	7.212	6.28E-06	chr:4:9603240
4	rs189998629	9423437	A	7.212	6.28E-06	chr:4:9603240
4	rs28569198	9417795	G	7.212	6.28E-06	chr:4:9603240
4	rs28588147	9420233	C	7.212	6.28E-06	chr:4:9603240
4	rs28699068	9422649	T	7.212	6.28E-06	chr:4:9603240
4	rs62412114	9557691	A	3.415	6.29E-06	chr:4:9603240
4	rs144362537	9135598	G	0.5389	6.29E-06	chr:4:9603240
4	rs35343103	9134625	C	0.5389	6.29E-06	chr:4:9603240
4	rs13142396	9722847	G	2.642	6.35E-06	chr:4:9603240
4	rs10000818	9758234	A	0.4982	6.40E-06	chr:4:9603240
4	rs34257804	9139679	G	0.5409	6.41E-06	chr:4:9603240
4	rs6814282	9692516	T	0.5655	6.53E-06	chr:4:9603240
4	rs143003203	9127749	C	0.5334	6.75E-06	chr:4:9603240
4	rs6858614	9889740	T	2.08	6.76E-06	chr:4:9603240
4	rs6825221	9589620	G	0.5501	6.83E-06	chr:4:9603240
4	rs1842494	9569460	A	0.3613	6.87E-06	chr:4:9603240
4	rs12500696	9554200	A	0.5435	7.03E-06	chr:4:9603240
4	rs7676603	9775710	G	0.5412	7.11E-06	chr:4:9603240
4	rs10008721	9568038	A	7.065	7.30E-06	chr:4:9603240
4	rs140042917	9564766	A	7.065	7.30E-06	chr:4:9603240
4	rs9884663	9562978	T	7.065	7.30E-06	chr:4:9603240
4	rs146768165	9142282	G	0.5149	7.33E-06	chr:4:9603240
4	rs13104360	9684804	C	2.631	7.43E-06	chr:4:9603240
4	rs188754540	9397975	T	3.59	7.48E-06	chr:4:9603240
4	rs10027585	9424582	T	7.091	7.56E-06	chr:4:9603240
4	rs544975809	9640380	T	0.3984	7.63E-06	chr:4:9603240
4	rs114501308	9523606	C	0.3686	7.69E-06	chr:4:9603240
4	rs141104069	9418469	G	7.078	7.73E-06	chr:4:9603240
4	rs10029901	9506829	T	0.5473	8.32E-06	chr:4:9603240
4	rs3857166	9136261	G	0.5445	8.33E-06	chr:4:9603240

4	rs142136009	9715455	T	0.3231	8.33E-06	chr:4:9603240
4	rs2865028	9499342	A	0.2337	8.38E-06	chr:4:9603240
4	rs139343792	9572589	C	7.008	8.39E-06	chr:4:9603240
4	rs28379551	9583509	C	7.008	8.39E-06	chr:4:9603240
4	rs28569802	9587761	T	7.008	8.39E-06	chr:4:9603240
4	rs9990816	9587343	A	7.008	8.39E-06	chr:4:9603240
4	rs113168681	9692858	T	0.401	8.50E-06	chr:4:9603240
4	rs181340952	9670537	T	0.401	8.50E-06	chr:4:9603240
4	rs188176101	9631903	A	3.248	8.59E-06	chr:4:9603240
4	rs28566607	9542165	A	6.948	8.72E-06	chr:4:9603240
4	rs7437720	9510396	A	0.5188	8.80E-06	chr:4:9603240
4	rs10034040	9413585	A	6.984	9.02E-06	chr:4:9603240
4	rs10213123	9595433	G	8.201	9.14E-06	chr:4:9603240
4	rs116801488	9994132	A	199.8	3.44E-23	chr:4:9981605
4	rs116802845	10002728	G	199.8	3.44E-23	chr:4:9981605
4	rs16891964	9997686	G	199.8	3.44E-23	chr:4:9981605
4	rs79070322	9989896	C	199.8	3.44E-23	chr:4:9981605
4	rs16892124	10006832	A	225.4	1.34E-22	chr:4:9981605
4	rs79695094	10007924	T	225.4	1.34E-22	chr:4:9981605
4	rs78009136	9991231	A	285.9	3.96E-22	chr:4:9981605
4	rs79014420	9999406	G	280.4	4.21E-22	chr:4:9981605
4	rs115809052	9998603	C	339.2	4.13E-21	chr:4:9981605
4	rs79893309	9985629	G	339.2	4.13E-21	chr:4:9981605
4	rs13116337	9947669	A	35.68	3.80E-20	chr:4:9981605
4	rs77459722	9947200	A	35.68	3.80E-20	chr:4:9981605
4	rs114219058	9821684	T	59.84	7.82E-20	chr:4:9981605
4	rs75315521	9832084	T	59.84	7.82E-20	chr:4:9981605
4	rs75815764	9827738	C	59.84	7.82E-20	chr:4:9981605
4	rs79056900	9825695	C	59.84	7.82E-20	chr:4:9981605
4	rs115462081	9891797	A	65.03	8.01E-20	chr:4:9981605
4	rs115989654	9846120	G	65.03	8.01E-20	chr:4:9981605
4	rs116459080	9875776	G	65.03	8.01E-20	chr:4:9981605
4	rs139372859	9886571	T	65.03	8.01E-20	chr:4:9981605
4	rs6847291	9848487	T	65.03	8.01E-20	chr:4:9981605
4	rs76482463	9853573	C	65.03	8.01E-20	chr:4:9981605
4	rs7690760	9881572	A	65.03	8.01E-20	chr:4:9981605
4	rs79026415	9873601	A	65.03	8.01E-20	chr:4:9981605
4	rs115968953	9981233	T	430.6	8.66E-20	chr:4:9981605
4	rs7669698	9942866	A	430.6	8.66E-20	chr:4:9981605
4	rs77091057	9960471	C	430.6	8.66E-20	chr:4:9981605
4	rs78676381	9947593	G	430.6	8.66E-20	chr:4:9981605
4	rs79384197	9967504	G	430.6	8.66E-20	chr:4:9981605
4	rs116574712	9860662	A	65.29	2.80E-19	chr:4:9981605

4	rs115124464	9806079	A	60.07	4.35E-19	chr:4:9981605
4	rs76685428	9802355	G	60.07	4.35E-19	chr:4:9981605
4	rs112432698	9817787	A	36.11	1.59E-18	chr:4:9981605
4	rs76318215	9814434	T	36.11	1.59E-18	chr:4:9981605
4	rs141052118	9792197	C	31.91	3.26E-18	chr:4:9981605
4	rs2139238	9818655	A	30.75	3.77E-18	chr:4:9981605
4	rs145718821	9792518	A	29.53	6.77E-18	chr:4:9981605
4	rs7698519	9796745	A	29.53	6.77E-18	chr:4:9981605
4	rs3796840	10008121	T	119.2	9.02E-18	chr:4:9981605
4	rs78465681	9810274	C	104.1	1.86E-17	chr:4:9981605
4	rs74986450	9949603	A	17.83	1.73E-16	chr:4:9981605
4	rs16891971	9998376	A	24.55	1.86E-15	chr:4:9981605
4	rs67968745	10008749	A	21.32	1.05E-14	chr:4:9981605
4	rs73805854	9792921	C	14.55	2.40E-14	chr:4:9981605
4	rs7678287	10000501	A	11.18	2.80E-14	chr:4:9981605
4	rs36058283	10008512	A	30.35	3.06E-14	chr:4:9981605
4	rs78550522	9974844	A	97.99	7.68E-14	chr:4:9981605
4	rs78685278	9975590	T	97.99	7.68E-14	chr:4:9981605
4	rs4697891	9822319	G	11.64	9.00E-14	chr:4:9981605
4	rs9991278	10002665	T	12.17	1.18E-13	chr:4:9981605
4	rs116662392	9995726	T	309	1.24E-13	chr:4:9981605
4	rs148448488	9969238	A	309	1.24E-13	chr:4:9981605
4	rs150088572	9930273	A	309	1.24E-13	chr:4:9981605
4	rs75985854	9898051	C	309	1.24E-13	chr:4:9981605
4	rs79989591	10002517	C	309	1.24E-13	chr:4:9981605
4	rs13113918	9998493	A	9.303	2.11E-13	chr:4:9981605
4	rs28592748	9998605	T	9.303	2.11E-13	chr:4:9981605
4	rs7683856	10000947	A	9.303	2.11E-13	chr:4:9981605
4	rs115474850	9848833	C	293.4	2.38E-13	chr:4:9981605
4	rs74991865	9887610	C	293.4	2.38E-13	chr:4:9981605
4	rs76504720	9879304	A	293.4	2.38E-13	chr:4:9981605
4	rs78190988	9865366	T	293.4	2.38E-13	chr:4:9981605
4	rs7696983	9995829	A	9.303	2.45E-13	chr:4:9981605
4	rs7694997	9947811	A	7.481	4.34E-13	chr:4:9981605
4	rs4622999	10003395	G	8.395	4.50E-13	chr:4:9981605
4	rs6817961	9850779	A	276.4	4.52E-13	chr:4:9981605
4	rs6849962	9986903	A	7.77	4.83E-13	chr:4:9981605
4	rs1122142	9947548	G	6.926	1.00E-12	chr:4:9981605
4	rs2018643	9947121	T	6.926	1.00E-12	chr:4:9981605
4	rs4339211	9947658	C	6.926	1.00E-12	chr:4:9981605
4	rs185949181	9764482	A	41.89	1.10E-12	chr:4:9981605
4	rs78921276	10002138	A	80.92	1.12E-12	chr:4:9981605
4	rs141292564	9785521	A	36.09	1.16E-12	chr:4:9981605

4	rs150796194	9785584	G	36.09	1.16E-12	chr:4:9981605
4	rs3733590	9987226	C	7.348	1.50E-12	chr:4:9981605
4	rs3775949	9987667	G	7.348	1.50E-12	chr:4:9981605
4	rs6836200	9986915	C	7.348	1.50E-12	chr:4:9981605
4	rs73100621	9986339	G	7.348	1.50E-12	chr:4:9981605
4	rs6849736	9986783	A	7.316	1.72E-12	chr:4:9981605
4	rs35954357	9978758	C	6.645	3.10E-12	chr:4:9981605
4	rs7696971	9884528	T	6.579	4.08E-12	chr:4:9981605
4	rs6449158	9960459	T	6.285	6.61E-12	chr:4:9981605
4	rs13145554	9979589	T	6.351	6.74E-12	chr:4:9981605
4	rs10001964	9959275	C	6.289	6.76E-12	chr:4:9981605
4	rs10939636	9972640	T	6.289	6.76E-12	chr:4:9981605
4	rs113439739	9957984	G	6.289	6.76E-12	chr:4:9981605
4	rs150258593	9957950	A	6.289	6.76E-12	chr:4:9981605
4	rs4305511	9955545	T	6.289	6.76E-12	chr:4:9981605
4	rs4455410	9953297	T	6.289	6.76E-12	chr:4:9981605
4	rs4473653	9971058	A	6.289	6.76E-12	chr:4:9981605
4	rs6449171	9965998	T	6.289	6.76E-12	chr:4:9981605
4	rs6449174	9966422	C	6.289	6.76E-12	chr:4:9981605
4	rs6449178	9968684	C	6.289	6.76E-12	chr:4:9981605
4	rs6850684	9964380	G	6.289	6.76E-12	chr:4:9981605
4	rs6852441	9973744	C	6.289	6.76E-12	chr:4:9981605
4	rs7376948	9954708	G	6.289	6.76E-12	chr:4:9981605
4	rs7437120	9967683	T	6.289	6.76E-12	chr:4:9981605
4	rs9994937	9964799	G	6.289	6.76E-12	chr:4:9981605
4	rs114799904	10419671	A	61.18	7.59E-12	chr:4:9981605
4	rs115133815	10437198	T	61.18	7.59E-12	chr:4:9981605
4	rs150360090	10434441	C	61.18	7.59E-12	chr:4:9981605
4	rs13103497	9979262	G	6.361	7.78E-12	chr:4:9981605
4	rs74513897	9979852	T	157.8	7.83E-12	chr:4:9981605
4	rs6449201	9973894	C	6.277	8.50E-12	chr:4:9981605
4	rs147685156	9735702	G	22.68	9.25E-12	chr:4:9981605
4	rs56296130	9975461	T	6.069	1.17E-11	chr:4:9981605
4	rs112890065	9746653	A	18.49	1.31E-11	chr:4:9981605
4	rs4276278	9948870	T	6.039	1.49E-11	chr:4:9981605
4	rs4408959	9948868	C	6.039	1.49E-11	chr:4:9981605
4	rs17245723	9962218	A	5.914	1.55E-11	chr:4:9981605
4	rs112674844	9807999	T	7.694	1.95E-11	chr:4:9981605
4	rs111502994	9767409	T	17.69	2.24E-11	chr:4:9981605
4	rs149377109	9716721	A	19.39	2.28E-11	chr:4:9981605
4	rs185579962	9709152	T	19.39	2.28E-11	chr:4:9981605
4	rs113426638	9777694	A	17.4	2.33E-11	chr:4:9981605
4	rs10018663	10004797	C	7.386	2.40E-11	chr:4:9981605

4	rs751092	10005897	C	6.954	2.65E-11	chr:4:9981605
4	rs13141248	9845790	T	7.89	3.86E-11	chr:4:9981605
4	rs150659411	9845787	T	7.89	3.86E-11	chr:4:9981605
4	rs188926585	9710824	T	18.76	3.87E-11	chr:4:9981605
4	rs141360566	9716030	A	7.905	5.48E-11	chr:4:9981605
4	rs116063400	9733661	A	21.73	8.07E-11	chr:4:9981605
4	rs1014290	10001861	G	5.696	1.03E-10	chr:4:9981605
4	rs11943372	9999346	T	5.245	1.34E-10	chr:4:9981605
4	rs4529048	9997112	C	5.245	1.34E-10	chr:4:9981605
4	rs62293225	9777387	T	33.68	1.40E-10	chr:4:9981605
4	rs10939650	9998440	C	5.246	1.44E-10	chr:4:9981605
4	rs3733588	9997303	G	5.24	1.60E-10	chr:4:9981605
4	rs145096741	9697413	T	7.334	1.79E-10	chr:4:9981605
4	rs7686538	9948077	C	5.354	2.19E-10	chr:4:9981605
4	rs112889423	9743135	T	12.96	3.18E-10	chr:4:9981605
4	rs142272392	9743090	A	12.96	3.18E-10	chr:4:9981605
4	rs188814890	9740038	C	12.96	3.18E-10	chr:4:9981605
4	rs12511438	9702289	C	8.027	3.36E-10	chr:4:9981605
4	rs1401437	9819763	G	6.624	3.48E-10	chr:4:9981605
4	rs1401441	9832304	T	6.624	3.48E-10	chr:4:9981605
4	rs1401442	9839447	C	6.624	3.48E-10	chr:4:9981605
4	rs1519092	9826801	T	6.624	3.48E-10	chr:4:9981605
4	rs1519093	9824019	T	6.624	3.48E-10	chr:4:9981605
4	rs1878277	9825143	T	6.624	3.48E-10	chr:4:9981605
4	rs7656266	9820644	A	6.624	3.48E-10	chr:4:9981605
4	rs939131	9820950	A	6.624	3.48E-10	chr:4:9981605
4	rs3775946	9995256	A	4.908	5.55E-10	chr:4:9981605
4	rs3775947	9995240	C	4.908	5.55E-10	chr:4:9981605
4	rs7677006	10476374	T	7.363	6.52E-10	chr:4:9981605
4	rs78934234	9804560	A	5.954	7.66E-10	chr:4:9981605
4	rs114537931	10190611	A	37.43	9.16E-10	chr:4:9981605
4	rs114739867	10195016	T	37.43	9.16E-10	chr:4:9981605
4	rs35201034	10007451	C	4.553	1.05E-09	chr:4:9981605
4	rs138027354	9923403	G	28.1	1.11E-09	chr:4:9981605
4	rs139343650	9924976	C	28.1	1.11E-09	chr:4:9981605
4	rs140338772	9919724	G	28.1	1.11E-09	chr:4:9981605
4	rs143698928	9926104	A	28.1	1.11E-09	chr:4:9981605
4	rs149517836	9923805	C	28.1	1.11E-09	chr:4:9981605
4	rs150440092	9923303	A	28.1	1.11E-09	chr:4:9981605
4	rs16891038	9925788	G	28.1	1.11E-09	chr:4:9981605
4	rs181639772	9936791	T	28.1	1.11E-09	chr:4:9981605
4	rs183991328	9928890	C	28.1	1.11E-09	chr:4:9981605
4	rs540546895	9924547	G	28.1	1.11E-09	chr:4:9981605

4	rs551569822	9925918	T	28.1	1.11E-09	chr:4:9981605
4	rs938560	9920545	A	28.1	1.11E-09	chr:4:9981605
4	rs138679148	9738111	C	11.18	1.12E-09	chr:4:9981605
4	rs149055696	9701327	A	15.92	1.61E-09	chr:4:9981605
4	rs16890933	9918457	G	24.42	1.80E-09	chr:4:9981605
4	rs16890938	9918845	T	24.42	1.80E-09	chr:4:9981605
4	rs114235948	9772094	T	45.25	1.91E-09	chr:4:9981605
4	rs114803892	9638698	T	45.25	1.91E-09	chr:4:9981605
4	rs115102455	9691658	T	45.25	1.91E-09	chr:4:9981605
4	rs116156748	9722016	A	45.25	1.91E-09	chr:4:9981605
4	rs116532033	9762981	G	45.25	1.91E-09	chr:4:9981605
4	rs138512934	9606124	A	45.25	1.91E-09	chr:4:9981605
4	rs139128075	9604885	C	45.25	1.91E-09	chr:4:9981605
4	rs139668726	9642133	T	45.25	1.91E-09	chr:4:9981605
4	rs140014892	9682547	T	45.25	1.91E-09	chr:4:9981605
4	rs140552624	9671544	T	45.25	1.91E-09	chr:4:9981605
4	rs141975845	9687223	A	45.25	1.91E-09	chr:4:9981605
4	rs143830660	9732131	A	45.25	1.91E-09	chr:4:9981605
4	rs145616091	9669664	T	45.25	1.91E-09	chr:4:9981605
4	rs145895721	9779900	T	45.25	1.91E-09	chr:4:9981605
4	rs146196374	9678205	T	45.25	1.91E-09	chr:4:9981605
4	rs148258101	9620330	A	45.25	1.91E-09	chr:4:9981605
4	rs150608490	9660937	A	45.25	1.91E-09	chr:4:9981605
4	rs150744301	9751219	A	45.25	1.91E-09	chr:4:9981605
4	rs150798057	9748311	A	45.25	1.91E-09	chr:4:9981605
4	rs151251975	9603489	A	45.25	1.91E-09	chr:4:9981605
4	rs181352641	9640935	G	45.25	1.91E-09	chr:4:9981605
4	rs185328312	9592216	G	45.25	1.91E-09	chr:4:9981605
4	rs187417701	9646431	A	45.25	1.91E-09	chr:4:9981605
4	rs187998987	9753216	A	45.25	1.91E-09	chr:4:9981605
4	rs188063506	9679214	A	45.25	1.91E-09	chr:4:9981605
4	rs192115928	9682144	T	45.25	1.91E-09	chr:4:9981605
4	rs547498536	9666233	A	45.25	1.91E-09	chr:4:9981605
4	rs555538409	9667589	G	45.25	1.91E-09	chr:4:9981605
4	rs572808723	9680927	C	45.25	1.91E-09	chr:4:9981605
4	rs6836477	9656054	C	45.25	1.91E-09	chr:4:9981605
4	rs7655043	9762216	T	45.25	1.91E-09	chr:4:9981605
4	rs7665831	9596933	G	45.25	1.91E-09	chr:4:9981605
4	rs7671802	9745503	A	45.25	1.91E-09	chr:4:9981605
4	rs7698544	9766720	A	45.25	1.91E-09	chr:4:9981605
4	rs577897040	10137859	A	62.1	2.01E-09	chr:4:9981605
4	rs1519094	9817286	T	5.833	2.08E-09	chr:4:9981605
4	rs115020739	9925821	T	117.5	2.09E-09	chr:4:9981605

4	rs7658183	9922657	T	117.5	2.09E-09	chr:4:9981605
4	rs4697691	9858485	A	4.582	2.63E-09	chr:4:9981605
4	rs4697900	9859976	A	4.582	2.63E-09	chr:4:9981605
4	rs10939561	9851542	T	4.587	2.64E-09	chr:4:9981605
4	rs1980219	9850621	T	4.586	2.66E-09	chr:4:9981605
4	rs6812007	9850220	C	4.586	2.66E-09	chr:4:9981605
4	rs6828040	9850480	T	4.586	2.66E-09	chr:4:9981605
4	rs6847858	9853059	T	4.586	2.66E-09	chr:4:9981605
4	rs6847871	9853074	T	4.586	2.66E-09	chr:4:9981605
4	rs7683831	9845079	T	4.573	2.92E-09	chr:4:9981605
4	rs4613568	9862902	T	4.57	2.96E-09	chr:4:9981605
4	rs6852425	9856859	G	4.57	2.96E-09	chr:4:9981605
4	rs1519099	9874130	T	5.567	3.19E-09	chr:4:9981605
4	rs150393841	10142737	A	18.92	3.35E-09	chr:4:9981605
4	rs3775948	9995182	G	4.399	3.45E-09	chr:4:9981605
4	rs28591077	9863678	A	4.548	3.46E-09	chr:4:9981605
4	rs9684729	9862036	C	4.548	3.46E-09	chr:4:9981605
4	rs146607046	9725354	A	10.07	3.98E-09	chr:4:9981605
4	rs146524492	9712174	G	32.57	3.99E-09	chr:4:9981605
4	rs149849338	9688109	C	32.57	3.99E-09	chr:4:9981605
4	rs150078827	9770058	G	14.81	4.25E-09	chr:4:9981605
4	rs116432931	9931162	C	6.212	5.64E-09	chr:4:9981605
4	rs116658821	9931161	C	6.212	5.64E-09	chr:4:9981605
4	rs114757726	9770040	G	13.68	6.10E-09	chr:4:9981605
4	rs115503187	9782440	T	13.68	6.10E-09	chr:4:9981605
4	rs141539501	9767488	T	13.68	6.10E-09	chr:4:9981605
4	rs143543860	9773487	A	13.68	6.10E-09	chr:4:9981605
4	rs150887297	9757360	T	13.68	6.10E-09	chr:4:9981605
4	rs79155112	9764669	C	13.68	6.10E-09	chr:4:9981605
4	rs75000154	9653588	A	17.3	6.34E-09	chr:4:9981605
4	rs59024311	9810123	G	4.842	6.89E-09	chr:4:9981605
4	rs141649879	9734132	G	57.34	6.99E-09	chr:4:9981605
4	rs2867385	9795782	G	5.35	8.66E-09	chr:4:9981605
4	rs181110448	9600723	A	16.69	8.84E-09	chr:4:9981605
4	rs115531234	9755596	A	13.06	9.33E-09	chr:4:9981605
4	rs7663032	9993838	C	4.141	1.04E-08	chr:4:9981605
4	rs10006684	9694706	T	4.118	1.08E-08	chr:4:9981605
4	rs114684248	9773367	G	8.102	1.10E-08	chr:4:9981605
4	rs146491327	9772211	T	8.102	1.10E-08	chr:4:9981605
4	rs182426026	9772485	C	8.102	1.10E-08	chr:4:9981605
4	rs13136903	9694522	T	4.11	1.20E-08	chr:4:9981605
4	rs115508089	10447254	A	6	1.22E-08	chr:4:9981605
4	rs6813899	9654942	A	5.955	1.22E-08	chr:4:9981605

4	rs4621431	9947590	G	3.804	1.38E-08	chr:4:9981605
4	rs11941979	9664162	T	5.655	1.44E-08	chr:4:9981605
4	rs151313439	9920006	A	23.92	1.55E-08	chr:4:9981605
4	rs543937324	9764685	T	8.499	1.63E-08	chr:4:9981605
4	rs115383005	9687419	T	10.88	1.81E-08	chr:4:9981605
4	rs116272919	9688351	A	10.88	1.81E-08	chr:4:9981605
4	rs143429481	9690318	C	10.88	1.81E-08	chr:4:9981605
4	rs143715911	9684005	A	10.88	1.81E-08	chr:4:9981605
4	rs187432052	9681383	T	10.88	1.81E-08	chr:4:9981605
4	rs10939600	9886931	A	4.05	1.82E-08	chr:4:9981605
4	rs6449089	9884258	G	4.05	1.82E-08	chr:4:9981605
4	rs6449090	9884536	T	4.05	1.82E-08	chr:4:9981605
4	rs6838891	9884114	C	4.05	1.82E-08	chr:4:9981605
4	rs6848689	9884197	A	4.05	1.82E-08	chr:4:9981605
4	rs7697692	9600852	C	29.21	2.00E-08	chr:4:9981605
4	rs145767910	9671051	T	10.29	2.73E-08	chr:4:9981605
4	rs147221486	9669099	A	10.29	2.73E-08	chr:4:9981605
4	rs190436636	9679893	G	10.29	2.73E-08	chr:4:9981605
4	rs6858093	9681650	G	10.43	2.98E-08	chr:4:9981605
4	rs77017317	10462791	T	5.788	3.13E-08	chr:4:9981605
4	rs12506622	9690369	T	10.02	4.24E-08	chr:4:9981605
4	rs6823671	9692517	A	10.02	4.24E-08	chr:4:9981605
4	rs6829292	9692892	A	10.02	4.24E-08	chr:4:9981605
4	rs6829800	9685856	A	10.02	4.24E-08	chr:4:9981605
4	rs7678732	9693253	T	10.02	4.24E-08	chr:4:9981605
4	rs144261609	9561844	A	27.51	5.06E-08	chr:4:9981605
4	rs188966668	9510024	T	27.51	5.06E-08	chr:4:9981605
4	rs4697908	9893282	T	3.901	5.43E-08	chr:4:9981605
4	rs140456990	9569392	C	27.35	5.45E-08	chr:4:9981605
4	rs187419952	9753263	T	7.438	5.64E-08	chr:4:9981605
4	rs548255756	10136124	C	5.526	5.73E-08	chr:4:9981605
4	rs4077929	9698730	A	4.542	5.81E-08	chr:4:9981605
4	rs4554077	9674736	G	9.479	6.08E-08	chr:4:9981605
4	rs6812811	9672000	T	9.479	6.08E-08	chr:4:9981605
4	rs7688167	9893506	G	3.883	6.25E-08	chr:4:9981605
4	rs883041	9868346	C	3.703	6.40E-08	chr:4:9981605
4	rs116608329	9697295	T	9.425	6.46E-08	chr:4:9981605
4	rs75772786	10467331	G	5.25	7.27E-08	chr:4:9981605
4	rs114957772	9730698	C	6.938	7.83E-08	chr:4:9981605
4	rs144742219	9700280	A	9.205	7.92E-08	chr:4:9981605
4	rs79238430	9492635	T	5.383	8.57E-08	chr:4:9981605
4	rs6448728	9541220	T	13.81	9.87E-08	chr:4:9981605
4	rs10031836	9506539	C	5.602	1.01E-07	chr:4:9981605

4	rs10031907	9506545	C	5.602	1.01E-07	chr:4:9981605
4	rs10034585	9506548	C	5.602	1.01E-07	chr:4:9981605
4	rs149367087	9609347	A	4.954	1.02E-07	chr:4:9981605
4	rs114758567	9739117	T	6.687	1.03E-07	chr:4:9981605
4	rs115727367	9740960	G	6.687	1.03E-07	chr:4:9981605
4	rs116528676	9733207	A	6.687	1.03E-07	chr:4:9981605
4	rs138694626	9736408	T	6.687	1.03E-07	chr:4:9981605
4	rs144508872	9734039	A	6.687	1.03E-07	chr:4:9981605
4	rs145460666	9735643	A	6.687	1.03E-07	chr:4:9981605
4	rs181763499	9737693	T	6.687	1.03E-07	chr:4:9981605
4	rs115100475	9716472	T	43.41	1.07E-07	chr:4:9981605
4	rs538733592	9643095	G	43.41	1.07E-07	chr:4:9981605
4	rs544602607	9647853	C	43.41	1.07E-07	chr:4:9981605
4	rs556137923	9643083	A	43.41	1.07E-07	chr:4:9981605
4	rs560642590	9569518	T	43.41	1.07E-07	chr:4:9981605
4	rs4697903	9865426	G	3.57	1.11E-07	chr:4:9981605
4	rs28437340	9490580	T	5.428	1.15E-07	chr:4:9981605
4	rs13107864	10418989	A	4.326	1.17E-07	chr:4:9981605
4	rs6810406	10418431	A	4.326	1.17E-07	chr:4:9981605
4	rs6858614	9889740	T	3.931	1.18E-07	chr:4:9981605
4	rs10016075	10006663	G	3.622	1.28E-07	chr:4:9981605
4	rs138559943	10043590	G	69	1.32E-07	chr:4:9981605
4	rs143467853	10185209	G	69	1.32E-07	chr:4:9981605
4	rs144470017	10065738	G	69	1.32E-07	chr:4:9981605
4	rs145947196	10330045	G	69	1.32E-07	chr:4:9981605
4	rs181858096	10396022	G	69	1.32E-07	chr:4:9981605
4	rs551329964	10275829	C	69	1.32E-07	chr:4:9981605
4	rs28371074	9492403	T	5.367	1.41E-07	chr:4:9981605
4	rs7669444	9893577	A	3.755	1.46E-07	chr:4:9981605
4	rs28880651	9493311	A	5.347	1.47E-07	chr:4:9981605
4	rs114360895	10154974	T	13.07	1.72E-07	chr:4:9981605
4	rs143048489	9492085	C	5.425	1.76E-07	chr:4:9981605
4	rs6847375	9612811	G	9.379	1.83E-07	chr:4:9981605
4	rs189941972	9792811	A	90.63	1.83E-07	chr:4:9981605
4	rs10018119	9505877	A	5.25	1.87E-07	chr:4:9981605
4	rs10027121	9506061	T	5.25	1.87E-07	chr:4:9981605
4	rs114065417	9496389	T	5.25	1.87E-07	chr:4:9981605
4	rs116049471	9497345	T	5.25	1.87E-07	chr:4:9981605
4	rs142324132	9505654	A	5.25	1.87E-07	chr:4:9981605
4	rs145592249	9509476	A	5.25	1.87E-07	chr:4:9981605
4	rs148246352	9496314	A	5.25	1.87E-07	chr:4:9981605
4	rs28409436	9503519	A	5.25	1.87E-07	chr:4:9981605
4	rs28501113	9500762	A	5.25	1.87E-07	chr:4:9981605

4	rs28536787	9498858	T	5.25	1.87E-07	chr:4:9981605
4	rs28551614	9500307	T	5.25	1.87E-07	chr:4:9981605
4	rs28580561	9503589	C	5.25	1.87E-07	chr:4:9981605
4	rs28628561	9494374	T	5.25	1.87E-07	chr:4:9981605
4	rs28683702	9498629	A	5.25	1.87E-07	chr:4:9981605
4	rs28685834	9498475	C	5.25	1.87E-07	chr:4:9981605
4	rs28709857	9497873	A	5.25	1.87E-07	chr:4:9981605
4	rs28744575	9500593	T	5.25	1.87E-07	chr:4:9981605
4	rs9992409	9503798	T	5.25	1.87E-07	chr:4:9981605
4	rs9992781	9504194	A	5.25	1.87E-07	chr:4:9981605
4	rs114232342	9509298	C	5.364	2.07E-07	chr:4:9981605
4	rs4575994	9997979	G	0.2324	2.21E-07	chr:4:9981605
4	rs114576054	9496139	A	5.328	2.21E-07	chr:4:9981605
4	rs146525859	9496391	A	5.328	2.21E-07	chr:4:9981605
4	rs28421272	9497885	A	5.139	2.29E-07	chr:4:9981605
4	rs4697906	9889896	T	246.3	2.62E-07	chr:4:9981605
4	rs186692764	9626105	G	20.34	2.92E-07	chr:4:9981605
4	rs574102947	9631433	G	20.34	2.92E-07	chr:4:9981605
4	rs6826254	9814766	C	3.655	2.92E-07	chr:4:9981605
4	rs115120277	10408446	T	23.62	3.38E-07	chr:4:9981605
4	rs150790389	9862815	T	27.38	4.13E-07	chr:4:9981605
4	rs7669088	9699627	T	8.303	4.22E-07	chr:4:9981605
4	rs7686375	9734974	A	5.853	5.15E-07	chr:4:9981605
4	rs116615514	10113806	C	23.01	5.39E-07	chr:4:9981605
4	rs140448238	10116398	C	23.01	5.39E-07	chr:4:9981605
4	rs141154634	10089680	C	23.01	5.39E-07	chr:4:9981605
4	rs143195615	10089618	A	23.01	5.39E-07	chr:4:9981605
4	rs563248843	10089839	T	23.01	5.39E-07	chr:4:9981605
4	rs189549531	9535368	G	5.426	5.84E-07	chr:4:9981605
4	rs28441988	9686419	T	5.48	6.04E-07	chr:4:9981605
4	rs28532184	9638350	A	5.48	6.04E-07	chr:4:9981605
4	rs144984865	9863240	T	36.02	6.16E-07	chr:4:9981605
4	rs147530507	9855802	T	36.02	6.16E-07	chr:4:9981605
4	rs147579671	9860554	T	36.02	6.16E-07	chr:4:9981605
4	rs150266310	9861178	C	36.02	6.16E-07	chr:4:9981605
4	rs180932754	9855760	G	36.02	6.16E-07	chr:4:9981605
4	rs114137702	10440735	C	13.5	6.39E-07	chr:4:9981605
4	rs146838297	10467706	G	13.5	6.39E-07	chr:4:9981605
4	rs75900771	10478872	C	13.5	6.39E-07	chr:4:9981605
4	rs147526376	9542349	T	22.31	6.48E-07	chr:4:9981605
4	rs79503885	10236369	C	4.612	6.49E-07	chr:4:9981605
4	rs56113653	9898052	G	3	6.58E-07	chr:4:9981605
4	rs12649073	9694490	A	3.295	6.66E-07	chr:4:9981605

4	rs184691520	10012086	T	46.97	6.78E-07	chr:4:9981605
4	rs542277037	9569512	C	7.448	6.98E-07	chr:4:9981605
4	rs192094959	9504170	G	4.814	7.03E-07	chr:4:9981605
4	rs10022499	10006537	C	3.379	7.11E-07	chr:4:9981605
4	rs6853437	10005435	G	3.379	7.11E-07	chr:4:9981605
4	rs9291640	10007086	C	3.379	7.11E-07	chr:4:9981605
4	rs9291641	10007259	C	3.379	7.11E-07	chr:4:9981605
4	rs4697902	9863936	T	3.247	7.56E-07	chr:4:9981605
4	rs10000913	9511660	T	5.257	7.70E-07	chr:4:9981605
4	rs10007087	9521800	T	5.257	7.70E-07	chr:4:9981605
4	rs10010068	9520235	T	5.257	7.70E-07	chr:4:9981605
4	rs10028800	9524734	A	5.257	7.70E-07	chr:4:9981605
4	rs114146209	9515191	A	5.257	7.70E-07	chr:4:9981605
4	rs114586406	9511557	T	5.257	7.70E-07	chr:4:9981605
4	rs12331300	9521411	A	5.257	7.70E-07	chr:4:9981605
4	rs12331712	9521609	G	5.257	7.70E-07	chr:4:9981605
4	rs142968757	9519884	G	5.257	7.70E-07	chr:4:9981605
4	rs146238242	9520489	A	5.257	7.70E-07	chr:4:9981605
4	rs147542152	9522495	A	5.257	7.70E-07	chr:4:9981605
4	rs150463076	9518435	G	5.257	7.70E-07	chr:4:9981605
4	rs187749189	9512003	C	5.257	7.70E-07	chr:4:9981605
4	rs193097823	9518697	G	5.257	7.70E-07	chr:4:9981605
4	rs371770940	9512400	A	5.257	7.70E-07	chr:4:9981605
4	rs3975611	9522913	C	5.257	7.70E-07	chr:4:9981605
4	rs9992237	9511902	A	5.257	7.70E-07	chr:4:9981605
4	rs10012790	10421725	G	3.734	7.86E-07	chr:4:9981605
4	rs150759864	9620565	T	6.516	8.09E-07	chr:4:9981605
4	rs11726102	9951664	C	0.2652	8.16E-07	chr:4:9981605
4	rs11735831	9951591	A	0.2652	8.16E-07	chr:4:9981605
4	rs10001324	9520425	T	5.225	8.32E-07	chr:4:9981605
4	rs28832644	9510188	G	5.214	8.69E-07	chr:4:9981605
4	rs116084164	9536050	A	5.193	9.27E-07	chr:4:9981605
4	rs145749037	9535232	A	5.193	9.27E-07	chr:4:9981605
4	rs28609060	9533954	A	5.193	9.27E-07	chr:4:9981605
4	rs28644422	9502675	T	4.761	9.53E-07	chr:4:9981605
4	rs10939702	10096692	T	6.475	9.59E-07	chr:4:9981605
4	rs7674723	9916202	C	3.066	1.01E-06	chr:4:9981605
4	rs9994216	9984541	T	3.334	1.16E-06	chr:4:9981605
4	rs114155565	10143654	G	11.7	1.19E-06	chr:4:9981605
4	rs115226953	10133399	G	11.7	1.19E-06	chr:4:9981605
4	rs115613509	10159536	G	11.7	1.19E-06	chr:4:9981605
4	rs116036122	10143370	T	11.7	1.19E-06	chr:4:9981605
4	rs138501326	10136903	T	11.7	1.19E-06	chr:4:9981605

4	rs116138123	9648821	G	6.502	1.23E-06	chr:4:9981605
4	rs28566607	9542165	A	5.023	1.25E-06	chr:4:9981605
4	rs13150941	9694401	A	0.2256	1.25E-06	chr:4:9981605
4	rs114137844	9948482	T	82.87	1.29E-06	chr:4:9981605
4	rs114865151	9965145	A	82.87	1.29E-06	chr:4:9981605
4	rs116477885	9969931	C	82.87	1.29E-06	chr:4:9981605
4	rs140602684	9981019	A	82.87	1.29E-06	chr:4:9981605
4	rs147608714	9960013	C	82.87	1.29E-06	chr:4:9981605
4	rs181435538	9966797	C	82.87	1.29E-06	chr:4:9981605
4	rs11734219	9977154	C	0.2876	1.31E-06	chr:4:9981605
4	rs116189390	10178169	A	11.3	1.35E-06	chr:4:9981605
4	rs4697699	9945722	C	0.2724	1.37E-06	chr:4:9981605
4	rs140701304	9636731	A	6.477	1.38E-06	chr:4:9981605
4	rs140787992	9637047	A	6.477	1.38E-06	chr:4:9981605
4	rs4697700	9945792	C	0.2753	1.38E-06	chr:4:9981605
4	rs142806317	9865940	T	56.82	1.42E-06	chr:4:9981605
4	rs4580649	9948461	G	3.035	1.47E-06	chr:4:9981605
4	rs4697755	10436395	T	3.491	1.48E-06	chr:4:9981605
4	rs7698991	10437334	C	3.491	1.48E-06	chr:4:9981605
4	rs10939637	9977577	A	0.2901	1.52E-06	chr:4:9981605
4	rs10939638	9977675	T	0.2901	1.52E-06	chr:4:9981605
4	rs13102218	9976113	G	0.2901	1.52E-06	chr:4:9981605
4	rs7682751	9976294	A	0.2901	1.52E-06	chr:4:9981605
4	rs10023068	10004832	A	3.236	1.55E-06	chr:4:9981605
4	rs13125209	9982044	A	0.2905	1.57E-06	chr:4:9981605
4	rs13145758	9981997	G	0.2905	1.57E-06	chr:4:9981605
4	rs28479838	9619548	A	3.097	1.57E-06	chr:4:9981605
4	rs4475146	9946656	A	0.2759	1.59E-06	chr:4:9981605
4	rs10008721	9568038	A	4.959	1.60E-06	chr:4:9981605
4	rs140042917	9564766	A	4.959	1.60E-06	chr:4:9981605
4	rs9884663	9562978	T	4.959	1.60E-06	chr:4:9981605
4	rs149501985	9894727	G	57.36	1.66E-06	chr:4:9981605
4	rs187030607	9652686	T	5.419	1.71E-06	chr:4:9981605
4	rs28451938	9652691	T	5.419	1.71E-06	chr:4:9981605
4	rs28705142	9652682	C	5.419	1.71E-06	chr:4:9981605
4	rs10014973	10438430	G	3.043	1.75E-06	chr:4:9981605
4	rs113257452	9537457	T	4.518	1.78E-06	chr:4:9981605
4	rs187100546	9913827	C	165.6	1.84E-06	chr:4:9981605
4	rs557170772	9846892	G	19.11	1.86E-06	chr:4:9981605
4	rs60111228	9825823	A	19.11	1.86E-06	chr:4:9981605
4	rs10018666	10004805	C	3.214	1.89E-06	chr:4:9981605
4	rs2192094	10291968	C	7.701	1.93E-06	chr:4:9981605
4	rs114726622	10012802	A	10.69	1.93E-06	chr:4:9981605

4	rs115250951	10042556	C	10.69	1.93E-06	chr:4:9981605
4	rs116479950	10043188	T	10.69	1.93E-06	chr:4:9981605
4	rs139376229	10023659	T	10.69	1.93E-06	chr:4:9981605
4	rs190290723	10046675	C	10.69	1.93E-06	chr:4:9981605
4	rs142144404	9641021	T	5.288	1.93E-06	chr:4:9981605
4	rs182393147	9681451	A	5.288	1.93E-06	chr:4:9981605
4	rs28582691	9637824	A	5.288	1.93E-06	chr:4:9981605
4	rs28880963	9665259	A	5.288	1.93E-06	chr:4:9981605
4	rs6843809	9655294	G	6.245	1.97E-06	chr:4:9981605
4	rs13141264	9698240	A	2.987	2.03E-06	chr:4:9981605
4	rs56293131	9652279	G	6.149	2.10E-06	chr:4:9981605
4	rs7440232	9651744	T	6.149	2.10E-06	chr:4:9981605
4	rs116203571	10329158	A	51.49	2.13E-06	chr:4:9981605
4	rs114489981	10064751	A	5.606	2.19E-06	chr:4:9981605
4	rs115281176	10064638	C	5.606	2.19E-06	chr:4:9981605
4	rs56741911	9826492	A	16.27	2.25E-06	chr:4:9981605
4	rs60026409	9826847	C	16.27	2.25E-06	chr:4:9981605
4	rs73804391	9829784	A	16.27	2.25E-06	chr:4:9981605
4	rs6814282	9692516	T	0.2185	2.27E-06	chr:4:9981605
4	rs116613188	9634463	A	5.217	2.27E-06	chr:4:9981605
4	rs74659337	9895235	G	8.814	2.30E-06	chr:4:9981605
4	rs76065252	10405534	T	4.151	2.34E-06	chr:4:9981605
4	rs9684173	9691519	A	0.2192	2.37E-06	chr:4:9981605
4	rs10002859	9653384	T	5.115	2.56E-06	chr:4:9981605
4	rs143078048	9657322	T	5.115	2.56E-06	chr:4:9981605
4	rs28461318	9653361	A	5.115	2.56E-06	chr:4:9981605
4	rs12510091	9708661	A	3.039	2.61E-06	chr:4:9981605
4	rs77233756	9837438	T	15.94	2.67E-06	chr:4:9981605
4	rs13151395	9709144	T	3.026	2.89E-06	chr:4:9981605
4	rs116348943	9852618	T	13.67	3.13E-06	chr:4:9981605
4	rs73804390	9828756	G	13.67	3.13E-06	chr:4:9981605
4	rs73804392	9830280	T	13.67	3.13E-06	chr:4:9981605
4	rs73804734	9834978	G	13.67	3.13E-06	chr:4:9981605
4	rs73804735	9838790	C	13.67	3.13E-06	chr:4:9981605
4	rs73804738	9843552	A	13.67	3.13E-06	chr:4:9981605
4	rs116612404	9748398	G	6.347	3.14E-06	chr:4:9981605
4	rs145703344	9753072	A	6.347	3.14E-06	chr:4:9981605
4	rs150571547	9746210	T	6.347	3.14E-06	chr:4:9981605
4	rs150725431	9632263	T	4.454	3.19E-06	chr:4:9981605
4	rs7698535	9744864	A	6.322	3.28E-06	chr:4:9981605
4	rs10024256	9523614	G	4.776	3.36E-06	chr:4:9981605
4	rs6839302	9884324	C	2.888	3.39E-06	chr:4:9981605
4	rs139968201	9590506	C	24.88	3.42E-06	chr:4:9981605

4	rs142964081	9668827	A	24.88	3.42E-06	chr:4:9981605
4	rs139343792	9572589	C	4.882	3.52E-06	chr:4:9981605
4	rs28379551	9583509	C	4.882	3.52E-06	chr:4:9981605
4	rs28569802	9587761	T	4.882	3.52E-06	chr:4:9981605
4	rs9990816	9587343	A	4.882	3.52E-06	chr:4:9981605
4	rs9998156	9886560	A	2.886	3.54E-06	chr:4:9981605
4	rs10004353	10438091	A	3.338	3.55E-06	chr:4:9981605
4	rs28552686	9488838	C	3.861	3.68E-06	chr:4:9981605
4	rs544702874	9671629	C	25.21	3.77E-06	chr:4:9981605
4	rs544881013	9674615	A	25.21	3.77E-06	chr:4:9981605
4	rs563493121	9654221	T	25.21	3.77E-06	chr:4:9981605
4	rs66534644	9711634	G	2.947	3.87E-06	chr:4:9981605
4	rs184211452	9497126	A	3.655	3.93E-06	chr:4:9981605
4	rs6447956	10428207	A	3.304	4.18E-06	chr:4:9981605
4	rs16868313	10068064	T	3.277	4.37E-06	chr:4:9981605
4	rs181305798	9755199	G	157	4.50E-06	chr:4:9981605
4	rs2867388	9796495	C	3.459	4.55E-06	chr:4:9981605
4	rs139245561	9489991	A	3.801	4.55E-06	chr:4:9981605
4	rs190560945	9489537	A	3.801	4.55E-06	chr:4:9981605
4	rs10005931	9714347	C	3.088	4.56E-06	chr:4:9981605
4	rs181141933	9577434	T	32.81	4.60E-06	chr:4:9981605
4	rs182114516	9580619	C	32.81	4.60E-06	chr:4:9981605
4	rs184957234	9644112	C	32.81	4.60E-06	chr:4:9981605
4	rs186847077	9688974	T	32.81	4.60E-06	chr:4:9981605
4	rs190891681	9610244	A	32.81	4.60E-06	chr:4:9981605
4	rs527470627	9575377	A	32.81	4.60E-06	chr:4:9981605
4	rs536266752	9682815	T	32.81	4.60E-06	chr:4:9981605
4	rs546881120	9682807	T	32.81	4.60E-06	chr:4:9981605
4	rs553986940	9643253	C	32.81	4.60E-06	chr:4:9981605
4	rs569869716	9701016	C	32.81	4.60E-06	chr:4:9981605
4	rs60106886	9814238	C	3.157	4.61E-06	chr:4:9981605
4	rs535524011	9708745	G	32.71	4.67E-06	chr:4:9981605
4	rs13136075	9714968	A	2.923	4.72E-06	chr:4:9981605
4	rs188176101	9631903	A	2.938	4.80E-06	chr:4:9981605
4	rs2867386	9795784	G	3.391	4.92E-06	chr:4:9981605
4	rs2867387	9795786	G	3.391	4.92E-06	chr:4:9981605
4	rs4543113	10008305	A	2.923	4.96E-06	chr:4:9981605
4	rs28378623	9488791	A	3.673	5.08E-06	chr:4:9981605
4	rs28526410	9488632	G	3.673	5.08E-06	chr:4:9981605
4	rs146073677	9672178	T	4.79	5.08E-06	chr:4:9981605
4	rs6843276	9714147	T	2.915	5.14E-06	chr:4:9981605
4	rs145615124	9644276	A	5.448	5.33E-06	chr:4:9981605
4	rs6842702	9626659	C	2.914	5.73E-06	chr:4:9981605

4	rs6448969	9700056	A	2.747	5.90E-06	chr:4:9981605
4	rs115299967	10081399	A	8.764	5.90E-06	chr:4:9981605
4	rs11942223	9962765	C	0.2952	5.93E-06	chr:4:9981605
4	rs138225173	9492829	A	3.601	6.46E-06	chr:4:9981605
4	rs28504038	9603671	T	4.596	6.53E-06	chr:4:9981605
4	rs6811809	9918557	A	2.793	6.71E-06	chr:4:9981605
4	rs140141642	9858536	C	51.77	6.82E-06	chr:4:9981605
4	rs145359792	9816627	A	51.77	6.82E-06	chr:4:9981605
4	rs187736581	9863971	A	51.77	6.82E-06	chr:4:9981605
4	rs187946156	9892226	T	51.77	6.82E-06	chr:4:9981605
4	rs542855663	9809871	C	51.77	6.82E-06	chr:4:9981605
4	rs150081498	9738059	T	4.495	7.24E-06	chr:4:9981605
4	rs114982594	9599260	G	4.548	7.36E-06	chr:4:9981605
4	rs7671787	10439993	C	3.269	7.39E-06	chr:4:9981605
4	rs13131257	9981889	C	0.3124	7.51E-06	chr:4:9981605
4	rs141646409	9904232	A	136.4	7.66E-06	chr:4:9981605
4	rs149796755	9914445	T	136.4	7.66E-06	chr:4:9981605
4	rs184663216	9913204	T	136.4	7.66E-06	chr:4:9981605
4	rs186349586	9899122	G	136.4	7.66E-06	chr:4:9981605
4	rs561329337	9913604	C	136.4	7.66E-06	chr:4:9981605
4	rs6449173	9966105	G	0.3017	7.87E-06	chr:4:9981605
4	rs7439210	9971749	G	0.3017	7.87E-06	chr:4:9981605
4	rs7442295	9966380	G	0.3017	7.87E-06	chr:4:9981605
4	rs115747737	9635174	A	4.733	7.87E-06	chr:4:9981605
4	rs192870338	9634856	G	4.733	7.87E-06	chr:4:9981605
4	rs74381295	9613314	C	4.733	7.87E-06	chr:4:9981605
4	rs6848533	9690289	T	0.1943	8.00E-06	chr:4:9981605
4	rs1071988	9974638	A	0.3149	8.45E-06	chr:4:9981605
4	rs11722229	9980697	A	0.3149	8.45E-06	chr:4:9981605
4	rs16868246	9978305	G	0.3149	8.45E-06	chr:4:9981605
4	rs114230286	10282863	G	8.147	8.60E-06	chr:4:9981605
4	rs114418153	10300229	T	8.147	8.60E-06	chr:4:9981605
4	rs116711448	10269790	A	8.147	8.60E-06	chr:4:9981605
4	rs140190261	10259211	C	8.147	8.60E-06	chr:4:9981605
4	rs146359800	10278742	C	8.147	8.60E-06	chr:4:9981605
4	rs79855320	10261700	C	8.147	8.60E-06	chr:4:9981605
4	rs138840439	9891695	T	126.7	9.01E-06	chr:4:9981605
4	rs140689554	9869100	A	126.7	9.01E-06	chr:4:9981605
4	rs140762984	9879233	T	126.7	9.01E-06	chr:4:9981605
4	rs141750553	9892413	A	126.7	9.01E-06	chr:4:9981605
4	rs142451746	9876410	T	126.7	9.01E-06	chr:4:9981605
4	rs143245597	9870146	C	126.7	9.01E-06	chr:4:9981605
4	rs144094429	9882600	A	126.7	9.01E-06	chr:4:9981605

4	rs144303771	9887017	T	126.7	9.01E-06	chr:4:9981605
4	rs144552028	9866346	A	126.7	9.01E-06	chr:4:9981605
4	rs146225768	9878037	T	126.7	9.01E-06	chr:4:9981605
4	rs147228890	9867790	G	126.7	9.01E-06	chr:4:9981605
4	rs148082502	9888882	A	126.7	9.01E-06	chr:4:9981605
4	rs149485235	9885828	T	126.7	9.01E-06	chr:4:9981605
4	rs150633689	9889012	C	126.7	9.01E-06	chr:4:9981605
4	rs185169758	9880838	A	126.7	9.01E-06	chr:4:9981605
4	rs186371963	9879387	C	126.7	9.01E-06	chr:4:9981605
4	rs192106108	9880818	C	126.7	9.01E-06	chr:4:9981605
4	rs115188329	10173954	T	132.2	9.03E-06	chr:4:9981605
4	rs115663056	10283083	T	132.2	9.03E-06	chr:4:9981605
4	rs116140216	10160436	G	132.2	9.03E-06	chr:4:9981605
4	rs145265906	10199082	C	132.2	9.03E-06	chr:4:9981605
4	rs140671181	9700932	A	6.173	9.46E-06	chr:4:9981605
4	rs6448959	9683927	T	3.497	1.01E-05	chr:4:9981605
4	rs139416443	9977850	A	121.7	1.02E-05	chr:4:9981605
4	rs28729253	9602291	A	4.419	1.04E-05	chr:4:9981605
4	rs13125646	9982330	A	0.3086	1.07E-05	chr:4:9981605
4	rs6448960	9686586	T	3.485	1.08E-05	chr:4:9981605
4	rs7680572	9687543	A	3.485	1.08E-05	chr:4:9981605
4	rs10433800	10433708	T	3.356	1.09E-05	chr:4:9981605
4	rs4467560	10427048	G	3.116	1.09E-05	chr:4:9981605
4	rs6838644	10252871	C	6.851	1.13E-05	chr:4:9981605
4	rs17409460	10286427	C	6.834	1.18E-05	chr:4:9981605
4	rs17475334	10287170	G	6.834	1.18E-05	chr:4:9981605
4	rs62286662	10290650	C	6.834	1.18E-05	chr:4:9981605
4	rs62288124	10287550	G	6.834	1.18E-05	chr:4:9981605
4	rs62288125	10287987	G	6.834	1.18E-05	chr:4:9981605
4	rs6834574	10300814	G	6.834	1.18E-05	chr:4:9981605
4	rs73224405	10287415	A	6.834	1.18E-05	chr:4:9981605
4	rs73224422	10303417	T	6.834	1.18E-05	chr:4:9981605
4	rs116028495	10047412	T	9.6	1.21E-05	chr:4:9981605
4	rs139032162	10388243	G	123.8	1.22E-05	chr:4:9981605
4	rs139118727	10080782	T	123.8	1.22E-05	chr:4:9981605
4	rs141488118	10122292	G	123.8	1.22E-05	chr:4:9981605
4	rs141545818	10083901	G	123.8	1.22E-05	chr:4:9981605
4	rs143690141	10126080	T	123.8	1.22E-05	chr:4:9981605
4	rs143883059	10193917	C	123.8	1.22E-05	chr:4:9981605
4	rs144039186	10167766	T	123.8	1.22E-05	chr:4:9981605
4	rs146172769	10080777	T	123.8	1.22E-05	chr:4:9981605
4	rs146567373	10274690	A	123.8	1.22E-05	chr:4:9981605
4	rs147119263	10112073	A	123.8	1.22E-05	chr:4:9981605

4	rs150149824	10351048	T	123.8	1.22E-05	chr:4:9981605
4	rs150328613	10098275	T	123.8	1.22E-05	chr:4:9981605
4	rs35313378	10089535	T	123.8	1.22E-05	chr:4:9981605
4	rs555294301	10106310	G	123.8	1.22E-05	chr:4:9981605
4	rs570462561	10171777	C	123.8	1.22E-05	chr:4:9981605
4	rs575327145	10095974	G	123.8	1.22E-05	chr:4:9981605
4	rs10008035	9999335	T	0.2837	1.38E-05	chr:4:9981605
4	rs3733587	9997434	G	0.2837	1.38E-05	chr:4:9981605
4	rs4547795	9997060	T	0.2837	1.38E-05	chr:4:9981605
4	rs7696536	10000236	T	0.2837	1.38E-05	chr:4:9981605
4	rs138724702	9632058	T	28.58	1.40E-05	chr:4:9981605
4	rs187370048	9619538	G	28.58	1.40E-05	chr:4:9981605
4	rs189459090	9626265	T	28.58	1.40E-05	chr:4:9981605
4	rs557786500	9627195	T	28.58	1.40E-05	chr:4:9981605
4	rs113997128	9728531	T	5.098	1.42E-05	chr:4:9981605
4	rs146868354	9723645	G	5.098	1.42E-05	chr:4:9981605
4	rs149630066	9740919	T	5.098	1.42E-05	chr:4:9981605
4	rs6837336	9932510	A	0.3272	1.43E-05	chr:4:9981605
5	rs7722775	4535127	A	38.8	2.44E-12	chr:5:4537604
5	rs146484535	4514639	T	74.64	1.65E-10	chr:5:4537604
5	rs146421892	4476399	T	96.57	3.47E-10	chr:5:4537604
5	rs73039356	4517661	G	17.55	7.19E-10	chr:5:4537604
5	rs138653012	4494823	T	27.26	8.92E-10	chr:5:4537604
5	rs139649600	4493454	C	27.26	8.92E-10	chr:5:4537604
5	rs182198218	4551131	A	104.1	9.39E-10	chr:5:4537604
5	rs59307706	4566661	T	104.1	9.39E-10	chr:5:4537604
5	rs141543792	4479625	A	144.5	9.39E-10	chr:5:4537604
5	rs144166982	4492103	C	144.5	9.39E-10	chr:5:4537604
5	rs151052133	4493723	C	144.5	9.39E-10	chr:5:4537604
5	rs139538106	4571719	A	164.9	1.05E-09	chr:5:4537604
5	rs77079202	4516476	A	15.43	1.22E-09	chr:5:4537604
5	rs114260245	4517351	G	15.64	1.92E-09	chr:5:4537604
5	rs16873544	4517207	T	15.64	1.92E-09	chr:5:4537604
5	rs16873547	4517314	G	15.64	1.92E-09	chr:5:4537604
5	rs16873551	4517352	C	15.64	1.92E-09	chr:5:4537604
5	rs73039355	4517412	A	15.57	2.02E-09	chr:5:4537604
5	rs921391	4515695	G	14.71	2.04E-09	chr:5:4537604
5	rs144712042	4504759	T	32.44	2.13E-09	chr:5:4537604
5	rs16873584	4533197	G	23.69	2.78E-09	chr:5:4537604
5	rs149248923	4482110	C	28.19	3.63E-09	chr:5:4537604
5	rs111880700	4565630	G	33.31	5.00E-09	chr:5:4537604
5	rs113720915	4567814	T	33.31	5.00E-09	chr:5:4537604
5	rs112424100	4463039	C	46.27	5.25E-09	chr:5:4537604

5	rs559503215	4470756	C	46.27	5.25E-09	chr:5:4537604
5	rs73035319	4453910	C	46.27	5.25E-09	chr:5:4537604
5	rs73037348	4464580	A	46.27	5.25E-09	chr:5:4537604
5	rs73037349	4465113	T	46.27	5.25E-09	chr:5:4537604
5	rs16873592	4534318	G	16.75	1.55E-08	chr:5:4537604
5	rs138768507	4548011	C	667.2	2.14E-08	chr:5:4537604
5	rs145671511	4549537	C	667.2	2.14E-08	chr:5:4537604
5	rs544972940	4549560	G	667.2	2.14E-08	chr:5:4537604
5	rs569731758	4564002	T	667.2	2.14E-08	chr:5:4537604
5	rs34338737	4537914	A	8.112	2.25E-08	chr:5:4537604
5	rs930681	4537821	A	8.112	2.25E-08	chr:5:4537604
5	rs113107730	4558326	G	21.21	3.15E-08	chr:5:4537604
5	rs16873492	4509493	C	15.76	3.55E-08	chr:5:4537604
5	rs16873570	4530191	C	9.075	3.75E-08	chr:5:4537604
5	rs16873572	4530609	T	9.075	3.75E-08	chr:5:4537604
5	rs16873573	4531250	G	9.075	3.75E-08	chr:5:4537604
5	rs16873581	4532484	G	8.988	4.26E-08	chr:5:4537604
5	rs73043042	4528737	T	8.988	4.26E-08	chr:5:4537604
5	rs73037359	4473160	T	29.82	4.63E-08	chr:5:4537604
5	rs6861833	4515052	T	9.852	5.67E-08	chr:5:4537604
5	rs114402363	4522350	C	14.18	9.56E-08	chr:5:4537604
5	rs1897625	4467952	T	10.32	1.28E-07	chr:5:4537604
5	rs542306089	4554998	T	9.826	1.86E-07	chr:5:4537604
5	rs560585512	4554999	T	9.826	1.86E-07	chr:5:4537604
5	rs139338078	4675339	T	60.23	2.28E-07	chr:5:4537604
5	rs112379326	4555701	C	17.55	3.35E-07	chr:5:4537604
5	rs79019298	4533927	C	9.327	3.44E-07	chr:5:4537604
5	rs921392	4515553	C	8.491	3.96E-07	chr:5:4537604
5	rs112295341	4550276	T	23.82	5.36E-07	chr:5:4537604
5	rs75109680	4545782	C	27.3	5.98E-07	chr:5:4537604
5	rs147209721	4639365	T	80.95	6.10E-07	chr:5:4537604
5	rs144120378	5037511	T	23.99	7.56E-07	chr:5:4537604
5	rs138591903	5014686	G	38.59	1.08E-06	chr:5:4537604
5	rs139046999	5015324	T	38.59	1.08E-06	chr:5:4537604
5	rs139155662	5018264	C	38.59	1.08E-06	chr:5:4537604
5	rs139179575	5016665	T	38.59	1.08E-06	chr:5:4537604
5	rs140931123	5011709	A	38.59	1.08E-06	chr:5:4537604
5	rs141058381	5018999	C	38.59	1.08E-06	chr:5:4537604
5	rs142008226	5014883	T	38.59	1.08E-06	chr:5:4537604
5	rs142908515	5006256	A	38.59	1.08E-06	chr:5:4537604
5	rs143350759	5012985	G	38.59	1.08E-06	chr:5:4537604
5	rs144332423	5008884	A	38.59	1.08E-06	chr:5:4537604
5	rs145457117	5012490	T	38.59	1.08E-06	chr:5:4537604

5	rs145882382	5006625	A	38.59	1.08E-06	chr:5:4537604
5	rs145976967	5015598	T	38.59	1.08E-06	chr:5:4537604
5	rs146098924	5003253	G	38.59	1.08E-06	chr:5:4537604
5	rs146724599	5016224	T	38.59	1.08E-06	chr:5:4537604
5	rs149429735	5019214	C	38.59	1.08E-06	chr:5:4537604
5	rs369284119	5018551	T	38.59	1.08E-06	chr:5:4537604
5	rs373574890	5016756	G	38.59	1.08E-06	chr:5:4537604
5	rs73737852	4417914	C	23.81	1.67E-06	chr:5:4537604
5	rs138041255	4535737	T	272.9	2.03E-06	chr:5:4537604
5	rs140027317	4541442	G	292.6	2.73E-06	chr:5:4537604
5	rs76899300	4520100	A	9.749	3.14E-06	chr:5:4537604
5	rs144086962	5034018	C	14.67	3.43E-06	chr:5:4537604
5	rs138704568	4540505	A	41.66	3.48E-06	chr:5:4537604
5	rs141919616	4540076	A	41.66	3.48E-06	chr:5:4537604
5	rs139032928	4529123	A	63.83	3.61E-06	chr:5:4537604
5	rs150098946	4529718	A	63.83	3.61E-06	chr:5:4537604
5	rs539219236	4532020	T	63.83	3.61E-06	chr:5:4537604
5	rs554334358	4532021	G	63.83	3.61E-06	chr:5:4537604
5	rs6555295	4514732	A	8.694	4.20E-06	chr:5:4537604
5	rs73039400	4526380	C	6.544	4.93E-06	chr:5:4537604
5	rs73039402	4526401	G	6.544	4.93E-06	chr:5:4537604
5	rs75702524	4526348	G	6.544	4.93E-06	chr:5:4537604
5	rs7730143	4513357	A	8.494	5.29E-06	chr:5:4537604
5	rs6862133	4515370	A	5.9	5.37E-06	chr:5:4537604
5	rs570393286	4526155	A	6.454	5.39E-06	chr:5:4537604
5	rs7709410	4537293	G	4.704	5.76E-06	chr:5:4537604
5	rs73043054	4532584	A	9.008	5.77E-06	chr:5:4537604
5	rs6894984	4907084	A	5.602	5.78E-06	chr:5:4537604
5	rs7712214	4514243	C	8.281	6.51E-06	chr:5:4537604
5	rs9313085	4489021	T	4.9	6.74E-06	chr:5:4537604
5	rs112660579	4523769	C	8.669	7.22E-06	chr:5:4537604
5	rs112716024	4528763	T	8.669	7.22E-06	chr:5:4537604
5	rs73039388	4523923	T	8.669	7.22E-06	chr:5:4537604
5	rs73039391	4523979	A	8.669	7.22E-06	chr:5:4537604
5	rs73043055	4533215	C	8.669	7.22E-06	chr:5:4537604
5	rs16873575	4532072	T	8.666	7.23E-06	chr:5:4537604
5	rs112044989	4525870	A	6.31	7.52E-06	chr:5:4537604
5	rs113112159	4525949	C	6.31	7.52E-06	chr:5:4537604
5	rs73039394	4525290	C	6.31	7.52E-06	chr:5:4537604
5	rs73039395	4525365	A	6.31	7.52E-06	chr:5:4537604
5	rs73041207	4526541	A	6.31	7.52E-06	chr:5:4537604
5	rs671030	4941410	G	31.7	7.62E-06	chr:5:4537604
5	rs115255006	4520066	A	8.6	8.36E-06	chr:5:4537604

5	rs73039360	4520105	A	8.6	8.36E-06	chr:5:4537604
5	rs73039362	4520146	T	8.6	8.36E-06	chr:5:4537604
5	rs141292695	4637983	C	13.01	1.06E-05	chr:5:4537604
5	rs73737853	4421310	A	11.52	1.09E-05	chr:5:4537604
5	rs6555315	4750666	T	5.172	1.12E-05	chr:5:4537604
5	rs7710050	4750730	G	5.172	1.12E-05	chr:5:4537604
5	rs7727795	4751073	C	5.172	1.12E-05	chr:5:4537604
5	rs111505481	4520715	A	8.244	1.12E-05	chr:5:4537604
5	rs112048759	4521305	G	8.244	1.12E-05	chr:5:4537604
5	rs112475023	4521281	G	8.244	1.12E-05	chr:5:4537604
5	rs113871707	4522075	C	8.244	1.12E-05	chr:5:4537604
5	rs73039364	4520275	T	8.244	1.12E-05	chr:5:4537604
5	rs73039365	4520288	C	8.244	1.12E-05	chr:5:4537604
5	rs73039366	4520444	G	8.244	1.12E-05	chr:5:4537604
5	rs73039370	4520710	C	8.244	1.12E-05	chr:5:4537604
5	rs73039371	4520797	C	8.244	1.12E-05	chr:5:4537604
5	rs73039376	4523237	A	8.244	1.12E-05	chr:5:4537604
5	rs73039380	4523327	C	8.244	1.12E-05	chr:5:4537604
5	rs73039382	4523362	T	8.244	1.12E-05	chr:5:4537604
5	rs78794518	4522827	T	8.244	1.12E-05	chr:5:4537604
5	rs111513588	4749648	C	5.157	1.19E-05	chr:5:4537604
5	rs112815273	4740927	T	5.157	1.19E-05	chr:5:4537604
5	rs113758744	4740653	T	5.157	1.19E-05	chr:5:4537604
5	rs116242275	4740897	A	5.157	1.19E-05	chr:5:4537604
5	rs16873996	4740551	A	5.157	1.19E-05	chr:5:4537604
5	rs6870892	4738172	C	5.157	1.19E-05	chr:5:4537604
5	rs6875309	4738400	C	5.157	1.19E-05	chr:5:4537604
5	rs6875828	4738666	C	5.157	1.19E-05	chr:5:4537604
5	rs6876688	4739386	G	5.157	1.19E-05	chr:5:4537604
5	rs6890678	4738540	T	5.157	1.19E-05	chr:5:4537604
5	rs6891595	4739059	A	5.157	1.19E-05	chr:5:4537604
5	rs6894981	4739336	A	5.157	1.19E-05	chr:5:4537604
5	rs6896324	4739385	T	5.157	1.19E-05	chr:5:4537604
5	rs75313462	4743171	G	5.157	1.19E-05	chr:5:4537604
5	rs76871683	4742976	G	5.157	1.19E-05	chr:5:4537604
5	rs78391862	4741034	G	5.157	1.19E-05	chr:5:4537604
5	rs293089	4561821	C	4.5	1.21E-05	chr:5:4537604
5	rs78751262	4737980	A	5.141	1.27E-05	chr:5:4537604
5	rs6871795	4738913	G	5.123	1.32E-05	chr:5:4537604
5	rs142072081	4653080	C	165.4	1.33E-05	chr:5:4537604
5	rs293100	4571817	C	4.485	1.34E-05	chr:5:4537604
5	rs73737857	4429097	A	6.235	1.37E-05	chr:5:4537604
5	rs139568198	4919340	T	23.19	1.38E-05	chr:5:4537604

5	rs184123477	4926596	T	23.19	1.38E-05	chr:5:4537604
5	rs77244417	4432654	A	11.12	1.42E-05	chr:5:4537604
5	rs7707506	4535291	C	9.154	1.66E-05	chr:5:4537604
5	rs111603903	4965417	C	10.48	1.66E-05	chr:5:4537604
5	rs112546024	4974965	A	10.48	1.66E-05	chr:5:4537604
5	rs542980736	4866361	T	54.77	1.68E-05	chr:5:4537604
5	rs1450820	4544462	A	4.395	1.80E-05	chr:5:4537604
5	rs75956977	4746187	T	5.203	1.87E-05	chr:5:4537604
5	rs144689697	4825503	C	58.77	1.87E-05	chr:5:4537604
5	rs190013092	4816418	T	58.77	1.87E-05	chr:5:4537604
5	rs7728048	4487664	C	8.503	1.92E-05	chr:5:4537604
5	rs111562111	4555434	G	11.93	2.22E-05	chr:5:4537604
5	rs184212488	4995779	A	10.8	2.25E-05	chr:5:4537604
5	rs142421101	4496287	A	8.276	2.29E-05	chr:5:4537604
5	rs17678610	4509088	C	8.276	2.29E-05	chr:5:4537604
5	rs4701907	4503738	A	8.276	2.29E-05	chr:5:4537604
5	rs62343516	4495407	A	8.276	2.29E-05	chr:5:4537604
5	rs973156	4505421	C	8.276	2.29E-05	chr:5:4537604
5	rs4701877	4455368	C	8.538	2.80E-05	chr:5:4537604
5	rs145552461	4909599	A	9.593	2.89E-05	chr:5:4537604
5	rs75139273	5031595	G	10.03	3.18E-05	chr:5:4537604
5	rs7709922	4483433	C	7.832	3.63E-05	chr:5:4537604
5	rs6898706	64415987	A	87.02	5.02E-33	chr:5:64388440
5	rs73103441	64421362	G	87.02	5.02E-33	chr:5:64388440
5	rs7729539	64417678	C	87.02	5.02E-33	chr:5:64388440
5	rs55853058	64436298	T	37.71	8.60E-33	chr:5:64388440
5	rs56246735	64434968	A	37.71	8.60E-33	chr:5:64388440
5	rs67024543	64439173	C	37.71	8.60E-33	chr:5:64388440
5	rs72758879	64437191	G	37.71	8.60E-33	chr:5:64388440
5	rs79124463	64437182	T	36.35	1.19E-32	chr:5:64388440
5	rs73095600	64373529	C	27.38	3.59E-31	chr:5:64388440
5	rs6865147	64395387	A	310.6	2.50E-30	chr:5:64388440
5	rs78329642	64401237	A	48.36	2.77E-29	chr:5:64388440
5	rs73103448	64427080	C	75.13	8.04E-29	chr:5:64388440
5	rs73101500	64401736	T	167.7	9.90E-29	chr:5:64388440
5	rs73097406	64377619	T	382.6	1.34E-28	chr:5:64388440
5	rs73097412	64383581	G	359.6	2.53E-28	chr:5:64388440
5	rs75867121	64439878	A	21.08	5.07E-28	chr:5:64388440
5	rs7710434	64388099	A	1594	3.47E-26	chr:5:64388440
5	rs61619383	64453265	T	18.76	4.02E-26	chr:5:64388440
5	rs371938786	64460027	A	16.03	9.89E-26	chr:5:64388440
5	rs73103483	64459559	T	16.03	9.89E-26	chr:5:64388440
5	rs7710427	64388091	T	1539	1.15E-25	chr:5:64388440

5	rs140224833	64474892	T	14.57	3.29E-24	chr:5:64388440
5	rs264757	64380583	T	14.12	5.35E-24	chr:5:64388440
5	rs264760	64382737	A	12.75	7.74E-23	chr:5:64388440
5	rs73105104	64479565	G	12.58	1.16E-22	chr:5:64388440
5	rs264770	64391134	T	19.34	2.17E-22	chr:5:64388440
5	rs264775	64395359	G	21.07	2.65E-22	chr:5:64388440
5	rs4700662	64392906	C	20.8	2.76E-22	chr:5:64388440
5	rs190543	64391933	G	20.91	2.96E-22	chr:5:64388440
5	rs521074	64392198	C	20.91	2.96E-22	chr:5:64388440
5	rs111528700	64320024	T	16.85	3.44E-22	chr:5:64388440
5	rs73097416	64385303	T	205.8	5.79E-22	chr:5:64388440
5	rs65407	64375128	C	15.03	9.96E-21	chr:5:64388440
5	rs264752	64375502	T	23.99	1.92E-20	chr:5:64388440
5	rs155337	64375371	A	23.92	2.19E-20	chr:5:64388440
5	rs197138	64376615	G	23.92	2.19E-20	chr:5:64388440
5	rs264753	64375657	T	23.92	2.19E-20	chr:5:64388440
5	rs264754	64377290	A	23.92	2.19E-20	chr:5:64388440
5	rs264756	64380395	G	23.92	2.19E-20	chr:5:64388440
5	rs264762	64384386	A	23.92	2.19E-20	chr:5:64388440
5	rs264763	64384431	C	23.92	2.19E-20	chr:5:64388440
5	rs264766	64388979	T	23.92	2.19E-20	chr:5:64388440
5	rs73095522	64362357	G	164.6	2.96E-20	chr:5:64388440
5	rs73109219	64563151	A	11.68	1.63E-19	chr:5:64388440
5	rs533184079	64571243	G	20.81	2.93E-19	chr:5:64388440
5	rs73103471	64454874	T	7.406	3.75E-19	chr:5:64388440
5	rs73103478	64457644	C	7.792	4.25E-19	chr:5:64388440
5	rs73095585	64365707	T	138.4	6.10E-19	chr:5:64388440
5	rs73103472	64454906	T	7.334	6.15E-19	chr:5:64388440
5	rs144677474	64470809	T	7.164	1.66E-18	chr:5:64388440
5	rs6875154	64471415	A	7.164	1.66E-18	chr:5:64388440
5	rs73103487	64470165	T	7.164	1.66E-18	chr:5:64388440
5	rs11742800	64403371	T	6.573	1.83E-18	chr:5:64388440
5	rs73103481	64458204	T	7.122	2.52E-18	chr:5:64388440
5	rs111418391	64716056	G	15.84	3.05E-18	chr:5:64388440
5	rs112593123	64708584	T	15.84	3.05E-18	chr:5:64388440
5	rs73111166	64721188	T	15.84	3.05E-18	chr:5:64388440
5	rs7736365	64413591	C	7.082	3.58E-18	chr:5:64388440
5	rs73093236	64761742	G	15.24	4.23E-18	chr:5:64388440
5	rs192129646	64319677	A	14.29	5.59E-18	chr:5:64388440
5	rs58905231	64335255	A	14.29	5.59E-18	chr:5:64388440
5	rs73093175	64339354	A	14.29	5.59E-18	chr:5:64388440
5	rs73102795	64316619	G	14.29	5.59E-18	chr:5:64388440
5	rs73102800	64317492	A	14.29	5.59E-18	chr:5:64388440

5	rs73104706	64319024	C	14.29	5.59E-18	chr:5:64388440
5	rs73104721	64322896	T	14.29	5.59E-18	chr:5:64388440
5	rs73104725	64326735	A	14.29	5.59E-18	chr:5:64388440
5	rs73104729	64329399	A	14.29	5.59E-18	chr:5:64388440
5	rs73104735	64331054	C	14.29	5.59E-18	chr:5:64388440
5	rs73104738	64331990	G	14.29	5.59E-18	chr:5:64388440
5	rs73104740	64333955	T	14.29	5.59E-18	chr:5:64388440
5	rs73104745	64337158	G	14.29	5.59E-18	chr:5:64388440
5	rs74998301	64318760	A	14.29	5.59E-18	chr:5:64388440
5	rs73093170	64338376	A	13.21	1.06E-17	chr:5:64388440
5	rs111294603	64475047	T	6.801	1.38E-17	chr:5:64388440
5	rs264774	64394535	G	18.34	1.57E-17	chr:5:64388440
5	rs73111136	64690636	C	11.18	2.25E-17	chr:5:64388440
5	rs73093219	64751698	C	11.71	5.51E-17	chr:5:64388440
5	rs264758	64381433	A	5.333	7.56E-17	chr:5:64388440
5	rs264765	64388548	C	5.324	8.69E-17	chr:5:64388440
5	rs6870898	64396495	C	5.442	9.80E-17	chr:5:64388440
5	rs6890401	64396791	T	5.442	9.80E-17	chr:5:64388440
5	rs73101485	64398297	G	5.442	9.80E-17	chr:5:64388440
5	rs113981089	64404264	A	5.369	1.11E-16	chr:5:64388440
5	rs6870195	64405273	G	5.369	1.11E-16	chr:5:64388440
5	rs6876719	64406481	T	5.369	1.11E-16	chr:5:64388440
5	rs73104743	64336890	C	8.856	1.68E-16	chr:5:64388440
5	rs264778	64395979	A	5.708	1.93E-16	chr:5:64388440
5	rs111398089	64686368	A	6.996	4.03E-16	chr:5:64388440
5	rs111792241	64691133	A	6.996	4.03E-16	chr:5:64388440
5	rs112491888	64684807	A	6.996	4.03E-16	chr:5:64388440
5	rs73111131	64688247	G	6.996	4.03E-16	chr:5:64388440
5	rs73111133	64690144	G	6.996	4.03E-16	chr:5:64388440
5	rs73111141	64692779	C	6.996	4.03E-16	chr:5:64388440
5	rs113193051	64410219	T	5.161	4.24E-16	chr:5:64388440
5	rs6449771	64425328	A	5.126	5.51E-16	chr:5:64388440
5	rs6883161	64407936	A	5.126	5.51E-16	chr:5:64388440
5	rs73103420	64409881	A	5.126	5.51E-16	chr:5:64388440
5	rs73103440	64420014	T	5.126	5.51E-16	chr:5:64388440
5	rs73103445	64426837	T	5.126	5.51E-16	chr:5:64388440
5	rs7716646	64413666	C	5.126	5.51E-16	chr:5:64388440
5	rs73111144	64694191	C	6.858	5.88E-16	chr:5:64388440
5	rs7716553	64397419	C	4.937	8.04E-16	chr:5:64388440
5	rs16893403	64421920	T	5.064	8.73E-16	chr:5:64388440
5	rs264779	64398181	C	4.956	1.20E-15	chr:5:64388440
5	rs7733210	64492383	A	6.192	1.48E-15	chr:5:64388440
5	rs111512786	64410024	G	4.765	3.67E-15	chr:5:64388440

5	rs62370998	64322176	C	5.526	5.38E-15	chr:5:64388440
5	rs112112811	64302520	A	33.24	6.26E-15	chr:5:64388440
5	rs73093027	64283680	A	33.24	6.26E-15	chr:5:64388440
5	rs73093068	64293956	G	33.24	6.26E-15	chr:5:64388440
5	rs76208888	64300951	A	33.24	6.26E-15	chr:5:64388440
5	rs397044	64391972	C	4.566	6.97E-15	chr:5:64388440
5	rs111758288	64403718	A	5.11	1.15E-14	chr:5:64388440
5	rs73109580	64256585	G	31.12	2.13E-14	chr:5:64388440
5	rs73109582	64257492	G	31.12	2.13E-14	chr:5:64388440
5	rs73093221	64752599	G	6.857	1.01E-13	chr:5:64388440
5	rs58241836	64403328	C	4.033	1.08E-13	chr:5:64388440
5	rs6873085	64400885	G	4.033	1.08E-13	chr:5:64388440
5	rs12152909	64362074	C	3.993	6.12E-13	chr:5:64388440
5	rs7702739	64368803	T	3.993	6.12E-13	chr:5:64388440
5	rs111639834	64751882	C	6.041	7.75E-13	chr:5:64388440
5	rs77697244	64331684	C	4.432	9.75E-13	chr:5:64388440
5	rs441077	64356759	C	3.546	1.86E-12	chr:5:64388440
5	rs7713874	64330265	G	4.213	2.19E-12	chr:5:64388440
5	rs73097124	64874720	G	12.85	5.33E-12	chr:5:64388440
5	rs73097133	64878551	G	12.85	5.33E-12	chr:5:64388440
5	rs57076602	64310759	C	5.397	5.83E-12	chr:5:64388440
5	rs6867287	64312009	T	5.397	5.83E-12	chr:5:64388440
5	rs6889690	64308363	C	5.397	5.83E-12	chr:5:64388440
5	rs577544074	64526194	G	4.516	8.64E-12	chr:5:64388440
5	rs264739	64366053	C	3.383	9.92E-12	chr:5:64388440
5	rs264743	64368525	G	3.383	9.92E-12	chr:5:64388440
5	rs264745	64370547	G	3.383	9.92E-12	chr:5:64388440
5	rs264748	64371017	G	3.383	9.92E-12	chr:5:64388440
5	rs264749	64371183	C	3.377	1.21E-11	chr:5:64388440
5	rs264738	64363816	G	3.352	1.26E-11	chr:5:64388440
5	rs57237698	64790316	C	10.62	1.31E-11	chr:5:64388440
5	rs57788107	64790432	C	10.62	1.31E-11	chr:5:64388440
5	rs73107301	64239339	T	15.34	2.10E-11	chr:5:64388440
5	rs73094913	64826891	G	9.929	2.23E-11	chr:5:64388440
5	rs10940015	64318142	G	3.849	2.93E-11	chr:5:64388440
5	rs61571832	64318906	T	3.849	2.93E-11	chr:5:64388440
5	rs62370999	64322395	A	3.849	2.93E-11	chr:5:64388440
5	rs6882488	64319949	G	3.849	2.93E-11	chr:5:64388440
5	rs78822923	64359878	G	3.452	2.98E-11	chr:5:64388440
5	rs73094910	64822581	A	9.73	3.07E-11	chr:5:64388440
5	rs35841260	64360517	T	3.255	3.10E-11	chr:5:64388440
5	rs111889269	64091293	A	13.78	3.12E-11	chr:5:64388440
5	rs60609925	64217208	C	13.78	3.12E-11	chr:5:64388440

5	rs6891515	64120706	T	13.78	3.12E-11	chr:5:64388440
5	rs73101363	64140059	A	13.78	3.12E-11	chr:5:64388440
5	rs73107206	64183606	T	13.78	3.12E-11	chr:5:64388440
5	rs73107280	64222633	C	13.78	3.12E-11	chr:5:64388440
5	rs11749451	64675233	C	4.998	3.17E-11	chr:5:64388440
5	rs11741552	64473033	G	3.363	3.23E-11	chr:5:64388440
5	rs73093173	64338468	A	4.354	3.38E-11	chr:5:64388440
5	rs438698	64358052	A	3.226	3.58E-11	chr:5:64388440
5	rs264734	64361949	G	3.223	3.85E-11	chr:5:64388440
5	rs266568	64358639	G	3.223	3.85E-11	chr:5:64388440
5	rs266569	64358540	A	3.223	3.85E-11	chr:5:64388440
5	rs403958	64357631	C	3.223	3.85E-11	chr:5:64388440
5	rs439327	64356942	T	3.177	5.43E-11	chr:5:64388440
5	rs266571	64358152	A	3.134	1.06E-10	chr:5:64388440
5	rs11749829	64467554	T	3.188	1.29E-10	chr:5:64388440
5	rs113576877	64850257	A	8.057	1.36E-10	chr:5:64388440
5	rs113654962	64857097	A	8.057	1.36E-10	chr:5:64388440
5	rs73097110	64852719	T	8.057	1.36E-10	chr:5:64388440
5	rs150630356	64384299	C	26.81	1.79E-10	chr:5:64388440
5	rs77079494	64345577	T	3.211	1.81E-10	chr:5:64388440
5	rs114436292	64385381	A	25.09	4.25E-10	chr:5:64388440
5	rs115073561	64377414	A	25.09	4.25E-10	chr:5:64388440
5	rs11743242	64375379	C	25.09	4.25E-10	chr:5:64388440
5	rs11746094	64387612	T	25.09	4.25E-10	chr:5:64388440
5	rs11746360	64375515	G	25.09	4.25E-10	chr:5:64388440
5	rs77434451	64380180	C	25.09	4.25E-10	chr:5:64388440
5	rs77669131	64385985	C	25.09	4.25E-10	chr:5:64388440
5	rs77982418	64383897	C	25.09	4.25E-10	chr:5:64388440
5	rs11741777	64377897	T	25.04	4.34E-10	chr:5:64388440
5	rs11743343	64467626	T	3.045	4.69E-10	chr:5:64388440
5	rs11747084	64501304	C	3.462	6.14E-10	chr:5:64388440
5	rs111682017	64090207	A	4.988	1.84E-09	chr:5:64388440
5	rs2194218	64347662	C	2.712	2.00E-09	chr:5:64388440
5	rs369965566	64398886	T	16.82	2.34E-09	chr:5:64388440
5	rs11953426	64497837	T	3.46	2.72E-09	chr:5:64388440
5	rs11959921	64497937	G	3.46	2.72E-09	chr:5:64388440
5	rs60924651	64519047	G	3.46	2.86E-09	chr:5:64388440
5	rs139126213	64355569	T	18.07	2.88E-09	chr:5:64388440
5	rs75261655	64354080	G	18.07	2.88E-09	chr:5:64388440
5	rs10940019	64501468	T	3.426	3.49E-09	chr:5:64388440
5	rs62369565	64499949	G	3.426	3.49E-09	chr:5:64388440
5	rs7708553	64505744	A	3.426	3.49E-09	chr:5:64388440
5	rs7720673	64502727	T	3.426	3.49E-09	chr:5:64388440

5	rs7728957	64505977	G	3.426	3.49E-09	chr:5:64388440
5	rs78676550	64504629	T	3.426	3.49E-09	chr:5:64388440
5	rs56385905	64515187	T	3.357	4.12E-09	chr:5:64388440
5	rs7707398	64518254	T	3.357	4.12E-09	chr:5:64388440
5	rs62369571	64514734	C	3.392	4.50E-09	chr:5:64388440
5	rs73105135	64514187	G	3.392	4.50E-09	chr:5:64388440
5	rs7714577	64330480	C	3.096	4.92E-09	chr:5:64388440
5	rs10940018	64496326	T	3.326	4.92E-09	chr:5:64388440
5	rs182061015	64380725	A	20.42	6.29E-09	chr:5:64388440
5	rs192546362	64380724	C	20.42	6.29E-09	chr:5:64388440
5	rs11958777	64509115	C	3.253	7.12E-09	chr:5:64388440
5	rs73101344	64122953	G	4.733	7.42E-09	chr:5:64388440
5	rs59929214	64065184	A	6.562	1.72E-08	chr:5:64388440
5	rs113164834	64233211	G	6.198	2.28E-08	chr:5:64388440
5	rs11738990	64487371	A	3.105	2.55E-08	chr:5:64388440
5	rs58631792	63968421	C	5.854	4.43E-08	chr:5:64388440
5	rs434882	64353312	G	0.3763	4.44E-08	chr:5:64388440
5	rs10065275	64380353	T	0.3413	5.02E-08	chr:5:64388440
5	rs62368036	64486143	C	3.025	5.07E-08	chr:5:64388440
5	rs17206232	64433400	A	14.19	7.51E-08	chr:5:64388440
5	rs4700668	64435732	A	14.19	7.51E-08	chr:5:64388440
5	rs55762587	64436355	C	14.19	7.51E-08	chr:5:64388440
5	rs72758880	64440519	A	14.19	7.51E-08	chr:5:64388440
5	rs7718684	64371841	G	2.503	8.24E-08	chr:5:64388440
5	rs264750	64373264	T	0.3566	9.14E-08	chr:5:64388440
5	rs400088	64355340	C	0.3678	9.28E-08	chr:5:64388440
5	rs423139	64355873	T	0.3678	9.28E-08	chr:5:64388440
5	rs60464373	64366609	G	2.477	1.03E-07	chr:5:64388440
5	rs1423352	64450308	A	2.436	1.07E-07	chr:5:64388440
5	rs1117707	64389665	G	0.3394	1.09E-07	chr:5:64388440
5	rs10434530	64376348	C	0.3396	1.11E-07	chr:5:64388440
5	rs11746271	64522124	C	2.76	1.32E-07	chr:5:64388440
5	rs6884602	64529521	C	2.738	1.64E-07	chr:5:64388440
5	rs7735639	64337793	T	0.3998	1.86E-07	chr:5:64388440
5	rs12656072	64535627	C	2.694	1.96E-07	chr:5:64388440
5	rs112645559	64051127	C	5.199	2.18E-07	chr:5:64388440
5	rs73097700	64051869	T	5.199	2.18E-07	chr:5:64388440
5	rs73099505	64055838	G	5.199	2.18E-07	chr:5:64388440
5	rs398407	64357523	C	0.3387	2.19E-07	chr:5:64388440
5	rs111451874	64231443	A	4.846	2.31E-07	chr:5:64388440
5	rs111565859	64135412	A	4.846	2.31E-07	chr:5:64388440
5	rs111595032	64125848	T	4.846	2.31E-07	chr:5:64388440
5	rs111833618	64224034	T	4.846	2.31E-07	chr:5:64388440

5	rs111852072	64207658	C	4.846	2.31E-07	chr:5:64388440
5	rs112142048	64155007	T	4.846	2.31E-07	chr:5:64388440
5	rs112720600	64227271	G	4.846	2.31E-07	chr:5:64388440
5	rs116662026	64164730	A	4.846	2.31E-07	chr:5:64388440
5	rs116756607	64164729	T	4.846	2.31E-07	chr:5:64388440
5	rs149392464	64230924	G	4.846	2.31E-07	chr:5:64388440
5	rs57519034	64085801	G	4.846	2.31E-07	chr:5:64388440
5	rs57625412	64188178	T	4.846	2.31E-07	chr:5:64388440
5	rs59674870	64125266	G	4.846	2.31E-07	chr:5:64388440
5	rs73101310	64094856	G	4.846	2.31E-07	chr:5:64388440
5	rs73101321	64099565	C	4.846	2.31E-07	chr:5:64388440
5	rs73101347	64124354	T	4.846	2.31E-07	chr:5:64388440
5	rs73101359	64135506	T	4.846	2.31E-07	chr:5:64388440
5	rs73103381	64143471	G	4.846	2.31E-07	chr:5:64388440
5	rs73103385	64148814	G	4.846	2.31E-07	chr:5:64388440
5	rs73103391	64151470	T	4.846	2.31E-07	chr:5:64388440
5	rs73105205	64158117	C	4.846	2.31E-07	chr:5:64388440
5	rs73105207	64160560	T	4.846	2.31E-07	chr:5:64388440
5	rs73105209	64163334	T	4.846	2.31E-07	chr:5:64388440
5	rs73105224	64168437	C	4.846	2.31E-07	chr:5:64388440
5	rs73105301	64181380	A	4.846	2.31E-07	chr:5:64388440
5	rs73107213	64186153	A	4.846	2.31E-07	chr:5:64388440
5	rs73107218	64195349	T	4.846	2.31E-07	chr:5:64388440
5	rs73107224	64197482	T	4.846	2.31E-07	chr:5:64388440
5	rs73107248	64207769	T	4.846	2.31E-07	chr:5:64388440
5	rs73107259	64211768	T	4.846	2.31E-07	chr:5:64388440
5	rs73107282	64223646	C	4.846	2.31E-07	chr:5:64388440
5	rs73107283	64224030	T	4.846	2.31E-07	chr:5:64388440
5	rs73107295	64228871	T	4.846	2.31E-07	chr:5:64388440
5	rs73107296	64229609	T	4.846	2.31E-07	chr:5:64388440
5	rs7705022	64086067	A	4.846	2.31E-07	chr:5:64388440
5	rs7714464	64124895	T	4.846	2.31E-07	chr:5:64388440
5	rs7716164	64161328	A	4.846	2.31E-07	chr:5:64388440
5	rs10514977	64373956	T	0.3565	2.40E-07	chr:5:64388440
5	rs395850	64356894	A	0.3418	2.66E-07	chr:5:64388440
5	rs16893587	64547732	A	2.32	2.75E-07	chr:5:64388440
5	rs112033424	64058256	G	4.657	3.04E-07	chr:5:64388440
5	rs112703509	64442318	A	26.96	3.05E-07	chr:5:64388440
5	rs114159386	64441782	T	26.96	3.05E-07	chr:5:64388440
5	rs150497068	64519615	T	10.13	3.38E-07	chr:5:64388440
5	rs57522743	64058911	T	4.559	3.81E-07	chr:5:64388440
5	rs11746174	64470594	C	0.426	4.24E-07	chr:5:64388440
5	rs111987601	63985624	G	4.697	4.61E-07	chr:5:64388440

5	rs73097612	63997090	T	4.697	4.61E-07	chr:5:64388440
5	rs73097629	64025457	C	4.697	4.61E-07	chr:5:64388440
5	rs3776042	64558111	C	2.625	5.01E-07	chr:5:64388440
5	rs17811610	64428506	C	25.45	5.17E-07	chr:5:64388440
5	rs7719056	64542430	A	2.262	5.38E-07	chr:5:64388440
5	rs4019318	64403833	T	0.4241	5.77E-07	chr:5:64388440
5	rs10940022	64549606	A	2.61	5.81E-07	chr:5:64388440
5	rs62369607	64553490	A	2.61	5.81E-07	chr:5:64388440
5	rs34272107	64401030	T	0.4274	6.45E-07	chr:5:64388440
5	rs4700664	64401205	G	0.4274	6.45E-07	chr:5:64388440
5	rs62369611	64558325	G	2.602	6.53E-07	chr:5:64388440
5	rs62369581	64548881	A	2.277	6.87E-07	chr:5:64388440
5	rs166473	64340353	G	2.336	7.09E-07	chr:5:64388440
5	rs2886900	64405243	T	0.4282	7.20E-07	chr:5:64388440
5	rs34202375	64405041	T	0.4282	7.20E-07	chr:5:64388440
5	rs6650949	64408372	G	0.4282	7.20E-07	chr:5:64388440
5	rs10940003	64027108	G	4.509	7.30E-07	chr:5:64388440
5	rs111731999	64010858	C	4.509	7.30E-07	chr:5:64388440
5	rs112604774	63996319	G	4.509	7.30E-07	chr:5:64388440
5	rs28460304	64011903	C	4.509	7.30E-07	chr:5:64388440
5	rs57045767	64024842	G	4.509	7.30E-07	chr:5:64388440
5	rs57517937	64024850	C	4.509	7.30E-07	chr:5:64388440
5	rs57758714	64013212	C	4.509	7.30E-07	chr:5:64388440
5	rs58202736	64010242	A	4.509	7.30E-07	chr:5:64388440
5	rs61133380	64024141	A	4.509	7.30E-07	chr:5:64388440
5	rs73097631	64025654	C	4.509	7.30E-07	chr:5:64388440
5	rs73097692	64027248	A	4.509	7.30E-07	chr:5:64388440
5	rs80216318	64028142	A	4.509	7.30E-07	chr:5:64388440
5	rs10035191	64411156	T	0.4299	7.43E-07	chr:5:64388440
5	rs10040605	64410558	T	0.4299	7.43E-07	chr:5:64388440
5	rs12187984	64411249	T	0.4299	7.43E-07	chr:5:64388440
5	rs16893246	64220712	G	3.536	7.71E-07	chr:5:64388440
5	rs59349085	64216048	A	3.536	7.71E-07	chr:5:64388440
5	rs73097633	64026118	C	4.485	8.00E-07	chr:5:64388440
5	rs12186551	64411371	G	0.4306	8.17E-07	chr:5:64388440
5	rs1035489	64424183	G	0.4306	8.23E-07	chr:5:64388440
5	rs10461501	64329894	C	0.4275	8.42E-07	chr:5:64388440
5	rs10061727	64426461	C	0.4309	8.68E-07	chr:5:64388440
5	rs111843652	64043866	G	4.413	9.08E-07	chr:5:64388440
5	rs113667536	64053681	C	4.413	9.08E-07	chr:5:64388440
5	rs1812553	64474640	T	2.446	1.04E-06	chr:5:64388440
5	rs62369608	64554899	A	2.262	1.19E-06	chr:5:64388440
5	rs62370996	64319202	C	0.4394	1.19E-06	chr:5:64388440

5	rs7701134	64542365	T	0.3977	1.24E-06	chr:5:64388440
5	rs115751157	64477426	C	23.28	1.30E-06	chr:5:64388440
5	rs140009968	64448114	C	23.28	1.30E-06	chr:5:64388440
5	rs9291831	64419511	A	0.4375	1.32E-06	chr:5:64388440
5	rs115596035	64394182	A	40.35	1.34E-06	chr:5:64388440
5	rs11750581	64392324	T	40.35	1.34E-06	chr:5:64388440
5	rs114005099	64540574	A	11.45	1.43E-06	chr:5:64388440
5	rs114736328	64536310	T	11.45	1.43E-06	chr:5:64388440
5	rs114939548	64534078	C	11.45	1.43E-06	chr:5:64388440
5	rs115360677	64534860	T	11.45	1.43E-06	chr:5:64388440
5	rs115362675	64541831	T	11.45	1.43E-06	chr:5:64388440
5	rs116147829	64547379	C	11.45	1.43E-06	chr:5:64388440
5	rs116316884	64545764	A	11.45	1.43E-06	chr:5:64388440
5	rs138022970	64536118	G	11.45	1.43E-06	chr:5:64388440
5	rs138415199	64523850	T	11.45	1.43E-06	chr:5:64388440
5	rs143018035	64523951	G	11.45	1.43E-06	chr:5:64388440
5	rs150774675	64528745	T	11.45	1.43E-06	chr:5:64388440
5	rs150897403	64543939	G	11.45	1.43E-06	chr:5:64388440
5	rs13153005	64460096	T	0.4142	1.50E-06	chr:5:64388440
5	rs11960938	64322549	A	0.4411	1.50E-06	chr:5:64388440
5	rs12653052	64544676	C	2.473	1.50E-06	chr:5:64388440
5	rs41268423	64556697	C	2.468	1.61E-06	chr:5:64388440
5	rs16893613	64552199	T	2.487	1.66E-06	chr:5:64388440
5	rs17206008	64427970	A	38.73	1.66E-06	chr:5:64388440
5	rs1862565	64457083	C	0.4518	1.70E-06	chr:5:64388440
5	rs2081115	64459861	C	0.4518	1.70E-06	chr:5:64388440
5	rs7445643	64459475	G	0.4518	1.70E-06	chr:5:64388440
5	rs1549249	64455558	C	0.4524	1.82E-06	chr:5:64388440
5	rs10940021	64542667	T	2.454	1.85E-06	chr:5:64388440
5	rs10461506	64828559	G	2.361	1.97E-06	chr:5:64388440
5	rs2367239	64459691	A	0.3991	2.20E-06	chr:5:64388440
5	rs112624903	64014536	A	4.252	2.30E-06	chr:5:64388440
5	rs16893338	64361357	T	0.4481	2.61E-06	chr:5:64388440
5	rs61174743	64464557	A	0.4515	2.61E-06	chr:5:64388440
5	rs114007286	64556450	A	21.61	2.72E-06	chr:5:64388440
5	rs114808253	64549430	G	21.61	2.72E-06	chr:5:64388440
5	rs76294348	64401590	C	36.05	2.94E-06	chr:5:64388440
5	rs79066328	64400793	T	36.05	2.94E-06	chr:5:64388440
5	rs28582519	63986449	A	4.552	2.95E-06	chr:5:64388440
5	rs56724345	64475749	C	21.76	2.96E-06	chr:5:64388440
5	rs528375954	64460151	A	21.23	3.05E-06	chr:5:64388440
5	rs6880009	64535590	A	2.129	3.26E-06	chr:5:64388440
5	rs59167602	63989036	G	3.714	3.40E-06	chr:5:64388440

5	rs245525	64761316	C	2.16	4.05E-06	chr:5:64388440
5	rs60278863	64021519	C	3.646	4.28E-06	chr:5:64388440
5	rs6889661	64522219	T	2.101	4.33E-06	chr:5:64388440
5	rs56666062	63974265	G	2.637	5.36E-06	chr:5:64388440
5	rs62369575	64519862	C	2.091	5.43E-06	chr:5:64388440
5	rs9291824	64084504	A	0.4567	5.47E-06	chr:5:64388440
5	rs10471310	64548961	T	0.389	5.78E-06	chr:5:64388440
5	rs531905188	64351431	T	33.08	5.97E-06	chr:5:64388440
5	rs562948183	64351430	G	33.08	5.97E-06	chr:5:64388440
5	rs266597	64342992	C	0.4234	7.26E-06	chr:5:64388440
5	rs11739864	64550037	C	2.024	7.72E-06	chr:5:64388440
5	rs116457699	64342570	T	8.373	7.80E-06	chr:5:64388440
5	rs1309513	64341929	C	0.4261	8.16E-06	chr:5:64388440
5	rs181752	64791876	A	2.201	9.23E-06	chr:5:64388440
5	rs245519	64792839	A	2.201	9.23E-06	chr:5:64388440
5	rs245520	64792219	A	2.201	9.23E-06	chr:5:64388440
5	rs1309512	64342040	T	0.4297	9.71E-06	chr:5:64388440
5	rs245521	64789949	G	2.187	1.09E-05	chr:5:64388440
5	rs13157797	64830790	C	2.185	1.15E-05	chr:5:64388440
5	rs66889039	64843262	T	2.185	1.15E-05	chr:5:64388440
5	rs66971305	64832439	A	2.185	1.15E-05	chr:5:64388440
5	rs150864020	64342989	T	8.96	1.22E-05	chr:5:64388440
5	rs186573598	64342180	C	8.96	1.22E-05	chr:5:64388440
5	rs6861406	64599365	T	0.3286	1.44E-05	chr:5:64388440
5	rs13170899	64848738	C	2.155	1.47E-05	chr:5:64388440
5	rs16893364	64372897	G	0.3866	1.61E-05	chr:5:64388440
5	rs1423427	64560698	A	2.146	1.74E-05	chr:5:64388440
5	rs28412757	64597775	T	0.3415	1.96E-05	chr:5:64388440
5	rs115677911	64328851	C	28.39	1.98E-05	chr:5:64388440
5	rs149269547	64327102	T	28.39	1.98E-05	chr:5:64388440
5	rs149208	64865405	A	2.144	1.99E-05	chr:5:64388440
5	rs10940017	64475275	T	0.4186	2.00E-05	chr:5:64388440
5	rs62371025	64359888	A	0.3916	2.01E-05	chr:5:64388440
5	rs371636404	64766521	A	2.215	2.01E-05	chr:5:64388440
5	rs2047071	64604810	T	0.337	2.21E-05	chr:5:64388440
5	rs10474649	80461296	A	57.71	5.20E-12	chr:5:80442266
5	rs10474027	80464374	T	30.71	3.71E-09	chr:5:80442266
5	rs10474650	80463398	C	30.71	3.71E-09	chr:5:80442266
5	rs140600790	80414322	G	88.52	9.77E-08	chr:5:80442266
5	rs575940861	80419646	A	88.52	9.77E-08	chr:5:80442266
5	rs76184782	80446151	T	17.28	2.59E-07	chr:5:80442266
5	rs144473584	80476321	C	34.96	3.36E-07	chr:5:80442266
5	rs185956472	80437592	G	392.7	9.23E-07	chr:5:80442266

5	rs539995180	80456405	T	22.47	1.73E-06	chr:5:80442266
5	rs182112055	80472411	T	20.54	3.39E-06	chr:5:80442266
5	rs6891857	80464684	G	6.859	3.64E-06	chr:5:80442266
5	rs16878472	80409407	G	6.89	9.57E-06	chr:5:80442266
5	rs76307934	80408244	C	6.89	9.57E-06	chr:5:80442266
5	rs151126633	80477390	G	14.08	1.11E-05	chr:5:80442266
5	rs13361744	80455667	G	7.059	1.29E-05	chr:5:80442266
5	rs150081879	80428497	A	191	1.43E-05	chr:5:80442266
5	rs190782674	80400793	A	9.729	2.81E-05	chr:5:80442266
5	rs114292556	80356685	T	19.18	3.35E-05	chr:5:80442266
5	rs114628076	80340625	A	19.18	3.35E-05	chr:5:80442266
5	rs115564889	80434343	A	11.74	3.85E-05	chr:5:80442266
5	rs140963806	80389585	A	8.983	4.21E-05	chr:5:80442266
5	rs7703538	80386534	G	8.983	4.21E-05	chr:5:80442266
5	rs17213955	80437241	T	6.341	4.73E-05	chr:5:80442266
5	rs145371027	80263753	A	70.68	4.99E-05	chr:5:80442266
5	rs80193320	8941404	C	0.07778	2.76E-22	chr:5:8937853
5	rs6555586	8935800	A	0.0628	6.29E-22	chr:5:8937853
5	rs13160367	8936774	A	0.05714	6.81E-22	chr:5:8937853
5	rs6861103	8928381	A	0.06194	9.76E-22	chr:5:8937853
5	rs11949503	8937804	C	0.05805	1.10E-21	chr:5:8937853
5	rs11955429	8941715	A	0.05805	1.10E-21	chr:5:8937853
5	rs12186849	8933088	C	0.05805	1.10E-21	chr:5:8937853
5	rs13157678	8931378	C	0.05805	1.10E-21	chr:5:8937853
5	rs13166892	8931212	C	0.05805	1.10E-21	chr:5:8937853
5	rs13168801	8943312	A	0.05805	1.10E-21	chr:5:8937853
5	rs13169109	8943532	C	0.05805	1.10E-21	chr:5:8937853
5	rs13170524	8934862	G	0.05805	1.10E-21	chr:5:8937853
5	rs13180198	8936074	C	0.05805	1.10E-21	chr:5:8937853
5	rs13181039	8936810	C	0.05805	1.10E-21	chr:5:8937853
5	rs13181188	8936871	G	0.05805	1.10E-21	chr:5:8937853
5	rs13185206	8943046	G	0.05805	1.10E-21	chr:5:8937853
5	rs13186994	8930953	C	0.05805	1.10E-21	chr:5:8937853
5	rs34108904	8943745	T	0.05805	1.10E-21	chr:5:8937853
5	rs35376107	8942188	G	0.05805	1.10E-21	chr:5:8937853
5	rs35951584	8943375	A	0.05805	1.10E-21	chr:5:8937853
5	rs36075104	8937389	A	0.05805	1.10E-21	chr:5:8937853
5	rs4495147	8933733	T	0.05805	1.10E-21	chr:5:8937853
5	rs4505924	8936629	A	0.05805	1.10E-21	chr:5:8937853
5	rs4526080	8941529	G	0.05805	1.10E-21	chr:5:8937853
5	rs4563593	8933794	G	0.05805	1.10E-21	chr:5:8937853
5	rs4585412	8941705	G	0.05805	1.10E-21	chr:5:8937853
5	rs6555580	8932263	C	0.05805	1.10E-21	chr:5:8937853

5	rs6555585	8935651	G	0.05805	1.10E-21	chr:5:8937853
5	rs6862593	8928550	T	0.05805	1.10E-21	chr:5:8937853
5	rs6866047	8932660	T	0.05805	1.10E-21	chr:5:8937853
5	rs6870451	8938885	G	0.05805	1.10E-21	chr:5:8937853
5	rs6870477	8938935	G	0.05805	1.10E-21	chr:5:8937853
5	rs6884096	8932523	A	0.05805	1.10E-21	chr:5:8937853
5	rs6885789	8932782	T	0.05805	1.10E-21	chr:5:8937853
5	rs6888819	8929239	C	0.05805	1.10E-21	chr:5:8937853
5	rs6894834	8930468	G	0.05805	1.10E-21	chr:5:8937853
5	rs71611354	8942703	T	0.05805	1.10E-21	chr:5:8937853
5	rs76109783	8939954	A	0.05805	1.10E-21	chr:5:8937853
5	rs7735641	8935206	A	0.05805	1.10E-21	chr:5:8937853
5	rs34251442	8942645	C	0.05821	1.24E-21	chr:5:8937853
5	rs35495516	8942635	C	0.05821	1.24E-21	chr:5:8937853
5	rs13160595	8929697	C	0.05838	1.41E-21	chr:5:8937853
5	rs13189384	8943321	G	0.0588	1.58E-21	chr:5:8937853
5	rs34626780	8943339	T	0.0588	1.58E-21	chr:5:8937853
5	rs13165792	8943070	T	0.059	1.70E-21	chr:5:8937853
5	rs2962735	8942095	G	0.07901	1.44E-20	chr:5:8937853
5	rs6555581	8932337	T	0.1199	1.83E-20	chr:5:8937853
5	rs36068583	8942585	A	0.1206	2.24E-20	chr:5:8937853
5	rs13155565	8965540	T	0.1458	2.36E-19	chr:5:8937853
5	rs7730808	8977997	T	0.1462	3.09E-19	chr:5:8937853
5	rs7730809	8977998	C	0.1462	3.09E-19	chr:5:8937853
5	rs6883368	8968144	C	0.1462	3.25E-19	chr:5:8937853
5	rs1947583	8980189	C	0.15	4.24E-19	chr:5:8937853
5	rs6876180	8977231	T	0.1511	4.55E-19	chr:5:8937853
5	rs12513755	8958879	A	0.1581	4.62E-19	chr:5:8937853
5	rs6863109	8966159	T	0.1356	4.63E-19	chr:5:8937853
5	rs10040864	8965550	A	0.1394	9.91E-19	chr:5:8937853
5	rs13355363	8960849	A	0.1394	9.91E-19	chr:5:8937853
5	rs6861946	8966209	T	0.1394	9.91E-19	chr:5:8937853
5	rs7723950	8959250	C	0.1395	1.14E-18	chr:5:8937853
5	rs7728475	8969172	A	0.1446	1.37E-18	chr:5:8937853
5	rs59676981	8968807	T	0.1446	1.44E-18	chr:5:8937853
5	rs6869638	8968467	G	0.1446	1.44E-18	chr:5:8937853
5	rs6872524	8972999	T	0.1445	1.59E-18	chr:5:8937853
5	rs7726068	8971413	G	0.1445	1.59E-18	chr:5:8937853
5	rs34352770	8970561	A	0.1487	3.30E-18	chr:5:8937853
5	rs6869307	8968310	G	0.1673	6.17E-18	chr:5:8937853
5	rs34814654	8958042	A	0.168	6.61E-18	chr:5:8937853
5	rs2963382	8948523	C	0.1679	2.07E-17	chr:5:8937853
5	rs2963363	8928893	A	0.1811	2.10E-17	chr:5:8937853

5	rs2963326	8933452	A	0.1959	1.31E-16	chr:5:8937853
5	rs2962650	8937165	G	0.2122	4.34E-16	chr:5:8937853
5	rs2963376	8942864	T	0.2122	4.64E-16	chr:5:8937853
5	rs2963327	8934159	G	0.2231	2.65E-15	chr:5:8937853
5	rs2963380	8945194	A	0.2231	2.82E-15	chr:5:8937853
5	rs2962654	8931393	A	0.2158	6.67E-15	chr:5:8937853
5	rs2963384	8950534	T	0.2297	7.71E-15	chr:5:8937853
5	rs6863266	8966162	T	0.2143	1.03E-14	chr:5:8937853
5	rs6883897	8966181	G	0.2143	1.03E-14	chr:5:8937853
5	rs6862506	8965836	T	0.2205	1.64E-14	chr:5:8937853
5	rs10038936	8965436	A	0.2208	1.97E-14	chr:5:8937853
5	rs1585204	8974023	A	0.2379	2.10E-14	chr:5:8937853
5	rs10056871	8964318	C	0.2206	2.33E-14	chr:5:8937853
5	rs10058520	8964357	C	0.2206	2.33E-14	chr:5:8937853
5	rs10512999	8963885	C	0.2206	2.33E-14	chr:5:8937853
5	rs13175700	8967537	T	0.2206	2.33E-14	chr:5:8937853
5	rs13185652	8967225	C	0.2206	2.33E-14	chr:5:8937853
5	rs1902806	8965016	C	0.2206	2.33E-14	chr:5:8937853
5	rs4431316	8964778	T	0.2206	2.33E-14	chr:5:8937853
5	rs4432854	8966865	C	0.2206	2.33E-14	chr:5:8937853
5	rs6866139	8966309	C	0.2206	2.33E-14	chr:5:8937853
5	rs6875463	8963376	C	0.2206	2.33E-14	chr:5:8937853
5	rs9313264	8961561	T	0.2206	2.33E-14	chr:5:8937853
5	rs9313265	8964069	G	0.2206	2.33E-14	chr:5:8937853
5	rs1902807	8964963	A	0.2206	2.45E-14	chr:5:8937853
5	rs1902808	8964937	T	0.2206	2.45E-14	chr:5:8937853
5	rs4624764	8958031	T	0.2207	2.48E-14	chr:5:8937853
5	rs12521690	8967716	A	0.2251	3.50E-14	chr:5:8937853
5	rs2388006	8968700	C	0.2251	3.50E-14	chr:5:8937853
5	rs2388007	8968699	C	0.2251	3.50E-14	chr:5:8937853
5	rs2388008	8968479	C	0.2251	3.50E-14	chr:5:8937853
5	rs34958715	8970889	G	0.2251	3.50E-14	chr:5:8937853
5	rs58854317	8968867	C	0.2251	3.50E-14	chr:5:8937853
5	rs6555587	8969296	G	0.2251	3.50E-14	chr:5:8937853
5	rs6880066	8969679	C	0.2251	3.50E-14	chr:5:8937853
5	rs6880574	8969997	G	0.2251	3.50E-14	chr:5:8937853
5	rs6884680	8968261	T	0.2251	3.50E-14	chr:5:8937853
5	rs13188103	8964519	T	0.2245	3.51E-14	chr:5:8937853
5	rs6871960	8963871	C	0.2245	3.51E-14	chr:5:8937853
5	rs10062754	8972259	C	0.225	3.84E-14	chr:5:8937853
5	rs16881756	8976859	G	0.225	3.84E-14	chr:5:8937853
5	rs1947587	8974402	A	0.225	3.84E-14	chr:5:8937853
5	rs2219397	8971713	T	0.225	3.84E-14	chr:5:8937853

5	rs2219398	8971683	C	0.225	3.84E-14	chr:5:8937853
5	rs3921964	8972146	G	0.225	3.84E-14	chr:5:8937853
5	rs6555588	8977866	G	0.225	3.84E-14	chr:5:8937853
5	rs6870017	8977512	A	0.225	3.84E-14	chr:5:8937853
5	rs6880494	8977180	G	0.225	3.84E-14	chr:5:8937853
5	rs13164654	8972492	T	0.2251	4.37E-14	chr:5:8937853
5	rs6884293	8968011	T	0.2275	4.68E-14	chr:5:8937853
5	rs1947586	8974532	C	0.2275	5.12E-14	chr:5:8937853
5	rs6877048	8973265	T	0.2284	5.52E-14	chr:5:8937853
5	rs4627964	8968639	C	0.2282	5.61E-14	chr:5:8937853
5	rs2962651	8935871	A	0.2289	6.44E-14	chr:5:8937853
5	rs2962733	8945736	C	0.2289	6.44E-14	chr:5:8937853
5	rs11954862	8949344	C	0.213	7.16E-14	chr:5:8937853
5	rs6873594	8930306	A	0.213	7.16E-14	chr:5:8937853
5	rs1947584	8980099	C	0.2055	8.17E-14	chr:5:8937853
5	rs74482613	8927592	C	0.2149	9.51E-14	chr:5:8937853
5	rs2963325	8933269	T	0.2467	1.01E-13	chr:5:8937853
5	rs6889183	8968532	A	0.2122	2.11E-13	chr:5:8937853
5	rs75014751	8965335	C	0.2122	2.11E-13	chr:5:8937853
5	rs75281373	8964076	A	0.2122	2.11E-13	chr:5:8937853
5	rs79740539	8969856	C	0.2122	2.11E-13	chr:5:8937853
5	rs115606914	8942429	T	0.2024	2.25E-13	chr:5:8937853
5	rs140461872	8942428	C	0.2024	2.25E-13	chr:5:8937853
5	rs2963370	8940538	A	4.16	3.16E-13	chr:5:8937853
5	rs2963371	8940701	A	4.16	3.16E-13	chr:5:8937853
5	rs2963337	8935632	T	4.16	4.29E-13	chr:5:8937853
5	rs2963324	8933130	C	0.2653	4.45E-13	chr:5:8937853
5	rs76169939	8934848	T	0.2116	4.76E-13	chr:5:8937853
5	rs13163334	8962662	T	4.127	5.43E-13	chr:5:8937853
5	rs12658301	8927212	C	0.2937	1.49E-12	chr:5:8937853
5	rs2085514	9001414	A	0.2891	4.27E-12	chr:5:8937853
5	rs6868644	8981136	C	0.2987	1.89E-11	chr:5:8937853
5	rs2963375	8942567	A	3.541	8.29E-11	chr:5:8937853
5	rs577492066	8948517	T	0.2452	8.86E-11	chr:5:8937853
5	rs61036506	8981340	A	3.197	3.62E-10	chr:5:8937853
5	rs2925903	8983680	C	0.328	3.98E-10	chr:5:8937853
5	rs2925905	8985794	T	0.3278	4.52E-10	chr:5:8937853
5	rs2925908	8991487	T	0.3344	7.05E-10	chr:5:8937853
5	rs2925909	8992098	C	0.3346	7.51E-10	chr:5:8937853
5	rs2962705	8997304	C	0.3346	7.51E-10	chr:5:8937853
5	rs2100641	9014968	A	0.3423	7.57E-10	chr:5:8937853
5	rs12188723	8952085	C	0.1657	7.74E-10	chr:5:8937853
5	rs75949059	8948201	T	0.1657	7.74E-10	chr:5:8937853

5	rs4452535	8958034	T	3.183	1.10E-09	chr:5:8937853
5	rs2962707	8990084	G	0.344	1.29E-09	chr:5:8937853
5	rs16881678	8920805	T	0.3188	1.57E-09	chr:5:8937853
5	rs2962724	8952042	C	2.931	7.77E-09	chr:5:8937853
5	rs2925910	8992745	C	2.881	9.99E-09	chr:5:8937853
5	rs111603494	8976335	T	0.1284	1.03E-08	chr:5:8937853
5	rs113779456	8954029	C	0.1284	1.03E-08	chr:5:8937853
5	rs112164098	8961299	A	0.1355	1.54E-08	chr:5:8937853
5	rs112735467	8956946	T	0.1355	1.54E-08	chr:5:8937853
5	rs113044593	8961853	T	0.1355	1.54E-08	chr:5:8937853
5	rs114049874	8958877	G	0.2492	1.93E-08	chr:5:8937853
5	rs11750358	8999971	T	0.3929	3.35E-08	chr:5:8937853
5	rs16881803	8983995	C	0.3417	3.64E-08	chr:5:8937853
5	rs11750315	8999860	A	0.3491	4.96E-08	chr:5:8937853
5	rs77037743	8991632	T	0.3491	4.96E-08	chr:5:8937853
5	rs11750361	9000007	G	0.3972	5.07E-08	chr:5:8937853
5	rs11738920	8991968	C	0.3495	5.14E-08	chr:5:8937853
5	rs7720782	8994422	C	0.3511	5.79E-08	chr:5:8937853
5	rs114656264	8942423	T	0.3104	1.47E-07	chr:5:8937853
5	rs115883187	8942422	C	0.3104	1.47E-07	chr:5:8937853
5	rs2963349	8915030	C	0.2575	1.55E-07	chr:5:8937853
5	rs3111057	8915303	C	0.2575	1.55E-07	chr:5:8937853
5	rs4702602	9014396	G	0.43	2.23E-07	chr:5:8937853
5	rs2963341	8908533	G	0.2375	2.39E-07	chr:5:8937853
5	rs2963344	8909428	G	0.2375	2.39E-07	chr:5:8937853
5	rs2963345	8909508	T	0.2375	2.39E-07	chr:5:8937853
5	rs2963347	8910873	C	0.2375	2.39E-07	chr:5:8937853
5	rs112110914	9006527	C	0.4314	2.55E-07	chr:5:8937853
5	rs12517597	9002094	C	0.4314	2.55E-07	chr:5:8937853
5	rs1379567	9016944	C	0.4314	2.55E-07	chr:5:8937853
5	rs1379568	9016763	G	0.4314	2.55E-07	chr:5:8937853
5	rs1379569	9016719	C	0.4314	2.55E-07	chr:5:8937853
5	rs1379570	9016694	C	0.4314	2.55E-07	chr:5:8937853
5	rs1379573	9013983	T	0.4314	2.55E-07	chr:5:8937853
5	rs1598825	9021566	C	0.4314	2.55E-07	chr:5:8937853
5	rs2085512	9014812	C	0.4314	2.55E-07	chr:5:8937853
5	rs2925919	9001913	G	0.4314	2.55E-07	chr:5:8937853
5	rs2925920	9004597	T	0.4314	2.55E-07	chr:5:8937853
5	rs2925921	9005353	T	0.4314	2.55E-07	chr:5:8937853
5	rs2925922	9010282	T	0.4314	2.55E-07	chr:5:8937853
5	rs2925923	9012388	T	0.4314	2.55E-07	chr:5:8937853
5	rs2925924	9019816	C	0.4314	2.55E-07	chr:5:8937853
5	rs2925928	9032400	G	0.4314	2.55E-07	chr:5:8937853

5	rs2962718	9008955	G	0.4314	2.55E-07	chr:5:8937853
5	rs2962719	9009465	G	0.4314	2.55E-07	chr:5:8937853
5	rs2962720	9009675	C	0.4314	2.55E-07	chr:5:8937853
5	rs2962721	9010331	A	0.4314	2.55E-07	chr:5:8937853
5	rs74980194	9003526	T	0.4314	2.55E-07	chr:5:8937853
5	rs78490007	9003525	G	0.4314	2.55E-07	chr:5:8937853
5	rs79211599	9003402	A	0.4314	2.55E-07	chr:5:8937853
5	rs146470969	9001510	C	0.4061	2.57E-07	chr:5:8937853
5	rs148681757	9001509	C	0.4061	2.57E-07	chr:5:8937853
5	rs2962702	9025608	G	2.533	2.72E-07	chr:5:8937853
5	rs10037735	9004192	T	2.519	3.20E-07	chr:5:8937853
5	rs2962675	8913429	C	0.3882	3.59E-07	chr:5:8937853
5	rs1505067	9036790	T	2.499	3.61E-07	chr:5:8937853
5	rs2100642	9014917	T	2.482	4.37E-07	chr:5:8937853
5	rs142622218	9001560	C	0.2534	5.18E-07	chr:5:8937853
5	rs34968754	8959664	G	0.2051	9.41E-07	chr:5:8937853
5	rs72739858	8983300	G	2.412	9.88E-07	chr:5:8937853
5	rs10041221	9004208	T	0.4585	1.06E-06	chr:5:8937853
5	rs6883135	8944432	T	0.2184	1.12E-06	chr:5:8937853
5	rs376060904	9001673	A	0.3997	1.14E-06	chr:5:8937853
5	rs12517703	9011519	T	0.4005	1.19E-06	chr:5:8937853
5	rs16881838	9012115	T	0.4005	1.19E-06	chr:5:8937853
5	rs111387677	9004023	A	0.4025	1.36E-06	chr:5:8937853
5	rs143252128	9006280	T	0.4025	1.36E-06	chr:5:8937853
5	rs6897203	9002771	C	0.4025	1.36E-06	chr:5:8937853
5	rs75432582	9010227	T	0.4025	1.36E-06	chr:5:8937853
5	rs112854697	9006793	C	0.4043	1.52E-06	chr:5:8937853
5	rs10512996	8916550	A	0.1805	1.55E-06	chr:5:8937853
5	rs35191363	8912793	T	0.1805	1.55E-06	chr:5:8937853
5	rs71611351	8896933	T	0.1805	1.55E-06	chr:5:8937853
5	rs16881671	8917970	G	0.2601	1.75E-06	chr:5:8937853
5	rs16881672	8918651	G	0.2601	1.75E-06	chr:5:8937853
5	rs6555577	8924770	A	0.2601	1.75E-06	chr:5:8937853
5	rs9313260	8919349	G	0.2601	1.75E-06	chr:5:8937853
5	rs35592900	8942245	T	0.2164	2.63E-06	chr:5:8937853
5	rs35817870	8939620	G	0.2164	2.63E-06	chr:5:8937853
5	rs4583857	8941558	G	0.2164	2.63E-06	chr:5:8937853
5	rs71611355	8950617	A	0.2164	2.63E-06	chr:5:8937853
5	rs77781846	8941171	G	0.2164	2.63E-06	chr:5:8937853
5	rs2925902	8982517	T	0.4565	2.65E-06	chr:5:8937853
5	rs11134341	8989290	C	0.4655	2.93E-06	chr:5:8937853
5	rs7703582	8988714	T	0.4655	2.93E-06	chr:5:8937853
5	rs7723456	8989021	G	0.4655	2.93E-06	chr:5:8937853

5	rs7727402	8988961	G	0.4655	2.93E-06	chr:5:8937853
5	rs77709148	8988682	G	0.4655	2.93E-06	chr:5:8937853
5	rs11134342	8991036	A	0.4689	3.39E-06	chr:5:8937853
5	rs7712850	8990532	A	0.4689	3.39E-06	chr:5:8937853
5	rs7733108	8990306	G	0.4689	3.39E-06	chr:5:8937853
5	rs6861563	8926391	G	0.4041	3.47E-06	chr:5:8937853
5	rs11134343	8999304	C	0.4698	3.66E-06	chr:5:8937853
5	rs11951651	8995163	C	0.4698	3.66E-06	chr:5:8937853
5	rs11952933	8993747	T	0.4698	3.66E-06	chr:5:8937853
5	rs12186781	8998527	G	0.4698	3.66E-06	chr:5:8937853
5	rs146404047	8998963	T	0.4698	3.66E-06	chr:5:8937853
5	rs6870588	8993002	C	0.4698	3.66E-06	chr:5:8937853
5	rs74864061	8993179	G	0.4698	3.66E-06	chr:5:8937853
5	rs77430208	8997524	G	0.4698	3.66E-06	chr:5:8937853
5	rs79649573	8997977	T	0.4698	3.66E-06	chr:5:8937853
5	rs1354570	9059223	G	0.4038	3.87E-06	chr:5:8937853
5	rs12189149	8998699	G	0.4707	3.93E-06	chr:5:8937853
5	rs556660023	8990404	A	0.1459	4.00E-06	chr:5:8937853
5	rs10512995	8916360	G	0.3215	4.62E-06	chr:5:8937853
5	rs1457768	9034674	G	0.2248	4.72E-06	chr:5:8937853
5	rs1457773	8978530	A	0.2294	5.44E-06	chr:5:8937853
5	rs112032434	9014459	T	0.2231	6.51E-06	chr:5:8937853
5	rs114501856	8991577	A	0.2231	6.51E-06	chr:5:8937853
5	rs12189367	8987361	A	0.2231	6.51E-06	chr:5:8937853
5	rs17235037	9013608	T	0.2231	6.51E-06	chr:5:8937853
5	rs74984858	8986951	C	0.2231	6.51E-06	chr:5:8937853
5	rs7709752	8990196	G	0.2231	6.51E-06	chr:5:8937853
5	rs77207173	8984484	A	0.2231	6.51E-06	chr:5:8937853
5	rs77213924	8984873	G	0.2231	6.51E-06	chr:5:8937853
5	rs77865922	9034181	C	0.2231	6.51E-06	chr:5:8937853
5	rs139408148	8956642	G	0.1269	7.73E-06	chr:5:8937853
5	rs114493044	8980394	A	0.1625	8.23E-06	chr:5:8937853
5	rs17237125	9097230	G	0.2743	8.63E-06	chr:5:8937853
5	rs17309469	9100211	A	0.2743	8.63E-06	chr:5:8937853
5	rs76447168	8967849	G	0.1842	8.85E-06	chr:5:8937853
5	rs143959340	9002474	A	0.2317	8.90E-06	chr:5:8937853
5	rs75952224	9153291	C	0.221	1.01E-05	chr:5:8937853
5	rs2963351	8915179	T	0.4563	1.07E-05	chr:5:8937853
5	rs79101386	8996698	T	0.2496	1.09E-05	chr:5:8937853
5	rs73740342	9128407	C	0.2961	1.15E-05	chr:5:8937853
5	rs116598534	8929687	G	0.12	1.17E-05	chr:5:8937853
5	rs10037162	8902858	T	0.3845	1.31E-05	chr:5:8937853
5	rs10061810	8898595	A	0.3845	1.31E-05	chr:5:8937853

5	rs73740340	9127330	T	0.3018	1.44E-05	chr:5:8937853
5	rs79499339	9051312	T	0.3044	1.48E-05	chr:5:8937853
5	rs17237055	9093482	G	0.2335	1.57E-05	chr:5:8937853
5	rs17316598	9120407	T	0.2335	1.57E-05	chr:5:8937853
5	rs41283163	9108135	A	0.2335	1.57E-05	chr:5:8937853
5	rs79065199	9091308	A	0.2335	1.57E-05	chr:5:8937853
5	rs73740338	9127236	C	0.3055	1.67E-05	chr:5:8937853
5	rs74385970	9013525	T	0.2764	1.68E-05	chr:5:8937853
5	rs12518501	8982887	T	0.4594	1.71E-05	chr:5:8937853
5	rs12521160	8982888	G	0.4594	1.71E-05	chr:5:8937853
5	rs75435196	8987064	G	0.4697	1.74E-05	chr:5:8937853
5	rs17238609	9136646	G	0.2923	1.83E-05	chr:5:8937853
5	rs61181903	9135540	A	0.2923	1.83E-05	chr:5:8937853
5	rs73740343	9129804	T	0.2923	1.83E-05	chr:5:8937853
5	rs73740345	9135285	A	0.2923	1.83E-05	chr:5:8937853
5	rs73740346	9136174	A	0.2923	1.83E-05	chr:5:8937853
5	rs76859700	9135470	C	0.2923	1.83E-05	chr:5:8937853
5	rs372303759	8927200	C	0.1983	1.89E-05	chr:5:8937853
5	rs115127228	8913358	T	0.385	1.92E-05	chr:5:8937853
5	rs113334166	9135393	T	0.2991	2.38E-05	chr:5:8937853
5	rs74370667	8988253	C	0.4734	2.38E-05	chr:5:8937853
6	rs73603096	161272870	C	37.5	1.08E-32	chr:6:161270898
6	rs75211522	161266022	T	0.06158	1.70E-28	chr:6:161270898
6	rs13208565	161268025	A	0.05062	5.36E-28	chr:6:161270898
6	rs13208570	161268029	A	0.05062	5.36E-28	chr:6:161270898
6	rs13208572	161268036	A	0.05062	5.36E-28	chr:6:161270898
6	rs76259860	161263334	G	0.06149	2.24E-27	chr:6:161270898
6	rs6908103	161274560	T	16.17	4.77E-27	chr:6:161270898
6	rs28515783	161268702	C	0.06368	8.67E-27	chr:6:161270898
6	rs376433866	161274602	G	0.04402	2.16E-26	chr:6:161270898
6	rs9346839	161255046	C	0.07091	4.76E-26	chr:6:161270898
6	rs373446157	161274587	A	0.0332	6.50E-26	chr:6:161270898
6	rs62435319	161274589	A	0.0332	6.50E-26	chr:6:161270898
6	rs146201099	161274582	T	0.03305	6.53E-26	chr:6:161270898
6	rs28594774	161271666	T	0.02575	9.90E-26	chr:6:161270898
6	rs9456600	161272696	T	0.01332	7.09E-25	chr:6:161270898
6	rs9765937	161268118	C	0.026	9.93E-25	chr:6:161270898
6	rs9766526	161268119	T	0.026	9.93E-25	chr:6:161270898
6	rs139452685	161263330	C	0.02423	1.08E-23	chr:6:161270898
6	rs147841394	161263331	A	0.02423	1.08E-23	chr:6:161270898
6	rs5013439	161283060	G	0.06193	4.38E-23	chr:6:161270898
6	rs5013440	161283064	A	0.06193	4.38E-23	chr:6:161270898
6	rs5013441	161283071	A	0.06193	4.38E-23	chr:6:161270898

6	rs5013438	161283051	A	0.06216	5.05E-23	chr:6:161270898
6	rs13220254	161269958	T	0.02295	5.45E-23	chr:6:161270898
6	rs144015366	161274617	A	0.01873	1.15E-22	chr:6:161270898
6	rs370624331	161274612	T	0.01873	1.15E-22	chr:6:161270898
6	rs538674856	161274614	T	0.01873	1.15E-22	chr:6:161270898
6	rs558873783	161274615	A	0.01873	1.15E-22	chr:6:161270898
6	rs573457182	161274613	G	0.01873	1.15E-22	chr:6:161270898
6	rs144320744	161265076	T	10.46	1.67E-22	chr:6:161270898
6	rs75664940	161265073	C	10.46	1.67E-22	chr:6:161270898
6	rs76731838	161265072	T	10.46	1.67E-22	chr:6:161270898
6	rs765351	161252685	C	0.1198	4.10E-22	chr:6:161270898
6	rs2465859	161282713	C	0.05121	6.75E-22	chr:6:161270898
6	rs2489951	161259799	T	0.01467	7.63E-22	chr:6:161270898
6	rs76926711	161270283	C	0.01665	1.25E-21	chr:6:161270898
6	rs59536962	161264131	A	0.01578	1.52E-21	chr:6:161270898
6	rs2953494	161283178	G	0.1444	2.60E-21	chr:6:161270898
6	rs2953495	161283183	C	0.1444	2.60E-21	chr:6:161270898
6	rs2997094	161283135	T	0.1474	3.46E-21	chr:6:161270898
6	rs2997095	161283204	A	0.1478	3.50E-21	chr:6:161270898
6	rs7383738	161276047	A	0.05768	6.08E-21	chr:6:161270898
6	rs2489938	161282377	G	0.05808	7.21E-21	chr:6:161270898
6	rs2997093	161283133	T	0.1535	7.84E-21	chr:6:161270898
6	rs2489948	161259549	G	0.1312	8.39E-21	chr:6:161270898
6	rs2465846	161258027	C	0.1314	9.66E-21	chr:6:161270898
6	rs2489942	161257961	G	0.1314	9.66E-21	chr:6:161270898
6	rs28544460	161271777	T	0.014	1.20E-20	chr:6:161270898
6	rs28655734	161271783	T	0.014	1.20E-20	chr:6:161270898
6	rs66561051	161265144	C	0.01408	1.22E-20	chr:6:161270898
6	rs67613705	161265155	T	0.01408	1.22E-20	chr:6:161270898
6	rs2489965	161262607	C	0.0142	1.33E-20	chr:6:161270898
6	rs9347453	161270866	C	0.01415	1.36E-20	chr:6:161270898
6	rs9347454	161270870	C	0.01415	1.36E-20	chr:6:161270898
6	rs73604919	161277020	A	0.014	1.36E-20	chr:6:161270898
6	rs9347458	161277046	C	0.01405	1.37E-20	chr:6:161270898
6	rs67368974	161264124	A	0.01339	1.53E-20	chr:6:161270898
6	rs1652471	161254403	T	0.1377	2.79E-20	chr:6:161270898
6	rs1937479	161255544	C	0.1377	2.79E-20	chr:6:161270898
6	rs67391812	161273234	T	0.05544	8.97E-20	chr:6:161270898
6	rs2465857	161282500	C	0.03407	1.04E-19	chr:6:161270898
6	rs10945703	161279258	G	0.1839	1.29E-19	chr:6:161270898
6	rs59450759	161279302	A	0.1839	1.29E-19	chr:6:161270898
6	rs1892347	161281358	A	0.01123	1.50E-19	chr:6:161270898
6	rs62435318	161274301	C	0.01123	2.04E-19	chr:6:161270898

6	rs9347456	161276965	C	0.01134	2.08E-19	chr:6:161270898
6	rs7383363	161274962	A	0.01137	2.19E-19	chr:6:161270898
6	rs2489961	161261383	T	0.01141	2.25E-19	chr:6:161270898
6	rs2489958	161261109	T	0.01139	2.26E-19	chr:6:161270898
6	rs12153973	161273891	T	0.01138	2.27E-19	chr:6:161270898
6	rs9355844	161268967	T	0.0114	2.30E-19	chr:6:161270898
6	rs2489969	161264604	T	0.01145	2.31E-19	chr:6:161270898
6	rs55968870	161264803	A	0.01145	2.31E-19	chr:6:161270898
6	rs10945701	161266812	C	0.01142	2.33E-19	chr:6:161270898
6	rs10945702	161267174	A	0.01142	2.33E-19	chr:6:161270898
6	rs11753103	161267086	A	0.01142	2.33E-19	chr:6:161270898
6	rs11753537	161267022	T	0.01142	2.33E-19	chr:6:161270898
6	rs11753560	161267122	T	0.01142	2.33E-19	chr:6:161270898
6	rs12153845	161273987	C	0.01142	2.33E-19	chr:6:161270898
6	rs12173853	161266428	A	0.01142	2.33E-19	chr:6:161270898
6	rs12173859	161266513	A	0.01142	2.33E-19	chr:6:161270898
6	rs12176296	161266407	C	0.01142	2.33E-19	chr:6:161270898
6	rs12189741	161263528	T	0.01142	2.33E-19	chr:6:161270898
6	rs12191314	161263721	T	0.01142	2.33E-19	chr:6:161270898
6	rs12194479	161263485	G	0.01142	2.33E-19	chr:6:161270898
6	rs12196112	161264064	G	0.01142	2.33E-19	chr:6:161270898
6	rs12209829	161263938	C	0.01142	2.33E-19	chr:6:161270898
6	rs12215233	161263453	A	0.01142	2.33E-19	chr:6:161270898
6	rs13192981	161275824	T	0.01142	2.33E-19	chr:6:161270898
6	rs13200798	161275510	C	0.01142	2.33E-19	chr:6:161270898
6	rs13203412	161275871	G	0.01142	2.33E-19	chr:6:161270898
6	rs13203427	161275892	G	0.01142	2.33E-19	chr:6:161270898
6	rs13219765	161275516	T	0.01142	2.33E-19	chr:6:161270898
6	rs2465855	161270619	G	0.01142	2.33E-19	chr:6:161270898
6	rs2465856	161271369	C	0.01142	2.33E-19	chr:6:161270898
6	rs2465861	161261486	T	0.01142	2.33E-19	chr:6:161270898
6	rs2465862	161261198	G	0.01142	2.33E-19	chr:6:161270898
6	rs2465863	161260969	A	0.01142	2.33E-19	chr:6:161270898
6	rs2465864	161273785	G	0.01142	2.33E-19	chr:6:161270898
6	rs2465865	161273576	G	0.01142	2.33E-19	chr:6:161270898
6	rs2465866	161273489	G	0.01142	2.33E-19	chr:6:161270898
6	rs2489930	161271311	G	0.01142	2.33E-19	chr:6:161270898
6	rs2489931	161273628	A	0.01142	2.33E-19	chr:6:161270898
6	rs2489932	161273637	C	0.01142	2.33E-19	chr:6:161270898
6	rs2489960	161261315	C	0.01142	2.33E-19	chr:6:161270898
6	rs2489966	161262874	T	0.01142	2.33E-19	chr:6:161270898
6	rs2489968	161264408	G	0.01142	2.33E-19	chr:6:161270898
6	rs28448569	161271706	T	0.01142	2.33E-19	chr:6:161270898

6	rs28488549	161266035	A	0.01142	2.33E-19	chr:6:161270898
6	rs28533078	161268730	G	0.01142	2.33E-19	chr:6:161270898
6	rs28597670	161271643	C	0.01142	2.33E-19	chr:6:161270898
6	rs28670241	161268736	C	0.01142	2.33E-19	chr:6:161270898
6	rs28698869	161265892	C	0.01142	2.33E-19	chr:6:161270898
6	rs28711930	161272157	A	0.01142	2.33E-19	chr:6:161270898
6	rs28715036	161266045	G	0.01142	2.33E-19	chr:6:161270898
6	rs2997097	161263200	G	0.01142	2.33E-19	chr:6:161270898
6	rs55900705	161264920	G	0.01142	2.33E-19	chr:6:161270898
6	rs56238146	161264866	G	0.01142	2.33E-19	chr:6:161270898
6	rs57127570	161272996	C	0.01142	2.33E-19	chr:6:161270898
6	rs59942091	161263402	A	0.01142	2.33E-19	chr:6:161270898
6	rs62435277	161265332	C	0.01142	2.33E-19	chr:6:161270898
6	rs62435278	161265459	C	0.01142	2.33E-19	chr:6:161270898
6	rs62435280	161265620	C	0.01142	2.33E-19	chr:6:161270898
6	rs62435284	161268570	A	0.01142	2.33E-19	chr:6:161270898
6	rs66533808	161273302	C	0.01142	2.33E-19	chr:6:161270898
6	rs66940842	161265190	C	0.01142	2.33E-19	chr:6:161270898
6	rs67136969	161273433	T	0.01142	2.33E-19	chr:6:161270898
6	rs67591610	161268474	C	0.01142	2.33E-19	chr:6:161270898
6	rs67721200	161265209	C	0.01142	2.33E-19	chr:6:161270898
6	rs67876803	161268321	G	0.01142	2.33E-19	chr:6:161270898
6	rs67998011	161273327	G	0.01142	2.33E-19	chr:6:161270898
6	rs68027339	161272813	G	0.01142	2.33E-19	chr:6:161270898
6	rs68142848	161273257	T	0.01142	2.33E-19	chr:6:161270898
6	rs73029748	161268408	C	0.01142	2.33E-19	chr:6:161270898
6	rs7381442	161276215	T	0.01142	2.33E-19	chr:6:161270898
6	rs7381975	161275254	G	0.01142	2.33E-19	chr:6:161270898
6	rs7382318	161276128	G	0.01142	2.33E-19	chr:6:161270898
6	rs7382435	161276434	C	0.01142	2.33E-19	chr:6:161270898
6	rs7382456	161276583	C	0.01142	2.33E-19	chr:6:161270898
6	rs74199274	161274567	G	0.01142	2.33E-19	chr:6:161270898
6	rs9295132	161267909	G	0.01142	2.33E-19	chr:6:161270898
6	rs9347447	161269371	G	0.01142	2.33E-19	chr:6:161270898
6	rs9347451	161270100	T	0.01142	2.33E-19	chr:6:161270898
6	rs9347452	161270827	C	0.01142	2.33E-19	chr:6:161270898
6	rs9347455	161276752	C	0.01142	2.33E-19	chr:6:161270898
6	rs9355320	161269256	A	0.01142	2.33E-19	chr:6:161270898
6	rs9355841	161267335	A	0.01142	2.33E-19	chr:6:161270898
6	rs9355842	161267442	C	0.01142	2.33E-19	chr:6:161270898
6	rs9355845	161269097	A	0.01142	2.33E-19	chr:6:161270898
6	rs9355846	161269204	C	0.01142	2.33E-19	chr:6:161270898
6	rs9365219	161270070	A	0.01142	2.33E-19	chr:6:161270898

6	rs9365221	161276676	G	0.01142	2.33E-19	chr:6:161270898
6	rs9456598	161272283	A	0.01142	2.33E-19	chr:6:161270898
6	rs9456599	161272401	A	0.01142	2.33E-19	chr:6:161270898
6	rs9458045	161262083	A	0.01142	2.33E-19	chr:6:161270898
6	rs9458046	161262133	G	0.01142	2.33E-19	chr:6:161270898
6	rs9458047	161262336	A	0.01142	2.33E-19	chr:6:161270898
6	rs9458048	161272317	A	0.01142	2.33E-19	chr:6:161270898
6	rs9765938	161268135	C	0.01142	2.33E-19	chr:6:161270898
6	rs9766397	161267843	A	0.01142	2.33E-19	chr:6:161270898
6	rs9766412	161268255	A	0.01142	2.33E-19	chr:6:161270898
6	rs9766533	161268200	T	0.01142	2.33E-19	chr:6:161270898
6	rs2953496	161263032	A	0.01134	2.34E-19	chr:6:161270898
6	rs1806449	161280932	G	0.01146	2.35E-19	chr:6:161270898
6	rs1892348	161281390	T	0.01146	2.35E-19	chr:6:161270898
6	rs2243944	161280766	C	0.01146	2.35E-19	chr:6:161270898
6	rs2465848	161280023	C	0.01146	2.35E-19	chr:6:161270898
6	rs2465849	161280167	C	0.01146	2.35E-19	chr:6:161270898
6	rs2465850	161281175	T	0.01146	2.35E-19	chr:6:161270898
6	rs2489936	161280325	G	0.01146	2.35E-19	chr:6:161270898
6	rs2489949	161259718	T	0.01146	2.35E-19	chr:6:161270898
6	rs2186128	161269751	T	0.01145	2.37E-19	chr:6:161270898
6	rs9347448	161269737	C	0.01145	2.37E-19	chr:6:161270898
6	rs9347449	161269740	C	0.01145	2.37E-19	chr:6:161270898
6	rs28446193	161272078	A	0.01147	2.39E-19	chr:6:161270898
6	rs13216274	161275309	A	0.01146	2.40E-19	chr:6:161270898
6	rs12196838	161272765	A	0.01147	2.42E-19	chr:6:161270898
6	rs3949208	161269749	T	0.01144	2.43E-19	chr:6:161270898
6	rs10945699	161266748	G	0.01148	2.46E-19	chr:6:161270898
6	rs10945700	161266764	T	0.01148	2.46E-19	chr:6:161270898
6	rs9355843	161268805	G	0.01239	3.29E-19	chr:6:161270898
6	rs9365215	161268788	A	0.01239	3.29E-19	chr:6:161270898
6	rs9365216	161268818	G	0.01239	3.29E-19	chr:6:161270898
6	rs11753819	161264775	G	0.01216	3.58E-19	chr:6:161270898
6	rs9365217	161269849	T	0.01214	3.61E-19	chr:6:161270898
6	rs12176297	161266417	C	0.01186	3.66E-19	chr:6:161270898
6	rs9347450	161269890	A	0.01217	3.66E-19	chr:6:161270898
6	rs9365218	161269903	A	0.01217	3.66E-19	chr:6:161270898
6	rs13201433	161269959	C	0.01222	3.76E-19	chr:6:161270898
6	rs13217026	161269951	A	0.01222	3.76E-19	chr:6:161270898
6	rs2489967	161262877	G	0.01174	3.79E-19	chr:6:161270898
6	rs377289957	161277231	C	6.946	6.30E-19	chr:6:161270898
6	rs1652463	161237204	C	0.161	6.86E-19	chr:6:161270898
6	rs783154	161229780	G	0.1606	2.86E-18	chr:6:161270898

6	rs812482	161227652	G	0.1725	3.62E-18	chr:6:161270898
6	rs1740448	161230370	T	0.1672	5.43E-18	chr:6:161270898
6	rs60507137	161273066	A	0.174	1.15E-17	chr:6:161270898
6	rs66542006	161273252	C	0.174	1.15E-17	chr:6:161270898
6	rs2465860	161261813	C	0.2271	8.16E-17	chr:6:161270898
6	rs9456593	161261833	C	0.2164	1.23E-16	chr:6:161270898
6	rs13206496	161279478	C	5.208	1.47E-16	chr:6:161270898
6	rs1406890	161187335	C	0.2155	1.54E-16	chr:6:161270898
6	rs62435345	161283600	T	0.22	1.56E-16	chr:6:161270898
6	rs1247555	161189018	A	0.212	1.61E-16	chr:6:161270898
6	rs1406891	161187080	T	0.2164	1.82E-16	chr:6:161270898
6	rs1782625	161290812	T	0.2144	2.31E-16	chr:6:161270898
6	rs9347462	161279599	A	5.736	2.58E-16	chr:6:161270898
6	rs1782624	161290595	G	0.2152	2.85E-16	chr:6:161270898
6	rs1937484	161286950	T	0.2152	2.85E-16	chr:6:161270898
6	rs2489943	161288640	C	0.2152	2.85E-16	chr:6:161270898
6	rs2953492	161289895	G	0.2152	2.85E-16	chr:6:161270898
6	rs9456594	161261839	A	4.521	4.94E-16	chr:6:161270898
6	rs2802358	161243584	C	0.2049	4.97E-16	chr:6:161270898
6	rs1629794	161246303	G	0.2079	5.98E-16	chr:6:161270898
6	rs2802357	161242350	T	0.2059	6.09E-16	chr:6:161270898
6	rs2565724	161241889	A	0.208	6.11E-16	chr:6:161270898
6	rs1247541	161253485	T	0.2086	6.37E-16	chr:6:161270898
6	rs71551166;rs149448086	161266025	G	0.005193	7.70E-16	chr:6:161270898
6	rs9365224	161279539	A	5.608	8.38E-16	chr:6:161270898
6	rs202074609	161261844	C	4.431	8.70E-16	chr:6:161270898
6	rs9456595	161261845	T	4.431	8.70E-16	chr:6:161270898
6	rs1247553	161188103	A	0.2352	8.95E-16	chr:6:161270898
6	rs2953493	161291617	C	0.2253	1.10E-15	chr:6:161270898
6	rs2802354	161245752	G	0.2092	1.23E-15	chr:6:161270898
6	rs9365226	161279734	G	5.834	1.72E-15	chr:6:161270898
6	rs9347463	161279690	T	5.788	2.04E-15	chr:6:161270898
6	rs62435324	161276911	T	6.217	4.20E-15	chr:6:161270898
6	rs72501802	161263496	C	6.217	4.20E-15	chr:6:161270898
6	rs76838390	161272755	G	6.217	4.20E-15	chr:6:161270898
6	rs62435300	161269523	T	6.204	4.21E-15	chr:6:161270898
6	rs2489952	161260530	G	6.207	4.26E-15	chr:6:161270898
6	rs9346838	161247649	G	0.2461	9.96E-15	chr:6:161270898
6	rs200406291	161261860	G	4.395	1.02E-14	chr:6:161270898
6	rs9456596	161261870	A	4.395	1.02E-14	chr:6:161270898
6	rs2565721	161235098	A	0.2372	1.95E-14	chr:6:161270898
6	rs4709473	161278938	C	4.921	2.22E-14	chr:6:161270898
6	rs9689560	161279081	T	0.3413	2.85E-14	chr:6:161270898

6	rs1782618	161230368	A	0.2451	3.49E-14	chr:6:161270898
6	rs9689453	161278910	G	4.951	5.40E-14	chr:6:161270898
6	rs59754710	161187850	C	0.2726	5.69E-14	chr:6:161270898
6	rs2465868	161287333	C	0.2617	8.06E-14	chr:6:161270898
6	rs201418433	161261865	A	4.433	8.24E-14	chr:6:161270898
6	rs58508475	161279395	A	0.3021	1.21E-13	chr:6:161270898
6	rs1937487	161284779	T	0.2637	3.22E-13	chr:6:161270898
6	rs9365225	161279685	G	7.064	3.63E-13	chr:6:161270898
6	rs783153	161229578	T	0.2816	6.33E-13	chr:6:161270898
6	rs783156	161231177	T	0.2816	6.33E-13	chr:6:161270898
6	rs1652482	161197885	A	4	9.13E-13	chr:6:161270898
6	rs148378598	161290870	C	0.2151	2.19E-12	chr:6:161270898
6	rs79538248	161291744	A	0.2151	2.19E-12	chr:6:161270898
6	rs62435283	161267489	T	5.292	5.45E-12	chr:6:161270898
6	rs1740432	161210470	A	0.2637	7.34E-12	chr:6:161270898
6	rs1247558	161190199	A	0.3035	9.29E-12	chr:6:161270898
6	rs1247556	161189595	G	0.3036	1.01E-11	chr:6:161270898
6	rs62436729	161210806	T	0.2767	1.32E-11	chr:6:161270898
6	rs9456592	161231592	T	4.926	1.57E-11	chr:6:161270898
6	rs9458040	161232224	C	4.908	1.83E-11	chr:6:161270898
6	rs1247520	161312344	T	0.2422	2.33E-11	chr:6:161270898
6	rs1247570	161208957	T	0.2797	2.51E-11	chr:6:161270898
6	rs2255792	161284473	C	0.3305	5.07E-11	chr:6:161270898
6	rs4252181	161175004	T	0.2099	6.14E-11	chr:6:161270898
6	rs9456580	161185380	T	0.2099	6.14E-11	chr:6:161270898
6	rs73782325	161295855	C	0.241	7.02E-11	chr:6:161270898
6	rs73782326	161306601	G	0.242	7.59E-11	chr:6:161270898
6	rs73782327	161308469	G	0.242	7.59E-11	chr:6:161270898
6	rs1621801	161196966	A	0.3385	7.82E-11	chr:6:161270898
6	rs1614840	161197845	A	0.3389	8.51E-11	chr:6:161270898
6	rs1740442	161197558	T	0.3389	8.51E-11	chr:6:161270898
6	rs1652466	161249569	T	0.3216	8.58E-11	chr:6:161270898
6	rs1782632	161249412	A	0.3216	8.58E-11	chr:6:161270898
6	rs115067610	161274512	T	5.55	1.12E-10	chr:6:161270898
6	rs74200112	161274513	C	5.55	1.12E-10	chr:6:161270898
6	rs2565725	161244359	A	0.3235	1.13E-10	chr:6:161270898
6	rs56262039	161183455	A	0.2182	1.19E-10	chr:6:161270898
6	rs57848140	161299240	C	0.2523	1.72E-10	chr:6:161270898
6	rs1937486	161284845	G	0.3463	1.90E-10	chr:6:161270898
6	rs13219777	161275735	G	5.357	2.02E-10	chr:6:161270898
6	rs60198722	161181980	A	0.2262	2.23E-10	chr:6:161270898
6	rs1937485	161284895	A	0.3428	2.39E-10	chr:6:161270898
6	rs66507368	161271537	T	5.296	2.46E-10	chr:6:161270898

6	rs111906830	161242033	A	0.1987	2.56E-10	chr:6:161270898
6	rs76436475	161247828	T	0.1987	2.56E-10	chr:6:161270898
6	rs112603661	161276925	T	0.2039	2.62E-10	chr:6:161270898
6	rs151060299	161281678	C	0.2039	2.62E-10	chr:6:161270898
6	rs79090443	161269153	A	0.2039	2.62E-10	chr:6:161270898
6	rs1652465	161248552	T	0.3344	2.92E-10	chr:6:161270898
6	rs71551165	161263039	G	5.248	3.09E-10	chr:6:161270898
6	rs112898058	161274433	A	5.207	3.25E-10	chr:6:161270898
6	rs2802356	161238215	G	0.3356	3.42E-10	chr:6:161270898
6	rs12190057	161264201	G	5.217	3.62E-10	chr:6:161270898
6	rs12195948	161263790	A	5.217	3.62E-10	chr:6:161270898
6	rs9355840	161251350	C	5.194	3.83E-10	chr:6:161270898
6	rs28402939	161168949	T	0.228	3.87E-10	chr:6:161270898
6	rs9347445	161257418	G	5.19	3.97E-10	chr:6:161270898
6	rs66514671	161268507	T	4.485	4.50E-10	chr:6:161270898
6	rs66746245	161270505	G	4.485	4.50E-10	chr:6:161270898
6	rs9365214	161267556	C	4.485	4.50E-10	chr:6:161270898
6	rs9365222	161276870	T	4.485	4.50E-10	chr:6:161270898
6	rs10945698	161237551	C	5.155	4.57E-10	chr:6:161270898
6	rs6925542	161241864	A	5.155	4.57E-10	chr:6:161270898
6	rs9458022	161171682	A	0.2429	5.08E-10	chr:6:161270898
6	rs783145	161152449	G	0.3325	5.17E-10	chr:6:161270898
6	rs4708894	161243370	C	4.959	5.18E-10	chr:6:161270898
6	rs6690	161174138	A	0.3562	6.01E-10	chr:6:161270898
6	rs783168	161174594	A	0.3562	6.01E-10	chr:6:161270898
6	rs783171	161173610	A	0.3562	6.01E-10	chr:6:161270898
6	rs11060	161173946	T	0.3565	6.47E-10	chr:6:161270898
6	rs783173	161172942	C	0.3565	6.47E-10	chr:6:161270898
6	rs77409175	161278861	A	0.2177	8.40E-10	chr:6:161270898
6	rs13218423	161267283	T	4.365	8.98E-10	chr:6:161270898
6	rs9355847	161276847	G	4.365	8.98E-10	chr:6:161270898
6	rs56055227	161264940	G	4.359	8.99E-10	chr:6:161270898
6	rs1247513	161280379	T	0.3059	1.12E-09	chr:6:161270898
6	rs6926947	161272676	T	0.1672	1.62E-09	chr:6:161270898
6	rs73029749	161268411	T	0.1672	1.62E-09	chr:6:161270898
6	rs36162229	161265356	A	4.172	1.70E-09	chr:6:161270898
6	rs1620921	161197087	A	0.3657	1.73E-09	chr:6:161270898
6	rs1740443	161197042	T	0.3657	1.73E-09	chr:6:161270898
6	rs2024839	161233418	C	4.377	2.40E-09	chr:6:161270898
6	rs1835345	161240330	T	4.949	2.51E-09	chr:6:161270898
6	rs1317216	161282102	C	5.086	4.28E-09	chr:6:161270898
6	rs4252096	161134273	G	0.2391	5.43E-09	chr:6:161270898
6	rs4252107	161137518	C	0.2391	5.43E-09	chr:6:161270898

6	rs62439804	161134805	C	0.2391	5.43E-09	chr:6:161270898
6	rs1261931	161311849	T	0.2866	5.48E-09	chr:6:161270898
6	rs114581531	161237090	T	5.253	6.50E-09	chr:6:161270898
6	rs115032617	161237091	G	5.253	6.50E-09	chr:6:161270898
6	rs143687018	161237088	C	5.253	6.50E-09	chr:6:161270898
6	rs370751318	161237096	G	5.253	6.50E-09	chr:6:161270898
6	rs373974575	161237097	A	5.253	6.50E-09	chr:6:161270898
6	rs376125603	161237094	C	5.253	6.50E-09	chr:6:161270898
6	rs13202192	161233181	T	4.305	7.43E-09	chr:6:161270898
6	rs1247519	161303720	A	0.2914	7.73E-09	chr:6:161270898
6	rs2997096	161298263	T	0.2914	7.73E-09	chr:6:161270898
6	rs4252066	161127125	A	0.2447	7.77E-09	chr:6:161270898
6	rs4252130	161153080	C	0.247	7.89E-09	chr:6:161270898
6	rs4252134	161153527	C	0.247	7.89E-09	chr:6:161270898
6	rs12191929	161234926	G	4.24	1.02E-08	chr:6:161270898
6	rs56387679	161154878	A	0.2621	1.05E-08	chr:6:161270898
6	rs1247557	161189670	G	2.671	1.43E-08	chr:6:161270898
6	rs9458041	161235710	T	3.953	1.61E-08	chr:6:161270898
6	rs45485198	161237998	C	0.1893	1.69E-08	chr:6:161270898
6	rs4252135	161154232	T	0.2555	1.93E-08	chr:6:161270898
6	rs56093624	161156667	A	0.2555	1.93E-08	chr:6:161270898
6	rs9458019	161153561	C	0.2555	1.93E-08	chr:6:161270898
6	rs9458020	161156296	T	0.2555	1.93E-08	chr:6:161270898
6	rs783155	161229797	G	0.3387	2.29E-08	chr:6:161270898
6	rs783177	161169815	C	0.4128	2.70E-08	chr:6:161270898
6	rs783178	161169739	C	0.4128	2.70E-08	chr:6:161270898
6	rs4252126	161152294	G	0.267	4.13E-08	chr:6:161270898
6	rs4252117	161143376	G	0.2667	5.47E-08	chr:6:161270898
6	rs9456578	161147368	T	0.2667	5.47E-08	chr:6:161270898
6	rs9458016	161144800	G	0.2667	5.47E-08	chr:6:161270898
6	rs34620274	161266021	T	7.781	6.78E-08	chr:6:161270898
6	rs73782329	161312474	G	0.2932	7.23E-08	chr:6:161270898
6	rs3846778	161230801	C	0.1371	9.06E-08	chr:6:161270898
6	rs114592559	161259990	C	4.039	1.01E-07	chr:6:161270898
6	rs1084655	161181923	T	2.421	1.16E-07	chr:6:161270898
6	rs1247551	161187517	C	2.421	1.16E-07	chr:6:161270898
6	rs783162	161182954	G	2.421	1.16E-07	chr:6:161270898
6	rs783165	161177842	C	2.421	1.16E-07	chr:6:161270898
6	rs45475797	161237891	A	0.1309	1.21E-07	chr:6:161270898
6	rs34052962	161283211	G	4.544	1.23E-07	chr:6:161270898
6	rs112806054	161183522	A	0.0345	1.35E-07	chr:6:161270898
6	rs1084659	161163777	A	0.4317	1.43E-07	chr:6:161270898
6	rs1084658	161180477	A	2.358	2.38E-07	chr:6:161270898

6	rs1247552	161187744	T	2.358	2.38E-07	chr:6:161270898
6	rs783159	161185325	A	2.358	2.38E-07	chr:6:161270898
6	rs1247514	161283586	C	0.1288	2.42E-07	chr:6:161270898
6	rs2144727	161194814	C	3.126	2.47E-07	chr:6:161270898
6	rs12194271	161195895	G	3.128	2.58E-07	chr:6:161270898
6	rs12214168	161195801	T	3.128	2.58E-07	chr:6:161270898
6	rs12214254	161195932	A	3.128	2.58E-07	chr:6:161270898
6	rs9885597	161227692	A	4.5	2.64E-07	chr:6:161270898
6	rs150380099	161314966	G	0.3066	2.66E-07	chr:6:161270898
6	rs1084654	161182100	A	2.35	3.04E-07	chr:6:161270898
6	rs1344730	161204995	A	3.102	3.10E-07	chr:6:161270898
6	rs9458039	161225730	T	3.789	3.60E-07	chr:6:161270898
6	rs1853020	161221417	G	3.764	4.23E-07	chr:6:161270898
6	rs111629110	161269927	A	0.2606	7.34E-07	chr:6:161270898
6	rs783174	161171919	T	2.272	7.63E-07	chr:6:161270898
6	rs9458044	161259988	C	3.766	7.89E-07	chr:6:161270898
6	rs783180	161168961	T	2.262	8.12E-07	chr:6:161270898
6	rs783182	161168548	A	2.262	8.12E-07	chr:6:161270898
6	rs75415422	161280642	A	5.255	8.58E-07	chr:6:161270898
6	rs4252047	161121513	A	0.3112	8.96E-07	chr:6:161270898
6	rs11902	161174871	G	2.257	9.06E-07	chr:6:161270898
6	rs1247565	161206910	A	2.995	1.72E-06	chr:6:161270898
6	rs783169	161174579	C	2.197	1.92E-06	chr:6:161270898
6	rs149318841	161123894	A	0.3172	2.01E-06	chr:6:161270898
6	rs560909528	161262726	A	0.04542	2.02E-06	chr:6:161270898
6	rs1247518	161305051	T	0.4706	2.12E-06	chr:6:161270898
6	rs1652455	161210349	C	0.4654	2.27E-06	chr:6:161270898
6	rs783163	161182401	T	2.863	2.38E-06	chr:6:161270898
6	rs1652454	161210256	G	0.4659	2.92E-06	chr:6:161270898
6	rs1247308	161337202	G	2.719	2.94E-06	chr:6:161270898
6	rs4252043	161121161	C	0.3226	3.30E-06	chr:6:161270898
6	rs4252048	161121514	T	0.3226	3.30E-06	chr:6:161270898
6	rs73782431	161125556	C	0.3288	3.52E-06	chr:6:161270898
6	rs1615527	161211095	G	0.3769	3.70E-06	chr:6:161270898
6	rs7771298	161239477	C	0.04999	4.83E-06	chr:6:161270898
6	rs13206066	161167452	G	2.4	5.78E-06	chr:6:161270898
6	rs112580052	161249898	T	0.2745	5.85E-06	chr:6:161270898
6	rs73782311	161259297	G	0.3322	6.06E-06	chr:6:161270898
6	rs73782315	161265102	A	0.3322	6.06E-06	chr:6:161270898
6	rs79548693	161249510	T	0.2906	6.22E-06	chr:6:161270898
6	rs74854775	161268389	G	0.339	7.13E-06	chr:6:161270898
6	rs783160	161183722	C	0.3874	7.36E-06	chr:6:161270898
6	rs7454268	161166502	T	2.363	8.33E-06	chr:6:161270898

6	rs61200572	161282034	A	4.565	8.47E-06	chr:6:161270898
6	rs73782366	161345303	A	0.4209	9.22E-06	chr:6:161270898
6	rs4252076	161128922	G	0.4841	9.24E-06	chr:6:161270898
6	rs4440440	161183424	C	2.428	9.27E-06	chr:6:161270898
6	rs7450979	161186889	T	2.428	9.27E-06	chr:6:161270898
6	rs9458023	161176256	A	2.428	9.27E-06	chr:6:161270898
6	rs73782428	161112870	T	0.4277	9.52E-06	chr:6:161270898
6	rs4252165	161161559	A	0.422	1.00E-05	chr:6:161270898
6	rs9456579	161148937	G	0.1444	1.05E-05	chr:6:161270898
6	rs577902960	161285239	C	0.1058	1.08E-05	chr:6:161270898
6	rs112880342	161137155	C	0.1457	1.15E-05	chr:6:161270898
6	rs9689609	161101652	A	0.4374	1.27E-05	chr:6:161270898
6	rs73782425	161109672	A	0.4371	1.32E-05	chr:6:161270898
6	rs73782424	161109242	T	0.4445	1.36E-05	chr:6:161270898
6	rs4252072	161128264	T	0.4929	1.50E-05	chr:6:161270898
6	rs7760010	161106603	A	0.4374	1.54E-05	chr:6:161270898
6	rs115279925	161269479	A	4.402	1.59E-05	chr:6:161270898
6	rs783161	161183665	A	0.3868	1.65E-05	chr:6:161270898
6	rs1247539	161252795	T	0.3095	1.68E-05	chr:6:161270898
6	rs62436701	161194298	T	0.4391	1.74E-05	chr:6:161270898
6	rs62436702	161196562	C	0.4391	1.74E-05	chr:6:161270898
6	rs150098363	161289826	C	3.201	1.84E-05	chr:6:161270898
6	rs62436933	161346830	A	0.2402	1.85E-05	chr:6:161270898
6	rs1740446	161194278	C	0.5143	2.16E-05	chr:6:161270898
6	rs1620875	161201175	A	0.4234	2.27E-05	chr:6:161270898
6	rs143110183	161273008	T	0.07656	2.31E-05	chr:6:161270898
6	rs143577715	161236112	T	0.07656	2.31E-05	chr:6:161270898
6	rs144714092	161215008	T	0.07656	2.31E-05	chr:6:161270898
6	rs146619810	161218089	G	0.07656	2.31E-05	chr:6:161270898
6	rs146721784	161206112	A	0.07656	2.31E-05	chr:6:161270898
6	rs148742681	161237753	G	0.07656	2.31E-05	chr:6:161270898
6	rs151100736	161197737	T	0.07656	2.31E-05	chr:6:161270898
6	rs370897911	161210487	A	0.07656	2.31E-05	chr:6:161270898
6	rs578009304	161289118	C	0.07656	2.31E-05	chr:6:161270898
6	rs79772203	161224947	A	0.07656	2.31E-05	chr:6:161270898
6	rs598306	161357756	A	0.3092	2.43E-05	chr:6:161270898
6	rs622215	161363231	T	0.3092	2.43E-05	chr:6:161270898
6	rs114987387	32502131	A	0.2405	5.41E-15	chr:6:32505702
6	rs116316731	32502097	C	0.2399	7.00E-15	chr:6:32505702
6	rs9269561	32544405	C	0.2368	7.05E-15	chr:6:32505702
6	rs9269560	32544371	A	0.241	8.88E-15	chr:6:32505702
6	rs35957455	32521195	G	0.233	9.38E-15	chr:6:32505702
6	rs74485644	32501421	C	0.2486	9.73E-15	chr:6:32505702

6	rs114034298	32505797	C	2.848	1.01E-14	chr:6:32505702
6	rs72492314	32512421	T	0.2441	1.11E-14	chr:6:32505702
6	rs112457394	32469073	T	0.2359	1.13E-14	chr:6:32505702
6	rs66497315	32512407	T	0.2439	1.17E-14	chr:6:32505702
6	rs114302967	32503839	T	0.3137	1.22E-14	chr:6:32505702
6	rs73727960	32508989	A	0.2395	1.24E-14	chr:6:32505702
6	rs113225456	32521406	C	0.2337	1.34E-14	chr:6:32505702
6	rs72844187	32521419	C	0.2337	1.34E-14	chr:6:32505702
6	rs76054694	32503840	G	0.316	1.54E-14	chr:6:32505702
6	rs112155110	32501417	C	0.2475	1.66E-14	chr:6:32505702
6	rs72851025	32501459	T	0.2458	1.68E-14	chr:6:32505702
6	rs72844188	32521427	T	0.2351	1.76E-14	chr:6:32505702
6	rs73729745	32469136	T	0.2442	1.88E-14	chr:6:32505702
6	rs72853983	32511854	G	0.2376	1.91E-14	chr:6:32505702
6	rs72844152	32519652	T	0.2427	1.95E-14	chr:6:32505702
6	rs5016923	32502028	A	0.2375	1.98E-14	chr:6:32505702
6	rs74937819	32502037	G	0.2375	1.98E-14	chr:6:32505702
6	rs114353707	32513535	T	0.237	2.04E-14	chr:6:32505702
6	rs116817148	32513534	A	0.237	2.04E-14	chr:6:32505702
6	rs73727980	32511774	T	0.2372	2.08E-14	chr:6:32505702
6	rs73727981	32511791	A	0.2372	2.08E-14	chr:6:32505702
6	rs72853927	32508474	A	0.3253	2.11E-14	chr:6:32505702
6	rs115806221	32514401	A	0.2367	2.15E-14	chr:6:32505702
6	rs66521709	32509829	G	0.2365	2.18E-14	chr:6:32505702
6	rs34615209	32513862	G	0.2368	2.19E-14	chr:6:32505702
6	rs35687625	32513840	T	0.2368	2.19E-14	chr:6:32505702
6	rs112180912	32518436	C	0.2647	2.19E-14	chr:6:32505702
6	rs72508464	32504341	C	0.2644	2.25E-14	chr:6:32505702
6	rs115712701	32501737	T	0.2372	2.26E-14	chr:6:32505702
6	rs66706092	32513915	C	0.2369	2.26E-14	chr:6:32505702
6	rs72844111	32513938	T	0.2369	2.26E-14	chr:6:32505702
6	rs67529500	32504757	G	0.2471	2.26E-14	chr:6:32505702
6	rs66534143	32512601	T	0.2477	2.32E-14	chr:6:32505702
6	rs34369284	32514144	C	0.2368	2.41E-14	chr:6:32505702
6	rs72492319	32511987	C	0.2368	2.41E-14	chr:6:32505702
6	rs75141783	32511944	G	0.2368	2.41E-14	chr:6:32505702
6	rs72844171	32520749	G	0.2366	2.46E-14	chr:6:32505702
6	rs113460787	32518089	C	0.24	2.57E-14	chr:6:32505702
6	rs66796862	32521664	T	0.2389	2.60E-14	chr:6:32505702
6	rs77783340	32514313	C	0.2437	2.64E-14	chr:6:32505702
6	rs73397416	32521951	A	0.2383	2.69E-14	chr:6:32505702
6	rs74207695	32502631	C	2.688	2.70E-14	chr:6:32505702
6	rs74194578	32517590	C	0.2418	2.71E-14	chr:6:32505702

6	rs75861594	32518103	T	0.2398	2.71E-14	chr:6:32505702
6	rs35301884	32513638	T	0.2383	2.75E-14	chr:6:32505702
6	rs67081540	32512359	T	0.2379	2.78E-14	chr:6:32505702
6	rs115123240	32519919	A	0.2385	2.80E-14	chr:6:32505702
6	rs67721312	32517556	C	0.2417	2.86E-14	chr:6:32505702
6	rs111340421	32517174	C	0.2376	2.88E-14	chr:6:32505702
6	rs72851044	32501886	T	0.2392	2.90E-14	chr:6:32505702
6	rs72492327	32520465	A	0.2387	3.01E-14	chr:6:32505702
6	rs66738149	32501898	T	0.2389	3.05E-14	chr:6:32505702
6	rs72853989	32512321	C	0.2463	3.09E-14	chr:6:32505702
6	rs73728002	32513993	G	0.2397	3.22E-14	chr:6:32505702
6	rs113017160	32503834	A	0.3233	3.24E-14	chr:6:32505702
6	rs72853928	32508486	C	0.3319	3.26E-14	chr:6:32505702
6	rs66924955	32507855	A	0.2736	3.27E-14	chr:6:32505702
6	rs72853919	32507852	T	0.2736	3.27E-14	chr:6:32505702
6	rs72851077	32504624	T	0.315	3.42E-14	chr:6:32505702
6	rs200263528	32509009	G	0.2565	3.50E-14	chr:6:32505702
6	rs72851028	32501615	C	0.2459	3.51E-14	chr:6:32505702
6	rs79364663	32504085	C	2.896	3.51E-14	chr:6:32505702
6	rs67774869	32508794	T	0.2534	3.52E-14	chr:6:32505702
6	rs534064478	32512189	C	0.2967	3.61E-14	chr:6:32505702
6	rs546382275	32512190	A	0.2967	3.61E-14	chr:6:32505702
6	rs67717173	32520137	C	0.2405	3.65E-14	chr:6:32505702
6	rs72492325	32520151	T	0.2405	3.65E-14	chr:6:32505702
6	rs67916930	32508823	A	0.2538	3.73E-14	chr:6:32505702
6	rs76723065	32510591	C	0.2544	3.74E-14	chr:6:32505702
6	rs116132127	32503328	G	0.3305	3.75E-14	chr:6:32505702
6	rs78622201	32518405	G	0.2584	3.82E-14	chr:6:32505702
6	rs114840252	32504071	T	2.848	3.93E-14	chr:6:32505702
6	rs114821537	32503522	C	0.3062	4.20E-14	chr:6:32505702
6	rs72851029	32501639	C	0.235	4.22E-14	chr:6:32505702
6	rs73727973	32509691	A	0.2476	4.24E-14	chr:6:32505702
6	rs73727961	32509022	G	0.2561	4.27E-14	chr:6:32505702
6	rs79604868	32504073	A	2.847	4.28E-14	chr:6:32505702
6	rs115296342	32503526	A	0.3063	4.31E-14	chr:6:32505702
6	rs115217308	32520296	C	0.2426	4.38E-14	chr:6:32505702
6	rs116173033	32520295	A	0.2426	4.38E-14	chr:6:32505702
6	rs111559749	32509012	C	0.2562	4.40E-14	chr:6:32505702
6	rs3828852	32520595	G	0.2539	4.48E-14	chr:6:32505702
6	rs73729138	32520854	A	0.2439	4.57E-14	chr:6:32505702
6	rs72853979	32511572	A	0.2454	4.60E-14	chr:6:32505702
6	rs73729131	32520313	G	0.2424	4.61E-14	chr:6:32505702
6	rs73729122	32519777	A	0.2684	4.66E-14	chr:6:32505702

6	rs369883062	32519734	T	0.2672	4.70E-14	chr:6:32505702
6	rs76317804	32469375	T	2.869	4.83E-14	chr:6:32505702
6	rs68175425	32517446	A	0.2503	4.83E-14	chr:6:32505702
6	rs115202951	32501311	C	0.2744	4.86E-14	chr:6:32505702
6	rs529580232	32512136	T	0.2892	4.95E-14	chr:6:32505702
6	rs551034683	32512138	G	0.2892	4.95E-14	chr:6:32505702
6	rs113850533	32516839	G	0.2436	4.98E-14	chr:6:32505702
6	rs114520912	32517504	C	0.2582	5.04E-14	chr:6:32505702
6	rs116645664	32517505	T	0.2582	5.04E-14	chr:6:32505702
6	rs115303801	32512229	T	0.2911	5.12E-14	chr:6:32505702
6	rs28819746	32509579	G	0.2473	5.21E-14	chr:6:32505702
6	rs73729111	32517525	G	0.2481	5.23E-14	chr:6:32505702
6	rs545949191	32502900	A	2.872	5.25E-14	chr:6:32505702
6	rs115878723	32503335	A	2.919	5.28E-14	chr:6:32505702
6	rs116225018	32503380	T	0.3397	5.28E-14	chr:6:32505702
6	rs531982544	32503334	C	2.919	5.28E-14	chr:6:32505702
6	rs72851063	32503901	C	0.2697	5.29E-14	chr:6:32505702
6	rs72508473	32508399	A	0.3004	5.30E-14	chr:6:32505702
6	rs202231764	32508412	A	0.3005	5.35E-14	chr:6:32505702
6	rs75820413	32508505	G	2.947	5.40E-14	chr:6:32505702
6	rs112458679	32512167	C	0.2969	5.50E-14	chr:6:32505702
6	rs68126668	32509603	T	0.2471	5.59E-14	chr:6:32505702
6	rs66532036	32519319	T	0.2478	5.64E-14	chr:6:32505702
6	rs112709345	32501541	C	0.2428	5.80E-14	chr:6:32505702
6	rs72492303	32509061	G	0.256	5.85E-14	chr:6:32505702
6	rs193201719	32505807	T	2.618	5.86E-14	chr:6:32505702
6	rs75174683	32504101	G	2.881	5.96E-14	chr:6:32505702
6	rs116675816	32512131	T	0.2892	6.02E-14	chr:6:32505702
6	rs111901878	32516893	C	0.2451	6.07E-14	chr:6:32505702
6	rs7765211	32502346	A	2.764	6.11E-14	chr:6:32505702
6	rs62405640	32516729	C	0.2459	6.15E-14	chr:6:32505702
6	rs76033762	32516702	T	0.2459	6.15E-14	chr:6:32505702
6	rs72492315	32512466	T	0.3071	6.22E-14	chr:6:32505702
6	rs72851052	32502249	C	0.2927	6.46E-14	chr:6:32505702
6	rs72851053	32502250	A	0.2927	6.46E-14	chr:6:32505702
6	rs114769197	32538217	A	2.826	6.62E-14	chr:6:32505702
6	rs115724613	32538216	C	2.826	6.62E-14	chr:6:32505702
6	rs74950807	32513375	C	0.2566	6.88E-14	chr:6:32505702
6	rs76818741	32513378	G	0.2566	6.88E-14	chr:6:32505702
6	rs75519123	32469404	G	2.83	6.96E-14	chr:6:32505702
6	rs73727944	32504713	C	0.2984	7.15E-14	chr:6:32505702
6	rs74211958	32469040	A	0.3302	7.38E-14	chr:6:32505702
6	rs112349327	32502948	A	2.85	7.41E-14	chr:6:32505702

6	rs114015220	32510599	C	0.2629	7.46E-14	chr:6:32505702
6	rs114472333	32510598	G	0.2629	7.46E-14	chr:6:32505702
6	rs112815590	32504110	T	2.876	7.56E-14	chr:6:32505702
6	rs111945767	32517045	G	0.2488	7.66E-14	chr:6:32505702
6	rs111289870	32512269	A	0.2652	7.70E-14	chr:6:32505702
6	rs7743846	32505600	T	0.3332	7.95E-14	chr:6:32505702
6	rs114198395	32502961	T	2.84	8.11E-14	chr:6:32505702
6	rs72853940	32508945	G	0.2648	8.21E-14	chr:6:32505702
6	rs9269749	32548136	T	0.2326	8.39E-14	chr:6:32505702
6	rs115095329	32503497	G	0.3258	8.52E-14	chr:6:32505702
6	rs116133469	32503492	C	0.3258	8.52E-14	chr:6:32505702
6	rs72851056	32502944	A	2.847	8.58E-14	chr:6:32505702
6	rs74394760	32513078	T	0.2641	8.80E-14	chr:6:32505702
6	rs72851046	32501957	T	0.2509	9.21E-14	chr:6:32505702
6	rs72851047	32501962	G	0.2509	9.21E-14	chr:6:32505702
6	rs74221689	32501936	C	0.2509	9.21E-14	chr:6:32505702
6	rs72853926	32508451	A	0.322	9.31E-14	chr:6:32505702
6	rs111626267	32502273	G	0.3008	1.00E-13	chr:6:32505702
6	rs74375951	32500923	A	0.2603	1.01E-13	chr:6:32505702
6	rs72492311	32509979	C	0.2565	1.02E-13	chr:6:32505702
6	rs201439475	32502968	A	2.836	1.02E-13	chr:6:32505702
6	rs66678933	32509320	C	0.2502	1.03E-13	chr:6:32505702
6	rs72492304	32509311	T	0.2502	1.03E-13	chr:6:32505702
6	rs116190325	32510039	C	0.2564	1.06E-13	chr:6:32505702
6	rs116405199	32504367	C	0.2886	1.08E-13	chr:6:32505702
6	rs78994392	32504362	G	0.2886	1.08E-13	chr:6:32505702
6	rs115573660	32502277	T	0.3007	1.09E-13	chr:6:32505702
6	rs3828848	32520665	A	0.2559	1.09E-13	chr:6:32505702
6	rs547409849	32503365	T	2.861	1.11E-13	chr:6:32505702
6	rs565770331	32503366	C	2.861	1.11E-13	chr:6:32505702
6	rs72849282	32469335	C	0.3378	1.11E-13	chr:6:32505702
6	rs115077688	32503287	C	0.2733	1.12E-13	chr:6:32505702
6	rs62405642	32517759	A	0.2504	1.16E-13	chr:6:32505702
6	rs114653292	32503516	T	0.3206	1.18E-13	chr:6:32505702
6	rs199506689	32504701	C	0.3133	1.18E-13	chr:6:32505702
6	rs75088009	32504884	A	0.2951	1.21E-13	chr:6:32505702
6	rs111979375	32538231	T	2.746	1.21E-13	chr:6:32505702
6	rs112004746	32518155	C	0.2594	1.22E-13	chr:6:32505702
6	rs35697584	32522151	C	0.2594	1.23E-13	chr:6:32505702
6	rs142393802	32508021	T	2.475	1.24E-13	chr:6:32505702
6	rs555706008	32507953	G	2.455	1.31E-13	chr:6:32505702
6	rs140538649	32507946	T	2.455	1.35E-13	chr:6:32505702
6	rs539226504	32507944	A	2.455	1.35E-13	chr:6:32505702

6	rs571430728	32507938	T	2.455	1.35E-13	chr:6:32505702
6	rs76034941	32503498	A	0.331	1.36E-13	chr:6:32505702
6	rs67041740	32509273	T	0.2547	1.37E-13	chr:6:32505702
6	rs76662624	32503880	G	0.3096	1.38E-13	chr:6:32505702
6	rs113209512	32508875	C	0.2775	1.42E-13	chr:6:32505702
6	rs532050307	32469431	C	2.729	1.46E-13	chr:6:32505702
6	rs571525080	32469429	A	2.729	1.46E-13	chr:6:32505702
6	rs72492310	32509867	A	0.2688	1.46E-13	chr:6:32505702
6	rs530662953	32504587	G	0.3283	1.46E-13	chr:6:32505702
6	rs72851060	32503560	A	0.2991	1.46E-13	chr:6:32505702
6	rs147668426	32507931	G	2.453	1.48E-13	chr:6:32505702
6	rs185520080	32505872	C	2.321	1.48E-13	chr:6:32505702
6	rs115524254	32503565	A	0.2992	1.50E-13	chr:6:32505702
6	rs72851061	32503672	T	0.2906	1.52E-13	chr:6:32505702
6	rs545379090	32469285	C	0.3412	1.54E-13	chr:6:32505702
6	rs376327303	32520719	G	0.2663	1.56E-13	chr:6:32505702
6	rs115663137	32502992	C	2.789	1.57E-13	chr:6:32505702
6	rs73727954	32508902	G	0.2773	1.60E-13	chr:6:32505702
6	rs112471393	32538228	T	2.739	1.61E-13	chr:6:32505702
6	rs78806801	32503403	C	2.848	1.62E-13	chr:6:32505702
6	rs112300533	32517237	C	0.2583	1.63E-13	chr:6:32505702
6	rs74821931	32517233	G	0.2583	1.63E-13	chr:6:32505702
6	rs67860869	32508349	T	0.3083	1.64E-13	chr:6:32505702
6	rs72851100	32505511	T	0.3061	1.72E-13	chr:6:32505702
6	rs73397403	32519849	A	0.276	1.73E-13	chr:6:32505702
6	rs111725169	32512223	A	0.3067	1.76E-13	chr:6:32505702
6	rs115726960	32503171	C	0.2811	1.76E-13	chr:6:32505702
6	rs72492322	32519628	A	0.2671	1.77E-13	chr:6:32505702
6	rs79338946	32502822	T	2.569	1.82E-13	chr:6:32505702
6	rs376217655	32505666	T	2.395	1.82E-13	chr:6:32505702
6	rs550051341	32505851	C	2.316	1.84E-13	chr:6:32505702
6	rs571586981	32505852	A	2.316	1.84E-13	chr:6:32505702
6	rs78278896	32508647	A	0.281	1.89E-13	chr:6:32505702
6	rs116474488	32469226	T	2.777	1.92E-13	chr:6:32505702
6	rs540049194	32503078	A	2.521	1.92E-13	chr:6:32505702
6	rs536146370	32519759	G	0.2856	2.02E-13	chr:6:32505702
6	rs72492306	32509510	T	0.2649	2.04E-13	chr:6:32505702
6	rs72492307	32509519	T	0.2649	2.04E-13	chr:6:32505702
6	rs114638791	32538268	C	0.352	2.10E-13	chr:6:32505702
6	rs374408836	32507582	C	2.62	2.12E-13	chr:6:32505702
6	rs72851064	32503928	G	0.2772	2.14E-13	chr:6:32505702
6	rs72492317	32512501	G	0.3022	2.18E-13	chr:6:32505702
6	rs201795916	32514567	A	0.2719	2.33E-13	chr:6:32505702

6	rs72844118	32514539	C	0.2719	2.33E-13	chr:6:32505702
6	rs192164737	32508091	G	2.394	2.43E-13	chr:6:32505702
6	rs72853968	32509883	T	0.2775	2.47E-13	chr:6:32505702
6	rs73726852	32538295	T	0.3421	2.54E-13	chr:6:32505702
6	rs113900769	32503887	G	0.3103	2.56E-13	chr:6:32505702
6	rs113353313	32538242	A	0.3462	2.56E-13	chr:6:32505702
6	rs114831840	32501301	G	0.2968	2.57E-13	chr:6:32505702
6	rs115303308	32501296	G	0.2968	2.57E-13	chr:6:32505702
6	rs115664796	32508758	T	0.2812	2.60E-13	chr:6:32505702
6	rs115696138	32504460	T	0.3335	2.64E-13	chr:6:32505702
6	rs112944992	32510457	T	0.3265	2.64E-13	chr:6:32505702
6	rs113648142	32508762	G	0.2812	2.68E-13	chr:6:32505702
6	rs147023195	32518295	C	0.288	2.76E-13	chr:6:32505702
6	rs74535936	32510547	A	0.2868	2.82E-13	chr:6:32505702
6	rs556337500	32503531	A	2.784	2.86E-13	chr:6:32505702
6	rs571499647	32503532	G	2.784	2.86E-13	chr:6:32505702
6	rs114362560	32504503	C	0.3513	2.89E-13	chr:6:32505702
6	rs114961271	32503352	A	0.357	2.91E-13	chr:6:32505702
6	rs114629945	32503717	G	2.669	2.99E-13	chr:6:32505702
6	rs116103612	32503716	A	2.669	2.99E-13	chr:6:32505702
6	rs116422126	32504500	T	0.3514	3.11E-13	chr:6:32505702
6	rs150423066	32506995	A	2.696	3.11E-13	chr:6:32505702
6	rs180942055	32507976	T	2.392	3.17E-13	chr:6:32505702
6	rs186170828	32507977	G	2.392	3.17E-13	chr:6:32505702
6	rs573629697	32507971	G	2.392	3.17E-13	chr:6:32505702
6	rs562624040	32513024	T	0.3135	3.22E-13	chr:6:32505702
6	rs74786163	32507780	T	2.548	3.40E-13	chr:6:32505702
6	rs111649944	32518268	C	0.2837	3.41E-13	chr:6:32505702
6	rs112515515	32513422	G	0.2763	3.45E-13	chr:6:32505702
6	rs557288207	32504452	C	0.34	3.55E-13	chr:6:32505702
6	rs74205903	32501249	T	0.3052	3.60E-13	chr:6:32505702
6	rs544353633	32507596	G	2.578	3.61E-13	chr:6:32505702
6	rs116520213	32504424	A	0.3402	3.65E-13	chr:6:32505702
6	rs546636941	32504421	C	0.3402	3.65E-13	chr:6:32505702
6	rs66502326	32509411	C	0.2676	3.66E-13	chr:6:32505702
6	rs76940575	32502590	T	0.3526	3.72E-13	chr:6:32505702
6	rs72853949	32509435	A	0.2649	3.74E-13	chr:6:32505702
6	rs74197151	32469035	C	0.3576	3.75E-13	chr:6:32505702
6	rs76509141	32504529	T	0.3501	3.83E-13	chr:6:32505702
6	rs9269252	32454293	T	0.225	3.84E-13	chr:6:32505702
6	rs116609655	32503059	C	2.498	4.08E-13	chr:6:32505702
6	rs532618689	32508064	T	2.385	4.16E-13	chr:6:32505702
6	rs549780202	32508055	T	2.385	4.16E-13	chr:6:32505702

6	rs564974789	32508061	A	2.385	4.16E-13	chr:6:32505702
6	rs180756398	32458010	A	2.513	4.28E-13	chr:6:32505702
6	rs115084652	32468978	T	2.62	4.30E-13	chr:6:32505702
6	rs2157337	32501144	C	0.2992	4.31E-13	chr:6:32505702
6	rs542536566	32457959	T	2.47	4.35E-13	chr:6:32505702
6	rs561971374	32503086	A	2.477	4.72E-13	chr:6:32505702
6	rs114246255	32509461	A	0.2849	4.75E-13	chr:6:32505702
6	rs115704383	32509460	C	0.2849	4.75E-13	chr:6:32505702
6	rs72853950	32509472	G	0.2849	4.75E-13	chr:6:32505702
6	rs186217224	32457967	C	2.468	4.84E-13	chr:6:32505702
6	rs530370261	32519826	C	0.2966	4.94E-13	chr:6:32505702
6	rs113727677	32517010	T	0.269	4.98E-13	chr:6:32505702
6	rs113434238	32501204	C	0.3127	4.99E-13	chr:6:32505702
6	rs141020324	32469001	A	2.553	5.01E-13	chr:6:32505702
6	rs144975609	32468997	A	2.553	5.01E-13	chr:6:32505702
6	rs116209683	32510508	T	0.298	5.02E-13	chr:6:32505702
6	rs74211571	32502566	C	0.3547	5.19E-13	chr:6:32505702
6	rs113085907	32510224	C	0.3436	5.32E-13	chr:6:32505702
6	rs7765332	32502374	A	2.537	5.33E-13	chr:6:32505702
6	rs73726810	32522857	G	0.2378	5.50E-13	chr:6:32505702
6	rs73726819	32526540	A	0.2575	5.57E-13	chr:6:32505702
6	rs72508474	32508738	T	0.2896	5.59E-13	chr:6:32505702
6	rs199636459	32507758	A	2.394	5.71E-13	chr:6:32505702
6	rs72853970	32509919	G	0.2802	5.76E-13	chr:6:32505702
6	rs72853971	32509938	G	0.2802	5.76E-13	chr:6:32505702
6	rs113232784	32518256	G	0.2875	5.77E-13	chr:6:32505702
6	rs113220298	32510369	C	0.3191	5.96E-13	chr:6:32505702
6	rs535043779	32497358	A	2.367	6.01E-13	chr:6:32505702
6	rs552507937	32497354	A	2.367	6.01E-13	chr:6:32505702
6	rs570739482	32497356	C	2.367	6.01E-13	chr:6:32505702
6	rs34405098	32523269	C	0.2798	6.03E-13	chr:6:32505702
6	rs73726814	32523235	T	0.2798	6.03E-13	chr:6:32505702
6	rs72851065	32504213	C	0.3366	7.13E-13	chr:6:32505702
6	rs72851066	32504216	A	0.3366	7.13E-13	chr:6:32505702
6	rs113787446	32518630	A	0.298	7.20E-13	chr:6:32505702
6	rs114776163	32518625	A	0.298	7.20E-13	chr:6:32505702
6	rs115910196	32502304	C	0.3572	7.58E-13	chr:6:32505702
6	rs78942674	32509897	G	0.2888	7.69E-13	chr:6:32505702
6	rs149589923	32500818	G	0.2996	7.71E-13	chr:6:32505702
6	rs544527733	32503091	T	2.479	7.77E-13	chr:6:32505702
6	rs115380393	32503020	T	2.649	7.83E-13	chr:6:32505702
6	rs67461697	32518767	T	0.2927	7.93E-13	chr:6:32505702
6	rs538116343	32521742	T	3.026	8.03E-13	chr:6:32505702

6	rs114565678	32469224	G	0.3673	8.16E-13	chr:6:32505702
6	rs114233058	32517386	C	0.285	8.21E-13	chr:6:32505702
6	rs115916707	32517387	A	0.285	8.21E-13	chr:6:32505702
6	rs114682516	32503702	C	2.674	8.34E-13	chr:6:32505702
6	rs72853925	32508330	A	0.3229	8.84E-13	chr:6:32505702
6	rs555383676	32507771	A	2.382	8.97E-13	chr:6:32505702
6	rs35179057	32523349	T	0.2676	9.11E-13	chr:6:32505702
6	rs113133909	32457928	T	2.456	9.34E-13	chr:6:32505702
6	rs534859262	32457931	T	2.456	9.34E-13	chr:6:32505702
6	rs77173653	32508587	G	2.608	9.53E-13	chr:6:32505702
6	rs540194362	32507768	G	2.362	9.67E-13	chr:6:32505702
6	rs80083020	32510439	T	0.3403	1.00E-12	chr:6:32505702
6	rs116048339	32468982	T	2.507	1.02E-12	chr:6:32505702
6	rs116380301	32468983	T	2.507	1.02E-12	chr:6:32505702
6	rs67816706	32517266	A	0.2738	1.02E-12	chr:6:32505702
6	rs3828844	32520755	A	0.3024	1.06E-12	chr:6:32505702
6	rs116224274	32508583	C	2.605	1.07E-12	chr:6:32505702
6	rs537977627	32510140	G	0.3314	1.08E-12	chr:6:32505702
6	rs77149458	32487130	A	3.517	1.10E-12	chr:6:32505702
6	rs9270121	32555009	A	0.2433	1.11E-12	chr:6:32505702
6	rs547631481	32507531	A	2.441	1.14E-12	chr:6:32505702
6	rs373664556	32508561	C	2.569	1.18E-12	chr:6:32505702
6	rs553897781	32505335	A	2.406	1.20E-12	chr:6:32505702
6	rs111890942	32458131	T	0.3056	1.20E-12	chr:6:32505702
6	rs74202712	32502546	A	0.3683	1.21E-12	chr:6:32505702
6	rs116241189	32506751	G	0.356	1.21E-12	chr:6:32505702
6	rs72853911	32506759	A	0.356	1.21E-12	chr:6:32505702
6	rs9269750	32548141	T	0.2605	1.25E-12	chr:6:32505702
6	rs74290599	32518785	A	0.2973	1.28E-12	chr:6:32505702
6	rs71534520	32510146	C	0.3347	1.29E-12	chr:6:32505702
6	rs112111526	32510317	G	0.3417	1.30E-12	chr:6:32505702
6	rs76325796	32507653	A	2.621	1.31E-12	chr:6:32505702
6	rs77077499	32507652	T	2.621	1.31E-12	chr:6:32505702
6	rs112280675	32522066	G	0.278	1.34E-12	chr:6:32505702
6	rs201954564	32497824	C	0.2401	1.37E-12	chr:6:32505702
6	rs73727946	32504967	C	2.608	1.37E-12	chr:6:32505702
6	rs140120971	32510343	T	0.3417	1.42E-12	chr:6:32505702
6	rs143822887	32510344	G	0.3417	1.42E-12	chr:6:32505702
6	rs9269327	32540001	C	0.3423	1.45E-12	chr:6:32505702
6	rs538879571	32503698	A	2.662	1.47E-12	chr:6:32505702
6	rs68009841	32507731	T	2.582	1.48E-12	chr:6:32505702
6	rs76376937	32508568	G	2.564	1.49E-12	chr:6:32505702
6	rs184358099	32500832	A	0.3056	1.51E-12	chr:6:32505702

6	rs144411837	32510337	T	0.3419	1.57E-12	chr:6:32505702
6	rs571341495	32505326	G	2.401	1.61E-12	chr:6:32505702
6	rs191423596	32506888	C	2.24	1.63E-12	chr:6:32505702
6	rs116413420	32509466	C	0.292	1.73E-12	chr:6:32505702
6	rs114899212	32524951	A	0.3032	1.77E-12	chr:6:32505702
6	rs72849267	32468923	A	0.3635	1.77E-12	chr:6:32505702
6	rs112465099	32510430	A	0.348	1.82E-12	chr:6:32505702
6	rs112103446	32522679	C	6.995	1.82E-12	chr:6:32505702
6	rs556173675	32508202	G	2.285	1.97E-12	chr:6:32505702
6	rs572581776	32526776	A	0.3349	2.01E-12	chr:6:32505702
6	rs538727527	32505332	T	2.372	2.01E-12	chr:6:32505702
6	rs183777110	32505375	T	2.358	2.06E-12	chr:6:32505702
6	rs77413629	32505374	G	2.358	2.06E-12	chr:6:32505702
6	rs562852671	32512142	T	2.403	2.16E-12	chr:6:32505702
6	rs182987687	32506870	C	2.223	2.27E-12	chr:6:32505702
6	rs73727940	32503738	C	0.3689	2.28E-12	chr:6:32505702
6	rs115673628	32505419	G	2.333	2.36E-12	chr:6:32505702
6	rs538718414	32508207	T	2.28	2.39E-12	chr:6:32505702
6	rs9269985	32552532	C	0.3035	2.41E-12	chr:6:32505702
6	rs188426982	32506878	A	2.222	2.51E-12	chr:6:32505702
6	rs368398419	32510709	C	2.43	2.54E-12	chr:6:32505702
6	rs371989616	32510718	T	2.43	2.54E-12	chr:6:32505702
6	rs6905161	32505249	A	2.382	2.56E-12	chr:6:32505702
6	rs189968693	32506863	A	2.214	2.59E-12	chr:6:32505702
6	rs114015803	32485879	T	2.957	2.60E-12	chr:6:32505702
6	rs114693948	32485873	T	2.957	2.60E-12	chr:6:32505702
6	rs544548886	32505381	T	2.351	2.66E-12	chr:6:32505702
6	rs182374257	32525306	A	0.3726	2.74E-12	chr:6:32505702
6	rs72849265	32458208	C	0.3303	2.75E-12	chr:6:32505702
6	rs80216275	32469383	G	0.3617	2.95E-12	chr:6:32505702
6	rs9269563	32544456	C	0.2638	3.00E-12	chr:6:32505702
6	rs72851055	32502814	C	2.427	3.04E-12	chr:6:32505702
6	rs566710022	32507505	T	2.389	3.13E-12	chr:6:32505702
6	rs114171624	32514706	A	0.3342	3.31E-12	chr:6:32505702
6	rs111590055	32510177	T	0.3697	3.33E-12	chr:6:32505702
6	rs569509899	32507490	G	2.365	3.36E-12	chr:6:32505702
6	rs74197584	32502717	T	2.191	3.50E-12	chr:6:32505702
6	rs72508469	32505309	C	2.405	3.56E-12	chr:6:32505702
6	rs73726842	32533029	G	0.3167	3.65E-12	chr:6:32505702
6	rs73726843	32533042	G	0.3167	3.65E-12	chr:6:32505702
6	rs529484704	32507478	C	2.363	3.78E-12	chr:6:32505702
6	rs9269328	32540018	G	0.3507	3.86E-12	chr:6:32505702
6	rs562045282	32502681	T	2.173	4.12E-12	chr:6:32505702

6	rs111896611	32511183	G	0.3784	4.26E-12	chr:6:32505702
6	rs187395433	32510968	G	2.346	4.31E-12	chr:6:32505702
6	rs74197204	32507695	A	0.3806	4.32E-12	chr:6:32505702
6	rs73401349	32458094	G	0.3677	4.45E-12	chr:6:32505702
6	rs114020016	32524824	T	2.459	4.50E-12	chr:6:32505702
6	rs76571178	32525159	G	0.3974	4.55E-12	chr:6:32505702
6	rs9270529	32559658	C	0.3182	4.55E-12	chr:6:32505702
6	rs114435848	32532546	C	0.3545	4.79E-12	chr:6:32505702
6	rs115104630	32502387	A	2.451	4.84E-12	chr:6:32505702
6	rs147266303	32510268	G	0.3521	4.91E-12	chr:6:32505702
6	rs150117381	32505388	C	2.296	5.01E-12	chr:6:32505702
6	rs72853923	32508222	G	2.264	5.05E-12	chr:6:32505702
6	rs541499994	32510983	C	2.315	5.14E-12	chr:6:32505702
6	rs534024500	32507307	G	2.176	5.15E-12	chr:6:32505702
6	rs116085924	32514884	C	0.3868	5.40E-12	chr:6:32505702
6	rs72657675	32532955	A	0.362	5.49E-12	chr:6:32505702
6	rs72508465	32505141	C	0.388	5.57E-12	chr:6:32505702
6	rs116335331	32532577	C	0.3548	5.75E-12	chr:6:32505702
6	rs67011649	32532599	A	0.339	5.77E-12	chr:6:32505702
6	rs112427949	32511164	C	0.3824	5.86E-12	chr:6:32505702
6	rs116102092	32538672	A	3.354	5.92E-12	chr:6:32505702
6	rs72853924	32508239	G	2.277	5.97E-12	chr:6:32505702
6	rs543093016	32532533	T	0.3555	6.00E-12	chr:6:32505702
6	rs114905947	32533016	G	0.3231	6.05E-12	chr:6:32505702
6	rs72508466	32505166	G	2.54	6.08E-12	chr:6:32505702
6	rs76235954	32512815	A	2.484	6.08E-12	chr:6:32505702
6	rs116473597	32482374	T	2.176	6.28E-12	chr:6:32505702
6	rs556568526	32482353	T	2.176	6.28E-12	chr:6:32505702
6	rs578020847	32482368	C	2.176	6.28E-12	chr:6:32505702
6	rs112780153	32510301	A	0.3643	6.66E-12	chr:6:32505702
6	rs536387398	32507125	A	2.203	6.86E-12	chr:6:32505702
6	rs573273966	32507131	C	2.203	6.86E-12	chr:6:32505702
6	rs115137792	32510114	T	0.3481	7.03E-12	chr:6:32505702
6	rs9270570	32560978	A	0.2511	7.15E-12	chr:6:32505702
6	rs68146539	32532928	A	0.3629	7.19E-12	chr:6:32505702
6	rs72508468	32505288	C	2.386	7.68E-12	chr:6:32505702
6	rs9270045	32553662	A	0.2637	7.87E-12	chr:6:32505702
6	rs199616440	32510115	A	0.3502	7.90E-12	chr:6:32505702
6	rs550643609	32502794	A	2.278	7.97E-12	chr:6:32505702
6	rs76259812	32524672	A	0.3852	8.07E-12	chr:6:32505702
6	rs142475334	32533988	G	0.3774	8.17E-12	chr:6:32505702
6	rs66467908	32509787	C	0.2158	8.34E-12	chr:6:32505702
6	rs72492309	32509754	T	0.2158	8.34E-12	chr:6:32505702

6	rs114084676	32505216	C	2.494	8.36E-12	chr:6:32505702
6	rs73729106	32514288	G	0.2482	8.37E-12	chr:6:32505702
6	rs9269324	32539978	A	0.347	8.95E-12	chr:6:32505702
6	rs72492332	32523085	C	0.2738	8.98E-12	chr:6:32505702
6	rs9269565	32544500	G	0.2846	9.05E-12	chr:6:32505702
6	rs79740987	32507712	G	2.513	9.17E-12	chr:6:32505702
6	rs545256869	32512858	T	2.402	9.39E-12	chr:6:32505702
6	rs72508413	32454597	A	0.303	9.59E-12	chr:6:32505702
6	rs183703563	32498913	A	2.214	1.00E-11	chr:6:32505702
6	rs147819405	32533096	G	0.3517	1.02E-11	chr:6:32505702
6	rs535381236	32468870	G	2.382	1.04E-11	chr:6:32505702
6	rs559249402	32468864	C	2.382	1.04E-11	chr:6:32505702
6	rs577621741	32468866	A	2.382	1.04E-11	chr:6:32505702
6	rs73727912	32496944	T	0.3747	1.07E-11	chr:6:32505702
6	rs546964381	32511275	C	0.3298	1.14E-11	chr:6:32505702
6	rs558956689	32511276	A	0.3298	1.14E-11	chr:6:32505702
6	rs200819822	32505189	G	2.462	1.14E-11	chr:6:32505702
6	rs116585903	32487688	T	3.398	1.14E-11	chr:6:32505702
6	rs370864292	32532975	C	0.382	1.16E-11	chr:6:32505702
6	rs560693076	32532972	G	0.382	1.25E-11	chr:6:32505702
6	rs114327439	32505202	A	2.459	1.28E-11	chr:6:32505702
6	rs72508430	32487172	T	2.75	1.28E-11	chr:6:32505702
6	rs67515384	32505271	A	2.372	1.31E-11	chr:6:32505702
6	rs116526477	32468879	C	0.4063	1.32E-11	chr:6:32505702
6	rs114164335	32532128	T	0.3634	1.38E-11	chr:6:32505702
6	rs74715621	32508285	G	2.315	1.38E-11	chr:6:32505702
6	rs181244719	32498916	C	2.199	1.39E-11	chr:6:32505702
6	rs184303918	32529976	C	0.2694	1.40E-11	chr:6:32505702
6	rs72845745	32529961	C	0.2694	1.40E-11	chr:6:32505702
6	rs113871699	32509693	A	0.2512	1.48E-11	chr:6:32505702
6	rs73727972	32509683	C	0.2512	1.48E-11	chr:6:32505702
6	rs9270556	32560606	G	0.264	1.49E-11	chr:6:32505702
6	rs78085354	32529981	A	0.2692	1.53E-11	chr:6:32505702
6	rs34508462	32523476	T	0.2995	1.57E-11	chr:6:32505702
6	rs9270589	32561390	C	0.2623	1.60E-11	chr:6:32505702
6	rs9270590	32561392	G	0.2623	1.60E-11	chr:6:32505702
6	rs9270592	32561429	A	0.2623	1.60E-11	chr:6:32505702
6	rs9270593	32561445	A	0.2623	1.60E-11	chr:6:32505702
6	rs9270578	32561093	G	0.2643	1.68E-11	chr:6:32505702
6	rs374264352	32532969	C	0.3828	1.70E-11	chr:6:32505702
6	rs536463096	32510951	G	2.295	1.70E-11	chr:6:32505702
6	rs36218423	32524832	C	2.373	1.71E-11	chr:6:32505702
6	rs9270572	32561010	G	0.266	1.71E-11	chr:6:32505702

6	rs115946871	32530011	T	0.2712	1.73E-11	chr:6:32505702
6	rs9269254	32454327	C	0.2639	1.75E-11	chr:6:32505702
6	rs9270558	32560667	G	0.2635	1.77E-11	chr:6:32505702
6	rs9269549	32544202	T	0.2356	1.78E-11	chr:6:32505702
6	rs9269532	32543825	T	0.288	1.80E-11	chr:6:32505702
6	rs114715427	32538850	C	0.3431	1.82E-11	chr:6:32505702
6	rs9269250	32454269	C	0.243	1.83E-11	chr:6:32505702
6	rs113405235	32521796	C	2.904	1.83E-11	chr:6:32505702
6	rs72844144	32519125	A	3.402	1.85E-11	chr:6:32505702
6	rs112720311	32529995	G	0.2683	1.86E-11	chr:6:32505702
6	rs115077617	32532645	G	0.3391	1.94E-11	chr:6:32505702
6	rs114106672	32532692	G	0.2699	1.97E-11	chr:6:32505702
6	rs74615215	32458389	A	0.4083	2.01E-11	chr:6:32505702
6	rs113432958	32538366	C	0.3283	2.07E-11	chr:6:32505702
6	rs536038224	32523371	G	0.3029	2.08E-11	chr:6:32505702
6	rs111383925	32532741	T	0.2568	2.10E-11	chr:6:32505702
6	rs111566749	32532746	G	0.2568	2.10E-11	chr:6:32505702
6	rs116541769	32524870	T	2.399	2.11E-11	chr:6:32505702
6	rs115081905	32508276	T	2.264	2.12E-11	chr:6:32505702
6	rs139726276	32524769	G	2.392	2.12E-11	chr:6:32505702
6	rs9270574	32561028	G	0.2686	2.21E-11	chr:6:32505702
6	rs115978041	32522923	T	0.3212	2.27E-11	chr:6:32505702
6	rs183134414	32511069	T	2.28	2.38E-11	chr:6:32505702
6	rs112780983	32511348	C	0.2605	2.38E-11	chr:6:32505702
6	rs111786536	32523589	T	0.3004	2.40E-11	chr:6:32505702
6	rs9269880	32550916	A	0.3026	2.42E-11	chr:6:32505702
6	rs187316544	32500462	C	2.085	2.45E-11	chr:6:32505702
6	rs113504992	32531418	G	0.2503	2.48E-11	chr:6:32505702
6	rs146436875	32498948	G	2.172	2.57E-11	chr:6:32505702
6	rs148879657	32498940	G	2.172	2.57E-11	chr:6:32505702
6	rs527678609	32530435	G	0.3011	2.74E-11	chr:6:32505702
6	rs9269555	32544311	G	0.267	2.75E-11	chr:6:32505702
6	rs113573823	32532719	G	0.2681	2.78E-11	chr:6:32505702
6	rs9270576	32561068	C	0.2719	2.86E-11	chr:6:32505702
6	rs9269276	32539244	T	0.3035	2.88E-11	chr:6:32505702
6	rs115127109	32530038	C	0.2811	2.98E-11	chr:6:32505702
6	rs9270562	32560757	G	0.278	3.00E-11	chr:6:32505702
6	rs72845755	32530385	A	0.2519	3.07E-11	chr:6:32505702
6	rs72845749	32530081	C	0.255	3.10E-11	chr:6:32505702
6	rs73729140	32521290	A	0.3078	3.14E-11	chr:6:32505702
6	rs114328264	32534675	C	0.4006	3.16E-11	chr:6:32505702
6	rs181549666	32482352	A	2.144	3.16E-11	chr:6:32505702
6	rs72853904	32505575	C	0.2976	3.23E-11	chr:6:32505702

6	rs72845750	32530093	T	0.2544	3.35E-11	chr:6:32505702
6	rs139201366	32513186	G	0.3248	3.36E-11	chr:6:32505702
6	rs80011743	32531770	A	0.2922	3.39E-11	chr:6:32505702
6	rs142670190	32532172	C	0.3889	3.40E-11	chr:6:32505702
6	rs144412121	32532179	A	0.3889	3.40E-11	chr:6:32505702
6	rs115010442	32505229	C	2.401	3.42E-11	chr:6:32505702
6	rs537722644	32525112	C	0.4289	3.48E-11	chr:6:32505702
6	rs9269275	32539172	A	0.2755	3.52E-11	chr:6:32505702
6	rs111342306	32531091	T	0.3348	3.52E-11	chr:6:32505702
6	rs113209820	32531088	C	0.3348	3.52E-11	chr:6:32505702
6	rs556200726	32525900	T	0.4297	3.58E-11	chr:6:32505702
6	rs9269274	32539170	T	0.2753	3.65E-11	chr:6:32505702
6	rs142127023	32532178	C	0.389	3.66E-11	chr:6:32505702
6	rs9270363	32558137	C	0.2758	3.78E-11	chr:6:32505702
6	rs542648047	32535499	T	2.346	3.82E-11	chr:6:32505702
6	rs113051760	32521691	G	2.709	3.86E-11	chr:6:32505702
6	rs540951197	32530439	G	0.3111	3.86E-11	chr:6:32505702
6	rs562335725	32530443	G	0.3111	3.86E-11	chr:6:32505702
6	rs35255303	32536356	G	0.3039	3.91E-11	chr:6:32505702
6	rs9269317	32539767	C	0.3508	4.01E-11	chr:6:32505702
6	rs72845747	32530054	C	0.28	4.02E-11	chr:6:32505702
6	rs537678001	32527268	G	2.515	4.04E-11	chr:6:32505702
6	rs76454806	32530145	T	0.267	4.08E-11	chr:6:32505702
6	rs80142174	32530144	A	0.267	4.08E-11	chr:6:32505702
6	rs72845752	32530285	C	0.2526	4.28E-11	chr:6:32505702
6	rs72845754	32530286	A	0.2526	4.28E-11	chr:6:32505702
6	rs74201443	32530321	C	0.2526	4.28E-11	chr:6:32505702
6	rs72492356	32538899	G	0.3283	4.31E-11	chr:6:32505702
6	rs9270538	32559758	C	0.2946	4.34E-11	chr:6:32505702
6	rs114758808	32457997	C	0.4318	4.49E-11	chr:6:32505702
6	rs546033671	32498932	G	2.17	4.61E-11	chr:6:32505702
6	rs9269515	32543469	T	0.2794	4.65E-11	chr:6:32505702
6	rs77501456	32523627	A	0.3403	4.71E-11	chr:6:32505702
6	rs72845759	32531182	A	0.334	4.89E-11	chr:6:32505702
6	rs111632178	32511149	C	2.448	4.92E-11	chr:6:32505702
6	rs9270583	32561229	A	0.2746	4.95E-11	chr:6:32505702
6	rs142142101	32534657	T	0.4074	4.98E-11	chr:6:32505702
6	rs9270547	32559951	A	0.3515	5.01E-11	chr:6:32505702
6	rs3828832	32521083	T	0.3282	5.04E-11	chr:6:32505702
6	rs115469967	32530190	G	0.2657	5.08E-11	chr:6:32505702
6	rs77340266	32530200	G	0.2657	5.08E-11	chr:6:32505702
6	rs113955073	32522932	A	0.3301	5.11E-11	chr:6:32505702
6	rs73397495	32534640	T	0.4061	5.17E-11	chr:6:32505702

6	rs9269578	32544743	A	0.3369	5.19E-11	chr:6:32505702
6	rs111948873	32531081	A	0.344	5.42E-11	chr:6:32505702
6	rs111847217	32511131	G	2.445	5.44E-11	chr:6:32505702
6	rs72845757	32530563	G	0.2756	5.58E-11	chr:6:32505702
6	rs67283303	32531253	C	0.2986	5.61E-11	chr:6:32505702
6	rs182771482	32500178	C	2.06	5.64E-11	chr:6:32505702
6	rs116180191	32534588	T	0.3526	5.65E-11	chr:6:32505702
6	rs537366043	32531076	A	0.3531	5.73E-11	chr:6:32505702
6	rs570295314	32531074	A	0.3531	5.73E-11	chr:6:32505702
6	rs9269314	32539715	A	0.3405	5.78E-11	chr:6:32505702
6	rs115278245	32531057	G	0.3345	5.84E-11	chr:6:32505702
6	rs115407919	32531056	A	0.3345	5.84E-11	chr:6:32505702
6	rs115065497	32530466	T	0.3112	6.02E-11	chr:6:32505702
6	rs111596698	32487123	G	2.899	6.25E-11	chr:6:32505702
6	rs9270598	32561618	T	0.2744	6.34E-11	chr:6:32505702
6	rs72492343	32532385	C	0.3022	6.34E-11	chr:6:32505702
6	rs139128138	32533420	T	0.3817	6.35E-11	chr:6:32505702
6	rs576314890	32523379	A	0.2751	6.35E-11	chr:6:32505702
6	rs111843909	32531043	C	0.3242	6.46E-11	chr:6:32505702
6	rs559286232	32527271	C	2.497	6.60E-11	chr:6:32505702
6	rs73726834	32531111	T	0.3344	6.61E-11	chr:6:32505702
6	rs541088090	32533411	G	0.3835	6.74E-11	chr:6:32505702
6	rs9270548	32559960	C	0.3523	6.75E-11	chr:6:32505702
6	rs78274956	32526351	C	0.3523	6.87E-11	chr:6:32505702
6	rs141404431	32538578	T	2.841	6.92E-11	chr:6:32505702
6	rs143027966	32538579	T	2.841	6.92E-11	chr:6:32505702
6	rs151286796	32538577	T	2.841	6.92E-11	chr:6:32505702
6	rs115089463	32525092	G	0.4261	6.95E-11	chr:6:32505702
6	rs72845758	32531178	T	0.3434	6.98E-11	chr:6:32505702
6	rs116465569	32536986	T	0.3689	7.16E-11	chr:6:32505702
6	rs112310670	32531136	T	0.3337	7.31E-11	chr:6:32505702
6	rs9269277	32539265	G	0.3378	7.31E-11	chr:6:32505702
6	rs115640078	32538839	T	2.849	7.33E-11	chr:6:32505702
6	rs139796058	32531715	T	0.3342	7.36E-11	chr:6:32505702
6	rs11752506	32520273	A	2.646	7.41E-11	chr:6:32505702
6	rs149932905	32533421	A	0.3837	7.49E-11	chr:6:32505702
6	rs116350000	32523418	C	0.3134	7.67E-11	chr:6:32505702
6	rs112649230	32531140	A	0.3337	7.81E-11	chr:6:32505702
6	rs9270530	32559686	G	0.3552	7.86E-11	chr:6:32505702
6	rs9269465	32542247	A	0.2741	7.94E-11	chr:6:32505702
6	rs115141943	32538830	C	2.843	8.47E-11	chr:6:32505702
6	rs3828826	32485988	A	2.963	8.47E-11	chr:6:32505702
6	rs9270565	32560774	C	0.2988	8.51E-11	chr:6:32505702

6	rs9270566	32560775	A	0.2988	8.51E-11	chr:6:32505702
6	rs66754072	32532402	G	0.3013	8.63E-11	chr:6:32505702
6	rs9269299	32539541	T	0.3645	8.68E-11	chr:6:32505702
6	rs112083186	32531320	T	0.2891	8.77E-11	chr:6:32505702
6	rs138253052	32536103	C	0.3835	8.78E-11	chr:6:32505702
6	rs72492351	32538451	C	3.236	8.87E-11	chr:6:32505702
6	rs9269886	32550976	T	0.348	9.14E-11	chr:6:32505702
6	rs17424325	32521644	T	2.643	9.22E-11	chr:6:32505702
6	rs112715773	32530608	T	0.2836	9.34E-11	chr:6:32505702
6	rs9270549	32560440	G	0.2975	9.54E-11	chr:6:32505702
6	rs9270550	32560441	A	0.2975	9.54E-11	chr:6:32505702
6	rs9270567	32560801	T	0.285	9.63E-11	chr:6:32505702
6	rs148619571	32536155	T	0.3564	9.63E-11	chr:6:32505702
6	rs111548649	32533365	A	0.3783	9.87E-11	chr:6:32505702
6	rs9269885	32550971	A	0.348	9.92E-11	chr:6:32505702
6	rs9270580	32561123	T	0.2832	9.94E-11	chr:6:32505702
6	rs563850361	32531744	A	0.3434	9.94E-11	chr:6:32505702
6	rs9270126	32555052	G	0.3249	1.00E-10	chr:6:32505702
6	rs112302151	32530628	C	0.2858	1.02E-10	chr:6:32505702
6	rs12660259	32531709	G	0.3382	1.05E-10	chr:6:32505702
6	rs140209363	32492971	G	0.4267	1.05E-10	chr:6:32505702
6	rs373666709	32531748	G	0.3433	1.05E-10	chr:6:32505702
6	rs182090170	32537941	G	2.28	1.05E-10	chr:6:32505702
6	rs190345143	32537926	G	2.279	1.06E-10	chr:6:32505702
6	rs111749494	32532432	T	0.3006	1.08E-10	chr:6:32505702
6	rs112501266	32532428	A	0.3006	1.08E-10	chr:6:32505702
6	rs112236587	32530615	T	0.2856	1.08E-10	chr:6:32505702
6	rs145177101	32536632	A	0.419	1.10E-10	chr:6:32505702
6	rs535694878	32512216	G	0.3473	1.11E-10	chr:6:32505702
6	rs556986229	32512217	A	0.3473	1.11E-10	chr:6:32505702
6	rs9270521	32559529	A	0.3005	1.14E-10	chr:6:32505702
6	rs72492340	32531385	T	0.2953	1.15E-10	chr:6:32505702
6	rs72845768	32531377	T	0.2953	1.15E-10	chr:6:32505702
6	rs148612288	32498953	G	2.134	1.16E-10	chr:6:32505702
6	rs114633631	32531706	C	0.3336	1.16E-10	chr:6:32505702
6	rs146621647	32531602	C	0.3217	1.19E-10	chr:6:32505702
6	rs9269642	32545877	G	0.2412	1.19E-10	chr:6:32505702
6	rs201947932	32534556	A	0.364	1.24E-10	chr:6:32505702
6	rs9269278	32539269	G	0.3523	1.24E-10	chr:6:32505702
6	rs138940568	32536635	C	0.4195	1.29E-10	chr:6:32505702
6	rs556948697	32531729	T	0.353	1.29E-10	chr:6:32505702
6	rs184981897	32486966	A	2.255	1.30E-10	chr:6:32505702
6	rs143742917	32533480	G	0.4055	1.30E-10	chr:6:32505702

6	rs9269281	32539309	G	0.3523	1.30E-10	chr:6:32505702
6	rs35479797	32535900	C	0.3407	1.36E-10	chr:6:32505702
6	rs151210153	32531734	G	0.3528	1.36E-10	chr:6:32505702
6	rs115980757	32536914	A	0.4184	1.36E-10	chr:6:32505702
6	rs9270004	32552795	G	0.2904	1.38E-10	chr:6:32505702
6	rs116017457	32521909	T	2.546	1.40E-10	chr:6:32505702
6	rs113908137	32523500	A	0.3247	1.40E-10	chr:6:32505702
6	rs34166207	32523510	G	0.3247	1.40E-10	chr:6:32505702
6	rs111425203	32534872	C	0.3979	1.40E-10	chr:6:32505702
6	rs371994404	32532317	G	0.4085	1.41E-10	chr:6:32505702
6	rs575209356	32525806	G	0.4459	1.43E-10	chr:6:32505702
6	rs113737511	32533392	A	0.3852	1.43E-10	chr:6:32505702
6	rs72492335	32526460	T	0.383	1.45E-10	chr:6:32505702
6	rs142592433	32511392	C	0.2941	1.51E-10	chr:6:32505702
6	rs138129967	32532324	G	0.4086	1.51E-10	chr:6:32505702
6	rs9270533	32559709	C	0.3658	1.52E-10	chr:6:32505702
6	rs115018431	32534360	A	0.402	1.53E-10	chr:6:32505702
6	rs146783438	32533483	C	0.4076	1.54E-10	chr:6:32505702
6	rs148743468	32533479	T	0.4076	1.54E-10	chr:6:32505702
6	rs9269921	32551568	C	0.3864	1.58E-10	chr:6:32505702
6	rs9270540	32559848	T	0.3319	1.61E-10	chr:6:32505702
6	rs545853065	32531737	A	0.3583	1.63E-10	chr:6:32505702
6	rs140569663	32530944	G	0.3869	1.64E-10	chr:6:32505702
6	rs149685121	32530946	T	0.3869	1.64E-10	chr:6:32505702
6	rs72845756	32530545	T	0.3086	1.64E-10	chr:6:32505702
6	rs111338570	32533670	T	0.32	1.67E-10	chr:6:32505702
6	rs113042858	32533666	C	0.32	1.67E-10	chr:6:32505702
6	rs139013960	32531606	C	0.3235	1.71E-10	chr:6:32505702
6	rs186325882	32499990	A	2.007	1.72E-10	chr:6:32505702
6	rs72492344	32532494	G	0.3327	1.74E-10	chr:6:32505702
6	rs72492341	32531636	C	0.3062	1.76E-10	chr:6:32505702
6	rs112999094	32511386	C	0.296	1.77E-10	chr:6:32505702
6	rs139868705	32532075	C	0.3947	1.80E-10	chr:6:32505702
6	rs142162694	32532071	C	0.3947	1.80E-10	chr:6:32505702
6	rs115277180	32533468	T	0.4079	1.81E-10	chr:6:32505702
6	rs112719175	32533401	C	0.3938	1.83E-10	chr:6:32505702
6	rs199893280	32531226	A	0.3587	1.83E-10	chr:6:32505702
6	rs9269506	32543187	G	0.2863	1.87E-10	chr:6:32505702
6	rs187085470	32533998	T	0.4301	1.89E-10	chr:6:32505702
6	rs9269279	32539274	A	0.3616	1.90E-10	chr:6:32505702
6	rs111938555	32509707	T	2.528	1.97E-10	chr:6:32505702
6	rs78107833	32531807	A	0.3327	2.02E-10	chr:6:32505702
6	rs142891616	32534325	C	0.4317	2.05E-10	chr:6:32505702

6	rs544574563	32534337	A	0.4317	2.05E-10	chr:6:32505702
6	rs9269508	32543248	C	0.2701	2.06E-10	chr:6:32505702
6	rs182767311	32533523	T	0.4272	2.06E-10	chr:6:32505702
6	rs77178107	32533511	A	0.4209	2.07E-10	chr:6:32505702
6	rs200402299	32458349	T	0.4336	2.10E-10	chr:6:32505702
6	rs116152468	32536921	C	0.4157	2.10E-10	chr:6:32505702
6	rs115711695	32495994	A	2.072	2.12E-10	chr:6:32505702
6	rs114452447	32532262	G	0.4396	2.13E-10	chr:6:32505702
6	rs543107154	32532351	T	0.4097	2.17E-10	chr:6:32505702
6	rs561232678	32532352	G	0.4097	2.17E-10	chr:6:32505702
6	rs569119670	32532359	T	0.4097	2.17E-10	chr:6:32505702
6	rs201766754	32531843	A	0.3607	2.18E-10	chr:6:32505702
6	rs9269280	32539279	T	0.3616	2.21E-10	chr:6:32505702
6	rs76415310	32513232	T	2.525	2.23E-10	chr:6:32505702
6	rs182890820	32527218	T	2.286	2.31E-10	chr:6:32505702
6	rs9270154	32555389	A	0.364	2.33E-10	chr:6:32505702
6	rs138991250	32534855	A	0.413	2.35E-10	chr:6:32505702
6	rs193053660	32530503	G	0.341	2.40E-10	chr:6:32505702
6	rs533498287	32530506	T	0.341	2.40E-10	chr:6:32505702
6	rs566699769	32530505	C	0.341	2.40E-10	chr:6:32505702
6	rs115156211	32533440	C	0.4033	2.43E-10	chr:6:32505702
6	rs113655203	32533247	C	0.3328	2.43E-10	chr:6:32505702
6	rs9270597	32561598	T	0.2903	2.44E-10	chr:6:32505702
6	rs115232480	32536206	A	0.3636	2.44E-10	chr:6:32505702
6	rs78918008	32532281	G	0.4347	2.47E-10	chr:6:32505702
6	rs77814554	32533509	T	0.4245	2.51E-10	chr:6:32505702
6	rs189233809	32530497	A	0.3409	2.54E-10	chr:6:32505702
6	rs9269873	32550796	C	0.2762	2.59E-10	chr:6:32505702
6	rs552472567	32533447	A	0.4034	2.63E-10	chr:6:32505702
6	rs570478088	32533448	G	0.4034	2.63E-10	chr:6:32505702
6	rs9270535	32559714	G	0.3729	2.66E-10	chr:6:32505702
6	rs190614023	32500626	G	2.057	2.73E-10	chr:6:32505702
6	rs9270387	32558317	T	0.2992	2.76E-10	chr:6:32505702
6	rs9270559	32560698	T	0.2873	2.84E-10	chr:6:32505702
6	rs72844157	32520071	A	0.2476	2.91E-10	chr:6:32505702
6	rs145219524	32533964	A	0.4356	2.91E-10	chr:6:32505702
6	rs533614924	32532227	T	0.4407	2.92E-10	chr:6:32505702
6	rs145245177	32530955	G	0.3879	2.94E-10	chr:6:32505702
6	rs145520715	32530952	C	0.3879	2.94E-10	chr:6:32505702
6	rs148812784	32530954	T	0.3879	2.94E-10	chr:6:32505702
6	rs537686051	32531213	C	0.3846	2.97E-10	chr:6:32505702
6	rs552586675	32531215	T	0.3846	2.97E-10	chr:6:32505702
6	rs571171259	32531218	A	0.3846	2.97E-10	chr:6:32505702

6	rs138912409	32533684	C	0.35	2.98E-10	chr:6:32505702
6	rs143506542	32533686	G	0.35	2.98E-10	chr:6:32505702
6	rs75503609	32530929	G	0.3958	3.03E-10	chr:6:32505702
6	rs113204386	32531339	A	0.3297	3.03E-10	chr:6:32505702
6	rs75404103	32531348	T	0.3297	3.03E-10	chr:6:32505702
6	rs9270504	32559184	C	0.301	3.08E-10	chr:6:32505702
6	rs114451225	32538025	T	0.387	3.09E-10	chr:6:32505702
6	rs9269481	32542582	G	0.2547	3.13E-10	chr:6:32505702
6	rs116299701	32534068	G	0.4493	3.15E-10	chr:6:32505702
6	rs73403117	32468956	T	2.191	3.25E-10	chr:6:32505702
6	rs534655111	32531220	A	0.3848	3.25E-10	chr:6:32505702
6	rs201648725	32538024	T	0.387	3.29E-10	chr:6:32505702
6	rs181939518	32533977	T	0.4364	3.31E-10	chr:6:32505702
6	rs9270317	32557643	G	0.2799	3.34E-10	chr:6:32505702
6	rs9270524	32559588	G	0.3839	3.34E-10	chr:6:32505702
6	rs9270003	32552779	C	0.299	3.43E-10	chr:6:32505702
6	rs34966632	32536306	A	0.3914	3.43E-10	chr:6:32505702
6	rs73727909	32496036	T	2.074	3.44E-10	chr:6:32505702
6	rs9269237	32453583	C	0.3178	3.68E-10	chr:6:32505702
6	rs9269574	32544711	T	0.3774	3.72E-10	chr:6:32505702
6	rs66501603	32538002	G	0.3915	3.77E-10	chr:6:32505702
6	rs9270503	32559181	C	0.3031	3.77E-10	chr:6:32505702
6	rs12665549	32517141	A	2.502	3.82E-10	chr:6:32505702
6	rs536708130	32518949	G	2.658	3.90E-10	chr:6:32505702
6	rs74997284	32518934	G	2.658	3.90E-10	chr:6:32505702
6	rs78326230	32518935	A	2.658	3.90E-10	chr:6:32505702
6	rs189832895	32533972	C	0.4408	4.06E-10	chr:6:32505702
6	rs575033700	32533973	C	0.4408	4.06E-10	chr:6:32505702
6	rs199798245	32530708	T	0.3772	4.10E-10	chr:6:32505702
6	rs200856733	32530709	G	0.3772	4.10E-10	chr:6:32505702
6	rs543318785	32530714	T	0.3772	4.10E-10	chr:6:32505702
6	rs572746069	32530713	G	0.3772	4.10E-10	chr:6:32505702
6	rs9270541	32559864	G	0.3594	4.11E-10	chr:6:32505702
6	rs9270599	32561656	G	0.2834	4.23E-10	chr:6:32505702
6	rs7771747	32498931	G	0.4576	4.28E-10	chr:6:32505702
6	rs533779530	32453612	G	2.742	4.31E-10	chr:6:32505702
6	rs372618919	32529918	T	0.395	4.32E-10	chr:6:32505702
6	rs201024350	32536869	T	0.4046	4.34E-10	chr:6:32505702
6	rs9270115	32554934	C	0.3036	4.52E-10	chr:6:32505702
6	rs76533559	32554897	C	0.3509	4.79E-10	chr:6:32505702
6	rs185436529	32530740	C	0.394	4.84E-10	chr:6:32505702
6	rs531336617	32530792	T	0.4307	4.95E-10	chr:6:32505702
6	rs549516674	32530793	G	0.4307	4.95E-10	chr:6:32505702

6	rs567265210	32530790	C	0.4307	4.95E-10	chr:6:32505702
6	rs112009323	32532001	T	0.434	4.99E-10	chr:6:32505702
6	rs112562347	32532004	C	0.434	4.99E-10	chr:6:32505702
6	rs149581890	32530684	T	0.3405	5.00E-10	chr:6:32505702
6	rs11757123	32520094	T	0.3848	5.00E-10	chr:6:32505702
6	rs202075303	32496326	T	2.111	5.15E-10	chr:6:32505702
6	rs12661707	32531245	C	0.4296	5.22E-10	chr:6:32505702
6	rs193192981	32533553	A	0.4477	5.37E-10	chr:6:32505702
6	rs9269272	32539114	G	0.3477	5.46E-10	chr:6:32505702
6	rs543726509	32493208	G	0.3675	5.53E-10	chr:6:32505702
6	rs76666064	32530841	G	0.4299	5.71E-10	chr:6:32505702
6	rs115774308	32530778	G	0.423	5.85E-10	chr:6:32505702
6	rs138296162	32536852	A	0.4068	5.90E-10	chr:6:32505702
6	rs557163190	32526172	C	0.4467	5.98E-10	chr:6:32505702
6	rs369369329	32533784	T	0.452	6.06E-10	chr:6:32505702
6	rs9269651	32546074	C	0.2703	6.09E-10	chr:6:32505702
6	rs74207277	32560091	G	0.4382	6.28E-10	chr:6:32505702
6	rs112882980	32533222	G	0.359	6.92E-10	chr:6:32505702
6	rs115742569	32516464	C	3.334	7.04E-10	chr:6:32505702
6	rs185884622	32526169	T	0.4418	7.14E-10	chr:6:32505702
6	rs9270520	32559493	A	0.3033	7.35E-10	chr:6:32505702
6	rs147315656	32530869	A	0.4247	7.38E-10	chr:6:32505702
6	rs145896370	32533718	C	0.4095	7.58E-10	chr:6:32505702
6	rs563246799	32533766	A	0.4599	7.69E-10	chr:6:32505702
6	rs72847615	32536863	G	0.4076	7.83E-10	chr:6:32505702
6	rs73729129	32520092	T	0.2578	7.84E-10	chr:6:32505702
6	rs9270555	32560584	C	0.2944	7.90E-10	chr:6:32505702
6	rs9269879	32550906	A	0.3309	7.93E-10	chr:6:32505702
6	rs116609988	32522988	A	2.463	7.95E-10	chr:6:32505702
6	rs552961071	32533756	C	0.4612	8.22E-10	chr:6:32505702
6	rs574369672	32533757	A	0.4612	8.22E-10	chr:6:32505702
6	rs112621731	32501245	G	2.303	8.25E-10	chr:6:32505702
6	rs575744181	32533771	G	0.4602	8.33E-10	chr:6:32505702
6	rs34645444	32522634	T	0.2508	8.52E-10	chr:6:32505702
6	rs116748457	32514768	A	0.3407	8.52E-10	chr:6:32505702
6	rs116490940	32503056	T	2.183	8.64E-10	chr:6:32505702
6	rs68152293	32561020	C	0.4361	9.14E-10	chr:6:32505702
6	rs9269636	32545772	A	0.266	9.22E-10	chr:6:32505702
6	rs528792740	32533803	A	0.4584	9.22E-10	chr:6:32505702
6	rs546913114	32533805	A	0.4584	9.22E-10	chr:6:32505702
6	rs532508742	32530749	T	0.4105	9.26E-10	chr:6:32505702
6	rs9270525	32559595	A	0.4101	9.31E-10	chr:6:32505702
6	rs9270526	32559597	G	0.4101	9.31E-10	chr:6:32505702

6	rs9270527	32559600	G	0.4101	9.31E-10	chr:6:32505702
6	rs9270528	32559603	T	0.4101	9.31E-10	chr:6:32505702
6	rs114293611	32487353	T	0.4183	9.43E-10	chr:6:32505702
6	rs116551340	32527461	C	2.653	9.52E-10	chr:6:32505702
6	rs9270276	32557240	A	0.2785	9.56E-10	chr:6:32505702
6	rs536343459	32468799	C	0.4316	9.62E-10	chr:6:32505702
6	rs565374501	32468798	C	0.4316	9.62E-10	chr:6:32505702
6	rs183667786	32533749	T	0.4623	9.92E-10	chr:6:32505702
6	rs558887953	32533750	G	0.4623	9.92E-10	chr:6:32505702
6	rs73729339	32560017	C	0.4402	9.99E-10	chr:6:32505702
6	rs545154111	32530754	T	0.4107	9.99E-10	chr:6:32505702
6	rs111769716	32530819	C	0.4378	1.00E-09	chr:6:32505702
6	rs562082330	32533812	A	0.4589	1.01E-09	chr:6:32505702
6	rs151297998	32530904	T	0.4273	1.03E-09	chr:6:32505702
6	rs548006827	32468802	T	0.4319	1.04E-09	chr:6:32505702
6	rs9269813	32549681	G	0.2392	1.05E-09	chr:6:32505702
6	rs79638048	32519391	A	2.431	1.06E-09	chr:6:32505702
6	rs72492350	32538088	A	0.3655	1.06E-09	chr:6:32505702
6	rs9269635	32545720	A	0.2679	1.06E-09	chr:6:32505702
6	rs561942189	32496432	T	0.4269	1.09E-09	chr:6:32505702
6	rs558696616	32530898	T	0.4325	1.13E-09	chr:6:32505702
6	rs577240453	32530899	G	0.4325	1.13E-09	chr:6:32505702
6	rs143426018	32536888	A	0.4231	1.15E-09	chr:6:32505702
6	rs72845725	32525566	T	0.4168	1.15E-09	chr:6:32505702
6	rs9270485	32559064	T	0.351	1.15E-09	chr:6:32505702
6	rs576810845	32530891	C	0.4329	1.17E-09	chr:6:32505702
6	rs115493349	32527153	G	2.337	1.19E-09	chr:6:32505702
6	rs73729340	32560155	G	0.4483	1.21E-09	chr:6:32505702
6	rs9269392	32541163	T	0.4374	1.21E-09	chr:6:32505702
6	rs541874222	32533761	T	0.4669	1.21E-09	chr:6:32505702
6	rs551009500	32533836	C	0.4599	1.23E-09	chr:6:32505702
6	rs569299936	32533837	G	0.4599	1.23E-09	chr:6:32505702
6	rs34539464	32560339	A	0.4499	1.25E-09	chr:6:32505702
6	rs72492347	32533142	G	2.114	1.28E-09	chr:6:32505702
6	rs141503043	32485915	T	2.698	1.30E-09	chr:6:32505702
6	rs146222234	32485916	G	2.698	1.30E-09	chr:6:32505702
6	rs115573308	32533118	T	2.113	1.30E-09	chr:6:32505702
6	rs114418909	32531852	T	0.413	1.38E-09	chr:6:32505702
6	rs116479032	32531853	G	0.413	1.38E-09	chr:6:32505702
6	rs140527559	32536500	A	0.4417	1.39E-09	chr:6:32505702
6	rs147611303	32536497	G	0.4417	1.39E-09	chr:6:32505702
6	rs9270080	32554197	T	0.3581	1.40E-09	chr:6:32505702
6	rs145319447	32536492	T	0.4418	1.40E-09	chr:6:32505702

6	rs75019606	32531924	A	0.4561	1.42E-09	chr:6:32505702
6	rs541505856	32490394	C	0.4576	1.44E-09	chr:6:32505702
6	rs189557518	32526205	G	0.462	1.45E-09	chr:6:32505702
6	rs116659316	32527386	A	0.2944	1.45E-09	chr:6:32505702
6	rs72492338	32527408	G	2.817	1.47E-09	chr:6:32505702
6	rs188327097	32533734	T	0.4658	1.48E-09	chr:6:32505702
6	rs144064415	32536140	C	0.3781	1.49E-09	chr:6:32505702
6	rs72492329	32521136	A	3.327	1.51E-09	chr:6:32505702
6	rs113725551	32511420	A	0.3074	1.51E-09	chr:6:32505702
6	rs73726177	32490762	C	0.3737	1.53E-09	chr:6:32505702
6	rs146382271	32511291	T	2.206	1.56E-09	chr:6:32505702
6	rs559903629	32510984	A	2.032	1.57E-09	chr:6:32505702
6	rs71542414	32560247	A	0.4511	1.62E-09	chr:6:32505702
6	rs9270505	32559216	A	0.3078	1.64E-09	chr:6:32505702
6	rs111529629	32533843	A	0.4613	1.70E-09	chr:6:32505702
6	rs115055593	32468827	T	0.4398	1.73E-09	chr:6:32505702
6	rs71542413	32560240	C	0.4517	1.79E-09	chr:6:32505702
6	rs9269284	32539381	A	0.4064	1.80E-09	chr:6:32505702
6	rs28366259	32559516	T	0.3236	1.80E-09	chr:6:32505702
6	rs112479577	32533856	C	0.4604	1.81E-09	chr:6:32505702
6	rs71542415	32560298	T	0.4565	1.82E-09	chr:6:32505702
6	rs17203839	32555926	C	3.71	1.84E-09	chr:6:32505702
6	rs36207014	32560332	G	0.4565	1.85E-09	chr:6:32505702
6	rs34376317	32536678	T	2.3	1.96E-09	chr:6:32505702
6	rs527285388	32535496	T	2.044	1.97E-09	chr:6:32505702
6	rs74921648	32560307	G	0.4569	1.99E-09	chr:6:32505702
6	rs628754	32574787	G	0.3908	2.03E-09	chr:6:32505702
6	rs9270861	32570979	G	0.4127	2.07E-09	chr:6:32505702
6	rs9270862	32570981	T	0.4127	2.07E-09	chr:6:32505702
6	rs9270863	32570982	G	0.4127	2.07E-09	chr:6:32505702
6	rs34265294	32489118	G	0.4001	2.09E-09	chr:6:32505702
6	rs9269293	32539463	A	0.4148	2.15E-09	chr:6:32505702
6	rs11758321	32533413	G	2.089	2.21E-09	chr:6:32505702
6	rs9269393	32541222	G	0.4465	2.26E-09	chr:6:32505702
6	rs114020553	32527480	A	0.3956	2.27E-09	chr:6:32505702
6	rs538174489	32508201	T	1.989	2.32E-09	chr:6:32505702
6	rs76483802	32454913	A	0.3884	2.36E-09	chr:6:32505702
6	rs540141541	32487480	G	2.346	2.43E-09	chr:6:32505702
6	rs150860760	32486709	T	0.3816	2.45E-09	chr:6:32505702
6	rs201840950	32487091	G	3.212	2.47E-09	chr:6:32505702
6	rs201917227	32531870	A	0.4482	2.48E-09	chr:6:32505702
6	rs73726176	32490760	A	0.3833	2.53E-09	chr:6:32505702
6	rs111631740	32518161	G	0.2692	2.55E-09	chr:6:32505702

6	rs9270171	32555685	A	0.2875	2.55E-09	chr:6:32505702
6	rs9270172	32555687	G	0.2875	2.55E-09	chr:6:32505702
6	rs34824555	32525680	C	0.3419	2.62E-09	chr:6:32505702
6	rs116160472	32485643	G	2.094	2.66E-09	chr:6:32505702
6	rs369376349	32458425	G	0.4248	2.67E-09	chr:6:32505702
6	rs111705290	32513412	A	2.372	2.70E-09	chr:6:32505702
6	rs183231933	32524320	C	0.475	2.75E-09	chr:6:32505702
6	rs141124032	32533299	A	0.4261	2.82E-09	chr:6:32505702
6	rs9270743	32568242	A	0.3386	2.85E-09	chr:6:32505702
6	rs185087225	32480024	C	0.4777	2.90E-09	chr:6:32505702
6	rs138636844	32486961	A	2.256	3.00E-09	chr:6:32505702
6	rs72844120	32514540	A	2.318	3.04E-09	chr:6:32505702
6	rs9269326	32539996	A	0.351	3.18E-09	chr:6:32505702
6	rs146280841	32533291	T	0.427	3.23E-09	chr:6:32505702
6	rs148731245	32533293	T	0.427	3.23E-09	chr:6:32505702
6	rs143321288	32531487	G	0.4014	3.25E-09	chr:6:32505702
6	rs9270481	32559045	T	0.3658	3.30E-09	chr:6:32505702
6	rs9270474	32559003	A	0.3717	3.31E-09	chr:6:32505702
6	rs144230348	32512832	G	0.4812	3.54E-09	chr:6:32505702
6	rs533688361	32480016	A	0.4789	3.55E-09	chr:6:32505702
6	rs9269580	32544784	C	0.3757	3.66E-09	chr:6:32505702
6	rs9270034	32553418	T	0.3368	3.74E-09	chr:6:32505702
6	rs566973051	32531876	T	0.4559	3.86E-09	chr:6:32505702
6	rs9269582	32544787	C	0.3958	3.89E-09	chr:6:32505702
6	rs539796570	32526182	A	0.4553	3.92E-09	chr:6:32505702
6	rs142198554	32531887	C	0.4664	4.14E-09	chr:6:32505702
6	rs546856170	32531888	A	0.4664	4.14E-09	chr:6:32505702
6	rs139029897	32454882	A	0.4462	4.26E-09	chr:6:32505702
6	rs9270782	32569119	A	0.4192	4.32E-09	chr:6:32505702
6	rs71542412	32560190	T	0.4584	4.36E-09	chr:6:32505702
6	rs9269310	32539628	G	0.4344	4.46E-09	chr:6:32505702
6	rs679243	32570959	C	0.3884	4.49E-09	chr:6:32505702
6	rs111290332	32533327	G	0.4313	4.58E-09	chr:6:32505702
6	rs28584364	32512048	C	0.3106	4.62E-09	chr:6:32505702
6	rs149234868	32454921	A	0.4393	4.81E-09	chr:6:32505702
6	rs74208414	32501649	G	2.288	4.99E-09	chr:6:32505702
6	rs112348509	32523585	T	0.3413	5.03E-09	chr:6:32505702
6	rs149088066	32453495	C	0.2638	5.07E-09	chr:6:32505702
6	rs79140398	32527093	C	2.591	5.09E-09	chr:6:32505702
6	rs115833135	32489672	A	2.77	5.11E-09	chr:6:32505702
6	rs9270157	32555411	C	0.3099	5.33E-09	chr:6:32505702
6	rs114758692	32523431	G	0.3046	5.44E-09	chr:6:32505702
6	rs115010858	32523430	T	0.3046	5.44E-09	chr:6:32505702

6	rs568869869	32502799	G	1.993	5.48E-09	chr:6:32505702
6	rs116640755	32532556	G	0.4075	5.53E-09	chr:6:32505702
6	rs9269629	32545635	G	0.329	5.59E-09	chr:6:32505702
6	rs9269630	32545644	C	0.329	5.59E-09	chr:6:32505702
6	rs28613510	32518507	G	0.3245	5.61E-09	chr:6:32505702
6	rs191442117	32525886	T	0.471	5.62E-09	chr:6:32505702
6	rs9378212	32445691	T	0.2703	5.69E-09	chr:6:32505702
6	rs34452456	32450678	C	0.2455	5.75E-09	chr:6:32505702
6	rs9269501	32543118	C	0.3276	5.77E-09	chr:6:32505702
6	rs561017744	32547528	C	0.4109	5.89E-09	chr:6:32505702
6	rs115627609	32485579	T	2.038	5.90E-09	chr:6:32505702
6	rs72492339	32527472	G	0.4062	5.92E-09	chr:6:32505702
6	rs72508441	32490752	G	0.3738	6.00E-09	chr:6:32505702
6	rs34041546	32522872	C	0.2868	6.01E-09	chr:6:32505702
6	rs148866368	32531582	A	2.034	6.09E-09	chr:6:32505702
6	rs181190479	32533905	A	0.4832	6.10E-09	chr:6:32505702
6	rs188265524	32533903	A	0.4832	6.10E-09	chr:6:32505702
6	rs647467	32576726	T	2.32	6.23E-09	chr:6:32505702
6	rs72844196	32522843	C	0.2868	6.31E-09	chr:6:32505702
6	rs116542866	32521907	T	0.2917	6.40E-09	chr:6:32505702
6	rs9269292	32539450	C	0.4325	6.52E-09	chr:6:32505702
6	rs142886879	32497484	C	2.032	6.62E-09	chr:6:32505702
6	rs73727917	32497510	A	2.032	6.71E-09	chr:6:32505702
6	rs551270191	32453854	T	2.715	6.76E-09	chr:6:32505702
6	rs553177248	32529916	A	2.053	6.78E-09	chr:6:32505702
6	rs647455	32576721	A	2.317	6.83E-09	chr:6:32505702
6	rs531476760	32490069	C	2.019	6.94E-09	chr:6:32505702
6	rs540907103	32525757	T	0.4621	6.97E-09	chr:6:32505702
6	rs111823263	32482045	T	0.4735	6.97E-09	chr:6:32505702
6	rs554443954	32496557	C	0.4494	7.08E-09	chr:6:32505702
6	rs112507860	32496083	G	0.4968	7.13E-09	chr:6:32505702
6	rs34801804	32555892	G	4.484	7.15E-09	chr:6:32505702
6	rs73729756	32482020	C	0.4637	7.15E-09	chr:6:32505702
6	rs115022629	32534590	A	0.3449	7.24E-09	chr:6:32505702
6	rs72844197	32523020	T	0.2892	7.34E-09	chr:6:32505702
6	rs116541446	32497494	T	2.029	7.45E-09	chr:6:32505702
6	rs35767951	32522982	C	0.2893	7.49E-09	chr:6:32505702
6	rs72492323	32519715	G	0.2966	7.50E-09	chr:6:32505702
6	rs71536568	32531151	G	0.3608	7.60E-09	chr:6:32505702
6	rs73728000	32512552	C	0.2875	7.63E-09	chr:6:32505702
6	rs112219021	32518085	C	0.2743	7.69E-09	chr:6:32505702
6	rs112940468	32536389	G	0.3593	7.72E-09	chr:6:32505702
6	rs34559116	32536304	T	0.3864	7.72E-09	chr:6:32505702

6	rs114769669	32532149	G	0.3999	7.91E-09	chr:6:32505702
6	rs9270657	32566021	T	0.3763	8.04E-09	chr:6:32505702
6	rs77712892	32503570	T	2.195	8.12E-09	chr:6:32505702
6	rs11751146	32514899	T	2.063	8.14E-09	chr:6:32505702
6	rs139849303	32511307	G	0.3613	8.17E-09	chr:6:32505702
6	rs146103980	32493373	G	2.561	8.24E-09	chr:6:32505702
6	rs2760994	32574308	C	0.3136	8.32E-09	chr:6:32505702
6	rs72508426	32485598	G	2.026	8.34E-09	chr:6:32505702
6	rs72851018	32501379	C	0.2829	8.34E-09	chr:6:32505702
6	rs111672423	32523581	A	0.3459	8.40E-09	chr:6:32505702
6	rs34668202	32523477	A	0.3319	8.53E-09	chr:6:32505702
6	rs148573031	32530691	T	0.4601	8.55E-09	chr:6:32505702
6	rs549042477	32547505	A	0.4279	8.76E-09	chr:6:32505702
6	rs111774342	32532112	C	0.3907	8.80E-09	chr:6:32505702
6	rs9269998	32552721	A	0.4321	9.20E-09	chr:6:32505702
6	rs552461365	32482115	C	1.95	9.31E-09	chr:6:32505702
6	rs150598903	32489395	A	0.4556	9.42E-09	chr:6:32505702
6	rs115333128	32485804	T	0.4713	9.52E-09	chr:6:32505702
6	rs72853909	32505798	G	0.3847	9.52E-09	chr:6:32505702
6	rs144415016	32511298	A	0.3724	9.53E-09	chr:6:32505702
6	rs67534237	32513587	G	0.2817	9.68E-09	chr:6:32505702
6	rs73727927	32501214	A	0.3681	9.76E-09	chr:6:32505702
6	rs190202109	32525736	A	0.4587	1.01E-08	chr:6:32505702
6	rs572428148	32532532	A	0.4162	1.01E-08	chr:6:32505702
6	rs9270806	32569563	C	0.3768	1.01E-08	chr:6:32505702
6	rs9270807	32569572	G	0.3768	1.01E-08	chr:6:32505702
6	rs9270787	32569174	C	0.4247	1.02E-08	chr:6:32505702
6	rs187058069	32486665	C	0.4687	1.03E-08	chr:6:32505702
6	rs9270884	32571734	T	0.426	1.04E-08	chr:6:32505702
6	rs561623787	32452602	T	0.268	1.04E-08	chr:6:32505702
6	rs191239160	32525992	G	0.4874	1.07E-08	chr:6:32505702
6	rs476775	32569722	G	0.3747	1.09E-08	chr:6:32505702
6	rs476602	32569792	T	0.3746	1.10E-08	chr:6:32505702
6	rs9269673	32546447	C	0.3005	1.11E-08	chr:6:32505702
6	rs41287328	32552698	G	2.242	1.12E-08	chr:6:32505702
6	rs1136756	32552134	G	2.871	1.13E-08	chr:6:32505702
6	rs77087101	32534605	A	0.3565	1.16E-08	chr:6:32505702
6	rs547797725	32487827	G	0.4613	1.17E-08	chr:6:32505702
6	rs67704526	32536650	T	2.007	1.17E-08	chr:6:32505702
6	rs555503640	32486477	C	0.4939	1.18E-08	chr:6:32505702
6	rs11751496	32533549	T	1.921	1.20E-08	chr:6:32505702
6	rs9269286	32539396	G	0.4466	1.21E-08	chr:6:32505702
6	rs148620529	32525509	A	0.4803	1.21E-08	chr:6:32505702

6	rs72851098	32505477	T	0.3549	1.22E-08	chr:6:32505702
6	rs75999763	32523206	G	0.284	1.25E-08	chr:6:32505702
6	rs112996372	32517005	G	0.2832	1.25E-08	chr:6:32505702
6	rs115623335	32532578	A	0.4185	1.26E-08	chr:6:32505702
6	rs111372196	32516995	A	0.2829	1.28E-08	chr:6:32505702
6	rs115249219	32479677	C	0.4308	1.28E-08	chr:6:32505702
6	rs9270543	32559881	C	0.435	1.29E-08	chr:6:32505702
6	rs9270544	32559882	C	0.435	1.29E-08	chr:6:32505702
6	rs66931410	32518235	T	0.3298	1.29E-08	chr:6:32505702
6	rs140993369	32535968	G	0.3684	1.29E-08	chr:6:32505702
6	rs115005201	32501172	G	0.3813	1.31E-08	chr:6:32505702
6	rs673336	32577858	G	0.3851	1.31E-08	chr:6:32505702
6	rs116611418	32459595	A	0.4996	1.35E-08	chr:6:32505702
6	rs73397417	32521988	G	0.2734	1.36E-08	chr:6:32505702
6	rs139910587	32490483	A	0.4333	1.36E-08	chr:6:32505702
6	rs113369856	32532508	G	0.376	1.36E-08	chr:6:32505702
6	rs9270739	32568150	C	0.4106	1.37E-08	chr:6:32505702
6	rs9269697	32546953	G	0.2825	1.38E-08	chr:6:32505702
6	rs67672162	32503251	T	0.2992	1.38E-08	chr:6:32505702
6	rs113409465	32511143	T	2.116	1.39E-08	chr:6:32505702
6	rs113787140	32502901	T	0.4239	1.41E-08	chr:6:32505702
6	rs9270876	32571314	T	0.4243	1.41E-08	chr:6:32505702
6	rs7452937	32446484	A	0.2704	1.41E-08	chr:6:32505702
6	rs68148149	32511725	C	0.2741	1.42E-08	chr:6:32505702
6	rs138921502	32538132	A	0.3595	1.43E-08	chr:6:32505702
6	rs9271492	32589122	A	0.3885	1.45E-08	chr:6:32505702
6	rs11970219	32500925	C	2.189	1.48E-08	chr:6:32505702
6	rs115707905	32516930	G	0.2811	1.49E-08	chr:6:32505702
6	rs116184728	32516935	T	0.2811	1.49E-08	chr:6:32505702
6	rs67497288	32503221	A	0.2968	1.50E-08	chr:6:32505702
6	rs68174286	32503204	T	0.2968	1.50E-08	chr:6:32505702
6	rs34720636	32523260	C	0.2815	1.50E-08	chr:6:32505702
6	rs28732246	32447219	A	0.2721	1.51E-08	chr:6:32505702
6	rs29001620	32447216	T	0.2721	1.51E-08	chr:6:32505702
6	rs28895249	32444021	T	0.2716	1.53E-08	chr:6:32505702
6	rs115563246	32489734	A	2.68	1.54E-08	chr:6:32505702
6	rs9269381	32540927	T	0.4347	1.55E-08	chr:6:32505702
6	rs116531157	32547565	A	0.4049	1.56E-08	chr:6:32505702
6	rs67408570	32508874	C	0.3055	1.57E-08	chr:6:32505702
6	rs531128975	32497352	C	2.474	1.61E-08	chr:6:32505702
6	rs112512219	32532021	G	1.966	1.64E-08	chr:6:32505702
6	rs116830840	32537945	C	0.4822	1.65E-08	chr:6:32505702
6	rs9269493	32542990	T	0.2839	1.68E-08	chr:6:32505702

6	rs72844166	32520632	G	0.2799	1.68E-08	chr:6:32505702
6	rs114437875	32534943	C	0.4559	1.72E-08	chr:6:32505702
6	rs149521915	32489639	T	2.554	1.73E-08	chr:6:32505702
6	rs72844167	32520642	T	0.2791	1.74E-08	chr:6:32505702
6	rs77993032	32518093	C	0.2789	1.74E-08	chr:6:32505702
6	rs36088464	32513497	A	0.2788	1.75E-08	chr:6:32505702
6	rs111739306	32516848	T	0.2788	1.77E-08	chr:6:32505702
6	rs9269288	32539408	G	0.4553	1.78E-08	chr:6:32505702
6	rs9269484	32542711	C	0.2888	1.78E-08	chr:6:32505702
6	rs9269717	32547303	G	0.4293	1.78E-08	chr:6:32505702
6	rs72492345	32532615	T	0.4015	1.79E-08	chr:6:32505702
6	rs72853984	32511866	C	0.2794	1.79E-08	chr:6:32505702
6	rs66466672	32508913	A	0.304	1.84E-08	chr:6:32505702
6	rs71534519	32508893	A	0.304	1.84E-08	chr:6:32505702
6	rs67426377	32514457	C	0.2784	1.85E-08	chr:6:32505702
6	rs28732244	32444096	T	0.275	1.85E-08	chr:6:32505702
6	rs9270632	32565533	G	0.4073	1.85E-08	chr:6:32505702
6	rs185236820	32488776	A	0.4882	1.86E-08	chr:6:32505702
6	rs190526848	32488781	T	0.4882	1.86E-08	chr:6:32505702
6	rs559294646	32525834	T	0.4855	1.86E-08	chr:6:32505702
6	rs369885796	32511529	G	0.3326	1.87E-08	chr:6:32505702
6	rs2858873;rs531438466	32566769	G	0.3705	1.90E-08	chr:6:32505702
6	rs12194148	32444198	T	0.2746	1.90E-08	chr:6:32505702
6	rs12195589	32444584	T	0.2746	1.90E-08	chr:6:32505702
6	rs12207473	32444330	C	0.2746	1.90E-08	chr:6:32505702
6	rs13194665	32451526	G	0.2746	1.90E-08	chr:6:32505702
6	rs13211921	32442836	G	0.2746	1.90E-08	chr:6:32505702
6	rs17203619	32446922	C	0.2746	1.90E-08	chr:6:32505702
6	rs17203626	32446994	A	0.2746	1.90E-08	chr:6:32505702
6	rs17203636	32447014	A	0.2746	1.90E-08	chr:6:32505702
6	rs28895247	32443890	A	0.2746	1.90E-08	chr:6:32505702
6	rs28895255	32445079	T	0.2746	1.90E-08	chr:6:32505702
6	rs28895257	32445114	G	0.2746	1.90E-08	chr:6:32505702
6	rs28895258	32445117	T	0.2746	1.90E-08	chr:6:32505702
6	rs28895259	32445258	G	0.2746	1.90E-08	chr:6:32505702
6	rs28895264	32445792	T	0.2746	1.90E-08	chr:6:32505702
6	rs28895265	32445793	G	0.2746	1.90E-08	chr:6:32505702
6	rs28895266	32445799	G	0.2746	1.90E-08	chr:6:32505702
6	rs29001478	32447162	A	0.2746	1.90E-08	chr:6:32505702
6	rs29001568	32447341	A	0.2746	1.90E-08	chr:6:32505702
6	rs29001652	32447111	G	0.2746	1.90E-08	chr:6:32505702
6	rs4994854	32447900	C	0.2746	1.90E-08	chr:6:32505702
6	rs4994855	32447873	G	0.2746	1.90E-08	chr:6:32505702

6	rs554832027	32444708	A	0.2746	1.90E-08	chr:6:32505702
6	rs71543453	32452361	T	0.2746	1.90E-08	chr:6:32505702
6	rs9378213	32448398	G	0.2742	1.90E-08	chr:6:32505702
6	rs9378264	32443451	A	0.2746	1.90E-08	chr:6:32505702
6	rs9391879	32443172	T	0.2746	1.90E-08	chr:6:32505702
6	rs9391881	32443432	G	0.2746	1.90E-08	chr:6:32505702
6	rs72844114	32514030	G	0.2782	1.90E-08	chr:6:32505702
6	rs72492326	32520420	T	0.2782	1.90E-08	chr:6:32505702
6	rs68017248	32519262	A	0.2769	1.91E-08	chr:6:32505702
6	rs72851102	32505568	A	0.328	1.92E-08	chr:6:32505702
6	rs112582516	32481392	A	0.5269	1.93E-08	chr:6:32505702
6	rs35882239	32522576	A	0.2791	1.94E-08	chr:6:32505702
6	rs2516050	32570352	G	0.3849	1.94E-08	chr:6:32505702
6	rs538439966	32536033	T	0.417	1.95E-08	chr:6:32505702
6	rs553961126	32536034	A	0.417	1.95E-08	chr:6:32505702
6	rs572166536	32536035	T	0.417	1.95E-08	chr:6:32505702
6	rs9270796	32569293	G	0.4273	1.96E-08	chr:6:32505702
6	rs3828840	32520907	T	0.2779	1.96E-08	chr:6:32505702
6	rs9270894	32571872	A	0.4398	2.01E-08	chr:6:32505702
6	rs141563474	32536045	T	0.4152	2.09E-08	chr:6:32505702
6	rs71543454	32452399	A	0.2737	2.12E-08	chr:6:32505702
6	rs71543455	32452409	C	0.2737	2.12E-08	chr:6:32505702
6	rs79401736	32503843	A	0.3616	2.13E-08	chr:6:32505702
6	rs17203514	32446071	G	0.2758	2.14E-08	chr:6:32505702
6	rs17209887	32446051	C	0.2758	2.14E-08	chr:6:32505702
6	rs618095	32574736	G	0.431	2.15E-08	chr:6:32505702
6	rs28895242	32443666	C	0.2768	2.16E-08	chr:6:32505702
6	rs72844177	32520990	G	0.2766	2.16E-08	chr:6:32505702
6	rs111662020	32518444	A	0.3279	2.17E-08	chr:6:32505702
6	rs34207893	32447616	C	0.2715	2.18E-08	chr:6:32505702
6	rs67870913	32503258	T	0.3093	2.20E-08	chr:6:32505702
6	rs9270210	32556475	C	0.4272	2.20E-08	chr:6:32505702
6	rs4457186	32447653	G	0.2723	2.24E-08	chr:6:32505702
6	rs112080536	32521594	A	0.3112	2.24E-08	chr:6:32505702
6	rs34523106	32513724	C	0.2804	2.24E-08	chr:6:32505702
6	rs4410767	32448129	C	0.2763	2.25E-08	chr:6:32505702
6	rs17209866	32445992	A	0.2751	2.25E-08	chr:6:32505702
6	rs9270272	32557213	G	0.2827	2.26E-08	chr:6:32505702
6	rs566775437	32525993	G	0.4994	2.28E-08	chr:6:32505702
6	rs373289038	32535643	A	2.089	2.29E-08	chr:6:32505702
6	rs72851021	32501395	T	0.2961	2.29E-08	chr:6:32505702
6	rs9269579	32544772	G	0.3902	2.30E-08	chr:6:32505702
6	rs112532723	32518151	G	0.2972	2.30E-08	chr:6:32505702

6	rs4994859	32447715	G	0.2772	2.32E-08	chr:6:32505702
6	rs17203563	32446459	A	0.2775	2.33E-08	chr:6:32505702
6	rs34673795	32513988	G	0.2808	2.34E-08	chr:6:32505702
6	rs201211315	32525040	A	0.4653	2.37E-08	chr:6:32505702
6	rs9394099	32449160	T	0.2738	2.41E-08	chr:6:32505702
6	rs112403925	32525183	G	0.4587	2.42E-08	chr:6:32505702
6	rs149705942	32532078	T	0.4291	2.43E-08	chr:6:32505702
6	rs140747244	32488784	T	0.4898	2.43E-08	chr:6:32505702
6	rs79912140	32519729	A	0.3137	2.46E-08	chr:6:32505702
6	rs9270877	32571318	A	0.4332	2.46E-08	chr:6:32505702
6	rs9270878	32571320	G	0.4332	2.46E-08	chr:6:32505702
6	rs28662053	32453107	G	0.2749	2.50E-08	chr:6:32505702
6	rs62405566	32453095	T	0.2749	2.50E-08	chr:6:32505702
6	rs9269302	32539574	G	0.4593	2.53E-08	chr:6:32505702
6	rs72844134	32518196	G	0.2963	2.54E-08	chr:6:32505702
6	rs141056608	32537440	C	1.926	2.56E-08	chr:6:32505702
6	rs146946148	32537444	T	1.926	2.56E-08	chr:6:32505702
6	rs35772438	32530407	C	0.2719	2.57E-08	chr:6:32505702
6	rs9270302	32557479	A	3.459	2.58E-08	chr:6:32505702
6	rs35321667	32501534	C	0.2915	2.60E-08	chr:6:32505702
6	rs9269653	32546080	A	0.2983	2.60E-08	chr:6:32505702
6	rs77498463	32517198	T	0.2965	2.62E-08	chr:6:32505702
6	rs5020947	32450033	T	0.2723	2.69E-08	chr:6:32505702
6	rs5020946	32450089	T	0.2727	2.69E-08	chr:6:32505702
6	rs1964995	32449411	C	0.2733	2.70E-08	chr:6:32505702
6	rs72844151	32519650	A	0.2953	2.73E-08	chr:6:32505702
6	rs79881121	32502324	G	0.4271	2.75E-08	chr:6:32505702
6	rs112234874	32527439	G	0.3287	2.78E-08	chr:6:32505702
6	rs9269413	32541458	C	0.301	2.79E-08	chr:6:32505702
6	rs5018660	32449293	G	0.2748	2.80E-08	chr:6:32505702
6	rs112861810	32494029	T	0.4633	2.84E-08	chr:6:32505702
6	rs147034082	32539600	G	0.4599	2.85E-08	chr:6:32505702
6	rs9269965	32552219	G	2.298	2.86E-08	chr:6:32505702
6	rs374248993	32496534	G	0.4604	2.88E-08	chr:6:32505702
6	rs67587495	32518728	A	0.2943	3.00E-08	chr:6:32505702
6	rs5020944	32450140	G	0.2723	3.04E-08	chr:6:32505702
6	rs9269405	32541348	A	0.3596	3.06E-08	chr:6:32505702
6	rs2395192	32447644	T	0.2827	3.06E-08	chr:6:32505702
6	rs9269709	32547237	G	0.3664	3.07E-08	chr:6:32505702
6	rs73729117	32519600	A	0.294	3.20E-08	chr:6:32505702
6	rs62405573	32458239	G	0.4659	3.20E-08	chr:6:32505702
6	rs13194770	32451570	T	0.2712	3.21E-08	chr:6:32505702
6	rs377599643	32536107	C	0.4207	3.21E-08	chr:6:32505702

6	rs9269390	32541123	C	0.4249	3.23E-08	chr:6:32505702
6	rs9269708	32547232	C	0.3664	3.26E-08	chr:6:32505702
6	rs76566406	32518913	T	0.3141	3.28E-08	chr:6:32505702
6	rs5020950	32449965	C	0.2704	3.29E-08	chr:6:32505702
6	rs79268162	32517493	G	0.3104	3.30E-08	chr:6:32505702
6	rs9270423	32558580	G	0.4655	3.31E-08	chr:6:32505702
6	rs72492308	32509570	T	0.2937	3.32E-08	chr:6:32505702
6	rs560625026	32546591	C	0.2898	3.33E-08	chr:6:32505702
6	rs371747477	32489266	C	0.4147	3.35E-08	chr:6:32505702
6	rs371638245	32525191	G	0.4643	3.40E-08	chr:6:32505702
6	rs78279385	32451758	A	0.2698	3.40E-08	chr:6:32505702
6	rs9269586	32544844	T	0.3842	3.40E-08	chr:6:32505702
6	rs201885513	32512459	C	0.3803	3.42E-08	chr:6:32505702
6	rs549610120	32525308	G	0.4636	3.43E-08	chr:6:32505702
6	rs72853943	32509260	C	0.2939	3.43E-08	chr:6:32505702
6	rs28895254	32444946	T	0.2825	3.43E-08	chr:6:32505702
6	rs531127489	32453036	A	0.2652	3.44E-08	chr:6:32505702
6	rs552565118	32453037	T	0.2652	3.44E-08	chr:6:32505702
6	rs113260943	32509550	T	0.2934	3.46E-08	chr:6:32505702
6	rs112323850	32521804	T	0.3476	3.52E-08	chr:6:32505702
6	rs73395587	32518339	T	0.3117	3.56E-08	chr:6:32505702
6	rs9270631	32565505	A	0.4278	3.57E-08	chr:6:32505702
6	rs376459798	32535644	A	2.065	3.59E-08	chr:6:32505702
6	rs9269713	32547252	G	0.39	3.59E-08	chr:6:32505702
6	rs9269311	32539654	G	0.4474	3.59E-08	chr:6:32505702
6	rs9270869	32571155	T	0.425	3.60E-08	chr:6:32505702
6	rs9270871	32571160	A	0.425	3.60E-08	chr:6:32505702
6	rs75259234	32453338	T	0.2728	3.67E-08	chr:6:32505702
6	rs181664429	32525903	T	0.4961	3.71E-08	chr:6:32505702
6	rs72508418	32459868	C	0.431	3.71E-08	chr:6:32505702
6	rs9269969	32552272	C	2.547	3.72E-08	chr:6:32505702
6	rs28895261	32445306	A	0.2881	3.72E-08	chr:6:32505702
6	rs111485252	32518374	T	0.3115	3.74E-08	chr:6:32505702
6	rs28732247	32447474	G	0.2829	3.76E-08	chr:6:32505702
6	rs35007143	32447518	A	0.2829	3.76E-08	chr:6:32505702
6	rs9270626	32565443	G	0.4298	3.77E-08	chr:6:32505702
6	rs115187291	32525223	G	0.4661	3.80E-08	chr:6:32505702
6	rs116088388	32488961	G	0.4515	3.82E-08	chr:6:32505702
6	rs139007218	32488958	G	0.4515	3.82E-08	chr:6:32505702
6	rs72508422	32481498	G	0.5354	3.83E-08	chr:6:32505702
6	rs28895253	32444910	A	0.2835	3.89E-08	chr:6:32505702
6	rs9269562	32544407	A	0.2839	3.89E-08	chr:6:32505702
6	rs146123957	32525913	T	0.4919	3.93E-08	chr:6:32505702

6	rs185112674	32525915	A	0.4919	3.93E-08	chr:6:32505702
6	rs115729463	32514301	C	0.3043	3.94E-08	chr:6:32505702
6	rs79138148	32516351	C	0.2922	3.96E-08	chr:6:32505702
6	rs182191708	32490480	A	0.4527	3.97E-08	chr:6:32505702
6	rs547043452	32525326	G	0.4659	4.02E-08	chr:6:32505702
6	rs9270433	32558670	G	0.4844	4.06E-08	chr:6:32505702
6	rs9270773	32568990	A	0.4305	4.07E-08	chr:6:32505702
6	rs117090280	32489265	A	0.4751	4.08E-08	chr:6:32505702
6	rs576554734	32525219	C	0.4673	4.11E-08	chr:6:32505702
6	rs72844137	32518615	G	0.3108	4.11E-08	chr:6:32505702
6	rs9270803	32569415	G	0.4212	4.13E-08	chr:6:32505702
6	rs68109409	32518552	G	0.3107	4.14E-08	chr:6:32505702
6	rs74217355	32519018	A	0.3257	4.19E-08	chr:6:32505702
6	rs528864352	32452626	G	0.2785	4.19E-08	chr:6:32505702
6	rs182968054	32536067	T	0.4409	4.19E-08	chr:6:32505702
6	rs190321460	32536065	G	0.4409	4.19E-08	chr:6:32505702
6	rs184792502	32525876	C	0.4942	4.22E-08	chr:6:32505702
6	rs186639906	32525878	T	0.4942	4.22E-08	chr:6:32505702
6	rs659204	32577001	G	2.295	4.22E-08	chr:6:32505702
6	rs9270250	32557001	C	2.783	4.30E-08	chr:6:32505702
6	rs9270251	32557003	A	2.783	4.30E-08	chr:6:32505702
6	rs73727962	32509050	A	0.3109	4.30E-08	chr:6:32505702
6	rs76722140	32509053	C	0.3109	4.30E-08	chr:6:32505702
6	rs72845727	32525674	A	0.3717	4.31E-08	chr:6:32505702
6	rs77236357	32459844	G	0.4318	4.33E-08	chr:6:32505702
6	rs62405572	32458232	A	0.4617	4.36E-08	chr:6:32505702
6	rs72849274	32469068	T	0.2931	4.39E-08	chr:6:32505702
6	rs73729116	32519558	C	0.2919	4.41E-08	chr:6:32505702
6	rs9269291	32539445	G	0.4681	4.41E-08	chr:6:32505702
6	rs9269304	32539585	C	0.4679	4.44E-08	chr:6:32505702
6	rs9270046	32553683	A	0.3359	4.46E-08	chr:6:32505702
6	rs28454881	32451351	G	0.2714	4.56E-08	chr:6:32505702
6	rs529763782	32493737	C	0.4277	4.56E-08	chr:6:32505702
6	rs9380309	32449820	G	0.2726	4.59E-08	chr:6:32505702
6	rs9269874	32550828	G	0.287	4.59E-08	chr:6:32505702
6	rs9394098	32449138	T	0.2793	4.75E-08	chr:6:32505702
6	rs9269518	32543594	T	0.2937	4.78E-08	chr:6:32505702
6	rs67642277	32538067	G	0.4009	4.79E-08	chr:6:32505702
6	rs558057632	32526072	G	0.4891	4.86E-08	chr:6:32505702
6	rs76517385	32519067	A	0.3095	4.87E-08	chr:6:32505702
6	rs559109541	32493714	C	0.4319	4.93E-08	chr:6:32505702
6	rs72844190	32521785	T	0.3032	4.93E-08	chr:6:32505702
6	rs554833162	32525208	G	0.472	4.95E-08	chr:6:32505702

6	rs71536524	32520841	T	0.3559	5.07E-08	chr:6:32505702
6	rs545350177	32565668	T	0.4449	5.09E-08	chr:6:32505702
6	rs9391786	32448561	G	0.2684	5.11E-08	chr:6:32505702
6	rs192638533	32526085	T	0.4911	5.16E-08	chr:6:32505702
6	rs9269524	32543671	T	0.4095	5.20E-08	chr:6:32505702
6	rs35875942	32447563	C	0.2846	5.23E-08	chr:6:32505702
6	rs73403121	32469012	C	0.4117	5.25E-08	chr:6:32505702
6	rs62405645	32517934	G	0.303	5.34E-08	chr:6:32505702
6	rs148633289	32487229	T	2.626	5.40E-08	chr:6:32505702
6	rs9269783	32549001	A	0.2826	5.41E-08	chr:6:32505702
6	rs36189266	32526768	G	0.4131	5.43E-08	chr:6:32505702
6	rs9270728	32567956	T	0.4165	5.50E-08	chr:6:32505702
6	rs17203570	32446674	T	0.2831	5.52E-08	chr:6:32505702
6	rs111353892	32525928	T	0.492	5.56E-08	chr:6:32505702
6	rs547755130	32486547	T	0.5003	5.59E-08	chr:6:32505702
6	rs565956329	32486548	G	0.5003	5.59E-08	chr:6:32505702
6	rs78241440	32509180	G	0.3059	5.61E-08	chr:6:32505702
6	rs78650231	32525698	G	0.3942	5.65E-08	chr:6:32505702
6	rs182651216	32526060	G	0.4885	5.68E-08	chr:6:32505702
6	rs74528572	32525502	G	0.4988	5.69E-08	chr:6:32505702
6	rs76087961	32525501	A	0.4988	5.69E-08	chr:6:32505702
6	rs115772619	32519757	C	0.3472	5.73E-08	chr:6:32505702
6	rs9270658	32566072	T	0.4227	5.76E-08	chr:6:32505702
6	rs9270769	32568907	T	0.4199	5.79E-08	chr:6:32505702
6	rs9270770	32568910	T	0.4199	5.79E-08	chr:6:32505702
6	rs9270369	32558183	T	0.3269	5.84E-08	chr:6:32505702
6	rs74324607	32525646	A	0.385	5.90E-08	chr:6:32505702
6	rs116151010	32514763	G	0.3801	5.90E-08	chr:6:32505702
6	rs114924039	32514794	C	0.3801	5.95E-08	chr:6:32505702
6	rs9270642	32565707	G	0.4269	5.97E-08	chr:6:32505702
6	rs79251508	32453632	G	0.2806	6.01E-08	chr:6:32505702
6	rs146363280	32524765	A	0.4751	6.03E-08	chr:6:32505702
6	rs17209907	32446261	T	0.2807	6.07E-08	chr:6:32505702
6	rs188008023	32526074	T	0.4921	6.12E-08	chr:6:32505702
6	rs28732268	32548352	G	0.3088	6.21E-08	chr:6:32505702
6	rs541172392	32525830	T	0.4955	6.31E-08	chr:6:32505702
6	rs9269269	32539055	G	0.4717	6.42E-08	chr:6:32505702
6	rs116258010	32524772	A	0.4755	6.45E-08	chr:6:32505702
6	rs78987802	32503728	T	0.3858	6.47E-08	chr:6:32505702
6	rs9270374	32558216	G	0.3027	6.61E-08	chr:6:32505702
6	rs9269725	32547359	T	0.4562	6.61E-08	chr:6:32505702
6	rs188912067	32525865	G	0.505	6.61E-08	chr:6:32505702
6	rs36165851	32489981	G	0.492	6.68E-08	chr:6:32505702

6	rs72492336	32527112	C	0.3081	6.68E-08	chr:6:32505702
6	rs534705706	32562023	A	2.054	6.72E-08	chr:6:32505702
6	rs573046310	32525827	A	0.4958	6.73E-08	chr:6:32505702
6	rs565566670	32525229	C	0.4762	6.76E-08	chr:6:32505702
6	rs80048124	32503729	G	0.3857	6.79E-08	chr:6:32505702
6	rs67062152	32527025	T	0.3081	6.79E-08	chr:6:32505702
6	rs73401326	32454783	A	0.4395	6.91E-08	chr:6:32505702
6	rs541391839	32525244	T	0.4763	7.01E-08	chr:6:32505702
6	rs9269726	32547360	G	0.4564	7.03E-08	chr:6:32505702
6	rs9270634	32565625	C	0.4198	7.08E-08	chr:6:32505702
6	rs9270282	32557271	A	0.2817	7.08E-08	chr:6:32505702
6	rs9270621	32565283	G	0.4196	7.11E-08	chr:6:32505702
6	rs190044159	32525843	T	0.5014	7.11E-08	chr:6:32505702
6	rs9269968	32552251	G	0.4138	7.20E-08	chr:6:32505702
6	rs79346779	32453655	A	0.2827	7.21E-08	chr:6:32505702
6	rs116708952	32486590	T	0.4986	7.37E-08	chr:6:32505702
6	rs67738935	32521230	C	0.2695	7.47E-08	chr:6:32505702
6	rs9270436	32558693	C	0.5008	7.51E-08	chr:6:32505702
6	rs9269962	32552177	A	2.651	7.52E-08	chr:6:32505702
6	rs9270653	32565940	G	0.4198	7.55E-08	chr:6:32505702
6	rs9270654	32565946	T	0.4198	7.55E-08	chr:6:32505702
6	rs9270655	32565966	A	0.4198	7.55E-08	chr:6:32505702
6	rs535866271	32486668	C	0.4926	7.59E-08	chr:6:32505702
6	rs9270772	32568967	C	2.319	7.63E-08	chr:6:32505702
6	rs9269547	32544173	T	0.267	7.77E-08	chr:6:32505702
6	rs78387908	32503391	A	0.3922	7.81E-08	chr:6:32505702
6	rs74587827	32567757	G	0.4185	7.84E-08	chr:6:32505702
6	rs78896800	32525473	A	0.5037	7.84E-08	chr:6:32505702
6	rs115817940	32489735	T	2.344	7.84E-08	chr:6:32505702
6	rs143796398	32536259	T	1.929	7.87E-08	chr:6:32505702
6	rs559593099	32525759	T	0.4963	7.91E-08	chr:6:32505702
6	rs77469652	32453532	C	0.284	7.91E-08	chr:6:32505702
6	rs182032087	32525821	T	0.4967	8.06E-08	chr:6:32505702
6	rs79485855	32527253	T	0.4421	8.11E-08	chr:6:32505702
6	rs28895267	32445916	T	0.3292	8.13E-08	chr:6:32505702
6	rs370779031	32535688	A	2.037	8.19E-08	chr:6:32505702
6	rs375377896	32535693	A	2.037	8.19E-08	chr:6:32505702
6	rs111338793	32486248	C	0.5163	8.22E-08	chr:6:32505702
6	rs539141868	32524620	C	0.4944	8.23E-08	chr:6:32505702
6	rs9270651	32565884	C	0.4204	8.31E-08	chr:6:32505702
6	rs9270652	32565905	C	0.4204	8.31E-08	chr:6:32505702
6	rs113523485	32489302	C	2.044	8.40E-08	chr:6:32505702
6	rs9269296	32539509	G	1.982	8.40E-08	chr:6:32505702

6	rs116670844	32490925	T	1.884	8.43E-08	chr:6:32505702
6	rs200582841	32453810	A	2.721	8.46E-08	chr:6:32505702
6	rs554566424	32496605	C	0.4829	8.51E-08	chr:6:32505702
6	rs75518178	32505100	T	0.3675	8.57E-08	chr:6:32505702
6	rs112112734	32453853	C	0.2931	8.73E-08	chr:6:32505702
6	rs116504658	32537906	A	0.4868	8.87E-08	chr:6:32505702
6	rs76415507	32492589	T	0.5054	8.87E-08	chr:6:32505702
6	rs549643018	32525126	G	0.4854	8.99E-08	chr:6:32505702
6	rs62405632	32514561	G	0.3244	9.17E-08	chr:6:32505702
6	rs9268839	32428772	G	0.2945	9.25E-08	chr:6:32505702
6	rs9270591	32561424	C	0.2864	9.35E-08	chr:6:32505702
6	rs71534530	32511211	G	0.4305	9.35E-08	chr:6:32505702
6	rs137953098	32534097	T	0.5071	9.36E-08	chr:6:32505702
6	rs646159	32576446	C	0.4347	9.46E-08	chr:6:32505702
6	rs1064717	32546615	G	0.322	9.63E-08	chr:6:32505702
6	rs680061	32571098	A	0.4197	9.64E-08	chr:6:32505702
6	rs9270585	32561300	C	0.2869	9.65E-08	chr:6:32505702
6	rs9270588	32561327	C	0.2869	9.65E-08	chr:6:32505702
6	rs78533241	32502317	A	0.3908	9.67E-08	chr:6:32505702
6	rs75252378	32502610	T	0.402	9.79E-08	chr:6:32505702
6	rs9270659	32566076	C	0.4322	9.87E-08	chr:6:32505702
6	rs9270660	32566077	A	0.4322	9.87E-08	chr:6:32505702
6	rs148817433	32493266	T	0.4242	9.92E-08	chr:6:32505702
6	rs73726821	32526767	G	0.4126	1.01E-07	chr:6:32505702
6	rs142518779	32524817	G	0.4913	1.02E-07	chr:6:32505702
6	rs9378266	32448189	T	0.3241	1.05E-07	chr:6:32505702
6	rs66499101	32517332	A	0.3184	1.05E-07	chr:6:32505702
6	rs137976440	32489630	T	2.408	1.05E-07	chr:6:32505702
6	rs34665982	32560306	T	0.4957	1.06E-07	chr:6:32505702
6	rs9270368	32558182	C	0.3335	1.06E-07	chr:6:32505702
6	rs57172910	32529365	C	0.537	1.06E-07	chr:6:32505702
6	rs9270661	32566087	A	0.4326	1.07E-07	chr:6:32505702
6	rs9270746	32568284	C	0.45	1.07E-07	chr:6:32505702
6	rs141572443	32525395	C	0.5106	1.07E-07	chr:6:32505702
6	rs9269934	32551721	T	0.4623	1.07E-07	chr:6:32505702
6	rs147613123	32518296	A	0.3547	1.07E-07	chr:6:32505702
6	rs481245	32576114	A	0.4239	1.09E-07	chr:6:32505702
6	rs72508417	32459813	G	0.4544	1.09E-07	chr:6:32505702
6	rs2858867	32575325	A	0.4303	1.10E-07	chr:6:32505702
6	rs541195	32567321	A	0.4311	1.11E-07	chr:6:32505702
6	rs9270636	32565648	C	2.173	1.11E-07	chr:6:32505702
6	rs9270637	32565650	G	2.173	1.11E-07	chr:6:32505702
6	rs9270638	32565657	C	2.173	1.11E-07	chr:6:32505702

6	rs115789421	32526805	A	0.4138	1.11E-07	chr:6:32505702
6	rs9270414	32558497	C	0.4714	1.11E-07	chr:6:32505702
6	rs74999184	32508306	C	0.382	1.12E-07	chr:6:32505702
6	rs67405590	32525542	T	0.4763	1.12E-07	chr:6:32505702
6	rs540520708	32534266	G	0.4818	1.12E-07	chr:6:32505702
6	rs573056492	32534265	T	0.4818	1.12E-07	chr:6:32505702
6	rs74290590	32510539	C	0.3373	1.12E-07	chr:6:32505702
6	rs112740757	32481965	A	0.479	1.13E-07	chr:6:32505702
6	rs9269301	32539561	C	0.4818	1.13E-07	chr:6:32505702
6	rs115009756	32526800	A	0.4139	1.13E-07	chr:6:32505702
6	rs9270647	32565805	A	0.4341	1.13E-07	chr:6:32505702
6	rs9270649	32565808	G	0.4341	1.13E-07	chr:6:32505702
6	rs9269792	32549128	C	0.2393	1.14E-07	chr:6:32505702
6	rs566861	32576538	T	0.4285	1.14E-07	chr:6:32505702
6	rs113068532	32518301	G	0.3547	1.15E-07	chr:6:32505702
6	rs9270667	32566306	T	0.4246	1.15E-07	chr:6:32505702
6	rs114500718	32524694	G	0.4412	1.15E-07	chr:6:32505702
6	rs9270575	32561049	A	0.2588	1.16E-07	chr:6:32505702
6	rs28404276	32510355	G	0.413	1.17E-07	chr:6:32505702
6	rs2858866	32575391	G	0.4243	1.18E-07	chr:6:32505702
6	rs9271142	32577090	T	0.3033	1.18E-07	chr:6:32505702
6	rs112483249	32526924	A	0.4025	1.19E-07	chr:6:32505702
6	rs143442815	32489863	C	2.138	1.19E-07	chr:6:32505702
6	rs9270618	32565236	C	0.4261	1.19E-07	chr:6:32505702
6	rs9270413	32558495	C	0.4718	1.20E-07	chr:6:32505702
6	rs542233442	32535702	A	2.013	1.20E-07	chr:6:32505702
6	rs143237673	32525386	A	0.5124	1.21E-07	chr:6:32505702
6	rs72845733	32527305	A	0.4217	1.22E-07	chr:6:32505702
6	rs530218062	32525091	A	0.4924	1.22E-07	chr:6:32505702
6	rs114146939	32459890	C	0.5239	1.23E-07	chr:6:32505702
6	rs116587947	32459889	C	0.5239	1.23E-07	chr:6:32505702
6	rs73726822	32527272	G	0.4396	1.24E-07	chr:6:32505702
6	rs554894601	32497146	C	0.4036	1.24E-07	chr:6:32505702
6	rs553260480	32538534	A	0.2614	1.24E-07	chr:6:32505702
6	rs11752277	32537771	G	1.84	1.24E-07	chr:6:32505702
6	rs9270190	32556064	G	0.2573	1.25E-07	chr:6:32505702
6	rs74894552	32502589	C	0.4027	1.26E-07	chr:6:32505702
6	rs71549282	32504906	C	0.4612	1.26E-07	chr:6:32505702
6	rs28838605	32510279	C	0.3781	1.27E-07	chr:6:32505702
6	rs17209873	32446010	C	0.3421	1.27E-07	chr:6:32505702
6	rs71549283	32505038	A	0.3687	1.28E-07	chr:6:32505702
6	rs146356328	32535965	C	0.5059	1.31E-07	chr:6:32505702
6	rs9270612	32565106	T	0.4323	1.32E-07	chr:6:32505702

6	rs9270614	32565115	C	0.4323	1.32E-07	chr:6:32505702
6	rs615719	32574204	G	0.4344	1.35E-07	chr:6:32505702
6	rs28357078	32556991	G	0.2875	1.36E-07	chr:6:32505702
6	rs9270248	32556993	A	0.2875	1.36E-07	chr:6:32505702
6	rs9270249	32556996	C	0.2875	1.36E-07	chr:6:32505702
6	rs576371337	32493199	C	0.3633	1.36E-07	chr:6:32505702
6	rs549069478	32526275	A	0.43	1.38E-07	chr:6:32505702
6	rs34596578	32536450	G	0.4807	1.39E-07	chr:6:32505702
6	rs615698	32574190	A	0.4291	1.40E-07	chr:6:32505702
6	rs687675	32567316	T	0.4329	1.40E-07	chr:6:32505702
6	rs9270771	32568964	G	0.4407	1.44E-07	chr:6:32505702
6	rs486912	32575493	C	0.4257	1.45E-07	chr:6:32505702
6	rs9269388	32541102	T	2.06	1.47E-07	chr:6:32505702
6	rs9270663	32566101	A	0.4349	1.48E-07	chr:6:32505702
6	rs2858875	32566649	T	0.4288	1.49E-07	chr:6:32505702
6	rs114590821	32526035	A	0.5033	1.50E-07	chr:6:32505702
6	rs566252979	32496600	G	0.493	1.51E-07	chr:6:32505702
6	rs614437	32573941	A	0.4276	1.52E-07	chr:6:32505702
6	rs74945612	32505901	C	0.5206	1.53E-07	chr:6:32505702
6	rs12665158	32482007	C	0.516	1.53E-07	chr:6:32505702
6	rs367997563	32535716	C	1.999	1.55E-07	chr:6:32505702
6	rs560908329	32535706	G	1.999	1.55E-07	chr:6:32505702
6	rs680151	32571166	A	0.4589	1.55E-07	chr:6:32505702
6	rs115572650	32524778	T	0.4916	1.58E-07	chr:6:32505702
6	rs615672	32574171	G	0.4282	1.58E-07	chr:6:32505702
6	rs185231254	32535257	A	0.499	1.58E-07	chr:6:32505702
6	rs9271527	32589867	T	2.238	1.58E-07	chr:6:32505702
6	rs545827965	32525807	A	0.51	1.60E-07	chr:6:32505702
6	rs62405562	32451834	G	0.3173	1.60E-07	chr:6:32505702
6	rs115428373	32526116	G	0.5057	1.63E-07	chr:6:32505702
6	rs9270976	32573728	A	0.4363	1.65E-07	chr:6:32505702
6	rs114133813	32526300	T	0.4041	1.66E-07	chr:6:32505702
6	rs536237965	32535621	C	0.4809	1.68E-07	chr:6:32505702
6	rs9269732	32547425	A	0.4875	1.68E-07	chr:6:32505702
6	rs114710684	32521237	G	7.281	1.69E-07	chr:6:32505702
6	rs72853912	32506766	T	0.3961	1.69E-07	chr:6:32505702
6	rs116100204	32531556	C	0.4104	1.69E-07	chr:6:32505702
6	rs72847623	32538044	A	0.4695	1.70E-07	chr:6:32505702
6	rs9271522	32589791	C	2.236	1.72E-07	chr:6:32505702
6	rs74223623	32492102	C	0.4692	1.75E-07	chr:6:32505702
6	rs79645566	32505836	T	0.4907	1.76E-07	chr:6:32505702
6	rs9269736	32547440	C	0.4878	1.80E-07	chr:6:32505702
6	rs9269394	32541275	C	0.4591	1.80E-07	chr:6:32505702

6	rs114624001	32525083	A	0.4991	1.80E-07	chr:6:32505702
6	rs113045771	32503092	A	0.4482	1.81E-07	chr:6:32505702
6	rs140036576	32478632	T	2.038	1.83E-07	chr:6:32505702
6	rs534227738	32525995	A	0.5153	1.84E-07	chr:6:32505702
6	rs557442991	32534238	C	0.4975	1.86E-07	chr:6:32505702
6	rs182965163	32454827	G	0.4844	1.88E-07	chr:6:32505702
6	rs202162787	32454828	C	0.4844	1.88E-07	chr:6:32505702
6	rs114835436	32524789	A	0.5088	1.89E-07	chr:6:32505702
6	rs369257183	32525068	G	0.4993	1.90E-07	chr:6:32505702
6	rs116525270	32522921	T	0.3937	1.90E-07	chr:6:32505702
6	rs111293660	32503087	C	0.4488	1.90E-07	chr:6:32505702
6	rs9269728	32547385	A	0.4697	1.91E-07	chr:6:32505702
6	rs9270451	32558838	G	0.4778	1.93E-07	chr:6:32505702
6	rs73731412	32454215	G	8.06	1.94E-07	chr:6:32505702
6	rs201624597	32527297	G	0.4326	1.95E-07	chr:6:32505702
6	rs545503784	32537034	A	1.916	1.95E-07	chr:6:32505702
6	rs560891265	32537038	C	1.916	1.95E-07	chr:6:32505702
6	rs578162447	32537032	G	1.916	1.95E-07	chr:6:32505702
6	rs17203612	32446853	T	0.3299	1.96E-07	chr:6:32505702
6	rs9270143	32555256	C	0.4252	1.99E-07	chr:6:32505702
6	rs71536560	32529163	A	0.5224	2.00E-07	chr:6:32505702
6	rs9269906	32551309	G	0.4911	2.00E-07	chr:6:32505702
6	rs192020001	32525799	T	0.5053	2.00E-07	chr:6:32505702
6	rs9269986	32552553	C	0.4012	2.02E-07	chr:6:32505702
6	rs9271379	32587333	T	2.243	2.04E-07	chr:6:32505702
6	rs145092566	32454838	G	0.494	2.07E-07	chr:6:32505702
6	rs9270560	32560741	T	0.3044	2.08E-07	chr:6:32505702
6	rs9269234	32451788	C	2.272	2.10E-07	chr:6:32505702
6	rs112993783	32478652	A	2.032	2.12E-07	chr:6:32505702
6	rs9270609	32565051	A	0.4425	2.13E-07	chr:6:32505702
6	rs78316776	32508012	T	0.4727	2.13E-07	chr:6:32505702
6	rs9270448	32558819	A	0.4836	2.14E-07	chr:6:32505702
6	rs75876220	32454297	C	0.2569	2.16E-07	chr:6:32505702
6	rs116229360	32526811	A	0.4255	2.16E-07	chr:6:32505702
6	rs544126617	32510976	A	0.4806	2.17E-07	chr:6:32505702
6	rs577174379	32510979	G	0.4806	2.17E-07	chr:6:32505702
6	rs9270613	32565109	T	0.2199	2.17E-07	chr:6:32505702
6	rs184149877	32525731	A	0.4912	2.17E-07	chr:6:32505702
6	rs116181884	32487509	T	0.4528	2.19E-07	chr:6:32505702
6	rs142795234	32487593	T	0.467	2.20E-07	chr:6:32505702
6	rs9270382	32558285	G	0.4228	2.21E-07	chr:6:32505702
6	rs529924829	32486993	G	2.649	2.22E-07	chr:6:32505702
6	rs141114903	32487814	C	0.4687	2.24E-07	chr:6:32505702

6	rs186648552	32525996	T	0.52	2.24E-07	chr:6:32505702
6	rs9270616	32565154	C	0.4383	2.25E-07	chr:6:32505702
6	rs560277143	32547480	T	0.514	2.27E-07	chr:6:32505702
6	rs9269502	32543121	G	2.579	2.27E-07	chr:6:32505702
6	rs540304678	32536503	C	1.893	2.28E-07	chr:6:32505702
6	rs72849269	32468939	A	0.3966	2.30E-07	chr:6:32505702
6	rs141022812	32533567	G	0.4616	2.33E-07	chr:6:32505702
6	rs9270026	32553262	A	0.2826	2.33E-07	chr:6:32505702
6	rs571832998	32535560	G	0.4533	2.37E-07	chr:6:32505702
6	rs141684647	32507289	C	0.5029	2.41E-07	chr:6:32505702
6	rs201843924	32486878	T	0.3864	2.48E-07	chr:6:32505702
6	rs9269720	32547319	T	0.4675	2.52E-07	chr:6:32505702
6	rs545728465	32547479	C	0.516	2.52E-07	chr:6:32505702
6	rs199507577	32524985	C	0.4155	2.52E-07	chr:6:32505702
6	rs201400520	32524983	C	0.4155	2.52E-07	chr:6:32505702
6	rs9269564	32544471	C	0.354	2.53E-07	chr:6:32505702
6	rs574264514	32547472	A	0.5153	2.53E-07	chr:6:32505702
6	rs569972115	32535638	G	0.4875	2.57E-07	chr:6:32505702
6	rs9270606	32564869	C	0.4389	2.62E-07	chr:6:32505702
6	rs374949924	32534231	G	0.5054	2.66E-07	chr:6:32505702
6	rs9269581	32544785	A	0.4176	2.67E-07	chr:6:32505702
6	rs80296745	32478683	T	2.025	2.69E-07	chr:6:32505702
6	rs78776915	32469380	T	0.3518	2.71E-07	chr:6:32505702
6	rs372568767	32535570	C	0.454	2.72E-07	chr:6:32505702
6	rs112652539	32503128	G	0.3919	2.73E-07	chr:6:32505702
6	rs9270582	32561161	T	0.2713	2.74E-07	chr:6:32505702
6	rs77932549	32524736	G	0.474	2.74E-07	chr:6:32505702
6	rs78025613	32524735	A	0.474	2.74E-07	chr:6:32505702
6	rs9270326	32557812	C	0.2905	2.74E-07	chr:6:32505702
6	rs140542936	32525780	C	0.4985	2.75E-07	chr:6:32505702
6	rs62405649	32534491	G	0.4004	2.76E-07	chr:6:32505702
6	rs146225755	32491807	G	0.5055	2.77E-07	chr:6:32505702
6	rs9270388	32558343	C	0.4944	2.79E-07	chr:6:32505702
6	rs116715716	32506910	C	0.5314	2.79E-07	chr:6:32505702
6	rs9271489	32589051	A	2.215	2.81E-07	chr:6:32505702
6	rs148728069	32496097	C	0.5269	2.81E-07	chr:6:32505702
6	rs181916895	32525947	G	1.82	2.82E-07	chr:6:32505702
6	rs542755566	32525942	G	1.82	2.82E-07	chr:6:32505702
6	rs116783886	32521236	T	7.02	2.82E-07	chr:6:32505702
6	rs59066278	32510239	T	0.4113	2.83E-07	chr:6:32505702
6	rs9270484	32559063	A	0.2748	2.85E-07	chr:6:32505702
6	rs72845730	32526731	A	0.3793	2.85E-07	chr:6:32505702
6	rs139447342	32526123	C	0.5256	2.86E-07	chr:6:32505702

6	rs115834108	32459908	G	0.5419	2.88E-07	chr:6:32505702
6	rs9269588	32544887	G	0.3954	2.89E-07	chr:6:32505702
6	rs9269589	32544890	C	0.3954	2.89E-07	chr:6:32505702
6	rs533418679	32526140	A	1.819	2.90E-07	chr:6:32505702
6	rs147962567;rs35980928	32557378	C	3.36	2.96E-07	chr:6:32505702
6	rs701831	32549401	T	0.2388	3.12E-07	chr:6:32505702
6	rs112114145	32535188	A	0.4985	3.14E-07	chr:6:32505702
6	rs139296575	32469233	A	0.3801	3.15E-07	chr:6:32505702
6	rs547245894	32469227	C	0.3801	3.15E-07	chr:6:32505702
6	rs146797212	32507206	G	0.5022	3.19E-07	chr:6:32505702
6	rs9271533	32589952	T	2.139	3.22E-07	chr:6:32505702
6	rs9269295	32539475	C	1.891	3.22E-07	chr:6:32505702
6	rs9269715	32547278	T	0.4962	3.22E-07	chr:6:32505702
6	rs9269723	32547336	T	0.4764	3.22E-07	chr:6:32505702
6	rs114278037	32507173	G	0.5025	3.24E-07	chr:6:32505702
6	rs115824930	32507172	T	0.5025	3.24E-07	chr:6:32505702
6	rs181471921	32507163	A	0.5025	3.24E-07	chr:6:32505702
6	rs28437337	32451484	T	0.2917	3.25E-07	chr:6:32505702
6	rs548599006	32522315	C	0.2926	3.31E-07	chr:6:32505702
6	rs9269587	32544880	C	0.3957	3.34E-07	chr:6:32505702
6	rs73729123	32519811	T	0.3904	3.37E-07	chr:6:32505702
6	rs9269964	32552212	T	2.213	3.41E-07	chr:6:32505702
6	rs115254515	32491895	C	0.5027	3.47E-07	chr:6:32505702
6	rs141961165	32506799	C	0.4693	3.48E-07	chr:6:32505702
6	rs9271539	32590028	A	2.185	3.49E-07	chr:6:32505702
6	rs9271508	32589410	T	2.193	3.51E-07	chr:6:32505702
6	rs73727915	32497046	C	0.446	3.52E-07	chr:6:32505702
6	rs9269380	32540922	A	0.4708	3.60E-07	chr:6:32505702
6	rs74583807	32469214	T	0.3839	3.60E-07	chr:6:32505702
6	rs80293621	32469218	T	0.3839	3.60E-07	chr:6:32505702
6	rs9271558	32590348	G	2.17	3.65E-07	chr:6:32505702
6	rs2760981	32565465	G	0.3466	3.65E-07	chr:6:32505702
6	rs2395225	32590624	C	2.186	3.67E-07	chr:6:32505702
6	rs189560008	32526138	T	0.5205	3.69E-07	chr:6:32505702
6	rs79601580	32507591	G	0.4541	3.76E-07	chr:6:32505702
6	rs9270406	32558448	G	0.4899	3.77E-07	chr:6:32505702
6	rs115176251	32538129	G	0.3743	3.80E-07	chr:6:32505702
6	rs144578241	32536479	A	1.877	3.81E-07	chr:6:32505702
6	rs9270965	32573471	A	0.2972	3.85E-07	chr:6:32505702
6	rs28952268	32446724	T	0.2996	3.86E-07	chr:6:32505702
6	rs575449137	32536491	A	1.875	3.87E-07	chr:6:32505702
6	rs79749596	32534021	T	0.4485	3.88E-07	chr:6:32505702
6	rs9268846	32429245	G	0.3543	3.91E-07	chr:6:32505702

6	rs28559730	32526366	T	0.2943	3.93E-07	chr:6:32505702
6	rs28438482	32451243	G	0.2805	3.94E-07	chr:6:32505702
6	rs28627501	32451242	G	0.2805	3.94E-07	chr:6:32505702
6	rs9270123	32555032	G	0.3672	3.98E-07	chr:6:32505702
6	rs9269523	32543647	C	0.3272	3.99E-07	chr:6:32505702
6	rs9269905	32551291	T	0.4317	4.00E-07	chr:6:32505702
6	rs112675983	32469251	T	0.3851	4.01E-07	chr:6:32505702
6	rs114944990	32526885	C	0.445	4.13E-07	chr:6:32505702
6	rs9269525	32543676	C	0.3271	4.16E-07	chr:6:32505702
6	rs9271535	32589959	T	2.127	4.17E-07	chr:6:32505702
6	rs673002	32577814	G	0.1904	4.20E-07	chr:6:32505702
6	rs113496464	32494011	A	0.4495	4.23E-07	chr:6:32505702
6	rs75287767	32521735	T	0.4005	4.29E-07	chr:6:32505702
6	rs9270366	32558175	C	0.3423	4.34E-07	chr:6:32505702
6	rs9270745	32568264	G	0.4727	4.35E-07	chr:6:32505702
6	rs371700873	32514900	A	0.4894	4.36E-07	chr:6:32505702
6	rs79878723	32505682	C	0.497	4.39E-07	chr:6:32505702
6	rs371337007	32493146	G	0.4431	4.40E-07	chr:6:32505702
6	rs9269541	32544010	A	0.3191	4.52E-07	chr:6:32505702
6	rs62405643	32517807	C	2.021	4.56E-07	chr:6:32505702
6	rs616187	32574305	G	0.4265	4.57E-07	chr:6:32505702
6	rs72508432	32487524	G	0.464	4.60E-07	chr:6:32505702
6	rs180713844	32526141	A	0.5221	4.65E-07	chr:6:32505702
6	rs58769847	32481122	C	0.563	4.68E-07	chr:6:32505702
6	rs528638684	32490600	G	0.5173	4.69E-07	chr:6:32505702
6	rs9270362	32558135	T	0.3234	4.69E-07	chr:6:32505702
6	rs9271130	32576894	A	0.4307	4.71E-07	chr:6:32505702
6	rs9269567	32544572	A	0.3239	4.76E-07	chr:6:32505702
6	rs9269568	32544577	C	0.3239	4.76E-07	chr:6:32505702
6	rs35121819	32453318	C	0.3616	4.78E-07	chr:6:32505702
6	rs9269722	32547328	A	0.4859	4.82E-07	chr:6:32505702
6	rs3021303	32575746	G	0.4465	4.92E-07	chr:6:32505702
6	rs9269566	32544561	G	0.3237	4.96E-07	chr:6:32505702
6	rs527679389	32526147	T	0.5225	4.98E-07	chr:6:32505702
6	rs115814063	32492093	T	0.4919	5.05E-07	chr:6:32505702
6	rs192994337	32511083	A	0.4951	5.11E-07	chr:6:32505702
6	rs112550762	32507514	A	0.4719	5.21E-07	chr:6:32505702
6	rs9270511	32559371	T	0.3579	5.25E-07	chr:6:32505702
6	rs9269409	32541426	G	0.3238	5.25E-07	chr:6:32505702
6	rs9269485	32542728	T	0.3235	5.25E-07	chr:6:32505702
6	rs36101847	32550431	T	0.2565	5.27E-07	chr:6:32505702
6	rs5004278	32582401	G	0.4208	5.31E-07	chr:6:32505702
6	rs5004279	32582406	G	0.4208	5.31E-07	chr:6:32505702

6	rs528723549	32490420	A	0.5265	5.33E-07	chr:6:32505702
6	rs9270357	32558092	C	0.3228	5.35E-07	chr:6:32505702
6	rs9270358	32558096	A	0.3228	5.35E-07	chr:6:32505702
6	rs530004672	32526841	T	0.473	5.35E-07	chr:6:32505702
6	rs77951594	32499128	A	0.5236	5.37E-07	chr:6:32505702
6	rs9271526	32589862	A	2.132	5.43E-07	chr:6:32505702
6	rs9269693	32546866	T	0.3229	5.48E-07	chr:6:32505702
6	rs9269527	32543691	A	0.3258	5.49E-07	chr:6:32505702
6	rs9269649	32546067	C	0.323	5.49E-07	chr:6:32505702
6	rs9269669	32546418	C	0.339	5.51E-07	chr:6:32505702
6	rs115024757	32526836	C	0.4567	5.51E-07	chr:6:32505702
6	rs1064701	32546795	T	0.3229	5.53E-07	chr:6:32505702
6	rs9269916	32551468	A	0.3709	5.54E-07	chr:6:32505702
6	rs9269917	32551469	G	0.3709	5.54E-07	chr:6:32505702
6	rs112013291	32536551	G	0.5481	5.56E-07	chr:6:32505702
6	rs75236519	32460670	T	0.5614	5.59E-07	chr:6:32505702
6	rs201614260	32557436	G	3.039	5.67E-07	chr:6:32505702
6	rs9270512	32559380	G	0.358	5.69E-07	chr:6:32505702
6	rs140190321	32511008	T	0.4998	5.74E-07	chr:6:32505702
6	rs572123865	32497098	G	0.4334	5.78E-07	chr:6:32505702
6	rs9269536	32543923	C	0.3167	5.78E-07	chr:6:32505702
6	rs9269486	32542791	C	0.3227	5.83E-07	chr:6:32505702
6	rs9269480	32542568	T	0.3184	5.83E-07	chr:6:32505702
6	rs77233532	32510740	A	0.4847	5.88E-07	chr:6:32505702
6	rs9270242	32556964	C	0.3076	5.97E-07	chr:6:32505702
6	rs9270243	32556965	T	0.3076	5.97E-07	chr:6:32505702
6	rs9270244	32556967	T	0.3076	5.97E-07	chr:6:32505702
6	rs9269478	32542551	G	0.3182	5.99E-07	chr:6:32505702
6	rs137863146	32489871	G	2.031	6.00E-07	chr:6:32505702
6	rs191795965	32455073	T	0.3952	6.00E-07	chr:6:32505702
6	rs538742897	32526773	C	0.4271	6.00E-07	chr:6:32505702
6	rs72849254	32454200	T	0.2451	6.05E-07	chr:6:32505702
6	rs373964410	32497086	C	0.4335	6.05E-07	chr:6:32505702
6	rs567608492	32526161	A	0.5213	6.07E-07	chr:6:32505702
6	rs9269705	32547182	T	2.274	6.08E-07	chr:6:32505702
6	rs9270507	32559326	T	0.3843	6.08E-07	chr:6:32505702
6	rs9269396	32541290	G	0.4928	6.08E-07	chr:6:32505702
6	rs9270508	32559347	G	0.3844	6.09E-07	chr:6:32505702
6	rs9270509	32559355	A	0.3844	6.09E-07	chr:6:32505702
6	rs9270514	32559418	G	0.3411	6.11E-07	chr:6:32505702
6	rs9269611	32545248	C	0.3412	6.16E-07	chr:6:32505702
6	rs9269454	32542086	A	0.3214	6.18E-07	chr:6:32505702
6	rs76133616	32506895	A	0.5106	6.23E-07	chr:6:32505702

6	rs9269476	32542501	T	0.318	6.27E-07	chr:6:32505702
6	rs73407208	32491721	T	1.978	6.29E-07	chr:6:32505702
6	rs9271328	32582548	A	0.4219	6.34E-07	chr:6:32505702
6	rs189723252	32494948	T	1.809	6.47E-07	chr:6:32505702
6	rs140849564	32490607	A	0.4953	6.51E-07	chr:6:32505702
6	rs9269655	32546132	G	0.3223	6.51E-07	chr:6:32505702
6	rs9270639	32565658	T	2.034	6.53E-07	chr:6:32505702
6	rs111779823	32491965	C	0.5022	6.56E-07	chr:6:32505702
6	rs549407206	32526160	G	0.527	6.57E-07	chr:6:32505702
6	rs9270391	32558361	G	0.5155	6.57E-07	chr:6:32505702
6	rs9269544	32544129	G	0.3259	6.61E-07	chr:6:32505702
6	rs34288859	32545996	A	0.322	6.69E-07	chr:6:32505702
6	rs72851096	32505357	A	0.475	6.74E-07	chr:6:32505702
6	rs9270695	32567154	A	0.2977	6.76E-07	chr:6:32505702
6	rs9270696	32567155	G	0.2977	6.76E-07	chr:6:32505702
6	rs199973717	32493600	A	0.4058	6.76E-07	chr:6:32505702
6	rs113321784	32493147	T	0.436	6.76E-07	chr:6:32505702
6	rs114895810	32473514	C	2.443	6.77E-07	chr:6:32505702
6	rs140070075	32498899	A	0.4499	6.79E-07	chr:6:32505702
6	rs116711613	32492041	C	0.5027	6.83E-07	chr:6:32505702
6	rs60378094	32493690	C	0.4527	6.87E-07	chr:6:32505702
6	rs9269545	32544145	T	0.3257	6.88E-07	chr:6:32505702
6	rs9269634	32545706	A	0.3216	6.90E-07	chr:6:32505702
6	rs74508474	32454156	C	0.2473	7.00E-07	chr:6:32505702
6	rs79973795	32505443	A	0.4966	7.01E-07	chr:6:32505702
6	rs9271340	32582932	T	0.4226	7.03E-07	chr:6:32505702
6	rs9269607	32545168	G	0.341	7.03E-07	chr:6:32505702
6	rs145606733	32491959	A	0.5026	7.05E-07	chr:6:32505702
6	rs113319840	32507441	T	0.4821	7.06E-07	chr:6:32505702
6	rs34544636	32503879	T	0.2752	7.13E-07	chr:6:32505702
6	rs9269348	32540474	C	0.4841	7.17E-07	chr:6:32505702
6	rs9269332	32540077	A	0.3522	7.18E-07	chr:6:32505702
6	rs75700544	32469300	T	0.4727	7.21E-07	chr:6:32505702
6	rs9269668	32546394	G	0.3417	7.24E-07	chr:6:32505702
6	rs114085331	32479430	A	2.006	7.33E-07	chr:6:32505702
6	rs35773267	32555944	T	0.2624	7.34E-07	chr:6:32505702
6	rs557756256	32526179	T	0.5282	7.41E-07	chr:6:32505702
6	rs1064707	32546743	C	0.3212	7.44E-07	chr:6:32505702
6	rs9269923	32551604	T	2.022	7.51E-07	chr:6:32505702
6	rs9269785	32549053	T	2.454	7.52E-07	chr:6:32505702
6	rs9269787	32549070	G	2.454	7.52E-07	chr:6:32505702
6	rs28895241	32443665	C	0.3735	7.54E-07	chr:6:32505702
6	rs9269553	32544287	C	0.3197	7.58E-07	chr:6:32505702

6	rs112062732	32453936	C	0.3059	7.65E-07	chr:6:32505702
6	rs186995982	32494270	C	0.5352	7.76E-07	chr:6:32505702
6	rs75953322	32506798	T	0.4304	7.80E-07	chr:6:32505702
6	rs9269881	32550918	C	0.3743	7.81E-07	chr:6:32505702
6	rs9269529	32543733	T	0.3226	7.92E-07	chr:6:32505702
6	rs9269530	32543734	T	0.3226	7.92E-07	chr:6:32505702
6	rs531950386	32486540	T	1.818	8.00E-07	chr:6:32505702
6	rs9269489	32542832	T	0.3199	8.17E-07	chr:6:32505702
6	rs145316959	32521201	T	5.098	8.18E-07	chr:6:32505702
6	rs9270487	32559076	G	2.012	8.18E-07	chr:6:32505702
6	rs75969927	32502416	T	0.4794	8.25E-07	chr:6:32505702
6	rs111478896	32507910	T	1.812	8.36E-07	chr:6:32505702
6	rs9269897	32551112	G	0.4352	8.45E-07	chr:6:32505702
6	rs9269876	32550860	T	0.3258	8.55E-07	chr:6:32505702
6	rs9269701	32547053	A	0.32	8.57E-07	chr:6:32505702
6	rs9271153	32577549	A	0.298	8.58E-07	chr:6:32505702
6	rs9271156	32577590	G	0.298	8.58E-07	chr:6:32505702
6	rs9269622	32545523	G	0.3204	8.61E-07	chr:6:32505702
6	rs9269471	32542322	A	0.3374	8.69E-07	chr:6:32505702
6	rs9270047	32553697	T	0.4574	8.71E-07	chr:6:32505702
6	rs35747774	32487050	C	2.578	8.74E-07	chr:6:32505702
6	rs9271225	32579420	C	0.3188	8.79E-07	chr:6:32505702
6	rs9271003	32574527	T	0.3423	8.80E-07	chr:6:32505702
6	rs9269492	32542924	C	0.3179	8.87E-07	chr:6:32505702
6	rs9271066	32575631	A	0.2985	8.92E-07	chr:6:32505702
6	rs9269891	32551017	C	0.5114	8.92E-07	chr:6:32505702
6	rs9269491	32542844	A	0.3189	8.94E-07	chr:6:32505702
6	rs75499602	32506794	C	0.4312	8.94E-07	chr:6:32505702
6	rs9270209	32556443	A	0.3582	8.98E-07	chr:6:32505702
6	rs113749865	32481973	T	0.5047	8.99E-07	chr:6:32505702
6	rs2760982	32565495	G	0.4452	9.18E-07	chr:6:32505702
6	rs9271063	32575584	A	0.2982	9.21E-07	chr:6:32505702
6	rs9271064	32575601	A	0.2982	9.21E-07	chr:6:32505702
6	rs9270446	32558775	C	0.5286	9.24E-07	chr:6:32505702
6	rs9269576	32544724	A	0.3994	9.29E-07	chr:6:32505702
6	rs9270563	32560764	T	0.3172	9.33E-07	chr:6:32505702
6	rs9269427	32541581	T	0.3402	9.41E-07	chr:6:32505702
6	rs9269431	32541655	C	0.3402	9.41E-07	chr:6:32505702
6	rs71549285	32505371	T	0.4769	9.63E-07	chr:6:32505702
6	rs9270516	32559468	T	0.3184	9.63E-07	chr:6:32505702
6	rs9270517	32559472	T	0.3184	9.63E-07	chr:6:32505702
6	rs539676807	32526174	A	0.53	9.66E-07	chr:6:32505702
6	rs569049066	32526173	A	0.53	9.66E-07	chr:6:32505702

6	rs9269505	32543169	T	0.3424	9.69E-07	chr:6:32505702
6	rs9269558	32544351	A	0.3203	9.86E-07	chr:6:32505702
6	rs113698180	32518798	C	0.4039	9.91E-07	chr:6:32505702
6	rs9271106	32576595	G	0.3175	9.91E-07	chr:6:32505702
6	rs9271107	32576604	A	0.3175	9.91E-07	chr:6:32505702
6	rs9270513	32559400	T	0.3575	9.98E-07	chr:6:32505702
6	rs9269734	32547433	T	0.5242	1.00E-06	chr:6:32505702
6	rs186020633	32535771	A	1.977	1.01E-06	chr:6:32505702
6	rs67407114	32526500	A	0.3927	1.01E-06	chr:6:32505702
6	rs9269470	32542305	G	0.3405	1.02E-06	chr:6:32505702
6	rs111685796	32504453	G	0.3798	1.02E-06	chr:6:32505702
6	rs143945008	32513183	T	2.063	1.02E-06	chr:6:32505702
6	rs192052326	32528176	T	1.678	1.03E-06	chr:6:32505702
6	rs59447698	32481125	G	1.72	1.03E-06	chr:6:32505702
6	rs9270271	32557209	T	0.3175	1.03E-06	chr:6:32505702
6	rs573138176	32526180	A	0.5305	1.03E-06	chr:6:32505702
6	rs112399032	32510947	T	0.4987	1.04E-06	chr:6:32505702
6	rs9271233	32579533	C	0.3177	1.06E-06	chr:6:32505702
6	rs41287329	32552728	G	1.91	1.08E-06	chr:6:32505702
6	rs41287330	32552730	A	1.91	1.08E-06	chr:6:32505702
6	rs9271108	32576633	G	0.3172	1.08E-06	chr:6:32505702
6	rs186911188	32510905	G	0.5054	1.09E-06	chr:6:32505702
6	rs9270054	32553871	A	0.3174	1.09E-06	chr:6:32505702
6	rs9270948	32572972	C	0.3377	1.09E-06	chr:6:32505702
6	rs9270949	32572975	T	0.3377	1.09E-06	chr:6:32505702
6	rs9269656	32546203	A	0.3377	1.10E-06	chr:6:32505702
6	rs9269657	32546205	A	0.3377	1.10E-06	chr:6:32505702
6	rs9269575	32544714	G	0.3996	1.10E-06	chr:6:32505702
6	rs9270624	32565413	G	0.3165	1.11E-06	chr:6:32505702
6	rs72849268	32468931	A	0.3723	1.11E-06	chr:6:32505702
6	rs9271234	32579549	G	0.3169	1.11E-06	chr:6:32505702
6	rs71536581	32533030	C	0.2992	1.13E-06	chr:6:32505702
6	rs9271210	32579070	C	0.3139	1.13E-06	chr:6:32505702
6	rs9271211	32579078	C	0.3139	1.13E-06	chr:6:32505702
6	rs9271212	32579079	A	0.3139	1.13E-06	chr:6:32505702
6	rs9271417	32587818	C	2.087	1.13E-06	chr:6:32505702
6	rs9271018	32574807	T	0.3701	1.14E-06	chr:6:32505702
6	rs9271019	32574808	G	0.3701	1.14E-06	chr:6:32505702
6	rs7756647	32582843	G	2.178	1.14E-06	chr:6:32505702
6	rs9270213	32556494	A	0.3995	1.14E-06	chr:6:32505702
6	rs536803934	32526907	T	2.145	1.14E-06	chr:6:32505702
6	rs9271105	32576577	T	0.3157	1.15E-06	chr:6:32505702
6	rs9269439	32541898	C	0.3384	1.15E-06	chr:6:32505702

6	rs9270648	32565807	A	0.3157	1.15E-06	chr:6:32505702
6	rs72508415	32459793	A	1.812	1.16E-06	chr:6:32505702
6	rs9271453	32588416	A	0.3871	1.17E-06	chr:6:32505702
6	rs563226460	32490851	G	0.5561	1.17E-06	chr:6:32505702
6	rs9269309	32539625	T	0.4472	1.17E-06	chr:6:32505702
6	rs9270335	32557913	A	0.3693	1.17E-06	chr:6:32505702
6	rs9271550	32590274	A	2.085	1.18E-06	chr:6:32505702
6	rs9271154	32577563	G	0.3178	1.18E-06	chr:6:32505702
6	rs72508434	32487563	C	0.4785	1.18E-06	chr:6:32505702
6	rs9270062	32553995	G	0.3729	1.19E-06	chr:6:32505702
6	rs9270232	32556811	C	0.3136	1.20E-06	chr:6:32505702
6	rs142206489	32536167	A	2.027	1.20E-06	chr:6:32505702
6	rs9269829	32549915	T	0.3357	1.21E-06	chr:6:32505702
6	rs9269830	32549936	G	0.3357	1.21E-06	chr:6:32505702
6	rs79996892	32507051	C	0.5261	1.21E-06	chr:6:32505702
6	rs9270646	32565779	A	0.3157	1.21E-06	chr:6:32505702
6	rs9270010	32552930	T	0.3334	1.21E-06	chr:6:32505702
6	rs75202782	32538419	A	0.3157	1.22E-06	chr:6:32505702
6	rs200864308	32518611	G	0.4102	1.23E-06	chr:6:32505702
6	rs71536570	32531422	C	0.3015	1.23E-06	chr:6:32505702
6	rs9271111	32576699	G	0.3702	1.24E-06	chr:6:32505702
6	rs28383200	32574540	A	0.3074	1.25E-06	chr:6:32505702
6	rs71534527	32510978	A	0.4857	1.25E-06	chr:6:32505702
6	rs76564265	32530582	G	0.3062	1.25E-06	chr:6:32505702
6	rs9269246	32454098	C	2.523	1.25E-06	chr:6:32505702
6	rs9269499	32543091	C	0.3389	1.26E-06	chr:6:32505702
6	rs6933740	32537804	T	1.762	1.26E-06	chr:6:32505702
6	rs9270841	32570418	T	0.3161	1.26E-06	chr:6:32505702
6	rs9270940	32572850	A	0.3154	1.27E-06	chr:6:32505702
6	rs9270941	32572851	G	0.3154	1.27E-06	chr:6:32505702
6	rs35855915	32532691	G	0.3297	1.27E-06	chr:6:32505702
6	rs9270077	32554178	T	0.3164	1.28E-06	chr:6:32505702
6	rs9271562	32590411	A	2.08	1.30E-06	chr:6:32505702
6	rs9270831	32570221	G	0.3153	1.31E-06	chr:6:32505702
6	rs9270832	32570228	T	0.3153	1.31E-06	chr:6:32505702
6	rs201854374	32507311	C	0.5292	1.31E-06	chr:6:32505702
6	rs9270211	32556487	T	0.4025	1.31E-06	chr:6:32505702
6	rs9271209	32579035	G	0.3153	1.32E-06	chr:6:32505702
6	rs9271412	32587685	T	2.087	1.32E-06	chr:6:32505702
6	rs9270944	32572911	A	0.3367	1.33E-06	chr:6:32505702
6	rs9270208	32556402	G	0.3674	1.33E-06	chr:6:32505702
6	rs569962367	32485726	T	0.5385	1.34E-06	chr:6:32505702
6	rs9271051	32575290	T	0.3148	1.35E-06	chr:6:32505702

6	rs9271020	32574840	T	0.3365	1.35E-06	chr:6:32505702
6	rs539509361	32594399	G	1.967	1.35E-06	chr:6:32505702
6	rs9271219	32579354	C	0.3571	1.35E-06	chr:6:32505702
6	rs9271220	32579355	A	0.3571	1.35E-06	chr:6:32505702
6	rs9270712	32567380	T	0.3359	1.36E-06	chr:6:32505702
6	rs9270713	32567381	G	0.3359	1.36E-06	chr:6:32505702
6	rs9269407	32541376	T	0.3572	1.36E-06	chr:6:32505702
6	rs9270162	32555560	A	2.693	1.37E-06	chr:6:32505702
6	rs149361627	32486988	G	0.416	1.38E-06	chr:6:32505702
6	rs187402303	32486990	A	0.416	1.38E-06	chr:6:32505702
6	rs542087123	32486994	A	0.416	1.38E-06	chr:6:32505702
6	rs180731180	32535770	C	1.964	1.38E-06	chr:6:32505702
6	rs9271049	32575265	T	0.3145	1.38E-06	chr:6:32505702
6	rs9271050	32575275	G	0.3145	1.38E-06	chr:6:32505702
6	rs9271218	32579327	G	0.357	1.39E-06	chr:6:32505702
6	rs9270236	32556887	C	0.3153	1.40E-06	chr:6:32505702
6	rs9270237	32556908	C	0.3153	1.40E-06	chr:6:32505702
6	rs9270158	32555460	G	0.3159	1.42E-06	chr:6:32505702
6	rs9270323	32557782	G	0.3154	1.42E-06	chr:6:32505702
6	rs67927015	32531442	T	0.3043	1.42E-06	chr:6:32505702
6	rs145437633	32487776	G	0.4804	1.43E-06	chr:6:32505702
6	rs9270233	32556829	G	0.3154	1.43E-06	chr:6:32505702
6	rs77117066	32535516	C	0.4857	1.43E-06	chr:6:32505702
6	rs9270854	32570809	A	0.3153	1.43E-06	chr:6:32505702
6	rs9270979	32573780	A	0.3152	1.43E-06	chr:6:32505702
6	rs9270833	32570238	T	0.3144	1.43E-06	chr:6:32505702
6	rs9270735	32568096	T	0.3094	1.43E-06	chr:6:32505702
6	rs9270736	32568097	G	0.3094	1.43E-06	chr:6:32505702
6	rs9270842	32570461	C	0.3139	1.43E-06	chr:6:32505702
6	rs9270843	32570478	A	0.3139	1.43E-06	chr:6:32505702
6	rs9271144	32577220	G	0.3146	1.44E-06	chr:6:32505702
6	rs9271145	32577224	C	0.3146	1.44E-06	chr:6:32505702
6	rs9271424	32587922	C	2.073	1.44E-06	chr:6:32505702
6	rs9271425	32587923	G	2.073	1.44E-06	chr:6:32505702
6	rs9270608	32565042	A	0.3162	1.44E-06	chr:6:32505702
6	rs9271439	32588234	G	2.048	1.44E-06	chr:6:32505702
6	rs9270225	32556683	G	0.3119	1.44E-06	chr:6:32505702
6	rs9270859	32570894	T	0.3145	1.46E-06	chr:6:32505702
6	rs9271227	32579480	A	0.3143	1.47E-06	chr:6:32505702
6	rs9271228	32579481	A	0.3143	1.47E-06	chr:6:32505702
6	rs559863405	32536118	G	1.968	1.47E-06	chr:6:32505702
6	rs9270498	32559134	A	0.3195	1.47E-06	chr:6:32505702
6	rs9270622	32565329	C	0.3142	1.47E-06	chr:6:32505702

6	rs9270852	32570797	G	0.315	1.47E-06	chr:6:32505702
6	rs9270853	32570800	G	0.315	1.47E-06	chr:6:32505702
6	rs71536584	32533116	C	0.412	1.49E-06	chr:6:32505702
6	rs565682004	32526900	G	2.129	1.49E-06	chr:6:32505702
6	rs66491634	32528457	G	0.5073	1.49E-06	chr:6:32505702
6	rs9270816	32569879	T	0.3094	1.49E-06	chr:6:32505702
6	rs9270817	32569911	A	0.3094	1.49E-06	chr:6:32505702
6	rs9270780	32569108	C	0.3878	1.49E-06	chr:6:32505702
6	rs9270962	32573434	C	0.318	1.49E-06	chr:6:32505702
6	rs9270963	32573441	A	0.318	1.49E-06	chr:6:32505702
6	rs9270964	32573442	T	0.318	1.49E-06	chr:6:32505702
6	rs9270753	32568429	C	0.3142	1.50E-06	chr:6:32505702
6	rs9270125	32555049	A	0.3102	1.51E-06	chr:6:32505702
6	rs9270610	32565053	C	0.318	1.51E-06	chr:6:32505702
6	rs9271437	32588166	A	2.071	1.51E-06	chr:6:32505702
6	rs189503580	32488022	G	2.491	1.51E-06	chr:6:32505702
6	rs577424330	32488029	T	2.491	1.51E-06	chr:6:32505702
6	rs9271086	32576165	T	0.3179	1.51E-06	chr:6:32505702
6	rs9271087	32576170	C	0.3179	1.51E-06	chr:6:32505702
6	rs9271088	32576175	A	0.3179	1.51E-06	chr:6:32505702
6	rs9271089	32576177	T	0.3179	1.51E-06	chr:6:32505702
6	rs376533971	32540407	T	1.782	1.51E-06	chr:6:32505702
6	rs9271001	32574493	A	0.3395	1.51E-06	chr:6:32505702
6	rs9271002	32574497	T	0.3395	1.51E-06	chr:6:32505702
6	rs9270830	32570181	G	0.3142	1.52E-06	chr:6:32505702
6	rs9270857	32570849	G	0.3142	1.52E-06	chr:6:32505702
6	rs9270858	32570859	C	0.3142	1.52E-06	chr:6:32505702
6	rs75219938	32454278	T	0.262	1.53E-06	chr:6:32505702
6	rs9271140	32577083	C	0.3111	1.53E-06	chr:6:32505702
6	rs9271141	32577084	T	0.3111	1.53E-06	chr:6:32505702
6	rs9270727	32567954	T	0.3145	1.53E-06	chr:6:32505702
6	rs9270729	32567960	G	0.3145	1.53E-06	chr:6:32505702
6	rs66859861	32539012	T	0.3169	1.53E-06	chr:6:32505702
6	rs7768875	32560752	T	2.007	1.54E-06	chr:6:32505702
6	rs9271249	32580229	G	0.318	1.54E-06	chr:6:32505702
6	rs9270931	32572547	A	0.3141	1.54E-06	chr:6:32505702
6	rs79128700	32454275	C	0.2615	1.54E-06	chr:6:32505702
6	rs9272012	32598156	A	1.84	1.55E-06	chr:6:32505702
6	rs9270851	32570776	C	0.3143	1.55E-06	chr:6:32505702
6	rs9269857	32550407	G	0.315	1.55E-06	chr:6:32505702
6	rs9269858	32550426	G	0.315	1.55E-06	chr:6:32505702
6	rs9270909	32572179	A	0.3185	1.56E-06	chr:6:32505702
6	rs73726198	32492262	T	0.4833	1.57E-06	chr:6:32505702

6	rs9270675	32566647	A	0.2982	1.57E-06	chr:6:32505702
6	rs73399473	32538959	C	0.3169	1.57E-06	chr:6:32505702
6	rs9269458	32542148	A	0.3572	1.58E-06	chr:6:32505702
6	rs9269461	32542161	C	0.3572	1.58E-06	chr:6:32505702
6	rs9269867	32550683	G	0.3374	1.58E-06	chr:6:32505702
6	rs9270322	32557776	C	0.3137	1.58E-06	chr:6:32505702
6	rs9270971	32573598	T	0.3175	1.59E-06	chr:6:32505702
6	rs9269282	32539339	A	0.4542	1.59E-06	chr:6:32505702
6	rs9270615	32565152	T	0.3138	1.59E-06	chr:6:32505702
6	rs9269661	32546259	C	0.4128	1.59E-06	chr:6:32505702
6	rs9270595	32561579	C	0.3179	1.60E-06	chr:6:32505702
6	rs9270168	32555641	A	0.3138	1.60E-06	chr:6:32505702
6	rs9270723	32567798	G	0.3134	1.60E-06	chr:6:32505702
6	rs9270724	32567820	A	0.3134	1.60E-06	chr:6:32505702
6	rs199514452	32557489	T	2.891	1.61E-06	chr:6:32505702
6	rs201540428	32557486	A	2.891	1.61E-06	chr:6:32505702
6	rs7758269	32598812	C	1.973	1.61E-06	chr:6:32505702
6	rs530242049	32536125	C	1.966	1.61E-06	chr:6:32505702
6	rs9270939	32572806	G	0.3136	1.61E-06	chr:6:32505702
6	rs9270180	32555805	A	0.314	1.61E-06	chr:6:32505702
6	rs9270754	32568471	G	0.3135	1.62E-06	chr:6:32505702
6	rs9270278	32557253	C	0.3141	1.63E-06	chr:6:32505702
6	rs9270281	32557267	A	0.3141	1.63E-06	chr:6:32505702
6	rs9271422	32587860	G	0.3173	1.63E-06	chr:6:32505702
6	rs9271083	32576102	T	0.3135	1.63E-06	chr:6:32505702
6	rs9271213	32579211	G	0.3135	1.63E-06	chr:6:32505702
6	rs9269391	32541128	G	0.468	1.64E-06	chr:6:32505702
6	rs9270670	32566533	A	0.3131	1.64E-06	chr:6:32505702
6	rs9270671	32566549	T	0.3131	1.64E-06	chr:6:32505702
6	rs9270672	32566557	G	0.3131	1.64E-06	chr:6:32505702
6	rs112221534	32453603	A	0.2914	1.64E-06	chr:6:32505702
6	rs9270937	32572757	A	0.3133	1.65E-06	chr:6:32505702
6	rs9271115	32576731	C	0.4001	1.65E-06	chr:6:32505702
6	rs9270856	32570839	A	0.3137	1.65E-06	chr:6:32505702
6	rs62405575	32458259	A	0.502	1.65E-06	chr:6:32505702
6	rs9270933	32572659	C	0.3132	1.65E-06	chr:6:32505702
6	rs9270934	32572695	G	0.3132	1.65E-06	chr:6:32505702
6	rs9270935	32572701	A	0.3132	1.65E-06	chr:6:32505702
6	rs4341046	32567880	C	0.3136	1.65E-06	chr:6:32505702
6	rs72508409	32451073	G	17.24	1.66E-06	chr:6:32505702
6	rs9271058	32575406	A	0.3131	1.66E-06	chr:6:32505702
6	rs9270778	32569050	A	0.3131	1.67E-06	chr:6:32505702
6	rs9270581	32561143	C	0.3137	1.67E-06	chr:6:32505702

6	rs9270813	32569790	C	0.3131	1.67E-06	chr:6:32505702
6	rs9271205	32578941	G	0.313	1.67E-06	chr:6:32505702
6	rs9270908	32572148	G	0.3178	1.68E-06	chr:6:32505702
6	rs9270786	32569164	C	0.3132	1.68E-06	chr:6:32505702
6	rs9270165	32555627	A	0.3143	1.68E-06	chr:6:32505702
6	rs9270166	32555633	G	0.3143	1.68E-06	chr:6:32505702
6	rs9270405	32558444	G	0.5389	1.68E-06	chr:6:32505702
6	rs9270875	32571310	A	0.358	1.68E-06	chr:6:32505702
6	rs9271079	32576052	G	0.3132	1.69E-06	chr:6:32505702
6	rs9271255	32580357	C	0.3121	1.69E-06	chr:6:32505702
6	rs9271068	32575674	C	0.313	1.69E-06	chr:6:32505702
6	rs9269815	32549726	A	0.3133	1.70E-06	chr:6:32505702
6	rs9269816	32549727	T	0.3133	1.70E-06	chr:6:32505702
6	rs9269817	32549746	A	0.3133	1.70E-06	chr:6:32505702
6	rs9269819	32549782	C	0.3133	1.70E-06	chr:6:32505702
6	rs9270781	32569111	G	1.922	1.70E-06	chr:6:32505702
6	rs9270579	32561098	A	0.313	1.70E-06	chr:6:32505702
6	rs9270924	32572408	G	0.3364	1.70E-06	chr:6:32505702
6	rs116360287	32482303	G	0.5193	1.71E-06	chr:6:32505702
6	rs9269854	32550331	T	0.3129	1.71E-06	chr:6:32505702
6	rs73726189	32492008	T	3.789	1.71E-06	chr:6:32505702
6	rs9271221	32579363	G	0.3566	1.72E-06	chr:6:32505702
6	rs9270811	32569721	A	0.3128	1.72E-06	chr:6:32505702
6	rs9271149	32577455	A	0.3134	1.72E-06	chr:6:32505702
6	rs9271150	32577470	T	0.3134	1.72E-06	chr:6:32505702
6	rs9271151	32577474	T	0.3134	1.72E-06	chr:6:32505702
6	rs9271239	32579769	T	0.3128	1.72E-06	chr:6:32505702
6	rs9271097	32576439	C	0.3134	1.73E-06	chr:6:32505702
6	rs9271098	32576440	T	0.3134	1.73E-06	chr:6:32505702
6	rs9270810	32569659	T	0.3128	1.73E-06	chr:6:32505702
6	rs9270791	32569232	C	0.3128	1.73E-06	chr:6:32505702
6	rs9270792	32569233	A	0.3128	1.73E-06	chr:6:32505702
6	rs9271135	32577002	A	0.3128	1.74E-06	chr:6:32505702
6	rs9270981	32573932	A	0.3134	1.74E-06	chr:6:32505702
6	rs9269774	32548802	G	0.3128	1.74E-06	chr:6:32505702
6	rs9270071	32554097	G	0.4716	1.74E-06	chr:6:32505702
6	rs9270719	32567586	A	0.3128	1.74E-06	chr:6:32505702
6	rs9270720	32567627	T	0.3128	1.74E-06	chr:6:32505702
6	rs9270569	32560899	C	0.3134	1.74E-06	chr:6:32505702
6	rs9270668	32566308	A	0.3128	1.75E-06	chr:6:32505702
6	rs9270914	32572249	A	0.3394	1.75E-06	chr:6:32505702
6	rs9270953	32573045	C	0.3129	1.75E-06	chr:6:32505702
6	rs9271139	32577038	T	0.3128	1.75E-06	chr:6:32505702

6	rs9270998	32574379	A	0.3129	1.76E-06	chr:6:32505702
6	rs9271021	32574863	G	0.3395	1.76E-06	chr:6:32505702
6	rs9271100	32576478	T	0.313	1.76E-06	chr:6:32505702
6	rs9271016	32574767	C	0.4003	1.77E-06	chr:6:32505702
6	rs9270124	32555040	C	0.3135	1.77E-06	chr:6:32505702
6	rs542465996	32485661	A	0.5358	1.77E-06	chr:6:32505702
6	rs563816645	32485662	C	0.5358	1.77E-06	chr:6:32505702
6	rs575576508	32485670	G	0.5358	1.77E-06	chr:6:32505702
6	rs113014982	32536382	T	0.5326	1.77E-06	chr:6:32505702
6	rs9271223	32579372	T	0.313	1.78E-06	chr:6:32505702
6	rs9270972	32573621	C	0.3166	1.78E-06	chr:6:32505702
6	rs9270552	32560520	G	0.3178	1.79E-06	chr:6:32505702
6	rs9270553	32560523	A	0.3178	1.79E-06	chr:6:32505702
6	rs9270983	32573973	T	0.3131	1.79E-06	chr:6:32505702
6	rs9271538	32589983	A	2.033	1.80E-06	chr:6:32505702
6	rs145260193	32455012	G	0.3349	1.80E-06	chr:6:32505702
6	rs3997874	32580741	A	0.3125	1.80E-06	chr:6:32505702
6	rs9270258	32557060	G	0.3125	1.80E-06	chr:6:32505702
6	rs9270584	32561277	C	0.3125	1.80E-06	chr:6:32505702
6	rs9270650	32565854	C	0.3125	1.80E-06	chr:6:32505702
6	rs9270678	32566686	T	0.3125	1.80E-06	chr:6:32505702
6	rs9270692	32567006	C	0.3125	1.80E-06	chr:6:32505702
6	rs9270693	32567007	A	0.3125	1.80E-06	chr:6:32505702
6	rs9270694	32567024	C	0.3125	1.80E-06	chr:6:32505702
6	rs9270768	32568805	T	0.3125	1.80E-06	chr:6:32505702
6	rs9270985	32574027	T	0.3125	1.80E-06	chr:6:32505702
6	rs9270987	32574127	T	0.3125	1.80E-06	chr:6:32505702
6	rs9270988	32574130	A	0.3125	1.80E-06	chr:6:32505702
6	rs9271029	32575077	A	0.3125	1.80E-06	chr:6:32505702
6	rs9271060	32575513	C	0.3125	1.80E-06	chr:6:32505702
6	rs9271073	32575803	G	0.3125	1.80E-06	chr:6:32505702
6	rs9271075	32575852	A	0.3125	1.80E-06	chr:6:32505702
6	rs9271076	32575873	G	0.3125	1.80E-06	chr:6:32505702
6	rs9271146	32577282	A	0.3125	1.80E-06	chr:6:32505702
6	rs9271240	32579833	T	0.3125	1.80E-06	chr:6:32505702
6	rs9271260	32580507	A	0.3125	1.80E-06	chr:6:32505702
6	rs9270922	32572388	T	0.3363	1.81E-06	chr:6:32505702
6	rs79119980	32505272	C	0.4714	1.81E-06	chr:6:32505702
6	rs9270805	32569556	T	0.3159	1.81E-06	chr:6:32505702
6	rs9270594	32561526	C	0.3159	1.83E-06	chr:6:32505702
6	rs9271112	32576717	T	0.3965	1.83E-06	chr:6:32505702
6	rs9271022	32574881	A	0.3393	1.83E-06	chr:6:32505702
6	rs9270554	32560546	C	0.3174	1.83E-06	chr:6:32505702

6	rs77115076	32454361	T	0.31	1.84E-06	chr:6:32505702
6	rs78358922	32454360	A	0.31	1.84E-06	chr:6:32505702
6	rs9270912	32572224	A	0.3391	1.84E-06	chr:6:32505702
6	rs9270913	32572229	A	0.3391	1.84E-06	chr:6:32505702
6	rs574714830	32494119	G	0.4963	1.85E-06	chr:6:32505702
6	rs372319444	32526876	A	0.4621	1.86E-06	chr:6:32505702
6	rs9271405	32587584	C	2.056	1.87E-06	chr:6:32505702
6	rs9270925	32572433	A	0.3361	1.88E-06	chr:6:32505702
6	rs9270926	32572435	C	0.3361	1.88E-06	chr:6:32505702
6	rs71536582	32533078	G	0.3825	1.89E-06	chr:6:32505702
6	rs9270808	32569607	G	0.3156	1.90E-06	chr:6:32505702
6	rs9270809	32569608	G	0.3156	1.90E-06	chr:6:32505702
6	rs74203767	32491593	G	2.4	1.90E-06	chr:6:32505702
6	rs9270551	32560501	G	0.3172	1.91E-06	chr:6:32505702
6	rs72492362	32543969	C	0.458	1.91E-06	chr:6:32505702
6	rs111842802	32538450	G	0.3116	1.91E-06	chr:6:32505702
6	rs112232311	32538454	A	0.3116	1.91E-06	chr:6:32505702
6	rs146618240	32482102	A	0.5662	1.93E-06	chr:6:32505702
6	rs191018606	32488081	T	0.4027	1.93E-06	chr:6:32505702
6	rs9271251	32580241	C	0.316	1.94E-06	chr:6:32505702
6	rs9271392	32587456	G	2.024	1.94E-06	chr:6:32505702
6	rs9271410	32587645	G	2.053	1.95E-06	chr:6:32505702
6	rs9270230	32556787	C	0.316	1.95E-06	chr:6:32505702
6	rs9271094	32576347	G	0.3159	1.96E-06	chr:6:32505702
6	rs9271250	32580234	G	0.3158	1.97E-06	chr:6:32505702
6	rs9270951	32573039	C	0.3359	1.97E-06	chr:6:32505702
6	rs9270950	32573027	T	0.3358	1.97E-06	chr:6:32505702
6	rs9270945	32572948	A	0.3545	1.98E-06	chr:6:32505702
6	rs9270946	32572952	G	0.3545	1.98E-06	chr:6:32505702
6	rs9270489	32559077	C	0.3157	1.99E-06	chr:6:32505702
6	rs9270081	32554243	T	0.3902	1.99E-06	chr:6:32505702
6	rs9270385	32558314	T	0.439	2.00E-06	chr:6:32505702
6	rs9270885	32571771	G	0.4128	2.00E-06	chr:6:32505702
6	rs9271252	32580288	C	0.3157	2.00E-06	chr:6:32505702
6	rs9269373	32540873	G	1.794	2.00E-06	chr:6:32505702
6	rs113395139	32538431	C	0.3235	2.01E-06	chr:6:32505702
6	rs116218325	32526611	G	0.4343	2.01E-06	chr:6:32505702
6	rs201277028	32526612	T	0.4343	2.01E-06	chr:6:32505702
6	rs77858388	32526619	A	0.4343	2.01E-06	chr:6:32505702
6	rs74973088	32457874	C	0.4773	2.01E-06	chr:6:32505702
6	rs9269888	32550980	C	0.4493	2.01E-06	chr:6:32505702
6	rs9270947	32572961	G	0.3546	2.01E-06	chr:6:32505702
6	rs9270078	32554179	A	0.4018	2.02E-06	chr:6:32505702

6	rs34812354	32530046	T	0.3475	2.03E-06	chr:6:32505702
6	rs9271091	32576275	G	0.3156	2.03E-06	chr:6:32505702
6	rs9271092	32576296	A	0.3156	2.03E-06	chr:6:32505702
6	rs9271162	32577715	G	0.3164	2.04E-06	chr:6:32505702
6	rs9271163	32577733	C	0.3164	2.04E-06	chr:6:32505702
6	rs9271174	32578040	C	0.3164	2.06E-06	chr:6:32505702
6	rs114375471	32493854	A	0.4791	2.07E-06	chr:6:32505702
6	rs116684175	32493853	C	0.4791	2.07E-06	chr:6:32505702
6	rs36152293	32526410	T	1.894	2.08E-06	chr:6:32505702
6	rs9271242	32579940	T	0.3151	2.08E-06	chr:6:32505702
6	rs9271243	32579951	C	0.3151	2.08E-06	chr:6:32505702
6	rs9271246	32580084	A	0.3151	2.08E-06	chr:6:32505702
6	rs9271247	32580112	A	0.3151	2.08E-06	chr:6:32505702
6	rs9271248	32580157	C	0.3151	2.08E-06	chr:6:32505702
6	rs9270952	32573040	A	0.3357	2.08E-06	chr:6:32505702
6	rs9270999	32574438	G	0.3157	2.09E-06	chr:6:32505702
6	rs9271023	32574895	T	0.3386	2.10E-06	chr:6:32505702
6	rs66814015	32505258	A	0.4722	2.10E-06	chr:6:32505702
6	rs35464393	32530198	T	0.3267	2.11E-06	chr:6:32505702
6	rs9270102	32554621	G	0.3151	2.11E-06	chr:6:32505702
6	rs9270605	32564861	A	0.3152	2.11E-06	chr:6:32505702
6	rs9271171	32577907	C	0.3162	2.11E-06	chr:6:32505702
6	rs9271172	32577975	C	0.3162	2.11E-06	chr:6:32505702
6	rs9271173	32577981	A	0.3162	2.11E-06	chr:6:32505702
6	rs9271170	32577889	T	0.3161	2.13E-06	chr:6:32505702
6	rs9271090	32576247	T	0.3153	2.13E-06	chr:6:32505702
6	rs9271192	32578530	C	0.3154	2.14E-06	chr:6:32505702
6	rs71534526	32510974	T	0.5367	2.15E-06	chr:6:32505702
6	rs28724008	32550550	G	0.2871	2.15E-06	chr:6:32505702
6	rs9269890	32551010	G	0.4703	2.16E-06	chr:6:32505702
6	rs71536585	32533131	G	0.4257	2.17E-06	chr:6:32505702
6	rs9270625	32565438	C	0.3552	2.17E-06	chr:6:32505702
6	rs9270627	32565447	T	0.3552	2.17E-06	chr:6:32505702
6	rs9270628	32565448	C	0.3552	2.17E-06	chr:6:32505702
6	rs9269884	32550969	A	0.4497	2.17E-06	chr:6:32505702
6	rs142972412	32454837	T	0.4548	2.18E-06	chr:6:32505702
6	rs9270586	32561302	G	0.3157	2.18E-06	chr:6:32505702
6	rs9270587	32561305	T	0.3157	2.18E-06	chr:6:32505702
6	rs9271164	32577784	C	0.3158	2.19E-06	chr:6:32505702
6	rs9271165	32577785	A	0.3158	2.19E-06	chr:6:32505702
6	rs9271166	32577811	C	0.3158	2.19E-06	chr:6:32505702
6	rs9271168	32577854	G	0.3158	2.19E-06	chr:6:32505702
6	rs12195582	32444544	T	0.4012	2.19E-06	chr:6:32505702

6	rs9270265	32557127	A	0.3151	2.19E-06	chr:6:32505702
6	rs9271201	32578818	C	0.316	2.20E-06	chr:6:32505702
6	rs9271202	32578837	A	0.316	2.20E-06	chr:6:32505702
6	rs9270557	32560645	A	0.3159	2.20E-06	chr:6:32505702
6	rs115830407	32482484	T	0.5551	2.20E-06	chr:6:32505702
6	rs9270118	32554983	C	0.3007	2.21E-06	chr:6:32505702
6	rs9270119	32554987	A	0.3007	2.21E-06	chr:6:32505702
6	rs9270120	32554990	C	0.3007	2.21E-06	chr:6:32505702
6	rs9271484	32588905	T	2.045	2.22E-06	chr:6:32505702
6	rs9270644	32565745	G	0.3345	2.25E-06	chr:6:32505702
6	rs9270887	32571789	C	0.413	2.25E-06	chr:6:32505702
6	rs71538524	32536592	G	0.5151	2.27E-06	chr:6:32505702
6	rs541082691	32536247	T	0.5301	2.27E-06	chr:6:32505702
6	rs9271053	32575319	C	0.332	2.28E-06	chr:6:32505702
6	rs9271077	32575925	G	0.3168	2.28E-06	chr:6:32505702
6	rs9271078	32575930	C	0.3168	2.28E-06	chr:6:32505702
6	rs9270954	32573058	C	0.3347	2.30E-06	chr:6:32505702
6	rs79788626	32490246	A	0.5439	2.32E-06	chr:6:32505702
6	rs9271407	32587605	T	2.037	2.32E-06	chr:6:32505702
6	rs9270086	32554283	G	0.4287	2.32E-06	chr:6:32505702
6	rs9271236	32579645	C	0.3195	2.34E-06	chr:6:32505702
6	rs145704749	32454744	C	0.2806	2.35E-06	chr:6:32505702
6	rs66988465	32523109	C	0.2468	2.35E-06	chr:6:32505702
6	rs113947393	32537950	C	0.5205	2.38E-06	chr:6:32505702
6	rs570595530	32525770	T	1.732	2.39E-06	chr:6:32505702
6	rs9270847	32570602	G	0.3292	2.39E-06	chr:6:32505702
6	rs9270848	32570609	C	0.3292	2.39E-06	chr:6:32505702
6	rs9272081	32599655	C	1.793	2.42E-06	chr:6:32505702
6	rs9269958	32552143	C	0.4206	2.42E-06	chr:6:32505702
6	rs9269959	32552144	A	0.4206	2.42E-06	chr:6:32505702
6	rs9270458	32558900	A	0.5274	2.43E-06	chr:6:32505702
6	rs78121285	32502174	A	0.3287	2.45E-06	chr:6:32505702
6	rs9270438	32558703	G	0.5322	2.47E-06	chr:6:32505702
6	rs9272285	32603688	G	2.012	2.49E-06	chr:6:32505702
6	rs9271185	32578286	G	0.3225	2.50E-06	chr:6:32505702
6	rs9271186	32578297	A	0.3225	2.50E-06	chr:6:32505702
6	rs111358198	32547489	T	0.438	2.50E-06	chr:6:32505702
6	rs9270927	32572451	A	0.334	2.51E-06	chr:6:32505702
6	rs71538525	32536630	A	0.5094	2.52E-06	chr:6:32505702
6	rs9270710	32567368	C	0.3531	2.53E-06	chr:6:32505702
6	rs9270231	32556788	A	0.3348	2.54E-06	chr:6:32505702
6	rs9271024	32574954	T	0.3378	2.55E-06	chr:6:32505702
6	rs9270687	32566871	G	0.3217	2.56E-06	chr:6:32505702

6	rs9270688	32566872	T	0.3217	2.56E-06	chr:6:32505702
6	rs9270839	32570375	A	0.3336	2.56E-06	chr:6:32505702
6	rs9270840	32570377	C	0.3336	2.56E-06	chr:6:32505702
6	rs9271178	32578191	C	0.3217	2.56E-06	chr:6:32505702
6	rs9271179	32578196	G	0.3217	2.56E-06	chr:6:32505702
6	rs9270690	32566948	G	0.3184	2.57E-06	chr:6:32505702
6	rs9270691	32566972	G	0.3184	2.57E-06	chr:6:32505702
6	rs9270069	32554080	T	0.4159	2.58E-06	chr:6:32505702
6	rs76731120	32454239	C	0.2117	2.58E-06	chr:6:32505702
6	rs3104416	32581502	C	0.3187	2.58E-06	chr:6:32505702
6	rs9269995	32552681	C	1.857	2.59E-06	chr:6:32505702
6	rs9270607	32564915	T	0.3387	2.60E-06	chr:6:32505702
6	rs9271117	32576795	C	0.4337	2.60E-06	chr:6:32505702
6	rs9271118	32576796	A	0.4337	2.60E-06	chr:6:32505702
6	rs9271193	32578566	T	0.3188	2.61E-06	chr:6:32505702
6	rs9271194	32578612	T	0.3188	2.61E-06	chr:6:32505702
6	rs9270097	32554506	C	0.3338	2.61E-06	chr:6:32505702
6	rs9270098	32554517	T	0.3338	2.61E-06	chr:6:32505702
6	rs569686853	32525767	G	1.73	2.62E-06	chr:6:32505702
6	rs111976080	32538814	T	0.3564	2.64E-06	chr:6:32505702
6	rs9270711	32567375	A	0.353	2.64E-06	chr:6:32505702
6	rs28366320	32561551	A	0.3408	2.65E-06	chr:6:32505702
6	rs9271104	32576561	G	0.3332	2.65E-06	chr:6:32505702
6	rs77102498	32572583	G	0.3341	2.65E-06	chr:6:32505702
6	rs9270845	32570571	T	0.3336	2.67E-06	chr:6:32505702
6	rs9271189	32578357	C	0.322	2.67E-06	chr:6:32505702
6	rs9271052	32575309	T	0.3339	2.68E-06	chr:6:32505702
6	rs73728637	32594420	C	2.023	2.69E-06	chr:6:32505702
6	rs9270564	32560773	G	0.3539	2.69E-06	chr:6:32505702
6	rs9270424	32558594	G	0.51	2.72E-06	chr:6:32505702
6	rs182336149	32494269	C	0.5255	2.73E-06	chr:6:32505702
6	rs9269385	32541036	C	0.3891	2.73E-06	chr:6:32505702
6	rs9270689	32566909	A	0.3212	2.73E-06	chr:6:32505702
6	rs9270788	32569205	G	0.3258	2.74E-06	chr:6:32505702
6	rs9270604	32564800	T	0.4799	2.74E-06	chr:6:32505702
6	rs3997871	32580456	G	0.3198	2.75E-06	chr:6:32505702
6	rs9271257	32580444	G	0.3198	2.75E-06	chr:6:32505702
6	rs9271258	32580446	G	0.3198	2.75E-06	chr:6:32505702
6	rs148940091	32507101	T	0.5694	2.75E-06	chr:6:32505702
6	rs146344255	32490158	C	0.5388	2.75E-06	chr:6:32505702
6	rs9270967	32573543	C	0.3368	2.75E-06	chr:6:32505702
6	rs9270968	32573554	C	0.3368	2.75E-06	chr:6:32505702
6	rs9270568	32560808	G	0.3335	2.79E-06	chr:6:32505702

6	rs9270978	32573754	T	0.3338	2.79E-06	chr:6:32505702
6	rs9269372	32540809	G	0.5485	2.79E-06	chr:6:32505702
6	rs1059546	32557552	A	0.1862	2.80E-06	chr:6:32505702
6	rs9270874	32571227	G	0.3335	2.80E-06	chr:6:32505702
6	rs72847626	32538224	G	0.4401	2.80E-06	chr:6:32505702
6	rs9271026	32574994	T	0.3373	2.80E-06	chr:6:32505702
6	rs147610934	32455033	C	0.3443	2.81E-06	chr:6:32505702
6	rs9270846	32570577	A	0.3335	2.81E-06	chr:6:32505702
6	rs9270844	32570550	T	0.3329	2.83E-06	chr:6:32505702
6	rs9270419	32558559	T	0.5171	2.84E-06	chr:6:32505702
6	rs9270396	32558385	T	0.5035	2.84E-06	chr:6:32505702
6	rs9270522	32559541	T	0.3543	2.84E-06	chr:6:32505702
6	rs9270955	32573167	G	0.3619	2.84E-06	chr:6:32505702
6	rs9270956	32573170	T	0.3619	2.84E-06	chr:6:32505702
6	rs17209280	32440269	G	2.611	2.85E-06	chr:6:32505702
6	rs9270991	32574200	G	0.3357	2.85E-06	chr:6:32505702
6	rs9270992	32574202	C	0.3357	2.85E-06	chr:6:32505702
6	rs9271143	32577165	T	0.3334	2.86E-06	chr:6:32505702
6	rs9270969	32573560	A	0.3366	2.87E-06	chr:6:32505702
6	rs114553448	32537139	A	0.5276	2.89E-06	chr:6:32505702
6	rs9272208	32601967	G	1.916	2.89E-06	chr:6:32505702
6	rs72508440	32489908	G	0.4942	2.90E-06	chr:6:32505702
6	rs9270738	32568118	T	0.3549	2.91E-06	chr:6:32505702
6	rs79267592	32578673	A	0.3218	2.91E-06	chr:6:32505702
6	rs9270732	32568018	C	0.3334	2.95E-06	chr:6:32505702
6	rs17204294	32492179	T	0.4715	2.96E-06	chr:6:32505702
6	rs9271229	32579515	A	0.3331	2.97E-06	chr:6:32505702
6	rs9271230	32579519	C	0.3331	2.97E-06	chr:6:32505702
6	rs9271231	32579520	T	0.3331	2.97E-06	chr:6:32505702
6	rs9270756	32568538	A	0.3324	2.97E-06	chr:6:32505702
6	rs9270827	32570132	C	0.333	2.97E-06	chr:6:32505702
6	rs9270828	32570144	T	0.333	2.97E-06	chr:6:32505702
6	rs72847627	32538512	A	0.3535	2.98E-06	chr:6:32505702
6	rs9269283	32539355	G	0.4671	2.99E-06	chr:6:32505702
6	rs9270834	32570266	C	0.3335	2.99E-06	chr:6:32505702
6	rs9270774	32569012	G	0.3816	2.99E-06	chr:6:32505702
6	rs9270303	32557483	T	0.3169	2.99E-06	chr:6:32505702
6	rs9270970	32573574	C	0.3364	2.99E-06	chr:6:32505702
6	rs572264676	32490134	T	0.5565	3.03E-06	chr:6:32505702
6	rs9270737	32568107	A	0.3548	3.04E-06	chr:6:32505702
6	rs9271103	32576540	T	0.3326	3.04E-06	chr:6:32505702
6	rs9271198	32578726	G	0.3215	3.05E-06	chr:6:32505702
6	rs9271199	32578735	A	0.3215	3.05E-06	chr:6:32505702

6	rs9271200	32578756	C	0.3215	3.05E-06	chr:6:32505702
6	rs9270466	32558929	C	0.5344	3.05E-06	chr:6:32505702
6	rs9270765	32568740	G	0.3532	3.06E-06	chr:6:32505702
6	rs9270766	32568763	G	0.3532	3.06E-06	chr:6:32505702
6	rs9270767	32568770	T	0.3532	3.06E-06	chr:6:32505702
6	rs189371992	32498895	A	0.4622	3.06E-06	chr:6:32505702
6	rs35652190	32544828	T	0.488	3.08E-06	chr:6:32505702
6	rs373652084	32562127	T	1.911	3.09E-06	chr:6:32505702
6	rs74503228	32454246	T	0.2149	3.09E-06	chr:6:32505702
6	rs9270426	32558632	T	0.5182	3.10E-06	chr:6:32505702
6	rs9270452	32558851	A	0.4665	3.13E-06	chr:6:32505702
6	rs9270453	32558852	C	0.4665	3.13E-06	chr:6:32505702
6	rs9270835	32570281	G	0.3333	3.13E-06	chr:6:32505702
6	rs9270838	32570353	C	0.3327	3.15E-06	chr:6:32505702
6	rs9270226	32556689	G	0.3298	3.15E-06	chr:6:32505702
6	rs9270450	32558837	T	0.4764	3.16E-06	chr:6:32505702
6	rs9270762	32568686	A	0.3424	3.18E-06	chr:6:32505702
6	rs9270206	32556367	G	0.3295	3.19E-06	chr:6:32505702
6	rs66869156	32531295	T	0.3449	3.19E-06	chr:6:32505702
6	rs9270401	32558414	T	0.5048	3.20E-06	chr:6:32505702
6	rs9270904	32572060	T	0.3927	3.25E-06	chr:6:32505702
6	rs9270974	32573713	C	0.3361	3.26E-06	chr:6:32505702
6	rs9271101	32576493	C	0.3324	3.27E-06	chr:6:32505702
6	rs9270755	32568522	G	0.3321	3.28E-06	chr:6:32505702
6	rs9270420	32558569	C	0.5181	3.29E-06	chr:6:32505702
6	rs9271712	32593432	G	0.5348	3.31E-06	chr:6:32505702
6	rs9270198	32556227	C	0.3965	3.31E-06	chr:6:32505702
6	rs553518894	32486504	C	0.521	3.31E-06	chr:6:32505702
6	rs34303755	32450613	C	0.3078	3.31E-06	chr:6:32505702
6	rs9270099	32554540	A	0.3323	3.32E-06	chr:6:32505702
6	rs72845704	32523228	T	0.2943	3.36E-06	chr:6:32505702
6	rs73726847	32537646	C	0.5434	3.36E-06	chr:6:32505702
6	rs67240795	32530458	C	0.3801	3.37E-06	chr:6:32505702
6	rs9270960	32573288	T	0.3304	3.37E-06	chr:6:32505702
6	rs9270961	32573289	G	0.3304	3.37E-06	chr:6:32505702
6	rs76675915	32555554	T	2.619	3.41E-06	chr:6:32505702
6	rs9270836	32570325	A	0.3323	3.41E-06	chr:6:32505702
6	rs9270392	32558372	A	0.5048	3.41E-06	chr:6:32505702
6	rs9269609	32545211	T	0.424	3.41E-06	chr:6:32505702
6	rs9269610	32545218	C	0.424	3.41E-06	chr:6:32505702
6	rs9270619	32565243	A	0.3322	3.43E-06	chr:6:32505702
6	rs541218925	32488717	A	0.5416	3.44E-06	chr:6:32505702
6	rs9269389	32541106	C	0.4885	3.45E-06	chr:6:32505702

6	rs9270577	32561074	A	0.3321	3.46E-06	chr:6:32505702
6	rs9270215	32556548	G	0.4415	3.48E-06	chr:6:32505702
6	rs9269913	32551406	A	0.4149	3.50E-06	chr:6:32505702
6	rs71534539	32512754	G	0.505	3.50E-06	chr:6:32505702
6	rs9270674	32566598	C	0.3319	3.51E-06	chr:6:32505702
6	rs9270417	32558546	T	0.5188	3.53E-06	chr:6:32505702
6	rs9270418	32558550	C	0.5188	3.53E-06	chr:6:32505702
6	rs9270421	32558574	T	0.5188	3.55E-06	chr:6:32505702
6	rs9270422	32558577	A	0.5188	3.55E-06	chr:6:32505702
6	rs9269907	32551324	A	0.5228	3.56E-06	chr:6:32505702
6	rs9271096	32576426	A	0.3542	3.61E-06	chr:6:32505702
6	rs138535752	32489520	G	1.921	3.64E-06	chr:6:32505702
6	rs9270066	32554025	A	0.4168	3.68E-06	chr:6:32505702
6	rs557048	32587001	T	2.023	3.69E-06	chr:6:32505702
6	rs200623105	32540757	G	0.4965	3.72E-06	chr:6:32505702
6	rs9270377	32558260	G	0.473	3.75E-06	chr:6:32505702
6	rs9270677	32566655	T	0.3317	3.75E-06	chr:6:32505702
6	rs9270340	32557969	C	0.3528	3.77E-06	chr:6:32505702
6	rs9270341	32557970	C	0.3528	3.77E-06	chr:6:32505702
6	rs9271011	32574676	A	0.3762	3.79E-06	chr:6:32505702
6	rs9270777	32569038	T	0.4117	3.79E-06	chr:6:32505702
6	rs9269914	32551416	G	0.4151	3.82E-06	chr:6:32505702
6	rs9270403	32558434	A	0.5097	3.85E-06	chr:6:32505702
6	rs9269727	32547371	T	0.5405	3.85E-06	chr:6:32505702
6	rs9270202	32556246	G	0.4119	3.85E-06	chr:6:32505702
6	rs9270425	32558627	G	0.5254	3.93E-06	chr:6:32505702
6	rs146758209	32496618	G	0.5109	3.93E-06	chr:6:32505702
6	rs9270734	32568085	T	0.3539	3.97E-06	chr:6:32505702
6	rs9270196	32556200	T	0.3943	4.00E-06	chr:6:32505702
6	rs9268960	32434130	C	0.3417	4.01E-06	chr:6:32505702
6	rs9268961	32434135	A	0.3417	4.01E-06	chr:6:32505702
6	rs9270864	32571001	T	0.3668	4.03E-06	chr:6:32505702
6	rs9270204	32556277	T	0.4036	4.11E-06	chr:6:32505702
6	rs9270714	32567488	A	0.3346	4.16E-06	chr:6:32505702
6	rs9270716	32567502	A	0.3346	4.16E-06	chr:6:32505702
6	rs113128948	32507129	C	0.5734	4.19E-06	chr:6:32505702
6	rs554654579	32644070	C	11.72	5.88E-38	chr:6:32648036
6	rs28755212	32644094	C	10.41	6.57E-38	chr:6:32648036
6	rs28755213	32644099	G	10.41	6.57E-38	chr:6:32648036
6	rs28755214	32644111	C	10.41	6.57E-38	chr:6:32648036
6	rs28755216	32644135	A	11.28	7.25E-38	chr:6:32648036
6	rs28755215	32644128	G	10.96	7.38E-38	chr:6:32648036
6	rs28755209	32644049	T	12.55	9.35E-38	chr:6:32648036

6	rs28755208	32644036	C	11.7	9.57E-38	chr:6:32648036
6	rs9275018	32643139	T	10.36	1.01E-37	chr:6:32648036
6	rs9275017	32643130	T	10.4	1.12E-37	chr:6:32648036
6	rs28755220	32644238	C	11.79	1.16E-37	chr:6:32648036
6	rs28755221	32644239	A	11.79	1.16E-37	chr:6:32648036
6	rs28755255	32644915	G	10.06	1.20E-37	chr:6:32648036
6	rs28755256	32644919	G	10.06	1.20E-37	chr:6:32648036
6	rs9275019	32643166	T	12.28	1.23E-37	chr:6:32648036
6	rs28755222	32644264	C	10.8	1.25E-37	chr:6:32648036
6	rs28755268	32645469	A	11.94	1.30E-37	chr:6:32648036
6	rs9275020	32643184	T	12.71	1.35E-37	chr:6:32648036
6	rs28755364	32647133	T	10.15	1.46E-37	chr:6:32648036
6	rs28755219	32644226	C	11.71	1.49E-37	chr:6:32648036
6	rs28755270	32645489	A	12.22	1.56E-37	chr:6:32648036
6	rs28755271	32645497	T	12.16	1.73E-37	chr:6:32648036
6	rs28755272	32645529	C	12.77	1.73E-37	chr:6:32648036
6	rs28755273	32645534	A	12.77	1.73E-37	chr:6:32648036
6	rs28371222	32647611	T	11.17	2.14E-37	chr:6:32648036
6	rs28371223	32647617	G	10.82	2.16E-37	chr:6:32648036
6	rs28371224	32647628	A	10.82	2.16E-37	chr:6:32648036
6	rs28371225	32647640	A	10.82	2.16E-37	chr:6:32648036
6	rs28755217	32644159	C	12.83	2.34E-37	chr:6:32648036
6	rs28755225	32644290	T	9.777	2.35E-37	chr:6:32648036
6	rs9275021	32643220	A	11.62	2.35E-37	chr:6:32648036
6	rs28755300	32646132	A	9.868	2.37E-37	chr:6:32648036
6	rs28755301	32646134	A	9.868	2.37E-37	chr:6:32648036
6	rs28755257	32644953	T	9.694	2.43E-37	chr:6:32648036
6	rs28755258	32644958	C	9.694	2.43E-37	chr:6:32648036
6	rs28755340	32646830	G	9.584	2.43E-37	chr:6:32648036
6	rs28755299	32646107	A	9.912	2.50E-37	chr:6:32648036
6	rs28755365	32647150	C	11.33	2.64E-37	chr:6:32648036
6	rs28755263	32645109	A	10.61	2.72E-37	chr:6:32648036
6	rs28755259	32644983	A	11.8	2.84E-37	chr:6:32648036
6	rs28755266	32645432	T	10.01	2.85E-37	chr:6:32648036
6	rs28371243	32647946	C	10.92	2.88E-37	chr:6:32648036
6	rs28755294	32646047	C	10.4	2.97E-37	chr:6:32648036
6	rs28755218	32644190	T	13.39	2.97E-37	chr:6:32648036
6	rs28755341	32646837	C	9.869	3.00E-37	chr:6:32648036
6	rs28755224	32644285	T	9.545	3.15E-37	chr:6:32648036
6	rs9275022	32643251	G	9.389	3.19E-37	chr:6:32648036
6	rs28755234	32644391	T	11.62	3.21E-37	chr:6:32648036
6	rs28755302	32646143	G	9.64	3.28E-37	chr:6:32648036
6	rs28755303	32646145	G	9.64	3.28E-37	chr:6:32648036

6	rs28755264	32645131	G	9.749	3.34E-37	chr:6:32648036
6	rs28755206	32644010	G	14.18	3.34E-37	chr:6:32648036
6	rs28755327	32646604	A	10.81	3.38E-37	chr:6:32648036
6	rs28371242	32647935	G	11.73	3.41E-37	chr:6:32648036
6	rs28755223	32644276	A	9.514	3.47E-37	chr:6:32648036
6	rs28755296	32646085	G	9.762	3.50E-37	chr:6:32648036
6	rs28755297	32646087	G	9.762	3.50E-37	chr:6:32648036
6	rs28755265	32645144	A	9.512	3.53E-37	chr:6:32648036
6	rs28755229	32644305	A	9.272	3.66E-37	chr:6:32648036
6	rs28371241	32647906	G	12.15	3.73E-37	chr:6:32648036
6	rs28755244	32644631	A	12.36	3.79E-37	chr:6:32648036
6	rs28755342	32646861	T	10.01	3.94E-37	chr:6:32648036
6	rs28755343	32646866	A	10.01	3.94E-37	chr:6:32648036
6	rs28755298	32646094	T	9.88	3.96E-37	chr:6:32648036
6	rs28755295	32646054	A	10.26	3.98E-37	chr:6:32648036
6	rs28755226	32644297	G	9.076	4.12E-37	chr:6:32648036
6	rs28755227	32644299	C	9.076	4.12E-37	chr:6:32648036
6	rs28755228	32644300	A	9.076	4.12E-37	chr:6:32648036
6	rs28755362	32647119	C	8.67	4.17E-37	chr:6:32648036
6	rs28755363	32647120	T	8.67	4.17E-37	chr:6:32648036
6	rs28755262	32645088	T	11.48	4.18E-37	chr:6:32648036
6	rs28755267	32645446	C	10.3	4.20E-37	chr:6:32648036
6	rs28755334	32646759	G	11.38	4.23E-37	chr:6:32648036
6	rs28755335	32646763	T	11.38	4.23E-37	chr:6:32648036
6	rs28755326	32646588	A	11.69	4.29E-37	chr:6:32648036
6	rs28755235	32644423	T	12.06	4.32E-37	chr:6:32648036
6	rs28755236	32644428	G	12.06	4.32E-37	chr:6:32648036
6	rs28755260	32645073	C	12.42	4.54E-37	chr:6:32648036
6	rs28755261	32645075	G	12.42	4.54E-37	chr:6:32648036
6	rs28371213	32646059	G	9.757	4.77E-37	chr:6:32648036
6	rs28371214	32646061	G	9.757	4.77E-37	chr:6:32648036
6	rs28755333	32646691	G	11.46	5.13E-37	chr:6:32648036
6	rs28755245	32644658	T	12.47	5.15E-37	chr:6:32648036
6	rs28755233	32644350	C	9.781	5.48E-37	chr:6:32648036
6	rs28755242	32644577	G	12.84	5.49E-37	chr:6:32648036
6	rs28755246	32644670	C	12.39	5.63E-37	chr:6:32648036
6	rs28755247	32644680	T	12.39	5.63E-37	chr:6:32648036
6	rs28755231	32644337	G	9.563	5.71E-37	chr:6:32648036
6	rs28755324	32646494	T	12.36	5.91E-37	chr:6:32648036
6	rs28755361	32647113	G	8.479	5.98E-37	chr:6:32648036
6	rs28755243	32644594	A	12.77	6.00E-37	chr:6:32648036
6	rs28755344	32646904	C	10.42	6.31E-37	chr:6:32648036
6	rs7775228	32658079	C	47.17	6.65E-37	chr:6:32648036

6	rs28371226	32647665	T	8.666	6.90E-37	chr:6:32648036
6	rs28371227	32647666	G	8.666	6.90E-37	chr:6:32648036
6	rs28755253	32644869	T	13.97	7.29E-37	chr:6:32648036
6	rs28755254	32644878	C	13.97	7.29E-37	chr:6:32648036
6	rs28755230	32644328	G	9.326	7.33E-37	chr:6:32648036
6	rs116493570	32643860	T	14.25	7.62E-37	chr:6:32648036
6	rs114557541	32643844	T	14.15	7.82E-37	chr:6:32648036
6	rs28755370	32647353	G	12.94	7.88E-37	chr:6:32648036
6	rs28755371	32647357	C	12.94	7.88E-37	chr:6:32648036
6	rs28371219	32647510	A	9.831	7.90E-37	chr:6:32648036
6	rs28755304	32646193	T	9.054	8.04E-37	chr:6:32648036
6	rs28755249	32644701	A	12.88	8.14E-37	chr:6:32648036
6	rs3129713	32657255	T	46.8	8.38E-37	chr:6:32648036
6	rs3129715	32657281	T	46.8	8.38E-37	chr:6:32648036
6	rs28755360	32647099	C	8.365	8.58E-37	chr:6:32648036
6	rs28755325	32646521	A	13.33	8.97E-37	chr:6:32648036
6	rs28371220	32647546	A	13.39	9.17E-37	chr:6:32648036
6	rs28371221	32647556	C	13.39	9.17E-37	chr:6:32648036
6	rs28755372	32647383	A	9.631	9.65E-37	chr:6:32648036
6	rs28371218	32647508	C	9.586	9.73E-37	chr:6:32648036
6	rs28755323	32646469	A	9.113	9.76E-37	chr:6:32648036
6	rs9275031	32643420	A	9.808	9.87E-37	chr:6:32648036
6	rs28755277	32645598	T	13.81	1.02E-36	chr:6:32648036
6	rs28755278	32645599	G	13.81	1.02E-36	chr:6:32648036
6	rs28755279	32645626	C	13.81	1.02E-36	chr:6:32648036
6	rs28755305	32646232	G	8.601	1.03E-36	chr:6:32648036
6	rs28755306	32646238	T	8.601	1.03E-36	chr:6:32648036
6	rs28755307	32646241	A	8.601	1.03E-36	chr:6:32648036
6	rs141967600	32643824	T	14.7	1.04E-36	chr:6:32648036
6	rs199945715	32645744	T	14.61	1.11E-36	chr:6:32648036
6	rs28755339	32646817	G	8.425	1.27E-36	chr:6:32648036
6	rs28755276	32645578	C	14.33	1.31E-36	chr:6:32648036
6	rs28755240	32644562	A	13.95	1.37E-36	chr:6:32648036
6	rs28755308	32646274	C	8.51	1.43E-36	chr:6:32648036
6	rs116005148	32645766	A	14.52	1.46E-36	chr:6:32648036
6	rs202178406	32645770	G	14.52	1.46E-36	chr:6:32648036
6	rs28755373	32647388	A	9.448	1.46E-36	chr:6:32648036
6	rs28755366	32647187	T	14.31	1.47E-36	chr:6:32648036
6	rs28755293	32646010	T	14.92	1.49E-36	chr:6:32648036
6	rs28755309	32646279	G	8.369	1.55E-36	chr:6:32648036
6	rs201805319	32645737	G	15.26	1.70E-36	chr:6:32648036
6	rs28755367	32647215	A	14.28	1.74E-36	chr:6:32648036
6	rs28371215	32647476	G	8.725	1.75E-36	chr:6:32648036

6	rs28755186	32643479	C	14.38	1.80E-36	chr:6:32648036
6	rs9275023	32643276	T	7.987	1.90E-36	chr:6:32648036
6	rs3129778	32646252	T	8.205	1.95E-36	chr:6:32648036
6	rs2858327	32658935	G	39.48	2.18E-36	chr:6:32648036
6	rs140232909	32643883	A	14.93	2.34E-36	chr:6:32648036
6	rs28755239	32644463	C	15	2.50E-36	chr:6:32648036
6	rs28755187	32643516	A	14.84	2.53E-36	chr:6:32648036
6	rs28755203	32643947	G	14.83	2.55E-36	chr:6:32648036
6	rs28755204	32643953	T	14.83	2.55E-36	chr:6:32648036
6	rs28371239	32647854	C	8.302	2.56E-36	chr:6:32648036
6	rs113838733	32645825	C	14.94	2.58E-36	chr:6:32648036
6	rs114954038	32645693	T	15.75	2.59E-36	chr:6:32648036
6	rs115932399	32645695	T	15.75	2.59E-36	chr:6:32648036
6	rs28755345	32646930	C	9.072	2.61E-36	chr:6:32648036
6	rs28755202	32643894	G	14.86	2.69E-36	chr:6:32648036
6	rs28371216	32647491	C	8.553	2.73E-36	chr:6:32648036
6	rs28371217	32647495	A	8.553	2.73E-36	chr:6:32648036
6	rs113898828	32643755	T	15.67	2.82E-36	chr:6:32648036
6	rs114642759	32643766	T	15.67	2.82E-36	chr:6:32648036
6	rs28755251	32644775	T	14.91	2.83E-36	chr:6:32648036
6	rs28755252	32644806	G	14.91	2.83E-36	chr:6:32648036
6	rs28755369	32647313	G	14.91	2.83E-36	chr:6:32648036
6	rs28755188	32643548	C	14.8	2.88E-36	chr:6:32648036
6	rs28755189	32643552	C	14.8	2.88E-36	chr:6:32648036
6	rs112838245	32645820	G	14.95	2.97E-36	chr:6:32648036
6	rs9275024	32643281	A	7.843	3.01E-36	chr:6:32648036
6	rs28755250	32644755	G	15.1	3.19E-36	chr:6:32648036
6	rs28371211	32637528	A	26.88	3.20E-36	chr:6:32648036
6	rs28755351	32647012	G	8.444	3.23E-36	chr:6:32648036
6	rs28755338	32646804	T	7.808	3.27E-36	chr:6:32648036
6	rs28755352	32647027	T	8.295	3.38E-36	chr:6:32648036
6	rs28755280	32645660	A	15.66	3.53E-36	chr:6:32648036
6	rs28755290	32645871	C	15.49	3.60E-36	chr:6:32648036
6	rs28755291	32645906	G	15.49	3.60E-36	chr:6:32648036
6	rs28755292	32645974	A	15.49	3.60E-36	chr:6:32648036
6	rs28755346	32646940	T	8.557	3.86E-36	chr:6:32648036
6	rs28755353	32647040	A	8.255	3.87E-36	chr:6:32648036
6	rs28755191	32643584	G	15.5	4.14E-36	chr:6:32648036
6	rs28755192	32643687	T	15.5	4.14E-36	chr:6:32648036
6	rs28755193	32643704	C	15.5	4.14E-36	chr:6:32648036
6	rs28755368	32647280	C	15.5	4.14E-36	chr:6:32648036
6	rs9275064	32648381	T	31.74	4.49E-36	chr:6:32648036
6	rs28755349	32646978	C	8.227	5.86E-36	chr:6:32648036

6	rs28371228	32647698	A	7.51	6.52E-36	chr:6:32648036
6	rs9275015	32643108	A	8.837	6.85E-36	chr:6:32648036
6	rs9275016	32643109	G	8.837	6.85E-36	chr:6:32648036
6	rs28371238	32647846	C	7.654	7.01E-36	chr:6:32648036
6	rs28755350	32646997	G	8.1	7.44E-36	chr:6:32648036
6	rs17212014	32637472	T	24.5	7.73E-36	chr:6:32648036
6	rs28755190	32643583	C	16.23	8.15E-36	chr:6:32648036
6	rs28755310	32646304	T	7.979	9.29E-36	chr:6:32648036
6	rs9275066	32648404	T	28.9	1.01E-35	chr:6:32648036
6	rs9275067	32648414	C	28.27	1.05E-35	chr:6:32648036
6	rs9275025	32643303	T	7.485	1.13E-35	chr:6:32648036
6	rs28755354	32647045	T	7.776	1.16E-35	chr:6:32648036
6	rs28755328	32646616	C	7.723	1.42E-35	chr:6:32648036
6	rs28755329	32646617	A	7.723	1.42E-35	chr:6:32648036
6	rs28755311	32646309	C	7.632	1.82E-35	chr:6:32648036
6	rs28755331	32646647	A	7.874	1.87E-35	chr:6:32648036
6	rs28755332	32646648	T	7.874	1.87E-35	chr:6:32648036
6	rs141881212	32639387	G	32.58	2.69E-35	chr:6:32648036
6	rs28755356	32647059	G	7.447	2.77E-35	chr:6:32648036
6	rs28755330	32646637	A	7.48	2.88E-35	chr:6:32648036
6	rs139025169	32639390	A	34.76	3.04E-35	chr:6:32648036
6	rs28755374	32647404	T	7.322	3.96E-35	chr:6:32648036
6	rs28755375	32647411	C	7.322	3.96E-35	chr:6:32648036
6	rs28755376	32647412	A	7.322	3.96E-35	chr:6:32648036
6	rs28755357	32647061	C	7.282	4.05E-35	chr:6:32648036
6	rs28755315	32646369	G	7.043	5.50E-35	chr:6:32648036
6	rs9275075	32648550	A	39.61	5.88E-35	chr:6:32648036
6	rs9275076	32648553	G	39.61	5.88E-35	chr:6:32648036
6	rs9274858	32640546	A	37.23	6.89E-35	chr:6:32648036
6	rs9274860	32640556	A	37.23	6.89E-35	chr:6:32648036
6	rs28755312	32646342	T	7.073	7.26E-35	chr:6:32648036
6	rs28755313	32646343	G	7.073	7.26E-35	chr:6:32648036
6	rs9274716	32637404	A	39.61	7.45E-35	chr:6:32648036
6	rs28755314	32646366	G	6.898	8.92E-35	chr:6:32648036
6	rs9274856	32640539	T	36.7	1.05E-34	chr:6:32648036
6	rs144949632	32639331	A	27.43	1.13E-34	chr:6:32648036
6	rs148651202	32639332	T	27.43	1.13E-34	chr:6:32648036
6	rs9274816	32639621	A	22.71	1.18E-34	chr:6:32648036
6	rs9274817	32639623	T	22.71	1.18E-34	chr:6:32648036
6	rs112819910	32639344	T	29.04	1.45E-34	chr:6:32648036
6	rs2856678	32655830	A	17.77	1.50E-34	chr:6:32648036
6	rs9275002	32642761	A	43.61	1.56E-34	chr:6:32648036
6	rs9275030	32643351	T	6.842	1.91E-34	chr:6:32648036

6	rs9274819	32639646	C	21	1.95E-34	chr:6:32648036
6	rs9275192	32656305	A	18.32	2.41E-34	chr:6:32648036
6	rs9275193	32656306	A	18.32	2.41E-34	chr:6:32648036
6	rs28371229	32647728	C	6.59	2.85E-34	chr:6:32648036
6	rs28371230	32647729	C	6.59	2.85E-34	chr:6:32648036
6	rs28371231	32647735	A	6.59	2.85E-34	chr:6:32648036
6	rs9275003	32642769	C	47.9	3.21E-34	chr:6:32648036
6	rs9275026	32643318	T	6.705	3.30E-34	chr:6:32648036
6	rs9275027	32643320	C	6.705	3.30E-34	chr:6:32648036
6	rs9275028	32643328	C	6.705	3.30E-34	chr:6:32648036
6	rs28755316	32646395	G	6.645	3.65E-34	chr:6:32648036
6	rs28755317	32646396	G	6.645	3.65E-34	chr:6:32648036
6	rs9274874	32640849	T	21.12	4.09E-34	chr:6:32648036
6	rs9274825	32639710	C	23.15	4.61E-34	chr:6:32648036
6	rs9274818	32639624	G	21.01	4.80E-34	chr:6:32648036
6	rs9274966	32642019	A	20.36	5.55E-34	chr:6:32648036
6	rs9274737	32637907	C	49.46	6.22E-34	chr:6:32648036
6	rs9274964	32641972	T	18.89	7.26E-34	chr:6:32648036
6	rs9274965	32641984	A	18.89	7.26E-34	chr:6:32648036
6	rs9274823	32639695	T	22.85	7.44E-34	chr:6:32648036
6	rs9274821	32639670	G	21.04	7.56E-34	chr:6:32648036
6	rs9274826	32639725	A	22.65	7.64E-34	chr:6:32648036
6	rs9274827	32639726	T	22.65	7.64E-34	chr:6:32648036
6	rs201341530	32639456	C	33.87	8.13E-34	chr:6:32648036
6	rs202086737	32639459	G	33.87	8.13E-34	chr:6:32648036
6	rs9275029	32643336	A	6.436	8.19E-34	chr:6:32648036
6	rs9274706	32637265	G	30.88	8.26E-34	chr:6:32648036
6	rs9274982	32642335	A	19.84	8.81E-34	chr:6:32648036
6	rs9274983	32642337	C	19.84	8.81E-34	chr:6:32648036
6	rs28755319	32646445	C	6.525	1.01E-33	chr:6:32648036
6	rs28755347	32646955	T	6.721	1.05E-33	chr:6:32648036
6	rs28755322	32646455	A	6.423	1.11E-33	chr:6:32648036
6	rs9274868	32640752	G	33.39	1.11E-33	chr:6:32648036
6	rs9274812	32639573	T	52.16	1.16E-33	chr:6:32648036
6	rs9274970	32642095	T	63.03	1.63E-33	chr:6:32648036
6	rs9274981	32642313	G	19.14	2.46E-33	chr:6:32648036
6	rs9274876	32640864	G	15.82	3.15E-33	chr:6:32648036
6	rs9275077	32648565	C	54.71	3.70E-33	chr:6:32648036
6	rs9274735	32637896	A	52.7	3.75E-33	chr:6:32648036
6	rs28755320	32646449	C	6.427	4.00E-33	chr:6:32648036
6	rs28755321	32646451	T	6.427	4.00E-33	chr:6:32648036
6	rs9274936	32641625	T	25.93	4.06E-33	chr:6:32648036
6	rs9274989	32642424	A	18.99	4.17E-33	chr:6:32648036

6	rs28755318	32646416	T	6.072	4.74E-33	chr:6:32648036
6	rs28371232	32647761	T	6.056	5.25E-33	chr:6:32648036
6	rs28371233	32647762	G	6.056	5.25E-33	chr:6:32648036
6	rs9274709	32637275	G	37.14	5.95E-33	chr:6:32648036
6	rs9274879	32640918	G	29.64	6.58E-33	chr:6:32648036
6	rs9275072	32648502	T	60.68	7.12E-33	chr:6:32648036
6	rs9274979	32642280	T	17.51	9.89E-33	chr:6:32648036
6	rs9274945	32641796	C	15.34	1.13E-32	chr:6:32648036
6	rs9274704	32637213	G	40.39	1.19E-32	chr:6:32648036
6	rs9275009	32642958	A	63.05	1.19E-32	chr:6:32648036
6	rs28755336	32646798	A	10.66	1.58E-32	chr:6:32648036
6	rs28755337	32646799	C	10.66	1.58E-32	chr:6:32648036
6	rs9275000	32642719	T	59.83	1.62E-32	chr:6:32648036
6	rs9274847	32640143	G	59.31	1.79E-32	chr:6:32648036
6	rs9274992	32642577	G	50.78	2.06E-32	chr:6:32648036
6	rs9274938	32641673	C	39.24	2.88E-32	chr:6:32648036
6	rs9274862	32640595	T	48.94	4.63E-32	chr:6:32648036
6	rs9274711	32637289	T	49.39	5.12E-32	chr:6:32648036
6	rs28371235	32647786	G	5.669	5.51E-32	chr:6:32648036
6	rs9274939	32641751	A	14.8	6.38E-32	chr:6:32648036
6	rs9274993	32642582	A	55.54	8.64E-32	chr:6:32648036
6	rs28371234	32647779	A	5.685	9.40E-32	chr:6:32648036
6	rs9274984	32642378	C	15.56	1.08E-31	chr:6:32648036
6	rs9274985	32642379	A	15.56	1.08E-31	chr:6:32648036
6	rs9274700	32637180	G	47.6	1.19E-31	chr:6:32648036
6	rs28371236	32647803	G	5.493	1.46E-31	chr:6:32648036
6	rs28371237	32647816	G	5.493	1.46E-31	chr:6:32648036
6	rs9274972	32642126	T	66.75	2.18E-31	chr:6:32648036
6	rs9274948	32641847	T	13.85	2.47E-31	chr:6:32648036
6	rs9274863	32640611	C	53.43	2.58E-31	chr:6:32648036
6	rs2935909	32655962	G	28.48	2.94E-31	chr:6:32648036
6	rs3021056	32655921	T	28.48	2.94E-31	chr:6:32648036
6	rs9274688	32636900	T	53.41	3.08E-31	chr:6:32648036
6	rs9274882	32641066	T	8.979	3.33E-31	chr:6:32648036
6	rs9274947	32641832	A	13.9	3.51E-31	chr:6:32648036
6	rs9274793	32639247	C	64.57	3.68E-31	chr:6:32648036
6	rs9274963	32641942	C	14.47	4.17E-31	chr:6:32648036
6	rs9274941	32641755	G	13.23	4.45E-31	chr:6:32648036
6	rs9275079	32648614	C	65.06	4.52E-31	chr:6:32648036
6	rs9275080	32648627	T	65.06	4.52E-31	chr:6:32648036
6	rs9274888	32641142	A	7.28	2.00E-30	chr:6:32648036
6	rs28755355	32647058	C	8.615	2.27E-30	chr:6:32648036
6	rs28755358	32647062	A	8.615	2.27E-30	chr:6:32648036

6	rs28755359	32647064	T	8.615	2.27E-30	chr:6:32648036
6	rs9274950	32641864	T	12.29	2.50E-30	chr:6:32648036
6	rs9274890	32641148	A	6.988	3.33E-30	chr:6:32648036
6	rs9275083	32648694	C	70.48	3.35E-30	chr:6:32648036
6	rs9274777	32638686	G	5.371	4.37E-30	chr:6:32648036
6	rs9274783	32638816	G	5.371	4.37E-30	chr:6:32648036
6	rs9274924	32641403	T	5.353	7.16E-30	chr:6:32648036
6	rs9274842	32640095	A	65.58	7.42E-30	chr:6:32648036
6	rs9274742	32638037	C	86.09	7.60E-30	chr:6:32648036
6	rs9275157	32652552	A	36.98	1.02E-29	chr:6:32648036
6	rs9275156	32652486	C	42.42	1.74E-29	chr:6:32648036
6	rs9274976	32642220	A	82.68	2.06E-29	chr:6:32648036
6	rs9274925	32641411	T	5.199	2.15E-29	chr:6:32648036
6	rs9274885	32641081	T	7.595	2.91E-29	chr:6:32648036
6	rs9274975	32642187	G	81.32	2.94E-29	chr:6:32648036
6	rs9274789	32639143	C	50.95	3.00E-29	chr:6:32648036
6	rs9274892	32641161	G	6.424	4.50E-29	chr:6:32648036
6	rs9275187	32656038	C	5.414	5.23E-29	chr:6:32648036
6	rs9274884	32641078	C	7.559	5.33E-29	chr:6:32648036
6	rs9275190	32656228	G	5.347	5.36E-29	chr:6:32648036
6	rs9275191	32656235	C	5.347	5.36E-29	chr:6:32648036
6	rs9275194	32656334	C	5.326	6.12E-29	chr:6:32648036
6	rs9275006	32642852	T	80.79	6.20E-29	chr:6:32648036
6	rs9274791	32639163	T	60.69	6.25E-29	chr:6:32648036
6	rs9274893	32641165	T	6.301	7.96E-29	chr:6:32648036
6	rs9274728	32637772	G	80.88	8.60E-29	chr:6:32648036
6	rs9274928	32641484	T	7.669	9.13E-29	chr:6:32648036
6	rs9274897	32641195	C	5.405	1.41E-28	chr:6:32648036
6	rs9274712	32637298	T	19.44	1.43E-28	chr:6:32648036
6	rs9274921	32641341	T	5.024	1.75E-28	chr:6:32648036
6	rs9275090	32648971	G	73.16	2.84E-28	chr:6:32648036
6	rs9274699	32637096	A	72.54	3.07E-28	chr:6:32648036
6	rs9274920	32641328	A	4.977	3.80E-28	chr:6:32648036
6	rs9274894	32641180	G	5.795	6.41E-28	chr:6:32648036
6	rs9275004	32642839	G	92.38	1.13E-27	chr:6:32648036
6	rs9274811	32639511	T	89.45	1.29E-27	chr:6:32648036
6	rs7750477	32651707	G	66.59	1.42E-27	chr:6:32648036
6	rs9274903	32641221	A	4.887	1.67E-27	chr:6:32648036
6	rs9275159	32652607	T	65.65	1.84E-27	chr:6:32648036
6	rs17205373	32626210	G	10.99	3.70E-27	chr:6:32648036
6	rs9273508	32628439	A	10.88	5.58E-27	chr:6:32648036
6	rs9274958	32641915	G	10.26	6.43E-27	chr:6:32648036
6	rs9274959	32641916	C	10.26	6.43E-27	chr:6:32648036

6	rs9274960	32641917	A	10.26	6.43E-27	chr:6:32648036
6	rs1048027	32609169	C	8.284	6.51E-27	chr:6:32648036
6	rs9275318	32666173	G	14.02	6.98E-27	chr:6:32648036
6	rs9275319	32666295	G	14.02	6.98E-27	chr:6:32648036
6	rs3021062	32651741	T	80.83	9.04E-27	chr:6:32648036
6	rs9275183	32654502	G	13.9	9.14E-27	chr:6:32648036
6	rs9273471	32628030	A	8.149	1.02E-26	chr:6:32648036
6	rs2516006	32650268	C	11.32	1.24E-26	chr:6:32648036
6	rs9275205	32657560	C	11.32	1.24E-26	chr:6:32648036
6	rs1049087	32629889	A	11.09	1.35E-26	chr:6:32648036
6	rs9274757	32638280	C	111.4	1.50E-26	chr:6:32648036
6	rs9275188	32656136	T	5.239	2.02E-26	chr:6:32648036
6	rs9275189	32656137	G	5.239	2.02E-26	chr:6:32648036
6	rs2647082	32628146	A	7.884	2.10E-26	chr:6:32648036
6	rs9274840	32640035	T	109.1	2.18E-26	chr:6:32648036
6	rs9274841	32640059	A	109.1	2.18E-26	chr:6:32648036
6	rs9274767	32638541	G	109.4	2.30E-26	chr:6:32648036
6	rs9274768	32638544	C	109.4	2.30E-26	chr:6:32648036
6	rs281874807	32631448	G	5.859	2.51E-26	chr:6:32648036
6	rs9273866	32629639	C	12.92	2.98E-26	chr:6:32648036
6	rs9274906	32641252	A	4.489	3.28E-26	chr:6:32648036
6	rs9274880	32641011	G	5.1	3.82E-26	chr:6:32648036
6	rs9274779	32638726	A	74.72	3.88E-26	chr:6:32648036
6	rs3021059	32652344	T	74.66	3.91E-26	chr:6:32648036
6	rs79471319	32627777	C	13.25	4.62E-26	chr:6:32648036
6	rs9274782	32638795	C	73.62	4.94E-26	chr:6:32648036
6	rs3830059	32634007	G	12.97	5.09E-26	chr:6:32648036
6	rs9274854	32640473	T	102.9	5.81E-26	chr:6:32648036
6	rs1130373	32634301	C	12.89	6.21E-26	chr:6:32648036
6	rs9273502	32628389	C	12.58	7.65E-26	chr:6:32648036
6	rs9273503	32628390	T	12.58	7.65E-26	chr:6:32648036
6	rs9274935	32641580	C	4.517	8.13E-26	chr:6:32648036
6	rs79350521	32503147	T	8.214	9.39E-26	chr:6:32648036
6	rs3828796	32635974	G	12.29	1.07E-25	chr:6:32648036
6	rs3828800	32636063	A	12.26	1.10E-25	chr:6:32648036
6	rs3828801	32636071	T	12.26	1.10E-25	chr:6:32648036
6	rs9275211	32657844	C	10.39	1.23E-25	chr:6:32648036
6	rs28383432	32608880	A	6.281	1.29E-25	chr:6:32648036
6	rs28746846	32634261	T	12.61	1.51E-25	chr:6:32648036
6	rs28746847	32634262	G	12.61	1.51E-25	chr:6:32648036
6	rs28371247	32648003	C	11.77	1.56E-25	chr:6:32648036
6	rs9274654	32636280	T	12.08	1.59E-25	chr:6:32648036
6	rs9274655	32636289	C	12.08	1.59E-25	chr:6:32648036

6	rs9274656	32636351	C	12.08	1.59E-25	chr:6:32648036
6	rs9274661	32636443	C	12.08	1.59E-25	chr:6:32648036
6	rs9274785	32638907	C	97.52	1.81E-25	chr:6:32648036
6	rs2856677	32656815	T	97.55	1.95E-25	chr:6:32648036
6	rs9274784	32638878	G	96.88	2.05E-25	chr:6:32648036
6	rs28746835	32633959	C	13.04	2.29E-25	chr:6:32648036
6	rs3828799	32636062	C	11.94	2.80E-25	chr:6:32648036
6	rs9274937	32641631	A	4.675	3.02E-25	chr:6:32648036
6	rs1694122	32642845	G	10.43	3.08E-25	chr:6:32648036
6	rs3828792	32635828	C	11.91	3.12E-25	chr:6:32648036
6	rs9275347	32667623	A	11.88	3.19E-25	chr:6:32648036
6	rs9275149	32651616	T	94.16	3.34E-25	chr:6:32648036
6	rs9275150	32651617	G	94.16	3.34E-25	chr:6:32648036
6	rs9273495	32628273	A	6.616	3.39E-25	chr:6:32648036
6	rs9274917	32641317	C	4.451	4.12E-25	chr:6:32648036
6	rs9274918	32641318	A	4.451	4.12E-25	chr:6:32648036
6	rs201639066	32516705	T	6.834	4.71E-25	chr:6:32648036
6	rs9275165	32652956	G	93.19	4.89E-25	chr:6:32648036
6	rs9275166	32652963	C	93.19	4.89E-25	chr:6:32648036
6	rs9274691	32636957	C	5.044	5.27E-25	chr:6:32648036
6	rs9274692	32636958	A	5.044	5.27E-25	chr:6:32648036
6	rs9275164	32652929	C	92.42	5.52E-25	chr:6:32648036
6	rs9274649	32636217	A	11.39	7.45E-25	chr:6:32648036
6	rs9274919	32641320	G	4.326	8.78E-25	chr:6:32648036
6	rs3828809	32636172	T	11.71	8.78E-25	chr:6:32648036
6	rs28746825	32633472	G	11.94	9.03E-25	chr:6:32648036
6	rs9272316	32604050	T	6.666	9.08E-25	chr:6:32648036
6	rs9274780	32638741	G	89.24	9.35E-25	chr:6:32648036
6	rs9275195	32656405	C	5.056	1.03E-24	chr:6:32648036
6	rs9275196	32656406	A	5.056	1.03E-24	chr:6:32648036
6	rs3828787	32635743	C	11.92	1.20E-24	chr:6:32648036
6	rs3828788	32635749	G	11.92	1.20E-24	chr:6:32648036
6	rs3828808	32636138	G	11.45	1.22E-24	chr:6:32648036
6	rs9274640	32636154	G	11.45	1.22E-24	chr:6:32648036
6	rs28746838	32634057	T	11.53	1.22E-24	chr:6:32648036
6	rs9274693	32636972	T	5.104	1.44E-24	chr:6:32648036
6	rs9274765	32638510	T	130.3	2.07E-24	chr:6:32648036
6	rs9274766	32638512	A	130.3	2.07E-24	chr:6:32648036
6	rs28383373	32607193	C	4.404	2.30E-24	chr:6:32648036
6	rs1049072	32634355	A	10.99	2.35E-24	chr:6:32648036
6	rs28383372	32607174	T	4.375	2.74E-24	chr:6:32648036
6	rs1049070	32634425	T	11	3.00E-24	chr:6:32648036
6	rs9274739	32637940	T	129.2	3.23E-24	chr:6:32648036

6	rs9273371	32626565	T	7.945	3.34E-24	chr:6:32648036
6	rs28724252	32629549	A	11.89	4.25E-24	chr:6:32648036
6	rs281863614	32630788	A	4.904	4.78E-24	chr:6:32648036
6	rs9274787	32639028	A	125.9	4.96E-24	chr:6:32648036
6	rs9274788	32639036	C	125.9	4.96E-24	chr:6:32648036
6	rs28746834	32633899	C	12.47	5.08E-24	chr:6:32648036
6	rs9274775	32638643	G	123	5.65E-24	chr:6:32648036
6	rs2856683	32655218	G	122.8	6.11E-24	chr:6:32648036
6	rs7774434	32657578	C	6.34	6.13E-24	chr:6:32648036
6	rs281874979	32631295	A	4.784	7.66E-24	chr:6:32648036
6	rs4947342	32653070	A	120.6	8.07E-24	chr:6:32648036
6	rs9275171	32653972	G	77.28	9.47E-24	chr:6:32648036
6	rs9275175	32654147	A	75.59	1.42E-23	chr:6:32648036
6	rs9275176	32654157	G	75.59	1.42E-23	chr:6:32648036
6	rs9275147	32651557	T	117	1.43E-23	chr:6:32648036
6	rs9273513	32628506	A	12.22	1.72E-23	chr:6:32648036
6	rs9273821	32629543	C	11.28	1.82E-23	chr:6:32648036
6	rs9274710	32637287	G	5.019	2.52E-23	chr:6:32648036
6	rs9273491	32628221	A	13.39	2.53E-23	chr:6:32648036
6	rs28746845	32634150	C	10.04	2.72E-23	chr:6:32648036
6	rs28746851	32634506	T	10.96	2.81E-23	chr:6:32648036
6	rs11568830	32634556	A	11.35	3.06E-23	chr:6:32648036
6	rs9275155	32652484	A	5.151	3.09E-23	chr:6:32648036
6	rs7745002	32615421	G	4.309	3.89E-23	chr:6:32648036
6	rs9274815	32639620	G	3.57	3.93E-23	chr:6:32648036
6	rs115749644	32627818	A	13.22	4.87E-23	chr:6:32648036
6	rs9272518	32606446	C	48.18	4.92E-23	chr:6:32648036
6	rs1770	32627833	G	5.169	5.02E-23	chr:6:32648036
6	rs1049073	32632717	A	5.974	5.63E-23	chr:6:32648036
6	rs9273537	32628765	T	4.898	5.92E-23	chr:6:32648036
6	rs3021057	32652363	T	5.196	5.98E-23	chr:6:32648036
6	rs9274696	32637007	C	5.108	6.11E-23	chr:6:32648036
6	rs9273528	32628633	C	10.67	6.29E-23	chr:6:32648036
6	rs281863219	32631570	A	10.33	6.59E-23	chr:6:32648036
6	rs76585788	32637493	A	13.02	7.34E-23	chr:6:32648036
6	rs9274702	32637184	A	5.335	7.39E-23	chr:6:32648036
6	rs28383356	32606646	C	3.916	7.72E-23	chr:6:32648036
6	rs11568831	32634545	A	11.07	7.98E-23	chr:6:32648036
6	rs281863304	32631473	T	9.915	8.76E-23	chr:6:32648036
6	rs9274748	32638086	A	5.502	1.01E-22	chr:6:32648036
6	rs9274749	32638094	A	5.502	1.01E-22	chr:6:32648036
6	rs9273489	32628189	G	14.24	1.05E-22	chr:6:32648036
6	rs28724258	32630093	T	7.583	1.09E-22	chr:6:32648036

6	rs9274062	32630094	G	7.583	1.09E-22	chr:6:32648036
6	rs9274065	32630099	T	7.583	1.09E-22	chr:6:32648036
6	rs9274697	32637016	G	5.087	1.21E-22	chr:6:32648036
6	rs9273492	32628229	A	11.77	1.23E-22	chr:6:32648036
6	rs281863734	32630620	T	8.787	1.29E-22	chr:6:32648036
6	rs28383400	32607950	A	4.697	1.47E-22	chr:6:32648036
6	rs9275158	32652582	A	5.043	1.56E-22	chr:6:32648036
6	rs9274705	32637245	A	4.654	1.79E-22	chr:6:32648036
6	rs9275154	32652435	T	5.138	1.83E-22	chr:6:32648036
6	rs3129783	32655730	G	5.372	1.87E-22	chr:6:32648036
6	rs28746829	32633767	T	11.23	2.00E-22	chr:6:32648036
6	rs28746830	32633781	T	11.23	2.00E-22	chr:6:32648036
6	rs9274731	32637823	G	4.733	2.38E-22	chr:6:32648036
6	rs9274707	32637266	G	4.471	2.88E-22	chr:6:32648036
6	rs9272365	32604621	C	4.276	3.05E-22	chr:6:32648036
6	rs9274730	32637817	A	4.729	3.20E-22	chr:6:32648036
6	rs28746843	32634137	C	9.66	3.23E-22	chr:6:32648036
6	rs28746844	32634139	T	9.66	3.23E-22	chr:6:32648036
6	rs28383387	32607575	T	3.773	3.28E-22	chr:6:32648036
6	rs9274778	32638694	A	5.146	3.36E-22	chr:6:32648036
6	rs9274708	32637267	A	4.461	3.58E-22	chr:6:32648036
6	rs28746841	32634094	C	9.252	3.60E-22	chr:6:32648036
6	rs28746842	32634098	G	9.252	3.60E-22	chr:6:32648036
6	rs9274907	32641281	A	5.016	4.29E-22	chr:6:32648036
6	rs28746852	32634593	C	10.86	4.70E-22	chr:6:32648036
6	rs2856688	32654640	T	163.4	4.97E-22	chr:6:32648036
6	rs9274750	32638114	C	164.3	4.97E-22	chr:6:32648036
6	rs9275113	32649825	G	162.2	5.13E-22	chr:6:32648036
6	rs9273818	32629531	G	10.49	5.55E-22	chr:6:32648036
6	rs28746839	32634090	C	9.373	5.84E-22	chr:6:32648036
6	rs28746840	32634091	C	9.373	5.84E-22	chr:6:32648036
6	rs17206238	32665761	T	10.53	6.28E-22	chr:6:32648036
6	rs1794495	32649347	C	10.53	6.28E-22	chr:6:32648036
6	rs1794497	32649180	C	10.53	6.28E-22	chr:6:32648036
6	rs2646994	32665813	T	10.53	6.28E-22	chr:6:32648036
6	rs2646995	32650492	A	10.53	6.28E-22	chr:6:32648036
6	rs2647002	32657082	G	10.53	6.28E-22	chr:6:32648036
6	rs2647005	32664760	G	10.53	6.28E-22	chr:6:32648036
6	rs2647007	32664677	G	10.53	6.28E-22	chr:6:32648036
6	rs2647014	32664279	T	10.53	6.28E-22	chr:6:32648036
6	rs2647015	32664093	G	10.53	6.28E-22	chr:6:32648036
6	rs2647017	32663772	G	10.53	6.28E-22	chr:6:32648036
6	rs28371252	32653732	A	10.53	6.28E-22	chr:6:32648036

6	rs28371258	32658513	C	10.53	6.28E-22	chr:6:32648036
6	rs28371267	32663578	T	10.53	6.28E-22	chr:6:32648036
6	rs2856669	32663360	A	10.53	6.28E-22	chr:6:32648036
6	rs2856673	32659822	T	10.53	6.28E-22	chr:6:32648036
6	rs2858303	32650550	C	10.53	6.28E-22	chr:6:32648036
6	rs2858313	32663919	G	10.53	6.28E-22	chr:6:32648036
6	rs2858325	32660366	G	10.53	6.28E-22	chr:6:32648036
6	rs74379773	32662724	C	10.53	6.28E-22	chr:6:32648036
6	rs3129784	32656608	T	5.109	6.31E-22	chr:6:32648036
6	rs2647025	32635949	A	162.6	6.49E-22	chr:6:32648036
6	rs2858316	32662501	T	10.32	6.78E-22	chr:6:32648036
6	rs9274740	32637968	T	5.778	7.11E-22	chr:6:32648036
6	rs9274878	32640889	G	3.992	7.18E-22	chr:6:32648036
6	rs9272362	32604564	T	4.18	7.44E-22	chr:6:32648036
6	rs9273558	32628965	G	4.918	8.32E-22	chr:6:32648036
6	rs9275144	32651218	A	157.2	8.42E-22	chr:6:32648036
6	rs28746827	32633636	A	12.41	8.45E-22	chr:6:32648036
6	rs9274690	32636940	C	4.913	8.65E-22	chr:6:32648036
6	rs9275068	32648418	C	10.04	8.82E-22	chr:6:32648036
6	rs2856691	32653435	A	155.9	9.41E-22	chr:6:32648036
6	rs1694111	32648753	T	11.12	1.01E-21	chr:6:32648036
6	rs9272358	32604538	A	4.12	1.01E-21	chr:6:32648036
6	rs2858323	32660707	T	10.36	1.02E-21	chr:6:32648036
6	rs9275140	32651018	A	155.4	1.03E-21	chr:6:32648036
6	rs9274715	32637391	A	4.321	1.08E-21	chr:6:32648036
6	rs9274689	32636930	C	4.9	1.18E-21	chr:6:32648036
6	rs9274698	32637095	C	5.603	1.22E-21	chr:6:32648036
6	rs9274679	32636653	C	5.023	1.25E-21	chr:6:32648036
6	rs9273526	32628617	C	9.815	1.32E-21	chr:6:32648036
6	rs28746853	32634646	C	10.74	1.40E-21	chr:6:32648036
6	rs9274713	32637341	T	4.962	1.56E-21	chr:6:32648036
6	rs28371265	32659263	C	10.31	1.60E-21	chr:6:32648036
6	rs9274790	32639155	G	4.989	1.63E-21	chr:6:32648036
6	rs9274683	32636780	A	5.6	1.66E-21	chr:6:32648036
6	rs9274684	32636785	T	5.6	1.66E-21	chr:6:32648036
6	rs28724250	32629530	T	10.05	1.88E-21	chr:6:32648036
6	rs1794491	32640041	A	10.69	1.95E-21	chr:6:32648036
6	rs9273525	32628605	C	9.869	2.16E-21	chr:6:32648036
6	rs3997848	32682394	G	5.964	2.32E-21	chr:6:32648036
6	rs28746861	32635120	A	10.65	2.46E-21	chr:6:32648036
6	rs9274875	32640863	G	3.597	2.50E-21	chr:6:32648036
6	rs522308	32581922	T	6.721	2.52E-21	chr:6:32648036
6	rs76164808	32637390	C	12.89	2.60E-21	chr:6:32648036

6	rs9274943	32641784	T	3.226	3.10E-21	chr:6:32648036
6	rs9274685	32636844	C	5.42	3.14E-21	chr:6:32648036
6	rs2856680	32655606	G	9.819	3.15E-21	chr:6:32648036
6	rs2856682	32655405	T	9.819	3.15E-21	chr:6:32648036
6	rs2647008	32661515	C	9.829	3.16E-21	chr:6:32648036
6	rs2647009	32661615	A	9.829	3.16E-21	chr:6:32648036
6	rs2647010	32661641	G	9.829	3.16E-21	chr:6:32648036
6	rs9275180	32654278	T	5.116	3.18E-21	chr:6:32648036
6	rs28746824	32633398	C	8.897	3.22E-21	chr:6:32648036
6	rs9274877	32640877	G	3.736	3.31E-21	chr:6:32648036
6	rs9274687	32636882	T	5.417	3.33E-21	chr:6:32648036
6	rs9275168	32653676	C	5.11	4.12E-21	chr:6:32648036
6	rs9275179	32654275	G	141.1	4.18E-21	chr:6:32648036
6	rs2856690	32654536	A	5.284	4.34E-21	chr:6:32648036
6	rs9274944	32641786	A	3.216	4.50E-21	chr:6:32648036
6	rs6927022	32612397	G	3.853	4.75E-21	chr:6:32648036
6	rs9274725	32637573	T	5.339	4.86E-21	chr:6:32648036
6	rs9274726	32637593	G	5.339	4.86E-21	chr:6:32648036
6	rs2935908	32652606	C	9.564	4.86E-21	chr:6:32648036
6	rs28746865	32635248	T	10.43	5.00E-21	chr:6:32648036
6	rs9274743	32638042	G	5.01	5.17E-21	chr:6:32648036
6	rs9272502	32606244	T	3.36	5.58E-21	chr:6:32648036
6	rs28371253	32654257	T	9.169	5.88E-21	chr:6:32648036
6	rs9273477	32628079	A	10.52	6.84E-21	chr:6:32648036
6	rs12192514	32674240	T	9.079	8.59E-21	chr:6:32648036
6	rs17212616	32674617	A	9.079	8.59E-21	chr:6:32648036
6	rs2647049	32672681	A	9.079	8.59E-21	chr:6:32648036
6	rs28371270	32674296	G	9.079	8.59E-21	chr:6:32648036
6	rs2856705	32670956	T	9.079	8.59E-21	chr:6:32648036
6	rs9275169	32653721	A	5.111	9.16E-21	chr:6:32648036
6	rs5019292	32733719	A	6.581	9.22E-21	chr:6:32648036
6	rs9275146	32651495	T	5.127	9.24E-21	chr:6:32648036
6	rs281863357	32631142	G	4.656	9.48E-21	chr:6:32648036
6	rs9275148	32651585	C	5.146	9.78E-21	chr:6:32648036
6	rs1826961	32603866	T	7.772	9.82E-21	chr:6:32648036
6	rs9275170	32653726	A	5.104	1.08E-20	chr:6:32648036
6	rs9275161	32652636	C	5.036	1.15E-20	chr:6:32648036
6	rs2856689	32654551	G	5.117	1.15E-20	chr:6:32648036
6	rs10456068	32659419	C	9.835	1.16E-20	chr:6:32648036
6	rs28371254	32657452	C	9.835	1.16E-20	chr:6:32648036
6	rs2856665	32665597	G	9.835	1.16E-20	chr:6:32648036
6	rs2856696	32651868	T	9.835	1.16E-20	chr:6:32648036
6	rs3021175	32651404	C	9.835	1.16E-20	chr:6:32648036

6	rs55673904	32645076	T	5.534	1.21E-20	chr:6:32648036
6	rs3021058	32652359	C	5.092	1.22E-20	chr:6:32648036
6	rs28371274	32674558	C	8.915	1.24E-20	chr:6:32648036
6	rs3997832	32637497	C	4.451	1.25E-20	chr:6:32648036
6	rs1130420	32627852	A	4.086	1.28E-20	chr:6:32648036
6	rs9274773	32638620	G	5.122	1.42E-20	chr:6:32648036
6	rs9274774	32638630	G	5.122	1.42E-20	chr:6:32648036
6	rs9274155	32630288	C	9.123	1.43E-20	chr:6:32648036
6	rs1694116	32647839	A	5.66	1.46E-20	chr:6:32648036
6	rs9275199	32656680	T	5.108	1.55E-20	chr:6:32648036
6	rs3021176	32652469	A	8.952	1.71E-20	chr:6:32648036
6	rs115341895	32649824	T	13.7	1.79E-20	chr:6:32648036
6	rs9274744	32638054	A	4.737	1.84E-20	chr:6:32648036
6	rs9274746	32638060	A	4.737	1.84E-20	chr:6:32648036
6	rs9274717	32637454	A	4.43	1.85E-20	chr:6:32648036
6	rs9274852	32640289	A	126.1	1.93E-20	chr:6:32648036
6	rs9273496	32628283	A	10.48	1.96E-20	chr:6:32648036
6	rs3104361	32652278	C	4.465	2.03E-20	chr:6:32648036
6	rs9275177	32654174	T	5.098	2.08E-20	chr:6:32648036
6	rs9275178	32654181	A	5.098	2.08E-20	chr:6:32648036
6	rs9274865	32640671	T	4.714	2.34E-20	chr:6:32648036
6	rs6928482	32626249	C	5.143	2.46E-20	chr:6:32648036
6	rs111903208	32661432	G	13.06	2.48E-20	chr:6:32648036
6	rs112117693	32662754	T	13.06	2.48E-20	chr:6:32648036
6	rs112317305	32667199	C	13.06	2.48E-20	chr:6:32648036
6	rs112535219	32662613	T	13.06	2.48E-20	chr:6:32648036
6	rs112574398	32658610	T	13.06	2.48E-20	chr:6:32648036
6	rs112608098	32665270	A	13.06	2.48E-20	chr:6:32648036
6	rs112670480	32661440	T	13.06	2.48E-20	chr:6:32648036
6	rs113138578	32661335	G	13.06	2.48E-20	chr:6:32648036
6	rs114218404	32664119	T	13.06	2.48E-20	chr:6:32648036
6	rs114325417	32666222	A	13.06	2.48E-20	chr:6:32648036
6	rs114744294	32660866	G	13.06	2.48E-20	chr:6:32648036
6	rs114801596	32663093	G	13.06	2.48E-20	chr:6:32648036
6	rs114996593	32658360	C	13.06	2.48E-20	chr:6:32648036
6	rs115262684	32666235	A	13.06	2.48E-20	chr:6:32648036
6	rs115577932	32663819	T	13.06	2.48E-20	chr:6:32648036
6	rs115777056	32664154	A	13.06	2.48E-20	chr:6:32648036
6	rs115842332	32666213	A	13.06	2.48E-20	chr:6:32648036
6	rs116147199	32658355	G	13.06	2.48E-20	chr:6:32648036
6	rs116459302	32666559	T	13.06	2.48E-20	chr:6:32648036
6	rs141748883	32663156	C	13.06	2.48E-20	chr:6:32648036
6	rs1694113	32650101	A	13.06	2.48E-20	chr:6:32648036

6	rs17427494	32665001	A	13.06	2.48E-20	chr:6:32648036
6	rs199923808	32663798	G	13.06	2.48E-20	chr:6:32648036
6	rs4713586	32659582	G	13.06	2.48E-20	chr:6:32648036
6	rs568935162	32662076	G	13.06	2.48E-20	chr:6:32648036
6	rs74756176	32664373	T	13.06	2.48E-20	chr:6:32648036
6	rs77537414	32664356	C	13.06	2.48E-20	chr:6:32648036
6	rs7754496	32657893	T	13.06	2.48E-20	chr:6:32648036
6	rs7775055	32657916	C	13.06	2.48E-20	chr:6:32648036
6	rs79951097	32662954	C	13.06	2.48E-20	chr:6:32648036
6	rs28371272	32674440	C	8.81	2.50E-20	chr:6:32648036
6	rs28371273	32674442	C	8.81	2.50E-20	chr:6:32648036
6	rs10456069	32659520	T	9.331	2.53E-20	chr:6:32648036
6	rs10456070	32659527	A	9.331	2.53E-20	chr:6:32648036
6	rs9272574	32607281	A	3.304	2.74E-20	chr:6:32648036
6	rs530423766	32610152	T	4.584	2.80E-20	chr:6:32648036
6	rs28746866	32635266	G	10.01	2.94E-20	chr:6:32648036
6	rs9274969	32642073	T	4.225	3.01E-20	chr:6:32648036
6	rs9274752	32638163	A	4.978	3.05E-20	chr:6:32648036
6	rs9274834	32639895	G	5.128	3.27E-20	chr:6:32648036
6	rs28746864	32635230	A	9.888	3.28E-20	chr:6:32648036
6	rs9274753	32638176	T	4.982	3.30E-20	chr:6:32648036
6	rs9274751	32638138	C	4.974	3.30E-20	chr:6:32648036
6	rs143607013	32658807	T	12.88	3.33E-20	chr:6:32648036
6	rs145250786	32658793	A	12.88	3.33E-20	chr:6:32648036
6	rs9275143	32651192	T	5.097	3.73E-20	chr:6:32648036
6	rs9274995	32642615	T	4.718	3.78E-20	chr:6:32648036
6	rs9275088	32648931	A	5.097	3.83E-20	chr:6:32648036
6	rs17211699	32626037	T	9.195	3.88E-20	chr:6:32648036
6	rs17211713	32626179	C	9.195	3.88E-20	chr:6:32648036
6	rs9275162	32652687	C	5.061	3.94E-20	chr:6:32648036
6	rs9275172	32653997	A	0.1957	3.98E-20	chr:6:32648036
6	rs9275173	32654037	T	0.1957	3.98E-20	chr:6:32648036
6	rs9275139	32651005	A	5.11	4.04E-20	chr:6:32648036
6	rs2858330	32658715	C	5.184	4.10E-20	chr:6:32648036
6	rs9275358	32667895	G	6.444	4.13E-20	chr:6:32648036
6	rs9274864	32640646	G	4.707	4.34E-20	chr:6:32648036
6	rs9274836	32639956	A	5.128	4.35E-20	chr:6:32648036
6	rs9275119	32650031	A	5.123	4.66E-20	chr:6:32648036
6	rs2647000	32656890	C	5.103	4.71E-20	chr:6:32648036
6	rs9275202	32656869	G	5.103	4.71E-20	chr:6:32648036
6	rs9275114	32649897	A	5.268	4.79E-20	chr:6:32648036
6	rs2856684	32655095	C	5.11	4.94E-20	chr:6:32648036
6	rs116540263	32671372	A	12.85	5.06E-20	chr:6:32648036

6	rs12527228	32679992	T	12.85	5.06E-20	chr:6:32648036
6	rs140882479	32663083	G	12.85	5.06E-20	chr:6:32648036
6	rs4713588	32659502	A	12.85	5.06E-20	chr:6:32648036
6	rs199884023	32639449	A	4.1	5.27E-20	chr:6:32648036
6	rs9274846	32640142	T	4.328	5.39E-20	chr:6:32648036
6	rs2856676	32656833	A	5.04	5.47E-20	chr:6:32648036
6	rs9275008	32642944	C	4.818	5.54E-20	chr:6:32648036
6	rs2856685	32655072	T	5.11	5.55E-20	chr:6:32648036
6	rs9274720	32637479	A	4.164	5.67E-20	chr:6:32648036
6	rs9274843	32640096	G	4.727	5.71E-20	chr:6:32648036
6	rs9274848	32640149	G	4.326	6.10E-20	chr:6:32648036
6	rs9274849	32640157	T	4.326	6.10E-20	chr:6:32648036
6	rs113194380	32671391	T	11.75	6.12E-20	chr:6:32648036
6	rs115276762	32674178	T	11.75	6.12E-20	chr:6:32648036
6	rs115911154	32674194	G	11.75	6.12E-20	chr:6:32648036
6	rs117296796	32674192	G	11.75	6.12E-20	chr:6:32648036
6	rs12525220	32675470	A	11.75	6.12E-20	chr:6:32648036
6	rs138256019	32673055	A	11.75	6.12E-20	chr:6:32648036
6	rs141345764	32673811	T	11.75	6.12E-20	chr:6:32648036
6	rs149005483	32674166	T	11.75	6.12E-20	chr:6:32648036
6	rs36030018	32667964	C	11.75	6.12E-20	chr:6:32648036
6	rs146335245	32662357	C	12.65	6.62E-20	chr:6:32648036
6	rs3129774	32603825	T	4.291	6.65E-20	chr:6:32648036
6	rs112032264	32660115	C	12.59	7.13E-20	chr:6:32648036
6	rs112466823	32660104	T	12.59	7.13E-20	chr:6:32648036
6	rs2647045	32668100	A	69.36	7.18E-20	chr:6:32648036
6	rs9274845	32640134	T	4.322	7.18E-20	chr:6:32648036
6	rs9273748	32629394	A	10.14	7.20E-20	chr:6:32648036
6	rs9275118	32649996	G	5.12	7.27E-20	chr:6:32648036
6	rs9275138	32650958	G	5.101	7.53E-20	chr:6:32648036
6	rs9275112	32649773	A	5.109	7.60E-20	chr:6:32648036
6	rs9274771	32638581	C	4.973	7.79E-20	chr:6:32648036
6	rs9274772	32638583	C	4.973	7.79E-20	chr:6:32648036
6	rs113529604	32652971	A	12.05	8.20E-20	chr:6:32648036
6	rs9275116	32649952	C	5.183	8.85E-20	chr:6:32648036
6	rs3210176	32627850	C	4.003	8.94E-20	chr:6:32648036
6	rs146797694	32658866	T	12.41	9.25E-20	chr:6:32648036
6	rs9272553	32607008	T	3.286	9.86E-20	chr:6:32648036
6	rs114079314	32580716	T	12.81	1.00E-19	chr:6:32648036
6	rs9274734	32637887	C	4.361	1.01E-19	chr:6:32648036
6	rs9272545	32606885	T	4.432	1.05E-19	chr:6:32648036
6	rs9275013	32643011	C	4.257	1.05E-19	chr:6:32648036
6	rs9274763	32638427	T	5.113	1.05E-19	chr:6:32648036

6	rs28755392	32678021	C	8.61	1.06E-19	chr:6:32648036
6	rs111937677	32639316	G	4.113	1.09E-19	chr:6:32648036
6	rs2856681	32655524	T	8.214	1.09E-19	chr:6:32648036
6	rs7754885	32657858	A	5.102	1.13E-19	chr:6:32648036
6	rs2856693	32653283	T	5.054	1.15E-19	chr:6:32648036
6	rs2856679	32655615	A	5.1	1.19E-19	chr:6:32648036
6	rs115064018	32643752	A	5.983	1.20E-19	chr:6:32648036
6	rs9274759	32638315	A	4.962	1.20E-19	chr:6:32648036
6	rs9275312	32665728	G	6.379	1.21E-19	chr:6:32648036
6	rs9275328	32666822	T	6.379	1.21E-19	chr:6:32648036
6	rs9275330	32666875	G	6.379	1.21E-19	chr:6:32648036
6	rs9275331	32666899	G	6.379	1.21E-19	chr:6:32648036
6	rs9275333	32666968	C	6.379	1.21E-19	chr:6:32648036
6	rs9275338	32667343	T	6.379	1.21E-19	chr:6:32648036
6	rs2856695	32651894	G	5.094	1.21E-19	chr:6:32648036
6	rs9274754	32638261	C	4.825	1.23E-19	chr:6:32648036
6	rs9274755	32638262	A	4.825	1.23E-19	chr:6:32648036
6	rs281874968	32631308	A	8.351	1.24E-19	chr:6:32648036
6	rs9274792	32639224	C	4.702	1.24E-19	chr:6:32648036
6	rs2858329	32658801	G	4.874	1.25E-19	chr:6:32648036
6	rs9275007	32642921	A	4.826	1.29E-19	chr:6:32648036
6	rs9275089	32648947	T	4.792	1.33E-19	chr:6:32648036
6	rs2856686	32654869	C	5.104	1.34E-19	chr:6:32648036
6	rs9275087	32648893	C	5.107	1.39E-19	chr:6:32648036
6	rs17212937	32681339	T	9.523	1.42E-19	chr:6:32648036
6	rs7764819	32680576	G	9.523	1.42E-19	chr:6:32648036
6	rs7765379	32680928	G	9.523	1.42E-19	chr:6:32648036
6	rs9275141	32651117	G	5.099	1.42E-19	chr:6:32648036
6	rs9275014	32643023	A	4.161	1.42E-19	chr:6:32648036
6	rs75098126	32636108	G	12.1	1.43E-19	chr:6:32648036
6	rs75128960	32636087	A	12.1	1.43E-19	chr:6:32648036
6	rs9274758	32638290	G	4.82	1.43E-19	chr:6:32648036
6	rs113374318	32639415	A	3.998	1.49E-19	chr:6:32648036
6	rs16898262	32677397	G	8.438	1.50E-19	chr:6:32648036
6	rs17212846	32675581	T	8.438	1.50E-19	chr:6:32648036
6	rs2856697	32673230	T	8.438	1.50E-19	chr:6:32648036
6	rs9274741	32637994	T	0.1961	1.51E-19	chr:6:32648036
6	rs9272380	32604732	A	3.519	1.51E-19	chr:6:32648036
6	rs9274718	32637469	C	3.99	1.53E-19	chr:6:32648036
6	rs9274719	32637470	C	3.99	1.53E-19	chr:6:32648036
6	rs2856667	32665079	C	5.103	1.55E-19	chr:6:32648036
6	rs9275145	32651363	C	0.1957	1.56E-19	chr:6:32648036
6	rs3104367	32603487	C	4.388	1.57E-19	chr:6:32648036

6	rs9275105	32649416	C	0.196	1.60E-19	chr:6:32648036
6	rs9275101	32649355	G	0.1959	1.62E-19	chr:6:32648036
6	rs9275102	32649361	G	0.1959	1.62E-19	chr:6:32648036
6	rs9274839	32640015	G	4.885	1.64E-19	chr:6:32648036
6	rs9275327	32666802	T	6.313	1.66E-19	chr:6:32648036
6	rs9274853	32640420	A	4.966	1.73E-19	chr:6:32648036
6	rs9275091	32648972	T	4.794	1.74E-19	chr:6:32648036
6	rs7754464	32657818	A	0.1961	1.76E-19	chr:6:32648036
6	rs9275103	32649386	T	0.1959	1.78E-19	chr:6:32648036
6	rs9275106	32649475	T	0.196	1.80E-19	chr:6:32648036
6	rs9275123	32650256	T	5.025	1.81E-19	chr:6:32648036
6	rs9275125	32650293	T	5.025	1.81E-19	chr:6:32648036
6	rs9275126	32650307	T	5.025	1.81E-19	chr:6:32648036
6	rs113666293	32656477	C	11.66	1.83E-19	chr:6:32648036
6	rs9275135	32650736	T	0.196	1.85E-19	chr:6:32648036
6	rs9275136	32650757	C	0.196	1.85E-19	chr:6:32648036
6	rs1049092	32629802	G	4.821	1.89E-19	chr:6:32648036
6	rs9275360	32667937	T	6.268	1.92E-19	chr:6:32648036
6	rs9275362	32667957	T	6.268	1.92E-19	chr:6:32648036
6	rs9275365	32668125	A	6.268	1.92E-19	chr:6:32648036
6	rs9274736	32637905	C	4.261	1.94E-19	chr:6:32648036
6	rs4642516	32657543	G	0.196	2.01E-19	chr:6:32648036
6	rs143801964	32645680	C	5.908	2.02E-19	chr:6:32648036
6	rs28746867	32635293	G	11.42	2.03E-19	chr:6:32648036
6	rs9272468	32605708	A	3.945	2.05E-19	chr:6:32648036
6	rs9274833	32639885	T	225.6	2.09E-19	chr:6:32648036
6	rs9274838	32639973	G	225.6	2.09E-19	chr:6:32648036
6	rs1049124	32627713	A	8.784	2.19E-19	chr:6:32648036
6	rs9272716	32609367	G	5.81	2.20E-19	chr:6:32648036
6	rs9275131	32650467	T	5.014	2.22E-19	chr:6:32648036
6	rs149999552	32658763	C	11.65	2.27E-19	chr:6:32648036
6	rs9274996	32642641	T	4.819	2.28E-19	chr:6:32648036
6	rs1794510	32652171	A	11.68	2.29E-19	chr:6:32648036
6	rs9274760	32638348	G	4.887	2.34E-19	chr:6:32648036
6	rs17843717	32630798	A	9.897	2.38E-19	chr:6:32648036
6	rs9274738	32637909	C	4.255	2.39E-19	chr:6:32648036
6	rs9274832	32639875	T	223.5	2.43E-19	chr:6:32648036
6	rs9274806	32639441	G	3.92	2.46E-19	chr:6:32648036
6	rs9275130	32650409	C	4.942	2.48E-19	chr:6:32648036
6	rs9274761	32638371	G	224.9	2.48E-19	chr:6:32648036
6	rs9274762	32638411	G	224.9	2.48E-19	chr:6:32648036
6	rs148781980	32667577	G	10.32	2.49E-19	chr:6:32648036
6	rs75084410	32636018	A	11.94	2.52E-19	chr:6:32648036

6	rs9274946	32641828	G	3.053	2.55E-19	chr:6:32648036
6	rs9274764	32638490	G	4.951	2.64E-19	chr:6:32648036
6	rs9274873	32640818	C	3.843	2.75E-19	chr:6:32648036
6	rs9274889	32641143	G	2.881	2.76E-19	chr:6:32648036
6	rs28383346	32605326	G	8.429	2.78E-19	chr:6:32648036
6	rs9275108	32649609	A	222.4	2.82E-19	chr:6:32648036
6	rs281863287	32631496	A	3.373	2.90E-19	chr:6:32648036
6	rs9274855	32640494	A	4.688	2.96E-19	chr:6:32648036
6	rs9275110	32649723	C	220.8	3.11E-19	chr:6:32648036
6	rs9275111	32649735	T	220.8	3.11E-19	chr:6:32648036
6	rs28746818	32633307	C	8.708	3.17E-19	chr:6:32648036
6	rs9275133	32650588	A	0.1993	3.22E-19	chr:6:32648036
6	rs9274805	32639429	T	3.917	3.23E-19	chr:6:32648036
6	rs9274756	32638269	T	4.679	3.27E-19	chr:6:32648036
6	rs9272467	32605705	T	3.94	3.37E-19	chr:6:32648036
6	rs2856692	32653385	G	218.9	3.46E-19	chr:6:32648036
6	rs3021060	32651846	C	218.9	3.46E-19	chr:6:32648036
6	rs3021061	32651839	G	218.9	3.46E-19	chr:6:32648036
6	rs3129785	32656724	T	218.9	3.46E-19	chr:6:32648036
6	rs9275100	32649308	G	218.9	3.46E-19	chr:6:32648036
6	rs9275104	32649388	C	218.9	3.46E-19	chr:6:32648036
6	rs9275107	32649508	G	218.9	3.46E-19	chr:6:32648036
6	rs9275121	32650185	G	218.9	3.46E-19	chr:6:32648036
6	rs9275122	32650233	C	218.9	3.46E-19	chr:6:32648036
6	rs9275127	32650314	T	218.9	3.46E-19	chr:6:32648036
6	rs9275132	32650500	A	218.9	3.46E-19	chr:6:32648036
6	rs9275134	32650612	G	218.9	3.46E-19	chr:6:32648036
6	rs9273408	32627221	G	10.96	3.66E-19	chr:6:32648036
6	rs77329518	32637136	T	12.34	3.69E-19	chr:6:32648036
6	rs9272736	32609671	A	4.351	3.70E-19	chr:6:32648036
6	rs9272737	32609673	A	4.351	3.70E-19	chr:6:32648036
6	rs9274973	32642147	G	4.83	3.72E-19	chr:6:32648036
6	rs9275115	32649941	C	217.3	3.81E-19	chr:6:32648036
6	rs9275117	32649965	T	217.3	3.81E-19	chr:6:32648036
6	rs9275093	32649047	T	217.2	3.83E-19	chr:6:32648036
6	rs9275094	32649062	C	217.2	3.83E-19	chr:6:32648036
6	rs9275096	32649096	T	217.2	3.83E-19	chr:6:32648036
6	rs3864306	32635012	A	9.659	4.09E-19	chr:6:32648036
6	rs9275086	32648809	G	4.937	4.11E-19	chr:6:32648036
6	rs9275182	32654390	T	216.5	4.20E-19	chr:6:32648036
6	rs3104362	32656697	G	215.7	4.21E-19	chr:6:32648036
6	rs28383306	32586739	T	8.47	4.25E-19	chr:6:32648036
6	rs9273354	32625952	G	4.167	4.25E-19	chr:6:32648036

6	rs9273355	32625960	G	4.165	4.35E-19	chr:6:32648036
6	rs9274727	32637696	G	215.2	4.46E-19	chr:6:32648036
6	rs9273370	32626492	G	0.2068	4.58E-19	chr:6:32648036
6	rs9501606	32196440	A	5.884	4.61E-19	chr:6:32648036
6	rs9275137	32650880	C	214.1	4.62E-19	chr:6:32648036
6	rs4321864	32399187	A	5.019	4.64E-19	chr:6:32648036
6	rs28383330	32600340	G	8.445	4.67E-19	chr:6:32648036
6	rs9275181	32654325	G	213.8	4.68E-19	chr:6:32648036
6	rs281863284	32631499	A	3.342	4.71E-19	chr:6:32648036
6	rs6914573	32639368	T	4.046	4.72E-19	chr:6:32648036
6	rs7774573	32657627	C	9.24	4.80E-19	chr:6:32648036
6	rs9274974	32642152	T	4.739	4.81E-19	chr:6:32648036
6	rs112453089	32639270	G	4.355	5.01E-19	chr:6:32648036
6	rs9275010	32642966	T	4.161	5.05E-19	chr:6:32648036
6	rs9275011	32642969	T	4.161	5.05E-19	chr:6:32648036
6	rs28383458	32609476	C	8.389	5.26E-19	chr:6:32648036
6	rs9274861	32640592	G	4.334	5.34E-19	chr:6:32648036
6	rs28383318	32590352	T	9.027	5.39E-19	chr:6:32648036
6	rs10947320	32581491	T	8.31	5.56E-19	chr:6:32648036
6	rs28746854	32634755	T	9.037	5.72E-19	chr:6:32648036
6	rs28746855	32634756	G	9.037	5.72E-19	chr:6:32648036
6	rs1140343	32629137	G	3.26	5.94E-19	chr:6:32648036
6	rs9272725	32609513	G	0.2135	5.99E-19	chr:6:32648036
6	rs2858308	32670000	T	8.49	6.09E-19	chr:6:32648036
6	rs3828806	32636130	T	4.33	6.10E-19	chr:6:32648036
6	rs3828807	32636131	C	4.33	6.10E-19	chr:6:32648036
6	rs9274769	32638547	C	4.663	7.09E-19	chr:6:32648036
6	rs9272441	32605380	T	0.2167	7.20E-19	chr:6:32648036
6	rs34719927	32609514	C	0.2167	7.58E-19	chr:6:32648036
6	rs281875029	32631237	A	8.108	7.59E-19	chr:6:32648036
6	rs1694123	32642759	G	9.839	7.86E-19	chr:6:32648036
6	rs9273374	32626614	A	0.2165	8.00E-19	chr:6:32648036
6	rs9275085	32648735	A	4.686	8.09E-19	chr:6:32648036
6	rs9272578	32607389	C	4.133	8.10E-19	chr:6:32648036
6	rs9274794	32639264	G	4.343	8.17E-19	chr:6:32648036
6	rs375846271	32640725	T	12.27	8.32E-19	chr:6:32648036
6	rs562092898	32629544	A	13.17	8.47E-19	chr:6:32648036
6	rs12192104	32580703	C	8.158	8.52E-19	chr:6:32648036
6	rs12206257	32576036	T	8.158	8.52E-19	chr:6:32648036
6	rs13193798	32574991	A	8.158	8.52E-19	chr:6:32648036
6	rs13206639	32578100	A	8.158	8.52E-19	chr:6:32648036
6	rs17211475	32602285	G	8.158	8.52E-19	chr:6:32648036
6	rs28383226	32583899	G	8.158	8.52E-19	chr:6:32648036

6	rs28383229	32584024	G	8.158	8.52E-19	chr:6:32648036
6	rs28383315	32587281	G	8.158	8.52E-19	chr:6:32648036
6	rs28383319	32591038	G	8.158	8.52E-19	chr:6:32648036
6	rs28383325	32596310	C	8.158	8.52E-19	chr:6:32648036
6	rs28383327	32599077	C	8.158	8.52E-19	chr:6:32648036
6	rs28383348	32605825	A	8.158	8.52E-19	chr:6:32648036
6	rs28383459	32609520	A	8.158	8.52E-19	chr:6:32648036
6	rs28383467	32610330	T	8.158	8.52E-19	chr:6:32648036
6	rs28752513	32583459	C	8.158	8.52E-19	chr:6:32648036
6	rs9273444	32627635	T	0.2225	8.70E-19	chr:6:32648036
6	rs9273372	32626590	C	0.2165	8.79E-19	chr:6:32648036
6	rs9273373	32626601	A	0.2165	8.79E-19	chr:6:32648036
6	rs147328574	32643801	G	5.947	8.80E-19	chr:6:32648036
6	rs2308891	32610009	G	4.467	8.89E-19	chr:6:32648036
6	rs113773636	32673719	G	11.16	8.94E-19	chr:6:32648036
6	rs143895212	32673761	T	11.16	8.94E-19	chr:6:32648036
6	rs9274660	32636434	A	0.2166	9.04E-19	chr:6:32648036
6	rs28383344	32605067	G	8.093	9.16E-19	chr:6:32648036
6	rs9274997	32642673	T	4.546	9.34E-19	chr:6:32648036
6	rs28383335	32602892	T	8.093	9.41E-19	chr:6:32648036
6	rs28477203	32599509	T	8.093	9.41E-19	chr:6:32648036
6	rs111335405	32655722	G	10.76	9.54E-19	chr:6:32648036
6	rs9273445	32627646	A	0.2226	9.73E-19	chr:6:32648036
6	rs6919468	32582903	T	13.59	1.02E-18	chr:6:32648036
6	rs1049086	32629904	A	0.2165	1.06E-18	chr:6:32648036
6	rs1130034	32605274	T	0.2165	1.06E-18	chr:6:32648036
6	rs9273053	32612050	T	2.968	1.10E-18	chr:6:32648036
6	rs281863425	32631044	A	9.189	1.13E-18	chr:6:32648036
6	rs281863426	32631043	C	9.189	1.13E-18	chr:6:32648036
6	rs9274881	32641065	C	3.059	1.14E-18	chr:6:32648036
6	rs111983899	32639389	T	3.827	1.21E-18	chr:6:32648036
6	rs28383345	32605234	A	8.022	1.21E-18	chr:6:32648036
6	rs9274891	32641152	C	2.809	1.21E-18	chr:6:32648036
6	rs114816840	32610214	C	3.955	1.28E-18	chr:6:32648036
6	rs116613962	32610220	G	3.955	1.28E-18	chr:6:32648036
6	rs28383300	32586611	T	8.891	1.35E-18	chr:6:32648036
6	rs9273509	32628473	T	0.2325	1.35E-18	chr:6:32648036
6	rs9273510	32628474	G	0.2325	1.35E-18	chr:6:32648036
6	rs9272528	32606617	G	2.992	1.37E-18	chr:6:32648036
6	rs28383321	32591411	C	8.156	1.39E-18	chr:6:32648036
6	rs1063355	32627714	T	0.2224	1.41E-18	chr:6:32648036
6	rs1794489	32641718	C	9.223	1.42E-18	chr:6:32648036
6	rs34826728	32610109	A	0.223	1.45E-18	chr:6:32648036

6	rs34843907	32610059	T	0.223	1.45E-18	chr:6:32648036
6	rs9272756	32610047	T	0.223	1.45E-18	chr:6:32648036
6	rs9274977	32642247	G	4.593	1.46E-18	chr:6:32648036
6	rs9273376	32626801	C	0.2229	1.48E-18	chr:6:32648036
6	rs184950714	32636728	A	10.66	1.56E-18	chr:6:32648036
6	rs114913214	32418321	A	11.66	1.58E-18	chr:6:32648036
6	rs115445135	32431116	C	11.66	1.58E-18	chr:6:32648036
6	rs115756433	32446937	A	11.66	1.58E-18	chr:6:32648036
6	rs181080594	32414647	G	11.66	1.58E-18	chr:6:32648036
6	rs6910553	32425778	G	11.66	1.58E-18	chr:6:32648036
6	rs17613340	32621966	G	4.541	1.63E-18	chr:6:32648036
6	rs9274106	32630176	A	3.674	1.64E-18	chr:6:32648036
6	rs9274501	32634032	G	4.252	1.75E-18	chr:6:32648036
6	rs3104364	32603968	G	4.148	1.77E-18	chr:6:32648036
6	rs558998942	32637290	G	12.37	1.80E-18	chr:6:32648036
6	rs9275084	32648722	G	4.55	1.81E-18	chr:6:32648036
6	rs2935907	32651136	A	10.54	1.84E-18	chr:6:32648036
6	rs199526678	32653823	G	9.947	1.85E-18	chr:6:32648036
6	rs114196722	32408151	A	11.31	1.90E-18	chr:6:32648036
6	rs114248983	32394443	T	11.31	1.90E-18	chr:6:32648036
6	rs114304312	32391470	G	11.31	1.90E-18	chr:6:32648036
6	rs114453949	32390377	T	11.31	1.90E-18	chr:6:32648036
6	rs114656303	32395244	G	11.31	1.90E-18	chr:6:32648036
6	rs114657744	32395626	G	11.31	1.90E-18	chr:6:32648036
6	rs114901373	32394802	T	11.31	1.90E-18	chr:6:32648036
6	rs115098399	32396787	A	11.31	1.90E-18	chr:6:32648036
6	rs115291739	32409286	T	11.31	1.90E-18	chr:6:32648036
6	rs115353337	32394827	A	11.31	1.90E-18	chr:6:32648036
6	rs115573784	32394279	C	11.31	1.90E-18	chr:6:32648036
6	rs115622881	32401908	A	11.31	1.90E-18	chr:6:32648036
6	rs115661515	32395428	T	11.31	1.90E-18	chr:6:32648036
6	rs115678364	32395201	C	11.31	1.90E-18	chr:6:32648036
6	rs115877957	32394547	G	11.31	1.90E-18	chr:6:32648036
6	rs115956975	32400230	G	11.31	1.90E-18	chr:6:32648036
6	rs116025413	32391800	A	11.31	1.90E-18	chr:6:32648036
6	rs116297629	32393915	G	11.31	1.90E-18	chr:6:32648036
6	rs116393604	32394448	A	11.31	1.90E-18	chr:6:32648036
6	rs116705089	32395914	G	11.31	1.90E-18	chr:6:32648036
6	rs116706331	32394622	C	11.31	1.90E-18	chr:6:32648036
6	rs116721964	32398322	T	11.31	1.90E-18	chr:6:32648036
6	rs140938009	32394121	A	11.31	1.90E-18	chr:6:32648036
6	rs141144670	32390484	T	11.31	1.90E-18	chr:6:32648036
6	rs144608450	32390924	T	11.31	1.90E-18	chr:6:32648036

6	rs145199103	32390559	G	11.31	1.90E-18	chr:6:32648036
6	rs148205919	32390464	G	11.31	1.90E-18	chr:6:32648036
6	rs16822567	32407260	A	11.31	1.90E-18	chr:6:32648036
6	rs16822586	32407773	C	11.31	1.90E-18	chr:6:32648036
6	rs16822587	32407855	C	11.31	1.90E-18	chr:6:32648036
6	rs16822602	32409697	A	11.31	1.90E-18	chr:6:32648036
6	rs16822614	32409953	G	11.31	1.90E-18	chr:6:32648036
6	rs3763324	32406901	G	11.31	1.90E-18	chr:6:32648036
6	rs6911791	32396288	T	11.31	1.90E-18	chr:6:32648036
6	rs6912072	32396265	A	11.31	1.90E-18	chr:6:32648036
6	rs6915350	32392720	C	11.31	1.90E-18	chr:6:32648036
6	rs6930615	32392205	A	11.31	1.90E-18	chr:6:32648036
6	rs6935319	32392570	T	11.31	1.90E-18	chr:6:32648036
6	rs6936170	32392821	A	11.31	1.90E-18	chr:6:32648036
6	rs7753500	32393354	G	11.31	1.90E-18	chr:6:32648036
6	rs7754263	32397096	G	11.31	1.90E-18	chr:6:32648036
6	rs7754635	32397028	A	11.31	1.90E-18	chr:6:32648036
6	rs7754753	32397401	T	11.31	1.90E-18	chr:6:32648036
6	rs9273050	32612035	G	4.579	1.97E-18	chr:6:32648036
6	rs9273014	32611650	A	3.954	1.99E-18	chr:6:32648036
6	rs28383235	32584184	C	8.24	2.03E-18	chr:6:32648036
6	rs114466578	32635501	G	9.085	2.04E-18	chr:6:32648036
6	rs114995226	32645799	A	5.315	2.14E-18	chr:6:32648036
6	rs13205658	32577607	T	7.972	2.14E-18	chr:6:32648036
6	rs7743412	32625884	T	13.49	2.20E-18	chr:6:32648036
6	rs281874783	32632660	G	4.682	2.29E-18	chr:6:32648036
6	rs9274390	32632659	T	4.682	2.29E-18	chr:6:32648036
6	rs28383320	32591368	C	7.921	2.36E-18	chr:6:32648036
6	rs116315927	32565977	T	11.41	2.42E-18	chr:6:32648036
6	rs9272498	32606217	A	4.285	2.45E-18	chr:6:32648036
6	rs28383399	32607872	G	8.047	2.53E-18	chr:6:32648036
6	rs9273352	32625928	T	3.875	2.62E-18	chr:6:32648036
6	rs9273523	32628571	T	10.31	2.66E-18	chr:6:32648036
6	rs28383438	32609038	T	7.877	2.76E-18	chr:6:32648036
6	rs16822610	32409844	A	10.84	2.83E-18	chr:6:32648036
6	rs9274857	32640542	G	3.864	2.83E-18	chr:6:32648036
6	rs9275348	32667638	A	5.746	2.85E-18	chr:6:32648036
6	rs28746860	32635033	G	8.703	2.87E-18	chr:6:32648036
6	rs9271394	32587480	T	5.14	2.99E-18	chr:6:32648036
6	rs9273356	32625989	G	4.15	3.12E-18	chr:6:32648036
6	rs1766	32627795	G	3.677	3.16E-18	chr:6:32648036
6	rs28383326	32598671	C	7.663	3.20E-18	chr:6:32648036
6	rs142420626	32630303	C	7.971	3.23E-18	chr:6:32648036

6	rs2647042	32667548	C	0.2158	3.32E-18	chr:6:32648036
6	rs9273584	32629064	T	2.989	3.40E-18	chr:6:32648036
6	rs9272368	32604642	G	3.497	3.43E-18	chr:6:32648036
6	rs9272370	32604644	A	3.497	3.43E-18	chr:6:32648036
6	rs9274999	32642709	A	4.368	3.49E-18	chr:6:32648036
6	rs28746798	32632558	C	5.759	3.49E-18	chr:6:32648036
6	rs28746859	32635007	A	8.162	3.54E-18	chr:6:32648036
6	rs116704735	32407206	T	11.12	3.61E-18	chr:6:32648036
6	rs3129730	32681839	G	7.811	3.75E-18	chr:6:32648036
6	rs28383347	32605689	A	7.53	3.76E-18	chr:6:32648036
6	rs28746863	32635172	T	9.826	3.78E-18	chr:6:32648036
6	rs3828811	32636221	G	4.102	3.85E-18	chr:6:32648036
6	rs9274967	32642030	C	3.573	3.88E-18	chr:6:32648036
6	rs9274949	32641855	G	2.979	3.90E-18	chr:6:32648036
6	rs13206219	32577847	T	7.658	3.96E-18	chr:6:32648036
6	rs28746809	32633159	C	7.209	4.20E-18	chr:6:32648036
6	rs180828183	32644430	T	5.694	4.40E-18	chr:6:32648036
6	rs185091810	32644431	A	5.694	4.40E-18	chr:6:32648036
6	rs28383410	32608289	A	4.773	4.51E-18	chr:6:32648036
6	rs3828798	32636038	A	0.2314	4.57E-18	chr:6:32648036
6	rs199617354	32644563	G	5.449	4.60E-18	chr:6:32648036
6	rs76384124	32653855	A	9.016	4.60E-18	chr:6:32648036
6	rs9274869	32640759	T	3.934	4.73E-18	chr:6:32648036
6	rs9274733	32637874	T	4.181	4.87E-18	chr:6:32648036
6	rs28383405	32608108	T	4.675	4.92E-18	chr:6:32648036
6	rs9275001	32642738	T	4.254	4.92E-18	chr:6:32648036
6	rs1694126	32640905	A	7.394	4.94E-18	chr:6:32648036
6	rs9274283	32631763	C	2.609	5.32E-18	chr:6:32648036
6	rs1130430	32629161	G	3.105	5.70E-18	chr:6:32648036
6	rs9274951	32641867	G	2.921	6.03E-18	chr:6:32648036
6	rs9274952	32641868	G	2.921	6.03E-18	chr:6:32648036
6	rs9272733	32609652	A	4.444	6.15E-18	chr:6:32648036
6	rs9272734	32609654	A	4.444	6.15E-18	chr:6:32648036
6	rs9272735	32609655	G	4.444	6.15E-18	chr:6:32648036
6	rs9271398	32587508	C	5.099	6.20E-18	chr:6:32648036
6	rs28755181	32642013	A	10.38	6.34E-18	chr:6:32648036
6	rs9272482	32605920	G	0.2381	6.44E-18	chr:6:32648036
6	rs28746819	32633320	C	7.89	6.51E-18	chr:6:32648036
6	rs9274867	32640745	A	3.929	6.53E-18	chr:6:32648036
6	rs28383317	32590281	A	7.851	6.65E-18	chr:6:32648036
6	rs9271416	32587812	A	5.056	6.68E-18	chr:6:32648036
6	rs9272411	32604980	C	3.535	6.76E-18	chr:6:32648036
6	rs3828790	32635809	T	0.232	6.78E-18	chr:6:32648036

6	rs3828791	32635813	A	0.232	6.78E-18	chr:6:32648036
6	rs28383411	32608308	C	4.734	6.81E-18	chr:6:32648036
6	rs9274257	32631415	C	9.743	6.91E-18	chr:6:32648036
6	rs28383357	32606732	A	3.152	7.04E-18	chr:6:32648036
6	rs28383390	32607657	C	8.893	7.06E-18	chr:6:32648036
6	rs28383466	32610277	G	7.615	7.15E-18	chr:6:32648036
6	rs9273013	32611649	T	3.468	7.37E-18	chr:6:32648036
6	rs28746810	32633185	T	7.208	7.54E-18	chr:6:32648036
6	rs9273021	32611725	G	2.969	7.66E-18	chr:6:32648036
6	rs28746808	32633130	A	7.195	7.70E-18	chr:6:32648036
6	rs9272586	32607492	C	3.195	7.72E-18	chr:6:32648036
6	rs28724246	32629422	C	8.432	7.89E-18	chr:6:32648036
6	rs9272994	32611450	T	2.744	8.20E-18	chr:6:32648036
6	rs9271408	32587613	T	4.986	8.28E-18	chr:6:32648036
6	rs28746815	32633253	G	7.436	8.36E-18	chr:6:32648036
6	rs28746816	32633261	A	7.436	8.36E-18	chr:6:32648036
6	rs9272492	32606052	G	4.021	8.46E-18	chr:6:32648036
6	rs9275073	32648513	G	4.406	8.49E-18	chr:6:32648036
6	rs9274745	32638058	T	4.168	8.98E-18	chr:6:32648036
6	rs78074767	32611472	G	2.681	9.02E-18	chr:6:32648036
6	rs9273020	32611723	T	2.964	9.04E-18	chr:6:32648036
6	rs9271411	32587661	A	4.988	9.04E-18	chr:6:32648036
6	rs2395191	32447085	C	4.482	9.30E-18	chr:6:32648036
6	rs9378268	32450915	C	4.482	9.30E-18	chr:6:32648036
6	rs549783155	32448180	C	11.1	9.37E-18	chr:6:32648036
6	rs9274922	32641350	G	2.632	9.53E-18	chr:6:32648036
6	rs281863638	32630750	A	10.18	9.85E-18	chr:6:32648036
6	rs9272442	32605400	C	0.2419	9.93E-18	chr:6:32648036
6	rs17843626	32621013	A	2.912	9.96E-18	chr:6:32648036
6	rs115211911	32389614	T	10.8	1.00E-17	chr:6:32648036
6	rs116227556	32389618	G	10.8	1.00E-17	chr:6:32648036
6	rs146771984	32390142	A	10.8	1.00E-17	chr:6:32648036
6	rs150022449	32389908	A	10.8	1.00E-17	chr:6:32648036
6	rs28383419	32608622	A	4.424	1.00E-17	chr:6:32648036
6	rs9501620	32383027	A	10.8	1.00E-17	chr:6:32648036
6	rs9272393	32604826	A	2.99	1.01E-17	chr:6:32648036
6	rs9296026	32368075	A	11.31	1.03E-17	chr:6:32648036
6	rs9469107	32366226	A	11.31	1.03E-17	chr:6:32648036
6	rs28383316	32589687	C	7.723	1.03E-17	chr:6:32648036
6	rs9275082	32648684	C	4.36	1.09E-17	chr:6:32648036
6	rs9272538	32606809	C	2.812	1.09E-17	chr:6:32648036
6	rs1071630	32609126	T	0.2422	1.10E-17	chr:6:32648036
6	rs9272416	32605043	T	3.814	1.10E-17	chr:6:32648036

6	rs28383401	32607973	A	4.342	1.12E-17	chr:6:32648036
6	rs28383463	32610105	G	7.415	1.16E-17	chr:6:32648036
6	rs9272556	32607046	T	3.068	1.17E-17	chr:6:32648036
6	rs116712006	32389671	C	10.03	1.17E-17	chr:6:32648036
6	rs9275071	32648490	A	4.348	1.20E-17	chr:6:32648036
6	rs34100078	32653792	G	7.123	1.20E-17	chr:6:32648036
6	rs114267247	32652593	T	9.257	1.20E-17	chr:6:32648036
6	rs9271409	32587628	C	4.92	1.25E-17	chr:6:32648036
6	rs9274954	32641878	T	2.977	1.26E-17	chr:6:32648036
6	rs115218431	32611504	C	2.712	1.27E-17	chr:6:32648036
6	rs6929590	32391851	G	9.409	1.28E-17	chr:6:32648036
6	rs2002778	32601647	C	7.102	1.32E-17	chr:6:32648036
6	rs9271540	32590029	C	4.862	1.33E-17	chr:6:32648036
6	rs28383408	32608251	G	4.599	1.37E-17	chr:6:32648036
6	rs551235432	32352762	A	11.18	1.38E-17	chr:6:32648036
6	rs6927077	32375909	A	11.18	1.38E-17	chr:6:32648036
6	rs6940690	32341367	T	11.18	1.38E-17	chr:6:32648036
6	rs7759813	32354073	G	11.18	1.38E-17	chr:6:32648036
6	rs7771136	32356409	C	11.18	1.38E-17	chr:6:32648036
6	rs9501619	32346075	A	11.18	1.38E-17	chr:6:32648036
6	rs727939	32623463	G	3.114	1.42E-17	chr:6:32648036
6	rs9271486	32588980	T	4.928	1.50E-17	chr:6:32648036
6	rs9273024	32611757	C	2.846	1.51E-17	chr:6:32648036
6	rs9271582	32590774	C	4.909	1.53E-17	chr:6:32648036
6	rs9271583	32590780	C	4.909	1.53E-17	chr:6:32648036
6	rs28383402	32607985	G	4.309	1.55E-17	chr:6:32648036
6	rs9271504	32589368	A	4.925	1.56E-17	chr:6:32648036
6	rs28383462	32609619	T	6.041	1.63E-17	chr:6:32648036
6	rs2858301	32643201	T	5.585	1.65E-17	chr:6:32648036
6	rs9272510	32606367	G	3.511	1.68E-17	chr:6:32648036
6	rs9275078	32648592	C	4.324	1.68E-17	chr:6:32648036
6	rs9271400	32587520	T	4.936	1.76E-17	chr:6:32648036
6	rs28746797	32632544	A	5.375	1.77E-17	chr:6:32648036
6	rs9272474	32605754	A	3.284	1.77E-17	chr:6:32648036
6	rs1129740	32609105	G	0.2515	1.91E-17	chr:6:32648036
6	rs28383415	32608473	A	4.712	1.92E-17	chr:6:32648036
6	rs28724244	32629398	C	8.789	1.96E-17	chr:6:32648036
6	rs28383324	32594211	T	7.314	1.97E-17	chr:6:32648036
6	rs9271511	32589483	A	4.876	1.98E-17	chr:6:32648036
6	rs9271584	32590870	C	4.876	1.98E-17	chr:6:32648036
6	rs9271585	32590879	A	4.876	1.98E-17	chr:6:32648036
6	rs9271589	32591128	A	4.876	1.98E-17	chr:6:32648036
6	rs9271592	32591173	T	4.876	1.98E-17	chr:6:32648036

6	rs9271607	32591566	G	4.876	1.98E-17	chr:6:32648036
6	rs9271609	32591597	C	4.876	1.98E-17	chr:6:32648036
6	rs9273488	32628159	G	9.505	2.03E-17	chr:6:32648036
6	rs28383417	32608507	G	8.782	2.06E-17	chr:6:32648036
6	rs9273052	32612041	G	4.741	2.10E-17	chr:6:32648036
6	rs143374644	32521059	A	11.11	2.13E-17	chr:6:32648036
6	rs9272366	32604622	A	3.484	2.19E-17	chr:6:32648036
6	rs9272584	32607470	T	3.229	2.23E-17	chr:6:32648036
6	rs28746792	32632503	T	2.55	2.25E-17	chr:6:32648036
6	rs9272476	32605759	A	3.28	2.25E-17	chr:6:32648036
6	rs3134993	32637778	G	0.2484	2.31E-17	chr:6:32648036
6	rs9272473	32605738	A	3.368	2.31E-17	chr:6:32648036
6	rs281863414	32631058	G	7.36	2.32E-17	chr:6:32648036
6	rs28383333	32601786	A	7.718	2.33E-17	chr:6:32648036
6	rs116685323	32611510	C	2.679	2.35E-17	chr:6:32648036
6	rs149654721	32611509	C	2.679	2.35E-17	chr:6:32648036
6	rs28383404	32608090	T	7.39	2.37E-17	chr:6:32648036
6	rs9274895	32641184	G	2.602	2.38E-17	chr:6:32648036
6	rs9274896	32641185	G	2.602	2.38E-17	chr:6:32648036
6	rs9272583	32607453	T	3.416	2.39E-17	chr:6:32648036
6	rs28746823	32633373	T	7.331	2.39E-17	chr:6:32648036
6	rs9274934	32641529	T	2.695	2.44E-17	chr:6:32648036
6	rs3828789	32635754	G	0.243	2.48E-17	chr:6:32648036
6	rs9273033	32611873	C	2.981	2.53E-17	chr:6:32648036
6	rs9273034	32611874	T	2.981	2.53E-17	chr:6:32648036
6	rs28383413	32608417	A	4.603	2.54E-17	chr:6:32648036
6	rs9273535	32628760	A	7.993	2.56E-17	chr:6:32648036
6	rs9272484	32605943	G	0.2512	2.58E-17	chr:6:32648036
6	rs9271560	32590381	T	4.866	2.60E-17	chr:6:32648036
6	rs9272555	32607042	A	3.018	2.76E-17	chr:6:32648036
6	rs28383397	32607820	T	4.5	2.79E-17	chr:6:32648036
6	rs28383398	32607828	T	4.5	2.79E-17	chr:6:32648036
6	rs281863770	32630557	A	3.687	2.82E-17	chr:6:32648036
6	rs281863778	32630543	T	3.687	2.82E-17	chr:6:32648036
6	rs115081558	32681272	G	10.88	2.92E-17	chr:6:32648036
6	rs9272503	32606286	C	3.617	2.97E-17	chr:6:32648036
6	rs9273350	32625885	G	3.622	3.01E-17	chr:6:32648036
6	rs9271604	32591430	G	4.819	3.08E-17	chr:6:32648036
6	rs9271605	32591448	A	4.819	3.08E-17	chr:6:32648036
6	rs9273022	32611737	C	3.918	3.12E-17	chr:6:32648036
6	rs9274828	32639737	G	3.312	3.12E-17	chr:6:32648036
6	rs9271561	32590393	G	4.807	3.18E-17	chr:6:32648036
6	rs28383331	32600772	A	6.511	3.33E-17	chr:6:32648036

6	rs9272491	32606046	C	3.452	3.34E-17	chr:6:32648036
6	rs9274289	32631817	C	4.029	3.47E-17	chr:6:32648036
6	rs9273351	32625906	C	3.617	3.62E-17	chr:6:32648036
6	rs28746796	32632536	T	5.227	3.62E-17	chr:6:32648036
6	rs9273443	32627600	G	0.2551	3.68E-17	chr:6:32648036
6	rs9274898	32641199	T	2.584	3.80E-17	chr:6:32648036
6	rs9274899	32641203	T	2.584	3.80E-17	chr:6:32648036
6	rs28746793	32632528	T	4.664	3.83E-17	chr:6:32648036
6	rs9274900	32641207	A	2.585	3.86E-17	chr:6:32648036
6	rs2395520	32601462	G	6.707	3.86E-17	chr:6:32648036
6	rs28383414	32608467	A	4.614	3.98E-17	chr:6:32648036
6	rs9273541	32628787	T	7.695	4.08E-17	chr:6:32648036
6	rs9271554	32590317	G	4.785	4.18E-17	chr:6:32648036
6	rs9272347	32604394	C	3.557	4.25E-17	chr:6:32648036
6	rs28746805	32633050	G	7.005	4.27E-17	chr:6:32648036
6	rs1130381	32632691	A	8.007	4.41E-17	chr:6:32648036
6	rs9271488	32589000	T	4.753	4.44E-17	chr:6:32648036
6	rs9272793	32610495	G	3.861	4.46E-17	chr:6:32648036
6	rs281863735	32630619	A	3.486	4.48E-17	chr:6:32648036
6	rs9271497	32589245	T	4.808	4.59E-17	chr:6:32648036
6	rs9271485	32588953	G	4.745	4.66E-17	chr:6:32648036
6	rs116567952	32601006	C	9.544	4.69E-17	chr:6:32648036
6	rs28383349	32606266	C	6.439	4.73E-17	chr:6:32648036
6	rs9273800	32629496	T	3.8	4.82E-17	chr:6:32648036
6	rs9274883	32641072	T	2.802	4.90E-17	chr:6:32648036
6	rs28746807	32633085	G	6.795	4.99E-17	chr:6:32648036
6	rs9273049	32612034	T	2.704	5.04E-17	chr:6:32648036
6	rs9274871	32640792	G	3.472	5.07E-17	chr:6:32648036
6	rs9274872	32640793	G	3.472	5.07E-17	chr:6:32648036
6	rs9272389	32604793	C	2.87	5.19E-17	chr:6:32648036
6	rs9275081	32648669	T	4.117	5.25E-17	chr:6:32648036
6	rs7751376	32605437	G	3.752	5.32E-17	chr:6:32648036
6	rs9271595	32591235	G	4.639	5.33E-17	chr:6:32648036
6	rs9271596	32591237	G	4.639	5.33E-17	chr:6:32648036
6	rs9271426	32587973	A	4.758	5.36E-17	chr:6:32648036
6	rs1140342	32629141	T	3.097	5.49E-17	chr:6:32648036
6	rs9274607	32635713	T	0.2497	5.58E-17	chr:6:32648036
6	rs114213887	32681343	C	9.689	5.77E-17	chr:6:32648036
6	rs563774374	32506022	T	2.696	5.90E-17	chr:6:32648036
6	rs9274352	32632433	G	5.289	6.04E-17	chr:6:32648036
6	rs9272568	32607209	G	2.685	6.07E-17	chr:6:32648036
6	rs9272847	32610695	T	3.476	6.24E-17	chr:6:32648036
6	rs9273046	32612003	T	2.8	6.29E-17	chr:6:32648036

6	rs9274351	32632425	A	5.191	6.56E-17	chr:6:32648036
6	rs9272387	32604776	C	2.838	6.59E-17	chr:6:32648036
6	rs9274278	32631711	T	2.679	6.78E-17	chr:6:32648036
6	rs114279634	32392481	G	8.234	6.85E-17	chr:6:32648036
6	rs17612640	32615754	A	3.664	7.08E-17	chr:6:32648036
6	rs1064173	32627480	A	5.112	7.09E-17	chr:6:32648036
6	rs28383416	32608483	C	4.559	7.11E-17	chr:6:32648036
6	rs116080026	32637492	T	11.39	7.13E-17	chr:6:32648036
6	rs9271430	32588002	C	4.726	7.25E-17	chr:6:32648036
6	rs9272435	32605336	A	4.532	7.29E-17	chr:6:32648036
6	rs17612802	32620359	T	2.83	7.29E-17	chr:6:32648036
6	rs1130382	32632672	A	8.075	7.39E-17	chr:6:32648036
6	rs12208101	32576812	A	7.549	7.73E-17	chr:6:32648036
6	rs77032877	32501811	C	5.081	7.84E-17	chr:6:32648036
6	rs28383418	32608537	C	4.504	7.85E-17	chr:6:32648036
6	rs281861609	32633350	A	7.254	7.86E-17	chr:6:32648036
6	rs28746821	32633348	G	7.254	7.86E-17	chr:6:32648036
6	rs114001299	32394511	A	8.147	8.12E-17	chr:6:32648036
6	rs114188689	32391589	A	8.147	8.12E-17	chr:6:32648036
6	rs114404972	32393686	A	8.147	8.12E-17	chr:6:32648036
6	rs114594015	32394690	C	8.147	8.12E-17	chr:6:32648036
6	rs114637082	32392282	A	8.147	8.12E-17	chr:6:32648036
6	rs114913290	32390676	T	8.147	8.12E-17	chr:6:32648036
6	rs115045614	32394615	C	8.147	8.12E-17	chr:6:32648036
6	rs115472206	32392433	T	8.147	8.12E-17	chr:6:32648036
6	rs115617611	32391301	G	8.147	8.12E-17	chr:6:32648036
6	rs115861456	32391503	T	8.147	8.12E-17	chr:6:32648036
6	rs116103816	32395236	A	8.147	8.12E-17	chr:6:32648036
6	rs116276080	32391514	C	8.147	8.12E-17	chr:6:32648036
6	rs116414794	32391485	C	8.147	8.12E-17	chr:6:32648036
6	rs116514685	32393768	T	8.147	8.12E-17	chr:6:32648036
6	rs116610730	32394370	G	8.147	8.12E-17	chr:6:32648036
6	rs148258492	32394177	C	8.147	8.12E-17	chr:6:32648036
6	rs148519138	32390934	C	8.147	8.12E-17	chr:6:32648036
6	rs186973048	32391050	C	8.147	8.12E-17	chr:6:32648036
6	rs191868117	32391084	C	8.147	8.12E-17	chr:6:32648036
6	rs6457579	32393093	G	8.147	8.12E-17	chr:6:32648036
6	rs6457580	32393141	T	8.147	8.12E-17	chr:6:32648036
6	rs6929953	32391988	T	8.147	8.12E-17	chr:6:32648036
6	rs6930108	32392054	T	8.147	8.12E-17	chr:6:32648036
6	rs6936008	32392764	C	8.147	8.12E-17	chr:6:32648036
6	rs7771308	32393259	T	8.147	8.12E-17	chr:6:32648036
6	rs9272514	32606385	T	3.41	8.39E-17	chr:6:32648036

6	rs9271624	32591921	T	4.619	8.47E-17	chr:6:32648036
6	rs28383308	32586782	T	6.221	8.49E-17	chr:6:32648036
6	rs28383309	32586783	T	6.221	8.49E-17	chr:6:32648036
6	rs6938008	32604743	C	2.818	8.51E-17	chr:6:32648036
6	rs28383338	32603897	G	6.366	8.57E-17	chr:6:32648036
6	rs9271574	32590590	G	4.638	8.77E-17	chr:6:32648036
6	rs9271576	32590612	C	4.638	8.77E-17	chr:6:32648036
6	rs570194309	32590602	C	4.66	8.79E-17	chr:6:32648036
6	rs9271549	32590248	C	4.684	8.80E-17	chr:6:32648036
6	rs28371245	32647982	C	2.853	8.92E-17	chr:6:32648036
6	rs3104363	32604000	T	3.745	9.02E-17	chr:6:32648036
6	rs9272525	32606559	A	2.665	9.08E-17	chr:6:32648036
6	rs28383336	32603729	G	6.082	9.28E-17	chr:6:32648036
6	rs9272420	32605101	G	4.518	9.35E-17	chr:6:32648036
6	rs6900039	32623235	T	11.24	9.60E-17	chr:6:32648036
6	rs9272443	32605418	A	4.187	9.76E-17	chr:6:32648036
6	rs9272444	32605419	C	4.187	9.76E-17	chr:6:32648036
6	rs28383391	32607659	T	4.521	1.00E-16	chr:6:32648036
6	rs28383392	32607660	G	4.521	1.00E-16	chr:6:32648036
6	rs28383403	32608035	C	4.21	1.01E-16	chr:6:32648036
6	rs28383440	32609046	T	7.147	1.06E-16	chr:6:32648036
6	rs9274662	32636494	T	0.2378	1.06E-16	chr:6:32648036
6	rs377552899	32630344	C	6.295	1.06E-16	chr:6:32648036
6	rs535051941	32635364	C	3.123	1.06E-16	chr:6:32648036
6	rs28746790	32632459	C	5.008	1.07E-16	chr:6:32648036
6	rs9274926	32641416	C	2.627	1.16E-16	chr:6:32648036
6	rs9274927	32641419	C	2.627	1.16E-16	chr:6:32648036
6	rs1794500	32647442	C	4.914	1.17E-16	chr:6:32648036
6	rs28383457	32609456	T	4.535	1.17E-16	chr:6:32648036
6	rs9271563	32590428	C	4.601	1.24E-16	chr:6:32648036
6	rs9271628	32591971	G	4.524	1.24E-16	chr:6:32648036
6	rs9273324	32623156	T	2.614	1.26E-16	chr:6:32648036
6	rs9274930	32641505	C	2.625	1.28E-16	chr:6:32648036
6	rs9274931	32641506	A	2.625	1.28E-16	chr:6:32648036
6	rs9274932	32641509	G	2.625	1.28E-16	chr:6:32648036
6	rs9274933	32641513	C	2.625	1.28E-16	chr:6:32648036
6	rs9271544	32590120	A	4.61	1.29E-16	chr:6:32648036
6	rs1130391	32632624	T	8.129	1.30E-16	chr:6:32648036
6	rs9272323	32604151	T	3.738	1.30E-16	chr:6:32648036
6	rs28746802	32632980	T	5.623	1.31E-16	chr:6:32648036
6	rs28746803	32632983	T	5.623	1.31E-16	chr:6:32648036
6	rs3188543	32610403	A	4.453	1.34E-16	chr:6:32648036
6	rs9272488	32606005	G	3.319	1.38E-16	chr:6:32648036

6	rs9273048	32612008	C	4.308	1.38E-16	chr:6:32648036
6	rs9274886	32641086	C	2.738	1.39E-16	chr:6:32648036
6	rs9274887	32641089	G	2.738	1.39E-16	chr:6:32648036
6	rs9272379	32604727	C	2.715	1.43E-16	chr:6:32648036
6	rs28383386	32607567	A	3.159	1.44E-16	chr:6:32648036
6	rs9272661	32608680	T	3.106	1.45E-16	chr:6:32648036
6	rs9272350	32604410	A	3.024	1.46E-16	chr:6:32648036
6	rs9272563	32607158	C	2.64	1.49E-16	chr:6:32648036
6	rs9272472	32605724	G	3.944	1.49E-16	chr:6:32648036
6	rs9273329	32623242	A	3.656	1.51E-16	chr:6:32648036
6	rs537750078	32661051	A	5.502	1.51E-16	chr:6:32648036
6	rs17612625	32615702	C	3.581	1.54E-16	chr:6:32648036
6	rs17612633	32615710	C	3.581	1.54E-16	chr:6:32648036
6	rs28752536	32598140	T	6.276	1.55E-16	chr:6:32648036
6	rs9271472	32588700	T	4.591	1.59E-16	chr:6:32648036
6	rs9271474	32588707	A	4.591	1.59E-16	chr:6:32648036
6	rs9274357	32632457	G	4.836	1.63E-16	chr:6:32648036
6	rs9272565	32607169	G	2.639	1.65E-16	chr:6:32648036
6	rs9271674	32592845	C	4.39	1.66E-16	chr:6:32648036
6	rs9271735	32593649	A	3.642	1.67E-16	chr:6:32648036
6	rs28383365	32606942	A	3.295	1.69E-16	chr:6:32648036
6	rs116312062	32527045	G	7.472	1.72E-16	chr:6:32648036
6	rs9274955	32641890	G	2.861	1.74E-16	chr:6:32648036
6	rs9271516	32589668	A	4.562	1.76E-16	chr:6:32648036
6	rs28383304	32586720	T	6.108	1.77E-16	chr:6:32648036
6	rs9272647	32608508	T	3.501	1.93E-16	chr:6:32648036
6	rs9272567	32607200	C	2.639	1.94E-16	chr:6:32648036
6	rs28383381	32607288	A	3.458	1.95E-16	chr:6:32648036
6	rs535097245	32218625	A	9.62	1.95E-16	chr:6:32648036
6	rs28383389	32607596	A	4.384	1.96E-16	chr:6:32648036
6	rs9272637	32608213	A	3.204	1.96E-16	chr:6:32648036
6	rs9272318	32604059	G	4.128	2.01E-16	chr:6:32648036
6	rs116808893	32652418	C	9.345	2.02E-16	chr:6:32648036
6	rs1049057	32634350	C	3.507	2.04E-16	chr:6:32648036
6	rs372253974	32630329	C	6.123	2.06E-16	chr:6:32648036
6	rs28383383	32607368	C	4.6	2.09E-16	chr:6:32648036
6	rs1063348	32627923	G	3.651	2.10E-16	chr:6:32648036
6	rs9272731	32609645	C	4.199	2.14E-16	chr:6:32648036
6	rs9272732	32609647	G	4.199	2.14E-16	chr:6:32648036
6	rs9275062	32648337	C	3.794	2.18E-16	chr:6:32648036
6	rs9271769	32594217	T	4.453	2.22E-16	chr:6:32648036
6	rs3205916	32609106	C	4.526	2.22E-16	chr:6:32648036
6	rs28383350	32606320	A	6.262	2.23E-16	chr:6:32648036

6	rs3187964	32605207	G	3.601	2.24E-16	chr:6:32648036
6	rs9272463	32605646	G	4.236	2.27E-16	chr:6:32648036
6	rs9273441	32627573	G	3.572	2.27E-16	chr:6:32648036
6	rs9271438	32588193	G	4.553	2.28E-16	chr:6:32648036
6	rs3134998	32636757	T	0.2375	2.30E-16	chr:6:32648036
6	rs9273326	32623219	C	3.576	2.38E-16	chr:6:32648036
6	rs9271532	32589945	C	4.516	2.45E-16	chr:6:32648036
6	rs9273328	32623233	A	3.575	2.46E-16	chr:6:32648036
6	rs28746804	32632989	G	5.482	2.47E-16	chr:6:32648036
6	rs7760514	32746309	A	8.825	2.50E-16	chr:6:32648036
6	rs9271643	32592229	A	4.402	2.57E-16	chr:6:32648036
6	rs9274470	32633617	A	3.059	2.60E-16	chr:6:32648036
6	rs9272346	32604372	A	3.354	2.65E-16	chr:6:32648036
6	rs9271637	32592124	G	4.424	2.75E-16	chr:6:32648036
6	rs281863860	32630427	C	5.752	2.80E-16	chr:6:32648036
6	rs76282264	32628635	T	10.25	2.88E-16	chr:6:32648036
6	rs2856713	32620422	T	2.721	2.88E-16	chr:6:32648036
6	rs28383358	32606779	A	4.676	2.90E-16	chr:6:32648036
6	rs9275070	32648449	T	3.874	2.91E-16	chr:6:32648036
6	rs9271736	32593688	A	3.298	2.93E-16	chr:6:32648036
6	rs9272357	32604522	A	2.837	2.95E-16	chr:6:32648036
6	rs28366275	32560114	A	3.685	2.97E-16	chr:6:32648036
6	rs28383328	32599554	T	5.487	2.97E-16	chr:6:32648036
6	rs9272730	32609634	G	4.151	3.10E-16	chr:6:32648036
6	rs9272378	32604718	G	3.022	3.12E-16	chr:6:32648036
6	rs9271602	32591383	G	4.449	3.19E-16	chr:6:32648036
6	rs28383340	32604431	T	5.672	3.25E-16	chr:6:32648036
6	rs9271693	32593033	T	4.282	3.31E-16	chr:6:32648036
6	rs28383460	32609586	G	4.404	3.39E-16	chr:6:32648036
6	rs9273493	32628247	T	0.2725	3.46E-16	chr:6:32648036
6	rs17205647	32637418	A	3.446	3.48E-16	chr:6:32648036
6	rs9274669	32636591	A	0.2694	3.49E-16	chr:6:32648036
6	rs2856675	32658768	A	5.627	3.56E-16	chr:6:32648036
6	rs9271675	32592862	C	4.286	3.59E-16	chr:6:32648036
6	rs28383342	32604924	T	6.015	3.60E-16	chr:6:32648036
6	rs28383337	32603732	G	7.665	3.62E-16	chr:6:32648036
6	rs10947319	32581454	T	5.728	3.64E-16	chr:6:32648036
6	rs9274929	32641487	C	2.571	3.69E-16	chr:6:32648036
6	rs281861394	32633608	G	11.86	3.71E-16	chr:6:32648036
6	rs9272355	32604515	C	2.829	3.92E-16	chr:6:32648036
6	rs9272599	32607638	G	2.836	3.93E-16	chr:6:32648036
6	rs28383301	32586631	T	6.081	4.03E-16	chr:6:32648036
6	rs28383302	32586639	G	6.081	4.03E-16	chr:6:32648036

6	rs28383303	32586648	G	6.081	4.03E-16	chr:6:32648036
6	rs9273002	32611513	G	3.675	4.06E-16	chr:6:32648036
6	rs116105128	32511456	C	6.123	4.15E-16	chr:6:32648036
6	rs9273007	32611537	T	3.772	4.17E-16	chr:6:32648036
6	rs115487398	32390206	C	7.782	4.21E-16	chr:6:32648036
6	rs9273045	32611982	C	4.15	4.23E-16	chr:6:32648036
6	rs9272485	32605972	G	3.941	4.23E-16	chr:6:32648036
6	rs9271564	32590436	T	4.438	4.25E-16	chr:6:32648036
6	rs9271565	32590437	G	4.438	4.25E-16	chr:6:32648036
6	rs6932265	32579441	T	5.764	4.39E-16	chr:6:32648036
6	rs9272540	32606834	C	2.579	4.48E-16	chr:6:32648036
6	rs9272656	32608629	G	2.956	4.50E-16	chr:6:32648036
6	rs9272376	32604697	T	2.638	4.55E-16	chr:6:32648036
6	rs9272377	32604699	T	2.638	4.55E-16	chr:6:32648036
6	rs2133035	32596475	A	4.184	4.72E-16	chr:6:32648036
6	rs28400568	32610557	A	7.377	4.75E-16	chr:6:32648036
6	rs28400569	32610567	T	7.377	4.75E-16	chr:6:32648036
6	rs17613542	32630717	A	8.844	4.79E-16	chr:6:32648036
6	rs9272025	32598581	A	4.097	4.86E-16	chr:6:32648036
6	rs9272744	32609746	T	3.873	4.95E-16	chr:6:32648036
6	rs28383343	32604974	T	6.049	4.96E-16	chr:6:32648036
6	rs28383407	32608187	T	4.07	5.00E-16	chr:6:32648036
6	rs35979303	32450721	C	3.719	5.00E-16	chr:6:32648036
6	rs28371240	32647856	A	5.167	5.13E-16	chr:6:32648036
6	rs1048414	32610478	G	4.342	5.17E-16	chr:6:32648036
6	rs1048419	32610481	C	4.342	5.17E-16	chr:6:32648036
6	rs9273036	32611898	G	4.044	5.22E-16	chr:6:32648036
6	rs9272581	32607411	A	3.221	5.22E-16	chr:6:32648036
6	rs12212951	32581233	C	5.628	5.43E-16	chr:6:32648036
6	rs3134996	32636866	A	0.2449	5.57E-16	chr:6:32648036
6	rs9273044	32611971	C	4.111	5.60E-16	chr:6:32648036
6	rs28383382	32607320	C	3.795	5.87E-16	chr:6:32648036
6	rs17843573	32615400	T	3.227	5.88E-16	chr:6:32648036
6	rs9272601	32607652	T	2.802	5.88E-16	chr:6:32648036
6	rs1129735	32605295	T	4.28	5.88E-16	chr:6:32648036
6	rs75076975	32610134	A	4.199	6.03E-16	chr:6:32648036
6	rs17205640	32637413	A	3.11	6.04E-16	chr:6:32648036
6	rs17843578	32615520	A	2.978	6.09E-16	chr:6:32648036
6	rs9274565	32635067	A	2.8	6.15E-16	chr:6:32648036
6	rs28383406	32608179	C	4.34	6.15E-16	chr:6:32648036
6	rs17843625	32620909	G	2.834	6.19E-16	chr:6:32648036
6	rs9272642	32608303	C	2.766	6.20E-16	chr:6:32648036
6	rs9272643	32608304	A	2.766	6.20E-16	chr:6:32648036

6	rs17612858	32620622	T	2.886	6.21E-16	chr:6:32648036
6	rs568480451	32609789	G	7.604	6.25E-16	chr:6:32648036
6	rs28383395	32607713	T	4.223	6.25E-16	chr:6:32648036
6	rs28383396	32607714	G	4.223	6.25E-16	chr:6:32648036
6	rs28383426	32608785	C	8.406	6.35E-16	chr:6:32648036
6	rs28383313	32587117	G	4.292	6.41E-16	chr:6:32648036
6	rs9271542	32590091	G	4.394	6.58E-16	chr:6:32648036
6	rs9272513	32606380	G	2.766	6.59E-16	chr:6:32648036
6	rs17843577	32615510	T	2.976	6.60E-16	chr:6:32648036
6	rs9272546	32606941	C	2.897	6.71E-16	chr:6:32648036
6	rs28383393	32607698	G	7.455	6.72E-16	chr:6:32648036
6	rs28383394	32607700	T	7.455	6.72E-16	chr:6:32648036
6	rs13204672	32582796	G	5.648	6.93E-16	chr:6:32648036
6	rs13212015	32580580	C	5.648	6.93E-16	chr:6:32648036
6	rs28383216	32580596	C	5.648	6.93E-16	chr:6:32648036
6	rs28383222	32583405	A	5.648	6.93E-16	chr:6:32648036
6	rs28383225	32583824	C	5.648	6.93E-16	chr:6:32648036
6	rs28383227	32583982	A	5.648	6.93E-16	chr:6:32648036
6	rs28383228	32583993	A	5.648	6.93E-16	chr:6:32648036
6	rs28383232	32584108	T	5.648	6.93E-16	chr:6:32648036
6	rs28752517	32584583	A	5.648	6.93E-16	chr:6:32648036
6	rs28752518	32584588	G	5.648	6.93E-16	chr:6:32648036
6	rs28752521	32584767	T	5.648	6.93E-16	chr:6:32648036
6	rs570600579	32584768	A	5.648	6.93E-16	chr:6:32648036
6	rs9273524	32628595	T	0.2763	7.07E-16	chr:6:32648036
6	rs9461744	32388317	G	7.496	7.08E-16	chr:6:32648036
6	rs9272335	32604259	T	3.223	7.15E-16	chr:6:32648036
6	rs9271621	32591841	A	4.384	7.18E-16	chr:6:32648036
6	rs9272774	32610255	A	4.186	7.32E-16	chr:6:32648036
6	rs9272507	32606324	C	2.676	7.33E-16	chr:6:32648036
6	rs9272776	32610270	T	0.2713	7.38E-16	chr:6:32648036
6	rs9272871	32610747	C	4.142	7.43E-16	chr:6:32648036
6	rs9271764	32594159	A	4.318	7.44E-16	chr:6:32648036
6	rs9271765	32594166	G	4.318	7.44E-16	chr:6:32648036
6	rs9271773	32594304	C	3.479	7.48E-16	chr:6:32648036
6	rs9272602	32607666	A	2.795	7.55E-16	chr:6:32648036
6	rs28752524	32584862	A	5.703	7.74E-16	chr:6:32648036
6	rs28752525	32584868	C	5.703	7.74E-16	chr:6:32648036
6	rs281861581	32633380	A	2.637	8.21E-16	chr:6:32648036
6	rs28383329	32599645	T	4.536	8.40E-16	chr:6:32648036
6	rs28383439	32609042	A	4.27	8.41E-16	chr:6:32648036
6	rs28383369	32607087	T	5.89	8.47E-16	chr:6:32648036
6	rs9272976	32611341	A	2.584	8.73E-16	chr:6:32648036

6	rs9272865	32610736	A	4.1	9.05E-16	chr:6:32648036
6	rs9271676	32592868	C	4.172	9.06E-16	chr:6:32648036
6	rs9273042	32611964	T	4.015	9.08E-16	chr:6:32648036
6	rs9273040	32611958	G	3.994	9.15E-16	chr:6:32648036
6	rs9274459	32633409	A	2.786	9.38E-16	chr:6:32648036
6	rs9273043	32611968	T	2.786	9.42E-16	chr:6:32648036
6	rs17612555	32615366	A	3.235	9.48E-16	chr:6:32648036
6	rs115793615	32206725	T	8.425	9.55E-16	chr:6:32648036
6	rs9272310	32603939	T	3.221	9.67E-16	chr:6:32648036
6	rs9272509	32606357	A	2.757	9.87E-16	chr:6:32648036
6	rs28371261	32658727	A	5.267	9.95E-16	chr:6:32648036
6	rs17843584	32615668	T	3.244	1.01E-15	chr:6:32648036
6	rs9274510	32634184	G	2.956	1.03E-15	chr:6:32648036
6	rs28371168	32610751	G	4.091	1.05E-15	chr:6:32648036
6	rs28383263	32585843	T	6.104	1.05E-15	chr:6:32648036
6	rs9274561	32634953	G	3.358	1.09E-15	chr:6:32648036
6	rs9271523	32589806	G	4.336	1.10E-15	chr:6:32648036
6	rs2308890	32610008	T	4.095	1.11E-15	chr:6:32648036
6	rs138512769	32324390	G	8.291	1.13E-15	chr:6:32648036
6	rs541367648	32251332	C	8.291	1.13E-15	chr:6:32648036
6	rs9501169	32232184	G	8.291	1.13E-15	chr:6:32648036
6	rs9272645	32608331	G	2.748	1.18E-15	chr:6:32648036
6	rs9271469	32588630	C	4.351	1.19E-15	chr:6:32648036
6	rs9271470	32588662	T	4.322	1.20E-15	chr:6:32648036
6	rs28383247	32584525	A	5.565	1.23E-15	chr:6:32648036
6	rs534644711	32552719	C	6.327	1.24E-15	chr:6:32648036
6	rs1063349	32627906	C	3.477	1.27E-15	chr:6:32648036
6	rs28383339	32604395	G	5.291	1.28E-15	chr:6:32648036
6	rs10080962	32214285	C	8.01	1.35E-15	chr:6:32648036
6	rs114165089	32219611	T	8.01	1.35E-15	chr:6:32648036
6	rs114901188	32316209	A	8.01	1.35E-15	chr:6:32648036
6	rs115169690	32313182	G	8.01	1.35E-15	chr:6:32648036
6	rs115739548	32245836	T	8.01	1.35E-15	chr:6:32648036
6	rs116054853	32315807	G	8.01	1.35E-15	chr:6:32648036
6	rs13437404	32277153	C	8.01	1.35E-15	chr:6:32648036
6	rs2395148	32321554	T	8.01	1.35E-15	chr:6:32648036
6	rs375566997	32297653	T	8.01	1.35E-15	chr:6:32648036
6	rs6914179	32309876	C	8.01	1.35E-15	chr:6:32648036
6	rs9469099	32308908	A	8.01	1.35E-15	chr:6:32648036
6	rs9501171	32233354	T	8.01	1.35E-15	chr:6:32648036
6	rs9501175	32283333	C	8.01	1.35E-15	chr:6:32648036
6	rs9271503	32589326	A	4.362	1.35E-15	chr:6:32648036
6	rs9275043	32648061	C	2.866	1.37E-15	chr:6:32648036

6	rs9272319	32604108	C	3.724	1.38E-15	chr:6:32648036
6	rs281863884	32630401	T	2.471	1.40E-15	chr:6:32648036
6	rs9272407	32604931	A	3.54	1.42E-15	chr:6:32648036
6	rs28383271	32586134	T	5.545	1.46E-15	chr:6:32648036
6	rs9275069	32648437	G	3.675	1.48E-15	chr:6:32648036
6	rs9271677	32592898	A	3.913	1.49E-15	chr:6:32648036
6	rs17612788	32620344	A	2.608	1.50E-15	chr:6:32648036
6	rs3020625	32628835	C	2.822	1.51E-15	chr:6:32648036
6	rs9272317	32604051	T	4.2	1.52E-15	chr:6:32648036
6	rs9273710	32629314	A	2.708	1.56E-15	chr:6:32648036
6	rs9271767	32594176	T	4.184	1.57E-15	chr:6:32648036
6	rs9272547	32606949	C	2.815	1.57E-15	chr:6:32648036
6	rs114263604	32644859	T	4.977	1.60E-15	chr:6:32648036
6	rs1130161	32610994	A	4.143	1.63E-15	chr:6:32648036
6	rs9272609	32607750	C	3.069	1.65E-15	chr:6:32648036
6	rs13204933	32583026	G	5.508	1.66E-15	chr:6:32648036
6	rs9273009	32611579	G	3.843	1.66E-15	chr:6:32648036
6	rs9274279	32631712	G	2.516	1.73E-15	chr:6:32648036
6	rs28752519	32584705	C	5.501	1.75E-15	chr:6:32648036
6	rs28383429	32608844	G	4.505	1.76E-15	chr:6:32648036
6	rs28752528	32585007	T	6.152	1.78E-15	chr:6:32648036
6	rs17843580	32615551	G	2.885	1.80E-15	chr:6:32648036
6	rs28383273	32586221	T	5.664	1.81E-15	chr:6:32648036
6	rs9273544	32628833	G	2.803	1.84E-15	chr:6:32648036
6	rs9272779	32610314	C	3.396	1.84E-15	chr:6:32648036
6	rs3134988	32640772	G	0.2863	1.94E-15	chr:6:32648036
6	rs9271644	32592337	A	3.888	1.96E-15	chr:6:32648036
6	rs28383272	32586207	A	5.648	1.96E-15	chr:6:32648036
6	rs9274962	32641936	A	2.785	1.98E-15	chr:6:32648036
6	rs17843575	32615427	T	2.816	2.03E-15	chr:6:32648036
6	rs17843576	32615431	A	2.816	2.03E-15	chr:6:32648036
6	rs28383368	32607047	G	3.241	2.09E-15	chr:6:32648036
6	rs6925976	32755092	G	8.583	2.12E-15	chr:6:32648036
6	rs9501212	32760006	G	8.583	2.12E-15	chr:6:32648036
6	rs9501213	32760383	T	8.583	2.12E-15	chr:6:32648036
6	rs17612583	32615470	A	2.798	2.15E-15	chr:6:32648036
6	rs17840121	32577693	C	5.377	2.19E-15	chr:6:32648036
6	rs9271571	32590483	T	4.25	2.19E-15	chr:6:32648036
6	rs75490552	32389552	G	7.069	2.20E-15	chr:6:32648036
6	rs28371212	32637538	A	3.125	2.23E-15	chr:6:32648036
6	rs28383334	32601969	A	4.737	2.25E-15	chr:6:32648036
6	rs9272497	32606176	T	3.342	2.26E-15	chr:6:32648036
6	rs28383431	32608856	C	4.521	2.27E-15	chr:6:32648036

6	rs28383341	32604744	A	4.871	2.28E-15	chr:6:32648036
6	rs1048726	32611012	T	2.416	2.32E-15	chr:6:32648036
6	rs9272613	32607829	G	3.217	2.35E-15	chr:6:32648036
6	rs281863879	32630407	G	2.446	2.38E-15	chr:6:32648036
6	rs1065036	32611029	C	2.418	2.39E-15	chr:6:32648036
6	rs9272905	32610836	T	3.965	2.41E-15	chr:6:32648036
6	rs281863167	32631632	A	2.605	2.42E-15	chr:6:32648036
6	rs9272974	32611315	T	2.546	2.46E-15	chr:6:32648036
6	rs9273008	32611572	A	3.81	2.48E-15	chr:6:32648036
6	rs558693707	32644578	A	5.079	2.49E-15	chr:6:32648036
6	rs28724240	32629328	A	9.436	2.51E-15	chr:6:32648036
6	rs28724241	32629330	T	9.436	2.51E-15	chr:6:32648036
6	rs9271568	32590463	A	4.219	2.59E-15	chr:6:32648036
6	rs622137	32569852	A	4.17	2.61E-15	chr:6:32648036
6	rs9273025	32611760	T	3.566	2.65E-15	chr:6:32648036
6	rs9273027	32611783	G	2.565	2.72E-15	chr:6:32648036
6	rs28383332	32601128	A	5.231	2.82E-15	chr:6:32648036
6	rs28383246	32584482	A	5.437	2.92E-15	chr:6:32648036
6	rs9273056	32612097	G	2.738	2.96E-15	chr:6:32648036
6	rs9271682	32592923	G	3.907	2.99E-15	chr:6:32648036
6	rs28383262	32585773	T	5.521	3.06E-15	chr:6:32648036
6	rs9273038	32611931	T	2.776	3.11E-15	chr:6:32648036
6	rs2516051	32570184	T	4.128	3.13E-15	chr:6:32648036
6	rs9273547	32628844	C	2.806	3.15E-15	chr:6:32648036
6	rs9273084	32612396	C	2.684	3.18E-15	chr:6:32648036
6	rs3104389	32595097	A	3.812	3.19E-15	chr:6:32648036
6	rs11752027	32512534	A	5.571	3.24E-15	chr:6:32648036
6	rs617578	32574603	A	9.153	3.24E-15	chr:6:32648036
6	rs9271776	32594341	C	3.35	3.34E-15	chr:6:32648036
6	rs9272290	32603713	C	3.281	3.36E-15	chr:6:32648036
6	rs281863177	32631620	T	2.649	3.43E-15	chr:6:32648036
6	rs9271480	32588831	A	4.233	3.47E-15	chr:6:32648036
6	rs17843606	32620399	T	2.536	3.52E-15	chr:6:32648036
6	rs28383243	32584410	G	5.395	3.54E-15	chr:6:32648036
6	rs9271521	32589783	T	4.187	3.56E-15	chr:6:32648036
6	rs114544105	32635629	A	4.442	3.72E-15	chr:6:32648036
6	rs9274348	32632381	C	2.397	3.72E-15	chr:6:32648036
6	rs114816309	32182875	C	6.44	3.73E-15	chr:6:32648036
6	rs114484741	32699757	C	8.998	3.79E-15	chr:6:32648036
6	rs9273086	32612410	T	2.679	3.89E-15	chr:6:32648036
6	rs28371250	32648039	T	2.706	4.09E-15	chr:6:32648036
6	rs17843605	32620391	T	2.532	4.16E-15	chr:6:32648036
6	rs397843773	32610171	T	3.921	4.21E-15	chr:6:32648036

6	rs9271685	32592970	C	3.874	4.22E-15	chr:6:32648036
6	rs9273321	32623096	G	2.399	4.42E-15	chr:6:32648036
6	rs2516052	32570147	G	4.09	4.43E-15	chr:6:32648036
6	rs9273419	32627365	C	2.804	4.50E-15	chr:6:32648036
6	rs17612576	32615458	A	2.615	4.57E-15	chr:6:32648036
6	rs9274824	32639702	T	3.094	4.69E-15	chr:6:32648036
6	rs3129766	32594441	G	3.659	4.72E-15	chr:6:32648036
6	rs9271734	32593639	A	4.499	4.78E-15	chr:6:32648036
6	rs9272899	32610809	T	3.999	4.83E-15	chr:6:32648036
6	rs28752531	32585031	T	7.661	4.89E-15	chr:6:32648036
6	rs9272670	32608820	T	2.992	4.94E-15	chr:6:32648036
6	rs6457594	32443961	A	0.2397	5.12E-15	chr:6:32648036
6	rs28383181	32571669	T	4.107	5.16E-15	chr:6:32648036
6	rs559469172	32503489	A	5.499	5.26E-15	chr:6:32648036
6	rs114201912	32519431	A	9.665	5.31E-15	chr:6:32648036
6	rs9272990	32611427	A	3.606	5.31E-15	chr:6:32648036
6	rs477515	32569691	A	4.047	5.37E-15	chr:6:32648036
6	rs9272664	32608710	T	2.943	5.52E-15	chr:6:32648036
6	rs116022266	32610201	A	3.898	5.57E-15	chr:6:32648036
6	rs79351547	32610196	A	3.898	5.57E-15	chr:6:32648036
6	rs28383367	32607031	T	3.166	5.62E-15	chr:6:32648036
6	rs9273264	32614303	G	2.346	5.71E-15	chr:6:32648036
6	rs568924277	32606505	C	5.555	5.82E-15	chr:6:32648036
6	rs9501609	32209157	A	7.099	5.87E-15	chr:6:32648036
6	rs9272520	32606475	A	2.593	5.96E-15	chr:6:32648036
6	rs17612562	32615446	G	2.61	5.97E-15	chr:6:32648036
6	rs9274503	32634103	C	2.742	6.10E-15	chr:6:32648036
6	rs76858292	32776358	G	8.195	6.14E-15	chr:6:32648036
6	rs9273424	32627394	T	2.741	6.19E-15	chr:6:32648036
6	rs9273425	32627396	T	2.741	6.19E-15	chr:6:32648036
6	rs9273319	32623050	T	2.38	6.22E-15	chr:6:32648036
6	rs28371169	32610819	G	3.945	6.26E-15	chr:6:32648036
6	rs9272948	32611107	C	2.322	6.35E-15	chr:6:32648036
6	rs9273320	32623072	G	2.388	6.36E-15	chr:6:32648036
6	rs9273418	32627362	C	2.779	6.48E-15	chr:6:32648036
6	rs9274564	32635048	A	2.741	6.52E-15	chr:6:32648036
6	rs9273497	32628297	C	3.313	6.54E-15	chr:6:32648036
6	rs28371256	32658366	C	4.769	6.62E-15	chr:6:32648036
6	rs28371257	32658375	G	4.769	6.62E-15	chr:6:32648036
6	rs9275046	32648080	C	2.813	6.71E-15	chr:6:32648036
6	rs9275047	32648084	G	2.813	6.71E-15	chr:6:32648036
6	rs9272611	32607780	T	3.075	7.02E-15	chr:6:32648036
6	rs78556970	32626865	C	2.976	7.12E-15	chr:6:32648036

6	rs9273417	32627352	G	2.774	7.22E-15	chr:6:32648036
6	rs28383244	32584462	A	5.297	7.29E-15	chr:6:32648036
6	rs28383245	32584479	C	5.297	7.29E-15	chr:6:32648036
6	rs9271853	32595117	T	3.415	7.29E-15	chr:6:32648036
6	rs80108580	32763128	G	8.082	7.35E-15	chr:6:32648036
6	rs9296045	32766405	A	8.082	7.35E-15	chr:6:32648036
6	rs9501214	32771929	T	8.082	7.35E-15	chr:6:32648036
6	rs9986640	32764970	A	8.082	7.35E-15	chr:6:32648036
6	rs112464670	32683018	C	7.72	7.48E-15	chr:6:32648036
6	rs564176274	32642025	T	10.18	7.50E-15	chr:6:32648036
6	rs9271498	32589282	A	4.164	8.11E-15	chr:6:32648036
6	rs28383264	32585901	T	5.423	8.13E-15	chr:6:32648036
6	rs28383265	32585924	T	5.423	8.13E-15	chr:6:32648036
6	rs9272962	32611229	T	2.72	8.20E-15	chr:6:32648036
6	rs9272896	32610806	C	3.977	8.23E-15	chr:6:32648036
6	rs9274506	32634141	C	2.782	8.23E-15	chr:6:32648036
6	rs9272940	32611081	C	4.307	8.23E-15	chr:6:32648036
6	rs61729683	32185818	T	6.022	8.24E-15	chr:6:32648036
6	rs28755178	32641503	G	7.134	8.37E-15	chr:6:32648036
6	rs9273132	32612800	G	2.506	8.47E-15	chr:6:32648036
6	rs9272634	32608184	T	2.944	8.59E-15	chr:6:32648036
6	rs113140854	32634588	T	10.23	8.79E-15	chr:6:32648036
6	rs9272995	32611466	G	3.458	8.88E-15	chr:6:32648036
6	rs9272950	32611117	G	2.305	8.94E-15	chr:6:32648036
6	rs28383351	32606464	C	4.713	9.17E-15	chr:6:32648036
6	rs2858300	32643261	A	4.725	9.48E-15	chr:6:32648036
6	rs28383366	32606980	A	4.543	9.57E-15	chr:6:32648036
6	rs17843620	32620800	G	2.708	9.62E-15	chr:6:32648036
6	rs28746794	32632532	C	2.403	9.65E-15	chr:6:32648036
6	rs28383380	32607253	A	3.099	9.65E-15	chr:6:32648036
6	rs9271762	32594028	G	3.942	9.73E-15	chr:6:32648036
6	rs9273704	32629297	C	2.619	9.78E-15	chr:6:32648036
6	rs3207983	32609213	A	4.359	9.79E-15	chr:6:32648036
6	rs9272943	32611092	G	2.306	9.83E-15	chr:6:32648036
6	rs535440044	32683306	G	7.174	9.86E-15	chr:6:32648036
6	rs2858878	32620487	G	2.494	9.88E-15	chr:6:32648036
6	rs3104393	32594403	T	3.146	1.01E-14	chr:6:32648036
6	rs5002178	32611590	G	0.3108	1.05E-14	chr:6:32648036
6	rs9274371	32632553	G	2.446	1.05E-14	chr:6:32648036
6	rs9274372	32632555	A	2.446	1.05E-14	chr:6:32648036
6	rs9271660	32592602	G	3.555	1.07E-14	chr:6:32648036
6	rs9272989	32611418	C	3.54	1.07E-14	chr:6:32648036
6	rs201414107	32647866	A	4.334	1.08E-14	chr:6:32648036

6	rs9271758	32593997	A	2.496	1.10E-14	chr:6:32648036
6	rs9271759	32594006	A	2.496	1.10E-14	chr:6:32648036
6	rs28383378	32607239	A	3.126	1.10E-14	chr:6:32648036
6	rs28383379	32607242	A	3.126	1.10E-14	chr:6:32648036
6	rs9273174	32613237	G	4.203	1.11E-14	chr:6:32648036
6	rs17843699	32622238	G	2.285	1.17E-14	chr:6:32648036
6	rs9274986	32642386	A	3.069	1.17E-14	chr:6:32648036
6	rs185812018	32632514	T	2.402	1.17E-14	chr:6:32648036
6	rs12192221	32580928	G	5.241	1.19E-14	chr:6:32648036
6	rs9272371	32604654	C	2.568	1.24E-14	chr:6:32648036
6	rs9271520	32589771	G	4.053	1.24E-14	chr:6:32648036
6	rs9272598	32607611	T	2.58	1.24E-14	chr:6:32648036
6	rs9272494	32606085	T	3.021	1.27E-14	chr:6:32648036
6	rs28383274	32586246	G	5.409	1.28E-14	chr:6:32648036
6	rs199705602	32606584	G	2.522	1.32E-14	chr:6:32648036
6	rs28383251	32585567	T	5.246	1.32E-14	chr:6:32648036
6	rs28383252	32585574	T	5.246	1.32E-14	chr:6:32648036
6	rs28383377	32607227	G	3.146	1.32E-14	chr:6:32648036
6	rs9271760	32594012	T	2.49	1.36E-14	chr:6:32648036
6	rs9273330	32623300	T	2.651	1.37E-14	chr:6:32648036
6	rs9274368	32632518	G	2.398	1.37E-14	chr:6:32648036
6	rs28383250	32585525	T	5.321	1.38E-14	chr:6:32648036
6	rs1056316	32627494	C	4.187	1.41E-14	chr:6:32648036
6	rs9272983	32611392	T	3.411	1.42E-14	chr:6:32648036
6	rs9272984	32611393	G	3.411	1.42E-14	chr:6:32648036
6	rs9272614	32607859	T	3.115	1.44E-14	chr:6:32648036
6	rs9272597	32607605	G	2.577	1.45E-14	chr:6:32648036
6	rs9273268	32614334	C	2.333	1.46E-14	chr:6:32648036
6	rs9272607	32607735	A	2.772	1.47E-14	chr:6:32648036
6	rs1064993	32611181	A	3.618	1.48E-14	chr:6:32648036
6	rs9271661	32592605	A	3.53	1.49E-14	chr:6:32648036
6	rs9274987	32642405	T	3.006	1.50E-14	chr:6:32648036
6	rs9274988	32642406	T	3.006	1.50E-14	chr:6:32648036
6	rs585305	32572048	T	4.099	1.51E-14	chr:6:32648036
6	rs17843701	32622256	T	2.263	1.53E-14	chr:6:32648036
6	rs9272533	32606665	T	2.556	1.54E-14	chr:6:32648036
6	rs9273185	32613340	G	2.471	1.54E-14	chr:6:32648036
6	rs9272958	32611170	A	2.445	1.57E-14	chr:6:32648036
6	rs9271432	32588056	T	4.045	1.58E-14	chr:6:32648036
6	rs9271433	32588057	G	4.045	1.58E-14	chr:6:32648036
6	rs9500908	32770666	A	7.861	1.67E-14	chr:6:32648036
6	rs28383360	32606824	A	4.237	1.70E-14	chr:6:32648036
6	rs9273257	32614240	G	2.293	1.70E-14	chr:6:32648036

6	rs9272981	32611373	A	2.44	1.75E-14	chr:6:32648036
6	rs77287921	32816216	C	7.286	1.77E-14	chr:6:32648036
6	rs187265184	32525769	G	3.006	1.83E-14	chr:6:32648036
6	rs9273685	32629269	T	8.239	1.87E-14	chr:6:32648036
6	rs9272998	32611474	A	3.356	1.89E-14	chr:6:32648036
6	rs9272339	32604274	A	0.3382	1.92E-14	chr:6:32648036
6	rs281863704	32630661	C	2.746	1.94E-14	chr:6:32648036
6	rs191032570	32610632	A	5.226	1.98E-14	chr:6:32648036
6	rs9272987	32611403	A	3.366	1.99E-14	chr:6:32648036
6	rs9273380	32626895	A	2.86	1.99E-14	chr:6:32648036
6	rs9271517	32589740	G	4.032	2.04E-14	chr:6:32648036
6	rs9271519	32589751	C	4.032	2.04E-14	chr:6:32648036
6	rs9273331	32623307	T	2.579	2.04E-14	chr:6:32648036
6	rs9273772	32629438	C	2.829	2.06E-14	chr:6:32648036
6	rs9272571	32607251	T	2.519	2.07E-14	chr:6:32648036
6	rs9273172	32613224	C	2.978	2.08E-14	chr:6:32648036
6	rs17843613	32620658	T	2.364	2.09E-14	chr:6:32648036
6	rs9273266	32614326	A	2.316	2.09E-14	chr:6:32648036
6	rs115624624	32599083	A	4.979	2.09E-14	chr:6:32648036
6	rs116202168	32599088	T	4.979	2.09E-14	chr:6:32648036
6	rs9273030	32611805	A	2.459	2.18E-14	chr:6:32648036
6	rs35779483	32626702	G	3.296	2.18E-14	chr:6:32648036
6	rs7744001	32626086	A	3.296	2.18E-14	chr:6:32648036
6	rs7744020	32626130	A	3.296	2.18E-14	chr:6:32648036
6	rs9271761	32594022	A	3.619	2.20E-14	chr:6:32648036
6	rs707947	32611068	G	4.219	2.25E-14	chr:6:32648036
6	rs28383384	32607414	A	3.981	2.28E-14	chr:6:32648036
6	rs28383385	32607416	A	3.981	2.28E-14	chr:6:32648036
6	rs9273274	32614405	T	2.298	2.29E-14	chr:6:32648036
6	rs9273275	32614407	G	2.298	2.29E-14	chr:6:32648036
6	rs9271465	32588554	T	4.041	2.31E-14	chr:6:32648036
6	rs12722061	32609221	C	4.34	2.37E-14	chr:6:32648036
6	rs9273317	32623003	C	2.314	2.38E-14	chr:6:32648036
6	rs9273318	32623004	C	2.314	2.38E-14	chr:6:32648036
6	rs9273244	32614108	G	2.523	2.41E-14	chr:6:32648036
6	rs9273245	32614110	A	2.523	2.41E-14	chr:6:32648036
6	rs28383253	32585589	T	5.159	2.42E-14	chr:6:32648036
6	rs9273267	32614330	C	2.312	2.46E-14	chr:6:32648036
6	rs9273088	32612488	C	2.417	2.49E-14	chr:6:32648036
6	rs9271744	32593755	A	3.161	2.50E-14	chr:6:32648036
6	rs281874798	32631843	T	2.329	2.54E-14	chr:6:32648036
6	rs9272851	32610704	C	2.991	2.60E-14	chr:6:32648036
6	rs115601017	32191428	G	4.552	2.61E-14	chr:6:32648036

6	rs17843586	32615801	G	2.355	2.64E-14	chr:6:32648036
6	rs17843587	32615802	T	2.355	2.64E-14	chr:6:32648036
6	rs17843588	32615807	G	2.355	2.64E-14	chr:6:32648036
6	rs17843614	32620661	G	2.348	2.64E-14	chr:6:32648036
6	rs115691564	32610122	T	5.226	2.67E-14	chr:6:32648036
6	rs17843607	32620435	A	2.458	2.74E-14	chr:6:32648036
6	rs28383435	32608972	T	4.007	2.77E-14	chr:6:32648036
6	rs9273269	32614343	A	2.3	2.91E-14	chr:6:32648036
6	rs2228110	32816889	A	6.834	2.91E-14	chr:6:32648036
6	rs9273026	32611769	C	3.428	2.92E-14	chr:6:32648036
6	rs28724138	32552617	A	3.858	2.93E-14	chr:6:32648036
6	rs9273259	32614269	G	2.28	2.94E-14	chr:6:32648036
6	rs9273260	32614270	G	2.28	2.94E-14	chr:6:32648036
6	rs76855996	32880673	A	6.653	2.95E-14	chr:6:32648036
6	rs28371246	32647995	C	2.483	2.95E-14	chr:6:32648036
6	rs9273261	32614284	G	2.273	2.96E-14	chr:6:32648036
6	rs9272675	32608888	T	2.9	2.96E-14	chr:6:32648036
6	rs9272955	32611145	A	3.582	2.99E-14	chr:6:32648036
6	rs1064985	32611034	A	4.161	3.11E-14	chr:6:32648036
6	rs9268159	32269487	T	4.159	3.13E-14	chr:6:32648036
6	rs1140321	32632581	G	2.44	3.13E-14	chr:6:32648036
6	rs1140322	32632578	G	2.44	3.13E-14	chr:6:32648036
6	rs1140318	32632593	T	2.44	3.17E-14	chr:6:32648036
6	rs1140319	32632592	G	2.44	3.17E-14	chr:6:32648036
6	rs1140320	32632589	G	2.44	3.17E-14	chr:6:32648036
6	rs9273311	32622967	A	2.321	3.17E-14	chr:6:32648036
6	rs28383420	32608707	C	3.822	3.18E-14	chr:6:32648036
6	rs28383254	32585638	C	5.163	3.23E-14	chr:6:32648036
6	rs28383255	32585641	A	5.163	3.23E-14	chr:6:32648036
6	rs17613292	32621861	A	2.345	3.33E-14	chr:6:32648036
6	rs368699425	32602553	A	4.982	3.33E-14	chr:6:32648036
6	rs9273767	32629432	G	2.814	3.35E-14	chr:6:32648036
6	rs9272054	32599164	A	0.286	3.44E-14	chr:6:32648036
6	rs147774651	32853253	C	6.583	3.44E-14	chr:6:32648036
6	rs9273314	32622986	C	2.324	3.46E-14	chr:6:32648036
6	rs9273315	32622990	T	2.324	3.46E-14	chr:6:32648036
6	rs6936669	32799554	C	7.79	3.47E-14	chr:6:32648036
6	rs9274623	32635998	T	4.449	3.49E-14	chr:6:32648036
6	rs9272951	32611125	C	3.731	3.55E-14	chr:6:32648036
6	rs9273556	32628953	G	2.551	3.58E-14	chr:6:32648036
6	rs9273271	32614382	C	2.29	3.58E-14	chr:6:32648036
6	rs191329273	32627227	C	10.34	3.64E-14	chr:6:32648036
6	rs115910284	32601655	A	4.717	3.65E-14	chr:6:32648036

6	rs9271707	32593356	T	0.2826	3.66E-14	chr:6:32648036
6	rs9272957	32611152	A	3.558	3.67E-14	chr:6:32648036
6	rs9273559	32628967	T	2.517	3.69E-14	chr:6:32648036
6	rs28383370	32607152	C	3.128	3.76E-14	chr:6:32648036
6	rs28383371	32607154	T	3.128	3.76E-14	chr:6:32648036
6	rs3020628	32628784	A	2.817	3.76E-14	chr:6:32648036
6	rs17843600	32620148	G	2.261	3.81E-14	chr:6:32648036
6	rs9271741	32593734	T	2.452	3.82E-14	chr:6:32648036
6	rs17612654	32615813	T	2.324	3.82E-14	chr:6:32648036
6	rs17612661	32615814	G	2.324	3.82E-14	chr:6:32648036
6	rs115139056	32603425	A	4.861	3.85E-14	chr:6:32648036
6	rs535476317	32570019	A	3.783	3.94E-14	chr:6:32648036
6	rs9273415	32627310	T	2.662	3.96E-14	chr:6:32648036
6	rs9272971	32611302	C	2.42	4.01E-14	chr:6:32648036
6	rs141274999	32608808	G	4.727	4.09E-14	chr:6:32648036
6	rs9273273	32614392	T	2.28	4.16E-14	chr:6:32648036
6	rs9273096	32612540	C	2.337	4.30E-14	chr:6:32648036
6	rs114027542	32602231	T	5.023	4.34E-14	chr:6:32648036
6	rs114799674	32602880	A	4.885	4.36E-14	chr:6:32648036
6	rs115503527	32602899	A	4.885	4.36E-14	chr:6:32648036
6	rs115573799	32602884	C	4.885	4.36E-14	chr:6:32648036
6	rs9275054	32648208	G	2.786	4.45E-14	chr:6:32648036
6	rs9275055	32648220	C	2.786	4.45E-14	chr:6:32648036
6	rs28421666	32592737	G	4.877	4.46E-14	chr:6:32648036
6	rs9273382	32626925	G	2.825	4.50E-14	chr:6:32648036
6	rs12205706	32580999	T	4.915	4.51E-14	chr:6:32648036
6	rs9274453	32633369	A	2.409	4.76E-14	chr:6:32648036
6	rs559673115	32629239	G	2.43	4.76E-14	chr:6:32648036
6	rs9272662	32608681	G	2.836	4.76E-14	chr:6:32648036
6	rs576909786	32569270	T	9.247	4.77E-14	chr:6:32648036
6	rs16822516	32552092	T	4.137	4.81E-14	chr:6:32648036
6	rs9273432	32627485	C	2.669	4.89E-14	chr:6:32648036
6	rs9273433	32627489	G	2.669	4.89E-14	chr:6:32648036
6	rs114307619	32596625	A	4.895	5.10E-14	chr:6:32648036
6	rs148819373	32598624	G	4.895	5.10E-14	chr:6:32648036
6	rs201980192	32609063	T	4.895	5.10E-14	chr:6:32648036
6	rs564621439	32598986	A	4.895	5.10E-14	chr:6:32648036
6	rs78798979	32593094	T	4.895	5.10E-14	chr:6:32648036
6	rs574582728	32608989	T	4.665	5.18E-14	chr:6:32648036
6	rs36206058	32626920	G	2.82	5.26E-14	chr:6:32648036
6	rs9273313	32622979	C	2.303	5.26E-14	chr:6:32648036
6	rs9273534	32628751	T	2.81	5.30E-14	chr:6:32648036
6	rs17613424	32622195	T	2.233	5.34E-14	chr:6:32648036

6	rs9274300	32631970	G	2.238	5.37E-14	chr:6:32648036
6	rs9273414	32627299	T	2.622	5.39E-14	chr:6:32648036
6	rs576530137	32629015	C	2.296	5.44E-14	chr:6:32648036
6	rs116178934	32605904	T	5.116	5.44E-14	chr:6:32648036
6	rs17612489	32614749	A	2.233	5.51E-14	chr:6:32648036
6	rs145878889	32609446	C	5.262	5.70E-14	chr:6:32648036
6	rs533028614	32609452	C	5.262	5.70E-14	chr:6:32648036
6	rs28383256	32585675	C	5.301	5.72E-14	chr:6:32648036
6	rs28383257	32585680	T	5.301	5.72E-14	chr:6:32648036
6	rs28383449	32609228	A	7.79	5.73E-14	chr:6:32648036
6	rs3188043	32609233	C	7.79	5.73E-14	chr:6:32648036
6	rs139025277	32646859	G	4.21	5.75E-14	chr:6:32648036
6	rs73738964	32900760	A	6.418	5.79E-14	chr:6:32648036
6	rs9273339	32623430	A	2.3	5.92E-14	chr:6:32648036
6	rs28383362	32606864	C	2.97	5.95E-14	chr:6:32648036
6	rs28383363	32606868	C	2.97	5.95E-14	chr:6:32648036
6	rs3104385	32597538	G	2.769	6.11E-14	chr:6:32648036
6	rs9274358	32632469	C	2.354	6.11E-14	chr:6:32648036
6	rs9272561	32607141	A	2.563	6.23E-14	chr:6:32648036
6	rs9274980	32642290	T	3.062	6.28E-14	chr:6:32648036
6	rs9273255	32614228	G	2.255	6.36E-14	chr:6:32648036
6	rs17613437	32622282	C	2.209	6.38E-14	chr:6:32648036
6	rs9271742	32593747	T	2.431	6.62E-14	chr:6:32648036
6	rs9271743	32593751	G	2.431	6.62E-14	chr:6:32648036
6	rs17843616	32620667	C	2.305	6.64E-14	chr:6:32648036
6	rs9273309	32622949	G	2.265	6.68E-14	chr:6:32648036
6	rs707951	32609813	T	0.3019	6.70E-14	chr:6:32648036
6	rs115421755	32647108	A	4.25	6.73E-14	chr:6:32648036
6	rs9273332	32623317	T	2.495	6.77E-14	chr:6:32648036
6	rs9273333	32623325	C	2.495	6.77E-14	chr:6:32648036
6	rs185835704	32728576	A	5.592	6.98E-14	chr:6:32648036
6	rs9267858	32196575	T	3.473	7.05E-14	chr:6:32648036
6	rs28383258	32585701	T	5.23	7.08E-14	chr:6:32648036
6	rs555685178	32609581	T	5.204	7.12E-14	chr:6:32648036
6	rs9271893	32595859	T	0.2897	7.13E-14	chr:6:32648036
6	rs9273277	32614414	A	2.253	7.26E-14	chr:6:32648036
6	rs9273278	32614415	C	2.253	7.26E-14	chr:6:32648036
6	rs9273134	32612807	T	2.407	7.33E-14	chr:6:32648036
6	rs9271745	32593786	G	2.416	7.36E-14	chr:6:32648036
6	rs28752533	32585058	A	6.319	7.45E-14	chr:6:32648036
6	rs17206287	32674725	G	4.426	7.50E-14	chr:6:32648036
6	rs17612809	32620459	T	2.324	7.55E-14	chr:6:32648036
6	rs28383434	32608925	T	3.908	7.97E-14	chr:6:32648036

6	rs17843615	32620663	C	2.299	7.97E-14	chr:6:32648036
6	rs9273243	32614091	A	2.521	7.98E-14	chr:6:32648036
6	rs28383424	32608756	G	3.842	8.07E-14	chr:6:32648036
6	rs28383425	32608761	C	3.842	8.07E-14	chr:6:32648036
6	rs9273413	32627292	G	2.591	8.09E-14	chr:6:32648036
6	rs115296161	32603463	T	4.812	8.31E-14	chr:6:32648036
6	rs143117432	32598186	T	4.372	8.34E-14	chr:6:32648036
6	rs9272629	32608096	T	2.975	8.37E-14	chr:6:32648036
6	rs371537406	32602839	G	4.738	8.43E-14	chr:6:32648036
6	rs9272044	32598971	A	0.2912	8.51E-14	chr:6:32648036
6	rs9273250	32614180	T	2.25	8.58E-14	chr:6:32648036
6	rs144238786	32601770	T	4.845	8.67E-14	chr:6:32648036
6	rs186564411	32601771	G	4.845	8.67E-14	chr:6:32648036
6	rs9271912	32596207	T	0.2941	8.69E-14	chr:6:32648036
6	rs116728358	32600062	G	4.734	8.75E-14	chr:6:32648036
6	rs115315696	32599028	G	4.664	8.77E-14	chr:6:32648036
6	rs139692935	32598983	A	4.664	8.77E-14	chr:6:32648036
6	rs546460367	32598985	A	4.664	8.77E-14	chr:6:32648036
6	rs550642974	32599041	G	4.664	8.77E-14	chr:6:32648036
6	rs9271757	32593969	A	2.399	8.79E-14	chr:6:32648036
6	rs28383279	32586318	T	6.143	8.84E-14	chr:6:32648036
6	rs9271907	32596123	T	0.2931	8.95E-14	chr:6:32648036
6	rs9271908	32596131	T	0.2931	8.95E-14	chr:6:32648036
6	rs9273416	32627321	G	2.629	8.95E-14	chr:6:32648036
6	rs116334170	32598500	A	4.18	9.14E-14	chr:6:32648036
6	rs116517496	32595773	A	4.708	9.17E-14	chr:6:32648036
6	rs9273279	32614426	T	2.246	9.22E-14	chr:6:32648036
6	rs9273514	32628538	T	2.823	9.26E-14	chr:6:32648036
6	rs9273515	32628539	A	2.823	9.26E-14	chr:6:32648036
6	rs114352945	32602648	T	4.809	9.31E-14	chr:6:32648036
6	rs28383375	32607203	A	3.076	9.33E-14	chr:6:32648036
6	rs9273312	32622973	T	2.28	9.37E-14	chr:6:32648036
6	rs17613410	32622186	C	2.209	9.43E-14	chr:6:32648036
6	rs17613417	32622190	G	2.209	9.43E-14	chr:6:32648036
6	rs9271464	32588544	T	3.859	9.52E-14	chr:6:32648036
6	rs9273335	32623348	G	2.288	9.60E-14	chr:6:32648036
6	rs114025574	32603230	A	4.835	9.60E-14	chr:6:32648036
6	rs114774960	32603234	T	4.835	9.60E-14	chr:6:32648036
6	rs9273101	32612564	A	2.307	9.68E-14	chr:6:32648036
6	rs9272628	32608093	C	2.972	9.72E-14	chr:6:32648036
6	rs28383179	32571116	A	3.204	9.72E-14	chr:6:32648036
6	rs28383180	32571118	C	3.204	9.72E-14	chr:6:32648036
6	rs9469093	32191544	T	3.917	9.78E-14	chr:6:32648036

6	rs9273135	32612816	G	2.387	9.80E-14	chr:6:32648036
6	rs3115567	32227506	T	3.792	9.82E-14	chr:6:32648036
6	rs9274902	32641220	C	2.358	9.88E-14	chr:6:32648036
6	rs2647023	32643325	T	4.466	9.94E-14	chr:6:32648036
6	rs114929610	32602075	A	5.069	1.00E-13	chr:6:32648036
6	rs17613431	32622203	T	2.215	1.00E-13	chr:6:32648036
6	rs6909790	32282979	G	3.642	1.00E-13	chr:6:32648036
6	rs1130162	32610997	G	3.981	1.01E-13	chr:6:32648036
6	rs9269140	32445249	T	3.293	1.01E-13	chr:6:32648036
6	rs9273209	32613610	T	2.207	1.01E-13	chr:6:32648036
6	rs9273242	32614080	C	2.532	1.04E-13	chr:6:32648036
6	rs576628433	32598750	C	4.231	1.07E-13	chr:6:32648036
6	rs9272055	32599176	A	0.2964	1.07E-13	chr:6:32648036
6	rs113695046	32522072	T	6.731	1.08E-13	chr:6:32648036
6	rs9273306	32622928	A	2.246	1.12E-13	chr:6:32648036
6	rs9273307	32622929	C	2.246	1.12E-13	chr:6:32648036
6	rs9274537	32634655	A	2.617	1.12E-13	chr:6:32648036
6	rs281875048	32631217	T	8.084	1.12E-13	chr:6:32648036
6	rs9273191	32613424	T	2.29	1.12E-13	chr:6:32648036
6	rs182585640	32644906	C	4.715	1.14E-13	chr:6:32648036
6	rs28383423	32608752	T	3.802	1.14E-13	chr:6:32648036
6	rs115596571	32602104	T	4.906	1.14E-13	chr:6:32648036
6	rs17612496	32614752	G	2.207	1.15E-13	chr:6:32648036
6	rs143229898	32214785	A	5.758	1.17E-13	chr:6:32648036
6	rs17612935	32620992	G	2.237	1.17E-13	chr:6:32648036
6	rs9296013	32216147	C	5.758	1.17E-13	chr:6:32648036
6	rs9273412	32627263	C	2.586	1.18E-13	chr:6:32648036
6	rs536322397	32605366	C	4.767	1.18E-13	chr:6:32648036
6	rs77455945	32608798	G	4.612	1.18E-13	chr:6:32648036
6	rs9274362	32632486	C	2.335	1.19E-13	chr:6:32648036
6	rs9272913	32610860	T	3.67	1.20E-13	chr:6:32648036
6	rs9272914	32610863	G	3.67	1.20E-13	chr:6:32648036
6	rs114762033	32602753	A	4.762	1.20E-13	chr:6:32648036
6	rs1065043	32611234	T	3.452	1.21E-13	chr:6:32648036
6	rs1065044	32611243	T	3.452	1.21E-13	chr:6:32648036
6	rs373813924	32602332	T	4.97	1.21E-13	chr:6:32648036
6	rs2854273	32628776	A	2.725	1.21E-13	chr:6:32648036
6	rs9272980	32611357	T	3.21	1.21E-13	chr:6:32648036
6	rs9272064	32599338	T	0.2947	1.22E-13	chr:6:32648036
6	rs9273031	32611849	C	3.52	1.22E-13	chr:6:32648036
6	rs9380317	32620740	T	2.33	1.23E-13	chr:6:32648036
6	rs79473311	32601382	G	4.468	1.23E-13	chr:6:32648036
6	rs79573551	32601416	T	4.468	1.23E-13	chr:6:32648036

6	rs114612036	32607350	C	4.984	1.24E-13	chr:6:32648036
6	rs9272108	32600228	C	0.3005	1.26E-13	chr:6:32648036
6	rs17843654	32621378	A	2.239	1.26E-13	chr:6:32648036
6	rs17843570	32614946	G	2.188	1.33E-13	chr:6:32648036
6	rs116172932	32596385	T	4.676	1.35E-13	chr:6:32648036
6	rs28383433	32608887	G	3.922	1.35E-13	chr:6:32648036
6	rs1130151	32610935	G	2.267	1.36E-13	chr:6:32648036
6	rs9274978	32642277	C	2.997	1.37E-13	chr:6:32648036
6	rs72851058	32503213	T	3.494	1.38E-13	chr:6:32648036
6	rs9273105	32612601	A	2.347	1.39E-13	chr:6:32648036
6	rs9501166	32202999	G	4.564	1.39E-13	chr:6:32648036
6	rs114310549	32600248	A	4.576	1.40E-13	chr:6:32648036
6	rs28724234	32627535	T	2.573	1.40E-13	chr:6:32648036
6	rs115938440	32503420	A	4.974	1.41E-13	chr:6:32648036
6	rs116765893	32503445	A	4.974	1.41E-13	chr:6:32648036
6	rs115493697	32603359	A	4.742	1.42E-13	chr:6:32648036
6	rs115240119	32605945	T	5.035	1.43E-13	chr:6:32648036
6	rs9272146	32600914	C	0.301	1.44E-13	chr:6:32648036
6	rs79136294	32899882	A	5.703	1.45E-13	chr:6:32648036
6	rs9394107	32620720	G	2.328	1.45E-13	chr:6:32648036
6	rs9272917	32610892	G	2.289	1.46E-13	chr:6:32648036
6	rs9272918	32610893	A	2.289	1.46E-13	chr:6:32648036
6	rs17612928	32620976	G	2.23	1.46E-13	chr:6:32648036
6	rs17612748	32620174	G	2.254	1.46E-13	chr:6:32648036
6	rs17612816	32620469	G	2.307	1.47E-13	chr:6:32648036
6	rs9272305	32603879	A	4.987	1.47E-13	chr:6:32648036
6	rs9274298	32631902	T	2.266	1.48E-13	chr:6:32648036
6	rs2157051	32658624	G	3.224	1.49E-13	chr:6:32648036
6	rs9271722	32593522	G	3.001	1.50E-13	chr:6:32648036
6	rs9274562	32635000	G	2.816	1.51E-13	chr:6:32648036
6	rs9273239	32613998	T	2.324	1.53E-13	chr:6:32648036
6	rs115609519	32596968	G	4.775	1.53E-13	chr:6:32648036
6	rs9271615	32591686	G	0.2985	1.54E-13	chr:6:32648036
6	rs28383361	32606846	A	2.789	1.54E-13	chr:6:32648036
6	rs9271909	32596177	T	0.2996	1.55E-13	chr:6:32648036
6	rs9274540	32634676	T	2.573	1.55E-13	chr:6:32648036
6	rs17843608	32620454	C	2.294	1.56E-13	chr:6:32648036
6	rs17613381	32622130	T	2.195	1.56E-13	chr:6:32648036
6	rs114260654	32871520	G	6.057	1.57E-13	chr:6:32648036
6	rs114814601	32871521	T	6.057	1.57E-13	chr:6:32648036
6	rs17612831	32620507	T	2.324	1.57E-13	chr:6:32648036
6	rs17843624	32620885	T	2.203	1.57E-13	chr:6:32648036
6	rs9274438	32633181	C	2.748	1.57E-13	chr:6:32648036

6	rs115711192	32602161	T	4.9	1.59E-13	chr:6:32648036
6	rs9272973	32611312	T	3.129	1.59E-13	chr:6:32648036
6	rs28383290	32586475	T	6.149	1.61E-13	chr:6:32648036
6	rs28383291	32586476	G	6.149	1.61E-13	chr:6:32648036
6	rs1048173	32609855	C	3.592	1.65E-13	chr:6:32648036
6	rs9273098	32612556	G	2.283	1.67E-13	chr:6:32648036
6	rs9273100	32612559	G	2.283	1.67E-13	chr:6:32648036
6	rs17843696	32622178	G	2.188	1.67E-13	chr:6:32648036
6	rs9279900	32607583	T	2.375	1.72E-13	chr:6:32648036
6	rs9271913	32596222	A	0.3019	1.73E-13	chr:6:32648036
6	rs17843692	32622103	G	2.192	1.74E-13	chr:6:32648036
6	rs17612533	32615307	G	2.31	1.74E-13	chr:6:32648036
6	rs17612541	32615308	T	2.31	1.74E-13	chr:6:32648036
6	rs17612548	32615312	A	2.31	1.74E-13	chr:6:32648036
6	rs9271945	32597034	A	0.2971	1.77E-13	chr:6:32648036
6	rs9273427	32627441	C	2.561	1.81E-13	chr:6:32648036
6	rs17613285	32621828	A	2.342	1.82E-13	chr:6:32648036
6	rs9271910	32596183	C	0.3018	1.83E-13	chr:6:32648036
6	rs35789108	32550126	G	6.809	1.84E-13	chr:6:32648036
6	rs9273251	32614193	G	2.215	1.86E-13	chr:6:32648036
6	rs281874991	32631281	T	2.649	1.87E-13	chr:6:32648036
6	rs9272067	32599401	A	0.2991	1.87E-13	chr:6:32648036
6	rs28383427	32608792	T	3.849	1.87E-13	chr:6:32648036
6	rs17843609	32620513	C	2.32	1.90E-13	chr:6:32648036
6	rs9271919	32596423	G	0.3007	1.91E-13	chr:6:32648036
6	rs9273188	32613384	T	2.386	1.92E-13	chr:6:32648036
6	rs9272059	32599223	C	0.2995	1.93E-13	chr:6:32648036
6	rs9273438	32627523	G	2.563	1.93E-13	chr:6:32648036
6	rs9273240	32614000	T	2.318	1.94E-13	chr:6:32648036
6	rs17612590	32615578	A	2.351	1.95E-13	chr:6:32648036
6	rs9273436	32627517	G	2.562	1.95E-13	chr:6:32648036
6	rs60601161	32783387	T	4.345	1.97E-13	chr:6:32648036
6	rs17843568	32614941	T	2.175	2.02E-13	chr:6:32648036
6	rs17612727	32616003	T	2.225	2.03E-13	chr:6:32648036
6	rs17612734	32616004	G	2.225	2.03E-13	chr:6:32648036
6	rs9271922	32596444	A	0.2985	2.04E-13	chr:6:32648036
6	rs116236894	32603461	A	4.602	2.04E-13	chr:6:32648036
6	rs9272120	32600501	G	0.3029	2.06E-13	chr:6:32648036
6	rs9273426	32627434	C	2.558	2.06E-13	chr:6:32648036
6	rs9272117	32600411	T	0.2978	2.06E-13	chr:6:32648036
6	rs9273430	32627460	G	2.57	2.07E-13	chr:6:32648036
6	rs9274306	32632006	G	2.217	2.09E-13	chr:6:32648036
6	rs17843593	32615950	C	2.226	2.09E-13	chr:6:32648036

6	rs17843594	32615952	C	2.226	2.09E-13	chr:6:32648036
6	rs9272049	32599045	G	0.3033	2.14E-13	chr:6:32648036
6	rs115214276	32600032	C	4.548	2.15E-13	chr:6:32648036
6	rs17612482	32614726	T	2.198	2.15E-13	chr:6:32648036
6	rs9273305	32622927	C	2.429	2.15E-13	chr:6:32648036
6	rs115231411	32600409	T	4.511	2.17E-13	chr:6:32648036
6	rs116485389	32600457	G	4.511	2.17E-13	chr:6:32648036
6	rs9272969	32611289	G	2.391	2.17E-13	chr:6:32648036
6	rs9273344	32623458	C	2.268	2.19E-13	chr:6:32648036
6	rs17612838	32620538	C	2.304	2.19E-13	chr:6:32648036
6	rs17843612	32620536	A	2.304	2.19E-13	chr:6:32648036
6	rs17612475	32614706	A	2.197	2.20E-13	chr:6:32648036
6	rs17843563	32614705	G	2.197	2.20E-13	chr:6:32648036
6	rs9274575	32635226	G	2.703	2.23E-13	chr:6:32648036
6	rs9273247	32614166	C	2.211	2.25E-13	chr:6:32648036
6	rs9273248	32614167	C	2.211	2.25E-13	chr:6:32648036
6	rs9273249	32614168	A	2.211	2.25E-13	chr:6:32648036
6	rs9273224	32613804	T	2.22	2.29E-13	chr:6:32648036
6	rs9272459	32605578	A	2.92	2.30E-13	chr:6:32648036
6	rs9268142	32256418	C	3.528	2.30E-13	chr:6:32648036
6	rs17612518	32614927	T	2.177	2.35E-13	chr:6:32648036
6	rs17613278	32621810	G	2.334	2.37E-13	chr:6:32648036
6	rs114309058	32594470	A	0.3096	2.39E-13	chr:6:32648036
6	rs10456411	32615904	A	3.567	2.40E-13	chr:6:32648036
6	rs1130158	32610972	A	2.219	2.43E-13	chr:6:32648036
6	rs114866528	32599267	C	4.496	2.44E-13	chr:6:32648036
6	rs116633385	32599268	A	4.496	2.44E-13	chr:6:32648036
6	rs199950886	32603075	G	4.686	2.45E-13	chr:6:32648036
6	rs28383278	32586312	C	5.917	2.49E-13	chr:6:32648036
6	rs685810	32584952	G	3.442	2.51E-13	chr:6:32648036
6	rs281875005	32631266	G	2.502	2.52E-13	chr:6:32648036
6	rs9272515	32606406	A	2.635	2.54E-13	chr:6:32648036
6	rs17612719	32615990	C	2.233	2.55E-13	chr:6:32648036
6	rs28383376	32607219	A	5.179	2.61E-13	chr:6:32648036
6	rs550204266	32615268	C	2.217	2.64E-13	chr:6:32648036
6	rs114308959	32599445	G	4.489	2.65E-13	chr:6:32648036
6	rs115290336	32605107	G	4.712	2.68E-13	chr:6:32648036
6	rs17612712	32615945	T	2.22	2.72E-13	chr:6:32648036
6	rs9271892	32595813	C	0.3032	2.72E-13	chr:6:32648036
6	rs3208105	32609222	T	2.651	2.75E-13	chr:6:32648036
6	rs28383455	32609380	T	4.246	2.77E-13	chr:6:32648036
6	rs17612597	32615582	C	2.33	2.79E-13	chr:6:32648036
6	rs9273136	32612823	C	2.346	2.82E-13	chr:6:32648036

6	rs9273137	32612827	A	2.346	2.82E-13	chr:6:32648036
6	rs1018434	32281360	C	3.544	2.83E-13	chr:6:32648036
6	rs9273410	32627250	A	2.538	2.85E-13	chr:6:32648036
6	rs9273343	32623445	C	2.243	2.86E-13	chr:6:32648036
6	rs9271896	32595907	A	0.3029	2.87E-13	chr:6:32648036
6	rs7747236	32502399	T	5.153	2.88E-13	chr:6:32648036
6	rs111247469	32503071	T	4.109	2.91E-13	chr:6:32648036
6	rs9272359	32604544	T	2.459	2.92E-13	chr:6:32648036
6	rs3096669	32236608	T	3.546	2.93E-13	chr:6:32648036
6	rs9271890	32595779	A	0.3043	2.97E-13	chr:6:32648036
6	rs9271891	32595783	T	0.3043	2.97E-13	chr:6:32648036
6	rs281875010	32631261	T	2.499	2.98E-13	chr:6:32648036
6	rs9271383	32587391	C	0.3087	2.99E-13	chr:6:32648036
6	rs17613153	32621505	G	2.173	3.05E-13	chr:6:32648036
6	rs17613167	32621513	T	2.173	3.05E-13	chr:6:32648036
6	rs17613174	32621515	A	2.173	3.05E-13	chr:6:32648036
6	rs17843610	32620529	C	2.295	3.08E-13	chr:6:32648036
6	rs17843611	32620531	C	2.295	3.08E-13	chr:6:32648036
6	rs9273287	32614495	G	2.163	3.10E-13	chr:6:32648036
6	rs9272028	32598706	T	0.3042	3.10E-13	chr:6:32648036
6	rs9272029	32598707	G	0.3042	3.10E-13	chr:6:32648036
6	rs9273560	32628996	A	2.321	3.10E-13	chr:6:32648036
6	rs9273225	32613817	G	2.21	3.11E-13	chr:6:32648036
6	rs9273226	32613824	T	2.21	3.11E-13	chr:6:32648036
6	rs17613326	32621949	C	2.169	3.15E-13	chr:6:32648036
6	rs17843683	32621947	C	2.169	3.15E-13	chr:6:32648036
6	rs17843684	32621948	A	2.169	3.15E-13	chr:6:32648036
6	rs9274529	32634514	G	2.714	3.16E-13	chr:6:32648036
6	rs9273482	32628127	C	2.798	3.18E-13	chr:6:32648036
6	rs9272625	32608014	C	2.647	3.20E-13	chr:6:32648036
6	rs192482979	32643386	G	4.616	3.22E-13	chr:6:32648036
6	rs9271914	32596238	C	0.3087	3.26E-13	chr:6:32648036
6	rs17612676	32615841	A	2.25	3.30E-13	chr:6:32648036
6	rs17843589	32615843	A	2.25	3.30E-13	chr:6:32648036
6	rs1130156	32610968	G	3.719	3.31E-13	chr:6:32648036
6	rs115368651	32602040	G	5.147	3.32E-13	chr:6:32648036
6	rs115170333	32552322	C	4.459	3.32E-13	chr:6:32648036
6	rs566656642	32552339	A	4.459	3.32E-13	chr:6:32648036
6	rs9273124	32612752	G	2.329	3.33E-13	chr:6:32648036
6	rs9273125	32612754	G	2.329	3.33E-13	chr:6:32648036
6	rs1130155	32610951	G	2.225	3.34E-13	chr:6:32648036
6	rs17613243	32621683	G	2.158	3.34E-13	chr:6:32648036
6	rs17843671	32621682	T	2.158	3.34E-13	chr:6:32648036

6	rs17613250	32621699	C	2.162	3.36E-13	chr:6:32648036
6	rs28720766	32595063	T	4.023	3.42E-13	chr:6:32648036
6	rs9272061	32599250	A	0.3052	3.44E-13	chr:6:32648036
6	rs9272593	32607558	G	2.395	3.45E-13	chr:6:32648036
6	rs9272594	32607561	T	2.395	3.45E-13	chr:6:32648036
6	rs9271889	32595747	A	0.3051	3.46E-13	chr:6:32648036
6	rs9271939	32596921	C	0.3061	3.46E-13	chr:6:32648036
6	rs2858328	32658911	C	0.2643	3.47E-13	chr:6:32648036
6	rs6905282	32626015	A	3.026	3.51E-13	chr:6:32648036
6	rs9273358	32626019	T	3.026	3.51E-13	chr:6:32648036
6	rs9272143	32600803	C	0.313	3.54E-13	chr:6:32648036
6	rs2515895	32628960	C	3.244	3.59E-13	chr:6:32648036
6	rs17613395	32622167	A	2.156	3.61E-13	chr:6:32648036
6	rs17843694	32622173	C	2.156	3.61E-13	chr:6:32648036
6	rs9270910	32572180	G	0.3016	3.65E-13	chr:6:32648036
6	rs558095162	32598504	T	0.3126	3.74E-13	chr:6:32648036
6	rs9273338	32623393	T	2.228	3.77E-13	chr:6:32648036
6	rs2454138	32570401	A	3.604	3.77E-13	chr:6:32648036
6	rs2516049	32570400	C	3.604	3.77E-13	chr:6:32648036
6	rs17613181	32621550	G	2.167	3.78E-13	chr:6:32648036
6	rs17843661	32621526	C	2.167	3.78E-13	chr:6:32648036
6	rs17843662	32621529	A	2.167	3.78E-13	chr:6:32648036
6	rs9273234	32613954	G	2.204	3.80E-13	chr:6:32648036
6	rs139025848	32613234	T	4.61	3.80E-13	chr:6:32648036
6	rs526784	32572030	A	3.602	3.81E-13	chr:6:32648036
6	rs112443719	32884252	A	5.705	3.81E-13	chr:6:32648036
6	rs112492913	32872978	T	5.705	3.81E-13	chr:6:32648036
6	rs76041302	32880374	T	5.705	3.81E-13	chr:6:32648036
6	rs78906256	32881325	A	5.705	3.81E-13	chr:6:32648036
6	rs9272013	32598224	T	0.306	3.83E-13	chr:6:32648036
6	rs9271897	32595954	A	0.3058	3.84E-13	chr:6:32648036
6	rs9271898	32595972	A	0.3058	3.84E-13	chr:6:32648036
6	rs9273399	32627117	A	2.318	3.87E-13	chr:6:32648036
6	rs9274957	32641914	C	2.439	3.90E-13	chr:6:32648036
6	rs9271404	32587553	A	3.373	3.95E-13	chr:6:32648036
6	rs17612878	32620733	T	2.226	3.96E-13	chr:6:32648036
6	rs114354029	32598764	G	0.3139	4.00E-13	chr:6:32648036
6	rs9268202	32279340	T	3.489	4.10E-13	chr:6:32648036
6	rs1142328	32609216	T	2.63	4.12E-13	chr:6:32648036
6	rs9273235	32613957	A	2.202	4.14E-13	chr:6:32648036
6	rs9273253	32614207	G	2.179	4.17E-13	chr:6:32648036
6	rs3021302	32623150	C	4	4.22E-13	chr:6:32648036
6	rs7142	32610825	G	2.366	4.24E-13	chr:6:32648036

6	rs9272422	32605132	C	3.411	4.26E-13	chr:6:32648036
6	rs9271918	32596352	T	0.306	4.28E-13	chr:6:32648036
6	rs28383277	32586300	T	6.049	4.28E-13	chr:6:32648036
6	rs9273290	32614552	T	2.148	4.30E-13	chr:6:32648036
6	rs9273291	32614555	C	2.148	4.30E-13	chr:6:32648036
6	rs544749594	32605025	G	5.168	4.35E-13	chr:6:32648036
6	rs17612781	32620311	C	2.513	4.36E-13	chr:6:32648036
6	rs371989651	32598510	C	3.688	4.37E-13	chr:6:32648036
6	rs9272775	32610257	C	3.34	4.37E-13	chr:6:32648036
6	rs9273214	32613703	T	2.164	4.43E-13	chr:6:32648036
6	rs9271906	32596048	A	0.3086	4.46E-13	chr:6:32648036
6	rs115682612	32848897	T	5.568	4.46E-13	chr:6:32648036
6	rs112313206	32598405	T	0.3132	4.47E-13	chr:6:32648036
6	rs74606007	32603973	C	4.739	4.47E-13	chr:6:32648036
6	rs9271699	32593179	G	0.3128	4.55E-13	chr:6:32648036
6	rs9273348	32623487	G	2.256	4.56E-13	chr:6:32648036
6	rs3129714	32657279	A	3.105	4.58E-13	chr:6:32648036
6	rs115908946	32600615	C	4.347	4.61E-13	chr:6:32648036
6	rs559861992	32603447	T	4.428	4.62E-13	chr:6:32648036
6	rs9274557	32634887	T	2.382	4.66E-13	chr:6:32648036
6	rs9273241	32614025	T	2.374	4.66E-13	chr:6:32648036
6	rs9272130	32600638	G	0.3094	4.67E-13	chr:6:32648036
6	rs9274664	32636528	G	2.922	4.76E-13	chr:6:32648036
6	rs9270911	32572202	C	0.3024	4.78E-13	chr:6:32648036
6	rs9274573	32635205	T	2.628	4.83E-13	chr:6:32648036
6	rs4538748	32657505	C	3.103	4.97E-13	chr:6:32648036
6	rs9272109	32600253	T	0.3131	5.00E-13	chr:6:32648036
6	rs17612887	32620739	T	2.213	5.01E-13	chr:6:32648036
6	rs115738641	32599980	G	4.358	5.02E-13	chr:6:32648036
6	rs28755177	32641412	A	4.623	5.06E-13	chr:6:32648036
6	rs9271888	32595732	C	0.3085	5.08E-13	chr:6:32648036
6	rs17613271	32621778	G	2.29	5.12E-13	chr:6:32648036
6	rs111618128	32830426	T	5.603	5.12E-13	chr:6:32648036
6	rs115273200	32826794	A	5.603	5.12E-13	chr:6:32648036
6	rs9271753	32593901	C	2.333	5.17E-13	chr:6:32648036
6	rs9271754	32593913	G	2.333	5.17E-13	chr:6:32648036
6	rs9272103	32599896	T	0.3142	5.21E-13	chr:6:32648036
6	rs9273236	32613973	C	2.197	5.23E-13	chr:6:32648036
6	rs9273237	32613976	T	2.197	5.23E-13	chr:6:32648036
6	rs115616896	32862370	C	5.605	5.25E-13	chr:6:32648036
6	rs149631651	32853458	A	5.605	5.25E-13	chr:6:32648036
6	rs76436617	32849339	C	5.605	5.25E-13	chr:6:32648036
6	rs78183691	32848569	C	5.605	5.25E-13	chr:6:32648036

6	rs146650659	32646807	G	3.947	5.28E-13	chr:6:32648036
6	rs9272026	32598618	G	0.3087	5.33E-13	chr:6:32648036
6	rs9272027	32598622	G	0.3087	5.33E-13	chr:6:32648036
6	rs114307375	32597081	G	4.066	5.36E-13	chr:6:32648036
6	rs115350662	32597078	T	4.066	5.36E-13	chr:6:32648036
6	rs115016190	32600959	C	4.104	5.37E-13	chr:6:32648036
6	rs9271387	32587424	C	0.3185	5.39E-13	chr:6:32648036
6	rs78692977	32601164	A	3.702	5.39E-13	chr:6:32648036
6	rs114088880	32600279	G	4.359	5.46E-13	chr:6:32648036
6	rs9273398	32627115	T	2.301	5.46E-13	chr:6:32648036
6	rs9271917	32596336	G	0.3085	5.46E-13	chr:6:32648036
6	rs17612741	32616036	G	2.178	5.46E-13	chr:6:32648036
6	rs9273346	32623475	G	2.252	5.48E-13	chr:6:32648036
6	rs9272141	32600797	A	0.3169	5.56E-13	chr:6:32648036
6	rs114968544	32596711	C	4.321	5.66E-13	chr:6:32648036
6	rs9501608	32201803	C	4.629	5.68E-13	chr:6:32648036
6	rs9273160	32613104	G	2.255	5.69E-13	chr:6:32648036
6	rs1018433	32281510	T	3.469	5.75E-13	chr:6:32648036
6	rs111257207	32226126	C	3.469	5.75E-13	chr:6:32648036
6	rs112845108	32226352	T	3.469	5.75E-13	chr:6:32648036
6	rs140566858	32226520	G	3.469	5.75E-13	chr:6:32648036
6	rs142071717	32226256	G	3.469	5.75E-13	chr:6:32648036
6	rs1559873	32243129	C	3.469	5.75E-13	chr:6:32648036
6	rs2069026	32281958	T	3.469	5.75E-13	chr:6:32648036
6	rs281863610	32630794	G	2.482	5.75E-13	chr:6:32648036
6	rs28367008	32283214	T	3.469	5.75E-13	chr:6:32648036
6	rs3096672	32237748	A	3.469	5.75E-13	chr:6:32648036
6	rs3096674	32238219	A	3.469	5.75E-13	chr:6:32648036
6	rs3096677	32239050	C	3.469	5.75E-13	chr:6:32648036
6	rs3096678	32239314	A	3.469	5.75E-13	chr:6:32648036
6	rs3115557	32239651	T	3.469	5.75E-13	chr:6:32648036
6	rs3115558	32239427	A	3.469	5.75E-13	chr:6:32648036
6	rs3115565	32230344	A	3.469	5.75E-13	chr:6:32648036
6	rs3130331	32231483	T	3.469	5.75E-13	chr:6:32648036
6	rs3130336	32238428	T	3.469	5.75E-13	chr:6:32648036
6	rs3130337	32241203	A	3.469	5.75E-13	chr:6:32648036
6	rs3132927	32231407	A	3.469	5.75E-13	chr:6:32648036
6	rs3132931	32235895	G	3.469	5.75E-13	chr:6:32648036
6	rs3864299	32271674	T	3.469	5.75E-13	chr:6:32648036
6	rs3864300	32271807	T	3.469	5.75E-13	chr:6:32648036
6	rs3864301	32271955	T	3.469	5.75E-13	chr:6:32648036
6	rs3864303	32281621	G	3.469	5.75E-13	chr:6:32648036
6	rs6457536	32273765	G	3.469	5.75E-13	chr:6:32648036

6	rs6910275	32283114	A	3.469	5.75E-13	chr:6:32648036
6	rs6915455	32283494	A	3.469	5.75E-13	chr:6:32648036
6	rs6915879	32273198	T	3.469	5.75E-13	chr:6:32648036
6	rs6927529	32258088	G	3.469	5.75E-13	chr:6:32648036
6	rs6927856	32258043	A	3.469	5.75E-13	chr:6:32648036
6	rs6934429	32279622	T	3.469	5.75E-13	chr:6:32648036
6	rs6934546	32279952	G	3.469	5.75E-13	chr:6:32648036
6	rs6934776	32279816	A	3.469	5.75E-13	chr:6:32648036
6	rs6939410	32280182	G	3.469	5.75E-13	chr:6:32648036
6	rs7341270	32273487	A	3.469	5.75E-13	chr:6:32648036
6	rs7742654	32271443	G	3.469	5.75E-13	chr:6:32648036
6	rs7757711	32256807	T	3.469	5.75E-13	chr:6:32648036
6	rs7758030	32257035	T	3.469	5.75E-13	chr:6:32648036
6	rs9268013	32224828	C	3.469	5.75E-13	chr:6:32648036
6	rs9268059	32230678	T	3.469	5.75E-13	chr:6:32648036
6	rs9268116	32249177	G	3.469	5.75E-13	chr:6:32648036
6	rs9268117	32249524	C	3.469	5.75E-13	chr:6:32648036
6	rs9268121	32251435	C	3.469	5.75E-13	chr:6:32648036
6	rs9268125	32252678	C	3.469	5.75E-13	chr:6:32648036
6	rs9268131	32254452	G	3.469	5.75E-13	chr:6:32648036
6	rs9268137	32255269	A	3.469	5.75E-13	chr:6:32648036
6	rs9268140	32255594	C	3.469	5.75E-13	chr:6:32648036
6	rs9268163	32270875	T	3.469	5.75E-13	chr:6:32648036
6	rs9268165	32272327	G	3.469	5.75E-13	chr:6:32648036
6	rs9268168	32272510	T	3.469	5.75E-13	chr:6:32648036
6	rs9268169	32272707	G	3.469	5.75E-13	chr:6:32648036
6	rs9268170	32272711	G	3.469	5.75E-13	chr:6:32648036
6	rs9268172	32273085	T	3.469	5.75E-13	chr:6:32648036
6	rs9268175	32274067	A	3.469	5.75E-13	chr:6:32648036
6	rs9268176	32274079	T	3.469	5.75E-13	chr:6:32648036
6	rs9268179	32275629	T	3.469	5.75E-13	chr:6:32648036
6	rs9268180	32275841	T	3.469	5.75E-13	chr:6:32648036
6	rs9268181	32276009	C	3.469	5.75E-13	chr:6:32648036
6	rs9268182	32276027	G	3.469	5.75E-13	chr:6:32648036
6	rs9268183	32276172	A	3.469	5.75E-13	chr:6:32648036
6	rs9268187	32276549	C	3.469	5.75E-13	chr:6:32648036
6	rs9268188	32276735	C	3.469	5.75E-13	chr:6:32648036
6	rs9268190	32276795	T	3.469	5.75E-13	chr:6:32648036
6	rs9268191	32277025	A	3.469	5.75E-13	chr:6:32648036
6	rs9268192	32277211	T	3.469	5.75E-13	chr:6:32648036
6	rs9268194	32277548	A	3.469	5.75E-13	chr:6:32648036
6	rs9268195	32277555	A	3.469	5.75E-13	chr:6:32648036
6	rs9268196	32277834	T	3.469	5.75E-13	chr:6:32648036

6	rs9268197	32277934	A	3.469	5.75E-13	chr:6:32648036
6	rs9268198	32278187	T	3.469	5.75E-13	chr:6:32648036
6	rs9268204	32279500	T	3.469	5.75E-13	chr:6:32648036
6	rs9268207	32280462	T	3.469	5.75E-13	chr:6:32648036
6	rs9268212	32281889	C	3.469	5.75E-13	chr:6:32648036
6	rs9268213	32282081	G	3.469	5.75E-13	chr:6:32648036
6	rs9268214	32282466	G	3.469	5.75E-13	chr:6:32648036
6	rs9268215	32282471	G	3.469	5.75E-13	chr:6:32648036
6	rs9268218	32283699	G	3.469	5.75E-13	chr:6:32648036
6	rs9268222	32285228	A	3.469	5.75E-13	chr:6:32648036
6	rs1130152	32610946	T	2.202	5.75E-13	chr:6:32648036
6	rs17612865	32620723	T	2.214	5.92E-13	chr:6:32648036
6	rs17612872	32620726	C	2.214	5.92E-13	chr:6:32648036
6	rs17843681	32621928	A	2.153	5.94E-13	chr:6:32648036
6	rs17843682	32621932	C	2.153	5.94E-13	chr:6:32648036
6	rs112008201	32884838	G	5.572	5.94E-13	chr:6:32648036
6	rs17612610	32615611	T	2.31	5.96E-13	chr:6:32648036
6	rs17612617	32615613	T	2.31	5.96E-13	chr:6:32648036
6	rs111477174	32594457	G	0.321	6.23E-13	chr:6:32648036
6	rs111634228	32594452	G	0.321	6.23E-13	chr:6:32648036
6	rs9272110	32600287	C	0.3138	6.35E-13	chr:6:32648036
6	rs9272113	32600291	C	0.3138	6.35E-13	chr:6:32648036
6	rs9273215	32613712	A	2.167	6.41E-13	chr:6:32648036
6	rs17613299	32621901	G	2.156	6.50E-13	chr:6:32648036
6	rs28383270	32586024	G	4.838	6.52E-13	chr:6:32648036
6	rs116135263	32517913	A	6.464	6.53E-13	chr:6:32648036
6	rs28371173	32612057	A	7.271	6.53E-13	chr:6:32648036
6	rs9271899	32596007	G	0.3109	6.53E-13	chr:6:32648036
6	rs9271901	32596027	T	0.3109	6.53E-13	chr:6:32648036
6	rs17612604	32615597	C	2.296	6.53E-13	chr:6:32648036
6	rs17843618	32620743	T	2.206	6.54E-13	chr:6:32648036
6	rs9272330	32604213	C	0.3435	6.56E-13	chr:6:32648036
6	rs9275059	32648298	A	2.898	6.69E-13	chr:6:32648036
6	rs9273294	32614575	A	2.131	6.84E-13	chr:6:32648036
6	rs9271915	32596294	C	0.3125	6.86E-13	chr:6:32648036
6	rs3096684	32234345	T	3.45	6.88E-13	chr:6:32648036
6	rs1063346	32627967	G	2.576	6.93E-13	chr:6:32648036
6	rs3204428	32627969	T	2.576	6.93E-13	chr:6:32648036
6	rs9271857	32595147	T	0.3204	6.95E-13	chr:6:32648036
6	rs9272719	32609387	A	2.285	7.00E-13	chr:6:32648036
6	rs9272720	32609389	T	2.285	7.00E-13	chr:6:32648036
6	rs9268096	32240611	G	3.451	7.00E-13	chr:6:32648036
6	rs4713589	32659435	G	0.2742	7.01E-13	chr:6:32648036

6	rs28383275	32586281	G	5.809	7.14E-13	chr:6:32648036
6	rs28383276	32586282	G	5.809	7.14E-13	chr:6:32648036
6	rs9271948	32597105	A	0.3113	7.14E-13	chr:6:32648036
6	rs9268135	32255230	G	3.442	7.16E-13	chr:6:32648036
6	rs9273213	32613696	A	2.159	7.16E-13	chr:6:32648036
6	rs3096687	32230056	T	3.448	7.19E-13	chr:6:32648036
6	rs9271960	32597385	T	0.3193	7.34E-13	chr:6:32648036
6	rs9273325	32623193	A	3.801	7.36E-13	chr:6:32648036
6	rs9273479	32628093	C	2.653	7.38E-13	chr:6:32648036
6	rs28534011	32602947	A	4.505	7.54E-13	chr:6:32648036
6	rs17205970	32651923	C	8.069	7.58E-13	chr:6:32648036
6	rs553340154	32525778	G	2.985	7.68E-13	chr:6:32648036
6	rs9268184	32276203	A	3.453	7.72E-13	chr:6:32648036
6	rs9273232	32613944	T	2.187	7.72E-13	chr:6:32648036
6	rs9273163	32613114	T	2.243	7.84E-13	chr:6:32648036
6	rs116573360	32598366	G	4.037	8.00E-13	chr:6:32648036
6	rs147493459	32598335	G	4.037	8.00E-13	chr:6:32648036
6	rs9271934	32596773	T	0.3151	8.13E-13	chr:6:32648036
6	rs3132951	32247315	T	3.427	8.13E-13	chr:6:32648036
6	rs8227	32610757	A	0.3366	8.19E-13	chr:6:32648036
6	rs9273337	32623377	T	2.205	8.30E-13	chr:6:32648036
6	rs9271935	32596831	C	0.3156	8.37E-13	chr:6:32648036
6	rs9273483	32628138	A	2.8	8.43E-13	chr:6:32648036
6	rs9276498	32719081	A	3.151	8.45E-13	chr:6:32648036
6	rs7754119	32449858	C	4.333	8.50E-13	chr:6:32648036
6	rs116109088	32599900	T	3.622	8.60E-13	chr:6:32648036
6	rs9271756	32593933	G	2.307	8.62E-13	chr:6:32648036
6	rs113192265	32511884	C	5.502	8.74E-13	chr:6:32648036
6	rs148437409	32598479	C	0.3225	8.79E-13	chr:6:32648036
6	rs17843679	32621888	G	2.148	8.97E-13	chr:6:32648036
6	rs17843680	32621893	G	2.148	8.97E-13	chr:6:32648036
6	rs9271959	32597377	C	0.3207	9.00E-13	chr:6:32648036
6	rs9273078	32612309	A	2.226	9.00E-13	chr:6:32648036
6	rs114895321	32603154	G	4.551	9.12E-13	chr:6:32648036
6	rs9271920	32596430	G	0.3106	9.15E-13	chr:6:32648036
6	rs9271921	32596431	G	0.3106	9.15E-13	chr:6:32648036
6	rs9274536	32634634	T	2.546	9.18E-13	chr:6:32648036
6	rs9272022	32598529	G	0.3222	9.19E-13	chr:6:32648036
6	rs17843567	32614866	G	2.137	9.19E-13	chr:6:32648036
6	rs9271739	32593692	T	0.3057	9.23E-13	chr:6:32648036
6	rs28755168	32621793	T	2.426	9.24E-13	chr:6:32648036
6	rs115222936	32605039	T	4.987	9.25E-13	chr:6:32648036
6	rs3130321	32223418	T	3.424	9.29E-13	chr:6:32648036

6	rs3132966	32223632	A	3.424	9.29E-13	chr:6:32648036
6	rs17843595	32615971	A	2.176	9.30E-13	chr:6:32648036
6	rs17843596	32615972	A	2.176	9.30E-13	chr:6:32648036
6	rs1130116	32610535	A	0.3516	9.40E-13	chr:6:32648036
6	rs9271927	32596647	C	0.3169	9.43E-13	chr:6:32648036
6	rs9271928	32596648	A	0.3169	9.43E-13	chr:6:32648036
6	rs17612510	32614883	T	2.136	9.46E-13	chr:6:32648036
6	rs17613257	32621726	T	2.13	9.47E-13	chr:6:32648036
6	rs17613264	32621727	G	2.13	9.47E-13	chr:6:32648036
6	rs9272009	32598063	T	0.315	9.52E-13	chr:6:32648036
6	rs9273123	32612733	A	2.293	9.83E-13	chr:6:32648036
6	rs115544552	32711318	T	4.715	9.86E-13	chr:6:32648036
6	rs17843621	32620827	T	2.166	9.87E-13	chr:6:32648036
6	rs7753228	32503794	T	4.838	9.89E-13	chr:6:32648036
6	rs9273231	32613937	A	2.174	9.94E-13	chr:6:32648036
6	rs9273288	32614511	A	2.129	1.01E-12	chr:6:32648036
6	rs9271937	32596870	G	0.3159	1.01E-12	chr:6:32648036
6	rs9271663	32592636	A	0.3143	1.01E-12	chr:6:32648036
6	rs28752526	32584918	G	3.22	1.01E-12	chr:6:32648036
6	rs7750783	32268080	T	3.404	1.02E-12	chr:6:32648036
6	rs76822178	32601188	T	3.593	1.03E-12	chr:6:32648036
6	rs9271755	32593926	C	2.303	1.04E-12	chr:6:32648036
6	rs9273065	32612205	G	2.356	1.04E-12	chr:6:32648036
6	rs9272076	32599624	T	0.2923	1.05E-12	chr:6:32648036
6	rs9272077	32599625	G	0.2923	1.05E-12	chr:6:32648036
6	rs1063343	32627992	C	2.593	1.07E-12	chr:6:32648036
6	rs74824383	32624731	G	2.118	1.07E-12	chr:6:32648036
6	rs9274543	32634695	A	2.423	1.09E-12	chr:6:32648036
6	rs9271955	32597281	A	0.3161	1.09E-12	chr:6:32648036
6	rs7341300	32275027	T	3.402	1.11E-12	chr:6:32648036
6	rs7341328	32275194	A	3.402	1.11E-12	chr:6:32648036
6	rs17613305	32621908	C	2.135	1.11E-12	chr:6:32648036
6	rs17613312	32621911	A	2.135	1.11E-12	chr:6:32648036
6	rs9271403	32587536	G	3.274	1.11E-12	chr:6:32648036
6	rs28383312	32587090	T	3.295	1.12E-12	chr:6:32648036
6	rs9272119	32600470	C	0.3175	1.13E-12	chr:6:32648036
6	rs9271930	32596677	G	0.318	1.13E-12	chr:6:32648036
6	rs17843619	32620775	A	2.182	1.14E-12	chr:6:32648036
6	rs1559874	32243155	A	3.395	1.15E-12	chr:6:32648036
6	rs9273081	32612336	A	2.221	1.15E-12	chr:6:32648036
6	rs9268166	32272433	C	3.399	1.15E-12	chr:6:32648036
6	rs9274555	32634882	T	2.33	1.16E-12	chr:6:32648036
6	rs9274556	32634883	T	2.33	1.16E-12	chr:6:32648036

6	rs9271961	32597412	A	0.3215	1.16E-12	chr:6:32648036
6	rs17843564	32614842	G	2.125	1.17E-12	chr:6:32648036
6	rs17843565	32614847	G	2.125	1.17E-12	chr:6:32648036
6	rs113011827	32513453	T	5.532	1.18E-12	chr:6:32648036
6	rs113568276	32513127	A	5.532	1.18E-12	chr:6:32648036
6	rs9273179	32613305	G	2.323	1.18E-12	chr:6:32648036
6	rs2395255	32721639	C	3.112	1.18E-12	chr:6:32648036
6	rs3667	32610934	G	3.527	1.20E-12	chr:6:32648036
6	rs9272134	32600705	G	0.3212	1.22E-12	chr:6:32648036
6	rs9272016	32598346	T	0.3199	1.22E-12	chr:6:32648036
6	rs9273121	32612725	T	2.266	1.24E-12	chr:6:32648036
6	rs9273122	32612726	G	2.266	1.24E-12	chr:6:32648036
6	rs12192620	32674318	A	3.056	1.25E-12	chr:6:32648036
6	rs28371271	32674343	A	3.056	1.25E-12	chr:6:32648036
6	rs17843676	32621759	G	2.156	1.27E-12	chr:6:32648036
6	rs75237145	32594295	T	0.3296	1.30E-12	chr:6:32648036
6	rs9272618	32607927	T	2.48	1.34E-12	chr:6:32648036
6	rs2114435	32242838	G	3.316	1.34E-12	chr:6:32648036
6	rs372332783	32603074	T	4.504	1.34E-12	chr:6:32648036
6	rs1065047	32611260	G	3.233	1.35E-12	chr:6:32648036
6	rs1065048	32611265	T	3.233	1.35E-12	chr:6:32648036
6	rs1063345	32627976	A	2.587	1.35E-12	chr:6:32648036
6	rs534490668	32433399	A	0.317	1.36E-12	chr:6:32648036
6	rs6931740	32647469	G	0.3681	1.37E-12	chr:6:32648036
6	rs9273082	32612339	T	2.216	1.38E-12	chr:6:32648036
6	rs536081339	32600597	T	4.044	1.38E-12	chr:6:32648036
6	rs9276448	32715629	G	3.137	1.39E-12	chr:6:32648036
6	rs116707510	32605552	C	4.881	1.39E-12	chr:6:32648036
6	rs17612893	32620836	T	2.148	1.42E-12	chr:6:32648036
6	rs9272031	32598779	C	0.3263	1.43E-12	chr:6:32648036
6	rs12722069	32609231	G	4.097	1.44E-12	chr:6:32648036
6	rs3207985	32609227	C	4.097	1.44E-12	chr:6:32648036
6	rs563316064	32602809	C	4.323	1.47E-12	chr:6:32648036
6	rs575430623	32602810	T	4.323	1.47E-12	chr:6:32648036
6	rs9273218	32613756	A	2.16	1.51E-12	chr:6:32648036
6	rs9273220	32613759	T	2.16	1.51E-12	chr:6:32648036
6	rs9274544	32634699	A	2.383	1.52E-12	chr:6:32648036
6	rs9274545	32634702	C	2.383	1.52E-12	chr:6:32648036
6	rs12527746	32620237	A	3.942	1.54E-12	chr:6:32648036
6	rs114598250	32605458	T	4.727	1.54E-12	chr:6:32648036
6	rs567798325	32605450	C	4.727	1.54E-12	chr:6:32648036
6	rs9273066	32612208	A	2.331	1.55E-12	chr:6:32648036
6	rs9271929	32596650	C	0.3151	1.57E-12	chr:6:32648036

6	rs9274554	32634870	G	2.335	1.60E-12	chr:6:32648036
6	rs9272798	32610574	T	2.456	1.60E-12	chr:6:32648036
6	rs9272799	32610576	T	2.456	1.60E-12	chr:6:32648036
6	rs9272800	32610581	G	2.456	1.60E-12	chr:6:32648036
6	rs9272801	32610584	C	2.456	1.60E-12	chr:6:32648036
6	rs28383269	32585995	C	4.697	1.63E-12	chr:6:32648036
6	rs281863631	32630761	T	2.353	1.64E-12	chr:6:32648036
6	rs367728179	32600771	G	3.813	1.66E-12	chr:6:32648036
6	rs9271957	32597335	C	0.3202	1.69E-12	chr:6:32648036
6	rs28362367	32590364	T	4.02	1.70E-12	chr:6:32648036
6	rs116230912	32601690	G	4.468	1.71E-12	chr:6:32648036
6	rs9271854	32595118	A	0.3293	1.72E-12	chr:6:32648036
6	rs9271855	32595119	C	0.3293	1.72E-12	chr:6:32648036
6	rs9271856	32595120	A	0.3293	1.72E-12	chr:6:32648036
6	rs139455771	32631775	T	4.323	1.75E-12	chr:6:32648036
6	rs9272032	32598782	G	0.3286	1.80E-12	chr:6:32648036
6	rs146406015	32598870	T	4.021	1.83E-12	chr:6:32648036
6	rs9272135	32600723	A	0.3249	1.84E-12	chr:6:32648036
6	rs9271752	32593868	C	2.274	1.85E-12	chr:6:32648036
6	rs9269079	32440910	T	0.3162	1.94E-12	chr:6:32648036
6	rs9271951	32597172	A	0.3271	1.94E-12	chr:6:32648036
6	rs9273083	32612343	C	2.202	1.94E-12	chr:6:32648036
6	rs58879393	32903733	T	5.584	1.95E-12	chr:6:32648036
6	rs116607676	32604139	A	4.591	1.96E-12	chr:6:32648036
6	rs9276461	32716167	A	3.062	1.97E-12	chr:6:32648036
6	rs9276462	32716170	C	3.062	1.97E-12	chr:6:32648036
6	rs9276463	32716181	C	3.062	1.97E-12	chr:6:32648036
6	rs9276464	32716294	T	3.062	1.97E-12	chr:6:32648036
6	rs9276465	32716409	G	3.062	1.97E-12	chr:6:32648036
6	rs9271932	32596714	C	0.3268	1.99E-12	chr:6:32648036
6	rs9271933	32596717	A	0.3268	1.99E-12	chr:6:32648036
6	rs9273310	32622954	A	2.633	2.00E-12	chr:6:32648036
6	rs4642463	32721783	T	3.07	2.03E-12	chr:6:32648036
6	rs763028	32721484	A	3.07	2.03E-12	chr:6:32648036
6	rs763029	32721174	C	3.07	2.03E-12	chr:6:32648036
6	rs7757475	32724499	C	3.07	2.03E-12	chr:6:32648036
6	rs7774929	32724147	A	3.07	2.03E-12	chr:6:32648036
6	rs7774954	32724189	A	3.07	2.03E-12	chr:6:32648036
6	rs7774973	32724267	G	3.07	2.03E-12	chr:6:32648036
6	rs7775664	32724447	C	3.07	2.03E-12	chr:6:32648036
6	rs9276545	32722020	T	3.07	2.03E-12	chr:6:32648036
6	rs9276554	32722702	T	3.07	2.03E-12	chr:6:32648036
6	rs9273397	32627109	G	2.275	2.04E-12	chr:6:32648036

6	rs9273187	32613376	T	4.035	2.07E-12	chr:6:32648036
6	rs17843603	32620241	A	2.415	2.08E-12	chr:6:32648036
6	rs77639852	32601094	T	3.734	2.11E-12	chr:6:32648036
6	rs142901825	32713602	C	7.792	2.14E-12	chr:6:32648036
6	rs80235202	32503831	G	4.291	2.15E-12	chr:6:32648036
6	rs187142834	32643333	A	4.22	2.17E-12	chr:6:32648036
6	rs28383267	32585978	G	4.703	2.20E-12	chr:6:32648036
6	rs375608727	32608735	G	4.243	2.22E-12	chr:6:32648036
6	rs7453649	32440160	T	0.3125	2.27E-12	chr:6:32648036
6	rs9271720	32593507	A	0.3094	2.30E-12	chr:6:32648036
6	rs9272007	32598038	T	0.3252	2.31E-12	chr:6:32648036
6	rs9272008	32598042	A	0.3252	2.31E-12	chr:6:32648036
6	rs9273074	32612287	G	2.214	2.31E-12	chr:6:32648036
6	rs9273075	32612288	G	2.214	2.31E-12	chr:6:32648036
6	rs9269109	32443266	T	0.3174	2.34E-12	chr:6:32648036
6	rs117055012	32602628	G	4.358	2.35E-12	chr:6:32648036
6	rs9268881	32431606	T	0.3148	2.36E-12	chr:6:32648036
6	rs9268883	32431638	T	0.3148	2.36E-12	chr:6:32648036
6	rs17612900	32620838	C	2.128	2.40E-12	chr:6:32648036
6	rs9273396	32627105	A	2.27	2.42E-12	chr:6:32648036
6	rs6457530	32248176	T	2.803	2.45E-12	chr:6:32648036
6	rs7757760	32247710	G	2.803	2.45E-12	chr:6:32648036
6	rs7776041	32247771	A	2.803	2.45E-12	chr:6:32648036
6	rs9268109	32247505	T	2.803	2.45E-12	chr:6:32648036
6	rs9275057	32648253	G	2.667	2.54E-12	chr:6:32648036
6	rs9268852	32429594	A	0.3164	2.59E-12	chr:6:32648036
6	rs115717621	32603163	T	4.38	2.61E-12	chr:6:32648036
6	rs185234644	32514792	C	5.995	2.66E-12	chr:6:32648036
6	rs9274583	32635361	A	2.463	2.71E-12	chr:6:32648036
6	rs77701583	32435699	C	0.333	2.74E-12	chr:6:32648036
6	rs4548006	32717604	A	3.047	2.75E-12	chr:6:32648036
6	rs4585553	32716736	G	3.047	2.75E-12	chr:6:32648036
6	rs9276453	32715775	C	3.047	2.75E-12	chr:6:32648036
6	rs9271718	32593465	T	2.737	2.77E-12	chr:6:32648036
6	rs9276488	32718602	G	3.046	2.82E-12	chr:6:32648036
6	rs9276489	32718620	T	3.046	2.82E-12	chr:6:32648036
6	rs9273069	32612233	T	2.211	2.87E-12	chr:6:32648036
6	rs2647011	32661915	C	0.3048	2.88E-12	chr:6:32648036
6	rs9271388	32587426	A	0.304	2.92E-12	chr:6:32648036
6	rs558652356	32608548	A	4.347	2.92E-12	chr:6:32648036
6	rs17612907	32620852	A	2.111	2.99E-12	chr:6:32648036
6	rs7755212	32441408	C	0.318	3.00E-12	chr:6:32648036
6	rs9273156	32613050	A	2.306	3.07E-12	chr:6:32648036

6	rs9273158	32613062	C	2.305	3.15E-12	chr:6:32648036
6	rs527369852	32453191	C	3.112	3.16E-12	chr:6:32648036
6	rs548656751	32601705	T	4.39	3.20E-12	chr:6:32648036
6	rs17843591	32615856	C	2.146	3.23E-12	chr:6:32648036
6	rs9271717	32593453	G	0.33	3.24E-12	chr:6:32648036
6	rs17612683	32615877	G	2.135	3.29E-12	chr:6:32648036
6	rs17612704	32615921	T	2.708	3.32E-12	chr:6:32648036
6	rs9274546	32634736	T	2.374	3.35E-12	chr:6:32648036
6	rs9269042	32438387	C	0.3192	3.35E-12	chr:6:32648036
6	rs9272970	32611301	T	2.894	3.37E-12	chr:6:32648036
6	rs9268209	32280620	C	3.316	3.38E-12	chr:6:32648036
6	rs2022544	32321004	T	4.374	3.38E-12	chr:6:32648036
6	rs2073048	32335433	A	4.374	3.38E-12	chr:6:32648036
6	rs2078290	32312655	A	4.374	3.38E-12	chr:6:32648036
6	rs55967581	32315496	C	4.374	3.38E-12	chr:6:32648036
6	rs6457549	32326844	G	4.374	3.38E-12	chr:6:32648036
6	rs6457553	32330695	G	4.374	3.38E-12	chr:6:32648036
6	rs6907757	32325358	T	4.374	3.38E-12	chr:6:32648036
6	rs7770048	32334754	T	4.374	3.38E-12	chr:6:32648036
6	rs10947251	32261952	A	4.611	3.40E-12	chr:6:32648036
6	rs11751697	32266425	T	4.611	3.40E-12	chr:6:32648036
6	rs11755505	32277479	T	4.611	3.40E-12	chr:6:32648036
6	rs11759575	32269848	T	4.611	3.40E-12	chr:6:32648036
6	rs11759612	32270009	T	4.611	3.40E-12	chr:6:32648036
6	rs28394230	32294069	A	4.611	3.40E-12	chr:6:32648036
6	rs3749967	32283844	C	4.611	3.40E-12	chr:6:32648036
6	rs529358641	32225729	A	4.611	3.40E-12	chr:6:32648036
6	rs9271691	32593006	G	0.3344	3.40E-12	chr:6:32648036
6	rs9271692	32593010	A	0.3344	3.40E-12	chr:6:32648036
6	rs9501173	32279902	A	4.611	3.40E-12	chr:6:32648036
6	rs9501174	32283014	A	4.611	3.40E-12	chr:6:32648036
6	rs9501176	32286944	C	4.611	3.40E-12	chr:6:32648036
6	rs9501178	32292917	C	4.611	3.40E-12	chr:6:32648036
6	rs980605	32289662	T	4.611	3.40E-12	chr:6:32648036
6	rs9268912	32432509	C	0.3193	3.40E-12	chr:6:32648036
6	rs4351242	32717722	T	3.033	3.42E-12	chr:6:32648036
6	rs4370403	32717515	A	3.033	3.42E-12	chr:6:32648036
6	rs4394272	32717771	T	3.033	3.42E-12	chr:6:32648036
6	rs4415152	32717024	C	3.033	3.42E-12	chr:6:32648036
6	rs5014418	32719381	C	3.033	3.42E-12	chr:6:32648036
6	rs5017093	32717339	A	3.033	3.42E-12	chr:6:32648036
6	rs555798526	32718907	T	3.033	3.42E-12	chr:6:32648036
6	rs9276436	32714083	C	3.033	3.42E-12	chr:6:32648036

6	rs9276438	32714516	A	3.033	3.42E-12	chr:6:32648036
6	rs9276439	32714603	T	3.033	3.42E-12	chr:6:32648036
6	rs9276442	32714901	T	3.033	3.42E-12	chr:6:32648036
6	rs9276446	32715448	G	3.033	3.42E-12	chr:6:32648036
6	rs9276449	32715653	T	3.033	3.42E-12	chr:6:32648036
6	rs9276450	32715682	A	3.033	3.42E-12	chr:6:32648036
6	rs9276451	32715685	T	3.033	3.42E-12	chr:6:32648036
6	rs9276452	32715722	G	3.033	3.42E-12	chr:6:32648036
6	rs9276455	32715933	A	3.033	3.42E-12	chr:6:32648036
6	rs9276457	32715993	T	3.033	3.42E-12	chr:6:32648036
6	rs9276458	32716000	A	3.033	3.42E-12	chr:6:32648036
6	rs9276459	32716034	C	3.033	3.42E-12	chr:6:32648036
6	rs9276460	32716127	G	3.033	3.42E-12	chr:6:32648036
6	rs9276472	32717224	G	3.033	3.42E-12	chr:6:32648036
6	rs9276483	32718329	C	3.033	3.42E-12	chr:6:32648036
6	rs115032171	32501241	G	7.121	3.43E-12	chr:6:32648036
6	rs9268932	32433290	T	0.3194	3.56E-12	chr:6:32648036
6	rs17843582	32615651	A	2.37	3.56E-12	chr:6:32648036
6	rs17843583	32615652	C	2.37	3.56E-12	chr:6:32648036
6	rs2858879	32719117	G	3.005	3.56E-12	chr:6:32648036
6	rs9272606	32607729	T	2.501	3.64E-12	chr:6:32648036
6	rs9274584	32635365	A	2.421	3.69E-12	chr:6:32648036
6	rs9273112	32612648	T	2.218	3.72E-12	chr:6:32648036
6	rs2647032	32629905	C	3.713	3.77E-12	chr:6:32648036
6	rs17843581	32615638	C	2.298	3.78E-12	chr:6:32648036
6	rs9271711	32593430	T	0.33	3.78E-12	chr:6:32648036
6	rs9271714	32593434	G	0.33	3.78E-12	chr:6:32648036
6	rs189546613	32601735	C	4.424	3.78E-12	chr:6:32648036
6	rs7739357	32441641	A	0.3207	3.80E-12	chr:6:32648036
6	rs7773030	32441396	G	0.3195	3.80E-12	chr:6:32648036
6	rs9268863	32430289	A	0.3195	3.80E-12	chr:6:32648036
6	rs9268888	32431867	G	0.3195	3.80E-12	chr:6:32648036
6	rs9268979	32435044	T	0.3195	3.80E-12	chr:6:32648036
6	rs9269082	32441178	A	0.3195	3.80E-12	chr:6:32648036
6	rs9269084	32441199	A	0.3195	3.80E-12	chr:6:32648036
6	rs9269086	32441268	G	0.3195	3.80E-12	chr:6:32648036
6	rs7749242	32449030	A	0.2959	3.89E-12	chr:6:32648036
6	rs9271947	32597095	T	0.3199	3.90E-12	chr:6:32648036
6	rs28724238	32629066	C	8.668	3.95E-12	chr:6:32648036
6	rs17612852	32620572	G	0.3135	3.99E-12	chr:6:32648036
6	rs9271653	32592556	C	0.3269	4.02E-12	chr:6:32648036
6	rs113208447	32503314	A	5.261	4.05E-12	chr:6:32648036
6	rs9272004	32597964	A	0.3299	4.12E-12	chr:6:32648036

6	rs4959031	32597411	T	3.558	4.16E-12	chr:6:32648036
6	rs9273113	32612655	G	2.224	4.34E-12	chr:6:32648036
6	rs5014413	32719502	G	2.99	4.35E-12	chr:6:32648036
6	rs9274587	32635377	A	2.429	4.36E-12	chr:6:32648036
6	rs1041885	32412809	A	3.803	4.42E-12	chr:6:32648036
6	rs1051336	32412592	A	3.803	4.42E-12	chr:6:32648036
6	rs1131541	32412566	A	3.803	4.42E-12	chr:6:32648036
6	rs3129884	32410210	C	3.803	4.42E-12	chr:6:32648036
6	rs3129885	32410215	C	3.803	4.42E-12	chr:6:32648036
6	rs3135386	32415426	G	3.803	4.42E-12	chr:6:32648036
6	rs7762077	32416467	A	3.803	4.42E-12	chr:6:32648036
6	rs9272562	32607151	G	2.252	4.43E-12	chr:6:32648036
6	rs9272001	32597901	G	0.3341	4.45E-12	chr:6:32648036
6	rs9273146	32612979	T	2.531	4.50E-12	chr:6:32648036
6	rs9273147	32612981	G	2.531	4.50E-12	chr:6:32648036
6	rs7763192	32338885	G	4.32	4.51E-12	chr:6:32648036
6	rs9276497	32719027	A	2.997	4.52E-12	chr:6:32648036
6	rs17843592	32615863	G	2.129	4.56E-12	chr:6:32648036
6	rs7749057	32448904	G	3.78	4.59E-12	chr:6:32648036
6	rs9272015	32598299	A	0.334	4.62E-12	chr:6:32648036
6	rs9271715	32593441	T	0.333	4.80E-12	chr:6:32648036
6	rs9271716	32593442	G	0.333	4.80E-12	chr:6:32648036
6	rs17612697	32615919	A	2.115	4.85E-12	chr:6:32648036
6	rs148909685	32550776	T	13.99	4.89E-12	chr:6:32648036
6	rs9273230	32613930	A	2.113	4.89E-12	chr:6:32648036
6	rs28362132	32595276	T	3.231	4.96E-12	chr:6:32648036
6	rs9272712	32609341	T	2.251	5.00E-12	chr:6:32648036
6	rs281864453	32628065	G	2.485	5.03E-12	chr:6:32648036
6	rs9273176	32613300	A	2.277	5.06E-12	chr:6:32648036
6	rs2021905	32281201	G	4.618	5.07E-12	chr:6:32648036
6	rs2395254	32716837	C	2.978	5.13E-12	chr:6:32648036
6	rs112616809	32946921	T	5.152	5.19E-12	chr:6:32648036
6	rs9272138	32600757	A	0.3399	5.25E-12	chr:6:32648036
6	rs9272622	32607986	C	2.641	5.34E-12	chr:6:32648036
6	rs9276496	32719017	A	2.986	5.35E-12	chr:6:32648036
6	rs113536666	32719362	G	2.976	5.41E-12	chr:6:32648036
6	rs5014411	32719634	G	2.976	5.41E-12	chr:6:32648036
6	rs5014412	32719573	A	2.976	5.41E-12	chr:6:32648036
6	rs5014414	32719440	G	2.976	5.41E-12	chr:6:32648036
6	rs527659718	32719414	A	2.976	5.41E-12	chr:6:32648036
6	rs535308210	32719439	T	2.976	5.41E-12	chr:6:32648036
6	rs572884610	32719411	C	2.976	5.41E-12	chr:6:32648036
6	rs9276502	32719666	C	2.976	5.41E-12	chr:6:32648036

6	rs386621302	32718908	C	3.012	5.43E-12	chr:6:32648036
6	rs9276484	32718452	G	2.99	5.44E-12	chr:6:32648036
6	rs9276485	32718479	T	2.99	5.44E-12	chr:6:32648036
6	rs9276486	32718481	C	2.99	5.44E-12	chr:6:32648036
6	rs28383453	32609358	T	3.852	5.51E-12	chr:6:32648036
6	rs7773182	32441422	T	0.3244	5.61E-12	chr:6:32648036
6	rs9272075	32599593	G	0.3312	5.62E-12	chr:6:32648036
6	rs2239806	32411307	T	3.767	5.67E-12	chr:6:32648036
6	rs116708054	32603761	C	4.02	5.69E-12	chr:6:32648036
6	rs3104405	32682308	C	0.3019	5.76E-12	chr:6:32648036
6	rs9275051	32648153	C	2.405	5.84E-12	chr:6:32648036
6	rs76391884	32604085	G	4.338	5.88E-12	chr:6:32648036
6	rs115223053	32602624	C	4.221	5.96E-12	chr:6:32648036
6	rs9269078	32440879	C	0.3201	6.04E-12	chr:6:32648036
6	rs17612690	32615918	C	2.105	6.09E-12	chr:6:32648036
6	rs115805770	32608432	T	4.247	6.21E-12	chr:6:32648036
6	rs116093550	32594395	G	0.3447	6.24E-12	chr:6:32648036
6	rs116361489	32594396	T	0.3447	6.24E-12	chr:6:32648036
6	rs76934421	32503722	T	4.657	6.24E-12	chr:6:32648036
6	rs2894291	32716827	C	2.964	6.36E-12	chr:6:32648036
6	rs6903608	32428285	C	0.3036	6.37E-12	chr:6:32648036
6	rs6923504	32428186	G	0.3036	6.37E-12	chr:6:32648036
6	rs9271954	32597216	C	0.3422	6.41E-12	chr:6:32648036
6	rs111365964	32517646	G	5.062	6.52E-12	chr:6:32648036
6	rs9268893	32431927	C	0.3277	6.61E-12	chr:6:32648036
6	rs9272454	32605525	C	0.332	6.85E-12	chr:6:32648036
6	rs9271999	32597832	G	0.3403	6.96E-12	chr:6:32648036
6	rs9272000	32597834	T	0.3403	6.96E-12	chr:6:32648036
6	rs28366362	32565213	C	3.005	6.96E-12	chr:6:32648036
6	rs28366352	32564993	C	2.992	7.15E-12	chr:6:32648036
6	rs28366353	32565004	G	2.992	7.15E-12	chr:6:32648036
6	rs9276487	32718594	A	2.966	7.22E-12	chr:6:32648036
6	rs139599717	32604992	T	4.779	7.30E-12	chr:6:32648036
6	rs536151678	32453543	G	2.895	7.37E-12	chr:6:32648036
6	rs9276491	32718827	G	3.001	7.39E-12	chr:6:32648036
6	rs9276492	32718828	T	3.001	7.39E-12	chr:6:32648036
6	rs9276493	32718869	G	3.001	7.39E-12	chr:6:32648036
6	rs9272151	32601005	G	0.3466	7.39E-12	chr:6:32648036
6	rs9272152	32601007	A	0.3466	7.39E-12	chr:6:32648036
6	rs9271648	32592358	T	0.341	7.40E-12	chr:6:32648036
6	rs9272136	32600730	T	0.3402	7.48E-12	chr:6:32648036
6	rs9271848	32595027	G	0.3432	7.51E-12	chr:6:32648036
6	rs9274286	32631802	A	2.175	7.51E-12	chr:6:32648036

6	rs184315023	32599644	T	3.035	7.55E-12	chr:6:32648036
6	rs181562347	32601738	G	4.225	7.63E-12	chr:6:32648036
6	rs183885674	32601732	G	4.225	7.63E-12	chr:6:32648036
6	rs191517186	32606859	C	5.001	7.70E-12	chr:6:32648036
6	rs535777	32577633	C	3.796	7.71E-12	chr:6:32648036
6	rs9271751	32593857	A	2.221	7.77E-12	chr:6:32648036
6	rs9268200	32278670	T	3.106	7.91E-12	chr:6:32648036
6	rs9268877	32431147	A	0.328	7.95E-12	chr:6:32648036
6	rs9274580	32635345	A	2.387	7.97E-12	chr:6:32648036
6	rs9274581	32635347	C	2.387	7.97E-12	chr:6:32648036
6	rs9274582	32635348	C	2.387	7.97E-12	chr:6:32648036
6	rs9272148	32600980	C	0.3485	8.02E-12	chr:6:32648036
6	rs9272156	32601081	C	0.3481	8.10E-12	chr:6:32648036
6	rs9274592	32635400	G	2.417	8.15E-12	chr:6:32648036
6	rs9271768	32594188	T	2.974	8.26E-12	chr:6:32648036
6	rs9276437	32714168	G	3.133	8.30E-12	chr:6:32648036
6	rs7739375	32441679	A	0.3214	8.33E-12	chr:6:32648036
6	rs3129882	32409530	G	0.3299	8.42E-12	chr:6:32648036
6	rs1129808	32609312	C	2.24	8.50E-12	chr:6:32648036
6	rs9272161	32601139	G	0.3472	8.70E-12	chr:6:32648036
6	rs9272162	32601140	A	0.3472	8.70E-12	chr:6:32648036
6	rs200951960	32510600	G	4.87	8.78E-12	chr:6:32648036
6	rs9272633	32608181	G	2.55	8.80E-12	chr:6:32648036
6	rs9271931	32596698	G	0.3108	8.85E-12	chr:6:32648036
6	rs561918072	32607733	A	4.329	9.03E-12	chr:6:32648036
6	rs9274550	32634795	A	2.27	9.21E-12	chr:6:32648036
6	rs9272174	32601307	T	0.3427	9.34E-12	chr:6:32648036
6	rs7739203	32441555	A	0.3303	9.41E-12	chr:6:32648036
6	rs4959110	32597599	C	3.99	9.42E-12	chr:6:32648036
6	rs9272153	32601018	C	0.3498	9.48E-12	chr:6:32648036
6	rs9272679	32608931	C	3.425	9.48E-12	chr:6:32648036
6	rs7600	32610769	A	0.3676	9.54E-12	chr:6:32648036
6	rs9406389	32517764	C	7.983	9.56E-12	chr:6:32648036
6	rs9274551	32634834	T	2.271	9.76E-12	chr:6:32648036
6	rs9274552	32634838	C	2.271	9.76E-12	chr:6:32648036
6	rs9269144	32445519	A	0.3058	9.83E-12	chr:6:32648036
6	rs115124923	32510482	A	4.937	9.85E-12	chr:6:32648036
6	rs9501179	32292993	A	4.38	9.91E-12	chr:6:32648036
6	rs9272522	32606490	A	2.528	1.01E-11	chr:6:32648036
6	rs6919855	32429011	C	0.308	1.03E-11	chr:6:32648036
6	rs9272150	32601000	A	0.3507	1.03E-11	chr:6:32648036
6	rs9272158	32601122	A	0.3489	1.04E-11	chr:6:32648036
6	rs3188642	32610553	A	3.506	1.06E-11	chr:6:32648036

6	rs28383182	32571811	A	3.25	1.07E-11	chr:6:32648036
6	rs9273119	32612699	A	2.188	1.07E-11	chr:6:32648036
6	rs9272128	32600601	A	0.347	1.08E-11	chr:6:32648036
6	rs9272129	32600602	T	0.347	1.08E-11	chr:6:32648036
6	rs9272163	32601146	T	0.3496	1.11E-11	chr:6:32648036
6	rs111374340	32509477	G	5.18	1.13E-11	chr:6:32648036
6	rs9271858	32595223	G	0.3391	1.16E-11	chr:6:32648036
6	rs9271859	32595231	G	0.3391	1.16E-11	chr:6:32648036
6	rs574901905	32452501	C	4.321	1.17E-11	chr:6:32648036
6	rs9272037	32598844	C	0.3485	1.19E-11	chr:6:32648036
6	rs2856687	32654822	T	0.2854	1.21E-11	chr:6:32648036
6	rs9273114	32612664	G	2.179	1.22E-11	chr:6:32648036
6	rs9271843	32594957	T	0.2592	1.24E-11	chr:6:32648036
6	rs9271794	32594855	A	0.3048	1.27E-11	chr:6:32648036
6	rs28393315	32603177	G	3.456	1.30E-11	chr:6:32648036
6	rs9272508	32606325	A	2.292	1.30E-11	chr:6:32648036
6	rs9271746	32593804	C	2.204	1.32E-11	chr:6:32648036
6	rs7453703	32440158	A	0.3218	1.35E-11	chr:6:32648036
6	rs75074972	32607264	G	5.302	1.37E-11	chr:6:32648036
6	rs28366351	32564990	A	2.939	1.37E-11	chr:6:32648036
6	rs9272521	32606479	A	2.512	1.39E-11	chr:6:32648036
6	rs9268882	32431623	T	0.3075	1.39E-11	chr:6:32648036
6	rs2894292	32721656	A	2.924	1.39E-11	chr:6:32648036
6	rs2858317	32662280	C	0.3171	1.40E-11	chr:6:32648036
6	rs9273554	32628941	C	6.324	1.46E-11	chr:6:32648036
6	rs62405567	32453372	G	3.165	1.50E-11	chr:6:32648036
6	rs9271846	32594980	A	0.263	1.50E-11	chr:6:32648036
6	rs72848212	32594898	T	0.2887	1.51E-11	chr:6:32648036
6	rs373880870	32599763	T	2.72	1.52E-11	chr:6:32648036
6	rs9272137	32600738	C	0.3499	1.52E-11	chr:6:32648036
6	rs9273407	32627211	C	2.288	1.54E-11	chr:6:32648036
6	rs9269135	32445030	T	0.3106	1.57E-11	chr:6:32648036
6	rs9273117	32612688	G	2.172	1.59E-11	chr:6:32648036
6	rs181107089	32599677	T	2.922	1.60E-11	chr:6:32648036
6	rs187272234	32599617	C	2.995	1.61E-11	chr:6:32648036
6	rs12191360	32451361	C	0.3095	1.62E-11	chr:6:32648036
6	rs9260	32610461	A	0.2747	1.62E-11	chr:6:32648036
6	rs9269080	32440969	G	0.335	1.73E-11	chr:6:32648036
6	rs9276513	32720144	G	2.907	1.75E-11	chr:6:32648036
6	rs9271842	32594953	A	0.2686	1.76E-11	chr:6:32648036
6	rs138238823	32598837	C	3.436	1.78E-11	chr:6:32648036
6	rs138816958	32598798	C	3.436	1.78E-11	chr:6:32648036
6	rs9269143	32445499	T	2.835	1.79E-11	chr:6:32648036

6	rs9271442	32588260	A	3.03	1.82E-11	chr:6:32648036
6	rs9271382	32587375	G	0.3315	1.85E-11	chr:6:32648036
6	rs2856670	32662904	G	0.2846	1.86E-11	chr:6:32648036
6	rs9276508	32720004	C	2.903	1.86E-11	chr:6:32648036
6	rs9276509	32720005	A	2.903	1.86E-11	chr:6:32648036
6	rs9276511	32720037	G	2.903	1.86E-11	chr:6:32648036
6	rs2858318	32661302	T	0.2876	1.89E-11	chr:6:32648036
6	rs2858319	32661294	T	0.2876	1.89E-11	chr:6:32648036
6	rs9268847	32429277	G	0.3244	1.90E-11	chr:6:32648036
6	rs9271962	32597434	C	0.3514	1.91E-11	chr:6:32648036
6	rs9273404	32627160	G	2.129	1.93E-11	chr:6:32648036
6	rs2647020	32663256	T	0.2847	1.95E-11	chr:6:32648036
6	rs2647021	32663151	T	0.2847	1.95E-11	chr:6:32648036
6	rs151304730	32554227	A	7.475	1.97E-11	chr:6:32648036
6	rs9269072	32440508	G	0.3228	1.98E-11	chr:6:32648036
6	rs9271788	32594734	G	0.3469	1.99E-11	chr:6:32648036
6	rs2647080	32566646	A	2.939	1.99E-11	chr:6:32648036
6	rs28752490	32567897	A	2.939	1.99E-11	chr:6:32648036
6	rs75783927	32566712	C	2.939	1.99E-11	chr:6:32648036
6	rs2646998	32665367	T	0.2846	1.99E-11	chr:6:32648036
6	rs2647001	32665086	C	0.2846	1.99E-11	chr:6:32648036
6	rs2647003	32664880	T	0.2846	1.99E-11	chr:6:32648036
6	rs2647013	32664284	C	0.2846	1.99E-11	chr:6:32648036
6	rs2647016	32663925	C	0.2846	1.99E-11	chr:6:32648036
6	rs2647018	32663447	T	0.2846	1.99E-11	chr:6:32648036
6	rs2647022	32663004	A	0.2846	1.99E-11	chr:6:32648036
6	rs2856666	32665285	G	0.2846	1.99E-11	chr:6:32648036
6	rs2856668	32663606	G	0.2846	1.99E-11	chr:6:32648036
6	rs2856727	32666397	C	0.2846	1.99E-11	chr:6:32648036
6	rs2858324	32660375	A	0.2846	1.99E-11	chr:6:32648036
6	rs9275212	32658327	G	0.2846	1.99E-11	chr:6:32648036
6	rs528827703	32452406	G	3.529	2.00E-11	chr:6:32648036
6	rs9276518	32720385	G	2.896	2.02E-11	chr:6:32648036
6	rs9276520	32720415	T	2.896	2.02E-11	chr:6:32648036
6	rs9276549	32722242	T	2.895	2.04E-11	chr:6:32648036
6	rs17612669	32615816	A	2.161	2.05E-11	chr:6:32648036
6	rs3135385	32416366	A	0.3107	2.08E-11	chr:6:32648036
6	rs7382085	32414420	G	0.3107	2.08E-11	chr:6:32648036
6	rs7382354	32414639	G	0.3107	2.08E-11	chr:6:32648036
6	rs28752496	32568321	C	2.87	2.08E-11	chr:6:32648036
6	rs536493456	32674838	G	0.2867	2.10E-11	chr:6:32648036
6	rs9271847	32594993	G	0.2882	2.10E-11	chr:6:32648036
6	rs9268671	32414290	A	0.313	2.14E-11	chr:6:32648036

6	rs9273551	32628913	T	6.538	2.16E-11	chr:6:32648036
6	rs9273552	32628916	A	6.538	2.16E-11	chr:6:32648036
6	rs9271970	32597550	A	0.342	2.18E-11	chr:6:32648036
6	rs9274349	32632408	T	2.181	2.19E-11	chr:6:32648036
6	rs9269175	32447451	G	0.3132	2.19E-11	chr:6:32648036
6	rs9269176	32447545	T	0.3132	2.19E-11	chr:6:32648036
6	rs9269178	32447598	A	0.3132	2.19E-11	chr:6:32648036
6	rs482162	32576019	C	2.95	2.20E-11	chr:6:32648036
6	rs482205	32576009	G	2.95	2.20E-11	chr:6:32648036
6	rs9272084	32599680	T	0.3294	2.21E-11	chr:6:32648036
6	rs9271968	32597529	G	0.3378	2.21E-11	chr:6:32648036
6	rs75016307	32435767	C	0.3591	2.22E-11	chr:6:32648036
6	rs9273115	32612672	A	2.167	2.24E-11	chr:6:32648036
6	rs2894261	32447769	C	0.3132	2.27E-11	chr:6:32648036
6	rs2894262	32447816	A	0.3132	2.27E-11	chr:6:32648036
6	rs9268880	32431358	T	0.3132	2.27E-11	chr:6:32648036
6	rs9269110	32443269	A	0.3132	2.27E-11	chr:6:32648036
6	rs9269114	32443425	A	0.3132	2.27E-11	chr:6:32648036
6	rs9269157	32446483	T	0.3132	2.27E-11	chr:6:32648036
6	rs9269172	32447154	C	0.3132	2.27E-11	chr:6:32648036
6	rs9269174	32447364	T	0.3132	2.27E-11	chr:6:32648036
6	rs9273409	32627235	T	2.283	2.29E-11	chr:6:32648036
6	rs28383310	32586930	A	3.617	2.30E-11	chr:6:32648036
6	rs4470886	32721020	T	2.886	2.35E-11	chr:6:32648036
6	rs4498415	32720986	T	2.886	2.35E-11	chr:6:32648036
6	rs7774644	32724075	G	2.886	2.35E-11	chr:6:32648036
6	rs9276516	32720302	C	2.886	2.35E-11	chr:6:32648036
6	rs9276517	32720366	C	2.886	2.35E-11	chr:6:32648036
6	rs9276526	32720589	T	2.886	2.35E-11	chr:6:32648036
6	rs9276527	32720637	A	2.886	2.35E-11	chr:6:32648036
6	rs9276528	32720934	T	2.886	2.35E-11	chr:6:32648036
6	rs9276529	32720971	T	2.886	2.35E-11	chr:6:32648036
6	rs9276546	32722129	T	2.886	2.35E-11	chr:6:32648036
6	rs9276547	32722155	A	2.886	2.35E-11	chr:6:32648036
6	rs9276548	32722220	C	2.886	2.35E-11	chr:6:32648036
6	rs17203846	32555989	A	3.637	2.37E-11	chr:6:32648036
6	rs9271782	32594562	A	0.326	2.39E-11	chr:6:32648036
6	rs9271784	32594704	A	0.3152	2.40E-11	chr:6:32648036
6	rs9271786	32594709	T	0.3152	2.40E-11	chr:6:32648036
6	rs9275032	32643839	T	0.3347	2.41E-11	chr:6:32648036
6	rs12722051	32609147	T	3.536	2.43E-11	chr:6:32648036
6	rs9271781	32594547	A	0.3293	2.44E-11	chr:6:32648036
6	rs9272072	32599563	G	0.3482	2.44E-11	chr:6:32648036

6	rs9272073	32599570	T	0.3482	2.44E-11	chr:6:32648036
6	rs9269142	32445423	G	0.3133	2.46E-11	chr:6:32648036
6	rs9272086	32599688	T	0.3326	2.46E-11	chr:6:32648036
6	rs28417660	32603044	T	4.014	2.49E-11	chr:6:32648036
6	rs28635391	32603048	A	4.014	2.49E-11	chr:6:32648036
6	rs9271386	32587416	T	0.3084	2.55E-11	chr:6:32648036
6	rs9272121	32600556	C	0.3564	2.55E-11	chr:6:32648036
6	rs7739432	32198474	T	2.862	2.56E-11	chr:6:32648036
6	rs375703547	32608217	C	4.838	2.59E-11	chr:6:32648036
6	rs9273258	32614268	G	2.05	2.59E-11	chr:6:32648036
6	rs9269137	32445147	C	0.3214	2.60E-11	chr:6:32648036
6	rs9269138	32445155	A	0.3214	2.60E-11	chr:6:32648036
6	rs9269139	32445156	G	0.3214	2.60E-11	chr:6:32648036
6	rs28383174	32566288	A	2.904	2.60E-11	chr:6:32648036
6	rs552704888	32554994	T	3.827	2.65E-11	chr:6:32648036
6	rs2647012	32664458	T	0.2875	2.66E-11	chr:6:32648036
6	rs9272165	32601170	C	0.3597	2.68E-11	chr:6:32648036
6	rs540590778	32453500	G	2.947	2.68E-11	chr:6:32648036
6	rs9273118	32612691	G	2.153	2.72E-11	chr:6:32648036
6	rs578222092	32720017	C	2.885	2.73E-11	chr:6:32648036
6	rs9269083	32441193	A	0.3412	2.75E-11	chr:6:32648036
6	rs9272035	32598814	T	0.3578	2.80E-11	chr:6:32648036
6	rs9271787	32594718	C	0.3393	2.86E-11	chr:6:32648036
6	rs9269141	32445270	C	0.315	2.87E-11	chr:6:32648036
6	rs9272803	32610591	T	3.123	2.89E-11	chr:6:32648036
6	rs115317719	32412005	T	3.657	2.91E-11	chr:6:32648036
6	rs539718048	32412004	C	3.657	2.91E-11	chr:6:32648036
6	rs202207567	32512533	T	3.128	2.92E-11	chr:6:32648036
6	rs377093	32194077	G	0.3431	2.96E-11	chr:6:32648036
6	rs3132948	32197219	T	2.8	2.96E-11	chr:6:32648036
6	rs9267857	32196569	A	2.8	2.96E-11	chr:6:32648036
6	rs9267861	32196648	G	2.8	2.96E-11	chr:6:32648036
6	rs5026743	32439964	G	0.3242	3.01E-11	chr:6:32648036
6	rs9271963	32597453	A	0.3456	3.03E-11	chr:6:32648036
6	rs72492305	32509333	C	0.3284	3.13E-11	chr:6:32648036
6	rs17843693	32622122	A	3.203	3.18E-11	chr:6:32648036
6	rs9272102	32599830	C	0.3551	3.20E-11	chr:6:32648036
6	rs9271748	32593830	G	2.167	3.21E-11	chr:6:32648036
6	rs9271749	32593836	T	2.167	3.21E-11	chr:6:32648036
6	rs9271750	32593837	G	2.167	3.21E-11	chr:6:32648036
6	rs9273057	32612104	T	3.61	3.22E-11	chr:6:32648036
6	rs9271823	32594915	G	0.2769	3.25E-11	chr:6:32648036
6	rs9271824	32594916	G	0.2769	3.25E-11	chr:6:32648036

6	rs9269234	32451788	C	0.3129	3.26E-11	chr:6:32648036
6	rs7755774	32444932	G	0.3137	3.33E-11	chr:6:32648036
6	rs9275109	32649676	G	0.2809	3.43E-11	chr:6:32648036
6	rs9271886	32595633	T	0.3576	3.44E-11	chr:6:32648036
6	rs536427156	32453103	G	3.03	3.45E-11	chr:6:32648036
6	rs643889	32575918	T	2.893	3.49E-11	chr:6:32648036
6	rs9273166	32613146	G	2.185	3.55E-11	chr:6:32648036
6	rs138487125	32599812	T	2.89	3.57E-11	chr:6:32648036
6	rs9273170	32613177	G	2.224	3.61E-11	chr:6:32648036
6	rs2856725	32666738	C	0.292	3.61E-11	chr:6:32648036
6	rs113422598	32626508	T	3.463	3.62E-11	chr:6:32648036
6	rs17211741	32626346	A	3.463	3.62E-11	chr:6:32648036
6	rs79638487	32626664	A	3.463	3.62E-11	chr:6:32648036
6	rs9271399	32587510	C	0.3485	3.62E-11	chr:6:32648036
6	rs9276503	32719731	A	2.835	3.62E-11	chr:6:32648036
6	rs9276504	32719745	C	2.835	3.62E-11	chr:6:32648036
6	rs550286428	32452407	T	4.167	3.66E-11	chr:6:32648036
6	rs601148	32573265	T	4.859	3.68E-11	chr:6:32648036
6	rs9269074	32440792	A	3.675	3.70E-11	chr:6:32648036
6	rs13193606	32575044	T	2.882	3.71E-11	chr:6:32648036
6	rs75872455	32502638	C	2.371	3.71E-11	chr:6:32648036
6	rs2840230	32596145	A	3.252	3.74E-11	chr:6:32648036
6	rs481139	32576145	A	2.901	3.75E-11	chr:6:32648036
6	rs139727756	32603562	A	4.029	3.81E-11	chr:6:32648036
6	rs549131306	32603558	T	4.029	3.81E-11	chr:6:32648036
6	rs9274548	32634785	G	2.194	3.82E-11	chr:6:32648036
6	rs9274549	32634786	G	2.194	3.82E-11	chr:6:32648036
6	rs9271780	32594535	C	0.3363	3.83E-11	chr:6:32648036
6	rs9273167	32613163	A	2.19	3.85E-11	chr:6:32648036
6	rs9274595	32635450	A	2.351	3.90E-11	chr:6:32648036
6	rs200004305	32605204	G	4.679	3.90E-11	chr:6:32648036
6	rs9271883	32595610	A	0.3607	3.91E-11	chr:6:32648036
6	rs560849611	32478729	A	5.485	3.92E-11	chr:6:32648036
6	rs17605238	32299908	C	3.848	3.94E-11	chr:6:32648036
6	rs2022536	32301960	C	3.848	3.94E-11	chr:6:32648036
6	rs7765585	32304025	G	3.848	3.94E-11	chr:6:32648036
6	rs9469098	32296382	T	3.848	3.94E-11	chr:6:32648036
6	rs28366363	32565355	A	5.625	3.96E-11	chr:6:32648036
6	rs28366344	32564813	C	2.822	3.96E-11	chr:6:32648036
6	rs2395184	32432646	A	3.307	4.03E-11	chr:6:32648036
6	rs6901541	32442261	C	2.726	4.03E-11	chr:6:32648036
6	rs28752497	32568481	A	2.879	4.04E-11	chr:6:32648036
6	rs9271964	32597464	C	0.3486	4.05E-11	chr:6:32648036

6	rs543713	32573934	T	2.873	4.09E-11	chr:6:32648036
6	rs28383461	32609607	A	3.593	4.13E-11	chr:6:32648036
6	rs138574269	32507114	A	2.285	4.13E-11	chr:6:32648036
6	rs9272171	32601249	G	0.3599	4.25E-11	chr:6:32648036
6	rs9271389	32587431	T	0.3487	4.27E-11	chr:6:32648036
6	rs9274346	32632367	T	2.113	4.29E-11	chr:6:32648036
6	rs9271966	32597507	T	0.3604	4.29E-11	chr:6:32648036
6	rs9272124	32600581	A	0.3622	4.32E-11	chr:6:32648036
6	rs28383173	32566287	C	2.848	4.46E-11	chr:6:32648036
6	rs9269085	32441202	C	0.3462	4.49E-11	chr:6:32648036
6	rs28383409	32608279	G	2.617	4.54E-11	chr:6:32648036
6	rs2858310	32668323	G	0.2917	4.73E-11	chr:6:32648036
6	rs576125530	32522109	G	3.618	4.73E-11	chr:6:32648036
6	rs9271881	32595604	A	0.3629	4.80E-11	chr:6:32648036
6	rs9271882	32595605	T	0.3629	4.80E-11	chr:6:32648036
6	rs28724264	32631937	G	2.176	4.81E-11	chr:6:32648036
6	rs2647040	32667280	A	0.2916	4.85E-11	chr:6:32648036
6	rs614348	32573877	C	2.863	4.99E-11	chr:6:32648036
6	rs9272005	32597992	G	0.3609	5.00E-11	chr:6:32648036
6	rs9272006	32597993	T	0.3609	5.00E-11	chr:6:32648036
6	rs9269102	32442741	C	2.725	5.01E-11	chr:6:32648036
6	rs2647046	32668336	A	0.2915	5.07E-11	chr:6:32648036
6	rs2647048	32668705	A	0.2915	5.07E-11	chr:6:32648036
6	rs2647051	32670897	T	0.2915	5.07E-11	chr:6:32648036
6	rs2856699	32671765	A	0.2915	5.07E-11	chr:6:32648036
6	rs2856717	32670308	A	0.2915	5.07E-11	chr:6:32648036
6	rs2858305	32670464	G	0.2915	5.07E-11	chr:6:32648036
6	rs2858309	32668713	C	0.2915	5.07E-11	chr:6:32648036
6	rs9275389	32669132	C	0.2915	5.07E-11	chr:6:32648036
6	rs28383311	32587011	T	3.528	5.10E-11	chr:6:32648036
6	rs1142331	32609254	T	2.242	5.11E-11	chr:6:32648036
6	rs1142332	32609255	T	2.242	5.11E-11	chr:6:32648036
6	rs9272702	32609253	A	2.242	5.11E-11	chr:6:32648036
6	rs7776032	32444896	C	0.316	5.11E-11	chr:6:32648036
6	rs9269126	32444915	C	0.316	5.11E-11	chr:6:32648036
6	rs9273168	32613173	C	2.201	5.23E-11	chr:6:32648036
6	rs28752493	32568165	C	2.838	5.26E-11	chr:6:32648036
6	rs9271884	32595628	C	0.3626	5.29E-11	chr:6:32648036
6	rs9271885	32595629	T	0.3626	5.29E-11	chr:6:32648036
6	rs6924676	32445727	T	0.3204	5.32E-11	chr:6:32648036
6	rs28752501	32568995	A	2.768	5.38E-11	chr:6:32648036
6	rs4193	32609236	A	2.281	5.43E-11	chr:6:32648036
6	rs538680043	32444933	T	0.3162	5.52E-11	chr:6:32648036

6	rs9272012	32598156	A	0.3598	5.79E-11	chr:6:32648036
6	rs9269118	32443641	G	0.314	5.81E-11	chr:6:32648036
6	rs563837736	32660582	A	0.3055	5.82E-11	chr:6:32648036
6	rs28752498	32568652	A	2.818	6.05E-11	chr:6:32648036
6	rs9272140	32600780	G	3.254	6.08E-11	chr:6:32648036
6	rs75351515	32635420	A	2.286	6.09E-11	chr:6:32648036
6	rs601481	32573293	C	2.84	6.10E-11	chr:6:32648036
6	rs9270797	32569297	A	0.3702	6.10E-11	chr:6:32648036
6	rs601006	32573173	A	2.825	6.11E-11	chr:6:32648036
6	rs9269163	32446833	A	0.3233	6.16E-11	chr:6:32648036
6	rs9269165	32446843	A	0.3233	6.16E-11	chr:6:32648036
6	rs2157334	32392543	C	3.344	6.28E-11	chr:6:32648036
6	rs2395170	32394313	C	3.344	6.28E-11	chr:6:32648036
6	rs2395171	32394537	T	3.344	6.28E-11	chr:6:32648036
6	rs3129842	32394060	C	3.344	6.28E-11	chr:6:32648036
6	rs3135349	32394027	G	3.344	6.28E-11	chr:6:32648036
6	rs541986750	32391192	C	3.344	6.28E-11	chr:6:32648036
6	rs9272770	32610226	A	3.285	6.29E-11	chr:6:32648036
6	rs9272166	32601209	C	0.3663	6.34E-11	chr:6:32648036
6	rs7770017	32237463	A	2.754	6.35E-11	chr:6:32648036
6	rs2856722	32667958	A	0.2946	6.42E-11	chr:6:32648036
6	rs116023697;rs28986256	32717799	C	3.217	6.45E-11	chr:6:32648036
6	rs9270664	32566149	G	0.3372	6.45E-11	chr:6:32648036
6	rs28383201	32574869	G	2.823	6.46E-11	chr:6:32648036
6	rs9275521	32674952	T	0.2951	6.77E-11	chr:6:32648036
6	rs2856723	32667762	A	0.2944	6.89E-11	chr:6:32648036
6	rs9272092	32599756	C	0.3241	6.95E-11	chr:6:32648036
6	rs139558884	32604167	C	4.098	6.99E-11	chr:6:32648036
6	rs2894260	32447058	T	2.689	7.03E-11	chr:6:32648036
6	rs4993690	32446971	A	2.689	7.03E-11	chr:6:32648036
6	rs550108049	32445145	G	2.689	7.03E-11	chr:6:32648036
6	rs9268861	32429894	A	2.689	7.03E-11	chr:6:32648036
6	rs9269049	32438850	C	2.689	7.03E-11	chr:6:32648036
6	rs9269171	32447120	A	2.689	7.03E-11	chr:6:32648036
6	rs28752500	32568769	T	2.824	7.12E-11	chr:6:32648036
6	rs602457	32573562	C	4.751	7.19E-11	chr:6:32648036
6	rs548718231	32641196	A	3.917	7.20E-11	chr:6:32648036
6	rs9271368	32586934	G	0.3331	7.31E-11	chr:6:32648036
6	rs3115574	32217413	T	2.841	7.34E-11	chr:6:32648036
6	rs6936204	32217092	T	2.841	7.34E-11	chr:6:32648036
6	rs9273059	32612111	A	3.543	7.49E-11	chr:6:32648036
6	rs17210202	32555987	C	3.675	7.72E-11	chr:6:32648036
6	rs28366354	32565044	G	2.779	7.96E-11	chr:6:32648036

6	rs370055	32194259	C	0.3592	7.97E-11	chr:6:32648036
6	rs9273149	32612987	A	3.199	8.21E-11	chr:6:32648036
6	rs200501057	32487088	T	5.706	8.24E-11	chr:6:32648036
6	rs7453498	32446717	T	0.3531	8.26E-11	chr:6:32648036
6	rs28366212	32557259	T	3.25	8.31E-11	chr:6:32648036
6	rs28366213	32557263	A	3.25	8.31E-11	chr:6:32648036
6	rs1612904	32669018	C	0.2876	8.37E-11	chr:6:32648036
6	rs34276369	32614037	T	3.356	8.40E-11	chr:6:32648036
6	rs9269201	32449345	C	0.314	8.55E-11	chr:6:32648036
6	rs9269164	32446840	C	2.682	8.59E-11	chr:6:32648036
6	rs190293957	32641526	A	4.601	8.64E-11	chr:6:32648036
6	rs574897295	32548550	A	5.805	9.02E-11	chr:6:32648036
6	rs112894757	32510777	T	2.431	9.02E-11	chr:6:32648036
6	rs9273139	32612860	G	2.219	9.25E-11	chr:6:32648036
6	rs447903	32193945	T	0.3601	9.29E-11	chr:6:32648036
6	rs499691	32194339	T	0.3601	9.29E-11	chr:6:32648036
6	rs535195653	32453064	G	3.79	9.33E-11	chr:6:32648036
6	rs2647043	32667549	A	0.2986	9.40E-11	chr:6:32648036
6	rs9269104	32443032	A	2.668	9.47E-11	chr:6:32648036
6	rs9269105	32443034	C	2.668	9.47E-11	chr:6:32648036
6	rs7450278	32439048	T	2.66	9.49E-11	chr:6:32648036
6	rs112628749	32510769	T	2.45	9.53E-11	chr:6:32648036
6	rs113766057	32510772	A	2.45	9.53E-11	chr:6:32648036
6	rs9271791	32594810	A	0.3719	9.53E-11	chr:6:32648036
6	rs9273406	32627180	C	2.084	9.81E-11	chr:6:32648036
6	rs4959112	32597747	T	2.537	9.99E-11	chr:6:32648036
6	rs9273405	32627175	C	2.083	1.01E-10	chr:6:32648036
6	rs28724262	32631889	C	2.159	1.03E-10	chr:6:32648036
6	rs376975457	32630317	G	2.335	1.04E-10	chr:6:32648036
6	rs6906021	32626311	T	0.369	1.08E-10	chr:6:32648036
6	rs3129721	32676251	C	0.2912	1.09E-10	chr:6:32648036
6	rs9273140	32612868	C	2.214	1.10E-10	chr:6:32648036
6	rs527493854	32675902	T	0.2935	1.12E-10	chr:6:32648036
6	rs113220099	32510823	T	2.447	1.12E-10	chr:6:32648036
6	rs201117812	32610286	A	3.314	1.13E-10	chr:6:32648036
6	rs28383170	32565722	T	2.778	1.13E-10	chr:6:32648036
6	rs28383171	32565735	T	2.778	1.13E-10	chr:6:32648036
6	rs549070097	32516282	G	3.068	1.15E-10	chr:6:32648036
6	rs9269119	32443797	G	2.69	1.17E-10	chr:6:32648036
6	rs28752492	32568138	A	2.779	1.17E-10	chr:6:32648036
6	rs28366341	32564784	C	2.724	1.19E-10	chr:6:32648036
6	rs28366343	32564803	C	2.724	1.19E-10	chr:6:32648036
6	rs9271989	32597729	T	0.3488	1.22E-10	chr:6:32648036

6	rs9272587	32607510	A	2.091	1.24E-10	chr:6:32648036
6	rs146130478	32499213	C	2.524	1.25E-10	chr:6:32648036
6	rs9273192	32613437	A	2.036	1.27E-10	chr:6:32648036
6	rs113663338	32624847	T	2.11	1.27E-10	chr:6:32648036
6	rs35983205	32624829	C	2.11	1.27E-10	chr:6:32648036
6	rs28383172	32565979	G	5.212	1.32E-10	chr:6:32648036
6	rs13193700	32575078	T	2.777	1.32E-10	chr:6:32648036
6	rs574170	32572904	A	2.792	1.33E-10	chr:6:32648036
6	rs4947345	32678260	T	0.3055	1.34E-10	chr:6:32648036
6	rs9269027	32437466	A	0.3265	1.39E-10	chr:6:32648036
6	rs9269081	32441100	A	0.3265	1.39E-10	chr:6:32648036
6	rs9271995	32597777	A	0.3671	1.40E-10	chr:6:32648036
6	rs9272718	32609379	T	2.072	1.41E-10	chr:6:32648036
6	rs28490182	32592476	T	0.3622	1.42E-10	chr:6:32648036
6	rs3132949	32195935	T	2.69	1.43E-10	chr:6:32648036
6	rs9271789	32594768	A	0.3778	1.43E-10	chr:6:32648036
6	rs9269159	32446691	G	0.3423	1.44E-10	chr:6:32648036
6	rs1894553	32502866	A	3.171	1.45E-10	chr:6:32648036
6	rs9274639	32636146	T	0.3208	1.46E-10	chr:6:32648036
6	rs387071	32193449	C	0.3649	1.46E-10	chr:6:32648036
6	rs3129758	32584625	A	0.3119	1.52E-10	chr:6:32648036
6	rs9394104	32620203	G	2.161	1.52E-10	chr:6:32648036
6	rs9394105	32620206	T	2.161	1.52E-10	chr:6:32648036
6	rs112838474	32295816	T	3.773	1.56E-10	chr:6:32648036
6	rs28383280	32586342	G	4.868	1.61E-10	chr:6:32648036
6	rs28383281	32586344	G	4.868	1.61E-10	chr:6:32648036
6	rs9274596	32635467	T	2.274	1.63E-10	chr:6:32648036
6	rs9271445	32588299	T	2.846	1.63E-10	chr:6:32648036
6	rs9275766	32689362	G	4.86	1.63E-10	chr:6:32648036
6	rs28752502	32569051	G	5.303	1.63E-10	chr:6:32648036
6	rs9269162	32446737	A	0.3551	1.66E-10	chr:6:32648036
6	rs1142338	32609322	T	4.132	1.66E-10	chr:6:32648036
6	rs118038025	32625692	C	3.566	1.67E-10	chr:6:32648036
6	rs9271990	32597735	T	0.3554	1.68E-10	chr:6:32648036
6	rs9271991	32597738	G	0.3554	1.68E-10	chr:6:32648036
6	rs185295622	32510840	A	2.327	1.70E-10	chr:6:32648036
6	rs9271988	32597725	A	0.3523	1.71E-10	chr:6:32648036
6	rs9272099	32599813	G	0.3657	1.71E-10	chr:6:32648036
6	rs9273286	32614494	A	1.949	1.75E-10	chr:6:32648036
6	rs9272093	32599777	T	0.314	1.76E-10	chr:6:32648036
6	rs78017592	32508455	C	3.365	1.76E-10	chr:6:32648036
6	rs9269116	32443458	T	0.3164	1.83E-10	chr:6:32648036
6	rs113534515	32460798	C	3.731	1.86E-10	chr:6:32648036

6	rs6905297	32446100	C	0.319	1.86E-10	chr:6:32648036
6	rs9273141	32612883	G	2.211	1.87E-10	chr:6:32648036
6	rs138222522	32510733	T	2.455	1.88E-10	chr:6:32648036
6	rs79936591	32503543	A	4.1	1.89E-10	chr:6:32648036
6	rs9273190	32613418	A	2.067	1.91E-10	chr:6:32648036
6	rs9273549	32628873	T	2.615	1.91E-10	chr:6:32648036
6	rs182013486	32603534	C	3.838	1.93E-10	chr:6:32648036
6	rs9273157	32613052	G	3.454	1.94E-10	chr:6:32648036
6	rs28366357	32565138	T	2.739	1.95E-10	chr:6:32648036
6	rs28366360	32565176	C	2.746	1.97E-10	chr:6:32648036
6	rs3130320	32223258	T	2.75	1.98E-10	chr:6:32648036
6	rs9275596	32681631	C	0.2955	2.03E-10	chr:6:32648036
6	rs9272097	32599786	T	0.3071	2.05E-10	chr:6:32648036
6	rs4999342	32448098	T	0.3274	2.07E-10	chr:6:32648036
6	rs28366361	32565197	A	2.733	2.07E-10	chr:6:32648036
6	rs116150265	32607526	C	2.051	2.13E-10	chr:6:32648036
6	rs9272589	32607524	C	2.051	2.13E-10	chr:6:32648036
6	rs1694129	32637743	T	6.031	2.16E-10	chr:6:32648036
6	rs9269076	32440805	C	0.3512	2.18E-10	chr:6:32648036
6	rs9269077	32440806	C	0.3512	2.18E-10	chr:6:32648036
6	rs74929336	32502585	A	2.502	2.19E-10	chr:6:32648036
6	rs9271434	32588058	A	2.802	2.19E-10	chr:6:32648036
6	rs17884945	32552029	T	3.168	2.25E-10	chr:6:32648036
6	rs9273142	32612886	C	2.205	2.28E-10	chr:6:32648036
6	rs2133037	32667596	A	5.915	2.32E-10	chr:6:32648036
6	rs9271924	32596498	A	0.313	2.35E-10	chr:6:32648036
6	rs9273143	32612899	G	2.219	2.35E-10	chr:6:32648036
6	rs28752494	32568240	A	2.72	2.36E-10	chr:6:32648036
6	rs9268914	32432556	C	0.3361	2.42E-10	chr:6:32648036
6	rs3134981	32646900	A	0.3765	2.44E-10	chr:6:32648036
6	rs115966492	32478811	T	3.749	2.46E-10	chr:6:32648036
6	rs80072299	32459923	G	2.167	2.46E-10	chr:6:32648036
6	rs28752491	32568127	C	5.198	2.52E-10	chr:6:32648036
6	rs3135362	32389747	G	3.192	2.55E-10	chr:6:32648036
6	rs7758269	32598812	C	0.3564	2.56E-10	chr:6:32648036
6	rs35984981	32554725	T	3.36	2.58E-10	chr:6:32648036
6	rs6922257	32595682	A	3.185	2.66E-10	chr:6:32648036
6	rs111343297	32520080	G	3.156	2.67E-10	chr:6:32648036
6	rs28366355	32565056	G	2.67	2.70E-10	chr:6:32648036
6	rs201181224	32429810	T	0.3073	2.72E-10	chr:6:32648036
6	rs28366308	32561043	T	3.3	2.74E-10	chr:6:32648036
6	rs28366309	32561048	T	3.3	2.74E-10	chr:6:32648036
6	rs28383456	32609453	T	3.959	2.80E-10	chr:6:32648036

6	rs9272095	32599782	A	0.3163	2.82E-10	chr:6:32648036
6	rs9272098	32599787	G	0.3163	2.82E-10	chr:6:32648036
6	rs9271986	32597700	A	0.353	2.92E-10	chr:6:32648036
6	rs3134975	32652581	T	0.2986	2.94E-10	chr:6:32648036
6	rs9274594	32635435	T	2.225	2.98E-10	chr:6:32648036
6	rs4713575	32627039	G	2.201	3.04E-10	chr:6:32648036
6	rs9271984	32597669	G	0.3398	3.05E-10	chr:6:32648036
6	rs28752495	32568315	T	2.692	3.14E-10	chr:6:32648036
6	rs3129780	32647701	T	0.3801	3.14E-10	chr:6:32648036
6	rs7749043	32574952	T	3.141	3.16E-10	chr:6:32648036
6	rs9271976	32597617	C	0.3681	3.23E-10	chr:6:32648036
6	rs9271977	32597623	A	0.3681	3.23E-10	chr:6:32648036
6	rs6925580	32446203	C	2.877	3.27E-10	chr:6:32648036
6	rs281874819	32631428	C	2.306	3.32E-10	chr:6:32648036
6	rs537205676	32586383	T	3.89	3.37E-10	chr:6:32648036
6	rs34075049	32554935	A	3.293	3.48E-10	chr:6:32648036
6	rs9269205	32449851	T	0.3166	3.53E-10	chr:6:32648036
6	rs9273062	32612161	A	3.189	3.60E-10	chr:6:32648036
6	rs9273063	32612166	T	3.189	3.60E-10	chr:6:32648036
6	rs9271987	32597716	G	0.3588	3.61E-10	chr:6:32648036
6	rs9272666	32608737	C	2.259	3.66E-10	chr:6:32648036
6	rs527371188	32453819	T	3.174	3.75E-10	chr:6:32648036
6	rs41287333	32555046	T	3.748	3.78E-10	chr:6:32648036
6	rs9271985	32597679	T	0.3456	3.92E-10	chr:6:32648036
6	rs28383359	32606794	C	2.093	3.94E-10	chr:6:32648036
6	rs9273155	32613042	C	3.371	3.98E-10	chr:6:32648036
6	rs115591310	32511009	A	2.329	4.06E-10	chr:6:32648036
6	rs554768713	32444943	G	0.3515	4.06E-10	chr:6:32648036
6	rs9269130	32444940	T	0.3515	4.06E-10	chr:6:32648036
6	rs1048381	32610445	A	3.049	4.08E-10	chr:6:32648036
6	rs9275511	32674329	A	0.3599	4.10E-10	chr:6:32648036
6	rs74719989	32502664	A	2.144	4.17E-10	chr:6:32648036
6	rs116513958	32478809	G	3.669	4.22E-10	chr:6:32648036
6	rs13213605	32647764	C	0.4649	4.26E-10	chr:6:32648036
6	rs28366347	32564908	G	2.604	4.28E-10	chr:6:32648036
6	rs556025	32570880	T	2.783	4.35E-10	chr:6:32648036
6	rs146825681	32512918	G	4.549	4.42E-10	chr:6:32648036
6	rs9272195	32601830	G	0.4047	4.51E-10	chr:6:32648036
6	rs9271981	32597639	C	0.3598	4.69E-10	chr:6:32648036
6	rs77697582	32502517	T	2.281	4.91E-10	chr:6:32648036
6	rs9271385	32587409	A	0.3611	4.95E-10	chr:6:32648036
6	rs9271420	32587852	A	0.3464	4.96E-10	chr:6:32648036
6	rs28366349	32564916	T	2.592	4.97E-10	chr:6:32648036

6	rs28366350	32564918	A	2.592	4.97E-10	chr:6:32648036
6	rs574335595	32564914	G	2.592	4.97E-10	chr:6:32648036
6	rs3210168	32629146	A	2.827	5.11E-10	chr:6:32648036
6	rs9269037	32437958	T	0.3716	5.11E-10	chr:6:32648036
6	rs35359983	32555022	A	3.469	5.11E-10	chr:6:32648036
6	rs1049163	32629193	T	2.345	5.25E-10	chr:6:32648036
6	rs28672722	32626537	T	0.303	5.30E-10	chr:6:32648036
6	rs7748454	32448698	C	3.575	5.31E-10	chr:6:32648036
6	rs148416818	32850055	T	3.479	5.33E-10	chr:6:32648036
6	rs28752504	32569162	C	2.654	5.35E-10	chr:6:32648036
6	rs148395623	32524801	A	2.952	5.47E-10	chr:6:32648036
6	rs9271878	32595474	C	0.3908	5.54E-10	chr:6:32648036
6	rs201326238	32517754	T	3.507	5.66E-10	chr:6:32648036
6	rs111838566	32517727	G	3.049	5.75E-10	chr:6:32648036
6	rs112296425	32521984	G	3.386	5.76E-10	chr:6:32648036
6	rs28445646	32593952	G	2.34	5.77E-10	chr:6:32648036
6	rs9269210	32450037	C	0.3149	5.83E-10	chr:6:32648036
6	rs28366312	32561175	C	3.231	5.89E-10	chr:6:32648036
6	rs28366313	32561186	A	3.231	5.89E-10	chr:6:32648036
6	rs116585338	32512812	G	4.398	5.96E-10	chr:6:32648036
6	rs9274094	32630155	A	2.432	6.03E-10	chr:6:32648036
6	rs28479136	32625051	A	3.122	6.29E-10	chr:6:32648036
6	rs111403760	32517303	C	3.01	6.36E-10	chr:6:32648036
6	rs9271877	32595469	C	0.3929	6.55E-10	chr:6:32648036
6	rs115754308	32518466	T	2.927	6.56E-10	chr:6:32648036
6	rs28366306	32560990	A	3.232	6.62E-10	chr:6:32648036
6	rs6457598	32444475	T	0.3288	6.66E-10	chr:6:32648036
6	rs564014805	32614277	A	3.547	6.70E-10	chr:6:32648036
6	rs3129717	32659308	T	0.276	6.90E-10	chr:6:32648036
6	rs9271978	32597632	C	0.3743	6.93E-10	chr:6:32648036
6	rs35095850	32549935	G	3.271	6.94E-10	chr:6:32648036
6	rs111665695	32479429	G	2.213	7.05E-10	chr:6:32648036
6	rs74324877	32517782	C	2.977	7.10E-10	chr:6:32648036
6	rs9269020	32436934	G	2.301	7.12E-10	chr:6:32648036
6	rs79207037	32509294	A	2.909	7.14E-10	chr:6:32648036
6	rs563648725	32613130	G	3.618	7.15E-10	chr:6:32648036
6	rs9269070	32440451	G	0.3372	7.16E-10	chr:6:32648036
6	rs28366315	32561207	T	3.207	7.22E-10	chr:6:32648036
6	rs9273542	32628812	T	2.648	7.34E-10	chr:6:32648036
6	rs28366316	32561246	C	3.194	7.41E-10	chr:6:32648036
6	rs28366359	32565169	T	2.642	7.43E-10	chr:6:32648036
6	rs281875165	32628559	C	2.765	7.63E-10	chr:6:32648036
6	rs539555344	32628560	T	2.765	7.63E-10	chr:6:32648036

6	rs9273519	32628558	A	2.765	7.63E-10	chr:6:32648036
6	rs34902284	32556300	T	3.268	7.71E-10	chr:6:32648036
6	rs111343881	32521022	C	3.269	7.72E-10	chr:6:32648036
6	rs113459411	32521026	G	3.269	7.72E-10	chr:6:32648036
6	rs3135363	32389648	G	3.008	7.89E-10	chr:6:32648036
6	rs34835435	32549966	G	3.16	7.91E-10	chr:6:32648036
6	rs28772724	32509357	T	2.905	7.96E-10	chr:6:32648036
6	rs79957603	32509348	A	2.905	7.96E-10	chr:6:32648036
6	rs9269111	32443358	G	2.531	8.01E-10	chr:6:32648036
6	rs9271879	32595489	T	0.3987	8.02E-10	chr:6:32648036
6	rs79508322	32512049	A	2.93	8.14E-10	chr:6:32648036
6	rs534262225	32646454	T	0.3888	8.21E-10	chr:6:32648036
6	rs9271876	32595448	G	0.3928	8.29E-10	chr:6:32648036
6	rs28366358	32565164	G	2.636	8.31E-10	chr:6:32648036
6	rs149690859	32511135	A	2.447	8.32E-10	chr:6:32648036
6	rs28371177	32612990	T	5.603	8.36E-10	chr:6:32648036
6	rs143811457	32507112	A	2.142	8.40E-10	chr:6:32648036
6	rs116333937	32517381	T	3.005	8.43E-10	chr:6:32648036
6	rs111621461	32520050	T	2.938	9.01E-10	chr:6:32648036
6	rs115135140	32554855	G	3.264	9.19E-10	chr:6:32648036
6	rs2647026	32635705	T	5.534	9.20E-10	chr:6:32648036
6	rs9269071	32440467	T	0.3685	9.24E-10	chr:6:32648036
6	rs12206125	32575605	A	2.99	9.26E-10	chr:6:32648036
6	rs9269207	32449896	C	0.3035	9.27E-10	chr:6:32648036
6	rs7748293	32448622	T	0.3097	9.27E-10	chr:6:32648036
6	rs113528607	32500928	G	2.828	9.68E-10	chr:6:32648036
6	rs1130399	32629755	A	2.624	9.71E-10	chr:6:32648036
6	rs114954902	32517768	A	2.95	9.72E-10	chr:6:32648036
6	rs28366365	32565560	C	2.606	9.84E-10	chr:6:32648036
6	rs28366367	32565571	A	2.606	9.84E-10	chr:6:32648036
6	rs28383168	32565588	C	2.606	9.84E-10	chr:6:32648036
6	rs3135006	32667119	T	0.2813	9.87E-10	chr:6:32648036
6	rs74635045	32502745	C	2.109	9.87E-10	chr:6:32648036
6	rs9271447	32588357	A	2.697	1.01E-09	chr:6:32648036
6	rs9271448	32588368	A	2.697	1.01E-09	chr:6:32648036
6	rs35448211	32555067	T	3.657	1.01E-09	chr:6:32648036
6	rs35456626	32555066	A	3.657	1.01E-09	chr:6:32648036
6	rs9269147	32445650	A	2.546	1.03E-09	chr:6:32648036
6	rs9269148	32445658	T	2.546	1.03E-09	chr:6:32648036
6	rs6457544	32296057	T	2.772	1.03E-09	chr:6:32648036
6	rs7452864	32439077	T	0.3561	1.03E-09	chr:6:32648036
6	rs369952885	32539033	C	2.535	1.04E-09	chr:6:32648036
6	rs35239661	32549923	T	3.251	1.04E-09	chr:6:32648036

6	rs75635252	32509332	G	2.88	1.05E-09	chr:6:32648036
6	rs554350950	32522108	A	2.974	1.05E-09	chr:6:32648036
6	rs9271983	32597647	T	0.3731	1.05E-09	chr:6:32648036
6	rs281863739	32630610	A	2.34	1.07E-09	chr:6:32648036
6	rs9271866	32595367	T	0.3823	1.07E-09	chr:6:32648036
6	rs9271867	32595368	G	0.3823	1.07E-09	chr:6:32648036
6	rs547505909	32478806	T	3.597	1.07E-09	chr:6:32648036
6	rs550717566	32609094	T	2.586	1.07E-09	chr:6:32648036
6	rs116384190	32520337	G	2.911	1.08E-09	chr:6:32648036
6	rs189711091	32478943	C	2.92	1.09E-09	chr:6:32648036
6	rs370791576	32517082	A	2.926	1.09E-09	chr:6:32648036
6	rs28366304	32560981	G	3.176	1.10E-09	chr:6:32648036
6	rs28366305	32560982	T	3.176	1.10E-09	chr:6:32648036
6	rs9268944	32433654	T	0.4336	1.10E-09	chr:6:32648036
6	rs113049351	32511024	T	2.278	1.11E-09	chr:6:32648036
6	rs1987305	32401996	T	2.494	1.14E-09	chr:6:32648036
6	rs4348358	32399092	A	2.494	1.14E-09	chr:6:32648036
6	rs4612206	32404093	T	2.494	1.14E-09	chr:6:32648036
6	rs7753264	32402440	G	2.494	1.14E-09	chr:6:32648036
6	rs7756262	32398675	A	2.494	1.14E-09	chr:6:32648036
6	rs7773756	32402464	C	2.494	1.14E-09	chr:6:32648036
6	rs7774047	32398563	A	2.494	1.14E-09	chr:6:32648036
6	rs9268588	32397794	G	2.494	1.14E-09	chr:6:32648036
6	rs9268589	32398202	A	2.494	1.14E-09	chr:6:32648036
6	rs9268605	32399833	A	2.494	1.14E-09	chr:6:32648036
6	rs9268606	32400070	A	2.494	1.14E-09	chr:6:32648036
6	rs9268607	32400538	G	2.494	1.14E-09	chr:6:32648036
6	rs9268608	32400865	T	2.494	1.14E-09	chr:6:32648036
6	rs9268609	32401261	A	2.494	1.14E-09	chr:6:32648036
6	rs9268610	32401263	C	2.494	1.14E-09	chr:6:32648036
6	rs9268613	32402692	T	2.494	1.14E-09	chr:6:32648036
6	rs9268615	32402889	A	2.494	1.14E-09	chr:6:32648036
6	rs9268634	32406530	A	2.494	1.14E-09	chr:6:32648036
6	rs9268645	32408527	G	2.494	1.14E-09	chr:6:32648036
6	rs960736	32402225	C	2.494	1.14E-09	chr:6:32648036
6	rs9276010	32694686	T	4.744	1.14E-09	chr:6:32648036
6	rs111517012	32554675	G	3.143	1.14E-09	chr:6:32648036
6	rs35675330	32554653	A	3.143	1.14E-09	chr:6:32648036
6	rs9273480	32628103	T	2.854	1.15E-09	chr:6:32648036
6	rs3129782	32648719	G	0.2874	1.16E-09	chr:6:32648036
6	rs76752213	32509416	A	2.846	1.17E-09	chr:6:32648036
6	rs28366364	32565532	G	2.594	1.19E-09	chr:6:32648036
6	rs9273608	32629110	T	2.361	1.21E-09	chr:6:32648036

6	rs199882626	32517752	A	2.925	1.24E-09	chr:6:32648036
6	rs74768610	32509868	C	2.797	1.25E-09	chr:6:32648036
6	rs79215033	32509858	G	2.797	1.25E-09	chr:6:32648036
6	rs9270697	32567296	G	0.384	1.26E-09	chr:6:32648036
6	rs112654908	32519968	C	2.897	1.27E-09	chr:6:32648036
6	rs112956435	32519929	C	2.897	1.27E-09	chr:6:32648036
6	rs281874906	32631381	T	2.273	1.30E-09	chr:6:32648036
6	rs28366307	32560995	C	3.146	1.31E-09	chr:6:32648036
6	rs115630465	32482032	A	2.382	1.32E-09	chr:6:32648036
6	rs111797109	32511650	T	2.91	1.35E-09	chr:6:32648036
6	rs9271406	32587588	G	2.548	1.36E-09	chr:6:32648036
6	rs28723989	32550116	T	3.484	1.38E-09	chr:6:32648036
6	rs548548482	32608153	A	3.884	1.39E-09	chr:6:32648036
6	rs9272739	32609698	C	3.215	1.39E-09	chr:6:32648036
6	rs6935999	32392757	A	3.244	1.41E-09	chr:6:32648036
6	rs78668102	32507091	T	2.114	1.41E-09	chr:6:32648036
6	rs112796795	32505471	C	2.383	1.41E-09	chr:6:32648036
6	rs28366311	32561137	G	3.165	1.42E-09	chr:6:32648036
6	rs6457595	32444040	A	2.655	1.44E-09	chr:6:32648036
6	rs9271865	32595360	T	0.3885	1.45E-09	chr:6:32648036
6	rs28366314	32561201	G	3.144	1.47E-09	chr:6:32648036
6	rs28407950	32626348	T	0.3284	1.48E-09	chr:6:32648036
6	rs28515515	32626432	T	0.3284	1.48E-09	chr:6:32648036
6	rs28724200	32556248	G	3.459	1.49E-09	chr:6:32648036
6	rs111432098	32520940	G	2.89	1.50E-09	chr:6:32648036
6	rs9274129	32630230	C	2.335	1.50E-09	chr:6:32648036
6	rs111989182	32510924	G	2.237	1.51E-09	chr:6:32648036
6	rs113547679	32510914	T	2.237	1.51E-09	chr:6:32648036
6	rs181435903	32510903	A	2.237	1.51E-09	chr:6:32648036
6	rs9274464	32633489	T	0.3194	1.51E-09	chr:6:32648036
6	rs28366274	32560107	T	3.411	1.51E-09	chr:6:32648036
6	rs9276300	32703185	T	4.653	1.52E-09	chr:6:32648036
6	rs28732284	32548667	C	3.257	1.57E-09	chr:6:32648036
6	rs537501315	32516973	A	3.39	1.57E-09	chr:6:32648036
6	rs75420369	32511920	A	2.929	1.58E-09	chr:6:32648036
6	rs3129767	32594975	G	0.3095	1.59E-09	chr:6:32648036
6	rs28366302	32560934	C	3.093	1.60E-09	chr:6:32648036
6	rs181351497	32478944	C	2.887	1.62E-09	chr:6:32648036
6	rs9271862	32595333	C	0.3848	1.64E-09	chr:6:32648036
6	rs9271863	32595334	A	0.3848	1.64E-09	chr:6:32648036
6	rs535852	32577604	T	2.506	1.66E-09	chr:6:32648036
6	rs3129720	32663631	T	0.2903	1.67E-09	chr:6:32648036
6	rs113586210	32517050	T	2.896	1.72E-09	chr:6:32648036

6	rs35840159	32556332	G	3.157	1.73E-09	chr:6:32648036
6	rs9269056	32439123	T	0.3695	1.74E-09	chr:6:32648036
6	rs9271868	32595375	C	0.3243	1.75E-09	chr:6:32648036
6	rs9271869	32595376	A	0.3243	1.75E-09	chr:6:32648036
6	rs9271870	32595377	T	0.3243	1.75E-09	chr:6:32648036
6	rs116254278	32521312	A	2.929	1.78E-09	chr:6:32648036
6	rs113521434	32520460	T	2.862	1.80E-09	chr:6:32648036
6	rs113686014	32509551	A	2.862	1.80E-09	chr:6:32648036
6	rs202038991	32509105	C	2.926	1.81E-09	chr:6:32648036
6	rs565113427	32647098	C	0.414	1.82E-09	chr:6:32648036
6	rs9274150	32630276	G	2.295	1.82E-09	chr:6:32648036
6	rs78733108	32517511	G	2.874	1.83E-09	chr:6:32648036
6	rs28366328	32561766	G	3.08	1.85E-09	chr:6:32648036
6	rs28366356	32565091	T	2.56	1.90E-09	chr:6:32648036
6	rs80247743	32527206	G	2.613	1.90E-09	chr:6:32648036
6	rs28366345	32564896	G	2.499	1.92E-09	chr:6:32648036
6	rs28366346	32564898	G	2.499	1.92E-09	chr:6:32648036
6	rs3828805	32636120	T	0.3044	1.95E-09	chr:6:32648036
6	rs659244	32577033	C	2.475	1.98E-09	chr:6:32648036
6	rs3129719	32661779	T	0.2934	2.00E-09	chr:6:32648036
6	rs190411898	32511180	T	2.385	2.00E-09	chr:6:32648036
6	rs17612845	32620541	A	2.045	2.04E-09	chr:6:32648036
6	rs1049133	32629847	A	0.2929	2.09E-09	chr:6:32648036
6	rs9273841	32629582	C	0.2929	2.09E-09	chr:6:32648036
6	rs78182729	32502322	T	2.344	2.10E-09	chr:6:32648036
6	rs2621320	32790940	A	2.582	2.14E-09	chr:6:32648036
6	rs28724260	32630240	G	2.311	2.14E-09	chr:6:32648036
6	rs115872586	32604933	A	3.939	2.17E-09	chr:6:32648036
6	rs11966146	32487739	A	3.215	2.17E-09	chr:6:32648036
6	rs1135085	32522477	C	3.823	2.18E-09	chr:6:32648036
6	rs28366300	32560875	T	3.153	2.19E-09	chr:6:32648036
6	rs28366301	32560883	A	3.153	2.19E-09	chr:6:32648036
6	rs375350961	32521793	G	2.966	2.21E-09	chr:6:32648036
6	rs28366317	32561411	T	3.019	2.25E-09	chr:6:32648036
6	rs59350153	32509935	T	2.787	2.29E-09	chr:6:32648036
6	rs113961302	32514219	G	2.833	2.33E-09	chr:6:32648036
6	rs3200830	32520774	T	2.833	2.33E-09	chr:6:32648036
6	rs34481656	32501664	G	2.833	2.33E-09	chr:6:32648036
6	rs62405598	32513708	C	2.833	2.33E-09	chr:6:32648036
6	rs62405629	32513885	T	2.833	2.33E-09	chr:6:32648036
6	rs75170544	32513285	T	2.833	2.33E-09	chr:6:32648036
6	rs78450703	32513216	T	2.833	2.33E-09	chr:6:32648036
6	rs9271461	32588500	A	2.621	2.33E-09	chr:6:32648036

6	rs574642751	32525837	T	3.194	2.33E-09	chr:6:32648036
6	rs536810	32577497	T	2.483	2.36E-09	chr:6:32648036
6	rs147317423	32516905	T	2.87	2.40E-09	chr:6:32648036
6	rs189139309	32516915	G	2.87	2.40E-09	chr:6:32648036
6	rs190685297	32458514	C	2.97	2.42E-09	chr:6:32648036
6	rs9269054	32439120	T	0.3745	2.45E-09	chr:6:32648036
6	rs112692718	32685027	T	3.248	2.46E-09	chr:6:32648036
6	rs3828849	32520664	A	2.824	2.48E-09	chr:6:32648036
6	rs34127991	32559131	A	2.953	2.49E-09	chr:6:32648036
6	rs34788621	32559124	C	2.953	2.49E-09	chr:6:32648036
6	rs281863362	32631133	T	2.152	2.49E-09	chr:6:32648036
6	rs62405638	32516715	T	2.833	2.52E-09	chr:6:32648036
6	rs62405639	32516724	C	2.833	2.52E-09	chr:6:32648036
6	rs3129771	32597202	G	0.2911	2.58E-09	chr:6:32648036
6	rs546788946	32511223	A	2.373	2.62E-09	chr:6:32648036
6	rs34614226	32554963	A	3.034	2.65E-09	chr:6:32648036
6	rs9269016	32436670	G	0.4189	2.66E-09	chr:6:32648036
6	rs114277609	32478731	T	2.432	2.66E-09	chr:6:32648036
6	rs28724031	32550819	A	3.215	2.66E-09	chr:6:32648036
6	rs202015091	32520214	C	2.828	2.68E-09	chr:6:32648036
6	rs28383183	32571829	A	2.971	2.69E-09	chr:6:32648036
6	rs9270891	32571828	G	2.971	2.69E-09	chr:6:32648036
6	rs9270892	32571830	T	2.971	2.69E-09	chr:6:32648036
6	rs28366299	32560870	A	3.128	2.72E-09	chr:6:32648036
6	rs9274626	32636040	T	0.3031	2.80E-09	chr:6:32648036
6	rs586610	32572311	C	3.042	2.80E-09	chr:6:32648036
6	rs77789453	32512013	C	2.913	2.81E-09	chr:6:32648036
6	rs540912133	32553011	G	3.388	2.83E-09	chr:6:32648036
6	rs573504840	32553010	T	3.388	2.83E-09	chr:6:32648036
6	rs9269068	32440270	A	0.3384	2.84E-09	chr:6:32648036
6	rs151027857	32521366	C	3.067	2.84E-09	chr:6:32648036
6	rs4713534	32445926	T	2.869	2.85E-09	chr:6:32648036
6	rs552189104	32485744	T	3.018	2.85E-09	chr:6:32648036
6	rs9273494	32628256	T	2.734	2.88E-09	chr:6:32648036
6	rs9273154	32613028	A	3.036	2.90E-09	chr:6:32648036
6	rs113484779	32514162	T	2.797	2.91E-09	chr:6:32648036
6	rs9273127	32612762	A	3.154	2.94E-09	chr:6:32648036
6	rs6457543	32296029	G	2.646	2.96E-09	chr:6:32648036
6	rs1049055	32634387	C	0.3206	2.97E-09	chr:6:32648036
6	rs36172419	32507766	A	2.147	2.98E-09	chr:6:32648036
6	rs9273093	32612514	A	3.401	2.99E-09	chr:6:32648036
6	rs34004019	32626403	G	0.3397	3.01E-09	chr:6:32648036
6	rs34983241	32626397	T	0.3397	3.01E-09	chr:6:32648036

6	rs568483267	32626392	C	0.3397	3.01E-09	chr:6:32648036
6	rs9273070	32612241	T	3.12	3.01E-09	chr:6:32648036
6	rs281874937	32631345	C	2.217	3.06E-09	chr:6:32648036
6	rs28366323	32561609	G	3.084	3.11E-09	chr:6:32648036
6	rs77506702	32518704	G	2.784	3.14E-09	chr:6:32648036
6	rs3830058	32634010	G	0.3065	3.16E-09	chr:6:32648036
6	rs9273079	32612326	T	3.2	3.17E-09	chr:6:32648036
6	rs9273103	32612576	C	3.447	3.19E-09	chr:6:32648036
6	rs28366310	32561128	C	3.076	3.25E-09	chr:6:32648036
6	rs9274339	32632319	A	1.851	3.28E-09	chr:6:32648036
6	rs9269073	32440771	G	2.615	3.31E-09	chr:6:32648036
6	rs79136305	32505292	G	2.255	3.31E-09	chr:6:32648036
6	rs77495163	32517859	G	2.757	3.37E-09	chr:6:32648036
6	rs116519496	32607762	T	3.809	3.38E-09	chr:6:32648036
6	rs74958579	32459971	T	2.977	3.38E-09	chr:6:32648036
6	rs76870762	32459972	G	2.977	3.38E-09	chr:6:32648036
6	rs9273073	32612279	C	3.117	3.40E-09	chr:6:32648036
6	rs552085814	32554985	T	4.222	3.43E-09	chr:6:32648036
6	rs57588792	32510012	A	2.774	3.44E-09	chr:6:32648036
6	rs28732302	32548855	C	3.015	3.45E-09	chr:6:32648036
6	rs115712530	32517414	A	2.898	3.54E-09	chr:6:32648036
6	rs77594561	32501273	G	2.816	3.54E-09	chr:6:32648036
6	rs77604461	32501282	C	2.816	3.54E-09	chr:6:32648036
6	rs28680981	32606579	A	2.189	3.57E-09	chr:6:32648036
6	rs111484816	32509034	G	2.824	3.57E-09	chr:6:32648036
6	rs115622534	32553023	G	3.299	3.64E-09	chr:6:32648036
6	rs138825368	32518375	A	2.782	3.65E-09	chr:6:32648036
6	rs559236302	32486992	G	2.709	3.68E-09	chr:6:32648036
6	rs3104376	32600003	T	0.3072	3.69E-09	chr:6:32648036
6	rs9269067	32440242	G	0.339	3.77E-09	chr:6:32648036
6	rs77891198	32506670	A	2	3.79E-09	chr:6:32648036
6	rs62405631	32514500	C	2.764	3.80E-09	chr:6:32648036
6	rs9276179	32699090	A	2.442	3.93E-09	chr:6:32648036
6	rs532385	32195359	A	0.398	3.95E-09	chr:6:32648036
6	rs370787440	32521596	G	2.798	3.96E-09	chr:6:32648036
6	rs555862602	32565799	G	12.41	3.97E-09	chr:6:32648036
6	rs9269202	32449523	T	0.35	3.98E-09	chr:6:32648036
6	rs9273217	32613747	G	1.929	3.99E-09	chr:6:32648036
6	rs9273133	32612801	C	3.054	4.00E-09	chr:6:32648036
6	rs532162239	32558725	T	3.222	4.01E-09	chr:6:32648036
6	rs547202137	32558728	A	3.222	4.01E-09	chr:6:32648036
6	rs140215683	32607806	T	3.99	4.04E-09	chr:6:32648036
6	rs145266520	32607812	G	3.99	4.04E-09	chr:6:32648036

6	rs528153410	32507211	G	2.047	4.05E-09	chr:6:32648036
6	rs28383169	32565652	G	2.339	4.05E-09	chr:6:32648036
6	rs9273129	32612774	A	3.132	4.07E-09	chr:6:32648036
6	rs9273130	32612775	A	3.132	4.07E-09	chr:6:32648036
6	rs1049225	32627747	A	0.3054	4.18E-09	chr:6:32648036
6	rs34282199	32556218	T	3.281	4.23E-09	chr:6:32648036
6	rs3135001	32670136	T	0.2998	4.33E-09	chr:6:32648036
6	rs62405630	32513942	T	2.781	4.34E-09	chr:6:32648036
6	rs34594152	32556124	C	4.015	4.35E-09	chr:6:32648036
6	rs34756259	32556121	T	4.015	4.35E-09	chr:6:32648036
6	rs9274488	32633852	A	0.3799	4.37E-09	chr:6:32648036
6	rs2894257	32433276	G	0.2577	4.40E-09	chr:6:32648036
6	rs79890132	32517668	A	2.756	4.42E-09	chr:6:32648036
6	rs9268833	32428062	T	2.688	4.46E-09	chr:6:32648036
6	rs9268834	32428079	A	2.688	4.46E-09	chr:6:32648036
6	rs9268835	32428115	A	2.688	4.46E-09	chr:6:32648036
6	rs2395196	32450154	T	6.182	4.48E-09	chr:6:32648036
6	rs3828851	32520621	A	2.767	4.48E-09	chr:6:32648036
6	rs557371502	32520639	A	2.767	4.48E-09	chr:6:32648036
6	rs9274490	32633879	G	2.707	4.52E-09	chr:6:32648036
6	rs76207819	32507365	G	1.997	4.54E-09	chr:6:32648036
6	rs111410640	32521831	A	2.773	4.59E-09	chr:6:32648036
6	rs9271997	32597801	A	0.3665	4.59E-09	chr:6:32648036
6	rs202187184	32519848	C	2.803	4.61E-09	chr:6:32648036
6	rs530790448	32519846	T	2.803	4.61E-09	chr:6:32648036
6	rs17612942	32620999	G	1.914	4.72E-09	chr:6:32648036
6	rs559062197	32452802	T	0.306	4.73E-09	chr:6:32648036
6	rs9272071	32599487	C	0.3805	4.92E-09	chr:6:32648036
6	rs9394106	32620232	G	2.055	5.01E-09	chr:6:32648036
6	rs112360905	32509019	G	2.786	5.05E-09	chr:6:32648036
6	rs138925158	32493114	A	3.309	5.05E-09	chr:6:32648036
6	rs9273102	32612566	C	3.4	5.06E-09	chr:6:32648036
6	rs28732291	32548697	G	3.031	5.08E-09	chr:6:32648036
6	rs58537387	32510176	T	2.365	5.24E-09	chr:6:32648036
6	rs9269160	32446711	C	2.427	5.27E-09	chr:6:32648036
6	rs3135190	32667946	T	0.303	5.28E-09	chr:6:32648036
6	rs3891176	32634318	A	2.718	5.35E-09	chr:6:32648036
6	rs3134966	32674298	T	0.3035	5.39E-09	chr:6:32648036
6	rs75928817	32559212	G	2.879	5.41E-09	chr:6:32648036
6	rs9273219	32613758	A	1.919	5.43E-09	chr:6:32648036
6	rs3134997	32672039	T	0.3034	5.54E-09	chr:6:32648036
6	rs201150249	32522103	C	2.752	5.54E-09	chr:6:32648036
6	rs9273128	32612769	C	3.08	5.59E-09	chr:6:32648036

6	rs112158360	32514050	G	2.794	5.60E-09	chr:6:32648036
6	rs112587701	32514041	A	2.794	5.60E-09	chr:6:32648036
6	rs206017	32176211	G	2.347	5.69E-09	chr:6:32648036
6	rs3130290	32174957	T	2.347	5.69E-09	chr:6:32648036
6	rs9273071	32612251	G	3.008	5.73E-09	chr:6:32648036
6	rs646984	32576592	C	2.407	5.84E-09	chr:6:32648036
6	rs9273668	32629237	G	2.509	5.85E-09	chr:6:32648036
6	rs114580448	32507538	T	2.157	5.93E-09	chr:6:32648036
6	rs138296677	32713598	C	3.68	5.95E-09	chr:6:32648036
6	rs79905009	32479493	G	2.116	5.97E-09	chr:6:32648036
6	rs111757290	32517856	T	3.403	6.07E-09	chr:6:32648036
6	rs77048791	32503986	T	2.62	6.09E-09	chr:6:32648036
6	rs9271667	32592710	C	0.3626	6.11E-09	chr:6:32648036
6	rs9378165	32510651	G	2.623	6.12E-09	chr:6:32648036
6	rs9274637	32636137	A	0.3289	6.19E-09	chr:6:32648036
6	rs373804788	32521591	T	2.775	6.23E-09	chr:6:32648036
6	rs562347537	32446227	A	0.243	6.31E-09	chr:6:32648036
6	rs28366319	32561495	A	2.928	6.36E-09	chr:6:32648036
6	rs1894411	32792973	C	2.498	6.40E-09	chr:6:32648036
6	rs76270534	32497232	G	2.418	6.47E-09	chr:6:32648036
6	rs200930495	32612460	A	3.218	6.48E-09	chr:6:32648036
6	rs28746993	32654030	C	0.3332	6.50E-09	chr:6:32648036
6	rs111899294	32517912	C	3.071	6.54E-09	chr:6:32648036
6	rs546722932	32507223	T	2.027	6.56E-09	chr:6:32648036
6	rs77794095	32502471	T	2.023	6.71E-09	chr:6:32648036
6	rs28366294	32560715	A	2.97	6.71E-09	chr:6:32648036
6	rs28366295	32560716	C	2.97	6.71E-09	chr:6:32648036
6	rs28366296	32560727	A	2.97	6.71E-09	chr:6:32648036
6	rs281863349	32631152	A	2.1	6.75E-09	chr:6:32648036
6	rs28366303	32560963	G	2.948	6.85E-09	chr:6:32648036
6	rs436388	32186264	T	2.355	6.90E-09	chr:6:32648036
6	rs17210258	32556130	T	3.889	6.97E-09	chr:6:32648036
6	rs2857107	32785515	T	2.498	7.00E-09	chr:6:32648036
6	rs10807106	32450851	G	2.736	7.03E-09	chr:6:32648036
6	rs28366327	32561678	A	2.96	7.06E-09	chr:6:32648036
6	rs281863514	32630930	G	0.3547	7.10E-09	chr:6:32648036
6	rs9270092	32554420	T	0.3207	7.33E-09	chr:6:32648036
6	rs113096063	32503161	T	2.579	7.35E-09	chr:6:32648036
6	rs113351668	32503167	A	2.579	7.35E-09	chr:6:32648036
6	rs28533694	32587235	T	0.3767	7.37E-09	chr:6:32648036
6	rs28383364	32606912	G	2.388	7.37E-09	chr:6:32648036
6	rs9273104	32612595	T	3.289	7.40E-09	chr:6:32648036
6	rs9271703	32593280	T	0.3606	7.47E-09	chr:6:32648036

6	rs75876652	32505224	T	2.351	7.54E-09	chr:6:32648036
6	rs115885380	32502972	A	2.247	7.59E-09	chr:6:32648036
6	rs116299636	32502974	C	2.247	7.59E-09	chr:6:32648036
6	rs9271708	32593386	A	0.3507	7.60E-09	chr:6:32648036
6	rs9271709	32593392	A	0.3507	7.60E-09	chr:6:32648036
6	rs114680412	32520248	A	2.764	7.66E-09	chr:6:32648036
6	rs115706291	32520256	A	2.764	7.66E-09	chr:6:32648036
6	rs6457663	32739312	A	4.397	7.72E-09	chr:6:32648036
6	rs6919798	32732890	G	4.397	7.72E-09	chr:6:32648036
6	rs557630457	32554837	T	3.183	7.73E-09	chr:6:32648036
6	rs576005883	32554840	T	3.183	7.73E-09	chr:6:32648036
6	rs28724164	32555762	C	3.56	7.76E-09	chr:6:32648036
6	rs9271925	32596540	G	0.3332	7.95E-09	chr:6:32648036
6	rs9271926	32596566	T	0.3332	7.95E-09	chr:6:32648036
6	rs35399335	32556338	G	2.841	8.02E-09	chr:6:32648036
6	rs540527828	32459250	T	2.961	8.17E-09	chr:6:32648036
6	rs9273108	32612626	T	3.215	8.20E-09	chr:6:32648036
6	rs62405591	32508704	A	2.711	8.22E-09	chr:6:32648036
6	rs9273786	32629462	A	2.562	8.40E-09	chr:6:32648036
6	rs9271618	32591752	A	0.3673	8.58E-09	chr:6:32648036
6	rs281863226	32631563	T	0.3618	8.81E-09	chr:6:32648036
6	rs4367411	32571929	T	2.45	8.84E-09	chr:6:32648036
6	rs28366298	32560859	C	2.956	8.87E-09	chr:6:32648036
6	rs116752065	32482564	G	3.098	8.89E-09	chr:6:32648036
6	rs28746817	32633287	T	2.199	8.97E-09	chr:6:32648036
6	rs192090456	32482101	C	2.065	8.98E-09	chr:6:32648036
6	rs113154956	32537479	C	5.925	9.04E-09	chr:6:32648036
6	rs9271894	32595874	T	0.3665	9.20E-09	chr:6:32648036
6	rs9273111	32612643	A	3.142	9.29E-09	chr:6:32648036
6	rs74498391	32505267	C	2.206	9.37E-09	chr:6:32648036
6	rs78543540	32457762	A	2.466	9.48E-09	chr:6:32648036
6	rs79044903	32457771	A	2.466	9.48E-09	chr:6:32648036
6	rs115466102	32507168	C	1.981	9.55E-09	chr:6:32648036
6	rs115272081	32519782	A	2.782	9.59E-09	chr:6:32648036
6	rs115888968	32521309	G	2.63	9.59E-09	chr:6:32648036
6	rs9273110	32612633	C	3.13	9.61E-09	chr:6:32648036
6	rs9273107	32612620	G	3.172	9.72E-09	chr:6:32648036
6	rs9269129	32444938	G	0.267	9.76E-09	chr:6:32648036
6	rs9273660	32629227	T	2.482	9.77E-09	chr:6:32648036
6	rs67612510	32526436	A	2.19	9.80E-09	chr:6:32648036
6	rs3129726	32678206	G	0.3166	1.01E-08	chr:6:32648036
6	rs9271376	32587113	G	0.3266	1.01E-08	chr:6:32648036
6	rs647035	32576626	A	2.372	1.01E-08	chr:6:32648036

6	rs9273403	32627159	T	1.915	1.01E-08	chr:6:32648036
6	rs9271662	32592628	A	0.3446	1.01E-08	chr:6:32648036
6	rs9269204	32449797	C	0.3331	1.01E-08	chr:6:32648036
6	rs28366325	32561659	C	2.936	1.02E-08	chr:6:32648036
6	rs534964517	32517795	C	3.421	1.02E-08	chr:6:32648036
6	rs570640158	32517793	C	3.421	1.02E-08	chr:6:32648036
6	rs551251088	32507235	A	2.013	1.03E-08	chr:6:32648036
6	rs112895453	32510602	G	2.588	1.05E-08	chr:6:32648036
6	rs114645331	32554776	T	3.194	1.05E-08	chr:6:32648036
6	rs281863522	32630920	C	2.282	1.06E-08	chr:6:32648036
6	rs78294251	32503336	A	2.238	1.06E-08	chr:6:32648036
6	rs9271733	32593633	A	0.3382	1.07E-08	chr:6:32648036
6	rs41270929	32630868	T	0.3578	1.07E-08	chr:6:32648036
6	rs113823631	32553037	T	3.127	1.08E-08	chr:6:32648036
6	rs113623173	32510438	T	2.37	1.08E-08	chr:6:32648036
6	rs536189184	32558823	A	3.031	1.11E-08	chr:6:32648036
6	rs9273539	32628779	T	2.488	1.11E-08	chr:6:32648036
6	rs138087636	32517031	T	2.729	1.12E-08	chr:6:32648036
6	rs185282713	32517018	C	2.729	1.12E-08	chr:6:32648036
6	rs28772272	32656980	T	0.3494	1.12E-08	chr:6:32648036
6	rs9378274	32656983	A	0.3494	1.12E-08	chr:6:32648036
6	rs9391891	32656975	A	0.3494	1.12E-08	chr:6:32648036
6	rs9275642	32684935	C	0.4377	1.12E-08	chr:6:32648036
6	rs75211336	32502934	G	2.202	1.15E-08	chr:6:32648036
6	rs28366267	32559781	C	2.736	1.15E-08	chr:6:32648036
6	rs9273807	32629514	A	2.523	1.19E-08	chr:6:32648036
6	rs35081742	32553608	T	3.208	1.19E-08	chr:6:32648036
6	rs74587162	32509455	C	2.647	1.19E-08	chr:6:32648036
6	rs4570006	32657257	T	0.3529	1.21E-08	chr:6:32648036
6	rs17210293	32556165	T	3.504	1.22E-08	chr:6:32648036
6	rs74791653	32499185	T	1.937	1.24E-08	chr:6:32648036
6	rs9273402	32627155	A	1.908	1.24E-08	chr:6:32648036
6	rs71549252	32501613	C	2.904	1.24E-08	chr:6:32648036
6	rs114969562	32594299	A	0.3406	1.26E-08	chr:6:32648036
6	rs71549253	32501882	C	2.826	1.27E-08	chr:6:32648036
6	rs71549254	32501887	G	2.826	1.27E-08	chr:6:32648036
6	rs1626993	32636991	T	2.217	1.28E-08	chr:6:32648036
6	rs9273229	32613914	C	1.895	1.28E-08	chr:6:32648036
6	rs35971290	32550235	G	3.234	1.29E-08	chr:6:32648036
6	rs9273498	32628301	T	0.3316	1.30E-08	chr:6:32648036
6	rs9469195	32585012	G	0.2798	1.33E-08	chr:6:32648036
6	rs538187149	32591690	C	0.3177	1.33E-08	chr:6:32648036
6	rs74323244	32503921	C	2.544	1.33E-08	chr:6:32648036

6	rs9271694	32593051	C	0.3724	1.33E-08	chr:6:32648036
6	rs28366256	32559499	G	2.994	1.34E-08	chr:6:32648036
6	rs28746964	32653083	G	0.3523	1.34E-08	chr:6:32648036
6	rs114031016	32520035	T	3.093	1.34E-08	chr:6:32648036
6	rs62405643	32517807	C	0.3993	1.34E-08	chr:6:32648036
6	rs111680800	32507452	A	2.053	1.35E-08	chr:6:32648036
6	rs111897614	32503177	T	2.54	1.35E-08	chr:6:32648036
6	rs7749092	32449050	T	0.3414	1.37E-08	chr:6:32648036
6	rs1049213	32627773	A	0.331	1.40E-08	chr:6:32648036
6	rs17212104	32650883	G	0.3528	1.41E-08	chr:6:32648036
6	rs140590517	32518282	A	2.652	1.41E-08	chr:6:32648036
6	rs34977583	32555377	A	3.935	1.45E-08	chr:6:32648036
6	rs9273391	32627043	G	4.651	1.47E-08	chr:6:32648036
6	rs17212279	32651713	G	0.3515	1.50E-08	chr:6:32648036
6	rs9272608	32607737	C	3.258	1.50E-08	chr:6:32648036
6	rs9271623	32591914	C	0.377	1.54E-08	chr:6:32648036
6	rs113141487	32524887	G	2.959	1.55E-08	chr:6:32648036
6	rs28891493	32662509	T	0.355	1.55E-08	chr:6:32648036
6	rs9276557	32723614	T	4.201	1.55E-08	chr:6:32648036
6	rs9274514	32634243	A	2.622	1.55E-08	chr:6:32648036
6	rs77122291	32559019	A	2.674	1.64E-08	chr:6:32648036
6	rs28366288	32560432	T	2.768	1.65E-08	chr:6:32648036
6	rs9268945	32433701	T	0.4494	1.65E-08	chr:6:32648036
6	rs113182435	32518236	A	2.632	1.65E-08	chr:6:32648036
6	rs9273504	32628407	C	2.618	1.65E-08	chr:6:32648036
6	rs28747006	32654453	T	0.3565	1.66E-08	chr:6:32648036
6	rs28747007	32654475	A	0.3565	1.66E-08	chr:6:32648036
6	rs9271630	32591989	G	0.3761	1.66E-08	chr:6:32648036
6	rs28747028	32654989	T	0.3568	1.66E-08	chr:6:32648036
6	rs79476956	32508173	G	1.997	1.68E-08	chr:6:32648036
6	rs17205696	32650587	T	0.3594	1.68E-08	chr:6:32648036
6	rs13208475	32583255	T	2.632	1.69E-08	chr:6:32648036
6	rs13208697	32583274	T	2.632	1.69E-08	chr:6:32648036
6	rs17210265	32556141	A	3.691	1.70E-08	chr:6:32648036
6	rs547758749	32558755	T	3.043	1.70E-08	chr:6:32648036
6	rs28746963	32653079	T	0.3556	1.71E-08	chr:6:32648036
6	rs116813230	32524784	A	2.778	1.72E-08	chr:6:32648036
6	rs112177836	32503658	A	2.527	1.76E-08	chr:6:32648036
6	rs28746910	32649127	G	0.3571	1.76E-08	chr:6:32648036
6	rs75046889	32507835	C	3.103	1.77E-08	chr:6:32648036
6	rs28746994	32654094	A	0.3483	1.79E-08	chr:6:32648036
6	rs28366258	32559513	C	2.985	1.81E-08	chr:6:32648036
6	rs554943670	32516971	G	2.696	1.82E-08	chr:6:32648036

6	rs6901665	32442167	G	2.863	1.86E-08	chr:6:32648036
6	rs149253766	32507305	T	1.974	1.88E-08	chr:6:32648036
6	rs28746916	32649579	A	0.3603	1.89E-08	chr:6:32648036
6	rs9271900	32596013	G	0.3756	1.94E-08	chr:6:32648036
6	rs113654440	32596946	A	0.3773	1.95E-08	chr:6:32648036
6	rs28724149	32555334	G	3.849	1.95E-08	chr:6:32648036
6	rs17212062	32650648	G	0.3603	2.01E-08	chr:6:32648036
6	rs17212069	32650667	G	0.3603	2.01E-08	chr:6:32648036
6	rs17212090	32650809	T	0.3603	2.01E-08	chr:6:32648036
6	rs4538747	32657334	A	0.3603	2.01E-08	chr:6:32648036
6	rs75375346	32596908	C	0.3768	2.01E-08	chr:6:32648036
6	rs9273803	32629504	T	2.48	2.05E-08	chr:6:32648036
6	rs3828829	32521233	T	3.296	2.06E-08	chr:6:32648036
6	rs9272002	32597924	G	0.4002	2.07E-08	chr:6:32648036
6	rs9272003	32597940	G	0.4002	2.07E-08	chr:6:32648036
6	rs9273536	32628764	T	2.447	2.08E-08	chr:6:32648036
6	rs534819727	32481692	T	3.068	2.10E-08	chr:6:32648036
6	rs376271339	32517804	C	3.277	2.10E-08	chr:6:32648036
6	rs17202787	32428397	A	4.304	2.12E-08	chr:6:32648036
6	rs28752487	32417433	T	4.304	2.12E-08	chr:6:32648036
6	rs28752488	32419006	A	4.304	2.12E-08	chr:6:32648036
6	rs62405588	32508468	G	2.205	2.13E-08	chr:6:32648036
6	rs78539884	32513224	A	2.594	2.16E-08	chr:6:32648036
6	rs116427677	32493456	T	2.508	2.16E-08	chr:6:32648036
6	rs206018	32177880	G	2.421	2.16E-08	chr:6:32648036
6	rs149038762	32475948	A	1.915	2.18E-08	chr:6:32648036
6	rs3129299	32900787	T	2.268	2.19E-08	chr:6:32648036
6	rs111409670	32518069	C	2.682	2.19E-08	chr:6:32648036
6	rs9273873	32629650	C	2.595	2.20E-08	chr:6:32648036
6	rs9273902	32629716	C	2.595	2.20E-08	chr:6:32648036
6	rs9271979	32597635	C	0.3463	2.22E-08	chr:6:32648036
6	rs9273695	32629281	T	2.455	2.23E-08	chr:6:32648036
6	rs9273696	32629282	T	2.455	2.23E-08	chr:6:32648036
6	rs9348889	32620255	C	2.722	2.24E-08	chr:6:32648036
6	rs9348890	32620256	C	2.722	2.24E-08	chr:6:32648036
6	rs116432881	32514385	G	2.688	2.26E-08	chr:6:32648036
6	rs9271654	32592565	A	0.3518	2.28E-08	chr:6:32648036
6	rs9271657	32592582	C	0.3518	2.28E-08	chr:6:32648036
6	rs9405042	32657020	G	0.3622	2.34E-08	chr:6:32648036
6	rs541764084	32522223	G	3.585	2.34E-08	chr:6:32648036
6	rs281863686	32630689	C	2.092	2.35E-08	chr:6:32648036
6	rs281863689	32630684	T	2.092	2.35E-08	chr:6:32648036
6	rs2023222	32194083	T	2.299	2.36E-08	chr:6:32648036

6	rs3132950	32194740	C	2.299	2.36E-08	chr:6:32648036
6	rs9267845	32193698	A	2.299	2.36E-08	chr:6:32648036
6	rs17205717	32650746	C	0.3623	2.37E-08	chr:6:32648036
6	rs17212076	32650724	G	0.3623	2.37E-08	chr:6:32648036
6	rs28746903	32648768	T	0.3641	2.37E-08	chr:6:32648036
6	rs28746904	32648773	C	0.3641	2.37E-08	chr:6:32648036
6	rs28746936	32651393	A	0.3574	2.38E-08	chr:6:32648036
6	rs28746937	32651401	T	0.3574	2.38E-08	chr:6:32648036
6	rs79005639	32510582	C	2.515	2.39E-08	chr:6:32648036
6	rs75924712	32506761	A	2.252	2.40E-08	chr:6:32648036
6	rs75387045	32508213	T	2.004	2.42E-08	chr:6:32648036
6	rs537206245	32514754	C	2.636	2.46E-08	chr:6:32648036
6	rs539579029	32514764	T	2.636	2.46E-08	chr:6:32648036
6	rs28746970	32653259	C	0.3587	2.51E-08	chr:6:32648036
6	rs113712140	32485972	G	3.186	2.53E-08	chr:6:32648036
6	rs9271972	32597567	T	0.3838	2.53E-08	chr:6:32648036
6	rs113938016	32517898	C	3.165	2.55E-08	chr:6:32648036
6	rs4587163	32593167	T	2.313	2.55E-08	chr:6:32648036
6	rs28747004	32654379	A	0.3625	2.55E-08	chr:6:32648036
6	rs28747005	32654401	G	0.3625	2.55E-08	chr:6:32648036
6	rs28772280	32673899	T	0.3626	2.56E-08	chr:6:32648036
6	rs28772285	32674380	T	0.3626	2.56E-08	chr:6:32648036
6	rs28772286	32674385	C	0.3626	2.56E-08	chr:6:32648036
6	rs28772287	32674400	C	0.3626	2.56E-08	chr:6:32648036
6	rs28437335	32657644	C	0.363	2.56E-08	chr:6:32648036
6	rs9405119	32657681	G	0.363	2.56E-08	chr:6:32648036
6	rs9273698	32629289	A	2.439	2.57E-08	chr:6:32648036
6	rs3104391	32594901	T	0.3439	2.58E-08	chr:6:32648036
6	rs28746996	32654214	C	0.3482	2.60E-08	chr:6:32648036
6	rs28746912	32649451	G	0.3644	2.64E-08	chr:6:32648036
6	rs146702867	32524800	G	2.625	2.65E-08	chr:6:32648036
6	rs28675927	32603488	G	0.355	2.66E-08	chr:6:32648036
6	rs281863331	32631178	A	2.046	2.68E-08	chr:6:32648036
6	rs551468481	32518258	G	2.594	2.68E-08	chr:6:32648036
6	rs75658193	32503375	A	2.156	2.69E-08	chr:6:32648036
6	rs17205738	32650762	G	0.3639	2.71E-08	chr:6:32648036
6	rs62405574	32458246	G	2.544	2.71E-08	chr:6:32648036
6	rs535188034	32558790	T	2.946	2.72E-08	chr:6:32648036
6	rs553643750	32558791	G	2.946	2.72E-08	chr:6:32648036
6	rs28772284	32674313	T	0.3633	2.77E-08	chr:6:32648036
6	rs28746911	32649200	C	0.3641	2.78E-08	chr:6:32648036
6	rs28891483	32649254	T	0.3641	2.78E-08	chr:6:32648036
6	rs28891484	32649261	A	0.3641	2.78E-08	chr:6:32648036

6	rs28891485	32649274	T	0.3641	2.78E-08	chr:6:32648036
6	rs28747026	32654860	G	0.3624	2.80E-08	chr:6:32648036
6	rs28746939	32651416	C	0.3596	2.83E-08	chr:6:32648036
6	rs111875855	32489668	T	3.339	2.84E-08	chr:6:32648036
6	rs28746971	32653272	A	0.3617	2.87E-08	chr:6:32648036
6	rs411326	32211317	T	2.284	2.89E-08	chr:6:32648036
6	rs9271887	32595679	A	0.3814	2.90E-08	chr:6:32648036
6	rs28366339	32564725	G	2.382	2.91E-08	chr:6:32648036
6	rs28366340	32564727	G	2.382	2.91E-08	chr:6:32648036
6	rs28724146	32555303	G	3.809	2.92E-08	chr:6:32648036
6	rs9271738	32593691	G	0.3427	2.92E-08	chr:6:32648036
6	rs35130701	32594199	G	0.3843	2.95E-08	chr:6:32648036
6	rs5020949	32449974	T	3.028	2.97E-08	chr:6:32648036
6	rs28746966	32653145	C	0.3642	3.00E-08	chr:6:32648036
6	rs33927408	32550420	T	3.283	3.02E-08	chr:6:32648036
6	rs115286318	32573911	A	11.01	3.05E-08	chr:6:32648036
6	rs150548684	32507293	T	1.954	3.06E-08	chr:6:32648036
6	rs17205747	32650911	T	0.3642	3.09E-08	chr:6:32648036
6	rs28746905	32648842	T	0.3659	3.09E-08	chr:6:32648036
6	rs76867075	32459748	T	2.308	3.10E-08	chr:6:32648036
6	rs5000634	32663564	G	2.244	3.13E-08	chr:6:32648036
6	rs17843709	32622409	G	1.977	3.21E-08	chr:6:32648036
6	rs28891486	32649408	T	0.3667	3.21E-08	chr:6:32648036
6	rs9271850	32595060	G	0.3762	3.21E-08	chr:6:32648036
6	rs12055602	32622382	C	1.97	3.21E-08	chr:6:32648036
6	rs6935578	32683552	G	2.946	3.26E-08	chr:6:32648036
6	rs7770879	32598423	A	0.3171	3.27E-08	chr:6:32648036
6	rs7771138	32598419	A	0.3171	3.27E-08	chr:6:32648036
6	rs74395243	32608116	A	3.324	3.32E-08	chr:6:32648036
6	rs28747050	32655674	C	0.3645	3.33E-08	chr:6:32648036
6	rs28826696	32617628	C	1.804	3.34E-08	chr:6:32648036
6	rs574347579	32514817	A	2.594	3.35E-08	chr:6:32648036
6	rs116384287	32482031	T	2.131	3.38E-08	chr:6:32648036
6	rs572418566	32670172	C	2.394	3.38E-08	chr:6:32648036
6	rs9275446	32671841	G	2.287	3.39E-08	chr:6:32648036
6	rs9275448	32671848	T	2.287	3.39E-08	chr:6:32648036
6	rs9275449	32671869	T	2.287	3.39E-08	chr:6:32648036
6	rs531218774	32629372	A	2.482	3.41E-08	chr:6:32648036
6	rs9273736	32629371	G	2.482	3.41E-08	chr:6:32648036
6	rs113602321	32468804	A	2.039	3.41E-08	chr:6:32648036
6	rs12055445	32626739	A	0.4396	3.42E-08	chr:6:32648036
6	rs28746913	32649517	A	0.3688	3.45E-08	chr:6:32648036
6	rs28746914	32649536	C	0.3688	3.45E-08	chr:6:32648036

6	rs28746915	32649543	A	0.3688	3.45E-08	chr:6:32648036
6	rs9271454	32588422	T	2.479	3.48E-08	chr:6:32648036
6	rs9271455	32588423	G	2.479	3.48E-08	chr:6:32648036
6	rs78512156	32458168	C	3.338	3.49E-08	chr:6:32648036
6	rs78947187	32458169	A	3.338	3.49E-08	chr:6:32648036
6	rs28747047	32655578	T	0.3681	3.53E-08	chr:6:32648036
6	rs28747013	32654596	A	0.3674	3.53E-08	chr:6:32648036
6	rs76342965	32508008	T	2	3.55E-08	chr:6:32648036
6	rs9271688	32592992	G	0.3827	3.61E-08	chr:6:32648036
6	rs2395106	32193220	A	2.269	3.61E-08	chr:6:32648036
6	rs3096691	32194854	G	2.269	3.61E-08	chr:6:32648036
6	rs564551505	32644523	C	0.3537	3.62E-08	chr:6:32648036
6	rs28746906	32648863	A	0.3678	3.63E-08	chr:6:32648036
6	rs35744187	32637756	A	0.365	3.63E-08	chr:6:32648036
6	rs17205949	32651816	A	0.3688	3.65E-08	chr:6:32648036
6	rs17212286	32651819	A	0.3688	3.65E-08	chr:6:32648036
6	rs35877117	32501719	T	3.071	3.66E-08	chr:6:32648036
6	rs3104377	32599689	C	0.3724	3.68E-08	chr:6:32648036
6	rs78273580	32506779	T	2.233	3.69E-08	chr:6:32648036
6	rs4403294	32634922	A	0.379	3.69E-08	chr:6:32648036
6	rs9268805	32423399	G	0.4184	3.70E-08	chr:6:32648036
6	rs75459649	32505390	G	2.065	3.70E-08	chr:6:32648036
6	rs1475960	32194751	T	2.27	3.73E-08	chr:6:32648036
6	rs1475961	32194609	A	2.27	3.73E-08	chr:6:32648036
6	rs114618396	32608053	T	3.399	3.74E-08	chr:6:32648036
6	rs9273442	32627582	T	0.3566	3.74E-08	chr:6:32648036
6	rs28746934	32650436	C	0.3713	3.75E-08	chr:6:32648036
6	rs28746931	32650194	G	0.3705	3.80E-08	chr:6:32648036
6	rs28746932	32650217	A	0.3705	3.80E-08	chr:6:32648036
6	rs28746883	32638392	T	0.3696	3.81E-08	chr:6:32648036
6	rs28746884	32638395	T	0.3696	3.81E-08	chr:6:32648036
6	rs9274326	32632237	C	0.5277	3.84E-08	chr:6:32648036
6	rs9269041	32438243	G	0.3735	3.84E-08	chr:6:32648036
6	rs75730517	32503697	A	2.168	3.85E-08	chr:6:32648036
6	rs17204659	32557321	T	3.305	3.88E-08	chr:6:32648036
6	rs9271681	32592918	T	0.3966	3.91E-08	chr:6:32648036
6	rs3104383	32598784	G	0.3228	3.91E-08	chr:6:32648036
6	rs28746925	32649932	G	0.368	3.93E-08	chr:6:32648036
6	rs9272018	32598460	T	0.3267	3.95E-08	chr:6:32648036
6	rs572081630	32512882	T	2.542	3.96E-08	chr:6:32648036
6	rs147842166	32485952	G	3.225	3.97E-08	chr:6:32648036
6	rs147192780	32489642	C	3.11	3.98E-08	chr:6:32648036
6	rs28747048	32655584	T	0.3694	3.98E-08	chr:6:32648036

6	rs9271727	32593585	A	0.3599	3.99E-08	chr:6:32648036
6	rs28891489	32650362	C	0.3799	4.02E-08	chr:6:32648036
6	rs1109771	32187605	A	2.177	4.02E-08	chr:6:32648036
6	rs28891488	32650345	T	0.3735	4.03E-08	chr:6:32648036
6	rs72848204	32594073	T	0.3907	4.08E-08	chr:6:32648036
6	rs112530924	32552590	C	2.783	4.14E-08	chr:6:32648036
6	rs9272266	32603416	A	0.3579	4.17E-08	chr:6:32648036
6	rs78923832	32507947	A	1.998	4.23E-08	chr:6:32648036
6	rs9274112	32630194	T	0.3962	4.23E-08	chr:6:32648036
6	rs9273138	32612843	T	2.811	4.24E-08	chr:6:32648036
6	rs35635216	32501743	A	3.055	4.25E-08	chr:6:32648036
6	rs2395105	32192978	T	2.253	4.28E-08	chr:6:32648036
6	rs28891403	32640257	C	0.3698	4.28E-08	chr:6:32648036
6	rs28891404	32640268	A	0.3698	4.28E-08	chr:6:32648036
6	rs9275052	32648156	G	0.4829	4.29E-08	chr:6:32648036
6	rs28891487	32650312	T	0.3727	4.30E-08	chr:6:32648036
6	rs186302965	32498947	A	1.969	4.31E-08	chr:6:32648036
6	rs190230688	32498937	T	1.969	4.31E-08	chr:6:32648036
6	rs17205942	32651798	T	0.3704	4.31E-08	chr:6:32648036
6	rs9273440	32627561	T	0.3679	4.32E-08	chr:6:32648036
6	rs9271378	32587300	G	2.233	4.33E-08	chr:6:32648036
6	rs542371727	32554817	T	2.969	4.34E-08	chr:6:32648036
6	rs9275447	32671843	C	2.273	4.34E-08	chr:6:32648036
6	rs9272154	32601019	A	0.3447	4.37E-08	chr:6:32648036
6	rs17205858	32651297	C	0.3701	4.38E-08	chr:6:32648036
6	rs28747064	32656770	G	0.3709	4.38E-08	chr:6:32648036
6	rs4993691	32446981	G	0.3019	4.39E-08	chr:6:32648036
6	rs28746997	32654225	T	0.3549	4.39E-08	chr:6:32648036
6	rs17212160	32651097	G	0.3609	4.39E-08	chr:6:32648036
6	rs28746962	32652979	A	0.3614	4.41E-08	chr:6:32648036
6	rs28746967	32653231	C	0.3691	4.46E-08	chr:6:32648036
6	rs6689	32627700	G	0.3616	4.48E-08	chr:6:32648036
6	rs28366265	32559708	A	2.46	4.53E-08	chr:6:32648036
6	rs113046257	32512753	T	2.55	4.55E-08	chr:6:32648036
6	rs113085203	32512789	G	2.55	4.55E-08	chr:6:32648036
6	rs9273206	32613573	T	0.3908	4.60E-08	chr:6:32648036
6	rs28747032	32655180	C	0.372	4.65E-08	chr:6:32648036
6	rs28746968	32653247	T	0.3673	4.66E-08	chr:6:32648036
6	rs28746969	32653248	G	0.3673	4.66E-08	chr:6:32648036
6	rs76264710	32569305	A	0.4101	4.72E-08	chr:6:32648036
6	rs28747023	32654807	A	0.3756	4.74E-08	chr:6:32648036
6	rs9271728	32593592	C	0.3622	4.75E-08	chr:6:32648036
6	rs532946202	32537465	T	6.025	4.80E-08	chr:6:32648036

6	rs28747024	32654830	A	0.3682	4.81E-08	chr:6:32648036
6	rs28747025	32654831	C	0.3682	4.81E-08	chr:6:32648036
6	rs28357082	32558176	A	2.693	4.83E-08	chr:6:32648036
6	rs76931568	32459665	A	2.002	4.86E-08	chr:6:32648036
6	rs530246116	32517244	C	2.611	4.94E-08	chr:6:32648036
6	rs9269011	32436299	C	1.969	4.95E-08	chr:6:32648036
6	rs17205928	32651737	G	0.3715	4.95E-08	chr:6:32648036
6	rs28891405	32640288	T	0.3716	4.96E-08	chr:6:32648036
6	rs28891406	32640336	A	0.3716	4.96E-08	chr:6:32648036
6	rs17212350	32652173	G	0.3652	4.96E-08	chr:6:32648036
6	rs17212357	32652174	T	0.3652	4.96E-08	chr:6:32648036
6	rs17212364	32652176	G	0.3652	4.96E-08	chr:6:32648036
6	rs542891748	32526778	C	2.604	4.97E-08	chr:6:32648036
6	rs78097306	32499148	T	1.891	4.97E-08	chr:6:32648036
6	rs115736509	32554782	T	4.535	5.03E-08	chr:6:32648036
6	rs28747031	32655125	A	0.3711	5.05E-08	chr:6:32648036
6	rs28747066	32656817	A	0.3694	5.08E-08	chr:6:32648036
6	rs75262655	32459811	G	2.291	5.09E-08	chr:6:32648036
6	rs79420263	32459803	C	2.291	5.09E-08	chr:6:32648036
6	rs28747049	32655659	C	0.3721	5.11E-08	chr:6:32648036
6	rs28746998	32654246	T	0.3546	5.11E-08	chr:6:32648036
6	rs9275488	32673269	A	2.265	5.13E-08	chr:6:32648036
6	rs9275489	32673272	T	2.265	5.13E-08	chr:6:32648036
6	rs17212343	32652147	G	0.3715	5.14E-08	chr:6:32648036
6	rs370710514	32562498	G	2.025	5.16E-08	chr:6:32648036
6	rs9275451	32671908	T	2.262	5.17E-08	chr:6:32648036
6	rs539509361	32594399	G	0.3497	5.19E-08	chr:6:32648036
6	rs72848263	32602065	C	0.3624	5.20E-08	chr:6:32648036
6	rs79591888	32503704	A	2.141	5.22E-08	chr:6:32648036
6	rs28414666	32626451	A	0.3393	5.22E-08	chr:6:32648036
6	rs17205809	32651135	A	0.3683	5.23E-08	chr:6:32648036
6	rs28747014	32654658	C	0.3731	5.26E-08	chr:6:32648036
6	rs28747015	32654673	T	0.3731	5.26E-08	chr:6:32648036
6	rs62405568	32453409	C	2.565	5.27E-08	chr:6:32648036
6	rs17206005	32652069	G	0.3736	5.31E-08	chr:6:32648036
6	rs17206012	32652077	C	0.3736	5.31E-08	chr:6:32648036
6	rs9274115	32630205	T	0.4063	5.41E-08	chr:6:32648036
6	rs542744272	32556027	A	16.2	5.41E-08	chr:6:32648036
6	rs62405589	32508516	G	2.142	5.42E-08	chr:6:32648036
6	rs9271729	32593600	A	0.3639	5.44E-08	chr:6:32648036
6	rs9274247	32631332	A	0.4066	5.47E-08	chr:6:32648036
6	rs552154060	32458032	G	4.12	5.50E-08	chr:6:32648036
6	rs9275501	32673920	T	2.261	5.50E-08	chr:6:32648036

6	rs9275502	32673921	G	2.261	5.50E-08	chr:6:32648036
6	rs9275504	32673986	T	2.261	5.50E-08	chr:6:32648036
6	rs17212251	32651629	C	0.3693	5.56E-08	chr:6:32648036
6	rs201699945	32485856	C	3.802	5.60E-08	chr:6:32648036
6	rs376789262	32727002	G	3.223	5.62E-08	chr:6:32648036
6	rs17846798	32150498	A	2.732	5.65E-08	chr:6:32648036
6	rs28747051	32655686	T	0.3728	5.78E-08	chr:6:32648036
6	rs28747052	32655703	T	0.3728	5.78E-08	chr:6:32648036
6	rs9273529	32628698	T	2.483	5.79E-08	chr:6:32648036
6	rs9273530	32628699	G	2.483	5.79E-08	chr:6:32648036
6	rs201628145	32489913	G	5.646	5.83E-08	chr:6:32648036
6	rs570919834	32481691	T	2.959	5.83E-08	chr:6:32648036
6	rs76902085	32507945	C	1.98	5.84E-08	chr:6:32648036
6	rs17205977	32651927	C	0.3731	5.87E-08	chr:6:32648036
6	rs28891407	32640359	T	0.374	5.93E-08	chr:6:32648036
6	rs17212153	32651092	T	0.3661	5.99E-08	chr:6:32648036
6	rs75684124	32503488	T	2.212	6.02E-08	chr:6:32648036
6	rs28891410	32640424	G	0.3743	6.04E-08	chr:6:32648036
6	rs77587106	32508001	A	1.977	6.08E-08	chr:6:32648036
6	rs28746961	32652968	G	0.3656	6.15E-08	chr:6:32648036
6	rs17205754	32650930	C	0.3738	6.19E-08	chr:6:32648036
6	rs17205761	32650934	A	0.3738	6.19E-08	chr:6:32648036
6	rs17205768	32650937	C	0.3738	6.19E-08	chr:6:32648036
6	rs28746933	32650411	G	0.3791	6.20E-08	chr:6:32648036
6	rs9272024	32598559	A	0.3235	6.28E-08	chr:6:32648036
6	rs36046522	32550931	A	3.149	6.29E-08	chr:6:32648036
6	rs6902805	32193568	T	0.4158	6.29E-08	chr:6:32648036
6	rs9296012	32195332	T	0.4158	6.29E-08	chr:6:32648036
6	rs75438654	32459749	G	2.406	6.41E-08	chr:6:32648036
6	rs542882860	32670178	C	2.343	6.62E-08	chr:6:32648036
6	rs28891408	32640382	A	0.3754	6.66E-08	chr:6:32648036
6	rs28891409	32640401	G	0.3754	6.66E-08	chr:6:32648036
6	rs34061722	32599337	T	0.3534	6.71E-08	chr:6:32648036
6	rs9271414	32587782	C	0.3899	6.71E-08	chr:6:32648036
6	rs9271415	32587786	C	0.3899	6.71E-08	chr:6:32648036
6	rs28747061	32656653	T	0.3673	6.72E-08	chr:6:32648036
6	rs28747062	32656661	C	0.3673	6.72E-08	chr:6:32648036
6	rs28747063	32656668	C	0.3673	6.72E-08	chr:6:32648036
6	rs28772268	32656872	A	0.3719	6.73E-08	chr:6:32648036
6	rs28772269	32656878	C	0.3719	6.73E-08	chr:6:32648036
6	rs28772270	32656881	T	0.3719	6.73E-08	chr:6:32648036
6	rs9273757	32629417	C	2.369	6.76E-08	chr:6:32648036
6	rs376740117	32506081	T	1.897	6.76E-08	chr:6:32648036

6	rs17205844	32651254	G	0.3763	6.81E-08	chr:6:32648036
6	rs17205851	32651265	C	0.3763	6.81E-08	chr:6:32648036
6	rs28746918	32649769	T	0.3772	6.87E-08	chr:6:32648036
6	rs281875058	32631207	T	2.017	6.90E-08	chr:6:32648036
6	rs4568495	32672246	C	2.248	6.91E-08	chr:6:32648036
6	rs9275377	32668667	G	2.248	6.91E-08	chr:6:32648036
6	rs9275378	32668729	G	2.248	6.91E-08	chr:6:32648036
6	rs9275379	32668804	T	2.248	6.91E-08	chr:6:32648036
6	rs9275396	32669528	A	2.248	6.91E-08	chr:6:32648036
6	rs9275397	32669552	G	2.248	6.91E-08	chr:6:32648036
6	rs9275399	32669712	G	2.248	6.91E-08	chr:6:32648036
6	rs9275464	32672082	G	2.248	6.91E-08	chr:6:32648036
6	rs9275468	32672412	A	2.248	6.91E-08	chr:6:32648036
6	rs9275469	32672430	G	2.248	6.91E-08	chr:6:32648036
6	rs9275481	32672913	A	2.248	6.91E-08	chr:6:32648036
6	rs9275482	32672932	T	2.248	6.91E-08	chr:6:32648036
6	rs9275485	32673077	A	2.248	6.91E-08	chr:6:32648036
6	rs9275487	32673174	T	2.248	6.91E-08	chr:6:32648036
6	rs9275491	32673424	T	2.248	6.91E-08	chr:6:32648036
6	rs9275492	32673446	T	2.248	6.91E-08	chr:6:32648036
6	rs9275507	32674130	A	2.248	6.91E-08	chr:6:32648036
6	rs9275508	32674159	G	2.248	6.91E-08	chr:6:32648036
6	rs28746972	32653406	G	0.378	6.93E-08	chr:6:32648036
6	rs113453333	32522490	A	2.588	6.93E-08	chr:6:32648036
6	rs77931438	32505762	A	2.08	6.94E-08	chr:6:32648036
6	rs28746999	32654258	A	0.3621	7.02E-08	chr:6:32648036
6	rs78434421	32627652	G	2.233	7.10E-08	chr:6:32648036
6	rs9275483	32673051	C	2.252	7.11E-08	chr:6:32648036
6	rs9275484	32673052	T	2.252	7.11E-08	chr:6:32648036
6	rs9274507	32634152	G	0.4105	7.12E-08	chr:6:32648036
6	rs17211601	32602817	G	0.3554	7.13E-08	chr:6:32648036
6	rs28533091	32602864	A	0.3554	7.13E-08	chr:6:32648036
6	rs75563047	32485492	T	2.264	7.15E-08	chr:6:32648036
6	rs552547617	32479301	A	1.883	7.17E-08	chr:6:32648036
6	rs28357084	32559483	G	2.842	7.22E-08	chr:6:32648036
6	rs28366254	32559481	G	2.842	7.22E-08	chr:6:32648036
6	rs28366255	32559482	T	2.842	7.22E-08	chr:6:32648036
6	rs9273650	32629194	A	3.213	7.25E-08	chr:6:32648036
6	rs144537734	32513476	A	0.4031	7.27E-08	chr:6:32648036
6	rs9273546	32628841	A	0.4182	7.32E-08	chr:6:32648036
6	rs79705220	32507930	C	1.971	7.32E-08	chr:6:32648036
6	rs17191234	32564681	C	2.314	7.36E-08	chr:6:32648036
6	rs17212132	32651008	A	0.3738	7.38E-08	chr:6:32648036

6	rs3763317	32376788	C	0.4513	7.43E-08	chr:6:32648036
6	rs28746946	32652715	A	0.3692	7.44E-08	chr:6:32648036
6	rs6926894	32612430	C	2.405	7.46E-08	chr:6:32648036
6	rs2071288	32149260	T	2.699	7.66E-08	chr:6:32648036
6	rs17211510	32602430	A	0.3554	7.70E-08	chr:6:32648036
6	rs28747033	32655213	C	0.3793	7.71E-08	chr:6:32648036
6	rs9271293	32581459	G	0.4474	7.85E-08	chr:6:32648036
6	rs9271294	32581461	A	0.4474	7.85E-08	chr:6:32648036
6	rs9271295	32581467	T	0.4474	7.85E-08	chr:6:32648036
6	rs9273270	32614361	C	0.5192	7.86E-08	chr:6:32648036
6	rs9271456	32588435	T	2.401	7.99E-08	chr:6:32648036
6	rs28746944	32652651	A	0.3719	8.00E-08	chr:6:32648036
6	rs113600317	32553125	T	2.782	8.06E-08	chr:6:32648036
6	rs35331833	32556635	A	3.23	8.07E-08	chr:6:32648036
6	rs28747035	32655316	C	0.3789	8.08E-08	chr:6:32648036
6	rs9273817	32629529	T	0.3627	8.12E-08	chr:6:32648036
6	rs9271871	32595383	C	0.3178	8.13E-08	chr:6:32648036
6	rs115976249	32608013	A	3.868	8.13E-08	chr:6:32648036
6	rs28746928	32650060	G	0.3809	8.15E-08	chr:6:32648036
6	rs17205802	32651072	G	0.3687	8.28E-08	chr:6:32648036
6	rs28371174	32612524	T	5.748	8.29E-08	chr:6:32648036
6	rs28746995	32654116	A	0.3706	8.32E-08	chr:6:32648036
6	rs9271874	32595424	T	0.3437	8.34E-08	chr:6:32648036
6	rs9268948	32433746	G	0.4663	8.38E-08	chr:6:32648036
6	rs79100516	32503452	T	2.086	8.40E-08	chr:6:32648036
6	rs4311549	32635091	T	0.4276	8.43E-08	chr:6:32648036
6	rs9273463	32627934	A	2.494	8.45E-08	chr:6:32648036
6	rs138081466	32607906	C	4.16	8.47E-08	chr:6:32648036
6	rs560708392	32607901	C	4.16	8.47E-08	chr:6:32648036
6	rs28746973	32653442	A	0.3803	8.57E-08	chr:6:32648036
6	rs28746974	32653443	A	0.3803	8.57E-08	chr:6:32648036
6	rs9271441	32588245	T	0.4245	8.60E-08	chr:6:32648036
6	rs3135387	32415109	T	0.3834	8.61E-08	chr:6:32648036
6	rs28366297	32560829	C	2.743	8.62E-08	chr:6:32648036
6	rs17212188	32651216	A	0.3814	8.68E-08	chr:6:32648036
6	rs200526004	32486059	T	3.054	8.71E-08	chr:6:32648036
6	rs67838634	32662128	G	0.4288	8.74E-08	chr:6:32648036
6	rs17205907	32651649	G	0.3764	8.85E-08	chr:6:32648036
6	rs547199103	32606006	A	3.463	8.85E-08	chr:6:32648036
6	rs561917905	32606007	G	3.463	8.85E-08	chr:6:32648036
6	rs17205782	32651051	T	0.3718	8.87E-08	chr:6:32648036
6	rs17205788	32651056	A	0.3718	8.87E-08	chr:6:32648036
6	rs28747003	32654322	C	0.3791	8.88E-08	chr:6:32648036

6	rs3104380	32599042	G	0.3218	8.90E-08	chr:6:32648036
6	rs7764856	32680640	A	2.229	8.91E-08	chr:6:32648036
6	rs28772271	32656901	C	0.3768	8.95E-08	chr:6:32648036
6	rs9469220	32658310	G	0.4428	8.97E-08	chr:6:32648036
6	rs17205177	32602518	G	0.3575	9.02E-08	chr:6:32648036
6	rs28747043	32655508	G	0.3737	9.02E-08	chr:6:32648036
6	rs28747038	32655368	A	0.3813	9.02E-08	chr:6:32648036
6	rs9271490	32589060	A	0.3895	9.06E-08	chr:6:32648036
6	rs9271491	32589061	A	0.3895	9.06E-08	chr:6:32648036
6	rs9271493	32589158	A	0.3895	9.06E-08	chr:6:32648036
6	rs9271512	32589510	A	0.3895	9.06E-08	chr:6:32648036
6	rs9274079	32630131	T	2.453	9.10E-08	chr:6:32648036
6	rs74867743	32502819	T	2.017	9.11E-08	chr:6:32648036
6	rs17211105	32557606	A	3.951	9.14E-08	chr:6:32648036
6	rs114852415	32524759	A	2.558	9.25E-08	chr:6:32648036
6	rs9273713	32629320	A	0.4399	9.31E-08	chr:6:32648036
6	rs9272451	32605501	G	2.193	9.33E-08	chr:6:32648036
6	rs35779452	32599272	G	0.3582	9.50E-08	chr:6:32648036
6	rs28891480	32642843	G	0.3846	9.55E-08	chr:6:32648036
6	rs28718232	32626750	G	0.3523	9.66E-08	chr:6:32648036
6	rs9271705	32593321	T	0.3978	9.69E-08	chr:6:32648036
6	rs9271704	32593317	G	0.3914	9.72E-08	chr:6:32648036
6	rs9269069	32440362	C	0.4244	9.73E-08	chr:6:32648036
6	rs10093	32609173	G	2.176	9.78E-08	chr:6:32648036
6	rs34927613	32600211	A	0.3533	9.78E-08	chr:6:32648036
6	rs28366270	32559934	G	2.352	9.79E-08	chr:6:32648036
6	rs28747034	32655257	T	0.3821	9.81E-08	chr:6:32648036
6	rs34801804	32555892	G	0.3475	9.85E-08	chr:6:32648036
6	rs28362368	32594040	G	0.4021	9.93E-08	chr:6:32648036
6	rs11963017	32657863	C	0.3845	9.94E-08	chr:6:32648036
6	rs11967921	32657855	A	0.3845	9.94E-08	chr:6:32648036
6	rs11967958	32657849	A	0.3845	9.94E-08	chr:6:32648036
6	rs17205114	32602196	G	0.3603	9.94E-08	chr:6:32648036
6	rs17205128	32602217	G	0.3603	9.94E-08	chr:6:32648036
6	rs17205135	32602222	G	0.3603	9.94E-08	chr:6:32648036
6	rs9271956	32597313	T	0.3212	9.95E-08	chr:6:32648036
6	rs17205795	32651066	T	0.3721	9.98E-08	chr:6:32648036
6	rs192002545	32517227	A	2.536	1.00E-07	chr:6:32648036
6	rs145365399	32517715	G	8.11	1.01E-07	chr:6:32648036
6	rs9275471	32672491	A	2.219	1.01E-07	chr:6:32648036
6	rs9275472	32672494	A	2.219	1.01E-07	chr:6:32648036
6	rs9275473	32672523	T	2.219	1.01E-07	chr:6:32648036
6	rs9275474	32672529	T	2.219	1.01E-07	chr:6:32648036

6	rs9275475	32672556	G	2.219	1.01E-07	chr:6:32648036
6	rs2395517	32597792	A	0.3213	1.01E-07	chr:6:32648036
6	rs183870834	32517228	C	2.526	1.03E-07	chr:6:32648036
6	rs9271557	32590331	T	0.4015	1.03E-07	chr:6:32648036
6	rs28746929	32650068	G	0.3843	1.04E-07	chr:6:32648036
6	rs28746930	32650074	A	0.3843	1.04E-07	chr:6:32648036
6	rs9274442	32633231	C	2.442	1.04E-07	chr:6:32648036
6	rs436845	32197736	G	0.3997	1.04E-07	chr:6:32648036
6	rs3104370	32602341	G	0.3212	1.04E-07	chr:6:32648036
6	rs968155	32380715	C	0.4521	1.04E-07	chr:6:32648036
6	rs9271291	32581335	G	0.4497	1.05E-07	chr:6:32648036
6	rs9271292	32581350	T	0.4497	1.05E-07	chr:6:32648036
6	rs9271880	32595596	C	0.399	1.06E-07	chr:6:32648036
6	rs2213287	32605508	T	2.187	1.07E-07	chr:6:32648036
6	rs9275478	32672654	T	2.216	1.07E-07	chr:6:32648036
6	rs9275479	32672659	T	2.216	1.07E-07	chr:6:32648036
6	rs9275480	32672668	A	2.216	1.07E-07	chr:6:32648036
6	rs9271684	32592953	T	0.4002	1.07E-07	chr:6:32648036
6	rs201415661	32526386	T	2.708	1.07E-07	chr:6:32648036
6	rs35366682	32594354	C	0.3627	1.08E-07	chr:6:32648036
6	rs28747044	32655516	C	0.3763	1.09E-07	chr:6:32648036
6	rs28891377	32638888	C	0.3804	1.09E-07	chr:6:32648036
6	rs28891378	32638894	T	0.3804	1.09E-07	chr:6:32648036
6	rs28747037	32655336	C	0.384	1.10E-07	chr:6:32648036
6	rs199516615	32493510	A	2.382	1.10E-07	chr:6:32648036
6	rs28746902	32648713	A	0.3873	1.11E-07	chr:6:32648036
6	rs17205837	32651222	C	0.3848	1.11E-07	chr:6:32648036
6	rs17211580	32602754	A	0.3639	1.11E-07	chr:6:32648036
6	rs9271286	32581221	G	0.4523	1.11E-07	chr:6:32648036
6	rs9271287	32581253	A	0.4523	1.11E-07	chr:6:32648036
6	rs9271288	32581260	A	0.4523	1.11E-07	chr:6:32648036
6	rs9273651	32629199	C	3.156	1.11E-07	chr:6:32648036
6	rs114202038	32493438	C	2.433	1.12E-07	chr:6:32648036
6	rs11751908	32599862	G	0.3392	1.12E-07	chr:6:32648036
6	rs9271336	32582877	G	0.4543	1.12E-07	chr:6:32648036
6	rs9271337	32582881	C	0.4543	1.12E-07	chr:6:32648036
6	rs9271338	32582885	T	0.4543	1.12E-07	chr:6:32648036
6	rs9271339	32582891	T	0.4543	1.12E-07	chr:6:32648036
6	rs111437237	32537608	C	1.861	1.13E-07	chr:6:32648036
6	rs379464	32186348	T	2.339	1.13E-07	chr:6:32648036
6	rs116292100	32482576	C	1.867	1.14E-07	chr:6:32648036
6	rs3104386	32596937	G	0.3209	1.14E-07	chr:6:32648036
6	rs112716963	32485815	A	2.32	1.14E-07	chr:6:32648036

6	rs78510027	32505075	G	2.298	1.15E-07	chr:6:32648036
6	rs2395223	32581778	C	0.4531	1.15E-07	chr:6:32648036
6	rs28746899	32648646	A	0.3908	1.16E-07	chr:6:32648036
6	rs28746900	32648652	G	0.3908	1.16E-07	chr:6:32648036
6	rs34290324	32550947	G	2.988	1.16E-07	chr:6:32648036
6	rs28746901	32648681	T	0.3909	1.17E-07	chr:6:32648036
6	rs28747067	32656832	T	0.3831	1.18E-07	chr:6:32648036
6	rs113522240	32512591	G	2.512	1.19E-07	chr:6:32648036
6	rs9271646	32592351	G	0.4006	1.19E-07	chr:6:32648036
6	rs9271647	32592354	G	0.4006	1.19E-07	chr:6:32648036
6	rs9271649	32592359	G	0.4006	1.19E-07	chr:6:32648036
6	rs527606121	32452051	G	2.247	1.21E-07	chr:6:32648036
6	rs79186433	32479883	T	1.9	1.23E-07	chr:6:32648036
6	rs28891376	32638788	C	0.3517	1.24E-07	chr:6:32648036
6	rs17205998	32652050	G	0.3857	1.24E-07	chr:6:32648036
6	rs9271296	32581501	A	0.4524	1.25E-07	chr:6:32648036
6	rs17211454	32602179	A	0.3625	1.25E-07	chr:6:32648036
6	rs3104379	32599114	G	0.3205	1.27E-07	chr:6:32648036
6	rs28694749	32603029	C	0.3687	1.27E-07	chr:6:32648036
6	rs9271182	32578230	A	0.3429	1.28E-07	chr:6:32648036
6	rs77456373	32598853	T	0.3068	1.28E-07	chr:6:32648036
6	rs28407322	32603355	C	0.3652	1.28E-07	chr:6:32648036
6	rs34403271	32550220	C	2.768	1.29E-07	chr:6:32648036
6	rs113296631	32478885	T	1.881	1.29E-07	chr:6:32648036
6	rs9271289	32581305	C	0.4536	1.29E-07	chr:6:32648036
6	rs576836627	32624327	T	3.333	1.29E-07	chr:6:32648036
6	rs34232122	32556706	C	3.106	1.30E-07	chr:6:32648036
6	rs9271302	32581621	T	0.4559	1.32E-07	chr:6:32648036
6	rs17212265	32651694	T	0.3796	1.32E-07	chr:6:32648036
6	rs28747000	32654280	A	0.3718	1.32E-07	chr:6:32648036
6	rs28747001	32654284	G	0.3718	1.32E-07	chr:6:32648036
6	rs9274478	32633744	C	0.3982	1.32E-07	chr:6:32648036
6	rs482044	32576064	C	2.203	1.33E-07	chr:6:32648036
6	rs3104406	32682443	G	0.4585	1.33E-07	chr:6:32648036
6	rs2856698	32636376	G	0.3986	1.34E-07	chr:6:32648036
6	rs9271436	32588110	C	0.3997	1.34E-07	chr:6:32648036
6	rs9271431	32588031	A	2.172	1.34E-07	chr:6:32648036
6	rs9272068	32599404	C	0.3127	1.36E-07	chr:6:32648036
6	rs3891175	32634467	T	2.46	1.36E-07	chr:6:32648036
6	rs28747022	32654785	A	0.3857	1.37E-07	chr:6:32648036
6	rs9271507	32589394	C	0.3992	1.38E-07	chr:6:32648036
6	rs79428469	32602034	C	0.3715	1.38E-07	chr:6:32648036
6	rs28747045	32655537	C	0.3818	1.38E-07	chr:6:32648036

6	rs28747046	32655539	G	0.3818	1.38E-07	chr:6:32648036
6	rs9273595	32629091	G	2.245	1.39E-07	chr:6:32648036
6	rs9275499	32673778	G	2.192	1.39E-07	chr:6:32648036
6	rs28747065	32656806	C	0.3868	1.41E-07	chr:6:32648036
6	rs28747016	32654706	A	0.3869	1.42E-07	chr:6:32648036
6	rs77339454	32503567	A	2.259	1.44E-07	chr:6:32648036
6	rs9296035	32657888	G	0.3902	1.44E-07	chr:6:32648036
6	rs1063321	32629955	T	2.205	1.44E-07	chr:6:32648036
6	rs28746885	32638871	G	0.3854	1.46E-07	chr:6:32648036
6	rs3104392	32594425	C	0.3384	1.47E-07	chr:6:32648036
6	rs111848494	32486020	G	2.801	1.47E-07	chr:6:32648036
6	rs9271505	32589375	T	0.402	1.48E-07	chr:6:32648036
6	rs9271506	32589380	C	0.402	1.48E-07	chr:6:32648036
6	rs28746886	32639083	C	0.3861	1.50E-07	chr:6:32648036
6	rs111357164	32482638	A	1.889	1.50E-07	chr:6:32648036
6	rs28747029	32655096	A	0.3869	1.50E-07	chr:6:32648036
6	rs28747030	32655104	A	0.3869	1.50E-07	chr:6:32648036
6	rs116176269	32490519	C	2.596	1.51E-07	chr:6:32648036
6	rs6929057	32597005	C	0.3821	1.51E-07	chr:6:32648036
6	rs9271327	32582533	G	0.4555	1.51E-07	chr:6:32648036
6	rs9275496	32673714	C	2.181	1.53E-07	chr:6:32648036
6	rs9275497	32673726	G	2.181	1.53E-07	chr:6:32648036
6	rs9275498	32673749	T	2.181	1.53E-07	chr:6:32648036
6	rs9271620	32591777	T	2.21	1.54E-07	chr:6:32648036
6	rs281863486	32630967	C	0.4047	1.56E-07	chr:6:32648036
6	rs112550762	32507514	A	0.4792	1.57E-07	chr:6:32648036
6	rs1465651	32901351	T	2.36	1.58E-07	chr:6:32648036
6	rs74768985	32518175	C	2.76	1.58E-07	chr:6:32648036
6	rs9274465	32633490	C	2.322	1.58E-07	chr:6:32648036
6	rs9271591	32591141	A	2.208	1.60E-07	chr:6:32648036
6	rs2894275	32581754	T	0.4573	1.60E-07	chr:6:32648036
6	rs28747018	32654739	C	0.3879	1.61E-07	chr:6:32648036
6	rs146080923	32605997	C	3.321	1.62E-07	chr:6:32648036
6	rs9271510	32589467	T	0.4004	1.62E-07	chr:6:32648036
6	rs34145408	32602124	T	0.3722	1.63E-07	chr:6:32648036
6	rs35360625	32599435	C	0.3684	1.63E-07	chr:6:32648036
6	rs35744886	32600084	T	0.3515	1.63E-07	chr:6:32648036
6	rs9274364	32632495	G	1.804	1.63E-07	chr:6:32648036
6	rs17882028	32551911	A	2.304	1.65E-07	chr:6:32648036
6	rs17885482	32551912	C	2.304	1.65E-07	chr:6:32648036
6	rs28366268	32559874	G	2.314	1.65E-07	chr:6:32648036
6	rs531642165	32472248	A	3.689	1.65E-07	chr:6:32648036
6	rs561310579	32472247	T	3.689	1.65E-07	chr:6:32648036

6	rs9271284	32581190	T	0.4576	1.66E-07	chr:6:32648036
6	rs9271285	32581193	A	0.4576	1.66E-07	chr:6:32648036
6	rs28746887	32639096	T	0.3873	1.66E-07	chr:6:32648036
6	rs28891379	32639107	C	0.3873	1.66E-07	chr:6:32648036
6	rs9273148	32612982	A	2.902	1.66E-07	chr:6:32648036
6	rs1063323	32629891	T	2.197	1.66E-07	chr:6:32648036
6	rs1130398	32629764	T	2.197	1.66E-07	chr:6:32648036
6	rs9271482	32588880	C	0.4092	1.68E-07	chr:6:32648036
6	rs9271483	32588882	G	0.4092	1.68E-07	chr:6:32648036
6	rs9273392	32627046	A	3.004	1.68E-07	chr:6:32648036
6	rs9273393	32627050	A	3.004	1.68E-07	chr:6:32648036
6	rs3135461	32680122	G	0.3499	1.69E-07	chr:6:32648036
6	rs9275376	32668633	T	2.191	1.69E-07	chr:6:32648036
6	rs7451962	32582435	G	0.4563	1.70E-07	chr:6:32648036
6	rs9271324	32582482	C	0.4563	1.70E-07	chr:6:32648036
6	rs28891373	32638744	A	0.3587	1.72E-07	chr:6:32648036
6	rs9271419	32587846	T	2.166	1.72E-07	chr:6:32648036
6	rs17205914	32651680	G	0.3833	1.73E-07	chr:6:32648036
6	rs17205921	32651685	T	0.3833	1.73E-07	chr:6:32648036
6	rs17212258	32651690	T	0.3833	1.73E-07	chr:6:32648036
6	rs9270430	32558661	T	4.773	1.76E-07	chr:6:32648036
6	rs554680493	32611362	T	3.329	1.77E-07	chr:6:32648036
6	rs17211545	32602608	C	0.3709	1.78E-07	chr:6:32648036
6	rs34107231	32600074	G	0.3532	1.78E-07	chr:6:32648036
6	rs115508098	32608981	G	1.918	1.78E-07	chr:6:32648036
6	rs9273777	32629443	T	0.4134	1.79E-07	chr:6:32648036
6	rs9271555	32590319	T	0.4128	1.80E-07	chr:6:32648036
6	rs9271556	32590324	T	0.4128	1.80E-07	chr:6:32648036
6	rs9271277	32580998	T	0.4779	1.80E-07	chr:6:32648036
6	rs3134982	32646884	T	0.4274	1.81E-07	chr:6:32648036
6	rs9271458	32588453	A	2.331	1.81E-07	chr:6:32648036
6	rs9271586	32590899	T	2.201	1.82E-07	chr:6:32648036
6	rs9271587	32590916	G	2.201	1.82E-07	chr:6:32648036
6	rs9271588	32590953	C	2.201	1.82E-07	chr:6:32648036
6	rs567470347	32525111	C	2.694	1.83E-07	chr:6:32648036
6	rs41285651	32551785	G	0.4716	1.83E-07	chr:6:32648036
6	rs115405728	32481749	C	1.817	1.83E-07	chr:6:32648036
6	rs74345507	32459572	C	1.983	1.83E-07	chr:6:32648036
6	rs79893879	32459554	A	1.983	1.83E-07	chr:6:32648036
6	rs35242582	32600057	G	0.357	1.85E-07	chr:6:32648036
6	rs9274107	32630178	A	2.383	1.86E-07	chr:6:32648036
6	rs28891398	32639857	A	0.3908	1.86E-07	chr:6:32648036
6	rs28891399	32639876	C	0.3908	1.86E-07	chr:6:32648036

6	rs2858859	32582275	G	0.4571	1.87E-07	chr:6:32648036
6	rs5004275	32582190	G	0.4571	1.87E-07	chr:6:32648036
6	rs5004276	32582332	G	0.4571	1.87E-07	chr:6:32648036
6	rs5004277	32582354	C	0.4571	1.87E-07	chr:6:32648036
6	rs521512	32581982	T	0.4571	1.87E-07	chr:6:32648036
6	rs641685	32582003	G	0.4571	1.87E-07	chr:6:32648036
6	rs9271331	32582672	C	0.4571	1.87E-07	chr:6:32648036
6	rs9271332	32582674	T	0.4571	1.87E-07	chr:6:32648036
6	rs11970000	32598291	G	0.3655	1.89E-07	chr:6:32648036
6	rs9271278	32581016	A	0.4782	1.89E-07	chr:6:32648036
6	rs9271279	32581018	A	0.4782	1.89E-07	chr:6:32648036
6	rs2894266	32485924	A	3.376	1.91E-07	chr:6:32648036
6	rs9271268	32580806	G	0.4608	1.91E-07	chr:6:32648036
6	rs77572094	32502885	T	2.075	1.91E-07	chr:6:32648036
6	rs28366283	32560288	C	2.064	1.92E-07	chr:6:32648036
6	rs28746984	32653819	A	0.3737	1.93E-07	chr:6:32648036
6	rs9273505	32628420	T	2.18	1.93E-07	chr:6:32648036
6	rs9273781	32629447	A	0.4137	1.94E-07	chr:6:32648036
6	rs183872958	32552538	T	2.993	1.94E-07	chr:6:32648036
6	rs9272320	32604124	A	2.065	1.94E-07	chr:6:32648036
6	rs9273265	32614318	G	0.5295	1.95E-07	chr:6:32648036
6	rs73728637	32594420	C	0.3597	1.96E-07	chr:6:32648036
6	rs35635298	32600079	C	0.3546	1.97E-07	chr:6:32648036
6	rs3997849	32682402	T	2.357	1.98E-07	chr:6:32648036
6	rs281863660	32630721	G	1.964	1.99E-07	chr:6:32648036
6	rs281863661	32630720	T	1.964	1.99E-07	chr:6:32648036
6	rs79293578	32503561	T	2.223	2.02E-07	chr:6:32648036
6	rs9271299	32581537	G	0.4592	2.03E-07	chr:6:32648036
6	rs180852758	32459251	G	2.554	2.04E-07	chr:6:32648036
6	rs9271724	32593552	C	0.3903	2.07E-07	chr:6:32648036
6	rs116586023	32473199	T	2.135	2.07E-07	chr:6:32648036
6	rs2395195	32447989	A	0.3641	2.08E-07	chr:6:32648036
6	rs9271393	32587457	G	0.4122	2.08E-07	chr:6:32648036
6	rs3134976	32652305	A	0.346	2.09E-07	chr:6:32648036
6	rs2002779	32601684	T	0.3402	2.10E-07	chr:6:32648036
6	rs4988889	32634952	T	2.379	2.11E-07	chr:6:32648036
6	rs113319840	32507441	T	0.4887	2.12E-07	chr:6:32648036
6	rs141506183	32485776	A	2.58	2.13E-07	chr:6:32648036
6	rs9271495	32589242	C	0.41	2.13E-07	chr:6:32648036
6	rs9271496	32589244	C	0.41	2.13E-07	chr:6:32648036
6	rs9273754	32629409	A	0.4344	2.16E-07	chr:6:32648036
6	rs281875047	32631218	G	1.963	2.17E-07	chr:6:32648036
6	rs3134992	32638783	T	0.3465	2.18E-07	chr:6:32648036

6	rs9274905	32641251	T	0.5582	2.19E-07	chr:6:32648036
6	rs28747017	32654723	G	0.3938	2.19E-07	chr:6:32648036
6	rs28891375	32638757	A	0.3631	2.21E-07	chr:6:32648036
6	rs9271307	32581694	G	0.4646	2.23E-07	chr:6:32648036
6	rs281863485	32630968	C	2.147	2.24E-07	chr:6:32648036
6	rs62405569	32454061	T	2.257	2.24E-07	chr:6:32648036
6	rs540965243	32511378	C	4.56	2.25E-07	chr:6:32648036
6	rs576708145	32511375	C	4.56	2.25E-07	chr:6:32648036
6	rs35426875	32600135	C	0.3708	2.25E-07	chr:6:32648036
6	rs9275452	32671930	T	2.158	2.26E-07	chr:6:32648036
6	rs77296290	32635296	T	2.391	2.28E-07	chr:6:32648036
6	rs74955625	32502871	A	2.065	2.31E-07	chr:6:32648036
6	rs75288201	32541031	A	2.41	2.34E-07	chr:6:32648036
6	rs72844151	32519650	A	0.3773	2.37E-07	chr:6:32648036
6	rs17212336	32652026	A	0.395	2.38E-07	chr:6:32648036
6	rs535905056	32511341	G	4.108	2.38E-07	chr:6:32648036
6	rs553395545	32511337	A	4.108	2.38E-07	chr:6:32648036
6	rs571140115	32511322	T	4.108	2.38E-07	chr:6:32648036
6	rs574932946	32511340	T	4.108	2.38E-07	chr:6:32648036
6	rs9273144	32612915	C	2.62	2.39E-07	chr:6:32648036
6	rs9274579	32635312	A	0.4139	2.42E-07	chr:6:32648036
6	rs28746926	32650026	C	0.3969	2.42E-07	chr:6:32648036
6	rs28746927	32650029	T	0.3969	2.42E-07	chr:6:32648036
6	rs28747021	32654772	G	0.3945	2.44E-07	chr:6:32648036
6	rs9271610	32591601	T	0.4192	2.45E-07	chr:6:32648036
6	rs9271611	32591609	G	0.4192	2.45E-07	chr:6:32648036
6	rs34180045	32600066	G	0.3613	2.46E-07	chr:6:32648036
6	rs9271494	32589240	C	6.973	2.47E-07	chr:6:32648036
6	rs35305772	32556662	C	3.04	2.49E-07	chr:6:32648036
6	rs9275454	32671939	A	2.152	2.51E-07	chr:6:32648036
6	rs17212216	32651509	C	0.3878	2.51E-07	chr:6:32648036
6	rs2395204	32452005	G	2.356	2.51E-07	chr:6:32648036
6	rs28746921	32649831	A	0.398	2.53E-07	chr:6:32648036
6	rs114217086	32552954	G	2.794	2.56E-07	chr:6:32648036
6	rs116743472	32552953	T	2.794	2.56E-07	chr:6:32648036
6	rs28747019	32654757	A	0.3953	2.57E-07	chr:6:32648036
6	rs28747020	32654760	C	0.3953	2.57E-07	chr:6:32648036
6	rs28891414	32640593	T	0.3886	2.62E-07	chr:6:32648036
6	rs188413732	32498914	T	2.163	2.65E-07	chr:6:32648036
6	rs28366330	32561846	C	2.693	2.65E-07	chr:6:32648036
6	rs35321667	32501534	C	0.3751	2.67E-07	chr:6:32648036
6	rs17205879	32651576	C	0.3892	2.69E-07	chr:6:32648036
6	rs17205886	32651578	A	0.3892	2.69E-07	chr:6:32648036

6	rs3134967	32659315	A	0.3223	2.69E-07	chr:6:32648036
6	rs9271276	32580971	A	0.4828	2.70E-07	chr:6:32648036
6	rs35828545	32600099	C	0.3682	2.73E-07	chr:6:32648036
6	rs9271487	32588985	G	0.4118	2.73E-07	chr:6:32648036
6	rs9275033	32644388	A	0.4301	2.74E-07	chr:6:32648036
6	rs28746989	32653873	T	0.3768	2.75E-07	chr:6:32648036
6	rs28371354	32710555	G	4.178	2.75E-07	chr:6:32648036
6	rs28371355	32710557	T	4.178	2.75E-07	chr:6:32648036
6	rs28891369	32638602	T	0.3976	2.76E-07	chr:6:32648036
6	rs71536523	32520594	A	0.3394	2.76E-07	chr:6:32648036
6	rs75287767	32521735	T	0.4254	2.78E-07	chr:6:32648036
6	rs28891374	32638750	G	0.3668	2.80E-07	chr:6:32648036
6	rs9273501	32628361	A	2.16	2.81E-07	chr:6:32648036
6	rs75496002	32459855	C	2.084	2.83E-07	chr:6:32648036
6	rs9273067	32612213	C	0.5043	2.85E-07	chr:6:32648036
6	rs28599891	32452470	C	0.3927	2.87E-07	chr:6:32648036
6	rs13211123	32600249	C	0.3717	2.90E-07	chr:6:32648036
6	rs3134987	32642050	A	0.3485	2.93E-07	chr:6:32648036
6	rs9271329	32582635	A	0.4665	2.98E-07	chr:6:32648036
6	rs73729117	32519600	A	0.3773	2.98E-07	chr:6:32648036
6	rs17212230	32651588	A	0.3926	3.00E-07	chr:6:32648036
6	rs543622960	32511362	C	4.236	3.00E-07	chr:6:32648036
6	rs558497977	32511365	A	4.236	3.00E-07	chr:6:32648036
6	rs2239802	32411846	C	0.3832	3.03E-07	chr:6:32648036
6	rs2395182	32413317	G	0.3832	3.03E-07	chr:6:32648036
6	rs7196	32412571	A	0.3832	3.03E-07	chr:6:32648036
6	rs241410	32861651	C	2.12	3.03E-07	chr:6:32648036
6	rs112337463	32479229	G	1.851	3.05E-07	chr:6:32648036
6	rs28746992	32653919	G	0.3777	3.05E-07	chr:6:32648036
6	rs35326979	32653917	G	0.3777	3.05E-07	chr:6:32648036
6	rs75640879	32541021	T	2.39	3.06E-07	chr:6:32648036
6	rs74729326	32506796	T	2.085	3.07E-07	chr:6:32648036
6	rs9271553	32590299	T	0.422	3.08E-07	chr:6:32648036
6	rs75251583	32889082	G	2.452	3.08E-07	chr:6:32648036
6	rs281863466	32630996	T	2.359	3.08E-07	chr:6:32648036
6	rs540002313	32599555	A	0.3333	3.10E-07	chr:6:32648036
6	rs28746919	32649806	A	0.4011	3.11E-07	chr:6:32648036
6	rs28746920	32649813	A	0.4011	3.11E-07	chr:6:32648036
6	rs4959108	32591922	G	0.3178	3.12E-07	chr:6:32648036
6	rs12204922	32451613	C	2.427	3.13E-07	chr:6:32648036
6	rs74729795	32892138	C	2.452	3.16E-07	chr:6:32648036
6	rs419132	32210799	A	2.133	3.17E-07	chr:6:32648036
6	rs68148149	32511725	C	0.3728	3.18E-07	chr:6:32648036

6	rs28746960	32652938	G	0.3919	3.18E-07	chr:6:32648036
6	rs6908032	32643739	A	0.3799	3.19E-07	chr:6:32648036
6	rs145649164	32601918	A	0.2879	3.21E-07	chr:6:32648036
6	rs3830060	32633971	G	6.479	3.25E-07	chr:6:32648036
6	rs9271971	32597561	A	0.4032	3.27E-07	chr:6:32648036
6	rs6916779	32684317	C	0.4299	3.28E-07	chr:6:32648036
6	rs9271269	32580820	G	0.4699	3.29E-07	chr:6:32648036
6	rs114335201	32473206	C	2.062	3.29E-07	chr:6:32648036
6	rs9275638	32684760	T	0.427	3.33E-07	chr:6:32648036
6	rs9271687	32592985	A	0.3384	3.33E-07	chr:6:32648036
6	rs4246055	32592360	T	2.31	3.35E-07	chr:6:32648036
6	rs144353907	32552226	C	3.256	3.36E-07	chr:6:32648036
6	rs149614319	32552223	A	3.256	3.36E-07	chr:6:32648036
6	rs1383265	32739888	C	0.449	3.36E-07	chr:6:32648036
6	rs17205872	32651558	A	0.3896	3.44E-07	chr:6:32648036
6	rs571321362	32183599	T	0.4053	3.46E-07	chr:6:32648036
6	rs915896	32187721	T	0.4551	3.48E-07	chr:6:32648036
6	rs72492308	32509570	T	0.378	3.51E-07	chr:6:32648036
6	rs9271390	32587440	A	0.423	3.52E-07	chr:6:32648036
6	rs9271391	32587442	G	0.423	3.52E-07	chr:6:32648036
6	rs115462544	32552229	A	3.656	3.53E-07	chr:6:32648036
6	rs73397417	32521988	G	0.3746	3.55E-07	chr:6:32648036
6	rs115397315	32525087	C	2.574	3.57E-07	chr:6:32648036
6	rs535667342	32485778	T	2.54	3.58E-07	chr:6:32648036
6	rs552798572	32485775	C	2.54	3.58E-07	chr:6:32648036
6	rs28383206	32575167	G	2.696	3.60E-07	chr:6:32648036
6	rs9271374	32587041	G	0.3473	3.60E-07	chr:6:32648036
6	rs72853984	32511866	C	0.3784	3.61E-07	chr:6:32648036
6	rs9271303	32581647	A	0.4733	3.62E-07	chr:6:32648036
6	rs9271304	32581648	G	0.4733	3.62E-07	chr:6:32648036
6	rs9271305	32581649	C	0.4733	3.62E-07	chr:6:32648036
6	rs9271306	32581658	A	0.4733	3.62E-07	chr:6:32648036
6	rs9271548	32590234	A	0.4166	3.63E-07	chr:6:32648036
6	rs3132132	32901934	A	2.297	3.63E-07	chr:6:32648036
6	rs113260943	32509550	T	0.3779	3.64E-07	chr:6:32648036
6	rs9271861	32595287	T	0.4901	3.64E-07	chr:6:32648036
6	rs28366329	32561819	A	2.63	3.64E-07	chr:6:32648036
6	rs72844167	32520642	T	0.378	3.65E-07	chr:6:32648036
6	rs9274526	32634485	A	0.4263	3.67E-07	chr:6:32648036
6	rs552324616	32569207	T	9.39	3.74E-07	chr:6:32648036
6	rs559285850	32435549	A	1.92	3.74E-07	chr:6:32648036
6	rs9271980	32597636	A	0.3685	3.76E-07	chr:6:32648036
6	rs111372196	32516995	A	0.3945	3.77E-07	chr:6:32648036

6	rs77993032	32518093	C	0.378	3.78E-07	chr:6:32648036
6	rs915894	32190390	G	0.4556	3.78E-07	chr:6:32648036
6	rs9272159	32601129	A	0.3507	3.80E-07	chr:6:32648036
6	rs147613123	32518296	A	0.4132	3.81E-07	chr:6:32648036
6	rs36088464	32513497	A	0.378	3.81E-07	chr:6:32648036
6	rs28747011	32654554	G	0.3963	3.83E-07	chr:6:32648036
6	rs28747012	32654558	C	0.3963	3.83E-07	chr:6:32648036
6	rs561142110	32654552	G	0.3963	3.83E-07	chr:6:32648036
6	rs9271873	32595421	A	0.39	3.85E-07	chr:6:32648036
6	rs17205823	32651168	T	0.4013	3.88E-07	chr:6:32648036
6	rs17205830	32651180	C	0.4013	3.88E-07	chr:6:32648036
6	rs28371353	32710510	G	4.047	3.95E-07	chr:6:32648036
6	rs9274498	32633941	G	2.329	3.96E-07	chr:6:32648036
6	rs4959030	32591751	A	0.3208	3.97E-07	chr:6:32648036
6	rs113068532	32518301	G	0.4133	4.00E-07	chr:6:32648036
6	rs281863148	32631652	A	0.5016	4.01E-07	chr:6:32648036
6	rs9271578	32590635	T	2.136	4.03E-07	chr:6:32648036
6	rs1265795	32184024	T	0.4098	4.03E-07	chr:6:32648036
6	rs206014	32183701	G	0.4098	4.03E-07	chr:6:32648036
6	rs384247	32184574	A	0.4098	4.03E-07	chr:6:32648036
6	rs404860	32184345	C	0.4098	4.03E-07	chr:6:32648036
6	rs431541	32186193	C	0.4098	4.03E-07	chr:6:32648036
6	rs74347248	32459878	T	2.074	4.04E-07	chr:6:32648036
6	rs28746909	32648975	A	0.4035	4.08E-07	chr:6:32648036
6	rs74728615	32459866	A	2.048	4.10E-07	chr:6:32648036
6	rs111940905	32473454	G	2.17	4.12E-07	chr:6:32648036
6	rs67426377	32514457	C	0.3782	4.14E-07	chr:6:32648036
6	rs9274535	32634620	T	0.4356	4.16E-07	chr:6:32648036
6	rs3134970	32654202	T	0.3441	4.25E-07	chr:6:32648036
6	rs28724247	32629467	A	2.074	4.25E-07	chr:6:32648036
6	rs113276642	32525000	G	2.996	4.27E-07	chr:6:32648036
6	rs35063846	32550733	C	2.954	4.28E-07	chr:6:32648036
6	rs9271737	32593690	C	1.933	4.29E-07	chr:6:32648036
6	rs9274114	32630201	C	2.313	4.30E-07	chr:6:32648036
6	rs9271275	32580961	A	0.4909	4.33E-07	chr:6:32648036
6	rs28891411	32640509	A	0.4032	4.40E-07	chr:6:32648036
6	rs9275222	32659516	T	0.4547	4.41E-07	chr:6:32648036
6	rs3129871	32406342	C	2.127	4.45E-07	chr:6:32648036
6	rs72492326	32520420	T	0.3783	4.46E-07	chr:6:32648036
6	rs28746907	32648937	A	0.405	4.46E-07	chr:6:32648036
6	rs143722527	32479580	C	1.909	4.51E-07	chr:6:32648036
6	rs281863143	32631657	T	0.5025	4.53E-07	chr:6:32648036
6	rs28891435	32641241	G	0.5094	4.56E-07	chr:6:32648036

6	rs17613367	32622063	A	1.723	4.57E-07	chr:6:32648036
6	rs550053442	32599693	A	0.2974	4.57E-07	chr:6:32648036
6	rs2760985	32566398	A	7.035	4.58E-07	chr:6:32648036
6	rs114803358	32601708	A	0.3937	4.59E-07	chr:6:32648036
6	rs77233532	32510740	A	0.5071	4.60E-07	chr:6:32648036
6	rs3828840	32520907	T	0.3783	4.60E-07	chr:6:32648036
6	rs2256594	32186872	G	0.4115	4.61E-07	chr:6:32648036
6	rs438475	32186245	A	0.4114	4.62E-07	chr:6:32648036
6	rs9271982	32597640	A	0.372	4.63E-07	chr:6:32648036
6	rs281863676	32630701	T	1.941	4.64E-07	chr:6:32648036
6	rs9268830	32427572	G	0.3736	4.64E-07	chr:6:32648036
6	rs9273500	32628342	T	0.3323	4.66E-07	chr:6:32648036
6	rs114567049	32601719	A	0.3951	4.66E-07	chr:6:32648036
6	rs9271696	32593085	C	0.4304	4.73E-07	chr:6:32648036
6	rs116079706	32479557	C	1.911	4.74E-07	chr:6:32648036
6	rs28891471	32642473	G	0.413	4.79E-07	chr:6:32648036
6	rs72848261	32601790	C	0.3971	4.79E-07	chr:6:32648036
6	rs9271872	32595418	G	0.394	4.79E-07	chr:6:32648036
6	rs552568377	32511319	C	3.859	4.80E-07	chr:6:32648036
6	rs115707905	32516930	G	0.3912	4.82E-07	chr:6:32648036
6	rs116184728	32516935	T	0.3912	4.82E-07	chr:6:32648036
6	rs260547	32907278	C	2.512	4.83E-07	chr:6:32648036
6	rs9272328	32604196	T	0.394	4.86E-07	chr:6:32648036
6	rs113608847	32554284	G	2.871	4.87E-07	chr:6:32648036
6	rs114034156	32479305	G	1.83	4.91E-07	chr:6:32648036
6	rs34523106	32513724	C	0.3807	4.91E-07	chr:6:32648036
6	rs201854374	32507311	C	0.535	4.92E-07	chr:6:32648036
6	rs281863430	32631039	A	0.4234	4.96E-07	chr:6:32648036
6	rs9274497	32633928	A	2.308	4.98E-07	chr:6:32648036
6	rs28724237	32629046	C	2.044	5.06E-07	chr:6:32648036
6	rs4502931	32380782	T	0.4482	5.11E-07	chr:6:32648036
6	rs5007259	32379101	T	0.4482	5.11E-07	chr:6:32648036
6	rs5007263	32378982	A	0.4482	5.11E-07	chr:6:32648036
6	rs5007264	32378877	C	0.4482	5.11E-07	chr:6:32648036
6	rs5007265	32378866	T	0.4482	5.11E-07	chr:6:32648036
6	rs5007266	32378834	T	0.4482	5.11E-07	chr:6:32648036
6	rs6911383	32379682	T	0.4482	5.11E-07	chr:6:32648036
6	rs6918317	32381210	T	0.4482	5.11E-07	chr:6:32648036
6	rs6932542	32380262	A	0.4482	5.11E-07	chr:6:32648036
6	rs6932810	32380190	G	0.4482	5.11E-07	chr:6:32648036
6	rs6938337	32381041	C	0.4482	5.11E-07	chr:6:32648036
6	rs7759742	32381736	T	0.4482	5.11E-07	chr:6:32648036
6	rs9268503	32377061	C	0.4482	5.11E-07	chr:6:32648036

6	rs9268504	32377116	C	0.4482	5.11E-07	chr:6:32648036
6	rs9268506	32377470	C	0.4482	5.11E-07	chr:6:32648036
6	rs9268507	32377539	A	0.4482	5.11E-07	chr:6:32648036
6	rs9268508	32377588	T	0.4482	5.11E-07	chr:6:32648036
6	rs9268513	32378787	T	0.4482	5.11E-07	chr:6:32648036
6	rs9268518	32380818	A	0.4482	5.11E-07	chr:6:32648036
6	rs9268519	32380826	G	0.4482	5.11E-07	chr:6:32648036
6	rs9268520	32380860	C	0.4482	5.11E-07	chr:6:32648036
6	rs968154	32380785	G	0.4482	5.11E-07	chr:6:32648036
6	rs28891481	32642899	A	0.4092	5.12E-07	chr:6:32648036
6	rs28891482	32642906	A	0.4092	5.12E-07	chr:6:32648036
6	rs9271965	32597473	C	0.4053	5.14E-07	chr:6:32648036
6	rs77158649	32459875	C	2.06	5.15E-07	chr:6:32648036
6	rs78091375	32459877	G	2.06	5.15E-07	chr:6:32648036
6	rs9273561	32628998	C	2.07	5.17E-07	chr:6:32648036
6	rs78241440	32509180	G	0.3877	5.29E-07	chr:6:32648036
6	rs181280314	32481838	C	5.275	5.30E-07	chr:6:32648036
6	rs569932079	32481837	T	5.275	5.30E-07	chr:6:32648036
6	rs9274430	32633054	C	0.5214	5.31E-07	chr:6:32648036
6	rs76924329	32541094	G	2.287	5.31E-07	chr:6:32648036
6	rs28746908	32648963	A	0.4076	5.35E-07	chr:6:32648036
6	rs17208902	32379547	T	2.246	5.37E-07	chr:6:32648036
6	rs9275203	32656947	A	6.887	5.40E-07	chr:6:32648036
6	rs553858906	32183602	C	0.4112	5.41E-07	chr:6:32648036
6	rs9274747	32638071	A	6.867	5.42E-07	chr:6:32648036
6	rs545019763	32183815	A	0.4109	5.44E-07	chr:6:32648036
6	rs9271543	32590097	A	0.4208	5.44E-07	chr:6:32648036
6	rs73729116	32519558	C	0.3787	5.48E-07	chr:6:32648036
6	rs5007258	32379239	A	0.4488	5.52E-07	chr:6:32648036
6	rs5007260	32379047	G	0.4488	5.52E-07	chr:6:32648036
6	rs5007261	32379031	G	0.4488	5.52E-07	chr:6:32648036
6	rs5007262	32379011	A	0.4488	5.52E-07	chr:6:32648036
6	rs3104373	32600375	T	0.3267	5.55E-07	chr:6:32648036
6	rs28891472	32642515	C	0.4154	5.62E-07	chr:6:32648036
6	rs28891473	32642525	T	0.4154	5.62E-07	chr:6:32648036
6	rs9271281	32581059	C	0.4913	5.63E-07	chr:6:32648036
6	rs9271282	32581062	T	0.4913	5.63E-07	chr:6:32648036
6	rs541685416	32558854	C	2.583	5.66E-07	chr:6:32648036
6	rs563619413	32558855	A	2.583	5.66E-07	chr:6:32648036
6	rs431722	32187722	C	2.048	5.66E-07	chr:6:32648036
6	rs13202749	32585299	G	0.3406	5.68E-07	chr:6:32648036
6	rs9271514	32589613	A	0.4219	5.72E-07	chr:6:32648036
6	rs9271515	32589645	A	0.4219	5.72E-07	chr:6:32648036

6	rs28371176	32612839	G	4.679	5.72E-07	chr:6:32648036
6	rs9271274	32580935	A	0.4915	5.74E-07	chr:6:32648036
6	rs9271176	32578127	A	0.3607	5.74E-07	chr:6:32648036
6	rs3134979	32650178	A	0.3431	5.78E-07	chr:6:32648036
6	rs1136787	32487427	A	0.4953	5.78E-07	chr:6:32648036
6	rs35117964	32568146	G	6.145	5.80E-07	chr:6:32648036
6	rs72844177	32520990	G	0.3784	5.83E-07	chr:6:32648036
6	rs72848254	32601375	G	0.364	5.83E-07	chr:6:32648036
6	rs9275151	32651936	T	6.26	5.84E-07	chr:6:32648036
6	rs9275167	32653263	G	6.26	5.84E-07	chr:6:32648036
6	rs28746922	32649844	G	0.4121	5.87E-07	chr:6:32648036
6	rs577650925	32183813	G	0.4174	5.92E-07	chr:6:32648036
6	rs79350467	32527476	T	1.952	5.97E-07	chr:6:32648036
6	rs3763349	32808232	A	2.07	5.98E-07	chr:6:32648036
6	rs116575354	32524989	C	2.939	5.98E-07	chr:6:32648036
6	rs28471056	32603563	G	0.3548	5.98E-07	chr:6:32648036
6	rs2647066	32571122	T	6.132	6.00E-07	chr:6:32648036
6	rs2760990	32571110	A	6.132	6.00E-07	chr:6:32648036
6	rs111480903	32478848	A	1.815	6.02E-07	chr:6:32648036
6	rs28724243	32629347	C	2.028	6.06E-07	chr:6:32648036
6	rs28746882	32638175	A	0.4072	6.07E-07	chr:6:32648036
6	rs281874998	32631273	T	1.9	6.08E-07	chr:6:32648036
6	rs112218095	32490813	T	4.69	6.09E-07	chr:6:32648036
6	rs112572344	32473495	G	1.926	6.11E-07	chr:6:32648036
6	rs28746943	32652634	A	0.4035	6.14E-07	chr:6:32648036
6	rs17212329	32652005	T	0.4102	6.15E-07	chr:6:32648036
6	rs111813052	32554277	A	2.892	6.18E-07	chr:6:32648036
6	rs72844166	32520632	G	0.3918	6.20E-07	chr:6:32648036
6	rs6906730	32379445	T	0.4515	6.20E-07	chr:6:32648036
6	rs35597441	32555545	T	8.048	6.20E-07	chr:6:32648036
6	rs73728598	32601447	G	0.3702	6.21E-07	chr:6:32648036
6	rs281874818	32631430	G	0.4462	6.21E-07	chr:6:32648036
6	rs112996372	32517005	G	0.4072	6.24E-07	chr:6:32648036
6	rs28746923	32649849	G	0.4123	6.26E-07	chr:6:32648036
6	rs28746924	32649854	A	0.4123	6.26E-07	chr:6:32648036
6	rs116277371	32392330	A	6.012	6.28E-07	chr:6:32648036
6	rs5024432	32684468	T	2.224	6.32E-07	chr:6:32648036
6	rs527409566	32684514	G	2.224	6.32E-07	chr:6:32648036
6	rs76733997	32453590	A	2.27	6.32E-07	chr:6:32648036
6	rs68109409	32518552	G	0.4035	6.52E-07	chr:6:32648036
6	rs73727962	32509050	A	0.4038	6.52E-07	chr:6:32648036
6	rs76722140	32509053	C	0.4038	6.52E-07	chr:6:32648036
6	rs113301125	32458328	G	0.4788	6.52E-07	chr:6:32648036

6	rs9275476	32672624	C	6.733	6.61E-07	chr:6:32648036
6	rs9275477	32672641	C	6.733	6.61E-07	chr:6:32648036
6	rs111830089	32496843	A	7.49	6.65E-07	chr:6:32648036
6	rs17879995	32552072	G	7.49	6.65E-07	chr:6:32648036
6	rs72845733	32527305	A	0.4876	6.65E-07	chr:6:32648036
6	rs76699014	32555838	T	0.4001	6.68E-07	chr:6:32648036
6	rs28619202	32451650	T	0.4548	6.70E-07	chr:6:32648036
6	rs3134994	32637680	T	0.3426	6.75E-07	chr:6:32648036
6	rs281863431	32631038	G	2.293	6.78E-07	chr:6:32648036
6	rs72853943	32509260	C	0.3921	6.84E-07	chr:6:32648036
6	rs28371352	32708220	T	3.996	6.84E-07	chr:6:32648036
6	rs143845848	32481954	A	2.119	6.90E-07	chr:6:32648036
6	rs199720289	32481962	A	2.119	6.90E-07	chr:6:32648036
6	rs201894254	32481961	C	2.119	6.90E-07	chr:6:32648036
6	rs35670775	32544455	T	2.456	6.97E-07	chr:6:32648036
6	rs3129962	32379383	C	2.228	6.99E-07	chr:6:32648036
6	rs72492336	32527112	C	0.3915	6.99E-07	chr:6:32648036
6	rs9271283	32581092	T	0.4861	7.02E-07	chr:6:32648036
6	rs72844134	32518196	G	0.4041	7.02E-07	chr:6:32648036
6	rs533779530	32453612	G	0.4627	7.18E-07	chr:6:32648036
6	rs28891437	32641342	A	0.5119	7.20E-07	chr:6:32648036
6	rs9268501	32376882	T	0.453	7.22E-07	chr:6:32648036
6	rs9268502	32376932	C	0.453	7.22E-07	chr:6:32648036
6	rs281875006	32631265	T	1.889	7.23E-07	chr:6:32648036
6	rs77932549	32524736	G	0.5212	7.24E-07	chr:6:32648036
6	rs78025613	32524735	A	0.5212	7.24E-07	chr:6:32648036
6	rs2454139	32566635	C	6.418	7.33E-07	chr:6:32648036
6	rs2647059	32569362	C	6.418	7.33E-07	chr:6:32648036
6	rs2760983	32565601	G	6.418	7.33E-07	chr:6:32648036
6	rs34039593	32570311	G	6.418	7.33E-07	chr:6:32648036
6	rs34331363	32568439	T	6.418	7.33E-07	chr:6:32648036
6	rs35226637	32568448	A	6.418	7.33E-07	chr:6:32648036
6	rs687308	32567256	T	6.418	7.33E-07	chr:6:32648036
6	rs28746880	32638146	A	0.4114	7.35E-07	chr:6:32648036
6	rs553392189	32441275	C	5.364	7.39E-07	chr:6:32648036
6	rs577192394	32451875	G	5.364	7.39E-07	chr:6:32648036
6	rs9269562	32544407	A	0.3763	7.39E-07	chr:6:32648036
6	rs552672286	32554258	C	2.932	7.41E-07	chr:6:32648036
6	rs113556768	32496926	G	8.1	7.47E-07	chr:6:32648036
6	rs74290590	32510539	C	0.4124	7.55E-07	chr:6:32648036
6	rs3763314	32376618	C	0.448	7.55E-07	chr:6:32648036
6	rs9272337	32604273	A	2.921	7.61E-07	chr:6:32648036
6	rs560259947	32458033	C	2.437	7.62E-07	chr:6:32648036

6	rs200094698	32532973	A	2.468	7.76E-07	chr:6:32648036
6	rs34350244	32561465	T	7.284	7.80E-07	chr:6:32648036
6	rs35118762	32560631	T	7.284	7.80E-07	chr:6:32648036
6	rs35265698	32561334	G	7.284	7.80E-07	chr:6:32648036
6	rs35294087	32561466	G	7.284	7.80E-07	chr:6:32648036
6	rs35525122	32561283	A	7.284	7.80E-07	chr:6:32648036
6	rs111662020	32518444	A	0.4435	7.81E-07	chr:6:32648036
6	rs28746955	32652858	C	0.3897	7.82E-07	chr:6:32648036
6	rs34928543	32560695	C	7.915	7.87E-07	chr:6:32648036
6	rs138002663	32473542	C	1.949	8.07E-07	chr:6:32648036
6	rs199690134	32487449	T	2.95	8.09E-07	chr:6:32648036
6	rs371225543	32601968	T	0.4076	8.10E-07	chr:6:32648036
6	rs28746947	32652747	A	0.4013	8.13E-07	chr:6:32648036
6	rs534494500	32709338	G	4.777	8.18E-07	chr:6:32648036
6	rs549426719	32709341	G	4.777	8.18E-07	chr:6:32648036
6	rs567649680	32709343	G	4.777	8.18E-07	chr:6:32648036
6	rs9274284	32631783	G	0.5306	8.18E-07	chr:6:32648036
6	rs116218325	32526611	G	0.445	8.26E-07	chr:6:32648036
6	rs201277028	32526612	T	0.445	8.26E-07	chr:6:32648036
6	rs77858388	32526619	A	0.445	8.26E-07	chr:6:32648036
6	rs409687	32195750	T	0.4584	8.30E-07	chr:6:32648036
6	rs9271446	32588344	A	0.4558	8.34E-07	chr:6:32648036
6	rs9268505	32377408	T	0.3847	8.38E-07	chr:6:32648036
6	rs111739306	32516848	T	0.3925	8.47E-07	chr:6:32648036
6	rs9270161	32555558	A	0.3937	8.49E-07	chr:6:32648036
6	rs111693298	32518251	T	2.245	8.50E-07	chr:6:32648036
6	rs9274436	32633131	C	0.4703	8.62E-07	chr:6:32648036
6	rs9274428	32633041	T	0.5285	8.62E-07	chr:6:32648036
6	rs6905975	32626139	C	0.4685	8.63E-07	chr:6:32648036
6	rs3129759	32585163	G	0.4823	8.70E-07	chr:6:32648036
6	rs35882239	32522576	A	0.3967	8.73E-07	chr:6:32648036
6	rs373544996	32663873	G	6.336	8.73E-07	chr:6:32648036
6	rs3957146	32681530	C	6.336	8.73E-07	chr:6:32648036
6	rs4273728	32678491	C	6.336	8.73E-07	chr:6:32648036
6	rs9274619	32635954	A	6.336	8.73E-07	chr:6:32648036
6	rs9274830	32639836	A	6.336	8.73E-07	chr:6:32648036
6	rs9274835	32639931	T	6.336	8.73E-07	chr:6:32648036
6	rs9274837	32639972	T	6.336	8.73E-07	chr:6:32648036
6	rs9275092	32648987	T	6.336	8.73E-07	chr:6:32648036
6	rs9275095	32649088	G	6.336	8.73E-07	chr:6:32648036
6	rs9275097	32649126	G	6.336	8.73E-07	chr:6:32648036
6	rs9275098	32649161	T	6.336	8.73E-07	chr:6:32648036
6	rs9275184	32654714	C	6.336	8.73E-07	chr:6:32648036

6	rs9275185	32655305	T	6.336	8.73E-07	chr:6:32648036
6	rs9275201	32656723	A	6.336	8.73E-07	chr:6:32648036
6	rs9275206	32657565	G	6.336	8.73E-07	chr:6:32648036
6	rs9275207	32657710	G	6.336	8.73E-07	chr:6:32648036
6	rs9275213	32658335	C	6.336	8.73E-07	chr:6:32648036
6	rs9275214	32658665	A	6.336	8.73E-07	chr:6:32648036
6	rs9275221	32659099	C	6.336	8.73E-07	chr:6:32648036
6	rs9275223	32659839	A	6.336	8.73E-07	chr:6:32648036
6	rs9275275	32662607	A	6.336	8.73E-07	chr:6:32648036
6	rs9275293	32663308	C	6.336	8.73E-07	chr:6:32648036
6	rs9275296	32663431	C	6.336	8.73E-07	chr:6:32648036
6	rs9275300	32664126	G	6.336	8.73E-07	chr:6:32648036
6	rs9275307	32664990	T	6.336	8.73E-07	chr:6:32648036
6	rs9275308	32665255	A	6.336	8.73E-07	chr:6:32648036
6	rs9275310	32665319	G	6.336	8.73E-07	chr:6:32648036
6	rs9275311	32665640	T	6.336	8.73E-07	chr:6:32648036
6	rs9275313	32665759	T	6.336	8.73E-07	chr:6:32648036
6	rs9275315	32665912	A	6.336	8.73E-07	chr:6:32648036
6	rs9275334	32667107	C	6.336	8.73E-07	chr:6:32648036
6	rs9275356	32667850	G	6.336	8.73E-07	chr:6:32648036
6	rs9275387	32669013	C	6.336	8.73E-07	chr:6:32648036
6	rs9275394	32669454	C	6.336	8.73E-07	chr:6:32648036
6	rs9275395	32669483	G	6.336	8.73E-07	chr:6:32648036
6	rs9275400	32669761	G	6.336	8.73E-07	chr:6:32648036
6	rs9275422	32670548	A	6.336	8.73E-07	chr:6:32648036
6	rs9275426	32670912	C	6.336	8.73E-07	chr:6:32648036
6	rs9275429	32671057	A	6.336	8.73E-07	chr:6:32648036
6	rs9275430	32671086	T	6.336	8.73E-07	chr:6:32648036
6	rs9275434	32671247	T	6.336	8.73E-07	chr:6:32648036
6	rs9275435	32671332	C	6.336	8.73E-07	chr:6:32648036
6	rs9275437	32671412	T	6.336	8.73E-07	chr:6:32648036
6	rs9275442	32671755	A	6.336	8.73E-07	chr:6:32648036
6	rs9275445	32671811	A	6.336	8.73E-07	chr:6:32648036
6	rs9275486	32673099	A	6.336	8.73E-07	chr:6:32648036
6	rs9275495	32673574	T	6.336	8.73E-07	chr:6:32648036
6	rs9275503	32673931	G	6.336	8.73E-07	chr:6:32648036
6	rs9275530	32675523	C	6.336	8.73E-07	chr:6:32648036
6	rs9275532	32675634	G	6.336	8.73E-07	chr:6:32648036
6	rs9275541	32676159	G	6.336	8.73E-07	chr:6:32648036
6	rs9275583	32680299	A	6.336	8.73E-07	chr:6:32648036
6	rs9275592	32680620	T	6.336	8.73E-07	chr:6:32648036
6	rs9273200	32613537	C	0.5726	8.73E-07	chr:6:32648036
6	rs72844190	32521785	T	0.3982	8.76E-07	chr:6:32648036

6	rs72844114	32514030	G	0.3924	8.81E-07	chr:6:32648036
6	rs28629976	32625221	C	2.997	8.81E-07	chr:6:32648036
6	rs9272458	32605558	G	0.3813	8.89E-07	chr:6:32648036
6	rs59281193	32423601	T	0.3897	8.90E-07	chr:6:32648036
6	rs79138148	32516351	C	0.3922	9.00E-07	chr:6:32648036
6	rs3763313	32376471	C	2.24	9.03E-07	chr:6:32648036
6	rs28747056	32656544	G	0.4081	9.06E-07	chr:6:32648036
6	rs9275608	32683708	G	0.4499	9.06E-07	chr:6:32648036
6	rs9275609	32683713	G	0.4499	9.06E-07	chr:6:32648036
6	rs28891479	32642750	A	0.4249	9.07E-07	chr:6:32648036
6	rs564968289	32558618	A	2.978	9.07E-07	chr:6:32648036
6	rs142244845	32472941	A	3.058	9.08E-07	chr:6:32648036
6	rs4377824	32635491	T	0.4454	9.13E-07	chr:6:32648036
6	rs241413	32859000	G	0.4643	9.20E-07	chr:6:32648036
6	rs28724142	32555235	A	2.92	9.24E-07	chr:6:32648036
6	rs28724143	32555236	T	2.92	9.24E-07	chr:6:32648036
6	rs34855541	32559825	G	7.155	9.27E-07	chr:6:32648036
6	rs6907291	32950934	T	2.455	9.28E-07	chr:6:32648036
6	rs9276943	32956454	C	2.455	9.28E-07	chr:6:32648036
6	rs73395587	32518339	T	0.4161	9.32E-07	chr:6:32648036
6	rs9271272	32580894	T	0.4963	9.40E-07	chr:6:32648036
6	rs9271273	32580902	G	0.4963	9.40E-07	chr:6:32648036
6	rs114281696	32525925	G	3.207	9.51E-07	chr:6:32648036
6	rs9274304	32631986	G	0.5586	9.54E-07	chr:6:32648036
6	rs9275163	32652880	C	6.203	9.55E-07	chr:6:32648036
6	rs112382456	32554296	C	2.82	9.57E-07	chr:6:32648036
6	rs281863401	32631075	C	1.912	9.68E-07	chr:6:32648036
6	rs9273531	32628712	T	6.723	9.69E-07	chr:6:32648036
6	rs115729463	32514301	C	0.4084	9.69E-07	chr:6:32648036
6	rs28772273	32659314	T	0.4097	9.81E-07	chr:6:32648036
6	rs57723712	32423409	C	0.3901	9.82E-07	chr:6:32648036
6	rs6457590	32421798	T	0.3901	9.82E-07	chr:6:32648036
6	rs6938382	32418227	A	0.3901	9.82E-07	chr:6:32648036
6	rs7760260	32421071	A	0.3901	9.82E-07	chr:6:32648036
6	rs7764351	32421631	G	0.3901	9.82E-07	chr:6:32648036
6	rs7776297	32424108	T	0.3901	9.82E-07	chr:6:32648036
6	rs9461748	32418948	C	0.3901	9.82E-07	chr:6:32648036
6	rs9767620	32426100	C	0.3901	9.82E-07	chr:6:32648036
6	rs9275416	32670196	T	2.068	9.83E-07	chr:6:32648036
6	rs201624597	32527297	G	0.4968	1.00E-06	chr:6:32648036
6	rs601945	32573415	G	6.575	1.00E-06	chr:6:32648036
6	rs111485252	32518374	T	0.4163	1.00E-06	chr:6:32648036
6	rs112927854	32663259	T	0.346	1.01E-06	chr:6:32648036

6	rs530460066	32629240	G	0.5043	1.01E-06	chr:6:32648036
6	rs2395206	32452176	G	0.458	1.01E-06	chr:6:32648036
6	rs149432428	32485861	G	2.197	1.02E-06	chr:6:32648036
6	rs9273394	32627082	A	0.516	1.02E-06	chr:6:32648036
6	rs9274443	32633239	C	0.4969	1.03E-06	chr:6:32648036
6	rs9275610	32683750	C	0.4509	1.03E-06	chr:6:32648036
6	rs546173503	32523200	C	2.571	1.04E-06	chr:6:32648036
6	rs28891397	32639777	A	0.4215	1.05E-06	chr:6:32648036
6	rs9268926	32433067	G	2.682	1.05E-06	chr:6:32648036
6	rs17613535	32630674	C	0.4781	1.05E-06	chr:6:32648036
6	rs200864308	32518611	G	0.4358	1.06E-06	chr:6:32648036
6	rs28746956	32652877	G	0.3983	1.07E-06	chr:6:32648036
6	rs116630455	32601946	C	0.4094	1.08E-06	chr:6:32648036
6	rs67587495	32518728	A	0.4049	1.09E-06	chr:6:32648036
6	rs111405624	32554254	T	2.867	1.09E-06	chr:6:32648036
6	rs34673795	32513988	G	0.3962	1.10E-06	chr:6:32648036
6	rs28746881	32638153	T	0.4177	1.11E-06	chr:6:32648036
6	rs72844137	32518615	G	0.4164	1.11E-06	chr:6:32648036
6	rs28746897	32648396	G	0.4408	1.11E-06	chr:6:32648036
6	rs113014982	32536382	T	0.5486	1.11E-06	chr:6:32648036
6	rs62405645	32517934	G	0.401	1.12E-06	chr:6:32648036
6	rs546068737	32517854	C	5.405	1.13E-06	chr:6:32648036
6	rs17206049	32652336	A	0.3973	1.13E-06	chr:6:32648036
6	rs9268143	32256473	G	2.059	1.14E-06	chr:6:32648036
6	rs28747057	32656553	T	0.4116	1.14E-06	chr:6:32648036
6	rs28747058	32656554	G	0.4116	1.14E-06	chr:6:32648036
6	rs28747059	32656558	G	0.4116	1.14E-06	chr:6:32648036
6	rs79996892	32507051	C	0.548	1.14E-06	chr:6:32648036
6	rs191277720	32458048	C	2.416	1.14E-06	chr:6:32648036
6	rs9276931	32928984	G	2.192	1.14E-06	chr:6:32648036
6	rs72848256	32601531	G	0.3832	1.16E-06	chr:6:32648036
6	rs28724242	32629331	G	1.983	1.16E-06	chr:6:32648036
6	rs111685796	32504453	G	0.4132	1.17E-06	chr:6:32648036
6	rs28746957	32652893	C	0.4063	1.17E-06	chr:6:32648036
6	rs9274237	32631256	G	2.324	1.17E-06	chr:6:32648036
6	rs563197321	32458258	T	2.374	1.18E-06	chr:6:32648036
6	rs28746958	32652913	C	0.409	1.20E-06	chr:6:32648036
6	rs28746959	32652914	A	0.409	1.20E-06	chr:6:32648036
6	rs9275340	32667458	T	2.034	1.21E-06	chr:6:32648036
6	rs533524385	32522291	G	2.33	1.21E-06	chr:6:32648036
6	rs559757855	32522285	C	2.33	1.21E-06	chr:6:32648036
6	rs5020945	32450134	C	3.35	1.21E-06	chr:6:32648036
6	rs184149877	32525731	A	0.5483	1.21E-06	chr:6:32648036

6	rs9271467	32588613	T	0.4468	1.21E-06	chr:6:32648036
6	rs9271468	32588616	G	0.4468	1.21E-06	chr:6:32648036
6	rs34553045	32561565	C	7.619	1.21E-06	chr:6:32648036
6	rs28747008	32654530	T	0.4169	1.21E-06	chr:6:32648036
6	rs28747009	32654531	A	0.4169	1.21E-06	chr:6:32648036
6	rs146320947	32534206	A	2.411	1.22E-06	chr:6:32648036
6	rs28688825	32587157	G	0.349	1.24E-06	chr:6:32648036
6	rs368833717	32485705	G	2.031	1.25E-06	chr:6:32648036
6	rs112216801	32601838	A	0.4261	1.27E-06	chr:6:32648036
6	rs56051108	32645530	A	6.528	1.28E-06	chr:6:32648036
6	rs112532723	32518151	G	0.4209	1.30E-06	chr:6:32648036
6	rs28366331	32561880	G	2.554	1.31E-06	chr:6:32648036
6	rs28366332	32561885	A	2.554	1.31E-06	chr:6:32648036
6	rs28366333	32561887	C	2.554	1.31E-06	chr:6:32648036
6	rs112740890	32473461	G	2.031	1.31E-06	chr:6:32648036
6	rs9274295	32631866	A	0.5363	1.31E-06	chr:6:32648036
6	rs547043452	32525326	G	0.5551	1.32E-06	chr:6:32648036
6	rs17212371	32652259	A	0.4036	1.32E-06	chr:6:32648036
6	rs77274878	32523348	A	2.322	1.33E-06	chr:6:32648036
6	rs55722612	32647158	G	5.802	1.34E-06	chr:6:32648036
6	rs9271534	32589953	C	0.4631	1.34E-06	chr:6:32648036
6	rs9274361	32632483	A	0.5438	1.35E-06	chr:6:32648036
6	rs9273423	32627390	T	0.48	1.35E-06	chr:6:32648036
6	rs6905775	32626021	A	5.427	1.36E-06	chr:6:32648036
6	rs9274528	32634508	C	0.4507	1.37E-06	chr:6:32648036
6	rs113441266	32479312	A	2.416	1.38E-06	chr:6:32648036
6	rs148598843	32479311	A	2.416	1.38E-06	chr:6:32648036
6	rs2395179	32407302	G	2.149	1.38E-06	chr:6:32648036
6	rs2395180	32407310	G	2.149	1.38E-06	chr:6:32648036
6	rs2395181	32407404	C	2.149	1.38E-06	chr:6:32648036
6	rs3129845	32396277	G	2.149	1.38E-06	chr:6:32648036
6	rs3129846	32396475	G	2.149	1.38E-06	chr:6:32648036
6	rs3129875	32407468	C	2.149	1.38E-06	chr:6:32648036
6	rs3129879	32408907	A	2.149	1.38E-06	chr:6:32648036
6	rs3135343	32396313	T	2.149	1.38E-06	chr:6:32648036
6	rs34117221	32568324	G	6.185	1.39E-06	chr:6:32648036
6	rs67062152	32527025	T	0.4057	1.39E-06	chr:6:32648036
6	rs28732242	32444078	A	2.363	1.39E-06	chr:6:32648036
6	rs72851021	32501395	T	0.4174	1.40E-06	chr:6:32648036
6	rs62405632	32514561	G	0.4173	1.40E-06	chr:6:32648036
6	rs34977123	32555736	G	7.336	1.41E-06	chr:6:32648036
6	rs62405649	32534491	G	0.4613	1.41E-06	chr:6:32648036
6	rs78316776	32508012	T	0.5332	1.42E-06	chr:6:32648036

6	rs79579660	32543433	G	2.565	1.42E-06	chr:6:32648036
6	rs9274195	32630758	A	1.886	1.44E-06	chr:6:32648036
6	rs17879599	32551959	G	0.3486	1.44E-06	chr:6:32648036
6	rs9273131	32612793	T	1.779	1.46E-06	chr:6:32648036
6	rs145586726	32489796	T	3.221	1.46E-06	chr:6:32648036
6	rs28891372	32638727	A	0.403	1.47E-06	chr:6:32648036
6	rs28891400	32640074	T	0.4222	1.48E-06	chr:6:32648036
6	rs28891401	32640075	G	0.4222	1.48E-06	chr:6:32648036
6	rs679242	32570958	T	5.834	1.48E-06	chr:6:32648036
6	rs553195173	32566329	C	6.147	1.48E-06	chr:6:32648036
6	rs72845730	32526731	A	0.4459	1.49E-06	chr:6:32648036
6	rs9274505	32634126	A	2.327	1.49E-06	chr:6:32648036
6	rs78387908	32503391	A	0.4879	1.49E-06	chr:6:32648036
6	rs115965987	32538598	G	0.4157	1.49E-06	chr:6:32648036
6	rs116555998	32538590	T	0.4157	1.49E-06	chr:6:32648036
6	rs200591586	32485857	A	2.16	1.49E-06	chr:6:32648036
6	rs6926737	32375745	G	0.4598	1.50E-06	chr:6:32648036
6	rs35599691	32543117	G	0.4721	1.50E-06	chr:6:32648036
6	rs9274336	32632287	G	1.663	1.52E-06	chr:6:32648036
6	rs151005270	32601874	T	0.4284	1.53E-06	chr:6:32648036
6	rs76517385	32519067	A	0.4171	1.54E-06	chr:6:32648036
6	rs9272336	32604264	G	0.4266	1.54E-06	chr:6:32648036
6	rs9271444	32588281	A	0.4608	1.54E-06	chr:6:32648036
6	rs59066278	32510239	T	0.4766	1.54E-06	chr:6:32648036
6	rs28636516	32606701	G	0.4637	1.54E-06	chr:6:32648036
6	rs4713572	32626952	C	2.129	1.57E-06	chr:6:32648036
6	rs533608307	32633023	T	0.5382	1.58E-06	chr:6:32648036
6	rs543964824	32677572	T	0.3593	1.58E-06	chr:6:32648036
6	rs6917212	32583005	C	0.4898	1.59E-06	chr:6:32648036
6	rs9469108	32380892	T	2.153	1.59E-06	chr:6:32648036
6	rs28746983	32653760	T	0.4215	1.59E-06	chr:6:32648036
6	rs1049053	32634405	C	0.4756	1.59E-06	chr:6:32648036
6	rs35653258	32568783	A	6.519	1.59E-06	chr:6:32648036
6	rs9271530	32589938	A	0.4666	1.60E-06	chr:6:32648036
6	rs9271531	32589939	A	0.4666	1.60E-06	chr:6:32648036
6	rs56347793	32646963	T	6.009	1.60E-06	chr:6:32648036
6	rs17206035	32652224	C	0.4125	1.61E-06	chr:6:32648036
6	rs68017248	32519262	A	0.3902	1.63E-06	chr:6:32648036
6	rs2647079	32566502	A	2.157	1.65E-06	chr:6:32648036
6	rs146797212	32507206	G	0.5536	1.65E-06	chr:6:32648036
6	rs17211091	32557585	C	17.38	1.65E-06	chr:6:32648036
6	rs9274447	32633282	C	2.252	1.65E-06	chr:6:32648036
6	rs3129886	32410576	T	0.4156	1.65E-06	chr:6:32648036

6	rs9268652	32409056	A	0.4156	1.65E-06	chr:6:32648036
6	rs5000807	32496471	A	1.945	1.65E-06	chr:6:32648036
6	rs75876639	32457809	T	2.266	1.66E-06	chr:6:32648036
6	rs113861133	32601911	C	0.4204	1.66E-06	chr:6:32648036
6	rs28891474	32642565	T	0.4343	1.67E-06	chr:6:32648036
6	rs28891475	32642570	C	0.4343	1.67E-06	chr:6:32648036
6	rs3129724	32677560	A	0.359	1.68E-06	chr:6:32648036
6	rs35062987	32590735	T	0.3934	1.68E-06	chr:6:32648036
6	rs28772275	32663439	A	0.4123	1.70E-06	chr:6:32648036
6	rs5000632	32663688	C	0.4123	1.70E-06	chr:6:32648036
6	rs6935069	32666156	A	0.4123	1.70E-06	chr:6:32648036
6	rs9348891	32664971	G	0.4123	1.70E-06	chr:6:32648036
6	rs9357152	32664960	G	0.4123	1.70E-06	chr:6:32648036
6	rs9394110	32658471	G	0.4123	1.70E-06	chr:6:32648036
6	rs2395202	32451906	T	0.4783	1.72E-06	chr:6:32648036
6	rs11967891	32500957	T	2.03	1.72E-06	chr:6:32648036
6	rs79912140	32519729	A	0.4398	1.73E-06	chr:6:32648036
6	rs3129883	32410137	T	0.4157	1.74E-06	chr:6:32648036
6	rs3132970	32230208	A	2.033	1.76E-06	chr:6:32648036
6	rs35074855	32561140	G	7.489	1.76E-06	chr:6:32648036
6	rs9268073	32232758	C	2.042	1.77E-06	chr:6:32648036
6	rs115773472	32486484	G	2.918	1.78E-06	chr:6:32648036
6	rs558721	32570573	T	6.069	1.79E-06	chr:6:32648036
6	rs1130456	32627379	T	0.4886	1.80E-06	chr:6:32648036
6	rs28732243	32444088	G	2.359	1.80E-06	chr:6:32648036
6	rs9272410	32604969	A	0.4306	1.80E-06	chr:6:32648036
6	rs9269211	32450058	C	0.4447	1.81E-06	chr:6:32648036
6	rs2071465	32805470	G	0.4739	1.82E-06	chr:6:32648036
6	rs78055135	32517412	A	2.207	1.82E-06	chr:6:32648036
6	rs114849159	32552788	C	2.561	1.82E-06	chr:6:32648036
6	rs9269661	32546259	C	0.4415	1.82E-06	chr:6:32648036
6	rs78232591	32458419	T	1.805	1.83E-06	chr:6:32648036
6	rs73726822	32527272	G	0.5197	1.83E-06	chr:6:32648036
6	rs535514039	32490360	C	3.617	1.84E-06	chr:6:32648036
6	rs79438035	32457830	G	2.234	1.84E-06	chr:6:32648036
6	rs79584190	32457822	T	2.234	1.84E-06	chr:6:32648036
6	rs281874787	32632165	A	0.5635	1.84E-06	chr:6:32648036
6	rs9275314	32665909	A	5.748	1.84E-06	chr:6:32648036
6	rs112324351	32457338	C	2.081	1.85E-06	chr:6:32648036
6	rs140190321	32511008	T	0.5462	1.86E-06	chr:6:32648036
6	rs2395232	32640217	A	0.3604	1.88E-06	chr:6:32648036
6	rs79601580	32507591	G	0.5141	1.89E-06	chr:6:32648036
6	rs17211965	32637355	G	0.419	1.90E-06	chr:6:32648036

6	rs3134995	32672089	T	0.359	1.92E-06	chr:6:32648036
6	rs12528622	32658259	A	0.3602	1.92E-06	chr:6:32648036
6	rs13206007	32663442	G	0.3602	1.92E-06	chr:6:32648036
6	rs13206018	32663497	T	0.3602	1.92E-06	chr:6:32648036
6	rs17206252	32665915	C	0.3602	1.92E-06	chr:6:32648036
6	rs17427431	32663644	A	0.3602	1.92E-06	chr:6:32648036
6	rs35122881	32663677	T	0.3602	1.92E-06	chr:6:32648036
6	rs68067315	32663340	A	0.3602	1.92E-06	chr:6:32648036
6	rs546957361	32224456	C	2.017	1.93E-06	chr:6:32648036
6	rs568672155	32224457	A	2.017	1.93E-06	chr:6:32648036
6	rs6908464	32224445	G	2.017	1.93E-06	chr:6:32648036
6	rs6908903	32224439	A	2.017	1.93E-06	chr:6:32648036
6	rs6931322	32224463	C	2.017	1.93E-06	chr:6:32648036
6	rs28724245	32629400	G	1.958	1.93E-06	chr:6:32648036
6	rs112323850	32521804	T	0.4444	1.94E-06	chr:6:32648036
6	rs6934185	32673233	G	0.4147	1.94E-06	chr:6:32648036
6	rs6934347	32673223	G	0.4147	1.94E-06	chr:6:32648036
6	rs9273472	32628045	C	0.5105	1.94E-06	chr:6:32648036
6	rs28895250	32444022	C	2.316	1.95E-06	chr:6:32648036
6	rs28891476	32642584	A	0.4337	1.95E-06	chr:6:32648036
6	rs200582841	32453810	A	0.4321	1.96E-06	chr:6:32648036
6	rs41270871	32628224	A	0.4155	1.96E-06	chr:6:32648036
6	rs9271440	32588240	C	0.4624	1.96E-06	chr:6:32648036
6	rs68027833	32630028	A	0.4689	1.98E-06	chr:6:32648036
6	rs9275402	32669837	C	1.988	1.99E-06	chr:6:32648036
6	rs9275403	32669838	T	1.988	1.99E-06	chr:6:32648036
6	rs28732205	32377712	T	2.12	1.99E-06	chr:6:32648036
6	rs114746879	32487080	T	5.965	1.99E-06	chr:6:32648036
6	rs116067927	32487074	G	5.965	1.99E-06	chr:6:32648036
6	rs535648472	32487066	T	5.965	1.99E-06	chr:6:32648036
6	rs9275324	32666635	T	6.056	2.00E-06	chr:6:32648036
6	rs9275325	32666651	T	6.056	2.00E-06	chr:6:32648036
6	rs9275326	32666660	T	6.056	2.00E-06	chr:6:32648036
6	rs28772281	32674086	A	0.4149	2.01E-06	chr:6:32648036
6	rs554742089	32451822	T	2.585	2.03E-06	chr:6:32648036
6	rs567852525	32452110	T	2.585	2.03E-06	chr:6:32648036
6	rs17205170	32602483	T	0.3784	2.05E-06	chr:6:32648036
6	rs547199864	32558886	T	2.453	2.05E-06	chr:6:32648036
6	rs183847206	32490350	T	2.32	2.06E-06	chr:6:32648036
6	rs111675168	32487104	G	5.052	2.06E-06	chr:6:32648036
6	rs112080536	32521594	A	0.4472	2.09E-06	chr:6:32648036
6	rs4713598	32806786	G	0.4635	2.10E-06	chr:6:32648036
6	rs377506388	32487038	G	6.095	2.10E-06	chr:6:32648036

6	rs1130457	32627367	T	0.4898	2.10E-06	chr:6:32648036
6	rs13191875	32663403	A	0.3616	2.11E-06	chr:6:32648036
6	rs532817093	32522191	G	2.332	2.11E-06	chr:6:32648036
6	rs9275351	32667793	A	5.995	2.12E-06	chr:6:32648036
6	rs17212643	32674865	T	0.4151	2.12E-06	chr:6:32648036
6	rs28772282	32674136	G	0.4151	2.12E-06	chr:6:32648036
6	rs6906698	32673425	T	0.4151	2.12E-06	chr:6:32648036
6	rs6907114	32673361	A	0.4151	2.12E-06	chr:6:32648036
6	rs9272349	32604402	G	0.4427	2.13E-06	chr:6:32648036
6	rs544126617	32510976	A	0.5474	2.13E-06	chr:6:32648036
6	rs577174379	32510979	G	0.5474	2.13E-06	chr:6:32648036
6	rs2760980	32565201	A	5.86	2.13E-06	chr:6:32648036
6	rs536757162	32684641	A	1.978	2.16E-06	chr:6:32648036
6	rs552183536	32684630	C	1.978	2.16E-06	chr:6:32648036
6	rs77592650	32457835	C	2.219	2.16E-06	chr:6:32648036
6	rs28891490	32660697	A	0.4167	2.17E-06	chr:6:32648036
6	rs28891491	32661080	A	0.4165	2.17E-06	chr:6:32648036
6	rs77867566	32485524	A	2.575	2.17E-06	chr:6:32648036
6	rs9275401	32669827	G	1.983	2.17E-06	chr:6:32648036
6	rs112219021	32518085	C	0.4204	2.18E-06	chr:6:32648036
6	rs538742897	32526773	C	0.482	2.20E-06	chr:6:32648036
6	rs28746965	32653136	G	0.4154	2.21E-06	chr:6:32648036
6	rs3096670	32236609	G	2.013	2.21E-06	chr:6:32648036
6	rs114500718	32524694	G	0.5279	2.22E-06	chr:6:32648036
6	rs34535888	32561527	C	7.327	2.23E-06	chr:6:32648036
6	rs28891470	32642454	T	0.4439	2.23E-06	chr:6:32648036
6	rs9274674	32636610	A	0.4375	2.24E-06	chr:6:32648036
6	rs17206042	32652244	A	0.4174	2.24E-06	chr:6:32648036
6	rs531329164	32496942	C	7.52	2.25E-06	chr:6:32648036
6	rs9368738	32658750	A	0.4168	2.26E-06	chr:6:32648036
6	rs9268130	32254312	C	2.011	2.28E-06	chr:6:32648036
6	rs141890570	32667412	A	0.3605	2.30E-06	chr:6:32648036
6	rs9275607	32683645	G	0.4688	2.30E-06	chr:6:32648036
6	rs601020	32573179	A	2.505	2.34E-06	chr:6:32648036
6	rs372568412	32523041	T	2.606	2.35E-06	chr:6:32648036
6	rs200519021	32537816	T	0.5817	2.35E-06	chr:6:32648036
6	rs113176001	32571483	C	1.708	2.36E-06	chr:6:32648036
6	rs28891492	32662451	T	0.4135	2.41E-06	chr:6:32648036
6	rs9274130	32630232	A	2.168	2.41E-06	chr:6:32648036
6	rs9274134	32630242	C	2.168	2.41E-06	chr:6:32648036
6	rs3135002	32668439	A	0.3636	2.43E-06	chr:6:32648036
6	rs375955233	32603002	T	3.356	2.43E-06	chr:6:32648036
6	rs34153824	32627627	T	0.3927	2.43E-06	chr:6:32648036

6	rs115795737	32459515	C	4.081	2.44E-06	chr:6:32648036
6	rs9269986	32552553	C	0.4841	2.44E-06	chr:6:32648036
6	rs9274384	32632635	C	1.757	2.45E-06	chr:6:32648036
6	rs17843590	32615850	G	1.719	2.46E-06	chr:6:32648036
6	rs2647058	32569304	T	2.117	2.47E-06	chr:6:32648036
6	rs9391747	32806949	T	0.4648	2.48E-06	chr:6:32648036
6	rs112477119	32489269	T	2.382	2.48E-06	chr:6:32648036
6	rs78633917	32501658	A	2.014	2.48E-06	chr:6:32648036
6	rs9368739	32669945	T	0.4177	2.49E-06	chr:6:32648036
6	rs9501648	32668886	G	0.4177	2.49E-06	chr:6:32648036
6	rs552525356	32453000	G	2.538	2.50E-06	chr:6:32648036
6	rs4988888	32635197	C	2.206	2.50E-06	chr:6:32648036
6	rs74973088	32457874	C	1.72	2.50E-06	chr:6:32648036
6	rs28383282	32586375	T	2.38	2.52E-06	chr:6:32648036
6	rs17205612	32637333	T	0.4264	2.52E-06	chr:6:32648036
6	rs74999184	32508306	C	0.4856	2.55E-06	chr:6:32648036
6	rs28724008	32550550	G	2.237	2.55E-06	chr:6:32648036
6	rs56435053	32623277	T	4.701	2.57E-06	chr:6:32648036
6	rs113923037	32457315	A	2.052	2.58E-06	chr:6:32648036
6	rs17881965	32551961	C	0.3629	2.59E-06	chr:6:32648036
6	rs17885388	32551962	T	0.3629	2.59E-06	chr:6:32648036
6	rs34958241	32559575	G	5.718	2.60E-06	chr:6:32648036
6	rs557617439	32523181	G	2.557	2.62E-06	chr:6:32648036
6	rs281874914	32631372	G	4.484	2.63E-06	chr:6:32648036
6	rs434841	32191041	A	0.4631	2.64E-06	chr:6:32648036
6	rs140539100	32227258	A	2.001	2.66E-06	chr:6:32648036
6	rs200269610	32227259	G	2.001	2.66E-06	chr:6:32648036
6	rs6908927	32224489	A	2.001	2.66E-06	chr:6:32648036
6	rs9268034	32227262	G	2.001	2.66E-06	chr:6:32648036
6	rs28746982	32653703	A	0.433	2.68E-06	chr:6:32648036
6	rs192994337	32511083	A	0.5551	2.69E-06	chr:6:32648036
6	rs9394113	32665167	G	0.4206	2.69E-06	chr:6:32648036
6	rs549954104	32554096	A	2.75	2.70E-06	chr:6:32648036
6	rs34784936	32559648	T	6.433	2.71E-06	chr:6:32648036
6	rs9274226	32631458	C	5.496	2.72E-06	chr:6:32648036
6	rs3115569	32224139	A	2.003	2.73E-06	chr:6:32648036
6	rs9394111	32658744	T	0.4218	2.73E-06	chr:6:32648036
6	rs549610120	32525308	G	0.5678	2.74E-06	chr:6:32648036
6	rs2071544	32806121	C	0.4945	2.76E-06	chr:6:32648036
6	rs9270523	32559584	T	0.4389	2.76E-06	chr:6:32648036
6	rs498422	32286761	G	0.4582	2.77E-06	chr:6:32648036
6	rs114651315	32601278	T	0.3904	2.78E-06	chr:6:32648036
6	rs72848251	32601288	A	0.3904	2.78E-06	chr:6:32648036

6	rs34400217	32637750	T	0.4173	2.78E-06	chr:6:32648036
6	rs77498463	32517198	T	0.4322	2.79E-06	chr:6:32648036
6	rs28891412	32640538	T	0.4314	2.79E-06	chr:6:32648036
6	rs28772303	32680558	A	0.4157	2.79E-06	chr:6:32648036
6	rs9268560	32389512	C	1.984	2.80E-06	chr:6:32648036
6	rs1018430	32281688	T	1.998	2.81E-06	chr:6:32648036
6	rs183972385	32497183	G	2.289	2.82E-06	chr:6:32648036
6	rs28891413	32640548	T	0.4412	2.83E-06	chr:6:32648036
6	rs200330306	32496048	A	1.754	2.84E-06	chr:6:32648036
6	rs557355024	32630464	T	1.733	2.86E-06	chr:6:32648036
6	rs114211044	32394840	G	4.627	2.87E-06	chr:6:32648036
6	rs141377480	32426020	A	4.627	2.87E-06	chr:6:32648036
6	rs3129876	32408012	A	2.083	2.90E-06	chr:6:32648036
6	rs17843708	32622396	A	1.734	2.90E-06	chr:6:32648036
6	rs2646993	32620489	T	1.757	2.92E-06	chr:6:32648036
6	rs2647062	32570417	C	5.921	2.93E-06	chr:6:32648036
6	rs17209950	32446588	A	2.211	2.94E-06	chr:6:32648036
6	rs3134969	32674027	C	0.3674	2.95E-06	chr:6:32648036
6	rs199980256	32540924	A	1.856	3.00E-06	chr:6:32648036
6	rs1049107	32629936	T	0.3932	3.00E-06	chr:6:32648036
6	rs9271763	32594061	G	1.983	3.01E-06	chr:6:32648036
6	rs9274450	32633334	A	0.5321	3.01E-06	chr:6:32648036
6	rs17212427	32652425	C	0.4191	3.03E-06	chr:6:32648036
6	rs34647096	32561681	A	6.662	3.03E-06	chr:6:32648036
6	rs28724151	32555378	T	2.919	3.04E-06	chr:6:32648036
6	rs35113548	32546299	A	2.815	3.05E-06	chr:6:32648036
6	rs28731358	32626883	C	0.515	3.05E-06	chr:6:32648036
6	rs281863375	32631113	C	0.4924	3.06E-06	chr:6:32648036
6	rs76566406	32518913	T	0.4437	3.07E-06	chr:6:32648036
6	rs34415150	32560477	G	6.686	3.07E-06	chr:6:32648036
6	rs34496598	32560455	T	6.686	3.07E-06	chr:6:32648036
6	rs34924558	32560446	T	6.686	3.07E-06	chr:6:32648036
6	rs113786400	32485794	T	1.967	3.09E-06	chr:6:32648036
6	rs9275357	32667891	C	1.984	3.10E-06	chr:6:32648036
7	rs67340855	158028782	C	79.16	3.35E-35	chr:7:158029482
7	rs67515424	158028692	T	79.16	3.35E-35	chr:7:158029482
7	rs67463190	158029292	T	32.9	7.38E-33	chr:7:158029482
7	rs55821220	158022462	G	17.51	2.06E-18	chr:7:158029482
7	rs73176110	158016190	T	33.17	2.72E-18	chr:7:158029482
7	rs73176115	158017202	C	33.17	2.72E-18	chr:7:158029482
7	rs73176116	158018041	T	33.17	2.72E-18	chr:7:158029482
7	rs73176117	158018530	A	33.17	2.72E-18	chr:7:158029482
7	rs73176118	158018536	A	33.17	2.72E-18	chr:7:158029482

7	rs73176123	158026203	G	37.73	4.88E-18	chr:7:158029482
7	rs73173702	157996475	G	52.83	5.56E-17	chr:7:158029482
7	rs73173694	157983465	A	12.46	1.26E-16	chr:7:158029482
7	rs73173701	157993472	C	8.654	1.47E-15	chr:7:158029482
7	rs571148131	158047093	A	8.709	2.83E-15	chr:7:158029482
7	rs73176109	158014524	G	8.071	5.72E-15	chr:7:158029482
7	rs55633973	158000392	A	7.314	3.01E-14	chr:7:158029482
7	rs12154635	158000947	G	6.885	1.02E-13	chr:7:158029482
7	rs73176108	158009978	A	6.545	2.18E-13	chr:7:158029482
7	rs56215385	158008987	A	6.372	3.70E-13	chr:7:158029482
7	rs10245306	158029340	G	3.53	5.62E-13	chr:7:158029482
7	rs114212832	158010048	A	15.99	2.67E-12	chr:7:158029482
7	rs114397225	158026708	T	15.99	2.67E-12	chr:7:158029482
7	rs116425675	158025376	G	15.99	2.67E-12	chr:7:158029482
7	rs146336615	158005141	T	15.99	2.67E-12	chr:7:158029482
7	rs148230980	158027778	G	15.99	2.67E-12	chr:7:158029482
7	rs149721773	158023785	A	15.99	2.67E-12	chr:7:158029482
7	rs185955717	158027592	C	15.99	2.67E-12	chr:7:158029482
7	rs75127652	158002441	C	15.99	2.67E-12	chr:7:158029482
7	rs78431013	158011436	G	15.99	2.67E-12	chr:7:158029482
7	rs79488857	158006433	G	15.99	2.67E-12	chr:7:158029482
7	rs76837643	157998303	T	27.36	2.80E-12	chr:7:158029482
7	rs114730739	158018607	C	5.454	4.13E-12	chr:7:158029482
7	rs114814237	158007454	T	32.72	6.40E-12	chr:7:158029482
7	rs114925757	158010221	C	32.72	6.40E-12	chr:7:158029482
7	rs148551986	158005447	C	32.72	6.40E-12	chr:7:158029482
7	rs78028556	158020232	A	32.72	6.40E-12	chr:7:158029482
7	rs78569708	158019350	A	32.72	6.40E-12	chr:7:158029482
7	rs116662371	157983459	A	15.5	6.54E-12	chr:7:158029482
7	rs6952594	157983739	G	15.5	6.54E-12	chr:7:158029482
7	rs6956043	157984240	T	15.5	6.54E-12	chr:7:158029482
7	rs114073477	157987166	A	13.91	9.55E-12	chr:7:158029482
7	rs147418646	157996189	A	13.91	9.55E-12	chr:7:158029482
7	rs147434829	157993831	T	13.91	9.55E-12	chr:7:158029482
7	rs6945026	157989420	A	13.91	9.55E-12	chr:7:158029482
7	rs6953725	157986343	C	13.91	9.55E-12	chr:7:158029482
7	rs6966499	157986139	A	13.91	9.55E-12	chr:7:158029482
7	rs6968048	157986390	T	13.91	9.55E-12	chr:7:158029482
7	rs6972865	157987793	A	13.91	9.55E-12	chr:7:158029482
7	rs6977406	157988246	A	13.91	9.55E-12	chr:7:158029482
7	rs75229314	157996659	T	13.91	9.55E-12	chr:7:158029482
7	rs77498718	157992348	A	13.91	9.55E-12	chr:7:158029482
7	rs79422776	157992820	A	13.91	9.55E-12	chr:7:158029482

7	rs79575378	157988653	A	13.91	9.55E-12	chr:7:158029482
7	rs191668503	157879438	C	12.54	1.20E-11	chr:7:158029482
7	rs115782456	157886102	A	15.51	1.44E-11	chr:7:158029482
7	rs146283516	157921007	G	15.51	1.44E-11	chr:7:158029482
7	rs180930685	157919353	T	15.51	1.44E-11	chr:7:158029482
7	rs185372713	157906466	T	15.51	1.44E-11	chr:7:158029482
7	rs536608866	157917813	T	15.51	1.44E-11	chr:7:158029482
7	rs554321308	157921838	T	15.51	1.44E-11	chr:7:158029482
7	rs554322023	157925176	T	15.51	1.44E-11	chr:7:158029482
7	rs74911888	157903474	A	15.51	1.44E-11	chr:7:158029482
7	rs76336600	157927761	A	15.51	1.44E-11	chr:7:158029482
7	rs76534082	157886828	A	15.51	1.44E-11	chr:7:158029482
7	rs78068415	157919982	T	15.51	1.44E-11	chr:7:158029482
7	rs78118820	157895991	A	15.51	1.44E-11	chr:7:158029482
7	rs6972212	157952535	A	21.57	1.49E-11	chr:7:158029482
7	rs74355306	157971701	G	21.57	1.49E-11	chr:7:158029482
7	rs79391313	157962842	A	21.57	1.49E-11	chr:7:158029482
7	rs74703160	157985626	A	12.33	1.72E-11	chr:7:158029482
7	rs73176112	158016362	G	3.344	1.96E-11	chr:7:158029482
7	rs76642817	157890996	T	13.85	2.33E-11	chr:7:158029482
7	rs75174369	157977058	T	12.3	3.10E-11	chr:7:158029482
7	rs78576374	157972674	T	12.3	3.10E-11	chr:7:158029482
7	rs115154220	157992431	A	10.83	3.45E-11	chr:7:158029482
7	rs116101671	158002764	G	39.88	3.53E-11	chr:7:158029482
7	rs145872346	158030555	C	39.88	3.53E-11	chr:7:158029482
7	rs74746824	158024336	C	39.88	3.53E-11	chr:7:158029482
7	rs114513220	157967575	T	14.26	3.78E-11	chr:7:158029482
7	rs114729797	157937056	A	14.26	3.78E-11	chr:7:158029482
7	rs116265641	157939554	A	14.26	3.78E-11	chr:7:158029482
7	rs116369472	157969228	A	14.26	3.78E-11	chr:7:158029482
7	rs138770773	157937061	A	14.26	3.78E-11	chr:7:158029482
7	rs6971835	157952257	C	14.26	3.78E-11	chr:7:158029482
7	rs74559595	157933765	T	14.26	3.78E-11	chr:7:158029482
7	rs75482412	157935378	T	14.26	3.78E-11	chr:7:158029482
7	rs78774372	157968332	A	11.95	4.69E-11	chr:7:158029482
7	rs17847433	157997673	A	4.778	4.87E-11	chr:7:158029482
7	rs114958462	157903797	T	14.1	6.25E-11	chr:7:158029482
7	rs55917540	157999549	C	4.646	9.25E-11	chr:7:158029482
7	rs6964342	158031807	G	3.349	9.41E-11	chr:7:158029482
7	rs115300653	157932042	T	10.68	1.09E-10	chr:7:158029482
7	rs10271837	157985998	C	0.2872	1.38E-10	chr:7:158029482
7	rs10268228	157985479	A	0.2896	1.70E-10	chr:7:158029482
7	rs10271711	157985854	A	0.2896	1.70E-10	chr:7:158029482

7	rs1806611	157986440	T	0.2955	2.20E-10	chr:7:158029482
7	rs80332916	157934646	A	9.959	2.70E-10	chr:7:158029482
7	rs3800856	157984992	A	0.2912	3.13E-10	chr:7:158029482
7	rs3800855	157985149	G	0.2918	3.35E-10	chr:7:158029482
7	rs10263846	157990941	T	0.2931	3.92E-10	chr:7:158029482
7	rs10275911	157990608	G	0.2931	3.92E-10	chr:7:158029482
7	rs10949702	157986125	C	0.2944	4.03E-10	chr:7:158029482
7	rs10249710	158030463	A	0.3018	4.60E-10	chr:7:158029482
7	rs77954122	157994745	G	6.971	5.91E-10	chr:7:158029482
7	rs10258997	158016559	G	0.2999	6.58E-10	chr:7:158029482
7	rs142201058	158020635	T	10.81	7.72E-10	chr:7:158029482
7	rs78090661	158020353	A	10.81	7.72E-10	chr:7:158029482
7	rs73512359	157906542	T	11.56	1.00E-09	chr:7:158029482
7	rs114392747	157994452	C	6.561	1.09E-09	chr:7:158029482
7	rs116046269	157994801	C	6.561	1.09E-09	chr:7:158029482
7	rs116368464	157995072	G	6.561	1.09E-09	chr:7:158029482
7	rs142912440	157994234	C	6.561	1.09E-09	chr:7:158029482
7	rs144548501	157993316	T	6.561	1.09E-09	chr:7:158029482
7	rs148965653	157994024	G	6.561	1.09E-09	chr:7:158029482
7	rs73520513	158033774	T	7.908	1.12E-09	chr:7:158029482
7	rs67598038	158028778	A	0.3104	1.18E-09	chr:7:158029482
7	rs59804289	158006512	G	4.123	1.76E-09	chr:7:158029482
7	rs150310484	158033865	A	15.31	2.02E-09	chr:7:158029482
7	rs6950346	158025658	T	0.311	3.36E-09	chr:7:158029482
7	rs10231253	157983353	A	0.3177	3.54E-09	chr:7:158029482
7	rs4909061	158103810	C	2.674	4.18E-09	chr:7:158029482
7	rs185047359	158032606	C	23.36	4.57E-09	chr:7:158029482
7	rs7789111	158021519	C	0.319	5.25E-09	chr:7:158029482
7	rs112569024	157944883	T	6.283	5.67E-09	chr:7:158029482
7	rs115431284	158014743	G	9.959	6.87E-09	chr:7:158029482
7	rs76699966	158008670	C	9.959	6.87E-09	chr:7:158029482
7	rs185271170	158047448	G	19.6	6.88E-09	chr:7:158029482
7	rs375602633	158040173	T	19.6	6.88E-09	chr:7:158029482
7	rs376264880	158041701	T	19.6	6.88E-09	chr:7:158029482
7	rs75391892	158044882	A	19.6	6.88E-09	chr:7:158029482
7	rs113800399	157954168	A	14.3	1.18E-08	chr:7:158029482
7	rs10227009	158027455	C	0.3346	1.42E-08	chr:7:158029482
7	rs58825421	158021882	A	5.52	1.49E-08	chr:7:158029482
7	rs116112670	157978049	G	6.008	1.65E-08	chr:7:158029482
7	rs192283162	157955561	T	12.28	2.00E-08	chr:7:158029482
7	rs59137399	158027849	T	5.331	2.12E-08	chr:7:158029482
7	rs114084009	158039743	T	40.03	2.47E-08	chr:7:158029482
7	rs114913762	158044106	A	40.03	2.47E-08	chr:7:158029482

7	rs147332266	158041045	A	40.03	2.47E-08	chr:7:158029482
7	rs74895395	158039244	T	40.03	2.47E-08	chr:7:158029482
7	rs116453618	158034494	A	8.108	2.56E-08	chr:7:158029482
7	rs57418975	158008236	T	5.179	2.84E-08	chr:7:158029482
7	rs73518440	158004550	T	5.179	2.84E-08	chr:7:158029482
7	rs114658547	158019418	T	66.26	3.78E-08	chr:7:158029482
7	rs139241072	158020035	G	66.26	3.78E-08	chr:7:158029482
7	rs139449578	158019174	T	66.26	3.78E-08	chr:7:158029482
7	rs147886434	158024207	T	66.26	3.78E-08	chr:7:158029482
7	rs75833406	158019784	A	66.26	3.78E-08	chr:7:158029482
7	rs79437632	158025064	G	66.26	3.78E-08	chr:7:158029482
7	rs139962222	157976021	A	15.85	4.60E-08	chr:7:158029482
7	rs369667396	157964187	T	15.62	5.34E-08	chr:7:158029482
7	rs59308052	158012401	T	4.544	5.35E-08	chr:7:158029482
7	rs138941200	158026684	G	63.77	5.56E-08	chr:7:158029482
7	rs4416779	157987251	T	2.55	7.12E-08	chr:7:158029482
7	rs185089183	157986725	T	62.08	7.88E-08	chr:7:158029482
7	rs546693958	157991425	C	62.08	7.88E-08	chr:7:158029482
7	rs78436390	157989783	A	62.08	7.88E-08	chr:7:158029482
7	rs115563191	158006928	G	61.26	8.54E-08	chr:7:158029482
7	rs115010930	157967313	A	14.93	1.16E-07	chr:7:158029482
7	rs137902222	157968097	A	14.93	1.16E-07	chr:7:158029482
7	rs181064733	157967576	A	14.93	1.16E-07	chr:7:158029482
7	rs184836979	157946720	A	14.93	1.16E-07	chr:7:158029482
7	rs368277902	157967275	G	14.93	1.16E-07	chr:7:158029482
7	rs369886862	157959528	T	14.93	1.16E-07	chr:7:158029482
7	rs77448247	157960029	A	14.93	1.16E-07	chr:7:158029482
7	rs79350926	157955419	T	14.93	1.16E-07	chr:7:158029482
7	rs10949707	158070635	C	3.226	1.26E-07	chr:7:158029482
7	rs4909062	158103844	A	2.377	1.51E-07	chr:7:158029482
7	rs12533146	158073096	A	3.077	1.53E-07	chr:7:158029482
7	rs13231012	158072540	A	3.136	1.62E-07	chr:7:158029482
7	rs114246372	157934539	C	12.56	1.69E-07	chr:7:158029482
7	rs150094352	157935774	A	12.56	1.69E-07	chr:7:158029482
7	rs186893056	157929280	G	12.56	1.69E-07	chr:7:158029482
7	rs182904034	157955513	A	12.39	1.87E-07	chr:7:158029482
7	rs13230783	158072330	A	3.111	1.88E-07	chr:7:158029482
7	rs73176114	158017101	G	3.043	2.05E-07	chr:7:158029482
7	rs115380396	157978423	A	4.4	2.20E-07	chr:7:158029482
7	rs79707592	158039220	T	7.325	2.50E-07	chr:7:158029482
7	rs146404159	157905389	T	19.17	3.96E-07	chr:7:158029482
7	rs148418754	157910241	C	19.17	3.96E-07	chr:7:158029482
7	rs78055072	157935566	T	3.937	4.13E-07	chr:7:158029482

7	rs115829202	157943365	T	7.176	4.29E-07	chr:7:158029482
7	rs569803815	158046905	T	4.674	4.70E-07	chr:7:158029482
7	rs192147010	157942364	T	26.35	4.93E-07	chr:7:158029482
7	rs114251824	157925408	A	11.68	4.96E-07	chr:7:158029482
7	rs17847428	157926349	G	0.4331	5.16E-07	chr:7:158029482
7	rs60639062	157982851	T	18.36	5.99E-07	chr:7:158029482
7	rs34664051	158069257	A	2.945	6.11E-07	chr:7:158029482
7	rs141625384	157966763	G	7.224	6.25E-07	chr:7:158029482
7	rs147187810	157952409	T	8.104	6.81E-07	chr:7:158029482
7	rs9691217	158044083	C	6.838	8.05E-07	chr:7:158029482
7	rs4909162	158058663	T	13.68	9.46E-07	chr:7:158029482
7	rs143899134	157960718	C	39.95	1.39E-06	chr:7:158029482
7	rs10247985	158017478	C	0.4211	1.39E-06	chr:7:158029482
7	rs111360395	158046892	C	4.74	1.50E-06	chr:7:158029482
7	rs76583176	157969959	A	23.39	1.55E-06	chr:7:158029482
7	rs150519493	158096747	C	27.95	1.58E-06	chr:7:158029482
7	rs112290031	157914431	A	10.74	1.59E-06	chr:7:158029482
7	rs148636805	157960723	C	6.84	1.64E-06	chr:7:158029482
7	rs149077451	157958690	T	6.84	1.64E-06	chr:7:158029482
7	rs73176107	158004804	G	2.749	1.73E-06	chr:7:158029482
7	rs114057722	157932924	A	7.567	1.91E-06	chr:7:158029482
7	rs116058122	157935823	A	7.567	1.91E-06	chr:7:158029482
7	rs141237994	157934588	A	7.567	1.91E-06	chr:7:158029482
7	rs369805479	157900543	T	8.463	2.02E-06	chr:7:158029482
7	rs113100148	158046896	G	3.795	2.08E-06	chr:7:158029482
7	rs7784655	157880105	C	2.138	2.43E-06	chr:7:158029482
7	rs191914941	157939369	T	6.625	2.88E-06	chr:7:158029482
7	rs531614647	157917975	T	8.586	3.11E-06	chr:7:158029482
7	rs116828492	158023888	C	129.7	3.47E-06	chr:7:158029482
7	rs141144502	158022984	T	129.7	3.47E-06	chr:7:158029482
7	rs183214705	158002555	G	129.7	3.47E-06	chr:7:158029482
7	rs190367380	157996656	T	129.7	3.47E-06	chr:7:158029482
7	rs547208450	157999698	A	129.7	3.47E-06	chr:7:158029482
7	rs1982450	158074146	T	2.52	4.19E-06	chr:7:158029482
7	rs6963124	158023589	T	0.3921	4.65E-06	chr:7:158029482
7	rs111618217	157946585	C	0.3495	4.79E-06	chr:7:158029482
7	rs10215745	158015262	T	2.07	4.83E-06	chr:7:158029482
7	rs2335834;rs528512480	157967182	G	3.095	5.07E-06	chr:7:158029482
7	rs111852013	157973020	T	2.107	5.16E-06	chr:7:158029482
7	rs12698190	158051753	T	2.135	5.25E-06	chr:7:158029482
7	rs546309641	157919286	A	7.034	5.36E-06	chr:7:158029482
7	rs77030049	157970376	T	2.109	5.47E-06	chr:7:158029482
7	rs535124812	158119083	G	24.05	5.52E-06	chr:7:158029482

7	rs10215991	158015263	G	2.057	5.72E-06	chr:7:158029482
7	rs10215992	158015273	G	2.057	5.72E-06	chr:7:158029482
7	rs77439951	157850032	A	5.897	6.08E-06	chr:7:158029482
7	rs56211164	158016764	A	2.084	6.64E-06	chr:7:158029482
7	rs10257230	158015030	T	2.041	7.48E-06	chr:7:158029482
7	rs10215289	158015301	C	2.032	8.60E-06	chr:7:158029482
7	rs140744744	157861117	C	6.347	8.83E-06	chr:7:158029482
7	rs142686554	157850685	C	6.347	8.83E-06	chr:7:158029482
7	rs10238207	157870788	C	2.715	1.14E-05	chr:7:158029482
7	rs146059562	157919343	G	0.4835	1.15E-05	chr:7:158029482
7	rs140405611	157911297	G	6.012	1.21E-05	chr:7:158029482
7	rs141581691	157913360	C	6.012	1.21E-05	chr:7:158029482
7	rs73173689	157981830	A	2.484	1.31E-05	chr:7:158029482
7	rs13234832	158014979	C	2.011	1.43E-05	chr:7:158029482
7	rs140231094	158056865	A	105.5	1.55E-05	chr:7:158029482
7	rs538008579	158061457	A	105.5	1.55E-05	chr:7:158029482
7	rs145200244	157931404	G	4.881	1.59E-05	chr:7:158029482
7	rs150955375	158010507	C	100.1	1.60E-05	chr:7:158029482
7	rs28501236	157913697	C	2.915	1.98E-05	chr:7:158029482
7	rs3752372	158031556	C	0.4876	2.01E-05	chr:7:158029482
7	rs138665655	157889846	A	5.551	2.16E-05	chr:7:158029482
7	rs11763127	157931893	C	2.662	2.27E-05	chr:7:158029482
7	rs10228083	157912213	T	2.625	2.29E-05	chr:7:158029482
7	rs3800860	157903504	T	2.625	2.29E-05	chr:7:158029482
7	rs10277112	157916913	A	2.685	2.38E-05	chr:7:158029482
7	rs553467466	157568780	T	3.983	2.41E-05	chr:7:158029482
7	rs10262127	158016574	T	0.3996	2.46E-05	chr:7:158029482
7	rs138523799	158793571	A	102.3	1.12E-13	chr:7:158773385
7	rs148191983	158794102	T	102.3	1.12E-13	chr:7:158773385
7	rs142970495	158771425	G	176.8	3.67E-13	chr:7:158773385
7	rs115752605	158807960	T	106.9	1.00E-12	chr:7:158773385
7	rs138320299	158808687	C	106.9	1.00E-12	chr:7:158773385
7	rs138720434	158812703	C	106.9	1.00E-12	chr:7:158773385
7	rs139925274	158799701	T	106.9	1.00E-12	chr:7:158773385
7	rs143423400	158775838	T	244.5	1.06E-12	chr:7:158773385
7	rs145602338	158785863	T	244.5	1.06E-12	chr:7:158773385
7	rs149504192	158779093	A	244.5	1.06E-12	chr:7:158773385
7	rs149754911	158785737	A	244.5	1.06E-12	chr:7:158773385
7	rs189570458	158776040	T	244.5	1.06E-12	chr:7:158773385
7	rs535727050	158782451	G	244.5	1.06E-12	chr:7:158773385
7	rs555673551	158782453	C	244.5	1.06E-12	chr:7:158773385
7	rs139015389	158813767	A	67.48	1.45E-12	chr:7:158773385
7	rs180729474	158766864	T	38.03	3.24E-12	chr:7:158773385

7	rs115436222	158819451	A	298.8	3.70E-11	chr:7:158773385
7	rs116064411	158807651	C	298.8	3.70E-11	chr:7:158773385
7	rs139689953	158819670	C	298.8	3.70E-11	chr:7:158773385
7	rs141350254	158857415	T	48.11	5.44E-10	chr:7:158773385
7	rs114801328	158695613	A	23.45	2.20E-09	chr:7:158773385
7	rs149349671	158682570	T	23.45	2.20E-09	chr:7:158773385
7	rs558399897	158685625	G	23.45	2.20E-09	chr:7:158773385
7	rs139692502	158766761	C	13.49	2.37E-09	chr:7:158773385
7	rs140852796	158656731	T	40.84	9.99E-09	chr:7:158773385
7	rs151115445	158710907	G	48.95	1.03E-08	chr:7:158773385
7	rs147534657	158751295	G	67.32	1.11E-08	chr:7:158773385
7	rs142012004	158670477	G	84.78	1.14E-08	chr:7:158773385
7	rs139436013	158897519	C	17.94	1.23E-08	chr:7:158773385
7	rs190931491	158897523	T	17.94	1.23E-08	chr:7:158773385
7	rs144174529	158752191	G	37.22	1.51E-08	chr:7:158773385
7	rs115496394	158645732	C	10.75	3.75E-08	chr:7:158773385
7	rs116285782	158645630	T	10.75	3.75E-08	chr:7:158773385
7	rs114910333	158657200	C	13.82	4.32E-08	chr:7:158773385
7	rs140088911	158655377	A	13.82	4.32E-08	chr:7:158773385
7	rs76689482	158654079	T	13.4	5.15E-08	chr:7:158773385
7	rs140244843	158782360	T	244.9	5.36E-08	chr:7:158773385
7	rs140596100	158677277	T	66.76	8.06E-08	chr:7:158773385
7	rs138414941	158756362	A	75.82	1.05E-07	chr:7:158773385
7	rs149141966	158785912	C	75.82	1.05E-07	chr:7:158773385
7	rs568688013	158789844	C	75.82	1.05E-07	chr:7:158773385
7	rs115563954	158741968	G	17.28	1.48E-07	chr:7:158773385
7	rs148690898	158757302	G	31.04	1.65E-07	chr:7:158773385
7	rs576890241	158671184	C	117.7	1.73E-07	chr:7:158773385
7	rs2730234	158782584	A	6.602	2.14E-07	chr:7:158773385
7	rs140376692	158750624	T	18.4	2.18E-07	chr:7:158773385
7	rs114018982	158723083	C	16.16	2.36E-07	chr:7:158773385
7	rs114093570	158662154	T	15.81	2.63E-07	chr:7:158773385
7	rs114305528	158695812	C	15.81	2.63E-07	chr:7:158773385
7	rs115035685	158662412	A	15.81	2.63E-07	chr:7:158773385
7	rs116479089	158658864	A	15.81	2.63E-07	chr:7:158773385
7	rs116625827	158662642	A	15.81	2.63E-07	chr:7:158773385
7	rs116627160	158657594	G	15.81	2.63E-07	chr:7:158773385
7	rs138339913	158666510	T	15.81	2.63E-07	chr:7:158773385
7	rs151236919	158696868	G	15.81	2.63E-07	chr:7:158773385
7	rs59277300	158686315	G	15.81	2.63E-07	chr:7:158773385
7	rs61212889	158665399	G	15.81	2.63E-07	chr:7:158773385
7	rs6948415	158763881	G	6.017	3.13E-07	chr:7:158773385
7	rs2657407	158782543	T	6.363	3.24E-07	chr:7:158773385

7	rs114650465	158755171	C	10.95	4.15E-07	chr:7:158773385
7	rs2247274	158765682	G	6.527	4.16E-07	chr:7:158773385
7	rs142443822	158704746	G	14.01	4.47E-07	chr:7:158773385
7	rs138066846	158676646	T	8.976	5.76E-07	chr:7:158773385
7	rs147478471	158836620	A	38.92	1.00E-06	chr:7:158773385
7	rs370299428	158831322	T	38.92	1.00E-06	chr:7:158773385
7	rs150871010	158689749	A	9.68	2.00E-06	chr:7:158773385
7	rs114154718	158675517	G	8.144	2.54E-06	chr:7:158773385
7	rs6459916	158770117	A	5.618	2.54E-06	chr:7:158773385
7	rs116277468	158673748	C	8.078	2.88E-06	chr:7:158773385
7	rs116397049	158701546	C	7.872	3.58E-06	chr:7:158773385
7	rs114580586	158701147	G	7.82	3.89E-06	chr:7:158773385
7	rs262136	158764915	T	5.429	4.59E-06	chr:7:158773385
7	rs10259521	158740307	T	9.714	5.37E-06	chr:7:158773385
7	rs2657399	158784591	C	5.846	5.83E-06	chr:7:158773385
7	rs114475809	158716221	C	9.465	6.61E-06	chr:7:158773385
7	rs115436921	158716224	C	9.465	6.61E-06	chr:7:158773385
7	rs116339161	158710422	T	9.465	6.61E-06	chr:7:158773385
7	rs10256002	158739841	T	9.333	7.37E-06	chr:7:158773385
7	rs10276354	158737815	T	9.333	7.37E-06	chr:7:158773385
7	rs28770291	158733181	A	9.333	7.37E-06	chr:7:158773385
7	rs565059270	158775061	T	179.1	7.79E-06	chr:7:158773385
7	rs116206273	158895513	T	7.843	8.76E-06	chr:7:158773385
7	rs10247405	158790004	G	6.036	9.68E-06	chr:7:158773385
7	rs147867742	158671306	A	6.679	1.95E-05	chr:7:158773385
7	rs115022047	158881022	A	7.954	2.11E-05	chr:7:158773385
7	rs143508100	158864823	C	6.884	2.22E-05	chr:7:158773385
7	rs7791538	158855367	G	6.219	2.25E-05	chr:7:158773385
7	rs565435826	158527283	T	107.5	2.27E-05	chr:7:158773385
7	rs115551956	158869490	T	7.754	2.52E-05	chr:7:158773385
7	rs116529881	158847310	C	7.672	2.55E-05	chr:7:158773385
7	rs532960886	158882893	T	7.063	2.60E-05	chr:7:158773385
7	rs114653951	158845976	A	7.618	2.65E-05	chr:7:158773385
7	rs113277054	158775082	T	52.97	2.88E-05	chr:7:158773385
7	rs28854817	158688729	G	5.944	2.92E-05	chr:7:158773385
7	rs114465751	158902783	A	7.579	2.92E-05	chr:7:158773385
7	rs116546283	158906836	C	7.579	2.92E-05	chr:7:158773385
7	rs561469257	158893794	A	7.579	2.92E-05	chr:7:158773385
7	rs115435859	158850790	A	7.4	3.49E-05	chr:7:158773385
7	rs115332713	158861394	T	7.401	3.52E-05	chr:7:158773385
7	rs140553521	158864478	T	7.401	3.52E-05	chr:7:158773385
7	rs567437775	158779892	C	81.65	3.53E-05	chr:7:158773385
7	rs115459879	158890347	A	7.316	3.92E-05	chr:7:158773385

7	rs78569357	16241435	C	0.1767	2.85E-11	chr:7:16237347
7	rs80148995	16240992	A	0.1767	2.85E-11	chr:7:16237347
7	rs114237814	16203354	A	0.1964	1.49E-10	chr:7:16237347
7	rs139906888	16202233	G	0.1995	2.00E-10	chr:7:16237347
7	rs149192706	16200558	C	0.1995	2.00E-10	chr:7:16237347
7	rs17169288	16193288	A	0.1995	2.00E-10	chr:7:16237347
7	rs17169290	16194207	G	0.1995	2.00E-10	chr:7:16237347
7	rs17169295	16206239	A	0.1995	2.00E-10	chr:7:16237347
7	rs59124001	16191337	C	0.1995	2.00E-10	chr:7:16237347
7	rs59951504	16201410	A	0.1995	2.00E-10	chr:7:16237347
7	rs60412407	16189893	G	0.1995	2.00E-10	chr:7:16237347
7	rs74666326	16188224	G	0.1995	2.00E-10	chr:7:16237347
7	rs75093879	16200177	A	0.1995	2.00E-10	chr:7:16237347
7	rs75798894	16204714	A	0.1995	2.00E-10	chr:7:16237347
7	rs76635530	16220088	A	0.1983	2.85E-10	chr:7:16237347
7	rs17169327	16231028	C	0.2148	3.49E-10	chr:7:16237347
7	rs59510115	16150162	A	0.2052	4.33E-10	chr:7:16237347
7	rs7787953	16160986	C	0.2052	4.33E-10	chr:7:16237347
7	rs79608769	16159898	A	0.2052	4.33E-10	chr:7:16237347
7	rs59618653	16186627	T	0.2064	4.63E-10	chr:7:16237347
7	rs60082144	16184639	G	0.2064	4.63E-10	chr:7:16237347
7	rs75844841	16184554	C	0.2064	4.63E-10	chr:7:16237347
7	rs57213749	16147064	C	0.2238	5.43E-10	chr:7:16237347
7	rs77332294	16145345	A	0.2238	5.43E-10	chr:7:16237347
7	rs143528089	16142507	A	0.2076	5.44E-10	chr:7:16237347
7	rs59529774	16141140	G	0.2076	5.44E-10	chr:7:16237347
7	rs60525181	16148477	A	0.2076	5.44E-10	chr:7:16237347
7	rs61059482	16147246	C	0.2076	5.44E-10	chr:7:16237347
7	rs75872145	16169991	A	0.2095	5.90E-10	chr:7:16237347
7	rs7809306	16225174	A	0.252	8.36E-10	chr:7:16237347
7	rs76015838	16128754	C	0.2084	9.75E-10	chr:7:16237347
7	rs79391915	16138889	A	0.2084	9.75E-10	chr:7:16237347
7	rs79430054	16137524	A	0.2084	9.75E-10	chr:7:16237347
7	rs116237660	16180993	A	0.2115	1.03E-09	chr:7:16237347
7	rs10487914	16219670	G	0.2803	1.93E-09	chr:7:16237347
7	rs113407083	16199382	G	0.2803	1.93E-09	chr:7:16237347
7	rs17169286	16182262	T	0.2188	2.35E-09	chr:7:16237347
7	rs73304822	16216110	T	0.2829	2.47E-09	chr:7:16237347
7	rs114368585	16313273	A	0.1806	4.98E-09	chr:7:16237347
7	rs111701134	16292180	A	0.1912	1.15E-08	chr:7:16237347
7	rs111940941	16289931	G	0.1912	1.15E-08	chr:7:16237347
7	rs112143154	16293361	T	0.1912	1.15E-08	chr:7:16237347
7	rs112414690	16307612	T	0.1912	1.15E-08	chr:7:16237347

7	rs146152412	16290109	A	0.1912	1.15E-08	chr:7:16237347
7	rs78681820	16304439	G	0.1912	1.15E-08	chr:7:16237347
7	rs79555541	16301626	A	0.1912	1.15E-08	chr:7:16237347
7	rs79796679	16301634	C	0.1912	1.15E-08	chr:7:16237347
7	rs1034960	16212032	T	0.2624	1.16E-08	chr:7:16237347
7	rs115298402	16308143	A	0.1886	1.40E-08	chr:7:16237347
7	rs116677605	16312795	G	0.1886	1.40E-08	chr:7:16237347
7	rs75096853	16108857	G	0.182	1.45E-08	chr:7:16237347
7	rs79939484	16099542	A	0.182	1.45E-08	chr:7:16237347
7	rs77137406	16251332	C	0.1822	1.50E-08	chr:7:16237347
7	rs62440440	16294388	A	0.1942	2.04E-08	chr:7:16237347
7	rs140036716	16313848	C	0.1969	2.52E-08	chr:7:16237347
7	rs75499884	16072209	G	0.1813	3.79E-08	chr:7:16237347
7	rs12670694	16233741	G	0.3153	5.38E-08	chr:7:16237347
7	rs75658037	16065241	G	0.1665	5.58E-08	chr:7:16237347
7	rs115011307	16286071	C	0.1961	6.05E-08	chr:7:16237347
7	rs141283607	16290730	G	0.1961	6.05E-08	chr:7:16237347
7	rs148517152	16296005	G	0.1961	6.05E-08	chr:7:16237347
7	rs150186974	16291439	G	0.1961	6.05E-08	chr:7:16237347
7	rs77398067	16296632	G	0.1961	6.05E-08	chr:7:16237347
7	rs6963052	16211529	T	0.3708	7.36E-08	chr:7:16237347
7	rs12699772	16226615	T	0.322	7.64E-08	chr:7:16237347
7	rs35978678	16232776	T	0.3164	7.90E-08	chr:7:16237347
7	rs201756228	16128987	G	0.324	1.40E-07	chr:7:16237347
7	rs13237999	16126709	T	0.2403	1.41E-07	chr:7:16237347
7	rs199541295	16128992	T	0.3177	1.64E-07	chr:7:16237347
7	rs200989147	16128994	C	0.3177	1.64E-07	chr:7:16237347
7	rs201774908	16128995	A	0.3177	1.64E-07	chr:7:16237347
7	rs76570626	16158229	C	0.2036	2.07E-07	chr:7:16237347
7	rs537249407	16171775	T	0.3566	2.12E-07	chr:7:16237347
7	rs558512300	16171776	G	0.3566	2.12E-07	chr:7:16237347
7	rs569856115	16171774	C	0.3566	2.12E-07	chr:7:16237347
7	rs79447373	16147444	C	0.2085	2.77E-07	chr:7:16237347
7	rs11760631	16159780	T	0.4014	3.26E-07	chr:7:16237347
7	rs11760717	16159827	T	0.4014	3.26E-07	chr:7:16237347
7	rs77772901	16285138	T	0.2189	3.42E-07	chr:7:16237347
7	rs1527205	16160617	G	0.4033	3.72E-07	chr:7:16237347
7	rs78057051	16220568	A	0.204	3.78E-07	chr:7:16237347
7	rs115842586	16219510	T	0.2092	5.20E-07	chr:7:16237347
7	rs140445980	16293927	A	0.2198	7.99E-07	chr:7:16237347
7	rs76453672	16314532	G	0.2198	7.99E-07	chr:7:16237347
7	rs6976322	16231830	A	0.3955	8.89E-07	chr:7:16237347
7	rs4721478	16227094	A	0.4017	1.17E-06	chr:7:16237347

7	rs10950602	16136846	C	0.4032	1.30E-06	chr:7:16237347
7	rs17355113	16073067	G	0.3173	1.62E-06	chr:7:16237347
7	rs12216568	16261975	T	0.3491	1.93E-06	chr:7:16237347
7	rs12699773	16268937	C	0.3491	1.93E-06	chr:7:16237347
7	rs1589987	16267776	C	0.3491	1.93E-06	chr:7:16237347
7	rs7790551	16270388	A	0.3491	1.93E-06	chr:7:16237347
7	rs7807183	16267316	G	0.3491	1.93E-06	chr:7:16237347
7	rs78667415	15958939	T	0.2355	2.70E-06	chr:7:16237347
7	rs74350728	16272186	C	0.3379	3.38E-06	chr:7:16237347
7	rs78114589	16271386	G	0.3379	3.38E-06	chr:7:16237347
7	rs78251392	16264027	G	0.3379	3.38E-06	chr:7:16237347
7	rs79531512	16269690	C	0.3379	3.38E-06	chr:7:16237347
7	rs529813497	15917542	C	0.2597	3.97E-06	chr:7:16237347
7	rs1528145	16062820	A	0.3249	4.25E-06	chr:7:16237347
7	rs78947322	15895364	C	0.2352	4.29E-06	chr:7:16237347
7	rs79140626	16282404	A	0.34	4.40E-06	chr:7:16237347
7	rs78334563	15893721	C	0.2439	4.43E-06	chr:7:16237347
7	rs111449414	16278514	A	0.3411	4.56E-06	chr:7:16237347
7	rs74901096	16262857	C	0.3411	4.56E-06	chr:7:16237347
7	rs75942460	16274783	C	0.3411	4.56E-06	chr:7:16237347
7	rs78374272	16255641	C	0.3411	4.56E-06	chr:7:16237347
7	rs79096699	16275120	C	0.3411	4.56E-06	chr:7:16237347
7	rs80024336	16280063	C	0.228	4.56E-06	chr:7:16237347
7	rs28602776	16398883	C	0.3055	4.70E-06	chr:7:16237347
7	rs13240803	16227939	C	0.45	4.93E-06	chr:7:16237347
7	rs77256425	15881557	G	0.2469	5.15E-06	chr:7:16237347
7	rs74974254	16304404	C	0.1799	5.25E-06	chr:7:16237347
7	rs78294159	16275741	G	0.3441	5.51E-06	chr:7:16237347
7	rs553363007	16256767	T	0.3384	5.91E-06	chr:7:16237347
7	rs6461230	16242151	T	0.4548	9.83E-06	chr:7:16237347
7	rs12673171	16387848	C	0.3777	1.05E-05	chr:7:16237347
7	rs6461229	16238691	C	0.4579	1.09E-05	chr:7:16237347
7	rs17286602	16152174	A	0.4409	1.18E-05	chr:7:16237347
7	rs1918854	16073545	C	0.358	1.36E-05	chr:7:16237347
7	rs2389578	16066289	T	0.3492	1.41E-05	chr:7:16237347
7	rs7459076	16066542	T	0.3492	1.41E-05	chr:7:16237347
7	rs1528164	16077871	G	0.3503	2.51E-05	chr:7:16237347
7	rs35331274	16077251	C	0.3503	2.51E-05	chr:7:16237347
7	rs12699784	16396248	C	0.3964	2.70E-05	chr:7:16237347
7	rs17169439	16394128	A	0.3082	2.79E-05	chr:7:16237347
7	rs6461239	16371533	T	0.3082	2.79E-05	chr:7:16237347
7	rs6946720	16377435	T	0.3082	2.79E-05	chr:7:16237347
7	rs77301988	16387646	G	0.3059	3.58E-05	chr:7:16237347

7	rs4236287	16393464	C	0.4031	3.66E-05	chr:7:16237347
8	rs2129702	18656090	G	17.92	5.50E-37	chr:8:18651457
8	rs28696248	18656314	G	52.48	4.66E-33	chr:8:18651457
8	rs13261634	18653020	T	121.9	6.50E-32	chr:8:18651457
8	rs2632852	18656224	T	13.25	7.93E-32	chr:8:18651457
8	rs335256	18650834	C	8.888	2.17E-30	chr:8:18651457
8	rs10111304	18650904	C	0.05162	1.10E-29	chr:8:18651457
8	rs13254153	18655330	C	9.798	2.56E-29	chr:8:18651457
8	rs17467874	18652744	C	0.1002	3.75E-29	chr:8:18651457
8	rs11203998	18654206	C	157.1	1.63E-28	chr:8:18651457
8	rs10503635	18654329	T	97.48	2.28E-28	chr:8:18651457
8	rs11996790	18656238	C	0.1264	3.96E-28	chr:8:18651457
8	rs12549858	18654478	A	0.1142	5.54E-28	chr:8:18651457
8	rs60746929	18654480	T	0.1183	6.60E-28	chr:8:18651457
8	rs11777761	18652934	T	0.01971	1.51E-27	chr:8:18651457
8	rs60508084	18652637	T	0.01971	1.51E-27	chr:8:18651457
8	rs10503633	18653742	A	0.02702	9.61E-27	chr:8:18651457
8	rs10503634	18654293	T	0.01271	1.01E-26	chr:8:18651457
8	rs10094591	18653299	C	0.02297	2.48E-26	chr:8:18651457
8	rs11785249	18653313	A	0.02297	2.48E-26	chr:8:18651457
8	rs13250313	18652812	A	9.313	1.28E-25	chr:8:18651457
8	rs11780872	18651376	T	248.6	4.16E-24	chr:8:18651457
8	rs11780185	18651428	A	248.1	4.35E-24	chr:8:18651457
8	rs57290482	18652460	T	248.1	4.35E-24	chr:8:18651457
8	rs57931874	18652499	C	248.1	4.35E-24	chr:8:18651457
8	rs73199983	18628075	C	9.282	1.79E-21	chr:8:18651457
8	rs335234	18637444	C	4.387	3.37E-21	chr:8:18651457
8	rs335238	18642319	G	7.631	2.45E-20	chr:8:18651457
8	rs7824662	18635135	C	0.2182	6.87E-19	chr:8:18651457
8	rs6586764	18632093	T	0.2611	1.97E-17	chr:8:18651457
8	rs6586763	18632062	T	0.2692	4.59E-17	chr:8:18651457
8	rs11775676	18665492	C	0.2428	8.67E-17	chr:8:18651457
8	rs11774752	18664654	C	0.2451	1.23E-16	chr:8:18651457
8	rs12680447	18654653	T	3.649	3.23E-16	chr:8:18651457
8	rs335244	18645147	A	0.3201	2.06E-15	chr:8:18651457
8	rs2632845	18660720	C	0.3046	2.56E-15	chr:8:18651457
8	rs2632848	18659780	T	0.3135	9.36E-15	chr:8:18651457
8	rs79153013	18632687	T	0.2705	1.63E-14	chr:8:18651457
8	rs335232	18636126	A	0.3419	2.09E-14	chr:8:18651457
8	rs4921961	18638070	T	6.049	2.57E-14	chr:8:18651457
8	rs11775742	18665710	C	0.287	2.82E-14	chr:8:18651457
8	rs4921958	18634096	A	6.046	2.99E-14	chr:8:18651457
8	rs11786921	18657985	C	0.2784	3.36E-14	chr:8:18651457

8	rs11786923	18658007	G	0.2784	3.36E-14	chr:8:18651457
8	rs11986109	18657632	C	0.2784	3.36E-14	chr:8:18651457
8	rs11993401	18657427	T	0.2784	3.36E-14	chr:8:18651457
8	rs11993467	18657661	T	0.2784	3.36E-14	chr:8:18651457
8	rs11997879	18657450	A	0.2784	3.36E-14	chr:8:18651457
8	rs13276530	18658346	G	0.2784	3.36E-14	chr:8:18651457
8	rs34059664	18657698	G	0.2784	3.36E-14	chr:8:18651457
8	rs11774165	18658950	G	0.275	3.52E-14	chr:8:18651457
8	rs73595755	18661545	T	0.2728	4.80E-14	chr:8:18651457
8	rs73595757	18661575	A	0.2728	4.80E-14	chr:8:18651457
8	rs4341203	18646154	C	2.95	8.10E-14	chr:8:18651457
8	rs36085278	18659470	G	0.2943	1.13E-13	chr:8:18651457
8	rs28656462	18606367	T	3.105	1.90E-13	chr:8:18651457
8	rs73666715	18639853	C	5.186	1.96E-13	chr:8:18651457
8	rs56062960	18637485	C	5.178	2.15E-13	chr:8:18651457
8	rs10108047	18650597	T	10.88	2.18E-13	chr:8:18651457
8	rs28791302	18629428	T	3.545	2.23E-13	chr:8:18651457
8	rs10086769	18664962	C	7.006	2.91E-13	chr:8:18651457
8	rs3988322	18614526	A	0.3296	3.13E-13	chr:8:18651457
8	rs74614121	18666968	G	0.2725	3.46E-13	chr:8:18651457
8	rs12676642	18637208	C	4.59	4.15E-13	chr:8:18651457
8	rs7836892	18635256	C	4.579	4.23E-13	chr:8:18651457
8	rs73199955	18607756	C	3.059	5.14E-13	chr:8:18651457
8	rs112738876	18633882	A	4.524	5.69E-13	chr:8:18651457
8	rs61255116	18663146	G	0.2772	7.30E-13	chr:8:18651457
8	rs67028998	18628162	A	3.274	7.52E-13	chr:8:18651457
8	rs10102692	18665202	C	6.774	7.85E-13	chr:8:18651457
8	rs79441119	18632939	G	5.03	8.97E-13	chr:8:18651457
8	rs12335185	18623326	G	0.3599	9.19E-13	chr:8:18651457
8	rs58325516	18662764	T	0.2789	9.97E-13	chr:8:18651457
8	rs2638625	18659269	C	0.3593	1.07E-12	chr:8:18651457
8	rs73595748	18658096	C	0.2838	1.56E-12	chr:8:18651457
8	rs73595749	18658207	C	0.2838	1.56E-12	chr:8:18651457
8	rs7831166	18639675	T	8.521	1.64E-12	chr:8:18651457
8	rs12682382	18637762	G	8.511	1.66E-12	chr:8:18651457
8	rs73595731	18647294	T	0.2512	1.74E-12	chr:8:18651457
8	rs11993322	18657165	G	0.2857	2.09E-12	chr:8:18651457
8	rs111241636	18623207	C	8.004	2.50E-12	chr:8:18651457
8	rs1386692	18658750	A	0.368	2.75E-12	chr:8:18651457
8	rs335248	18646733	C	0.3818	3.19E-12	chr:8:18651457
8	rs57557661	18637442	C	4.094	3.44E-12	chr:8:18651457
8	rs4921959	18635578	G	4.086	3.44E-12	chr:8:18651457
8	rs7830088	18636444	C	4.086	3.44E-12	chr:8:18651457

8	rs12677280	18638660	C	4.048	4.25E-12	chr:8:18651457
8	rs10100475	18642757	G	4.581	5.16E-12	chr:8:18651457
8	rs73666724	18662204	A	7.47	8.85E-12	chr:8:18651457
8	rs73595733	18651305	G	0.2715	1.54E-11	chr:8:18651457
8	rs13439022	18636649	C	3.717	2.79E-11	chr:8:18651457
8	rs10093710	18611809	G	2.582	3.03E-11	chr:8:18651457
8	rs28502684	18608723	A	2.594	3.33E-11	chr:8:18651457
8	rs9792382	18620570	T	0.3933	3.80E-11	chr:8:18651457
8	rs335231	18629355	C	0.401	3.89E-11	chr:8:18651457
8	rs35764685	18661342	A	6.042	4.02E-11	chr:8:18651457
8	rs6994455	18618764	G	2.573	4.38E-11	chr:8:18651457
8	rs73595770	18665183	C	0.321	4.75E-11	chr:8:18651457
8	rs151157085	18608740	C	2.573	4.84E-11	chr:8:18651457
8	rs73595741	18654254	C	0.2835	5.15E-11	chr:8:18651457
8	rs335237	18641291	C	0.4096	6.00E-11	chr:8:18651457
8	rs2638650	18661521	T	0.3951	6.89E-11	chr:8:18651457
8	rs111514843	18608851	A	2.543	6.90E-11	chr:8:18651457
8	rs13255143	18661844	G	5.238	7.27E-11	chr:8:18651457
8	rs4921960	18635804	A	2.478	8.27E-11	chr:8:18651457
8	rs6987329	18616376	T	0.4017	9.03E-11	chr:8:18651457
8	rs7001013	18615974	C	0.4017	9.03E-11	chr:8:18651457
8	rs6998281	18618557	T	0.4014	9.10E-11	chr:8:18651457
8	rs7012496	18618427	C	0.4014	9.10E-11	chr:8:18651457
8	rs7012651	18618539	C	0.4014	9.10E-11	chr:8:18651457
8	rs1487745	18621332	C	0.4056	1.10E-10	chr:8:18651457
8	rs2638653	18666210	A	0.4059	1.20E-10	chr:8:18651457
8	rs78292221	18621253	A	0.255	1.35E-10	chr:8:18651457
8	rs7012633	18618503	C	0.4104	1.39E-10	chr:8:18651457
8	rs4921611	18617581	T	0.4076	1.53E-10	chr:8:18651457
8	rs6996801	18615696	G	2.483	1.86E-10	chr:8:18651457
8	rs73595761	18662625	G	0.3369	2.43E-10	chr:8:18651457
8	rs2638654	18666503	G	0.4305	2.52E-10	chr:8:18651457
8	rs12544207	18619231	A	0.4144	4.04E-10	chr:8:18651457
8	rs73199965	18616604	A	2.431	4.48E-10	chr:8:18651457
8	rs35809948	18647701	G	3.322	4.61E-10	chr:8:18651457
8	rs73666719	18648350	T	3.948	8.05E-10	chr:8:18651457
8	rs79076024	18614548	A	0.3942	8.07E-10	chr:8:18651457
8	rs73580705	18670105	C	0.3569	8.30E-10	chr:8:18651457
8	rs74936387	18649527	T	3.931	9.70E-10	chr:8:18651457
8	rs7814472	18632776	C	2.912	9.97E-10	chr:8:18651457
8	rs11988744	18617621	T	0.2841	1.08E-09	chr:8:18651457
8	rs10106772	18612008	A	2.397	1.16E-09	chr:8:18651457
8	rs78564754	18650296	C	4.215	1.26E-09	chr:8:18651457

8	rs73199966	18616606	A	2.351	1.42E-09	chr:8:18651457
8	rs6586762	18615706	C	0.3261	1.57E-09	chr:8:18651457
8	rs4320588	18615090	G	0.4327	2.82E-09	chr:8:18651457
8	rs55884038	18618142	C	2.307	3.14E-09	chr:8:18651457
8	rs7012188	18618272	T	2.307	3.14E-09	chr:8:18651457
8	rs335216	18627417	T	0.4322	3.22E-09	chr:8:18651457
8	rs79683508	18649812	A	4.074	3.32E-09	chr:8:18651457
8	rs28702887	18609522	C	2.288	3.88E-09	chr:8:18651457
8	rs10110943	18612612	A	2.258	7.06E-09	chr:8:18651457
8	rs73670009	18611236	A	4.514	7.77E-09	chr:8:18651457
8	rs73670007	18605496	C	4.272	1.35E-08	chr:8:18651457
8	rs10097648	18606611	T	0.4745	2.38E-08	chr:8:18651457
8	rs74525630	18612870	A	4.657	2.71E-08	chr:8:18651457
8	rs4921956	18620761	A	2.254	8.87E-08	chr:8:18651457
8	rs73670008	18606909	C	3.603	1.18E-07	chr:8:18651457
8	rs2306825	18656805	A	2.445	1.38E-07	chr:8:18651457
8	rs11778064	18665597	T	2.578	1.49E-07	chr:8:18651457
8	rs60806935	18656644	T	4.696	2.92E-07	chr:8:18651457
8	rs73666721	18648435	T	3.54	5.69E-07	chr:8:18651457
8	rs335243	18643985	C	19.14	6.73E-07	chr:8:18651457
8	rs335240	18642610	G	19.03	7.02E-07	chr:8:18651457
8	rs7839578	18640791	A	2.117	7.73E-07	chr:8:18651457
8	rs79655476	18630599	A	0.3788	1.07E-06	chr:8:18651457
8	rs335249	18646833	T	18.05	1.11E-06	chr:8:18651457
8	rs335253	18648477	T	4.18	1.19E-06	chr:8:18651457
8	rs111637875	18648034	G	0.3032	1.20E-06	chr:8:18651457
8	rs335242	18642711	A	17.85	1.25E-06	chr:8:18651457
8	rs77385050	18651205	C	0.2449	1.42E-06	chr:8:18651457
8	rs79606369	18657012	G	0.3344	1.43E-06	chr:8:18651457
8	rs111852071	18650213	C	0.2627	1.55E-06	chr:8:18651457
8	rs335226	18632945	T	0.5059	1.73E-06	chr:8:18651457
8	rs73593598	18627240	C	0.2185	2.00E-06	chr:8:18651457
8	rs11988686	18617442	C	0.5349	2.90E-06	chr:8:18651457
8	rs335233	18636629	A	4.75	3.36E-06	chr:8:18651457
8	rs59437212	18610782	T	1.844	3.90E-06	chr:8:18651457
8	rs335255	18649785	C	3.565	4.12E-06	chr:8:18651457
8	rs565693019	18609240	A	2.346	4.20E-06	chr:8:18651457
8	rs2638621	18656416	T	15.15	5.01E-06	chr:8:18651457
8	rs718418	18552276	T	0.5493	5.03E-06	chr:8:18651457
8	rs7000474	18551363	G	0.5508	5.32E-06	chr:8:18651457
8	rs182460095	18610167	A	3.365	5.74E-06	chr:8:18651457
8	rs57625991	18646741	G	2.767	6.05E-06	chr:8:18651457
8	rs17127179	18632112	T	2.179	6.11E-06	chr:8:18651457

8	rs77553985	18629003	C	0.2493	6.27E-06	chr:8:18651457
8	rs73199994	18646365	T	0.3874	6.41E-06	chr:8:18651457
8	rs335254	18649213	C	3.354	6.69E-06	chr:8:18651457
8	rs7837043	18577584	C	2.637	6.94E-06	chr:8:18651457
8	rs2016076	18552328	C	0.5546	7.38E-06	chr:8:18651457
8	rs77492095	18635805	T	0.2962	7.38E-06	chr:8:18651457
8	rs73593542	18593078	T	2.432	8.99E-06	chr:8:18651457
8	rs10503636	18654794	C	0.2952	1.09E-05	chr:8:18651457
8	rs335252	18647969	C	2.503	1.17E-05	chr:8:18651457
8	rs2632846	18660016	C	2.041	1.19E-05	chr:8:18651457
8	rs10503629	18556729	T	0.5403	1.23E-05	chr:8:18651457
8	rs57109550	18581347	C	2.62	1.37E-05	chr:8:18651457
8	rs7837572	18665140	T	1.802	1.38E-05	chr:8:18651457
8	rs77656612	18644639	C	0.2622	1.42E-05	chr:8:18651457
8	rs1492285	18655251	A	22.74	1.51E-05	chr:8:18651457
8	rs62495853	18604017	A	0.5187	1.64E-05	chr:8:18651457
8	rs1976160	18366489	C	1.804	1.67E-05	chr:8:18651457
8	rs1487748	18580153	A	2.55	1.75E-05	chr:8:18651457
8	rs73666698	18578566	A	2.55	1.75E-05	chr:8:18651457
8	rs17127182	18645394	C	0.2409	1.79E-05	chr:8:18651457
8	rs73595725	18643037	C	0.2409	1.79E-05	chr:8:18651457
8	rs150105022	18625560	A	0.2725	1.85E-05	chr:8:18651457
8	rs78614964	18555581	T	0.542	1.91E-05	chr:8:18651457
8	rs78680425	18651256	T	0.2615	2.02E-05	chr:8:18651457
8	rs1826767	18366560	G	1.79	2.06E-05	chr:8:18651457
8	rs17383368	18655746	T	0.3016	2.16E-05	chr:8:18651457
8	rs111916415	18656221	T	0.3042	2.31E-05	chr:8:18651457
8	rs35884486	18656382	C	0.3042	2.31E-05	chr:8:18651457
8	rs335225	18633305	A	21.1	2.37E-05	chr:8:18651457
8	rs66672830	18601318	C	2.343	2.38E-05	chr:8:18651457
8	rs1487765	18543277	T	0.5637	2.39E-05	chr:8:18651457
8	rs233197	18640628	A	20.88	2.51E-05	chr:8:18651457
8	rs527270	37059026	A	41.74	6.60E-47	chr:8:37050885
8	rs519407	37062657	T	23.83	6.14E-43	chr:8:37050885
8	rs574667	37070657	C	21.89	6.97E-42	chr:8:37050885
8	rs575360	37072211	T	21.89	6.97E-42	chr:8:37050885
8	rs507136	37066554	C	21.23	1.37E-41	chr:8:37050885
8	rs562346	37058605	C	30.74	1.70E-41	chr:8:37050885
8	rs514719	37063207	T	20.69	1.98E-41	chr:8:37050885
8	rs56097134	37067371	G	20.6	2.27E-41	chr:8:37050885
8	rs551464	37068041	T	20.15	3.59E-41	chr:8:37050885
8	rs496362	37073368	T	18.84	2.92E-40	chr:8:37050885
8	rs526765	37080954	A	18.25	3.07E-40	chr:8:37050885

8	rs2697894	37076650	C	17.67	7.87E-40	chr:8:37050885
8	rs2843834	37078884	T	17.67	7.87E-40	chr:8:37050885
8	rs501761	37073910	C	17.67	7.87E-40	chr:8:37050885
8	rs547855	37078106	A	17.67	7.87E-40	chr:8:37050885
8	rs575589	37077120	A	17.67	7.87E-40	chr:8:37050885
8	rs2843835	37078820	T	17.37	8.11E-40	chr:8:37050885
8	rs571788	37078413	T	17.41	1.29E-39	chr:8:37050885
8	rs473160	37073161	C	17.37	1.47E-39	chr:8:37050885
8	rs500960	37073841	G	17.37	1.47E-39	chr:8:37050885
8	rs473969	37055831	G	38.33	2.39E-39	chr:8:37050885
8	rs571824	37077544	G	16.98	3.28E-39	chr:8:37050885
8	rs577113	37077012	G	16.98	3.28E-39	chr:8:37050885
8	rs491607	37054032	G	36.82	9.26E-39	chr:8:37050885
8	rs510561	37055719	T	36.82	9.26E-39	chr:8:37050885
8	rs240524	37088271	T	15.64	1.83E-38	chr:8:37050885
8	rs495428	37079790	A	15.45	3.71E-38	chr:8:37050885
8	rs509011	37084128	C	15.45	3.71E-38	chr:8:37050885
8	rs525751	37080834	A	15.45	3.71E-38	chr:8:37050885
8	rs579858	37072747	C	15.45	3.71E-38	chr:8:37050885
8	rs493477	37053824	C	34.09	6.03E-38	chr:8:37050885
8	rs539797	37077235	G	15.25	6.35E-38	chr:8:37050885
8	rs240521	37086908	A	14.8	1.43E-37	chr:8:37050885
8	rs240522	37087609	T	14.8	1.43E-37	chr:8:37050885
8	rs240518	37085509	C	13.9	6.66E-37	chr:8:37050885
8	rs493539	37069825	G	19.7	6.75E-28	chr:8:37050885
8	rs567469	37064847	G	6.061	5.92E-26	chr:8:37050885
8	rs4739484	37082138	A	16.48	1.12E-24	chr:8:37050885
8	rs28452965	37062127	C	0.2204	2.88E-20	chr:8:37050885
8	rs537583	37058174	G	815.5	2.55E-19	chr:8:37050885
8	rs473731	37082568	A	0.2458	1.38E-18	chr:8:37050885
8	rs494545	37079696	T	0.2458	1.38E-18	chr:8:37050885
8	rs543162	37078419	T	0.2458	1.38E-18	chr:8:37050885
8	rs2697887	37078928	A	0.2462	1.53E-18	chr:8:37050885
8	rs553163	37085269	G	0.2458	1.63E-18	chr:8:37050885
8	rs240520	37086850	G	0.2458	1.98E-18	chr:8:37050885
8	rs240523	37087732	C	0.2458	1.98E-18	chr:8:37050885
8	rs6468397	37087364	C	0.2689	6.08E-18	chr:8:37050885
8	rs7014438	37088100	G	0.2689	6.08E-18	chr:8:37050885
8	rs10504000	37075320	A	0.2641	6.31E-18	chr:8:37050885
8	rs7010606	37088266	G	0.2698	1.18E-17	chr:8:37050885
8	rs542245	37062505	T	465.5	4.40E-17	chr:8:37050885
8	rs10107112	37079810	G	0.2793	5.93E-17	chr:8:37050885
8	rs6993545	37084686	G	0.3013	5.02E-16	chr:8:37050885

8	rs7012524	37084943	T	0.3035	7.11E-16	chr:8:37050885
8	rs7818972	37083118	C	0.3051	7.98E-16	chr:8:37050885
8	rs74571086	37049958	G	18.68	1.22E-15	chr:8:37050885
8	rs10105918	37084184	T	0.3108	1.41E-15	chr:8:37050885
8	rs10092203	37084145	G	0.3164	2.43E-15	chr:8:37050885
8	rs6468396	37083079	T	0.3164	2.43E-15	chr:8:37050885
8	rs34456013	37049781	A	22.25	2.78E-14	chr:8:37050885
8	rs990711	37058871	T	0.3146	4.13E-14	chr:8:37050885
8	rs16886251	37086493	A	18.8	4.37E-14	chr:8:37050885
8	rs113776709	37057199	A	15.4	7.73E-14	chr:8:37050885
8	rs13439621	37058555	C	0.2761	1.19E-13	chr:8:37050885
8	rs2843836	37076582	C	0.3095	8.16E-13	chr:8:37050885
8	rs115677542	37056997	A	14.9	2.59E-12	chr:8:37050885
8	rs41464350	37061196	T	14.6	3.80E-12	chr:8:37050885
8	rs78334371	37053704	T	14.27	6.37E-12	chr:8:37050885
8	rs75081645	37065559	C	18.81	3.40E-11	chr:8:37050885
8	rs9942819	37077283	G	18.81	3.40E-11	chr:8:37050885
8	rs116544019	37099310	A	4.969	4.58E-11	chr:8:37050885
8	rs511629	37060493	T	8.663	6.14E-11	chr:8:37050885
8	rs10098775	37027048	C	3.014	2.77E-10	chr:8:37050885
8	rs16886107	37021020	A	3.014	2.77E-10	chr:8:37050885
8	rs16886109	37022009	C	3.014	2.77E-10	chr:8:37050885
8	rs28445234	37025250	T	3.014	2.77E-10	chr:8:37050885
8	rs10088440	37034372	T	2.966	3.49E-10	chr:8:37050885
8	rs9886466	37123919	T	3.617	3.64E-10	chr:8:37050885
8	rs16886104	37018915	A	2.976	3.90E-10	chr:8:37050885
8	rs146959665	37031849	G	2.928	4.78E-10	chr:8:37050885
8	rs471141	37067066	G	3.522	1.26E-09	chr:8:37050885
8	rs490827	37079275	G	3.522	1.26E-09	chr:8:37050885
8	rs526525	37080867	C	3.522	1.26E-09	chr:8:37050885
8	rs16886139	37037716	A	13.96	2.82E-09	chr:8:37050885
8	rs16886310	37117101	A	5.213	3.19E-09	chr:8:37050885
8	rs10109088	37114613	G	5.879	3.53E-09	chr:8:37050885
8	rs114445131	37119249	T	5.879	3.53E-09	chr:8:37050885
8	rs116372178	37119598	A	5.879	3.53E-09	chr:8:37050885
8	rs9642715	37038245	T	0.3938	4.34E-09	chr:8:37050885
8	rs115350687	37098527	A	5.102	4.41E-09	chr:8:37050885
8	rs16886292	37103781	G	5.129	4.71E-09	chr:8:37050885
8	rs76313110	37107769	T	21.39	7.33E-09	chr:8:37050885
8	rs12675666	37166328	T	3.037	7.90E-09	chr:8:37050885
8	rs77533443	37107398	G	20.82	9.26E-09	chr:8:37050885
8	rs16886110	37022683	G	10.86	1.08E-08	chr:8:37050885
8	rs17388043	37069029	A	20.36	1.28E-08	chr:8:37050885

8	rs114786140	37097021	T	20.06	1.40E-08	chr:8:37050885
8	rs114825594	37100090	G	20.06	1.40E-08	chr:8:37050885
8	rs4739483	37076718	C	0.3738	1.59E-08	chr:8:37050885
8	rs479974	37039730	C	6.111	2.39E-08	chr:8:37050885
8	rs16886161	37067053	A	18.88	3.14E-08	chr:8:37050885
8	rs511544	37076504	A	0.4854	4.81E-08	chr:8:37050885
8	rs16886316	37118921	C	2.154	5.61E-08	chr:8:37050885
8	rs116055059	37060727	A	25.57	8.39E-08	chr:8:37050885
8	rs116182831	37058103	C	25.57	8.39E-08	chr:8:37050885
8	rs116371587	37061780	C	25.57	8.39E-08	chr:8:37050885
8	rs116452276	37058758	T	25.57	8.39E-08	chr:8:37050885
8	rs16886146	37054359	G	25.57	8.39E-08	chr:8:37050885
8	rs11985899	37083414	C	16.88	9.78E-08	chr:8:37050885
8	rs80144412	37001208	G	8.271	1.04E-07	chr:8:37050885
8	rs490027	37079215	T	0.4959	1.04E-07	chr:8:37050885
8	rs80074131	37088052	A	24.74	1.06E-07	chr:8:37050885
8	rs532022	37074938	C	0.4979	1.32E-07	chr:8:37050885
8	rs557361	37075388	G	0.4979	1.32E-07	chr:8:37050885
8	rs114935497	37075718	A	23.8	1.56E-07	chr:8:37050885
8	rs115614079	37073903	A	23.8	1.56E-07	chr:8:37050885
8	rs16875922	37048590	T	23.8	1.56E-07	chr:8:37050885
8	rs16886184	37075925	C	23.8	1.56E-07	chr:8:37050885
8	rs16886208	37078627	A	23.8	1.56E-07	chr:8:37050885
8	rs16886221	37079378	T	23.8	1.56E-07	chr:8:37050885
8	rs16886224	37079569	G	23.8	1.56E-07	chr:8:37050885
8	rs16886246	37083636	C	23.8	1.56E-07	chr:8:37050885
8	rs534138	37038476	T	4.818	1.92E-07	chr:8:37050885
8	rs540668	37037736	G	2.222	2.01E-07	chr:8:37050885
8	rs240517	37085448	G	0.505	2.28E-07	chr:8:37050885
8	rs16886046	37008070	A	3.106	2.32E-07	chr:8:37050885
8	rs41523746	37038507	A	2.891	3.14E-07	chr:8:37050885
8	rs7016400	37041193	A	2.904	3.48E-07	chr:8:37050885
8	rs74815246	37052254	A	21.46	3.66E-07	chr:8:37050885
8	rs16875904	37042654	G	2.86	4.38E-07	chr:8:37050885
8	rs536020	37038246	A	4.224	6.26E-07	chr:8:37050885
8	rs114890013	37127765	C	14.14	7.67E-07	chr:8:37050885
8	rs116120465	37124624	T	14.14	7.67E-07	chr:8:37050885
8	rs79314359	37127740	A	14.14	7.67E-07	chr:8:37050885
8	rs77212681	37075679	C	35.61	1.04E-06	chr:8:37050885
8	rs79340345	37101154	C	19.46	1.11E-06	chr:8:37050885
8	rs78663547	37171192	T	13.51	1.26E-06	chr:8:37050885
8	rs114268034	37128838	C	19.06	1.36E-06	chr:8:37050885
8	rs115621925	37128756	A	19.06	1.36E-06	chr:8:37050885

8	rs114935936	37091894	A	19	1.39E-06	chr:8:37050885
8	rs116729280	37075079	T	10.24	1.45E-06	chr:8:37050885
8	rs573219083	37037395	T	2.337	1.98E-06	chr:8:37050885
8	rs28631916	37085115	T	0.5427	2.30E-06	chr:8:37050885
8	rs115838954	37035473	T	9.52	3.34E-06	chr:8:37050885
8	rs140102131	36996904	T	9.591	3.34E-06	chr:8:37050885
8	rs16886306	37114117	C	4.481	3.74E-06	chr:8:37050885
8	rs16886317	37119197	C	4.481	3.74E-06	chr:8:37050885
8	rs76369532	37109388	A	4.481	3.74E-06	chr:8:37050885
8	rs7843213;rs565336389	37045329	A	0.4494	3.79E-06	chr:8:37050885
8	rs240519	37085713	C	0.5509	3.89E-06	chr:8:37050885
8	rs114187905	37354897	T	4.435	4.01E-06	chr:8:37050885
8	rs10092766	37141088	A	4.806	4.50E-06	chr:8:37050885
8	rs10098526	37129657	A	4.806	4.50E-06	chr:8:37050885
8	rs13439666	37125761	A	4.806	4.50E-06	chr:8:37050885
8	rs139794406	37131663	T	4.806	4.50E-06	chr:8:37050885
8	rs150650554	37131662	T	4.806	4.50E-06	chr:8:37050885
8	rs143997725	37345866	T	3.969	7.63E-06	chr:8:37050885
8	rs576019	37044187	T	2.075	7.91E-06	chr:8:37050885
8	rs12334605	37045753	G	2.084	8.00E-06	chr:8:37050885
8	rs7835227	37076680	T	0.2916	8.29E-06	chr:8:37050885
8	rs405459	37170113	A	1.853	8.84E-06	chr:8:37050885
8	rs12334611	37045986	C	2.061	9.27E-06	chr:8:37050885
8	rs16875909	37043868	T	2.058	1.15E-05	chr:8:37050885
8	rs116542566	37064744	T	0.3489	1.18E-05	chr:8:37050885
8	rs75672461	37053797	A	24.46	1.22E-05	chr:8:37050885
8	rs16886141	37038562	T	0.337	1.51E-05	chr:8:37050885
8	rs4739476	37046991	A	2.003	1.60E-05	chr:8:37050885
8	rs72639371	37210037	G	2.129	1.68E-05	chr:8:37050885
8	rs140888150	37351085	T	3.591	1.69E-05	chr:8:37050885
8	rs562322	37036994	C	0.5799	2.25E-05	chr:8:37050885
8	rs4237086	37088022	G	0.3844	2.70E-05	chr:8:37050885
8	rs59787506	37113679	T	0.4626	2.86E-05	chr:8:37050885
8	rs73595262	37108512	A	0.4626	2.86E-05	chr:8:37050885
8	rs73595266	37108993	T	0.4626	2.86E-05	chr:8:37050885
8	rs73595270	37109513	T	0.4626	2.86E-05	chr:8:37050885
8	rs73673154	37112782	A	0.4626	2.86E-05	chr:8:37050885
8	rs3958649	37174113	A	0.3353	2.87E-05	chr:8:37050885
8	rs11776866	37137268	A	3.483	2.91E-05	chr:8:37050885
8	rs7814754	37128956	A	3.483	2.91E-05	chr:8:37050885
8	rs75881701	37072693	A	0.4192	3.17E-05	chr:8:37050885
8	rs80257076	37072523	T	0.4192	3.17E-05	chr:8:37050885
8	rs111999509	7355419	A	32.55	3.46E-28	chr:8:7355397

8	rs62639800	7372247	T	5.349	1.06E-27	chr:8:7355397
8	rs76206197	7371208	C	4.992	1.79E-27	chr:8:7355397
8	rs74787406	7360893	G	41.41	4.08E-27	chr:8:7355397
8	rs562847555	7354734	A	39.85	3.83E-23	chr:8:7355397
8	rs28548808	7341290	C	6.647	4.43E-21	chr:8:7355397
8	rs2737983	7365975	A	4.492	8.50E-20	chr:8:7355397
8	rs144460864	7336269	C	5.157	2.94E-18	chr:8:7355397
8	rs148764079	7370001	A	8.299	6.67E-17	chr:8:7355397
8	rs544637792	7352785	C	4.096	8.65E-17	chr:8:7355397
8	rs370867537	7371467	A	5.623	2.80E-16	chr:8:7355397
8	rs140959028	7443705	T	4.32	1.01E-13	chr:8:7355397
8	rs141973795	7663771	A	4.061	6.89E-13	chr:8:7355397
8	rs566529784	7360744	G	5.428	2.07E-11	chr:8:7355397
8	rs76122916	7455141	C	2.745	4.15E-11	chr:8:7355397
8	rs74375471	7455070	A	2.655	8.94E-11	chr:8:7355397
8	rs191332947	7364475	G	47.17	2.66E-10	chr:8:7355397
8	rs189903431	7373837	C	21.64	1.87E-08	chr:8:7355397
8	rs369910328	7454478	G	2.183	2.55E-08	chr:8:7355397
8	rs527934971	7467707	A	3.247	5.10E-08	chr:8:7355397
8	rs149317882	7781800	T	14.7	5.39E-08	chr:8:7355397
8	rs62639797	7367263	G	2.185	2.38E-07	chr:8:7355397
8	rs201256319	7353480	T	0.2541	3.11E-07	chr:8:7355397
8	rs115213243	7334665	G	5.814	3.73E-07	chr:8:7355397
8	rs529245338	7553177	G	2.929	3.98E-07	chr:8:7355397
8	rs572078497	7448887	T	0.328	4.66E-07	chr:8:7355397
8	rs552674485	7375209	G	2.164	7.34E-07	chr:8:7355397
8	rs77333779	7375207	A	2.164	7.34E-07	chr:8:7355397
8	rs141402280	7388108	A	0.3034	1.02E-06	chr:8:7355397
8	rs76675735	7463745	G	2.039	1.13E-06	chr:8:7355397
8	rs148154904	7385844	T	0.3588	1.18E-06	chr:8:7355397
8	rs60249228	7693605	C	2.404	2.22E-06	chr:8:7355397
8	rs11786478	7388186	A	0.3556	3.97E-06	chr:8:7355397
8	rs536198816	7674567	G	1.862	6.31E-06	chr:8:7355397
8	rs369528899	7154084	G	2.072	1.40E-05	chr:8:7355397
8	rs553568302	7046965	G	10.99	1.73E-05	chr:8:7355397
8	rs569623292	7292914	T	2.687	1.85E-05	chr:8:7355397
8	rs145467665	7009245	T	4.518	1.92E-05	chr:8:7355397
8	rs141701100	7684747	A	1.776	2.10E-05	chr:8:7355397
8	rs150530577	7684750	T	1.776	2.10E-05	chr:8:7355397
8	rs77605644	7448886	T	0.3191	3.27E-05	chr:8:7355397
8	rs77049685	7723630	T	1.77	4.88E-05	chr:8:7355397
8	rs62636823	7239240	T	0.3144	5.06E-05	chr:8:7355397
8	rs2680513	7691765	T	1.793	5.65E-05	chr:8:7355397

Table S3: Associations of hiSNPs with polymprphic HERV-K insertions with Amerindian super popul

CHROM	RSID	POS	ALT	OR	P-VAL	HERV
10	rs1854455	135354874	A	0.2105	6.41E-08	chr:10:135355522
10	rs4615946	135355394	G	0.2105	6.41E-08	chr:10:135355522
10	rs8192781	135354251	T	0.2105	6.41E-08	chr:10:135355522
10	rs2480256	135352514	A	0.2097	6.46E-08	chr:10:135355522
10	rs10857739	135352697	G	0.2101	6.49E-08	chr:10:135355522
10	rs8192780	135354125	T	0.2101	6.49E-08	chr:10:135355522
10	rs1952467	135353499	T	0.2169	9.80E-08	chr:10:135355522
10	rs2480258	135352100	T	0.2169	9.80E-08	chr:10:135355522
10	rs2480259	135352076	A	0.2169	9.80E-08	chr:10:135355522
10	rs2515642	135352013	C	0.2169	9.80E-08	chr:10:135355522
10	rs2515644	135353079	A	0.2169	9.80E-08	chr:10:135355522
10	rs4838771	135355007	C	0.2211	1.24E-07	chr:10:135355522
10	rs2987794	135378612	G	0.2195	1.29E-07	chr:10:135355522
10	rs2480255	135352855	T	0.2289	1.61E-07	chr:10:135355522
10	rs8192776	135348187	T	0.2367	1.70E-07	chr:10:135355522
10	rs4838693	135354714	G	0.2325	1.91E-07	chr:10:135355522
10	rs61868336	135350060	C	0.2386	2.00E-07	chr:10:135355522
10	rs4240522	135355557	C	0.2458	2.91E-07	chr:10:135355522
10	rs4363513	135355534	A	0.2458	2.91E-07	chr:10:135355522
10	rs4542321	135355481	T	0.2458	2.91E-07	chr:10:135355522
10	rs1536826	135357239	A	0.2415	2.93E-07	chr:10:135355522
10	rs2480254	135352869	T	0.2455	2.98E-07	chr:10:135355522
10	rs2480257	135352509	T	0.2451	3.01E-07	chr:10:135355522
10	rs10857768	135402703	G	0.2495	3.26E-07	chr:10:135355522
10	rs3020513	135364915	T	0.2462	3.33E-07	chr:10:135355522
10	rs2987792	135371466	T	0.2459	3.34E-07	chr:10:135355522
10	rs4512750	135358664	T	0.2459	3.34E-07	chr:10:135355522
10	rs3020501	135381592	C	0.2485	4.02E-07	chr:10:135355522
10	rs11101867	135400250	A	0.2554	4.29E-07	chr:10:135355522
10	rs11101890	135421321	A	0.2554	4.29E-07	chr:10:135355522
10	rs4129223	135403948	G	0.2554	4.29E-07	chr:10:135355522
10	rs4133041	135408387	A	0.2554	4.29E-07	chr:10:135355522
10	rs4259784	135399192	A	0.2554	4.29E-07	chr:10:135355522
10	rs4306261	135410989	G	0.2554	4.29E-07	chr:10:135355522
10	rs4351776	135415572	T	0.2554	4.29E-07	chr:10:135355522
10	rs7084384	135412322	G	0.2554	4.29E-07	chr:10:135355522
10	rs7084391	135412344	C	0.2554	4.29E-07	chr:10:135355522
10	rs77382433	135389919	C	0.2554	4.29E-07	chr:10:135355522
10	rs7907135	135410660	C	0.2554	4.29E-07	chr:10:135355522
10	rs7093170	135402904	T	0.2551	4.31E-07	chr:10:135355522
10	rs2987799	135380499	T	0.2487	4.63E-07	chr:10:135355522

10	rs10857765	135394613	C	0.2568	4.87E-07	chr:10:135355522
10	rs10857769	135405047	C	0.2568	4.87E-07	chr:10:135355522
10	rs4335490	135402350	C	0.2568	4.87E-07	chr:10:135355522
10	rs6537613	135412414	G	0.2568	4.87E-07	chr:10:135355522
10	rs7075572	135404358	C	0.2568	4.87E-07	chr:10:135355522
10	rs9629936	135398783	G	0.2568	4.87E-07	chr:10:135355522
10	rs9630012	135397511	T	0.2568	4.87E-07	chr:10:135355522
10	rs9630033	135418505	T	0.2568	4.87E-07	chr:10:135355522
10	rs7922540	135395468	T	0.2566	4.89E-07	chr:10:135355522
10	rs9629935	135398306	C	0.2578	5.28E-07	chr:10:135355522
10	rs4556495	135422209	T	0.266	7.06E-07	chr:10:135355522
10	rs117220052	135388573	A	0.288	9.97E-07	chr:10:135355522
10	rs12218790	135434551	A	0.2361	1.08E-06	chr:10:135355522
10	rs9418991	135362114	C	0.2556	1.14E-06	chr:10:135355522
10	rs61868695	135380408	G	0.2757	1.38E-06	chr:10:135355522
10	rs61868696	135380409	C	0.2757	1.38E-06	chr:10:135355522
10	rs61868697	135380410	A	0.2757	1.38E-06	chr:10:135355522
10	rs2987785	135365003	C	0.283	1.60E-06	chr:10:135355522
10	rs11101857	135395359	G	0.2933	2.36E-06	chr:10:135355522
10	rs7094588	135401586	G	0.2933	2.36E-06	chr:10:135355522
10	rs2243909	135279810	T	0.2566	2.64E-06	chr:10:135355522
10	rs11101870	135402702	T	0.2318	3.26E-06	chr:10:135355522
10	rs1536828	135342315	G	0.3138	3.70E-06	chr:10:135355522
10	rs7092584	135348257	T	0.2215	4.12E-06	chr:10:135355522
10	rs2026047	135329616	C	0.3099	7.90E-06	chr:10:135355522
10	rs1854458	135313815	G	0.3156	9.34E-06	chr:10:135355522
10	rs2274665	135295630	T	0.3156	9.34E-06	chr:10:135355522
10	rs4838687	135295396	C	0.3156	9.34E-06	chr:10:135355522
10	rs7097581	135295350	A	0.3156	9.34E-06	chr:10:135355522
10	rs9418982	135320622	C	0.3156	9.34E-06	chr:10:135355522
10	rs9418984	135322356	G	0.3156	9.34E-06	chr:10:135355522
10	rs9418987	135324409	A	0.3156	9.34E-06	chr:10:135355522
10	rs9419072	135310445	G	0.3156	9.34E-06	chr:10:135355522
10	rs142168980	135389786	C	0.3309	9.87E-06	chr:10:135355522
10	rs2070673	135340567	A	0.3137	1.13E-05	chr:10:135355522
10	rs2247214	135285337	C	0.3069	1.31E-05	chr:10:135355522
10	rs9419079	135326985	G	0.3239	1.35E-05	chr:10:135355522
10	rs2070675	135346696	T	0.3021	1.39E-05	chr:10:135355522
10	rs1581934	135327410	T	0.3088	1.84E-05	chr:10:135355522
10	rs4543900	135310808	G	0.3036	1.92E-05	chr:10:135355522
10	rs9418986	135324276	T	0.3046	2.04E-05	chr:10:135355522
10	rs11101794	135307667	C	0.3064	2.15E-05	chr:10:135355522
10	rs7907225	135320905	G	0.3061	2.17E-05	chr:10:135355522

10	rs9419082	135333772	A	0.2728	2.31E-05	chr:10:135355522
10	rs10857730	135326822	G	0.3217	2.55E-05	chr:10:135355522
10	rs10857736	135336514	G	0.2886	2.86E-05	chr:10:135355522
10	rs9418990	135337966	C	0.3474	2.87E-05	chr:10:135355522
10	rs10776685	135327317	C	0.3245	2.90E-05	chr:10:135355522
10	rs11815623	135329133	G	0.3245	2.90E-05	chr:10:135355522
10	rs7095379	135328532	A	0.3245	2.90E-05	chr:10:135355522
10	rs7095996	135328948	A	0.3245	2.90E-05	chr:10:135355522
10	rs9418988	135327632	C	0.3473	2.91E-05	chr:10:135355522
10	rs915907	135346927	A	0.3016	3.28E-05	chr:10:135355522
10	rs2247229	135285880	T	0.331	3.72E-05	chr:10:135355522
10	rs1590820	135296816	A	0.3557	3.95E-05	chr:10:135355522
10	rs2149618	135288814	G	0.3309	4.36E-05	chr:10:135355522
10	rs6537609	135323084	G	0.3406	4.77E-05	chr:10:135355522
10	rs7083073	135322956	A	0.3406	4.77E-05	chr:10:135355522
10	rs7092634	135322977	A	0.3406	4.77E-05	chr:10:135355522
10	rs7911139	135323525	C	0.3406	4.77E-05	chr:10:135355522
10	rs8192766	135339385	G	0.3018	5.11E-05	chr:10:135355522
10	rs10857735	135336230	A	0.3014	5.13E-05	chr:10:135355522
10	rs10857737	135336716	A	0.3014	5.13E-05	chr:10:135355522
10	rs11101807	135335647	C	0.3014	5.13E-05	chr:10:135355522
10	rs11101809	135335746	T	0.3014	5.13E-05	chr:10:135355522
10	rs7077457	135324370	A	0.3445	5.29E-05	chr:10:135355522
11	rs140837952	60315830	C	140.2	4.74E-22	chr:11:60449890
11	rs80331353	60276293	T	132.8	1.58E-21	chr:11:60449890
11	rs4939385	60275473	T	94.98	3.38E-21	chr:11:60449890
11	rs4939386	60275719	A	118	3.71E-21	chr:11:60449890
11	rs17155107	60275273	C	110.7	3.91E-21	chr:11:60449890
11	rs1941022	60272887	A	61.3	3.81E-20	chr:11:60449890
11	rs76860074	60472292	G	313.5	4.66E-20	chr:11:60449890
11	rs376103871	60434536	G	454.4	7.09E-20	chr:11:60449890
11	rs61898831	60518070	A	40.87	9.52E-20	chr:11:60449890
11	rs139710185	60386332	C	329.3	1.05E-19	chr:11:60449890
11	rs74976810	60280059	T	82.59	2.21E-19	chr:11:60449890
11	rs76701960	60281519	C	82.59	2.21E-19	chr:11:60449890
11	rs569629432	60514937	G	42.95	3.22E-19	chr:11:60449890
11	rs61898828	60513780	T	42.95	3.22E-19	chr:11:60449890
11	rs4939434	60413170	T	358.1	3.32E-19	chr:11:60449890
11	rs150026789	60240149	C	32.99	3.46E-19	chr:11:60449890
11	rs17155034	60237378	A	32.99	3.46E-19	chr:11:60449890
11	rs17155041	60237414	G	32.99	3.46E-19	chr:11:60449890
11	rs185777027	60252475	A	32.99	3.46E-19	chr:11:60449890
11	rs4617613	60250648	T	32.99	3.46E-19	chr:11:60449890

11	rs4939365	60238824	A	32.99	3.46E-19	chr:11:60449890
11	rs7112881	60235600	C	32.99	3.46E-19	chr:11:60449890
11	rs7129314	60235459	G	32.99	3.46E-19	chr:11:60449890
11	rs74237978	60254725	G	32.99	3.46E-19	chr:11:60449890
11	rs76814174	60256208	A	32.99	3.46E-19	chr:11:60449890
11	rs77828735	60251575	C	32.99	3.46E-19	chr:11:60449890
11	rs75976166	60511961	T	250.6	5.12E-19	chr:11:60449890
11	rs925010	60512786	T	250.6	5.12E-19	chr:11:60449890
11	rs925011	60512497	T	250.6	5.12E-19	chr:11:60449890
11	rs150759751	60243215	C	28.6	5.69E-19	chr:11:60449890
11	rs557131820	60243025	G	28.6	5.69E-19	chr:11:60449890
11	rs1395401	60415354	C	475	6.84E-19	chr:11:60449890
11	rs146864929	60419881	T	475	6.84E-19	chr:11:60449890
11	rs183839528	60419770	C	475	6.84E-19	chr:11:60449890
11	rs4939436	60424565	A	475	6.84E-19	chr:11:60449890
11	rs75177700	60420926	A	475	6.84E-19	chr:11:60449890
11	rs75606468	60413595	G	475	6.84E-19	chr:11:60449890
11	rs78401374	60425155	G	475	6.84E-19	chr:11:60449890
11	rs79053314	60409541	A	475	6.84E-19	chr:11:60449890
11	rs80322405	60425758	T	475	6.84E-19	chr:11:60449890
11	rs76753770	60426674	A	444.4	7.22E-19	chr:11:60449890
11	rs78897592	60426552	T	444.4	7.22E-19	chr:11:60449890
11	rs111662795	60420336	T	436.7	8.91E-19	chr:11:60449890
11	rs112026902	60420507	T	436.7	8.91E-19	chr:11:60449890
11	rs112061705	60414568	T	436.7	8.91E-19	chr:11:60449890
11	rs112285293	60419923	C	436.7	8.91E-19	chr:11:60449890
11	rs112410934	60420346	A	436.7	8.91E-19	chr:11:60449890
11	rs113304615	60420369	C	436.7	8.91E-19	chr:11:60449890
11	rs113359775	60413824	C	436.7	8.91E-19	chr:11:60449890
11	rs113646818	60420205	T	436.7	8.91E-19	chr:11:60449890
11	rs114299280	60419780	T	436.7	8.91E-19	chr:11:60449890
11	rs114357683	60422696	G	436.7	8.91E-19	chr:11:60449890
11	rs115069163	60419768	T	436.7	8.91E-19	chr:11:60449890
11	rs115943468	60413681	C	436.7	8.91E-19	chr:11:60449890
11	rs1354770	60416509	A	436.7	8.91E-19	chr:11:60449890
11	rs1354771	60416483	C	436.7	8.91E-19	chr:11:60449890
11	rs1354772	60416327	C	436.7	8.91E-19	chr:11:60449890
11	rs1354773	60416315	A	436.7	8.91E-19	chr:11:60449890
11	rs138028975	60420106	C	436.7	8.91E-19	chr:11:60449890
11	rs1395399	60415721	T	436.7	8.91E-19	chr:11:60449890
11	rs1395400	60415572	T	436.7	8.91E-19	chr:11:60449890
11	rs1395402	60421420	A	436.7	8.91E-19	chr:11:60449890
11	rs1395404	60421807	A	436.7	8.91E-19	chr:11:60449890

11	rs1395405	60422296	T	436.7	8.91E-19	chr:11:60449890
11	rs140613292	60418035	T	436.7	8.91E-19	chr:11:60449890
11	rs143370276	60420110	A	436.7	8.91E-19	chr:11:60449890
11	rs17155185	60415989	C	436.7	8.91E-19	chr:11:60449890
11	rs17155186	60416072	C	436.7	8.91E-19	chr:11:60449890
11	rs17155188	60416175	G	436.7	8.91E-19	chr:11:60449890
11	rs2129786	60423103	G	436.7	8.91E-19	chr:11:60449890
11	rs2129787	60423827	T	436.7	8.91E-19	chr:11:60449890
11	rs2129788	60423946	T	436.7	8.91E-19	chr:11:60449890
11	rs2171485	60422921	A	436.7	8.91E-19	chr:11:60449890
11	rs4304796	60415413	G	436.7	8.91E-19	chr:11:60449890
11	rs4938968	60422399	T	436.7	8.91E-19	chr:11:60449890
11	rs4938969	60422434	G	436.7	8.91E-19	chr:11:60449890
11	rs4939429	60412257	T	436.7	8.91E-19	chr:11:60449890
11	rs4939430	60412276	A	436.7	8.91E-19	chr:11:60449890
11	rs4939431	60412331	T	436.7	8.91E-19	chr:11:60449890
11	rs4939432	60412583	G	436.7	8.91E-19	chr:11:60449890
11	rs4939435	60419373	G	436.7	8.91E-19	chr:11:60449890
11	rs74237988	60419683	A	436.7	8.91E-19	chr:11:60449890
11	rs74551327	60422947	C	436.7	8.91E-19	chr:11:60449890
11	rs74658643	60425757	G	436.7	8.91E-19	chr:11:60449890
11	rs7479031	60415822	C	436.7	8.91E-19	chr:11:60449890
11	rs74793391	60420807	T	436.7	8.91E-19	chr:11:60449890
11	rs7483244	60421757	C	436.7	8.91E-19	chr:11:60449890
11	rs75598587	60422757	G	436.7	8.91E-19	chr:11:60449890
11	rs75841265	60420812	T	436.7	8.91E-19	chr:11:60449890
11	rs76365878	60425214	C	436.7	8.91E-19	chr:11:60449890
11	rs77026665	60417687	A	436.7	8.91E-19	chr:11:60449890
11	rs77125902	60425141	T	436.7	8.91E-19	chr:11:60449890
11	rs77663416	60426250	G	436.7	8.91E-19	chr:11:60449890
11	rs77726795	60425419	G	436.7	8.91E-19	chr:11:60449890
11	rs78392907	60411069	C	436.7	8.91E-19	chr:11:60449890
11	rs78474464	60418171	T	436.7	8.91E-19	chr:11:60449890
11	rs78637342	60426005	C	436.7	8.91E-19	chr:11:60449890
11	rs79513455	60426156	A	436.7	8.91E-19	chr:11:60449890
11	rs79568121	60426280	C	436.7	8.91E-19	chr:11:60449890
11	rs80082757	60420883	A	436.7	8.91E-19	chr:11:60449890
11	rs80102522	60425369	G	436.7	8.91E-19	chr:11:60449890
11	rs80255668	60415835	T	436.7	8.91E-19	chr:11:60449890
11	rs36043646	60404929	A	168.9	1.16E-18	chr:11:60449890
11	rs4939428	60404458	G	168.9	1.16E-18	chr:11:60449890
11	rs11823491	60398174	T	166.7	1.31E-18	chr:11:60449890
11	rs181323223	60389622	G	166.7	1.31E-18	chr:11:60449890

11	rs12223063	60309438	T	104.9	1.60E-18	chr:11:60449890
11	rs4938955	60298963	G	104.9	1.60E-18	chr:11:60449890
11	rs4939398	60298580	C	104.9	1.60E-18	chr:11:60449890
11	rs79002350	60285803	T	104.9	1.60E-18	chr:11:60449890
11	rs61898829	60514367	C	49.17	1.63E-18	chr:11:60449890
11	rs73492994	60515078	T	49.17	1.63E-18	chr:11:60449890
11	rs10501387	60386979	T	444.7	1.76E-18	chr:11:60449890
11	rs375531665	60389366	T	444.7	1.76E-18	chr:11:60449890
11	rs536155208	60389985	T	444.7	1.76E-18	chr:11:60449890
11	rs552861910	60390547	A	444.7	1.76E-18	chr:11:60449890
11	rs74237985	60397751	C	444.7	1.76E-18	chr:11:60449890
11	rs74362668	60377300	A	444.7	1.76E-18	chr:11:60449890
11	rs74796030	60373039	C	444.7	1.76E-18	chr:11:60449890
11	rs75858171	60377478	C	444.7	1.76E-18	chr:11:60449890
11	rs76118857	60397070	G	444.7	1.76E-18	chr:11:60449890
11	rs77082032	60380341	C	444.7	1.76E-18	chr:11:60449890
11	rs77624740	60364445	T	444.7	1.76E-18	chr:11:60449890
11	rs77713821	60394593	G	444.7	1.76E-18	chr:11:60449890
11	rs77948025	60385189	C	444.7	1.76E-18	chr:11:60449890
11	rs79192023	60398204	C	444.7	1.76E-18	chr:11:60449890
11	rs79250688	60397495	A	444.7	1.76E-18	chr:11:60449890
11	rs79465298	60403208	G	444.7	1.76E-18	chr:11:60449890
11	rs79519698	60384232	A	444.7	1.76E-18	chr:11:60449890
11	rs80043843	60368375	C	444.7	1.76E-18	chr:11:60449890
11	rs75071466	60405537	C	405.2	1.85E-18	chr:11:60449890
11	rs1395403	60421492	A	427.6	2.34E-18	chr:11:60449890
11	rs1588034	60406972	A	359.4	4.40E-18	chr:11:60449890
11	rs1588035	60407020	A	359.4	4.40E-18	chr:11:60449890
11	rs76524444	60405169	C	359.4	4.40E-18	chr:11:60449890
11	rs4589322	60357024	T	101.1	4.61E-18	chr:11:60449890
11	rs12284014	60391203	G	115.2	9.98E-18	chr:11:60449890
11	rs184359733	60389486	C	115.2	9.98E-18	chr:11:60449890
11	rs475320	60394279	C	115.2	9.98E-18	chr:11:60449890
11	rs79718080	60379115	T	241.3	1.00E-17	chr:11:60449890
11	rs12271356	60379030	T	114.4	1.39E-17	chr:11:60449890
11	rs78827449	60338319	G	155.2	1.68E-17	chr:11:60449890
11	rs10897100	60345552	A	146.3	2.40E-17	chr:11:60449890
11	rs11230386	60333542	C	146.3	2.40E-17	chr:11:60449890
11	rs11230393	60346890	A	146.3	2.40E-17	chr:11:60449890
11	rs11230410	60358407	T	146.3	2.40E-17	chr:11:60449890
11	rs149005040	60349048	T	146.3	2.40E-17	chr:11:60449890
11	rs4939409	60331285	A	146.3	2.40E-17	chr:11:60449890
11	rs4939411	60331584	C	146.3	2.40E-17	chr:11:60449890

11	rs4939419	60363677	A	146.3	2.40E-17	chr:11:60449890
11	rs880517	60327093	C	146.3	2.40E-17	chr:11:60449890
11	rs11535815	60322719	C	135.4	2.51E-17	chr:11:60449890
11	rs12222676	60320591	T	135.4	2.51E-17	chr:11:60449890
11	rs148027662	60319226	C	135.4	2.51E-17	chr:11:60449890
11	rs4939401	60313437	T	135.4	2.51E-17	chr:11:60449890
11	rs11230416	60358917	T	128.3	5.64E-17	chr:11:60449890
11	rs112420146	60352340	A	128.3	5.64E-17	chr:11:60449890
11	rs79994929	60342896	A	128.3	5.64E-17	chr:11:60449890
11	rs80197220	60360315	T	128.3	5.64E-17	chr:11:60449890
11	rs12269769	60378488	G	63.44	1.45E-16	chr:11:60449890
11	rs117052092	60277975	T	65.96	1.76E-16	chr:11:60449890
11	rs78293538	60313053	T	106.3	1.86E-16	chr:11:60449890
11	rs1075193	60357882	G	117.9	1.92E-16	chr:11:60449890
11	rs10897099	60345446	A	117.9	1.92E-16	chr:11:60449890
11	rs112266959	60350831	T	117.9	1.92E-16	chr:11:60449890
11	rs11230389	60344066	A	117.9	1.92E-16	chr:11:60449890
11	rs11230390	60345907	C	117.9	1.92E-16	chr:11:60449890
11	rs11230392	60346342	T	117.9	1.92E-16	chr:11:60449890
11	rs11230395	60347928	C	117.9	1.92E-16	chr:11:60449890
11	rs11230396	60348289	C	117.9	1.92E-16	chr:11:60449890
11	rs11230398	60348665	A	117.9	1.92E-16	chr:11:60449890
11	rs11230401	60354064	T	117.9	1.92E-16	chr:11:60449890
11	rs11230402	60354355	G	117.9	1.92E-16	chr:11:60449890
11	rs11230404	60354440	T	117.9	1.92E-16	chr:11:60449890
11	rs11230405	60354446	G	117.9	1.92E-16	chr:11:60449890
11	rs11230406	60355032	G	117.9	1.92E-16	chr:11:60449890
11	rs11230407	60355215	T	117.9	1.92E-16	chr:11:60449890
11	rs11230408	60356188	T	117.9	1.92E-16	chr:11:60449890
11	rs11230409	60356955	C	117.9	1.92E-16	chr:11:60449890
11	rs11230411	60358502	C	117.9	1.92E-16	chr:11:60449890
11	rs11230412	60358517	G	117.9	1.92E-16	chr:11:60449890
11	rs11230413	60358600	G	117.9	1.92E-16	chr:11:60449890
11	rs11230414	60358669	C	117.9	1.92E-16	chr:11:60449890
11	rs11230415	60358744	T	117.9	1.92E-16	chr:11:60449890
11	rs113438455	60352321	C	117.9	1.92E-16	chr:11:60449890
11	rs12574473	60344427	C	117.9	1.92E-16	chr:11:60449890
11	rs12577546	60350014	T	117.9	1.92E-16	chr:11:60449890
11	rs192160182	60359598	A	117.9	1.92E-16	chr:11:60449890
11	rs4938959	60341383	C	117.9	1.92E-16	chr:11:60449890
11	rs4939414	60355673	C	117.9	1.92E-16	chr:11:60449890
11	rs75074202	60361575	G	117.9	1.92E-16	chr:11:60449890
11	rs75259753	60360747	C	117.9	1.92E-16	chr:11:60449890

11	rs75847459	60361128	A	117.9	1.92E-16	chr:11:60449890
11	rs76962588	60361563	G	117.9	1.92E-16	chr:11:60449890
11	rs77815918	60342648	G	117.9	1.92E-16	chr:11:60449890
11	rs73492997	60515364	T	28.09	2.44E-16	chr:11:60449890
11	rs11230417	60359982	T	112	2.54E-16	chr:11:60449890
11	rs11230420	60363961	A	116.3	2.79E-16	chr:11:60449890
11	rs55752280	60239957	T	14.78	4.96E-16	chr:11:60449890
11	rs7124085	60239723	G	14.78	4.96E-16	chr:11:60449890
11	rs7124717	60243714	C	14.78	4.96E-16	chr:11:60449890
11	rs60684765	60472310	C	30.55	1.38E-15	chr:11:60449890
11	rs10897102	60364051	G	56.83	1.95E-15	chr:11:60449890
11	rs111747950	60495986	G	23.34	3.53E-15	chr:11:60449890
11	rs17155019	60229677	T	20.76	6.41E-15	chr:11:60449890
11	rs78619545	60230270	T	20.76	6.41E-15	chr:11:60449890
11	rs1443249	60224044	G	18.61	9.96E-15	chr:11:60449890
11	rs74478918	60221936	A	18.61	9.96E-15	chr:11:60449890
11	rs79350662	60222269	G	18.61	9.96E-15	chr:11:60449890
11	rs1567083	60473821	T	13.68	1.46E-14	chr:11:60449890
11	rs10736709	60455094	C	13.88	1.48E-14	chr:11:60449890
11	rs10897107	60438001	T	13.88	1.48E-14	chr:11:60449890
11	rs474347	60394379	A	11.6	1.49E-14	chr:11:60449890
11	rs533273	60392597	G	11.6	1.49E-14	chr:11:60449890
11	rs556374	60395210	G	11.6	1.49E-14	chr:11:60449890
11	rs58686875	60389219	G	11.6	1.49E-14	chr:11:60449890
11	rs59782110	60388974	C	11.6	1.49E-14	chr:11:60449890
11	rs79794327	60408059	C	16.49	1.53E-14	chr:11:60449890
11	rs1532900	60486121	G	13.46	1.81E-14	chr:11:60449890
11	rs11230434	60379726	A	11.56	1.83E-14	chr:11:60449890
11	rs12274091	60375565	C	11.56	1.83E-14	chr:11:60449890
11	rs7119769	60372678	A	11.56	1.83E-14	chr:11:60449890
11	rs6591614	60419949	C	15.76	1.83E-14	chr:11:60449890
11	rs4939433	60412685	G	12.72	1.83E-14	chr:11:60449890
11	rs7128058	60409995	C	12.72	1.83E-14	chr:11:60449890
11	rs7130322	60414821	G	12.72	1.83E-14	chr:11:60449890
11	rs1588033	60406661	C	12.68	2.10E-14	chr:11:60449890
11	rs72643556	60397090	C	12.68	2.10E-14	chr:11:60449890
11	rs2087236	60411080	A	15.72	2.17E-14	chr:11:60449890
11	rs484858	60439530	C	16.57	2.31E-14	chr:11:60449890
11	rs518778	60440909	C	16.57	2.31E-14	chr:11:60449890
11	rs528002	60461496	A	16.36	2.64E-14	chr:11:60449890
11	rs664114	60470538	A	16.36	2.64E-14	chr:11:60449890
11	rs6591612	60407822	C	17.81	3.07E-14	chr:11:60449890
11	rs10897101	60347964	C	10.75	3.75E-14	chr:11:60449890

11	rs4939412	60346987	T	10.75	3.75E-14	chr:11:60449890
11	rs12222298	60289836	T	11.99	3.89E-14	chr:11:60449890
11	rs2868222	60344075	T	10.74	4.14E-14	chr:11:60449890
11	rs7113073	60478793	G	18.39	4.78E-14	chr:11:60449890
11	rs2306836	60482329	C	18.53	4.86E-14	chr:11:60449890
11	rs10897089	60316296	A	8.012	5.11E-14	chr:11:60449890
11	rs11494164	60316659	C	8.012	5.11E-14	chr:11:60449890
11	rs2509514	60318700	C	8.012	5.11E-14	chr:11:60449890
11	rs7948426	60314241	G	8.012	5.11E-14	chr:11:60449890
11	rs2186916	60332013	C	8.871	5.54E-14	chr:11:60449890
11	rs2847210	60337966	C	8.871	5.54E-14	chr:11:60449890
11	rs6591606	60351183	T	8.871	5.54E-14	chr:11:60449890
11	rs12278643	60352288	C	8.874	6.00E-14	chr:11:60449890
11	rs12285680	60361616	A	8.854	6.20E-14	chr:11:60449890
11	rs11230365	60298171	T	8.222	6.45E-14	chr:11:60449890
11	rs4121607	60306301	G	8.222	6.45E-14	chr:11:60449890
11	rs6591595	60291413	C	8.222	6.45E-14	chr:11:60449890
11	rs72643555	60280001	A	10.54	7.33E-14	chr:11:60449890
11	rs10750940	60282019	G	7.985	9.26E-14	chr:11:60449890
11	rs10897076	60278980	C	7.985	9.26E-14	chr:11:60449890
11	rs17155261	60511789	G	11.43	9.91E-14	chr:11:60449890
11	rs1941026	60312462	A	9.751	1.02E-13	chr:11:60449890
11	rs10897079	60290960	G	9.002	1.12E-13	chr:11:60449890
11	rs10897080	60294184	A	9.002	1.12E-13	chr:11:60449890
11	rs10897081	60295202	G	9.002	1.12E-13	chr:11:60449890
11	rs10897082	60295234	G	9.002	1.12E-13	chr:11:60449890
11	rs10897083	60299838	G	9.002	1.12E-13	chr:11:60449890
11	rs11230375	60306767	A	9.002	1.12E-13	chr:11:60449890
11	rs11230376	60306877	A	9.002	1.12E-13	chr:11:60449890
11	rs12282328	60289291	C	9.002	1.12E-13	chr:11:60449890
11	rs12284816	60291548	G	9.002	1.12E-13	chr:11:60449890
11	rs12286993	60289473	T	9.002	1.12E-13	chr:11:60449890
11	rs12578017	60299330	A	9.002	1.12E-13	chr:11:60449890
11	rs2077468	60308202	T	9.002	1.12E-13	chr:11:60449890
11	rs4938952	60290348	T	9.002	1.12E-13	chr:11:60449890
11	rs4938953	60295986	T	9.002	1.12E-13	chr:11:60449890
11	rs4938954	60298660	T	9.002	1.12E-13	chr:11:60449890
11	rs4938956	60299129	T	9.002	1.12E-13	chr:11:60449890
11	rs4939393	60290352	C	9.002	1.12E-13	chr:11:60449890
11	rs4939395	60298443	C	9.002	1.12E-13	chr:11:60449890
11	rs4939396	60298541	C	9.002	1.12E-13	chr:11:60449890
11	rs4939397	60298564	T	9.002	1.12E-13	chr:11:60449890
11	rs4939399	60298745	G	9.002	1.12E-13	chr:11:60449890

11	rs750939	60308039	C	9.002	1.12E-13	chr:11:60449890
11	rs4939391	60290223	T	8.989	1.28E-13	chr:11:60449890
11	rs12277712	60282204	T	8.298	1.32E-13	chr:11:60449890
11	rs4939388	60280204	G	8.298	1.32E-13	chr:11:60449890
11	rs59358103	60279328	T	8.298	1.32E-13	chr:11:60449890
11	rs66506129	60279921	T	8.298	1.32E-13	chr:11:60449890
11	rs766111	60280875	A	8.298	1.32E-13	chr:11:60449890
11	rs10897109	60518182	T	7.548	1.42E-13	chr:11:60449890
11	rs75655826	60516738	T	9.488	1.43E-13	chr:11:60449890
11	rs10897086	60309071	G	8.938	1.50E-13	chr:11:60449890
11	rs11230358	60292172	G	8.325	1.53E-13	chr:11:60449890
11	rs7934771	60286038	A	7.372	1.54E-13	chr:11:60449890
11	rs10897078	60285528	G	7.849	2.54E-13	chr:11:60449890
11	rs11230355	60284522	G	7.849	2.54E-13	chr:11:60449890
11	rs7110684	60500843	C	23.48	2.71E-13	chr:11:60449890
11	rs113789171	60481588	G	23.27	4.24E-13	chr:11:60449890
11	rs61898830	60515774	G	7.793	6.27E-13	chr:11:60449890
11	rs7948556	60517887	G	7.53	2.15E-12	chr:11:60449890
11	rs575559715	60301959	T	39.07	5.68E-12	chr:11:60449890
11	rs549412940	60303581	G	8.836	1.58E-11	chr:11:60449890
11	rs116954371	60484942	A	2269	3.45E-11	chr:11:60449890
11	rs149904169	60218249	A	10.95	7.56E-11	chr:11:60449890
11	rs150164831	60214698	T	10.95	7.56E-11	chr:11:60449890
11	rs77182118	60214076	A	10.95	7.56E-11	chr:11:60449890
11	rs77831294	60216599	T	10.95	7.56E-11	chr:11:60449890
11	rs372464052	60218891	G	10.5	1.14E-10	chr:11:60449890
11	rs76907727	60215737	C	10.44	1.18E-10	chr:11:60449890
11	rs576832473	60219175	C	10.12	1.33E-10	chr:11:60449890
11	rs113897778	60461655	C	984.5	1.69E-10	chr:11:60449890
11	rs11822213	60452469	C	984.5	1.69E-10	chr:11:60449890
11	rs4938975	60465691	A	984.5	1.69E-10	chr:11:60449890
11	rs6591617	60443177	A	984.5	1.69E-10	chr:11:60449890
11	rs78504056	60464475	G	984.5	1.69E-10	chr:11:60449890
11	rs74973858	60473529	T	946.1	2.16E-10	chr:11:60449890
11	rs17155222	60467175	G	890.6	2.17E-10	chr:11:60449890
11	rs74588793	60479991	G	917.8	2.22E-10	chr:11:60449890
11	rs191364573	60430925	G	28.74	8.55E-10	chr:11:60449890
11	rs148096621	60460770	G	596.7	1.42E-09	chr:11:60449890
11	rs573441235	60515380	T	7.301	2.30E-09	chr:11:60449890
11	rs111523940	60472332	C	368.3	1.17E-08	chr:11:60449890
11	rs75851712	60535363	A	5.168	3.87E-08	chr:11:60449890
11	rs532683805	60241237	T	20.3	7.35E-08	chr:11:60449890
11	rs4534600	60203177	A	7.799	7.42E-08	chr:11:60449890

11	rs75272964	60277488	T	0.2742	7.96E-08	chr:11:60449890
11	rs12417644	60277063	C	0.2742	1.08E-07	chr:11:60449890
11	rs143837385	60178507	T	7.301	1.10E-07	chr:11:60449890
11	rs1443243	60184840	C	7.301	1.10E-07	chr:11:60449890
11	rs145918292	60180189	A	7.301	1.10E-07	chr:11:60449890
11	rs17154981	60201102	A	7.301	1.10E-07	chr:11:60449890
11	rs4939355	60182571	G	7.301	1.10E-07	chr:11:60449890
11	rs4939356	60189627	C	7.301	1.10E-07	chr:11:60449890
11	rs7935330	60185334	C	7.301	1.10E-07	chr:11:60449890
11	rs150289543	60210476	C	6.625	1.21E-07	chr:11:60449890
11	rs57765637	60320019	T	0.2675	1.40E-07	chr:11:60449890
11	rs78443804	60322247	G	0.2675	1.40E-07	chr:11:60449890
11	rs11230450	60470119	A	0.223	1.43E-07	chr:11:60449890
11	rs1224311	60417361	A	0.2259	1.55E-07	chr:11:60449890
11	rs523318	60414669	T	0.2259	1.55E-07	chr:11:60449890
11	rs12362061	60446715	C	0.2232	1.64E-07	chr:11:60449890
11	rs1848089	60429426	G	0.2232	1.64E-07	chr:11:60449890
11	rs11230454	60484893	A	0.2261	1.75E-07	chr:11:60449890
11	rs111330485	60810157	C	4.923	1.79E-07	chr:11:60449890
11	rs2233247	60154167	C	6.324	1.80E-07	chr:11:60449890
11	rs2233255	60160423	C	6.324	1.80E-07	chr:11:60449890
11	rs72472148	60157500	C	6.324	1.80E-07	chr:11:60449890
11	rs74693377	60162233	T	6.324	1.80E-07	chr:11:60449890
11	rs75408079	60162813	A	6.324	1.80E-07	chr:11:60449890
11	rs76402001	60151580	G	6.324	1.80E-07	chr:11:60449890
11	rs77256998	60155557	C	6.324	1.80E-07	chr:11:60449890
11	rs78824219	60158249	G	6.324	1.80E-07	chr:11:60449890
11	rs80196453	60158841	T	6.324	1.80E-07	chr:11:60449890
11	rs141950944	60151021	C	6.085	2.36E-07	chr:11:60449890
11	rs12420937	60323768	C	0.2723	2.37E-07	chr:11:60449890
11	rs75538311	60185044	C	6.461	2.50E-07	chr:11:60449890
11	rs76274884	60196877	T	6.461	2.50E-07	chr:11:60449890
11	rs77626314	60191284	G	6.461	2.50E-07	chr:11:60449890
11	rs534362559	60460600	C	16.55	2.51E-07	chr:11:60449890
11	rs139686528	60172693	T	6.065	2.58E-07	chr:11:60449890
11	rs75332833	60165502	A	6.065	2.58E-07	chr:11:60449890
11	rs76943117	60165816	A	6.065	2.58E-07	chr:11:60449890
11	rs4939449	60520624	G	3.426	2.64E-07	chr:11:60449890
11	rs482386	60400154	C	0.2383	2.92E-07	chr:11:60449890
11	rs716562	60312456	A	0.2865	3.22E-07	chr:11:60449890
11	rs2226681	60311515	T	0.2862	3.28E-07	chr:11:60449890
11	rs116841458	60148566	T	5.825	3.43E-07	chr:11:60449890
11	rs117655631	60133639	A	5.825	3.43E-07	chr:11:60449890

11	rs117961609	60148570	T	5.825	3.43E-07	chr:11:60449890
11	rs138775550	60143481	T	5.825	3.43E-07	chr:11:60449890
11	rs146149101	60147398	A	5.825	3.43E-07	chr:11:60449890
11	rs17154830	60122229	A	5.825	3.43E-07	chr:11:60449890
11	rs2017738	60110746	T	5.825	3.43E-07	chr:11:60449890
11	rs4938938	60119633	C	5.825	3.43E-07	chr:11:60449890
11	rs4939341	60111430	A	5.825	3.43E-07	chr:11:60449890
11	rs4939351	60139906	A	5.825	3.43E-07	chr:11:60449890
11	rs7112972	60139846	T	5.825	3.43E-07	chr:11:60449890
11	rs74603923	60141495	A	5.825	3.43E-07	chr:11:60449890
11	rs74836746	60147294	T	5.825	3.43E-07	chr:11:60449890
11	rs75301877	60149538	G	5.825	3.43E-07	chr:11:60449890
11	rs75443562	60144484	T	5.825	3.43E-07	chr:11:60449890
11	rs75812155	60126923	G	5.825	3.43E-07	chr:11:60449890
11	rs77833123	60128200	A	5.825	3.43E-07	chr:11:60449890
11	rs77904860	60115695	T	5.825	3.43E-07	chr:11:60449890
11	rs78474912	60127722	A	5.825	3.43E-07	chr:11:60449890
11	rs78843981	60146055	A	5.825	3.43E-07	chr:11:60449890
11	rs79926162	60132686	G	5.825	3.43E-07	chr:11:60449890
11	rs962813	60110542	A	5.825	3.43E-07	chr:11:60449890
11	rs17154674	60051768	C	3.956	3.82E-07	chr:11:60449890
11	rs556448	60395181	G	0.239	4.05E-07	chr:11:60449890
11	rs12417095	60295245	A	0.288	4.13E-07	chr:11:60449890
11	rs12419383	60289988	G	0.288	4.13E-07	chr:11:60449890
11	rs7105958	60289057	G	0.288	4.13E-07	chr:11:60449890
11	rs112073301	60371040	G	0.2387	4.27E-07	chr:11:60449890
11	rs61900812	60322946	A	0.2783	4.27E-07	chr:11:60449890
11	rs17154846	60126725	C	5.613	4.54E-07	chr:11:60449890
11	rs76757698	60148991	A	5.559	4.90E-07	chr:11:60449890
11	rs11230283	60112476	T	5.574	4.93E-07	chr:11:60449890
11	rs4938940	60169827	T	5.087	4.99E-07	chr:11:60449890
11	rs1552474	60388078	A	0.2426	5.00E-07	chr:11:60449890
11	rs61900774	60299652	T	0.2923	5.29E-07	chr:11:60449890
11	rs11230424	60368782	C	0.2425	5.33E-07	chr:11:60449890
11	rs1238233	60392174	T	0.2425	5.33E-07	chr:11:60449890
11	rs1994457	60387600	G	0.2425	5.33E-07	chr:11:60449890
11	rs7479522	60390389	A	0.2425	5.33E-07	chr:11:60449890
11	rs78540235	60389656	T	0.2425	5.33E-07	chr:11:60449890
11	rs7933702	60364916	C	0.2425	5.33E-07	chr:11:60449890
11	rs2054851	60380272	T	0.2424	5.59E-07	chr:11:60449890
11	rs75627175	60005825	C	3.746	5.91E-07	chr:11:60449890
11	rs6591572	60115559	A	4.717	7.09E-07	chr:11:60449890
11	rs766112	60280544	C	0.3059	8.79E-07	chr:11:60449890

11	rs2299651	60757542	T	3.39	9.23E-07	chr:11:60449890
11	rs540028	60836408	A	0.2805	1.04E-06	chr:11:60449890
11	rs12421597	60505846	A	0.2409	1.04E-06	chr:11:60449890
11	rs11230378	60310083	T	0.3017	1.06E-06	chr:11:60449890
11	rs11230394	60346932	G	0.2556	1.23E-06	chr:11:60449890
11	rs4614472	60336949	C	0.2556	1.23E-06	chr:11:60449890
11	rs59379022	60328417	C	0.2556	1.23E-06	chr:11:60449890
11	rs190728254	60025977	T	5.305	1.29E-06	chr:11:60449890
11	rs1373061	60125647	A	4.569	1.29E-06	chr:11:60449890
11	rs1373062	60125591	A	4.569	1.29E-06	chr:11:60449890
11	rs1899573	60123837	T	4.569	1.29E-06	chr:11:60449890
11	rs2034080	60121869	T	4.569	1.29E-06	chr:11:60449890
11	rs2034081	60121670	C	4.569	1.29E-06	chr:11:60449890
11	rs7945371	60308128	A	0.3066	1.36E-06	chr:11:60449890
11	rs507550	60804962	A	3.351	1.40E-06	chr:11:60449890
11	rs4938977	60521424	A	0.3163	1.41E-06	chr:11:60449890
11	rs11230548	60751569	A	3.253	1.93E-06	chr:11:60449890
11	rs187852876	60051142	C	5.242	2.19E-06	chr:11:60449890
11	rs185807105	59989832	G	5.06	2.32E-06	chr:11:60449890
11	rs509936	60858031	G	0.3379	2.54E-06	chr:11:60449890
11	rs55929020	60391817	T	0.2749	2.73E-06	chr:11:60449890
11	rs59497469	60390801	C	0.2749	2.73E-06	chr:11:60449890
11	rs7937466	60389305	A	0.2749	2.73E-06	chr:11:60449890
11	rs61900768	60283627	G	0.32	2.89E-06	chr:11:60449890
11	rs7482102	60390467	A	0.2749	3.00E-06	chr:11:60449890
11	rs199581697	60369457	T	0.2956	3.15E-06	chr:11:60449890
11	rs2054849	60364127	C	0.2779	3.26E-06	chr:11:60449890
11	rs56183826	60368846	A	0.2779	3.26E-06	chr:11:60449890
11	rs61898779	60371542	T	0.2779	3.26E-06	chr:11:60449890
11	rs12421253	60889474	C	7.838	3.99E-06	chr:11:60449890
11	rs12418015	60343295	A	0.2742	4.03E-06	chr:11:60449890
11	rs12274840	60275870	G	0.3583	4.34E-06	chr:11:60449890
11	rs61898795	60441783	C	0.2891	4.92E-06	chr:11:60449890
11	rs612342	60495124	T	0.2895	5.40E-06	chr:11:60449890
11	rs2509511	60335761	A	0.2735	5.91E-06	chr:11:60449890
11	rs2633279	60337668	A	0.2735	5.91E-06	chr:11:60449890
11	rs2847216	60332894	A	0.2735	5.91E-06	chr:11:60449890
11	rs1050922	60785352	G	0.3124	5.93E-06	chr:11:60449890
11	rs2074232	60785038	G	0.3124	5.93E-06	chr:11:60449890
11	rs485303	60590145	A	0.3427	5.93E-06	chr:11:60449890
11	rs520569	60590947	G	0.3427	5.93E-06	chr:11:60449890
11	rs11604104	60257301	G	0.3099	6.08E-06	chr:11:60449890
11	rs4939366	60255867	C	0.3099	6.08E-06	chr:11:60449890

11	rs4939368	60256048	A	0.3099	6.08E-06	chr:11:60449890
11	rs4939370	60257400	C	0.3099	6.08E-06	chr:11:60449890
11	rs55820209	60256974	G	0.3099	6.08E-06	chr:11:60449890
11	rs55924472	60254603	A	0.3099	6.08E-06	chr:11:60449890
11	rs150826565	60389460	T	0.2907	7.21E-06	chr:11:60449890
11	rs199639640	60259428	G	0.3141	7.26E-06	chr:11:60449890
11	rs7951049	60262342	G	0.3141	7.26E-06	chr:11:60449890
11	rs145490027	59956241	A	3.228	7.89E-06	chr:11:60449890
11	rs6591611	60405172	C	0.3036	8.60E-06	chr:11:60449890
11	rs2051770	60804710	A	2.963	9.17E-06	chr:11:60449890
11	rs999297	60801310	A	2.963	9.17E-06	chr:11:60449890
11	rs4939372	60257801	C	0.3173	9.27E-06	chr:11:60449890
11	rs11230344	60264409	G	0.3221	9.65E-06	chr:11:60449890
11	rs3019558	60784275	G	0.3222	1.06E-05	chr:11:60449890
11	rs188388424	60259290	C	0.3165	1.19E-05	chr:11:60449890
11	rs191577916	60259291	C	0.3165	1.19E-05	chr:11:60449890
11	rs11230388	60340422	A	0.2983	1.24E-05	chr:11:60449890
11	rs113104783	60348915	C	0.2983	1.24E-05	chr:11:60449890
11	rs612415	60859886	T	0.3595	1.30E-05	chr:11:60449890
11	rs3016171	60803588	G	0.3448	1.34E-05	chr:11:60449890
11	rs202037444	60258570	G	0.3228	1.38E-05	chr:11:60449890
11	rs2000636	60220947	A	2.461	1.41E-05	chr:11:60449890
11	rs7944716	60579522	A	0.3604	1.41E-05	chr:11:60449890
11	rs12576721	60442427	T	0.3129	1.51E-05	chr:11:60449890
11	rs4939446	60465161	T	0.3129	1.51E-05	chr:11:60449890
11	rs2298559	60274640	T	0.3439	1.53E-05	chr:11:60449890
11	rs2298558	60274393	G	0.3457	1.58E-05	chr:11:60449890
11	rs61898791	60400024	T	0.3067	1.60E-05	chr:11:60449890
11	rs146548256	60259218	T	0.3195	1.60E-05	chr:11:60449890
11	rs1080719	60422891	A	0.3069	1.65E-05	chr:11:60449890
11	rs4939383	60272374	G	0.323	1.68E-05	chr:11:60449890
11	rs117239525	60755203	C	0.337	1.75E-05	chr:11:60449890
11	rs138993612	60755200	T	0.337	1.75E-05	chr:11:60449890
11	rs141668624	60755201	T	0.337	1.75E-05	chr:11:60449890
11	rs151039602	60755202	T	0.337	1.75E-05	chr:11:60449890
11	rs2905510	60757857	C	0.337	1.75E-05	chr:11:60449890
11	rs3019538	60756175	G	0.337	1.75E-05	chr:11:60449890
11	rs75152709	60755197	C	0.337	1.75E-05	chr:11:60449890
11	rs78689918	60755196	T	0.337	1.75E-05	chr:11:60449890
11	rs61198976	60538309	A	2.575	1.81E-05	chr:11:60449890
11	rs113289170	60259320	A	0.3234	1.86E-05	chr:11:60449890
11	rs10897105	60431482	C	0.2634	1.88E-05	chr:11:60449890
11	rs11533252	60439134	G	0.2634	1.88E-05	chr:11:60449890

11	rs8181503	60264303	C	0.3208	1.91E-05	chr:11:60449890
11	rs10897069	60250742	A	0.3237	1.91E-05	chr:11:60449890
11	rs10897072	60258082	A	0.3237	2.00E-05	chr:11:60449890
11	rs10897073	60258169	C	0.3237	2.00E-05	chr:11:60449890
11	rs4939367	60255890	A	0.3237	2.00E-05	chr:11:60449890
11	rs4939369	60257335	A	0.3237	2.00E-05	chr:11:60449890
11	rs4939371	60257542	T	0.3237	2.00E-05	chr:11:60449890
11	rs56077595	60254796	A	0.3237	2.00E-05	chr:11:60449890
11	rs7937986	60256130	T	0.3237	2.00E-05	chr:11:60449890
11	rs9667216	60259689	G	0.3232	2.03E-05	chr:11:60449890
11	rs4471445	60232838	A	0.3249	2.13E-05	chr:11:60449890
11	rs4633490	60232766	T	0.3249	2.13E-05	chr:11:60449890
11	rs112292723	60258868	C	0.3277	2.20E-05	chr:11:60449890
11	rs113783397	60258658	T	0.3277	2.20E-05	chr:11:60449890
11	rs4394855	60262591	A	0.3488	2.21E-05	chr:11:60449890
11	rs17627788	60384540	A	0.3209	2.24E-05	chr:11:60449890
11	rs1506682	60549966	A	2.538	2.24E-05	chr:11:60449890
11	rs2089343	60547604	A	2.538	2.24E-05	chr:11:60449890
11	rs2905507	60751277	G	0.3429	2.24E-05	chr:11:60449890
11	rs112740539	60259113	G	0.3287	2.34E-05	chr:11:60449890
11	rs11230343	60262251	C	0.328	2.37E-05	chr:11:60449890
11	rs11604619	60261376	A	0.328	2.37E-05	chr:11:60449890
11	rs140618814	60258923	C	0.328	2.37E-05	chr:11:60449890
11	rs200899867	60258569	T	0.328	2.37E-05	chr:11:60449890
11	rs4939374	60261732	G	0.328	2.37E-05	chr:11:60449890
11	rs4939375	60261738	T	0.328	2.37E-05	chr:11:60449890
11	rs4939376	60261906	C	0.328	2.37E-05	chr:11:60449890
11	rs4939377	60262172	A	0.328	2.37E-05	chr:11:60449890
11	rs6591592	60260570	T	0.328	2.37E-05	chr:11:60449890
11	rs6591593	60262382	C	0.328	2.37E-05	chr:11:60449890
11	rs7941935	60263157	A	0.328	2.37E-05	chr:11:60449890
11	rs7942553	60259981	G	0.328	2.37E-05	chr:11:60449890
11	rs7942906	60260318	G	0.328	2.37E-05	chr:11:60449890
11	rs7951316	60262627	G	0.328	2.37E-05	chr:11:60449890
11	rs9666114	60259845	C	0.328	2.37E-05	chr:11:60449890
11	rs1941032	60247925	G	0.3285	2.46E-05	chr:11:60449890
11	rs58707312	60254591	G	0.3285	2.46E-05	chr:11:60449890
11	rs7396755	60240699	A	0.3285	2.46E-05	chr:11:60449890
11	rs75805701	60141605	T	3.591	2.51E-05	chr:11:60449890
11	rs4939373	60257821	G	0.3276	2.51E-05	chr:11:60449890
11	rs139608122	60562904	T	2.915	2.58E-05	chr:11:60449890
11	rs151164269	60562903	C	2.915	2.58E-05	chr:11:60449890
11	rs2298551	60264755	G	0.3197	2.58E-05	chr:11:60449890

11	rs111971056	60258633	A	0.3325	2.69E-05	chr:11:60449890
11	rs145082841	60566525	C	2.903	2.73E-05	chr:11:60449890
11	rs4939392	60290257	C	0.3519	2.78E-05	chr:11:60449890
11	rs9666659	60259733	A	0.3328	2.89E-05	chr:11:60449890
11	rs34752191	60262644	T	0.3414	3.21E-05	chr:11:60449890
12	rs78072538	124074848	T	48.04	2.02E-12	chr:12:124066477
12	rs117887064	124074320	T	35.58	5.76E-12	chr:12:124066477
12	rs137909390	124078791	A	35.58	5.76E-12	chr:12:124066477
12	rs141542281	124075268	A	35.58	5.76E-12	chr:12:124066477
12	rs34099549	124085062	A	35.58	5.76E-12	chr:12:124066477
12	rs146502362	124041729	C	42.44	7.58E-12	chr:12:124066477
12	rs77699179	124092437	A	34.33	8.64E-12	chr:12:124066477
12	rs111516966	124035781	A	40.65	1.06E-11	chr:12:124066477
12	rs11615043	124056715	C	33.37	1.14E-11	chr:12:124066477
12	rs143130669	124046424	T	33.37	1.14E-11	chr:12:124066477
12	rs188848881	124042516	A	33.37	1.14E-11	chr:12:124066477
12	rs74844896	124068924	C	33.37	1.14E-11	chr:12:124066477
12	rs76100851	124067280	T	33.37	1.14E-11	chr:12:124066477
12	rs78067009	124019231	T	27.51	2.16E-11	chr:12:124066477
12	rs111790379	124022967	T	38.36	2.17E-11	chr:12:124066477
12	rs145594864	124024519	A	38.36	2.17E-11	chr:12:124066477
12	rs28691687	124023020	T	38.36	2.17E-11	chr:12:124066477
12	rs6488879	124027372	T	38.36	2.17E-11	chr:12:124066477
12	rs10082874	124051951	A	26.64	3.61E-11	chr:12:124066477
12	rs2458449	124053767	C	26.64	3.61E-11	chr:12:124066477
12	rs786455	124054766	T	26.64	3.61E-11	chr:12:124066477
12	rs11057311	124121429	T	41.54	6.21E-11	chr:12:124066477
12	rs11572966	124134802	T	41.54	6.21E-11	chr:12:124066477
12	rs10846527	124043296	C	35.03	6.66E-11	chr:12:124066477
12	rs79370578	124007431	T	23.6	1.35E-10	chr:12:124066477
12	rs2048614	124011429	T	21.47	1.71E-10	chr:12:124066477
12	rs28361405	124012714	A	21.47	1.71E-10	chr:12:124066477
12	rs147460725	124038535	T	28.36	1.86E-10	chr:12:124066477
12	rs28880346	124038995	C	28.36	1.86E-10	chr:12:124066477
12	rs118111778	124083600	T	164.8	6.45E-10	chr:12:124066477
12	rs117209788	124014251	T	22.51	8.53E-10	chr:12:124066477
12	rs143928387	124013220	T	22.51	8.53E-10	chr:12:124066477
12	rs883503	124008897	C	16.77	1.00E-09	chr:12:124066477
12	rs786426	124086086	C	17.17	1.25E-09	chr:12:124066477
12	rs884892	124097528	G	61.93	3.79E-09	chr:12:124066477
12	rs530657685	124084061	G	18.93	6.72E-09	chr:12:124066477
12	rs7316613	124045580	T	28.35	6.78E-09	chr:12:124066477
12	rs112002563	124077902	T	19.82	7.09E-09	chr:12:124066477

12	rs9697379	124005129	A	14.85	8.69E-09	chr:12:124066477
12	rs3748268	124097824	G	57.61	1.06E-08	chr:12:124066477
12	rs61953538	124005708	T	13.43	1.11E-08	chr:12:124066477
12	rs10846531	124098612	G	11.82	1.27E-08	chr:12:124066477
12	rs11057298	124057112	A	86	1.54E-08	chr:12:124066477
12	rs4930715	124042949	C	39.5	1.58E-08	chr:12:124066477
12	rs28454032	124034703	G	24.78	2.53E-08	chr:12:124066477
12	rs5027860	124032819	G	24.78	2.53E-08	chr:12:124066477
12	rs542038648	124086080	A	13.88	2.63E-08	chr:12:124066477
12	rs7307227	124020098	T	12.46	2.72E-08	chr:12:124066477
12	rs2048802	124098780	T	10.94	2.95E-08	chr:12:124066477
12	rs7965466	124036188	C	71	4.13E-08	chr:12:124066477
12	rs7314792	124036984	G	69.74	4.68E-08	chr:12:124066477
12	rs11057302	124070013	A	429.6	6.09E-08	chr:12:124066477
12	rs111507681	124088418	T	429.6	6.09E-08	chr:12:124066477
12	rs143269373	124071864	A	429.6	6.09E-08	chr:12:124066477
12	rs146539578	124071144	T	429.6	6.09E-08	chr:12:124066477
12	rs146936648	124074744	G	429.6	6.09E-08	chr:12:124066477
12	rs7301377	124076254	T	429.6	6.09E-08	chr:12:124066477
12	rs7303554	124079920	G	429.6	6.09E-08	chr:12:124066477
12	rs74643748	124075915	T	429.6	6.09E-08	chr:12:124066477
12	rs74996347	124086528	G	429.6	6.09E-08	chr:12:124066477
12	rs77592647	124084160	G	429.6	6.09E-08	chr:12:124066477
12	rs77895354	124075111	G	429.6	6.09E-08	chr:12:124066477
12	rs7952920	124085011	T	429.6	6.09E-08	chr:12:124066477
12	rs7970545	124085080	G	429.6	6.09E-08	chr:12:124066477
12	rs79933835	124073911	T	429.6	6.09E-08	chr:12:124066477
12	rs952360	124076806	C	429.6	6.09E-08	chr:12:124066477
12	rs71458805	124017254	G	8.985	6.20E-08	chr:12:124066477
12	rs7965334	124036026	C	35.28	6.32E-08	chr:12:124066477
12	rs28673982	124016464	C	10.74	6.42E-08	chr:12:124066477
12	rs7315326	124033885	G	6.979	6.72E-08	chr:12:124066477
12	rs2292500	124008073	A	10.69	6.87E-08	chr:12:124066477
12	rs2063629	124006842	C	10.6	8.06E-08	chr:12:124066477
12	rs9723048	124006318	G	10.6	8.06E-08	chr:12:124066477
12	rs111625397	124058009	A	395.3	8.34E-08	chr:12:124066477
12	rs11610013	124069614	A	395.3	8.34E-08	chr:12:124066477
12	rs11834369	124059546	T	395.3	8.34E-08	chr:12:124066477
12	rs11837236	124065116	G	395.3	8.34E-08	chr:12:124066477
12	rs12099986	124065978	G	395.3	8.34E-08	chr:12:124066477
12	rs138708987	124058169	C	395.3	8.34E-08	chr:12:124066477
12	rs1877365	124061397	T	395.3	8.34E-08	chr:12:124066477
12	rs2340691	124061421	T	395.3	8.34E-08	chr:12:124066477

12	rs78955489	124068910	A	395.3	8.34E-08	chr:12:124066477
12	rs7954782	124064537	G	395.3	8.34E-08	chr:12:124066477
12	rs7958153	124064998	G	395.3	8.34E-08	chr:12:124066477
12	rs7960812	124062742	T	395.3	8.34E-08	chr:12:124066477
12	rs7968040	124058937	A	395.3	8.34E-08	chr:12:124066477
12	rs7971779	124064918	T	395.3	8.34E-08	chr:12:124066477
12	rs10846528	124052985	A	7.184	8.96E-08	chr:12:124066477
12	rs12099981	124065428	C	390.5	9.92E-08	chr:12:124066477
12	rs112455213	124055507	A	379.3	1.00E-07	chr:12:124066477
12	rs116716383	124055605	G	379.3	1.00E-07	chr:12:124066477
12	rs6488881	124056441	G	379.3	1.00E-07	chr:12:124066477
12	rs7136101	124056685	A	379.3	1.00E-07	chr:12:124066477
12	rs61678733	124094174	C	323.7	1.09E-07	chr:12:124066477
12	rs73414262	124093859	G	323.7	1.09E-07	chr:12:124066477
12	rs786428	124086998	C	323.7	1.09E-07	chr:12:124066477
12	rs786429	124087394	T	323.7	1.09E-07	chr:12:124066477
12	rs786430	124087988	G	323.7	1.09E-07	chr:12:124066477
12	rs786431	124088366	T	323.7	1.09E-07	chr:12:124066477
12	rs786432	124088532	T	323.7	1.09E-07	chr:12:124066477
12	rs786433	124089582	A	323.7	1.09E-07	chr:12:124066477
12	rs786434	124089793	T	323.7	1.09E-07	chr:12:124066477
12	rs786435	124090208	G	323.7	1.09E-07	chr:12:124066477
12	rs786437	124090814	G	323.7	1.09E-07	chr:12:124066477
12	rs786438	124091218	A	323.7	1.09E-07	chr:12:124066477
12	rs786439	124091460	G	323.7	1.09E-07	chr:12:124066477
12	rs786450	124071765	C	323.7	1.09E-07	chr:12:124066477
12	rs28834793	124040064	G	6.406	1.19E-07	chr:12:124066477
12	rs28711456	124012763	T	8.606	1.21E-07	chr:12:124066477
12	rs7313179	124009190	T	8.606	1.21E-07	chr:12:124066477
12	rs7959727	124022758	G	6.924	1.21E-07	chr:12:124066477
12	rs4930716	124013306	A	7.989	1.23E-07	chr:12:124066477
12	rs2458115	124053902	T	6.375	1.36E-07	chr:12:124066477
12	rs12817861	124046540	T	6.367	1.38E-07	chr:12:124066477
12	rs2048616	124049966	G	6.375	1.41E-07	chr:12:124066477
12	rs11057306	124097766	G	314.3	1.71E-07	chr:12:124066477
12	rs4310694	124050760	A	6.08	3.24E-07	chr:12:124066477
12	rs786454	124055358	C	5.157	6.53E-07	chr:12:124066477
12	rs7305701	124150013	C	5.567	6.71E-07	chr:12:124066477
12	rs34996665	124134656	T	5.559	1.02E-06	chr:12:124066477
12	rs786452	124057169	C	5.387	1.13E-06	chr:12:124066477
12	rs10744161	124231355	A	4.797	1.23E-06	chr:12:124066477
12	rs11615300	124100423	G	4.991	1.29E-06	chr:12:124066477
12	rs7301065	124154838	A	5.07	1.53E-06	chr:12:124066477

12	rs7295552	124223844	T	4.847	1.57E-06	chr:12:124066477
12	rs35158437	124160764	T	5.074	1.63E-06	chr:12:124066477
12	rs7133199	124159185	C	5.074	1.63E-06	chr:12:124066477
12	rs7134074	124159885	T	5.074	1.63E-06	chr:12:124066477
12	rs7313140	124157188	G	5.074	1.63E-06	chr:12:124066477
12	rs7313261	124166937	A	5.074	1.63E-06	chr:12:124066477
12	rs1051793	124144395	G	5.039	1.64E-06	chr:12:124066477
12	rs10846537	124138346	T	5.039	1.64E-06	chr:12:124066477
12	rs4930708	124145697	C	5.039	1.64E-06	chr:12:124066477
12	rs11610473	124149975	G	5.112	1.74E-06	chr:12:124066477
12	rs11834141	124150696	C	4.972	2.09E-06	chr:12:124066477
12	rs7296804	124153863	C	4.972	2.09E-06	chr:12:124066477
12	rs28752288	124020681	A	6.147	2.21E-06	chr:12:124066477
12	rs11612479	124155526	G	4.971	2.23E-06	chr:12:124066477
12	rs7398082	124225073	A	4.62	2.31E-06	chr:12:124066477
12	rs12580486	124224335	T	4.612	2.39E-06	chr:12:124066477
12	rs7308398	124226800	T	4.612	2.39E-06	chr:12:124066477
12	rs7399128	124225211	G	4.612	2.39E-06	chr:12:124066477
12	rs7135660	124174234	A	4.745	2.68E-06	chr:12:124066477
12	rs66647364	124197280	C	4.881	2.73E-06	chr:12:124066477
12	rs7960951	124165829	A	4.728	2.79E-06	chr:12:124066477
12	rs147639128	124176516	G	14.5	2.85E-06	chr:12:124066477
12	rs74936044	124177506	C	14.5	2.85E-06	chr:12:124066477
12	rs10773031	124222909	G	4.559	3.02E-06	chr:12:124066477
12	rs11829958	124173005	G	4.709	3.23E-06	chr:12:124066477
12	rs12825800	124193666	C	4.709	3.23E-06	chr:12:124066477
12	rs12828633	124179620	A	4.709	3.23E-06	chr:12:124066477
12	rs35114195	124176013	C	4.709	3.23E-06	chr:12:124066477
12	rs6488897	124195483	C	4.709	3.23E-06	chr:12:124066477
12	rs7138503	124183827	G	4.709	3.23E-06	chr:12:124066477
12	rs7298831	124172555	T	4.709	3.23E-06	chr:12:124066477
12	rs7306549	124156897	G	4.709	3.23E-06	chr:12:124066477
12	rs7964443	124166500	C	4.709	3.23E-06	chr:12:124066477
12	rs28478308	124017569	A	4.516	3.25E-06	chr:12:124066477
12	rs28662057	124017568	A	4.516	3.25E-06	chr:12:124066477
12	rs35649708	124151439	G	4.68	3.45E-06	chr:12:124066477
12	rs11609591	124174065	A	4.707	3.48E-06	chr:12:124066477
12	rs11614659	124167956	T	4.707	3.48E-06	chr:12:124066477
12	rs12819746	124193574	G	4.707	3.48E-06	chr:12:124066477
12	rs7297576	124171323	C	4.707	3.48E-06	chr:12:124066477
12	rs7308862	124171151	T	4.707	3.48E-06	chr:12:124066477
12	rs7313032	124172731	A	4.707	3.48E-06	chr:12:124066477
12	rs7958693	124162750	A	4.707	3.48E-06	chr:12:124066477

12	rs7974279	124166411	T	4.707	3.48E-06	chr:12:124066477
12	rs2222060	124194438	G	4.691	3.68E-06	chr:12:124066477
12	rs10846549	124222687	C	4.39	3.87E-06	chr:12:124066477
12	rs10846550	124222695	T	4.384	3.99E-06	chr:12:124066477
12	rs786441	124062743	G	4.508	4.10E-06	chr:12:124066477
12	rs11611354	124153639	T	4.668	4.16E-06	chr:12:124066477
12	rs2333838	124220354	A	4.356	4.49E-06	chr:12:124066477
12	rs113310757	124161494	G	4.662	4.58E-06	chr:12:124066477
12	rs114208992	124169242	C	4.662	4.58E-06	chr:12:124066477
12	rs11612030	124188064	T	4.662	4.58E-06	chr:12:124066477
12	rs11613632	124184065	A	4.662	4.58E-06	chr:12:124066477
12	rs11614468	124187924	C	4.662	4.58E-06	chr:12:124066477
12	rs118004082	124169240	T	4.662	4.58E-06	chr:12:124066477
12	rs11830462	124178665	T	4.662	4.58E-06	chr:12:124066477
12	rs11831536	124176748	G	4.662	4.58E-06	chr:12:124066477
12	rs11833604	124176472	A	4.662	4.58E-06	chr:12:124066477
12	rs11834492	124180011	C	4.662	4.58E-06	chr:12:124066477
12	rs11834808	124190582	T	4.662	4.58E-06	chr:12:124066477
12	rs11837256	124190393	T	4.662	4.58E-06	chr:12:124066477
12	rs12817757	124156487	T	4.662	4.58E-06	chr:12:124066477
12	rs12818596	124180474	C	4.662	4.58E-06	chr:12:124066477
12	rs12820195	124162047	C	4.662	4.58E-06	chr:12:124066477
12	rs12821242	124178329	A	4.662	4.58E-06	chr:12:124066477
12	rs12822546	124157247	A	4.662	4.58E-06	chr:12:124066477
12	rs12823866	124165111	C	4.662	4.58E-06	chr:12:124066477
12	rs12824075	124157335	T	4.662	4.58E-06	chr:12:124066477
12	rs12827074	124190836	C	4.662	4.58E-06	chr:12:124066477
12	rs12828408	124198718	C	4.662	4.58E-06	chr:12:124066477
12	rs12829984	124172307	T	4.662	4.58E-06	chr:12:124066477
12	rs12831081	124179739	G	4.662	4.58E-06	chr:12:124066477
12	rs2203500	124194710	G	4.662	4.58E-06	chr:12:124066477
12	rs34534928	124160834	C	4.662	4.58E-06	chr:12:124066477
12	rs35416308	124169461	G	4.662	4.58E-06	chr:12:124066477
12	rs35946281	124177016	A	4.662	4.58E-06	chr:12:124066477
12	rs3817309	124198721	T	4.662	4.58E-06	chr:12:124066477
12	rs6488890	124158971	T	4.662	4.58E-06	chr:12:124066477
12	rs6488891	124163512	T	4.662	4.58E-06	chr:12:124066477
12	rs6488892	124171826	C	4.662	4.58E-06	chr:12:124066477
12	rs6488893	124171903	G	4.662	4.58E-06	chr:12:124066477
12	rs6488894	124185124	T	4.662	4.58E-06	chr:12:124066477
12	rs66471742	124199006	A	4.662	4.58E-06	chr:12:124066477
12	rs66690784	124165677	G	4.662	4.58E-06	chr:12:124066477
12	rs67178535	124196299	A	4.662	4.58E-06	chr:12:124066477

12	rs7133543	124159411	G	4.662	4.58E-06	chr:12:124066477
12	rs7135267	124199120	C	4.662	4.58E-06	chr:12:124066477
12	rs7138209	124157052	A	4.662	4.58E-06	chr:12:124066477
12	rs71458814	124196891	G	4.662	4.58E-06	chr:12:124066477
12	rs7298440	124181049	T	4.662	4.58E-06	chr:12:124066477
12	rs7302649	124159766	A	4.662	4.58E-06	chr:12:124066477
12	rs7302788	124167021	C	4.662	4.58E-06	chr:12:124066477
12	rs7304052	124177643	A	4.662	4.58E-06	chr:12:124066477
12	rs7304837	124177538	T	4.662	4.58E-06	chr:12:124066477
12	rs7305838	124193471	A	4.662	4.58E-06	chr:12:124066477
12	rs7307008	124160021	G	4.662	4.58E-06	chr:12:124066477
12	rs7307343	124174365	G	4.662	4.58E-06	chr:12:124066477
12	rs7308234	124183804	T	4.662	4.58E-06	chr:12:124066477
12	rs7309528	124171658	T	4.662	4.58E-06	chr:12:124066477
12	rs7310918	124174656	C	4.662	4.58E-06	chr:12:124066477
12	rs73219037	124184482	C	4.662	4.58E-06	chr:12:124066477
12	rs7397160	124196219	A	4.662	4.58E-06	chr:12:124066477
12	rs7397230	124182659	G	4.662	4.58E-06	chr:12:124066477
12	rs7397930	124188581	A	4.662	4.58E-06	chr:12:124066477
12	rs7398152	124184639	G	4.662	4.58E-06	chr:12:124066477
12	rs7398298	124192728	T	4.662	4.58E-06	chr:12:124066477
12	rs7399224	124195856	T	4.662	4.58E-06	chr:12:124066477
12	rs7953596	124158450	C	4.662	4.58E-06	chr:12:124066477
12	rs7953599	124158455	C	4.662	4.58E-06	chr:12:124066477
12	rs7959553	124162705	T	4.662	4.58E-06	chr:12:124066477
12	rs7963498	124158502	A	4.662	4.58E-06	chr:12:124066477
12	rs7963968	124166041	C	4.662	4.58E-06	chr:12:124066477
12	rs7968029	124182120	T	4.662	4.58E-06	chr:12:124066477
12	rs7969130	124161056	G	4.662	4.58E-06	chr:12:124066477
12	rs7969234	124161110	G	4.662	4.58E-06	chr:12:124066477
12	rs7969964	124165213	A	4.662	4.58E-06	chr:12:124066477
12	rs7973155	124185948	G	4.662	4.58E-06	chr:12:124066477
12	rs7973695	124162477	G	4.662	4.58E-06	chr:12:124066477
12	rs7975119	124157674	T	4.662	4.58E-06	chr:12:124066477
12	rs7975233	124157745	G	4.662	4.58E-06	chr:12:124066477
12	rs7978447	124158080	G	4.662	4.58E-06	chr:12:124066477
12	rs7979528	124181820	G	4.662	4.58E-06	chr:12:124066477
12	rs1040156	124223364	A	4.349	4.64E-06	chr:12:124066477
12	rs10732574	124219121	A	4.349	4.64E-06	chr:12:124066477
12	rs10846551	124222811	G	4.349	4.64E-06	chr:12:124066477
12	rs2271661	124219910	T	4.349	4.64E-06	chr:12:124066477
12	rs2333835	124220879	T	4.349	4.64E-06	chr:12:124066477
12	rs2333839	124216852	T	4.349	4.64E-06	chr:12:124066477

12	rs7300721	124219595	T	4.349	4.64E-06	chr:12:124066477
12	rs2271660	124220055	T	4.345	4.68E-06	chr:12:124066477
12	rs117222175	124244936	T	54.45	5.09E-06	chr:12:124066477
12	rs2333836	124220543	C	4.346	5.12E-06	chr:12:124066477
12	rs786443	124063891	T	4.358	5.39E-06	chr:12:124066477
12	rs786445	124068361	C	4.358	5.39E-06	chr:12:124066477
12	rs786447	124069667	A	4.358	5.39E-06	chr:12:124066477
12	rs35900897	124148892	G	4.594	5.49E-06	chr:12:124066477
12	rs2048615	124011285	C	4.327	5.80E-06	chr:12:124066477
12	rs6488888	124142591	G	4.586	6.02E-06	chr:12:124066477
12	rs7294984	124141749	G	4.586	6.02E-06	chr:12:124066477
12	rs4456350	124058489	A	4.251	6.12E-06	chr:12:124066477
12	rs908373	124113319	G	4.334	6.21E-06	chr:12:124066477
12	rs12813758	124201294	T	4.597	7.21E-06	chr:12:124066477
12	rs28580741	124013216	C	4.173	7.35E-06	chr:12:124066477
12	rs28741988	124013264	C	4.173	7.35E-06	chr:12:124066477
12	rs13303364	124006100	G	4.541	7.44E-06	chr:12:124066477
12	rs9697536	123995023	T	4.469	7.72E-06	chr:12:124066477
12	rs1057879	124013406	A	4.17	7.89E-06	chr:12:124066477
12	rs2333831	124012316	G	4.17	7.89E-06	chr:12:124066477
12	rs28371124	124012558	T	4.17	7.89E-06	chr:12:124066477
12	rs28430750	124012197	G	4.17	7.89E-06	chr:12:124066477
12	rs28450070	124013120	G	4.17	7.89E-06	chr:12:124066477
12	rs28478446	124014177	T	4.17	7.89E-06	chr:12:124066477
12	rs28497190	124012223	T	4.17	7.89E-06	chr:12:124066477
12	rs28535055	124014073	C	4.17	7.89E-06	chr:12:124066477
12	rs28605072	124011804	G	4.17	7.89E-06	chr:12:124066477
12	rs28701527	124011923	C	4.17	7.89E-06	chr:12:124066477
12	rs28707421	124010185	T	4.17	7.89E-06	chr:12:124066477
12	rs1060637	124110122	T	4.458	8.22E-06	chr:12:124066477
12	rs1060639	124109988	A	4.458	8.22E-06	chr:12:124066477
12	rs11572920	124119983	A	4.458	8.22E-06	chr:12:124066477
12	rs36018806	124114552	A	4.458	8.22E-06	chr:12:124066477
12	rs7302183	124104460	G	4.16	8.23E-06	chr:12:124066477
12	rs11522338	124004918	C	4.174	8.26E-06	chr:12:124066477
12	rs11522339	124004948	A	4.174	8.26E-06	chr:12:124066477
12	rs28496079	124006841	C	4.174	8.26E-06	chr:12:124066477
12	rs28712858	124007762	C	4.174	8.26E-06	chr:12:124066477
12	rs7970602	124165056	T	4.372	8.35E-06	chr:12:124066477
12	rs35911711	124201087	T	4.405	8.50E-06	chr:12:124066477
12	rs1515815	124111999	C	4.458	8.68E-06	chr:12:124066477
12	rs11057326	124148478	T	4.294	9.14E-06	chr:12:124066477
12	rs34586028	124187082	C	4.438	9.86E-06	chr:12:124066477

12	rs12830927	124120991	T	4.432	1.01E-05	chr:12:124066477
12	rs786444	124065416	T	4.083	1.04E-05	chr:12:124066477
12	rs11572952	124129809	A	4.341	1.13E-05	chr:12:124066477
12	rs11611450	124148207	G	4.341	1.13E-05	chr:12:124066477
12	rs11612927	124131727	C	4.341	1.13E-05	chr:12:124066477
12	rs11615162	124131929	A	4.341	1.13E-05	chr:12:124066477
12	rs12817576	124126339	T	4.341	1.13E-05	chr:12:124066477
12	rs2017181	124117257	G	4.266	1.18E-05	chr:12:124066477
12	rs111707562	124164355	C	4.333	1.22E-05	chr:12:124066477
12	rs11614155	124167175	T	4.333	1.22E-05	chr:12:124066477
12	rs12811354	124192415	A	4.333	1.22E-05	chr:12:124066477
12	rs12829893	124166978	T	4.333	1.22E-05	chr:12:124066477
12	rs35804181	124162344	C	4.333	1.22E-05	chr:12:124066477
12	rs7133734	124196735	C	4.333	1.22E-05	chr:12:124066477
12	rs7136939	124189657	C	4.333	1.22E-05	chr:12:124066477
12	rs11057307	124098213	C	4.01	1.24E-05	chr:12:124066477
12	rs3748269	124099925	C	4.01	1.24E-05	chr:12:124066477
12	rs786422	124083562	G	4.01	1.24E-05	chr:12:124066477
12	rs786423	124083898	G	4.01	1.24E-05	chr:12:124066477
12	rs1139320	124197099	T	4.219	1.29E-05	chr:12:124066477
12	rs1543075	124086448	G	3.926	1.35E-05	chr:12:124066477
12	rs1543076	124086449	C	3.926	1.35E-05	chr:12:124066477
12	rs12813346	124201114	G	4.222	1.38E-05	chr:12:124066477
12	rs11522337	124004843	C	4	1.39E-05	chr:12:124066477
12	rs6488883	124101347	C	3.919	1.41E-05	chr:12:124066477
12	rs6551	124082240	C	3.919	1.41E-05	chr:12:124066477
12	rs7134896	124101858	A	3.919	1.41E-05	chr:12:124066477
12	rs786448	124070214	A	3.919	1.41E-05	chr:12:124066477
12	rs786449	124071728	C	3.919	1.41E-05	chr:12:124066477
12	rs10846540	124169093	G	4.227	1.44E-05	chr:12:124066477
12	rs1969272	124170293	G	4.227	1.44E-05	chr:12:124066477
12	rs2222061	124194929	T	4.227	1.44E-05	chr:12:124066477
12	rs56176939	124184829	G	4.227	1.44E-05	chr:12:124066477
12	rs7137946	124177289	C	4.227	1.44E-05	chr:12:124066477
12	rs7139193	124171176	G	4.227	1.44E-05	chr:12:124066477
12	rs7301907	124172342	C	4.227	1.44E-05	chr:12:124066477
12	rs7302449	124172746	C	4.227	1.44E-05	chr:12:124066477
12	rs7316011	124185212	T	4.227	1.44E-05	chr:12:124066477
12	rs10846535	124130388	G	4.201	1.46E-05	chr:12:124066477
12	rs11057320	124141362	G	4.201	1.46E-05	chr:12:124066477
12	rs7971452	124126821	G	4.201	1.46E-05	chr:12:124066477
12	rs7966882	124128965	A	4.193	1.53E-05	chr:12:124066477
12	rs10846541	124176286	T	4.225	1.54E-05	chr:12:124066477

12	rs7960017	124165059	C	4.414	1.68E-05	chr:12:124066477
12	rs111482432	124078585	G	22.85	1.72E-05	chr:12:124066477
12	rs113088709	124095455	G	22.85	1.72E-05	chr:12:124066477
12	rs113620982	124086656	T	22.85	1.72E-05	chr:12:124066477
12	rs114613914	124089740	G	22.85	1.72E-05	chr:12:124066477
12	rs12099985	124065667	C	22.85	1.72E-05	chr:12:124066477
12	rs3850953	124091412	T	22.85	1.72E-05	chr:12:124066477
12	rs75700739	124084424	A	22.85	1.72E-05	chr:12:124066477
12	rs78068178	124059962	T	22.85	1.72E-05	chr:12:124066477
12	rs7964934	124058639	A	22.85	1.72E-05	chr:12:124066477
12	rs7965292	124081779	T	22.85	1.72E-05	chr:12:124066477
12	rs7973187	124068015	C	22.85	1.72E-05	chr:12:124066477
12	rs7973689	124090655	T	22.85	1.72E-05	chr:12:124066477
12	rs79847496	124089348	G	22.85	1.72E-05	chr:12:124066477
12	rs12821219	124178290	C	4.09	1.75E-05	chr:12:124066477
12	rs67516712	124201524	A	3.981	1.84E-05	chr:12:124066477
12	rs11057376	124348065	T	15.11	1.84E-05	chr:12:124066477
12	rs6488898	124203832	G	15.53	1.86E-05	chr:12:124066477
12	rs137963709	124020685	A	11.07	1.89E-05	chr:12:124066477
12	rs11837257	124190394	C	4.312	1.90E-05	chr:12:124066477
12	rs10846546	124187886	G	4.199	1.90E-05	chr:12:124066477
12	rs7966867	124179417	C	4.199	1.90E-05	chr:12:124066477
12	rs1515814	124108699	A	3.903	2.05E-05	chr:12:124066477
12	rs117336016	124196606	A	5.514	2.07E-05	chr:12:124066477
12	rs146184147	124196604	A	5.514	2.07E-05	chr:12:124066477
12	rs11611540	124187606	T	11.54	2.41E-05	chr:12:124066477
12	rs117871720	124052384	G	22.45	2.54E-05	chr:12:124066477
12	rs7972413	124108296	C	3.8	2.68E-05	chr:12:124066477
12	rs7135624	124147831	G	3.998	2.71E-05	chr:12:124066477
12	rs138318001	124054104	C	20.77	2.73E-05	chr:12:124066477
12	rs6488882	124056572	T	20.77	2.73E-05	chr:12:124066477
12	rs7306906	124100486	A	20.77	2.73E-05	chr:12:124066477
12	rs75468015	124054109	C	20.77	2.73E-05	chr:12:124066477
12	rs78702455	124054015	G	20.77	2.73E-05	chr:12:124066477
12	rs4040225	124096673	A	3.805	2.94E-05	chr:12:124066477
12	rs786425	124085502	G	3.805	2.94E-05	chr:12:124066477
12	rs12815746	124126086	T	4.249	3.01E-05	chr:12:124066477
12	rs10744162	124243707	T	3.513	3.89E-05	chr:12:124066477
12	rs10773033	124231368	T	3.513	3.89E-05	chr:12:124066477
12	rs10773034	124234253	A	3.513	3.89E-05	chr:12:124066477
12	rs10744160	124225542	A	3.554	3.90E-05	chr:12:124066477
12	rs7313628	124107015	A	3.639	4.00E-05	chr:12:124066477
12	rs6488886	124120205	A	15.65	4.03E-05	chr:12:124066477

12	rs10785482	44342910	T	152.2	8.43E-18	chr:12:44313657
12	rs10880597	44336172	A	152.2	8.43E-18	chr:12:44313657
12	rs10880600	44343641	T	152.2	8.43E-18	chr:12:44313657
12	rs10880601	44343768	A	152.2	8.43E-18	chr:12:44313657
12	rs11182339	44331841	A	152.2	8.43E-18	chr:12:44313657
12	rs11182341	44339216	T	152.2	8.43E-18	chr:12:44313657
12	rs11182346	44350097	T	152.2	8.43E-18	chr:12:44313657
12	rs7312373	44332843	C	152.2	8.43E-18	chr:12:44313657
12	rs10880605	44349669	A	141.9	1.30E-17	chr:12:44313657
12	rs10785473	44316584	G	202.8	6.85E-17	chr:12:44313657
12	rs10785474	44318428	G	202.8	6.85E-17	chr:12:44313657
12	rs10785477	44318723	G	202.8	6.85E-17	chr:12:44313657
12	rs10785478	44320156	T	202.8	6.85E-17	chr:12:44313657
12	rs10880586	44310604	G	202.8	6.85E-17	chr:12:44313657
12	rs11182327	44310418	A	202.8	6.85E-17	chr:12:44313657
12	rs4417371	44327407	A	202.8	6.85E-17	chr:12:44313657
12	rs4768544	44309919	C	269.1	2.03E-16	chr:12:44313657
12	rs11182358	44363335	T	32.72	2.79E-16	chr:12:44313657
12	rs200356917	44301684	A	6.282	3.06E-16	chr:12:44313657
12	rs201614323	44301725	T	5.828	1.21E-15	chr:12:44313657
12	rs1731447	44289250	A	32.29	1.25E-15	chr:12:44313657
12	rs77056193	44225895	T	46.77	1.72E-15	chr:12:44313657
12	rs9668752	44302117	T	5.715	2.05E-15	chr:12:44313657
12	rs10785467	44309433	T	21.07	2.63E-15	chr:12:44313657
12	rs7350559	44362805	G	31.74	2.95E-15	chr:12:44313657
12	rs7979391	44295117	G	20.64	3.72E-15	chr:12:44313657
12	rs10736017	44300037	A	19.9	5.42E-15	chr:12:44313657
12	rs10880580	44297492	G	19.9	5.42E-15	chr:12:44313657
12	rs1731421	44285207	A	19.9	5.42E-15	chr:12:44313657
12	rs1731427	44281449	T	19.9	5.42E-15	chr:12:44313657
12	rs1731429	44280899	T	19.9	5.42E-15	chr:12:44313657
12	rs2614394	44282166	A	19.9	5.42E-15	chr:12:44313657
12	rs4238090	44299833	T	19.9	5.42E-15	chr:12:44313657
12	rs4354753	44293620	G	19.9	5.42E-15	chr:12:44313657
12	rs4768052	44308910	T	19.9	5.42E-15	chr:12:44313657
12	rs4768534	44298821	T	19.9	5.42E-15	chr:12:44313657
12	rs7296222	44238104	A	39.2	5.51E-15	chr:12:44313657
12	rs75842033	44228587	A	42.28	6.42E-15	chr:12:44313657
12	rs9668657	44302174	A	6.222	6.59E-15	chr:12:44313657
12	rs7973886	44306134	G	6.436	6.77E-15	chr:12:44313657
12	rs7961078	44306167	A	19.71	7.00E-15	chr:12:44313657
12	rs12318365	44352760	T	55.15	8.73E-15	chr:12:44313657
12	rs7963981	44306066	T	5.891	9.20E-15	chr:12:44313657

12	rs7973769	44306028	G	5.891	9.20E-15	chr:12:44313657
12	rs199957779	44302961	T	6.595	1.18E-14	chr:12:44313657
12	rs201324668	44302939	C	6.595	1.18E-14	chr:12:44313657
12	rs7973774	44306044	G	5.847	1.29E-14	chr:12:44313657
12	rs12302508	44303914	A	23.44	1.30E-14	chr:12:44313657
12	rs199914146	44303005	G	6.154	1.43E-14	chr:12:44313657
12	rs201261552	44302974	C	6.154	1.43E-14	chr:12:44313657
12	rs9668647	44301830	A	5.741	1.52E-14	chr:12:44313657
12	rs9669053	44301883	G	5.542	1.55E-14	chr:12:44313657
12	rs11182298	44248189	A	49.85	1.68E-14	chr:12:44313657
12	rs11182300	44249025	T	49.85	1.68E-14	chr:12:44313657
12	rs11182301	44249779	C	49.85	1.68E-14	chr:12:44313657
12	rs11182304	44250895	A	49.85	1.68E-14	chr:12:44313657
12	rs11182305	44250958	G	49.85	1.68E-14	chr:12:44313657
12	rs113386845	44239075	T	49.85	1.68E-14	chr:12:44313657
12	rs113451227	44247014	G	49.85	1.68E-14	chr:12:44313657
12	rs115601996	44242554	T	49.85	1.68E-14	chr:12:44313657
12	rs116608528	44239556	C	49.85	1.68E-14	chr:12:44313657
12	rs116609893	44241652	G	49.85	1.68E-14	chr:12:44313657
12	rs117577677	44246366	C	49.85	1.68E-14	chr:12:44313657
12	rs12230284	44245217	G	49.85	1.68E-14	chr:12:44313657
12	rs12230296	44245365	T	49.85	1.68E-14	chr:12:44313657
12	rs12230727	44247063	C	49.85	1.68E-14	chr:12:44313657
12	rs12230751	44247312	C	49.85	1.68E-14	chr:12:44313657
12	rs12231953	44245366	G	49.85	1.68E-14	chr:12:44313657
12	rs140628578	44239252	T	49.85	1.68E-14	chr:12:44313657
12	rs142324451	44246163	C	49.85	1.68E-14	chr:12:44313657
12	rs144999308	44241733	A	49.85	1.68E-14	chr:12:44313657
12	rs146784200	44239223	T	49.85	1.68E-14	chr:12:44313657
12	rs149174850	44246158	T	49.85	1.68E-14	chr:12:44313657
12	rs151064202	44242351	C	49.85	1.68E-14	chr:12:44313657
12	rs61930682	44240177	T	49.85	1.68E-14	chr:12:44313657
12	rs74373254	44240769	A	49.85	1.68E-14	chr:12:44313657
12	rs74775231	44246985	T	49.85	1.68E-14	chr:12:44313657
12	rs76240216	44244168	T	49.85	1.68E-14	chr:12:44313657
12	rs77161382	44241297	T	49.85	1.68E-14	chr:12:44313657
12	rs77556291	44246646	C	49.85	1.68E-14	chr:12:44313657
12	rs77708558	44240597	G	49.85	1.68E-14	chr:12:44313657
12	rs78339833	44236763	G	49.85	1.68E-14	chr:12:44313657
12	rs78388987	44244204	C	49.85	1.68E-14	chr:12:44313657
12	rs79074543	44246752	C	49.85	1.68E-14	chr:12:44313657
12	rs79425253	44241126	T	49.85	1.68E-14	chr:12:44313657
12	rs79716923	44236764	C	49.85	1.68E-14	chr:12:44313657

12	rs79970551	44244859	A	49.85	1.68E-14	chr:12:44313657
12	rs80099331	44240349	T	49.85	1.68E-14	chr:12:44313657
12	rs202162002	44301820	A	5.492	1.77E-14	chr:12:44313657
12	rs367898590	44301818	C	5.492	1.77E-14	chr:12:44313657
12	rs375152458	44301817	T	5.492	1.77E-14	chr:12:44313657
12	rs11533717	44304261	G	21.86	1.92E-14	chr:12:44313657
12	rs11182303	44250800	A	45.85	2.40E-14	chr:12:44313657
12	rs150307762	44246320	G	47.71	2.82E-14	chr:12:44313657
12	rs10880604	44349398	G	135	3.12E-14	chr:12:44313657
12	rs4519146	44327240	A	67.33	3.73E-14	chr:12:44313657
12	rs201066998	44303041	G	5.338	3.90E-14	chr:12:44313657
12	rs11182299	44248269	A	46.96	5.56E-14	chr:12:44313657
12	rs7968199	44212237	G	15.11	5.89E-14	chr:12:44313657
12	rs7971516	44353473	G	115.6	1.08E-13	chr:12:44313657
12	rs10785468	44309434	G	20.84	1.65E-13	chr:12:44313657
12	rs10785469	44309459	G	20.84	1.65E-13	chr:12:44313657
12	rs7956245	44143520	T	16.33	1.88E-13	chr:12:44313657
12	rs3990338	44300626	T	18.92	1.97E-13	chr:12:44313657
12	rs766961	44236021	G	39.73	2.09E-13	chr:12:44313657
12	rs7965240	44309591	A	12.73	2.29E-13	chr:12:44313657
12	rs7965330	44309602	A	25.39	2.30E-13	chr:12:44313657
12	rs11520309	44303103	A	19.01	2.40E-13	chr:12:44313657
12	rs7971628	44353583	G	14.78	2.49E-13	chr:12:44313657
12	rs3990339	44300728	G	13.42	2.50E-13	chr:12:44313657
12	rs11182326	44310333	G	51.51	2.54E-13	chr:12:44313657
12	rs11182328	44311277	G	51.51	2.54E-13	chr:12:44313657
12	rs11182345	44347202	C	51.51	2.54E-13	chr:12:44313657
12	rs11829881	44322395	G	51.51	2.54E-13	chr:12:44313657
12	rs12301015	44321160	T	51.51	2.54E-13	chr:12:44313657
12	rs12303804	44340018	A	51.51	2.54E-13	chr:12:44313657
12	rs12309681	44343202	T	51.51	2.54E-13	chr:12:44313657
12	rs12315094	44345773	A	51.51	2.54E-13	chr:12:44313657
12	rs7969641	44310268	T	51.51	2.54E-13	chr:12:44313657
12	rs4768539	44303524	C	16.8	2.72E-13	chr:12:44313657
12	rs17093971	44359265	T	32.03	2.76E-13	chr:12:44313657
12	rs7308588	44356119	A	32.03	2.76E-13	chr:12:44313657
12	rs2407787	44300709	G	13.91	2.77E-13	chr:12:44313657
12	rs10748377	44308151	T	18.22	2.86E-13	chr:12:44313657
12	rs10748378	44308153	A	18.22	2.86E-13	chr:12:44313657
12	rs10785462	44299067	A	18.22	2.86E-13	chr:12:44313657
12	rs10785463	44299093	T	18.22	2.86E-13	chr:12:44313657
12	rs10785466	44308052	A	18.22	2.86E-13	chr:12:44313657
12	rs10880579	44297491	A	18.22	2.86E-13	chr:12:44313657

12	rs10880585	44307982	C	18.22	2.86E-13	chr:12:44313657
12	rs11182324	44307868	C	18.22	2.86E-13	chr:12:44313657
12	rs11182325	44307930	G	18.22	2.86E-13	chr:12:44313657
12	rs1358705	44287850	C	18.22	2.86E-13	chr:12:44313657
12	rs1609549	44306994	A	18.22	2.86E-13	chr:12:44313657
12	rs1610291	44307158	A	18.22	2.86E-13	chr:12:44313657
12	rs1614042	44290018	C	18.22	2.86E-13	chr:12:44313657
12	rs1675756	44279540	T	18.22	2.86E-13	chr:12:44313657
12	rs1675781	44292513	A	18.22	2.86E-13	chr:12:44313657
12	rs1675789	44290545	A	18.22	2.86E-13	chr:12:44313657
12	rs1675790	44290447	C	18.22	2.86E-13	chr:12:44313657
12	rs1731430	44280809	A	18.22	2.86E-13	chr:12:44313657
12	rs1731456	44286932	A	18.22	2.86E-13	chr:12:44313657
12	rs1731457	44286499	T	18.22	2.86E-13	chr:12:44313657
12	rs1731458	44285969	T	18.22	2.86E-13	chr:12:44313657
12	rs1731459	44285844	C	18.22	2.86E-13	chr:12:44313657
12	rs2407783	44300247	G	18.22	2.86E-13	chr:12:44313657
12	rs2407786	44300467	A	18.22	2.86E-13	chr:12:44313657
12	rs4238089	44299688	A	18.22	2.86E-13	chr:12:44313657
12	rs4238091	44299900	T	18.22	2.86E-13	chr:12:44313657
12	rs4363674	44307404	C	18.22	2.86E-13	chr:12:44313657
12	rs4469953	44307349	T	18.22	2.86E-13	chr:12:44313657
12	rs4572198	44298071	G	18.22	2.86E-13	chr:12:44313657
12	rs4572199	44298378	G	18.22	2.86E-13	chr:12:44313657
12	rs4609659	44298550	C	18.22	2.86E-13	chr:12:44313657
12	rs4768053	44309069	C	18.22	2.86E-13	chr:12:44313657
12	rs4768532	44279222	C	18.22	2.86E-13	chr:12:44313657
12	rs4768535	44298917	C	18.22	2.86E-13	chr:12:44313657
12	rs4768538	44300011	A	18.22	2.86E-13	chr:12:44313657
12	rs7295151	44308194	G	18.22	2.86E-13	chr:12:44313657
12	rs7974045	44306266	G	18.22	2.86E-13	chr:12:44313657
12	rs7974046	44306269	G	18.22	2.86E-13	chr:12:44313657
12	rs7954640	44301081	C	12.42	2.97E-13	chr:12:44313657
12	rs10880578	44291513	C	22.27	2.99E-13	chr:12:44313657
12	rs1675787	44290928	T	22.27	2.99E-13	chr:12:44313657
12	rs1731432	44279783	T	22.27	2.99E-13	chr:12:44313657
12	rs1731435	44292195	A	22.27	2.99E-13	chr:12:44313657
12	rs2447113	44282785	G	22.27	2.99E-13	chr:12:44313657
12	rs2951435	44291174	G	22.27	2.99E-13	chr:12:44313657
12	rs1675764	44285480	T	17.33	3.12E-13	chr:12:44313657
12	rs1731433	44279776	G	17.33	3.12E-13	chr:12:44313657
12	rs4537822	44299389	C	17.33	3.12E-13	chr:12:44313657
12	rs4768543	44308978	A	17.33	3.12E-13	chr:12:44313657

12	rs2614395	44291851	G	19.3	3.19E-13	chr:12:44313657
12	rs1406787	44274699	T	18.9	3.61E-13	chr:12:44313657
12	rs79916589	44289680	T	15.53	3.63E-13	chr:12:44313657
12	rs5019975	44299370	G	17.98	3.65E-13	chr:12:44313657
12	rs2407784	44300347	T	17.97	3.69E-13	chr:12:44313657
12	rs2407785	44300358	A	17.97	3.69E-13	chr:12:44313657
12	rs151265567	44318897	T	54.56	4.21E-13	chr:12:44313657
12	rs7965873	44354797	A	20.17	4.34E-13	chr:12:44313657
12	rs7954130	44301327	G	11.86	4.76E-13	chr:12:44313657
12	rs1731423	44278469	G	21.99	4.89E-13	chr:12:44313657
12	rs1675755	44278773	G	25.83	5.09E-13	chr:12:44313657
12	rs147015726	44303495	G	53.1	5.61E-13	chr:12:44313657
12	rs1731455	44260900	A	14.14	5.71E-13	chr:12:44313657
12	rs11182331	44317153	C	49.93	5.86E-13	chr:12:44313657
12	rs11182334	44318590	G	49.93	5.86E-13	chr:12:44313657
12	rs11182336	44320611	G	49.93	5.86E-13	chr:12:44313657
12	rs1057486	44260041	G	20.56	5.99E-13	chr:12:44313657
12	rs11182293	44231482	A	20.56	5.99E-13	chr:12:44313657
12	rs1621279	44249011	C	20.56	5.99E-13	chr:12:44313657
12	rs1675765	44244855	T	20.56	5.99E-13	chr:12:44313657
12	rs1675769	44248400	T	20.56	5.99E-13	chr:12:44313657
12	rs1731450	44246041	T	20.56	5.99E-13	chr:12:44313657
12	rs1731452	44249745	C	20.56	5.99E-13	chr:12:44313657
12	rs1731454	44251107	T	20.56	5.99E-13	chr:12:44313657
12	rs7305609	44240652	A	20.56	5.99E-13	chr:12:44313657
12	rs73096934	44241279	G	20.56	5.99E-13	chr:12:44313657
12	rs2219629	44235406	T	15.65	6.28E-13	chr:12:44313657
12	rs1623667	44249258	T	20.23	6.78E-13	chr:12:44313657
12	rs1675767	44247135	G	20.23	6.78E-13	chr:12:44313657
12	rs1731434	44272915	A	20.23	6.78E-13	chr:12:44313657
12	rs11182323	44306528	G	48.67	7.79E-13	chr:12:44313657
12	rs116930265	44299125	C	48.67	7.79E-13	chr:12:44313657
12	rs12230228	44303195	A	48.67	7.79E-13	chr:12:44313657
12	rs139946355	44282948	C	48.67	7.79E-13	chr:12:44313657
12	rs28758958	44306264	A	48.67	7.79E-13	chr:12:44313657
12	rs367990382	44301261	T	48.67	7.79E-13	chr:12:44313657
12	rs528423997	44303078	G	48.67	7.79E-13	chr:12:44313657
12	rs542685597	44303937	T	48.67	7.79E-13	chr:12:44313657
12	rs566058745	44300960	A	48.67	7.79E-13	chr:12:44313657
12	rs75158834	44280690	T	48.67	7.79E-13	chr:12:44313657
12	rs79110324	44296458	G	48.67	7.79E-13	chr:12:44313657
12	rs1609548	44306947	G	25.29	8.05E-13	chr:12:44313657
12	rs1623829	44262745	A	25.24	8.97E-13	chr:12:44313657

12	rs11182340	44335190	G	40.39	1.05E-12	chr:12:44313657
12	rs11182343	44344206	A	40.39	1.05E-12	chr:12:44313657
12	rs11182347	44353617	G	40.39	1.05E-12	chr:12:44313657
12	rs11182349	44357759	G	40.39	1.05E-12	chr:12:44313657
12	rs11182351	44358078	T	40.39	1.05E-12	chr:12:44313657
12	rs11182352	44361158	G	40.39	1.05E-12	chr:12:44313657
12	rs116884551	44340736	C	40.39	1.05E-12	chr:12:44313657
12	rs117528618	44350676	G	40.39	1.05E-12	chr:12:44313657
12	rs140180348	44336849	T	40.39	1.05E-12	chr:12:44313657
12	rs146427438	44339234	T	40.39	1.05E-12	chr:12:44313657
12	rs150694122	44336742	A	40.39	1.05E-12	chr:12:44313657
12	rs186989426	44344787	C	40.39	1.05E-12	chr:12:44313657
12	rs200338856	44340179	G	40.39	1.05E-12	chr:12:44313657
12	rs78675197	44360787	A	40.39	1.05E-12	chr:12:44313657
12	rs79112229	44361616	G	40.39	1.05E-12	chr:12:44313657
12	rs79163779	44361704	T	40.39	1.05E-12	chr:12:44313657
12	rs80120468	44344737	G	40.39	1.05E-12	chr:12:44313657
12	rs80251371	44328497	T	40.39	1.05E-12	chr:12:44313657
12	rs146868110	44293933	G	43.48	1.08E-12	chr:12:44313657
12	rs61930734	44304400	C	11.26	1.39E-12	chr:12:44313657
12	rs1622590	44288061	G	30.85	2.10E-12	chr:12:44313657
12	rs1675758	44280027	G	30.85	2.10E-12	chr:12:44313657
12	rs1675785	44292747	G	30.85	2.10E-12	chr:12:44313657
12	rs1731431	44279870	G	30.85	2.10E-12	chr:12:44313657
12	rs7955615	44295861	C	30.85	2.10E-12	chr:12:44313657
12	rs10785460	44264906	T	11.16	2.40E-12	chr:12:44313657
12	rs6582490	44329957	G	29.29	2.50E-12	chr:12:44313657
12	rs7979691	44142588	A	14.14	2.53E-12	chr:12:44313657
12	rs4768546	44336686	C	28.88	2.72E-12	chr:12:44313657
12	rs4768057	44332707	G	29.03	2.80E-12	chr:12:44313657
12	rs12298711	44206759	T	10.18	2.84E-12	chr:12:44313657
12	rs11182337	44320875	A	210.1	2.85E-12	chr:12:44313657
12	rs4238093	44315925	A	210.1	2.85E-12	chr:12:44313657
12	rs10880573	44226111	G	9.409	2.96E-12	chr:12:44313657
12	rs112105382	44241680	T	12.32	3.26E-12	chr:12:44313657
12	rs4768536	44298961	A	17.59	3.60E-12	chr:12:44313657
12	rs7972025	44222722	T	9.311	4.50E-12	chr:12:44313657
12	rs11182314	44260848	T	41.84	5.53E-12	chr:12:44313657
12	rs4238092	44315762	C	15.47	6.91E-12	chr:12:44313657
12	rs115482982	44250485	G	28.91	8.94E-12	chr:12:44313657
12	rs12229940	44244636	A	28.91	8.94E-12	chr:12:44313657
12	rs137888888	44246356	C	28.91	8.94E-12	chr:12:44313657
12	rs138541393	44246210	C	28.91	8.94E-12	chr:12:44313657

12	rs143317429	44239623	T	28.91	8.94E-12	chr:12:44313657
12	rs76048883	44250719	T	28.91	8.94E-12	chr:12:44313657
12	rs76350700	44240040	G	28.91	8.94E-12	chr:12:44313657
12	rs77952333	44240015	A	28.91	8.94E-12	chr:12:44313657
12	rs78170106	44250279	G	28.91	8.94E-12	chr:12:44313657
12	rs78179678	44239907	C	28.91	8.94E-12	chr:12:44313657
12	rs79392152	44250626	C	28.91	8.94E-12	chr:12:44313657
12	rs79783862	44244432	A	28.91	8.94E-12	chr:12:44313657
12	rs10880587	44311477	T	37.04	1.17E-11	chr:12:44313657
12	rs1881926	44319047	G	37.04	1.17E-11	chr:12:44313657
12	rs1881927	44318922	G	37.04	1.17E-11	chr:12:44313657
12	rs4768055	44317208	G	37.04	1.17E-11	chr:12:44313657
12	rs4768056	44322826	T	37.04	1.17E-11	chr:12:44313657
12	rs7974214	44319517	T	37.04	1.17E-11	chr:12:44313657
12	rs10785481	44325061	G	36.5	1.28E-11	chr:12:44313657
12	rs5023324	44327148	C	36.5	1.28E-11	chr:12:44313657
12	rs7311847	44315590	A	36.5	1.28E-11	chr:12:44313657
12	rs112006889	44196125	C	18.28	1.48E-11	chr:12:44313657
12	rs202009693	44267522	T	31.25	1.54E-11	chr:12:44313657
12	rs12315211	44133668	A	10.89	1.68E-11	chr:12:44313657
12	rs11182306	44251130	A	39.29	2.11E-11	chr:12:44313657
12	rs7954384	44301587	G	5.16	2.22E-11	chr:12:44313657
12	rs7969754	44301580	A	5.16	2.22E-11	chr:12:44313657
12	rs11504469	44096699	A	10.4	2.31E-11	chr:12:44313657
12	rs12301056	44098701	G	10.4	2.31E-11	chr:12:44313657
12	rs60029414	44096996	G	10.4	2.31E-11	chr:12:44313657
12	rs201859105	44232358	C	11.83	2.35E-11	chr:12:44313657
12	rs117709201	44224088	T	22.89	2.58E-11	chr:12:44313657
12	rs11182308	44252969	G	38	2.62E-11	chr:12:44313657
12	rs11182310	44255992	A	38	2.62E-11	chr:12:44313657
12	rs116861561	44256819	T	38	2.62E-11	chr:12:44313657
12	rs117137831	44254126	G	38	2.62E-11	chr:12:44313657
12	rs117543763	44264051	A	38	2.62E-11	chr:12:44313657
12	rs118062126	44243952	T	38	2.62E-11	chr:12:44313657
12	rs12227646	44245640	C	38	2.62E-11	chr:12:44313657
12	rs12229491	44255465	A	38	2.62E-11	chr:12:44313657
12	rs12231136	44268262	T	38	2.62E-11	chr:12:44313657
12	rs140988631	44242531	C	38	2.62E-11	chr:12:44313657
12	rs17093804	44272622	G	38	2.62E-11	chr:12:44313657
12	rs183971901	44228578	A	38	2.62E-11	chr:12:44313657
12	rs186673154	44261933	T	38	2.62E-11	chr:12:44313657
12	rs1948516	44238723	A	38	2.62E-11	chr:12:44313657
12	rs2199390	44235245	C	38	2.62E-11	chr:12:44313657

12	rs3829807	44255108	A	38	2.62E-11	chr:12:44313657
12	rs720944	44237303	C	38	2.62E-11	chr:12:44313657
12	rs74423632	44229154	T	38	2.62E-11	chr:12:44313657
12	rs74888682	44237950	C	38	2.62E-11	chr:12:44313657
12	rs76030944	44226085	A	38	2.62E-11	chr:12:44313657
12	rs76737056	44244225	G	38	2.62E-11	chr:12:44313657
12	rs11182219	44099521	T	14.73	3.38E-11	chr:12:44313657
12	rs141598641	44249695	T	36.72	3.54E-11	chr:12:44313657
12	rs17093639	44132928	C	10.64	3.85E-11	chr:12:44313657
12	rs12228658	44277314	C	11.94	4.05E-11	chr:12:44313657
12	rs147802186	44187010	T	27.94	5.56E-11	chr:12:44313657
12	rs10785485	44355693	A	12.47	7.75E-11	chr:12:44313657
12	rs118045375	44185257	A	18.61	8.28E-11	chr:12:44313657
12	rs141317022	44195765	G	18.61	8.28E-11	chr:12:44313657
12	rs150213649	44195782	A	18.61	8.28E-11	chr:12:44313657
12	rs17093732	44191475	G	18.61	8.28E-11	chr:12:44313657
12	rs17093734	44194265	G	18.61	8.28E-11	chr:12:44313657
12	rs1802561	44189129	C	18.61	8.28E-11	chr:12:44313657
12	rs79866180	44193532	G	18.61	8.28E-11	chr:12:44313657
12	rs17121283	44185593	T	7.255	9.42E-11	chr:12:44313657
12	rs6582484	44190630	C	7.135	9.81E-11	chr:12:44313657
12	rs17093747	44205459	G	17.06	1.12E-10	chr:12:44313657
12	rs17093754	44209423	A	17.06	1.12E-10	chr:12:44313657
12	rs2166063	44202450	G	17.06	1.12E-10	chr:12:44313657
12	rs4251475	44167093	G	15.11	1.38E-10	chr:12:44313657
12	rs7961537	44102333	T	14.55	1.53E-10	chr:12:44313657
12	rs9849	44182951	C	7.087	1.55E-10	chr:12:44313657
12	rs145873486	44401507	T	27.58	1.75E-10	chr:12:44313657
12	rs10880598	44336375	T	1191	1.95E-10	chr:12:44313657
12	rs12305959	44332618	G	1191	1.95E-10	chr:12:44313657
12	rs73100975	44339016	T	1191	1.95E-10	chr:12:44313657
12	rs73100933	44314453	T	1426	2.33E-10	chr:12:44313657
12	rs73100935	44314454	G	1426	2.33E-10	chr:12:44313657
12	rs11182290	44221032	A	6.17	2.44E-10	chr:12:44313657
12	rs11182280	44209584	C	6.075	2.93E-10	chr:12:44313657
12	rs12301506	44129636	G	6.074	3.43E-10	chr:12:44313657
12	rs10506231	44206027	T	5.787	5.18E-10	chr:12:44313657
12	rs11182275	44206289	G	5.787	5.18E-10	chr:12:44313657
12	rs3764004	44201451	C	5.787	5.18E-10	chr:12:44313657
12	rs10880599	44337662	T	789.1	5.38E-10	chr:12:44313657
12	rs11182273	44202901	C	5.769	5.60E-10	chr:12:44313657
12	rs142119333	44462402	C	20.09	6.36E-10	chr:12:44313657
12	rs12312338	44340209	T	8.514	7.00E-10	chr:12:44313657

12	rs75725665	44340195	T	8.514	7.00E-10	chr:12:44313657
12	rs10880571	44207279	C	5.317	1.23E-09	chr:12:44313657
12	rs116999385	44173427	A	13.45	1.53E-09	chr:12:44313657
12	rs117008835	44158656	G	13.45	1.53E-09	chr:12:44313657
12	rs117068074	44160759	T	13.45	1.53E-09	chr:12:44313657
12	rs117132545	44103892	A	13.45	1.53E-09	chr:12:44313657
12	rs117307608	44088553	A	13.45	1.53E-09	chr:12:44313657
12	rs117344023	44136757	T	13.45	1.53E-09	chr:12:44313657
12	rs117417075	44108549	T	13.45	1.53E-09	chr:12:44313657
12	rs117903595	44137098	T	13.45	1.53E-09	chr:12:44313657
12	rs118070124	44132815	C	13.45	1.53E-09	chr:12:44313657
12	rs11832281	44128220	C	13.45	1.53E-09	chr:12:44313657
12	rs138820383	44180086	G	13.45	1.53E-09	chr:12:44313657
12	rs139229724	44098042	A	13.45	1.53E-09	chr:12:44313657
12	rs139612761	44126233	C	13.45	1.53E-09	chr:12:44313657
12	rs140672847	44089882	T	13.45	1.53E-09	chr:12:44313657
12	rs144126457	44121102	A	13.45	1.53E-09	chr:12:44313657
12	rs144270751	44098631	G	13.45	1.53E-09	chr:12:44313657
12	rs144539565	44089765	C	13.45	1.53E-09	chr:12:44313657
12	rs146605165	44141210	C	13.45	1.53E-09	chr:12:44313657
12	rs150424915	44158977	G	13.45	1.53E-09	chr:12:44313657
12	rs17093630	44125736	C	13.45	1.53E-09	chr:12:44313657
12	rs17093632	44126508	A	13.45	1.53E-09	chr:12:44313657
12	rs181407925	44112509	T	13.45	1.53E-09	chr:12:44313657
12	rs1870765	44147491	C	13.45	1.53E-09	chr:12:44313657
12	rs191905705	44158574	C	13.45	1.53E-09	chr:12:44313657
12	rs3805199	44176015	G	13.45	1.53E-09	chr:12:44313657
12	rs4251490	44169965	A	13.45	1.53E-09	chr:12:44313657
12	rs532722540	44158576	A	13.45	1.53E-09	chr:12:44313657
12	rs55973711	44167023	A	13.45	1.53E-09	chr:12:44313657
12	rs74890729	44143065	G	13.45	1.53E-09	chr:12:44313657
12	rs74928789	44138445	G	13.45	1.53E-09	chr:12:44313657
12	rs75519831	44113847	T	13.45	1.53E-09	chr:12:44313657
12	rs75860385	44137050	C	13.45	1.53E-09	chr:12:44313657
12	rs76779572	44162600	G	13.45	1.53E-09	chr:12:44313657
12	rs79154645	44173982	T	13.45	1.53E-09	chr:12:44313657
12	rs79595727	44122193	A	13.45	1.53E-09	chr:12:44313657
12	rs79734937	44098365	G	13.45	1.53E-09	chr:12:44313657
12	rs1057190	44148259	C	5.709	2.05E-09	chr:12:44313657
12	rs10880557	44131697	C	5.709	2.05E-09	chr:12:44313657
12	rs10880563	44141593	T	5.709	2.05E-09	chr:12:44313657
12	rs10880566	44146392	G	5.709	2.05E-09	chr:12:44313657
12	rs11182246	44139805	T	5.709	2.05E-09	chr:12:44313657

12	rs11182249	44145654	C	5.709	2.05E-09	chr:12:44313657
12	rs12312327	44134417	C	5.709	2.05E-09	chr:12:44313657
12	rs12319024	44135647	A	5.709	2.05E-09	chr:12:44313657
12	rs7311465	44141164	T	5.709	2.05E-09	chr:12:44313657
12	rs7969729	44142547	A	5.709	2.05E-09	chr:12:44313657
12	rs9783418	44144643	A	5.709	2.05E-09	chr:12:44313657
12	rs4251497	44171131	A	5.469	2.52E-09	chr:12:44313657
12	rs4251495	44171096	G	5.29	2.83E-09	chr:12:44313657
12	rs4251496	44171097	C	5.29	2.83E-09	chr:12:44313657
12	rs3794262	44165430	A	5.349	3.14E-09	chr:12:44313657
12	rs3805198	44175934	T	5.349	3.14E-09	chr:12:44313657
12	rs4251459	44162566	C	5.349	3.14E-09	chr:12:44313657
12	rs4251460	44162768	C	5.349	3.14E-09	chr:12:44313657
12	rs4251465	44164169	T	5.349	3.14E-09	chr:12:44313657
12	rs4251532	44178083	T	5.349	3.14E-09	chr:12:44313657
12	rs4251533	44178175	G	5.349	3.14E-09	chr:12:44313657
12	rs4251555	44182312	C	5.349	3.14E-09	chr:12:44313657
12	rs11182250	44147174	G	5.342	5.28E-09	chr:12:44313657
12	rs11182242	44135219	C	5.389	5.84E-09	chr:12:44313657
12	rs7954843	44113010	A	4.566	1.61E-08	chr:12:44313657
12	rs4251489	44169945	T	5.287	1.93E-08	chr:12:44313657
12	rs7955148	44301433	C	5.856	2.16E-08	chr:12:44313657
12	rs2464103	44279211	T	12.71	2.69E-08	chr:12:44313657
12	rs544503047	44291853	G	9.136	2.94E-08	chr:12:44313657
12	rs4251463	44163295	T	5.086	3.17E-08	chr:12:44313657
12	rs4251545	44180295	A	5.086	3.17E-08	chr:12:44313657
12	rs10880565	44146248	C	4.635	3.78E-08	chr:12:44313657
12	rs4768054	44309743	A	6.235	3.89E-08	chr:12:44313657
12	rs4251540	44179579	T	4.987	4.21E-08	chr:12:44313657
12	rs59709721	44362347	A	27.51	4.65E-08	chr:12:44313657
12	rs10880562	44140311	G	4.605	5.55E-08	chr:12:44313657
12	rs11182240	44134755	C	4.605	5.55E-08	chr:12:44313657
12	rs12308611	44132831	G	4.605	5.55E-08	chr:12:44313657
12	rs12310585	44133972	G	4.605	5.55E-08	chr:12:44313657
12	rs11182332	44317599	A	30.91	6.42E-08	chr:12:44313657
12	rs73085705	44345490	T	30.91	6.42E-08	chr:12:44313657
12	rs10880558	44132250	A	4.536	6.97E-08	chr:12:44313657
12	rs10785489	44364261	C	5.999	7.14E-08	chr:12:44313657
12	rs144535012	44182278	A	7.636	7.32E-08	chr:12:44313657
12	rs12311962	44237272	A	5.92	8.20E-08	chr:12:44313657
12	rs12314126	44236455	G	5.92	8.20E-08	chr:12:44313657
12	rs1731422	44276369	G	9.327	8.85E-08	chr:12:44313657
12	rs12302873	44146579	T	4.352	1.04E-07	chr:12:44313657

12	rs10880559	44137338	G	4.538	1.20E-07	chr:12:44313657
12	rs10880560	44137382	C	4.538	1.20E-07	chr:12:44313657
12	rs10880561	44137426	T	4.538	1.20E-07	chr:12:44313657
12	rs73098836	44286253	A	9.769	1.26E-07	chr:12:44313657
12	rs7957146	44306759	G	9.769	1.26E-07	chr:12:44313657
12	rs7958314	44302430	T	9.769	1.26E-07	chr:12:44313657
12	rs7954870	44143149	A	4.374	1.28E-07	chr:12:44313657
12	rs4251466	44164441	T	4.598	1.66E-07	chr:12:44313657
12	rs11182294	44231539	T	9.916	2.24E-07	chr:12:44313657
12	rs4251473	44166318	A	4.413	3.72E-07	chr:12:44313657
12	rs11182419	44500239	T	9.822	3.73E-07	chr:12:44313657
12	rs4251431	44154690	T	4.273	3.96E-07	chr:12:44313657
12	rs10880583	44305868	T	0.2112	4.47E-07	chr:12:44313657
12	rs1675778	44263162	G	5.428	4.59E-07	chr:12:44313657
12	rs1675760	44282981	G	8.182	4.73E-07	chr:12:44313657
12	rs1675786	44292760	C	8.182	4.73E-07	chr:12:44313657
12	rs1675791	44290267	G	8.182	4.73E-07	chr:12:44313657
12	rs1731425	44282630	T	8.182	4.73E-07	chr:12:44313657
12	rs7966024	44297013	G	8.182	4.73E-07	chr:12:44313657
12	rs148798873	44653722	G	9.509	1.07E-06	chr:12:44313657
12	rs10748373	44253386	A	5.024	1.14E-06	chr:12:44313657
12	rs1675771	44252960	A	5.024	1.14E-06	chr:12:44313657
12	rs1675774	44266575	G	5.024	1.14E-06	chr:12:44313657
12	rs1675776	44265242	A	5.024	1.14E-06	chr:12:44313657
12	rs1675777	44264215	G	5.024	1.14E-06	chr:12:44313657
12	rs1675782	44258841	C	5.024	1.14E-06	chr:12:44313657
12	rs1675784	44257601	A	5.024	1.14E-06	chr:12:44313657
12	rs1675788	44273779	C	5.024	1.14E-06	chr:12:44313657
12	rs1731439	44266559	G	5.024	1.14E-06	chr:12:44313657
12	rs1731440	44265605	T	5.024	1.14E-06	chr:12:44313657
12	rs2447115	44254494	A	5.024	1.14E-06	chr:12:44313657
12	rs2447116	44262529	A	5.024	1.14E-06	chr:12:44313657
12	rs2951436	44261618	A	5.024	1.14E-06	chr:12:44313657
12	rs4251513	44174204	C	3.256	1.95E-06	chr:12:44313657
12	rs11830410	44322978	C	28.45	2.15E-06	chr:12:44313657
12	rs12371940	44319060	C	28.45	2.15E-06	chr:12:44313657
12	rs73100979	44345424	T	28.45	2.15E-06	chr:12:44313657
12	rs7976722	44312149	T	28.45	2.15E-06	chr:12:44313657
12	rs11182287	44218539	C	21.25	2.59E-06	chr:12:44313657
12	rs1675775	44265524	A	5.584	4.24E-06	chr:12:44313657
12	rs1731441	44265523	G	5.584	4.24E-06	chr:12:44313657
12	rs1731449	44244877	T	5.584	4.24E-06	chr:12:44313657
12	rs1731453	44250528	G	5.584	4.24E-06	chr:12:44313657

12	rs7953229	44239350	T	5.584	4.24E-06	chr:12:44313657
12	rs7965635	44238903	A	5.584	4.24E-06	chr:12:44313657
12	rs149139340	44304397	T	43.29	4.43E-06	chr:12:44313657
12	rs7315408	44308709	C	43.29	4.43E-06	chr:12:44313657
12	rs11182371	44394939	C	26.57	4.74E-06	chr:12:44313657
12	rs12320155	44394433	T	26.57	4.74E-06	chr:12:44313657
12	rs2407782	44278489	A	3.061	4.96E-06	chr:12:44313657
12	rs11608602	44215732	C	5.472	5.02E-06	chr:12:44313657
12	rs11182363	44381348	C	18.52	6.61E-06	chr:12:44313657
12	rs10082995	44232340	T	5.335	6.62E-06	chr:12:44313657
12	rs2699992	44278013	C	5.283	8.24E-06	chr:12:44313657
12	rs145822768	44350947	T	33.88	9.06E-06	chr:12:44313657
12	rs111789075	44352673	C	10.97	9.95E-06	chr:12:44313657
12	rs11182348	44354835	C	10.97	9.95E-06	chr:12:44313657
12	rs11182353	44361688	T	10.97	9.95E-06	chr:12:44313657
12	rs11182354	44361712	G	10.97	9.95E-06	chr:12:44313657
12	rs11182355	44362091	G	10.97	9.95E-06	chr:12:44313657
12	rs11182359	44363956	T	10.97	9.95E-06	chr:12:44313657
12	rs1675766	44244919	C	5.596	1.07E-05	chr:12:44313657
12	rs112350998	44141631	A	9.694	1.08E-05	chr:12:44313657
12	rs1842044	44113667	G	2.846	1.25E-05	chr:12:44313657
12	rs2083956	44100278	G	2.846	1.25E-05	chr:12:44313657
12	rs2407659	44099786	C	2.846	1.25E-05	chr:12:44313657
12	rs6582480	44103230	A	2.846	1.25E-05	chr:12:44313657
12	rs11182284	44217312	A	5.038	1.56E-05	chr:12:44313657
12	rs11182286	44217342	C	5.038	1.56E-05	chr:12:44313657
12	rs12230695	44215935	G	5.038	1.56E-05	chr:12:44313657
12	rs11182221	44101967	G	2.739	2.14E-05	chr:12:44313657
12	rs1585716	44103302	G	2.739	2.14E-05	chr:12:44313657
12	rs1814173	44101092	A	2.739	2.14E-05	chr:12:44313657
12	rs1824817	44098594	G	2.739	2.14E-05	chr:12:44313657
12	rs6582481	44103477	T	2.739	2.14E-05	chr:12:44313657
12	rs10506233	44376031	C	11.1	2.21E-05	chr:12:44313657
12	rs547527570	44301398	C	0.2518	2.46E-05	chr:12:44313657
12	rs7485053	55770126	C	0.1152	8.23E-11	chr:12:55727213
12	rs61957915	55742266	C	0.12	8.87E-11	chr:12:55727213
12	rs59466585	55722736	G	0.1119	9.23E-11	chr:12:55727213
12	rs61957887	55721734	T	0.1119	9.23E-11	chr:12:55727213
12	rs7960760	55722311	G	0.1119	9.23E-11	chr:12:55727213
12	rs7971053	55729947	C	0.1119	9.23E-11	chr:12:55727213
12	rs7484522	55738240	G	0.1243	1.02E-10	chr:12:55727213
12	rs11171416	55731490	G	0.1204	1.02E-10	chr:12:55727213
12	rs11831714	55741742	G	0.1204	1.02E-10	chr:12:55727213

12	rs12099811	55731200	A	0.1204	1.02E-10	chr:12:55727213
12	rs55659159	55747754	T	0.1204	1.02E-10	chr:12:55727213
12	rs59136392	55748204	T	0.1204	1.02E-10	chr:12:55727213
12	rs59676560	55745425	C	0.1204	1.02E-10	chr:12:55727213
12	rs61957898	55730423	C	0.1204	1.02E-10	chr:12:55727213
12	rs61957924	55744100	C	0.1204	1.02E-10	chr:12:55727213
12	rs61957933	55744710	G	0.1204	1.02E-10	chr:12:55727213
12	rs61957936	55745719	G	0.1204	1.02E-10	chr:12:55727213
12	rs7314118	55737403	C	0.1204	1.02E-10	chr:12:55727213
12	rs7955088	55732963	T	0.1204	1.02E-10	chr:12:55727213
12	rs61957902	55738064	C	0.1208	1.16E-10	chr:12:55727213
12	rs7960719	55722035	A	0.1122	1.41E-10	chr:12:55727213
12	rs11171435	55758053	C	0.1217	2.02E-10	chr:12:55727213
12	rs12318683	55755024	C	0.1217	2.02E-10	chr:12:55727213
12	rs146150551	55751301	C	0.1217	2.02E-10	chr:12:55727213
12	rs56677471	55749967	A	0.1217	2.02E-10	chr:12:55727213
12	rs59347627	55758129	G	0.1217	2.02E-10	chr:12:55727213
12	rs61957943	55749330	G	0.1217	2.02E-10	chr:12:55727213
12	rs79104523	55749220	C	0.1217	2.02E-10	chr:12:55727213
12	rs537017437	55742569	T	0.1322	2.09E-10	chr:12:55727213
12	rs6581037	55767310	T	0.1238	2.10E-10	chr:12:55727213
12	rs57757372	55718780	G	0.1234	2.10E-10	chr:12:55727213
12	rs58709231	55719090	C	0.1234	2.10E-10	chr:12:55727213
12	rs59097302	55719030	T	0.1234	2.10E-10	chr:12:55727213
12	rs60142559	55719192	G	0.1234	2.10E-10	chr:12:55727213
12	rs60220253	55717410	G	0.1234	2.10E-10	chr:12:55727213
12	rs61957879	55716971	C	0.1234	2.10E-10	chr:12:55727213
12	rs12296692	55756851	C	0.124	2.49E-10	chr:12:55727213
12	rs149898597	55753436	A	0.123	2.49E-10	chr:12:55727213
12	rs7310335	55884439	T	0.1348	2.54E-10	chr:12:55727213
12	rs61249728	55728922	G	0.1222	2.64E-10	chr:12:55727213
12	rs7311751	55748847	G	0.1228	2.68E-10	chr:12:55727213
12	rs55898514	55736238	A	0.1334	3.48E-10	chr:12:55727213
12	rs61957899	55731971	T	0.1334	3.48E-10	chr:12:55727213
12	rs73118998	55736659	C	0.1334	3.48E-10	chr:12:55727213
12	rs73119000	55736662	A	0.1334	3.48E-10	chr:12:55727213
12	rs7313465	55736863	T	0.1334	3.48E-10	chr:12:55727213
12	rs7484556	55738427	G	0.1334	3.48E-10	chr:12:55727213
12	rs201554506	55826869	A	0.1268	3.63E-10	chr:12:55727213
12	rs112331945	55781657	T	0.1312	4.47E-10	chr:12:55727213
12	rs61566486	55781694	G	0.1312	4.47E-10	chr:12:55727213
12	rs115471673	55766516	G	0.1348	4.49E-10	chr:12:55727213
12	rs12099665	55731191	T	0.1348	4.49E-10	chr:12:55727213

12	rs12099808	55731178	A	0.1348	4.49E-10	chr:12:55727213
12	rs12578879	55797773	T	0.1348	4.49E-10	chr:12:55727213
12	rs12579346	55786856	G	0.1348	4.49E-10	chr:12:55727213
12	rs12580661	55803129	A	0.1348	4.49E-10	chr:12:55727213
12	rs12581060	55797484	C	0.1348	4.49E-10	chr:12:55727213
12	rs12581916	55764409	C	0.1348	4.49E-10	chr:12:55727213
12	rs12582005	55772837	A	0.1348	4.49E-10	chr:12:55727213
12	rs189794726	55743934	C	0.1348	4.49E-10	chr:12:55727213
12	rs3935215	55792401	A	0.1348	4.49E-10	chr:12:55727213
12	rs4448777	55724981	C	0.1348	4.49E-10	chr:12:55727213
12	rs4590978	55725086	T	0.1348	4.49E-10	chr:12:55727213
12	rs55637917	55747565	C	0.1348	4.49E-10	chr:12:55727213
12	rs58254871	55750281	A	0.1348	4.49E-10	chr:12:55727213
12	rs58780687	55736498	C	0.1348	4.49E-10	chr:12:55727213
12	rs59213907	55728911	T	0.1348	4.49E-10	chr:12:55727213
12	rs60230359	55736550	C	0.1348	4.49E-10	chr:12:55727213
12	rs60249205	55758333	G	0.1348	4.49E-10	chr:12:55727213
12	rs60586348	55745151	T	0.1348	4.49E-10	chr:12:55727213
12	rs60774348	55757717	G	0.1348	4.49E-10	chr:12:55727213
12	rs61556775	55745640	C	0.1348	4.49E-10	chr:12:55727213
12	rs61956269	55768023	T	0.1348	4.49E-10	chr:12:55727213
12	rs61956272	55779559	T	0.1348	4.49E-10	chr:12:55727213
12	rs61956275	55786423	A	0.1348	4.49E-10	chr:12:55727213
12	rs61956290	55793833	G	0.1348	4.49E-10	chr:12:55727213
12	rs61956291	55797964	C	0.1348	4.49E-10	chr:12:55727213
12	rs61956292	55803346	C	0.1348	4.49E-10	chr:12:55727213
12	rs61957937	55746850	C	0.1348	4.49E-10	chr:12:55727213
12	rs61957938	55747266	G	0.1348	4.49E-10	chr:12:55727213
12	rs61957941	55748058	A	0.1348	4.49E-10	chr:12:55727213
12	rs61957946	55755778	C	0.1348	4.49E-10	chr:12:55727213
12	rs66902680	55724080	C	0.1348	4.49E-10	chr:12:55727213
12	rs67015069	55759968	A	0.1348	4.49E-10	chr:12:55727213
12	rs67934812	55785591	G	0.1348	4.49E-10	chr:12:55727213
12	rs73326725	55749222	C	0.1348	4.49E-10	chr:12:55727213
12	rs74844018	55766871	A	0.1348	4.49E-10	chr:12:55727213
12	rs7485979	55789043	A	0.1348	4.49E-10	chr:12:55727213
12	rs7485991	55789129	C	0.1348	4.49E-10	chr:12:55727213
12	rs7487419	55769801	G	0.1348	4.49E-10	chr:12:55727213
12	rs7487895	55796720	C	0.1348	4.49E-10	chr:12:55727213
12	rs7488940	55802591	T	0.1348	4.49E-10	chr:12:55727213
12	rs7489047	55759851	G	0.1348	4.49E-10	chr:12:55727213
12	rs7959124	55726957	G	0.1348	4.49E-10	chr:12:55727213
12	rs7976828	55726450	T	0.1348	4.49E-10	chr:12:55727213

12	rs7959837	55734511	A	0.1371	4.64E-10	chr:12:55727213
12	rs61957765	55868561	C	0.1351	4.95E-10	chr:12:55727213
12	rs67290413	55869876	C	0.1351	4.95E-10	chr:12:55727213
12	rs7294659	55866519	C	0.1351	4.95E-10	chr:12:55727213
12	rs61957914	55742041	A	0.1401	5.52E-10	chr:12:55727213
12	rs12579408	55764348	A	0.1374	5.59E-10	chr:12:55727213
12	rs61071395	55721228	C	0.1336	6.35E-10	chr:12:55727213
12	rs11171431	55757609	T	0.1349	6.61E-10	chr:12:55727213
12	rs11171432	55757696	C	0.1349	6.61E-10	chr:12:55727213
12	rs11171434	55758018	T	0.1349	6.61E-10	chr:12:55727213
12	rs11832308	55755506	A	0.1349	6.61E-10	chr:12:55727213
12	rs13377995	55757380	G	0.1349	6.61E-10	chr:12:55727213
12	rs200704134	55879280	T	0.1402	6.82E-10	chr:12:55727213
12	rs201855666	55879919	A	0.1402	6.82E-10	chr:12:55727213
12	rs61957788	55883248	A	0.1356	7.09E-10	chr:12:55727213
12	rs60991522	55781529	T	0.1346	7.09E-10	chr:12:55727213
12	rs142753382	55872931	T	0.1534	7.81E-10	chr:12:55727213
12	rs12582120	55830064	G	0.1384	7.89E-10	chr:12:55727213
12	rs201409163	55827180	C	0.1384	7.89E-10	chr:12:55727213
12	rs4318592	55837124	A	0.1384	7.89E-10	chr:12:55727213
12	rs55797706	55836585	A	0.1384	7.89E-10	chr:12:55727213
12	rs61956325	55827707	A	0.1384	7.89E-10	chr:12:55727213
12	rs61956326	55827735	A	0.1384	7.89E-10	chr:12:55727213
12	rs61956327	55828708	T	0.1384	7.89E-10	chr:12:55727213
12	rs6581047	55832001	G	0.1384	7.89E-10	chr:12:55727213
12	rs7311929	55834833	G	0.1384	7.89E-10	chr:12:55727213
12	rs7957446	55839631	C	0.1384	7.89E-10	chr:12:55727213
12	rs11171430	55755254	G	0.1433	8.32E-10	chr:12:55727213
12	rs12578577	55716443	T	0.14	8.48E-10	chr:12:55727213
12	rs58480409	55719221	T	0.14	8.48E-10	chr:12:55727213
12	rs61957878	55716754	T	0.14	8.48E-10	chr:12:55727213
12	rs61957884	55720177	G	0.14	8.48E-10	chr:12:55727213
12	rs61957885	55720876	G	0.14	8.48E-10	chr:12:55727213
12	rs4130004	55860773	T	0.1432	8.87E-10	chr:12:55727213
12	rs4343668	55868936	T	0.1432	8.87E-10	chr:12:55727213
12	rs4356858	55867290	T	0.1432	8.87E-10	chr:12:55727213
12	rs56840674	55887691	T	0.1432	8.87E-10	chr:12:55727213
12	rs60066834	55877998	T	0.1432	8.87E-10	chr:12:55727213
12	rs60450959	55861373	T	0.1432	8.87E-10	chr:12:55727213
12	rs60683621	55863224	G	0.1432	8.87E-10	chr:12:55727213
12	rs61957764	55867916	A	0.1432	8.87E-10	chr:12:55727213
12	rs7304753	55886489	A	0.1432	8.87E-10	chr:12:55727213
12	rs61956300	55812640	G	0.1394	9.46E-10	chr:12:55727213

12	rs12579852	55716569	A	0.144	9.98E-10	chr:12:55727213
12	rs60873827	55717769	C	0.144	9.98E-10	chr:12:55727213
12	rs61957763	55867540	A	0.1436	1.03E-09	chr:12:55727213
12	rs60643523	55842928	G	0.139	1.10E-09	chr:12:55727213
12	rs61956331	55840856	A	0.139	1.10E-09	chr:12:55727213
12	rs7966770	55844717	C	0.139	1.10E-09	chr:12:55727213
12	rs7971742	55848486	G	0.139	1.10E-09	chr:12:55727213
12	rs7980098	55844694	G	0.139	1.10E-09	chr:12:55727213
12	rs61957760	55859957	T	0.146	1.18E-09	chr:12:55727213
12	rs11171433	55757797	T	0.1115	1.21E-09	chr:12:55727213
12	rs145535298	55873030	C	0.1539	1.25E-09	chr:12:55727213
12	rs4376077	55872442	G	0.1539	1.25E-09	chr:12:55727213
12	rs66516917	55749131	G	0.1393	1.33E-09	chr:12:55727213
12	rs12579181	55887087	T	0.1393	1.43E-09	chr:12:55727213
12	rs59003108	55888940	T	0.1393	1.43E-09	chr:12:55727213
12	rs7316594	55887407	A	0.1393	1.43E-09	chr:12:55727213
12	rs59438785	55811665	G	0.1493	1.69E-09	chr:12:55727213
12	rs61547125	55814082	G	0.1493	1.69E-09	chr:12:55727213
12	rs61956303	55818071	A	0.1493	1.69E-09	chr:12:55727213
12	rs57361193	55883572	A	0.1433	1.73E-09	chr:12:55727213
12	rs57050414	55776926	A	0.1474	1.75E-09	chr:12:55727213
12	rs4453888	55887808	C	0.1392	1.79E-09	chr:12:55727213
12	rs112892991	55808749	T	0.1502	1.99E-09	chr:12:55727213
12	rs12579005	55853230	A	0.1499	2.01E-09	chr:12:55727213
12	rs61956358	55852286	T	0.1499	2.01E-09	chr:12:55727213
12	rs4309772	55888672	C	0.151	2.05E-09	chr:12:55727213
12	rs4479639	55888719	G	0.1521	3.23E-09	chr:12:55727213
12	rs67246762	55889642	T	0.1678	4.25E-09	chr:12:55727213
12	rs143455470	55879325	A	0.1788	5.64E-09	chr:12:55727213
12	rs12581959	55787311	A	0.1766	1.64E-08	chr:12:55727213
12	rs11171583	55980116	G	0.1807	2.19E-08	chr:12:55727213
12	rs11534917	55984049	C	0.1807	2.19E-08	chr:12:55727213
12	rs11536179	55997655	C	0.1807	2.19E-08	chr:12:55727213
12	rs58285384	55981220	A	0.1807	2.19E-08	chr:12:55727213
12	rs9669106	55975508	C	0.1807	2.19E-08	chr:12:55727213
12	rs201131895	55740725	A	0.2114	3.14E-08	chr:12:55727213
12	rs376987469	55740755	G	0.2114	3.14E-08	chr:12:55727213
12	rs148541886	55996016	G	0.2118	5.77E-08	chr:12:55727213
12	rs11171610	55998903	T	0.1939	6.93E-08	chr:12:55727213
12	rs11531173	55987253	G	0.1939	6.93E-08	chr:12:55727213
12	rs11531175	55993045	T	0.1939	6.93E-08	chr:12:55727213
12	rs11532451	55993192	T	0.1939	6.93E-08	chr:12:55727213
12	rs11537921	55985909	T	0.1939	6.93E-08	chr:12:55727213

12	rs11537924	55986259	C	0.1939	6.93E-08	chr:12:55727213
12	rs9668674	55975665	C	0.1939	6.93E-08	chr:12:55727213
12	rs1316899	55966101	A	0.2244	1.96E-07	chr:12:55727213
12	rs61957868	55695527	A	0.283	5.95E-07	chr:12:55727213
12	rs373941107	55740754	T	0.219	9.27E-07	chr:12:55727213
12	rs12579030	55689194	C	0.2728	1.02E-06	chr:12:55727213
12	rs12580194	55696558	A	0.2728	1.02E-06	chr:12:55727213
12	rs12582734	55689572	A	0.2728	1.02E-06	chr:12:55727213
12	rs7971451	55699950	A	0.2728	1.02E-06	chr:12:55727213
12	rs4262806	55711562	A	0.2753	1.22E-06	chr:12:55727213
12	rs58332180	55711353	A	0.2753	1.22E-06	chr:12:55727213
12	rs7308316	55709162	A	0.2753	1.22E-06	chr:12:55727213
12	rs12579915	55696789	C	0.2938	1.25E-06	chr:12:55727213
12	rs4491341	55697838	A	0.2938	1.25E-06	chr:12:55727213
12	rs56380243	55690727	G	0.2938	1.25E-06	chr:12:55727213
12	rs58408480	55699186	T	0.2938	1.25E-06	chr:12:55727213
12	rs61539173	55703206	T	0.2938	1.25E-06	chr:12:55727213
12	rs7968881	55694434	C	0.2938	1.25E-06	chr:12:55727213
12	rs7974051	55713210	C	0.3024	2.24E-06	chr:12:55727213
12	rs7974512	55713606	G	0.3128	3.00E-06	chr:12:55727213
12	rs7486167	55783942	T	0.306	4.41E-06	chr:12:55727213
12	rs11171441	55766596	T	0.3291	4.50E-06	chr:12:55727213
12	rs10783728	55762519	G	0.3267	4.58E-06	chr:12:55727213
12	rs10783730	55763463	G	0.3267	4.58E-06	chr:12:55727213
12	rs7133826	55939629	G	0.3293	4.72E-06	chr:12:55727213
12	rs11171523	55901413	T	0.329	4.81E-06	chr:12:55727213
12	rs1124741	55967941	T	0.2401	4.87E-06	chr:12:55727213
12	rs7487315	55782690	C	0.3189	5.43E-06	chr:12:55727213
12	rs7487832	55782814	A	0.3189	5.43E-06	chr:12:55727213
12	rs7487833	55782819	A	0.3189	5.43E-06	chr:12:55727213
12	rs11171576	55972314	C	0.2315	5.56E-06	chr:12:55727213
12	rs7313943	55972705	T	0.2315	5.56E-06	chr:12:55727213
12	rs7294677	55800713	T	0.3212	5.90E-06	chr:12:55727213
12	rs10747746	55769082	T	0.3211	5.99E-06	chr:12:55727213
12	rs10747747	55772188	T	0.3211	5.99E-06	chr:12:55727213
12	rs10747748	55774581	G	0.3211	5.99E-06	chr:12:55727213
12	rs7488140	55770771	G	0.3211	5.99E-06	chr:12:55727213
12	rs7488178	55770918	G	0.3211	5.99E-06	chr:12:55727213
12	rs11171625	56026816	T	0.259	6.22E-06	chr:12:55727213
12	rs10783731	55767702	T	0.3228	6.27E-06	chr:12:55727213
12	rs10876749	55754085	G	0.3228	6.27E-06	chr:12:55727213
12	rs11171437	55761078	A	0.3228	6.27E-06	chr:12:55727213
12	rs10747752	55793235	A	0.3221	6.46E-06	chr:12:55727213

12	rs10783737	55802733	G	0.3221	6.46E-06	chr:12:55727213
12	rs11171449	55789447	A	0.3221	6.46E-06	chr:12:55727213
12	rs28881605	55914354	G	0.3199	7.14E-06	chr:12:55727213
12	rs4759147	55770418	A	0.3357	7.31E-06	chr:12:55727213
12	rs10747743	55755226	A	0.3246	7.38E-06	chr:12:55727213
12	rs10876750	55757559	C	0.3246	7.38E-06	chr:12:55727213
12	rs10876751	55759878	A	0.3246	7.38E-06	chr:12:55727213
12	rs10876754	55767295	G	0.3246	7.38E-06	chr:12:55727213
12	rs11171444	55766896	C	0.3246	7.38E-06	chr:12:55727213
12	rs4758999	55756597	T	0.3246	7.38E-06	chr:12:55727213
12	rs11534885	55993114	C	0.2332	7.53E-06	chr:12:55727213
12	rs11534920	55989137	T	0.2332	7.53E-06	chr:12:55727213
12	rs10735862	55763034	T	0.3349	7.88E-06	chr:12:55727213
12	rs10735861	55762857	G	0.3459	7.92E-06	chr:12:55727213
12	rs10876837	56028546	A	0.2437	7.94E-06	chr:12:55727213
12	rs58316942	55718919	A	0.2488	8.04E-06	chr:12:55727213
12	rs61957889	55724456	T	0.2488	8.04E-06	chr:12:55727213
12	rs10747744	55768014	T	0.3379	8.34E-06	chr:12:55727213
12	rs10747745	55768086	A	0.3379	8.34E-06	chr:12:55727213
12	rs10876753	55767068	T	0.3379	8.34E-06	chr:12:55727213
12	rs4759000	55766037	C	0.3379	8.34E-06	chr:12:55727213
12	rs4759145	55769542	A	0.3379	8.34E-06	chr:12:55727213
12	rs4759146	55770047	T	0.3379	8.34E-06	chr:12:55727213
12	rs12305626	55441747	G	0.2132	8.53E-06	chr:12:55727213
12	rs10783756	55920314	C	0.3442	8.72E-06	chr:12:55727213
12	rs11171521	55901314	T	0.344	8.93E-06	chr:12:55727213
12	rs181265801	55900112	C	0.344	8.93E-06	chr:12:55727213
12	rs7138601	55895730	A	0.344	8.93E-06	chr:12:55727213
12	rs11171427	55742270	T	0.3297	9.02E-06	chr:12:55727213
12	rs11171631	56031611	G	0.2626	9.42E-06	chr:12:55727213
12	rs10431547	55745950	G	0.3303	9.75E-06	chr:12:55727213
12	rs10735860	55732664	C	0.3303	9.75E-06	chr:12:55727213
12	rs10783721	55732060	G	0.3303	9.75E-06	chr:12:55727213
12	rs10783723	55738137	G	0.3303	9.75E-06	chr:12:55727213
12	rs10783724	55746910	G	0.3303	9.75E-06	chr:12:55727213
12	rs10876741	55723575	C	0.3303	9.75E-06	chr:12:55727213
12	rs10876742	55737616	T	0.3303	9.75E-06	chr:12:55727213
12	rs10876747	55745162	A	0.3303	9.75E-06	chr:12:55727213
12	rs4759140	55733080	A	0.3303	9.75E-06	chr:12:55727213
12	rs7138676	55734240	G	0.3303	9.75E-06	chr:12:55727213
12	rs7486665	55741380	A	0.3303	9.75E-06	chr:12:55727213
12	rs9795610	55740956	A	0.3303	9.75E-06	chr:12:55727213
12	rs7960547	55898639	C	0.3453	1.01E-05	chr:12:55727213

12	rs10876806	55914022	G	0.3458	1.02E-05	chr:12:55727213
12	rs11171411	55719478	C	0.3311	1.15E-05	chr:12:55727213
12	rs12301041	55718970	C	0.3311	1.15E-05	chr:12:55727213
12	rs4511372	55722251	C	0.3311	1.15E-05	chr:12:55727213
12	rs7958843	55726716	T	0.3311	1.15E-05	chr:12:55727213
12	rs11543034	55959971	C	0.3472	1.16E-05	chr:12:55727213
12	rs1896044	55953670	A	0.3472	1.16E-05	chr:12:55727213
12	rs1896045	55953568	G	0.3472	1.16E-05	chr:12:55727213
12	rs4302411	55938276	T	0.3472	1.16E-05	chr:12:55727213
12	rs6581066	55926695	A	0.3472	1.16E-05	chr:12:55727213
12	rs7297323	55941692	C	0.3472	1.16E-05	chr:12:55727213
12	rs7963839	55936468	G	0.3472	1.16E-05	chr:12:55727213
12	rs7964180	55957169	G	0.3472	1.16E-05	chr:12:55727213
12	rs4336160	55891377	T	0.3321	1.21E-05	chr:12:55727213
12	rs7304684	55895538	T	0.3321	1.21E-05	chr:12:55727213
12	rs10876767	55797331	C	0.3314	1.22E-05	chr:12:55727213
12	rs7960126	55813605	G	0.257	1.26E-05	chr:12:55727213
12	rs10747742	55748509	A	0.3409	1.32E-05	chr:12:55727213
12	rs11171535	55914635	A	0.3341	1.39E-05	chr:12:55727213
12	rs11532447	55957772	T	0.3341	1.39E-05	chr:12:55727213
12	rs12230513	55916780	G	0.3341	1.39E-05	chr:12:55727213
12	rs139464426	55958278	C	0.3341	1.39E-05	chr:12:55727213
12	rs146073724	55958414	A	0.3341	1.39E-05	chr:12:55727213
12	rs183973202	55935021	A	0.3341	1.39E-05	chr:12:55727213
12	rs76179834	55936746	T	0.3341	1.39E-05	chr:12:55727213
12	rs76783572	55936752	A	0.3341	1.39E-05	chr:12:55727213
12	rs11171556	55949852	A	0.3448	1.63E-05	chr:12:55727213
12	rs4324500	55935029	A	0.3448	1.63E-05	chr:12:55727213
12	rs77755732	55924293	T	0.3448	1.63E-05	chr:12:55727213
12	rs10876836	56028489	T	0.2824	1.74E-05	chr:12:55727213
12	rs11171629	56029005	C	0.2824	1.74E-05	chr:12:55727213
12	rs3922689	55647750	A	0.1994	1.83E-05	chr:12:55727213
12	rs7978249	55649103	A	0.1994	1.83E-05	chr:12:55727213
12	rs11171561	55960184	T	0.3564	1.86E-05	chr:12:55727213
12	rs11171562	55961414	C	0.3564	1.86E-05	chr:12:55727213
12	rs11171569	55967018	G	0.3399	2.13E-05	chr:12:55727213
12	rs10783734	55799682	G	0.3444	2.31E-05	chr:12:55727213
12	rs10876764	55789304	G	0.3444	2.31E-05	chr:12:55727213
12	rs10876768	55797728	A	0.3444	2.31E-05	chr:12:55727213
12	rs10876758	55781712	G	0.3669	2.46E-05	chr:12:55727213
12	rs10876759	55781717	A	0.3669	2.46E-05	chr:12:55727213
12	rs11171550	55933641	C	0.3653	2.52E-05	chr:12:55727213
12	rs7976741	55696028	A	0.382	2.62E-05	chr:12:55727213

12	rs10506341	55448678	A	0.1876	2.73E-05	chr:12:55727213
12	rs12297677	55460614	T	0.1876	2.73E-05	chr:12:55727213
12	rs17116877	55451189	C	0.1876	2.73E-05	chr:12:55727213
12	rs11171426	55741827	T	0.3511	2.74E-05	chr:12:55727213
12	rs11611990	55644365	G	0.1444	2.83E-05	chr:12:55727213
12	rs73322883	55641920	A	0.1444	2.83E-05	chr:12:55727213
12	rs79812609	55645396	A	0.1444	2.83E-05	chr:12:55727213
13	rs112632269	90731067	G	120	3.40E-15	chr:13:90743183
13	rs78721625	90744516	T	120	3.40E-15	chr:13:90743183
13	rs16943826	90710255	C	117.6	1.00E-14	chr:13:90743183
13	rs77191234	90719556	G	117.6	1.00E-14	chr:13:90743183
13	rs75921720	90708825	C	212.2	3.20E-14	chr:13:90743183
13	rs74507286	90738994	T	73.24	3.67E-14	chr:13:90743183
13	rs79785623	90752549	T	67.62	4.68E-14	chr:13:90743183
13	rs2805748	90747577	A	79.88	5.16E-14	chr:13:90743183
13	rs112945355	90680625	T	65.7	1.14E-13	chr:13:90743183
13	rs113169741	90685650	A	65.7	1.14E-13	chr:13:90743183
13	rs116763682	90743619	A	65.7	1.14E-13	chr:13:90743183
13	rs137989181	90676229	T	65.7	1.14E-13	chr:13:90743183
13	rs1987095	90681873	C	65.7	1.14E-13	chr:13:90743183
13	rs1987156	90681662	A	65.7	1.14E-13	chr:13:90743183
13	rs57346044	90704533	C	65.7	1.14E-13	chr:13:90743183
13	rs74565296	90691322	T	65.7	1.14E-13	chr:13:90743183
13	rs75410608	90693188	A	65.7	1.14E-13	chr:13:90743183
13	rs75805202	90735664	G	65.7	1.14E-13	chr:13:90743183
13	rs76202013	90714744	C	65.7	1.14E-13	chr:13:90743183
13	rs76901628	90699709	C	65.7	1.14E-13	chr:13:90743183
13	rs78545676	90677712	G	65.7	1.14E-13	chr:13:90743183
13	rs78645261	90754325	G	65.7	1.14E-13	chr:13:90743183
13	rs78975634	90720218	G	65.7	1.14E-13	chr:13:90743183
13	rs79671279	90682347	T	65.7	1.14E-13	chr:13:90743183
13	rs80014975	90678526	G	65.7	1.14E-13	chr:13:90743183
13	rs80233688	90723496	G	65.7	1.14E-13	chr:13:90743183
13	rs79110442	90748308	G	53.73	3.11E-13	chr:13:90743183
13	rs114498245	90667379	A	57.97	3.38E-13	chr:13:90743183
13	rs115029999	90669505	G	57.97	3.38E-13	chr:13:90743183
13	rs75635330	90668043	C	57.97	3.38E-13	chr:13:90743183
13	rs78585383	90673119	T	57.97	3.38E-13	chr:13:90743183
13	rs78693129	90673027	T	57.97	3.38E-13	chr:13:90743183
13	rs7985369	90671522	A	57.97	3.38E-13	chr:13:90743183
13	rs16943737	90663135	G	48.65	1.22E-12	chr:13:90743183
13	rs16943738	90664242	G	48.65	1.22E-12	chr:13:90743183
13	rs41322051	90646840	G	48.65	1.22E-12	chr:13:90743183

13	rs41361347	90652322	G	48.65	1.22E-12	chr:13:90743183
13	rs74422803	90653835	T	48.65	1.22E-12	chr:13:90743183
13	rs74568779	90660687	A	48.65	1.22E-12	chr:13:90743183
13	rs76111549	90651195	A	48.65	1.22E-12	chr:13:90743183
13	rs76925939	90646777	T	48.65	1.22E-12	chr:13:90743183
13	rs77555222	90662826	C	48.65	1.22E-12	chr:13:90743183
13	rs78076983	90651105	G	48.65	1.22E-12	chr:13:90743183
13	rs78078484	90646090	A	48.65	1.22E-12	chr:13:90743183
13	rs78172895	90663325	A	48.65	1.22E-12	chr:13:90743183
13	rs78641456	90653738	G	48.65	1.22E-12	chr:13:90743183
13	rs78680587	90654218	A	48.65	1.22E-12	chr:13:90743183
13	rs78905293	90647677	T	48.65	1.22E-12	chr:13:90743183
13	rs78992908	90651318	A	48.65	1.22E-12	chr:13:90743183
13	rs79494540	90648492	C	48.65	1.22E-12	chr:13:90743183
13	rs79603316	90660753	A	48.65	1.22E-12	chr:13:90743183
13	rs80350675	90647983	G	48.65	1.22E-12	chr:13:90743183
13	rs2805699	90723189	A	326.7	1.32E-12	chr:13:90743183
13	rs74387961	90647017	A	44.84	1.90E-12	chr:13:90743183
13	rs80247605	90664690	G	43.62	2.15E-12	chr:13:90743183
13	rs76821422	90696710	T	38	1.33E-11	chr:13:90743183
13	rs74593387	90592567	A	35.34	1.34E-11	chr:13:90743183
13	rs117605767	90638149	G	32.85	5.40E-11	chr:13:90743183
13	rs76245692	90623318	T	30.72	5.64E-11	chr:13:90743183
13	rs1341647	90653212	G	29.89	1.31E-10	chr:13:90743183
13	rs1361601	90651537	T	29.89	1.31E-10	chr:13:90743183
13	rs1417931	90652059	C	29.89	1.31E-10	chr:13:90743183
13	rs1417932	90651802	C	29.89	1.31E-10	chr:13:90743183
13	rs1578539	90647385	A	29.89	1.31E-10	chr:13:90743183
13	rs1857016	90653003	C	29.89	1.31E-10	chr:13:90743183
13	rs1935181	90649956	C	29.89	1.31E-10	chr:13:90743183
13	rs1935182	90649766	T	29.89	1.31E-10	chr:13:90743183
13	rs2805658	90652699	A	29.89	1.31E-10	chr:13:90743183
13	rs2805659	90652722	A	29.89	1.31E-10	chr:13:90743183
13	rs4773545	90650689	T	29.89	1.31E-10	chr:13:90743183
13	rs6492467	90646581	T	29.89	1.31E-10	chr:13:90743183
13	rs6492468	90650308	T	29.89	1.31E-10	chr:13:90743183
13	rs6492469	90650326	A	29.89	1.31E-10	chr:13:90743183
13	rs7987787	90648902	A	29.89	1.31E-10	chr:13:90743183
13	rs7987946	90648926	A	29.89	1.31E-10	chr:13:90743183
13	rs7987947	90648931	A	29.89	1.31E-10	chr:13:90743183
13	rs9522811	90647140	C	29.89	1.31E-10	chr:13:90743183
13	rs1341654	90661565	A	25.67	1.32E-10	chr:13:90743183
13	rs76200599	90606676	T	26.97	2.60E-10	chr:13:90743183

13	rs7321841	90645244	A	27.29	2.99E-10	chr:13:90743183
13	rs7326085	90645471	C	27.29	2.99E-10	chr:13:90743183
13	rs7322297	90720522	A	683.2	1.60E-09	chr:13:90743183
13	rs1491024	90715598	G	28.82	2.95E-09	chr:13:90743183
13	rs9588850	90717634	A	28.82	2.95E-09	chr:13:90743183
13	rs9588852	90719948	G	28.82	2.95E-09	chr:13:90743183
13	rs1987096	90681823	T	10.08	6.31E-08	chr:13:90743183
13	rs11842540	90634897	G	10.35	1.47E-07	chr:13:90743183
13	rs11842101	90633545	G	10.14	1.65E-07	chr:13:90743183
13	rs76649938	90799618	T	16.23	1.94E-07	chr:13:90743183
13	rs11620232	90642326	G	9.934	2.04E-07	chr:13:90743183
13	rs11841913	90637307	T	9.934	2.04E-07	chr:13:90743183
13	rs12855674	90638634	G	9.934	2.04E-07	chr:13:90743183
13	rs12857525	90638814	G	9.934	2.04E-07	chr:13:90743183
13	rs34788777	90640624	G	9.934	2.04E-07	chr:13:90743183
13	rs35159467	90631767	A	9.934	2.04E-07	chr:13:90743183
13	rs7322351	90639552	T	9.934	2.04E-07	chr:13:90743183
13	rs2805689	90715153	G	6.953	2.88E-07	chr:13:90743183
13	rs186610268	90795129	A	14.2	3.63E-07	chr:13:90743183
13	rs1602410	90699029	A	7.397	4.94E-07	chr:13:90743183
13	rs4142563	90722674	G	7.041	5.76E-07	chr:13:90743183
13	rs77533677	90798005	T	12.86	5.77E-07	chr:13:90743183
13	rs2637398	90722150	T	5.837	6.09E-07	chr:13:90743183
13	rs2637421	90740082	T	5.837	6.09E-07	chr:13:90743183
13	rs2637426	90741000	A	5.837	6.09E-07	chr:13:90743183
13	rs2637439	90732217	G	5.837	6.09E-07	chr:13:90743183
13	rs2805704	90727635	A	5.837	6.09E-07	chr:13:90743183
13	rs2805728	90742666	A	5.837	6.09E-07	chr:13:90743183
13	rs8001179	90613977	G	8.84	7.94E-07	chr:13:90743183
13	rs12858780	90735185	T	6.004	1.16E-06	chr:13:90743183
13	rs1491012	90738253	C	6.004	1.16E-06	chr:13:90743183
13	rs1586740	90747887	C	6.004	1.16E-06	chr:13:90743183
13	rs2350300	90733811	G	6.004	1.16E-06	chr:13:90743183
13	rs35414148	90749088	A	6.004	1.16E-06	chr:13:90743183
13	rs3864991	90752677	G	6.004	1.16E-06	chr:13:90743183
13	rs4093505	90755052	G	6.004	1.16E-06	chr:13:90743183
13	rs4771804	90756276	G	6.004	1.16E-06	chr:13:90743183
13	rs4773550	90754699	T	6.004	1.16E-06	chr:13:90743183
13	rs7330958	90724514	T	6.004	1.16E-06	chr:13:90743183
13	rs7992099	90737810	G	6.004	1.16E-06	chr:13:90743183
13	rs7994352	90737852	A	6.004	1.16E-06	chr:13:90743183
13	rs9515679	90723917	T	6.004	1.16E-06	chr:13:90743183
13	rs9515680	90726064	G	6.004	1.16E-06	chr:13:90743183

13	rs9515684	90735792	A	6.004	1.16E-06	chr:13:90743183
13	rs9522843	90735691	A	6.004	1.16E-06	chr:13:90743183
13	rs9522844	90738863	T	6.004	1.16E-06	chr:13:90743183
13	rs9560547	90725963	T	6.004	1.16E-06	chr:13:90743183
13	rs113355173	90805755	A	12.7	1.25E-06	chr:13:90743183
13	rs74734778	90805574	G	12.7	1.25E-06	chr:13:90743183
13	rs11619601	90630160	G	7.942	1.51E-06	chr:13:90743183
13	rs11839415	90579846	T	7.942	1.51E-06	chr:13:90743183
13	rs12854096	90614128	G	7.942	1.51E-06	chr:13:90743183
13	rs12872730	90628330	T	7.942	1.51E-06	chr:13:90743183
13	rs17633242	90603523	A	7.942	1.51E-06	chr:13:90743183
13	rs34072176	90595015	G	7.942	1.51E-06	chr:13:90743183
13	rs34721005	90628659	C	7.942	1.51E-06	chr:13:90743183
13	rs35019682	90600049	G	7.942	1.51E-06	chr:13:90743183
13	rs35152474	90623202	T	7.942	1.51E-06	chr:13:90743183
13	rs36012183	90604597	C	7.942	1.51E-06	chr:13:90743183
13	rs67181673	90624858	G	7.942	1.51E-06	chr:13:90743183
13	rs67842803	90583574	G	7.942	1.51E-06	chr:13:90743183
13	rs950956	90581710	C	7.942	1.51E-06	chr:13:90743183
13	rs2637400	90723289	T	159	1.91E-06	chr:13:90743183
13	rs2805701	90723284	A	159	1.91E-06	chr:13:90743183
13	rs12870592	90616121	T	7.599	2.48E-06	chr:13:90743183
13	rs4357809	90752644	C	5.304	2.86E-06	chr:13:90743183
13	rs7991374	90713284	G	4.383	6.14E-06	chr:13:90743183
13	rs1388186	90720423	A	4.86	6.70E-06	chr:13:90743183
13	rs8001217	90717865	C	4.86	6.70E-06	chr:13:90743183
13	rs9515678	90718301	A	4.86	6.70E-06	chr:13:90743183
13	rs9522836	90718114	T	4.86	6.70E-06	chr:13:90743183
13	rs113524432	90747494	T	33.38	7.27E-06	chr:13:90743183
13	rs113894290	90729661	C	3450	1.54E-05	chr:13:90743183
13	rs1388190	90739471	A	3450	1.54E-05	chr:13:90743183
13	rs112552157	90523437	T	27.13	2.22E-05	chr:13:90743183
13	rs112892478	90523407	A	27.13	2.22E-05	chr:13:90743183
14	rs78783511	20555396	C	19.14	8.21E-16	chr:14:20552746
14	rs7153404	20550111	C	14.36	3.74E-15	chr:14:20552746
14	rs58368472	20561378	T	15.77	3.82E-15	chr:14:20552746
14	rs4144238	20561971	C	14.96	3.86E-15	chr:14:20552746
14	rs12590521	20558876	T	14.34	3.91E-15	chr:14:20552746
14	rs4144240	20561753	C	15.57	3.99E-15	chr:14:20552746
14	rs12588910	20558938	C	16.15	4.06E-15	chr:14:20552746
14	rs12890274	20560354	T	17.07	4.20E-15	chr:14:20552746
14	rs4144239	20561922	C	17.45	4.38E-15	chr:14:20552746
14	rs57793822	20561481	A	17.45	4.38E-15	chr:14:20552746

14	rs969774	20562080	T	17.45	4.38E-15	chr:14:20552746
14	rs7152272	20559234	A	20.27	4.45E-15	chr:14:20552746
14	rs11158661	20556366	T	19.62	4.67E-15	chr:14:20552746
14	rs12433363	20561341	A	17.5	4.67E-15	chr:14:20552746
14	rs4981728	20560512	C	24.72	4.98E-15	chr:14:20552746
14	rs12586923	20559937	A	20.13	5.21E-15	chr:14:20552746
14	rs12587536	20559906	T	20.13	5.21E-15	chr:14:20552746
14	rs7158086	20559451	T	20.13	5.21E-15	chr:14:20552746
14	rs12881697	20560434	G	20.2	5.50E-15	chr:14:20552746
14	rs7148411	20559282	G	20.2	5.50E-15	chr:14:20552746
14	rs4297617	20557961	T	31.3	6.38E-15	chr:14:20552746
14	rs4360850	20558013	A	31.3	6.38E-15	chr:14:20552746
14	rs4438225	20557810	A	31.3	6.38E-15	chr:14:20552746
14	rs4572311	20557971	C	31.3	6.38E-15	chr:14:20552746
14	rs1958722	20562590	G	24.7	6.51E-15	chr:14:20552746
14	rs10136466	20550347	C	40.08	8.28E-15	chr:14:20552746
14	rs11158659	20556212	A	38.61	1.21E-14	chr:14:20552746
14	rs11158660	20556259	C	38.61	1.21E-14	chr:14:20552746
14	rs12434437	20556447	T	38.61	1.21E-14	chr:14:20552746
14	rs12434463	20556513	T	38.61	1.21E-14	chr:14:20552746
14	rs6573756	20555675	G	38.61	1.21E-14	chr:14:20552746
14	rs6573757	20555744	A	38.61	1.21E-14	chr:14:20552746
14	rs6573759	20555817	C	38.61	1.21E-14	chr:14:20552746
14	rs6573762	20555920	A	38.61	1.21E-14	chr:14:20552746
14	rs7151579	20556265	G	38.61	1.21E-14	chr:14:20552746
14	rs7401094	20555950	C	38.61	1.21E-14	chr:14:20552746
14	rs12897224	20560235	C	5.903	1.28E-10	chr:14:20552746
14	rs72669071	20568482	C	12.19	2.56E-09	chr:14:20552746
14	rs369097102	20621303	G	10.66	6.15E-09	chr:14:20552746
14	rs1959651	20548729	C	4.432	1.91E-08	chr:14:20552746
14	rs61993646	20574305	G	4.726	3.88E-08	chr:14:20552746
14	rs7144896	20563540	A	3.521	6.34E-08	chr:14:20552746
14	rs7158044	20563508	C	3.439	8.74E-08	chr:14:20552746
14	rs113297121	20546983	T	4.602	8.99E-08	chr:14:20552746
14	rs2225388	20563664	C	3.403	1.18E-07	chr:14:20552746
14	rs7156270	20564435	T	3.403	1.18E-07	chr:14:20552746
14	rs7156816	20564737	T	3.403	1.18E-07	chr:14:20552746
14	rs7150862	20564461	A	3.417	1.19E-07	chr:14:20552746
14	rs58438833	20552415	C	2.828	1.23E-07	chr:14:20552746
14	rs7140825	20563466	C	0.2914	1.50E-07	chr:14:20552746
14	rs7150250	20563495	C	0.2914	1.50E-07	chr:14:20552746
14	rs12890060	20565461	G	3.346	1.78E-07	chr:14:20552746
14	rs1958724	20565487	T	3.346	1.78E-07	chr:14:20552746

14	rs1958725	20565515	G	3.346	1.78E-07	chr:14:20552746
14	rs4303403	20565381	C	3.346	1.78E-07	chr:14:20552746
14	rs12892241	20563896	C	0.2966	2.09E-07	chr:14:20552746
14	rs10150358	20552946	G	2.735	2.12E-07	chr:14:20552746
14	rs12895600	20566286	A	0.2974	2.43E-07	chr:14:20552746
14	rs1958726	20565683	C	0.3031	3.24E-07	chr:14:20552746
14	rs1958727	20565936	C	0.3031	3.24E-07	chr:14:20552746
14	rs7152008	20566893	A	0.3027	3.70E-07	chr:14:20552746
14	rs4981088	20665840	A	0.3047	3.98E-07	chr:14:20552746
14	rs2318480	20591214	A	3.573	4.42E-07	chr:14:20552746
14	rs4981822	20665790	A	0.3093	4.88E-07	chr:14:20552746
14	rs35344717	20568046	T	0.3085	4.93E-07	chr:14:20552746
14	rs4981066	20570809	A	0.3085	4.93E-07	chr:14:20552746
14	rs60649626	20568452	T	0.3085	4.93E-07	chr:14:20552746
14	rs67680230	20569553	A	0.3085	4.93E-07	chr:14:20552746
14	rs9707258	20573142	T	0.3085	4.93E-07	chr:14:20552746
14	rs17242467	20608329	A	3.411	1.08E-06	chr:14:20552746
14	rs61993653	20633961	A	3.411	1.08E-06	chr:14:20552746
14	rs72638470	20636475	T	3.411	1.08E-06	chr:14:20552746
14	rs36126932	20576705	T	0.329	1.31E-06	chr:14:20552746
14	rs36142459	20576716	T	0.329	1.31E-06	chr:14:20552746
14	rs10149475	20565183	T	0.3311	1.33E-06	chr:14:20552746
14	rs11844863	20568135	C	2.943	1.81E-06	chr:14:20552746
14	rs149352702	20634977	C	9.273	1.88E-06	chr:14:20552746
14	rs202152920	20575655	G	2.857	2.07E-06	chr:14:20552746
14	rs12887942	20577454	T	0.3378	2.19E-06	chr:14:20552746
14	rs7159862	20569410	G	0.3378	2.19E-06	chr:14:20552746
14	rs374481570	20550995	A	0.05707	3.12E-06	chr:14:20552746
14	rs2210191	20563840	G	2.812	3.47E-06	chr:14:20552746
14	rs111429016	20577519	A	0.3461	3.48E-06	chr:14:20552746
14	rs145789052	20577567	G	0.3461	3.48E-06	chr:14:20552746
14	rs7143710	20577918	T	2.803	3.52E-06	chr:14:20552746
14	rs7159989	20577910	A	2.803	3.52E-06	chr:14:20552746
14	rs113231632	20551103	A	3.11	3.57E-06	chr:14:20552746
14	rs11850994	20577628	A	2.741	5.01E-06	chr:14:20552746
14	rs7151455	20577818	G	2.741	5.01E-06	chr:14:20552746
14	rs201183120	20575571	G	2.65	5.85E-06	chr:14:20552746
14	rs72669057	20548996	G	3.638	5.99E-06	chr:14:20552746
14	rs72669049	20538585	G	3.22	6.44E-06	chr:14:20552746
14	rs72669051	20540257	T	3.22	6.44E-06	chr:14:20552746
14	rs17122640	20503541	A	3.502	6.46E-06	chr:14:20552746
14	rs9743620	20573576	A	2.66	7.61E-06	chr:14:20552746
14	rs112874053	20572537	A	2.624	9.87E-06	chr:14:20552746

14	rs190993235	20574230	A	2.624	9.87E-06	chr:14:20552746
14	rs9743609	20573071	A	2.624	9.87E-06	chr:14:20552746
14	rs3939209	20578244	G	2.619	1.07E-05	chr:14:20552746
14	rs28887469	20577050	C	0.4027	1.51E-05	chr:14:20552746
14	rs375935149	20576375	T	0.2807	1.51E-05	chr:14:20552746
14	rs573217071	20576361	G	0.2807	1.51E-05	chr:14:20552746
14	rs72669052	20540663	T	2.995	1.59E-05	chr:14:20552746
14	rs72669053	20540701	G	2.995	1.59E-05	chr:14:20552746
14	rs36074339	20572229	T	2.576	1.74E-05	chr:14:20552746
14	rs111268396	20546863	G	3.645	1.79E-05	chr:14:20552746
14	rs7156327	20578170	G	2.542	2.09E-05	chr:14:20552746
14	rs56327453	20529148	C	3.075	2.21E-05	chr:14:20552746
14	rs7152931	20527032	T	3.075	2.21E-05	chr:14:20552746
14	rs7161355	20526287	C	3.075	2.21E-05	chr:14:20552746
14	rs72669025	20526239	C	3.075	2.21E-05	chr:14:20552746
14	rs72669031	20527127	C	3.075	2.21E-05	chr:14:20552746
14	rs72669032	20527202	C	3.075	2.21E-05	chr:14:20552746
14	rs72669033	20527443	C	3.075	2.21E-05	chr:14:20552746
14	rs72669037	20529550	A	3.075	2.21E-05	chr:14:20552746
14	rs78059363	20529431	T	3.075	2.21E-05	chr:14:20552746
14	rs28750505	20577068	G	0.4148	2.65E-05	chr:14:20552746
14	rs45584133	20528250	T	3.06	2.84E-05	chr:14:20552746
14	rs45585336	20528682	C	3.06	2.84E-05	chr:14:20552746
14	rs55893849	20524035	C	3.06	2.84E-05	chr:14:20552746
14	rs72669017	20523568	G	3.06	2.84E-05	chr:14:20552746
14	rs72669018	20523598	T	3.06	2.84E-05	chr:14:20552746
14	rs72669019	20524309	C	3.06	2.84E-05	chr:14:20552746
14	rs72669021	20524956	A	3.06	2.84E-05	chr:14:20552746
14	rs72669022	20525093	G	3.06	2.84E-05	chr:14:20552746
14	rs72638473	20650246	A	2.816	2.96E-05	chr:14:20552746
14	rs72669013	20518709	C	3.266	3.05E-05	chr:14:20552746
14	rs72669014	20518781	C	3.266	3.05E-05	chr:14:20552746
14	rs76503330	20576401	C	0.3263	4.33E-05	chr:14:20552746
14	rs72669042	20533319	G	2.807	4.79E-05	chr:14:20552746
14	rs995249	20534313	A	2.807	4.79E-05	chr:14:20552746
15	rs2937855	63374677	G	0.09013	4.03E-16	chr:15:63374594
15	rs2652805	63374129	A	0.1001	4.16E-16	chr:15:63374594
15	rs2652806	63374127	C	0.1001	4.16E-16	chr:15:63374594
15	rs2652804	63374256	T	0.09427	5.41E-16	chr:15:63374594
15	rs2729805	63374289	A	0.09427	5.41E-16	chr:15:63374594
15	rs2729806	63373329	T	0.1147	1.41E-15	chr:15:63374594
15	rs2937857	63369571	A	0.162	6.27E-14	chr:15:63374594
15	rs1071646	63351840	C	0.1662	1.28E-13	chr:15:63374594

15	rs1966032	63365471	C	0.1662	1.28E-13	chr:15:63374594
15	rs1966033	63365444	T	0.1662	1.28E-13	chr:15:63374594
15	rs11071726	63390622	T	0.1912	1.44E-13	chr:15:63374594
15	rs2729811	63364783	T	0.1723	1.44E-13	chr:15:63374594
15	rs76119457	63376829	A	0.1571	2.01E-13	chr:15:63374594
15	rs6738	63363901	C	0.1749	2.09E-13	chr:15:63374594
15	rs11071722	63350422	A	0.1771	2.30E-13	chr:15:63374594
15	rs1574311	63346320	G	0.1779	2.38E-13	chr:15:63374594
15	rs4238371	63347403	G	0.1779	2.38E-13	chr:15:63374594
15	rs6494387	63345526	A	0.1846	2.52E-13	chr:15:63374594
15	rs1135403	63344958	G	0.185	2.79E-13	chr:15:63374594
15	rs8519	63363654	A	0.179	2.98E-13	chr:15:63374594
15	rs16946372	63347876	G	0.1793	3.04E-13	chr:15:63374594
15	rs16953467	63347997	A	0.1793	3.04E-13	chr:15:63374594
15	rs4450350	63349952	A	0.1793	3.04E-13	chr:15:63374594
15	rs7166724	63348395	G	0.1793	3.04E-13	chr:15:63374594
15	rs7173072	63349564	C	0.1793	3.04E-13	chr:15:63374594
15	rs8037564	63379822	A	0.1656	4.32E-13	chr:15:63374594
15	rs2729807	63370329	A	0.189	1.32E-12	chr:15:63374594
15	rs17826629	63365680	G	0.1885	5.70E-12	chr:15:63374594
15	rs76862276	63380427	A	0.1847	7.30E-12	chr:15:63374594
15	rs17826683	63365911	A	0.1941	1.13E-11	chr:15:63374594
15	rs2652814	63401020	T	0.1959	2.21E-11	chr:15:63374594
15	rs80349772	63350471	A	0.2101	2.86E-11	chr:15:63374594
15	rs9888699	63387709	T	0.2021	3.31E-11	chr:15:63374594
15	rs8042050	63355871	G	0.2223	3.67E-11	chr:15:63374594
15	rs2729803	63376661	T	4.183	3.80E-11	chr:15:63374594
15	rs146600192	63385070	A	0.1989	4.24E-11	chr:15:63374594
15	rs28485524	63349132	A	0.2291	8.09E-11	chr:15:63374594
15	rs62012880	63527162	A	0.2749	8.48E-11	chr:15:63374594
15	rs2652841	63392707	C	0.214	9.18E-11	chr:15:63374594
15	rs28862017	63393846	G	0.214	9.18E-11	chr:15:63374594
15	rs17753220	63349726	A	0.2303	9.25E-11	chr:15:63374594
15	rs28645615	63348601	T	0.2303	9.25E-11	chr:15:63374594
15	rs2729840	63391744	G	0.2155	9.43E-11	chr:15:63374594
15	rs28660606	63356181	A	0.2309	9.79E-11	chr:15:63374594
15	rs75449003	63355797	T	0.2298	1.01E-10	chr:15:63374594
15	rs77372534	63360126	T	0.2298	1.01E-10	chr:15:63374594
15	rs12440281	63382513	G	0.1926	1.07E-10	chr:15:63374594
15	rs117932747	63399767	A	0.2152	1.30E-10	chr:15:63374594
15	rs34607951	63397625	A	0.2152	1.30E-10	chr:15:63374594
15	rs11630639	63399250	T	0.217	1.35E-10	chr:15:63374594
15	rs11630737	63399556	G	0.217	1.35E-10	chr:15:63374594

15	rs77273812	63391248	A	0.2174	1.39E-10	chr:15:63374594
15	rs76960849	63389834	G	0.2189	1.42E-10	chr:15:63374594
15	rs2729804	63376659	T	3.949	1.73E-10	chr:15:63374594
15	rs2937856	63369742	G	0.2423	1.91E-10	chr:15:63374594
15	rs80220017	63366161	T	0.2467	2.16E-10	chr:15:63374594
15	rs2729793	63483977	T	0.2762	2.17E-10	chr:15:63374594
15	rs376848493	63365312	G	0.241	2.24E-10	chr:15:63374594
15	rs28531385	63350604	C	0.2367	2.38E-10	chr:15:63374594
15	rs11636549	63357288	G	0.2475	2.48E-10	chr:15:63374594
15	rs28512289	63359185	A	0.2475	2.48E-10	chr:15:63374594
15	rs17826341	63359302	A	0.2491	2.88E-10	chr:15:63374594
15	rs78729321	63366160	G	0.2491	2.88E-10	chr:15:63374594
15	rs8040219	63360814	G	0.2491	2.88E-10	chr:15:63374594
15	rs1050255	63363631	T	0.2528	3.19E-10	chr:15:63374594
15	rs7178040	63364098	T	0.2528	3.19E-10	chr:15:63374594
15	rs9920576	63363013	C	0.2528	3.19E-10	chr:15:63374594
15	rs3803502	63352140	T	0.249	3.19E-10	chr:15:63374594
15	rs28735896	63359199	A	0.2491	3.32E-10	chr:15:63374594
15	rs1017545	63553994	C	3.616	3.33E-10	chr:15:63374594
15	rs707602	63363737	C	0.2617	9.68E-10	chr:15:63374594
15	rs2729786	63401605	A	0.2577	1.41E-09	chr:15:63374594
15	rs12912328	63494235	A	0.3063	1.47E-09	chr:15:63374594
15	rs7176597	63494482	G	0.3063	1.47E-09	chr:15:63374594
15	rs12595312	63494159	T	0.3143	2.25E-09	chr:15:63374594
15	rs149883367	63372180	C	0.205	3.92E-09	chr:15:63374594
15	rs2729835	63433766	G	0.3324	4.50E-09	chr:15:63374594
15	rs8468	63434110	C	0.327	4.57E-09	chr:15:63374594
15	rs9672258	63532459	C	0.3053	4.68E-09	chr:15:63374594
15	rs8032533	63516033	A	0.3038	5.02E-09	chr:15:63374594
15	rs10152434	63545352	C	0.3137	5.47E-09	chr:15:63374594
15	rs12439988	63547217	A	0.3137	5.47E-09	chr:15:63374594
15	rs2588862	63500195	A	0.3093	5.65E-09	chr:15:63374594
15	rs2652817	63483290	A	0.3016	5.92E-09	chr:15:63374594
15	rs6494396	63441248	C	0.3193	6.87E-09	chr:15:63374594
15	rs13313493	63523855	C	0.3071	6.92E-09	chr:15:63374594
15	rs55976323	63523586	A	0.3071	6.92E-09	chr:15:63374594
15	rs4619348	63359564	C	3.354	8.04E-09	chr:15:63374594
15	rs16946692	63531915	G	0.3143	8.68E-09	chr:15:63374594
15	rs7175974	63510113	G	0.3143	8.68E-09	chr:15:63374594
15	rs112196574	63545156	A	0.3241	9.49E-09	chr:15:63374594
15	rs2134263	63365394	T	3.262	1.05E-08	chr:15:63374594
15	rs78356111	63436955	C	0.3294	1.15E-08	chr:15:63374594
15	rs2729791	63493057	G	0.3162	1.19E-08	chr:15:63374594

15	rs4775613	63349096	A	3.227	1.25E-08	chr:15:63374594
15	rs7163254	63439403	C	0.3286	1.28E-08	chr:15:63374594
15	rs59768255	63436960	A	0.3486	1.42E-08	chr:15:63374594
15	rs61187203	63436958	C	0.3486	1.42E-08	chr:15:63374594
15	rs12591282	63539475	G	0.3139	1.48E-08	chr:15:63374594
15	rs28369547	63528924	T	0.3139	1.48E-08	chr:15:63374594
15	rs4774478	63434763	A	0.3327	1.50E-08	chr:15:63374594
15	rs16953475	63348087	G	3.158	1.89E-08	chr:15:63374594
15	rs6494394	63440938	A	0.3382	2.21E-08	chr:15:63374594
15	rs12148828	63355339	T	3.147	2.62E-08	chr:15:63374594
15	rs60160488	63587233	G	0.3452	2.64E-08	chr:15:63374594
15	rs55729424	63571779	T	0.3491	2.74E-08	chr:15:63374594
15	rs2062727	63472681	A	0.3282	2.75E-08	chr:15:63374594
15	rs8023653	63571209	T	0.337	3.50E-08	chr:15:63374594
15	rs58698989	63582095	T	0.3528	4.09E-08	chr:15:63374594
15	rs11855609	63522691	C	0.347	4.12E-08	chr:15:63374594
15	rs16946690	63530492	A	0.3343	4.56E-08	chr:15:63374594
15	rs7170409	63567238	T	0.3592	6.01E-08	chr:15:63374594
15	rs11858666	63526239	C	2.881	6.10E-08	chr:15:63374594
15	rs2652818	63502378	C	0.3372	6.11E-08	chr:15:63374594
15	rs4984257	63561160	T	2.864	6.90E-08	chr:15:63374594
15	rs72747098	63525249	A	2.845	6.94E-08	chr:15:63374594
15	rs11071739	63585896	T	0.3633	6.95E-08	chr:15:63374594
15	rs12904537	63379726	T	2.835	7.30E-08	chr:15:63374594
15	rs7177692	63379733	T	2.835	7.30E-08	chr:15:63374594
15	rs7177694	63379737	T	2.835	7.30E-08	chr:15:63374594
15	rs3803501	63352092	A	2.899	8.29E-08	chr:15:63374594
15	rs11857703	63379592	A	2.86	8.61E-08	chr:15:63374594
15	rs2068143	63588662	A	0.3756	8.91E-08	chr:15:63374594
15	rs959125	63583159	G	0.3677	9.19E-08	chr:15:63374594
15	rs11071735	63468114	T	0.3496	9.94E-08	chr:15:63374594
15	rs7165016	63439590	T	0.3979	1.11E-07	chr:15:63374594
15	rs28386949	63596440	A	0.3574	1.57E-07	chr:15:63374594
15	rs1027647	63374825	A	3.061	2.25E-07	chr:15:63374594
15	rs146054382	63508433	T	2.669	2.37E-07	chr:15:63374594
15	rs28565028	63397038	T	2.715	2.87E-07	chr:15:63374594
15	rs6494397	63460009	A	0.378	3.59E-07	chr:15:63374594
15	rs3803499	63343318	C	0.3677	3.92E-07	chr:15:63374594
15	rs17762798	63600603	A	0.3652	3.95E-07	chr:15:63374594
15	rs11071732	63407656	A	0.3384	4.00E-07	chr:15:63374594
15	rs12441822	63424532	T	0.3384	4.00E-07	chr:15:63374594
15	rs11853804	63528256	A	2.626	5.40E-07	chr:15:63374594
15	rs55703462	63395428	G	2.609	6.78E-07	chr:15:63374594

15	rs2018400	63626540	T	0.412	6.95E-07 chr:15:63374594
15	rs11071733	63416481	G	2.506	8.80E-07 chr:15:63374594
15	rs12593078	63517347	G	2.545	9.88E-07 chr:15:63374594
15	rs369161040	63573742	C	0.3885	1.13E-06 chr:15:63374594
15	rs10851727	63603486	G	0.4064	1.41E-06 chr:15:63374594
15	rs62011183	63413119	G	0.3659	1.76E-06 chr:15:63374594
15	rs4774476	63407390	T	2.433	1.81E-06 chr:15:63374594
15	rs7162890	63492027	A	2.472	2.22E-06 chr:15:63374594
15	rs12708477	63605954	A	0.4206	2.91E-06 chr:15:63374594
15	rs8041454	63485976	C	2.438	3.08E-06 chr:15:63374594
15	rs16946801	63605080	T	2.171	5.36E-06 chr:15:63374594
15	rs117973732	63530247	G	0.3363	7.18E-06 chr:15:63374594
15	rs34367270	63410912	A	2.256	9.03E-06 chr:15:63374594
15	rs1039289	63636121	A	0.443	1.06E-05 chr:15:63374594
15	rs35763222	63641361	G	0.4385	1.16E-05 chr:15:63374594
15	rs2729802	63377304	T	0.451	1.67E-05 chr:15:63374594
15	rs200962701	63417378	T	2.171	2.21E-05 chr:15:63374594
15	rs920864	63474391	T	2.051	2.86E-05 chr:15:63374594
15	rs4775635	63456307	C	2.45	3.24E-05 chr:15:63374594
19	rs12461975	21862108	A	102.6	1.61E-13 chr:19:21841536
19	rs117865432	21866980	G	108.1	9.78E-13 chr:19:21841536
19	rs12461755	21874224	G	108.1	9.78E-13 chr:19:21841536
19	rs138289834	21852693	C	108.1	9.78E-13 chr:19:21841536
19	rs75639671	21875393	C	108.1	9.78E-13 chr:19:21841536
19	rs1589279	21847351	C	178	1.13E-12 chr:19:21841536
19	rs58621185	21858531	A	178	1.13E-12 chr:19:21841536
19	rs12052180	21850463	C	266.7	1.86E-12 chr:19:21841536
19	rs12460252	21846543	A	266.7	1.86E-12 chr:19:21841536
19	rs12461654	21849302	A	266.7	1.86E-12 chr:19:21841536
19	rs12462750	21850703	G	266.7	1.86E-12 chr:19:21841536
19	rs146500963	21849138	G	266.7	1.86E-12 chr:19:21841536
19	rs1829501	21847506	C	266.7	1.86E-12 chr:19:21841536
19	rs58339212	21861075	T	266.7	1.86E-12 chr:19:21841536
19	rs59984853	21848526	A	266.7	1.86E-12 chr:19:21841536
19	rs73546506	21860575	A	266.7	1.86E-12 chr:19:21841536
19	rs79961580	21860730	A	266.7	1.86E-12 chr:19:21841536
19	rs114434735	21866989	T	235.6	1.97E-12 chr:19:21841536
19	rs115046417	21854616	G	235.6	1.97E-12 chr:19:21841536
19	rs116818044	21857932	A	235.6	1.97E-12 chr:19:21841536
19	rs144768538	21848501	G	235.6	1.97E-12 chr:19:21841536
19	rs145365305	21843419	A	235.6	1.97E-12 chr:19:21841536
19	rs59896423	21848375	T	130	2.14E-12 chr:19:21841536
19	rs12232863	21832380	G	16.34	2.27E-12 chr:19:21841536

19	rs59724594	21831057	C	16.34	2.27E-12	chr:19:21841536
19	rs113367038	21827121	G	19.45	2.29E-12	chr:19:21841536
19	rs116521177	21831596	T	19.49	2.56E-12	chr:19:21841536
19	rs186336934	21890720	G	109.1	3.41E-12	chr:19:21841536
19	rs8105433	21840606	A	243.3	3.48E-12	chr:19:21841536
19	rs12459784	21941306	C	44.4	5.57E-12	chr:19:21841536
19	rs12459852	21935851	A	44.4	5.57E-12	chr:19:21841536
19	rs12461775	21932062	C	44.4	5.57E-12	chr:19:21841536
19	rs12463030	21947607	C	44.4	5.57E-12	chr:19:21841536
19	rs17701305	21948020	G	44.4	5.57E-12	chr:19:21841536
19	rs75204354	21913614	A	44.4	5.57E-12	chr:19:21841536
19	rs2139533	21832590	T	21.21	9.20E-12	chr:19:21841536
19	rs10413152	21937846	A	37.37	1.00E-11	chr:19:21841536
19	rs11671415	21944782	A	37.37	1.00E-11	chr:19:21841536
19	rs146261958	21911514	A	37.37	1.00E-11	chr:19:21841536
19	rs7258769	21942872	A	37.37	1.00E-11	chr:19:21841536
19	rs74814265	21912203	G	37.37	1.00E-11	chr:19:21841536
19	rs115824809	21831481	T	21.59	1.29E-11	chr:19:21841536
19	rs12232815	21832363	C	21.59	1.29E-11	chr:19:21841536
19	rs2139531	21832736	A	21.59	1.29E-11	chr:19:21841536
19	rs2139532	21832597	C	21.59	1.29E-11	chr:19:21841536
19	rs7253108	21828955	G	21.59	1.29E-11	chr:19:21841536
19	rs111405107	21824157	A	146.9	1.43E-11	chr:19:21841536
19	rs111977553	21824165	T	146.9	1.43E-11	chr:19:21841536
19	rs1520075	21833741	C	21.16	1.48E-11	chr:19:21841536
19	rs117740054	21825672	T	21.13	1.50E-11	chr:19:21841536
19	rs111517079	21838995	T	381.8	1.86E-11	chr:19:21841536
19	rs138320974	21834230	C	381.8	1.86E-11	chr:19:21841536
19	rs28669973	21842253	A	381.8	1.86E-11	chr:19:21841536
19	rs34069114	21838272	C	381.8	1.86E-11	chr:19:21841536
19	rs60312928	21838295	G	381.8	1.86E-11	chr:19:21841536
19	rs78554651	21838518	G	381.8	1.86E-11	chr:19:21841536
19	rs80147175	21839022	G	381.8	1.86E-11	chr:19:21841536
19	rs113277169	21843728	T	356.6	2.58E-11	chr:19:21841536
19	rs186258664	21844201	T	362.6	2.83E-11	chr:19:21841536
19	rs112076231	21834099	G	360.8	3.98E-11	chr:19:21841536
19	rs75322575	21833798	A	28.77	4.06E-11	chr:19:21841536
19	rs111714111	21827774	A	26.19	4.37E-11	chr:19:21841536
19	rs112867628	21828269	A	26.19	4.37E-11	chr:19:21841536
19	rs113033092	21827530	C	26.19	4.37E-11	chr:19:21841536
19	rs35933524	21828299	G	26.19	4.37E-11	chr:19:21841536
19	rs58039129	21827133	A	26.19	4.37E-11	chr:19:21841536
19	rs77728877	21827833	G	26.19	4.37E-11	chr:19:21841536

19	rs111811317	21831260	A	26.36	4.69E-11	chr:19:21841536
19	rs12232857	21832320	A	26.36	4.69E-11	chr:19:21841536
19	rs35188146	21831014	G	26.36	4.69E-11	chr:19:21841536
19	rs35837179	21831140	A	26.36	4.69E-11	chr:19:21841536
19	rs57155685	21831116	T	26.36	4.69E-11	chr:19:21841536
19	rs57973828	21831115	C	26.36	4.69E-11	chr:19:21841536
19	rs59571905	21830875	T	26.36	4.69E-11	chr:19:21841536
19	rs61517956	21833407	A	26.36	4.69E-11	chr:19:21841536
19	rs7250456	21831585	T	26.36	4.69E-11	chr:19:21841536
19	rs7253097	21828936	C	26.36	4.69E-11	chr:19:21841536
19	rs7253361	21829123	G	26.36	4.69E-11	chr:19:21841536
19	rs7254224	21828967	C	26.36	4.69E-11	chr:19:21841536
19	rs7257314	21829542	A	26.36	4.69E-11	chr:19:21841536
19	rs7257929	21829488	C	26.36	4.69E-11	chr:19:21841536
19	rs7258332	21829769	G	26.36	4.69E-11	chr:19:21841536
19	rs117182815	21825628	A	25.9	4.95E-11	chr:19:21841536
19	rs12460950	21824781	C	25.9	4.95E-11	chr:19:21841536
19	rs34891363	21825550	C	25.9	4.95E-11	chr:19:21841536
19	rs7250875	21831905	T	26.45	6.19E-11	chr:19:21841536
19	rs4809168	21828794	A	26.47	6.40E-11	chr:19:21841536
19	rs67673622	21825103	C	26.02	6.41E-11	chr:19:21841536
19	rs11085493	21948748	T	19.29	1.92E-10	chr:19:21841536
19	rs7250867	21831884	T	31.5	2.46E-10	chr:19:21841536
19	rs78823434	21982409	G	25.76	2.53E-10	chr:19:21841536
19	rs10414424	21858966	C	100.8	4.52E-10	chr:19:21841536
19	rs12462082	21974486	T	19.18	1.47E-09	chr:19:21841536
19	rs9917005	21853097	G	32.43	1.81E-09	chr:19:21841536
19	rs75823388	21385874	A	24.7	2.86E-09	chr:19:21841536
19	rs2139534	21832567	G	57.33	4.80E-09	chr:19:21841536
19	rs112439638	21962740	A	20.06	6.08E-09	chr:19:21841536
19	rs111912937	21844349	C	1074	8.62E-09	chr:19:21841536
19	rs113005217	21845172	T	1074	8.62E-09	chr:19:21841536
19	rs113310470	21844534	A	1074	8.62E-09	chr:19:21841536
19	rs116001081	21842717	T	1074	8.62E-09	chr:19:21841536
19	rs12232782	21835070	C	1074	8.62E-09	chr:19:21841536
19	rs12461057	21839205	G	1074	8.62E-09	chr:19:21841536
19	rs12463094	21839243	G	1074	8.62E-09	chr:19:21841536
19	rs144847660	21823693	T	1074	8.62E-09	chr:19:21841536
19	rs147688845	21842839	A	1074	8.62E-09	chr:19:21841536
19	rs149505407	21839099	A	1074	8.62E-09	chr:19:21841536
19	rs28537769	21842349	A	1074	8.62E-09	chr:19:21841536
19	rs28640930	21842166	T	1074	8.62E-09	chr:19:21841536
19	rs57660329	21841175	C	1074	8.62E-09	chr:19:21841536

19	rs58919976	21836187	A	1074	8.62E-09	chr:19:21841536
19	rs61023946	21843077	T	1074	8.62E-09	chr:19:21841536
19	rs7256336	21842016	A	1074	8.62E-09	chr:19:21841536
19	rs7260114	21842561	A	1074	8.62E-09	chr:19:21841536
19	rs78207079	21838528	T	1074	8.62E-09	chr:19:21841536
19	rs79867595	21837552	C	1074	8.62E-09	chr:19:21841536
19	rs8101178	21839402	A	1074	8.62E-09	chr:19:21841536
19	rs8101397	21839802	G	1074	8.62E-09	chr:19:21841536
19	rs8105107	21840531	G	1074	8.62E-09	chr:19:21841536
19	rs8105700	21840972	T	1074	8.62E-09	chr:19:21841536
19	rs8105732	21841123	G	1074	8.62E-09	chr:19:21841536
19	rs8105957	21841052	A	1074	8.62E-09	chr:19:21841536
19	rs141892265	21965652	C	17.34	1.24E-08	chr:19:21841536
19	rs145782450	22007917	A	50.39	1.25E-08	chr:19:21841536
19	rs17769057	22026170	C	50.39	1.25E-08	chr:19:21841536
19	rs12979778	21843988	G	9.366	1.32E-08	chr:19:21841536
19	rs57699265	21844083	C	9.366	1.32E-08	chr:19:21841536
19	rs115635903	21844038	T	935	1.33E-08	chr:19:21841536
19	rs4569404	21924445	A	8.934	2.31E-08	chr:19:21841536
19	rs117229321	21875814	T	48.2	2.35E-08	chr:19:21841536
19	rs10403795	21919081	A	8.785	2.90E-08	chr:19:21841536
19	rs10405142	21918928	T	8.785	2.90E-08	chr:19:21841536
19	rs10422982	21918221	C	8.785	2.90E-08	chr:19:21841536
19	rs113293163	21914189	A	8.785	2.90E-08	chr:19:21841536
19	rs4352176	21924392	C	8.785	2.90E-08	chr:19:21841536
19	rs4372817	21922642	A	8.785	2.90E-08	chr:19:21841536
19	rs4808297	21935700	A	8.785	2.90E-08	chr:19:21841536
19	rs77464908	21898805	A	8.785	2.90E-08	chr:19:21841536
19	rs8101035	21934556	C	8.785	2.90E-08	chr:19:21841536
19	rs12979139	21843901	C	8.675	3.03E-08	chr:19:21841536
19	rs3795247	21906428	C	8.556	4.08E-08	chr:19:21841536
19	rs4330044	21905838	T	8.556	4.08E-08	chr:19:21841536
19	rs7252777	21907988	T	8.556	4.08E-08	chr:19:21841536
19	rs117301126	21965998	A	19.87	4.42E-08	chr:19:21841536
19	rs144038920	21965965	A	19.87	4.42E-08	chr:19:21841536
19	rs145830617	21965007	A	19.87	4.42E-08	chr:19:21841536
19	rs56904798	21962115	A	19.87	4.42E-08	chr:19:21841536
19	rs75477359	21966566	A	19.87	4.42E-08	chr:19:21841536
19	rs6511279	21838240	C	8.184	4.49E-08	chr:19:21841536
19	rs10418334	21823931	T	8.246	4.83E-08	chr:19:21841536
19	rs10401456	21834185	T	8.252	5.03E-08	chr:19:21841536
19	rs10404030	21839783	A	8.252	5.03E-08	chr:19:21841536
19	rs10405733	21834482	C	8.252	5.03E-08	chr:19:21841536

19	rs10422946	21838785	C	8.252	5.03E-08	chr:19:21841536
19	rs4808287	21835411	T	8.252	5.03E-08	chr:19:21841536
19	rs4808289	21836844	T	8.252	5.03E-08	chr:19:21841536
19	rs4808290	21837075	C	8.252	5.03E-08	chr:19:21841536
19	rs4809170	21836678	T	8.252	5.03E-08	chr:19:21841536
19	rs6511280	21838492	C	8.252	5.03E-08	chr:19:21841536
19	rs6511281	21838565	T	8.252	5.03E-08	chr:19:21841536
19	rs7351087	21838147	A	8.252	5.03E-08	chr:19:21841536
19	rs7351088	21838146	C	8.252	5.03E-08	chr:19:21841536
19	rs8105369	21840734	C	8.252	5.03E-08	chr:19:21841536
19	rs8105649	21840765	G	8.252	5.03E-08	chr:19:21841536
19	rs8106449	21835586	C	8.252	5.03E-08	chr:19:21841536
19	rs9807876	21837709	G	8.252	5.03E-08	chr:19:21841536
19	rs12978741	21843568	G	7.93	6.97E-08	chr:19:21841536
19	rs12978776	21843616	G	7.93	6.97E-08	chr:19:21841536
19	rs12980130	21843569	T	7.93	6.97E-08	chr:19:21841536
19	rs28432246	21842163	T	7.93	6.97E-08	chr:19:21841536
19	rs28520521	21842340	T	7.93	6.97E-08	chr:19:21841536
19	rs4808292	21841626	C	7.93	6.97E-08	chr:19:21841536
19	rs4808293	21841796	G	7.93	6.97E-08	chr:19:21841536
19	rs4809171	21841170	A	7.93	6.97E-08	chr:19:21841536
19	rs12460998	21973146	C	16.39	8.83E-08	chr:19:21841536
19	rs12462120	21974940	T	16.39	8.83E-08	chr:19:21841536
19	rs17701347	21980434	T	16.39	8.83E-08	chr:19:21841536
19	rs59958574	21971589	T	16.39	8.83E-08	chr:19:21841536
19	rs75614465	21980134	A	16.39	8.83E-08	chr:19:21841536
19	rs77340617	21982326	T	16.39	8.83E-08	chr:19:21841536
19	rs78077628	21971443	C	16.39	8.83E-08	chr:19:21841536
19	rs12984244	21833966	A	7.694	8.87E-08	chr:19:21841536
19	rs12984552	21834092	A	7.117	1.06E-07	chr:19:21841536
19	rs183320661	21641850	G	20.09	1.11E-07	chr:19:21841536
19	rs12979269	21843781	G	7.978	1.21E-07	chr:19:21841536
19	rs192495107	21383454	T	17.56	1.73E-07	chr:19:21841536
19	rs117010520	22016401	A	14.83	1.82E-07	chr:19:21841536
19	rs117329296	21988033	T	14.83	1.82E-07	chr:19:21841536
19	rs117733188	22016400	A	14.83	1.82E-07	chr:19:21841536
19	rs117783733	22006570	T	14.83	1.82E-07	chr:19:21841536
19	rs118189822	21984558	C	14.83	1.82E-07	chr:19:21841536
19	rs12460775	21985091	C	14.83	1.82E-07	chr:19:21841536
19	rs12463064	21985132	G	14.83	1.82E-07	chr:19:21841536
19	rs141171541	22011873	T	14.83	1.82E-07	chr:19:21841536
19	rs142185394	21984590	A	14.83	1.82E-07	chr:19:21841536
19	rs147875950	21988064	A	14.83	1.82E-07	chr:19:21841536

19	rs17701366	21985740	G	14.83	1.82E-07	chr:19:21841536
19	rs3764576	22005000	G	14.83	1.82E-07	chr:19:21841536
19	rs60272092	22006953	A	14.83	1.82E-07	chr:19:21841536
19	rs74606132	22019517	T	14.83	1.82E-07	chr:19:21841536
19	rs75290521	22000435	G	14.83	1.82E-07	chr:19:21841536
19	rs77086661	22032924	G	14.83	1.82E-07	chr:19:21841536
19	rs77310041	21986116	G	14.83	1.82E-07	chr:19:21841536
19	rs77751145	22006138	G	14.83	1.82E-07	chr:19:21841536
19	rs78033062	21986636	A	14.83	1.82E-07	chr:19:21841536
19	rs78773989	22009807	G	14.83	1.82E-07	chr:19:21841536
19	rs1520074	21833928	G	6.188	1.87E-07	chr:19:21841536
19	rs12973147	21860793	C	6.727	1.95E-07	chr:19:21841536
19	rs71333857	21844171	G	7.125	2.32E-07	chr:19:21841536
19	rs11085492	21945186	G	8.21	2.59E-07	chr:19:21841536
19	rs10405075	21943707	C	7.542	2.84E-07	chr:19:21841536
19	rs10412225	21944450	G	7.542	2.84E-07	chr:19:21841536
19	rs12979060	21843727	G	6.961	3.06E-07	chr:19:21841536
19	rs17768510	21349536	C	9.385	3.09E-07	chr:19:21841536
19	rs9304989	21824313	A	16.95	3.31E-07	chr:19:21841536
19	rs10423041	21933943	C	7.368	3.55E-07	chr:19:21841536
19	rs8105002	21919934	A	7.368	3.55E-07	chr:19:21841536
19	rs10854059	21859805	G	5.889	3.96E-07	chr:19:21841536
19	rs11668774	21859863	A	5.889	3.96E-07	chr:19:21841536
19	rs12461329	21860597	G	5.889	3.96E-07	chr:19:21841536
19	rs12973337	21860723	G	5.889	3.96E-07	chr:19:21841536
19	rs1818693	21858687	A	5.889	3.96E-07	chr:19:21841536
19	rs2015476	21859110	A	5.889	3.96E-07	chr:19:21841536
19	rs2015481	21858994	A	5.889	3.96E-07	chr:19:21841536
19	rs2015715	21858115	C	5.889	3.96E-07	chr:19:21841536
19	rs2015736	21857927	C	5.889	3.96E-07	chr:19:21841536
19	rs118006675	22030918	T	12.66	4.38E-07	chr:19:21841536
19	rs12462709	21989391	C	12.66	4.38E-07	chr:19:21841536
19	rs58664716	21993467	G	12.66	4.38E-07	chr:19:21841536
19	rs74762678	22019157	C	12.66	4.38E-07	chr:19:21841536
19	rs75937847	22031912	A	12.66	4.38E-07	chr:19:21841536
19	rs76621431	22014914	C	12.66	4.38E-07	chr:19:21841536
19	rs77115676	21985611	A	12.66	4.38E-07	chr:19:21841536
19	rs77216709	21992940	G	12.66	4.38E-07	chr:19:21841536
19	rs79327994	21993765	T	12.66	4.38E-07	chr:19:21841536
19	rs8111923	22021828	A	12.66	4.38E-07	chr:19:21841536
19	rs2359392	21854136	A	5.716	4.39E-07	chr:19:21841536
19	rs12459622	21852799	A	5.705	4.47E-07	chr:19:21841536
19	rs12459640	21852807	C	5.705	4.47E-07	chr:19:21841536

19	rs11879157	21900898	T	6.141	4.48E-07	chr:19:21841536
19	rs4808296	21931214	A	6.141	4.48E-07	chr:19:21841536
19	rs147379978	21544008	T	9.856	4.55E-07	chr:19:21841536
19	rs12975447	21860974	T	5.671	4.59E-07	chr:19:21841536
19	rs10164296	21856328	A	5.699	4.87E-07	chr:19:21841536
19	rs10164399	21856722	C	5.699	4.87E-07	chr:19:21841536
19	rs10164402	21856407	T	5.699	4.87E-07	chr:19:21841536
19	rs10164404	21856699	T	5.699	4.87E-07	chr:19:21841536
19	rs12459069	21852101	A	5.699	4.87E-07	chr:19:21841536
19	rs12459105	21852322	A	5.699	4.87E-07	chr:19:21841536
19	rs12459145	21852274	G	5.699	4.87E-07	chr:19:21841536
19	rs12459146	21852286	G	5.699	4.87E-07	chr:19:21841536
19	rs12459607	21852601	C	5.699	4.87E-07	chr:19:21841536
19	rs12459653	21852679	G	5.699	4.87E-07	chr:19:21841536
19	rs12459663	21852921	C	5.699	4.87E-07	chr:19:21841536
19	rs12460255	21853648	G	5.699	4.87E-07	chr:19:21841536
19	rs12460259	21853849	C	5.699	4.87E-07	chr:19:21841536
19	rs12460275	21853896	C	5.699	4.87E-07	chr:19:21841536
19	rs12461611	21856280	G	5.699	4.87E-07	chr:19:21841536
19	rs12463364	21851610	A	5.699	4.87E-07	chr:19:21841536
19	rs12972399	21850149	T	5.699	4.87E-07	chr:19:21841536
19	rs12983456	21855124	G	5.699	4.87E-07	chr:19:21841536
19	rs1818696	21850359	C	5.699	4.87E-07	chr:19:21841536
19	rs1850931	21855226	G	5.699	4.87E-07	chr:19:21841536
19	rs1850932	21855223	C	5.699	4.87E-07	chr:19:21841536
19	rs1850934	21854928	T	5.699	4.87E-07	chr:19:21841536
19	rs1850935	21854623	T	5.699	4.87E-07	chr:19:21841536
19	rs2037356	21849559	C	5.699	4.87E-07	chr:19:21841536
19	rs2359388	21851081	T	5.699	4.87E-07	chr:19:21841536
19	rs2359389	21851157	A	5.699	4.87E-07	chr:19:21841536
19	rs2359398	21854389	C	5.699	4.87E-07	chr:19:21841536
19	rs2359399	21854394	C	5.699	4.87E-07	chr:19:21841536
19	rs2359400	21855574	C	5.699	4.87E-07	chr:19:21841536
19	rs2359401	21855732	G	5.699	4.87E-07	chr:19:21841536
19	rs2359403	21855836	C	5.699	4.87E-07	chr:19:21841536
19	rs2359404	21855839	G	5.699	4.87E-07	chr:19:21841536
19	rs2359405	21856187	G	5.699	4.87E-07	chr:19:21841536
19	rs34449776	21856309	A	5.699	4.87E-07	chr:19:21841536
19	rs35091166	21856300	G	5.699	4.87E-07	chr:19:21841536
19	rs3946575	21848698	C	5.699	4.87E-07	chr:19:21841536
19	rs4026206	21855856	G	5.699	4.87E-07	chr:19:21841536
19	rs4302166	21850861	C	5.699	4.87E-07	chr:19:21841536
19	rs4310994	21850923	A	5.699	4.87E-07	chr:19:21841536

19	rs4321314	21849668	C	5.699	4.87E-07	chr:19:21841536
19	rs4490112	21850753	T	5.699	4.87E-07	chr:19:21841536
19	rs4500887	21850985	G	5.699	4.87E-07	chr:19:21841536
19	rs7259640	21848238	G	5.699	4.87E-07	chr:19:21841536
19	rs8108212	21845320	G	6.128	4.95E-07	chr:19:21841536
19	rs2359394	21854169	A	5.537	5.58E-07	chr:19:21841536
19	rs2359395	21854176	G	5.537	5.58E-07	chr:19:21841536
19	rs2359396	21854179	G	5.537	5.58E-07	chr:19:21841536
19	rs12982600	21847694	C	5.58	6.41E-07	chr:19:21841536
19	rs12984472	21847713	T	5.58	6.41E-07	chr:19:21841536
19	rs8108289	21846845	A	5.58	6.41E-07	chr:19:21841536
19	rs12971926	21837430	C	6.365	7.12E-07	chr:19:21841536
19	rs12972184	21835628	T	6.365	7.12E-07	chr:19:21841536
19	rs12975766	21840005	G	6.365	7.12E-07	chr:19:21841536
19	rs1850930	21839462	A	6.365	7.12E-07	chr:19:21841536
19	rs2139527	21839211	C	6.365	7.12E-07	chr:19:21841536
19	rs28711730	21842387	G	6.365	7.12E-07	chr:19:21841536
19	rs7258859	21942723	G	6.544	7.33E-07	chr:19:21841536
19	rs10469371	21893614	T	6.482	7.46E-07	chr:19:21841536
19	rs10421113	21929885	T	6.402	9.21E-07	chr:19:21841536
19	rs10421342	21930003	T	6.402	9.21E-07	chr:19:21841536
19	rs4436888	21916489	C	6.402	9.21E-07	chr:19:21841536
19	rs4547465	21901056	A	6.402	9.21E-07	chr:19:21841536
19	rs4613201	21927706	C	6.402	9.21E-07	chr:19:21841536
19	rs4808295	21930317	A	6.402	9.21E-07	chr:19:21841536
19	rs4809175	21938744	G	6.402	9.21E-07	chr:19:21841536
19	rs8111464	21917939	G	6.402	9.21E-07	chr:19:21841536
19	rs2359390	21851459	G	5.254	1.14E-06	chr:19:21841536
19	rs4319879	21916767	G	6.321	1.16E-06	chr:19:21841536
19	rs58748216	21914122	T	6.306	1.17E-06	chr:19:21841536
19	rs1397579	21976964	C	6.847	1.28E-06	chr:19:21841536
19	rs56742849	21914115	T	6.222	1.41E-06	chr:19:21841536
19	rs62109860	21833294	T	7.093	1.68E-06	chr:19:21841536
19	rs11085494	21952631	G	5.244	2.00E-06	chr:19:21841536
19	rs11670736	21952736	G	6.297	2.03E-06	chr:19:21841536
19	rs7248525	21996571	A	10.14	2.05E-06	chr:19:21841536
19	rs149927315	22084628	A	21.3	2.59E-06	chr:19:21841536
19	rs34810593	21825360	A	7.196	3.08E-06	chr:19:21841536
19	rs8113652	21921709	T	4.379	4.36E-06	chr:19:21841536
19	rs76369868	21653264	G	6.651	5.15E-06	chr:19:21841536
19	rs143275412	21494843	C	6.525	6.01E-06	chr:19:21841536
19	rs12232841	21857227	T	4.762	6.70E-06	chr:19:21841536
19	rs12984679	21856993	A	4.762	6.70E-06	chr:19:21841536

19	rs149586901	21901959	A	26.84	7.54E-06	chr:19:21841536
19	rs150839878	22023299	A	26.84	7.54E-06	chr:19:21841536
19	rs181060267	22027447	T	26.84	7.54E-06	chr:19:21841536
19	rs4381721	21951581	C	4.763	8.72E-06	chr:19:21841536
19	rs4522535	21951615	A	4.763	8.72E-06	chr:19:21841536
19	rs184125080	21694128	A	6.542	9.24E-06	chr:19:21841536
19	rs6511294	21978569	G	4.588	1.04E-05	chr:19:21841536
19	rs10412066	21950337	C	4.828	1.50E-05	chr:19:21841536
19	rs66562280	21833014	T	4.863	1.56E-05	chr:19:21841536
19	rs6511291	21950402	T	4.113	1.63E-05	chr:19:21841536
19	rs8102320	21936885	A	4.065	1.94E-05	chr:19:21841536
19	rs7252798	21908053	T	4.061	1.97E-05	chr:19:21841536
19	rs3813591	22321256	C	69.26	1.94E-27	chr:19:22414379
19	rs62119970	22417828	G	152.2	2.20E-26	chr:19:22414379
19	rs1830173	22388037	T	158.2	2.53E-26	chr:19:22414379
19	rs387604	22332265	A	158.2	2.53E-26	chr:19:22414379
19	rs451793	22361286	G	158.2	2.53E-26	chr:19:22414379
19	rs62112446	22368828	A	158.2	2.53E-26	chr:19:22414379
19	rs7253425	22381476	G	158.2	2.53E-26	chr:19:22414379
19	rs7343144	22369824	T	158.2	2.53E-26	chr:19:22414379
19	rs420218	22353589	T	156.8	3.05E-26	chr:19:22414379
19	rs17554725	22317759	G	59.61	3.28E-26	chr:19:22414379
19	rs7253490	22293706	A	53.5	4.76E-26	chr:19:22414379
19	rs281177	22394727	T	149.7	4.82E-26	chr:19:22414379
19	rs281160	22418046	T	182.4	8.33E-26	chr:19:22414379
19	rs281162	22418472	A	182.4	8.33E-26	chr:19:22414379
19	rs281170	22423336	T	182.4	8.33E-26	chr:19:22414379
19	rs12460025	22318230	G	59.27	9.31E-26	chr:19:22414379
19	rs8113700	22319421	A	59.27	9.31E-26	chr:19:22414379
19	rs7257051	22319292	G	58.87	1.07E-25	chr:19:22414379
19	rs7248898	22245354	A	47.41	1.87E-25	chr:19:22414379
19	rs1922685	22287970	C	66.51	2.76E-25	chr:19:22414379
19	rs281163	22419302	G	176.7	3.24E-25	chr:19:22414379
19	rs281158	22416013	T	256.3	4.25E-25	chr:19:22414379
19	rs57346017	22427336	G	149.9	4.64E-25	chr:19:22414379
19	rs2548937	22413188	T	255.5	5.91E-25	chr:19:22414379
19	rs4025837	22413189	A	255.5	5.91E-25	chr:19:22414379
19	rs808246	22430516	A	148.2	8.41E-25	chr:19:22414379
19	rs62120010	22428480	A	147.4	1.00E-24	chr:19:22414379
19	rs2606266	22409041	T	256.2	1.44E-24	chr:19:22414379
19	rs2694445	22408997	C	256.2	1.44E-24	chr:19:22414379
19	rs281156	22414691	T	259.5	1.67E-24	chr:19:22414379
19	rs2606267	22409260	A	290.5	2.11E-24	chr:19:22414379

19	rs1840718	22427586	T	152	2.38E-24	chr:19:22414379
19	rs2606274	22413650	G	288.4	2.40E-24	chr:19:22414379
19	rs2694449	22404047	A	242.7	3.12E-24	chr:19:22414379
19	rs281193	22400609	A	242.7	3.12E-24	chr:19:22414379
19	rs281194	22400802	A	242.7	3.12E-24	chr:19:22414379
19	rs281195	22402480	T	242.7	3.12E-24	chr:19:22414379
19	rs281199	22405501	T	240.4	3.61E-24	chr:19:22414379
19	rs7343077	22367925	T	221.5	5.33E-24	chr:19:22414379
19	rs11671416	22427254	C	179.3	9.82E-24	chr:19:22414379
19	rs1961316	22426154	A	165.2	1.18E-23	chr:19:22414379
19	rs283163	22455077	C	35.96	1.60E-23	chr:19:22414379
19	rs1912576	22121293	C	22.33	2.40E-23	chr:19:22414379
19	rs2021560	22373470	C	162.8	3.40E-23	chr:19:22414379
19	rs8105767	22215441	G	20.57	4.51E-23	chr:19:22414379
19	rs1969193	22120269	C	19.74	7.35E-23	chr:19:22414379
19	rs10419384	22391239	G	305.2	8.43E-23	chr:19:22414379
19	rs113493783	22333975	G	305.2	8.43E-23	chr:19:22414379
19	rs142745046	22334261	C	305.2	8.43E-23	chr:19:22414379
19	rs1918161	22374023	T	305.2	8.43E-23	chr:19:22414379
19	rs2360002	22383362	A	305.2	8.43E-23	chr:19:22414379
19	rs281181	22396809	T	305.2	8.43E-23	chr:19:22414379
19	rs379619	22341905	C	305.2	8.43E-23	chr:19:22414379
19	rs3934143	22392238	G	305.2	8.43E-23	chr:19:22414379
19	rs411557	22333324	C	305.2	8.43E-23	chr:19:22414379
19	rs436431	22360919	T	305.2	8.43E-23	chr:19:22414379
19	rs459017	22330531	C	305.2	8.43E-23	chr:19:22414379
19	rs459539	22330699	T	305.2	8.43E-23	chr:19:22414379
19	rs462577	22335500	A	305.2	8.43E-23	chr:19:22414379
19	rs462671	22328850	T	305.2	8.43E-23	chr:19:22414379
19	rs465978	22342421	A	305.2	8.43E-23	chr:19:22414379
19	rs78408635	22339824	G	305.2	8.43E-23	chr:19:22414379
19	rs1634466	22103726	G	20.15	8.92E-23	chr:19:22414379
19	rs79196382	22339817	A	250	1.49E-22	chr:19:22414379
19	rs1852418	22324418	A	294.3	2.85E-22	chr:19:22414379
19	rs17769372	22114704	C	14.79	8.20E-22	chr:19:22414379
19	rs1848994	22111366	A	14.79	8.20E-22	chr:19:22414379
19	rs385774	22096075	A	14.79	8.20E-22	chr:19:22414379
19	rs10404322	22110669	G	15.02	1.14E-21	chr:19:22414379
19	rs369128	22107866	T	14.53	1.26E-21	chr:19:22414379
19	rs440597	22096519	C	14.78	1.61E-21	chr:19:22414379
19	rs461804	22328560	C	297.5	2.83E-21	chr:19:22414379
19	rs8108027	22115901	C	14.22	3.96E-21	chr:19:22414379
19	rs10416248	22115865	A	13.4	1.47E-20	chr:19:22414379

19	rs283147	22438069	C	20.18	1.68E-19	chr:19:22414379
19	rs283142	22445651	T	15.74	1.94E-19	chr:19:22414379
19	rs283140	22447450	G	14.46	2.73E-19	chr:19:22414379
19	rs283139	22448069	T	14.68	3.15E-19	chr:19:22414379
19	rs10425845	22370672	A	560.8	6.40E-19	chr:19:22414379
19	rs283144	22439446	T	15.87	1.67E-18	chr:19:22414379
19	rs283145	22438782	T	17.03	3.17E-18	chr:19:22414379
19	rs452556	22469057	A	0.1029	3.45E-18	chr:19:22414379
19	rs808373	22469233	G	8.263	1.84E-17	chr:19:22414379
19	rs36123644	22444780	C	0.1465	5.15E-17	chr:19:22414379
19	rs553185985	22444776	T	0.1465	5.15E-17	chr:19:22414379
19	rs570214927	22444775	C	0.1465	5.15E-17	chr:19:22414379
19	rs1987409	22647287	T	8.465	5.41E-17	chr:19:22414379
19	rs8104705	22498943	T	0.1328	5.81E-17	chr:19:22414379
19	rs808369	22474282	T	0.1232	5.92E-17	chr:19:22414379
19	rs539301358	22444826	A	0.1653	7.11E-17	chr:19:22414379
19	rs557677690	22444827	A	0.1653	7.11E-17	chr:19:22414379
19	rs71333870	22444823	G	0.1653	7.11E-17	chr:19:22414379
19	rs1975174	22515251	G	0.1332	7.35E-17	chr:19:22414379
19	rs12459636	22542439	A	0.1312	1.04E-16	chr:19:22414379
19	rs12608536	22542058	A	0.1312	1.04E-16	chr:19:22414379
19	rs7255829	22537311	C	0.1325	1.10E-16	chr:19:22414379
19	rs2163846	22505844	C	0.1399	1.17E-16	chr:19:22414379
19	rs7259376	22507705	A	0.1399	1.17E-16	chr:19:22414379
19	rs11085524	22536422	G	0.1295	1.28E-16	chr:19:22414379
19	rs11673212	22536050	C	0.1295	1.28E-16	chr:19:22414379
19	rs11879791	22536939	A	0.1295	1.28E-16	chr:19:22414379
19	rs59598661	22537029	T	0.1295	1.28E-16	chr:19:22414379
19	rs6511344	22535673	C	0.1295	1.28E-16	chr:19:22414379
19	rs11879966	22443852	G	0.1288	1.30E-16	chr:19:22414379
19	rs7252056	22494052	T	0.1404	1.47E-16	chr:19:22414379
19	rs12972854	22444812	C	0.14	1.56E-16	chr:19:22414379
19	rs12973144	22439646	A	0.1331	1.71E-16	chr:19:22414379
19	rs1975173	22534538	C	0.1359	1.76E-16	chr:19:22414379
19	rs11668269	22513960	G	0.148	1.82E-16	chr:19:22414379
19	rs12985757	22444652	A	0.1398	1.84E-16	chr:19:22414379
19	rs12986108	22444577	T	0.1418	1.91E-16	chr:19:22414379
19	rs10409609	22439127	A	0.138	1.98E-16	chr:19:22414379
19	rs11666460	22442350	C	0.138	1.98E-16	chr:19:22414379
19	rs12608491	22541627	A	0.1383	2.35E-16	chr:19:22414379
19	rs2082479	22539847	T	0.1383	2.35E-16	chr:19:22414379
19	rs2082480	22538650	G	0.1383	2.35E-16	chr:19:22414379
19	rs2099354	22539532	C	0.1383	2.35E-16	chr:19:22414379

19	rs2195964	22538338	C	0.1383	2.35E-16	chr:19:22414379
19	rs2195965	22538111	C	0.1383	2.35E-16	chr:19:22414379
19	rs4932759	22540105	C	0.1383	2.35E-16	chr:19:22414379
19	rs4932760	22540455	T	0.1383	2.35E-16	chr:19:22414379
19	rs4932986	22540437	A	0.1383	2.35E-16	chr:19:22414379
19	rs57501688	22540597	G	0.1383	2.35E-16	chr:19:22414379
19	rs58170323	22539115	T	0.1383	2.35E-16	chr:19:22414379
19	rs6511345	22537436	T	0.1383	2.35E-16	chr:19:22414379
19	rs7259269	22441171	T	0.1388	2.53E-16	chr:19:22414379
19	rs12973509	22439581	A	0.1385	2.66E-16	chr:19:22414379
19	rs12982551	22439042	C	0.1385	2.66E-16	chr:19:22414379
19	rs8108630	22535177	G	0.1382	2.73E-16	chr:19:22414379
19	rs12610469	22454617	A	0.1305	2.87E-16	chr:19:22414379
19	rs7249374	22533465	C	0.1379	3.09E-16	chr:19:22414379
19	rs11667886	22430941	T	0.1368	3.88E-16	chr:19:22414379
19	rs56227660	22533543	A	0.14	4.22E-16	chr:19:22414379
19	rs13382114	22545739	G	0.1477	4.38E-16	chr:19:22414379
19	rs12610462	22551462	C	0.1468	4.52E-16	chr:19:22414379
19	rs16999064	22552602	A	0.1468	4.52E-16	chr:19:22414379
19	rs8111537	22535545	C	0.1472	6.40E-16	chr:19:22414379
19	rs10221483	22455112	G	0.1616	7.49E-16	chr:19:22414379
19	rs12460610	22291277	C	16.59	7.63E-16	chr:19:22414379
19	rs190030543	22291079	T	16.59	7.63E-16	chr:19:22414379
19	rs56320331	22297657	T	16.59	7.63E-16	chr:19:22414379
19	rs12608842	22548013	G	0.154	8.09E-16	chr:19:22414379
19	rs34556219	22382294	C	23.82	8.83E-16	chr:19:22414379
19	rs374309	22332298	G	23.82	8.83E-16	chr:19:22414379
19	rs62118584	22541142	G	0.1536	1.02E-15	chr:19:22414379
19	rs2168491	22425589	G	0.1464	1.04E-15	chr:19:22414379
19	rs2885035	22549124	T	0.1543	1.09E-15	chr:19:22414379
19	rs61657442	22552178	A	0.1543	1.09E-15	chr:19:22414379
19	rs7258446	22550341	A	0.1543	1.09E-15	chr:19:22414379
19	rs11085515	22426520	C	0.1524	1.14E-15	chr:19:22414379
19	rs10221484	22455170	G	0.1694	1.18E-15	chr:19:22414379
19	rs6511324	22283994	T	17.78	1.22E-15	chr:19:22414379
19	rs73019896	22286350	A	17.78	1.22E-15	chr:19:22414379
19	rs73019902	22286707	A	17.78	1.22E-15	chr:19:22414379
19	rs8108310	22287184	T	17.78	1.22E-15	chr:19:22414379
19	rs10409819	22602919	T	0.1599	1.26E-15	chr:19:22414379
19	rs12984257	22590664	A	0.1599	1.26E-15	chr:19:22414379
19	rs7248242	22575994	G	0.1599	1.26E-15	chr:19:22414379
19	rs281172	22424697	T	0.1525	1.35E-15	chr:19:22414379
19	rs436971	22461218	T	0.1757	1.41E-15	chr:19:22414379

19	rs808372	22470368	T	0.1757	1.41E-15	chr:19:22414379
19	rs11085526	22563880	C	0.1604	1.62E-15	chr:19:22414379
19	rs111724992	22282718	C	16.08	1.70E-15	chr:19:22414379
19	rs12459299	22296261	G	16.08	1.70E-15	chr:19:22414379
19	rs12460028	22284598	C	16.08	1.70E-15	chr:19:22414379
19	rs12460058	22284531	A	16.08	1.70E-15	chr:19:22414379
19	rs12461491	22304761	G	16.08	1.70E-15	chr:19:22414379
19	rs12461499	22304581	A	16.08	1.70E-15	chr:19:22414379
19	rs56359043	22295283	G	16.08	1.70E-15	chr:19:22414379
19	rs7260631	22282906	G	16.08	1.70E-15	chr:19:22414379
19	rs73021825	22313577	A	16.08	1.70E-15	chr:19:22414379
19	rs76995772	22311909	A	16.08	1.70E-15	chr:19:22414379
19	rs7256745	22537287	T	0.1721	1.70E-15	chr:19:22414379
19	rs4024623	22568065	A	0.1557	1.71E-15	chr:19:22414379
19	rs4932951	22246352	C	14.36	1.77E-15	chr:19:22414379
19	rs73019874	22267259	G	14.36	1.77E-15	chr:19:22414379
19	rs73021803	22286898	T	14.23	1.79E-15	chr:19:22414379
19	rs458515	22330126	T	0.147	1.85E-15	chr:19:22414379
19	rs10418203	22567144	A	0.1669	1.89E-15	chr:19:22414379
19	rs11671470	22599468	T	0.1669	1.89E-15	chr:19:22414379
19	rs11673228	22596084	A	0.1669	1.89E-15	chr:19:22414379
19	rs11881957	22573251	T	0.1669	1.89E-15	chr:19:22414379
19	rs12978477	22577709	G	0.1669	1.89E-15	chr:19:22414379
19	rs1865073	22554980	T	0.1669	1.89E-15	chr:19:22414379
19	rs1865074	22554949	G	0.1669	1.89E-15	chr:19:22414379
19	rs7256749	22592289	G	0.1669	1.89E-15	chr:19:22414379
19	rs7260210	22504803	A	0.1619	2.07E-15	chr:19:22414379
19	rs281164	22419523	T	0.1457	2.07E-15	chr:19:22414379
19	rs281166	22420798	G	0.1457	2.07E-15	chr:19:22414379
19	rs281168	22422638	A	0.1457	2.07E-15	chr:19:22414379
19	rs10425699	22576384	T	0.1617	2.08E-15	chr:19:22414379
19	rs605085	22355832	T	0.1527	2.12E-15	chr:19:22414379
19	rs172183	22405532	A	0.1514	2.15E-15	chr:19:22414379
19	rs1830175	22382080	A	0.1514	2.15E-15	chr:19:22414379
19	rs281196	22403293	G	0.1514	2.15E-15	chr:19:22414379
19	rs281198	22405169	G	0.1514	2.15E-15	chr:19:22414379
19	rs281203	22406377	C	0.1514	2.15E-15	chr:19:22414379
19	rs431628	22359024	T	0.1514	2.15E-15	chr:19:22414379
19	rs73021823	22310164	A	15.93	2.21E-15	chr:19:22414379
19	rs8103765	22569105	T	0.1611	2.23E-15	chr:19:22414379
19	rs12461719	22292841	T	14.03	2.27E-15	chr:19:22414379
19	rs4436884	22285163	C	14.03	2.27E-15	chr:19:22414379
19	rs55635227	22298116	C	14.03	2.27E-15	chr:19:22414379

19	rs56242139	22295279	A	14.03	2.27E-15	chr:19:22414379
19	rs34364151	22393367	T	0.1485	2.31E-15	chr:19:22414379
19	rs281169	22423276	T	0.1516	2.32E-15	chr:19:22414379
19	rs10419506	22561519	G	0.1682	2.61E-15	chr:19:22414379
19	rs73019829	22239600	A	15.24	2.65E-15	chr:19:22414379
19	rs73019831	22242013	T	15.24	2.65E-15	chr:19:22414379
19	rs1091182	22475622	A	0.163	2.67E-15	chr:19:22414379
19	rs12609473	22378606	G	0.1529	3.00E-15	chr:19:22414379
19	rs2522317	22339626	G	0.1529	3.00E-15	chr:19:22414379
19	rs3951502	22368852	A	0.1529	3.00E-15	chr:19:22414379
19	rs424912	22347371	C	0.1529	3.00E-15	chr:19:22414379
19	rs435638	22333121	A	0.1529	3.00E-15	chr:19:22414379
19	rs448861	22353606	A	0.1529	3.00E-15	chr:19:22414379
19	rs9304995	22366432	A	0.1529	3.00E-15	chr:19:22414379
19	rs2217666	22565048	A	0.1734	3.06E-15	chr:19:22414379
19	rs10426106	22323268	T	0.1474	3.08E-15	chr:19:22414379
19	rs1865067	22323069	T	0.1474	3.08E-15	chr:19:22414379
19	rs1897613	22573575	G	0.1679	3.09E-15	chr:19:22414379
19	rs10402396	22601809	C	0.1679	3.11E-15	chr:19:22414379
19	rs10406199	22584530	G	0.1679	3.11E-15	chr:19:22414379
19	rs10407050	22584914	G	0.1679	3.11E-15	chr:19:22414379
19	rs10407485	22585087	T	0.1679	3.11E-15	chr:19:22414379
19	rs10408428	22578023	T	0.1679	3.11E-15	chr:19:22414379
19	rs10413240	22591317	C	0.1679	3.11E-15	chr:19:22414379
19	rs10414591	22600122	A	0.1679	3.11E-15	chr:19:22414379
19	rs10417380	22596625	T	0.1679	3.11E-15	chr:19:22414379
19	rs10420380	22600624	T	0.1679	3.11E-15	chr:19:22414379
19	rs10423736	22596132	T	0.1679	3.11E-15	chr:19:22414379
19	rs10454118	22559605	G	0.1679	3.11E-15	chr:19:22414379
19	rs12151261	22587929	T	0.1679	3.11E-15	chr:19:22414379
19	rs12461060	22566085	T	0.1679	3.11E-15	chr:19:22414379
19	rs12977605	22566997	T	0.1679	3.11E-15	chr:19:22414379
19	rs12983542	22590585	G	0.1679	3.11E-15	chr:19:22414379
19	rs1368476	22586192	G	0.1679	3.11E-15	chr:19:22414379
19	rs139019650	22566671	T	0.1679	3.11E-15	chr:19:22414379
19	rs16999542	22573092	A	0.1679	3.11E-15	chr:19:22414379
19	rs17391718	22554154	A	0.1679	3.11E-15	chr:19:22414379
19	rs1865075	22554854	T	0.1679	3.11E-15	chr:19:22414379
19	rs2360622	22570622	T	0.1679	3.11E-15	chr:19:22414379
19	rs2360625	22592860	G	0.1679	3.11E-15	chr:19:22414379
19	rs2957848	22583747	G	0.1679	3.11E-15	chr:19:22414379
19	rs4024624	22568264	C	0.1679	3.11E-15	chr:19:22414379
19	rs5014503	22568340	A	0.1679	3.11E-15	chr:19:22414379

19	rs56027264	22554354	T	0.1679	3.11E-15	chr:19:22414379
19	rs56376504	22562520	C	0.1679	3.11E-15	chr:19:22414379
19	rs58374468	22562522	C	0.1679	3.11E-15	chr:19:22414379
19	rs6511350	22594697	G	0.1679	3.11E-15	chr:19:22414379
19	rs7247085	22589367	G	0.1679	3.11E-15	chr:19:22414379
19	rs7247220	22589489	G	0.1679	3.11E-15	chr:19:22414379
19	rs7247735	22575488	T	0.1679	3.11E-15	chr:19:22414379
19	rs7250541	22557093	G	0.1679	3.11E-15	chr:19:22414379
19	rs7250710	22557231	G	0.1679	3.11E-15	chr:19:22414379
19	rs7253315	22594439	T	0.1679	3.11E-15	chr:19:22414379
19	rs7255934	22596865	C	0.1679	3.11E-15	chr:19:22414379
19	rs7508099	22556203	T	0.1679	3.11E-15	chr:19:22414379
19	rs75653200	22579503	T	0.1679	3.11E-15	chr:19:22414379
19	rs8100077	22593112	T	0.1679	3.11E-15	chr:19:22414379
19	rs8106611	22569618	G	0.1679	3.11E-15	chr:19:22414379
19	rs8107513	22564534	C	0.1679	3.11E-15	chr:19:22414379
19	rs8107704	22564531	G	0.1679	3.11E-15	chr:19:22414379
19	rs8111665	22588559	T	0.1679	3.11E-15	chr:19:22414379
19	rs8112246	22567447	C	0.1679	3.11E-15	chr:19:22414379
19	rs368652	22356811	A	0.1514	3.32E-15	chr:19:22414379
19	rs55983151	22552936	T	0.1632	3.33E-15	chr:19:22414379
19	rs8110854	22549453	C	0.1632	3.33E-15	chr:19:22414379
19	rs56064722	22312293	T	17.25	3.67E-15	chr:19:22414379
19	rs437284	22354875	T	0.1512	3.82E-15	chr:19:22414379
19	rs7259586	22537862	C	0.1706	3.83E-15	chr:19:22414379
19	rs201005223	22574818	C	0.1515	3.84E-15	chr:19:22414379
19	rs4024619	22586839	G	0.1687	3.87E-15	chr:19:22414379
19	rs12459277	22235908	T	13.9	4.10E-15	chr:19:22414379
19	rs34878133	22230331	A	13.9	4.10E-15	chr:19:22414379
19	rs3752168	22235478	C	13.9	4.10E-15	chr:19:22414379
19	rs56183023	22269366	C	13.9	4.10E-15	chr:19:22414379
19	rs73019812	22232291	T	13.9	4.10E-15	chr:19:22414379
19	rs8101997	22568432	A	0.1686	4.23E-15	chr:19:22414379
19	rs56036277	22257574	A	15	4.25E-15	chr:19:22414379
19	rs73019871	22265915	T	15	4.25E-15	chr:19:22414379
19	rs201113571	22290840	T	14.79	4.30E-15	chr:19:22414379
19	rs4358065	22521132	A	0.1613	4.36E-15	chr:19:22414379
19	rs7256861	22561484	T	0.1697	4.40E-15	chr:19:22414379
19	rs61237480	22306505	T	13.27	4.59E-15	chr:19:22414379
19	rs73021816	22306321	A	13.27	4.59E-15	chr:19:22414379
19	rs11669885	22397422	A	0.153	4.71E-15	chr:19:22414379
19	rs12980808	22374080	G	0.153	4.71E-15	chr:19:22414379
19	rs397538	22329889	A	0.153	4.71E-15	chr:19:22414379

19	rs617609	22326000	A	0.153	4.71E-15	chr:19:22414379
19	rs464038	22335569	G	0.1597	5.02E-15	chr:19:22414379
19	rs56226428	22229075	C	15.09	5.10E-15	chr:19:22414379
19	rs404793	22329632	A	0.1607	5.21E-15	chr:19:22414379
19	rs4932955	22272845	A	12.3	5.35E-15	chr:19:22414379
19	rs4932956	22273797	G	12.3	5.35E-15	chr:19:22414379
19	rs11085514	22425837	A	0.165	5.38E-15	chr:19:22414379
19	rs4932763	22603922	A	0.1696	5.59E-15	chr:19:22414379
19	rs2195967	22564922	T	0.1727	6.25E-15	chr:19:22414379
19	rs12461745	22262667	T	13.66	6.63E-15	chr:19:22414379
19	rs56138218	22279031	A	13.66	6.63E-15	chr:19:22414379
19	rs73019862	22260969	T	13.66	6.63E-15	chr:19:22414379
19	rs1985135	22523578	A	0.164	7.19E-15	chr:19:22414379
19	rs12971375	22599963	A	0.1795	7.36E-15	chr:19:22414379
19	rs73019807	22231484	G	14.76	8.27E-15	chr:19:22414379
19	rs10420149	22582925	G	0.1704	8.41E-15	chr:19:22414379
19	rs1808668	22518657	A	0.1854	8.52E-15	chr:19:22414379
19	rs3862494	22519522	G	0.1854	8.52E-15	chr:19:22414379
19	rs1975175	22515239	G	0.1905	9.69E-15	chr:19:22414379
19	rs10409520	22512477	C	0.1848	9.69E-15	chr:19:22414379
19	rs2116919	22491706	C	0.1848	9.69E-15	chr:19:22414379
19	rs71333873	22581471	T	0.1767	9.70E-15	chr:19:22414379
19	rs8100087	22601359	C	0.1767	9.70E-15	chr:19:22414379
19	rs8110636	22600827	A	0.1767	9.70E-15	chr:19:22414379
19	rs720893	22426404	G	0.1696	1.03E-14	chr:19:22414379
19	rs11085522	22505167	T	0.1929	1.05E-14	chr:19:22414379
19	rs4932990	22583691	A	0.1659	1.26E-14	chr:19:22414379
19	rs10404225	22493082	G	0.1929	1.33E-14	chr:19:22414379
19	rs10409947	22512518	G	0.1929	1.33E-14	chr:19:22414379
19	rs1991830	22514730	G	0.1929	1.33E-14	chr:19:22414379
19	rs4932755	22494816	A	0.1929	1.33E-14	chr:19:22414379
19	rs7254434	22500033	G	0.1929	1.33E-14	chr:19:22414379
19	rs8106639	22509614	C	0.1929	1.33E-14	chr:19:22414379
19	rs9304996	22486535	A	0.1929	1.33E-14	chr:19:22414379
19	rs12462177	22509486	G	13.79	1.39E-14	chr:19:22414379
19	rs61216675	22561510	C	0.1784	1.41E-14	chr:19:22414379
19	rs7257134	22540857	A	0.1711	1.43E-14	chr:19:22414379
19	rs283158	22458714	A	12.48	1.67E-14	chr:19:22414379
19	rs283159	22457776	T	12.48	1.67E-14	chr:19:22414379
19	rs383451	22463231	T	12.48	1.67E-14	chr:19:22414379
19	rs58164690	22504773	A	11.18	1.80E-14	chr:19:22414379
19	rs4932984	22533255	T	0.1759	1.91E-14	chr:19:22414379
19	rs10404620	22316577	A	0.1765	1.92E-14	chr:19:22414379

19	rs10411951	22309055	C	0.1765	1.92E-14	chr:19:22414379
19	rs4244914	22315389	A	0.1765	1.92E-14	chr:19:22414379
19	rs6511334	22307267	C	0.1765	1.92E-14	chr:19:22414379
19	rs12459631	22477395	G	13.67	1.98E-14	chr:19:22414379
19	rs4932975	22491072	C	13.67	1.98E-14	chr:19:22414379
19	rs4932976	22494878	T	13.67	1.98E-14	chr:19:22414379
19	rs56955437	22504518	T	13.67	1.98E-14	chr:19:22414379
19	rs7253033	22503203	C	13.67	1.98E-14	chr:19:22414379
19	rs12983035	22590392	T	0.1877	2.05E-14	chr:19:22414379
19	rs7255484	22184978	C	12.22	2.10E-14	chr:19:22414379
19	rs10407936	22312059	A	0.1769	2.49E-14	chr:19:22414379
19	rs10415817	22313214	C	0.1769	2.49E-14	chr:19:22414379
19	rs7245790	22320009	C	0.1769	2.49E-14	chr:19:22414379
19	rs8103471	22317314	T	0.1769	2.49E-14	chr:19:22414379
19	rs55701013	22479996	G	11.01	2.81E-14	chr:19:22414379
19	rs8103990	22476027	A	11.01	2.81E-14	chr:19:22414379
19	rs59870410	22483963	G	12.59	2.93E-14	chr:19:22414379
19	rs1975172	22534603	A	0.181	3.52E-14	chr:19:22414379
19	rs12459526	22216669	T	10.73	3.57E-14	chr:19:22414379
19	rs2359827	22220016	C	10.73	3.57E-14	chr:19:22414379
19	rs4932940	22216366	G	10.73	3.57E-14	chr:19:22414379
19	rs7251237	22220858	A	10.73	3.57E-14	chr:19:22414379
19	rs8103503	22216828	A	10.73	3.57E-14	chr:19:22414379
19	rs4932977	22520694	T	11.52	3.68E-14	chr:19:22414379
19	rs80117552	22171367	C	11.37	3.98E-14	chr:19:22414379
19	rs61590737	22094470	G	0.1736	3.98E-14	chr:19:22414379
19	rs12460715	22523304	A	11.94	4.34E-14	chr:19:22414379
19	rs17769191	22094348	C	0.1737	5.03E-14	chr:19:22414379
19	rs2435012	22093800	A	0.1737	5.03E-14	chr:19:22414379
19	rs1229924	22092288	A	0.1709	5.20E-14	chr:19:22414379
19	rs112237385	22162147	C	9.588	5.81E-14	chr:19:22414379
19	rs4456632	22153634	A	9.588	5.81E-14	chr:19:22414379
19	rs55674738	22196576	T	9.588	5.81E-14	chr:19:22414379
19	rs56408994	22162618	C	9.588	5.81E-14	chr:19:22414379
19	rs60871663	22194240	T	9.588	5.81E-14	chr:19:22414379
19	rs6511310	22197285	A	9.588	5.81E-14	chr:19:22414379
19	rs7246239	22183049	A	9.588	5.81E-14	chr:19:22414379
19	rs7246992	22192047	A	9.588	5.81E-14	chr:19:22414379
19	rs7248938	22166196	G	9.588	5.81E-14	chr:19:22414379
19	rs7258270	22155520	G	9.588	5.81E-14	chr:19:22414379
19	rs73017852	22189239	G	9.588	5.81E-14	chr:19:22414379
19	rs12460852	22226289	T	10.23	5.87E-14	chr:19:22414379
19	rs6511346	22537574	T	0.1916	5.91E-14	chr:19:22414379

19	rs12462668	22156943	A	10.45	6.48E-14	chr:19:22414379
19	rs2359829	22222155	G	10.45	6.48E-14	chr:19:22414379
19	rs67441682	22194829	C	10.45	6.48E-14	chr:19:22414379
19	rs67458228	22191841	T	10.45	6.48E-14	chr:19:22414379
19	rs68118096	22200804	T	10.45	6.48E-14	chr:19:22414379
19	rs8104333	22153801	C	10.45	6.48E-14	chr:19:22414379
19	rs73015567	22128077	A	10.81	6.75E-14	chr:19:22414379
19	rs73015578	22132084	A	10.81	6.75E-14	chr:19:22414379
19	rs10411720	22308919	C	0.1886	7.09E-14	chr:19:22414379
19	rs12462173	22120728	T	10.66	7.59E-14	chr:19:22414379
19	rs118032465	22521394	C	11.94	8.29E-14	chr:19:22414379
19	rs200200120	22521387	T	11.94	8.29E-14	chr:19:22414379
19	rs11881638	22107837	A	0.1805	8.46E-14	chr:19:22414379
19	rs1813242	22101473	G	0.1805	8.46E-14	chr:19:22414379
19	rs1848996	22111809	A	0.1805	8.46E-14	chr:19:22414379
19	rs1965611	22113197	A	0.1805	8.46E-14	chr:19:22414379
19	rs2203034	22101710	C	0.1805	8.46E-14	chr:19:22414379
19	rs413873	22100192	A	0.1805	8.46E-14	chr:19:22414379
19	rs438100	22101205	C	0.1805	8.46E-14	chr:19:22414379
19	rs807957	22115990	A	0.1805	8.46E-14	chr:19:22414379
19	rs807959	22115835	G	0.1805	8.46E-14	chr:19:22414379
19	rs807965	22114669	T	0.1805	8.46E-14	chr:19:22414379
19	rs975712	22098471	C	0.1805	8.46E-14	chr:19:22414379
19	rs281173	22424997	G	0.1918	9.38E-14	chr:19:22414379
19	rs34044431	22128382	G	9.962	1.11E-13	chr:19:22414379
19	rs4932854	22143597	G	9.962	1.11E-13	chr:19:22414379
19	rs68053498	22131813	A	9.962	1.11E-13	chr:19:22414379
19	rs12460903	22210184	C	9.598	1.11E-13	chr:19:22414379
19	rs12461536	22207464	G	9.598	1.11E-13	chr:19:22414379
19	rs12462824	22209767	G	9.598	1.11E-13	chr:19:22414379
19	rs1975167	22215066	T	9.598	1.11E-13	chr:19:22414379
19	rs55638635	22210647	A	9.598	1.11E-13	chr:19:22414379
19	rs55772746	22224720	G	9.598	1.11E-13	chr:19:22414379
19	rs58132720	22225014	A	9.598	1.11E-13	chr:19:22414379
19	rs7247687	22214420	T	9.598	1.11E-13	chr:19:22414379
19	rs7255179	22213130	G	9.598	1.11E-13	chr:19:22414379
19	rs7258843	22213701	C	9.598	1.11E-13	chr:19:22414379
19	rs7259460	22213945	A	9.598	1.11E-13	chr:19:22414379
19	rs7259527	22214257	G	9.598	1.11E-13	chr:19:22414379
19	rs8103879	22211437	G	9.598	1.11E-13	chr:19:22414379
19	rs8106352	22208611	T	9.598	1.11E-13	chr:19:22414379
19	rs807956	22117292	A	0.1822	1.14E-13	chr:19:22414379
19	rs11878178	22577079	A	0.2418	1.18E-13	chr:19:22414379

19	rs11883405	22577080	T	0.2418	1.18E-13	chr:19:22414379
19	rs11669254	22544805	A	0.2045	1.18E-13	chr:19:22414379
19	rs2078136	22113503	A	0.1857	1.19E-13	chr:19:22414379
19	rs7245491	22532793	C	0.1994	1.27E-13	chr:19:22414379
19	rs7245492	22532796	C	0.1994	1.27E-13	chr:19:22414379
19	rs1912575	22100319	A	0.1823	1.34E-13	chr:19:22414379
19	rs1912574	22125733	C	9.183	1.95E-13	chr:19:22414379
19	rs1995934	22128656	T	9.183	1.95E-13	chr:19:22414379
19	rs56061708	22128775	T	9.183	1.95E-13	chr:19:22414379
19	rs1229922	22092875	T	0.1851	1.98E-13	chr:19:22414379
19	rs2360621	22548669	C	0.2054	2.00E-13	chr:19:22414379
19	rs2079063	22160674	T	9.339	2.01E-13	chr:19:22414379
19	rs2359826	22214993	T	9.339	2.01E-13	chr:19:22414379
19	rs57286877	22172618	G	9.339	2.01E-13	chr:19:22414379
19	rs7260454	22166739	T	9.339	2.01E-13	chr:19:22414379
19	rs73538835	22161066	A	9.339	2.01E-13	chr:19:22414379
19	rs28420503	22316713	A	0.2045	2.39E-13	chr:19:22414379
19	rs2359828	22220287	G	8.987	2.48E-13	chr:19:22414379
19	rs57420056	22094309	A	5.52	2.56E-13	chr:19:22414379
19	rs113181884	22586084	C	0.2597	2.87E-13	chr:19:22414379
19	rs56768795	22586073	A	0.2597	2.87E-13	chr:19:22414379
19	rs4932983	22533167	A	0.2098	2.90E-13	chr:19:22414379
19	rs77808832	22120260	T	8.906	3.14E-13	chr:19:22414379
19	rs12461800	22145006	C	8.933	3.55E-13	chr:19:22414379
19	rs56058873	22142542	C	8.933	3.55E-13	chr:19:22414379
19	rs68061456	22142308	A	8.933	3.55E-13	chr:19:22414379
19	rs10417635	22146123	G	8.541	3.98E-13	chr:19:22414379
19	rs66814910	22148202	A	8.541	3.98E-13	chr:19:22414379
19	rs2885036	22560484	T	0.2204	5.51E-13	chr:19:22414379
19	rs2188967	22095513	A	0.2038	5.90E-13	chr:19:22414379
19	rs8111771	22287861	A	0.2129	5.96E-13	chr:19:22414379
19	rs2905572	22651073	T	7.244	6.39E-13	chr:19:22414379
19	rs2905574	22655531	G	7.244	6.39E-13	chr:19:22414379
19	rs2905575	22656127	A	7.244	6.39E-13	chr:19:22414379
19	rs2957811	22658228	T	7.244	6.39E-13	chr:19:22414379
19	rs34312380	22390071	T	0.1937	6.72E-13	chr:19:22414379
19	rs34407605	22390084	T	0.1937	6.72E-13	chr:19:22414379
19	rs4932932	22203214	T	7.297	6.80E-13	chr:19:22414379
19	rs8100117	22195322	C	7.297	6.80E-13	chr:19:22414379
19	rs8109237	22202739	G	7.297	6.80E-13	chr:19:22414379
19	rs4932988	22560235	A	0.2207	7.00E-13	chr:19:22414379
19	rs6511348	22557374	G	0.2207	7.00E-13	chr:19:22414379
19	rs7252785	22561287	G	0.2207	7.00E-13	chr:19:22414379

19	rs8113208	22553888	A	0.2207	7.00E-13	chr:19:22414379
19	rs11671804	22591216	C	0.2251	7.82E-13	chr:19:22414379
19	rs12973769	22593223	C	0.2251	7.82E-13	chr:19:22414379
19	rs3867187	22570220	C	0.2251	7.82E-13	chr:19:22414379
19	rs4932747	22279252	C	0.2078	7.86E-13	chr:19:22414379
19	rs7248507	22214366	C	8.137	7.89E-13	chr:19:22414379
19	rs7248542	22214447	C	8.137	7.89E-13	chr:19:22414379
19	rs7253170	22209228	G	8.137	7.89E-13	chr:19:22414379
19	rs73930941	22123495	C	8.043	9.86E-13	chr:19:22414379
19	rs807966	22113827	C	4.952	1.23E-12	chr:19:22414379
19	rs8100438	22301003	C	0.2229	1.32E-12	chr:19:22414379
19	rs987711	22300007	T	0.2229	1.32E-12	chr:19:22414379
19	rs28456336	22268779	T	0.2267	1.35E-12	chr:19:22414379
19	rs68168074	22120333	T	7.739	1.68E-12	chr:19:22414379
19	rs1019938	22280954	T	0.224	1.77E-12	chr:19:22414379
19	rs1560718	22292212	C	0.224	1.77E-12	chr:19:22414379
19	rs1978718	22293126	T	0.224	1.77E-12	chr:19:22414379
19	rs2099347	22282614	T	0.224	1.77E-12	chr:19:22414379
19	rs452707	22304145	T	0.224	1.77E-12	chr:19:22414379
19	rs4553749	22285063	C	0.224	1.77E-12	chr:19:22414379
19	rs6511323	22281914	T	0.224	1.77E-12	chr:19:22414379
19	rs6511325	22284357	A	0.224	1.77E-12	chr:19:22414379
19	rs6511326	22286679	T	0.224	1.77E-12	chr:19:22414379
19	rs6511329	22287905	T	0.224	1.77E-12	chr:19:22414379
19	rs4932954	22272454	T	0.2282	1.91E-12	chr:19:22414379
19	rs9304994	22272743	G	0.2282	1.91E-12	chr:19:22414379
19	rs2043318	22241615	G	0.2286	1.93E-12	chr:19:22414379
19	rs3752163	22256396	G	0.2286	1.93E-12	chr:19:22414379
19	rs4932953	22249045	G	0.2286	1.93E-12	chr:19:22414379
19	rs6511318	22245745	A	0.2286	1.93E-12	chr:19:22414379
19	rs7251108	22250689	G	0.2286	1.93E-12	chr:19:22414379
19	rs2116895	22266669	T	0.2298	1.94E-12	chr:19:22414379
19	rs6511320	22267122	C	0.2298	1.94E-12	chr:19:22414379
19	rs7258192	22264658	G	0.2298	1.94E-12	chr:19:22414379
19	rs2082478	22539848	A	8.01	2.08E-12	chr:19:22414379
19	rs4932758	22540094	A	8.01	2.08E-12	chr:19:22414379
19	rs55851405	22541686	C	8.01	2.08E-12	chr:19:22414379
19	rs1704019	22633165	T	6.018	2.20E-12	chr:19:22414379
19	rs1692595	22634726	T	5.983	2.20E-12	chr:19:22414379
19	rs55921217	22296943	C	0.2246	2.23E-12	chr:19:22414379
19	rs2957817	22637364	A	6.036	2.34E-12	chr:19:22414379
19	rs60200804	22283368	T	0.2266	2.42E-12	chr:19:22414379
19	rs12460293	22543380	A	7.782	2.48E-12	chr:19:22414379

19	rs403850	22229593	T	0.2296	2.52E-12	chr:19:22414379
19	rs417331	22234478	T	0.2296	2.52E-12	chr:19:22414379
19	rs4244913	22239919	A	0.2296	2.52E-12	chr:19:22414379
19	rs632709	22233096	C	0.2296	2.52E-12	chr:19:22414379
19	rs6511332	22288391	A	0.2272	2.52E-12	chr:19:22414379
19	rs8107039	22238990	G	0.2296	2.52E-12	chr:19:22414379
19	rs2359995	22280060	C	0.2298	2.55E-12	chr:19:22414379
19	rs12459747	22542982	G	7.666	2.73E-12	chr:19:22414379
19	rs4932748	22279318	T	0.2313	2.73E-12	chr:19:22414379
19	rs10403779	22258396	C	0.2317	2.77E-12	chr:19:22414379
19	rs28881618	22254692	G	0.2317	2.77E-12	chr:19:22414379
19	rs7254363	22257048	C	0.2317	2.77E-12	chr:19:22414379
19	rs7255666	22257264	T	0.2317	2.77E-12	chr:19:22414379
19	rs7258080	22257625	A	0.2317	2.77E-12	chr:19:22414379
19	rs1692593	22633259	T	5.852	2.90E-12	chr:19:22414379
19	rs2453888	22632145	G	5.852	2.90E-12	chr:19:22414379
19	rs639356	22104986	T	0.2156	3.08E-12	chr:19:22414379
19	rs1692598	22635052	A	5.869	3.11E-12	chr:19:22414379
19	rs1036235	22279541	T	0.2324	3.49E-12	chr:19:22414379
19	rs366849	22234941	G	0.2319	3.50E-12	chr:19:22414379
19	rs6511333	22298363	T	0.2344	4.42E-12	chr:19:22414379
19	rs8103088	22298322	G	0.2344	4.42E-12	chr:19:22414379
19	rs56149942	22541575	G	7.49	4.62E-12	chr:19:22414379
19	rs73027904	22533823	T	7.593	4.73E-12	chr:19:22414379
19	rs12462488	22565096	C	6.924	5.45E-12	chr:19:22414379
19	rs1603765	22635852	T	5.622	5.81E-12	chr:19:22414379
19	rs1704015	22636585	G	5.622	5.81E-12	chr:19:22414379
19	rs1230299	22624040	C	5.518	6.03E-12	chr:19:22414379
19	rs10408261	22298225	T	0.236	6.04E-12	chr:19:22414379
19	rs111498572	22583766	A	0.2356	8.18E-12	chr:19:22414379
19	rs1651558	22089575	T	5.333	8.89E-12	chr:19:22414379
19	rs4932992	22628842	T	5.26	9.20E-12	chr:19:22414379
19	rs1692590	22629695	T	5.269	1.06E-11	chr:19:22414379
19	rs1704017	22629868	C	5.312	1.13E-11	chr:19:22414379
19	rs281159	22416987	A	2576	1.16E-11	chr:19:22414379
19	rs2935281	22630650	A	5.154	1.34E-11	chr:19:22414379
19	rs73029815	22623948	T	6.328	1.59E-11	chr:19:22414379
19	rs7249109	22577764	C	6.73	1.62E-11	chr:19:22414379
19	rs903937	22609988	G	6.085	1.64E-11	chr:19:22414379
19	rs17461312	22554107	T	6.39	1.66E-11	chr:19:22414379
19	rs2957824	22579316	T	6.39	1.66E-11	chr:19:22414379
19	rs3867189	22581121	C	6.39	1.66E-11	chr:19:22414379
19	rs6511352	22599758	A	6.39	1.66E-11	chr:19:22414379

19	rs8107578	22564441	A	6.39	1.66E-11	chr:19:22414379
19	rs8108891	22595082	T	6.39	1.66E-11	chr:19:22414379
19	rs8112170	22565967	A	6.39	1.66E-11	chr:19:22414379
19	rs10401339	22210401	T	0.2497	1.67E-11	chr:19:22414379
19	rs403997	22220543	C	0.2497	1.67E-11	chr:19:22414379
19	rs424066	22222884	C	0.2497	1.67E-11	chr:19:22414379
19	rs437192	22219510	G	0.2497	1.67E-11	chr:19:22414379
19	rs452459	22216712	G	0.2497	1.67E-11	chr:19:22414379
19	rs1985744	22208601	T	0.2514	1.78E-11	chr:19:22414379
19	rs2158106	22186710	T	0.2514	1.78E-11	chr:19:22414379
19	rs417947	22224030	A	0.2514	1.78E-11	chr:19:22414379
19	rs12985553	22150868	T	0.2507	1.82E-11	chr:19:22414379
19	rs1809825	22608943	A	6.123	1.94E-11	chr:19:22414379
19	rs12975751	22156482	T	0.254	1.96E-11	chr:19:22414379
19	rs12979885	22160709	T	0.254	1.96E-11	chr:19:22414379
19	rs12980606	22169549	G	0.254	1.96E-11	chr:19:22414379
19	rs4265943	22173740	A	0.254	1.96E-11	chr:19:22414379
19	rs1692591	22632743	G	4.98	2.36E-11	chr:19:22414379
19	rs1692592	22632854	G	4.98	2.36E-11	chr:19:22414379
19	rs440539	22359330	A	2310	2.46E-11	chr:19:22414379
19	rs10410114	22192671	T	0.2539	2.46E-11	chr:19:22414379
19	rs11667684	22129638	T	0.2534	2.53E-11	chr:19:22414379
19	rs12971688	22144040	A	0.2534	2.53E-11	chr:19:22414379
19	rs2428382	22137765	G	0.2534	2.53E-11	chr:19:22414379
19	rs2666454	22127730	A	0.2534	2.53E-11	chr:19:22414379
19	rs2359816	22198676	A	0.2539	2.54E-11	chr:19:22414379
19	rs4458136	22197603	T	0.2539	2.54E-11	chr:19:22414379
19	rs113242209	22547737	T	6.28	2.63E-11	chr:19:22414379
19	rs12608935	22145147	C	0.256	2.78E-11	chr:19:22414379
19	rs1606063	22134408	A	0.256	2.78E-11	chr:19:22414379
19	rs1849005	22142743	C	0.256	2.78E-11	chr:19:22414379
19	rs2188972	22149458	G	0.256	2.78E-11	chr:19:22414379
19	rs2666455	22127535	G	0.256	2.78E-11	chr:19:22414379
19	rs11085508	22193986	C	0.2536	2.80E-11	chr:19:22414379
19	rs4550595	22153808	T	0.2536	2.80E-11	chr:19:22414379
19	rs12976630	22590340	A	6.342	3.22E-11	chr:19:22414379
19	rs6511351	22595741	C	6.342	3.22E-11	chr:19:22414379
19	rs67769716	22567459	A	6.342	3.22E-11	chr:19:22414379
19	rs7343076	22367903	C	2397	3.62E-11	chr:19:22414379
19	rs10403375	22389248	T	2613	3.77E-11	chr:19:22414379
19	rs2935279	22626467	C	4.726	4.25E-11	chr:19:22414379
19	rs2935280	22628754	C	4.742	4.39E-11	chr:19:22414379
19	rs2023338	22443261	T	20.87	4.46E-11	chr:19:22414379

19	rs374195570	22598418	T	5.405	4.82E-11	chr:19:22414379
19	rs377199228	22598420	C	5.405	4.82E-11	chr:19:22414379
19	rs150844285	22411568	T	3846	5.06E-11	chr:19:22414379
19	rs11668920	22182053	C	0.2612	5.31E-11	chr:19:22414379
19	rs699639	22120805	C	0.264	5.75E-11	chr:19:22414379
19	rs34643838	22608433	T	0.2866	6.21E-11	chr:19:22414379
19	rs10410914	22192884	A	0.2651	7.29E-11	chr:19:22414379
19	rs8105991	22193131	C	0.2651	7.29E-11	chr:19:22414379
19	rs2666441	22139230	A	0.2726	7.81E-11	chr:19:22414379
19	rs10415826	22248317	T	12.73	8.22E-11	chr:19:22414379
19	rs10415080	22150937	C	0.2661	8.63E-11	chr:19:22414379
19	rs10425763	22155918	C	0.2688	8.98E-11	chr:19:22414379
19	rs10426971	22158167	A	0.2688	8.98E-11	chr:19:22414379
19	rs2214298	22163606	C	0.2688	8.98E-11	chr:19:22414379
19	rs74776957	22172805	G	0.2688	8.98E-11	chr:19:22414379
19	rs75181699	22172720	G	0.2688	8.98E-11	chr:19:22414379
19	rs10404998	22608374	C	0.2946	9.34E-11	chr:19:22414379
19	rs10405295	22608556	C	0.2946	9.34E-11	chr:19:22414379
19	rs10416278	22607031	T	0.2946	9.34E-11	chr:19:22414379
19	rs8112041	22604565	T	0.2946	9.34E-11	chr:19:22414379
19	rs1624967	22634476	T	4.906	1.05E-10	chr:19:22414379
19	rs1849007	22142930	T	0.2711	1.28E-10	chr:19:22414379
19	rs2136888	22134791	G	0.2711	1.28E-10	chr:19:22414379
19	rs2175367	22125889	G	0.2711	1.28E-10	chr:19:22414379
19	rs2666451	22130481	C	0.2711	1.28E-10	chr:19:22414379
19	rs7251413	22609759	T	0.3018	1.35E-10	chr:19:22414379
19	rs10854064	22206129	T	0.2743	1.75E-10	chr:19:22414379
19	rs408124	22225337	G	0.2743	1.75E-10	chr:19:22414379
19	rs441017	22214122	C	0.2743	1.75E-10	chr:19:22414379
19	rs10408585	22613247	A	5.322	1.91E-10	chr:19:22414379
19	rs931608	22614122	A	5.322	1.91E-10	chr:19:22414379
19	rs2168492;rs568148804	22425645	T	0.207	1.94E-10	chr:19:22414379
19	rs11669179	22604729	G	0.3039	2.18E-10	chr:19:22414379
19	rs11669270	22604816	A	0.3039	2.18E-10	chr:19:22414379
19	rs11669353	22605103	C	0.3039	2.18E-10	chr:19:22414379
19	rs11879678	22607312	A	0.3039	2.18E-10	chr:19:22414379
19	rs11879697	22607206	A	0.3039	2.18E-10	chr:19:22414379
19	rs34787405	22608148	A	0.3039	2.18E-10	chr:19:22414379
19	rs1912572	22140878	C	0.2786	2.76E-10	chr:19:22414379
19	rs11669356	22605120	T	0.3075	2.76E-10	chr:19:22414379
19	rs1654509	22219163	T	0.2789	2.82E-10	chr:19:22414379
19	rs34187872	22607883	A	0.307	2.84E-10	chr:19:22414379
19	rs8101947	22605640	T	0.3074	3.10E-10	chr:19:22414379

19	rs2664067	22638066	C	0.3175	3.40E-10	chr:19:22414379
19	rs55992911	22397853	C	29.82	3.66E-10	chr:19:22414379
19	rs1230297	22622753	G	4.911	3.88E-10	chr:19:22414379
19	rs56144642	22421516	C	0.2493	4.03E-10	chr:19:22414379
19	rs10424932	22194604	T	0.2741	4.53E-10	chr:19:22414379
19	rs17470104	22424418	A	0.2539	5.18E-10	chr:19:22414379
19	rs10401167	22291942	A	12.64	5.85E-10	chr:19:22414379
19	rs10418804	22273015	C	12.64	5.85E-10	chr:19:22414379
19	rs10423457	22279267	C	12.64	5.85E-10	chr:19:22414379
19	rs10424447	22274042	G	12.64	5.85E-10	chr:19:22414379
19	rs2043319	22274595	G	12.64	5.85E-10	chr:19:22414379
19	rs28374572	22276478	C	12.64	5.85E-10	chr:19:22414379
19	rs62111040	22287511	A	12.64	5.85E-10	chr:19:22414379
19	rs6511321	22270068	G	12.64	5.85E-10	chr:19:22414379
19	rs6511328	22286979	A	12.64	5.85E-10	chr:19:22414379
19	rs8100768	22270468	C	12.64	5.85E-10	chr:19:22414379
19	rs2522097	22123140	C	0.2928	6.99E-10	chr:19:22414379
19	rs2666458	22123138	A	0.2928	6.99E-10	chr:19:22414379
19	rs7259807	22291476	G	12.33	8.56E-10	chr:19:22414379
19	rs11085534	22613229	T	4.552	9.85E-10	chr:19:22414379
19	rs10417916	22272615	C	10.08	9.88E-10	chr:19:22414379
19	rs10425828	22283077	G	10.08	9.88E-10	chr:19:22414379
19	rs10426159	22267324	A	10.08	9.88E-10	chr:19:22414379
19	rs1968446	22279047	C	10.08	9.88E-10	chr:19:22414379
19	rs28547927	22277137	A	10.08	9.88E-10	chr:19:22414379
19	rs28600030	22268443	A	10.08	9.88E-10	chr:19:22414379
19	rs7256462	22281993	C	10.08	9.88E-10	chr:19:22414379
19	rs8103279	22284340	G	10.08	9.88E-10	chr:19:22414379
19	rs8103374	22284319	T	10.08	9.88E-10	chr:19:22414379
19	rs8106762	22284736	T	10.08	9.88E-10	chr:19:22414379
19	rs10401922	22253252	A	12.15	9.94E-10	chr:19:22414379
19	rs10404669	22241218	C	12.15	9.94E-10	chr:19:22414379
19	rs10405613	22244772	T	12.15	9.94E-10	chr:19:22414379
19	rs10405673	22244890	G	12.15	9.94E-10	chr:19:22414379
19	rs10406119	22245009	A	12.15	9.94E-10	chr:19:22414379
19	rs10406485	22245002	T	12.15	9.94E-10	chr:19:22414379
19	rs10406551	22244520	A	12.15	9.94E-10	chr:19:22414379
19	rs10406553	22237801	T	12.15	9.94E-10	chr:19:22414379
19	rs10406653	22238023	T	12.15	9.94E-10	chr:19:22414379
19	rs10407173	22246663	T	12.15	9.94E-10	chr:19:22414379
19	rs10407250	22238151	A	12.15	9.94E-10	chr:19:22414379
19	rs10408150	22245225	C	12.15	9.94E-10	chr:19:22414379
19	rs10412953	22238482	G	12.15	9.94E-10	chr:19:22414379

19	rs10413784	22246191	G	12.15	9.94E-10	chr:19:22414379
19	rs10414685	22247749	T	12.15	9.94E-10	chr:19:22414379
19	rs10414861	22247761	G	12.15	9.94E-10	chr:19:22414379
19	rs10415794	22248221	T	12.15	9.94E-10	chr:19:22414379
19	rs10415930	22248088	A	12.15	9.94E-10	chr:19:22414379
19	rs10417285	22248184	C	12.15	9.94E-10	chr:19:22414379
19	rs10419142	22252262	T	12.15	9.94E-10	chr:19:22414379
19	rs10419184	22252342	G	12.15	9.94E-10	chr:19:22414379
19	rs10420019	22251905	A	12.15	9.94E-10	chr:19:22414379
19	rs10420898	22252331	C	12.15	9.94E-10	chr:19:22414379
19	rs10422289	22256060	T	12.15	9.94E-10	chr:19:22414379
19	rs10423114	22249289	G	12.15	9.94E-10	chr:19:22414379
19	rs10423522	22243448	T	12.15	9.94E-10	chr:19:22414379
19	rs10423881	22243422	A	12.15	9.94E-10	chr:19:22414379
19	rs2043316	22251204	T	12.15	9.94E-10	chr:19:22414379
19	rs2043317	22251096	C	12.15	9.94E-10	chr:19:22414379
19	rs2163835	22242679	T	12.15	9.94E-10	chr:19:22414379
19	rs2163836	22242277	T	12.15	9.94E-10	chr:19:22414379
19	rs2163837	22242236	A	12.15	9.94E-10	chr:19:22414379
19	rs2359833	22242221	G	12.15	9.94E-10	chr:19:22414379
19	rs28822164	22245108	A	12.15	9.94E-10	chr:19:22414379
19	rs28857211	22243104	C	12.15	9.94E-10	chr:19:22414379
19	rs28894669	22243264	A	12.15	9.94E-10	chr:19:22414379
19	rs4257328	22240090	A	12.15	9.94E-10	chr:19:22414379
19	rs4510150	22239669	G	12.15	9.94E-10	chr:19:22414379
19	rs4542784	22239751	A	12.15	9.94E-10	chr:19:22414379
19	rs6511315	22236503	A	12.15	9.94E-10	chr:19:22414379
19	rs6511316	22237629	C	12.15	9.94E-10	chr:19:22414379
19	rs7247147	22249858	C	12.15	9.94E-10	chr:19:22414379
19	rs7247228	22250136	T	12.15	9.94E-10	chr:19:22414379
19	rs7248305	22250271	A	12.15	9.94E-10	chr:19:22414379
19	rs7250852	22250510	A	12.15	9.94E-10	chr:19:22414379
19	rs7250905	22250743	T	12.15	9.94E-10	chr:19:22414379
19	rs7251660	22250614	C	12.15	9.94E-10	chr:19:22414379
19	rs7251884	22245530	T	12.15	9.94E-10	chr:19:22414379
19	rs7252023	22245646	G	12.15	9.94E-10	chr:19:22414379
19	rs7252550	22246017	T	12.15	9.94E-10	chr:19:22414379
19	rs7259971	22237319	G	12.15	9.94E-10	chr:19:22414379
19	rs8103518	22246952	C	12.15	9.94E-10	chr:19:22414379
19	rs8104144	22246913	C	12.15	9.94E-10	chr:19:22414379
19	rs8104630	22247277	C	12.15	9.94E-10	chr:19:22414379
19	rs8108651	22251464	G	12.15	9.94E-10	chr:19:22414379
19	rs8108976	22251701	A	12.15	9.94E-10	chr:19:22414379

19	rs9653142	22249596	G	12.15	9.94E-10	chr:19:22414379
19	rs9653157	22249490	C	12.15	9.94E-10	chr:19:22414379
19	rs9973249	22242160	A	12.15	9.94E-10	chr:19:22414379
19	rs13344143	22531045	T	4.762	1.02E-09	chr:19:22414379
19	rs28635943	22268712	T	9.977	1.04E-09	chr:19:22414379
19	rs17701615	22102137	G	0.2798	1.05E-09	chr:19:22414379
19	rs11671130	22329455	C	0.2586	1.13E-09	chr:19:22414379
19	rs11672659	22394767	T	0.2586	1.13E-09	chr:19:22414379
19	rs149070103	22340413	G	0.2586	1.13E-09	chr:19:22414379
19	rs1852420	22361902	T	0.2586	1.13E-09	chr:19:22414379
19	rs4390714	22369043	A	0.2586	1.13E-09	chr:19:22414379
19	rs4578792	22368982	G	0.2586	1.13E-09	chr:19:22414379
19	rs4602157	22339036	C	0.2586	1.13E-09	chr:19:22414379
19	rs4616412	22339005	G	0.2586	1.13E-09	chr:19:22414379
19	rs4932750	22333226	T	0.2586	1.13E-09	chr:19:22414379
19	rs76490109	22330225	A	0.2586	1.13E-09	chr:19:22414379
19	rs8110969	22240222	T	13.82	1.15E-09	chr:19:22414379
19	rs62112369	22313901	T	13.04	1.17E-09	chr:19:22414379
19	rs62119972	22425682	C	10.59	1.23E-09	chr:19:22414379
19	rs11668081	22425615	A	0.2684	1.35E-09	chr:19:22414379
19	rs8111854	22240213	C	11.96	1.35E-09	chr:19:22414379
19	rs4123726	22419677	T	0.2642	1.35E-09	chr:19:22414379
19	rs11085533	22612880	T	4.463	1.50E-09	chr:19:22414379
19	rs1830174	22344502	A	0.2641	1.67E-09	chr:19:22414379
19	rs4381717	22338981	A	0.2649	1.70E-09	chr:19:22414379
19	rs10411943	22259782	G	9.688	1.73E-09	chr:19:22414379
19	rs10420042	22251926	A	9.688	1.73E-09	chr:19:22414379
19	rs2043315	22252823	C	9.688	1.73E-09	chr:19:22414379
19	rs62110990	22260441	T	9.688	1.73E-09	chr:19:22414379
19	rs6511314	22236395	A	9.688	1.73E-09	chr:19:22414379
19	rs7247041	22249790	A	9.688	1.73E-09	chr:19:22414379
19	rs7249591	22245345	C	9.688	1.73E-09	chr:19:22414379
19	rs7253053	22245685	C	9.688	1.73E-09	chr:19:22414379
19	rs8110047	22239502	G	9.688	1.73E-09	chr:19:22414379
19	rs8110841	22239436	C	9.688	1.73E-09	chr:19:22414379
19	rs12609320	22390462	C	0.2656	1.85E-09	chr:19:22414379
19	rs1609037	22380261	C	0.2656	1.85E-09	chr:19:22414379
19	rs4932962	22372000	T	0.2656	1.85E-09	chr:19:22414379
19	rs6511327	22286899	A	13.14	1.90E-09	chr:19:22414379
19	rs11671580	22326439	A	0.2649	1.95E-09	chr:19:22414379
19	rs10411275	22259469	G	9.572	2.04E-09	chr:19:22414379
19	rs1230296	22622638	G	4.216	2.09E-09	chr:19:22414379
19	rs74416263	22342767	T	0.2681	2.54E-09	chr:19:22414379

19	rs10405452	22529868	T	3.966	3.03E-09	chr:19:22414379
19	rs16999012	22522950	G	3.925	3.04E-09	chr:19:22414379
19	rs11666326	22377109	A	0.2683	3.25E-09	chr:19:22414379
19	rs11672740	22394859	A	0.2683	3.25E-09	chr:19:22414379
19	rs4372816	22328376	G	0.2683	3.25E-09	chr:19:22414379
19	rs1230294	22620820	T	4.122	3.40E-09	chr:19:22414379
19	rs1230298	22622846	C	4.122	3.40E-09	chr:19:22414379
19	rs111790701	22438463	T	17.71	3.87E-09	chr:19:22414379
19	rs4123729	22418142	C	0.2748	3.98E-09	chr:19:22414379
19	rs55758909	22420558	A	0.2748	3.98E-09	chr:19:22414379
19	rs7246007	22240596	C	14.66	4.43E-09	chr:19:22414379
19	rs1230295	22621678	C	0.361	4.56E-09	chr:19:22414379
19	rs5827519	22280532	G	10.94	4.74E-09	chr:19:22414379
19	rs1976314	22608853	T	0.3463	4.77E-09	chr:19:22414379
19	rs17554694	22317148	A	23.03	4.93E-09	chr:19:22414379
19	rs7247584	22529565	T	3.834	5.06E-09	chr:19:22414379
19	rs78646027	22257410	T	10.94	5.12E-09	chr:19:22414379
19	rs1118049	22642131	T	0.367	5.34E-09	chr:19:22414379
19	rs1230304	22641598	C	0.367	5.34E-09	chr:19:22414379
19	rs1230305	22641917	C	0.367	5.34E-09	chr:19:22414379
19	rs1230307	22642684	T	0.367	5.34E-09	chr:19:22414379
19	rs1230308	22642916	C	0.367	5.34E-09	chr:19:22414379
19	rs11667657	22393601	C	0.2756	5.36E-09	chr:19:22414379
19	rs12977311	22606001	A	0.3055	5.45E-09	chr:19:22414379
19	rs62112373	22319825	A	22.83	5.52E-09	chr:19:22414379
19	rs2885037	22606672	A	0.3107	6.15E-09	chr:19:22414379
19	rs555185638	22290846	C	13.82	6.52E-09	chr:19:22414379
19	rs66562925	22158713	A	4.171	6.74E-09	chr:19:22414379
19	rs12982366	22449420	G	0.2712	6.89E-09	chr:19:22414379
19	rs2957849	22583761	A	0.3337	7.58E-09	chr:19:22414379
19	rs111684505	22401097	G	0.2825	8.45E-09	chr:19:22414379
19	rs2645164	22638080	T	0.3716	9.62E-09	chr:19:22414379
19	rs2957814	22637580	T	0.375	1.33E-08	chr:19:22414379
19	rs61616269	22397866	A	3.977	1.82E-08	chr:19:22414379
19	rs12986319	22364233	T	0.2763	2.06E-08	chr:19:22414379
19	rs11878866	22144367	C	15.84	2.12E-08	chr:19:22414379
19	rs12609595	22614478	T	0.3352	2.56E-08	chr:19:22414379
19	rs7260331	22291771	T	30.02	2.65E-08	chr:19:22414379
19	rs62112374	22329197	T	59.48	2.90E-08	chr:19:22414379
19	rs62112416	22343674	C	59.48	2.90E-08	chr:19:22414379
19	rs3867185	22413097	T	58.79	3.13E-08	chr:19:22414379
19	rs60866819	22334909	C	58.79	3.13E-08	chr:19:22414379
19	rs62112420	22351653	G	58.79	3.13E-08	chr:19:22414379

19	rs62112447	22379833	G	58.79	3.13E-08	chr:19:22414379
19	rs62118346	22396430	A	58.79	3.13E-08	chr:19:22414379
19	rs62119969	22405446	C	58.79	3.13E-08	chr:19:22414379
19	rs12976555	22434811	T	0.2842	3.26E-08	chr:19:22414379
19	rs58950778	22432275	T	57.87	3.57E-08	chr:19:22414379
19	rs12151358	22282608	A	19.26	3.68E-08	chr:19:22414379
19	rs145591522	22282893	G	19.26	3.68E-08	chr:19:22414379
19	rs16998834	22267037	C	19.26	3.68E-08	chr:19:22414379
19	rs1975169	22292098	A	19.26	3.68E-08	chr:19:22414379
19	rs62110993	22268260	A	19.26	3.68E-08	chr:19:22414379
19	rs62111027	22269019	C	19.26	3.68E-08	chr:19:22414379
19	rs62111028	22272397	T	19.26	3.68E-08	chr:19:22414379
19	rs62111029	22276601	A	19.26	3.68E-08	chr:19:22414379
19	rs62111030	22279375	A	19.26	3.68E-08	chr:19:22414379
19	rs62111032	22281349	G	19.26	3.68E-08	chr:19:22414379
19	rs62111033	22283395	A	19.26	3.68E-08	chr:19:22414379
19	rs62111034	22284720	C	19.26	3.68E-08	chr:19:22414379
19	rs62111037	22286563	T	19.26	3.68E-08	chr:19:22414379
19	rs62111039	22287336	G	19.26	3.68E-08	chr:19:22414379
19	rs62112362	22293113	G	19.26	3.68E-08	chr:19:22414379
19	rs62112364	22294903	A	19.26	3.68E-08	chr:19:22414379
19	rs7252547	22305481	C	19.26	3.68E-08	chr:19:22414379
19	rs7254050	22286741	A	19.26	3.68E-08	chr:19:22414379
19	rs959908	22281893	C	19.26	3.68E-08	chr:19:22414379
19	rs8104559	22205167	C	10.5	3.68E-08	chr:19:22414379
19	rs117638181	22163014	G	19.18	4.76E-08	chr:19:22414379
19	rs11881563	22144992	T	19.18	4.76E-08	chr:19:22414379
19	rs11882234	22145727	T	19.18	4.76E-08	chr:19:22414379
19	rs1849004	22142455	C	19.18	4.76E-08	chr:19:22414379
19	rs2359820	22208114	T	19.18	4.76E-08	chr:19:22414379
19	rs57912102	22136043	C	19.18	4.76E-08	chr:19:22414379
19	rs62112865	22172333	T	19.18	4.76E-08	chr:19:22414379
19	rs62112870	22175976	A	19.18	4.76E-08	chr:19:22414379
19	rs62112871	22185295	C	19.18	4.76E-08	chr:19:22414379
19	rs62112872	22192425	C	19.18	4.76E-08	chr:19:22414379
19	rs75167249	22128655	T	19.18	4.76E-08	chr:19:22414379
19	rs8100042	22127841	A	19.18	4.76E-08	chr:19:22414379
19	rs8113460	22128006	T	19.18	4.76E-08	chr:19:22414379
19	rs116220423	22237041	A	18.63	5.31E-08	chr:19:22414379
19	rs62121863	22504673	A	12.02	6.79E-08	chr:19:22414379
19	rs62120019	22493958	T	10.17	8.35E-08	chr:19:22414379
19	rs11881224	22220340	A	25.75	1.21E-07	chr:19:22414379
19	rs12150930	22223687	C	25.75	1.21E-07	chr:19:22414379

19	rs17450788	22224755	C	25.75	1.21E-07	chr:19:22414379
19	rs2078180	22209846	A	25.75	1.21E-07	chr:19:22414379
19	rs60548522	22214874	A	25.75	1.21E-07	chr:19:22414379
19	rs62112907	22209569	G	25.75	1.21E-07	chr:19:22414379
19	rs62112908	22213956	G	25.75	1.21E-07	chr:19:22414379
19	rs62112913	22221067	G	25.75	1.21E-07	chr:19:22414379
19	rs62112915	22223179	A	25.75	1.21E-07	chr:19:22414379
19	rs6511313	22215905	A	25.75	1.21E-07	chr:19:22414379
19	rs8105895	22215457	T	25.75	1.21E-07	chr:19:22414379
19	rs8112751	22222458	T	25.75	1.21E-07	chr:19:22414379
19	rs369217217	22289248	C	25.19	1.22E-07	chr:19:22414379
19	rs62111036	22286451	G	8.712	1.40E-07	chr:19:22414379
19	rs76584077	22286457	C	8.712	1.40E-07	chr:19:22414379
19	rs59020942	22427750	T	0.3418	1.66E-07	chr:19:22414379
19	rs59574273	22427752	T	0.3418	1.66E-07	chr:19:22414379
19	rs111575233	22430482	A	0.3487	2.04E-07	chr:19:22414379
19	rs55784860	22431132	C	0.3487	2.04E-07	chr:19:22414379
19	rs148710998	22577980	G	3.419	2.53E-07	chr:19:22414379
19	rs550066332	22577974	C	3.419	2.53E-07	chr:19:22414379
19	rs60604817	22427565	G	0.3482	2.56E-07	chr:19:22414379
19	rs11561612	22432845	A	0.3462	3.15E-07	chr:19:22414379
19	rs28792372	22096971	C	0.3683	3.90E-07	chr:19:22414379
19	rs4932761	22546375	A	0.3272	4.55E-07	chr:19:22414379
19	rs11085516	22431751	T	0.3504	4.62E-07	chr:19:22414379
19	rs62118563	22529531	A	6.722	4.66E-07	chr:19:22414379
19	rs79005568	22288069	G	40.11	5.16E-07	chr:19:22414379
19	rs117976739	22445319	T	8.666	5.57E-07	chr:19:22414379
19	rs113023530	22261617	A	0.351	5.81E-07	chr:19:22414379
19	rs3813590	22321273	T	0.3522	6.34E-07	chr:19:22414379
19	rs3813592	22321245	G	0.3522	6.34E-07	chr:19:22414379
19	rs12104137	22659822	G	0.4335	6.43E-07	chr:19:22414379
19	rs10411905	22427035	G	0.392	6.88E-07	chr:19:22414379
19	rs1526884	22303651	G	0.3571	7.14E-07	chr:19:22414379
19	rs410794	22226159	A	0.3545	7.44E-07	chr:19:22414379
19	rs79846980	22141343	C	38.34	7.59E-07	chr:19:22414379
19	rs12610034	22314528	A	0.3588	7.69E-07	chr:19:22414379
19	rs8107022	22618698	T	2.966	1.03E-06	chr:19:22414379
19	rs111352604	22314567	G	0.3654	1.17E-06	chr:19:22414379
19	rs11669110	22296485	G	0.3654	1.17E-06	chr:19:22414379
19	rs11673195	22313814	C	0.3654	1.17E-06	chr:19:22414379
19	rs149975047	22287938	A	0.3654	1.17E-06	chr:19:22414379
19	rs191067451	22288201	A	0.3654	1.17E-06	chr:19:22414379
19	rs367972720	22291197	C	0.3654	1.17E-06	chr:19:22414379

19	rs372140746	22291198	C	0.3654	1.17E-06	chr:19:22414379
19	rs372252339	22291126	T	0.3654	1.17E-06	chr:19:22414379
19	rs4932957	22304745	T	0.3654	1.17E-06	chr:19:22414379
19	rs56207853	22292357	T	0.3654	1.17E-06	chr:19:22414379
19	rs61268244	22310130	T	0.3654	1.17E-06	chr:19:22414379
19	rs72481609	22296819	C	0.3654	1.17E-06	chr:19:22414379
19	rs73019900	22286680	A	0.3654	1.17E-06	chr:19:22414379
19	rs73021832	22316144	C	0.3654	1.17E-06	chr:19:22414379
19	rs62112863	22164515	G	35.93	1.19E-06	chr:19:22414379
19	rs1230293	22620302	T	2.924	1.58E-06	chr:19:22414379
19	rs17459246	22278121	C	0.3673	1.61E-06	chr:19:22414379
19	rs55886600	22276437	A	0.3673	1.61E-06	chr:19:22414379
19	rs56073035	22276298	G	0.3673	1.61E-06	chr:19:22414379
19	rs73019889	22277826	G	0.3673	1.61E-06	chr:19:22414379
19	rs12610367	22320848	T	0.3775	1.66E-06	chr:19:22414379
19	rs183543741	22289845	T	0.3774	1.69E-06	chr:19:22414379
19	rs193152926	22289796	A	0.3774	1.69E-06	chr:19:22414379
19	rs375012052	22289851	A	0.3774	1.69E-06	chr:19:22414379
19	rs199643795	22291325	A	0.3723	1.72E-06	chr:19:22414379
19	rs10426665	22428809	G	0.3999	2.10E-06	chr:19:22414379
19	rs283161	22456131	A	2.72	2.22E-06	chr:19:22414379
19	rs7246504	22532831	G	5.04	2.32E-06	chr:19:22414379
19	rs4932949	22238666	A	0.38	2.45E-06	chr:19:22414379
19	rs11671180	22184030	C	0.304	2.54E-06	chr:19:22414379
19	rs12609368	22263996	C	0.3756	2.58E-06	chr:19:22414379
19	rs1978719	22266101	A	0.3756	2.58E-06	chr:19:22414379
19	rs56369285	22267185	C	0.3756	2.58E-06	chr:19:22414379
19	rs283136	22466736	A	2.633	2.65E-06	chr:19:22414379
19	rs808371	22471055	T	2.633	2.65E-06	chr:19:22414379
19	rs12608810	22342002	A	0.314	3.13E-06	chr:19:22414379
19	rs4932746	22269847	T	0.3784	3.44E-06	chr:19:22414379
19	rs283137	22464916	G	2.608	3.66E-06	chr:19:22414379
19	rs11667209	22259467	G	0.3877	3.68E-06	chr:19:22414379
19	rs11667274	22259666	G	0.3877	3.68E-06	chr:19:22414379
19	rs149619063	22248189	G	0.3877	3.68E-06	chr:19:22414379
19	rs4932950	22243488	G	0.3877	3.68E-06	chr:19:22414379
19	rs4932952	22246788	A	0.3877	3.68E-06	chr:19:22414379
19	rs55735642	22241176	T	0.3877	3.68E-06	chr:19:22414379
19	rs73019838	22247732	A	0.3877	3.68E-06	chr:19:22414379
19	rs148747862	22177981	C	6.038	3.70E-06	chr:19:22414379
19	rs55728714	22262899	G	0.3825	3.71E-06	chr:19:22414379
19	rs112205850	22261876	A	0.3835	3.87E-06	chr:19:22414379
19	rs1091172	22432373	C	0.4103	4.38E-06	chr:19:22414379

19	rs145534553	22459643	T	6.223	4.62E-06	chr:19:22414379
19	rs1018912	22156585	G	0.3922	4.69E-06	chr:19:22414379
19	rs142623791	22173025	A	0.3922	4.69E-06	chr:19:22414379
19	rs2017521	22126138	T	0.3922	4.69E-06	chr:19:22414379
19	rs2175366	22128279	C	0.3922	4.69E-06	chr:19:22414379
19	rs2522104	22140849	T	0.3922	4.69E-06	chr:19:22414379
19	rs2666447	22132868	T	0.3922	4.69E-06	chr:19:22414379
19	rs4290587	22164646	A	0.3922	4.69E-06	chr:19:22414379
19	rs6511309	22174292	T	0.3922	4.69E-06	chr:19:22414379
19	rs765527	22129555	C	0.3922	4.69E-06	chr:19:22414379
19	rs9636187	22173370	T	0.3922	4.69E-06	chr:19:22414379
19	rs183691981	22291525	C	0.3995	5.54E-06	chr:19:22414379
19	rs73019842	22249909	G	0.4011	5.96E-06	chr:19:22414379
19	rs73019844	22249925	C	0.4011	5.96E-06	chr:19:22414379
19	rs1989120	22205528	T	0.4037	6.11E-06	chr:19:22414379
19	rs10420483	22190378	A	0.3945	6.17E-06	chr:19:22414379
19	rs11085509	22204573	T	0.3945	6.17E-06	chr:19:22414379
19	rs1654508	22219276	A	0.3945	6.17E-06	chr:19:22414379
19	rs2017962	22208751	T	0.3945	6.17E-06	chr:19:22414379
19	rs2078089	22209753	A	0.3945	6.17E-06	chr:19:22414379
19	rs2106869	22184693	C	0.3945	6.17E-06	chr:19:22414379
19	rs2188969	22202783	A	0.3945	6.17E-06	chr:19:22414379
19	rs2188970	22200382	G	0.3945	6.17E-06	chr:19:22414379
19	rs2359821	22208314	C	0.3945	6.17E-06	chr:19:22414379
19	rs374537	22222802	G	0.3945	6.17E-06	chr:19:22414379
19	rs404822	22215967	A	0.3945	6.17E-06	chr:19:22414379
19	rs4244912	22203237	T	0.3945	6.17E-06	chr:19:22414379
19	rs429184	22214352	A	0.3945	6.17E-06	chr:19:22414379
19	rs436039	22214873	T	0.3945	6.17E-06	chr:19:22414379
19	rs7249976	22183920	A	0.3945	6.17E-06	chr:19:22414379
19	rs76446294	22269313	G	5.799	6.50E-06	chr:19:22414379
19	rs2262909	22135270	A	0.4045	7.58E-06	chr:19:22414379
19	rs58038983	22544165	T	2.844	7.81E-06	chr:19:22414379
19	rs390730	22233509	C	0.4077	9.87E-06	chr:19:22414379
19	rs408687	22231123	T	0.4077	9.87E-06	chr:19:22414379
19	rs409439	22227432	C	0.4077	9.87E-06	chr:19:22414379
19	rs409835	22230671	T	0.4077	9.87E-06	chr:19:22414379
19	rs411020	22231102	G	0.4077	9.87E-06	chr:19:22414379
19	rs422344	22230956	G	0.4077	9.87E-06	chr:19:22414379
19	rs425781	22229306	A	0.4077	9.87E-06	chr:19:22414379
19	rs436662	22232107	C	0.4077	9.87E-06	chr:19:22414379
19	rs62110991	22260842	T	88.92	1.09E-05	chr:19:22414379
19	rs564862240	22397855	C	10.81	1.11E-05	chr:19:22414379

19	rs143692149	22478366	A	5.618	1.44E-05	chr:19:22414379
19	rs76695697	22344687	A	0.3056	1.58E-05	chr:19:22414379
19	rs1560716	22305901	G	81.27	1.64E-05	chr:19:22414379
19	rs1560717	22292247	G	81.27	1.64E-05	chr:19:22414379
19	rs1975168	22305826	T	81.27	1.64E-05	chr:19:22414379
19	rs62110992	22261516	G	81.27	1.64E-05	chr:19:22414379
19	rs62111038	22286614	G	81.27	1.64E-05	chr:19:22414379
19	rs62112368	22306775	A	81.27	1.64E-05	chr:19:22414379
19	rs2359835	22242690	G	79.01	1.87E-05	chr:19:22414379
19	rs368701052	22255803	A	79.01	1.87E-05	chr:19:22414379
19	rs377529013	22242455	A	79.01	1.87E-05	chr:19:22414379
19	rs62110979	22245152	C	79.01	1.87E-05	chr:19:22414379
19	rs62110980	22245191	A	79.01	1.87E-05	chr:19:22414379
19	rs62110981	22246013	G	79.01	1.87E-05	chr:19:22414379
19	rs62110984	22252332	A	79.01	1.87E-05	chr:19:22414379
19	rs62110985	22256077	G	79.01	1.87E-05	chr:19:22414379
19	rs62110986	22256771	A	79.01	1.87E-05	chr:19:22414379
19	rs62110987	22257295	C	79.01	1.87E-05	chr:19:22414379
19	rs62110988	22259956	T	79.01	1.87E-05	chr:19:22414379
19	rs62110989	22260299	G	79.01	1.87E-05	chr:19:22414379
19	rs62112957	22238259	T	79.01	1.87E-05	chr:19:22414379
19	rs62112958	22239118	G	79.01	1.87E-05	chr:19:22414379
19	rs7252220	22245564	A	79.01	1.87E-05	chr:19:22414379
19	rs7252380	22245715	A	79.01	1.87E-05	chr:19:22414379
19	rs7252772	22245459	A	79.01	1.87E-05	chr:19:22414379
19	rs8110289	22239717	G	79.01	1.87E-05	chr:19:22414379
19	rs8110975	22240233	G	77.73	2.03E-05	chr:19:22414379
19	rs62112917	22226580	T	77.87	2.05E-05	chr:19:22414379
19	rs371889380	22289791	T	0.3804	2.07E-05	chr:19:22414379
19	rs988211	22302536	T	74.51	2.39E-05	chr:19:22414379
19	rs62112956	22235910	A	74.1	2.48E-05	chr:19:22414379
19	rs200235951	22368291	A	0.2603	2.60E-05	chr:19:22414379
19	rs62112918	22227079	T	73.22	2.62E-05	chr:19:22414379
19	rs62112919	22227145	C	73.22	2.62E-05	chr:19:22414379
19	rs62112920	22227149	G	73.22	2.62E-05	chr:19:22414379
19	rs62112921	22227577	C	73.22	2.62E-05	chr:19:22414379
19	rs62112922	22228340	T	73.22	2.62E-05	chr:19:22414379
19	rs62112955	22234959	T	73.22	2.62E-05	chr:19:22414379
19	rs75025987	22230669	A	73.22	2.62E-05	chr:19:22414379
19	rs200026439	29845668	T	57.68	3.27E-25	chr:19:29855781
19	rs201163831	29845670	T	57.68	3.27E-25	chr:19:29855781
19	rs62109918	29845664	T	50.99	2.69E-24	chr:19:29855781
19	rs62109919	29845666	T	50.99	2.69E-24	chr:19:29855781

19	rs200273877	29841554	A	31.1	8.59E-24	chr:19:29855781
19	rs201714995	29841536	A	31.1	8.59E-24	chr:19:29855781
19	rs62104321	29866339	C	27.57	1.02E-23	chr:19:29855781
19	rs10426495	29866098	C	39.13	7.14E-23	chr:19:29855781
19	rs9789288	29806349	T	43.54	1.16E-22	chr:19:29855781
19	rs78286516	29843274	C	48.24	3.82E-22	chr:19:29855781
19	rs78902520	29843271	A	48.24	3.82E-22	chr:19:29855781
19	rs35900872	29871153	A	60.1	9.42E-22	chr:19:29855781
19	rs1981676	29868320	A	59.68	1.03E-21	chr:19:29855781
19	rs758770	29870136	G	59.68	1.03E-21	chr:19:29855781
19	rs10401757	29866045	T	58.9	1.21E-21	chr:19:29855781
19	rs79585427	29843225	C	17.36	2.03E-21	chr:19:29855781
19	rs1558363	29865313	C	70.42	4.49E-21	chr:19:29855781
19	rs200009518	29841360	G	15.99	4.62E-21	chr:19:29855781
19	rs200136359	29843211	G	15.87	4.94E-21	chr:19:29855781
19	rs200658849	29843213	T	15.87	4.94E-21	chr:19:29855781
19	rs76108985	29843218	C	15.87	4.94E-21	chr:19:29855781
19	rs2193501	29834999	A	78.42	4.96E-21	chr:19:29855781
19	rs9749537	29844679	A	58.18	5.30E-21	chr:19:29855781
19	rs7249606	29866089	C	51.72	6.07E-21	chr:19:29855781
19	rs10403590	29844676	C	84.52	6.22E-21	chr:19:29855781
19	rs6417157	29858422	C	63.08	6.35E-21	chr:19:29855781
19	rs10853798	29821124	A	79.02	7.18E-21	chr:19:29855781
19	rs1119169	29850778	T	17.32	1.31E-20	chr:19:29855781
19	rs10411752	29826775	C	65.73	1.64E-20	chr:19:29855781
19	rs35184298	29830558	A	99.79	1.80E-20	chr:19:29855781
19	rs4805368	29828621	G	99.79	1.80E-20	chr:19:29855781
19	rs12150940	29840750	C	110.3	2.32E-20	chr:19:29855781
19	rs12151167	29840748	T	110.3	2.32E-20	chr:19:29855781
19	rs2903117	29847324	T	105.7	2.92E-20	chr:19:29855781
19	rs7254854	29847388	A	105.7	2.92E-20	chr:19:29855781
19	rs7256256	29847424	G	105.7	2.92E-20	chr:19:29855781
19	rs4805366	29799264	G	35.02	3.01E-20	chr:19:29855781
19	rs10413728	29833591	C	99.27	3.08E-20	chr:19:29855781
19	rs11883012	29874196	G	51.2	3.15E-20	chr:19:29855781
19	rs7255472	29811968	C	50.3	3.41E-20	chr:19:29855781
19	rs10500239	29829980	C	16.6	3.93E-20	chr:19:29855781
19	rs12608817	29808821	A	16.32	3.94E-20	chr:19:29855781
19	rs12610707	29808907	C	16.32	3.94E-20	chr:19:29855781
19	rs62108213	29830870	A	114.7	4.19E-20	chr:19:29855781
19	rs62108214	29830872	C	114.7	4.19E-20	chr:19:29855781
19	rs62108215	29830873	T	114.7	4.19E-20	chr:19:29855781
19	rs150191335	29843058	T	16.51	4.49E-20	chr:19:29855781

19	rs67987964	29845533	G	16.51	4.49E-20	chr:19:29855781
19	rs7508357	29841738	C	16.51	4.49E-20	chr:19:29855781
19	rs10413814	29859044	A	15.71	4.63E-20	chr:19:29855781
19	rs10413925	29851859	T	15.71	4.63E-20	chr:19:29855781
19	rs34235250	29852282	C	15.71	4.63E-20	chr:19:29855781
19	rs8103504	29808196	C	53.02	5.21E-20	chr:19:29855781
19	rs9797726	29842789	T	108	5.35E-20	chr:19:29855781
19	rs10422443	29860582	A	15.88	5.62E-20	chr:19:29855781
19	rs10424790	29860630	G	15.88	5.62E-20	chr:19:29855781
19	rs11880983	29859865	C	15.88	5.62E-20	chr:19:29855781
19	rs11882773	29859829	A	15.88	5.62E-20	chr:19:29855781
19	rs12709912	29859443	C	15.88	5.62E-20	chr:19:29855781
19	rs13344917	29859503	C	15.88	5.62E-20	chr:19:29855781
19	rs7253279	29864696	A	14.93	6.48E-20	chr:19:29855781
19	rs1558362	29865141	T	14.84	6.76E-20	chr:19:29855781
19	rs1558364	29865345	A	14.84	6.76E-20	chr:19:29855781
19	rs9916951	29865388	G	14.84	6.76E-20	chr:19:29855781
19	rs10407706	29799613	C	41.13	6.80E-20	chr:19:29855781
19	rs10407716	29799624	G	41.13	6.80E-20	chr:19:29855781
19	rs10406127	29826758	T	15.46	6.90E-20	chr:19:29855781
19	rs1122022	29825214	A	15.46	6.90E-20	chr:19:29855781
19	rs10408748	29837390	T	15.26	7.05E-20	chr:19:29855781
19	rs10410330	29827372	C	15.26	7.05E-20	chr:19:29855781
19	rs10419817	29828620	T	15.26	7.05E-20	chr:19:29855781
19	rs2012095	29801230	G	45.6	7.72E-20	chr:19:29855781
19	rs10411489	29846018	C	15.18	7.94E-20	chr:19:29855781
19	rs10424375	29839089	A	15.18	7.94E-20	chr:19:29855781
19	rs10402522	29861084	A	15.1	8.05E-20	chr:19:29855781
19	rs10403684	29860887	T	15.1	8.05E-20	chr:19:29855781
19	rs10404520	29860937	G	15.1	8.05E-20	chr:19:29855781
19	rs10404941	29861103	C	15.1	8.05E-20	chr:19:29855781
19	rs6509385	29861469	C	15.1	8.05E-20	chr:19:29855781
19	rs1558365	29865746	C	14.72	8.06E-20	chr:19:29855781
19	rs1558366	29865847	T	14.72	8.06E-20	chr:19:29855781
19	rs6509388	29861882	G	15.15	8.63E-20	chr:19:29855781
19	rs8100899	29808524	G	57.68	8.86E-20	chr:19:29855781
19	rs8107456	29810347	C	57.68	8.86E-20	chr:19:29855781
19	rs10412642	29871720	A	14.13	1.09E-19	chr:19:29855781
19	rs7258038	29844001	G	85.43	1.17E-19	chr:19:29855781
19	rs10421910	29868808	A	14.06	1.20E-19	chr:19:29855781
19	rs10422144	29868886	A	14.06	1.20E-19	chr:19:29855781
19	rs10422587	29869112	A	14.06	1.20E-19	chr:19:29855781
19	rs12973626	29867364	C	14.06	1.20E-19	chr:19:29855781

19	rs12979074	29867103	A	14.06	1.20E-19	chr:19:29855781
19	rs12979587	29867327	A	14.06	1.20E-19	chr:19:29855781
19	rs12980588	29867736	A	14.06	1.20E-19	chr:19:29855781
19	rs12981862	29867527	G	14.06	1.20E-19	chr:19:29855781
19	rs12981877	29867555	T	14.06	1.20E-19	chr:19:29855781
19	rs1981675	29868188	T	14.06	1.20E-19	chr:19:29855781
19	rs1981677	29868499	A	14.06	1.20E-19	chr:19:29855781
19	rs4271695	29866742	A	14.06	1.20E-19	chr:19:29855781
19	rs4398199	29866833	C	14.06	1.20E-19	chr:19:29855781
19	rs4558517	29866993	T	14.06	1.20E-19	chr:19:29855781
19	rs4605284	29866673	T	14.06	1.20E-19	chr:19:29855781
19	rs4605285	29866853	G	14.06	1.20E-19	chr:19:29855781
19	rs8103925	29866287	G	14.06	1.20E-19	chr:19:29855781
19	rs10408576	29818131	A	14.29	1.22E-19	chr:19:29855781
19	rs11879307	29815370	A	14.29	1.22E-19	chr:19:29855781
19	rs2193499	29821859	A	14.29	1.22E-19	chr:19:29855781
19	rs10416302	29851854	C	118.4	1.24E-19	chr:19:29855781
19	rs7249741	29849562	G	118.4	1.24E-19	chr:19:29855781
19	rs13343404	29826240	A	14.22	1.36E-19	chr:19:29855781
19	rs12978558	29807938	C	14.28	1.37E-19	chr:19:29855781
19	rs17624933	29867883	T	13.88	1.37E-19	chr:19:29855781
19	rs8100334	29866137	T	13.99	1.42E-19	chr:19:29855781
19	rs8105692	29840119	C	87.75	1.42E-19	chr:19:29855781
19	rs10419653	29812484	G	14.87	1.44E-19	chr:19:29855781
19	rs10425007	29812397	C	14.87	1.44E-19	chr:19:29855781
19	rs10425611	29813263	T	14.87	1.44E-19	chr:19:29855781
19	rs11880387	29810986	A	14.87	1.44E-19	chr:19:29855781
19	rs2216341	29812294	T	14.87	1.44E-19	chr:19:29855781
19	rs727798	29811828	C	14.87	1.44E-19	chr:19:29855781
19	rs34786514	29878333	G	13.85	1.67E-19	chr:19:29855781
19	rs2111497	29822857	C	85.88	1.68E-19	chr:19:29855781
19	rs2193498	29821753	G	85.88	1.68E-19	chr:19:29855781
19	rs2865872	29822103	C	85.88	1.68E-19	chr:19:29855781
19	rs2903113	29821703	T	85.88	1.68E-19	chr:19:29855781
19	rs8113496	29820529	G	85.88	1.68E-19	chr:19:29855781
19	rs7258498	29860387	T	14.37	1.76E-19	chr:19:29855781
19	rs10411884	29814875	T	13.94	2.08E-19	chr:19:29855781
19	rs8102048	29814240	A	13.94	2.08E-19	chr:19:29855781
19	rs8100333	29866136	T	13.13	2.24E-19	chr:19:29855781
19	rs28573811	29867469	T	58.09	2.40E-19	chr:19:29855781
19	rs2193500	29834892	G	13.87	2.43E-19	chr:19:29855781
19	rs6509386	29861649	T	13.69	2.54E-19	chr:19:29855781
19	rs6509387	29861707	A	13.69	2.54E-19	chr:19:29855781

19	rs7246620	29861360	C	13.69	2.54E-19	chr:19:29855781
19	rs10403689	29862797	A	13.68	2.60E-19	chr:19:29855781
19	rs10404170	29863044	C	13.68	2.60E-19	chr:19:29855781
19	rs10423850	29862444	A	13.68	2.60E-19	chr:19:29855781
19	rs11883246	29862074	G	13.68	2.60E-19	chr:19:29855781
19	rs56048673	29862967	A	13.68	2.60E-19	chr:19:29855781
19	rs6509389	29861958	G	13.68	2.60E-19	chr:19:29855781
19	rs6509390	29862034	T	13.68	2.60E-19	chr:19:29855781
19	rs7248809	29863240	G	13.68	2.60E-19	chr:19:29855781
19	rs7248884	29863662	T	13.68	2.60E-19	chr:19:29855781
19	rs7252555	29863630	C	13.68	2.60E-19	chr:19:29855781
19	rs7259512	29862045	G	13.68	2.60E-19	chr:19:29855781
19	rs7507344	29863087	A	13.68	2.60E-19	chr:19:29855781
19	rs7507798	29863083	C	13.68	2.60E-19	chr:19:29855781
19	rs10853799	29821146	A	84.9	2.80E-19	chr:19:29855781
19	rs7351109	29816574	C	65.27	2.81E-19	chr:19:29855781
19	rs13345888	29842781	C	13.96	2.87E-19	chr:19:29855781
19	rs8113733	29807306	C	22.01	3.20E-19	chr:19:29855781
19	rs12610720	29808999	C	14.06	3.47E-19	chr:19:29855781
19	rs2111519	29794684	T	20.05	3.76E-19	chr:19:29855781
19	rs2080613	29811982	A	13.38	3.90E-19	chr:19:29855781
19	rs4805382	29873748	A	18.91	4.46E-19	chr:19:29855781
19	rs2160717	29822729	T	13.1	4.52E-19	chr:19:29855781
19	rs10405922	29813264	C	13.08	5.29E-19	chr:19:29855781
19	rs10415631	29810562	C	13.08	5.29E-19	chr:19:29855781
19	rs10418201	29810677	G	13.08	5.29E-19	chr:19:29855781
19	rs10423930	29810687	G	13.08	5.29E-19	chr:19:29855781
19	rs10424492	29813431	A	13.08	5.29E-19	chr:19:29855781
19	rs11880160	29810859	A	13.08	5.29E-19	chr:19:29855781
19	rs12978201	29805534	G	13.08	5.29E-19	chr:19:29855781
19	rs17773647	29813079	G	13.08	5.29E-19	chr:19:29855781
19	rs28581734	29813167	T	13.08	5.29E-19	chr:19:29855781
19	rs8099873	29808683	A	13.08	5.29E-19	chr:19:29855781
19	rs8108111	29809851	G	13.08	5.29E-19	chr:19:29855781
19	rs8111179	29809968	C	13.08	5.29E-19	chr:19:29855781
19	rs8113604	29813727	G	13.08	5.29E-19	chr:19:29855781
19	rs7258057	29844039	G	134	5.93E-19	chr:19:29855781
19	rs7507405	29841956	C	134.8	6.14E-19	chr:19:29855781
19	rs10424081	29832472	A	139.3	6.57E-19	chr:19:29855781
19	rs2216339	29821569	G	123.3	7.14E-19	chr:19:29855781
19	rs5007513	29821553	A	123.3	7.14E-19	chr:19:29855781
19	rs10413763	29873400	A	12.68	7.23E-19	chr:19:29855781
19	rs10404772	29821253	A	134.1	7.58E-19	chr:19:29855781

19	rs10408183	29823239	T	134.1	7.58E-19	chr:19:29855781
19	rs10409090	29823614	G	134.1	7.58E-19	chr:19:29855781
19	rs10415948	29824022	G	134.1	7.58E-19	chr:19:29855781
19	rs10417724	29819252	A	134.1	7.58E-19	chr:19:29855781
19	rs10423259	29820105	A	134.1	7.58E-19	chr:19:29855781
19	rs10853797	29821094	C	134.1	7.58E-19	chr:19:29855781
19	rs11083917	29821301	C	134.1	7.58E-19	chr:19:29855781
19	rs2111496	29822748	A	134.1	7.58E-19	chr:19:29855781
19	rs2111498	29822858	A	134.1	7.58E-19	chr:19:29855781
19	rs2160716	29822600	C	134.1	7.58E-19	chr:19:29855781
19	rs2193497	29821737	A	134.1	7.58E-19	chr:19:29855781
19	rs2216340	29823183	G	134.1	7.58E-19	chr:19:29855781
19	rs28488633	29823953	C	134.1	7.58E-19	chr:19:29855781
19	rs28546871	29821491	C	134.1	7.58E-19	chr:19:29855781
19	rs2865869	29821979	T	134.1	7.58E-19	chr:19:29855781
19	rs2865870	29821980	C	134.1	7.58E-19	chr:19:29855781
19	rs2865871	29822061	T	134.1	7.58E-19	chr:19:29855781
19	rs8102393	29820560	C	134.1	7.58E-19	chr:19:29855781
19	rs8107378	29823430	C	134.1	7.58E-19	chr:19:29855781
19	rs8113732	29820742	G	134.1	7.58E-19	chr:19:29855781
19	rs979412	29818376	A	134.1	7.58E-19	chr:19:29855781
19	rs10404259	29840188	C	139.3	7.71E-19	chr:19:29855781
19	rs35379433	29834698	A	129.8	8.37E-19	chr:19:29855781
19	rs1012613	29796344	A	13.19	9.18E-19	chr:19:29855781
19	rs4804878	29873579	A	17.23	9.22E-19	chr:19:29855781
19	rs72623834	29842510	A	128.1	1.10E-18	chr:19:29855781
19	rs979413	29818635	A	132.6	1.10E-18	chr:19:29855781
19	rs28857523	29844100	G	134.4	1.12E-18	chr:19:29855781
19	rs10404828	29826537	T	99.61	1.16E-18	chr:19:29855781
19	rs10411272	29826524	A	99.61	1.16E-18	chr:19:29855781
19	rs2865878	29825358	C	99.61	1.16E-18	chr:19:29855781
19	rs8111162	29824369	C	99.61	1.16E-18	chr:19:29855781
19	rs4805383	29873963	G	19.85	1.22E-18	chr:19:29855781
19	rs10853800	29821178	C	133	1.26E-18	chr:19:29855781
19	rs11083916	29821200	C	133	1.26E-18	chr:19:29855781
19	rs10421823	29835487	A	109.5	1.34E-18	chr:19:29855781
19	rs8103862	29830747	A	109.5	1.34E-18	chr:19:29855781
19	rs8109855	29828233	T	109.5	1.34E-18	chr:19:29855781
19	rs74838486	29842509	A	109.9	1.35E-18	chr:19:29855781
19	rs10420966	29816663	T	76.9	1.41E-18	chr:19:29855781
19	rs1558864	29823261	A	76.9	1.41E-18	chr:19:29855781
19	rs7253662	29815722	T	76.9	1.41E-18	chr:19:29855781
19	rs929691	29818042	C	76.9	1.41E-18	chr:19:29855781

19	rs10407026	29848625	C	108.6	1.45E-18	chr:19:29855781
19	rs10420835	29856760	T	108.6	1.45E-18	chr:19:29855781
19	rs13344183	29841038	T	108.6	1.45E-18	chr:19:29855781
19	rs144395360	29843445	T	108.6	1.45E-18	chr:19:29855781
19	rs2159564	29851751	A	108.6	1.45E-18	chr:19:29855781
19	rs2159565	29847643	A	108.6	1.45E-18	chr:19:29855781
19	rs4804875	29853497	G	108.6	1.45E-18	chr:19:29855781
19	rs4805373	29853498	C	108.6	1.45E-18	chr:19:29855781
19	rs7251831	29849926	A	108.6	1.45E-18	chr:19:29855781
19	rs7252072	29855010	G	108.6	1.45E-18	chr:19:29855781
19	rs8101272	29840005	C	108.6	1.45E-18	chr:19:29855781
19	rs8112567	29838763	G	108.6	1.45E-18	chr:19:29855781
19	rs9749055	29845247	G	108.6	1.45E-18	chr:19:29855781
19	rs2865883	29837946	T	108.6	1.47E-18	chr:19:29855781
19	rs28870995	29844110	C	134.6	1.64E-18	chr:19:29855781
19	rs7257439	29816242	A	79.32	1.66E-18	chr:19:29855781
19	rs62104320	29866335	T	70.05	1.71E-18	chr:19:29855781
19	rs10404884	29839279	A	24.85	1.87E-18	chr:19:29855781
19	rs10407922	29833571	C	109.5	2.17E-18	chr:19:29855781
19	rs1981678	29868519	G	67.24	2.72E-18	chr:19:29855781
19	rs8107989	29867796	G	67.24	2.72E-18	chr:19:29855781
19	rs1860573	29879749	A	10.45	3.34E-18	chr:19:29855781
19	rs10426484	29866090	A	11.63	4.95E-18	chr:19:29855781
19	rs4805381	29872627	C	102.9	6.36E-18	chr:19:29855781
19	rs10402726	29826292	T	159.7	7.75E-18	chr:19:29855781
19	rs10403475	29826676	C	159.7	7.75E-18	chr:19:29855781
19	rs10405159	29826311	G	159.7	7.75E-18	chr:19:29855781
19	rs10410622	29826229	G	159.7	7.75E-18	chr:19:29855781
19	rs10411073	29826427	C	159.7	7.75E-18	chr:19:29855781
19	rs13345551	29826197	G	159.7	7.75E-18	chr:19:29855781
19	rs13346790	29825511	A	159.7	7.75E-18	chr:19:29855781
19	rs2865874	29825020	A	159.7	7.75E-18	chr:19:29855781
19	rs2865875	29825068	A	159.7	7.75E-18	chr:19:29855781
19	rs2865876	29825093	A	159.7	7.75E-18	chr:19:29855781
19	rs2865877	29825180	C	159.7	7.75E-18	chr:19:29855781
19	rs8107784	29824307	T	159.7	7.75E-18	chr:19:29855781
19	rs12691091	29859430	C	97.92	7.76E-18	chr:19:29855781
19	rs3960886	29878149	C	26.99	8.79E-18	chr:19:29855781
19	rs6509401	29877643	C	26.99	8.79E-18	chr:19:29855781
19	rs8109600	29877328	G	26.99	8.79E-18	chr:19:29855781
19	rs2903115	29837927	T	97.21	9.54E-18	chr:19:29855781
19	rs35308361	29830552	T	97.21	9.54E-18	chr:19:29855781
19	rs10404303	29860663	C	96.36	1.02E-17	chr:19:29855781

19	rs10421211	29852874	A	96.36	1.02E-17	chr:19:29855781
19	rs1990391	29859164	T	96.36	1.02E-17	chr:19:29855781
19	rs4804876	29853605	A	96.36	1.02E-17	chr:19:29855781
19	rs61631634	29852485	C	96.36	1.02E-17	chr:19:29855781
19	rs6509383	29857637	C	96.36	1.02E-17	chr:19:29855781
19	rs7248644	29856939	C	96.36	1.02E-17	chr:19:29855781
19	rs7258342	29859947	C	96.36	1.02E-17	chr:19:29855781
19	rs8107741	29858602	G	96.36	1.02E-17	chr:19:29855781
19	rs10416173	29824125	T	152.9	1.09E-17	chr:19:29855781
19	rs10401849	29860785	T	91.39	1.32E-17	chr:19:29855781
19	rs10401431	29829429	G	183.3	1.41E-17	chr:19:29855781
19	rs10405212	29832150	G	183.3	1.41E-17	chr:19:29855781
19	rs10407240	29829500	C	183.3	1.41E-17	chr:19:29855781
19	rs10409875	29827166	A	183.3	1.41E-17	chr:19:29855781
19	rs10409965	29837251	T	183.3	1.41E-17	chr:19:29855781
19	rs10410499	29827426	T	183.3	1.41E-17	chr:19:29855781
19	rs10410585	29837528	A	183.3	1.41E-17	chr:19:29855781
19	rs10416004	29831681	T	183.3	1.41E-17	chr:19:29855781
19	rs10416725	29831649	C	183.3	1.41E-17	chr:19:29855781
19	rs10419949	29828341	G	183.3	1.41E-17	chr:19:29855781
19	rs10422084	29835604	G	183.3	1.41E-17	chr:19:29855781
19	rs10422614	29835503	G	183.3	1.41E-17	chr:19:29855781
19	rs10425546	29832398	T	183.3	1.41E-17	chr:19:29855781
19	rs10425993	29832603	G	183.3	1.41E-17	chr:19:29855781
19	rs11881314	29830193	A	183.3	1.41E-17	chr:19:29855781
19	rs11881407	29829948	T	183.3	1.41E-17	chr:19:29855781
19	rs11881459	29830092	T	183.3	1.41E-17	chr:19:29855781
19	rs11881508	29830309	G	183.3	1.41E-17	chr:19:29855781
19	rs12610959	29827626	C	183.3	1.41E-17	chr:19:29855781
19	rs17773922	29830629	C	183.3	1.41E-17	chr:19:29855781
19	rs2865884	29837991	C	183.3	1.41E-17	chr:19:29855781
19	rs35349289	29830485	G	183.3	1.41E-17	chr:19:29855781
19	rs35615835	29827061	A	183.3	1.41E-17	chr:19:29855781
19	rs62108163	29828752	T	183.3	1.41E-17	chr:19:29855781
19	rs62108164	29828755	C	183.3	1.41E-17	chr:19:29855781
19	rs8103034	29830764	A	183.3	1.41E-17	chr:19:29855781
19	rs8106354	29831100	A	183.3	1.41E-17	chr:19:29855781
19	rs35270224	29849348	G	182.1	1.44E-17	chr:19:29855781
19	rs10405177	29840592	C	181.7	1.53E-17	chr:19:29855781
19	rs10405226	29840658	A	181.7	1.53E-17	chr:19:29855781
19	rs10405460	29840781	A	181.7	1.53E-17	chr:19:29855781
19	rs10405550	29839527	C	181.7	1.53E-17	chr:19:29855781
19	rs10406707	29840277	C	181.7	1.53E-17	chr:19:29855781

19	rs10406756	29840591	T	181.7	1.53E-17	chr:19:29855781
19	rs10410829	29840787	T	181.7	1.53E-17	chr:19:29855781
19	rs10412879	29848889	A	181.7	1.53E-17	chr:19:29855781
19	rs10423486	29839393	A	181.7	1.53E-17	chr:19:29855781
19	rs10423515	29853162	T	181.7	1.53E-17	chr:19:29855781
19	rs10423608	29852639	G	181.7	1.53E-17	chr:19:29855781
19	rs10423847	29852955	T	181.7	1.53E-17	chr:19:29855781
19	rs10423994	29839675	A	181.7	1.53E-17	chr:19:29855781
19	rs10424058	29853036	G	181.7	1.53E-17	chr:19:29855781
19	rs10424145	29839694	T	181.7	1.53E-17	chr:19:29855781
19	rs10424499	29848304	T	181.7	1.53E-17	chr:19:29855781
19	rs10425325	29839190	C	181.7	1.53E-17	chr:19:29855781
19	rs10425388	29848770	T	181.7	1.53E-17	chr:19:29855781
19	rs10425705	29839737	T	181.7	1.53E-17	chr:19:29855781
19	rs10426238	29848399	A	181.7	1.53E-17	chr:19:29855781
19	rs111393122	29839832	C	181.7	1.53E-17	chr:19:29855781
19	rs111486428	29842638	T	181.7	1.53E-17	chr:19:29855781
19	rs1119168	29850635	C	181.7	1.53E-17	chr:19:29855781
19	rs113093811	29852096	A	181.7	1.53E-17	chr:19:29855781
19	rs140915866	29843043	G	181.7	1.53E-17	chr:19:29855781
19	rs147519792	29843490	G	181.7	1.53E-17	chr:19:29855781
19	rs148091885	29842847	A	181.7	1.53E-17	chr:19:29855781
19	rs2159563	29851209	C	181.7	1.53E-17	chr:19:29855781
19	rs2865888	29845818	C	181.7	1.53E-17	chr:19:29855781
19	rs2865889	29847080	A	181.7	1.53E-17	chr:19:29855781
19	rs2865890	29847162	T	181.7	1.53E-17	chr:19:29855781
19	rs2903118	29853727	C	181.7	1.53E-17	chr:19:29855781
19	rs35754631	29852145	C	181.7	1.53E-17	chr:19:29855781
19	rs4517901	29850859	G	181.7	1.53E-17	chr:19:29855781
19	rs4517902	29851078	C	181.7	1.53E-17	chr:19:29855781
19	rs4517903	29851162	C	181.7	1.53E-17	chr:19:29855781
19	rs4804877	29853608	A	181.7	1.53E-17	chr:19:29855781
19	rs4805371	29853296	T	181.7	1.53E-17	chr:19:29855781
19	rs4805372	29853415	C	181.7	1.53E-17	chr:19:29855781
19	rs62109915	29843088	T	181.7	1.53E-17	chr:19:29855781
19	rs62109922	29852078	T	181.7	1.53E-17	chr:19:29855781
19	rs713444	29847933	C	181.7	1.53E-17	chr:19:29855781
19	rs7249621	29849475	G	181.7	1.53E-17	chr:19:29855781
19	rs7249732	29849544	G	181.7	1.53E-17	chr:19:29855781
19	rs7252876	29851938	A	181.7	1.53E-17	chr:19:29855781
19	rs7253264	29849954	G	181.7	1.53E-17	chr:19:29855781
19	rs7253600	29850196	C	181.7	1.53E-17	chr:19:29855781
19	rs74181477	29840703	A	181.7	1.53E-17	chr:19:29855781

19	rs7507511	29842044	T	181.7	1.53E-17	chr:19:29855781
19	rs7507537	29841906	G	181.7	1.53E-17	chr:19:29855781
19	rs7507869	29841938	C	181.7	1.53E-17	chr:19:29855781
19	rs8105295	29846290	G	181.7	1.53E-17	chr:19:29855781
19	rs8105878	29846471	G	181.7	1.53E-17	chr:19:29855781
19	rs8106019	29846532	T	181.7	1.53E-17	chr:19:29855781
19	rs8108489	29846043	C	181.7	1.53E-17	chr:19:29855781
19	rs8108803	29846308	G	181.7	1.53E-17	chr:19:29855781
19	rs8108995	29838275	G	181.7	1.53E-17	chr:19:29855781
19	rs8109140	29846602	C	181.7	1.53E-17	chr:19:29855781
19	rs8109315	29838529	C	181.7	1.53E-17	chr:19:29855781
19	rs8112403	29838791	G	181.7	1.53E-17	chr:19:29855781
19	rs9748962	29844809	A	181.7	1.53E-17	chr:19:29855781
19	rs4805374	29862430	G	87.16	1.56E-17	chr:19:29855781
19	rs7249123	29870685	G	87.16	1.56E-17	chr:19:29855781
19	rs7252679	29863745	C	87.16	1.56E-17	chr:19:29855781
19	rs8107249	29834648	G	181.2	1.62E-17	chr:19:29855781
19	rs11670844	29800673	T	11.45	1.63E-17	chr:19:29855781
19	rs10415713	29831541	T	175	1.85E-17	chr:19:29855781
19	rs10405069	29833324	A	185.8	2.12E-17	chr:19:29855781
19	rs10405105	29833373	T	185.8	2.12E-17	chr:19:29855781
19	rs10406530	29832689	C	185.8	2.12E-17	chr:19:29855781
19	rs10406567	29832755	C	185.8	2.12E-17	chr:19:29855781
19	rs10406849	29833391	A	185.8	2.12E-17	chr:19:29855781
19	rs10407234	29833261	C	185.8	2.12E-17	chr:19:29855781
19	rs10413525	29833498	C	185.8	2.12E-17	chr:19:29855781
19	rs10405131	29845482	G	179.2	2.14E-17	chr:19:29855781
19	rs12150922	29879068	T	9.9	2.24E-17	chr:19:29855781
19	rs7259050	29799035	A	10.36	3.50E-17	chr:19:29855781
19	rs4591272	29841167	A	10.06	4.19E-17	chr:19:29855781
19	rs2011890	29801011	A	10.91	4.27E-17	chr:19:29855781
19	rs10424195	29883577	T	8.968	1.18E-16	chr:19:29855781
19	rs35980661	29798605	G	9.312	1.20E-16	chr:19:29855781
19	rs2865896	29872800	T	136.6	1.27E-16	chr:19:29855781
19	rs7253602	29864638	G	136.6	1.27E-16	chr:19:29855781
19	rs8107043	29864213	G	136.6	1.27E-16	chr:19:29855781
19	rs957166	29882508	T	8.399	2.58E-16	chr:19:29855781
19	rs10416313	29831461	G	287.6	5.83E-16	chr:19:29855781
19	rs10422053	29881070	T	8.187	7.93E-16	chr:19:29855781
19	rs13346371	29880687	G	8.187	7.93E-16	chr:19:29855781
19	rs17703317	29880615	A	8.187	7.93E-16	chr:19:29855781
19	rs55711025	29880437	G	8.187	7.93E-16	chr:19:29855781
19	rs9748993	29881310	C	8.187	7.93E-16	chr:19:29855781

19	rs111164000	29881600	C	7.378	1.20E-15	chr:19:29855781
19	rs56091016	29880529	C	7.378	1.20E-15	chr:19:29855781
19	rs17703299	29880277	A	7.353	1.45E-15	chr:19:29855781
19	rs8107864	29882147	T	7.549	1.48E-15	chr:19:29855781
19	rs28792508	29841216	C	8.747	1.95E-15	chr:19:29855781
19	rs56097453	29881537	A	7.215	2.14E-15	chr:19:29855781
19	rs13344861	29881433	A	7.211	2.68E-15	chr:19:29855781
19	rs2111518	29794622	C	6.909	3.30E-15	chr:19:29855781
19	rs10420190	29873997	A	6.977	3.59E-15	chr:19:29855781
19	rs957165	29882525	G	6.635	5.43E-15	chr:19:29855781
19	rs1076416	29879196	A	6.333	1.61E-14	chr:19:29855781
19	rs13344206	29880707	A	6.007	2.61E-14	chr:19:29855781
19	rs55645533	29880453	T	5.83	3.87E-14	chr:19:29855781
19	rs142004081	29841268	A	7.672	4.52E-14	chr:19:29855781
19	rs150522295	29841311	G	6.165	8.48E-13	chr:19:29855781
19	rs200964938	29841291	G	6.567	2.28E-12	chr:19:29855781
19	rs62111119	29795071	T	6.098	4.53E-12	chr:19:29855781
19	rs8103720	29800499	C	0.2124	5.62E-12	chr:19:29855781
19	rs10425210	29792094	A	5.862	1.05E-11	chr:19:29855781
19	rs10408404	29739729	T	4.063	2.32E-10	chr:19:29855781
19	rs7253429	29748848	T	4.065	2.71E-10	chr:19:29855781
19	rs4805357	29742669	A	3.977	3.47E-10	chr:19:29855781
19	rs741449	29738641	C	3.88	5.62E-10	chr:19:29855781
19	rs36032197	29813521	T	0.2593	5.70E-10	chr:19:29855781
19	rs71363971	29813517	C	0.2593	5.70E-10	chr:19:29855781
19	rs8101728	29883875	A	3.562	8.75E-10	chr:19:29855781
19	rs11671699	29882003	A	4.514	9.18E-10	chr:19:29855781
19	rs10401191	29792311	C	0.2834	2.18E-09	chr:19:29855781
19	rs17773425	29793493	A	10.82	4.34E-09	chr:19:29855781
19	rs75806877	29797051	A	10.82	4.34E-09	chr:19:29855781
19	rs12460892	29764708	T	3.159	6.07E-09	chr:19:29855781
19	rs10408136	29762996	C	3.149	7.43E-09	chr:19:29855781
19	rs12460869	29764587	T	3.149	7.43E-09	chr:19:29855781
19	rs888301	29761748	T	3.149	7.43E-09	chr:19:29855781
19	rs10425413	29752395	T	3.821	1.11E-08	chr:19:29855781
19	rs4805362	29785242	C	2.958	3.64E-08	chr:19:29855781
19	rs4805365	29786535	G	2.958	3.64E-08	chr:19:29855781
19	rs9789269	29784311	G	2.905	5.57E-08	chr:19:29855781
19	rs11083885	29767726	G	2.898	5.65E-08	chr:19:29855781
19	rs7254145	29851810	G	15.33	5.99E-08	chr:19:29855781
19	rs112915449	29743176	T	2.878	6.29E-08	chr:19:29855781
19	rs11879318	29745578	T	2.878	6.29E-08	chr:19:29855781
19	rs7246203	29748022	A	2.878	6.29E-08	chr:19:29855781

19	rs10407731	29769563	T	2.854	8.41E-08	chr:19:29855781
19	rs62109951	29854071	T	8.896	1.05E-07	chr:19:29855781
19	rs8182564	29873348	A	13.97	1.59E-07	chr:19:29855781
19	rs17624382	29788630	G	2.91	1.69E-07	chr:19:29855781
19	rs76154346	29894876	A	8.433	1.70E-07	chr:19:29855781
19	rs10404352	29787431	A	2.944	1.75E-07	chr:19:29855781
19	rs10409564	29787207	T	2.944	1.75E-07	chr:19:29855781
19	rs10423387	29788813	T	2.944	1.75E-07	chr:19:29855781
19	rs12981362	29787976	C	2.944	1.75E-07	chr:19:29855781
19	rs77238668	29865051	T	14.07	1.91E-07	chr:19:29855781
19	rs4805384	29874653	A	11.29	2.33E-07	chr:19:29855781
19	rs16962782	29864581	T	7.035	2.44E-07	chr:19:29855781
19	rs73925551	29864616	A	7.035	2.44E-07	chr:19:29855781
19	rs76396068	29864627	G	7.035	2.44E-07	chr:19:29855781
19	rs79014341	29864990	C	7.035	2.44E-07	chr:19:29855781
19	rs79187580	29864921	A	7.035	2.44E-07	chr:19:29855781
19	rs8107191	29864362	G	7.035	2.44E-07	chr:19:29855781
19	rs10406799	29783563	T	2.923	2.46E-07	chr:19:29855781
19	rs73024995	29822198	G	0.1985	2.92E-07	chr:19:29855781
19	rs12608692	29744597	C	0.387	3.64E-07	chr:19:29855781
19	rs17773524	29809488	G	12.88	4.71E-07	chr:19:29855781
19	rs117512512	29825745	A	12.75	5.19E-07	chr:19:29855781
19	rs138998402	29842631	C	12.75	5.19E-07	chr:19:29855781
19	rs139240069	29839857	A	12.75	5.19E-07	chr:19:29855781
19	rs149465522	29840076	T	12.75	5.19E-07	chr:19:29855781
19	rs17717108	29827335	T	12.75	5.19E-07	chr:19:29855781
19	rs190579845	29844147	A	12.75	5.19E-07	chr:19:29855781
19	rs4805375	29863126	T	12.75	5.19E-07	chr:19:29855781
19	rs4805378	29871813	T	12.75	5.19E-07	chr:19:29855781
19	rs4805379	29871922	T	12.75	5.19E-07	chr:19:29855781
19	rs4805380	29872523	A	12.75	5.19E-07	chr:19:29855781
19	rs57982463	29829642	G	12.75	5.19E-07	chr:19:29855781
19	rs740877	29869744	A	12.75	5.19E-07	chr:19:29855781
19	rs740878	29869768	T	12.75	5.19E-07	chr:19:29855781
19	rs740879	29869987	T	12.75	5.19E-07	chr:19:29855781
19	rs74453232	29833126	T	12.75	5.19E-07	chr:19:29855781
19	rs74578189	29836809	C	12.75	5.19E-07	chr:19:29855781
19	rs74647649	29868729	T	12.75	5.19E-07	chr:19:29855781
19	rs75224979	29864019	T	12.75	5.19E-07	chr:19:29855781
19	rs75530548	29856937	T	12.75	5.19E-07	chr:19:29855781
19	rs76278131	29867556	C	12.75	5.19E-07	chr:19:29855781
19	rs76807138	29868440	A	12.75	5.19E-07	chr:19:29855781
19	rs76963036	29833845	C	12.75	5.19E-07	chr:19:29855781

19	rs77337188	29869175	T	12.75	5.19E-07	chr:19:29855781
19	rs77430465	29824472	G	12.75	5.19E-07	chr:19:29855781
19	rs79470954	29854408	C	12.75	5.19E-07	chr:19:29855781
19	rs79857271	29865827	A	12.75	5.19E-07	chr:19:29855781
19	rs8182542	29873087	A	12.75	5.19E-07	chr:19:29855781
19	rs11881661	29858638	T	5.498	5.80E-07	chr:19:29855781
19	rs17716667	29789974	T	10.85	6.22E-07	chr:19:29855781
19	rs11083910	29814201	T	12.59	6.32E-07	chr:19:29855781
19	rs116957117	29805109	T	12.59	6.32E-07	chr:19:29855781
19	rs151173630	29805108	G	12.59	6.32E-07	chr:19:29855781
19	rs17773499	29807131	T	12.59	6.32E-07	chr:19:29855781
19	rs17773551	29810625	C	12.59	6.32E-07	chr:19:29855781
19	rs4804873	29802033	C	12.59	6.32E-07	chr:19:29855781
19	rs4804874	29802246	A	12.59	6.32E-07	chr:19:29855781
19	rs4805367	29808147	T	12.59	6.32E-07	chr:19:29855781
19	rs74575010	29809332	A	12.59	6.32E-07	chr:19:29855781
19	rs75040595	29806368	A	12.59	6.32E-07	chr:19:29855781
19	rs75553465	29823919	A	12.59	6.32E-07	chr:19:29855781
19	rs79702327	29820070	C	12.59	6.32E-07	chr:19:29855781
19	rs7253694	29858529	C	6.492	7.33E-07	chr:19:29855781
19	rs8106978	29864521	T	6.492	7.33E-07	chr:19:29855781
19	rs79988957	29818879	A	10.28	7.88E-07	chr:19:29855781
19	rs79137305	29784246	T	5.012	8.46E-07	chr:19:29855781
19	rs4805385	29875032	A	10.28	8.49E-07	chr:19:29855781
19	rs74792053	29877463	C	10.28	8.49E-07	chr:19:29855781
19	rs75131508	29877447	T	10.28	8.49E-07	chr:19:29855781
19	rs78214929	29876346	T	10.28	8.49E-07	chr:19:29855781
19	rs78258505	29877345	C	10.28	8.49E-07	chr:19:29855781
19	rs12984061	29885279	A	0.3559	9.31E-07	chr:19:29855781
19	rs4805387	29885553	C	0.3559	9.31E-07	chr:19:29855781
19	rs4805389	29885643	C	0.3559	9.31E-07	chr:19:29855781
19	rs4805390	29885676	G	0.3559	9.31E-07	chr:19:29855781
19	rs4805391	29885783	C	0.3559	9.31E-07	chr:19:29855781
19	rs75461522	29798875	A	10.09	9.33E-07	chr:19:29855781
19	rs186502397	29806312	G	12.04	1.02E-06	chr:19:29855781
19	rs73028769	29870748	C	0.2193	1.03E-06	chr:19:29855781
19	rs17716836	29812000	C	11.93	1.07E-06	chr:19:29855781
19	rs569196726	29881464	A	4.827	1.11E-06	chr:19:29855781
19	rs79231989	29790947	C	10.04	1.13E-06	chr:19:29855781
19	rs8102600	29793077	G	0.2302	2.48E-06	chr:19:29855781
19	rs148037882	29717889	T	3.66	3.05E-06	chr:19:29855781
19	rs76738117	29717976	T	3.66	3.05E-06	chr:19:29855781
19	rs11671788	29937565	G	2.338	3.51E-06	chr:19:29855781

19	rs59817498	29720661	T	3.518	5.40E-06	chr:19:29855781
19	rs8108536	29837645	T	0.3047	7.03E-06	chr:19:29855781
19	rs11670999	29730014	A	2.259	7.24E-06	chr:19:29855781
19	rs12972635	29897019	C	0.3834	7.38E-06	chr:19:29855781
19	rs11669309	29892297	T	0.3889	7.81E-06	chr:19:29855781
19	rs62109920	29846656	G	5.971	8.45E-06	chr:19:29855781
19	rs17717398	29902465	A	0.3902	8.80E-06	chr:19:29855781
19	rs111353402	29872155	A	0.3883	9.22E-06	chr:19:29855781
19	rs73027098	29850863	C	0.3883	9.22E-06	chr:19:29855781
19	rs73028737	29864112	T	0.3883	9.22E-06	chr:19:29855781
19	rs12460899	29899675	A	0.3868	9.53E-06	chr:19:29855781
19	rs36060857	29898561	A	0.3868	9.53E-06	chr:19:29855781
19	rs73014561	29899008	A	0.3868	9.53E-06	chr:19:29855781
19	rs1558880	29754331	C	2.384	9.98E-06	chr:19:29855781
19	rs11666114	29690571	T	3.364	1.08E-05	chr:19:29855781
19	rs34894094	29894875	T	0.3973	1.22E-05	chr:19:29855781
19	rs759632	29780738	T	2.168	1.33E-05	chr:19:29855781
19	rs73024933	29806342	T	0.4016	1.42E-05	chr:19:29855781
19	rs11083894	29784467	C	2.154	1.64E-05	chr:19:29855781
19	rs11083896	29784489	C	2.154	1.64E-05	chr:19:29855781
19	rs4805364	29786472	G	2.154	1.64E-05	chr:19:29855781
19	rs9789270	29784331	G	2.154	1.64E-05	chr:19:29855781
19	rs9789277	29784391	A	2.154	1.64E-05	chr:19:29855781
19	rs10426577	29731860	T	2.182	1.65E-05	chr:19:29855781
19	rs17691420	29730876	C	2.182	1.65E-05	chr:19:29855781
19	rs11083881	29760043	A	2.302	1.72E-05	chr:19:29855781
19	rs11880619	29759803	A	2.302	1.72E-05	chr:19:29855781
19	rs2193516	29757697	G	2.302	1.72E-05	chr:19:29855781
19	rs17691755	29793376	G	0.3222	1.84E-05	chr:19:29855781
19	rs73024931	29805085	A	0.4101	2.04E-05	chr:19:29855781
19	rs741450	29805084	A	0.4101	2.04E-05	chr:19:29855781
19	rs11667078	29610994	A	2.609	2.22E-05	chr:19:29855781
19	rs10421985	29771310	T	2.125	2.24E-05	chr:19:29855781
19	rs10422218	29771407	T	2.125	2.24E-05	chr:19:29855781
19	rs12462410	29771524	C	2.125	2.24E-05	chr:19:29855781
19	rs2016528	29770679	A	2.125	2.24E-05	chr:19:29855781
19	rs7408479	29784673	A	2.11	2.33E-05	chr:19:29855781
19	rs6509333	29776409	T	2.118	2.36E-05	chr:19:29855781
19	rs759629	29760667	T	2.27	2.66E-05	chr:19:29855781
19	rs759630	29760672	T	2.27	2.66E-05	chr:19:29855781
19	rs1014693	29775562	C	2.104	2.92E-05	chr:19:29855781
19	rs10405196	29774956	C	2.104	2.92E-05	chr:19:29855781
19	rs10406223	29775025	A	2.104	2.92E-05	chr:19:29855781

19	rs10426189	29778171	G	2.104	2.92E-05	chr:19:29855781
19	rs12983202	29773877	A	2.104	2.92E-05	chr:19:29855781
19	rs17691536	29773976	G	2.104	2.92E-05	chr:19:29855781
19	rs4343393	29781692	C	2.104	2.92E-05	chr:19:29855781
19	rs4524053	29781699	C	2.104	2.92E-05	chr:19:29855781
19	rs7249738	29779653	G	2.104	2.92E-05	chr:19:29855781
19	rs7252907	29776619	C	2.104	2.92E-05	chr:19:29855781
19	rs7260650	29777327	T	2.104	2.92E-05	chr:19:29855781
19	rs759631	29780521	C	2.104	2.92E-05	chr:19:29855781
19	rs888304	29781352	T	2.104	2.92E-05	chr:19:29855781
19	rs888305	29781623	G	2.104	2.92E-05	chr:19:29855781
19	rs888306	29781632	C	2.104	2.92E-05	chr:19:29855781
19	rs888307	29781673	G	2.104	2.92E-05	chr:19:29855781
19	rs2011889	29800978	T	5.838	2.96E-05	chr:19:29855781
19	rs8104456	29772205	C	2.093	3.19E-05	chr:19:29855781
19	rs546946831	389293	G	13.19	7.10E-07	chr:19:386675
19	rs571529934	389295	C	13.19	7.10E-07	chr:19:386675
19	rs111857853	387614	G	8.9	7.93E-07	chr:19:386675
19	rs73916946	387864	T	8.318	1.12E-06	chr:19:386675
19	rs9710605	398547	T	16.41	1.45E-06	chr:19:386675
19	rs374432840	387197	T	9.859	1.60E-06	chr:19:386675
19	rs369380681	388030	C	7.768	1.84E-06	chr:19:386675
19	rs529560773	388349	T	11.74	7.89E-06	chr:19:386675
19	rs549371497	388347	T	11.74	7.89E-06	chr:19:386675
19	rs562006336	388348	G	11.74	7.89E-06	chr:19:386675
19	rs115964175	405159	T	9.174	1.11E-05	chr:19:386675
19	rs731335	402491	A	9.012	1.12E-05	chr:19:386675
19	rs75887790	402953	A	9.012	1.12E-05	chr:19:386675
19	rs78950283	403202	A	9.012	1.12E-05	chr:19:386675
19	rs74564490	403591	A	8.649	1.37E-05	chr:19:386675
19	rs55749078	387847	C	6.551	1.64E-05	chr:19:386675
19	rs111688272	388012	G	7.07	4.15E-05	chr:19:386675
19	rs75787341	370630	A	12.6	5.08E-05	chr:19:386675
1	rs10857872	111838382	A	7.916	5.62E-16	chr:1:111802591
1	rs11102240	111844621	G	7.916	5.62E-16	chr:1:111802591
1	rs7411387	111837135	C	7.916	5.62E-16	chr:1:111802591
1	rs4259643	111814404	G	0.02528	6.03E-14	chr:1:111802591
1	rs11102233	111827749	T	6.215	9.71E-14	chr:1:111802591
1	rs11102229	111808446	G	0.1691	1.29E-13	chr:1:111802591
1	rs34487304	111804011	A	0.1691	1.29E-13	chr:1:111802591
1	rs3891601	111805489	G	0.1691	1.29E-13	chr:1:111802591
1	rs59772828	111808783	A	0.1691	1.29E-13	chr:1:111802591
1	rs2820057	111811175	A	0.01716	1.74E-13	chr:1:111802591

1	rs2820058	111811160	T	0.01716	1.74E-13	chr:1:111802591
1	rs3118208	111812709	G	0.01716	1.74E-13	chr:1:111802591
1	rs3118209	111812196	C	0.01716	1.74E-13	chr:1:111802591
1	rs4240528	111802958	C	0.1725	1.99E-13	chr:1:111802591
1	rs1575722	111803894	C	7.128	2.01E-13	chr:1:111802591
1	rs2477577	111809238	C	7.128	2.01E-13	chr:1:111802591
1	rs2764550	111808478	A	7.128	2.01E-13	chr:1:111802591
1	rs2820060	111807851	C	7.128	2.01E-13	chr:1:111802591
1	rs2494011	111806553	G	7.187	2.15E-13	chr:1:111802591
1	rs11588487	111812241	T	0.1868	6.19E-13	chr:1:111802591
1	rs12733155	111812412	T	0.1868	6.19E-13	chr:1:111802591
1	rs12724976	111813174	C	0.1941	1.20E-12	chr:1:111802591
1	rs2104408	111810753	C	95.01	1.23E-12	chr:1:111802591
1	rs12135216	111809857	G	0.2122	3.41E-12	chr:1:111802591
1	rs58982671	111826007	T	0.1951	3.73E-12	chr:1:111802591
1	rs11102232	111818450	C	0.1997	4.50E-12	chr:1:111802591
1	rs11589353	111822680	G	0.1997	4.50E-12	chr:1:111802591
1	rs11589375	111822887	G	0.1997	4.50E-12	chr:1:111802591
1	rs12411206	111822962	C	0.1997	4.50E-12	chr:1:111802591
1	rs12567865	111817250	A	0.1997	4.50E-12	chr:1:111802591
1	rs12758301	111822132	C	0.1997	4.50E-12	chr:1:111802591
1	rs35693821	111816294	G	0.1997	4.50E-12	chr:1:111802591
1	rs35884994	111816144	C	0.1997	4.50E-12	chr:1:111802591
1	rs4323713	111823220	T	0.1997	4.50E-12	chr:1:111802591
1	rs5029527	111818923	T	0.1997	4.50E-12	chr:1:111802591
1	rs12401376	111828474	A	0.195	4.57E-12	chr:1:111802591
1	rs3118204	111810952	A	5.532	4.79E-12	chr:1:111802591
1	rs17027272	111823851	A	5.334	7.55E-12	chr:1:111802591
1	rs1925358	111820367	T	5.334	7.55E-12	chr:1:111802591
1	rs2494014	111819921	G	5.334	7.55E-12	chr:1:111802591
1	rs4838895	111821459	T	5.334	7.55E-12	chr:1:111802591
1	rs61803493	111820997	C	5.334	7.55E-12	chr:1:111802591
1	rs9429593	111823315	T	5.334	7.55E-12	chr:1:111802591
1	rs12738135	111809571	G	0.2257	9.06E-12	chr:1:111802591
1	rs4272622	111825435	C	5.174	1.13E-11	chr:1:111802591
1	rs4351635	111825754	T	5.174	1.13E-11	chr:1:111802591
1	rs117495492	111815317	A	5.213	1.16E-11	chr:1:111802591
1	rs12028469	111822648	A	5.255	1.30E-11	chr:1:111802591
1	rs11102238	111843082	G	5.334	2.21E-11	chr:1:111802591
1	rs12026810	111846599	G	5.311	2.27E-11	chr:1:111802591
1	rs41281400	111827898	A	0.2163	2.69E-11	chr:1:111802591
1	rs2494012	111808937	T	179.3	2.80E-11	chr:1:111802591
1	rs10776720	111808276	A	162.8	3.18E-11	chr:1:111802591

1	rs10776719	111808180	T	162.6	4.75E-11	chr:1:111802591
1	rs1590499	111804651	A	162.6	4.75E-11	chr:1:111802591
1	rs1853784	111804717	C	162.6	4.75E-11	chr:1:111802591
1	rs2764538	111804370	A	162.6	4.75E-11	chr:1:111802591
1	rs2764549	111808546	C	162.6	4.75E-11	chr:1:111802591
1	rs2820062	111805966	G	162.6	4.75E-11	chr:1:111802591
1	rs76437095	111842681	C	0.2705	4.37E-10	chr:1:111802591
1	rs75510830	111842692	C	0.2792	8.31E-10	chr:1:111802591
1	rs79096750	111842693	A	0.2792	8.31E-10	chr:1:111802591
1	rs12752158	111833937	A	0.2771	1.03E-09	chr:1:111802591
1	rs12736391	111844599	C	0.2776	1.04E-09	chr:1:111802591
1	rs11578207	111837366	C	0.2877	2.15E-09	chr:1:111802591
1	rs11578209	111837382	A	0.2877	2.15E-09	chr:1:111802591
1	rs11584291	111846803	T	0.2877	2.15E-09	chr:1:111802591
1	rs12402658	111840855	G	0.2877	2.15E-09	chr:1:111802591
1	rs12410579	111835047	G	0.2877	2.15E-09	chr:1:111802591
1	rs12410848	111834984	C	0.2877	2.15E-09	chr:1:111802591
1	rs12562861	111838002	A	0.2877	2.15E-09	chr:1:111802591
1	rs12562929	111846331	G	0.2877	2.15E-09	chr:1:111802591
1	rs12563147	111846300	C	0.2877	2.15E-09	chr:1:111802591
1	rs12563158	111846416	G	0.2877	2.15E-09	chr:1:111802591
1	rs12727314	111846533	T	0.2877	2.15E-09	chr:1:111802591
1	rs12734016	111842985	C	0.2877	2.15E-09	chr:1:111802591
1	rs12735055	111842947	A	0.2877	2.15E-09	chr:1:111802591
1	rs12735944	111844348	T	0.2877	2.15E-09	chr:1:111802591
1	rs12739716	111836168	T	0.2877	2.15E-09	chr:1:111802591
1	rs12739896	111844175	G	0.2877	2.15E-09	chr:1:111802591
1	rs12751372	111846603	C	0.2877	2.15E-09	chr:1:111802591
1	rs12753801	111844522	C	0.2877	2.15E-09	chr:1:111802591
1	rs12755026	111844556	T	0.2877	2.15E-09	chr:1:111802591
1	rs12755376	111836023	T	0.2877	2.15E-09	chr:1:111802591
1	rs12758947	111842897	A	0.2877	2.15E-09	chr:1:111802591
1	rs28805711	111844103	T	0.2877	2.15E-09	chr:1:111802591
1	rs28826608	111843891	T	0.2877	2.15E-09	chr:1:111802591
1	rs28849976	111844011	A	0.2877	2.15E-09	chr:1:111802591
1	rs28857204	111844087	T	0.2877	2.15E-09	chr:1:111802591
1	rs28889692	111844007	G	0.2877	2.15E-09	chr:1:111802591
1	rs34971749	111845082	T	0.2877	2.15E-09	chr:1:111802591
1	rs35360118	111839319	T	0.2877	2.15E-09	chr:1:111802591
1	rs35764762	111843214	G	0.2877	2.15E-09	chr:1:111802591
1	rs35779360	111839515	G	0.2877	2.15E-09	chr:1:111802591
1	rs35888305	111845241	C	0.2877	2.15E-09	chr:1:111802591
1	rs4406630	111834733	T	0.2877	2.15E-09	chr:1:111802591

1	rs4546920	111836846	T	0.2877	2.15E-09	chr:1:111802591
1	rs4546921	111836868	T	0.2877	2.15E-09	chr:1:111802591
1	rs4562620	111835713	A	0.2877	2.15E-09	chr:1:111802591
1	rs4838901	111838605	G	0.2877	2.15E-09	chr:1:111802591
1	rs4838902	111838694	T	0.2877	2.15E-09	chr:1:111802591
1	rs4838903	111838832	T	0.2877	2.15E-09	chr:1:111802591
1	rs4838904	111838962	C	0.2877	2.15E-09	chr:1:111802591
1	rs4839117	111839037	G	0.2877	2.15E-09	chr:1:111802591
1	rs61382086	111842094	T	0.2877	2.15E-09	chr:1:111802591
1	rs66475510	111845588	A	0.2877	2.15E-09	chr:1:111802591
1	rs6669614	111840130	T	0.2877	2.15E-09	chr:1:111802591
1	rs6688167	111841315	C	0.2877	2.15E-09	chr:1:111802591
1	rs6688184	111841367	C	0.2877	2.15E-09	chr:1:111802591
1	rs6699184	111841409	A	0.2877	2.15E-09	chr:1:111802591
1	rs7413908	111842524	T	0.2877	2.15E-09	chr:1:111802591
1	rs7514165	111836591	G	0.2877	2.15E-09	chr:1:111802591
1	rs7525672	111836444	G	0.2877	2.15E-09	chr:1:111802591
1	rs7539071	111841089	G	0.2877	2.15E-09	chr:1:111802591
1	rs77805328	111842813	A	0.2877	2.15E-09	chr:1:111802591
1	rs78611689	111840605	G	0.2877	2.15E-09	chr:1:111802591
1	rs4460637	111847226	T	0.2952	3.30E-09	chr:1:111802591
1	rs34306809	111845903	A	0.2956	3.74E-09	chr:1:111802591
1	rs142458373	111842752	C	0.2971	4.08E-09	chr:1:111802591
1	rs6688274	111841418	G	0.3145	1.07E-08	chr:1:111802591
1	rs9659635	111808642	A	0.2585	4.61E-08	chr:1:111802591
1	rs11809538	111801853	A	0.2693	7.46E-08	chr:1:111802591
1	rs3927471	111802912	C	0.2693	7.46E-08	chr:1:111802591
1	rs9660405	111808946	C	0.2779	9.71E-08	chr:1:111802591
1	rs61808280	111807687	C	0.2805	1.70E-07	chr:1:111802591
1	rs7550145	111804157	T	0.2805	1.70E-07	chr:1:111802591
1	rs11800770	111813650	T	0.2752	1.72E-07	chr:1:111802591
1	rs12034487	111805474	G	5.656	2.81E-07	chr:1:111802591
1	rs6703883	111820644	G	0.2861	3.81E-07	chr:1:111802591
1	rs2764548	111808585	T	0.3115	3.82E-07	chr:1:111802591
1	rs2820059	111808392	G	0.3115	3.82E-07	chr:1:111802591
1	rs6671713	111815790	T	0.2985	6.08E-07	chr:1:111802591
1	rs10857867	111810878	A	5.29	6.12E-07	chr:1:111802591
1	rs6655936	111821516	G	0.3107	1.32E-06	chr:1:111802591
1	rs6698373	111816497	A	0.3107	1.32E-06	chr:1:111802591
1	rs6702380	111818052	A	0.3107	1.32E-06	chr:1:111802591
1	rs7512322	111818550	A	0.3107	1.32E-06	chr:1:111802591
1	rs2494013	111813821	A	0.3369	1.49E-06	chr:1:111802591
1	rs3118206	111814461	G	0.3369	1.49E-06	chr:1:111802591

1	rs3122594	111813735	T	0.3369	1.49E-06	chr:1:111802591
1	rs3122595	111813951	T	0.3369	1.49E-06	chr:1:111802591
1	rs78346400	111844342	G	0.408	1.68E-06	chr:1:111802591
1	rs11102231	111817468	A	0.3428	2.05E-06	chr:1:111802591
1	rs3122593	111810965	A	0.3421	2.05E-06	chr:1:111802591
1	rs3122591	111810174	C	0.348	2.80E-06	chr:1:111802591
1	rs6696410	111840987	A	0.3791	6.84E-06	chr:1:111802591
1	rs7525014	111835498	A	0.3791	6.84E-06	chr:1:111802591
1	rs10494131	111824711	C	0.3334	7.29E-06	chr:1:111802591
1	rs41282488	111828423	T	0.3334	7.29E-06	chr:1:111802591
1	rs6537690	111824209	A	0.3334	7.29E-06	chr:1:111802591
1	rs6688201	111826023	G	0.3334	7.29E-06	chr:1:111802591
1	rs6688325	111826175	T	0.3334	7.29E-06	chr:1:111802591
1	rs12048213	111821434	T	3.702	8.45E-06	chr:1:111802591
1	rs12139505	111816865	A	3.702	8.45E-06	chr:1:111802591
1	rs6537691	111824255	T	0.3394	9.75E-06	chr:1:111802591
1	rs2783441	111751534	T	2.302	1.04E-05	chr:1:111802591
1	rs2783442	111754616	T	2.292	1.05E-05	chr:1:111802591
1	rs10857870	111829220	C	0.3438	1.11E-05	chr:1:111802591
1	rs12064178	111841263	G	0.3592	1.18E-05	chr:1:111802591
1	rs113703527	111840317	A	0.367	1.69E-05	chr:1:111802591
1	rs2820082	111800472	T	0.347	1.77E-05	chr:1:111802591
1	rs1361049	111908713	C	0.4207	2.24E-05	chr:1:111802591
1	rs10494132	111832223	C	0.3756	2.36E-05	chr:1:111802591
1	rs12144594	111834306	A	0.3756	2.36E-05	chr:1:111802591
1	rs10857869	111822699	T	3.24	3.02E-05	chr:1:111802591
1	rs6687390	111825226	T	0.39	3.12E-05	chr:1:111802591
1	rs6537695	111854223	C	0.4452	4.10E-05	chr:1:111802591
1	rs80303510	111823697	C	0.1332	4.18E-05	chr:1:111802591
1	rs3886706	111788779	T	0.4697	4.24E-05	chr:1:111802591
1	rs2783440	111748488	G	0.4493	4.36E-05	chr:1:111802591
1	rs11485090	111846123	T	0.3939	4.87E-05	chr:1:111802591
1	rs6671210	111845463	T	0.3939	4.87E-05	chr:1:111802591
1	rs12082033	111798711	C	0.3422	5.49E-05	chr:1:111802591
3	rs78795877	125617617	T	4.022	8.07E-07	chr:3:125609298
3	rs11917669	125599068	T	3.954	1.16E-06	chr:3:125609298
3	rs11925437	125599216	A	3.954	1.16E-06	chr:3:125609298
3	rs12633485	125605577	T	3.954	1.16E-06	chr:3:125609298
3	rs12634512	125631766	T	3.954	1.16E-06	chr:3:125609298
3	rs12637469	125600407	T	3.954	1.16E-06	chr:3:125609298
3	rs200006127	125610368	G	3.954	1.16E-06	chr:3:125609298
3	rs6767408	125623666	T	3.954	1.16E-06	chr:3:125609298
3	rs6767618	125623941	G	3.954	1.16E-06	chr:3:125609298

3	rs6772193	125597432	A	3.954	1.16E-06	chr:3:125609298
3	rs74470729	125621098	T	3.954	1.16E-06	chr:3:125609298
3	rs74494751	125615642	G	3.954	1.16E-06	chr:3:125609298
3	rs75313595	125601479	A	3.954	1.16E-06	chr:3:125609298
3	rs75431854	125601942	A	3.954	1.16E-06	chr:3:125609298
3	rs75687938	125600858	T	3.954	1.16E-06	chr:3:125609298
3	rs77342220	125608463	T	3.954	1.16E-06	chr:3:125609298
3	rs78438430	125607169	C	3.954	1.16E-06	chr:3:125609298
3	rs79143534	125622688	G	3.954	1.16E-06	chr:3:125609298
3	rs79584539	125600024	T	3.954	1.16E-06	chr:3:125609298
3	rs79647471	125622158	A	3.954	1.16E-06	chr:3:125609298
3	rs79780713	125596589	G	3.954	1.16E-06	chr:3:125609298
3	rs9714319	125612678	T	3.954	1.16E-06	chr:3:125609298
3	rs34721907	125637285	A	3.938	1.40E-06	chr:3:125609298
3	rs58198667	125635719	T	3.938	1.40E-06	chr:3:125609298
3	rs74402623	125643173	A	3.938	1.40E-06	chr:3:125609298
3	rs80022059	125641856	A	3.938	1.40E-06	chr:3:125609298
3	rs76697478	125622811	A	3.88	1.45E-06	chr:3:125609298
3	rs75837707	125630833	T	3.779	2.29E-06	chr:3:125609298
3	rs79344588	125632117	A	3.749	2.33E-06	chr:3:125609298
3	rs60487455	125619863	G	3.784	2.46E-06	chr:3:125609298
3	rs60374286	125630173	T	3.475	4.26E-06	chr:3:125609298
3	rs112567450	125592200	T	3.532	5.32E-06	chr:3:125609298
3	rs11922653	125591569	A	3.532	5.32E-06	chr:3:125609298
3	rs35540257	125590518	A	3.532	5.32E-06	chr:3:125609298
3	rs11711436	125629149	T	3.363	6.60E-06	chr:3:125609298
3	rs9859596	125593349	T	3.277	1.28E-05	chr:3:125609298
3	rs11714778	129762082	C	0.1675	1.68E-14	chr:3:129776131
3	rs12495049	129810234	T	0.1675	1.68E-14	chr:3:129776131
3	rs73208686	129762898	C	0.1675	1.68E-14	chr:3:129776131
3	rs11716622	129809592	A	0.1671	1.77E-14	chr:3:129776131
3	rs12634803	129807284	T	0.1713	1.84E-14	chr:3:129776131
3	rs11718709	129811323	A	0.1822	6.54E-14	chr:3:129776131
3	rs111717650	129814301	A	0.1837	9.60E-14	chr:3:129776131
3	rs73210759	129813745	T	0.1859	9.98E-14	chr:3:129776131
3	rs9758049	129814863	A	0.1949	1.03E-12	chr:3:129776131
3	rs73866092	129815599	C	0.2471	5.40E-11	chr:3:129776131
3	rs75630148	129821465	T	0.2443	1.07E-10	chr:3:129776131
3	rs73210760	129818875	A	0.264	1.28E-10	chr:3:129776131
3	rs56088005	129821650	A	0.257	1.36E-10	chr:3:129776131
3	rs12632200	129816368	T	0.2507	1.50E-10	chr:3:129776131
3	rs113679585	129819332	A	0.2525	1.58E-10	chr:3:129776131
3	rs146549952	129816552	T	0.2527	1.87E-10	chr:3:129776131

3	rs6804887	129820568	A	0.2615	2.28E-10	chr:3:129776131
3	rs112581710	129820151	A	0.2615	2.58E-10	chr:3:129776131
3	rs12637557	129815930	A	0.2615	2.58E-10	chr:3:129776131
3	rs78806708	129822616	C	0.2636	3.14E-10	chr:3:129776131
3	rs9713340	129822492	A	0.2636	3.14E-10	chr:3:129776131
3	rs9713334	129822494	T	0.2639	3.72E-10	chr:3:129776131
3	rs118004362	129820231	T	0.2783	4.21E-10	chr:3:129776131
3	rs56218598	129823899	A	0.2725	5.02E-10	chr:3:129776131
3	rs9713344	129822552	A	0.2725	5.02E-10	chr:3:129776131
3	rs9713346	129822634	A	0.2725	5.02E-10	chr:3:129776131
3	rs111270043	129820915	T	0.2729	5.21E-10	chr:3:129776131
3	rs61073628	129817316	T	0.2731	5.43E-10	chr:3:129776131
3	rs74203764	129817379	T	0.2731	5.43E-10	chr:3:129776131
3	rs112577058	129818750	G	0.2858	6.98E-10	chr:3:129776131
3	rs12638292	129816961	T	0.2858	6.98E-10	chr:3:129776131
3	rs56039375	129818509	G	0.2858	6.98E-10	chr:3:129776131
3	rs56235703	129818389	G	0.2858	6.98E-10	chr:3:129776131
3	rs6788479	129817708	G	0.2899	8.37E-10	chr:3:129776131
3	rs34158162	129828454	C	0.2758	9.15E-10	chr:3:129776131
3	rs7428344	129826100	T	0.2758	9.15E-10	chr:3:129776131
3	rs9681064	129846313	C	0.2911	9.88E-10	chr:3:129776131
3	rs6804728	129820580	T	0.2786	1.06E-09	chr:3:129776131
3	rs73210763	129827901	G	0.2762	1.08E-09	chr:3:129776131
3	rs112987689	129824001	T	0.2962	1.28E-09	chr:3:129776131
3	rs6808888	129822441	A	0.2962	1.28E-09	chr:3:129776131
3	rs12497818	129843254	C	0.2807	1.30E-09	chr:3:129776131
3	rs9754694	129844166	T	0.2807	1.30E-09	chr:3:129776131
3	rs75177871	129821816	G	0.2847	1.42E-09	chr:3:129776131
3	rs111775688	129826402	T	0.2853	1.48E-09	chr:3:129776131
3	rs113674105	129830064	A	0.2853	1.48E-09	chr:3:129776131
3	rs137960198	129828560	T	0.2853	1.48E-09	chr:3:129776131
3	rs149652485	129830726	A	0.2853	1.48E-09	chr:3:129776131
3	rs7428346	129826002	T	0.2853	1.48E-09	chr:3:129776131
3	rs12491391	129843530	G	0.281	1.55E-09	chr:3:129776131
3	rs28477611	129844824	A	0.2994	1.55E-09	chr:3:129776131
3	rs28754684	129844800	C	0.2994	1.55E-09	chr:3:129776131
3	rs12495527	129822950	G	0.3023	1.91E-09	chr:3:129776131
3	rs113747501	129826513	C	0.2992	1.95E-09	chr:3:129776131
3	rs9713120	129850043	T	0.3029	2.04E-09	chr:3:129776131
3	rs10934908	129840300	T	0.2901	2.09E-09	chr:3:129776131
3	rs113421391	129830961	C	0.2901	2.09E-09	chr:3:129776131
3	rs12233562	129845749	A	0.2901	2.09E-09	chr:3:129776131
3	rs28442137	129840485	T	0.2901	2.09E-09	chr:3:129776131

3	rs4569696	129834986	A	0.2901	2.09E-09	chr:3:129776131
3	rs55908933	129835084	T	0.2901	2.09E-09	chr:3:129776131
3	rs56171265	129833240	T	0.2901	2.09E-09	chr:3:129776131
3	rs56292584	129833209	T	0.2901	2.09E-09	chr:3:129776131
3	rs56314950	129833031	T	0.2901	2.09E-09	chr:3:129776131
3	rs56957706	129845338	C	0.2901	2.09E-09	chr:3:129776131
3	rs60352193	129830922	C	0.2901	2.09E-09	chr:3:129776131
3	rs6809980	129847462	A	0.2901	2.09E-09	chr:3:129776131
3	rs9754720	129844403	T	0.2901	2.09E-09	chr:3:129776131
3	rs12497879	129843468	T	0.3034	2.29E-09	chr:3:129776131
3	rs6784328	129822059	G	0.3074	2.56E-09	chr:3:129776131
3	rs6769324	129820903	G	0.3055	2.61E-09	chr:3:129776131
3	rs112405295	129824844	A	0.2867	2.61E-09	chr:3:129776131
3	rs13083073	129819247	C	0.2959	2.66E-09	chr:3:129776131
3	rs59443817	129817290	G	0.2959	2.66E-09	chr:3:129776131
3	rs9815579	129755175	T	0.2927	2.87E-09	chr:3:129776131
3	rs113076563	129818689	C	0.2962	3.07E-09	chr:3:129776131
3	rs55773357	129849781	T	0.2915	3.23E-09	chr:3:129776131
3	rs56312715	129849847	A	0.2915	3.23E-09	chr:3:129776131
3	rs10934907	129830493	C	0.3105	3.84E-09	chr:3:129776131
3	rs12107141	129844617	C	0.3105	3.84E-09	chr:3:129776131
3	rs12233374	129845660	A	0.3105	3.84E-09	chr:3:129776131
3	rs12487314	129842009	C	0.3105	3.84E-09	chr:3:129776131
3	rs12487371	129842190	G	0.3105	3.84E-09	chr:3:129776131
3	rs12491387	129843518	G	0.3105	3.84E-09	chr:3:129776131
3	rs12491430	129843630	G	0.3105	3.84E-09	chr:3:129776131
3	rs12636674	129834567	A	0.3105	3.84E-09	chr:3:129776131
3	rs17735722	129843196	G	0.3105	3.84E-09	chr:3:129776131
3	rs28475731	129844692	T	0.3105	3.84E-09	chr:3:129776131
3	rs28567533	129845174	A	0.3105	3.84E-09	chr:3:129776131
3	rs28798137	129845247	A	0.3105	3.84E-09	chr:3:129776131
3	rs35543987	129842031	A	0.3105	3.84E-09	chr:3:129776131
3	rs376047039	129845958	G	0.3105	3.84E-09	chr:3:129776131
3	rs4072146	129829463	A	0.3105	3.84E-09	chr:3:129776131
3	rs4337672	129839907	T	0.3105	3.84E-09	chr:3:129776131
3	rs55977977	129831316	G	0.3105	3.84E-09	chr:3:129776131
3	rs56274321	129834254	A	0.3105	3.84E-09	chr:3:129776131
3	rs57173421	129843153	G	0.3105	3.84E-09	chr:3:129776131
3	rs59318782	129842972	G	0.3105	3.84E-09	chr:3:129776131
3	rs61433746	129836538	T	0.3105	3.84E-09	chr:3:129776131
3	rs6795939	129842885	T	0.3105	3.84E-09	chr:3:129776131
3	rs6796054	129842995	A	0.3105	3.84E-09	chr:3:129776131
3	rs6796132	129842958	A	0.3105	3.84E-09	chr:3:129776131

3	rs6796817	129837836	T	0.3105	3.84E-09	chr:3:129776131
3	rs6808274	129842797	C	0.3105	3.84E-09	chr:3:129776131
3	rs6808384	129842900	C	0.3105	3.84E-09	chr:3:129776131
3	rs72990317	129830425	G	0.3105	3.84E-09	chr:3:129776131
3	rs73210765	129841875	A	0.3105	3.84E-09	chr:3:129776131
3	rs7432233	129825955	G	0.3105	3.84E-09	chr:3:129776131
3	rs9289355	129838184	G	0.3105	3.84E-09	chr:3:129776131
3	rs9754713	129837151	C	0.3105	3.84E-09	chr:3:129776131
3	rs9757129	129844461	G	0.3105	3.84E-09	chr:3:129776131
3	rs11708901	129847846	C	0.3108	4.03E-09	chr:3:129776131
3	rs11720489	129848042	T	0.3108	4.03E-09	chr:3:129776131
3	rs11720599	129848148	A	0.3108	4.03E-09	chr:3:129776131
3	rs147534743	129849886	G	0.3108	4.03E-09	chr:3:129776131
3	rs34086701	129846916	C	0.3108	4.03E-09	chr:3:129776131
3	rs4491951	129850208	C	0.3108	4.03E-09	chr:3:129776131
3	rs4619822	129850342	A	0.3108	4.03E-09	chr:3:129776131
3	rs56279301	129848747	T	0.3108	4.03E-09	chr:3:129776131
3	rs59667515	129848796	C	0.3108	4.03E-09	chr:3:129776131
3	rs6776708	129847555	C	0.3108	4.03E-09	chr:3:129776131
3	rs74442213	129846119	A	0.3108	4.03E-09	chr:3:129776131
3	rs9682942	129846098	T	0.3108	4.03E-09	chr:3:129776131
3	rs9682954	129846247	G	0.3108	4.03E-09	chr:3:129776131
3	rs9713121	129850044	G	0.3108	4.03E-09	chr:3:129776131
3	rs9713819	129849956	G	0.3108	4.03E-09	chr:3:129776131
3	rs56086823	129841378	A	0.3106	4.29E-09	chr:3:129776131
3	rs10934909	129849297	T	0.3147	4.69E-09	chr:3:129776131
3	rs10934912	129849479	T	0.3171	6.10E-09	chr:3:129776131
3	rs35523538	129832419	C	0.2677	6.38E-09	chr:3:129776131
3	rs28726131	129844802	G	0.3245	7.21E-09	chr:3:129776131
3	rs34831897	129759914	T	0.3115	7.82E-09	chr:3:129776131
3	rs12497877	129843458	A	0.3316	9.94E-09	chr:3:129776131
3	rs72990312	129826675	C	0.311	1.09E-08	chr:3:129776131
3	rs11712662	129848252	G	0.3344	1.70E-08	chr:3:129776131
3	rs112319225	129826344	G	0.3353	1.71E-08	chr:3:129776131
3	rs113111443	129826394	C	0.3353	1.71E-08	chr:3:129776131
3	rs12233436	129845766	G	0.3353	1.71E-08	chr:3:129776131
3	rs6774812	129844022	G	0.3353	1.71E-08	chr:3:129776131
3	rs11721284;rs57423551	129844950	G	0.3061	1.73E-08	chr:3:129776131
3	rs6809088	129837584	C	0.3382	2.04E-08	chr:3:129776131
3	rs7428327	129826031	T	0.3363	2.51E-08	chr:3:129776131
3	rs57573544	129845394	G	0.3396	2.57E-08	chr:3:129776131
3	rs58593172	129845403	G	0.3396	2.57E-08	chr:3:129776131
3	rs61532278	129844947	G	0.2703	2.68E-08	chr:3:129776131

3	rs59803194	129845429	A	0.3507	4.44E-08	chr:3:129776131
3	rs55891359	129760185	C	2.81	4.91E-08	chr:3:129776131
3	rs187016983	129845427	A	0.3518	5.36E-08	chr:3:129776131
3	rs60351474	129845423	G	0.3518	5.36E-08	chr:3:129776131
3	rs10934910	129849334	G	0.3681	1.16E-07	chr:3:129776131
3	rs10934911	129849366	C	0.3681	1.16E-07	chr:3:129776131
3	rs34233228	129750876	A	2.707	1.26E-07	chr:3:129776131
3	rs35882657	129750735	C	2.707	1.26E-07	chr:3:129776131
3	rs56110318	129849681	G	0.3679	1.42E-07	chr:3:129776131
3	rs9713134	129850080	A	0.3677	1.70E-07	chr:3:129776131
3	rs13093111	129754672	A	2.562	2.59E-07	chr:3:129776131
3	rs2403477	129864875	T	0.3241	2.95E-07	chr:3:129776131
3	rs12488592	129752490	C	2.473	5.29E-07	chr:3:129776131
3	rs6799884	129814251	T	0.3094	1.29E-06	chr:3:129776131
3	rs9863859	129814862	T	2.763	2.13E-06	chr:3:129776131
3	rs35695300	129760923	T	2.806	2.42E-06	chr:3:129776131
3	rs9832290	129758522	C	2.806	2.42E-06	chr:3:129776131
3	rs9879964	129760340	T	2.806	2.42E-06	chr:3:129776131
3	rs11914710	129810346	C	2.894	2.57E-06	chr:3:129776131
3	rs13061782	129810800	A	2.894	2.57E-06	chr:3:129776131
3	rs13081624	129810799	G	2.894	2.57E-06	chr:3:129776131
3	rs6767964	129809899	A	2.894	2.57E-06	chr:3:129776131
3	rs6803922	129810158	T	2.894	2.57E-06	chr:3:129776131
3	rs6804080	129810128	A	2.894	2.57E-06	chr:3:129776131
3	rs7644173	129808755	T	2.894	2.57E-06	chr:3:129776131
3	rs9849970	129807203	T	2.894	2.57E-06	chr:3:129776131
3	rs9846907	129757554	T	2.279	2.59E-06	chr:3:129776131
3	rs9864424	129815139	A	2.625	4.89E-06	chr:3:129776131
3	rs6791923	129706392	G	2.273	6.39E-06	chr:3:129776131
3	rs12632095	129757870	C	2.232	8.54E-06	chr:3:129776131
3	rs4610271	129813301	G	2.221	9.02E-06	chr:3:129776131
3	rs9812409	129814502	T	2.795	1.03E-05	chr:3:129776131
3	rs11707425	129749980	G	2.402	1.15E-05	chr:3:129776131
3	rs10934906	129813062	T	2.181	1.21E-05	chr:3:129776131
3	rs4487280	129813361	G	2.181	1.21E-05	chr:3:129776131
3	rs10934901	129747902	T	2.219	1.25E-05	chr:3:129776131
3	rs58302754	129763329	A	2.168	1.52E-05	chr:3:129776131
3	rs7625942	129951454	G	2.392	1.69E-05	chr:3:129776131
3	rs11708560	129809131	A	2.093	2.54E-05	chr:3:129776131
3	rs6806210	129807027	T	2.093	2.54E-05	chr:3:129776131
3	rs7622131	129808869	A	2.093	2.54E-05	chr:3:129776131
3	rs12487635	195672459	T	0.02666	2.01E-09	chr:3:195654394
3	rs12497996	195672534	C	0.02666	2.01E-09	chr:3:195654394

3	rs141789999	195676214	G	0.02666	2.01E-09	chr:3:195654394
3	rs146447703	195672685	A	0.02666	2.01E-09	chr:3:195654394
3	rs72499654	195662770	C	0.01375	2.44E-09	chr:3:195654394
3	rs4927814	195684297	C	0.03707	2.82E-09	chr:3:195654394
3	rs111452973	195683167	C	0.02814	3.11E-09	chr:3:195654394
3	rs72499666	195683479	G	0.02399	3.12E-09	chr:3:195654394
3	rs111857020	195684226	A	0.03748	3.15E-09	chr:3:195654394
3	rs112372218	195684599	T	0.03748	3.15E-09	chr:3:195654394
3	rs12488793	195682872	G	0.03748	3.15E-09	chr:3:195654394
3	rs12489547	195682747	C	0.03748	3.15E-09	chr:3:195654394
3	rs77298109	195683022	G	0.03748	3.15E-09	chr:3:195654394
3	rs111237905	195683159	A	0.02838	3.16E-09	chr:3:195654394
3	rs111529852	195683186	C	0.02838	3.16E-09	chr:3:195654394
3	rs113181724	195683118	A	0.02838	3.16E-09	chr:3:195654394
3	rs111569452	195683174	C	0.02872	3.19E-09	chr:3:195654394
3	rs12489913	195673933	G	0.02946	3.26E-09	chr:3:195654394
3	rs7372023	195674317	T	0.03586	3.33E-09	chr:3:195654394
3	rs74406097	195658661	C	0.01484	3.34E-09	chr:3:195654394
3	rs74894130	195660697	T	0.01484	3.34E-09	chr:3:195654394
3	rs76268338	195661315	G	0.01484	3.34E-09	chr:3:195654394
3	rs77570361	195659561	G	0.01484	3.34E-09	chr:3:195654394
3	rs78128847	195653985	T	0.01484	3.34E-09	chr:3:195654394
3	rs78809271	195659736	A	0.01484	3.34E-09	chr:3:195654394
3	rs79026789	195660712	A	0.01484	3.34E-09	chr:3:195654394
3	rs79516196	195658662	A	0.01484	3.34E-09	chr:3:195654394
3	rs62285304	195675478	T	0.02905	4.21E-09	chr:3:195654394
3	rs7371976	195684515	A	0.04509	4.80E-09	chr:3:195654394
3	rs141586514	195688130	C	0.03577	6.35E-09	chr:3:195654394
3	rs112619958	195693191	G	0.03828	6.66E-09	chr:3:195654394
3	rs112957249	195693236	T	0.03828	6.66E-09	chr:3:195654394
3	rs149593867	195688363	C	0.03828	6.66E-09	chr:3:195654394
3	rs78320742	195689817	T	0.03828	6.66E-09	chr:3:195654394
3	rs113310924	195734614	T	0.02413	6.72E-09	chr:3:195654394
3	rs140057626	195728317	A	0.02413	6.72E-09	chr:3:195654394
3	rs146692935	195723648	C	0.02413	6.72E-09	chr:3:195654394
3	rs2593984	195725621	A	0.02413	6.72E-09	chr:3:195654394
3	rs34254399	195740067	T	0.02413	6.72E-09	chr:3:195654394
3	rs4927846	195732325	G	0.02413	6.72E-09	chr:3:195654394
3	rs4927847	195736627	C	0.02413	6.72E-09	chr:3:195654394
3	rs72499671	195724614	C	0.02413	6.72E-09	chr:3:195654394
3	rs72499672	195726839	G	0.02413	6.72E-09	chr:3:195654394
3	rs72499680	195730979	A	0.02413	6.72E-09	chr:3:195654394
3	rs75342758	195737511	T	0.02413	6.72E-09	chr:3:195654394

3	rs75962942	195728678	G	0.02413	6.72E-09	chr:3:195654394
3	rs76499913	195729028	T	0.02413	6.72E-09	chr:3:195654394
3	rs76655042	195736059	T	0.02413	6.72E-09	chr:3:195654394
3	rs78147044	195745484	A	0.02413	6.72E-09	chr:3:195654394
3	rs147267489	195693977	G	0.03869	6.77E-09	chr:3:195654394
3	rs188069625	195692975	A	0.02804	7.24E-09	chr:3:195654394
3	rs147038037	195688166	C	0.03667	7.28E-09	chr:3:195654394
3	rs538034368	195683992	A	0.06219	7.41E-09	chr:3:195654394
3	rs575022724	195684009	G	0.06219	7.41E-09	chr:3:195654394
3	rs114155151	195688314	G	0.03923	7.64E-09	chr:3:195654394
3	rs79698107	195760322	A	0.02842	8.59E-09	chr:3:195654394
3	rs4927856	195756474	T	0.02825	8.72E-09	chr:3:195654394
3	rs77192409	195751543	C	0.02825	8.72E-09	chr:3:195654394
3	rs79238342	195757954	A	0.02825	8.72E-09	chr:3:195654394
3	rs140893852	195691373	T	0.03993	9.32E-09	chr:3:195654394
3	rs143076671	195692843	A	0.03993	9.32E-09	chr:3:195654394
3	rs145435068	195693305	T	0.03993	9.32E-09	chr:3:195654394
3	rs148761326	195691637	A	0.03993	9.32E-09	chr:3:195654394
3	rs150157283	195691387	C	0.03993	9.32E-09	chr:3:195654394
3	rs201250247	195691199	G	0.03993	9.32E-09	chr:3:195654394
3	rs77759126	195692828	C	0.03993	9.32E-09	chr:3:195654394
3	rs368599720	195668122	A	0.01594	9.34E-09	chr:3:195654394
3	rs368905451	195729075	T	0.03402	9.38E-09	chr:3:195654394
3	rs138213397	195676057	A	0.03206	9.97E-09	chr:3:195654394
3	rs371452292	195710165	C	0.03956	1.11E-08	chr:3:195654394
3	rs183890720	195684486	G	0.05014	1.12E-08	chr:3:195654394
3	rs191821895	195684485	G	0.05014	1.12E-08	chr:3:195654394
3	rs7619087	195676518	A	0.02784	1.23E-08	chr:3:195654394
3	rs200631070	195671274	T	0.03369	1.43E-08	chr:3:195654394
3	rs189722989	195690118	A	0.03862	1.48E-08	chr:3:195654394
3	rs28660371	195690105	G	0.03862	1.48E-08	chr:3:195654394
3	rs12495891	195762306	G	0.03254	1.54E-08	chr:3:195654394
3	rs76633380	195762727	A	0.03254	1.54E-08	chr:3:195654394
3	rs75737544	195771900	G	0.0323	1.55E-08	chr:3:195654394
3	rs113488782	195684121	C	0.043	1.55E-08	chr:3:195654394
3	rs4927855	195754452	T	0.03213	1.61E-08	chr:3:195654394
3	rs1080211	195764711	C	0.0326	1.69E-08	chr:3:195654394
3	rs55990110	195768472	T	0.0326	1.69E-08	chr:3:195654394
3	rs62284005	195768079	T	0.0326	1.69E-08	chr:3:195654394
3	rs62284007	195770947	A	0.0326	1.69E-08	chr:3:195654394
3	rs62284008	195771155	A	0.0326	1.69E-08	chr:3:195654394
3	rs78587262	195717867	G	0.0419	1.82E-08	chr:3:195654394
3	rs200743347	195722294	A	0.04058	2.31E-08	chr:3:195654394

3	rs138904938	195667637	T	0.02068	2.84E-08	chr:3:195654394
3	rs79540055	195692516	T	0.05406	3.79E-08	chr:3:195654394
3	rs72499659	195667840	C	0.02053	3.85E-08	chr:3:195654394
3	rs113876944	195682180	A	0.04889	4.08E-08	chr:3:195654394
3	rs142455000	195831362	C	0.05184	4.24E-08	chr:3:195654394
3	rs9823320	195771629	G	0.07471	4.94E-08	chr:3:195654394
3	rs62284010	195773092	T	0.05218	5.19E-08	chr:3:195654394
3	rs73208016	195650120	A	0.05158	6.23E-08	chr:3:195654394
3	rs7372331	195684523	G	0.07047	6.80E-08	chr:3:195654394
3	rs112788096	195682140	T	0.05243	8.05E-08	chr:3:195654394
3	rs113786804	195682145	G	0.05243	8.05E-08	chr:3:195654394
3	rs73208017	195650428	G	0.08354	8.22E-08	chr:3:195654394
3	rs145575126	195683366	T	0.04069	8.45E-08	chr:3:195654394
3	rs3107709	195683255	C	0.06118	8.57E-08	chr:3:195654394
3	rs112586036	195762470	A	0.06276	8.63E-08	chr:3:195654394
3	rs9325402	195400498	T	18.53	9.59E-08	chr:3:195654394
3	rs114004364	195682365	G	0.06633	1.05E-07	chr:3:195654394
3	rs113809427	195762456	T	0.06427	1.07E-07	chr:3:195654394
3	rs200402998	195676577	A	0.0638	1.07E-07	chr:3:195654394
3	rs4927707	195771725	T	0.0815	1.07E-07	chr:3:195654394
3	rs4927705	195751397	T	0.0741	1.13E-07	chr:3:195654394
3	rs370983839	195668043	C	0.03364	1.28E-07	chr:3:195654394
3	rs7610260	195682526	A	0.09585	1.42E-07	chr:3:195654394
3	rs4927706	195771722	G	0.08023	1.46E-07	chr:3:195654394
3	rs2044599	195736740	G	0.09302	1.55E-07	chr:3:195654394
3	rs4927704	195738228	C	0.09302	1.55E-07	chr:3:195654394
3	rs4927848	195738406	A	0.09302	1.55E-07	chr:3:195654394
3	rs79088388	195812194	T	0.08549	1.55E-07	chr:3:195654394
3	rs10881563	195750225	A	0.09283	1.65E-07	chr:3:195654394
3	rs4632575	195744978	A	0.09283	1.65E-07	chr:3:195654394
3	rs1679296	195732647	A	0.09813	1.69E-07	chr:3:195654394
3	rs6799572	195740408	C	0.09244	1.71E-07	chr:3:195654394
3	rs62285392	195824420	C	0.08613	1.74E-07	chr:3:195654394
3	rs62285393	195824554	T	0.08613	1.74E-07	chr:3:195654394
3	rs9834211	195743762	A	0.09283	1.77E-07	chr:3:195654394
3	rs10881564	195750326	C	0.09224	1.80E-07	chr:3:195654394
3	rs7624638	195748729	C	0.09224	1.80E-07	chr:3:195654394
3	rs7624831	195748924	C	0.09224	1.80E-07	chr:3:195654394
3	rs7625570	195747398	A	0.09224	1.80E-07	chr:3:195654394
3	rs7627407	195749357	C	0.09224	1.80E-07	chr:3:195654394
3	rs201898153	195697848	C	0.0515	1.86E-07	chr:3:195654394
3	rs2300779	195790691	A	0.08793	1.92E-07	chr:3:195654394
3	rs3761716	195798097	G	0.08793	1.92E-07	chr:3:195654394

3	rs3804138	195807688	C	0.08793	1.92E-07	chr:3:195654394
3	rs3804141	195796908	T	0.08793	1.92E-07	chr:3:195654394
3	rs3827556	195796574	T	0.08793	1.92E-07	chr:3:195654394
3	rs41299394	195775824	T	0.08793	1.92E-07	chr:3:195654394
3	rs41300427	195804449	T	0.08793	1.92E-07	chr:3:195654394
3	rs41301353	195801426	A	0.08793	1.92E-07	chr:3:195654394
3	rs570	195776240	T	0.08793	1.92E-07	chr:3:195654394
3	rs62284053	195783481	T	0.08793	1.92E-07	chr:3:195654394
3	rs62285381	195806641	T	0.08793	1.92E-07	chr:3:195654394
3	rs6763092	195808024	G	0.08793	1.92E-07	chr:3:195654394
3	rs7645812	195781325	T	0.08793	1.92E-07	chr:3:195654394
3	rs1004860	195820247	A	0.08879	1.97E-07	chr:3:195654394
3	rs1004992	195821666	A	0.08879	1.97E-07	chr:3:195654394
3	rs12491771	195821441	G	0.08879	1.97E-07	chr:3:195654394
3	rs62285382	195813920	T	0.08879	1.97E-07	chr:3:195654394
3	rs62285387	195819505	A	0.08879	1.97E-07	chr:3:195654394
3	rs41259844	195781684	T	0.09033	2.17E-07	chr:3:195654394
3	rs41297523	195784648	C	0.1183	2.20E-07	chr:3:195654394
3	rs202025333	195695717	G	0.05005	2.26E-07	chr:3:195654394
3	rs4927792	195652315	C	0.08185	2.27E-07	chr:3:195654394
3	rs374823901	195685831	A	0.06181	2.49E-07	chr:3:195654394
3	rs376696582	195685832	G	0.06181	2.49E-07	chr:3:195654394
3	rs4927817	195693798	G	0.05098	2.82E-07	chr:3:195654394
3	rs13093426	195828806	T	0.0918	3.26E-07	chr:3:195654394
3	rs78219813	195831188	G	0.0918	3.26E-07	chr:3:195654394
3	rs12493933	195818769	T	0.09551	3.48E-07	chr:3:195654394
3	rs62285403	195834947	C	0.122	3.55E-07	chr:3:195654394
3	rs544737559	195825972	C	0.1028	3.76E-07	chr:3:195654394
3	rs78758916	195691752	G	0.07957	3.76E-07	chr:3:195654394
3	rs12490842	195816783	T	0.09655	3.77E-07	chr:3:195654394
3	rs200007926	195682077	T	0.07134	3.86E-07	chr:3:195654394
3	rs200191712	195682112	A	0.07134	3.86E-07	chr:3:195654394
3	rs201335223	195682102	T	0.07134	3.86E-07	chr:3:195654394
3	rs201963939	195682106	C	0.07134	3.86E-07	chr:3:195654394
3	rs557551051	195682108	T	0.07134	3.86E-07	chr:3:195654394
3	rs1975582	195751439	G	0.1009	3.93E-07	chr:3:195654394
3	rs4361313	195752260	A	0.1009	3.93E-07	chr:3:195654394
3	rs4927851	195751853	A	0.1009	3.93E-07	chr:3:195654394
3	rs7614767	195753451	G	0.1009	3.93E-07	chr:3:195654394
3	rs7627706	195753353	A	0.1009	3.93E-07	chr:3:195654394
3	rs7627868	195753473	A	0.1009	3.93E-07	chr:3:195654394
3	rs7630489	195753401	T	0.1009	3.93E-07	chr:3:195654394
3	rs7630875	195753766	T	0.1009	3.93E-07	chr:3:195654394

3	rs6800500	195833507	T	0.0795	4.31E-07	chr:3:195654394
3	rs9872347	195831237	T	0.0795	4.31E-07	chr:3:195654394
3	rs9877493	195832147	T	0.0795	4.31E-07	chr:3:195654394
3	rs7618864	195749561	G	0.1024	4.60E-07	chr:3:195654394
3	rs7624460	195748530	C	0.1024	4.60E-07	chr:3:195654394
3	rs2121818	195744988	G	0.1131	4.65E-07	chr:3:195654394
3	rs115223322	195733933	A	0.06151	4.73E-07	chr:3:195654394
3	rs147852061	195733925	C	0.06151	4.73E-07	chr:3:195654394
3	rs11709120	195671861	T	0.1339	5.76E-07	chr:3:195654394
3	rs12487702	195775991	A	0.1378	6.10E-07	chr:3:195654394
3	rs4927850	195751630	T	0.1019	6.50E-07	chr:3:195654394
3	rs2620600	195708880	C	23.93	6.74E-07	chr:3:195654394
3	rs3933	195784997	A	0.1075	6.81E-07	chr:3:195654394
3	rs41298097	195779155	T	0.1075	6.81E-07	chr:3:195654394
3	rs492288	195784210	T	0.1075	6.81E-07	chr:3:195654394
3	rs492349	195784192	A	0.1075	6.81E-07	chr:3:195654394
3	rs493661	195786489	A	0.1075	6.81E-07	chr:3:195654394
3	rs539816	195784076	A	0.1075	6.81E-07	chr:3:195654394
3	rs366268	195782177	G	0.1075	7.01E-07	chr:3:195654394
3	rs419059	195782184	T	0.1075	7.01E-07	chr:3:195654394
3	rs419068	195782176	T	0.1075	7.01E-07	chr:3:195654394
3	rs557527	195780709	A	0.1073	7.12E-07	chr:3:195654394
3	rs58381328	195442180	C	14.25	7.33E-07	chr:3:195654394
3	rs28427561	195733814	G	0.1136	7.39E-07	chr:3:195654394
3	rs406271	195776976	C	0.1117	8.38E-07	chr:3:195654394
3	rs12489052	195673049	G	0.1214	9.42E-07	chr:3:195654394
3	rs6772320	195774541	A	0.1809	1.02E-06	chr:3:195654394
3	rs7630825	195753740	T	0.1126	1.08E-06	chr:3:195654394
3	rs11925423	195734217	G	0.1247	1.95E-06	chr:3:195654394
3	rs34906439	195779706	G	0.1306	2.03E-06	chr:3:195654394
3	rs41298087	195779736	T	0.1306	2.03E-06	chr:3:195654394
3	rs62408943	195838810	G	0.1246	2.03E-06	chr:3:195654394
3	rs2638914	195663197	A	0.07859	2.20E-06	chr:3:195654394
3	rs4927711	195775449	T	0.09695	2.39E-06	chr:3:195654394
3	rs4927870	195820576	A	0.1684	2.95E-06	chr:3:195654394
3	rs191361199	195825973	G	0.1421	3.10E-06	chr:3:195654394
3	rs9817514	195775038	G	0.1373	3.33E-06	chr:3:195654394
3	rs9816784	195834303	T	0.1442	3.63E-06	chr:3:195654394
3	rs73082696	195414194	T	10.78	3.64E-06	chr:3:195654394
3	rs200021430	195684953	G	0.1064	3.69E-06	chr:3:195654394
3	rs201067908	195684954	G	0.1064	3.69E-06	chr:3:195654394
3	rs35653288	195684958	A	0.1064	3.69E-06	chr:3:195654394
3	rs71207509	195830355	C	0.1873	3.82E-06	chr:3:195654394

3	rs79368819	195720855	G	0.1685	4.08E-06	chr:3:195654394
3	rs11717368	195834357	G	0.149	4.86E-06	chr:3:195654394
3	rs28512347	195733884	C	0.1565	5.69E-06	chr:3:195654394
3	rs28594263	195733883	C	0.1565	5.69E-06	chr:3:195654394
3	rs73072305	195830690	T	0.1715	7.23E-06	chr:3:195654394
3	rs9878952	195410304	C	9.286	8.49E-06	chr:3:195654394
3	rs12490036	195816195	T	0.1643	9.43E-06	chr:3:195654394
3	rs139965737	195678158	G	0.1219	1.20E-05	chr:3:195654394
3	rs9866168	195830310	T	8.338	1.29E-05	chr:3:195654394
3	rs62408944	195843979	C	0.1874	1.48E-05	chr:3:195654394
3	rs150999632	195619120	C	0.1455	1.54E-05	chr:3:195654394
3	rs6769547	195416501	G	8.738	1.79E-05	chr:3:195654394
3	rs1965192	195852427	A	0.1866	2.59E-05	chr:3:195654394
3	rs60148746	195823008	T	0.1904	2.70E-05	chr:3:195654394
3	rs6778081	195819205	T	0.1853	2.78E-05	chr:3:195654394
3	rs6774560	195818126	G	0.1911	3.72E-05	chr:3:195654394
3	rs6784311	195733721	C	0.1176	3.88E-05	chr:3:195654394
3	rs12497208	195672011	A	0.09361	4.71E-05	chr:3:195654394
3	rs56134127	195646976	T	9.952	5.01E-05	chr:3:195654394
4	rs143457155	120263622	C	0.1072	6.90E-17	chr:4:120263688
4	rs10006688	120263526	C	0.1125	7.12E-17	chr:4:120263688
4	rs28622913	120261723	A	0.1048	7.72E-17	chr:4:120263688
4	rs28735977	120261738	T	0.1063	9.99E-17	chr:4:120263688
4	rs6833140	120266822	A	0.1003	1.37E-16	chr:4:120263688
4	rs13122153	120266585	C	0.1098	2.44E-16	chr:4:120263688
4	rs3864142	120266191	T	0.1037	2.44E-16	chr:4:120263688
4	rs13121928	120266518	C	0.1157	2.47E-16	chr:4:120263688
4	rs148893841	120263621	G	0.1055	2.70E-16	chr:4:120263688
4	rs28691602	120264770	C	0.1055	2.70E-16	chr:4:120263688
4	rs10000544	120270705	A	0.1057	2.75E-16	chr:4:120263688
4	rs10005542	120268302	C	0.1057	2.75E-16	chr:4:120263688
4	rs10012408	120270771	A	0.1057	2.75E-16	chr:4:120263688
4	rs10015579	120271802	C	0.1057	2.75E-16	chr:4:120263688
4	rs10015965	120268237	G	0.1057	2.75E-16	chr:4:120263688
4	rs10016241	120268553	C	0.1057	2.75E-16	chr:4:120263688
4	rs10022448	120270421	G	0.1057	2.75E-16	chr:4:120263688
4	rs10025925	120271744	T	0.1057	2.75E-16	chr:4:120263688
4	rs12502393	120271228	T	0.1057	2.75E-16	chr:4:120263688
4	rs12502524	120271414	A	0.1057	2.75E-16	chr:4:120263688
4	rs12506421	120271167	C	0.1057	2.75E-16	chr:4:120263688
4	rs12506445	120271237	C	0.1057	2.75E-16	chr:4:120263688
4	rs12506487	120271361	G	0.1057	2.75E-16	chr:4:120263688
4	rs12510133	120271344	C	0.1057	2.75E-16	chr:4:120263688

4	rs17009144	120270795	G	0.1057	2.75E-16	chr:4:120263688
4	rs28369518	120271630	C	0.1057	2.75E-16	chr:4:120263688
4	rs28396837	120271541	A	0.1057	2.75E-16	chr:4:120263688
4	rs28572003	120268891	G	0.1057	2.75E-16	chr:4:120263688
4	rs28589345	120269080	A	0.1057	2.75E-16	chr:4:120263688
4	rs28599836	120268809	T	0.1057	2.75E-16	chr:4:120263688
4	rs28714087	120270966	G	0.1057	2.75E-16	chr:4:120263688
4	rs3864144	120272605	T	0.1057	2.75E-16	chr:4:120263688
4	rs55820750	120269168	C	0.1057	2.75E-16	chr:4:120263688
4	rs10017001	120263293	G	0.1499	2.94E-16	chr:4:120263688
4	rs9994691	120263298	A	0.1499	2.94E-16	chr:4:120263688
4	rs12498657	120262866	C	0.1064	3.07E-16	chr:4:120263688
4	rs2175381	120265967	C	0.1104	3.25E-16	chr:4:120263688
4	rs10006192	120263022	C	0.1065	3.43E-16	chr:4:120263688
4	rs10010355	120261043	G	0.1065	3.43E-16	chr:4:120263688
4	rs10023641	120258410	G	0.1065	3.43E-16	chr:4:120263688
4	rs10028773	120265259	G	0.1065	3.43E-16	chr:4:120263688
4	rs114866537	120258608	T	0.1065	3.43E-16	chr:4:120263688
4	rs17517414	120262101	C	0.1065	3.43E-16	chr:4:120263688
4	rs17595727	120262074	C	0.1065	3.43E-16	chr:4:120263688
4	rs1814814	120258124	G	0.1065	3.43E-16	chr:4:120263688
4	rs2136911	120265859	A	0.1065	3.43E-16	chr:4:120263688
4	rs28437684	120264849	A	0.1065	3.43E-16	chr:4:120263688
4	rs28546691	120262832	A	0.1065	3.43E-16	chr:4:120263688
4	rs28551750	120264901	T	0.1065	3.43E-16	chr:4:120263688
4	rs28616635	120261861	C	0.1065	3.43E-16	chr:4:120263688
4	rs28726222	120262672	C	0.1065	3.43E-16	chr:4:120263688
4	rs3986377	120260270	A	0.1065	3.43E-16	chr:4:120263688
4	rs4001390	120265783	C	0.1065	3.43E-16	chr:4:120263688
4	rs4473640	120260437	A	0.1065	3.43E-16	chr:4:120263688
4	rs6815291	120266982	A	0.1065	3.43E-16	chr:4:120263688
4	rs6815934	120267310	C	0.1065	3.43E-16	chr:4:120263688
4	rs6838009	120267034	A	0.1065	3.43E-16	chr:4:120263688
4	rs6838457	120267367	G	0.1065	3.43E-16	chr:4:120263688
4	rs79606892	120259606	C	0.1065	3.43E-16	chr:4:120263688
4	rs9307470	120261214	A	0.1065	3.43E-16	chr:4:120263688
4	rs9307471	120261262	A	0.1065	3.43E-16	chr:4:120263688
4	rs9995716	120264996	C	0.1065	3.43E-16	chr:4:120263688
4	rs11735886	120290215	C	0.1228	4.16E-16	chr:4:120263688
4	rs4422403	120258194	T	0.1134	4.71E-16	chr:4:120263688
4	rs28584388	120261788	C	0.1078	4.88E-16	chr:4:120263688
4	rs555773279	120269424	T	0.1107	7.59E-16	chr:4:120263688
4	rs1814813	120258207	G	0.1156	7.63E-16	chr:4:120263688

4	rs3864143	120266428	C	0.1396	9.28E-16	chr:4:120263688
4	rs190883674	120269780	G	0.1693	1.64E-15	chr:4:120263688
4	rs201764844	120269768	C	0.1693	1.64E-15	chr:4:120263688
4	rs77466269	120269755	A	0.1724	2.02E-15	chr:4:120263688
4	rs200947437	120254755	C	0.1625	2.10E-15	chr:4:120263688
4	rs75420021	120246729	A	0.1444	3.82E-15	chr:4:120263688
4	rs2389874	120554991	A	0.1494	4.18E-15	chr:4:120263688
4	rs10212714	120254302	C	0.143	4.18E-15	chr:4:120263688
4	rs10212719	120254437	C	0.143	4.18E-15	chr:4:120263688
4	rs10212956	120254343	G	0.143	4.18E-15	chr:4:120263688
4	rs10213480	120254561	T	0.143	4.18E-15	chr:4:120263688
4	rs72676046	120254681	C	0.143	4.18E-15	chr:4:120263688
4	rs10006495	120263315	G	0.178	4.73E-15	chr:4:120263688
4	rs1546503	120241167	C	0.144	4.75E-15	chr:4:120263688
4	rs1546506	120241240	C	0.144	4.75E-15	chr:4:120263688
4	rs10003567	120241674	T	0.1444	5.54E-15	chr:4:120263688
4	rs10005237	120237897	C	0.1444	5.54E-15	chr:4:120263688
4	rs10006259	120242145	T	0.1444	5.54E-15	chr:4:120263688
4	rs10024933	120253582	A	0.1444	5.54E-15	chr:4:120263688
4	rs10212775	120239244	C	0.1444	5.54E-15	chr:4:120263688
4	rs10213158	120255829	T	0.1444	5.54E-15	chr:4:120263688
4	rs10213267	120239074	G	0.1444	5.54E-15	chr:4:120263688
4	rs10518300	120249499	G	0.1444	5.54E-15	chr:4:120263688
4	rs11098501	120252017	G	0.1444	5.54E-15	chr:4:120263688
4	rs11098502	120252063	A	0.1444	5.54E-15	chr:4:120263688
4	rs11724409	120252361	G	0.1444	5.54E-15	chr:4:120263688
4	rs11732686	120252330	A	0.1444	5.54E-15	chr:4:120263688
4	rs12498994	120250818	C	0.1444	5.54E-15	chr:4:120263688
4	rs12506610	120241659	C	0.1444	5.54E-15	chr:4:120263688
4	rs12507565	120251121	A	0.1444	5.54E-15	chr:4:120263688
4	rs12509621	120249660	C	0.1444	5.54E-15	chr:4:120263688
4	rs12510269	120241646	G	0.1444	5.54E-15	chr:4:120263688
4	rs12511640	120251248	C	0.1444	5.54E-15	chr:4:120263688
4	rs12513083	120249612	T	0.1444	5.54E-15	chr:4:120263688
4	rs12711071	120240934	C	0.1444	5.54E-15	chr:4:120263688
4	rs1511017	120250805	A	0.1444	5.54E-15	chr:4:120263688
4	rs1511025	120240238	C	0.1444	5.54E-15	chr:4:120263688
4	rs17049949	120255290	G	0.1444	5.54E-15	chr:4:120263688
4	rs1980024	120253047	T	0.1444	5.54E-15	chr:4:120263688
4	rs1980025	120252806	T	0.1444	5.54E-15	chr:4:120263688
4	rs1980026	120251643	A	0.1444	5.54E-15	chr:4:120263688
4	rs2002047	120257228	G	0.1444	5.54E-15	chr:4:120263688
4	rs2002049	120257417	G	0.1444	5.54E-15	chr:4:120263688

4	rs2036856	120249288	A	0.1444	5.54E-15	chr:4:120263688
4	rs2036860	120248934	G	0.1444	5.54E-15	chr:4:120263688
4	rs28363929	120257103	G	0.1444	5.54E-15	chr:4:120263688
4	rs28502463	120257023	C	0.1444	5.54E-15	chr:4:120263688
4	rs28643450	120245242	A	0.1444	5.54E-15	chr:4:120263688
4	rs28652763	120242312	T	0.1444	5.54E-15	chr:4:120263688
4	rs28713555	120251995	T	0.1444	5.54E-15	chr:4:120263688
4	rs2964	120240131	C	0.1444	5.54E-15	chr:4:120263688
4	rs34425882	120253177	A	0.1444	5.54E-15	chr:4:120263688
4	rs3956464	120256764	C	0.1444	5.54E-15	chr:4:120263688
4	rs4107728	120253910	A	0.1444	5.54E-15	chr:4:120263688
4	rs4443261	120249301	C	0.1444	5.54E-15	chr:4:120263688
4	rs7672594	120248543	C	0.1444	5.54E-15	chr:4:120263688
4	rs7672778	120248585	G	0.1444	5.54E-15	chr:4:120263688
4	rs7689729	120256192	G	0.1444	5.54E-15	chr:4:120263688
4	rs7690338	120256468	G	0.1444	5.54E-15	chr:4:120263688
4	rs878372	120238780	C	0.1444	5.54E-15	chr:4:120263688
4	rs878376	120237702	A	0.1444	5.54E-15	chr:4:120263688
4	rs907204	120238654	A	0.1444	5.54E-15	chr:4:120263688
4	rs907205	120238664	C	0.1444	5.54E-15	chr:4:120263688
4	rs938056	120237453	T	0.1444	5.54E-15	chr:4:120263688
4	rs9991166	120237851	A	0.1444	5.54E-15	chr:4:120263688
4	rs9991959	120253773	C	0.1444	5.54E-15	chr:4:120263688
4	rs17595608	120250506	T	0.1479	5.86E-15	chr:4:120263688
4	rs7438634	120245754	C	0.1439	5.94E-15	chr:4:120263688
4	rs60378962	120280991	A	0.1459	7.11E-15	chr:4:120263688
4	rs12507283	120247218	A	0.1517	7.13E-15	chr:4:120263688
4	rs1849457	120254355	G	0.1502	7.28E-15	chr:4:120263688
4	rs28373117	120253465	T	0.145	7.98E-15	chr:4:120263688
4	rs58583086	120556362	A	0.1489	8.09E-15	chr:4:120263688
4	rs534745130	120246059	C	0.1563	8.16E-15	chr:4:120263688
4	rs11732087	120239831	G	0.1514	8.27E-15	chr:4:120263688
4	rs12503171	120247196	G	0.152	8.34E-15	chr:4:120263688
4	rs10005973	120271958	T	0.1545	8.48E-15	chr:4:120263688
4	rs13108589	120268102	C	0.1545	8.48E-15	chr:4:120263688
4	rs4472123	120236630	A	0.1479	8.65E-15	chr:4:120263688
4	rs140387830	120247916	G	0.1502	8.90E-15	chr:4:120263688
4	rs145027390	120247827	G	0.1502	8.90E-15	chr:4:120263688
4	rs7671797	120248157	G	0.1502	8.90E-15	chr:4:120263688
4	rs7671978	120248218	G	0.1502	8.90E-15	chr:4:120263688
4	rs7672129	120248176	T	0.1502	8.90E-15	chr:4:120263688
4	rs140584472	120246695	G	0.1615	9.29E-15	chr:4:120263688
4	rs185481756	120246689	C	0.1615	9.29E-15	chr:4:120263688

4	rs2036857	120249240	C	0.1509	9.62E-15	chr:4:120263688
4	rs7671759	120248094	C	0.1539	9.84E-15	chr:4:120263688
4	rs10434028	120294464	G	0.1425	1.02E-14	chr:4:120263688
4	rs7673476	120248683	A	0.1615	1.07E-14	chr:4:120263688
4	rs10213554	120260785	A	0.1556	1.08E-14	chr:4:120263688
4	rs1980027	120251577	T	0.1556	1.08E-14	chr:4:120263688
4	rs66900435	120249425	A	0.1556	1.08E-14	chr:4:120263688
4	rs714899	120243035	A	0.1556	1.08E-14	chr:4:120263688
4	rs9993347	120258955	G	0.1556	1.08E-14	chr:4:120263688
4	rs9999724	120239944	T	0.1556	1.08E-14	chr:4:120263688
4	rs7672357	120248340	T	0.1533	1.09E-14	chr:4:120263688
4	rs10002914	120292081	A	0.1501	1.10E-14	chr:4:120263688
4	rs10021601	120282563	A	0.1501	1.10E-14	chr:4:120263688
4	rs10029303	120286755	A	0.1501	1.10E-14	chr:4:120263688
4	rs10517834	120286783	C	0.1501	1.10E-14	chr:4:120263688
4	rs10857066	120286596	T	0.1501	1.10E-14	chr:4:120263688
4	rs11098505	120284627	T	0.1501	1.10E-14	chr:4:120263688
4	rs12374244	120281972	T	0.1501	1.10E-14	chr:4:120263688
4	rs12374245	120282059	T	0.1501	1.10E-14	chr:4:120263688
4	rs12374346	120281705	A	0.1501	1.10E-14	chr:4:120263688
4	rs12374352	120281977	T	0.1501	1.10E-14	chr:4:120263688
4	rs12374396	120281619	C	0.1501	1.10E-14	chr:4:120263688
4	rs12499602	120284387	A	0.1501	1.10E-14	chr:4:120263688
4	rs12503082	120284317	G	0.1501	1.10E-14	chr:4:120263688
4	rs12510451	120289143	T	0.1501	1.10E-14	chr:4:120263688
4	rs12510487	120289231	T	0.1501	1.10E-14	chr:4:120263688
4	rs12513310	120288039	A	0.1501	1.10E-14	chr:4:120263688
4	rs13104572	120285568	C	0.1501	1.10E-14	chr:4:120263688
4	rs13105020	120285688	T	0.1501	1.10E-14	chr:4:120263688
4	rs13125526	120288019	A	0.1501	1.10E-14	chr:4:120263688
4	rs13132082	120285657	A	0.1501	1.10E-14	chr:4:120263688
4	rs28470894	120289405	G	0.1501	1.10E-14	chr:4:120263688
4	rs28488261	120289396	C	0.1501	1.10E-14	chr:4:120263688
4	rs28501434	120283773	T	0.1501	1.10E-14	chr:4:120263688
4	rs28581362	120283548	A	0.1501	1.10E-14	chr:4:120263688
4	rs28594160	120289456	G	0.1501	1.10E-14	chr:4:120263688
4	rs28668103	120291372	T	0.1501	1.10E-14	chr:4:120263688
4	rs28687057	120280812	G	0.1501	1.10E-14	chr:4:120263688
4	rs55845118	120290913	C	0.1501	1.10E-14	chr:4:120263688
4	rs67281037	120281157	C	0.1501	1.10E-14	chr:4:120263688
4	rs6824111	120285968	A	0.1501	1.10E-14	chr:4:120263688
4	rs6824169	120293175	C	0.1501	1.10E-14	chr:4:120263688
4	rs6846966	120293208	G	0.1501	1.10E-14	chr:4:120263688

4	rs6847586	120286069	A	0.1501	1.10E-14	chr:4:120263688
4	rs6847778	120293582	G	0.1501	1.10E-14	chr:4:120263688
4	rs6847862	120286371	T	0.1501	1.10E-14	chr:4:120263688
4	rs6848081	120286443	T	0.1501	1.10E-14	chr:4:120263688
4	rs6848380	120286403	A	0.1501	1.10E-14	chr:4:120263688
4	rs6857892	120282696	T	0.1501	1.10E-14	chr:4:120263688
4	rs71610270	120287436	G	0.1501	1.10E-14	chr:4:120263688
4	rs72676059	120290828	G	0.1501	1.10E-14	chr:4:120263688
4	rs73842616	120290683	A	0.1501	1.10E-14	chr:4:120263688
4	rs7677068	120291704	T	0.1501	1.10E-14	chr:4:120263688
4	rs9991221	120292107	C	0.1501	1.10E-14	chr:4:120263688
4	rs9995136	120286845	G	0.1501	1.10E-14	chr:4:120263688
4	rs78422072	120281042	T	0.1515	1.20E-14	chr:4:120263688
4	rs78971550	120281041	T	0.1515	1.20E-14	chr:4:120263688
4	rs3864141	120266007	A	0.1582	1.26E-14	chr:4:120263688
4	rs13101722	120555975	C	0.1605	1.30E-14	chr:4:120263688
4	rs59867181	120556467	C	0.1605	1.30E-14	chr:4:120263688
4	rs10213221	120255926	A	0.1584	1.37E-14	chr:4:120263688
4	rs11098507	120288286	A	0.1519	1.42E-14	chr:4:120263688
4	rs28787668	120554687	A	0.1616	1.44E-14	chr:4:120263688
4	rs7676296	120555687	G	0.1616	1.44E-14	chr:4:120263688
4	rs35280960	120257059	A	0.1593	1.48E-14	chr:4:120263688
4	rs56155624	120291026	G	0.1569	1.49E-14	chr:4:120263688
4	rs7672372	120248406	G	0.1565	1.52E-14	chr:4:120263688
4	rs9996494	120238880	A	0.1617	1.65E-14	chr:4:120263688
4	rs9996644	120238877	T	0.1617	1.65E-14	chr:4:120263688
4	rs2389879	120557684	G	0.1626	1.78E-14	chr:4:120263688
4	rs6534149	120559292	T	0.1626	1.78E-14	chr:4:120263688
4	rs28369503	120238925	A	0.167	1.82E-14	chr:4:120263688
4	rs28458294	120238924	C	0.167	1.82E-14	chr:4:120263688
4	rs9996501	120238918	A	0.167	1.82E-14	chr:4:120263688
4	rs10050092	120532085	C	0.1636	1.92E-14	chr:4:120263688
4	rs6832740	120546136	C	0.1636	1.92E-14	chr:4:120263688
4	rs6855918	120546299	T	0.1636	1.92E-14	chr:4:120263688
4	rs7693919	120540571	A	0.1636	1.92E-14	chr:4:120263688
4	rs113263911	120255134	T	0.1759	2.04E-14	chr:4:120263688
4	rs28850368	120554313	A	0.1649	2.36E-14	chr:4:120263688
4	rs34835603	120553428	T	0.1649	2.36E-14	chr:4:120263688
4	rs55777923	120553264	T	0.1739	2.65E-14	chr:4:120263688
4	rs4145952	120155806	A	0.1895	2.65E-14	chr:4:120263688
4	rs12507387	120247590	C	0.1507	2.88E-14	chr:4:120263688
4	rs11098534	120556772	G	0.1678	3.39E-14	chr:4:120263688
4	rs74693727	120330155	T	0.1616	3.62E-14	chr:4:120263688

4	rs10013652	120292256	G	0.1708	3.73E-14	chr:4:120263688
4	rs6820115	120398182	C	0.169	3.91E-14	chr:4:120263688
4	rs7692994	120427489	A	0.1655	4.12E-14	chr:4:120263688
4	rs10020256	120287265	T	0.1587	4.83E-14	chr:4:120263688
4	rs200290730	120287492	A	0.1587	4.83E-14	chr:4:120263688
4	rs34481394	120248374	T	0.1741	5.30E-14	chr:4:120263688
4	rs35231872	120248376	T	0.1741	5.30E-14	chr:4:120263688
4	rs35653026	120248378	G	0.1741	5.30E-14	chr:4:120263688
4	rs3806808	120551085	C	0.1714	6.78E-14	chr:4:120263688
4	rs201965045	120254952	G	0.1808	6.87E-14	chr:4:120263688
4	rs12503360	120247506	C	0.1781	7.61E-14	chr:4:120263688
4	rs10009566	120558608	C	0.1744	9.68E-14	chr:4:120263688
4	rs10012252	120559139	C	0.1744	9.68E-14	chr:4:120263688
4	rs2389880	120559870	A	0.1744	9.68E-14	chr:4:120263688
4	rs13134132	120324615	A	0.1834	1.41E-13	chr:4:120263688
4	rs10857068	120317549	G	0.1725	1.63E-13	chr:4:120263688
4	rs11098514	120317374	C	0.1725	1.63E-13	chr:4:120263688
4	rs13116768	120318254	C	0.1725	1.63E-13	chr:4:120263688
4	rs28572238	120316686	G	0.1725	1.63E-13	chr:4:120263688
4	rs28714195	120316950	T	0.1725	1.63E-13	chr:4:120263688
4	rs72676074	120359841	C	0.1739	1.84E-13	chr:4:120263688
4	rs9307475	120368556	A	0.19	1.86E-13	chr:4:120263688
4	rs4563482	120354480	G	0.1881	1.89E-13	chr:4:120263688
4	rs75199754	120365198	A	0.1765	1.98E-13	chr:4:120263688
4	rs35239065	120325130	C	0.1745	2.13E-13	chr:4:120263688
4	rs35265692	120325135	C	0.1745	2.13E-13	chr:4:120263688
4	rs56838032	120329894	A	0.2025	2.17E-13	chr:4:120263688
4	rs72918579	120333222	C	0.1762	2.25E-13	chr:4:120263688
4	rs55661135	120506153	G	0.1906	2.31E-13	chr:4:120263688
4	rs201664317	120330379	C	0.178	2.35E-13	chr:4:120263688
4	rs7695996	120322033	T	0.1795	2.73E-13	chr:4:120263688
4	rs11098513	120316075	G	0.175	2.77E-13	chr:4:120263688
4	rs9996940	120364542	T	0.1929	2.98E-13	chr:4:120263688
4	rs34954036	120366927	A	0.1792	3.04E-13	chr:4:120263688
4	rs10013032	120149419	C	0.2075	3.09E-13	chr:4:120263688
4	rs4833612	120147596	A	0.2075	3.09E-13	chr:4:120263688
4	rs2203039	120132347	T	0.201	3.13E-13	chr:4:120263688
4	rs10014719	120320715	A	0.1797	3.13E-13	chr:4:120263688
4	rs10016944	120320894	A	0.1797	3.13E-13	chr:4:120263688
4	rs10017543	120321420	A	0.1797	3.13E-13	chr:4:120263688
4	rs12504149	120318577	A	0.1797	3.13E-13	chr:4:120263688
4	rs12508173	120318526	C	0.1797	3.13E-13	chr:4:120263688
4	rs12509234	120319434	C	0.1797	3.13E-13	chr:4:120263688

4	rs12511625	120318135	G	0.1798	3.24E-13	chr:4:120263688
4	rs10002083	120311152	T	0.18	3.24E-13	chr:4:120263688
4	rs10005644	120298477	C	0.18	3.24E-13	chr:4:120263688
4	rs10010801	120310028	C	0.18	3.24E-13	chr:4:120263688
4	rs10011097	120310359	G	0.18	3.24E-13	chr:4:120263688
4	rs10014748	120300988	G	0.18	3.24E-13	chr:4:120263688
4	rs10014845	120301077	G	0.18	3.24E-13	chr:4:120263688
4	rs10016060	120298412	G	0.18	3.24E-13	chr:4:120263688
4	rs10016448	120298845	G	0.18	3.24E-13	chr:4:120263688
4	rs10024844	120311528	G	0.18	3.24E-13	chr:4:120263688
4	rs10025054	120311697	C	0.18	3.24E-13	chr:4:120263688
4	rs10027498	120311987	C	0.18	3.24E-13	chr:4:120263688
4	rs10027856	120304874	A	0.18	3.24E-13	chr:4:120263688
4	rs10027976	120305042	T	0.18	3.24E-13	chr:4:120263688
4	rs10222798	120302419	T	0.18	3.24E-13	chr:4:120263688
4	rs10440343	120309787	C	0.18	3.24E-13	chr:4:120263688
4	rs10440402	120307813	G	0.18	3.24E-13	chr:4:120263688
4	rs11098508	120306607	G	0.18	3.24E-13	chr:4:120263688
4	rs11098509	120306670	C	0.18	3.24E-13	chr:4:120263688
4	rs11098510	120306793	T	0.18	3.24E-13	chr:4:120263688
4	rs1112817	120297800	C	0.18	3.24E-13	chr:4:120263688
4	rs114019672	120306475	G	0.18	3.24E-13	chr:4:120263688
4	rs11726097	120301499	T	0.18	3.24E-13	chr:4:120263688
4	rs11729050	120300066	C	0.18	3.24E-13	chr:4:120263688
4	rs12498908	120308261	A	0.18	3.24E-13	chr:4:120263688
4	rs12506452	120308083	C	0.18	3.24E-13	chr:4:120263688
4	rs13113483	120309039	T	0.18	3.24E-13	chr:4:120263688
4	rs13127114	120314664	C	0.18	3.24E-13	chr:4:120263688
4	rs13151285	120314741	A	0.18	3.24E-13	chr:4:120263688
4	rs138611376	120306434	G	0.18	3.24E-13	chr:4:120263688
4	rs140816585	120306228	C	0.18	3.24E-13	chr:4:120263688
4	rs146523888	120308804	G	0.18	3.24E-13	chr:4:120263688
4	rs150376940	120305404	A	0.18	3.24E-13	chr:4:120263688
4	rs28429722	120300093	A	0.18	3.24E-13	chr:4:120263688
4	rs28668716	120309875	C	0.18	3.24E-13	chr:4:120263688
4	rs28751034	120307097	A	0.18	3.24E-13	chr:4:120263688
4	rs28793658	120307214	C	0.18	3.24E-13	chr:4:120263688
4	rs28812279	120304152	C	0.18	3.24E-13	chr:4:120263688
4	rs28884220	120307211	G	0.18	3.24E-13	chr:4:120263688
4	rs4309825	120314881	G	0.18	3.24E-13	chr:4:120263688
4	rs4345162	120312959	A	0.18	3.24E-13	chr:4:120263688
4	rs4373140	120307698	G	0.18	3.24E-13	chr:4:120263688
4	rs4463052	120313258	C	0.18	3.24E-13	chr:4:120263688

4	rs4507344	120307485	A	0.18	3.24E-13	chr:4:120263688
4	rs4604043	120314921	T	0.18	3.24E-13	chr:4:120263688
4	rs59866101	120296591	G	0.18	3.24E-13	chr:4:120263688
4	rs6832410	120296800	G	0.18	3.24E-13	chr:4:120263688
4	rs6838617	120297423	T	0.18	3.24E-13	chr:4:120263688
4	rs6858383	120296772	C	0.18	3.24E-13	chr:4:120263688
4	rs7437420	120312903	G	0.18	3.24E-13	chr:4:120263688
4	rs7658181	120310873	G	0.18	3.24E-13	chr:4:120263688
4	rs7674500	120303593	T	0.18	3.24E-13	chr:4:120263688
4	rs7674544	120303714	G	0.18	3.24E-13	chr:4:120263688
4	rs7677836	120310638	A	0.18	3.24E-13	chr:4:120263688
4	rs7678238	120310832	C	0.18	3.24E-13	chr:4:120263688
4	rs76990001	120302639	T	0.18	3.24E-13	chr:4:120263688
4	rs78036070	120302802	A	0.18	3.24E-13	chr:4:120263688
4	rs79704848	120309351	A	0.18	3.24E-13	chr:4:120263688
4	rs9307473	120300221	T	0.18	3.24E-13	chr:4:120263688
4	rs9307474	120302496	A	0.18	3.24E-13	chr:4:120263688
4	rs9994889	120305056	G	0.18	3.24E-13	chr:4:120263688
4	rs9996417	120295862	C	0.18	3.24E-13	chr:4:120263688
4	rs9996569	120299004	C	0.18	3.24E-13	chr:4:120263688
4	rs9996677	120299148	A	0.18	3.24E-13	chr:4:120263688
4	rs9998953	120296169	C	0.18	3.24E-13	chr:4:120263688
4	rs12648182	120503891	T	0.1939	3.27E-13	chr:4:120263688
4	rs11098516	120317978	C	0.1809	3.34E-13	chr:4:120263688
4	rs6829903	120506884	T	0.1837	3.37E-13	chr:4:120263688
4	rs28408407	120376030	G	0.181	3.44E-13	chr:4:120263688
4	rs28587175	120373172	C	0.181	3.44E-13	chr:4:120263688
4	rs72918577	120326701	A	0.181	3.44E-13	chr:4:120263688
4	rs72918584	120362445	C	0.181	3.44E-13	chr:4:120263688
4	rs9998174	120366312	G	0.181	3.44E-13	chr:4:120263688
4	rs7655048	120328284	A	0.1881	3.55E-13	chr:4:120263688
4	rs10013042	120244482	T	0.2049	3.83E-13	chr:4:120263688
4	rs13101526	120244456	T	0.2049	3.83E-13	chr:4:120263688
4	rs10031718	120295892	T	0.1797	3.86E-13	chr:4:120263688
4	rs10518329	120401835	G	0.1952	4.10E-13	chr:4:120263688
4	rs7665125	120402079	T	0.1952	4.10E-13	chr:4:120263688
4	rs10019522	120364728	G	0.183	4.30E-13	chr:4:120263688
4	rs149826784	120330643	A	0.183	4.30E-13	chr:4:120263688
4	rs9683604	120363592	T	0.183	4.30E-13	chr:4:120263688
4	rs9683609	120363692	G	0.183	4.30E-13	chr:4:120263688
4	rs9684938	120363595	G	0.183	4.30E-13	chr:4:120263688
4	rs192039339	120333191	C	0.1835	4.32E-13	chr:4:120263688
4	rs6851130	120133712	G	0.2079	4.33E-13	chr:4:120263688

4	rs202150896	120367650	T	0.1831	4.39E-13	chr:4:120263688
4	rs9760442	120367951	G	0.1831	4.39E-13	chr:4:120263688
4	rs550069983;rs77653089	120359650	G	0.1827	4.46E-13	chr:4:120263688
4	rs4296662	120313440	G	0.1852	4.50E-13	chr:4:120263688
4	rs4560394	120313435	G	0.1852	4.50E-13	chr:4:120263688
4	rs2127823	120485670	T	0.188	4.57E-13	chr:4:120263688
4	rs2322021	120295030	A	0.1857	4.60E-13	chr:4:120263688
4	rs71614437	120370156	C	0.1839	4.65E-13	chr:4:120263688
4	rs11722183	120280597	C	0.1823	4.69E-13	chr:4:120263688
4	rs4001305	120359236	T	0.1842	4.74E-13	chr:4:120263688
4	rs951570	120150467	T	0.2095	4.74E-13	chr:4:120263688
4	rs187271184	120330448	A	0.1827	4.82E-13	chr:4:120263688
4	rs190803766	120330449	A	0.1827	4.82E-13	chr:4:120263688
4	rs12502423	120424172	C	0.1857	4.86E-13	chr:4:120263688
4	rs28441656	120376115	G	0.1857	4.86E-13	chr:4:120263688
4	rs28571712	120375980	A	0.1857	4.86E-13	chr:4:120263688
4	rs370222454	120359691	G	0.1855	4.89E-13	chr:4:120263688
4	rs7441137	120133221	G	0.2099	4.97E-13	chr:4:120263688
4	rs9884728	120127079	C	0.2099	4.97E-13	chr:4:120263688
4	rs1546504	120241179	T	0.1833	5.09E-13	chr:4:120263688
4	rs11723757	120299669	T	0.1811	5.12E-13	chr:4:120263688
4	rs10018726	120394010	G	0.1851	5.14E-13	chr:4:120263688
4	rs10020027	120381879	C	0.1851	5.14E-13	chr:4:120263688
4	rs10034623	120397829	A	0.1851	5.14E-13	chr:4:120263688
4	rs13107475	120393011	T	0.1851	5.14E-13	chr:4:120263688
4	rs1809406	120377122	A	0.1851	5.14E-13	chr:4:120263688
4	rs2389803	120393511	A	0.1851	5.14E-13	chr:4:120263688
4	rs2389809	120377399	C	0.1851	5.14E-13	chr:4:120263688
4	rs3733520	120423480	G	0.1851	5.14E-13	chr:4:120263688
4	rs7356491	120381974	T	0.1851	5.14E-13	chr:4:120263688
4	rs9994810	120381590	G	0.1851	5.14E-13	chr:4:120263688
4	rs10008386	120318809	A	0.1857	5.39E-13	chr:4:120263688
4	rs10008392	120318839	A	0.1857	5.39E-13	chr:4:120263688
4	rs1155577	120449810	C	0.1982	5.45E-13	chr:4:120263688
4	rs13104219	120438174	T	0.1982	5.45E-13	chr:4:120263688
4	rs140982995	120455483	T	0.1982	5.45E-13	chr:4:120263688
4	rs142328963	120498647	T	0.1982	5.45E-13	chr:4:120263688
4	rs1480933	120433248	T	0.1982	5.45E-13	chr:4:120263688
4	rs2892869	120521297	T	0.1982	5.45E-13	chr:4:120263688
4	rs3775842	120427732	A	0.1982	5.45E-13	chr:4:120263688
4	rs56173225	120509972	G	0.1982	5.45E-13	chr:4:120263688
4	rs6844263	120433807	G	0.1982	5.45E-13	chr:4:120263688
4	rs72920701	120513230	G	0.1982	5.45E-13	chr:4:120263688

4	rs7678973	120511817	T	0.1982	5.45E-13	chr:4:120263688
4	rs12506148	120320348	T	0.1824	5.57E-13	chr:4:120263688
4	rs146743180	120497433	C	0.2023	5.71E-13	chr:4:120263688
4	rs36173322	120496491	C	0.2023	5.71E-13	chr:4:120263688
4	rs59516282	120502016	G	0.2023	5.71E-13	chr:4:120263688
4	rs80052400	120498134	G	0.2023	5.71E-13	chr:4:120263688
4	rs1552092	120488496	T	0.1876	5.75E-13	chr:4:120263688
4	rs4001207	120331782	C	0.1854	5.89E-13	chr:4:120263688
4	rs10222794	120302351	T	0.1862	5.90E-13	chr:4:120263688
4	rs10222801	120302350	T	0.1862	5.90E-13	chr:4:120263688
4	rs9997340	120364809	C	0.1866	6.15E-13	chr:4:120263688
4	rs11729521	120416788	C	0.1859	6.18E-13	chr:4:120263688
4	rs10440403	120308047	T	0.1872	6.21E-13	chr:4:120263688
4	rs10019406	120364619	G	0.1873	6.26E-13	chr:4:120263688
4	rs10020034	120294331	G	0.1873	6.26E-13	chr:4:120263688
4	rs10028669	120295197	T	0.1873	6.26E-13	chr:4:120263688
4	rs11098512	120314161	T	0.1873	6.26E-13	chr:4:120263688
4	rs11723839	120299673	A	0.1873	6.26E-13	chr:4:120263688
4	rs13435162	120304634	A	0.1873	6.26E-13	chr:4:120263688
4	rs200149630	120356941	C	0.1873	6.26E-13	chr:4:120263688
4	rs28551662	120309556	A	0.1873	6.26E-13	chr:4:120263688
4	rs34792640	120368627	A	0.1873	6.26E-13	chr:4:120263688
4	rs34913743	120324918	G	0.1873	6.26E-13	chr:4:120263688
4	rs35494005	120361142	C	0.1873	6.26E-13	chr:4:120263688
4	rs3890049	120326283	A	0.1873	6.26E-13	chr:4:120263688
4	rs3955380	120334410	A	0.1873	6.26E-13	chr:4:120263688
4	rs59394118	120295551	G	0.1873	6.26E-13	chr:4:120263688
4	rs67265404	120359270	T	0.1873	6.26E-13	chr:4:120263688
4	rs71610279	120326643	C	0.1873	6.26E-13	chr:4:120263688
4	rs71629403	120372567	T	0.1873	6.26E-13	chr:4:120263688
4	rs7674556	120303729	G	0.1873	6.26E-13	chr:4:120263688
4	rs7696465	120322114	A	0.1873	6.26E-13	chr:4:120263688
4	rs7696649	120322177	A	0.1873	6.26E-13	chr:4:120263688
4	rs7696857	120322321	A	0.1873	6.26E-13	chr:4:120263688
4	rs79446934	120326713	C	0.1873	6.26E-13	chr:4:120263688
4	rs9685784	120366008	T	0.1873	6.26E-13	chr:4:120263688
4	rs10004484	120442428	T	0.1902	6.31E-13	chr:4:120263688
4	rs10018280	120478139	G	0.1902	6.31E-13	chr:4:120263688
4	rs1155576	120450159	C	0.1902	6.31E-13	chr:4:120263688
4	rs17050695	120489527	T	0.1902	6.31E-13	chr:4:120263688
4	rs28735358	120482049	C	0.1902	6.31E-13	chr:4:120263688
4	rs3775858	120486028	G	0.1902	6.31E-13	chr:4:120263688
4	rs6534141	120485223	T	0.1902	6.31E-13	chr:4:120263688

4	rs6858592	120458692	A	0.1902	6.31E-13	chr:4:120263688
4	rs7680063	120479889	G	0.1902	6.31E-13	chr:4:120263688
4	rs9997631	120469995	G	0.1902	6.31E-13	chr:4:120263688
4	rs9759478	120367998	A	0.1836	6.33E-13	chr:4:120263688
4	rs7654611	120328304	G	0.1899	6.34E-13	chr:4:120263688
4	rs11731756	120478696	G	0.1906	6.38E-13	chr:4:120263688
4	rs11737395	120520704	G	0.1906	6.38E-13	chr:4:120263688
4	rs12498539	120468370	T	0.1906	6.38E-13	chr:4:120263688
4	rs201236087	120474546	T	0.1906	6.38E-13	chr:4:120263688
4	rs2170276	120485824	C	0.1906	6.38E-13	chr:4:120263688
4	rs2306457	120472839	A	0.1906	6.38E-13	chr:4:120263688
4	rs34308924	120481431	C	0.1906	6.38E-13	chr:4:120263688
4	rs3775854	120471971	T	0.1906	6.38E-13	chr:4:120263688
4	rs3822194	120471648	G	0.1906	6.38E-13	chr:4:120263688
4	rs6853998	120475860	A	0.1906	6.38E-13	chr:4:120263688
4	rs6858777	120475966	G	0.1906	6.38E-13	chr:4:120263688
4	rs7669520	120515278	C	0.1906	6.38E-13	chr:4:120263688
4	rs7680748	120479978	A	0.1906	6.38E-13	chr:4:120263688
4	rs9884402	120489982	G	0.1906	6.38E-13	chr:4:120263688
4	rs9995026	120490499	A	0.1906	6.38E-13	chr:4:120263688
4	rs114860385	120355584	C	0.1874	6.43E-13	chr:4:120263688
4	rs9996382	120151012	A	0.2129	6.51E-13	chr:4:120263688
4	rs28499576	120386677	A	0.194	6.62E-13	chr:4:120263688
4	rs10023569	120323316	T	0.1884	6.63E-13	chr:4:120263688
4	rs10518331	120323595	T	0.1884	6.63E-13	chr:4:120263688
4	rs115044484	120326887	G	0.1884	6.63E-13	chr:4:120263688
4	rs11934314	120376540	A	0.1884	6.63E-13	chr:4:120263688
4	rs13133522	120324424	T	0.1884	6.63E-13	chr:4:120263688
4	rs139379776	120332234	A	0.1884	6.63E-13	chr:4:120263688
4	rs140750089	120330539	A	0.1884	6.63E-13	chr:4:120263688
4	rs142609610	120327032	A	0.1884	6.63E-13	chr:4:120263688
4	rs149185281	120369277	A	0.1884	6.63E-13	chr:4:120263688
4	rs17009122	120362403	A	0.1884	6.63E-13	chr:4:120263688
4	rs200822665	120357250	A	0.1884	6.63E-13	chr:4:120263688
4	rs201742833	120356668	T	0.1884	6.63E-13	chr:4:120263688
4	rs2389802	120325732	A	0.1884	6.63E-13	chr:4:120263688
4	rs28394590	120373738	G	0.1884	6.63E-13	chr:4:120263688
4	rs28495013	120375831	C	0.1884	6.63E-13	chr:4:120263688
4	rs28529639	120373104	T	0.1884	6.63E-13	chr:4:120263688
4	rs28567426	120373354	G	0.1884	6.63E-13	chr:4:120263688
4	rs28626372	120373262	T	0.1884	6.63E-13	chr:4:120263688
4	rs28653183	120372276	G	0.1884	6.63E-13	chr:4:120263688
4	rs28714318	120375830	T	0.1884	6.63E-13	chr:4:120263688

4	rs34147052	120361591	C	0.1884	6.63E-13	chr:4:120263688
4	rs34378623	120368744	T	0.1884	6.63E-13	chr:4:120263688
4	rs34566984	120361270	A	0.1884	6.63E-13	chr:4:120263688
4	rs34965784	120361586	C	0.1884	6.63E-13	chr:4:120263688
4	rs35091806	120325529	A	0.1884	6.63E-13	chr:4:120263688
4	rs35111011	120366526	T	0.1884	6.63E-13	chr:4:120263688
4	rs35165976	120325630	A	0.1884	6.63E-13	chr:4:120263688
4	rs532415426	120359632	A	0.1884	6.63E-13	chr:4:120263688
4	rs545772518	120359643	G	0.1884	6.63E-13	chr:4:120263688
4	rs559457335	120359644	G	0.1884	6.63E-13	chr:4:120263688
4	rs563816604	120359627	G	0.1884	6.63E-13	chr:4:120263688
4	rs66506550	120371445	A	0.1884	6.63E-13	chr:4:120263688
4	rs6820376	120324476	G	0.1884	6.63E-13	chr:4:120263688
4	rs6845714	120326138	T	0.1884	6.63E-13	chr:4:120263688
4	rs6846442	120326323	A	0.1884	6.63E-13	chr:4:120263688
4	rs6846620	120326404	A	0.1884	6.63E-13	chr:4:120263688
4	rs71610280	120326648	G	0.1884	6.63E-13	chr:4:120263688
4	rs71612602	120332781	A	0.1884	6.63E-13	chr:4:120263688
4	rs71614422	120359340	A	0.1884	6.63E-13	chr:4:120263688
4	rs71614434	120368975	T	0.1884	6.63E-13	chr:4:120263688
4	rs71614438	120371252	A	0.1884	6.63E-13	chr:4:120263688
4	rs71629404	120372724	G	0.1884	6.63E-13	chr:4:120263688
4	rs72489565	120326819	C	0.1884	6.63E-13	chr:4:120263688
4	rs74772011	120332334	C	0.1884	6.63E-13	chr:4:120263688
4	rs75122014	120362426	C	0.1884	6.63E-13	chr:4:120263688
4	rs75396180	120375727	A	0.1884	6.63E-13	chr:4:120263688
4	rs75690486	120359788	A	0.1884	6.63E-13	chr:4:120263688
4	rs76525361	120359608	T	0.1884	6.63E-13	chr:4:120263688
4	rs7659501	120371552	T	0.1884	6.63E-13	chr:4:120263688
4	rs7659551	120371729	T	0.1884	6.63E-13	chr:4:120263688
4	rs7664458	120371967	G	0.1884	6.63E-13	chr:4:120263688
4	rs76651228	120359559	G	0.1884	6.63E-13	chr:4:120263688
4	rs77299866	120326716	T	0.1884	6.63E-13	chr:4:120263688
4	rs9683709	120365714	A	0.1884	6.63E-13	chr:4:120263688
4	rs9685777	120365965	A	0.1884	6.63E-13	chr:4:120263688
4	rs28479042	120386876	C	0.1916	6.67E-13	chr:4:120263688
4	rs34858317	120387110	C	0.1916	6.67E-13	chr:4:120263688
4	rs35063680	120387102	C	0.1916	6.67E-13	chr:4:120263688
4	rs71614448	120387026	A	0.1916	6.67E-13	chr:4:120263688
4	rs71614449	120387055	T	0.1916	6.67E-13	chr:4:120263688
4	rs34237553	120325273	C	0.1887	6.78E-13	chr:4:120263688
4	rs7665192	120247065	A	0.188	6.78E-13	chr:4:120263688
4	rs71610285	120329953	C	0.1442	6.87E-13	chr:4:120263688

4	rs142970247	120499656	T	0.2043	6.89E-13	chr:4:120263688
4	rs10026736	120384322	C	0.1862	7.00E-13	chr:4:120263688
4	rs17006190	120418838	G	0.1862	7.00E-13	chr:4:120263688
4	rs3733519	120423448	G	0.1862	7.00E-13	chr:4:120263688
4	rs10017371	120293776	G	0.1867	7.11E-13	chr:4:120263688
4	rs1002152	120273387	A	0.1867	7.11E-13	chr:4:120263688
4	rs10026436	120288904	A	0.1867	7.11E-13	chr:4:120263688
4	rs11729991	120292700	T	0.1867	7.11E-13	chr:4:120263688
4	rs13104396	120285649	T	0.1867	7.11E-13	chr:4:120263688
4	rs6848580	120286492	A	0.1867	7.11E-13	chr:4:120263688
4	rs1546505	120241224	G	0.1863	7.12E-13	chr:4:120263688
4	rs76541370	120498280	A	0.1998	7.15E-13	chr:4:120263688
4	rs2389873	120554714	A	0.2113	7.15E-13	chr:4:120263688
4	rs4834792	120555696	T	0.2113	7.15E-13	chr:4:120263688
4	rs11728616	120440676	A	0.1956	7.16E-13	chr:4:120263688
4	rs35111518	120440682	G	0.1956	7.16E-13	chr:4:120263688
4	rs79026312	120440677	A	0.1956	7.16E-13	chr:4:120263688
4	rs10006793	120390791	G	0.19	7.29E-13	chr:4:120263688
4	rs10031483	120422636	C	0.19	7.29E-13	chr:4:120263688
4	rs10031665	120422852	C	0.19	7.29E-13	chr:4:120263688
4	rs11098526	120390359	T	0.19	7.29E-13	chr:4:120263688
4	rs11098527	120399906	G	0.19	7.29E-13	chr:4:120263688
4	rs11934940	120389369	G	0.19	7.29E-13	chr:4:120263688
4	rs12499324	120393786	T	0.19	7.29E-13	chr:4:120263688
4	rs12505953	120385681	A	0.19	7.29E-13	chr:4:120263688
4	rs12510138	120423935	C	0.19	7.29E-13	chr:4:120263688
4	rs2306455	120421969	A	0.19	7.29E-13	chr:4:120263688
4	rs28685688	120420334	C	0.19	7.29E-13	chr:4:120263688
4	rs34004415	120381140	A	0.19	7.29E-13	chr:4:120263688
4	rs3733523	120423719	T	0.19	7.29E-13	chr:4:120263688
4	rs6842762	120398236	T	0.19	7.29E-13	chr:4:120263688
4	rs71614443	120379388	C	0.19	7.29E-13	chr:4:120263688
4	rs7687843	120421211	T	0.19	7.29E-13	chr:4:120263688
4	rs9994730	120381564	C	0.19	7.29E-13	chr:4:120263688
4	rs9998585	120396802	A	0.19	7.29E-13	chr:4:120263688
4	rs6849171	120409549	A	0.1885	7.45E-13	chr:4:120263688
4	rs55816173	120247014	T	0.2062	7.52E-13	chr:4:120263688
4	rs56843000	120516597	T	0.2035	7.69E-13	chr:4:120263688
4	rs7699346	120310542	T	0.1888	7.71E-13	chr:4:120263688
4	rs7654762	120328377	C	0.1902	8.27E-13	chr:4:120263688
4	rs116952757	120362969	C	0.1883	8.46E-13	chr:4:120263688
4	rs4001185	120376381	C	0.1906	8.59E-13	chr:4:120263688
4	rs6534130	120131339	A	0.2171	8.63E-13	chr:4:120263688

4	rs9995244	120321858	G	0.182	8.90E-13	chr:4:120263688
4	rs28549914	120322781	A	0.1905	8.98E-13	chr:4:120263688
4	rs4368585	120354482	G	0.1915	9.07E-13	chr:4:120263688
4	rs28655325	120372999	G	0.1891	9.08E-13	chr:4:120263688
4	rs12506546	120301618	C	0.188	9.09E-13	chr:4:120263688
4	rs199863129	120334013	T	0.188	9.09E-13	chr:4:120263688
4	rs7696875	120322358	T	0.188	9.09E-13	chr:4:120263688
4	rs10006877	120242793	T	0.189	9.22E-13	chr:4:120263688
4	rs1022145	120230979	G	0.189	9.22E-13	chr:4:120263688
4	rs12505469	120249585	T	0.189	9.22E-13	chr:4:120263688
4	rs1546502	120235898	G	0.189	9.22E-13	chr:4:120263688
4	rs2017057	120257711	G	0.189	9.22E-13	chr:4:120263688
4	rs6838236	120267229	G	0.189	9.22E-13	chr:4:120263688
4	rs878373	120237484	T	0.189	9.22E-13	chr:4:120263688
4	rs41464847	120532401	A	0.2049	9.31E-13	chr:4:120263688
4	rs59590064	120539753	T	0.2049	9.31E-13	chr:4:120263688
4	rs6817317	120542827	A	0.2049	9.31E-13	chr:4:120263688
4	rs7672519	120544112	C	0.2049	9.31E-13	chr:4:120263688
4	rs7681980	120533223	G	0.2049	9.31E-13	chr:4:120263688
4	rs58416902	120374639	G	0.1916	9.40E-13	chr:4:120263688
4	rs72676045	120254663	A	0.1873	9.48E-13	chr:4:120263688
4	rs3822195	120471660	T	0.1971	9.53E-13	chr:4:120263688
4	rs35138262	120361169	T	0.2134	9.66E-13	chr:4:120263688
4	rs3872807	120360251	A	0.2134	9.66E-13	chr:4:120263688
4	rs11934328	120376744	T	0.1913	9.91E-13	chr:4:120263688
4	rs10008459	120394231	C	0.1927	9.93E-13	chr:4:120263688
4	rs10009450	120391258	G	0.1927	9.93E-13	chr:4:120263688
4	rs10017389	120381412	A	0.1927	9.93E-13	chr:4:120263688
4	rs10026493	120384194	G	0.1927	9.93E-13	chr:4:120263688
4	rs10026516	120384253	A	0.1927	9.93E-13	chr:4:120263688
4	rs10026748	120384357	A	0.1927	9.93E-13	chr:4:120263688
4	rs10026952	120384564	A	0.1927	9.93E-13	chr:4:120263688
4	rs10032151	120391628	T	0.1927	9.93E-13	chr:4:120263688
4	rs10032158	120391632	T	0.1927	9.93E-13	chr:4:120263688
4	rs11098524	120390032	G	0.1927	9.93E-13	chr:4:120263688
4	rs11098525	120390152	A	0.1927	9.93E-13	chr:4:120263688
4	rs11941899	120382758	A	0.1927	9.93E-13	chr:4:120263688
4	rs11943877	120389047	T	0.1927	9.93E-13	chr:4:120263688
4	rs12502909	120377529	T	0.1927	9.93E-13	chr:4:120263688
4	rs148358770	120382262	C	0.1927	9.93E-13	chr:4:120263688
4	rs150657109	120377707	T	0.1927	9.93E-13	chr:4:120263688
4	rs17046116	120387259	A	0.1927	9.93E-13	chr:4:120263688
4	rs17046118	120387496	G	0.1927	9.93E-13	chr:4:120263688

4	rs185466280	120377740	T	0.1927	9.93E-13	chr:4:120263688
4	rs2127820	120394534	A	0.1927	9.93E-13	chr:4:120263688
4	rs2127821	120394535	C	0.1927	9.93E-13	chr:4:120263688
4	rs28444327	120387946	G	0.1927	9.93E-13	chr:4:120263688
4	rs28461559	120387932	A	0.1927	9.93E-13	chr:4:120263688
4	rs28665282	120384186	T	0.1927	9.93E-13	chr:4:120263688
4	rs2892848	120381341	T	0.1927	9.93E-13	chr:4:120263688
4	rs34858405	120381081	A	0.1927	9.93E-13	chr:4:120263688
4	rs4001190	120377619	A	0.1927	9.93E-13	chr:4:120263688
4	rs6837828	120392084	T	0.1927	9.93E-13	chr:4:120263688
4	rs6838814	120392443	A	0.1927	9.93E-13	chr:4:120263688
4	rs71614442	120379346	T	0.1927	9.93E-13	chr:4:120263688
4	rs7436506	120393769	C	0.1927	9.93E-13	chr:4:120263688
4	rs74602693	120377647	T	0.1927	9.93E-13	chr:4:120263688
4	rs7654529	120388230	A	0.1927	9.93E-13	chr:4:120263688
4	rs7654546	120388255	A	0.1927	9.93E-13	chr:4:120263688
4	rs7654587	120388406	T	0.1927	9.93E-13	chr:4:120263688
4	rs7659336	120385341	C	0.1927	9.93E-13	chr:4:120263688
4	rs7681214	120385320	A	0.1927	9.93E-13	chr:4:120263688
4	rs7681978	120383775	C	0.1927	9.93E-13	chr:4:120263688
4	rs7688613	120384803	C	0.1927	9.93E-13	chr:4:120263688
4	rs79870274	120382190	T	0.1927	9.93E-13	chr:4:120263688
4	rs9993199	120392873	A	0.1927	9.93E-13	chr:4:120263688
4	rs4001195	120329676	G	0.1896	1.04E-12	chr:4:120263688
4	rs10012456	120274039	T	0.1876	1.04E-12	chr:4:120263688
4	rs6847075	120286043	T	0.1876	1.04E-12	chr:4:120263688
4	rs2389875	120557014	G	0.2098	1.05E-12	chr:4:120263688
4	rs7655177	120328622	G	0.1926	1.05E-12	chr:4:120263688
4	rs1480931	120395809	A	0.1906	1.05E-12	chr:4:120263688
4	rs66887589	120509279	T	0.2004	1.06E-12	chr:4:120263688
4	rs10006706	120409152	C	0.1934	1.06E-12	chr:4:120263688
4	rs10518328	120401779	A	0.1934	1.06E-12	chr:4:120263688
4	rs11098530	120413154	T	0.1934	1.06E-12	chr:4:120263688
4	rs11734241	120416872	G	0.1934	1.06E-12	chr:4:120263688
4	rs11935596	120412457	C	0.1934	1.06E-12	chr:4:120263688
4	rs11940028	120411907	C	0.1934	1.06E-12	chr:4:120263688
4	rs12507964	120413061	A	0.1934	1.06E-12	chr:4:120263688
4	rs28753180	120405367	T	0.1934	1.06E-12	chr:4:120263688
4	rs28789446	120405366	C	0.1934	1.06E-12	chr:4:120263688
4	rs28845498	120405186	A	0.1934	1.06E-12	chr:4:120263688
4	rs4577559	120404043	G	0.1934	1.06E-12	chr:4:120263688
4	rs56270433	120406030	T	0.1934	1.06E-12	chr:4:120263688
4	rs6822679	120402702	C	0.1934	1.06E-12	chr:4:120263688

4	rs6834796	120414693	C	0.1934	1.06E-12	chr:4:120263688
4	rs6848389	120402622	C	0.1934	1.06E-12	chr:4:120263688
4	rs6849561	120409694	C	0.1934	1.06E-12	chr:4:120263688
4	rs7656252	120404268	C	0.1934	1.06E-12	chr:4:120263688
4	rs7681544	120411255	C	0.1934	1.06E-12	chr:4:120263688
4	rs10020141	120318870	C	0.193	1.08E-12	chr:4:120263688
4	rs6833815	120318921	A	0.193	1.08E-12	chr:4:120263688
4	rs10028423	120294909	T	0.1908	1.10E-12	chr:4:120263688
4	rs28491261	120294900	A	0.1908	1.10E-12	chr:4:120263688
4	rs80242894	120375752	A	0.1967	1.11E-12	chr:4:120263688
4	rs11723090	120490592	C	0.1919	1.12E-12	chr:4:120263688
4	rs4998488	120500841	C	0.1919	1.12E-12	chr:4:120263688
4	rs59732491	120489588	C	0.1919	1.12E-12	chr:4:120263688
4	rs71612595	120331428	T	0.1929	1.14E-12	chr:4:120263688
4	rs71612596	120331743	A	0.1929	1.14E-12	chr:4:120263688
4	rs71612597	120331845	C	0.1929	1.14E-12	chr:4:120263688
4	rs13139045	120168588	C	0.2172	1.14E-12	chr:4:120263688
4	rs10008791	120431469	C	0.1958	1.16E-12	chr:4:120263688
4	rs10009626	120470005	C	0.1958	1.16E-12	chr:4:120263688
4	rs10013305	120450424	A	0.1958	1.16E-12	chr:4:120263688
4	rs10030660	120436704	A	0.1958	1.16E-12	chr:4:120263688
4	rs10034450	120455649	G	0.1958	1.16E-12	chr:4:120263688
4	rs1010739	120463471	T	0.1958	1.16E-12	chr:4:120263688
4	rs1010740	120463409	C	0.1958	1.16E-12	chr:4:120263688
4	rs11098531	120465001	A	0.1958	1.16E-12	chr:4:120263688
4	rs11098532	120490726	A	0.1958	1.16E-12	chr:4:120263688
4	rs11736416	120431661	T	0.1958	1.16E-12	chr:4:120263688
4	rs11933966	120476715	A	0.1958	1.16E-12	chr:4:120263688
4	rs11947234	120474859	G	0.1958	1.16E-12	chr:4:120263688
4	rs12498599	120468503	T	0.1958	1.16E-12	chr:4:120263688
4	rs12502389	120454191	G	0.1958	1.16E-12	chr:4:120263688
4	rs13128602	120459366	G	0.1958	1.16E-12	chr:4:120263688
4	rs13134665	120426430	C	0.1958	1.16E-12	chr:4:120263688
4	rs13140391	120503437	G	0.1958	1.16E-12	chr:4:120263688
4	rs13140409	120503460	G	0.1958	1.16E-12	chr:4:120263688
4	rs13149407	120437825	G	0.1958	1.16E-12	chr:4:120263688
4	rs1383532	120434404	A	0.1958	1.16E-12	chr:4:120263688
4	rs1383533	120434576	A	0.1958	1.16E-12	chr:4:120263688
4	rs1480936	120462861	T	0.1958	1.16E-12	chr:4:120263688
4	rs1480939	120456927	C	0.1958	1.16E-12	chr:4:120263688
4	rs1480940	120457682	C	0.1958	1.16E-12	chr:4:120263688
4	rs1552095	120460306	C	0.1958	1.16E-12	chr:4:120263688
4	rs2086450	120496217	A	0.1958	1.16E-12	chr:4:120263688

4	rs2291185	120434833	A	0.1958	1.16E-12	chr:4:120263688
4	rs2306456	120472422	G	0.1958	1.16E-12	chr:4:120263688
4	rs28718422	120466304	A	0.1958	1.16E-12	chr:4:120263688
4	rs34868248	120442430	G	0.1958	1.16E-12	chr:4:120263688
4	rs36040693	120477616	G	0.1958	1.16E-12	chr:4:120263688
4	rs3733525	120447048	G	0.1958	1.16E-12	chr:4:120263688
4	rs3736115	120488703	A	0.1958	1.16E-12	chr:4:120263688
4	rs3775843	120427844	C	0.1958	1.16E-12	chr:4:120263688
4	rs3775844	120428033	A	0.1958	1.16E-12	chr:4:120263688
4	rs3775845	120432447	C	0.1958	1.16E-12	chr:4:120263688
4	rs3775847	120447642	C	0.1958	1.16E-12	chr:4:120263688
4	rs3775848	120447724	T	0.1958	1.16E-12	chr:4:120263688
4	rs3775849	120450908	C	0.1958	1.16E-12	chr:4:120263688
4	rs3775852	120454556	G	0.1958	1.16E-12	chr:4:120263688
4	rs3822190	120428098	A	0.1958	1.16E-12	chr:4:120263688
4	rs3822191	120428101	T	0.1958	1.16E-12	chr:4:120263688
4	rs4596225	120496444	G	0.1958	1.16E-12	chr:4:120263688
4	rs58452170	120459674	T	0.1958	1.16E-12	chr:4:120263688
4	rs6534139	120449456	A	0.1958	1.16E-12	chr:4:120263688
4	rs6534140	120455311	T	0.1958	1.16E-12	chr:4:120263688
4	rs6835635	120458867	C	0.1958	1.16E-12	chr:4:120263688
4	rs6847818	120469250	T	0.1958	1.16E-12	chr:4:120263688
4	rs7657849	120455494	C	0.1958	1.16E-12	chr:4:120263688
4	rs7664986	120429952	C	0.1958	1.16E-12	chr:4:120263688
4	rs7678400	120461957	A	0.1958	1.16E-12	chr:4:120263688
4	rs7688802	120451668	A	0.1958	1.16E-12	chr:4:120263688
4	rs7695620	120452776	A	0.1958	1.16E-12	chr:4:120263688
4	rs7699064	120428309	T	0.1958	1.16E-12	chr:4:120263688
4	rs71610282	120329093	C	0.194	1.17E-12	chr:4:120263688
4	rs7654700	120328125	A	0.194	1.17E-12	chr:4:120263688
4	rs7654984	120328520	G	0.194	1.17E-12	chr:4:120263688
4	rs7655723	120328666	A	0.194	1.17E-12	chr:4:120263688
4	rs79244813	120354847	C	0.1936	1.18E-12	chr:4:120263688
4	rs62322293	120330655	G	0.2145	1.19E-12	chr:4:120263688
4	rs73842633	120375464	A	0.1938	1.21E-12	chr:4:120263688
4	rs12505878	120385485	A	0.1938	1.22E-12	chr:4:120263688
4	rs7694483	120405929	A	0.2154	1.24E-12	chr:4:120263688
4	rs77934861	120324707	C	0.195	1.24E-12	chr:4:120263688
4	rs76568951	120355570	C	0.1949	1.25E-12	chr:4:120263688
4	rs150131449	120288605	G	0.1892	1.26E-12	chr:4:120263688
4	rs28374462	120386597	G	0.2015	1.27E-12	chr:4:120263688
4	rs28559989	120386627	T	0.2015	1.27E-12	chr:4:120263688
4	rs28587142	120386753	C	0.2015	1.27E-12	chr:4:120263688

4	rs28632018	120386730	C	0.2015	1.27E-12	chr:4:120263688
4	rs3733524	120423729	C	0.1888	1.27E-12	chr:4:120263688
4	rs71614444	120379436	G	0.1888	1.27E-12	chr:4:120263688
4	rs76837541	120375428	G	0.195	1.28E-12	chr:4:120263688
4	rs11722872	120233030	C	0.1899	1.35E-12	chr:4:120263688
4	rs10027517	120372234	G	0.2162	1.40E-12	chr:4:120263688
4	rs34818745	120257055	C	0.1938	1.41E-12	chr:4:120263688
4	rs10006525	120408931	G	0.1961	1.44E-12	chr:4:120263688
4	rs12508504	120410607	C	0.1961	1.44E-12	chr:4:120263688
4	rs13145352	120409963	C	0.1961	1.44E-12	chr:4:120263688
4	rs17005535	120411562	T	0.1961	1.44E-12	chr:4:120263688
4	rs10017335	120381523	G	0.1961	1.44E-12	chr:4:120263688
4	rs115227179	120355773	T	0.1991	1.49E-12	chr:4:120263688
4	rs7679468	120328333	C	0.202	1.50E-12	chr:4:120263688
4	rs71610281	120328001	C	0.1939	1.51E-12	chr:4:120263688
4	rs55825515	120486402	G	0.196	1.52E-12	chr:4:120263688
4	rs56372521	120374431	T	0.1901	1.61E-12	chr:4:120263688
4	rs79057525	120375389	G	0.1975	1.62E-12	chr:4:120263688
4	rs13134517	120405262	A	0.2164	1.62E-12	chr:4:120263688
4	rs6534135	120406302	G	0.2164	1.62E-12	chr:4:120263688
4	rs7661498	120408108	C	0.2164	1.62E-12	chr:4:120263688
4	rs9307477	120421809	A	0.2164	1.62E-12	chr:4:120263688
4	rs9684327	120365441	G	0.2164	1.62E-12	chr:4:120263688
4	rs9799664	120326678	T	0.2164	1.62E-12	chr:4:120263688
4	rs9996586	120326942	G	0.2164	1.62E-12	chr:4:120263688
4	rs7672493	120248260	A	0.1946	1.65E-12	chr:4:120263688
4	rs6843229	120424087	C	0.2143	1.65E-12	chr:4:120263688
4	rs6833334	120504227	T	0.1966	1.69E-12	chr:4:120263688
4	rs199865201	120332408	A	0.2223	1.77E-12	chr:4:120263688
4	rs12505876	120385472	A	0.2165	1.79E-12	chr:4:120263688
4	rs368938172	120330272	G	0.1926	1.80E-12	chr:4:120263688
4	rs71614435	120369711	A	0.2038	1.83E-12	chr:4:120263688
4	rs28403131	120285806	T	0.1901	1.90E-12	chr:4:120263688
4	rs28633994	120276037	G	0.1901	1.90E-12	chr:4:120263688
4	rs28634456	120375778	G	0.2029	1.92E-12	chr:4:120263688
4	rs78332141	120375782	T	0.2029	1.92E-12	chr:4:120263688
4	rs9995045	120321636	T	0.1938	1.93E-12	chr:4:120263688
4	rs11098506	120284971	G	0.1807	2.01E-12	chr:4:120263688
4	rs200898556	120500426	T	0.1943	2.02E-12	chr:4:120263688
4	rs3775841	120425777	C	0.2003	2.02E-12	chr:4:120263688
4	rs185413498	120327707	T	0.199	2.08E-12	chr:4:120263688
4	rs201087782	120327650	G	0.2005	2.20E-12	chr:4:120263688
4	rs4079144	120325432	T	0.2065	2.23E-12	chr:4:120263688

4	rs59182778	120374798	C	0.2018	2.29E-12	chr:4:120263688
4	rs28394116	120524283	G	0.2038	2.31E-12	chr:4:120263688
4	rs11098511	120306879	A	0.1952	2.40E-12	chr:4:120263688
4	rs13435370	120334666	C	0.1724	2.48E-12	chr:4:120263688
4	rs12504773	120562149	C	0.2012	2.50E-12	chr:4:120263688
4	rs13122709	120555356	A	0.2012	2.50E-12	chr:4:120263688
4	rs7680914	120563053	G	0.2012	2.50E-12	chr:4:120263688
4	rs10017189	120263523	C	0.2333	2.69E-12	chr:4:120263688
4	rs4001198	120325264	T	0.1981	2.71E-12	chr:4:120263688
4	rs4001175	120500667	A	0.2036	2.77E-12	chr:4:120263688
4	rs11735878	120290177	C	0.2093	2.82E-12	chr:4:120263688
4	rs10019309	120327085	T	0.2048	2.90E-12	chr:4:120263688
4	rs10019674	120443489	C	0.1989	2.93E-12	chr:4:120263688
4	rs3822192	120445720	C	0.1989	2.93E-12	chr:4:120263688
4	rs10003742	120271798	C	0.2199	3.30E-12	chr:4:120263688
4	rs10015883	120268146	G	0.2199	3.30E-12	chr:4:120263688
4	rs9637624	120274911	G	0.2199	3.30E-12	chr:4:120263688
4	rs13136462	120543173	T	0.2048	3.40E-12	chr:4:120263688
4	rs28535956	120536858	C	0.2048	3.40E-12	chr:4:120263688
4	rs28578366	120536905	T	0.2048	3.40E-12	chr:4:120263688
4	rs10031033	120151452	T	0.2407	3.41E-12	chr:4:120263688
4	rs12503276	120247329	A	0.2202	3.42E-12	chr:4:120263688
4	rs9995234	120321827	G	0.1986	3.43E-12	chr:4:120263688
4	rs1825061	120388902	T	0.2031	3.61E-12	chr:4:120263688
4	rs10027431	120372142	G	0.2381	3.84E-12	chr:4:120263688
4	rs10027432	120372143	C	0.2381	3.84E-12	chr:4:120263688
4	rs10004114	120259213	G	0.2207	3.89E-12	chr:4:120263688
4	rs10213542	120260650	T	0.2207	3.89E-12	chr:4:120263688
4	rs1814815	120258091	T	0.2207	3.89E-12	chr:4:120263688
4	rs2017058	120257739	C	0.2207	3.89E-12	chr:4:120263688
4	rs28439855	120262256	A	0.2207	3.89E-12	chr:4:120263688
4	rs4643791	120265619	A	0.2207	3.89E-12	chr:4:120263688
4	rs9307472	120261415	C	0.2207	3.89E-12	chr:4:120263688
4	rs28450134	120376318	C	0.2002	4.04E-12	chr:4:120263688
4	rs550821123	120327079	T	0.2064	4.07E-12	chr:4:120263688
4	rs6857105	120222298	G	0.2102	4.25E-12	chr:4:120263688
4	rs28571649	120388867	A	0.2065	4.43E-12	chr:4:120263688
4	rs9683520	120368199	C	0.2472	4.46E-12	chr:4:120263688
4	rs7695292	120558446	G	0.2038	4.54E-12	chr:4:120263688
4	rs9684878	120368175	C	0.2472	4.91E-12	chr:4:120263688
4	rs4001211	120331544	G	0.1723	5.01E-12	chr:4:120263688
4	rs28504370	120388890	A	0.2091	5.28E-12	chr:4:120263688
4	rs9760422	120354126	G	0.2441	5.28E-12	chr:4:120263688

4	rs3756156	120524841	A	0.2099	5.95E-12	chr:4:120263688
4	rs12505735	120532956	A	0.2105	6.15E-12	chr:4:120263688
4	rs12503309	120247397	A	0.226	6.47E-12	chr:4:120263688
4	rs12510923	120247381	G	0.226	6.47E-12	chr:4:120263688
4	rs13128933	120246249	A	0.2202	6.69E-12	chr:4:120263688
4	rs10518336	120522934	A	0.2411	7.32E-12	chr:4:120263688
4	rs78709015	120367728	T	0.2165	7.40E-12	chr:4:120263688
4	rs7674713	120322257	C	0.2263	7.52E-12	chr:4:120263688
4	rs13128774	120246282	T	0.1961	7.83E-12	chr:4:120263688
4	rs9762870	120367769	A	0.2165	8.78E-12	chr:4:120263688
4	rs11728730	120275003	C	0.2022	9.64E-12	chr:4:120263688
4	rs11098499	120187611	T	0.2219	1.01E-11	chr:4:120263688
4	rs11098500	120219239	A	0.2219	1.01E-11	chr:4:120263688
4	rs12499598	120189303	C	0.2219	1.01E-11	chr:4:120263688
4	rs12512517	120173245	G	0.2219	1.01E-11	chr:4:120263688
4	rs146531121	120174794	A	0.2219	1.01E-11	chr:4:120263688
4	rs1511018	120161580	T	0.2219	1.01E-11	chr:4:120263688
4	rs1511019	120166007	G	0.2219	1.01E-11	chr:4:120263688
4	rs199816242	120199351	T	0.2219	1.01E-11	chr:4:120263688
4	rs28419773	120211061	C	0.2219	1.01E-11	chr:4:120263688
4	rs28555550	120211040	G	0.2219	1.01E-11	chr:4:120263688
4	rs34585914	120189194	T	0.2219	1.01E-11	chr:4:120263688
4	rs35434465	120169378	G	0.2219	1.01E-11	chr:4:120263688
4	rs6814412	120170851	A	0.2219	1.01E-11	chr:4:120263688
4	rs6837898	120179154	T	0.2219	1.01E-11	chr:4:120263688
4	rs7659194	120207147	G	0.2219	1.01E-11	chr:4:120263688
4	rs7658368	120310922	T	0.2157	1.01E-11	chr:4:120263688
4	rs371048191	120246107	C	0.2053	1.03E-11	chr:4:120263688
4	rs58601355	120186367	C	0.2243	1.06E-11	chr:4:120263688
4	rs4001176	120500705	G	0.2193	1.24E-11	chr:4:120263688
4	rs531826432	120327073	T	0.2188	1.25E-11	chr:4:120263688
4	rs78958430	120338339	T	0.2224	1.26E-11	chr:4:120263688
4	rs79262354	120338335	T	0.2224	1.26E-11	chr:4:120263688
4	rs4511986	120354674	C	0.2145	1.27E-11	chr:4:120263688
4	rs13117947	120257060	G	0.2442	1.34E-11	chr:4:120263688
4	rs6851169	120117510	C	0.2226	1.59E-11	chr:4:120263688
4	rs68128210	120137819	G	0.2283	1.63E-11	chr:4:120263688
4	rs373699192	120246109	A	0.197	1.71E-11	chr:4:120263688
4	rs3956479	120360863	T	0.2795	1.76E-11	chr:4:120263688
4	rs3956480	120360861	T	0.2795	1.76E-11	chr:4:120263688
4	rs11726229	120211580	T	0.2317	1.79E-11	chr:4:120263688
4	rs13113112	120156040	T	0.2251	1.79E-11	chr:4:120263688
4	rs1011054	120202387	A	0.2254	1.89E-11	chr:4:120263688

4	rs3749591	120214030	G	0.2254	1.89E-11	chr:4:120263688
4	rs201085338	120347661	A	0.2237	1.99E-11	chr:4:120263688
4	rs6847248	120225955	G	0.2098	2.22E-11	chr:4:120263688
4	rs200207680	120338383	T	0.228	2.22E-11	chr:4:120263688
4	rs181331234	120327890	C	0.2142	2.23E-11	chr:4:120263688
4	rs13126596	120140729	G	0.2297	2.48E-11	chr:4:120263688
4	rs12501602	120287935	T	0.2252	2.99E-11	chr:4:120263688
4	rs10022508	120115228	G	0.2296	3.01E-11	chr:4:120263688
4	rs12502503	120116255	T	0.2296	3.01E-11	chr:4:120263688
4	rs12509054	120115075	C	0.2296	3.01E-11	chr:4:120263688
4	rs6822498	120120183	C	0.2296	3.01E-11	chr:4:120263688
4	rs6832670	120118792	G	0.2296	3.01E-11	chr:4:120263688
4	rs6838674	120119376	G	0.2296	3.01E-11	chr:4:120263688
4	rs4833613	120150156	A	0.2602	3.26E-11	chr:4:120263688
4	rs201495048	120333126	A	0.2267	3.72E-11	chr:4:120263688
4	rs4513552	120354657	T	0.2819	3.76E-11	chr:4:120263688
4	rs138401876	120370298	A	0.2375	4.10E-11	chr:4:120263688
4	rs6845702	120120453	G	0.2312	4.52E-11	chr:4:120263688
4	rs13123591	120105990	G	0.2347	4.62E-11	chr:4:120263688
4	rs17839089	120111069	G	0.2347	4.62E-11	chr:4:120263688
4	rs6826823	120112098	C	0.2347	4.62E-11	chr:4:120263688
4	rs7661020	120106982	C	0.2347	4.62E-11	chr:4:120263688
4	rs9995277	120108603	A	0.2347	4.62E-11	chr:4:120263688
4	rs117030010	120333095	C	0.2318	5.10E-11	chr:4:120263688
4	rs10006304	120124305	G	0.2357	5.34E-11	chr:4:120263688
4	rs13138782	120124474	A	0.2357	5.34E-11	chr:4:120263688
4	rs9684723	120367735	C	0.2957	5.62E-11	chr:4:120263688
4	rs10019421	120327198	T	0.287	5.81E-11	chr:4:120263688
4	rs10031129	120327201	C	0.287	5.81E-11	chr:4:120263688
4	rs56122576	120129336	G	0.2342	6.05E-11	chr:4:120263688
4	rs10213342	120175237	G	0.256	6.17E-11	chr:4:120263688
4	rs2715016	120452356	T	0.2529	6.40E-11	chr:4:120263688
4	rs2389885	120533931	T	0.2276	7.16E-11	chr:4:120263688
4	rs370990577	120327271	C	0.2381	7.88E-11	chr:4:120263688
4	rs552865294	120246034	A	0.2875	9.99E-11	chr:4:120263688
4	rs11723550	120289961	C	0.2583	1.01E-10	chr:4:120263688
4	rs28452522	120110784	G	0.2406	1.06E-10	chr:4:120263688
4	rs10010696	120164303	C	0.2642	1.17E-10	chr:4:120263688
4	rs10022262	120175152	G	0.2642	1.17E-10	chr:4:120263688
4	rs10029750	120172543	G	0.2642	1.17E-10	chr:4:120263688
4	rs11098496	120167466	G	0.2642	1.17E-10	chr:4:120263688
4	rs11729897	120174453	T	0.2642	1.17E-10	chr:4:120263688
4	rs11734519	120171071	G	0.2642	1.17E-10	chr:4:120263688

4	rs11944880	120159334	T	0.2642	1.17E-10	chr:4:120263688
4	rs12501610	120175763	T	0.2642	1.17E-10	chr:4:120263688
4	rs12506395	120185094	T	0.2642	1.17E-10	chr:4:120263688
4	rs13116504	120209412	C	0.2642	1.17E-10	chr:4:120263688
4	rs13133044	120201252	C	0.2642	1.17E-10	chr:4:120263688
4	rs13435802	120177960	T	0.2642	1.17E-10	chr:4:120263688
4	rs1397608	120161744	G	0.2642	1.17E-10	chr:4:120263688
4	rs1511015	120170473	C	0.2642	1.17E-10	chr:4:120263688
4	rs28374891	120183550	A	0.2642	1.17E-10	chr:4:120263688
4	rs34630083	120174569	G	0.2642	1.17E-10	chr:4:120263688
4	rs35197422	120169314	G	0.2642	1.17E-10	chr:4:120263688
4	rs35643470	120184948	C	0.2642	1.17E-10	chr:4:120263688
4	rs7684942	120162201	T	0.2642	1.17E-10	chr:4:120263688
4	rs6849889	120186348	C	0.2668	1.24E-10	chr:4:120263688
4	rs7685268	120162188	A	0.2685	1.61E-10	chr:4:120263688
4	rs368648502	120331079	G	0.2427	1.70E-10	chr:4:120263688
4	rs34616304	120169435	T	0.2664	1.80E-10	chr:4:120263688
4	rs11931312	120159023	C	0.2684	2.08E-10	chr:4:120263688
4	rs13114751	120156617	A	0.2684	2.08E-10	chr:4:120263688
4	rs13129661	120152909	G	0.2684	2.08E-10	chr:4:120263688
4	rs1551	120158500	G	0.2684	2.08E-10	chr:4:120263688
4	rs35271032	120156659	T	0.2684	2.08E-10	chr:4:120263688
4	rs35916640	120155852	C	0.2684	2.08E-10	chr:4:120263688
4	rs4145951	120155817	G	0.2684	2.08E-10	chr:4:120263688
4	rs56386062	120155135	C	0.2684	2.08E-10	chr:4:120263688
4	rs67073020	120152557	T	0.2684	2.08E-10	chr:4:120263688
4	rs6815725	120158410	A	0.2684	2.08E-10	chr:4:120263688
4	rs373735748	120244580	T	0.2736	2.34E-10	chr:4:120263688
4	rs55884612	120244577	T	0.2736	2.34E-10	chr:4:120263688
4	rs80259299	120336497	G	0.2052	2.56E-10	chr:4:120263688
4	rs140305554	120247693	C	0.2012	2.61E-10	chr:4:120263688
4	rs73844769	120254829	C	0.3173	2.69E-10	chr:4:120263688
4	rs368193355	120327846	C	0.2459	3.89E-10	chr:4:120263688
4	rs150408742	120122911	A	0.2546	4.49E-10	chr:4:120263688
4	rs10003931	120259052	G	0.2142	5.03E-10	chr:4:120263688
4	rs3956463	120245748	A	0.279	5.11E-10	chr:4:120263688
4	rs62319649	120522579	C	3.628	5.70E-10	chr:4:120263688
4	rs11724758	120239878	A	0.2852	6.12E-10	chr:4:120263688
4	rs74529999	120327854	C	0.2565	6.68E-10	chr:4:120263688
4	rs2036858	120249237	C	0.2875	7.46E-10	chr:4:120263688
4	rs12186259	120152039	T	0.283	8.47E-10	chr:4:120263688
4	rs200940395	120333454	C	0.1862	1.15E-09	chr:4:120263688
4	rs569705290	120333521	C	0.1898	1.20E-09	chr:4:120263688

4	rs4001284	120334957	G	0.3348	1.27E-09	chr:4:120263688
4	rs10014822	120280143	T	0.3021	1.43E-09	chr:4:120263688
4	rs11098493	120104113	G	0.294	1.43E-09	chr:4:120263688
4	rs9994651	120587803	A	0.2858	1.83E-09	chr:4:120263688
4	rs9994488	120587781	T	0.2893	2.52E-09	chr:4:120263688
4	rs4452411	120700609	A	0.2959	2.54E-09	chr:4:120263688
4	rs4287989	120700480	G	3.309	2.55E-09	chr:4:120263688
4	rs71612601	120332595	T	0.2921	3.30E-09	chr:4:120263688
4	rs11937425	120235999	C	3.499	3.30E-09	chr:4:120263688
4	rs11732403	120234725	G	3.469	3.67E-09	chr:4:120263688
4	rs34049297	120234722	C	3.469	3.67E-09	chr:4:120263688
4	rs10518301	120235125	C	3.462	4.21E-09	chr:4:120263688
4	rs11722847	120233121	T	3.462	4.21E-09	chr:4:120263688
4	rs13118936	120234402	A	3.462	4.21E-09	chr:4:120263688
4	rs13139118	120233640	C	3.462	4.21E-09	chr:4:120263688
4	rs4364243	120230860	T	3.462	4.21E-09	chr:4:120263688
4	rs9685057	120272333	G	0.3603	5.50E-09	chr:4:120263688
4	rs78624933	120338651	C	0.2658	6.09E-09	chr:4:120263688
4	rs72916543	120254961	T	0.3606	6.37E-09	chr:4:120263688
4	rs201764925	120334210	A	0.2001	6.76E-09	chr:4:120263688
4	rs11729978	120079387	A	0.3125	7.00E-09	chr:4:120263688
4	rs11731008	120079925	A	0.3125	7.00E-09	chr:4:120263688
4	rs11733525	120082417	T	0.3125	7.00E-09	chr:4:120263688
4	rs12508316	120081291	A	0.3125	7.00E-09	chr:4:120263688
4	rs4334737	120080346	C	0.3125	7.00E-09	chr:4:120263688
4	rs7696877	120081869	T	0.3125	7.00E-09	chr:4:120263688
4	rs370208580	120333619	G	0.2473	7.44E-09	chr:4:120263688
4	rs11098490	120086883	A	0.3185	9.66E-09	chr:4:120263688
4	rs11098486	120066810	A	0.3196	1.12E-08	chr:4:120263688
4	rs11098489	120081606	G	0.3196	1.12E-08	chr:4:120263688
4	rs11098491	120088542	T	0.3196	1.12E-08	chr:4:120263688
4	rs11098492	120095260	A	0.3196	1.12E-08	chr:4:120263688
4	rs11733239	120087698	T	0.3196	1.12E-08	chr:4:120263688
4	rs12513191	120096821	C	0.3196	1.12E-08	chr:4:120263688
4	rs13120560	120090246	C	0.3196	1.12E-08	chr:4:120263688
4	rs2036861	120102289	G	0.3196	1.12E-08	chr:4:120263688
4	rs4355373	120084955	C	0.3196	1.12E-08	chr:4:120263688
4	rs9993110	120092088	T	0.3196	1.12E-08	chr:4:120263688
4	rs186992684	120336473	T	0.3434	1.14E-08	chr:4:120263688
4	rs4834779	120425246	A	0.2539	1.19E-08	chr:4:120263688
4	rs6842973	120694059	A	3.197	1.25E-08	chr:4:120263688
4	rs376876178	120333615	G	0.2636	1.32E-08	chr:4:120263688
4	rs199515440	120372424	T	0.2295	1.46E-08	chr:4:120263688

4	rs1033087	120103401	C	0.3237	1.55E-08	chr:4:120263688
4	rs7678043	120698652	T	3.071	1.68E-08	chr:4:120263688
4	rs28567749	120329211	C	0.2872	1.93E-08	chr:4:120263688
4	rs11730138	120079800	A	0.3273	1.99E-08	chr:4:120263688
4	rs184174836	120196561	G	0.3452	2.05E-08	chr:4:120263688
4	rs4504231	120586891	A	0.3312	2.26E-08	chr:4:120263688
4	rs71595380	120329294	T	0.292	2.47E-08	chr:4:120263688
4	rs2389957	120695322	C	3.043	2.75E-08	chr:4:120263688
4	rs77222221	120329212	A	0.2939	3.36E-08	chr:4:120263688
4	rs10518337	120574874	C	3.049	3.60E-08	chr:4:120263688
4	rs4593113	120697811	A	2.936	3.86E-08	chr:4:120263688
4	rs527513020	120358891	C	0.2365	3.93E-08	chr:4:120263688
4	rs565835685	120358886	G	0.2365	3.93E-08	chr:4:120263688
4	rs202019903	120344304	C	0.275	4.24E-08	chr:4:120263688
4	rs12499377	120587215	A	0.3373	4.27E-08	chr:4:120263688
4	rs71627788	120255728	G	0.3736	5.75E-08	chr:4:120263688
4	rs11735819	120290194	C	2.82	5.95E-08	chr:4:120263688
4	rs2389958	120695256	C	2.916	6.44E-08	chr:4:120263688
4	rs2892892	120697553	G	2.916	6.44E-08	chr:4:120263688
4	rs4311295	120695280	A	2.916	6.44E-08	chr:4:120263688
4	rs4447844	120696623	A	2.916	6.44E-08	chr:4:120263688
4	rs1459055	120056815	A	0.3389	6.45E-08	chr:4:120263688
4	rs558588080	120348891	A	0.2531	6.65E-08	chr:4:120263688
4	rs13136593	120101772	A	0.3402	7.59E-08	chr:4:120263688
4	rs12512646	120585733	A	0.3443	7.69E-08	chr:4:120263688
4	rs4833624	120585497	T	0.3443	7.69E-08	chr:4:120263688
4	rs6822909	120691218	T	3.072	8.76E-08	chr:4:120263688
4	rs62320739	120279459	C	0.3609	1.15E-07	chr:4:120263688
4	rs10014947	120280077	A	0.3636	1.44E-07	chr:4:120263688
4	rs11737400	120279335	G	0.3636	1.44E-07	chr:4:120263688
4	rs13149954	120279041	A	0.3636	1.44E-07	chr:4:120263688
4	rs13150541	120279284	A	0.3636	1.44E-07	chr:4:120263688
4	rs28580295	120278873	T	0.3636	1.44E-07	chr:4:120263688
4	rs62320738	120279420	A	0.3636	1.44E-07	chr:4:120263688
4	rs62320740	120279486	A	0.3636	1.44E-07	chr:4:120263688
4	rs71627791	120278765	G	0.3636	1.44E-07	chr:4:120263688
4	rs1480934	120476006	C	3.121	1.46E-07	chr:4:120263688
4	rs2622496	120470255	T	3.121	1.46E-07	chr:4:120263688
4	rs140263405	120325820	A	0.2067	1.47E-07	chr:4:120263688
4	rs10001829	120665293	C	0.3492	1.55E-07	chr:4:120263688
4	rs13103043	120665371	C	0.3492	1.55E-07	chr:4:120263688
4	rs59319494	120696060	A	2.97	1.71E-07	chr:4:120263688
4	rs12512171	120662564	T	0.35	1.72E-07	chr:4:120263688

4	rs2714966	120677425	A	2.923	1.78E-07	chr:4:120263688
4	rs13122890	120601731	G	0.3456	1.84E-07	chr:4:120263688
4	rs2252708	120612158	A	0.3456	1.84E-07	chr:4:120263688
4	rs11731675	120212514	C	0.3663	2.03E-07	chr:4:120263688
4	rs10026625	120280136	C	0.3723	2.38E-07	chr:4:120263688
4	rs4337717	120643040	C	0.3586	2.50E-07	chr:4:120263688
4	rs12503809	120472922	T	0.1994	2.66E-07	chr:4:120263688
4	rs187593210	120549862	C	0.1994	2.66E-07	chr:4:120263688
4	rs12498792	120097358	T	0.1888	2.72E-07	chr:4:120263688
4	rs180904825	120127516	A	0.1902	2.74E-07	chr:4:120263688
4	rs2256834	120663939	C	0.3564	2.76E-07	chr:4:120263688
4	rs2714985	120664142	A	0.3564	2.76E-07	chr:4:120263688
4	rs7683474	120612853	C	0.3497	2.78E-07	chr:4:120263688
4	rs10518330	120401969	C	0.2018	2.94E-07	chr:4:120263688
4	rs200974711	120468265	G	0.3916	2.94E-07	chr:4:120263688
4	rs12506537	120220465	T	0.2031	3.12E-07	chr:4:120263688
4	rs6821717	120599661	T	0.3538	3.36E-07	chr:4:120263688
4	rs10032299	120206419	T	0.3759	3.45E-07	chr:4:120263688
4	rs1052633	120215314	G	0.3759	3.45E-07	chr:4:120263688
4	rs11098497	120187068	T	0.3759	3.45E-07	chr:4:120263688
4	rs11732621	120212883	G	0.3759	3.45E-07	chr:4:120263688
4	rs2175383	120215836	A	0.3759	3.45E-07	chr:4:120263688
4	rs6843509	120220196	A	0.3759	3.45E-07	chr:4:120263688
4	rs7659403	120207254	A	0.3759	3.45E-07	chr:4:120263688
4	rs2714965	120676814	T	2.842	4.17E-07	chr:4:120263688
4	rs2583602	120679347	A	2.725	4.32E-07	chr:4:120263688
4	rs12500145	120688312	A	0.3653	4.35E-07	chr:4:120263688
4	rs7655035	120617004	T	0.3676	5.08E-07	chr:4:120263688
4	rs4447843	120680950	G	0.3728	5.13E-07	chr:4:120263688
4	rs2389959	120689407	C	2.805	5.14E-07	chr:4:120263688
4	rs11098498	120187119	C	0.3821	5.40E-07	chr:4:120263688
4	rs6822808	120217365	A	0.3821	5.40E-07	chr:4:120263688
4	rs113409736	120195841	T	2.839	5.99E-07	chr:4:120263688
4	rs148150770	120421312	G	0.219	6.60E-07	chr:4:120263688
4	rs6534150	120685502	A	2.668	6.86E-07	chr:4:120263688
4	rs7675857	120680581	C	2.668	6.86E-07	chr:4:120263688
4	rs9997209	120683244	G	2.668	6.86E-07	chr:4:120263688
4	rs12507089	120025722	T	0.2063	6.97E-07	chr:4:120263688
4	rs2389903	120646281	G	0.3771	7.06E-07	chr:4:120263688
4	rs7693769	120658676	C	0.3724	7.09E-07	chr:4:120263688
4	rs140383661	120646775	G	0.2213	7.67E-07	chr:4:120263688
4	rs147458152	120640760	C	0.2213	7.67E-07	chr:4:120263688
4	rs62321169	120591368	C	0.3692	7.71E-07	chr:4:120263688

4	rs11736455	120685312	T	0.3787	7.83E-07	chr:4:120263688
4	rs7674894	120680189	G	0.3787	7.83E-07	chr:4:120263688
4	rs7676064	120680560	A	0.3787	7.83E-07	chr:4:120263688
4	rs7679245	120328196	T	2.91	8.10E-07	chr:4:120263688
4	rs11722624	120673096	T	2.649	8.90E-07	chr:4:120263688
4	rs11734863	120673259	G	2.649	8.90E-07	chr:4:120263688
4	rs2252621	120675026	C	2.649	8.90E-07	chr:4:120263688
4	rs2252625	120674899	T	2.649	8.90E-07	chr:4:120263688
4	rs28663988	120620388	C	0.3828	9.20E-07	chr:4:120263688
4	rs7664591	120627116	C	0.3835	9.99E-07	chr:4:120263688
4	rs9884655	120700717	C	2.612	1.17E-06	chr:4:120263688
4	rs144920716	120566010	G	0.2324	1.22E-06	chr:4:120263688
4	rs12647635	120226932	T	2.719	1.29E-06	chr:4:120263688
4	rs28421394	120229248	A	2.719	1.29E-06	chr:4:120263688
4	rs28848291	120229356	G	2.719	1.29E-06	chr:4:120263688
4	rs28864107	120229153	G	2.719	1.29E-06	chr:4:120263688
4	rs4834768	120228641	G	2.719	1.29E-06	chr:4:120263688
4	rs56344202	120228425	T	2.719	1.29E-06	chr:4:120263688
4	rs4094	120619421	G	0.388	1.54E-06	chr:4:120263688
4	rs2714973	120662172	A	2.606	1.56E-06	chr:4:120263688
4	rs2256054	120669517	C	2.546	1.61E-06	chr:4:120263688
4	rs2583596	120670120	A	2.546	1.61E-06	chr:4:120263688
4	rs2583597	120670150	A	2.546	1.61E-06	chr:4:120263688
4	rs2714978	120670213	G	2.546	1.61E-06	chr:4:120263688
4	rs2714980	120668765	T	2.546	1.61E-06	chr:4:120263688
4	rs12649773	120305262	A	2.807	1.71E-06	chr:4:120263688
4	rs142534042	120308936	C	2.807	1.71E-06	chr:4:120263688
4	rs4833617	120305176	A	2.807	1.71E-06	chr:4:120263688
4	rs55827028	120297059	A	2.807	1.71E-06	chr:4:120263688
4	rs62322286	120307242	G	2.807	1.71E-06	chr:4:120263688
4	rs2714970	120657355	T	2.586	1.88E-06	chr:4:120263688
4	rs725449	120656892	T	2.586	1.88E-06	chr:4:120263688
4	rs13111751	120050762	G	0.3684	1.95E-06	chr:4:120263688
4	rs2714976	120670555	G	2.59	1.98E-06	chr:4:120263688
4	rs13113885	120221176	C	0.3982	2.14E-06	chr:4:120263688
4	rs4834771	120242772	C	2.773	2.22E-06	chr:4:120263688
4	rs2583605	120659992	A	2.57	2.38E-06	chr:4:120263688
4	rs10023652	120662947	C	0.3952	2.43E-06	chr:4:120263688
4	rs11731571	120221185	A	0.4142	2.77E-06	chr:4:120263688
4	rs10011928	120662973	G	0.3973	2.86E-06	chr:4:120263688
4	rs874425	120664362	C	0.3973	2.86E-06	chr:4:120263688
4	rs878374	120237564	A	2.733	2.90E-06	chr:4:120263688
4	rs2583604	120660715	A	2.536	2.99E-06	chr:4:120263688

4	rs1511016	120170355	A	2.887	3.29E-06	chr:4:120263688
4	rs4496567	120175625	A	2.887	3.29E-06	chr:4:120263688
4	rs6534131	120180195	A	2.887	3.29E-06	chr:4:120263688
4	rs6823331	120180790	T	2.887	3.29E-06	chr:4:120263688
4	rs7676969	120173724	C	2.887	3.29E-06	chr:4:120263688
4	rs12510467	120618359	A	0.4004	3.34E-06	chr:4:120263688
4	rs5008452	120621752	T	0.4004	3.34E-06	chr:4:120263688
4	rs10034579	120244029	A	2.713	3.41E-06	chr:4:120263688
4	rs1397613	120242929	G	2.713	3.41E-06	chr:4:120263688
4	rs2282688	120243722	T	2.713	3.41E-06	chr:4:120263688
4	rs6823963	120242164	A	2.713	3.41E-06	chr:4:120263688
4	rs6857641	120243511	T	2.713	3.41E-06	chr:4:120263688
4	rs12642411	120580525	C	0.3975	3.50E-06	chr:4:120263688
4	rs6836219	120597941	T	2.504	3.53E-06	chr:4:120263688
4	rs2252565	120613111	T	2.5	3.58E-06	chr:4:120263688
4	rs76798497	120667915	C	3.053	3.71E-06	chr:4:120263688
4	rs17005489	120649070	A	2.488	3.77E-06	chr:4:120263688
4	rs531119820	120247694	A	0.164	3.94E-06	chr:4:120263688
4	rs4834772	120272316	A	0.4786	4.30E-06	chr:4:120263688
4	rs10031010	120035180	A	2.942	4.31E-06	chr:4:120263688
4	rs28455602	120034116	G	2.942	4.31E-06	chr:4:120263688
4	rs28690965	120034317	C	2.942	4.31E-06	chr:4:120263688
4	rs61391192	120036209	G	2.942	4.31E-06	chr:4:120263688
4	rs2102541	120115379	G	2.583	4.63E-06	chr:4:120263688
4	rs62326348	120114659	G	2.583	4.63E-06	chr:4:120263688
4	rs62326349	120116225	T	2.583	4.63E-06	chr:4:120263688
4	rs7699252	120659198	G	0.4054	4.63E-06	chr:4:120263688
4	rs10022185	120571765	G	0.4034	4.67E-06	chr:4:120263688
4	rs17051352	120581427	A	0.4034	4.67E-06	chr:4:120263688
4	rs2389882	120566733	C	0.4034	4.67E-06	chr:4:120263688
4	rs2389886	120570422	A	0.4034	4.67E-06	chr:4:120263688
4	rs2389887	120570644	C	0.4034	4.67E-06	chr:4:120263688
4	rs34278750	120571136	A	0.4034	4.67E-06	chr:4:120263688
4	rs7671096	120579317	A	0.4034	4.67E-06	chr:4:120263688
4	rs2250346	120648078	T	2.472	4.76E-06	chr:4:120263688
4	rs2583617	120644194	A	2.472	4.76E-06	chr:4:120263688
4	rs2583619	120652805	T	2.497	4.76E-06	chr:4:120263688
4	rs2714961	120628627	C	2.486	4.78E-06	chr:4:120263688
4	rs9884581	120699261	A	0.4082	5.05E-06	chr:4:120263688
4	rs55732167	120141507	T	2.586	5.22E-06	chr:4:120263688
4	rs10007283	120619656	G	0.4104	5.41E-06	chr:4:120263688
4	rs4834770	120241849	A	2.644	5.42E-06	chr:4:120263688
4	rs7437666	120653063	C	2.475	5.65E-06	chr:4:120263688

4	rs62326344	120106420	G	2.574	5.65E-06	chr:4:120263688
4	rs62326345	120107683	A	2.574	5.65E-06	chr:4:120263688
4	rs62326346	120108263	A	2.574	5.65E-06	chr:4:120263688
4	rs62326347	120109440	T	2.574	5.65E-06	chr:4:120263688
4	rs12645079	120194512	C	2.549	5.93E-06	chr:4:120263688
4	rs12645965	120204761	A	2.549	5.93E-06	chr:4:120263688
4	rs12649811	120183939	T	2.549	5.93E-06	chr:4:120263688
4	rs12650267	120195411	A	2.549	5.93E-06	chr:4:120263688
4	rs17049968	120159916	A	2.549	5.93E-06	chr:4:120263688
4	rs200134651	120200519	A	2.549	5.93E-06	chr:4:120263688
4	rs35372126	120181724	G	2.549	5.93E-06	chr:4:120263688
4	rs4833614	120176338	T	2.549	5.93E-06	chr:4:120263688
4	rs56072232	120193715	T	2.549	5.93E-06	chr:4:120263688
4	rs62328371	120165690	G	2.549	5.93E-06	chr:4:120263688
4	rs62328372	120166809	G	2.549	5.93E-06	chr:4:120263688
4	rs62328373	120167453	C	2.549	5.93E-06	chr:4:120263688
4	rs62328374	120168837	A	2.549	5.93E-06	chr:4:120263688
4	rs62328376	120171735	G	2.549	5.93E-06	chr:4:120263688
4	rs62328377	120173569	A	2.549	5.93E-06	chr:4:120263688
4	rs62328379	120178193	C	2.549	5.93E-06	chr:4:120263688
4	rs62328398	120201031	A	2.549	5.93E-06	chr:4:120263688
4	rs76969560	120206724	T	2.549	5.93E-06	chr:4:120263688
4	rs12644283	120133549	G	2.546	6.13E-06	chr:4:120263688
4	rs9684358	120622560	C	0.4125	6.33E-06	chr:4:120263688
4	rs2248806	120621423	A	2.441	6.36E-06	chr:4:120263688
4	rs2583609	120623111	T	2.441	6.36E-06	chr:4:120263688
4	rs2715026	120616805	C	2.441	6.36E-06	chr:4:120263688
4	rs4834802	120614296	C	0.4165	6.49E-06	chr:4:120263688
4	rs62328369	120151494	A	2.532	6.58E-06	chr:4:120263688
4	rs187392552	120200618	C	2.544	6.66E-06	chr:4:120263688
4	rs878375	120237574	T	2.581	6.83E-06	chr:4:120263688
4	rs2714960	120630872	C	2.434	6.84E-06	chr:4:120263688
4	rs12642042	120310479	T	2.629	6.92E-06	chr:4:120263688
4	rs62322279	120298548	T	2.629	6.92E-06	chr:4:120263688
4	rs62322287	120312172	A	2.629	6.92E-06	chr:4:120263688
4	rs1973598	120243419	A	2.551	7.17E-06	chr:4:120263688
4	rs907736	120289943	C	2.379	7.20E-06	chr:4:120263688
4	rs4834762	120132690	T	2.526	7.22E-06	chr:4:120263688
4	rs62326352	120127046	C	2.526	7.22E-06	chr:4:120263688
4	rs7664440	120578540	G	0.4123	7.50E-06	chr:4:120263688
4	rs4001200	120378665	G	0.1512	7.78E-06	chr:4:120263688
4	rs2245564	120625812	T	2.417	7.83E-06	chr:4:120263688
4	rs17642800	120219534	C	2.509	8.01E-06	chr:4:120263688

4	rs2583608	120620379	C	2.41	8.01E-06	chr:4:120263688
4	rs77377547	120217711	G	2.509	8.01E-06	chr:4:120263688
4	rs10018308	120657899	G	0.4042	8.77E-06	chr:4:120263688
4	rs532905339	120195910	T	2.553	9.06E-06	chr:4:120263688
4	rs749526	120663649	T	0.4234	9.60E-06	chr:4:120263688
4	rs7694773	120652993	A	0.413	9.97E-06	chr:4:120263688
4	rs28723049	120620507	A	0.4246	1.04E-05	chr:4:120263688
4	rs28869613	120620588	A	0.4246	1.04E-05	chr:4:120263688
4	rs4834749	120042718	A	2.769	1.04E-05	chr:4:120263688
4	rs17051356	120585308	T	0.4187	1.08E-05	chr:4:120263688
4	rs10029669	120619666	T	0.4281	1.08E-05	chr:4:120263688
4	rs12331776	120617412	A	0.4281	1.08E-05	chr:4:120263688
4	rs6534133	120270703	C	3.004	1.33E-05	chr:4:120263688
4	rs62326879	120029060	C	2.708	1.34E-05	chr:4:120263688
4	rs11098543	120748542	G	0.4201	1.41E-05	chr:4:120263688
4	rs4834817	120752566	C	0.4201	1.41E-05	chr:4:120263688
4	rs6534157	120753056	G	0.4201	1.41E-05	chr:4:120263688
4	rs6858155	120754216	T	0.4201	1.41E-05	chr:4:120263688
4	rs143726128	120348512	C	0.5065	1.58E-05	chr:4:120263688
4	rs62326359	120143266	A	2.244	1.67E-05	chr:4:120263688
4	rs13119815	120662321	A	0.435	1.68E-05	chr:4:120263688
4	rs144910036	120498353	C	0.3783	1.74E-05	chr:4:120263688
4	rs11737790	120751664	T	0.4248	1.83E-05	chr:4:120263688
4	rs2389919	120756095	G	0.4248	1.83E-05	chr:4:120263688
4	rs72682879	120754392	G	0.4248	1.83E-05	chr:4:120263688
4	rs2902921	9591436	A	0.07505	2.88E-15	chr:4:9603240
4	rs12645445	9599283	G	0.06264	3.26E-15	chr:4:9603240
4	rs1842487	9597264	C	0.06264	3.26E-15	chr:4:9603240
4	rs6448859	9596488	C	0.06264	3.26E-15	chr:4:9603240
4	rs6811235	9594627	G	0.06264	3.26E-15	chr:4:9603240
4	rs35735545	9600181	A	0.06312	4.20E-15	chr:4:9603240
4	rs34968063	9599535	T	0.06315	4.32E-15	chr:4:9603240
4	rs7664612	9595727	A	0.1076	8.87E-15	chr:4:9603240
4	rs78121242	9591433	A	0.0852	9.41E-15	chr:4:9603240
4	rs2865075	9591384	C	0.09316	1.22E-14	chr:4:9603240
4	rs10019130	9597356	A	0.09377	1.44E-14	chr:4:9603240
4	rs10212854	9590489	G	0.09377	1.44E-14	chr:4:9603240
4	rs11728108	9590027	C	0.09377	1.44E-14	chr:4:9603240
4	rs13103207	9603672	C	0.09377	1.44E-14	chr:4:9603240
4	rs1842488	9601268	G	0.09377	1.44E-14	chr:4:9603240
4	rs2865079	9600388	G	0.09377	1.44E-14	chr:4:9603240
4	rs6837173	9590700	G	0.09377	1.44E-14	chr:4:9603240
4	rs6855316	9596183	C	0.09377	1.44E-14	chr:4:9603240

4	rs9714994	9589698	C	0.0859	1.76E-14	chr:4:9603240
4	rs6856351	9590718	C	0.1028	1.79E-14	chr:4:9603240
4	rs111921266	9591246	A	0.1092	2.45E-14	chr:4:9603240
4	rs11729986	9605023	G	0.1455	6.67E-13	chr:4:9603240
4	rs6448969	9700056	G	0.1844	1.01E-12	chr:4:9603240
4	rs9714725	9589901	G	0.1144	1.61E-12	chr:4:9603240
4	rs12501341	9589915	A	0.1324	1.77E-12	chr:4:9603240
4	rs1871719	9594339	T	5.88	3.41E-12	chr:4:9603240
4	rs6811619	9613669	A	5.607	4.79E-12	chr:4:9603240
4	rs13141264	9698240	A	5.501	6.21E-12	chr:4:9603240
4	rs13134147	9611368	G	5.72	6.41E-12	chr:4:9603240
4	rs13133557	9609114	A	5.651	6.84E-12	chr:4:9603240
4	rs10939455	9672007	T	5.698	7.03E-12	chr:4:9603240
4	rs11726932	9656993	G	5.52	7.87E-12	chr:4:9603240
4	rs11732839	9597885	G	5.568	8.71E-12	chr:4:9603240
4	rs11730577	9592342	T	5.566	9.47E-12	chr:4:9603240
4	rs11737375	9607387	G	5.566	9.47E-12	chr:4:9603240
4	rs12510498	9606546	A	5.566	9.47E-12	chr:4:9603240
4	rs13105082	9604504	G	5.566	9.47E-12	chr:4:9603240
4	rs13108259	9603571	G	5.566	9.47E-12	chr:4:9603240
4	rs13120086	9599365	T	5.566	9.47E-12	chr:4:9603240
4	rs13123739	9604667	T	5.566	9.47E-12	chr:4:9603240
4	rs13124589	9590540	T	5.566	9.47E-12	chr:4:9603240
4	rs13141297	9602103	T	5.566	9.47E-12	chr:4:9603240
4	rs62409962	9600872	T	5.566	9.47E-12	chr:4:9603240
4	rs35497679	9636851	T	5.294	1.04E-11	chr:4:9603240
4	rs13147553	9610951	A	5.374	1.12E-11	chr:4:9603240
4	rs62409885	9642489	T	6.38	1.46E-11	chr:4:9603240
4	rs9761193	9669715	C	5.368	1.50E-11	chr:4:9603240
4	rs12511479	9650516	A	5.087	1.65E-11	chr:4:9603240
4	rs13129232	9573407	G	0.1853	1.67E-11	chr:4:9603240
4	rs13144634	9571273	A	0.1853	1.67E-11	chr:4:9603240
4	rs11723247	9656338	T	5.172	1.70E-11	chr:4:9603240
4	rs11726037	9657179	G	5.172	1.70E-11	chr:4:9603240
4	rs112404663	9624480	T	0.2103	1.86E-11	chr:4:9603240
4	rs34793939	9674154	A	5.177	1.92E-11	chr:4:9603240
4	rs6831476	9708183	T	4.732	1.98E-11	chr:4:9603240
4	rs10805307	9580141	G	0.1855	1.99E-11	chr:4:9603240
4	rs10939373	9579571	G	0.1855	1.99E-11	chr:4:9603240
4	rs11722510	9585639	C	0.1855	1.99E-11	chr:4:9603240
4	rs3864193	9584474	G	0.1855	1.99E-11	chr:4:9603240
4	rs4102945	9581863	G	0.1855	1.99E-11	chr:4:9603240
4	rs1812335	9608440	G	5.312	2.08E-11	chr:4:9603240

4	rs6856685	9665482	G	5.165	2.08E-11	chr:4:9603240
4	rs13122952	9707740	A	4.578	2.10E-11	chr:4:9603240
4	rs11736658	9585855	G	0.1855	2.16E-11	chr:4:9603240
4	rs13125486	9575367	C	0.186	2.68E-11	chr:4:9603240
4	rs9684683	9691507	T	4.868	3.09E-11	chr:4:9603240
4	rs62409900	9652583	A	4.872	3.11E-11	chr:4:9603240
4	rs1947868	9584808	G	0.1903	3.11E-11	chr:4:9603240
4	rs13103452	9694662	C	4.985	3.26E-11	chr:4:9603240
4	rs6815602	9592093	T	5.093	3.46E-11	chr:4:9603240
4	rs9684596	9695983	G	4.864	3.49E-11	chr:4:9603240
4	rs13117329	9684608	T	4.871	3.66E-11	chr:4:9603240
4	rs11734592	9641381	T	4.863	3.68E-11	chr:4:9603240
4	rs13127386	9636309	T	4.863	3.68E-11	chr:4:9603240
4	rs62411071	9640314	A	4.863	3.68E-11	chr:4:9603240
4	rs62409901	9652707	C	4.858	3.85E-11	chr:4:9603240
4	rs12233698	9680271	C	4.87	3.94E-11	chr:4:9603240
4	rs12645053	9668769	A	4.87	3.94E-11	chr:4:9603240
4	rs12647495	9668881	A	4.87	3.94E-11	chr:4:9603240
4	rs12649399	9668775	A	4.87	3.94E-11	chr:4:9603240
4	rs4274844	9676059	T	4.87	3.94E-11	chr:4:9603240
4	rs6827470	9667892	C	4.87	3.94E-11	chr:4:9603240
4	rs62409886	9642713	T	4.875	4.23E-11	chr:4:9603240
4	rs13132135	9583284	T	5.28	4.39E-11	chr:4:9603240
4	rs11943931	9706478	A	4.584	4.51E-11	chr:4:9603240
4	rs11733344	9670097	T	4.85	4.52E-11	chr:4:9603240
4	rs34172197	9634195	A	4.854	4.75E-11	chr:4:9603240
4	rs11728002	9632904	G	0.2243	4.90E-11	chr:4:9603240
4	rs11731299	9632932	C	0.2243	4.90E-11	chr:4:9603240
4	rs13107086	9628088	G	0.2363	5.82E-11	chr:4:9603240
4	rs150084672	9619998	C	0.2363	5.82E-11	chr:4:9603240
4	rs34407913	9627405	C	0.2363	5.82E-11	chr:4:9603240
4	rs76746438	9631273	C	0.2363	5.82E-11	chr:4:9603240
4	rs12502039	9658319	G	4.714	6.11E-11	chr:4:9603240
4	rs12647777	9664000	T	4.714	6.11E-11	chr:4:9603240
4	rs56141152	9661423	A	4.714	6.11E-11	chr:4:9603240
4	rs13115277	9683696	A	4.713	6.22E-11	chr:4:9603240
4	rs10939461	9681893	G	4.816	7.39E-11	chr:4:9603240
4	rs13141594	9690088	T	4.568	8.20E-11	chr:4:9603240
4	rs13102980	9697402	T	4.49	8.41E-11	chr:4:9603240
4	rs11731648	9691863	T	4.468	8.97E-11	chr:4:9603240
4	rs13135989	9692108	A	4.468	8.97E-11	chr:4:9603240
4	rs11724211	9695175	C	4.474	9.14E-11	chr:4:9603240
4	rs55634110	9643904	T	4.62	9.30E-11	chr:4:9603240

4	rs34113970	9686479	A	4.56	9.43E-11	chr:4:9603240
4	rs6821253	9690731	G	4.546	9.80E-11	chr:4:9603240
4	rs62411066	9638987	A	4.518	1.01E-10	chr:4:9603240
4	rs62411067	9639340	T	4.518	1.01E-10	chr:4:9603240
4	rs13101545	9684171	C	4.556	1.08E-10	chr:4:9603240
4	rs12509448	9577884	G	0.2096	1.15E-10	chr:4:9603240
4	rs1842497	9579673	A	0.2096	1.15E-10	chr:4:9603240
4	rs112714712	9643479	G	4.574	1.23E-10	chr:4:9603240
4	rs57746863	9645578	A	4.486	1.23E-10	chr:4:9603240
4	rs12510096	9646171	G	4.474	1.60E-10	chr:4:9603240
4	rs13141772	9575916	T	6.102	1.85E-10	chr:4:9603240
4	rs11727921	9632675	G	0.2525	1.96E-10	chr:4:9603240
4	rs13129861	9626802	A	0.2525	1.96E-10	chr:4:9603240
4	rs13130488	9627077	A	0.2525	1.96E-10	chr:4:9603240
4	rs13150819	9626909	G	0.2525	1.96E-10	chr:4:9603240
4	rs13151183	9627066	G	0.2525	1.96E-10	chr:4:9603240
4	rs74920290	9631759	A	0.2525	1.96E-10	chr:4:9603240
4	rs75000854	9631079	A	0.2525	1.96E-10	chr:4:9603240
4	rs75569331	9631942	G	0.2525	1.96E-10	chr:4:9603240
4	rs75957763	9631643	C	0.2525	1.96E-10	chr:4:9603240
4	rs7693695	9629380	A	0.2525	1.96E-10	chr:4:9603240
4	rs77675792	9632008	A	0.2525	1.96E-10	chr:4:9603240
4	rs78943848	9624005	T	0.2525	1.96E-10	chr:4:9603240
4	rs11734220	9634271	C	4.173	2.33E-10	chr:4:9603240
4	rs13140984	9637886	A	4.173	2.33E-10	chr:4:9603240
4	rs71615596	9635011	C	4.173	2.33E-10	chr:4:9603240
4	rs12510091	9708661	A	4.205	2.48E-10	chr:4:9603240
4	rs11732596	9569361	T	4.69	2.48E-10	chr:4:9603240
4	rs34554083	9634203	T	4.173	2.52E-10	chr:4:9603240
4	rs11733687	9643374	T	4.48	2.80E-10	chr:4:9603240
4	rs13151395	9709144	T	4.197	2.85E-10	chr:4:9603240
4	rs35737782	9674263	A	4.223	3.03E-10	chr:4:9603240
4	rs13115165	9624808	A	0.2488	3.14E-10	chr:4:9603240
4	rs13123398	9677837	A	4.219	3.38E-10	chr:4:9603240
4	rs62409888	9642756	A	4.133	3.49E-10	chr:4:9603240
4	rs13123294	9614786	C	4.101	3.92E-10	chr:4:9603240
4	rs13134507	9697356	A	4.11	4.04E-10	chr:4:9603240
4	rs13136075	9714968	A	3.971	8.39E-10	chr:4:9603240
4	rs66534644	9711634	G	3.971	8.39E-10	chr:4:9603240
4	rs6843276	9714147	T	3.971	8.39E-10	chr:4:9603240
4	rs12508394	9702186	T	3.947	9.41E-10	chr:4:9603240
4	rs112873333	9623915	T	3.531	1.18E-09	chr:4:9603240
4	rs143193345	9622281	A	3.531	1.18E-09	chr:4:9603240

4	rs13137196	9715456	A	3.855	1.42E-09	chr:4:9603240
4	rs9997490	9529506	G	0.175	1.44E-09	chr:4:9603240
4	rs13147802	9572720	A	3.882	1.52E-09	chr:4:9603240
4	rs4077929	9698730	A	3.843	1.55E-09	chr:4:9603240
4	rs11734448	9563381	T	0.2695	1.59E-09	chr:4:9603240
4	rs13145994	9623518	A	3.484	1.61E-09	chr:4:9603240
4	rs12233884	9550045	G	0.2699	1.67E-09	chr:4:9603240
4	rs13136973	9574572	A	3.876	1.80E-09	chr:4:9603240
4	rs12512447	9586313	A	3.868	2.09E-09	chr:4:9603240
4	rs12233701	9552436	C	0.2778	2.21E-09	chr:4:9603240
4	rs13152465	9556695	G	0.2747	2.48E-09	chr:4:9603240
4	rs10939447	9659957	T	3.728	2.68E-09	chr:4:9603240
4	rs11723541	9556165	C	0.28	2.85E-09	chr:4:9603240
4	rs11728441	9558041	T	0.28	2.85E-09	chr:4:9603240
4	rs12233677	9549872	A	0.28	2.85E-09	chr:4:9603240
4	rs12233678	9549951	A	0.28	2.85E-09	chr:4:9603240
4	rs12233771	9552965	T	0.28	2.85E-09	chr:4:9603240
4	rs13107393	9562217	C	0.28	2.85E-09	chr:4:9603240
4	rs13116764	9561759	A	0.28	2.85E-09	chr:4:9603240
4	rs13152653	9556775	G	0.28	2.85E-09	chr:4:9603240
4	rs7442338	9560945	C	0.28	2.85E-09	chr:4:9603240
4	rs9715222	9564999	T	0.28	2.85E-09	chr:4:9603240
4	rs6448964	9690292	G	4.336	3.10E-09	chr:4:9603240
4	rs1842490	9568457	G	0.2897	5.54E-09	chr:4:9603240
4	rs7434710	9700062	A	3.563	5.64E-09	chr:4:9603240
4	rs11735687	9523738	T	0.2878	5.67E-09	chr:4:9603240
4	rs12505958	9560131	A	0.2932	6.33E-09	chr:4:9603240
4	rs9917965	9614520	A	0.2377	6.59E-09	chr:4:9603240
4	rs13138961	9585147	T	3.687	7.01E-09	chr:4:9603240
4	rs9684707	9569057	G	0.2916	7.40E-09	chr:4:9603240
4	rs12641450	9529244	G	0.2921	8.29E-09	chr:4:9603240
4	rs13124998	9530491	G	0.2921	8.29E-09	chr:4:9603240
4	rs13136346	9528694	C	0.2921	8.29E-09	chr:4:9603240
4	rs13144880	9530438	C	0.2921	8.29E-09	chr:4:9603240
4	rs9684176	9716386	G	3.426	8.99E-09	chr:4:9603240
4	rs6820385	9606357	G	0.2423	9.72E-09	chr:4:9603240
4	rs12233883	9549927	G	0.3003	9.85E-09	chr:4:9603240
4	rs13110253	9558556	G	0.3003	9.85E-09	chr:4:9603240
4	rs13132153	9561479	G	0.3003	9.85E-09	chr:4:9603240
4	rs13138276	9562004	G	0.3003	9.85E-09	chr:4:9603240
4	rs1880831	9568145	C	0.3003	9.85E-09	chr:4:9603240
4	rs4102942	9568478	C	0.3003	9.85E-09	chr:4:9603240
4	rs113906552	9635435	C	4.207	1.07E-08	chr:4:9603240

4	rs13137016	9715392	A	3.508	1.10E-08	chr:4:9603240
4	rs11731863	9495756	C	3.351	1.15E-08	chr:4:9603240
4	rs7692046	9620128	G	0.2606	1.18E-08	chr:4:9603240
4	rs138499118	9631591	C	0.2622	1.21E-08	chr:4:9603240
4	rs6448856	9595797	A	0.2442	1.22E-08	chr:4:9603240
4	rs7672211	9597825	A	0.2442	1.22E-08	chr:4:9603240
4	rs10939182	9511068	A	0.2946	1.52E-08	chr:4:9603240
4	rs2127796	9510832	C	0.2946	1.52E-08	chr:4:9603240
4	rs10031125	9625879	A	0.2653	1.60E-08	chr:4:9603240
4	rs10049735	9626409	C	0.2653	1.60E-08	chr:4:9603240
4	rs114980491	9623328	T	0.2653	1.60E-08	chr:4:9603240
4	rs115870066	9622163	A	0.2653	1.60E-08	chr:4:9603240
4	rs147074482	9623653	T	0.2653	1.60E-08	chr:4:9603240
4	rs150602585	9621799	G	0.2653	1.60E-08	chr:4:9603240
4	rs151134492	9623039	A	0.2653	1.60E-08	chr:4:9603240
4	rs28491253	9629512	T	0.2653	1.60E-08	chr:4:9603240
4	rs28648949	9626132	C	0.2653	1.60E-08	chr:4:9603240
4	rs28851772	9630974	A	0.2653	1.60E-08	chr:4:9603240
4	rs56787100	9631153	T	0.2653	1.60E-08	chr:4:9603240
4	rs73082825	9627676	A	0.2653	1.60E-08	chr:4:9603240
4	rs13118864	9521311	T	0.3069	1.61E-08	chr:4:9603240
4	rs11733952	9508241	G	0.2978	1.69E-08	chr:4:9603240
4	rs12513044	9506678	A	0.2978	1.69E-08	chr:4:9603240
4	rs62410329	9504003	C	0.2978	1.69E-08	chr:4:9603240
4	rs28784595	9646182	C	0.252	1.90E-08	chr:4:9603240
4	rs13150941	9694401	G	0.2985	1.91E-08	chr:4:9603240
4	rs9790487	9516638	T	0.2964	2.06E-08	chr:4:9603240
4	rs6448858	9595918	T	0.2516	2.21E-08	chr:4:9603240
4	rs10939280	9529545	T	0.3029	2.29E-08	chr:4:9603240
4	rs11724460	9509698	A	0.2998	2.41E-08	chr:4:9603240
4	rs11722359	9508214	A	0.3036	2.52E-08	chr:4:9603240
4	rs11737117	9507890	C	0.3036	2.52E-08	chr:4:9603240
4	rs13120116	9694454	C	0.3158	2.99E-08	chr:4:9603240
4	rs11722239	9482356	C	3.337	3.04E-08	chr:4:9603240
4	rs6848533	9690289	T	3.742	3.17E-08	chr:4:9603240
4	rs10007689	9487133	G	3.339	4.17E-08	chr:4:9603240
4	rs1811570	9587999	A	0.2761	4.37E-08	chr:4:9603240
4	rs28694172	9628973	C	0.2669	4.43E-08	chr:4:9603240
4	rs11722202	9443894	T	3.263	4.84E-08	chr:4:9603240
4	rs12645738	9529669	A	0.3198	4.85E-08	chr:4:9603240
4	rs7437720	9510396	A	0.3007	4.94E-08	chr:4:9603240
4	rs7692776	9506201	G	3.239	5.00E-08	chr:4:9603240
4	rs6842850	9788598	A	0.3173	5.42E-08	chr:4:9603240

4	rs13147425	9492966	A	3.227	5.46E-08	chr:4:9603240
4	rs12506692	9596059	T	0.2715	6.13E-08	chr:4:9603240
4	rs12648831	9447802	A	3.201	6.55E-08	chr:4:9603240
4	rs10938806	9439761	C	3.286	6.60E-08	chr:4:9603240
4	rs6840249	9578971	G	0.2702	7.30E-08	chr:4:9603240
4	rs111613333	9592284	A	0.2722	7.62E-08	chr:4:9603240
4	rs12510176	9599593	G	0.2722	7.62E-08	chr:4:9603240
4	rs61485794	9592346	G	0.2722	7.62E-08	chr:4:9603240
4	rs73097911	9601929	A	0.2722	7.62E-08	chr:4:9603240
4	rs74183920	9603786	A	0.2722	7.62E-08	chr:4:9603240
4	rs13139813	9547169	C	0.3346	7.70E-08	chr:4:9603240
4	rs6814282	9692516	C	0.3326	7.83E-08	chr:4:9603240
4	rs9684173	9691519	G	0.3326	7.83E-08	chr:4:9603240
4	rs55760760	9503473	G	3.144	8.24E-08	chr:4:9603240
4	rs61175357	9434500	A	3.199	9.94E-08	chr:4:9603240
4	rs12501880	9717975	T	3.061	1.04E-07	chr:4:9603240
4	rs28851574	9644580	C	0.2707	1.07E-07	chr:4:9603240
4	rs11735679	9585384	G	0.2919	1.12E-07	chr:4:9603240
4	rs13118242	9585316	A	0.2919	1.12E-07	chr:4:9603240
4	rs1947866	9584747	G	0.2919	1.12E-07	chr:4:9603240
4	rs6855045	9583518	T	0.2919	1.12E-07	chr:4:9603240
4	rs9685853	9585655	G	0.2919	1.12E-07	chr:4:9603240
4	rs4698055	9627821	A	0.3459	1.15E-07	chr:4:9603240
4	rs6448981	9740325	G	0.3267	1.16E-07	chr:4:9603240
4	rs13119693	9451151	T	3.17	1.17E-07	chr:4:9603240
4	rs10938861	9450013	C	3.231	1.22E-07	chr:4:9603240
4	rs59507413	9606018	T	0.2749	1.35E-07	chr:4:9603240
4	rs1976792	9846309	A	0.2956	1.42E-07	chr:4:9603240
4	rs145014366	9588718	C	0.2783	1.45E-07	chr:4:9603240
4	rs6830692	9589873	T	0.1732	1.47E-07	chr:4:9603240
4	rs13114077	9413105	C	3.166	1.49E-07	chr:4:9603240
4	rs112839156	9601686	A	0.2826	1.57E-07	chr:4:9603240
4	rs12509677	9602724	A	0.2826	1.57E-07	chr:4:9603240
4	rs56934553	9594735	A	0.2826	1.57E-07	chr:4:9603240
4	rs60296945	9604068	T	0.2826	1.57E-07	chr:4:9603240
4	rs60990443	9601319	T	0.2826	1.57E-07	chr:4:9603240
4	rs73805539	9591699	C	0.2826	1.57E-07	chr:4:9603240
4	rs7691087	9595443	T	0.2826	1.57E-07	chr:4:9603240
4	rs11724549	9484511	A	3.129	1.59E-07	chr:4:9603240
4	rs10014826	9635408	C	0.279	1.71E-07	chr:4:9603240
4	rs12505906	9636906	A	0.279	1.71E-07	chr:4:9603240
4	rs143900004	9636160	T	0.279	1.71E-07	chr:4:9603240
4	rs150921853	9639169	T	0.279	1.71E-07	chr:4:9603240

4	rs28735278	9614378	A	0.279	1.71E-07	chr:4:9603240
4	rs6839392	9613589	T	0.279	1.71E-07	chr:4:9603240
4	rs73082901	9636762	C	0.279	1.71E-07	chr:4:9603240
4	rs73084810	9642699	G	0.279	1.71E-07	chr:4:9603240
4	rs76266909	9636303	C	0.279	1.71E-07	chr:4:9603240
4	rs142277140	9648076	C	0.2797	1.77E-07	chr:4:9603240
4	rs28577902	9650657	A	0.2797	1.77E-07	chr:4:9603240
4	rs73084818	9648875	T	0.2797	1.77E-07	chr:4:9603240
4	rs10011678	9688689	C	0.2803	1.80E-07	chr:4:9603240
4	rs13148606	9660864	C	0.2527	1.80E-07	chr:4:9603240
4	rs13131004	9612911	C	0.308	1.89E-07	chr:4:9603240
4	rs185725801	9588562	C	0.2465	2.02E-07	chr:4:9603240
4	rs112018355	9611157	A	0.2809	2.07E-07	chr:4:9603240
4	rs2865009	9522285	A	2.886	2.16E-07	chr:4:9603240
4	rs13110996	9726276	C	0.3373	2.18E-07	chr:4:9603240
4	rs12171434	9647060	T	0.2831	2.31E-07	chr:4:9603240
4	rs6817739	9442163	T	3.019	2.37E-07	chr:4:9603240
4	rs16898588	9595751	T	0.2884	2.38E-07	chr:4:9603240
4	rs59430272	9601484	C	0.2884	2.38E-07	chr:4:9603240
4	rs61267391	9600955	G	0.2884	2.38E-07	chr:4:9603240
4	rs73095794	9596566	A	0.2884	2.38E-07	chr:4:9603240
4	rs73802916	9604035	C	0.2884	2.38E-07	chr:4:9603240
4	rs28886660	9644540	G	0.284	2.40E-07	chr:4:9603240
4	rs11729203	9569091	T	2.949	2.53E-07	chr:4:9603240
4	rs10005582	9626791	A	0.3066	2.65E-07	chr:4:9603240
4	rs2865076	9591550	C	0.3041	2.65E-07	chr:4:9603240
4	rs6845333	9614058	C	0.3139	2.66E-07	chr:4:9603240
4	rs10939423	9627362	A	0.2711	2.95E-07	chr:4:9603240
4	rs113355114	9631117	A	0.2711	2.95E-07	chr:4:9603240
4	rs11939895	9630402	T	0.2711	2.95E-07	chr:4:9603240
4	rs6448881	9621124	T	0.2711	2.95E-07	chr:4:9603240
4	rs9995183	9625793	C	0.2711	2.95E-07	chr:4:9603240
4	rs9998663	9627053	C	0.2711	2.95E-07	chr:4:9603240
4	rs4482762	9615140	A	0.3125	3.00E-07	chr:4:9603240
4	rs10001941	9688516	A	0.2884	3.02E-07	chr:4:9603240
4	rs10024971	9694013	G	0.2884	3.02E-07	chr:4:9603240
4	rs12504246	9692932	A	0.2884	3.02E-07	chr:4:9603240
4	rs73088893	9695306	A	0.2884	3.02E-07	chr:4:9603240
4	rs139901069	9582722	A	0.2899	3.20E-07	chr:4:9603240
4	rs77648749	9583407	A	0.2899	3.20E-07	chr:4:9603240
4	rs10027164	9574763	A	0.2917	3.28E-07	chr:4:9603240
4	rs6448846	9580937	G	0.2917	3.28E-07	chr:4:9603240
4	rs73084845	9671025	T	0.2938	3.84E-07	chr:4:9603240

4	rs143582873	9624071	G	0.2782	3.87E-07	chr:4:9603240
4	rs36162019	9625137	C	0.2782	3.87E-07	chr:4:9603240
4	rs6837106	9626304	T	0.2782	3.87E-07	chr:4:9603240
4	rs6837700	9626577	T	0.2782	3.87E-07	chr:4:9603240
4	rs9999630	9625826	A	0.2782	3.87E-07	chr:4:9603240
4	rs73097914	9602324	T	0.2986	3.88E-07	chr:4:9603240
4	rs10026706	9674011	A	0.2933	4.02E-07	chr:4:9603240
4	rs12501304	9682548	T	0.2933	4.02E-07	chr:4:9603240
4	rs143459149	9674515	G	0.2933	4.02E-07	chr:4:9603240
4	rs201078836	9680043	T	0.2933	4.02E-07	chr:4:9603240
4	rs28546201	9684768	T	0.2933	4.02E-07	chr:4:9603240
4	rs28673739	9677529	T	0.2933	4.02E-07	chr:4:9603240
4	rs28691200	9684552	A	0.2933	4.02E-07	chr:4:9603240
4	rs80077135	9681299	G	0.2933	4.02E-07	chr:4:9603240
4	rs9291600	9675669	A	0.2933	4.02E-07	chr:4:9603240
4	rs28793127	9660482	T	0.2913	4.12E-07	chr:4:9603240
4	rs73805537	9588589	C	0.2556	4.30E-07	chr:4:9603240
4	rs7693691	9652934	C	0.2954	4.47E-07	chr:4:9603240
4	rs3864192	9573978	A	0.2985	4.64E-07	chr:4:9603240
4	rs373327909	9241924	T	2.944	4.86E-07	chr:4:9603240
4	rs10013863	9667436	T	0.2962	4.91E-07	chr:4:9603240
4	rs9884683	9677137	A	0.2962	4.91E-07	chr:4:9603240
4	rs59312718	9669434	T	0.2979	5.13E-07	chr:4:9603240
4	rs73084842	9671015	T	0.2979	5.13E-07	chr:4:9603240
4	rs884573	9869734	C	0.3205	5.17E-07	chr:4:9603240
4	rs4406018	9645618	A	0.2651	5.35E-07	chr:4:9603240
4	rs13129974	9779526	C	2.901	5.69E-07	chr:4:9603240
4	rs143079510	9665042	T	0.2988	5.76E-07	chr:4:9603240
4	rs150693830	9665588	T	0.2988	5.76E-07	chr:4:9603240
4	rs28575773	9656525	A	0.2988	5.76E-07	chr:4:9603240
4	rs28643508	9659221	C	0.2988	5.76E-07	chr:4:9603240
4	rs28718562	9659961	G	0.2988	5.76E-07	chr:4:9603240
4	rs4518243	9664634	C	0.2988	5.76E-07	chr:4:9603240
4	rs73802943	9663020	C	0.2988	5.76E-07	chr:4:9603240
4	rs77025480	9657754	A	0.2988	5.76E-07	chr:4:9603240
4	rs55988830	9588757	C	0.2622	5.94E-07	chr:4:9603240
4	rs73802915	9603200	A	0.2635	6.59E-07	chr:4:9603240
4	rs56323944	9642872	T	0.2657	6.97E-07	chr:4:9603240
4	rs10012148	9664294	A	0.3017	7.02E-07	chr:4:9603240
4	rs28796640	9663788	T	0.3017	7.02E-07	chr:4:9603240
4	rs146389512	9597589	A	0.264	7.14E-07	chr:4:9603240
4	rs6824573	9589256	A	0.3117	7.70E-07	chr:4:9603240
4	rs28380563	9658392	T	0.3008	7.82E-07	chr:4:9603240

4	rs12643412	9420974	G	2.799	7.95E-07	chr:4:9603240
4	rs12512543	9608445	A	0.3076	8.02E-07	chr:4:9603240
4	rs9996676	9678661	C	0.3084	8.04E-07	chr:4:9603240
4	rs11734925	9644507	G	0.2721	8.07E-07	chr:4:9603240
4	rs7668654	9744591	T	0.366	8.45E-07	chr:4:9603240
4	rs13112576	9781183	T	2.839	8.67E-07	chr:4:9603240
4	rs6835741	9778911	A	2.839	8.67E-07	chr:4:9603240
4	rs10002984	9680733	T	0.2738	8.82E-07	chr:4:9603240
4	rs10014571	9662181	C	0.2738	8.82E-07	chr:4:9603240
4	rs10939458	9677951	T	0.2738	8.82E-07	chr:4:9603240
4	rs6844051	9677232	C	0.2738	8.82E-07	chr:4:9603240
4	rs6855355	9672120	A	0.2738	8.82E-07	chr:4:9603240
4	rs4974853	9609115	C	0.273	8.82E-07	chr:4:9603240
4	rs4547793	9591708	C	0.2728	9.48E-07	chr:4:9603240
4	rs10939427	9628538	G	0.28	9.53E-07	chr:4:9603240
4	rs114344194	9606720	C	0.273	1.00E-06	chr:4:9603240
4	rs11724183	9609894	G	0.273	1.00E-06	chr:4:9603240
4	rs11726515	9611214	C	0.273	1.00E-06	chr:4:9603240
4	rs11733660	9608377	C	0.273	1.00E-06	chr:4:9603240
4	rs11735475	9614926	T	0.273	1.00E-06	chr:4:9603240
4	rs13128072	9636572	G	0.273	1.00E-06	chr:4:9603240
4	rs139590336	9612695	A	0.273	1.00E-06	chr:4:9603240
4	rs142443233	9605502	T	0.273	1.00E-06	chr:4:9603240
4	rs143734288	9611958	C	0.273	1.00E-06	chr:4:9603240
4	rs1961552	9608306	C	0.273	1.00E-06	chr:4:9603240
4	rs2077679	9608089	A	0.273	1.00E-06	chr:4:9603240
4	rs4974812	9614598	C	0.273	1.00E-06	chr:4:9603240
4	rs4974816	9613067	A	0.273	1.00E-06	chr:4:9603240
4	rs4974818	9612995	T	0.273	1.00E-06	chr:4:9603240
4	rs4974854	9609087	G	0.273	1.00E-06	chr:4:9603240
4	rs4974856	9607691	C	0.273	1.00E-06	chr:4:9603240
4	rs4974858	9607122	G	0.273	1.00E-06	chr:4:9603240
4	rs55934031	9613756	A	0.273	1.00E-06	chr:4:9603240
4	rs56746271	9610873	T	0.273	1.00E-06	chr:4:9603240
4	rs56873393	9606840	A	0.273	1.00E-06	chr:4:9603240
4	rs62409884	9642370	T	0.273	1.00E-06	chr:4:9603240
4	rs62411065	9638952	C	0.273	1.00E-06	chr:4:9603240
4	rs6836196	9633728	A	0.273	1.00E-06	chr:4:9603240
4	rs6837988	9634543	A	0.273	1.00E-06	chr:4:9603240
4	rs7375281	9609506	T	0.273	1.00E-06	chr:4:9603240
4	rs7375292	9609618	T	0.273	1.00E-06	chr:4:9603240
4	rs9799674	9606025	C	0.273	1.00E-06	chr:4:9603240
4	rs1048252	9870535	T	0.3294	1.01E-06	chr:4:9603240

4	rs57235613	9867708	T	0.3294	1.01E-06	chr:4:9603240
4	rs141090236	9597872	G	0.2695	1.02E-06	chr:4:9603240
4	rs28728462	9700392	A	0.3438	1.11E-06	chr:4:9603240
4	rs112878601	9643563	G	0.2731	1.11E-06	chr:4:9603240
4	rs7684741	9661843	A	0.3054	1.11E-06	chr:4:9603240
4	rs13131893	9744301	G	0.3785	1.20E-06	chr:4:9603240
4	rs114290083	9597994	T	0.2731	1.22E-06	chr:4:9603240
4	rs114945335	9598191	T	0.2731	1.22E-06	chr:4:9603240
4	rs115282360	9593376	T	0.2731	1.22E-06	chr:4:9603240
4	rs115893429	9593818	A	0.2731	1.22E-06	chr:4:9603240
4	rs116079424	9593204	C	0.2731	1.22E-06	chr:4:9603240
4	rs116509172	9594123	G	0.2731	1.22E-06	chr:4:9603240
4	rs116614494	9592153	T	0.2731	1.22E-06	chr:4:9603240
4	rs11730426	9600216	T	0.2731	1.22E-06	chr:4:9603240
4	rs11731628	9601348	T	0.2731	1.22E-06	chr:4:9603240
4	rs11734986	9601497	T	0.2731	1.22E-06	chr:4:9603240
4	rs11735905	9601757	G	0.2731	1.22E-06	chr:4:9603240
4	rs11737520	9601215	C	0.2731	1.22E-06	chr:4:9603240
4	rs117496616	9597238	C	0.2731	1.22E-06	chr:4:9603240
4	rs137855378	9594243	T	0.2731	1.22E-06	chr:4:9603240
4	rs138143241	9596395	T	0.2731	1.22E-06	chr:4:9603240
4	rs138542917	9592759	G	0.2731	1.22E-06	chr:4:9603240
4	rs139555537	9593067	T	0.2731	1.22E-06	chr:4:9603240
4	rs141001435	9598071	C	0.2731	1.22E-06	chr:4:9603240
4	rs142642407	9591168	A	0.2731	1.22E-06	chr:4:9603240
4	rs143023917	9596151	G	0.2731	1.22E-06	chr:4:9603240
4	rs143167350	9594342	T	0.2731	1.22E-06	chr:4:9603240
4	rs144619625	9593363	C	0.2731	1.22E-06	chr:4:9603240
4	rs147268581	9593254	T	0.2731	1.22E-06	chr:4:9603240
4	rs147777706	9597845	A	0.2731	1.22E-06	chr:4:9603240
4	rs148075169	9599421	A	0.2731	1.22E-06	chr:4:9603240
4	rs148536222	9592032	T	0.2731	1.22E-06	chr:4:9603240
4	rs149064572	9592378	A	0.2731	1.22E-06	chr:4:9603240
4	rs149095671	9597966	C	0.2731	1.22E-06	chr:4:9603240
4	rs149737988	9599039	A	0.2731	1.22E-06	chr:4:9603240
4	rs149930076	9591979	T	0.2731	1.22E-06	chr:4:9603240
4	rs150736073	9590365	C	0.2731	1.22E-06	chr:4:9603240
4	rs185210675	9598974	G	0.2731	1.22E-06	chr:4:9603240
4	rs190948268	9591497	T	0.2731	1.22E-06	chr:4:9603240
4	rs200437010	9592158	G	0.2731	1.22E-06	chr:4:9603240
4	rs34495260	9595324	C	0.2731	1.22E-06	chr:4:9603240
4	rs34587049	9594498	T	0.2731	1.22E-06	chr:4:9603240
4	rs35023412	9594927	G	0.2731	1.22E-06	chr:4:9603240

4	rs35228193	9595003	T	0.2731	1.22E-06	chr:4:9603240
4	rs35332449	9594812	G	0.2731	1.22E-06	chr:4:9603240
4	rs35509001	9594871	A	0.2731	1.22E-06	chr:4:9603240
4	rs35583251	9595042	C	0.2731	1.22E-06	chr:4:9603240
4	rs35870323	9600326	C	0.2731	1.22E-06	chr:4:9603240
4	rs4974823	9602719	A	0.2731	1.22E-06	chr:4:9603240
4	rs4974824	9602608	C	0.2731	1.22E-06	chr:4:9603240
4	rs4974825	9602228	T	0.2731	1.22E-06	chr:4:9603240
4	rs4974826	9602042	T	0.2731	1.22E-06	chr:4:9603240
4	rs4974859	9604519	T	0.2731	1.22E-06	chr:4:9603240
4	rs4974861	9604139	T	0.2731	1.22E-06	chr:4:9603240
4	rs4974863	9590979	T	0.2731	1.22E-06	chr:4:9603240
4	rs552645918	9594483	A	0.2731	1.22E-06	chr:4:9603240
4	rs55813730	9595618	A	0.2731	1.22E-06	chr:4:9603240
4	rs55916508	9589334	A	0.2731	1.22E-06	chr:4:9603240
4	rs56055745	9595453	T	0.2731	1.22E-06	chr:4:9603240
4	rs56411540	9595441	C	0.2731	1.22E-06	chr:4:9603240
4	rs57171822	9596455	A	0.2731	1.22E-06	chr:4:9603240
4	rs59747264	9595447	G	0.2731	1.22E-06	chr:4:9603240
4	rs73802912	9602497	A	0.2731	1.22E-06	chr:4:9603240
4	rs73805538	9591438	C	0.2731	1.22E-06	chr:4:9603240
4	rs73805540	9593964	G	0.2731	1.22E-06	chr:4:9603240
4	rs75043790	9597656	G	0.2731	1.22E-06	chr:4:9603240
4	rs76304666	9590479	A	0.2731	1.22E-06	chr:4:9603240
4	rs76469957	9597617	G	0.2731	1.22E-06	chr:4:9603240
4	rs76738832	9597809	A	0.2731	1.22E-06	chr:4:9603240
4	rs78422890	9600765	T	0.2731	1.22E-06	chr:4:9603240
4	rs9799500	9595891	A	0.2731	1.22E-06	chr:4:9603240
4	rs9799502	9595942	A	0.2731	1.22E-06	chr:4:9603240
4	rs9799575	9592812	A	0.2731	1.22E-06	chr:4:9603240
4	rs9799622	9595641	G	0.2731	1.22E-06	chr:4:9603240
4	rs9799684	9592993	T	0.2731	1.22E-06	chr:4:9603240
4	rs9799753	9592618	A	0.2731	1.22E-06	chr:4:9603240
4	rs9995735	9658789	T	0.3112	1.24E-06	chr:4:9603240
4	rs188796369	9854038	A	0.3342	1.27E-06	chr:4:9603240
4	rs73802906	9598448	T	0.2721	1.36E-06	chr:4:9603240
4	rs4974815	9614158	T	0.2735	1.37E-06	chr:4:9603240
4	rs13109135	9549749	T	2.616	1.41E-06	chr:4:9603240
4	rs1825043	9573740	G	0.3324	1.42E-06	chr:4:9603240
4	rs531217356	9591411	A	0.2769	1.44E-06	chr:4:9603240
4	rs60403942	9603452	G	0.2756	1.44E-06	chr:4:9603240
4	rs112123699	9624028	G	0.2833	1.59E-06	chr:4:9603240
4	rs7654169	9654071	T	0.2841	1.64E-06	chr:4:9603240

4	rs16898647	9599609	A	0.2801	1.66E-06	chr:4:9603240
4	rs149630666	9598726	T	0.2718	1.68E-06	chr:4:9603240
4	rs4563493	9743604	G	0.3835	1.70E-06	chr:4:9603240
4	rs7435681	9743362	T	0.3835	1.70E-06	chr:4:9603240
4	rs13119328	9751860	C	0.3752	1.81E-06	chr:4:9603240
4	rs13150238	9751763	G	0.3752	1.81E-06	chr:4:9603240
4	rs11942665	9742852	G	0.3886	1.90E-06	chr:4:9603240
4	rs9799747	9592874	C	0.281	1.92E-06	chr:4:9603240
4	rs9799779	9592870	A	0.281	1.92E-06	chr:4:9603240
4	rs56293131	9652279	G	0.3018	2.17E-06	chr:4:9603240
4	rs10014408	9695145	T	0.2924	2.24E-06	chr:4:9603240
4	rs6853779	9684468	A	0.2924	2.24E-06	chr:4:9603240
4	rs9761757	9696996	T	0.2924	2.24E-06	chr:4:9603240
4	rs6848710	9670562	T	0.3441	2.30E-06	chr:4:9603240
4	rs13101778	9521143	T	2.777	2.40E-06	chr:4:9603240
4	rs34441511	9738007	G	0.3926	2.41E-06	chr:4:9603240
4	rs4594723	9700910	A	0.3085	2.44E-06	chr:4:9603240
4	rs10022843	9604948	C	0.3506	2.69E-06	chr:4:9603240
4	rs11733905	9603159	A	0.291	2.70E-06	chr:4:9603240
4	rs142223268	9624727	C	0.329	2.73E-06	chr:4:9603240
4	rs4697892	9823837	A	0.366	2.94E-06	chr:4:9603240
4	rs57731161	9825847	T	0.3751	3.14E-06	chr:4:9603240
4	rs7440232	9651744	T	0.3038	3.25E-06	chr:4:9603240
4	rs10939390	9590105	G	0.3543	3.34E-06	chr:4:9603240
4	rs13101251	9588969	T	0.3543	3.34E-06	chr:4:9603240
4	rs6824806	9589427	G	0.3543	3.34E-06	chr:4:9603240
4	rs12506622	9690369	T	0.3057	3.85E-06	chr:4:9603240
4	rs10939424	9627379	G	0.346	3.85E-06	chr:4:9603240
4	rs13150882	9621101	T	2.626	4.11E-06	chr:4:9603240
4	rs56884786	9736751	C	0.4048	4.23E-06	chr:4:9603240
4	rs9291604	9696677	T	0.3087	4.34E-06	chr:4:9603240
4	rs10005112	9732610	G	0.4054	4.45E-06	chr:4:9603240
4	rs10939481	9735100	C	0.4054	4.45E-06	chr:4:9603240
4	rs10939482	9735217	C	0.4054	4.45E-06	chr:4:9603240
4	rs13127001	9731215	G	0.4054	4.45E-06	chr:4:9603240
4	rs13132835	9729181	C	0.4054	4.45E-06	chr:4:9603240
4	rs10939457	9677914	T	0.3594	4.46E-06	chr:4:9603240
4	rs13138869	9674967	T	0.3594	4.46E-06	chr:4:9603240
4	rs13139079	9675072	T	0.3594	4.46E-06	chr:4:9603240
4	rs7435906	9673736	A	0.3594	4.46E-06	chr:4:9603240
4	rs9884921	9676740	A	0.3594	4.46E-06	chr:4:9603240
4	rs13113655	9751629	C	0.398	4.58E-06	chr:4:9603240
4	rs13122793	9748511	G	0.4025	4.60E-06	chr:4:9603240

4	rs7677159	9745919	C	0.4025	4.60E-06	chr:4:9603240
4	rs58934247	9589730	C	0.3049	4.65E-06	chr:4:9603240
4	rs6448296	9478331	C	0.4233	4.68E-06	chr:4:9603240
4	rs16889260	9834896	T	0.3754	4.82E-06	chr:4:9603240
4	rs4554077	9674736	G	0.3128	5.15E-06	chr:4:9603240
4	rs6812811	9672000	T	0.3128	5.15E-06	chr:4:9603240
4	rs6843809	9655294	G	0.3128	5.15E-06	chr:4:9603240
4	rs6858093	9681650	G	0.3128	5.15E-06	chr:4:9603240
4	rs7690455	9758860	A	0.3975	5.29E-06	chr:4:9603240
4	rs3890174	9700774	G	0.3718	5.33E-06	chr:4:9603240
4	rs13140838	9732930	G	0.4105	5.38E-06	chr:4:9603240
4	rs1967550	9785265	T	2.544	5.52E-06	chr:4:9603240
4	rs11728148	9718971	C	0.3596	5.62E-06	chr:4:9603240
4	rs34027818	9605709	G	0.325	5.67E-06	chr:4:9603240
4	rs113957727	9840568	G	0.3717	5.73E-06	chr:4:9603240
4	rs7673959	9620145	G	0.3694	5.81E-06	chr:4:9603240
4	rs13106539	9797703	T	2.509	5.93E-06	chr:4:9603240
4	rs2280206	9799237	C	2.509	5.93E-06	chr:4:9603240
4	rs2280207	9799776	C	2.509	5.93E-06	chr:4:9603240
4	rs2867394	9872475	A	0.413	5.96E-06	chr:4:9603240
4	rs114809283	9578006	A	0.2607	6.21E-06	chr:4:9603240
4	rs9685067	9535838	T	0.2843	6.32E-06	chr:4:9603240
4	rs4554078	9701544	A	0.3126	6.67E-06	chr:4:9603240
4	rs950310	9842850	A	0.3749	6.91E-06	chr:4:9603240
4	rs13124305	9773273	G	0.4129	6.92E-06	chr:4:9603240
4	rs4697692	9893197	T	0.414	7.18E-06	chr:4:9603240
4	rs10009838	9833882	G	0.4225	7.64E-06	chr:4:9603240
4	rs10012516	9797178	A	2.496	7.68E-06	chr:4:9603240
4	rs1829413	9792339	A	2.496	7.68E-06	chr:4:9603240
4	rs58724508	9642207	G	0.3884	7.98E-06	chr:4:9603240
4	rs140355667	9578231	G	0.28	8.40E-06	chr:4:9603240
4	rs12644047	9893403	A	0.4225	8.48E-06	chr:4:9603240
4	rs6448880	9621023	T	0.373	8.78E-06	chr:4:9603240
4	rs374118392	9176144	G	0.5048	8.88E-06	chr:4:9603240
4	rs368238943	9590182	T	0.2959	8.92E-06	chr:4:9603240
4	rs6823671	9692517	A	0.3252	9.12E-06	chr:4:9603240
4	rs6829292	9692892	A	0.3252	9.12E-06	chr:4:9603240
4	rs7678732	9693253	T	0.3252	9.12E-06	chr:4:9603240
4	rs7671706	9749742	T	0.4111	9.13E-06	chr:4:9603240
4	rs10939567	9854749	A	0.4202	9.21E-06	chr:4:9603240
4	rs4622998	9621659	C	0.3831	9.32E-06	chr:4:9603240
4	rs10022923	9773438	G	0.4187	9.78E-06	chr:4:9603240
4	rs4621429	9826870	G	0.392	9.96E-06	chr:4:9603240

4	rs4697895	9828484	C	0.392	9.96E-06	chr:4:9603240
4	rs10939552	9838548	A	0.3991	1.00E-05	chr:4:9603240
4	rs12505312	9837090	G	0.3991	1.00E-05	chr:4:9603240
4	rs2139243	9867502	G	0.4281	1.05E-05	chr:4:9603240
4	rs115723792	9570902	G	0.2702	1.05E-05	chr:4:9603240
4	rs11729940	9574743	A	0.2702	1.05E-05	chr:4:9603240
4	rs140501910	9578396	C	0.2702	1.05E-05	chr:4:9603240
4	rs28729250	9584227	A	0.2702	1.05E-05	chr:4:9603240
4	rs4974828	9572320	G	0.2702	1.05E-05	chr:4:9603240
4	rs4974829	9571976	T	0.2702	1.05E-05	chr:4:9603240
4	rs4974865	9587937	A	0.2702	1.05E-05	chr:4:9603240
4	rs4974868	9572178	A	0.2702	1.05E-05	chr:4:9603240
4	rs11721988	9848899	G	0.3951	1.09E-05	chr:4:9603240
4	rs11723024	9848849	A	0.3951	1.09E-05	chr:4:9603240
4	rs2176644	9849528	T	0.3951	1.09E-05	chr:4:9603240
4	rs4697898	9851827	T	0.3951	1.09E-05	chr:4:9603240
4	rs4697899	9851875	A	0.3951	1.09E-05	chr:4:9603240
4	rs4407504	9717360	C	0.3303	1.09E-05	chr:4:9603240
4	rs10939599	9886860	A	0.4225	1.15E-05	chr:4:9603240
4	rs6840999	9880169	A	0.4225	1.15E-05	chr:4:9603240
4	rs938562	9891031	T	0.4225	1.15E-05	chr:4:9603240
4	rs10001006	9788498	T	2.443	1.15E-05	chr:4:9603240
4	rs10006201	9621908	G	0.3671	1.15E-05	chr:4:9603240
4	rs13118112	9794580	T	2.443	1.15E-05	chr:4:9603240
4	rs12507861	9777590	G	0.4212	1.17E-05	chr:4:9603240
4	rs6856661	9689011	T	0.3824	1.19E-05	chr:4:9603240
4	rs7671092	9687441	C	0.3824	1.19E-05	chr:4:9603240
4	rs7686078	9687410	T	0.3824	1.19E-05	chr:4:9603240
4	rs6829800	9685856	A	0.3323	1.20E-05	chr:4:9603240
4	rs7691166	9687673	T	0.3323	1.20E-05	chr:4:9603240
4	rs140729951	9521179	A	0.3449	1.22E-05	chr:4:9603240
4	rs10012224	9569324	A	0.3479	1.29E-05	chr:4:9603240
4	rs1107710	9890708	G	0.4246	1.35E-05	chr:4:9603240
4	rs6818572	9889448	A	0.4246	1.35E-05	chr:4:9603240
4	rs1980220	9824636	A	0.4009	1.36E-05	chr:4:9603240
4	rs77388911	9842207	A	0.4051	1.38E-05	chr:4:9603240
4	rs13105347	9537183	C	0.279	1.42E-05	chr:4:9603240
4	rs6822409	9536012	T	0.279	1.42E-05	chr:4:9603240
4	rs6815039	9777809	T	0.4236	1.42E-05	chr:4:9603240
4	rs1568318	9871541	C	0.4291	1.43E-05	chr:4:9603240
4	rs13124141	9773237	G	0.4259	1.46E-05	chr:4:9603240
4	rs6825221	9589620	G	0.3781	1.49E-05	chr:4:9603240
4	rs13143667	9781230	T	2.468	1.53E-05	chr:4:9603240

4	rs75856105	9666491	T	0.35	1.53E-05	chr:4:9603240
4	rs11729914	9721691	C	0.4356	1.53E-05	chr:4:9603240
4	rs28627624	9637607	T	0.4033	1.55E-05	chr:4:9603240
4	rs13141123	9757904	T	0.426	1.56E-05	chr:4:9603240
4	rs188525933	9575159	A	0.28	1.58E-05	chr:4:9603240
4	rs56205495	9789501	C	2.458	1.61E-05	chr:4:9603240
4	rs28430858	9626149	A	0.3856	1.63E-05	chr:4:9603240
4	rs1106058	9861524	A	0.4329	1.66E-05	chr:4:9603240
4	rs9685887	9706342	T	0.3436	1.73E-05	chr:4:9603240
4	rs6822889	9826757	C	0.4399	1.74E-05	chr:4:9603240
4	rs11726476	9858303	T	0.4301	1.77E-05	chr:4:9603240
4	rs6812563	9852757	C	0.4084	1.78E-05	chr:4:9603240
4	rs10939362	9553684	T	0.3651	1.79E-05	chr:4:9603240
4	rs192546093	9170261	T	0.5142	1.79E-05	chr:4:9603240
5	rs76603114	64451012	C	208.2	6.76E-12	chr:5:64388440
5	rs79627297	64431526	C	66.96	9.98E-12	chr:5:64388440
5	rs147069896	64474153	T	92.68	5.35E-11	chr:5:64388440
5	rs114436292	64385381	A	1453	1.11E-09	chr:5:64388440
5	rs369965566	64398886	T	1306	1.30E-09	chr:5:64388440
5	rs139126213	64355569	T	110.3	2.53E-09	chr:5:64388440
5	rs55853058	64436298	T	21.12	6.92E-09	chr:5:64388440
5	rs56246735	64434968	A	21.12	6.92E-09	chr:5:64388440
5	rs67024543	64439173	C	21.12	6.92E-09	chr:5:64388440
5	rs72758879	64437191	G	21.12	6.92E-09	chr:5:64388440
5	rs79124463	64437182	T	21.12	6.92E-09	chr:5:64388440
5	rs115073561	64377414	A	3117	1.40E-08	chr:5:64388440
5	rs11743242	64375379	C	3117	1.40E-08	chr:5:64388440
5	rs11746094	64387612	T	3117	1.40E-08	chr:5:64388440
5	rs11746360	64375515	G	3117	1.40E-08	chr:5:64388440
5	rs150630356	64384299	C	3117	1.40E-08	chr:5:64388440
5	rs182061015	64380725	A	3117	1.40E-08	chr:5:64388440
5	rs192546362	64380724	C	3117	1.40E-08	chr:5:64388440
5	rs77434451	64380180	C	3117	1.40E-08	chr:5:64388440
5	rs77669131	64385985	C	3117	1.40E-08	chr:5:64388440
5	rs77982418	64383897	C	3117	1.40E-08	chr:5:64388440
5	rs75261655	64354080	G	187.6	2.31E-08	chr:5:64388440
5	rs17206232	64433400	A	13.08	3.94E-08	chr:5:64388440
5	rs4700668	64435732	A	13.08	3.94E-08	chr:5:64388440
5	rs55762587	64436355	C	13.08	3.94E-08	chr:5:64388440
5	rs72758880	64440519	A	13.08	3.94E-08	chr:5:64388440
5	rs17812505	64454782	A	126	4.48E-08	chr:5:64388440
5	rs115153571	64501108	T	68.32	5.75E-08	chr:5:64388440
5	rs115596035	64394182	A	3679	9.03E-08	chr:5:64388440

5	rs11750581	64392324	T	3679	9.03E-08	chr:5:64388440
5	rs17206008	64427970	A	3679	9.03E-08	chr:5:64388440
5	rs17811610	64428506	C	3679	9.03E-08	chr:5:64388440
5	rs76294348	64401590	C	3679	9.03E-08	chr:5:64388440
5	rs79066328	64400793	T	3679	9.03E-08	chr:5:64388440
5	rs72758876	64434562	T	11.56	1.04E-07	chr:5:64388440
5	rs72758884	64452214	G	12.12	1.29E-07	chr:5:64388440
5	rs112170622	64456245	T	11.82	1.53E-07	chr:5:64388440
5	rs112408822	64469647	G	11.82	1.53E-07	chr:5:64388440
5	rs56375618	64471186	G	11.82	1.53E-07	chr:5:64388440
5	rs57027704	64470833	C	11.82	1.53E-07	chr:5:64388440
5	rs11742800	64403371	T	377.4	1.96E-07	chr:5:64388440
5	rs7736365	64413591	C	377.4	1.96E-07	chr:5:64388440
5	rs115434958	64475274	A	9.948	4.15E-07	chr:5:64388440
5	rs6861157	64428747	T	8.33	4.61E-07	chr:5:64388440
5	rs11739329	64429954	A	8.181	6.14E-07	chr:5:64388440
5	rs11742881	64373645	A	14.32	9.19E-07	chr:5:64388440
5	rs434882	64353312	A	6.453	4.73E-06	chr:5:64388440
5	rs140796023	64733914	T	15.14	7.28E-06	chr:5:64388440
5	rs2640715	64673742	T	8.456	1.21E-05	chr:5:64388440
5	rs114627939	64411854	A	132.6	1.25E-05	chr:5:64388440
5	rs264733	64359607	G	5.691	1.31E-05	chr:5:64388440
5	rs264737	64363583	A	5.65	1.48E-05	chr:5:64388440
5	rs264741	64367179	T	5.65	1.48E-05	chr:5:64388440
5	rs264742	64368149	T	5.65	1.48E-05	chr:5:64388440
5	rs266591	64347543	G	5.65	1.48E-05	chr:5:64388440
5	rs381652	64352334	C	5.65	1.48E-05	chr:5:64388440
5	rs143761847	64601150	T	13.14	1.50E-05	chr:5:64388440
5	rs190543	64391933	G	5.447	1.50E-05	chr:5:64388440
5	rs264770	64391134	T	5.447	1.50E-05	chr:5:64388440
5	rs264775	64395359	G	5.447	1.50E-05	chr:5:64388440
5	rs4700662	64392906	C	5.447	1.50E-05	chr:5:64388440
5	rs521074	64392198	C	5.447	1.50E-05	chr:5:64388440
5	rs264766	64388979	T	5.428	1.52E-05	chr:5:64388440
5	rs264778	64395979	A	5.291	1.52E-05	chr:5:64388440
5	rs1363921	64496183	T	6.058	1.54E-05	chr:5:64388440
5	rs155337	64375371	A	5.425	1.62E-05	chr:5:64388440
5	rs197138	64376615	G	5.425	1.62E-05	chr:5:64388440
5	rs264752	64375502	T	5.425	1.62E-05	chr:5:64388440
5	rs264753	64375657	T	5.425	1.62E-05	chr:5:64388440
5	rs264754	64377290	A	5.425	1.62E-05	chr:5:64388440
5	rs264756	64380395	G	5.425	1.62E-05	chr:5:64388440
5	rs264762	64384386	A	5.425	1.62E-05	chr:5:64388440

5	rs264763	64384431	C	5.425	1.62E-05	chr:5:64388440
5	rs65407	64375128	C	5.425	1.62E-05	chr:5:64388440
5	rs146117686	64347580	A	28.39	2.08E-05	chr:5:64388440
5	rs58335637	64506243	T	6.261	2.09E-05	chr:5:64388440
5	rs2161559	64493852	C	5.283	2.47E-05	chr:5:64388440
5	rs397044	64391972	C	4.982	2.78E-05	chr:5:64388440
5	rs264758	64381433	A	4.97	2.79E-05	chr:5:64388440
5	rs264765	64388548	C	4.97	2.79E-05	chr:5:64388440
5	rs264779	64398181	C	4.786	2.92E-05	chr:5:64388440
5	rs1862570	64480933	T	6.447	2.99E-05	chr:5:64388440
5	rs426628	64351318	T	4.906	3.03E-05	chr:5:64388440
5	rs266571	64358152	A	5.042	3.14E-05	chr:5:64388440
5	rs67057805	64497594	G	5.188	3.41E-05	chr:5:64388440
5	rs67872027	64517189	C	5.468	3.58E-05	chr:5:64388440
5	rs16893492	64494637	C	5.124	3.88E-05	chr:5:64388440
5	rs16893504	64495736	G	5.124	3.88E-05	chr:5:64388440
5	rs16893539	64506320	G	5.124	3.88E-05	chr:5:64388440
5	rs114465479	64572030	C	9.5	4.07E-05	chr:5:64388440
5	rs4700670	64505218	C	5.378	4.20E-05	chr:5:64388440
5	rs560140895	63996509	T	16.39	5.19E-05	chr:5:64388440
5	rs16893495	64494702	G	5.249	5.26E-05	chr:5:64388440
5	rs6867844	64503815	C	5.249	5.26E-05	chr:5:64388440
5	rs62364962	80465734	G	44.37	6.29E-10	chr:5:80442266
5	rs6881941	80467902	G	44.37	6.29E-10	chr:5:80442266
5	rs6880350	80470499	T	38.71	8.68E-10	chr:5:80442266
5	rs12697915	80458973	G	37.79	1.08E-09	chr:5:80442266
5	rs13159681	80467140	G	28.7	1.09E-09	chr:5:80442266
5	rs6895645	80470495	C	25.46	1.45E-09	chr:5:80442266
5	rs4144527	80467798	C	24.71	1.94E-09	chr:5:80442266
5	rs10474654	80464099	T	12.77	1.99E-09	chr:5:80442266
5	rs6872444	80464801	A	12.77	1.99E-09	chr:5:80442266
5	rs73130824	80475341	C	54.12	2.23E-09	chr:5:80442266
5	rs4704705	80463635	G	12.65	2.67E-09	chr:5:80442266
5	rs6890433	80463887	C	12.65	2.67E-09	chr:5:80442266
5	rs4703824	80466298	T	12.46	2.80E-09	chr:5:80442266
5	rs13159648	80467069	G	18.24	3.82E-09	chr:5:80442266
5	rs113138533	80516504	A	20.97	4.64E-09	chr:5:80442266
5	rs79837333	80512632	C	20.97	4.64E-09	chr:5:80442266
5	rs6891229	80448345	G	40.8	9.80E-09	chr:5:80442266
5	rs6863422	80474998	G	70.29	2.04E-08	chr:5:80442266
5	rs6878238	80474599	T	70.29	2.04E-08	chr:5:80442266
5	rs73130821	80474424	C	70.29	2.04E-08	chr:5:80442266
5	rs79215865	80476220	A	70.29	2.04E-08	chr:5:80442266

5	rs116063300	80444722	C	844.7	4.91E-08	chr:5:80442266
5	rs7707396	80433658	G	18.52	8.91E-08	chr:5:80442266
5	rs115860918	80393475	T	62.26	9.23E-08	chr:5:80442266
5	rs147665836	80382832	C	62.26	9.23E-08	chr:5:80442266
5	rs112680973	80546851	G	14.5	1.06E-07	chr:5:80442266
5	rs115017698	80424934	A	50.72	1.16E-07	chr:5:80442266
5	rs143086439	80424848	T	50.72	1.16E-07	chr:5:80442266
5	rs145198622	80414774	T	34.01	2.02E-07	chr:5:80442266
5	rs80015185	80435871	A	13.41	2.16E-07	chr:5:80442266
5	rs26897	80416882	C	10.97	4.40E-07	chr:5:80442266
5	rs111531198	80550110	T	26.12	6.10E-07	chr:5:80442266
5	rs115568758	80416113	C	16.67	1.15E-06	chr:5:80442266
5	rs116503403	80427691	A	12.24	2.45E-06	chr:5:80442266
5	rs111809751	80316223	G	15.2	2.79E-06	chr:5:80442266
5	rs11949363	80509137	G	5.516	3.69E-06	chr:5:80442266
5	rs76834320	80416108	G	12.59	3.94E-06	chr:5:80442266
5	rs540564	80403392	A	4.793	5.28E-06	chr:5:80442266
5	rs17212948	80406756	C	4.786	5.60E-06	chr:5:80442266
5	rs6882101	80458283	T	5.863	6.68E-06	chr:5:80442266
5	rs34121	80438915	G	4.996	7.82E-06	chr:5:80442266
5	rs111796768	80299334	A	8.189	1.23E-05	chr:5:80442266
5	rs12697916	80462439	G	4.992	1.27E-05	chr:5:80442266
5	rs13353953	80462407	A	4.993	1.29E-05	chr:5:80442266
5	rs190585232	80356070	T	13.28	1.29E-05	chr:5:80442266
5	rs10942943	80462935	G	4.929	1.46E-05	chr:5:80442266
5	rs10942944	80462963	G	4.929	1.46E-05	chr:5:80442266
5	rs39628	80075674	T	5.577	1.46E-05	chr:5:80442266
5	rs33010	80083631	G	5.519	1.66E-05	chr:5:80442266
5	rs190339	80431615	C	4.472	1.75E-05	chr:5:80442266
5	rs193786	80441637	T	4.516	1.85E-05	chr:5:80442266
5	rs34116	80441999	C	4.516	1.85E-05	chr:5:80442266
5	rs34120	80440146	G	4.516	1.85E-05	chr:5:80442266
5	rs245403	80069930	A	5.437	1.94E-05	chr:5:80442266
5	rs245408	80072451	G	5.437	1.94E-05	chr:5:80442266
5	rs40300	80067803	T	5.437	1.94E-05	chr:5:80442266
5	rs12188759	80462147	C	4.654	2.01E-05	chr:5:80442266
5	rs4703822	80411221	A	4.012	2.62E-05	chr:5:80442266
5	rs12186554	80462184	T	4.184	3.45E-05	chr:5:80442266
5	rs17277408	80379572	T	5.843	5.15E-05	chr:5:80442266
5	rs13160367	8936774	A	0.08443	6.41E-12	chr:5:8937853
5	rs2963382	8948523	C	0.1102	6.51E-12	chr:5:8937853
5	rs6861103	8928381	A	0.1008	6.63E-12	chr:5:8937853
5	rs11949503	8937804	C	0.08644	8.61E-12	chr:5:8937853

5	rs11955429	8941715	A	0.08644	8.61E-12	chr:5:8937853
5	rs12186849	8933088	C	0.08644	8.61E-12	chr:5:8937853
5	rs13157678	8931378	C	0.08644	8.61E-12	chr:5:8937853
5	rs13160595	8929697	C	0.08644	8.61E-12	chr:5:8937853
5	rs13165792	8943070	T	0.08644	8.61E-12	chr:5:8937853
5	rs13166892	8931212	C	0.08644	8.61E-12	chr:5:8937853
5	rs13169109	8943532	C	0.08644	8.61E-12	chr:5:8937853
5	rs13170524	8934862	G	0.08644	8.61E-12	chr:5:8937853
5	rs13180198	8936074	C	0.08644	8.61E-12	chr:5:8937853
5	rs13181039	8936810	C	0.08644	8.61E-12	chr:5:8937853
5	rs13181188	8936871	G	0.08644	8.61E-12	chr:5:8937853
5	rs13185206	8943046	G	0.08644	8.61E-12	chr:5:8937853
5	rs13186994	8930953	C	0.08644	8.61E-12	chr:5:8937853
5	rs2962735	8942095	G	0.08644	8.61E-12	chr:5:8937853
5	rs34108904	8943745	T	0.08644	8.61E-12	chr:5:8937853
5	rs35376107	8942188	G	0.08644	8.61E-12	chr:5:8937853
5	rs36075104	8937389	A	0.08644	8.61E-12	chr:5:8937853
5	rs4495147	8933733	T	0.08644	8.61E-12	chr:5:8937853
5	rs4505924	8936629	A	0.08644	8.61E-12	chr:5:8937853
5	rs4526080	8941529	G	0.08644	8.61E-12	chr:5:8937853
5	rs4563593	8933794	G	0.08644	8.61E-12	chr:5:8937853
5	rs4585412	8941705	G	0.08644	8.61E-12	chr:5:8937853
5	rs6555580	8932263	C	0.08644	8.61E-12	chr:5:8937853
5	rs6555585	8935651	G	0.08644	8.61E-12	chr:5:8937853
5	rs6555586	8935800	A	0.08644	8.61E-12	chr:5:8937853
5	rs6862593	8928550	T	0.08644	8.61E-12	chr:5:8937853
5	rs6866047	8932660	T	0.08644	8.61E-12	chr:5:8937853
5	rs6870451	8938885	G	0.08644	8.61E-12	chr:5:8937853
5	rs6870477	8938935	G	0.08644	8.61E-12	chr:5:8937853
5	rs6884096	8932523	A	0.08644	8.61E-12	chr:5:8937853
5	rs6885789	8932782	T	0.08644	8.61E-12	chr:5:8937853
5	rs6888819	8929239	C	0.08644	8.61E-12	chr:5:8937853
5	rs6894834	8930468	G	0.08644	8.61E-12	chr:5:8937853
5	rs71611354	8942703	T	0.08644	8.61E-12	chr:5:8937853
5	rs76109783	8939954	A	0.08644	8.61E-12	chr:5:8937853
5	rs7735641	8935206	A	0.08644	8.61E-12	chr:5:8937853
5	rs34251442	8942645	C	0.09611	1.03E-11	chr:5:8937853
[truncated: 477,246 more chars]
